# Identification and characterization of HPV-independent cervical cancers

**DOI:** 10.18632/oncotarget.14533

**Published:** 2017-01-06

**Authors:** Carolyn E. Banister, Changlong Liu, Lucia Pirisi, Kim E. Creek, Phillip J. Buckhaults

**Affiliations:** ^1^ University of South Carolina College of Pharmacy, Columbia, SC, Canada; ^2^ University of South Carolina School of Medicine, Columbia, SC, Canada

**Keywords:** HPV, cervical cancer, TP53, CTNNB1, APOBEC

## Abstract

**Background:**

Human papillomavirus (HPV) initiates cervical cancer, and continuous expression of HPV oncogenes E6 and E7 is thought to be necessary to maintain malignant growth. Current therapies target proliferating cells, rather than specific pathways, and most experimental therapies specifically target E6/E7. We investigated the presence and expression of HPV in cervical cancer, to correlate HPV oncogene expression with clinical and molecular features of these tumors that may be relevant to new targeted therapies.

**Results:**

While virtually all cervical cancers contained HPV DNA, and most expressed E6/E7 (HPV-active), a subset (8%) of HPV DNA-positive cervical cancers did not express HPV transcripts (HPV-inactive). HPV-inactive tumors occurred in older women (median 54 vs. 45 years, *p* = 0.02) and were associated with poorer survival (median 715 vs 3046 days, *p* = 0.0003). Gene expression profiles of HPV-active and -inactive tumors were distinct. HPV-active tumors expressed E2F target genes and increased AKT/MTOR signaling. HPV-inactive tumors had increased WNT/β-catenin and Sonic Hedgehog signaling. Substantial genome-wide differences in DNA methylation were observed. HPV-inactive tumors had a global decrease in DNA methylation; however, many promoter-associated CpGs were hypermethylated. Many inflammatory response genes showed promoter methylation and decreased expression. The somatic mutation landscapes were significantly different. HPV-active tumors carried few somatic mutations in driver genes, whereas HPV-inactive tumors were enriched for non-synonymous somatic mutations (*p*-value < 0.0000001) specifically targeting TP53, ARID, WNT, and PI3K pathways.

**Materials and Methods:**

The Cancer Genome Atlas (TCGA) cervical cancer data were analyzed.

**Conclusions:**

Many of the gene expression changes and somatic mutations found in HPV-inactive tumors alter pathways for which targeted therapeutics are available. Treatment strategies focused on WNT, PI3K, or TP53 mutations may be effective against HPV-inactive tumors and could improve survival for these cervical cancer patients.

## INTRODUCTION

Essentially all cervical cancers contain human papillomavirus (HPV) DNA [1] suggesting that HPV infection is necessary for cervical cancer initiation. Routine HPV testing has revealed that most HPV infections resolve, indicating that HPV infection is necessary, but not sufficient for the development of cervical cancer, and that additional events are required [2].

The molecular features of cervical cancers are beginning to be described [3–6]. Here we report a detailed analysis of TCGA cervical cancer gene expression, DNA methylation, and somatic mutation profiles. Similar to recent reports in head and neck cancers [7], we identified a subset of tumors, which no longer express HPV E6/E7 oncogenes (HPV-inactive). These tumors have gene expression, DNA methylation and somatic mutation signatures different from HPV-active tumors, and more similar to those of other, viral-independent cancers. Implications for cervical cancer progression, and opportunities for targeted therapy are discussed.

## RESULTS

### Identification of HPV-inactive tumors

**Figure 1:**
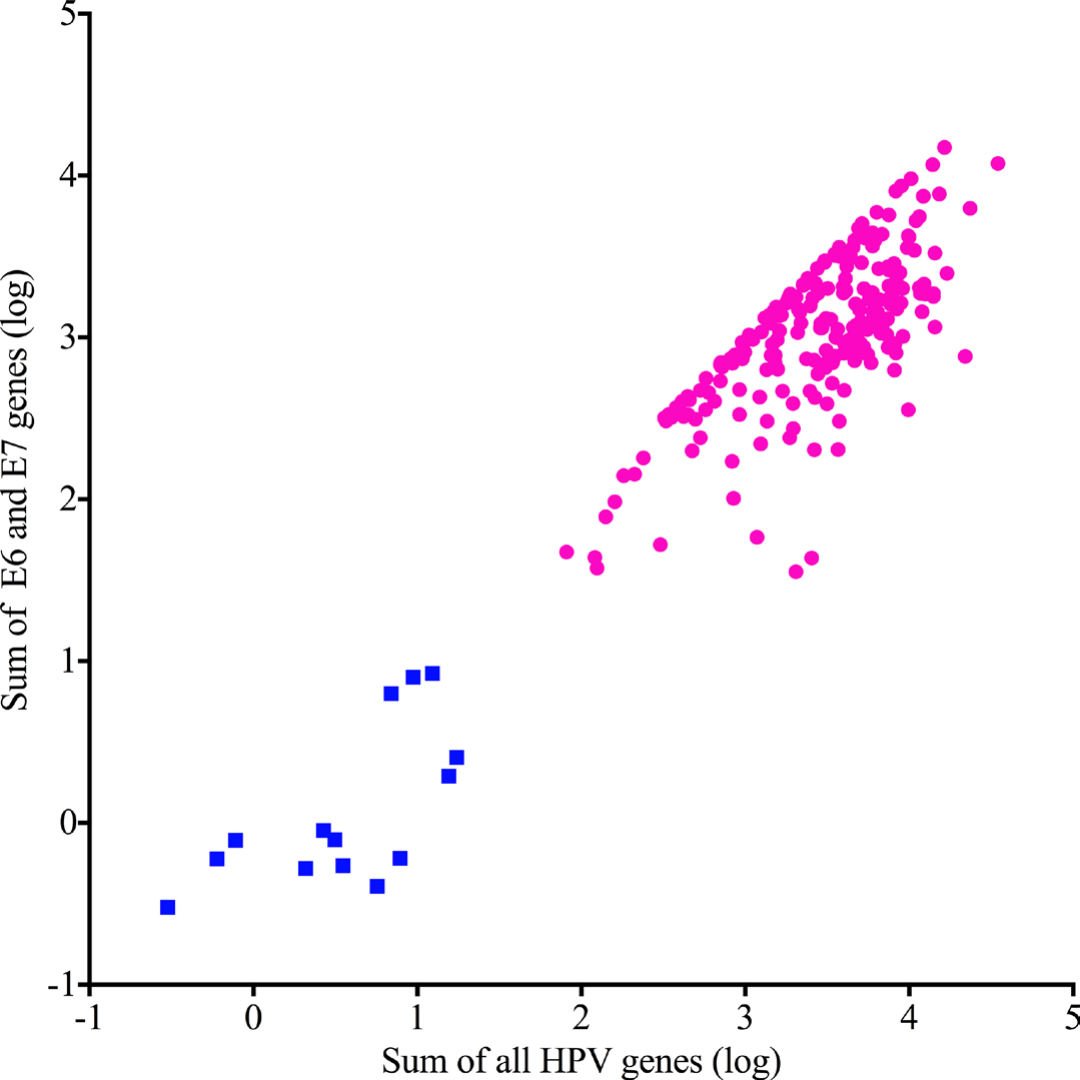
Unsupervised classification of cervical cancers by HPV gene expression HPV-active (circles) and HPV-inactive (squares) differ by total gene and oncogene expression levels.

We extracted all reads from 264 BAM files previously assembled to GRCh37-lite+HPV-Redux reference genome, and then re-assembled to a custom reference genome containing the HPV types most often associated with cervical cancer (Table 1). All but one of the cervical cancers analyzed (Supplementary Table 1) contained two or more normalized counts of Illumina DNA sequencing reads that assembled with HPV and were classified as HPV positive. In order to assign the HPV type, we used a more stringent cutoff of 50 normalized counts of HPV aligned reads, to classify a sample positive for a specific HPV type. We manually inspected all alignments and discarded instances of duplicated reads aligning to more than one HPV type. Using these criteria, there were 255 samples that we used to determine HPV type distribution (Table 1). Twenty-nine percent (75/255) of typed samples were positive for multiple HPV types. In order to characterize the gene expression landscapes of cervical cancers, we obtained the RNA-seq FASTQ files from the 261 patients with both tumor DNA and RNA sequencing data available, and determined expression levels of all genes (including HPV genes) (Supplementary Table 2A). To characterize the samples by HPV oncogene expression, we plotted the sum of the E6 and E7 expression against the sum of all HPV gene expression and performed unsupervised partitioning by K-Means clustering using the Euclidian distance function. This class discovery method revealed 2 distinct classes of cervical cancers (Figure 1), those with high levels of E6 and E7 oncogene expression (HPV-active, *n* = 241, 92.3%) and those with low, or zero E6/E7 expression (HPV-inactive, *n* = 20, 7.7%), similar to what we previously reported for head and neck cancer [7].

**Table 1:** Number of cervical cancer samples positive for each HPV type*

HPV type	Genome build	*n* (%)
HPV16	NC_001526.2.gb	188 (73.7)
HPV18	NC_001357.1.gb	63 (24.7)
HPV31	J04353.1.gb	18 (7.1)
HPV33	M12732.1.gb	11 (4.3)
HPV35	M74117.1.gb	6 (2.4)
HPV39	M62849.1.gb	11 (4.3)
HPV45	EF202167.1.gb	33 (12.9)
HPV51	M62877.1.gb	1 (0.4)
HPV52	GQ472848.1.gb	12 (4.7)
HPV53	NC_001593.1.gb	1 (0.4)
HPV56	EF177181.1.gb	7 (2.8)
HPV58	FJ385268.1.gb	19 (7.5)
HPV59	EU918767.1.gb	10 (3.9)
HPV66	U31794.1.gb	1 (0.4)
HPV68	FR751039.1	3 (1.2)
HPV72	X94164.1	0 (0.0)
HPV73	X94165.1.gb	1 (0.4)

* number of typed HPV tumors analyzed: 255.

### Demographic characteristics

**Figure 2:**
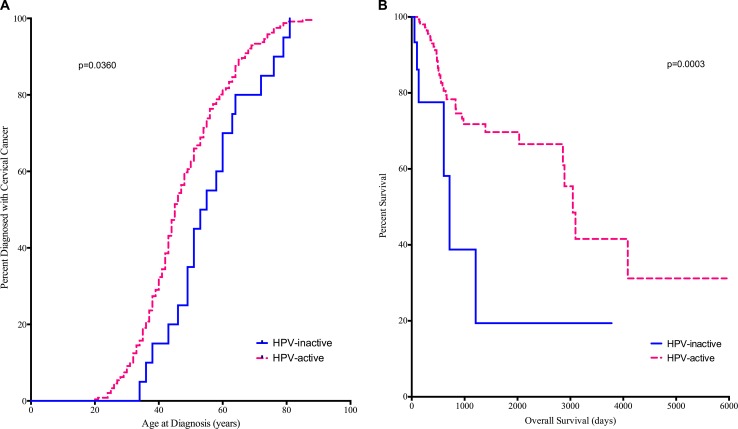
Diagnosis (A) and Survival (B) are compared between HPV-active (dashed line) and HPV-inactive (solid line) patients Patients with HPV-inactive tumors were on average 9 years older at diagnosis and died on average 6.4 years earlier.

The distributions of patient ancestry, tumor grade, and stage were not different between HPV-active and -inactive cancers (Table 2, Supplementary Table 3). However, patients with HPV-inactive tumors were significantly older at diagnosis (median age 54 vs 45, log rank Mantel-Cox *p* = 0.0360) (Figure 2A), and their median survival was dramatically shorter (715 days vs 3046 days, Gehan-Breslow-Wilcoxon *p* = 0.0003) (Figure 2B). Adenocarcinomas and adenosquamous carcinomas were significantly more common in the HPV-inactive than in the HPV-active tumors (Table 2, Supplementary Table 3) (*p* = 0.00022). Additionally, the percentage of HPV16 positive tumors was greater in the HPV-inactive tumors (81.8% vs 73.8%, Supplementary Table 1). Taken together, these differences point to the existence of a subtype of cervical cancers that silence HPV oncogene expression over time and have a worse prognosis.

**Table 2:** Patient characteristics

		Total	HPV inactive	HPV active	*p*-value
Total n		261	20	241	
Ethnicity n (%)					
	American Indian	7 (3.1)	1 (5.9)	6 (2.9)	0.89
	Asian and Pacific Islander	20 (8.9)	1 (5.9)	19 (9.1)	
	Black	25 (11.1)	2 (11.8)	23 (11.1)	
	White	173 (76.9)	13 (76.5)	160 (76.9)	
	Unknown	36	3	33	
Diagnosis n (%)					
	Adenocarcinoma	44 (16.9)	8 (40.0)	36 (14.9)	0.00022
	Adenosquamous	5 (1.9)	2 (10)	3 (1.2)	
	Squamous Cell Carcinoma	212 (81.2)	10 (50.0)	202 (83.8)	
Grade n (%)					
	G1	16 (7.0)	0 (0.0)	16 (7.6)	0.22
	G2	115 (50.2)	7 (36.8)	108 (51.4)	
	G3	97 (42.3)	12 (63.2)	85 (40.5)	
	G4	1 (0.4)	0 (0.0)	1 (0.5)	
	GX (Unknown)	32	1	31	
Stage n (%)					
	1	132 (52.0)	11 (55.0)	121 (51.7)	0.41
	2	64 (25.2)	5 (25.0)	59 (25.2)	
	3	38 (15.0)	1 (5.0)	37 (15.8)	
	4	20 (7.9)	3 (15.0)	17 (7.3)	
	Unknown	7	0	7	

### Gene expression differences

**Figure 3:**
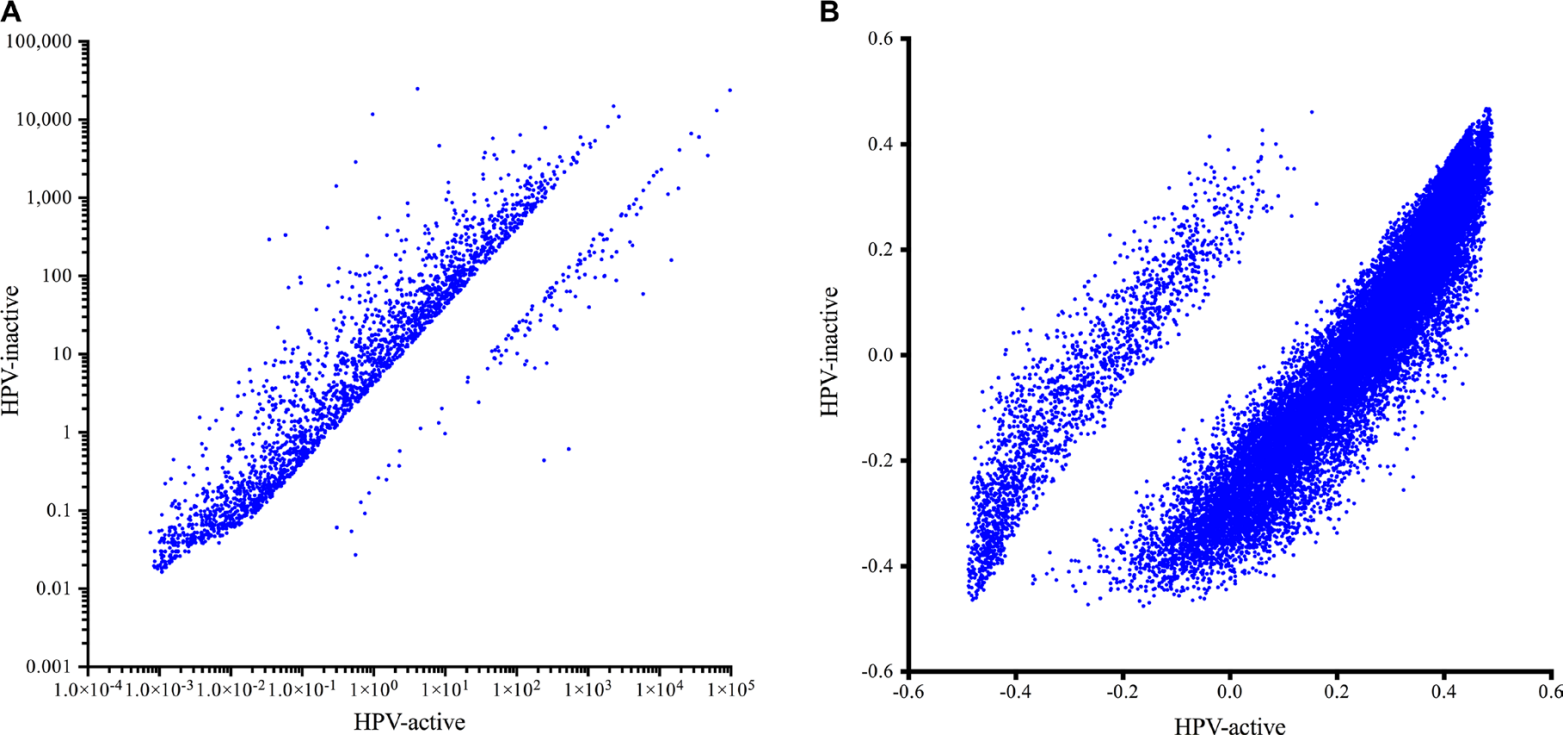
Gene expression (A) and DNA methylation (B) in HPV-active (horizontal axis) and HPV-inactive (vertical axis) cervical tumors The majority of significantly different genes had increased expression in HPV-inactive tumors. The majority of significantly different CpG loci were decreased in DNA methylation in HPV-inactive tumors.

We compared expression levels for 40,014 genomic elements and found that 2446 genes were significantly differentially expressed (4-fold up or down; FDR-adjusted *p* < 0.05) (Figure 3A, Supplementary Table 4). The majority of these most differentially expressed genes (94.6%) were overexpressed in the HPV-inactive tumors. In order to explore differences in biological themes, we performed Gene Set Enrichment Analysis (Partek Genomics Suite) using normalized gene expression data (Supplementary Table 2A) to query the hallmarks (h) and the chemical and genetic perturbations (cgp) gene sets [8]. Selected enriched gene sets are summarized in Table 3, enrichment scores and *p*-values for all gene sets are listed in Supplementary Tables 2B and 2C.

**Table 3:** Select gene sets enriched in HPV-inactive cervical cancers

Gene Set	Number of Genes	Enrichment Score	p-value (unadjusted)	FDR	Enrichment Direction
Ishida E2F Targets*	52	−0.5995	0.0303	0.1172	HPV-inactive down
Slebos head and neck cancer with HPV up*	83	−0.6997	0	0	HPV-inactive down
Pyeon HPV positive tumors up*	97	−0.6314	0	0.0356	HPV-inactive down
Hallmark WNT beta catenin signaling#	42	0.5692	0	0.1931	HPV-inactive up
Browne interferon responsive genes*	66	−0.7552	0	0.0208	HPV-inactive down
Sana response to INFG up*	75	−0.6749	0	0.0340	HPV-inactive down
Moserle INFA response*	31	−0.7897	0	0.0303	HPV-inactive down
Einav interferon signature in cancer*	26	−0.7544	0	0.0430	HPV-inactive down
Hecker IFNB1 targets*	95	−0.6298	0	0.0373	HPV-inactive down
Der inf alpha response up*	74	−0.5767	0	0.0417	HPV-inactive down
Der inf beta response up*	102	−0.5020	0	0.0548	HPV-inactive down
Der inf gamma response up*	71	−0.5439	0	0.0416	HPV-inactive down
Interferon alpha response#	94	−0.7239	0	0	HPV-inactive down
Interferon gamma response#	196	−0.6566	0	0	HPV-inactive down
Bosco interferon induced antiviral module*	76	−0.6342	0	0.0339	HPV-inactive down
Zhang interferon response*	23	−0.7519	0	0.0397	HPV-inactive down
Coldren Gefitinib resistance up *	80	0.6306	0.0127	0.1624	HPV-inactive up
Coldren Gefitinib resistance down *	223	−0.4051	0.05	0.2093	HPV-inactive down
Huang Dasatinib resistance up *	80	−0.5863	0	0.0344	HPV-inactive down
Huang Dasatinib resistance down *	66	0.4547	0.0133	0.4639	HPV-inactive up

Select gene sets that are enriched in HPV-inactive compared to HPV-active cervical cancers. Gene set enrichment analysis was performed in Partek Genomics Suite using the Hallmark (#) and the Chemical and Genetic Perturbations (*) gene sets.

As would be predicted for tumors that have become HPV-independent, E2F target gene sets are decreased in expression in the HPV-inactive tumors (Ishida_E2F_Targets-c2.cgp). The expression of HPV associated gene sets that characterize gene expression profiles of HPV-driven tumors of the oropharynx is also decreased in the HPV-inactive tumors (Slebos_head_and_neck_cancer_with_HPV_up-c2.cgp, Pyeon_HPV_positive_tumors_up–c2.cgp). These results support the conclusion that the HPV-inactive cervical tumors are more similar to HPV negative head and neck tumors than to HPV-positive tumors, and they show that the HPV-inactive tumor class is not only characterized by the absence of HPV transcripts, but also by the absence of expression of many other human genes normally positively correlated with the presence and expression of HPV.

HPV-inactive tumors have lost E6 and E7 oncogene expression, and have evolved alternative pathways to support cancerous growth. Gene sets associated with activated WNT/CTNNB1 signaling, a pathway important to other types of adenocarcinomas, are elevated in expression in the HPV-inactive tumor class (Hallmark_WNT_beta_catenin_signaling–h.all.v5.1).

Multiple interferon response gene sets are decreased in the HPV-inactive tumors, including gene sets that deal directly with the inflammatory reaction (Browne _interferon_responsive_genes–c2.cgp, Sana_response_to_INFG_up–c2.cgp, Moserle_INFA_response–c2.cgp, Einav _interferon_signature_in_cancer–c2.cgp, Hecker_IFNB1_targets–c2.cgp, Der_inf_alpha_response_up–c2.cgp, Der_inf_beta_response_up–c2.cgp, Der_inf_gamma_response _up–c2.cgp, interferon_alpha_response-h.all.v5.1, interferon _gamma_response-h.all.v5.1), and gene sets that deal indirectly with viral mediated inflammatory response (Bosco_interferon_induced_antiviral_module–c2.cgp, Zhang_interferon_response–c2.cgp). Thus, HPV-active tumors still have an active interferon associated inflammation response that is lost in HPV-inactive tumors. Robust inflammation present in the HPV-active tumors, along with higher expression of immune checkpoint inhibitor targets TIGIT, CTLA4 and PDL-1 (Supplementary Table 2A), suggest that immune checkpoint inhibitor therapy may be a productive approach for these virally driven cancers.

Differences in gefitinib resistance gene sets (Coldren_Gefitinib_resistance_up – c2.cgp, Coldren_Gefitinib_resistance_down – c2.cgp) indicate gefitinib would be more effective in HPV-active tumors, whereas differences in dasatinib resistance gene sets (Huang_Dasatinib_resistance_up – c2.cgp, Huang_Dasatinib_resistance_down – c2.cgp) indicate HPV-inactive tumors may be more sensitive to dasatinib therapy. Thus, informing treatment decisions based on HPV gene expression may improve outcomes for cervical cancer patients.

### Gene methylation differences

**Figure 4:**
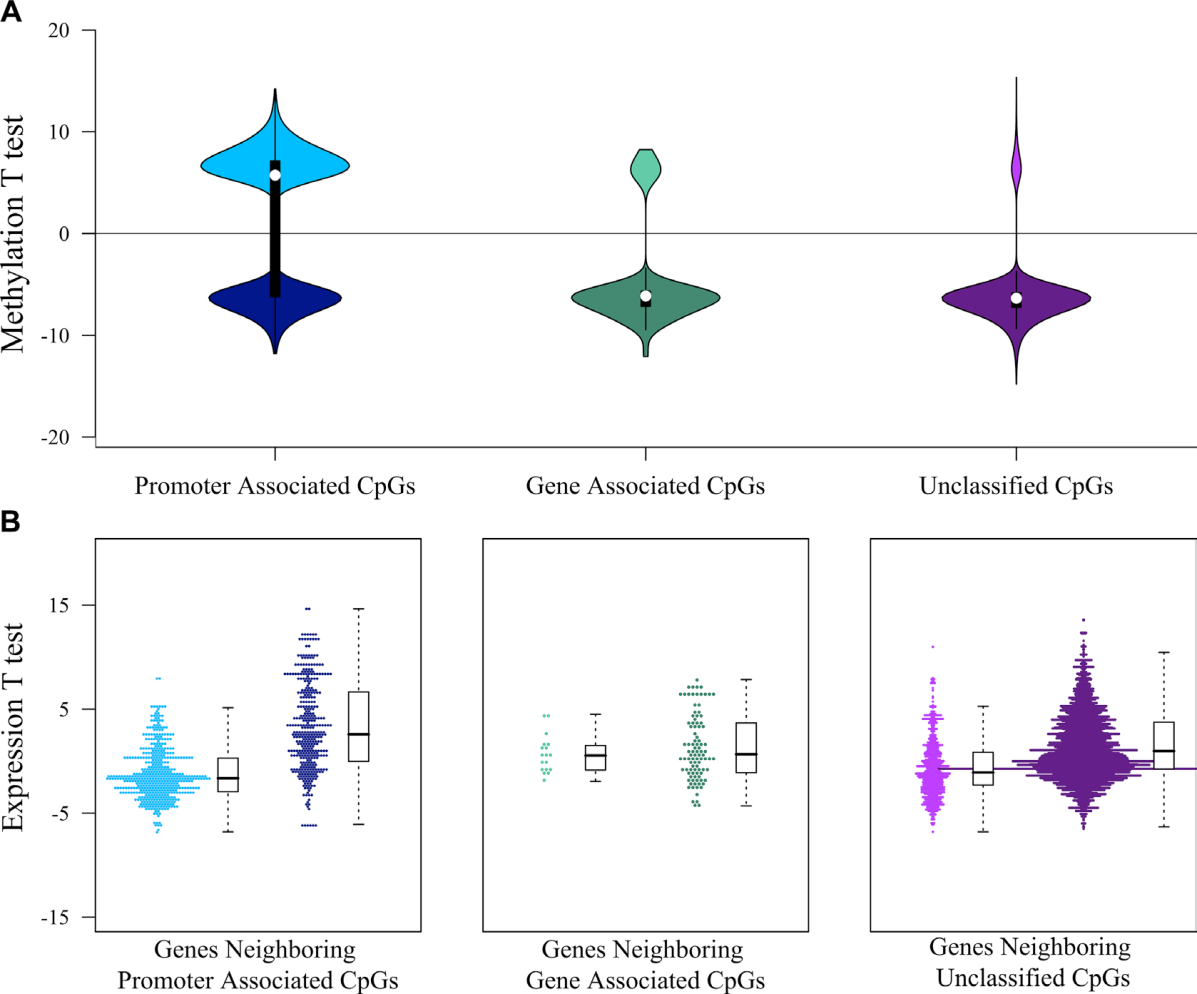
Interaction between DNA methylation and gene expression landscapes (**A**) Distribution of the DNA methylation test statistics (HPV-inactive vs. HPV-active) of the significant differently methylated CpGs. The majority of gene-associated and unclassified CpGs were hypomethylated in HPV-inactive tumors (negative test statistic), whereas promoter-associated CpGs were equally divided between hypermethylated (positive test statistic) and hypomethylated (negative test statistic). (**B**) Distribution of the gene expression test statistics (HPV-inactive vs. HPV-active) of the genes associated with significant differently methylated CpGs. Test statistics (and expression levels) of genes near gene-associated or unclassified CpGs were not correlated with CpG methylation levels, whereas test statistics (and expression levels) of genes near promoter-associated CpGs were inversely correlated with CpG methylation levels.

To explore mechanisms that could explain the differential gene expression observed, we compared DNA methylation and found a dramatic genome-wide loss of methylation in the HPV-inactive class, with 24,206 loci being significantly differentially methylated (Bonferroni-adjusted *p* < 0.05). Among these most differentially methylated loci, 77% were hypomethylated in HPV-inactive tumors. This is concordant with reports that viral independent cancers are often hypomethylated compared to the normal surrounding tissue [9] (Figure 3B, Supplementary Table 5). Loci associated with gene promoter regions were evenly split between increased and decreased methylation (Figure 4A) and we noted that the gene expression was inversely correlated with promoter methylation (Figure 4B). There were 164 genes that had increased promoter methylation and decreased gene expression in HPV-inactive tumors (Supplementary Table 6). These genes were significantly enriched for the interferon response gene sets (Hallmarks_Interferon_Gamma_Response, Hallmarks_Interferon_Alpha_Response).

### Somatic mutation landscape differences

We compared the numbers and types of somatic mutations between the two classes (Table 4). HPV-active tumors had on average 115 somatic mutations per tumor, whereas HPV-inactive tumors had 228 somatic mutations per tumor. The overall background rates of silent (synonymous) mutations were different. HPV-inactive tumors had, on average 1.7 times as many silent mutations as did HPV-active tumors (58.5 mutations per tumor vs 34.8 mutations per tumor). This is consistent with the older age of HPV-inactive patients, which may have allowed additional passenger mutations to accumulate in the progenitor cell that became the last common ancestor of the tumor. We also noted a larger difference in the numbers of non-synonymous somatic mutations. HPV-inactive tumors had on average twice as many non-synonymous mutations as did HPV-active tumors (169 non-synonymous mutations per tumor vs 80 non-synonymous mutations per tumor). The ratio of non-synonymous to synonymous mutations, which is a crude measure of driver mutation load, was significantly higher in the HPV-inactive tumors (2.9 vs 2.3, Chi square two-tailed *p* < 0.0000001, odds ratio = 1.26 CI = 1.17–1.35) (Table 4). The non-synonymous/synonymous ratio in the HPV-active tumors (2.3) is not significantly different from the 2.2 ratio that is expected to occur by chance alone [10], indicating that the somatic mutations in HPV-active tumors are not enriched for drivers. In contrast, the non-synonymous/synonymous ratio in the HPV-inactive tumors (2.9) is significantly greater than what is expected by chance alone, which indicates that these tumors are enriched for cancer driver mutations enabling them to evolve to HPV independence by acquiring non-synonymous driver mutations to cancer causing genes.

**Table 4:** Somatic mutation counts in cervical cancers

	HPV-active tumors	HPV-inactive tumors*	
n	239	19	
Total Mutations	27495	4326	
Mutations/person	115.0	227.7	
Nonsynonymous	19173	3215	
Synonymous	8322	1111	
NS/S ratio	2.30	2.89	*p*-value < 0.0000001

* Sample TCGA-2W-A8YY had 9,467 somatic mutations and was classified as a hyper-mutator and removed from the analysis.

We also investigated potential differences in the underlying mutation processes by enumerating and comparing each of the six types of nucleotide substitutions that can possibly occur (A:T > C:G, A:T > G:C, A:T > T:A, C:G > A:T, C:G > G:C, C:G > T:A) [11]. As has been seen in other epithelial cancers [12], the most common single nucleotide substitution observed was C:G > T:A, representing 44% of the mutational burden; however, this substitution was not different between the two groups of cervical cancer. C:G > T:A mutations have been previously reported in cervical cancers [4] and can occur by different mechanisms depending on the sequence context. For example, in the a CpG dinucleotide, the C > T mutation is due to the spontaneous deamination of methylated cytosines [13]. This type of mutation was significantly enriched in HPV-inactive tumors (HPV-inactive 638/4,326 vs. HPV-active 2,710/27,495; Chi Square *p*-value < 0.0000001). The PTEN tumor suppressor gene was more frequently the target of non-enzymatic deamination of methylated cytosine (3/5 mutations, 60%) in the HPV-inactive tumors, and less frequently the target of this process (3/14 mutations, 21%) in HPV-active tumors.

Alternatively, C:G > T:A mutations when the C is preceded by a T are caused by APOBEC family of cytidine deaminases [11, 14]. The activity of this enzyme is upregulated in HPV infected cells leading to a characteristic mutation pattern often seen in HPV-mediated cancers [15]. We found that HPV-active tumors have a significantly greater number of C:G > T:A mutations in a TpC context (HPV-active 7,342/27,495 vs HPV-inactive 808/4,326; Chi Square *p*-value < 0.0000001). The majority of the PIK3CA mutations observed in the HPV-active tumors were C:G > T:A substitutions (29/34, 85%) in which the C was preceded by a T (TpC), consistent with an APOBEC mediated mutational process [16]. In contrast, only 33% of the PIK3CA mutations in HPV-inactive tumors (2/6) were C:G > T:A substitutions in which the C is preceded by a T (TpC). These two different mutation patterns (C > T) suggest that HPV-active and HPV-inactive tumors have different mutational process histories.

**Figure 5:**
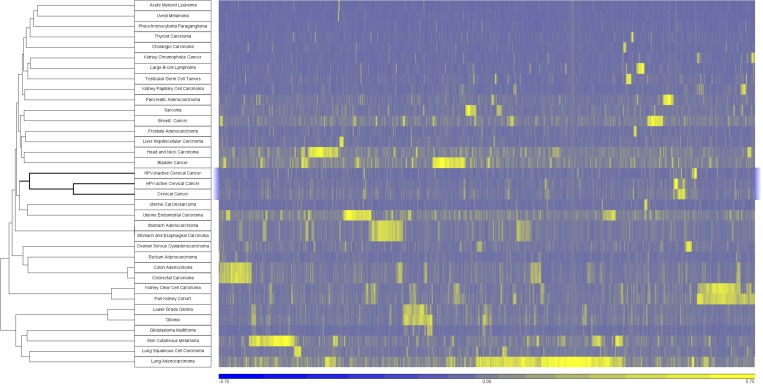
Cancers clustered by somatic mutation profiles Samples were clustered using the –log transformation of the p-value obtained from MutSigCV. Unsupervised clustering was performed using Pearson dissimilarity with complete linkage. A total of 7882 genes were significantly mutated (MutSigCV v0.9 p-value < 0.05) in at least one cohort.

In order to place cervical cancers of both classes into context based on somatic mutations, we performed a global comparison of select TCGA cancer cohorts according to the significance of somatic mutations [17]. MutSig outputs containing the *p*-values for every gene in select cancer cohorts were obtained from Firebrowse.org. There were 18,278 genes with one or more non-synonymous somatic mutations in any of the 33 select TCGA cohorts. MutSig analysis revealed that 7,882 genes are significant, with a nominal *p*-value less than 0.05 in at least one cohort (MutSigCV v0.9). We used the minus-log *p*-values of these genes to drive unsupervised hierarchical cluster analysis of the cohorts (Figure 5). In general, tumor cohorts clustered according to organ system such as GI tumors (stomach, colon and rectum), brain (glioma, lower grade glioma, glioma multiforme), and kidney (papillary, clear cell). The cervical cancers including both subsets of cervical cancers (HPV-active and HPV-inactive) are closely associated with uterine cancers (carcinosarcoma, endometrial carcinoma) with HPV-inactive cervical cancers mutationally more similar to uterine endometrial cancers.

**Figure 6:**
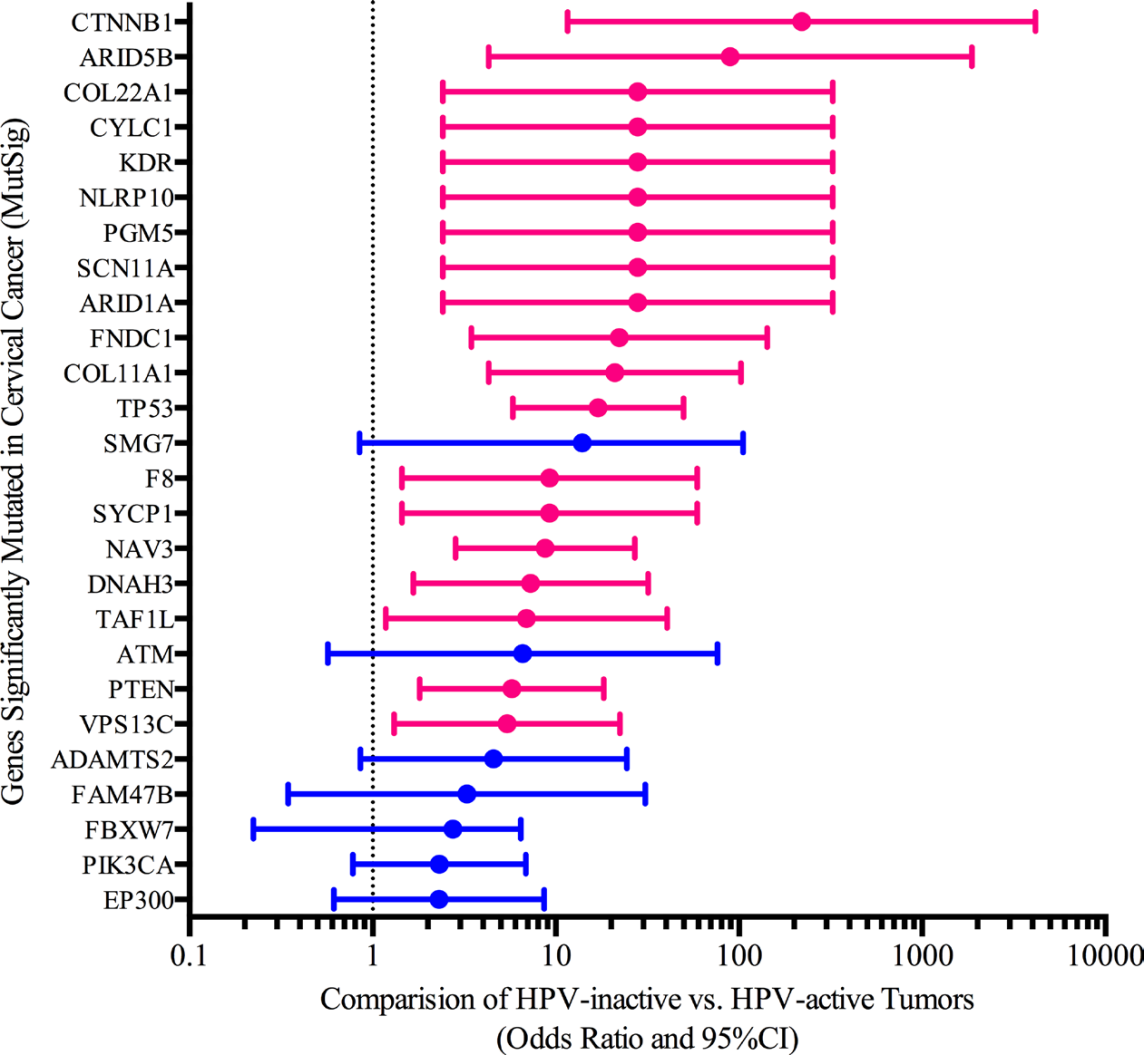
Somatic mutation differences between HPV-active and HPV-inactive cervical cancers Pink bars highlight significant genes with odds ratios larger than the 95% confidence interval.

In general, the frequencies with which MutSig significant cervical cancer genes are mutated are similar between the two classes of cervical cancer. For example, cervical cancer associated genes EP300 has on average 0.17 and 0.15 mutations per tumor (HPV-inactive, HPV-active respectively). Another commonly mutated gene, FBXW7, has 0.15 and 0.10 mutations per tumor (HPV-inactive, HPV-active respectively). However, there was a significantly different distribution in the mutation frequencies of other key cancer driver genes (Figure 6). We compared the mutational frequencies of the HPV-inactive and HPV-active tumors using our somatic mutation calls on 258 tumor/normal cervical cancer pairs. 361/468 genes had at least one somatic mutation identified by our variant calling pipeline. Twenty-six genes had different somatic mutation rates and all were more frequently mutated in the HPV-inactive tumors (Figure 6). TP53, a gene not commonly mutated in cervical cancers, was 17 times more likely (95% CI 5.8, 49.7) to be mutated in HPV-inactive tumors (47%) than in HPV-active tumors (4%). This is consistent with the absence of E6 HPV oncogene expression in inactive tumors. The enrichment of TP53 mutations in the HPV-inactive tumors provides convincing evidence that these tumors have escaped HPV dependence. Chromatin remodeling genes ARID1A and ARID5B, WNT signaling regulator CTNNB1, and the negative regulator of PI3K signaling PTEN, were among the notable genes significantly more mutated in HPV-inactive cervical cancers. Previously, TP53 and ARID1A have been reported as mutually exclusive in endometrial cancers [18]. We found that one tumor contained mutations in both of these genes and twenty-six contain mutations in one of these genes reinforcing the mutual exclusivity between these genes. In addition to PTEN mutational differences, the PIK3CA gene was more frequently mutated in the HPV-inactive tumors (26% vs 13%). Overall, AKT pathway mutations are more common in the HPV-inactive tumors, indicating that an AKT inhibitor may be more effective in treating these tumors [19]. Finally, the enrichment of the CTNNB1 mutations in HPV-inactive tumors is noteworthy because of the widespread up-regulation of WNT target gene expression that we detected in the HPV-inactive tumors by GSEA.

## DISCUSSION

Essentially all cervical cancers are HPV positive by DNA, and it is widely accepted that HPV oncogene expression is necessary for cervical cancer development. Here we show strong evidence that a fraction of cervical cancers can slowly evolve to HPV independence by accumulating somatic mutations to cancer driver genes. HPV-inactive cancers differ from HPV-active by more than just the expression of HPV E6/E7, diminishing the possibility of a trivial explanation such as a technical failure to detect HPV transcripts. The patients harboring HPV-active tumors are on average 9 years younger than the HPV-inactive patients, and they have dramatically longer overall survival (4.3 fold). Global gene expression changes are also apparent, with E2F target genes up-regulated in HPV-active tumors. Additionally, other gene expression differences mirror what has been reported for HPV-positive and HPV-negative head and neck cancers [7, 20]. There were also profound differences in global DNA methylation levels, with more methylation observed in HPV-active tumors than in HPV-inactive tumors. The somatic mutation profiles are also substantially different. The ratio of non-synonymous to synonymous mutations, which is a crude measure of driver gene mutation load, is low for TCGA cervical cancers, but HPV-inactive tumors are significantly enriched for non-synonymous changes, which often target important cancer driver genes. Somatic mutations consisting of C:G > T:A changes are common in both types of cervical cancers, but are unevenly distributed when subdivided according to the surrounding sequence context and mutational mechanism. For example, the PTEN tumor suppressor gene was more frequently mutated in HPV-inactive tumors, and it was more often the target of spontaneous, non-enzymatic deamination of methylated cytosines in a CpG context. In contrast, the PIK3CA oncogene was frequently mutated in both tumor types, but was more often the target of APOBEC-mediated deamination in the HPV-active tumors. Finally, the cancer driver genes TP53, CTNNB1, PTEN, and ARIDs were far more likely to be mutated in HPV-inactive tumors. In total, these clinical and molecular phenotypes strongly indicate that a subset of cervical cancer exists that is independent of HPV oncogene activity.

Personalized oncology promises to deliver therapies tailored to the genetic features of an individual's tumor, to improve outcome. Cervical cancers are usually treated with a combined regimen of platinum-based chemotherapy and radiation; however, few biomarkers of response to targeted therapies are available in this disease type. The differences between HPV-inactive and HPV-active tumors suggest the use of different targeted therapeutic approaches. For example, gefitinib, an EGF-receptor inhibitor, may be more effective in treating HPV-active cervical cancers, while dasatinib may be more effective in treating HPV-inactive tumors. Overall, somatic mutations in the genes upstream of AKT are more common in HPV-inactive tumors, suggesting that dual PI3K/MTOR inhibitors may be more effective for these patients. Finally, inflammation-associated gene sets are increased in HPV-active tumors, as are the targets for checkpoint inhibitor based immunotherapy such as, TIGIT, CTLA4, PDL-1 [21–23], indicating that immunotherapy, in combination with standard radiotherapy and DNA-damaging chemotherapy protocols may also be more effective in treating HPV-active tumors.

Most HPV infections do not develop into cervical cancer, but rather resolve within one or two years [24, 25]. Studies of immunosuppressed women have revealed higher incidences of cervical dysplasia, neoplasia, and cancers than in immunocompetent individuals, highlighting the role that the immune system plays in clearing HPV-infected cells [26, 27]. The small fraction of HPV infections that ultimately give rise to cervical cancer may do so only in individuals with genetic variants that attenuate some components of the immune response to HPV needed for clearance [28]. Our results suggest that, among the population of women who are immune surveillance competent, a small percentage of HPV-infected cells may acquire somatic mutations in cancer driver genes, rendering the cells ultimately independent of the expression of HPV, and allowing sub-clones to arise that become HPV-inactive tumors. Thus, women with HPV-inactive tumors may have a genetic background that is more similar to the majority of women who readily clear HPV infections. Careful analysis of the genomes of women without and with persistent HPV infection may reveal important cervical cancer risk alleles, and ultimately provide additional clues as to the origin and development of HPV-inactive cancers.

## MATERIALS AND METHODS

### Samples analyzed

All sequencing data were downloaded from TCGA repository CGHUB. We obtained TCGA CESC whole exome sequence data from tumors and matched normal samples that were assembled to the GrCH37-lite-+-HPV_Redux reference sequence. Tumor and matched normal BAM files, and RNA-seq FASTQ files that were available on February 10, 2015 at Cancer Genomics Hub (CGHub) were used. Access to this level data was approved by the National Cancer Institute. Normalized, mean centered DNA methylation data for the same samples were obtained from TCGA data portal.

### Statistical analysis of clinical data

Clinical information on the CESC samples analyzed were downloaded from the TCGA data portal (https://tcga-data.nci.nih.gov/tcga/). Statistical analyses presented in Table 2 were calculated using the Chi Square Test (R by C Table, OpenEpi, http://www.openepi.com/), unknown samples were not included in the test for significance. The log rank Mantel-Cox survival model was used to compare the age of disease-onset between the two classes (PRISM v6.0g). The Gehan-Breslow-Wilcoxon survival model was used to compare overall survival (PRISM v6.0g).

### Bioinformatics of DNA sequence analysis

We created a custom reference by concatenating hg19 human reference genome, and 18 human papillomavirus whole genome sequences as listed on Table 1. We extracted forward and reverse paired-end FASTQ reads from all tumor and normal BAM files using bam2fastq (https://gsl.hudsonalpha.org/information/software/bam2fastq). Reads were imported into CLC Genomics Workbench 8 and assembled to our custom reference sequence (hg19+HPV) with the following parameters: References = Genome (Genome), Masking mode = No masking, Mismatch cost = 2, Cost of insertions and deletions = Linear gap cost, Insertion cost = 3, Deletion cost = 3, Insertion open cost = 6, Insertion extend cost = 1, Deletion open cost = 6, Deletion extend cost = 1, Length fraction = 0.5, Similarity fraction = 0.8, Global alignment = No, Auto-detect paired distances = Yes, Non-specific match handling = Ignore, Output mode = Create reads track, Create report = Yes, Collect un-mapped reads = No.

HPV typing was determined by inspecting the number of normalized reads mapping to each HPV reference. HPV-reads were normalized by dividing the raw number of HPV mapping reads by the raw number of all mapped reads (viral and human) to control for the depth of sequencing for the different tumor samples. Resulting normalized reads were scaled down to the sample with the lowest depth of coverage (TCGA-MA-AA41, 38,688,500). The threshold for calling a specific HPV type was set at a normalized read count greater than 50 for that HPV type. Tumor variants were called using the following setting in CLC Genomics Workbench 8: Required significance (%) = 1.0, Ignore positions with coverage above = 100,000, Restrict calling to target regions = Not set, Ignore broken pairs = No, Ignore non-specific matches = No, Minimum coverage = 10, Minimum count = 2, Minimum frequency (%) = 1.0, Base quality filter = Yes, Neighborhood radius = 5, Minimum central quality = 20, Minimum neighborhood quality = 15, Read direction filter = No, Relative read direction filter = Yes, Significance (%) = 1.0, Read position filter = No, Remove pyro-error variants = No, Create track = Yes, Create annotated table = Yes, Create report = Yes.

To identify somatic variants we filtered each tumor's variants calls against the patient matched normal BAM file. Any variant appearing in 2 or more reads in the normal BAM file and covered by more than 20 reads was considered a germline variant and removed. The remaining variants were filtered for overlap with the coding sequence. Amino acid changes were identified using the standard codon table. We filtered marginal variant calls based on the following parameters: Minimum frequency (%) = 30.0, Minimum forward/reverse balance = 0.05, Minimum average base quality = 20.0, Variant frequency = Yes, Forward/reverse balance = Yes, Average base quality = Yes. Next, we annotated all variants with identical matches in the dbSNP, 1000 genomes phase 3, exome variant server (ESP6500), or COSMIC databases. We annotated all variants with overlapping gene name information. The initial germline variants filter failed to identify all germline variants due to regions of low coverage in the matched normal. The remaining germline variants were removed by filtering against exact matches in the dbSNP, 1000 genomes phase 3 and ESP6500 databases.

### Bioinformatics of RNA sequence analysis

The unassembled FASTQ files from CGHub were imported into CLC Genomics Workbench 8 and assembled to our custom reference sequence (HG19+HPV) using the RNA-Seq Analysis tool with the following parameters: Reference type = Genome annotated with genes and transcripts, Reference sequence = Genome (Genome), Gene track = Genome (Gene), mRNA track = Genome (mRNA), Mapping type = Also map to inter-genic regions, Mismatch cost = 2, Insertion cost = 3, Deletion cost = 3, Length fraction = 0.8, Similarity fraction = 0.8, Global alignment = No, Auto-detect paired distances = Yes, Strand specific = Both, Maximum number of hits for a read = 30, Count paired reads as two = No, Expression value = Total counts, Calculate RPKM for genes without transcripts = Yes, Minimum read count fusion gene table = 25, Create report = Yes, Create fusion gene table = Yes, Create list of unmapped reads = No. Gene expression levels were defined as the total number of reads assembling to the exons of the gene, normalized to the total number of reads that assembled to all exons of all genes.

### Gene expression data analysis

HPV classifier was determined using the normalized expression of all HPV reads against the sum of the HPV E6 and E7 oncogene reads. We utilized the unsupervised Partitioning Clustering tool in Partek Genomics Suite 6.5 to cluster the samples into two groups using the Euclidean distance function.

Differentially expressed human genes were identified using a one-way ANOVA and defined as greater than 4 fold different in either direction and FDR corrected *p* < 0.05. All genes were rank ordered by the *t*-test statistic and used to perform gene set enrichment analysis [8, 29].

### DNA methylation data analysis

We downloaded CESC Illumina 450k array mean-centered beta values from TCGA data portal (https://tcga-data.nci.nih.gov/tcga/). Differentially methylated human loci were identified using a one-way ANOVA and defined as Bonferroni-corrected *p*-value < 0.05.

### DNA mutation data analysis

Non-synonymous to synonymous ratios were compared using the Chi Squared test (OpenEpi, http://www.openepi.com/). Somatic mutation landscapes were compared between multiple tumor cohorts using variant calls and MutSig scores from firebrowse.org. We manually divided the samples in the CESC cohort by HPV expression class and ran MutSig CV0.9 (https://www.broadinstitute.org/cancer/cga/mutsig). The *p*-values were minus log p transformed and used to drive the unsupervised hierarchical clustering (Pearson dissimilarity, complete linkage) in Partek Genomics Suite 6.5.

The per gene somatic mutation frequencies (Figure 6) were compared between classes using a two-tailed Fisher's Exact *p*-value. The corresponding conditional maximum likelihood estimate of odds ratio and 95% CI (OpenEpi, http://www.openepi.com/ ) are plotted on Figure 6.

## SUPPLEMENTARY MATERIALS FIGURES AND TABLES



















## References

[1] Walboomers JM, Jacobs MV, Manos MM, Bosch FX, Kummer JA, Shah KV, Snijders PJ, Peto J, Meijer CJ, Munoz N (1999). Human papillomavirus is a necessary cause of invasive cervical cancer worldwide. J Pathol.

[2] Ho GY, Bierman R, Beardsley L, Chang CJ, Burk RD (1998). Natural history of cervicovaginal papillomavirus infection in young women. N Engl J Med.

[3] Akagi K, Li J, Broutian TR, Padilla-Nash H, Xiao W, Jiang B, Rocco JW, Teknos TN, Kumar B, Wangsa D, He D, Ried T, Symer DE (2014). Genome-wide analysis of HPV integration in human cancers reveals recurrent, focal genomic instability. Genome Res.

[4] Ojesina AI, Lichtenstein L, Freeman SS, Pedamallu CS, Imaz-Rosshandler I, Pugh TJ, Cherniack AD, Ambrogio L, Cibulskis K, Bertelsen B, Romero-Cordoba S, Trevino V, Vazquez-Santillan K (2014). Landscape of genomic alterations in cervical carcinomas. Nature.

[5] Tang KW, Alaei-Mahabadi B, Samuelsson T, Lindh M, Larsson E (2013). The landscape of viral expression and host gene fusion and adaptation in human cancer. Nat Commun.

[6] Rusan M, Li YY, Hammerman PS (2015). Genomic landscape of human papillomavirus-associated cancers. Clin Cancer Res.

[7] Tomar S, Graves CA, Altomare D, Kowli S, Kassler S, Sutkowski N, Gillespie MB, Creek KE, Pirisi L (2016). Human papillomavirus status and gene expression profiles of oropharyngeal and oral cancers from European American and African American patients. Head Neck.

[8] Subramanian A, Tamayo P, Mootha VK, Mukherjee S, Ebert BL, Gillette MA, Paulovich A, Pomeroy SL, Golub TR, Lander ES, Mesirov JP (2005). Gene set enrichment analysis: a knowledge-based approach for interpreting genome-wide expression profiles. Proc Natl Acad Sci USA.

[9] Suzuki H, Yamamoto E, Maruyama R, Niinuma T, Kai M (2014). Biological significance of the CpG island methylator phenotype. Biochem Biophys Res Commun.

[10] Stephens P, Edkins S, Davies H, Greenman C, Cox C, Hunter C, Bignell G, Teague J, Smith R, Stevens C, O'Meara S, Parker A, Tarpey P (2005). A screen of the complete protein kinase gene family identifies diverse patterns of somatic mutations in human breast cancer. Nat Genet.

[11] Alexandrov LB, Nik-Zainal S, Wedge DC, Campbell PJ, Stratton MR (2013). Deciphering signatures of mutational processes operative in human cancer. Cell Rep.

[12] Wood LD, Parsons DW, Jones S, Lin J, Sjoblom T, Leary RJ, Shen D, Boca SM, Barber T, Ptak J, Silliman N, Szabo S, Dezso Z (2007). The genomic landscapes of human breast and colorectal cancers. Science.

[13] Pfeifer GP (2000). p53 mutational spectra and the role of methylated CpG sequences. Mutat Res.

[14] Alexandrov LB (2015). Understanding the origins of human cancer. Science.

[15] Vartanian JP, Guetard D, Henry M, Wain-Hobson S (2008). Evidence for editing of human papillomavirus DNA by APOBEC3 in benign and precancerous lesions. Science.

[16] Henderson S, Chakravarthy A, Su X, Boshoff C, Fenton TR (2014). APOBEC-mediated cytosine deamination links PIK3CA helical domain mutations to human papillomavirus-driven tumor development. Cell Rep.

[17] Lawrence MS, Stojanov P, Polak P, Kryukov GV, Cibulskis K, Sivachenko A, Carter SL, Stewart C, Mermel CH, Roberts SA, Kiezun A, Hammerman PS, McKenna A (2013). Mutational heterogeneity in cancer and the search for new cancer-associated genes. Nature.

[18] Allo G, Bernardini MQ, Wu RC, M Shih Ie, Kalloger S, Pollett A, Gilks CB, Clarke BA (2014). ARID1A loss correlates with mismatch repair deficiency and intact p53 expression in high-grade endometrial carcinomas. Mod Pathol.

[19] Chappell WH, Steelman LS, Long JM, Kempf RC, Abrams SL, Franklin RA, Basecke J, Stivala F, Donia M, Fagone P, Malaponte G, Mazzarino MC, Nicoletti F (2011). Ras/Raf/MEK/ERK and PI3K/PTEN/Akt/mTOR inhibitors: rationale and importance to inhibiting these pathways in human health. Oncotarget.

[20] Slebos RJ, Yi Y, Ely K, Carter J, Evjen A, Zhang X, Shyr Y, Murphy BM, Cmelak AJ, Burkey BB, Netterville JL, Levy S, Yarbrough WG (2006). Gene expression differences associated with human papillomavirus status in head and neck squamous cell carcinoma. Clin Cancer Res.

[21] Johnston RJ, Yu X, Grogan JL (2015). The checkpoint inhibitor TIGIT limits antitumor and antiviral CD8+ T cell responses. Oncoimmunology.

[22] Rice AE, Latchman YE, Balint JP, Lee JH, Gabitzsch ES, Jones FR (2015). An HPV-E6/E7 immunotherapy plus PD-1 checkpoint inhibition results in tumor regression and reduction in PD-L1 expression. Cancer Gene Ther.

[23] Bartkowiak T, Singh S, Yang G, Galvan G, Haria D, Ai M, Allison JP, Sastry KJ, Curran MA (2015). Unique potential of 4-1BB agonist antibody to promote durable regression of HPV+ tumors when combined with an E6/E7 peptide vaccine. Proc Natl Acad Sci USA.

[24] Banister CE, Messersmith AR, Cai B, Spiryda LB, Glover SH, Pirisi L, Creek KE (2015). Disparity in the persistence of high-risk human papillomavirus genotypes between African American and European American women of college age. J Infect Dis.

[25] Sycuro LK, Xi LF, Hughes JP, Feng Q, Winer RL, Lee SK, O'Reilly S, Kiviat NB, Koutsky LA (2008). Persistence of genital human papillomavirus infection in a long-term follow-up study of female university students. J Infect Dis.

[26] Brickman C, Palefsky JM (2015). Human papillomavirus in the HIV-infected host: epidemiology and pathogenesis in the antiretroviral era. Curr HIV/AIDS Rep.

[27] Zhang L, Wu J, Ling MT, Zhao L, Zhao KN (2015). The role of the PI3K/Akt/mTOR signalling pathway in human cancers induced by infection with human papillomaviruses. Mol Cancer.

[28] Chen D, Enroth S, Ivansson E, Gyllensten U (2014). Pathway analysis of cervical cancer genome-wide association study highlights the MHC region and pathways involved in response to infection. Hum Mol Genet.

[29] Mootha VK, Lindgren CM, Eriksson KF, Subramanian A, Sihag S, Lehar J, Puigserver P, Carlsson E, Ridderstrale M, Laurila E, Houstis N, Daly MJ, Patterson N (2003). PGC-1alpha-responsive genes involved in oxidative phosphorylation are coordinately downregulated in human diabetes. Nat Genet.

