# Supplementary material for: Identification and characterization of HPV-independent cervical cancers
Source: Oncotarget. 2017 Jan 6;8(8):13375–86. doi: 10.18632/oncotarget.14533 (PMC5355105; doi:10.18632/oncotarget.14533)
Supplement: Supplementary file 1 [file oncotarget-08-13375-s001.pdf]

Identification and characterization of HPV-independent cervical cancers

Supplementary Materials

Supplementary Table 1: DNA Normalized HPV counts. See Supplementary_Table_1

Supplementary Table 2A: RNAseq normalized gene expression. See Supplementary_Table_2

Supplementary Table 2B: GSEA hallmarks gene sets. See Supplementary_Table_2B

Supplementary Table 2C: GSEA c2.cgp gene sets. See Supplementary_Table_2C

Supplementary Table 3: Clinical information. See Supplementary_Table_3

Supplementary Table 4: RNAseq data ANOVA. See Supplementary_Table_4

Supplementary Table 5: Methylation ANOVA short list. See Supplementary_Table_5

Supplementary Table 6: Genes methylated and silenced In HPV-inactive cervical cancers. See Supplementary_Table_6
