# Supplementary material for: Identification and characterization of HPV-independent cervical cancers
Source: Oncotarget. 2017 Jan 6;8(8):13375–86. doi: 10.18632/oncotarget.14533 (PMC5355105; doi:10.18632/oncotarget.14533)
Supplement: Supplementary file 2 [file oncotarget-08-13375-s002.docx]

Supplemental Table1. DNA Normalized HPV counts
patientID CESC_Tumor_DNA_Bamfile_ID HPV_express SumOfAllBamReads SumOfAllHPVReads HPV_16_RawCount HPV_18_RawCount HPV_31_RawCount HPV_33_RawCount HPV_35_RawCount HPV_39_RawCount HPV_45_RawCount HPV_51_RawCount HPV_52_RawCount HPV_53_RawCount HPV_56_RawCount HPV_58_RawCount HPV_59_RawCount HPV_66_RawCount HPV_68_RawCount HPV_72_RawCount HPV_73_RawCount AllHPVNormalizedReads HPV_16normalized HPV_18normalized HPV_31normalized HPV_33normalized HPV_35normalized HPV_39normalized HPV_45normalized HPV_51normalized HPV_52normalized HPV_53normalized HPV_56normalized HPV_58normalized HPV_59normalized HPV_66normalized HPV_68normalized_reads HPV_72normalized HPV_73normalized HPV_16_50positive HPV_18_50positive HPV_31_50positive HPV_33_50positive HPV_35_50positive HPV_39_50positive HPV_45_50positive HPV_51_50positive HPV_52_50positive HPV_53_50positive HPV_56_50positive HPV_58_50positive HPV_59_50positive HPV_66_50positive HPV_68_50positive HPV_72_50positive HPV_73_50positive TotalNumberHPV
TCGA-2W-A8YY 191e1f11c562128d1f2afb31f50e73b5 0 62179100 14 1 0 0 0 0 0 0 0 13 0 0 0 0 0 0 0 0 8.71 0.62 0 0 0 0 0 0 0 8.09 0 0 0 0 0 0 0 0 0 0 0 0 0 0 0 0 0 0 0 0 0 0 0 0 0 0
TCGA-4J-AA1J e67c78313b2bf9fa2f8ad9a664e45cda 1 85494200 6253077 7 6252800 1 3 27 8 216 1 0 0 0 0 8 1 5 0 0 2829691.01 3.17 2829565.66 0.45 1.36 12.22 3.62 *97.7459991438016 0.45 0 0 0 0 3.62 0.45 2.26 0 0 0 1 0 0 0 0 0 0 0 0 0 0 0 0 0 0 0 1
TCGA-C5-A1BQ f071077aa48a2da2216f8ee1aadf2bfd 1 119019000 4374789 58871 7733 4307940 3 12 0 4 1 1 0 0 223 1 0 0 0 0 1422075.67 19136.7 2513.7 1400345.63 0.98 3.9 0 1.3 0.33 0.33 0 0 72.49 0.33 0 0 0 0 1 1 1 0 0 0 0 0 0 0 0 1 0 0 0 0 0 4
TCGA-C5-A2LS bc852ce57cf1c7fdd9124d25afbf2bd4 1 69583000 276613 276606 0 3 0 0 0 0 0 2 0 0 0 1 0 1 0 0 153798.23 153794.34 0 1.67 0 0 0 0 0 1.11 0 0 0 0.56 0 0.56 0 0 1 0 0 0 0 0 0 0 0 0 0 0 0 0 0 0 0 1
TCGA-C5-A2LT 10c8aba03b79a473b049e2771a5f3a0f 1 68442400 37446 26220 221 45 1 1 4322 438 44 0 87 36 0 467 129 5355 0 80 21167.14 14821.4 *124.924878437927 25.44 0.57 0.57 *2443.10101632906 *247.588673103223 24.87 0 49.18 20.35 0 *263.981530454806 *72.9199516673875 *3027.02590061132 0 45.22 1 0 0 0 0 0 0 0 0 0 0 0 0 0 0 0 0 1
TCGA-C5-A2LV 568ca2acff10814c04ea071c0203e6d4 1 66659900 55237 55221 0 1 0 1 10 0 0 0 0 0 0 1 0 3 0 0 32058.8 32049.52 0 0.58 0 0.58 5.8 0 0 0 0 0 0 0.58 0 1.74 0 0 1 0 0 0 0 0 0 0 0 0 0 0 0 0 0 0 0 1
TCGA-C5-A2LX f0e926c6babf60f73aaa986b525e7402 1 82101200 10459371 10459300 4 16 6 13 9 2 0 4 0 3 3 2 1 8 0 0 4928763.22 4928729.76 1.88 7.54 2.83 6.13 4.24 0.94 0 1.88 0 1.41 1.41 0.94 0.47 3.77 0 0 1 0 0 0 0 0 0 0 0 0 0 0 0 0 0 0 0 1
TCGA-C5-A2LY 8cdf8451fea140219064e854091dc3ba 1 79291600 119121 119100 0 3 0 0 6 1 0 0 0 0 1 0 0 10 0 0 58122.33 58112.09 0 1.46 0 0 2.93 0.49 0 0 0 0 0.49 0 0 4.88 0 0 1 0 0 0 0 0 0 0 0 0 0 0 0 0 0 0 0 1
TCGA-C5-A2LZ 80866b62e505324b7f98146cd1d16037 1 167060000 1828023 1786360 12230 29103 1 9 0 0 1 3 0 0 314 0 0 2 0 0 423341.72 413693.22 2832.28 6739.8 0.23 2.08 0 0 0.23 0.69 0 0 72.72 0 0 0.46 0 0 1 1 1 0 0 0 0 0 0 0 0 1 0 0 0 0 0 4
TCGA-C5-A2M1 98fc2f7cb337eb55aa2a74d33b736b84 1 86965600 2216453 2216420 0 4 1 3 9 1 0 1 0 1 2 0 0 10 0 1 986036.34 986021.66 0 1.78 0.44 1.33 4 0.44 0 0.44 0 0.44 0.89 0 0 4.45 0 0.44 1 0 0 0 0 0 0 0 0 0 0 0 0 0 0 0 0 1
TCGA-C5-A2M2 ad170d2c078f01cacaa9f6a093cd9259 1 60863000 432585 412909 123 0 7 1 0 0 0 1 0 0 19544 0 0 0 0 0 274979.29 262471.94 78.19 0 4.45 0.64 0 0 0 0.64 0 0 12423.44 0 0 0 0 0 1 1 0 0 0 0 0 0 0 0 0 1 0 0 0 0 0 3
TCGA-C5-A3HD 446f35445214dc5665bee94ca731faca 1 150714000 4200209 4170450 8445 21077 1 6 1 5 0 4 0 0 215 2 1 2 0 0 1078199.68 1070560.5 2167.84 5410.5 0.26 1.54 0.26 1.28 0 1.03 0 0 55.19 0.51 0.26 0.51 0 0 1 1 1 0 0 0 0 0 0 0 0 1 0 0 0 0 0 4
TCGA-C5-A3HE d02f9be36c0343cbaf2178b16b0cd5e9 1 80185000 6223426 5087 6217900 2 2 0 8 401 1 0 0 1 1 10 0 12 1 0 3002743.86 2454.43 3000077.62 0.96 0.96 0 3.86 *193.478686786805 0.48 0 0 0.48 0.48 4.82 0 5.79 0.48 0 1 1 0 0 0 0 0 0 0 0 0 0 0 0 0 0 0 2
TCGA-C5-A3HF a6f9b4ac751667817f9547da4444cc61 1 140537000 1583029 30113 1495550 22438 0 0 2 86 2 0 0 0 34822 6 0 5 5 0 435792.83 8289.82 411710.7 6176.97 0 0 0.55 23.67 0.55 0 0 0 9586.17 1.65 0 1.38 1.38 0 1 1 1 0 0 0 0 0 0 0 0 1 0 0 0 0 0 4
TCGA-C5-A3HL acb04fe701d68b14d4b7cf4da6fd707e 1 124361000 1003283 361314 7249 634510 1 1 0 1 0 1 0 0 202 2 0 1 0 1 312119.67 112404.18 2255.15 197395 0.31 0.31 0 0.31 0 0.31 0 0 62.84 0.62 0 0.31 0 0.31 1 1 1 0 0 0 0 0 0 0 0 1 0 0 0 0 0 4
TCGA-C5-A7CG 970aa77d058b34f6993256b8d659ec34 1 57925200 7207 23 0 0 0 0 0 0 0 7184 0 0 0 0 0 0 0 0 4813.59 15.36 0 0 0 0 0 0 0 4798.23 0 0 0 0 0 0 0 0 0 0 0 0 0 0 0 0 1 0 0 0 0 0 0 0 0 1
TCGA-C5-A7CH 3a4780910e6b0fba7d8a783c63b0d3af 1 73096500 527874 527867 0 1 1 2 0 0 0 2 0 0 0 0 0 1 0 0 279393.04 279389.33 0 0.53 0.53 1.06 0 0 0 1.06 0 0 0 0 0 0.53 0 0 1 0 0 0 0 0 0 0 0 0 0 0 0 0 0 0 0 1
TCGA-C5-A7CJ cbb7d920c8a4b85b114ac8a888501e12 1 77765800 63191 63189 0 0 1 0 1 0 0 0 0 0 0 0 0 0 0 0 31437.53 31436.54 0 0 0.5 0 0.5 0 0 0 0 0 0 0 0 0 0 0 1 0 0 0 0 0 0 0 0 0 0 0 0 0 0 0 0 1
TCGA-C5-A7CK 5004b48690f50d5f7f038f247c9870cb 1 61169800 21210 21210 0 0 0 0 0 0 0 0 0 0 0 0 0 0 0 0 13414.84 13414.84 0 0 0 0 0 0 0 0 0 0 0 0 0 0 0 0 1 0 0 0 0 0 0 0 0 0 0 0 0 0 0 0 0 1
TCGA-C5-A7CL 4ce472f2e5dd51db52c5ac873d437e5c 1 52972500 34767 34767 0 0 0 0 0 0 0 0 0 0 0 0 0 0 0 0 25392.1 25392.1 0 0 0 0 0 0 0 0 0 0 0 0 0 0 0 0 1 0 0 0 0 0 0 0 0 0 0 0 0 0 0 0 0 1
TCGA-C5-A7CM 58af223cf4737372cca2914464d14d1e 1 68923800 70196 0 69869 0 0 0 0 327 0 0 0 0 0 0 0 0 0 0 39402.61 0 39219.06 0 0 0 0 *183.552553689727 0 0 0 0 0 0 0 0 0 0 0 1 0 0 0 0 0 0 0 0 0 0 0 0 0 0 0 1
TCGA-C5-A7CO 86f489add351f6226dd55936f25d64c2 1 68414200 199422 132 0 199287 0 2 0 0 0 0 0 0 1 0 0 0 0 0 112773.93 *74.6465207515399 0 112697.58 0 1.13 0 0 0 0 0 0 0.57 0 0 0 0 0 0 0 1 0 0 0 0 0 0 0 0 0 0 0 0 0 0 1
TCGA-C5-A7UC c8a5171162e0ac75e7be8312956393eb 1 109097000 10883 47 1 0 0 0 0 10833 0 0 0 0 1 1 0 0 0 0 3859.38 16.67 0.35 0 0 0 0 3841.65 0 0 0 0 0.35 0.35 0 0 0 0 0 0 0 0 0 0 1 0 0 0 0 0 0 0 0 0 0 1
TCGA-C5-A7UE 8389d504bf7c5afc147fee8adbc5c875 1 75132200 35095 35093 1 0 0 0 0 0 0 0 0 0 0 0 0 1 0 0 18071.78 18070.75 0.51 0 0 0 0 0 0 0 0 0 0 0 0 0.51 0 0 1 0 0 0 0 0 0 0 0 0 0 0 0 0 0 0 0 1
TCGA-C5-A7UH d403f4842fb79683464b18379bfa09b3 1 56157000 1975904 1975850 0 22 1 9 1 0 1 14 0 5 0 0 1 0 0 0 1361268.62 1361231.42 0 15.16 0.69 6.2 0.69 0 0.69 9.65 0 3.44 0 0 0.69 0 0 0 1 0 0 0 0 0 0 0 0 0 0 0 0 0 0 0 0 1
TCGA-C5-A7UI f2ca47fdccc2a91d326c340a1d3da4ee 1 59446300 27108 37 0 0 27070 0 0 0 0 0 0 0 1 0 0 0 0 0 17642.27 24.08 0 0 17617.54 0 0 0 0 0 0 0 0.65 0 0 0 0 0 0 0 0 1 0 0 0 0 0 0 0 0 0 0 0 0 0 1
TCGA-C5-A7X3 250c3eef8d7c52a45e6483758ce53247 1 66287000 46209 34 1 0 0 0 0 46174 0 0 0 0 0 0 0 0 0 0 26969.95 19.84 0.58 0 0 0 0 26949.52 0 0 0 0 0 0 0 0 0 0 0 0 0 0 0 0 1 0 0 0 0 0 0 0 0 0 0 1
TCGA-C5-A7X5 72b67922a0f259e44c69a41606cb143f 1 80428200 92283 7 1 862 0 91413 0 0 0 0 0 0 0 0 0 0 0 0 44391.03 3.37 0.48 *414.649177775954 0 43972.54 0 0 0 0 0 0 0 0 0 0 0 0 0 0 0 0 1 0 0 0 0 0 0 0 0 0 0 0 0 1
TCGA-C5-A7X8 7d2c1987ff0caa2e8a41cd3fe8a60511 1 102615000 65612 10 65597 0 0 1 0 4 0 0 0 0 0 0 0 0 0 0 24737.42 3.77 24731.76 0 0 0.38 0 1.51 0 0 0 0 0 0 0 0 0 0 0 1 0 0 0 0 0 0 0 0 0 0 0 0 0 0 0 1
TCGA-C5-A7XC faf0f8178c3c4154121f9c673ce81eb7 1 69437900 44030 44024 0 2 1 1 0 0 0 0 0 0 1 0 0 0 0 1 24532.06 24528.72 0 1.11 0.56 0.56 0 0 0 0 0 0 0.56 0 0 0 0 0.56 1 0 0 0 0 0 0 0 0 0 0 0 0 0 0 0 0 1
TCGA-C5-A8XH 2d4b4e590f0423cda9c91ca7440f72ec 1 56959600 93507 93496 0 0 0 0 0 0 0 11 0 0 0 0 0 0 0 0 63512.48 63505.01 0 0 0 0 0 0 0 7.47 0 0 0 0 0 0 0 0 1 0 0 0 0 0 0 0 0 0 0 0 0 0 0 0 0 1
TCGA-C5-A8XI 9af27bf34b33b6775dbf0c19b79d4e85 1 75238500 502553 3 0 0 4 5 0 0 1 502536 1 0 2 0 0 1 0 0 258418.52 1.54 0 0 2.06 2.57 0 0 0.51 258409.78 0.51 0 1.03 0 0 0.51 0 0 0 0 0 0 0 0 0 0 1 0 0 0 0 0 0 0 0 1
TCGA-C5-A8XJ e6a413a2f618b70e20e4c59d5f2dba24 1 49151700 72701 73 0 0 72628 0 0 0 0 0 0 0 0 0 0 0 0 0 57224.73 *57.460077677883 0 0 57167.27 0 0 0 0 0 0 0 0 0 0 0 0 0 0 0 0 1 0 0 0 0 0 0 0 0 0 0 0 0 0 1
TCGA-C5-A8XK 76e45595077a11a28987dc1329821606 1 59952400 1284420 1284380 1 15 3 6 0 1 0 6 1 0 3 2 0 2 0 0 828862.28 828836.47 0.65 9.68 1.94 3.87 0 0.65 0 3.87 0.65 0 1.94 1.29 0 1.29 0 0 1 0 0 0 0 0 0 0 0 0 0 0 0 0 0 0 0 1
TCGA-C5-A8YQ 0a503198d349883516bc5d64a42ff281 0 45086300 35 34 0 0 1 0 0 0 0 0 0 0 0 0 0 0 0 0 30.03 29.18 0 0 0.86 0 0 0 0 0 0 0 0 0 0 0 0 0 0 0 0 0 0 0 0 0 0 0 0 0 0 0 0 0 0 0
TCGA-C5-A8YR 3f02733722fc08b708d447abf2aed9d9 1 52850200 31127 50 0 0 4 0 0 31068 0 0 0 4 0 1 0 0 0 0 22786.23 36.6 0 0 2.93 0 0 22743.04 0 0 0 2.93 0 0.73 0 0 0 0 0 0 0 0 0 0 1 0 0 0 0 0 0 0 0 0 0 1
TCGA-C5-A8YT f3976f6b1380b1d497efd9de5cec5420 0 65429400 210 88 0 0 4 0 0 118 0 0 0 0 0 0 0 0 0 0 124.17 52.03 0 0 2.37 0 0 69.77 0 0 0 0 0 0 0 0 0 0 1 0 0 0 0 0 1 0 0 0 0 0 0 0 0 0 0 2
TCGA-C5-A8ZZ a69ff34e138b2104cc8c91f35d0b362c 1 38977300 9894 9890 0 0 1 0 0 3 0 0 0 0 0 0 0 0 0 0 9820.69 9816.72 0 0 0.99 0 0 2.98 0 0 0 0 0 0 0 0 0 0 1 0 0 0 0 0 0 0 0 0 0 0 0 0 0 0 0 1
TCGA-C5-A901 324cd14376920d5ce28ef50f6f2334ef 1 59854800 10651 10650 0 0 0 0 0 1 0 0 0 0 0 0 0 0 0 0 6884.51 6883.87 0 0 0 0 0 0.65 0 0 0 0 0 0 0 0 0 0 1 0 0 0 0 0 0 0 0 0 0 0 0 0 0 0 0 1
TCGA-C5-A902 4b6b9deb3aff721b3937f193ee146b88 1 65163000 456544 456535 0 2 3 3 0 0 0 1 0 0 0 0 0 0 0 0 271058.77 271053.43 0 1.19 1.78 1.78 0 0 0 0.59 0 0 0 0 0 0 0 0 1 0 0 0 0 0 0 0 0 0 0 0 0 0 0 0 0 1
TCGA-C5-A905 8883274c325b7009ee6d3ba70c59024b 1 65146000 236275 236257 0 6 6 2 0 3 0 0 0 0 0 0 1 0 0 0 140317.52 140306.83 0 3.56 3.56 1.19 0 1.78 0 0 0 0 0 0 0.59 0 0 0 1 0 0 0 0 0 0 0 0 0 0 0 0 0 0 0 0 1
TCGA-C5-A907 de71cdba66a51708134d5f5a9537632c 1 48757100 17072 0 17071 0 0 0 0 1 0 0 0 0 0 0 0 0 0 0 13546.54 0 13545.75 0 0 0 0 0.79 0 0 0 0 0 0 0 0 0 0 0 1 0 0 0 0 0 0 0 0 0 0 0 0 0 0 0 1
TCGA-DG-A2KH 8894ceddbee870f11da6f2ebb9b53edf 1 78049500 314463 1356 313085 0 0 1 1 19 0 0 0 0 0 1 0 0 0 0 155876.74 672.16 155193.68 0 0 0.5 0.5 9.42 0 0 0 0 0 0.5 0 0 0 0 1 1 0 0 0 0 0 0 0 0 0 0 0 0 0 0 0 2
TCGA-DG-A2KJ ff3fb0e4c8af80ed9c012bf598bca00a 1 73501000 194573 1423 165919 0 5 1 0 5 0 4 0 0 27216 0 0 0 0 0 102416.8 749.02 87334.28 0 2.63 0.53 0 2.63 0 2.11 0 0 14325.6 0 0 0 0 0 1 1 0 0 0 0 0 0 0 0 0 1 0 0 0 0 0 3
TCGA-DG-A2KK e044330aad5fcf2021b72594c6dad66c 1 81201300 68587 68520 0 0 0 0 0 0 0 0 0 0 0 0 0 67 0 0 32678.39 32646.47 0 0 0 0 0 0 0 0 0 0 0 0 0 31.92 0 0 1 0 0 0 0 0 0 0 0 0 0 0 0 0 0 0 0 1
TCGA-DG-A2KL 1c856226c74b31ef9fd30e3c2c3c6f75 1 73662500 1525828 1525720 0 1 0 4 0 0 0 1 0 0 1 0 0 101 0 0 801384.65 801327.92 0 0.53 0 2.1 0 0 0 0.53 0 0 0.53 0 0 53.05 0 0 1 0 0 0 0 0 0 0 0 0 0 0 0 0 1 0 0 2
TCGA-DG-A2KM fff6bc2b436912285fccae2bae94e7d2 1 69451500 327372 327367 0 2 0 1 0 0 0 0 0 1 1 0 0 0 0 0 182365.13 182362.34 0 1.11 0 0.56 0 0 0 0 0 0.56 0.56 0 0 0 0 0 1 0 0 0 0 0 0 0 0 0 0 0 0 0 0 0 0 1
TCGA-DS-A3LQ b720be98b40721dbe6aa7e0e4d17cf36 0 74440800 13152 517 46 140 121 28 263 8098 1807 8 404 393 61 662 141 70 92 301 6835.38 *268.696124974476 23.91 *72.7610396449259 *62.886327121686 14.55 *136.686810190111 4208.71 *939.137133131293 4.16 *209.967571546786 *204.250632717542 31.7 *344.05577317815 *73.2807613566754 36.38 47.81 *156.436235236591 0 0 0 0 0 0 1 0 0 0 0 0 0 0 0 0 0 1
TCGA-DS-A5RQ 61e2e09b88005aef9df903319565d268 1 48691600 172063 171954 0 0 0 0 1 0 0 0 0 0 0 108 0 0 0 0 136714.74 136628.13 0 0 0 0 0.79 0 0 0 0 0 0 85.81 0 0 0 0 1 0 0 0 0 0 0 0 0 0 0 0 1 0 0 0 0 2
TCGA-DS-A7WF b73b473daaab277d422bbe1fcb71e5a5 1 75086900 1934047 1934000 0 13 2 10 1 1 0 11 0 1 3 3 2 0 0 0 996517.07 996492.85 0 6.7 1.03 5.15 0.52 0.52 0 5.67 0 0.52 1.55 1.55 1.03 0 0 0 1 0 0 0 0 0 0 0 0 0 0 0 0 0 0 0 0 1
TCGA-DS-A7WH 7e96cd032d90584f6f0f45b6829e4e97 1 54863000 11258 11256 0 1 0 1 0 0 0 0 0 0 0 0 0 0 0 0 7938.96 7937.55 0 0.71 0 0.71 0 0 0 0 0 0 0 0 0 0 0 0 1 0 0 0 0 0 0 0 0 0 0 0 0 0 0 0 0 1
TCGA-DS-A7WI 4911f64fb4e718be1d336c3bc17c1493 1 85313300 61206 61202 0 0 0 1 0 3 0 0 0 0 0 0 0 0 0 0 27756.15 27754.33 0 0 0 0.45 0 1.36 0 0 0 0 0 0 0 0 0 0 1 0 0 0 0 0 0 0 0 0 0 0 0 0 0 0 0 1
TCGA-EA-A1QS 9cb6bab021af7360ee2c1ad8a1f5e309 1 67851400 62696 0 133 0 0 0 62562 1 0 0 0 0 0 0 0 0 0 0 35748.92 0 75.84 0 0 0 35672.51 0.57 0 0 0 0 0 0 0 0 0 0 0 1 0 0 0 1 0 0 0 0 0 0 0 0 0 0 0 2
TCGA-EA-A3HQ 290d582ed431b174031b61e2fbe96009 1 109848000 5355768 5355680 1 16 3 11 1 1 1 1 0 0 1 1 0 1 0 50 1886303.17 1886272.17 0.35 5.64 1.06 3.87 0.35 0.35 0.35 0.35 0 0 0.35 0.35 0 0.35 0 17.61 1 0 0 0 0 0 0 0 0 0 0 0 0 0 0 0 0 1
TCGA-EA-A3HR dd44d253e61da96546b7c66ba228dc78 1 85478700 48161 16607 0 0 0 0 0 0 0 0 0 0 0 0 0 0 0 31554 21798.14 7516.49 0 0 0 0 0 0 0 0 0 0 0 0 0 0 0 14281.65 1 0 0 0 0 0 0 0 0 0 0 0 0 0 0 0 1 2
TCGA-EA-A3HS 770158cfc6013ac94fc9f8da9bccadec 1 84378500 1030451 1030380 0 0 2 2 0 0 0 1 0 0 2 0 0 0 0 64 472473.48 472440.93 0 0 0.92 0.92 0 0 0 0.46 0 0 0.92 0 0 0 0 29.34 1 0 0 0 0 0 0 0 0 0 0 0 0 0 0 0 0 1
TCGA-EA-A3HT b19f44f56c5c16f24dfec3fe7e41a98c 1 68105600 414000 413433 0 564 0 1 0 0 0 2 0 0 0 0 0 0 0 0 235179.47 234857.38 0 320.39 0 0.57 0 0 0 1.14 0 0 0 0 0 0 0 0 1 0 1 0 0 0 0 0 0 0 0 0 0 0 0 0 0 2
TCGA-EA-A3HU f12a7b7251edfa076987f69fd8a15a55 1 72033700 2451523 2451440 1 4 2 5 0 1 1 0 1 1 0 1 0 0 0 66 1316685.77 1316641.19 0.54 2.15 1.07 2.69 0 0.54 0.54 0 0.54 0.54 0 0.54 0 0 0 35.45 1 0 0 0 0 0 0 0 0 0 0 0 0 0 0 0 0 1
TCGA-EA-A3QD 735c8442b96981e5c38215beb5112080 1 67972500 2642702 2642670 8 1 2 13 0 0 1 0 0 1 0 2 0 4 0 0 1504169.72 1504151.51 4.55 0.57 1.14 7.4 0 0 0.57 0 0 0.57 0 1.14 0 2.28 0 0 1 0 0 0 0 0 0 0 0 0 0 0 0 0 0 0 0 1
TCGA-EA-A3QE 949429624627d6c5ccc79d7e705e1c1e 1 62222400 405847 405386 0 458 1 0 1 0 0 0 0 1 0 0 0 0 0 0 252346.61 252059.97 0 284.77 0.62 0 0.62 0 0 0 0 0.62 0 0 0 0 0 0 1 0 1 0 0 0 0 0 0 0 0 0 0 0 0 0 0 2
TCGA-EA-A3Y4 4d04a789ce50c2c180748a053367dd4f 1 70921800 18572 1 0 0 0 0 1 18569 0 0 0 0 1 0 0 0 0 0 10131.2 0.55 0 0 0 0 0.55 10129.56 0 0 0 0 0.55 0 0 0 0 0 0 0 0 0 0 0 1 0 0 0 0 0 0 0 0 0 0 1
TCGA-EA-A410 9a6634616aaf1a4f289986b55cbb3ac2 0 67455100 33477 25021 8158 3 0 1 0 77 0 0 0 216 0 1 0 0 0 0 19200.55 14350.66 4678.98 1.72 0 0.57 0 44.16 0 0 0 123.89 0 0.57 0 0 0 0 1 1 0 0 0 0 0 0 0 0 1 0 0 0 0 0 0 3
TCGA-EA-A411 04ef90fa74a74829427e6a294f321d13 1 67894500 7614667 7607010 7350 25 5 15 3 57 1 1 0 188 3 2 2 1 1 3 4339085.56 4334722.35 4188.27 14.25 2.85 8.55 1.71 32.48 0.57 0.57 0 107.13 1.71 1.14 1.14 0.57 0.57 1.71 1 1 0 0 0 0 0 0 0 0 1 0 0 0 0 0 0 3
TCGA-EA-A439 4f42db906f5b41c390db77a9e2665696 1 71124300 4717901 25963 4691580 2 3 0 4 123 3 0 0 195 1 9 3 12 2 1 2566331.24 14122.73 2552013.77 1.09 1.63 0 2.18 66.91 1.63 0 0 106.07 0.54 4.9 1.63 6.53 1.09 0.54 1 1 0 0 0 0 1 0 0 0 1 0 0 0 0 0 0 4
TCGA-EA-A43B 7342659578b677706b3a29a6963eb35c 1 67771700 4712162 4702100 9663 12 4 12 0 106 0 0 0 260 1 0 0 4 0 0 2690008.95 2684264.9 5516.27 6.85 2.28 6.85 0 60.51 0 0 0 148.42 0.57 0 0 2.28 0 0 1 1 0 0 0 0 1 0 0 0 1 0 0 0 0 0 0 4
TCGA-EA-A44S f032036df8bd2e64ff18af19f019a214 1 61841900 50486 50485 0 0 0 0 0 0 0 0 0 0 0 0 0 1 0 0 31584.21 31583.59 0 0 0 0 0 0 0 0 0 0 0 0 0 0.63 0 0 1 0 0 0 0 0 0 0 0 0 0 0 0 0 0 0 0 1
TCGA-EA-A4BA e71b9cfb77d10bd244a537a2df55b1b8 0 54499900 5482 5481 0 0 0 0 0 1 0 0 0 0 0 0 0 0 0 0 3891.57 3890.86 0 0 0 0 0 0.71 0 0 0 0 0 0 0 0 0 0 1 0 0 0 0 0 0 0 0 0 0 0 0 0 0 0 0 1
TCGA-EA-A50E 8d7b032a63d02ab2359625a6e5aeaca3 1 60309700 9406 9406 0 0 0 0 0 0 0 0 0 0 0 0 0 0 0 0 6033.92 6033.92 0 0 0 0 0 0 0 0 0 0 0 0 0 0 0 0 1 0 0 0 0 0 0 0 0 0 0 0 0 0 0 0 0 1
TCGA-EA-A556 bd14966be2d361c4dab3d339da8b0ea2 0 84005000 4166 4165 0 0 0 0 0 0 0 0 1 0 0 0 0 0 0 0 1918.65 1918.19 0 0 0 0 0 0 0 0 0.46 0 0 0 0 0 0 0 1 0 0 0 0 0 0 0 0 0 0 0 0 0 0 0 0 1
TCGA-EA-A5FO 8ca85f98c6fb3551f2a6cbbb1428d06b 1 119929000 950757 950586 53 3 0 2 0 0 0 0 7 1 17 87 0 1 0 0 306709.49 306654.32 17.1 0.97 0 0.65 0 0 0 0 2.26 0.32 5.48 28.07 0 0.32 0 0 1 0 0 0 0 0 0 0 0 0 0 0 0 0 0 0 0 1
TCGA-EA-A5O9 972772b96b7ca7d405a7c6a2939665e8 1 57689300 608946 608854 22 0 0 2 0 0 0 0 9 2 7 47 3 0 0 0 408380.88 408319.19 14.75 0 0 1.34 0 0 0 0 6.04 1.34 4.69 31.52 2.01 0 0 0 1 0 0 0 0 0 0 0 0 0 0 0 0 0 0 0 0 1
TCGA-EA-A5ZD 5104268bec67e471523308029855cf7e 1 58206100 25321 2787 25 0 0 0 1 0 0 0 9 5 22459 33 2 0 0 0 16830.39 1852.47 16.62 0 0 0 0.66 0 0 0 5.98 3.32 14928.07 21.93 1.33 0 0 0 1 0 0 0 0 0 0 0 0 0 0 1 0 0 0 0 0 2
TCGA-EA-A5ZE 32abf93283596ca89616f2d4da149a95 1 71444400 49283 3281 34 0 0 0 0 0 0 1 3 4 16 45942 2 0 0 0 26687.68 1776.72 18.41 0 0 0 0 0 0 0.54 1.62 2.17 8.66 24878.47 1.08 0 0 0 1 0 0 0 0 0 0 0 0 0 0 0 1 0 0 0 0 2
TCGA-EA-A5ZF 33316aa3d72207fca24e7bdd0fb726e6 1 61411400 36215 2718 33437 0 0 0 0 0 0 0 4 0 13 39 4 0 0 0 22815.05 1712.31 21064.94 0 0 0 0 0 0 0 2.52 0 8.19 24.57 2.52 0 0 0 1 1 0 0 0 0 0 0 0 0 0 0 0 0 0 0 0 2
TCGA-EA-A6QX 88f8212f59e3298c9ab41f74b53f3b0d 1 73775400 308822 20 0 0 3 0 0 0 0 0 0 0 308799 0 0 0 0 0 161949.1 10.49 0 0 1.57 0 0 0 0 0 0 0 161937.04 0 0 0 0 0 0 0 0 0 0 0 0 0 0 0 0 1 0 0 0 0 0 1
TCGA-EA-A78R e241cffd2bdaf0f87aa4afb3eb695d41 1 61367100 11764 0 0 0 0 0 0 0 0 11764 0 0 0 0 0 0 0 0 7416.54 0 0 0 0 0 0 0 0 7416.54 0 0 0 0 0 0 0 0 0 0 0 0 0 0 0 0 1 0 0 0 0 0 0 0 0 1
TCGA-EA-A97N 2c104f047726df925f3c78498120769d 1 91841900 2373745 2373720 0 9 0 8 0 1 0 2 0 0 3 0 1 1 0 0 999942.66 999932.12 0 3.79 0 3.37 0 0.42 0 0.84 0 0 1.26 0 0.42 0.42 0 0 1 0 0 0 0 0 0 0 0 0 0 0 0 0 0 0 0 1
TCGA-EK-A2GZ 27cf2f92732fe848738cfb805c2d0b03 1 76656800 128579 1248 0 0 1 2 0 0 0 127328 0 0 0 0 0 0 0 0 64893.51 629.86 0 0 0.5 1.01 0 0 0 64262.13 0 0 0 0 0 0 0 0 1 0 0 0 0 0 0 0 1 0 0 0 0 0 0 0 0 2
TCGA-EK-A2H0 d926d42d2bbba23c6004d948cb132dbc 1 67474300 28084 8200 0 0 0 0 0 2 0 0 0 0 0 0 0 19882 0 0 16102.84 4701.73 0 0 0 0 0 1.15 0 0 0 0 0 0 0 11399.97 0 0 1 0 0 0 0 0 0 0 0 0 0 0 0 0 1 0 0 2
TCGA-EK-A2H1 b12ed5a0c4cf0634340d9ee13ad1b5b5 1 81106800 71100 71098 0 0 1 1 0 0 0 0 0 0 0 0 0 0 0 0 33915.19 33914.23 0 0 0.48 0.48 0 0 0 0 0 0 0 0 0 0 0 0 1 0 0 0 0 0 0 0 0 0 0 0 0 0 0 0 0 1
TCGA-EK-A2IP c1948ed7950cdc64cdb78ad9d75abe3e 1 74589600 258334 257698 0 0 0 1 0 1 0 626 0 0 0 0 8 0 0 0 133993.95 133664.06 0 0 0 0.52 0 0.52 0 324.7 0 0 0 0 4.15 0 0 0 1 0 0 0 0 0 0 0 1 0 0 0 0 0 0 0 0 2
TCGA-EK-A2PG 561f94fac36f7b09d9dd70290f9558ca 1 95724700 25505489 1872 216 15 1488 26 2 5 4 832 1 1 25501000 3 0 3 9 12 10308406.41 756.6 87.3 6.06 *601.396379408867 10.51 0.81 2.02 1.62 *336.264642250119 0.4 0.4 10306592.12 1.21 0 1.21 3.64 4.85 1 1 0 0 0 0 0 0 0 0 0 1 0 0 0 0 0 3
TCGA-EK-A2PI 20efd5a82fa9bb931ef431c1fd6aa4d4 1 69073100 589933 562722 153 2 7 1 0 1 0 2 0 1 27042 0 0 1 0 1 330427.08 315185.94 85.7 1.12 3.92 0.56 0 0.56 0 1.12 0 0.56 15146.48 0 0 0.56 0 0.56 1 1 0 0 0 0 0 0 0 0 0 1 0 0 0 0 0 3
TCGA-EK-A2PK 4ba0efd7e71851bf2cd0060a36895a41 1 67011700 41285 120 40958 0 0 0 108 4 0 84 0 0 11 0 0 0 0 0 23835.46 69.28 23646.67 0 0 0 62.35 2.31 0 48.5 0 0 6.35 0 0 0 0 0 1 1 0 0 0 1 0 0 0 0 0 0 0 0 0 0 0 3
TCGA-EK-A2PL 0e76da20852762ccc974b8df9295230e 1 72400600 85395 102 235 0 0 0 84952 1 0 95 0 0 10 0 0 0 0 0 45632.28 54.51 125.58 0 0 0 45395.56 0.53 0 50.76 0 0 5.34 0 0 0 0 0 1 1 0 0 0 1 0 0 1 0 0 0 0 0 0 0 0 4
TCGA-EK-A2PM 377c5d335435ade31ee00639b74f2905 0 71613500 482 78 194 0 0 0 98 1 0 92 0 0 19 0 0 0 0 0 260.4 42.14 104.81 0 0 0 52.94 0.54 0 49.7 0 0 10.26 0 0 0 0 0 0 1 0 0 0 1 0 0 0 0 0 0 0 0 0 0 0 2
TCGA-EK-A2R7 37d02d3e7193e64f59c3b441df19249d 1 65516400 113367 94 112335 0 0 0 126 694 0 107 0 0 11 0 0 0 0 0 66945.06 55.51 66335.64 0 0 0 74.41 *409.818289771721 0 63.19 0 0 6.5 0 0 0 0 0 1 1 0 0 0 1 0 0 1 0 0 0 0 0 0 0 0 4
TCGA-EK-A2R8 86bf3eb0b4e960f288e93544d598d981 1 77193700 235146 804 3 0 4057 0 1 230273 0 0 1 0 6 0 0 1 0 0 117852.18 402.95 1.5 0 2033.32 0 0.5 115409.9 0 0 0.5 0 3.01 0 0 0.5 0 0 1 0 0 1 0 0 1 0 0 0 0 0 0 0 0 0 0 3
TCGA-EK-A2R9 3c5a21da0edddbcd47c697592f40ba94 1 83547100 2749414 1033 1 1 2748070 0 0 292 0 5 0 0 12 0 0 0 0 0 1273182.47 478.36 0.46 0.46 1272560.1 0 0 135.22 0 2.32 0 0 5.56 0 0 0 0 0 1 0 0 1 0 0 1 0 0 0 0 0 0 0 0 0 0 3
TCGA-EK-A2RA 473031a5159239b496ca975a220fbd41 1 65670500 71034 82 207 0 1 0 125 1 0 70614 0 0 4 0 0 0 0 0 41848.3 48.31 121.95 0 0.59 0 73.64 0.59 0 41600.87 0 0 2.36 0 0 0 0 0 0 1 0 0 0 1 0 0 1 0 0 0 0 0 0 0 0 3
TCGA-EK-A2RB 329749d4af60c5e32c5d7e74d2e757f1 1 67561600 194752 190875 0 0 3609 0 0 265 0 0 0 1 2 0 0 0 0 0 111522.86 109302.73 0 0 2066.66 0 0 151.75 0 0 0 0.57 1.15 0 0 0 0 0 1 0 0 1 0 0 1 0 0 0 0 0 0 0 0 0 0 3
TCGA-EK-A2RC 627201cd597ef698d4b90ca7e3881bbf 1 74972400 28867 28533 118 0 0 0 0 0 0 0 0 0 2 214 0 0 0 0 14896.43 14724.07 60.89 0 0 0 0 0 0 0 0 0 1.03 110.43 0 0 0 0 1 1 0 0 0 0 0 0 0 0 0 0 1 0 0 0 0 3
TCGA-EK-A2RD e667d86f6e6edd00ae276de743811325 49748500 548280 548029 0 4 0 3 0 243 0 0 0 0 0 1 0 0 0 0 426387.34 426192.15 0 3.11 0 2.33 0 188.98 0 0 0 0 0 0.78 0 0 0 0 1 0 0 0 0 0 1 0 0 0 0 0 0 0 0 0 0 2
TCGA-EK-A2RE 68e077ee5e55c24b63f623c7590dd26a 1 70393700 205575 205264 101 0 2 0 0 1 0 0 0 0 0 207 0 0 0 0 112984.38 112813.45 55.51 0 1.1 0 0 0.55 0 0 0 0 0 113.77 0 0 0 0 1 1 0 0 0 0 0 0 0 0 0 0 1 0 0 0 0 3
TCGA-EK-A2RJ 889a1fa97c05824c916621c92b1ffb36 1 51444700 135232 4304 206 0 10 0 0 0 0 0 0 0 0 130712 0 0 0 0 101699.95 3236.78 154.92 0 7.52 0 0 0 0 0 0 0 0 98300.72 0 0 0 0 1 1 0 0 0 0 0 0 0 0 0 0 1 0 0 0 0 3
TCGA-EK-A2RK 93b883f5ac9ee78df800c56af53c57a1 1 61926300 67773 67345 190 0 0 0 112 1 0 119 0 0 6 0 0 0 0 0 42341.23 42073.84 118.7 0 0 0 69.97 0.62 0 74.35 0 0 3.75 0 0 0 0 0 1 1 0 0 0 1 0 0 1 0 0 0 0 0 0 0 0 4
TCGA-EK-A2RL 5db04494568d95a5c0bc4e382c4d5954 1 81512300 358741 355281 0 0 3157 0 0 302 0 0 0 0 1 0 0 0 0 0 170270.64 168628.4 0 0 1498.42 0 0 143.34 0 0 0 0 0.47 0 0 0 0 0 1 0 0 1 0 0 1 0 0 0 0 0 0 0 0 0 0 3
TCGA-EK-A2RM eddc943d62c9f457b039686c48afbd7a 1 78792900 114135 2513 111407 0 0 0 0 4 0 0 0 0 0 211 0 0 0 0 56042 1233.92 54702.51 0 0 0 0 1.96 0 0 0 0 0 103.6 0 0 0 0 1 1 0 0 0 0 0 0 0 0 0 0 1 0 0 0 0 3
TCGA-EK-A2RN 904e62aa0c337fcb865135f832c8eed8 1 55881800 309659 309107 51 423 1 1 0 0 0 0 0 0 76 0 0 0 0 0 214385.4 214003.24 35.31 292.85 0.69 0.69 0 0 0 0 0 0 52.62 0 0 0 0 0 1 0 1 0 0 0 0 0 0 0 0 1 0 0 0 0 0 3
TCGA-EK-A2RO 2e1cabf2aa171e09c6176e5f7480559c 1 77321600 79917 79647 80 0 2 0 0 0 0 0 0 0 0 188 0 0 0 0 39987.13 39852.03 40.03 0 1 0 0 0 0 0 0 0 0 94.07 0 0 0 0 1 0 0 0 0 0 0 0 0 0 0 0 1 0 0 0 0 2
TCGA-EK-A3GJ 589ef632efd56a3eb8bd671a8fd995d6 1 55562200 63908 4334 56 383 0 0 1 0 0 0 4 0 59130 0 0 0 0 0 44499.76 3017.81 38.99 266.69 0 0 0.7 0 0 0 2.79 0 41172.79 0 0 0 0 0 1 0 1 0 0 0 0 0 0 0 0 1 0 0 0 0 0 3
TCGA-EK-A3GK a513c8bb00c59cf527ef0e6226f78737 1 56087900 1984356 1983840 43 370 1 2 0 0 0 0 0 0 98 0 0 2 0 0 1368775.74 1368419.82 29.66 255.22 0.69 1.38 0 0 0 0 0 0 67.6 0 0 1.38 0 0 1 0 1 0 0 0 0 0 0 0 0 1 0 0 0 0 0 3
TCGA-EK-A3GM 71a06f94442d428c5924e119be90f02d 1 71067900 64281 1358 0 0 0 0 0 62923 0 0 0 0 0 0 0 0 0 0 34993.79 739.28 0 0 0 0 0 34254.52 0 0 0 0 0 0 0 0 0 0 1 0 0 0 0 0 1 0 0 0 0 0 0 0 0 0 0 2
TCGA-EK-A3GN ce23ab6e1031e220e267512426072332 1 59939000 708434 708136 0 0 0 1 0 297 0 0 0 0 0 0 0 0 0 0 457269.04 457076.69 0 0 0 0.65 0 191.7 0 0 0 0 0 0 0 0 0 0 1 0 0 0 0 0 1 0 0 0 0 0 0 0 0 0 0 2
TCGA-EX-A1H6 3722b3fb3b695e259a5d730c2eb80a49 1 74640500 28877 28876 0 0 0 0 0 0 0 0 0 0 1 0 0 0 0 0 14967.85 14967.33 0 0 0 0 0 0 0 0 0 0 0.52 0 0 0 0 0 1 0 0 0 0 0 0 0 0 0 0 0 0 0 0 0 0 1
TCGA-EX-A3L1 511b37bcb1e1cf5668c1e85b0a0fba96 1 63672800 8043 0 0 0 0 0 0 8043 0 0 0 0 0 0 0 0 0 0 4887.04 0 0 0 0 0 0 4887.04 0 0 0 0 0 0 0 0 0 0 0 0 0 0 0 0 1 0 0 0 0 0 0 0 0 0 0 1
TCGA-EX-A449 8a8f185b15a1d64bb4fdc6985f1e1780 1 68524600 396324 396209 107 3 0 4 0 0 0 0 0 0 0 0 0 0 0 1 223761.7 223696.77 60.41 1.69 0 2.26 0 0 0 0 0 0 0 0 0 0 0 0.56 1 1 0 0 0 0 0 0 0 0 0 0 0 0 0 0 0 2
TCGA-EX-A69L 7e55919974d507fc0f5acd112121815c 1 79366300 110020 109992 25 0 0 1 0 0 0 0 0 0 0 1 0 1 0 0 53631.19 53617.54 12.19 0 0 0.49 0 0 0 0 0 0 0 0.49 0 0.49 0 0 1 0 0 0 0 0 0 0 0 0 0 0 0 0 0 0 0 1
TCGA-EX-A69M ef2dae0714b09e1b3707f18ee1d55490 1 93486700 8205 13 32 0 0 0 0 0 0 8160 0 0 0 0 0 0 0 0 3395.55 5.38 13.24 0 0 0 0 0 0 3376.93 0 0 0 0 0 0 0 0 0 0 0 0 0 0 0 0 1 0 0 0 0 0 0 0 0 1
TCGA-EX-A8YF e987f58e7a43eb47f9e62f6dc47d01af 1 52325400 5478 1 5476 0 0 0 0 1 0 0 0 0 0 0 0 0 0 0 4050.34 0.74 4048.86 0 0 0 0 0.74 0 0 0 0 0 0 0 0 0 0 0 1 0 0 0 0 0 0 0 0 0 0 0 0 0 0 0 1
TCGA-FU-A2QG 1706f775b6ddf909be9892c6a9889057 1 86902900 2267521 2267130 146 2 3 2 0 0 0 0 0 1 1 236 0 0 0 0 1009482.84 1009308.77 65 0.89 1.34 0.89 0 0 0 0 0 0.45 0.45 105.07 0 0 0 0 1 1 0 0 0 0 0 0 0 0 0 0 1 0 0 0 0 3
TCGA-FU-A3EO 68b921f39e88c6a51c73c4e5574b10e9 1 62490900 275168 274919 0 3 0 0 0 242 0 2 0 0 2 0 0 0 0 0 170358.2 170204.04 0 1.86 0 0 0 149.82 0 1.24 0 0 1.24 0 0 0 0 0 1 0 0 0 0 0 1 0 0 0 0 0 0 0 0 0 0 2
TCGA-FU-A3HY 4df72e3ee9ffc4c72cf1d5af42b8a238 1 67669200 269328 269325 0 0 1 0 0 1 0 0 0 0 1 0 0 0 0 0 153982.85 153981.14 0 0 0.57 0 0 0.57 0 0 0 0 0.57 0 0 0 0 0 1 0 0 0 0 0 0 0 0 0 0 0 0 0 0 0 0 1
TCGA-FU-A3HZ 6fd7e112b560ed34950a8bc8dbfcf4cc 0 42896200 4885 4261 93 444 0 0 0 0 0 0 0 0 87 0 0 0 0 0 4405.83 3843.04 83.88 400.45 0 0 0 0 0 0 0 0 78.47 0 0 0 0 0 1 1 1 0 0 0 0 0 0 0 0 1 0 0 0 0 0 4
TCGA-FU-A3NI 660edfb2d63e66e58ecca66a204258ed 1 66613500 4556404 4556330 1 12 3 42 0 1 0 3 2 2 4 0 0 4 0 0 2646316.98 2646274 0.58 6.97 1.74 24.39 0 0.58 0 1.74 1.16 1.16 2.32 0 0 2.32 0 0 1 0 0 0 0 0 0 0 0 0 0 0 0 0 0 0 0 1
TCGA-FU-A3TQ d763d6a27e01993590abc524cf4a20b8 1 72431700 412923 386204 26712 0 0 1 0 5 0 1 0 0 0 0 0 0 0 0 220557.73 206286.11 14267.89 0 0 0.53 0 2.67 0 0.53 0 0 0 0 0 0 0 0 1 1 0 0 0 0 0 0 0 0 0 0 0 0 0 0 0 2
TCGA-FU-A3TX e2f63cea8d1081422440c71721801e5a 1 64713300 46606 47 46340 0 0 0 218 1 0 0 0 0 0 0 0 0 0 0 27863.15 28.1 27704.12 0 0 0 130.33 0.6 0 0 0 0 0 0 0 0 0 0 0 1 0 0 0 1 0 0 0 0 0 0 0 0 0 0 0 2
TCGA-FU-A3WB 024281e4ae144a27aeca510b2fb5bbba 1 76589900 101187 101186 0 1 0 0 0 0 0 0 0 0 0 0 0 0 0 0 51113.44 51112.93 0 0.51 0 0 0 0 0 0 0 0 0 0 0 0 0 0 1 0 0 0 0 0 0 0 0 0 0 0 0 0 0 0 0 1
TCGA-FU-A3YQ 13c5f9e05d7d7c9e9872d3d7ace85e12 1 67605200 44468 43357 1110 0 0 1 0 0 0 0 0 0 0 0 0 0 0 0 25447.75 24811.96 635.22 0 0 0.57 0 0 0 0 0 0 0 0 0 0 0 0 1 1 0 0 0 0 0 0 0 0 0 0 0 0 0 0 0 2
TCGA-FU-A40J 7842fe2c1082f850fed7d28c2fc68434 1 78719900 3471807 3471660 109 8 1 15 1 1 0 2 0 2 4 1 0 2 0 1 1706290.34 1706218.1 53.57 3.93 0.49 7.37 0.49 0.49 0 0.98 0 0.98 1.97 0.49 0 0.98 0 0.49 1 1 0 0 0 0 0 0 0 0 0 0 0 0 0 0 0 2
TCGA-FU-A57G a18fae2b151c5f22e63f5de7d8a20114 0 60275900 3658 3657 0 1 0 0 0 0 0 0 0 0 0 0 0 0 0 0 2347.91 2347.27 0 0.64 0 0 0 0 0 0 0 0 0 0 0 0 0 0 1 0 0 0 0 0 0 0 0 0 0 0 0 0 0 0 0 1
TCGA-FU-A5XV fa0c9ff54a61caa4f5735e440eda1cb8 1 77860300 40475 12620 27838 4 0 0 0 3 5 0 0 2 0 1 1 0 0 1 20111.88 6270.83 13832.6 1.99 0 0 0 1.49 2.48 0 0 0.99 0 0.5 0.5 0 0 0.5 1 1 0 0 0 0 0 0 0 0 0 0 0 0 0 0 0 2
TCGA-FU-A770 55d2df744993bcfe847851f06ae137af 1 64512800 7292 7291 1 0 0 0 0 0 0 0 0 0 0 0 0 0 0 0 4373.03 4372.43 0.6 0 0 0 0 0 0 0 0 0 0 0 0 0 0 0 1 0 0 0 0 0 0 0 0 0 0 0 0 0 0 0 0 1
TCGA-GH-A9DA 545ef7c8753511f0b5384d7314608ee1 1 46485600 13231 2 1 0 0 0 1 13227 0 0 0 0 0 0 0 0 0 0 11011.74 1.66 0.83 0 0 0 0.83 11008.42 0 0 0 0 0 0 0 0 0 0 0 0 0 0 0 0 1 0 0 0 0 0 0 0 0 0 0 1
TCGA-HG-A2PA 42f069766b5d12f6247876ed415fb98f 1 63715100 11223 10307 87 673 0 0 0 0 0 0 0 0 155 0 1 0 0 0 6814.73 6258.52 52.83 408.65 0 0 0 0 0 0 0 0 94.12 0 0.61 0 0 0 1 1 1 0 0 0 0 0 0 0 0 1 0 0 0 0 0 4
TCGA-HG-A9SC 6cf48282d909c96a8decc36cc1613155 75455100 36380 2 11 0 0 0 0 36367 0 0 0 0 0 0 0 0 0 0 18653.31 1.03 5.64 0 0 0 0 18646.65 0 0 0 0 0 0 0 0 0 0 0 0 0 0 0 0 1 0 0 0 0 0 0 0 0 0 0 1
TCGA-HM-A3JJ bb785efa00ba76ad28c8b752b91c11d8 1 54129000 73735 73696 0 1 0 38 0 0 0 0 0 0 0 0 0 0 0 0 52701.82 52673.94 0 0.71 0 27.16 0 0 0 0 0 0 0 0 0 0 0 0 1 0 0 0 0 0 0 0 0 0 0 0 0 0 0 0 0 1
TCGA-HM-A3JK 01a1ed50458ce9f1fbc171f0df629291 1 68055700 30185 15020 0 0 0 15165 0 0 0 0 0 0 0 0 0 0 0 0 17159.66 8538.61 0 0 0 8621.04 0 0 0 0 0 0 0 0 0 0 0 0 1 0 0 0 1 0 0 0 0 0 0 0 0 0 0 0 0 2
TCGA-HM-A4S6 8f21588ebf640ebe0660f06c8f56fc8b 1 67405600 503749 503747 0 0 0 1 0 0 0 1 0 0 0 0 0 0 0 0 289134.63 289133.48 0 0 0 0.57 0 0 0 0.57 0 0 0 0 0 0 0 0 1 0 0 0 0 0 0 0 0 0 0 0 0 0 0 0 0 1
TCGA-HM-A6W2 9318d72628b065be528a05763ddd672c 0 69991500 0 0 0 0 0 0 0 0 0 0 0 0 0 0 0 0 0 0 0 0 0 0 0 0 0 0 0 0 0 0 0 0 0 0 0 0 0 0 0 0 0 0 0 0 0 0 0 0 0 0 0 0 0 0
TCGA-IR-A3L7 ac72d0e13177a0c5abb516baa3e40c1c 1 44819600 40667 4926 35003 441 0 0 0 173 0 0 0 0 123 1 0 0 0 0 35103.96 4252.15 30214.76 380.67 0 0 0 *149.33445412275 0 0 0 0 106.17 0.86 0 0 0 0 1 1 1 0 0 0 0 0 0 0 0 1 0 0 0 0 0 4
TCGA-IR-A3LA 20e8a3e70f699221fcedcb31818dd250 0 63940400 179 177 2 0 0 0 0 0 0 0 0 0 0 0 0 0 0 0 108.31 107.1 1.21 0 0 0 0 0 0 0 0 0 0 0 0 0 0 0 1 0 0 0 0 0 0 0 0 0 0 0 0 0 0 0 0 1
TCGA-IR-A3LB d6dc49434dd5870d9bef02b9cab743de 1 55878200 79743 23625 7231 0 0 0 0 48673 0 0 0 214 0 0 0 0 0 0 55211.82 16357.29 5006.54 0 0 0 0 33699.82 0 0 0 148.17 0 0 0 0 0 0 1 1 0 0 0 0 1 0 0 0 1 0 0 0 0 0 0 4
TCGA-IR-A3LC db9bb0507d7839e9def3a34bbefaa6e3 1 59659900 63612 63420 0 0 0 0 0 190 0 0 0 0 2 0 0 0 0 0 41251.37 41126.87 0 0 0 0 0 123.21 0 0 0 0 1.3 0 0 0 0 0 1 0 0 0 0 0 1 0 0 0 0 0 0 0 0 0 0 2
TCGA-IR-A3LF cb8baa47b7f5ee3947c85d287ccab79c 1 73013000 443681 443678 0 2 0 0 0 0 0 0 0 0 0 0 0 1 0 0 235099.95 235098.36 0 1.06 0 0 0 0 0 0 0 0 0 0 0 0.53 0 0 1 0 0 0 0 0 0 0 0 0 0 0 0 0 0 0 0 1
TCGA-IR-A3LH 40f7fce159955ce77410d6b208cd7294 1 62541500 115040 5320 23 0 0 1 1 109695 0 0 0 0 0 0 0 0 0 0 71164.35 3290.98 14.23 0 0 0.62 0.62 67857.9 0 0 0 0 0 0 0 0 0 0 1 0 0 0 0 0 1 0 0 0 0 0 0 0 0 0 0 2
TCGA-IR-A3LI 8dc5fa617f8e5a909936cd355458e5aa 1 65593300 271654 271414 0 2 0 0 0 235 1 1 0 0 1 0 0 0 0 0 160228.04 160086.48 0 1.18 0 0 0 138.61 0.59 0.59 0 0 0.59 0 0 0 0 0 1 0 0 0 0 0 1 0 0 0 0 0 0 0 0 0 0 2
TCGA-IR-A3LK b757e4a6dd764dcc27c60bf847ab9c9e 1 74766300 236936 11369 141 225160 0 4 0 1 0 0 1 0 260 0 0 0 0 0 122604.68 5882.99 72.96 116511.08 0 2.07 0 0.52 0 0 0.52 0 134.54 0 0 0 0 0 1 1 1 0 0 0 0 0 0 0 0 1 0 0 0 0 0 4
TCGA-IR-A3LL abb2445df163d307960f469837fae2fd 1 41798200 432637 432630 1 3 0 1 0 0 0 1 0 0 0 0 0 1 0 0 400449.7 400443.22 0.93 2.78 0 0.93 0 0 0 0.93 0 0 0 0 0 0.93 0 0 1 0 0 0 0 0 0 0 0 0 0 0 0 0 0 0 0 1
TCGA-JW-A5VG a97e4cc4d969b7fd99ce5d338dad538d 1 61653000 71275 4225 0 0 0 0 0 0 0 0 0 1 0 67049 0 0 0 0 44726.5 2651.27 0 0 0 0 0 0 0 0 0 0.63 0 42074.6 0 0 0 0 1 0 0 0 0 0 0 0 0 0 0 0 1 0 0 0 0 2
TCGA-JW-A5VH fce742dc759ceae6b206e2a5fc039e1f 0 61652800 2886 2815 15 0 0 0 0 0 0 0 10 0 16 29 1 0 0 0 1811.03 1766.47 9.41 0 0 0 0 0 0 0 6.28 0 10.04 18.2 0.63 0 0 0 1 0 0 0 0 0 0 0 0 0 0 0 0 0 0 0 0 1
TCGA-JW-A5VI 590f62a3d1ff9ceb47c95271b7e83e41 1 63876400 12299 2763 131 2 6 45 16 0 5 0 6418 1560 26 26 1269 32 0 0 7449.23 1673.49 79.34 1.21 3.63 27.26 9.69 0 3.03 0 3887.24 944.86 15.75 15.75 768.6 19.38 0 0 1 1 0 0 0 0 0 0 0 1 1 0 0 1 0 0 0 5
TCGA-JW-A5VJ 9c19d78e7f69d4ef4ce1b35960d8ea40 1 65384800 30468 8997 21467 0 1 0 0 0 0 1 1 0 1 0 0 0 0 0 18028.06 5323.57 12702.13 0 0.59 0 0 0 0 0.59 0.59 0 0.59 0 0 0 0 0 1 1 0 0 0 0 0 0 0 0 0 0 0 0 0 0 0 2
TCGA-JW-A5VK da4f05458b71b3b9a1a196579286b56f 0 108458000 21108 15677 280 829 0 93 256 387 1124 472 421 153 54 488 541 93 0 240 7529.52 5592.21 99.88 *295.716005273931 0 *33.1744131368825 *91.3188146563647 *138.048364343801 *400.946670600601 *168.369064522672 *150.17664441535 *54.577260321968 19.26 *174.076490438695 *192.982338785521 33.17 0 *85.6113887403419 1 1 0 0 0 0 0 0 0 0 0 0 0 0 0 0 0 2
TCGA-JW-A5VL 1600506a2061e0763aa4c1ad5793cfde 1 71664800 236002 235935 62 0 0 0 1 1 1 0 1 0 0 1 0 0 0 0 127406.53 127370.36 33.47 0 0 0 0.54 0.54 0.54 0 0.54 0 0 0.54 0 0 0 0 1 0 0 0 0 0 0 0 0 0 0 0 0 0 0 0 0 1
TCGA-JW-A69B 83303a3a47422e828ffc09c4b31e958f 1 48652000 486992 486961 13 7 1 2 0 0 0 3 0 0 3 1 0 0 1 0 387260.34 387235.69 10.34 5.57 0.8 1.59 0 0 0 2.39 0 0 2.39 0.8 0 0 0.8 0 1 0 0 0 0 0 0 0 0 0 0 0 0 0 0 0 0 1
TCGA-JW-A852 c8622ce05d8ace4b4bb01922cffeebd5 1 61811100 49917 49916 0 0 0 1 0 0 0 0 0 0 0 0 0 0 0 0 31243.8 31243.18 0 0 0 0.63 0 0 0 0 0 0 0 0 0 0 0 0 1 0 0 0 0 0 0 0 0 0 0 0 0 0 0 0 0 1
TCGA-JW-AAVH b87f87504b3819de2b964457adc1c0ff 1 80779600 710978 710969 1 1 0 3 0 1 1 1 0 0 0 1 0 0 0 0 340515.08 340510.77 0.48 0.48 0 1.44 0 0.48 0.48 0.48 0 0 0 0.48 0 0 0 0 1 0 0 0 0 0 0 0 0 0 0 0 0 0 0 0 0 1
TCGA-JX-A3PZ e11bc9d3d2ef4c7c821c2aec1be17fc9 1 70466100 1026296 6 13 1 4 0 7 1026240 7 0 2 4 0 8 0 2 0 2 563474.53 3.29 7.14 0.55 2.2 0 3.84 563443.79 3.84 0 1.1 2.2 0 4.39 0 1.1 0 1.1 0 0 0 0 0 0 1 0 0 0 0 0 0 0 0 0 0 1
TCGA-JX-A3Q0 c03b6f5e373823f296da9f9054ffec89 1 64794300 138482 2422 0 136058 1 1 0 0 0 0 0 0 0 0 0 0 0 0 82687.22 1446.17 0 81239.86 0.6 0.6 0 0 0 0 0 0 0 0 0 0 0 0 1 0 1 0 0 0 0 0 0 0 0 0 0 0 0 0 0 2
TCGA-JX-A3Q8 91e3f223ac91c7ff7dac4e4a048c1cbe 1 55971400 830782 830777 0 1 1 2 0 1 0 0 0 0 0 0 0 0 0 0 574252.38 574248.92 0 0.69 0.69 1.38 0 0.69 0 0 0 0 0 0 0 0 0 0 1 0 0 0 0 0 0 0 0 0 0 0 0 0 0 0 0 1
TCGA-JX-A5QV e9429534fc68c39726fa95374fb2f43b 1 86606400 8067292 8067140 107 10 3 11 0 3 4 4 2 0 2 2 2 2 0 0 3603791.71 3603723.81 47.8 4.47 1.34 4.91 0 1.34 1.79 1.79 0.89 0 0.89 0.89 0.89 0.89 0 0 1 0 0 0 0 0 0 0 0 0 0 0 0 0 0 0 0 1
TCGA-LP-A4AU 13d92ef76d5e23806b6781720a6c7703 1 69285800 70116 18787 51325 1 0 0 1 2 0 0 0 0 0 0 0 0 0 0 39152.08 10490.47 28659.37 0.56 0 0 0.56 1.12 0 0 0 0 0 0 0 0 0 0 1 1 0 0 0 0 0 0 0 0 0 0 0 0 0 0 0 2
TCGA-LP-A4AV 25e5a97bad47b1865496303680191c42 1 41927100 116988 18129 5648 0 0 0 0 65 0 0 2 93142 0 0 2 0 0 0 107951.43 16728.65 5211.73 0 0 0 0 59.98 0 0 1.85 85947.38 0 0 1.85 0 0 0 1 1 0 0 0 0 1 0 0 0 1 0 0 0 0 0 0 4
TCGA-LP-A4AW 133fd8ca02175352839cbfe8b235d784 1 87338000 46779 46685 91 0 0 1 0 1 0 0 0 0 0 0 0 0 1 0 20721.9 20680.26 40.31 0 0 0.44 0 0.44 0 0 0 0 0 0 0 0 0.44 0 1 0 0 0 0 0 0 0 0 0 0 0 0 0 0 0 0 1
TCGA-LP-A4AX 2244dde449365764eaab3c445d5be9e5 1 64745100 5366265 5366150 68 11 2 16 2 1 0 2 1 3 3 0 2 2 0 2 3206617.08 3206548.36 40.63 6.57 1.2 9.56 1.2 0.6 0 1.2 0.6 1.79 1.79 0 1.2 1.2 0 1.2 1 0 0 0 0 0 0 0 0 0 0 0 0 0 0 0 0 1
TCGA-LP-A5U2 ba9728cd5f86d96524d0aa02570c304f 1 62973800 2993486 2993400 0 2 2 10 1 0 0 0 0 0 2 69 0 0 0 0 1839074.08 1839021.24 0 1.23 1.23 6.14 0.61 0 0 0 0 0 1.23 42.39 0 0 0 0 1 0 0 0 0 0 0 0 0 0 0 0 0 0 0 0 0 1
TCGA-LP-A5U3 8124cd2d1f63991379ac49f067e2d467 1 55314500 464711 464644 0 0 0 0 0 0 0 0 0 0 0 65 0 2 0 0 325031.8 324984.94 0 0 0 0 0 0 0 0 0 0 0 45.46 0 1.4 0 0 1 0 0 0 0 0 0 0 0 0 0 0 0 0 0 0 0 1
TCGA-LP-A7HU 3d89ae126826b86ed1f60dd85bc988ff 1 76571800 1458215 1458200 0 2 1 5 0 0 2 0 0 1 3 0 0 0 0 1 736774.52 736766.94 0 1.01 0.51 2.53 0 0 1.01 0 0 0.51 1.52 0 0 0 0 0.51 1 0 0 0 0 0 0 0 0 0 0 0 0 0 0 0 0 1
TCGA-MA-AA3W 8b535742a998ff4f77f7a927cb4d6228 1 87838100 101084 101081 0 0 0 1 0 1 0 0 0 0 1 0 0 0 0 0 44522.69 44521.37 0 0 0 0.44 0 0.44 0 0 0 0 0.44 0 0 0 0 0 1 0 0 0 0 0 0 0 0 0 0 0 0 0 0 0 0 1
TCGA-MA-AA3X d5b858b1a9bafefdd9b575d78055120c 1 60464600 1894353 1894320 1 0 0 4 11 0 0 0 0 0 0 2 1 14 0 0 1212108.84 1212087.72 0.64 0 0 2.56 7.04 0 0 0 0 0 0 1.28 0.64 8.96 0 0 1 0 0 0 0 0 0 0 0 0 0 0 0 0 0 0 0 1
TCGA-MA-AA3Y f89d3e0a2e2f1fdab582a1b68ce5899f 1 80523400 73268 4 1 0 0 0 73259 0 0 0 0 0 0 0 0 2 2 0 35202.55 1.92 0.48 0 0 0 35198.23 0 0 0 0 0 0 0 0 0.96 0.96 0 0 0 0 0 0 1 0 0 0 0 0 0 0 0 0 0 0 1
TCGA-MA-AA3Z 58a87612860d0dd4647ea643fee1afdd 1 94453100 5117663 5117610 3 8 6 12 1 2 1 5 1 0 6 2 0 6 0 0 2096222.41 2096200.7 1.23 3.28 2.46 4.92 0.41 0.82 0.41 2.05 0.41 0 2.46 0.82 0 2.46 0 0 1 0 0 0 0 0 0 0 0 0 0 0 0 0 0 0 0 1
TCGA-MA-AA41 3ca9caa610de513e2b0fd849369d5b14 1 38688500 160412 160410 0 0 1 1 0 0 0 0 0 0 0 0 0 0 0 0 160412 160410 0 0 1 1 0 0 0 0 0 0 0 0 0 0 0 0 1 0 0 0 0 0 0 0 0 0 0 0 0 0 0 0 0 1
TCGA-MA-AA42 aac021ebb11b4a0e893c1c60a5aa39bf 1 53008800 251888 251743 93 3 0 37 0 9 0 1 0 0 1 0 1 0 0 0 183840.59 183734.76 67.88 2.19 0 27 0 6.57 0 0.73 0 0 0.73 0 0.73 0 0 0 1 1 0 0 0 0 0 0 0 0 0 0 0 0 0 0 0 2
TCGA-MA-AA43 1f8d0f8570959fdd9f2ec329021305a2 1 73098400 2723 15 2708 0 0 0 0 0 0 0 0 0 0 0 0 0 0 0 1441.19 7.94 1433.25 0 0 0 0 0 0 0 0 0 0 0 0 0 0 0 0 1 0 0 0 0 0 0 0 0 0 0 0 0 0 0 0 1
TCGA-MU-A51Y 0ae47bde8ada60ad9af6b88181e8bc43 1 102844000 42267 42266 0 0 0 1 0 0 0 0 0 0 0 0 0 0 0 0 15900.26 15899.89 0 0 0 0.38 0 0 0 0 0 0 0 0 0 0 0 0 1 0 0 0 0 0 0 0 0 0 0 0 0 0 0 0 0 1
TCGA-MU-A5YI 93ce7fa24fe1703c2370cb5337e8e4eb 1 56009800 12747 12747 0 0 0 0 0 0 0 0 0 0 0 0 0 0 0 0 8804.93 8804.93 0 0 0 0 0 0 0 0 0 0 0 0 0 0 0 0 1 0 0 0 0 0 0 0 0 0 0 0 0 0 0 0 0 1
TCGA-MU-A8JM 1a9afce7ce5570d9537b40f8c7363967 1 102122000 17803 17799 1 0 0 3 0 0 0 0 0 0 0 0 0 0 0 0 6744.59 6743.08 0.38 0 0 1.14 0 0 0 0 0 0 0 0 0 0 0 0 1 0 0 0 0 0 0 0 0 0 0 0 0 0 0 0 0 1
TCGA-MY-A5BD 7c40e34864e2b2d13177e80096f38143 1 85813500 43259 43259 0 0 0 0 0 0 0 0 0 0 0 0 0 0 0 0 19503.06 19503.06 0 0 0 0 0 0 0 0 0 0 0 0 0 0 0 0 1 0 0 0 0 0 0 0 0 0 0 0 0 0 0 0 0 1
TCGA-MY-A5BE 3379d20228854c21eafc42fe9bc05dfc 1 72820600 101782 101782 0 0 0 0 0 0 0 0 0 0 0 0 0 0 0 0 54075.26 54075.26 0 0 0 0 0 0 0 0 0 0 0 0 0 0 0 0 1 0 0 0 0 0 0 0 0 0 0 0 0 0 0 0 0 1
TCGA-MY-A5BF cc2993e90031bc524da2f058c80eefd2 1 89538200 820416 820412 0 1 0 2 0 0 0 0 0 0 0 1 0 0 0 0 354492.99 354491.26 0 0.43 0 0.86 0 0 0 0 0 0 0 0.43 0 0 0 0 1 0 0 0 0 0 0 0 0 0 0 0 0 0 0 0 0 1
TCGA-MY-A913 c228d385ade2a44d75267d0233065c38 1 44064100 98009 2 11 0 0 0 0 97994 0 0 0 0 0 1 0 1 0 0 86052.39 1.76 9.66 0 0 0 0 86039.22 0 0 0 0 0 0.88 0 0.88 0 0 0 0 0 0 0 0 1 0 0 0 0 0 0 0 0 0 0 1
TCGA-PN-A8MA 3faa92a4ac2ac9c95b698c74b3993c89 1 83200600 704721 704698 0 4 0 3 0 0 0 9 0 1 3 1 0 2 0 0 327697.14 327686.44 0 1.86 0 1.4 0 0 0 4.19 0 0.47 1.4 0.47 0 0.93 0 0 1 0 0 0 0 0 0 0 0 0 0 0 0 0 0 0 0 1
TCGA-Q1-A5R1 132efc96e630dbcdb3cb2cb0703a137f 1 81390300 1913249 1913090 33 1 0 2 1 0 0 0 10 1 32 76 3 0 0 0 909454 909378.42 15.69 0.48 0 0.95 0.48 0 0 0 4.75 0.48 15.21 36.13 1.43 0 0 0 1 0 0 0 0 0 0 0 0 0 0 0 0 0 0 0 0 1
TCGA-Q1-A5R2 eb0f0ac8fbe80d63272d93326e9cbb1f 1 68914300 167535 167436 20 1 1 0 0 0 0 0 8 1 17 50 1 0 0 0 94054.18 93998.6 11.23 0.56 0.56 0 0 0 0 0 4.49 0.56 9.54 28.07 0.56 0 0 0 1 0 0 0 0 0 0 0 0 0 0 0 0 0 0 0 0 1
TCGA-Q1-A5R3 3d48f81b1916704edf9dfebfec6ec03b 1 74295200 31001 11022 19790 0 0 0 0 184 3 0 1 0 0 0 1 0 0 0 16143.47 5739.6 10305.45 0 0 0 0 *95.8162034694031 1.56 0 0.52 0 0 0 0.52 0 0 0 1 1 0 0 0 0 0 0 0 0 0 0 0 0 0 0 0 2
TCGA-Q1-A6DT 918e5eb1512d279ac604bd092639057c 1 79107000 5074802 5074740 1 17 4 21 0 1 0 7 0 2 4 4 1 0 0 0 2481910.29 2481879.97 0.49 8.31 1.96 10.27 0 0.49 0 3.42 0 0.98 1.96 1.96 0.49 0 0 0 1 0 0 0 0 0 0 0 0 0 0 0 0 0 0 0 0 1
TCGA-Q1-A6DV ba40c1c4fdddee58392970fb1c95f24e 1 61535300 164958 164956 0 1 0 1 0 0 0 0 0 0 0 0 0 0 0 0 103712.46 103711.21 0 0.63 0 0.63 0 0 0 0 0 0 0 0 0 0 0 0 1 0 0 0 0 0 0 0 0 0 0 0 0 0 0 0 0 1
TCGA-Q1-A6DW 6a59d8b5d43c66acc9de92c3b1299b69 1 65814600 19411 19408 0 1 0 2 0 0 0 0 0 0 0 0 0 0 0 0 11410.58 11408.81 0 0.59 0 1.18 0 0 0 0 0 0 0 0 0 0 0 0 1 0 0 0 0 0 0 0 0 0 0 0 0 0 0 0 0 1
TCGA-Q1-A73O 9268bd9fedee4b64102b19fc5d534c45 1 64285700 45708 1 45513 0 0 0 0 194 0 0 0 0 0 0 0 0 0 0 27508.05 0.6 27390.69 0 0 0 0 *116.753321500738 0 0 0 0 0 0 0 0 0 0 0 1 0 0 0 0 0 0 0 0 0 0 0 0 0 0 0 1
TCGA-Q1-A73P 365cdd248c35f915d9eff9a090b39896 1 82089500 1531299 8 1531190 0 22 0 1 71 0 2 0 0 0 2 0 3 0 0 721695.97 3.77 721644.6 0 10.37 0 0.47 33.46 0 0.94 0 0 0 0.94 0 1.41 0 0 0 1 0 0 0 0 0 0 0 0 0 0 0 0 0 0 0 1
TCGA-Q1-A73Q 34fdadd32d331e59c4cf59d4b9e31257 1 60327000 69095 0 2 1 69089 0 0 0 0 0 0 0 3 0 0 0 0 0 44311.53 0 1.28 0.64 44307.69 0 0 0 0 0 0 0 1.92 0 0 0 0 0 0 0 0 1 0 0 0 0 0 0 0 0 0 0 0 0 0 1
TCGA-Q1-A73R b723f04be0ee06c7b5ce190ecfaa6a00 1 76058500 1918503 1918490 0 1 2 3 0 0 0 3 0 0 1 1 0 2 0 0 975880.45 975873.84 0 0.51 1.02 1.53 0 0 0 1.53 0 0 0.51 0.51 0 1.02 0 0 1 0 0 0 0 0 0 0 0 0 0 0 0 0 0 0 0 1
TCGA-Q1-A73S c3a175cbeb6069575b4bc8680426a18f 1 61952900 99461 21 99427 0 0 0 0 13 0 0 0 0 0 0 0 0 0 0 62111.65 13.11 62090.42 0 0 0 0 8.12 0 0 0 0 0 0 0 0 0 0 0 1 0 0 0 0 0 0 0 0 0 0 0 0 0 0 0 1
TCGA-R2-A69V f900690c64475cc91dc9a2e7b070f710 1 55771000 2912 4 27 0 0 0 2881 0 0 0 0 0 0 0 0 0 0 0 2020.06 2.77 18.73 0 0 0 1998.56 0 0 0 0 0 0 0 0 0 0 0 0 0 0 0 0 1 0 0 0 0 0 0 0 0 0 0 0 1
TCGA-RA-A741 a39451b33caa9be5e5efd47b2370cd17 1 74885800 145547 145544 0 0 0 1 0 0 1 0 0 0 0 1 0 0 0 0 75194.43 75192.88 0 0 0 0.52 0 0 0.52 0 0 0 0 0.52 0 0 0 0 1 0 0 0 0 0 0 0 0 0 0 0 0 0 0 0 0 1
TCGA-UC-A7PD 6c1eaafa94b224fdc640b1fdde5dbd8d 1 81328700 22879 22877 0 1 1 0 0 0 0 0 0 0 0 0 0 0 0 0 10883.66 10882.71 0 0.48 0.48 0 0 0 0 0 0 0 0 0 0 0 0 0 1 0 0 0 0 0 0 0 0 0 0 0 0 0 0 0 0 1
TCGA-UC-A7PF 15d5856ca18b89468b32a24d25605235 1 65932200 100101 100100 0 0 0 0 0 1 0 0 0 0 0 0 0 0 0 0 58738.48 58737.9 0 0 0 0 0 0.59 0 0 0 0 0 0 0 0 0 0 1 0 0 0 0 0 0 0 0 0 0 0 0 0 0 0 0 1
TCGA-UC-A7PG c89c41ad364442929dba79a0508def59 1 123342000 29750 29736 6 0 0 0 0 8 0 0 0 0 0 0 0 0 0 0 9331.64 9327.25 1.88 0 0 0 0 2.51 0 0 0 0 0 0 0 0 0 0 1 0 0 0 0 0 0 0 0 0 0 0 0 0 0 0 0 1
TCGA-UC-A7PI b3cf4864641cbad52d3817e8c41c01c0 1 92253900 54595 42 54553 0 0 0 0 0 0 0 0 0 0 0 0 0 0 0 22895.49 17.61 22877.88 0 0 0 0 0 0 0 0 0 0 0 0 0 0 0 0 1 0 0 0 0 0 0 0 0 0 0 0 0 0 0 0 1
TCGA-VS-A8EB d704a3b054d00663c0bf5b5867b95d4f 1 110284000 1735813 1735790 2 6 0 4 0 0 0 3 1 0 0 6 0 1 0 0 608936.94 608928.87 0.7 2.1 0 1.4 0 0 0 1.05 0.35 0 0 2.1 0 0.35 0 0 1 0 0 0 0 0 0 0 0 0 0 0 0 0 0 0 0 1
TCGA-VS-A8EC 323e9427f78c7651d452207b7241cd5a 1 93944900 736764 736744 0 3 1 3 1 0 0 8 0 1 1 0 0 1 1 0 303415.02 303406.79 0 1.24 0.41 1.24 0.41 0 0 3.29 0 0.41 0.41 0 0 0.41 0.41 0 1 0 0 0 0 0 0 0 0 0 0 0 0 0 0 0 0 1
TCGA-VS-A8EG d249c9603cab4f8cdd1a0375fbe0a07b 1 73197800 27000 26999 0 1 0 0 0 0 0 0 0 0 0 0 0 0 0 0 14270.78 14270.25 0 0.53 0 0 0 0 0 0 0 0 0 0 0 0 0 0 1 0 0 0 0 0 0 0 0 0 0 0 0 0 0 0 0 1
TCGA-VS-A8EH 3f5807e42065a200bb33382e87bad824 1 88438500 711692 711673 2 6 1 5 0 0 0 2 0 0 0 1 0 2 0 0 311338.34 311330.03 0.87 2.62 0.44 2.19 0 0 0 0.87 0 0 0 0.44 0 0.87 0 0 1 0 0 0 0 0 0 0 0 0 0 0 0 0 0 0 0 1
TCGA-VS-A8EI 99d433eec59926450bbe0bf07aafbaca 1 49339700 51961 51958 0 0 0 0 0 3 0 0 0 0 0 0 0 0 0 0 40743.93 40741.57 0 0 0 0 0 2.35 0 0 0 0 0 0 0 0 0 0 1 0 0 0 0 0 0 0 0 0 0 0 0 0 0 0 0 1
TCGA-VS-A8EJ 7f0d0f8d17f1027832aab4ca3fe1153c 0 50153400 2 1 1 0 0 0 0 0 0 0 0 0 0 0 0 0 0 0 1.54 0.77 0.77 0 0 0 0 0 0 0 0 0 0 0 0 0 0 0 0 0 0 0 0 0 0 0 0 0 0 0 0 0 0 0 0 0
TCGA-VS-A8EK b962f9b999eff061b9e6d960bcd80d40 1 52113000 7473 1 0 35 0 7436 0 1 0 0 0 0 0 0 0 0 0 0 5547.93 0.74 0 25.98 0 5520.46 0 0.74 0 0 0 0 0 0 0 0 0 0 0 0 0 0 1 0 0 0 0 0 0 0 0 0 0 0 0 1
TCGA-VS-A8EL 1642ab824921436d7b1ab802490eecd0 1 59971600 19226 0 0 19225 0 0 0 1 0 0 0 0 0 0 0 0 0 0 12402.96 0 0 12402.31 0 0 0 0.65 0 0 0 0 0 0 0 0 0 0 0 0 1 0 0 0 0 0 0 0 0 0 0 0 0 0 0 1
TCGA-VS-A8Q8 f715f973136f243f932d2d494a5db714 1 74436500 25412 25410 0 0 0 0 0 2 0 0 0 0 0 0 0 0 0 0 13207.93 13206.89 0 0 0 0 0 1.04 0 0 0 0 0 0 0 0 0 0 1 0 0 0 0 0 0 0 0 0 0 0 0 0 0 0 0 1
TCGA-VS-A8Q9 e8eb8a4abcdf5d7e20f3c80edc68f119 1 89824200 184970 20 0 0 7 0 0 0 184942 0 1 0 0 0 0 0 0 0 79669.09 8.61 0 0 3.01 0 0 0 79657.03 0 0.43 0 0 0 0 0 0 0 0 0 0 0 0 0 0 1 0 0 0 0 0 0 0 0 0 1
TCGA-VS-A8QA 694020939f09263fd3ceeeccbb8c8924 1 60853100 99884 99868 0 0 11 0 0 0 4 0 0 0 0 0 0 1 0 0 63503.13 63492.95 0 0 6.99 0 0 0 2.54 0 0 0 0 0 0 0.64 0 0 1 0 0 0 0 0 0 0 0 0 0 0 0 0 0 0 0 1
TCGA-VS-A8QC 56f39672c072c2ff2b05d63e46085a9f 1 64334400 180829 180817 0 1 8 1 0 0 1 0 0 0 0 0 0 0 0 1 108744.35 108737.14 0 0.6 4.81 0.6 0 0 0.6 0 0 0 0 0 0 0 0 0.6 1 0 0 0 0 0 0 0 0 0 0 0 0 0 0 0 0 1
TCGA-VS-A8QF 7d2b5b3285ec0517030840cdfe72b581 1 56184500 40059 40050 0 2 4 1 0 0 1 1 0 0 0 0 0 0 0 0 27584.52 27578.33 0 1.38 2.75 0.69 0 0 0.69 0.69 0 0 0 0 0 0 0 0 1 0 0 0 0 0 0 0 0 0 0 0 0 0 0 0 0 1
TCGA-VS-A8QH b40970533c609c046b9add42ea7de1c0 0 62008000 20 16 0 0 3 0 0 0 1 0 0 0 0 0 0 0 0 0 12.48 9.98 0 0 1.87 0 0 0 0.62 0 0 0 0 0 0 0 0 0 0 0 0 0 0 0 0 0 0 0 0 0 0 0 0 0 0 0
TCGA-VS-A8QM ce0120949220cac24c911d7662f0be92 1 57490000 36332 36314 0 0 8 0 0 0 9 1 0 0 0 0 0 0 0 0 24450 24437.89 0 0 5.38 0 0 0 6.06 0.67 0 0 0 0 0 0 0 0 1 0 0 0 0 0 0 0 0 0 0 0 0 0 0 0 0 1
TCGA-VS-A94W 4f00286c36654cbeaa5bab5967d0ae4c 1 60096800 758306 758278 1 6 7 4 0 1 6 1 0 0 2 0 0 0 0 0 488174.44 488156.41 0.64 3.86 4.51 2.58 0 0.64 3.86 0.64 0 0 1.29 0 0 0 0 0 1 0 0 0 0 0 0 0 0 0 0 0 0 0 0 0 0 1
TCGA-VS-A94X f29994b324b2e89e34cf340092698857 1 86585600 367579 6 77 0 0 25 0 367469 0 0 0 0 0 1 0 1 0 0 164243.02 2.68 34.41 0 0 11.17 0 164193.87 0 0 0 0 0 0.45 0 0.45 0 0 0 0 0 0 0 0 1 0 0 0 0 0 0 0 0 0 0 1
TCGA-VS-A94Y 70f51593c06d2129cf3352223aeafbba 1 58735300 11472 3 74 0 0 15 0 11379 0 0 0 0 0 0 0 1 0 0 7556.52 1.98 48.74 0 0 9.88 0 7495.26 0 0 0 0 0 0 0 0.66 0 0 0 0 0 0 0 0 1 0 0 0 0 0 0 0 0 0 0 1
TCGA-VS-A94Z 776a530122b90f606c6f4da768dbfa18 1 61096400 4494 4494 0 0 0 0 0 0 0 0 0 0 0 0 0 0 0 0 2845.77 2845.77 0 0 0 0 0 0 0 0 0 0 0 0 0 0 0 0 1 0 0 0 0 0 0 0 0 0 0 0 0 0 0 0 0 1
TCGA-VS-A950 47ebd87baa31fe1f4ad03442c1cf2b9b 1 77521800 238201 2 7 1143 18 237029 0 0 0 1 0 0 1 0 0 0 0 0 118878.04 1 3.49 *570.432517046818 8.98 118293.13 0 0 0 0.5 0 0 0.5 0 0 0 0 0 0 0 0 0 1 0 0 0 0 0 0 0 0 0 0 0 0 1
TCGA-VS-A952 b88eec46fea6a60ff80fd37cc4e2ca56 1 70145400 197842 197838 0 2 0 0 1 0 0 1 0 0 0 0 0 0 0 0 109119.2 109117 0 1.1 0 0 0.55 0 0 0.55 0 0 0 0 0 0 0 0 1 0 0 0 0 0 0 0 0 0 0 0 0 0 0 0 0 1
TCGA-VS-A953 e8bc2fd61b8d6c06c2d5ab0de404bc7b 1 60309700 1697039 1697010 0 3 1 13 1 1 0 3 0 1 2 1 0 3 0 0 1088645.66 1088627.06 0 1.92 0.64 8.34 0.64 0.64 0 1.92 0 0.64 1.28 0.64 0 1.92 0 0 1 0 0 0 0 0 0 0 0 0 0 0 0 0 0 0 0 1
TCGA-VS-A954 4162cc523ca207b8dea4500d8fa12085 1 45205900 1691435 15 42 6920 3 1684440 0 7 1 1 0 0 2 1 0 1 0 2 1447578.37 12.84 35.94 *5922.33358919964 2.57 1441591.85 0 5.99 0.86 0.86 0 0 1.71 0.86 0 0.86 0 1.71 0 0 0 0 1 0 0 0 0 0 0 0 0 0 0 0 0 1
TCGA-VS-A957 f7ddac02106961d9670265d024158e38 1 59021000 846940 14 3 2 846913 2 0 0 0 0 0 0 6 0 0 0 0 0 555172.54 9.18 1.97 1.31 555154.84 1.31 0 0 0 0 0 0 3.93 0 0 0 0 0 0 0 0 1 0 0 0 0 0 0 0 0 0 0 0 0 0 1
TCGA-VS-A958 8214acf922254ac0c437a9a61fb7b7bc 1 138825000 4621 1 2 0 15 4 4597 1 0 0 0 0 1 0 0 0 0 0 1287.81 0.28 0.56 0 4.18 1.11 1281.12 0.28 0 0 0 0 0.28 0 0 0 0 0 0 0 0 0 0 1 0 0 0 0 0 0 0 0 0 0 0 1
TCGA-VS-A959 18c71ce4c3c5ee3ce6c92cdbbe1d2be3 1 79857200 200572 18 0 200550 0 0 0 4 0 0 0 0 0 0 0 0 0 0 97171.32 8.72 0 97160.67 0 0 0 1.94 0 0 0 0 0 0 0 0 0 0 0 0 1 0 0 0 0 0 0 0 0 0 0 0 0 0 0 1
TCGA-VS-A9U5 624abc2d9d07feb213600db0ee2dae24 1 91431700 644584 644564 0 6 1 2 0 4 1 3 0 0 2 0 0 1 0 0 272749.91 272741.45 0 2.54 0.42 0.85 0 1.69 0.42 1.27 0 0 0.85 0 0 0.42 0 0 1 0 0 0 0 0 0 0 0 0 0 0 0 0 0 0 0 1
TCGA-VS-A9U6 beed00d72a83a1b8f6b9eb96401824eb 1 60149900 21738 68 0 0 21667 0 0 0 0 3 0 0 0 0 0 0 0 0 13981.91 43.74 0 0 13936.24 0 0 0 0 1.93 0 0 0 0 0 0 0 0 0 0 0 1 0 0 0 0 0 0 0 0 0 0 0 0 0 1
TCGA-VS-A9U7 484b1dfd2bcd74a0039e82c5ee6bbe8f 1 79127000 564502 564486 0 5 1 2 0 2 0 3 0 0 0 2 1 0 0 0 276008.64 276000.82 0 2.44 0.49 0.98 0 0.98 0 1.47 0 0 0 0.98 0.49 0 0 0 1 0 0 0 0 0 0 0 0 0 0 0 0 0 0 0 0 1
TCGA-VS-A9UA 635c9b9c634e616c417bc95bd6e14c52 85782300 9657 35 0 0 0 0 0 3 0 0 0 0 0 9619 0 0 0 0 4355.38 15.79 0 0 0 0 0 1.35 0 0 0 0 0 4338.25 0 0 0 0 0 0 0 0 0 0 0 0 0 0 0 0 1 0 0 0 0 1
TCGA-VS-A9UB 02c72cb8c856275727f75bd0b12bd9cd 1 84851700 35812 17 0 0 0 0 0 35795 0 0 0 0 0 0 0 0 0 0 16328.64 7.75 0 0 0 0 0 16320.89 0 0 0 0 0 0 0 0 0 0 0 0 0 0 0 0 1 0 0 0 0 0 0 0 0 0 0 1
TCGA-VS-A9UC aa148c21fb79b49a448aa23d2a57ef68 1 141228000 1019837 3 3 0 1019820 0 0 1 0 1 0 0 7 0 1 1 0 0 279377.77 0.82 0.82 0 279373.11 0 0 0.27 0 0.27 0 0 1.92 0 0.27 0.27 0 0 0 0 0 1 0 0 0 0 0 0 0 0 0 0 0 0 0 1
TCGA-VS-A9UD 989fbb8c5efe92035c76d9762e820e63 1 128233000 793817 793785 8 1 2 2 0 8 0 2 0 1 5 2 0 0 1 0 239498.33 239488.67 2.41 0.3 0.6 0.6 0 2.41 0 0.6 0 0.3 1.51 0.6 0 0 0.3 0 1 0 0 0 0 0 0 0 0 0 0 0 0 0 0 0 0 1
TCGA-VS-A9UH 4e904701c6e511b0abd83529c6f7e75b 1 66804100 7527 4 1 0 0 0 0 7522 0 0 0 0 0 0 0 0 0 0 4359.14 2.32 0.58 0 0 0 0 4356.24 0 0 0 0 0 0 0 0 0 0 0 0 0 0 0 0 1 0 0 0 0 0 0 0 0 0 0 1
TCGA-VS-A9UI d75077fbcf48c045d49beec0713d6984 1 72408800 6689 18 0 0 1 0 0 0 0 6669 0 0 0 0 0 1 0 0 3573.98 9.62 0 0 0.53 0 0 0 0 3563.29 0 0 0 0 0 0.53 0 0 0 0 0 0 0 0 0 0 1 0 0 0 0 0 0 0 0 1
TCGA-VS-A9UJ 8580d3adcb95fd2f9e286f8f2659388a 0 55556500 4 1 2 0 0 0 0 1 0 0 0 0 0 0 0 0 0 0 2.79 0.7 1.39 0 0 0 0 0.7 0 0 0 0 0 0 0 0 0 0 0 0 0 0 0 0 0 0 0 0 0 0 0 0 0 0 0 0
TCGA-VS-A9UL 9b9cbb6a033c6700d3775e780e2ee200 1 72688600 276011 0 275995 2 0 0 0 14 0 0 0 0 0 0 0 0 0 0 146906.83 0 146898.31 1.06 0 0 0 7.45 0 0 0 0 0 0 0 0 0 0 0 1 0 0 0 0 0 0 0 0 0 0 0 0 0 0 0 1
TCGA-VS-A9UM a147ed80e7a020690ca1d744941fb864 1 73066800 88238 88233 4 1 0 0 0 0 0 0 0 0 0 0 0 0 0 0 46721.57 46718.93 2.12 0.53 0 0 0 0 0 0 0 0 0 0 0 0 0 0 1 0 0 0 0 0 0 0 0 0 0 0 0 0 0 0 0 1
TCGA-VS-A9UO 27a25d2cc0afcda50893415e7b62a47a 1 108246000 825108 14 825030 0 7 0 1 52 0 1 0 0 0 0 1 2 0 0 294904.12 5 294876.24 0 2.5 0 0.36 18.59 0 0.36 0 0 0 0 0.36 0.71 0 0 0 1 0 0 0 0 0 0 0 0 0 0 0 0 0 0 0 1
TCGA-VS-A9UP b730cfe0f00846ccd420121eaf8f3ab4 1 60894500 228118 1 228096 0 8 0 1 10 0 0 0 0 0 1 0 1 0 0 144931.7 0.64 144917.72 0 5.08 0 0.64 6.35 0 0 0 0 0 0.64 0 0.64 0 0 0 1 0 0 0 0 0 0 0 0 0 0 0 0 0 0 0 1
TCGA-VS-A9UQ 37df2617e853ab26c5ffcb5e6b184a13 1 64491400 190934 190928 0 3 1 1 0 0 0 1 0 0 0 0 0 0 0 0 114541.63 114538.03 0 1.8 0.6 0.6 0 0 0 0.6 0 0 0 0 0 0 0 0 1 0 0 0 0 0 0 0 0 0 0 0 0 0 0 0 0 1
TCGA-VS-A9UR 86d98de0fdf53c1684641472388d8f99 1 70870700 27369 27360 0 4 0 0 0 4 0 0 0 0 1 0 0 0 0 0 14940.81 14935.9 0 2.18 0 0 0 2.18 0 0 0 0 0.55 0 0 0 0 0 1 0 0 0 0 0 0 0 0 0 0 0 0 0 0 0 0 1
TCGA-VS-A9UT 9174de4b567bd84b1079daf36ef7c3a8 0 87896100 94 91 0 0 1 1 0 0 0 1 0 0 0 0 0 0 0 0 41.38 40.05 0 0 0.44 0.44 0 0 0 0.44 0 0 0 0 0 0 0 0 0 0 0 0 0 0 0 0 0 0 0 0 0 0 0 0 0 0
TCGA-VS-A9UU 28034a83d68f79f29631063a85718ee7 1 106824000 806483 806448 9 2 1 2 0 3 0 0 0 1 16 1 0 0 0 0 292084.34 292071.66 3.26 0.72 0.36 0.72 0 1.09 0 0 0 0.36 5.79 0.36 0 0 0 0 1 0 0 0 0 0 0 0 0 0 0 0 0 0 0 0 0 1
TCGA-VS-A9UV 2fad624c3fd5eda57b8bbaeb7c902cca 1 79128700 28925 83 0 0 1 4 0 0 0 28837 0 0 0 0 0 0 0 0 14142.34 40.58 0 0 0.49 1.96 0 0 0 14099.31 0 0 0 0 0 0 0 0 0 0 0 0 0 0 0 0 1 0 0 0 0 0 0 0 0 1
TCGA-VS-A9UY e5af499a13a91d6235a4c3456fe20624 1 127679000 37693 37684 2 0 7 0 0 0 0 0 0 0 0 0 0 0 0 0 11421.5 11418.77 0.61 0 2.12 0 0 0 0 0 0 0 0 0 0 0 0 0 1 0 0 0 0 0 0 0 0 0 0 0 0 0 0 0 0 1
TCGA-VS-A9UZ 94672d84a200c78898dfe5221e0339c0 1 72622300 306073 306050 2 4 1 2 0 0 0 1 0 0 13 0 0 0 0 0 163056.05 163043.8 1.07 2.13 0.53 1.07 0 0 0 0.53 0 0 6.93 0 0 0 0 0 1 0 0 0 0 0 0 0 0 0 0 0 0 0 0 0 0 1
TCGA-VS-A9V0 4e5e7fff018118e1fa05431a020ad4e3 0 76091200 91 88 0 0 1 0 0 0 0 1 0 0 0 0 0 1 0 0 46.27 44.74 0 0 0.51 0 0 0 0 0.51 0 0 0 0 0 0.51 0 0 0 0 0 0 0 0 0 0 0 0 0 0 0 0 0 0 0 0
TCGA-VS-A9V1 4a457a9fb33c32b8414a86164b84bb5b 1 126918000 61872 21 11 0 0 0 0 61840 0 0 0 0 0 0 0 0 0 0 18860.48 6.4 3.35 0 0 0 0 18850.73 0 0 0 0 0 0 0 0 0 0 0 0 0 0 0 0 1 0 0 0 0 0 0 0 0 0 0 1
TCGA-VS-A9V2 0637151f53553467d8d8d815af33a9db 1 60310300 743739 743692 0 3 1 0 40 0 0 1 0 0 1 1 0 0 0 0 477101.69 477071.54 0 1.92 0.64 0 25.66 0 0 0.64 0 0 0.64 0.64 0 0 0 0 1 0 0 0 0 0 0 0 0 0 0 0 0 0 0 0 0 1
TCGA-VS-A9V3 8d20ab99ebfc6be0715554bfd5a5587e 1 78998600 874417 123 0 0 18 1 0 0 1 2 0 1 874270 0 0 0 0 1 428233.94 *60.2375928181006 0 0 8.82 0.49 0 0 0.49 0.98 0 0.49 428161.95 0 0 0 0 0.49 0 0 0 0 0 0 0 0 0 0 0 1 0 0 0 0 0 1
TCGA-VS-A9V4 16e31f90e2805b6a8c798a7019d88609 0 132455000 12 4 3 0 3 0 0 1 0 0 0 0 1 0 0 0 0 0 3.51 1.17 0.88 0 0.88 0 0 0.29 0 0 0 0 0.29 0 0 0 0 0 0 0 0 0 0 0 0 0 0 0 0 0 0 0 0 0 0 0
TCGA-VS-A9V5 43f2cd21c8bf106981f52f14e7d55581 1 88759700 5666804 5666680 2 43 7 16 0 1 1 35 1 2 9 4 2 1 0 0 2470041.55 2469987.5 0.87 18.74 3.05 6.97 0 0.44 0.44 15.26 0.44 0.87 3.92 1.74 0.87 0.44 0 0 1 0 0 0 0 0 0 0 0 0 0 0 0 0 0 0 0 1
TCGA-VS-AA62 fae32cdfcd82b2e8dc7b0e0a68c6e7e1 1 109472000 543957 25 23 0 0 1 1 543904 0 0 0 0 0 1 0 2 0 0 192239.85 8.84 8.13 0 0 0.35 0.35 192221.12 0 0 0 0 0 0.35 0 0.71 0 0 0 0 0 0 0 0 1 0 0 0 0 0 0 0 0 0 0 1
TCGA-WL-A834 e59cda4e88beb5e0635fd815d1b973e2 1 62028000 4135005 4134950 1 13 3 8 0 0 1 13 0 2 4 6 0 2 0 2 2579111.71 2579077.4 0.62 8.11 1.87 4.99 0 0 0.62 8.11 0 1.25 2.49 3.74 0 1.25 0 1.25 1 0 0 0 0 0 0 0 0 0 0 0 0 0 0 0 0 1
TCGA-XS-A8TJ 1445e3f8dafbad4e47e724604a6381b5 1 84675700 50472 50467 0 1 0 2 0 0 0 2 0 0 0 0 0 0 0 0 23060.76 23058.48 0 0.46 0 0.91 0 0 0 0.91 0 0 0 0 0 0 0 0 1 0 0 0 0 0 0 0 0 0 0 0 0 0 0 0 0 1
TCGA-ZJ-A8QO 4da93329803ce72a5de740d9f36f87c6 1 52875200 551222 12 0 1 551203 0 0 0 2 2 0 0 2 0 0 0 0 0 403326.18 8.78 0 0.73 403312.28 0 0 0 1.46 1.46 0 0 1.46 0 0 0 0 0 0 0 0 1 0 0 0 0 0 0 0 0 0 0 0 0 0 1
TCGA-ZJ-A8QQ 9deafb99f73f9901911d9d255e3af856 1 62789300 4610 9 0 0 5 0 0 0 1 0 0 0 0 0 0 4595 0 0 2840.52 5.55 0 0 3.08 0 0 0 0.62 0 0 0 0 0 0 2831.27 0 0 0 0 0 0 0 0 0 0 0 0 0 0 0 0 1 0 0 1
TCGA-ZJ-A8QR f40758f74e5486f8ce125d1c799e14cd 1 67258500 678986 678943 0 2 1 1 0 0 0 38 0 0 0 0 0 1 0 0 390566.99 390542.25 0 1.15 0.58 0.58 0 0 0 21.86 0 0 0 0 0 0.58 0 0 1 0 0 0 0 0 0 0 0 0 0 0 0 0 0 0 0 1
TCGA-ZJ-AAX4 39a02712f64db40456f23d77cdcc80d5 1 64868200 10704 10688 1 0 12 3 0 0 0 0 0 0 0 0 0 0 0 0 6384.05 6374.51 0.6 0 7.16 1.79 0 0 0 0 0 0 0 0 0 0 0 0 1 0 0 0 0 0 0 0 0 0 0 0 0 0 0 0 0 1
TCGA-ZJ-AAX8 76e8bc4961b1f2d27b01308c5d7680d3 1 83927200 6511 110 129 41 0 34 2391 147 15 0 49 0 1 305 156 3133 0 0 3001.42 *50.7074583686814 59.47 18.9 0 15.67 *1102.19575417743 *67.7636034563288 6.91 0 22.59 0 0.46 *140.597952749526 *71.9123955046755 *1444.2406097189 0 0 0 1 0 0 0 0 0 0 0 0 0 0 0 0 0 0 0 1
TCGA-ZJ-AAXA 63d32953d4588039facb9509a2ba6511 1 58155600 512829 512818 0 3 0 1 0 6 0 1 0 0 0 0 0 0 0 0 341163.79 341156.47 0 2 0 0.67 0 3.99 0 0.67 0 0 0 0 0 0 0 0 1 0 0 0 0 0 0 0 0 0 0 0 0 0 0 0 0 1
TCGA-ZJ-AAXB 2cdb2d88eca9f1821e90f49e3c2d99eb 1 102256000 27103 18 27074 3 0 0 0 8 0 0 0 0 0 0 0 0 0 0 10254.4 6.81 10243.43 1.14 0 0 0 3.03 0 0 0 0 0 0 0 0 0 0 0 1 0 0 0 0 0 0 0 0 0 0 0 0 0 0 0 1
TCGA-ZJ-AAXD 408b9acdb3d5bc266d1e1baa510a64cd 1 51136500 162410 162404 0 4 0 1 0 0 0 0 0 0 0 0 0 1 0 0 122875.04 122870.5 0 3.03 0 0.76 0 0 0 0 0 0 0 0 0 0.76 0 0 1 0 0 0 0 0 0 0 0 0 0 0 0 0 0 0 0 1
TCGA-ZJ-AAXF a3362dab8973dce3de0f9c1be92aa840 1 148502000 33443 33422 4 0 16 0 0 0 0 0 0 0 1 0 0 0 0 0 8712.74 8707.27 1.04 0 4.17 0 0 0 0 0 0 0 0.26 0 0 0 0 0 1 0 0 0 0 0 0 0 0 0 0 0 0 0 0 0 0 1
TCGA-ZJ-AAXI 858ac937d016c8ca54ea2098d6eeca5c 1 69989300 2810637 2810610 0 4 1 5 0 1 0 1 1 1 11 0 0 2 0 0 1553656.48 1553641.56 0 2.21 0.55 2.76 0 0.55 0 0.55 0.55 0.55 6.08 0 0 1.11 0 0 1 0 0 0 0 0 0 0 0 0 0 0 0 0 0 0 0 1
TCGA-ZJ-AAXJ 1aa0eb146fbd8f2829c69f151471c130 1 94226900 2412180 2412140 0 7 0 2 1 0 0 3 0 0 24 2 0 1 0 0 990413.84 990397.42 0 2.87 0 0.82 0.41 0 0 1.23 0 0 9.85 0.82 0 0.41 0 0 1 0 0 0 0 0 0 0 0 0 0 0 0 0 0 0 0 1
TCGA-ZJ-AAXN 959ec19fda99e3acca44f2da66b3bbc6 1 91340700 205285 18 6 7 0 0 0 205252 0 0 0 0 0 1 0 0 1 0 86951.04 7.62 2.54 2.96 0 0 0 86937.06 0 0 0 0 0 0.42 0 0 0.42 0 0 0 0 0 0 0 1 0 0 0 0 0 0 0 0 0 0 1
TCGA-ZJ-AAXT 81b203b30b98899ae1decf51a58ed64f 1 86743700 21470 2 21458 0 9 0 0 1 0 0 0 0 0 0 0 0 0 0 9575.82 0.89 9570.47 0 4.01 0 0 0.45 0 0 0 0 0 0 0 0 0 0 0 1 0 0 0 0 0 0 0 0 0 0 0 0 0 0 0 1
TCGA-ZJ-AAXU c519a94a9e99053e09d60769895165e9 1 115311000 17479 17470 6 0 0 0 0 3 0 0 0 0 0 0 0 0 0 0 5864.46 5861.44 2.01 0 0 0 0 1.01 0 0 0 0 0 0 0 0 0 0 1 0 0 0 0 0 0 0 0 0 0 0 0 0 0 0 0 1
TCGA-ZJ-AB0H ff333b0f3f458f3453dd5a05f56777e5 1 149059000 72868 17 72094 24 8 0 0 724 0 0 1 0 0 0 0 0 0 0 18913.01 4.41 18712.11 6.23 2.08 0 0 *187.915348955783 0 0 0.26 0 0 0 0 0 0 0 0 1 0 0 0 0 0 0 0 0 0 0 0 0 0 0 0 1
TCGA-ZJ-AB0I 38c40366258a54ccba9765f7d4288457 1 109032000 85438 85413 11 1 8 0 0 3 0 1 0 0 0 1 0 0 0 0 30316.49 30307.62 3.9 0.35 2.84 0 0 1.06 0 0.35 0 0 0 0.35 0 0 0 0 1 0 0 0 0 0 0 0 0 0 0 0 0 0 0 0 0 1
TCGA-ZX-AA5X 223ed0e7a2a28b4888d48e27659ac4b0 1 96998800 1128828 4 0 6225 4 1122590 0 0 0 3 0 0 0 0 1 1 0 0 450239.2 1.6 0 *2482.87517474443 1.6 447751.14 0 0 0 1.2 0 0 0 0 0.4 0.4 0 0 0 0 0 0 1 0 0 0 0 0 0 0 0 0 0 0 0 1
