# Supplementary material for: Identification and characterization of HPV-independent cervical cancers
Source: Oncotarget. 2017 Jan 6;8(8):13375–86. doi: 10.18632/oncotarget.14533 (PMC5355105; doi:10.18632/oncotarget.14533)
Supplement: Supplementary file 4 [file oncotarget-08-13375-s004.docx]

Supplemental Table 2b. GSEA hallmarks gene sets
Gene Set Gene Set Description Number of Markers ES NES p-value FDR Description
HALLMARK_INTERFERON_ALPHA_RESPONSE http://www.broadinstitute.org/gsea/msigdb/cards/HALLMARK_INTERFERON_ALPHA_RESPONSE 94 -0.723884 -2.00065 0 0 0 down vs 1
HALLMARK_INTERFERON_GAMMA_RESPONSE http://www.broadinstitute.org/gsea/msigdb/cards/HALLMARK_INTERFERON_GAMMA_RESPONSE 196 -0.656559 -1.91217 0 0 0 down vs 1
HALLMARK_TNFA_SIGNALING_VIA_NFKB http://www.broadinstitute.org/gsea/msigdb/cards/HALLMARK_TNFA_SIGNALING_VIA_NFKB 199 -0.553988 -1.86767 0 0.00641675 0 down vs 1
HALLMARK_INFLAMMATORY_RESPONSE http://www.broadinstitute.org/gsea/msigdb/cards/HALLMARK_INFLAMMATORY_RESPONSE 199 -0.486977 -1.76047 0 0.0192503 0 down vs 1
HALLMARK_APOPTOSIS http://www.broadinstitute.org/gsea/msigdb/cards/HALLMARK_APOPTOSIS 160 -0.318223 -1.67342 0 0.0247503 0 down vs 1
HALLMARK_IL6_JAK_STAT3_SIGNALING http://www.broadinstitute.org/gsea/msigdb/cards/HALLMARK_IL6_JAK_STAT3_SIGNALING 83 -0.487353 -1.69715 0 0.025667 0 down vs 1
HALLMARK_P53_PATHWAY http://www.broadinstitute.org/gsea/msigdb/cards/HALLMARK_P53_PATHWAY 200 -0.419914 -1.72929 0 0.0269504 0 down vs 1
HALLMARK_ESTROGEN_RESPONSE_LATE http://www.broadinstitute.org/gsea/msigdb/cards/HALLMARK_ESTROGEN_RESPONSE_LATE 200 -0.308519 -1.56779 0 0.060157 0 down vs 1
HALLMARK_COMPLEMENT http://www.broadinstitute.org/gsea/msigdb/cards/HALLMARK_COMPLEMENT 195 -0.326789 -1.51125 0.0909091 0.077001 0 down vs 1
HALLMARK_WNT_BETA_CATENIN_SIGNALING http://www.broadinstitute.org/gsea/msigdb/cards/HALLMARK_WNT_BETA_CATENIN_SIGNALING 42 0.5692 1.81229 0 0.193122 0 up vs 1
HALLMARK_ALLOGRAFT_REJECTION http://www.broadinstitute.org/gsea/msigdb/cards/HALLMARK_ALLOGRAFT_REJECTION 200 -0.441768 -1.31815 0.2 0.232753 0 down vs 1
HALLMARK_APICAL_SURFACE http://www.broadinstitute.org/gsea/msigdb/cards/HALLMARK_APICAL_SURFACE 44 -0.312573 -1.33387 0.133333 0.238703 0 down vs 1
HALLMARK_ESTROGEN_RESPONSE_EARLY http://www.broadinstitute.org/gsea/msigdb/cards/HALLMARK_ESTROGEN_RESPONSE_EARLY 200 -0.271583 -1.29759 0 0.240628 0 down vs 1
HALLMARK_HEDGEHOG_SIGNALING http://www.broadinstitute.org/gsea/msigdb/cards/HALLMARK_HEDGEHOG_SIGNALING 36 0.516052 1.70519 0 0.243334 0 up vs 1
HALLMARK_HYPOXIA http://www.broadinstitute.org/gsea/msigdb/cards/HALLMARK_HYPOXIA 199 -0.245779 -1.22828 0.222222 0.296158 0 down vs 1
HALLMARK_E2F_TARGETS http://www.broadinstitute.org/gsea/msigdb/cards/HALLMARK_E2F_TARGETS 198 -0.400073 -1.21153 0.2 0.302504 0 down vs 1
HALLMARK_MYC_TARGETS_V2 http://www.broadinstitute.org/gsea/msigdb/cards/HALLMARK_MYC_TARGETS_V2 58 0.526543 1.3933 0.181818 0.32348 0 up vs 1
HALLMARK_IL2_STAT5_SIGNALING http://www.broadinstitute.org/gsea/msigdb/cards/HALLMARK_IL2_STAT5_SIGNALING 197 -0.245571 -1.1506 0.375 0.360621 0 down vs 1
HALLMARK_ADIPOGENESIS http://www.broadinstitute.org/gsea/msigdb/cards/HALLMARK_ADIPOGENESIS 196 0.37209 1.39458 0.0465116 0.365277 0 up vs 1
HALLMARK_APICAL_JUNCTION http://www.broadinstitute.org/gsea/msigdb/cards/HALLMARK_APICAL_JUNCTION 199 -0.22265 -1.05694 0.416667 0.409603 0 down vs 1
HALLMARK_NOTCH_SIGNALING http://www.broadinstitute.org/gsea/msigdb/cards/HALLMARK_NOTCH_SIGNALING 32 0.42332 1.39747 0.0609756 0.418432 0 up vs 1
HALLMARK_KRAS_SIGNALING_DN http://www.broadinstitute.org/gsea/msigdb/cards/HALLMARK_KRAS_SIGNALING_DN 196 -0.194706 -1.07086 0.4375 0.421241 0 down vs 1
HALLMARK_G2M_CHECKPOINT http://www.broadinstitute.org/gsea/msigdb/cards/HALLMARK_G2M_CHECKPOINT 199 -0.312955 -1.08337 0.428571 0.429521 0 down vs 1
HALLMARK_UNFOLDED_PROTEIN_RESPONSE http://www.broadinstitute.org/gsea/msigdb/cards/HALLMARK_UNFOLDED_PROTEIN_RESPONSE 112 0.434165 1.46473 0.0731707 0.438388 0 up vs 1
HALLMARK_MYC_TARGETS_V1 http://www.broadinstitute.org/gsea/msigdb/cards/HALLMARK_MYC_TARGETS_V1 199 0.478286 1.41737 0.171053 0.454224 0 up vs 1
HALLMARK_OXIDATIVE_PHOSPHORYLATION http://www.broadinstitute.org/gsea/msigdb/cards/HALLMARK_OXIDATIVE_PHOSPHORYLATION 198 0.45858 1.49511 0.057971 0.491818 0 up vs 1
HALLMARK_PEROXISOME http://www.broadinstitute.org/gsea/msigdb/cards/HALLMARK_PEROXISOME 103 0.338675 1.24838 0.16092 0.622283 0 up vs 1
HALLMARK_MITOTIC_SPINDLE http://www.broadinstitute.org/gsea/msigdb/cards/HALLMARK_MITOTIC_SPINDLE 199 -0.272401 -0.85178 0.615385 0.673759 0 down vs 1
HALLMARK_MTORC1_SIGNALING http://www.broadinstitute.org/gsea/msigdb/cards/HALLMARK_MTORC1_SIGNALING 199 0.325331 1.14989 0.296296 0.674443 0 up vs 1
HALLMARK_XENOBIOTIC_METABOLISM http://www.broadinstitute.org/gsea/msigdb/cards/HALLMARK_XENOBIOTIC_METABOLISM 199 0.288941 1.15137 0.317647 0.729359 0 up vs 1
HALLMARK_PANCREAS_BETA_CELLS http://www.broadinstitute.org/gsea/msigdb/cards/HALLMARK_PANCREAS_BETA_CELLS 40 0.338439 1.15166 0.253968 0.793557 0 up vs 1
HALLMARK_FATTY_ACID_METABOLISM http://www.broadinstitute.org/gsea/msigdb/cards/HALLMARK_FATTY_ACID_METABOLISM 158 0.325237 1.17009 0.25 0.811114 0 up vs 1
HALLMARK_SPERMATOGENESIS http://www.broadinstitute.org/gsea/msigdb/cards/HALLMARK_SPERMATOGENESIS 133 0.286718 1.08153 0.375 0.823253 0 up vs 1
HALLMARK_UV_RESPONSE_UP http://www.broadinstitute.org/gsea/msigdb/cards/HALLMARK_UV_RESPONSE_UP 156 0.258356 1.0252 0.528736 0.844285 0 up vs 1
HALLMARK_ANGIOGENESIS http://www.broadinstitute.org/gsea/msigdb/cards/HALLMARK_ANGIOGENESIS 36 0.319355 1.03459 0.388889 0.862774 0 up vs 1
HALLMARK_PROTEIN_SECRETION http://www.broadinstitute.org/gsea/msigdb/cards/HALLMARK_PROTEIN_SECRETION 94 0.322329 0.999301 0.444444 0.874201 0 up vs 1
HALLMARK_ANDROGEN_RESPONSE http://www.broadinstitute.org/gsea/msigdb/cards/HALLMARK_ANDROGEN_RESPONSE 101 0.187777 0.657341 0.904762 0.898405 0 up vs 1
HALLMARK_PI3K_AKT_MTOR_SIGNALING http://www.broadinstitute.org/gsea/msigdb/cards/HALLMARK_PI3K_AKT_MTOR_SIGNALING 105 0.187796 0.681864 0.908046 0.909939 0 up vs 1
HALLMARK_TGF_BETA_SIGNALING http://www.broadinstitute.org/gsea/msigdb/cards/HALLMARK_TGF_BETA_SIGNALING 53 0.187552 0.595688 0.940476 0.914029 0 up vs 1
HALLMARK_GLYCOLYSIS http://www.broadinstitute.org/gsea/msigdb/cards/HALLMARK_GLYCOLYSIS 199 0.282081 1.03592 0.451219 0.914627 0 up vs 1
HALLMARK_EPITHELIAL_MESENCHYMAL_TRANSITION http://www.broadinstitute.org/gsea/msigdb/cards/HALLMARK_EPITHELIAL_MESENCHYMAL_TRANSITION 198 0.22277 0.685645 0.835616 0.939678 0 up vs 1
HALLMARK_BILE_ACID_METABOLISM http://www.broadinstitute.org/gsea/msigdb/cards/HALLMARK_BILE_ACID_METABOLISM 112 0.255772 0.925948 0.634146 0.952479 0 up vs 1
HALLMARK_MYOGENESIS http://www.broadinstitute.org/gsea/msigdb/cards/HALLMARK_MYOGENESIS 200 0.188244 0.699505 0.835294 0.958745 0 up vs 1
HALLMARK_DNA_REPAIR http://www.broadinstitute.org/gsea/msigdb/cards/HALLMARK_DNA_REPAIR 143 0.269768 0.938143 0.604938 0.974556 0 up vs 1
HALLMARK_COAGULATION http://www.broadinstitute.org/gsea/msigdb/cards/HALLMARK_COAGULATION 135 0.187937 0.706985 0.858974 0.985815 0 up vs 1
HALLMARK_CHOLESTEROL_HOMEOSTASIS http://www.broadinstitute.org/gsea/msigdb/cards/HALLMARK_CHOLESTEROL_HOMEOSTASIS 73 0.226886 0.814422 0.75 1 0 up vs 1
HALLMARK_HEME_METABOLISM http://www.broadinstitute.org/gsea/msigdb/cards/HALLMARK_HEME_METABOLISM 197 0.200939 0.759503 0.755556 1 0 up vs 1
HALLMARK_KRAS_SIGNALING_UP http://www.broadinstitute.org/gsea/msigdb/cards/HALLMARK_KRAS_SIGNALING_UP 196 0.221268 0.821298 0.733333 1 0 up vs 1
HALLMARK_REACTIVE_OXIGEN_SPECIES_PATHWAY http://www.broadinstitute.org/gsea/msigdb/cards/HALLMARK_REACTIVE_OXIGEN_SPECIES_PATHWAY 47 0.215945 0.72284 0.830986 1 0 up vs 1
HALLMARK_UV_RESPONSE_DN http://www.broadinstitute.org/gsea/msigdb/cards/HALLMARK_UV_RESPONSE_DN 144 0.211013 0.734605 0.788889 1 0 up vs 1
