# Supplementary material for: Identification and characterization of HPV-independent cervical cancers
Source: Oncotarget. 2017 Jan 6;8(8):13375–86. doi: 10.18632/oncotarget.14533 (PMC5355105; doi:10.18632/oncotarget.14533)
Supplement: Supplementary file 5 [file oncotarget-08-13375-s005.docx]

Supplemental Table 2c. GSEA c2.cgp gene sets Gene Set Gene Set Description Number of Markers ES NES p-value FDR DescriptionKERLEY_RESPONSE_TO_CISPLATIN_UP http://www.broadinstitute.org/gsea/msigdb/cards/KERLEY_RESPONSE_TO_CISPLATIN_UP 43 -0.610745 -2.28411 0 0 0 down vs 1SLEBOS_HEAD_AND_NECK_CANCER_WITH_HPV_UP http://www.broadinstitute.org/gsea/msigdb/cards/SLEBOS_HEAD_AND_NECK_CANCER_WITH_HPV_UP 83 -0.699731 -2.24873 0 0 0 down vs 1STAMBOLSKY_TARGETS_OF_MUTATED_TP53_DN http://www.broadinstitute.org/gsea/msigdb/cards/STAMBOLSKY_TARGETS_OF_MUTATED_TP53_DN 46 -0.633999 -2.14893 0 0.0187456 0 down vs 1KRIEG_HYPOXIA_VIA_KDM3A http://www.broadinstitute.org/gsea/msigdb/cards/KRIEG_HYPOXIA_VIA_KDM3A 52 -0.532694 -2.15492 0 0.0195267 0 down vs 1BROWNE_INTERFERON_RESPONSIVE_GENES http://www.broadinstitute.org/gsea/msigdb/cards/BROWNE_INTERFERON_RESPONSIVE_GENES 66 -0.755241 -2.12932 0 0.0208284 0 down vs 1ONDER_CDH1_TARGETS_2_DN http://www.broadinstitute.org/gsea/msigdb/cards/ONDER_CDH1_TARGETS_2_DN 455 -0.511204 -2.17258 0 0.0208284 0 down vs 1GEISS_RESPONSE_TO_DSRNA_UP http://www.broadinstitute.org/gsea/msigdb/cards/GEISS_RESPONSE_TO_DSRNA_UP 38 -0.538785 -1.93124 0.0666667 0.0297781 0 down vs 1RADAEVA_RESPONSE_TO_IFNA1_UP http://www.broadinstitute.org/gsea/msigdb/cards/RADAEVA_RESPONSE_TO_IFNA1_UP 52 -0.617128 -1.91983 0 0.0299409 0 down vs 1RICKMAN_METASTASIS_DN http://www.broadinstitute.org/gsea/msigdb/cards/RICKMAN_METASTASIS_DN 249 -0.573789 -1.94222 0 0.0301653 0 down vs 1MOSERLE_IFNA_RESPONSE http://www.broadinstitute.org/gsea/msigdb/cards/MOSERLE_IFNA_RESPONSE 31 -0.789683 -1.9269 0 0.0302959 0 down vs 1DOANE_BREAST_CANCER_ESR1_DN http://www.broadinstitute.org/gsea/msigdb/cards/DOANE_BREAST_CANCER_ESR1_DN 48 -0.490883 -1.92652 0 0.0303237 0 down vs 1SARRIO_EPITHELIAL_MESENCHYMAL_TRANSITION_DN http://www.broadinstitute.org/gsea/msigdb/cards/SARRIO_EPITHELIAL_MESENCHYMAL_TRANSITION_DN 149 -0.486856 -1.9315 0 0.0307387 0 down vs 1BILD_HRAS_ONCOGENIC_SIGNATURE http://www.broadinstitute.org/gsea/msigdb/cards/BILD_HRAS_ONCOGENIC_SIGNATURE 254 -0.440437 -1.9246 0.05 0.0307963 0 down vs 1RASHI_RESPONSE_TO_IONIZING_RADIATION_2 http://www.broadinstitute.org/gsea/msigdb/cards/RASHI_RESPONSE_TO_IONIZING_RADIATION_2 125 -0.409596 -1.94322 0 0.0312426 0 down vs 1YAN_ESCAPE_FROM_ANOIKIS http://www.broadinstitute.org/gsea/msigdb/cards/YAN_ESCAPE_FROM_ANOIKIS 24 -0.709843 -1.93214 0 0.0317634 0 down vs 1XU_AKT1_TARGETS_6HR http://www.broadinstitute.org/gsea/msigdb/cards/XU_AKT1_TARGETS_6HR 27 -0.626277 -1.94357 0 0.0323998 0 down vs 1UROSEVIC_RESPONSE_TO_IMIQUIMOD http://www.broadinstitute.org/gsea/msigdb/cards/UROSEVIC_RESPONSE_TO_IMIQUIMOD 23 -0.774364 -1.89652 0 0.0327667 0 down vs 1CROMER_METASTASIS_DN http://www.broadinstitute.org/gsea/msigdb/cards/CROMER_METASTASIS_DN 80 -0.557973 -1.90024 0 0.0328048 0 down vs 1WUNDER_INFLAMMATORY_RESPONSE_AND_CHOLESTEROL_UP http://www.broadinstitute.org/gsea/msigdb/cards/WUNDER_INFLAMMATORY_RESPONSE_AND_CHOLESTEROL_UP 58 -0.57078 -1.94553 0.03125 0.0330451 0 down vs 1HINATA_NFKB_TARGETS_KERATINOCYTE_UP http://www.broadinstitute.org/gsea/msigdb/cards/HINATA_NFKB_TARGETS_KERATINOCYTE_UP 91 -0.555914 -1.94903 0 0.0331172 0 down vs 1LIANG_SILENCED_BY_METHYLATION_2 http://www.broadinstitute.org/gsea/msigdb/cards/LIANG_SILENCED_BY_METHYLATION_2 53 -0.550513 -1.90459 0.0285714 0.0332981 0 down vs 1GRAHAM_CML_QUIESCENT_VS_NORMAL_QUIESCENT_UP http://www.broadinstitute.org/gsea/msigdb/cards/GRAHAM_CML_QUIESCENT_VS_NORMAL_QUIESCENT_UP 84 -0.495918 -2.09036 0 0.0334743 0 down vs 1FURUKAWA_DUSP6_TARGETS_PCI35_UP http://www.broadinstitute.org/gsea/msigdb/cards/FURUKAWA_DUSP6_TARGETS_PCI35_UP 71 -0.469677 -1.90027 0.0434783 0.0336459 0 down vs 1NOJIMA_SFRP2_TARGETS_DN http://www.broadinstitute.org/gsea/msigdb/cards/NOJIMA_SFRP2_TARGETS_DN 24 -0.556851 -1.9076 0 0.0337758 0 down vs 1BOSCO_INTERFERON_INDUCED_ANTIVIRAL_MODULE http://www.broadinstitute.org/gsea/msigdb/cards/BOSCO_INTERFERON_INDUCED_ANTIVIRAL_MODULE 76 -0.634172 -1.88881 0 0.0338462 0 down vs 1SANA_RESPONSE_TO_IFNG_UP http://www.broadinstitute.org/gsea/msigdb/cards/SANA_RESPONSE_TO_IFNG_UP 75 -0.674922 -1.87824 0 0.0339594 0 down vs 1MURAKAMI_UV_RESPONSE_6HR_UP http://www.broadinstitute.org/gsea/msigdb/cards/MURAKAMI_UV_RESPONSE_6HR_UP 35 -0.516086 -1.9771 0 0.0343669 0 down vs 1HUANG_DASATINIB_RESISTANCE_UP http://www.broadinstitute.org/gsea/msigdb/cards/HUANG_DASATINIB_RESISTANCE_UP 80 -0.586307 -1.86642 0 0.0344307 0 down vs 1LINDSTEDT_DENDRITIC_CELL_MATURATION_B http://www.broadinstitute.org/gsea/msigdb/cards/LINDSTEDT_DENDRITIC_CELL_MATURATION_B 52 -0.575401 -1.94994 0 0.0344971 0 down vs 1BECKER_TAMOXIFEN_RESISTANCE_UP http://www.broadinstitute.org/gsea/msigdb/cards/BECKER_TAMOXIFEN_RESISTANCE_UP 50 -0.461148 -1.87854 0 0.0347141 0 down vs 1GOLDRATH_ANTIGEN_RESPONSE http://www.broadinstitute.org/gsea/msigdb/cards/GOLDRATH_ANTIGEN_RESPONSE 337 -0.437851 -1.87961 0 0.035148 0 down vs 1VERHAAK_GLIOBLASTOMA_MESENCHYMAL http://www.broadinstitute.org/gsea/msigdb/cards/VERHAAK_GLIOBLASTOMA_MESENCHYMAL 213 -0.502093 -1.86751 0.0333333 0.035148 0 down vs 1PYEON_HPV_POSITIVE_TUMORS_UP http://www.broadinstitute.org/gsea/msigdb/cards/PYEON_HPV_POSITIVE_TUMORS_UP 97 -0.631355 -1.88118 0 0.0356021 0 down vs 1MCDOWELL_ACUTE_LUNG_INJURY_UP http://www.broadinstitute.org/gsea/msigdb/cards/MCDOWELL_ACUTE_LUNG_INJURY_UP 45 -0.514261 -1.86789 0 0.0358958 0 down vs 1PHONG_TNF_TARGETS_UP http://www.broadinstitute.org/gsea/msigdb/cards/PHONG_TNF_TARGETS_UP 63 -0.620252 -1.95225 0 0.035997 0 down vs 1BENNETT_SYSTEMIC_LUPUS_ERYTHEMATOSUS http://www.broadinstitute.org/gsea/msigdb/cards/BENNETT_SYSTEMIC_LUPUS_ERYTHEMATOSUS 31 -0.720868 -1.97793 0 0.0361757 0 down vs 1TIAN_TNF_SIGNALING_VIA_NFKB http://www.broadinstitute.org/gsea/msigdb/cards/TIAN_TNF_SIGNALING_VIA_NFKB 28 -0.6735 -1.98699 0 0.0367561 0 down vs 1STREICHER_LSM1_TARGETS_DN http://www.broadinstitute.org/gsea/msigdb/cards/STREICHER_LSM1_TARGETS_DN 19 -0.627753 -1.86052 0 0.0368663 0 down vs 1JAEGER_METASTASIS_DN http://www.broadinstitute.org/gsea/msigdb/cards/JAEGER_METASTASIS_DN 254 -0.599395 -1.85645 0 0.0372509 0 down vs 1HECKER_IFNB1_TARGETS http://www.broadinstitute.org/gsea/msigdb/cards/HECKER_IFNB1_TARGETS 95 -0.629836 -1.98397 0 0.0373176 0 down vs 1CHARAFE_BREAST_CANCER_BASAL_VS_MESENCHYMAL_UP http://www.broadinstitute.org/gsea/msigdb/cards/CHARAFE_BREAST_CANCER_BASAL_VS_MESENCHYMAL_UP 116 -0.56882 -2.04448 0 0.0374912 0 down vs 1RASHI_NFKB1_TARGETS http://www.broadinstitute.org/gsea/msigdb/cards/RASHI_NFKB1_TARGETS 19 -0.604613 -1.95277 0 0.0376332 0 down vs 1STEIN_ESR1_TARGETS http://www.broadinstitute.org/gsea/msigdb/cards/STEIN_ESR1_TARGETS 84 -0.493113 -1.85739 0 0.0379813 0 down vs 1CHANG_IMMORTALIZED_BY_HPV31_DN http://www.broadinstitute.org/gsea/msigdb/cards/CHANG_IMMORTALIZED_BY_HPV31_DN 62 -0.515468 -1.95706 0 0.0386814 0 down vs 1KIM_ALL_DISORDERS_CALB1_CORR_DN http://www.broadinstitute.org/gsea/msigdb/cards/KIM_ALL_DISORDERS_CALB1_CORR_DN 34 -0.50266 -1.81098 0 0.0388363 0 down vs 1DAUER_STAT3_TARGETS_DN http://www.broadinstitute.org/gsea/msigdb/cards/DAUER_STAT3_TARGETS_DN 50 -0.635921 -1.82932 0 0.0390533 0 down vs 1NAKAMURA_METASTASIS_MODEL_DN http://www.broadinstitute.org/gsea/msigdb/cards/NAKAMURA_METASTASIS_MODEL_DN 42 -0.545029 -1.98702 0 0.0390533 0 down vs 1LEE_EARLY_T_LYMPHOCYTE_DN http://www.broadinstitute.org/gsea/msigdb/cards/LEE_EARLY_T_LYMPHOCYTE_DN 54 -0.550823 -1.80625 0 0.0391603 0 down vs 1LIN_SILENCED_BY_TUMOR_MICROENVIRONMENT http://www.broadinstitute.org/gsea/msigdb/cards/LIN_SILENCED_BY_TUMOR_MICROENVIRONMENT 107 -0.511859 -1.81308 0 0.0393833 0 down vs 1FERRANDO_HOX11_NEIGHBORS http://www.broadinstitute.org/gsea/msigdb/cards/FERRANDO_HOX11_NEIGHBORS 23 -0.677747 -2.01016 0 0.0396541 0 down vs 1BASSO_CD40_SIGNALING_UP http://www.broadinstitute.org/gsea/msigdb/cards/BASSO_CD40_SIGNALING_UP 100 -0.54989 -1.82964 0.037037 0.0396732 0 down vs 1ZHANG_INTERFERON_RESPONSE http://www.broadinstitute.org/gsea/msigdb/cards/ZHANG_INTERFERON_RESPONSE 23 -0.751863 -1.80339 0 0.0396866 0 down vs 1SEKI_INFLAMMATORY_RESPONSE_LPS_UP http://www.broadinstitute.org/gsea/msigdb/cards/SEKI_INFLAMMATORY_RESPONSE_LPS_UP 76 -0.513226 -1.82554 0.03125 0.0398945 0 down vs 1ZHAN_MULTIPLE_MYELOMA_LB_DN http://www.broadinstitute.org/gsea/msigdb/cards/ZHAN_MULTIPLE_MYELOMA_LB_DN 39 -0.574698 -1.81316 0.0285714 0.039946 0 down vs 1GRANDVAUX_IRF3_TARGETS_UP http://www.broadinstitute.org/gsea/msigdb/cards/GRANDVAUX_IRF3_TARGETS_UP 15 -0.708336 -1.81689 0 0.0399722 0 down vs 1AMIT_SERUM_RESPONSE_120_MCF10A http://www.broadinstitute.org/gsea/msigdb/cards/AMIT_SERUM_RESPONSE_120_MCF10A 64 -0.459428 -1.8305 0 0.0400611 0 down vs 1DORN_ADENOVIRUS_INFECTION_24HR_DN http://www.broadinstitute.org/gsea/msigdb/cards/DORN_ADENOVIRUS_INFECTION_24HR_DN 43 -0.487325 -1.81989 0.0416667 0.0401025 0 down vs 1DORN_ADENOVIRUS_INFECTION_32HR_DN http://www.broadinstitute.org/gsea/msigdb/cards/DORN_ADENOVIRUS_INFECTION_32HR_DN 39 -0.507703 -1.81402 0 0.0402985 0 down vs 1FUJII_YBX1_TARGETS_UP http://www.broadinstitute.org/gsea/msigdb/cards/FUJII_YBX1_TARGETS_UP 41 -0.501554 -1.99072 0 0.0406154 0 down vs 1BOWIE_RESPONSE_TO_EXTRACELLULAR_MATRIX http://www.broadinstitute.org/gsea/msigdb/cards/BOWIE_RESPONSE_TO_EXTRACELLULAR_MATRIX 17 -0.777841 -1.82039 0 0.0407101 0 down vs 1NIELSEN_LEIOMYOSARCOMA_UP http://www.broadinstitute.org/gsea/msigdb/cards/NIELSEN_LEIOMYOSARCOMA_UP 18 -0.6085 -1.83084 0.0263158 0.0407179 0 down vs 1AMIT_EGF_RESPONSE_40_HELA http://www.broadinstitute.org/gsea/msigdb/cards/AMIT_EGF_RESPONSE_40_HELA 42 -0.593879 -1.8468 0 0.0412639 0 down vs 1KRASNOSELSKAYA_ILF3_TARGETS_UP http://www.broadinstitute.org/gsea/msigdb/cards/KRASNOSELSKAYA_ILF3_TARGETS_UP 38 -0.654167 -1.99489 0 0.0412849 0 down vs 1MUELLER_METHYLATED_IN_GLIOBLASTOMA http://www.broadinstitute.org/gsea/msigdb/cards/MUELLER_METHYLATED_IN_GLIOBLASTOMA 40 -0.479253 -1.83624 0 0.0413828 0 down vs 1SANA_TNF_SIGNALING_UP http://www.broadinstitute.org/gsea/msigdb/cards/SANA_TNF_SIGNALING_UP 82 -0.571704 -1.83084 0 0.0413965 0 down vs 1VARELA_ZMPSTE24_TARGETS_UP http://www.broadinstitute.org/gsea/msigdb/cards/VARELA_ZMPSTE24_TARGETS_UP 39 -0.465361 -1.83521 0 0.0414773 0 down vs 1DER_IFN_GAMMA_RESPONSE_UP http://www.broadinstitute.org/gsea/msigdb/cards/DER_IFN_GAMMA_RESPONSE_UP 71 -0.543878 -1.83161 0 0.0415686 0 down vs 1DER_IFN_ALPHA_RESPONSE_UP http://www.broadinstitute.org/gsea/msigdb/cards/DER_IFN_ALPHA_RESPONSE_UP 74 -0.576727 -2.04605 0 0.0416569 0 down vs 1DORN_ADENOVIRUS_INFECTION_12HR_UP http://www.broadinstitute.org/gsea/msigdb/cards/DORN_ADENOVIRUS_INFECTION_12HR_UP 29 -0.51339 -1.8392 0 0.0417515 0 down vs 1HUMMERICH_SKIN_CANCER_PROGRESSION_UP http://www.broadinstitute.org/gsea/msigdb/cards/HUMMERICH_SKIN_CANCER_PROGRESSION_UP 88 -0.436219 -1.83745 0 0.0418428 0 down vs 1AIGNER_ZEB1_TARGETS http://www.broadinstitute.org/gsea/msigdb/cards/AIGNER_ZEB1_TARGETS 34 -0.554244 -1.84197 0 0.0422354 0 down vs 1XU_HGF_TARGETS_INDUCED_BY_AKT1_6HR http://www.broadinstitute.org/gsea/msigdb/cards/XU_HGF_TARGETS_INDUCED_BY_AKT1_6HR 18 -0.640636 -1.79548 0 0.042342 0 down vs 1CASTELLANO_NRAS_TARGETS_UP http://www.broadinstitute.org/gsea/msigdb/cards/CASTELLANO_NRAS_TARGETS_UP 67 -0.389267 -1.79394 0 0.042458 0 down vs 1BOWIE_RESPONSE_TO_TAMOXIFEN http://www.broadinstitute.org/gsea/msigdb/cards/BOWIE_RESPONSE_TO_TAMOXIFEN 18 -0.779422 -1.79673 0 0.04249 0 down vs 1WU_CELL_MIGRATION http://www.broadinstitute.org/gsea/msigdb/cards/WU_CELL_MIGRATION 181 -0.42448 -1.79204 0 0.0427115 0 down vs 1GAURNIER_PSMD4_TARGETS http://www.broadinstitute.org/gsea/msigdb/cards/GAURNIER_PSMD4_TARGETS 71 -0.616526 -1.79432 0.0344828 0.0428065 0 down vs 1EINAV_INTERFERON_SIGNATURE_IN_CANCER http://www.broadinstitute.org/gsea/msigdb/cards/EINAV_INTERFERON_SIGNATURE_IN_CANCER 26 -0.754393 -2.01043 0 0.0429586 0 down vs 1DUTTA_APOPTOSIS_VIA_NFKB http://www.broadinstitute.org/gsea/msigdb/cards/DUTTA_APOPTOSIS_VIA_NFKB 33 -0.505743 -1.78762 0 0.0443255 0 down vs 1AMIT_SERUM_RESPONSE_240_MCF10A http://www.broadinstitute.org/gsea/msigdb/cards/AMIT_SERUM_RESPONSE_240_MCF10A 56 -0.441777 -1.78564 0 0.0449354 0 down vs 1ZHANG_RESPONSE_TO_IKK_INHIBITOR_AND_TNF_UP http://www.broadinstitute.org/gsea/msigdb/cards/ZHANG_RESPONSE_TO_IKK_INHIBITOR_AND_TNF_UP 219 -0.521079 -2.01742 0 0.0454439 0 down vs 1AMIT_DELAYED_EARLY_GENES http://www.broadinstitute.org/gsea/msigdb/cards/AMIT_DELAYED_EARLY_GENES 18 -0.772831 -2.04812 0 0.046864 0 down vs 1HIRSCH_CELLULAR_TRANSFORMATION_SIGNATURE_UP http://www.broadinstitute.org/gsea/msigdb/cards/HIRSCH_CELLULAR_TRANSFORMATION_SIGNATURE_UP 236 -0.383961 -1.77921 0.05 0.046864 0 down vs 1STEIN_ESRRA_TARGETS_RESPONSIVE_TO_ESTROGEN_DN http://www.broadinstitute.org/gsea/msigdb/cards/STEIN_ESRRA_TARGETS_RESPONSIVE_TO_ESTROGEN_DN 41 -0.608133 -1.77586 0 0.0474286 0 down vs 1GRAHAM_CML_QUIESCENT_VS_NORMAL_DIVIDING_UP http://www.broadinstitute.org/gsea/msigdb/cards/GRAHAM_CML_QUIESCENT_VS_NORMAL_DIVIDING_UP 55 -0.469521 -1.77434 0 0.0477829 0 down vs 1DORN_ADENOVIRUS_INFECTION_48HR_DN http://www.broadinstitute.org/gsea/msigdb/cards/DORN_ADENOVIRUS_INFECTION_48HR_DN 40 -0.483079 -1.77471 0.0869565 0.0477938 0 down vs 1XU_RESPONSE_TO_TRETINOIN_AND_NSC682994_UP http://www.broadinstitute.org/gsea/msigdb/cards/XU_RESPONSE_TO_TRETINOIN_AND_NSC682994_UP 17 -0.60191 -1.76832 0 0.0508142 0 down vs 1KIM_LRRC3B_TARGETS http://www.broadinstitute.org/gsea/msigdb/cards/KIM_LRRC3B_TARGETS 30 -0.644647 -1.76925 0.030303 0.0508601 0 down vs 1RUAN_RESPONSE_TO_TNF_TROGLITAZONE_UP http://www.broadinstitute.org/gsea/msigdb/cards/RUAN_RESPONSE_TO_TNF_TROGLITAZONE_UP 17 -0.55691 -1.765 0 0.0509468 0 down vs 1CHEN_PDGF_TARGETS http://www.broadinstitute.org/gsea/msigdb/cards/CHEN_PDGF_TARGETS 19 -0.580258 -1.75126 0 0.0542455 0 down vs 1DER_IFN_BETA_RESPONSE_UP http://www.broadinstitute.org/gsea/msigdb/cards/DER_IFN_BETA_RESPONSE_UP 102 -0.50203 -1.75343 0 0.0547624 0 down vs 1KIM_WT1_TARGETS_UP http://www.broadinstitute.org/gsea/msigdb/cards/KIM_WT1_TARGETS_UP 211 -0.356937 -1.75136 0.0526316 0.0548482 0 down vs 1NAGASHIMA_NRG1_SIGNALING_UP http://www.broadinstitute.org/gsea/msigdb/cards/NAGASHIMA_NRG1_SIGNALING_UP 173 -0.433506 -1.7468 0 0.0555236 0 down vs 1BORLAK_LIVER_CANCER_EGF_UP http://www.broadinstitute.org/gsea/msigdb/cards/BORLAK_LIVER_CANCER_EGF_UP 57 -0.414979 -1.74414 0 0.0564383 0 down vs 1RICKMAN_TUMOR_DIFFERENTIATED_WELL_VS_POORLY_DN http://www.broadinstitute.org/gsea/msigdb/cards/RICKMAN_TUMOR_DIFFERENTIATED_WELL_VS_POORLY_DN 368 -0.541937 -1.73018 0 0.0616631 0 down vs 1RHEIN_ALL_GLUCOCORTICOID_THERAPY_UP http://www.broadinstitute.org/gsea/msigdb/cards/RHEIN_ALL_GLUCOCORTICOID_THERAPY_UP 77 -0.446231 -1.73028 0.0333333 0.0621529 0 down vs 1TARTE_PLASMA_CELL_VS_B_LYMPHOCYTE_DN http://www.broadinstitute.org/gsea/msigdb/cards/TARTE_PLASMA_CELL_VS_B_LYMPHOCYTE_DN 38 -0.551596 -1.72498 0.030303 0.0634516 0 down vs 1CHARAFE_BREAST_CANCER_LUMINAL_VS_BASAL_DN http://www.broadinstitute.org/gsea/msigdb/cards/CHARAFE_BREAST_CANCER_LUMINAL_VS_BASAL_DN 437 -0.490186 -1.72553 0 0.0639498 0 down vs 1HAHTOLA_SEZARY_SYNDROM_UP http://www.broadinstitute.org/gsea/msigdb/cards/HAHTOLA_SEZARY_SYNDROM_UP 95 -0.390468 -1.71625 0 0.0647826 0 down vs 1ZHU_CMV_8_HR_UP http://www.broadinstitute.org/gsea/msigdb/cards/ZHU_CMV_8_HR_UP 47 -0.540386 -1.71698 0 0.06496 0 down vs 1SHIN_B_CELL_LYMPHOMA_CLUSTER_7 http://www.broadinstitute.org/gsea/msigdb/cards/SHIN_B_CELL_LYMPHOMA_CLUSTER_7 27 -0.451714 -1.71371 0 0.0653669 0 down vs 1ALTEMEIER_RESPONSE_TO_LPS_WITH_MECHANICAL_VENTILATION http://www.broadinstitute.org/gsea/msigdb/cards/ALTEMEIER_RESPONSE_TO_LPS_WITH_MECHANICAL_VENTILATION 127 -0.513357 -1.71737 0.0277778 0.0654534 0 down vs 1LEE_LIVER_CANCER_MYC_E2F1_UP http://www.broadinstitute.org/gsea/msigdb/cards/LEE_LIVER_CANCER_MYC_E2F1_UP 56 -0.464701 -1.71922 0 0.0656411 0 down vs 1GHO_ATF5_TARGETS_DN http://www.broadinstitute.org/gsea/msigdb/cards/GHO_ATF5_TARGETS_DN 16 -0.604594 -1.72168 0 0.0656733 0 down vs 1SAGIV_CD24_TARGETS_DN http://www.broadinstitute.org/gsea/msigdb/cards/SAGIV_CD24_TARGETS_DN 46 -0.452544 -1.71089 0 0.0671417 0 down vs 1LI_INDUCED_T_TO_NATURAL_KILLER_UP http://www.broadinstitute.org/gsea/msigdb/cards/LI_INDUCED_T_TO_NATURAL_KILLER_UP 302 -0.375903 -1.70799 0.04 0.0685851 0 down vs 1DAUER_STAT3_TARGETS_UP http://www.broadinstitute.org/gsea/msigdb/cards/DAUER_STAT3_TARGETS_UP 49 -0.504253 -1.70663 0.0322581 0.0688222 0 down vs 1LEE_NAIVE_T_LYMPHOCYTE http://www.broadinstitute.org/gsea/msigdb/cards/LEE_NAIVE_T_LYMPHOCYTE 17 -0.540706 -1.70331 0 0.0706609 0 down vs 1SEITZ_NEOPLASTIC_TRANSFORMATION_BY_8P_DELETION_UP http://www.broadinstitute.org/gsea/msigdb/cards/SEITZ_NEOPLASTIC_TRANSFORMATION_BY_8P_DELETION_UP 72 -0.46527 -1.70103 0.03125 0.071887 0 down vs 1RAY_TUMORIGENESIS_BY_ERBB2_CDC25A_UP http://www.broadinstitute.org/gsea/msigdb/cards/RAY_TUMORIGENESIS_BY_ERBB2_CDC25A_UP 101 -0.333162 -1.69389 0 0.0759569 0 down vs 1ZHAN_MULTIPLE_MYELOMA_SPIKED http://www.broadinstitute.org/gsea/msigdb/cards/ZHAN_MULTIPLE_MYELOMA_SPIKED 21 -0.525403 -1.69119 0.025 0.0773966 0 down vs 1ZHENG_IL22_SIGNALING_UP http://www.broadinstitute.org/gsea/msigdb/cards/ZHENG_IL22_SIGNALING_UP 53 -0.418402 -1.68871 0 0.0789435 0 down vs 1LI_PROSTATE_CANCER_EPIGENETIC http://www.broadinstitute.org/gsea/msigdb/cards/LI_PROSTATE_CANCER_EPIGENETIC 30 -0.523547 -1.68885 0 0.0795139 0 down vs 1WIERENGA_STAT5A_TARGETS_GROUP2 http://www.broadinstitute.org/gsea/msigdb/cards/WIERENGA_STAT5A_TARGETS_GROUP2 59 -0.469839 -1.68148 0 0.0841893 0 down vs 1WORSCHECH_TUMOR_REJECTION_UP http://www.broadinstitute.org/gsea/msigdb/cards/WORSCHECH_TUMOR_REJECTION_UP 56 -0.50452 -1.6735 0.027027 0.085064 0 down vs 1BURTON_ADIPOGENESIS_3 http://www.broadinstitute.org/gsea/msigdb/cards/BURTON_ADIPOGENESIS_3 101 -0.563853 -1.67939 0 0.0850951 0 down vs 1NAKAMURA_METASTASIS_MODEL_UP http://www.broadinstitute.org/gsea/msigdb/cards/NAKAMURA_METASTASIS_MODEL_UP 42 -0.398757 -1.66751 0 0.0851614 0 down vs 1MARSHALL_VIRAL_INFECTION_RESPONSE_DN http://www.broadinstitute.org/gsea/msigdb/cards/MARSHALL_VIRAL_INFECTION_RESPONSE_DN 29 -0.543076 -1.66833 0.0322581 0.0855998 0 down vs 1HUMMERICH_BENIGN_SKIN_TUMOR_UP http://www.broadinstitute.org/gsea/msigdb/cards/HUMMERICH_BENIGN_SKIN_TUMOR_UP 18 -0.571496 -1.6738 0 0.0856525 0 down vs 1GILDEA_METASTASIS http://www.broadinstitute.org/gsea/msigdb/cards/GILDEA_METASTASIS 28 -0.569022 -1.66875 0 0.0857892 0 down vs 1ZHAN_EARLY_DIFFERENTIATION_GENES_DN http://www.broadinstitute.org/gsea/msigdb/cards/ZHAN_EARLY_DIFFERENTIATION_GENES_DN 42 -0.476357 -1.6701 0 0.0858527 0 down vs 1GHANDHI_DIRECT_IRRADIATION_UP http://www.broadinstitute.org/gsea/msigdb/cards/GHANDHI_DIRECT_IRRADIATION_UP 108 -0.423122 -1.67415 0.0833333 0.0863846 0 down vs 1SMIRNOV_RESPONSE_TO_IR_2HR_UP http://www.broadinstitute.org/gsea/msigdb/cards/SMIRNOV_RESPONSE_TO_IR_2HR_UP 50 -0.448311 -1.67024 0 0.086438 0 down vs 1KOKKINAKIS_METHIONINE_DEPRIVATION_96HR_DN http://www.broadinstitute.org/gsea/msigdb/cards/KOKKINAKIS_METHIONINE_DEPRIVATION_96HR_DN 75 -0.36023 -1.67514 0 0.0865286 0 down vs 1HUPER_BREAST_BASAL_VS_LUMINAL_UP http://www.broadinstitute.org/gsea/msigdb/cards/HUPER_BREAST_BASAL_VS_LUMINAL_UP 53 -0.619814 -1.67479 0.03125 0.0865906 0 down vs 1ONGUSAHA_TP53_TARGETS http://www.broadinstitute.org/gsea/msigdb/cards/ONGUSAHA_TP53_TARGETS 38 -0.412086 -1.65882 0 0.0917284 0 down vs 1AMUNDSON_DNA_DAMAGE_RESPONSE_TP53 http://www.broadinstitute.org/gsea/msigdb/cards/AMUNDSON_DNA_DAMAGE_RESPONSE_TP53 16 -0.564952 -1.65516 0.027027 0.0934839 0 down vs 1GRUETZMANN_PANCREATIC_CANCER_UP http://www.broadinstitute.org/gsea/msigdb/cards/GRUETZMANN_PANCREATIC_CANCER_UP 352 -0.332787 -1.65531 0 0.0939739 0 down vs 1DIRMEIER_LMP1_RESPONSE_LATE_UP http://www.broadinstitute.org/gsea/msigdb/cards/DIRMEIER_LMP1_RESPONSE_LATE_UP 57 -0.415164 -1.65542 0 0.0945958 0 down vs 1MISSIAGLIA_REGULATED_BY_METHYLATION_UP http://www.broadinstitute.org/gsea/msigdb/cards/MISSIAGLIA_REGULATED_BY_METHYLATION_UP 125 -0.403172 -1.65003 0 0.0969975 0 down vs 1WIELAND_UP_BY_HBV_INFECTION http://www.broadinstitute.org/gsea/msigdb/cards/WIELAND_UP_BY_HBV_INFECTION 99 -0.618864 -1.64713 0.03125 0.0972782 0 down vs 1VERNELL_RETINOBLASTOMA_PATHWAY_UP http://www.broadinstitute.org/gsea/msigdb/cards/VERNELL_RETINOBLASTOMA_PATHWAY_UP 69 -0.598942 -1.64802 0 0.0973054 0 down vs 1SUNG_METASTASIS_STROMA_DN http://www.broadinstitute.org/gsea/msigdb/cards/SUNG_METASTASIS_STROMA_DN 52 -0.482517 -1.64811 0 0.0980539 0 down vs 1MARKS_HDAC_TARGETS_UP http://www.broadinstitute.org/gsea/msigdb/cards/MARKS_HDAC_TARGETS_UP 23 -0.501225 -1.6403 0 0.102889 0 down vs 1KAMIKUBO_MYELOID_CEBPA_NETWORK http://www.broadinstitute.org/gsea/msigdb/cards/KAMIKUBO_MYELOID_CEBPA_NETWORK 27 -0.470727 -1.63657 0.0588235 0.105502 0 down vs 1ONDER_CDH1_TARGETS_3_DN http://www.broadinstitute.org/gsea/msigdb/cards/ONDER_CDH1_TARGETS_3_DN 58 -0.49144 -1.63269 0.04 0.109005 0 down vs 1HAN_JNK_SINGALING_UP http://www.broadinstitute.org/gsea/msigdb/cards/HAN_JNK_SINGALING_UP 35 -0.439917 -1.63338 0.0285714 0.109118 0 down vs 1ZHANG_ANTIVIRAL_RESPONSE_TO_RIBAVIRIN_UP http://www.broadinstitute.org/gsea/msigdb/cards/ZHANG_ANTIVIRAL_RESPONSE_TO_RIBAVIRIN_UP 27 -0.473147 -1.63068 0.0322581 0.110033 0 down vs 1KOKKINAKIS_METHIONINE_DEPRIVATION_48HR_UP http://www.broadinstitute.org/gsea/msigdb/cards/KOKKINAKIS_METHIONINE_DEPRIVATION_48HR_UP 127 -0.366092 -1.61794 0 0.113843 0 down vs 1TAVOR_CEBPA_TARGETS_UP http://www.broadinstitute.org/gsea/msigdb/cards/TAVOR_CEBPA_TARGETS_UP 48 -0.441673 -1.61953 0 0.114556 0 down vs 1ZHANG_ANTIVIRAL_RESPONSE_TO_RIBAVIRIN_DN http://www.broadinstitute.org/gsea/msigdb/cards/ZHANG_ANTIVIRAL_RESPONSE_TO_RIBAVIRIN_DN 50 -0.485642 -1.61801 0.0333333 0.114628 0 down vs 1NIELSEN_SYNOVIAL_SARCOMA_DN http://www.broadinstitute.org/gsea/msigdb/cards/NIELSEN_SYNOVIAL_SARCOMA_DN 20 -0.666714 -1.62509 0.0540541 0.114896 0 down vs 1BURTON_ADIPOGENESIS_PEAK_AT_16HR http://www.broadinstitute.org/gsea/msigdb/cards/BURTON_ADIPOGENESIS_PEAK_AT_16HR 40 -0.544087 -1.62135 0 0.115221 0 down vs 1LIANG_SILENCED_BY_METHYLATION_UP http://www.broadinstitute.org/gsea/msigdb/cards/LIANG_SILENCED_BY_METHYLATION_UP 32 -0.494439 -1.61977 0 0.115357 0 down vs 1SCHAVOLT_TARGETS_OF_TP53_AND_TP63 http://www.broadinstitute.org/gsea/msigdb/cards/SCHAVOLT_TARGETS_OF_TP53_AND_TP63 16 -0.548737 -1.62157 0 0.115709 0 down vs 1RICKMAN_TUMOR_DIFFERENTIATED_WELL_VS_MODERATELY_DN http://www.broadinstitute.org/gsea/msigdb/cards/RICKMAN_TUMOR_DIFFERENTIATED_WELL_VS_MODERATELY_DN 109 -0.577804 -1.62017 0 0.11573 0 down vs 1CHEOK_RESPONSE_TO_HD_MTX_UP http://www.broadinstitute.org/gsea/msigdb/cards/CHEOK_RESPONSE_TO_HD_MTX_UP 21 -0.464324 -1.62176 0.0333333 0.116317 0 down vs 1DUTERTRE_ESTRADIOL_RESPONSE_24HR_UP http://www.broadinstitute.org/gsea/msigdb/cards/DUTERTRE_ESTRADIOL_RESPONSE_24HR_UP 318 -0.500713 -1.61408 0 0.117054 0 down vs 1ISHIDA_E2F_TARGETS http://www.broadinstitute.org/gsea/msigdb/cards/ISHIDA_E2F_TARGETS 52 -0.599539 -1.61443 0.030303 0.117213 0 down vs 1HINATA_NFKB_TARGETS_KERATINOCYTE_DN http://www.broadinstitute.org/gsea/msigdb/cards/HINATA_NFKB_TARGETS_KERATINOCYTE_DN 22 -0.54656 -1.60834 0 0.122245 0 down vs 1WANG_TNF_TARGETS http://www.broadinstitute.org/gsea/msigdb/cards/WANG_TNF_TARGETS 24 -0.501566 -1.60361 0 0.124251 0 down vs 1RICKMAN_HEAD_AND_NECK_CANCER_C http://www.broadinstitute.org/gsea/msigdb/cards/RICKMAN_HEAD_AND_NECK_CANCER_C 109 -0.4695 -1.60423 0.0322581 0.12466 0 down vs 1CROMER_TUMORIGENESIS_DN http://www.broadinstitute.org/gsea/msigdb/cards/CROMER_TUMORIGENESIS_DN 51 -0.445768 -1.60451 0.027027 0.125075 0 down vs 1LEE_LIVER_CANCER_ACOX1_UP http://www.broadinstitute.org/gsea/msigdb/cards/LEE_LIVER_CANCER_ACOX1_UP 62 -0.35803 -1.59517 0.0454545 0.127329 0 down vs 1GENTLES_LEUKEMIC_STEM_CELL_DN http://www.broadinstitute.org/gsea/msigdb/cards/GENTLES_LEUKEMIC_STEM_CELL_DN 19 -0.510812 -1.59437 0 0.127411 0 down vs 1PRAMOONJAGO_SOX4_TARGETS_UP http://www.broadinstitute.org/gsea/msigdb/cards/PRAMOONJAGO_SOX4_TARGETS_UP 52 -0.44295 -1.5973 0.0344828 0.127474 0 down vs 1LOPES_METHYLATED_IN_COLON_CANCER_DN http://www.broadinstitute.org/gsea/msigdb/cards/LOPES_METHYLATED_IN_COLON_CANCER_DN 28 -0.426303 -1.59666 0.047619 0.127558 0 down vs 1BASAKI_YBX1_TARGETS_UP http://www.broadinstitute.org/gsea/msigdb/cards/BASAKI_YBX1_TARGETS_UP 279 -0.3786 -1.59559 0.05 0.12764 0 down vs 1GALINDO_IMMUNE_RESPONSE_TO_ENTEROTOXIN http://www.broadinstitute.org/gsea/msigdb/cards/GALINDO_IMMUNE_RESPONSE_TO_ENTEROTOXIN 84 -0.438782 -1.5987 0 0.128136 0 down vs 1HELLER_SILENCED_BY_METHYLATION_UP http://www.broadinstitute.org/gsea/msigdb/cards/HELLER_SILENCED_BY_METHYLATION_UP 277 -0.368475 -1.59742 0.0384615 0.128296 0 down vs 1KANNAN_TP53_TARGETS_UP http://www.broadinstitute.org/gsea/msigdb/cards/KANNAN_TP53_TARGETS_UP 57 -0.391312 -1.59776 0 0.128521 0 down vs 1SONG_TARGETS_OF_IE86_CMV_PROTEIN http://www.broadinstitute.org/gsea/msigdb/cards/SONG_TARGETS_OF_IE86_CMV_PROTEIN 60 -0.549571 -1.58892 0 0.131471 0 down vs 1AMIT_EGF_RESPONSE_60_HELA http://www.broadinstitute.org/gsea/msigdb/cards/AMIT_EGF_RESPONSE_60_HELA 44 -0.496526 -1.58839 0.0434783 0.131624 0 down vs 1LINDSTEDT_DENDRITIC_CELL_MATURATION_A http://www.broadinstitute.org/gsea/msigdb/cards/LINDSTEDT_DENDRITIC_CELL_MATURATION_A 67 -0.47346 -1.58556 0 0.131746 0 down vs 1SESTO_RESPONSE_TO_UV_C5 http://www.broadinstitute.org/gsea/msigdb/cards/SESTO_RESPONSE_TO_UV_C5 46 -0.491904 -1.58673 0.107143 0.131924 0 down vs 1WU_HBX_TARGETS_1_UP http://www.broadinstitute.org/gsea/msigdb/cards/WU_HBX_TARGETS_1_UP 16 -0.481465 -1.58738 0.0357143 0.132062 0 down vs 1RUIZ_TNC_TARGETS_DN http://www.broadinstitute.org/gsea/msigdb/cards/RUIZ_TNC_TARGETS_DN 138 -0.435086 -1.58557 0 0.132545 0 down vs 1MURATA_VIRULENCE_OF_H_PILORI http://www.broadinstitute.org/gsea/msigdb/cards/MURATA_VIRULENCE_OF_H_PILORI 24 -0.47935 -1.58304 0 0.13367 0 down vs 1FERRARI_RESPONSE_TO_FENRETINIDE_UP http://www.broadinstitute.org/gsea/msigdb/cards/FERRARI_RESPONSE_TO_FENRETINIDE_UP 22 -0.480627 -1.58026 0.0606061 0.134343 0 down vs 1RICKMAN_HEAD_AND_NECK_CANCER_E http://www.broadinstitute.org/gsea/msigdb/cards/RICKMAN_HEAD_AND_NECK_CANCER_E 89 -0.444108 -1.58046 0.0512821 0.134676 0 down vs 1KOHOUTEK_CCNT1_TARGETS http://www.broadinstitute.org/gsea/msigdb/cards/KOHOUTEK_CCNT1_TARGETS 48 -0.387606 -1.58144 0 0.134734 0 down vs 1GRAHAM_NORMAL_QUIESCENT_VS_NORMAL_DIVIDING_DN http://www.broadinstitute.org/gsea/msigdb/cards/GRAHAM_NORMAL_QUIESCENT_VS_NORMAL_DIVIDING_DN 87 -0.539733 -1.5776 0.0384615 0.137121 0 down vs 1MATTHEWS_SKIN_CARCINOGENESIS_VIA_JUN http://www.broadinstitute.org/gsea/msigdb/cards/MATTHEWS_SKIN_CARCINOGENESIS_VIA_JUN 16 -0.528599 -1.57021 0.03125 0.138759 0 down vs 1SARRIO_EPITHELIAL_MESENCHYMAL_TRANSITION_UP http://www.broadinstitute.org/gsea/msigdb/cards/SARRIO_EPITHELIAL_MESENCHYMAL_TRANSITION_UP 175 -0.421557 -1.57465 0 0.139237 0 down vs 1BOHN_PRIMARY_IMMUNODEFICIENCY_SYNDROM_DN http://www.broadinstitute.org/gsea/msigdb/cards/BOHN_PRIMARY_IMMUNODEFICIENCY_SYNDROM_DN 38 -0.426763 -1.57364 0.03125 0.139335 0 down vs 1SIMBULAN_UV_RESPONSE_NORMAL_DN http://www.broadinstitute.org/gsea/msigdb/cards/SIMBULAN_UV_RESPONSE_NORMAL_DN 33 -0.529899 -1.57033 0 0.139363 0 down vs 1HOFMANN_CELL_LYMPHOMA_DN http://www.broadinstitute.org/gsea/msigdb/cards/HOFMANN_CELL_LYMPHOMA_DN 39 -0.408565 -1.57491 0 0.139775 0 down vs 1MAGRANGEAS_MULTIPLE_MYELOMA_IGG_VS_IGA_UP http://www.broadinstitute.org/gsea/msigdb/cards/MAGRANGEAS_MULTIPLE_MYELOMA_IGG_VS_IGA_UP 22 -0.446056 -1.5721 0 0.139789 0 down vs 1FRIDMAN_IMMORTALIZATION_DN http://www.broadinstitute.org/gsea/msigdb/cards/FRIDMAN_IMMORTALIZATION_DN 33 -0.437585 -1.57054 0 0.139886 0 down vs 1JACKSON_DNMT1_TARGETS_UP http://www.broadinstitute.org/gsea/msigdb/cards/JACKSON_DNMT1_TARGETS_UP 76 -0.364416 -1.57075 0.0526316 0.140059 0 down vs 1NUTT_GBM_VS_AO_GLIOMA_DN http://www.broadinstitute.org/gsea/msigdb/cards/NUTT_GBM_VS_AO_GLIOMA_DN 44 0.643909 2.12068 0 0.140729 0 up vs 1CHEN_LVAD_SUPPORT_OF_FAILING_HEART_UP http://www.broadinstitute.org/gsea/msigdb/cards/CHEN_LVAD_SUPPORT_OF_FAILING_HEART_UP 101 -0.371007 -1.5619 0 0.148576 0 down vs 1SHIN_B_CELL_LYMPHOMA_CLUSTER_8 http://www.broadinstitute.org/gsea/msigdb/cards/SHIN_B_CELL_LYMPHOMA_CLUSTER_8 36 -0.467176 -1.56014 0.0625 0.149827 0 down vs 1NAGASHIMA_EGF_SIGNALING_UP http://www.broadinstitute.org/gsea/msigdb/cards/NAGASHIMA_EGF_SIGNALING_UP 57 -0.496775 -1.55857 0.0555556 0.151235 0 down vs 1BOYAULT_LIVER_CANCER_SUBCLASS_G6_DN http://www.broadinstitute.org/gsea/msigdb/cards/BOYAULT_LIVER_CANCER_SUBCLASS_G6_DN 18 -0.51536 -1.55607 0.08 0.153055 0 down vs 1ENK_UV_RESPONSE_EPIDERMIS_UP http://www.broadinstitute.org/gsea/msigdb/cards/ENK_UV_RESPONSE_EPIDERMIS_UP 283 -0.296503 -1.55237 0 0.153155 0 down vs 1FARMER_BREAST_CANCER_CLUSTER_1 http://www.broadinstitute.org/gsea/msigdb/cards/FARMER_BREAST_CANCER_CLUSTER_1 43 -0.667433 -1.55492 0.0222222 0.153412 0 down vs 1CHIARETTI_T_ALL_RELAPSE_PROGNOSIS http://www.broadinstitute.org/gsea/msigdb/cards/CHIARETTI_T_ALL_RELAPSE_PROGNOSIS 19 -0.548669 -1.55252 0.0322581 0.153804 0 down vs 1ACEVEDO_FGFR1_TARGETS_IN_PROSTATE_CANCER_MODEL_UP http://www.broadinstitute.org/gsea/msigdb/cards/ACEVEDO_FGFR1_TARGETS_IN_PROSTATE_CANCER_MODEL_UP 281 -0.330732 -1.55373 0 0.154018 0 down vs 1MAHADEVAN_RESPONSE_TO_MP470_DN http://www.broadinstitute.org/gsea/msigdb/cards/MAHADEVAN_RESPONSE_TO_MP470_DN 19 -0.566544 -1.55281 0.0487805 0.154041 0 down vs 1LIU_IL13_PRIMING_MODEL http://www.broadinstitute.org/gsea/msigdb/cards/LIU_IL13_PRIMING_MODEL 15 -0.469032 -1.55301 0.0294118 0.154366 0 down vs 1GRAHAM_CML_DIVIDING_VS_NORMAL_QUIESCENT_UP http://www.broadinstitute.org/gsea/msigdb/cards/GRAHAM_CML_DIVIDING_VS_NORMAL_QUIESCENT_UP 179 -0.399935 -1.54688 0.0769231 0.158667 0 down vs 1PARK_TRETINOIN_RESPONSE_AND_PML_RARA_FUSION http://www.broadinstitute.org/gsea/msigdb/cards/PARK_TRETINOIN_RESPONSE_AND_PML_RARA_FUSION 30 -0.509389 -1.54736 0.0645161 0.158844 0 down vs 1GU_PDEF_TARGETS_DN http://www.broadinstitute.org/gsea/msigdb/cards/GU_PDEF_TARGETS_DN 39 -0.373051 -1.54367 0.0434783 0.161583 0 down vs 1COLDREN_GEFITINIB_RESISTANCE_UP http://www.broadinstitute.org/gsea/msigdb/cards/COLDREN_GEFITINIB_RESISTANCE_UP 80 0.630548 2.09589 0.0126582 0.16238 0 up vs 1PHONG_TNF_RESPONSE_NOT_VIA_P38 http://www.broadinstitute.org/gsea/msigdb/cards/PHONG_TNF_RESPONSE_NOT_VIA_P38 334 -0.3323 -1.54169 0 0.163417 0 down vs 1SMIRNOV_RESPONSE_TO_IR_6HR_UP http://www.broadinstitute.org/gsea/msigdb/cards/SMIRNOV_RESPONSE_TO_IR_6HR_UP 158 -0.372177 -1.5399 0 0.164304 0 down vs 1DELPUECH_FOXO3_TARGETS_UP http://www.broadinstitute.org/gsea/msigdb/cards/DELPUECH_FOXO3_TARGETS_UP 68 -0.371706 -1.54035 0 0.164507 0 down vs 1CROONQUIST_NRAS_SIGNALING_DN http://www.broadinstitute.org/gsea/msigdb/cards/CROONQUIST_NRAS_SIGNALING_DN 72 -0.564338 -1.53904 0.0666667 0.164901 0 down vs 1TSAI_RESPONSE_TO_IONIZING_RADIATION http://www.broadinstitute.org/gsea/msigdb/cards/TSAI_RESPONSE_TO_IONIZING_RADIATION 149 -0.347516 -1.53676 0 0.166046 0 down vs 1ENGELMANN_CANCER_PROGENITORS_DN http://www.broadinstitute.org/gsea/msigdb/cards/ENGELMANN_CANCER_PROGENITORS_DN 68 -0.340281 -1.53544 0 0.166497 0 down vs 1ZHOU_CELL_CYCLE_GENES_IN_IR_RESPONSE_6HR http://www.broadinstitute.org/gsea/msigdb/cards/ZHOU_CELL_CYCLE_GENES_IN_IR_RESPONSE_6HR 82 -0.546551 -1.53438 0.0333333 0.166836 0 down vs 1LY_AGING_PREMATURE_DN http://www.broadinstitute.org/gsea/msigdb/cards/LY_AGING_PREMATURE_DN 30 -0.474193 -1.53556 0 0.167101 0 down vs 1TAKEDA_TARGETS_OF_NUP98_HOXA9_FUSION_10D_UP http://www.broadinstitute.org/gsea/msigdb/cards/TAKEDA_TARGETS_OF_NUP98_HOXA9_FUSION_10D_UP 187 -0.359592 -1.53296 0.0322581 0.167327 0 down vs 1MURAKAMI_UV_RESPONSE_24HR http://www.broadinstitute.org/gsea/msigdb/cards/MURAKAMI_UV_RESPONSE_24HR 20 -0.485075 -1.53116 0.0294118 0.16747 0 down vs 1AMIT_EGF_RESPONSE_120_HELA http://www.broadinstitute.org/gsea/msigdb/cards/AMIT_EGF_RESPONSE_120_HELA 67 -0.377235 -1.53131 0.1 0.168064 0 down vs 1RASHI_RESPONSE_TO_IONIZING_RADIATION_1 http://www.broadinstitute.org/gsea/msigdb/cards/RASHI_RESPONSE_TO_IONIZING_RADIATION_1 44 -0.336166 -1.5297 0.047619 0.168329 0 down vs 1YAO_TEMPORAL_RESPONSE_TO_PROGESTERONE_CLUSTER_6 http://www.broadinstitute.org/gsea/msigdb/cards/YAO_TEMPORAL_RESPONSE_TO_PROGESTERONE_CLUSTER_6 74 -0.325675 -1.53153 0 0.168509 0 down vs 1FURUKAWA_DUSP6_TARGETS_PCI35_DN http://www.broadinstitute.org/gsea/msigdb/cards/FURUKAWA_DUSP6_TARGETS_PCI35_DN 69 -0.462613 -1.52428 0.0769231 0.169614 0 down vs 1LEE_LIVER_CANCER_DENA_UP http://www.broadinstitute.org/gsea/msigdb/cards/LEE_LIVER_CANCER_DENA_UP 59 -0.333501 -1.52621 0.0909091 0.169732 0 down vs 1BROWNE_HCMV_INFECTION_8HR_DN http://www.broadinstitute.org/gsea/msigdb/cards/BROWNE_HCMV_INFECTION_8HR_DN 46 -0.386126 -1.52186 0.030303 0.169873 0 down vs 1GRAHAM_NORMAL_QUIESCENT_VS_NORMAL_DIVIDING_UP http://www.broadinstitute.org/gsea/msigdb/cards/GRAHAM_NORMAL_QUIESCENT_VS_NORMAL_DIVIDING_UP 66 -0.39509 -1.5267 0.0666667 0.170023 0 down vs 1KOBAYASHI_EGFR_SIGNALING_24HR_DN http://www.broadinstitute.org/gsea/msigdb/cards/KOBAYASHI_EGFR_SIGNALING_24HR_DN 245 -0.454334 -1.52464 0.0714286 0.170049 0 down vs 1WARTERS_IR_RESPONSE_5GY http://www.broadinstitute.org/gsea/msigdb/cards/WARTERS_IR_RESPONSE_5GY 47 -0.386711 -1.52294 0.0454545 0.170074 0 down vs 1VANTVEER_BREAST_CANCER_ESR1_DN http://www.broadinstitute.org/gsea/msigdb/cards/VANTVEER_BREAST_CANCER_ESR1_DN 229 -0.313488 -1.52531 0 0.17019 0 down vs 1AMIT_EGF_RESPONSE_60_MCF10A http://www.broadinstitute.org/gsea/msigdb/cards/AMIT_EGF_RESPONSE_60_MCF10A 38 -0.451838 -1.52314 0.0333333 0.170287 0 down vs 1DELYS_THYROID_CANCER_UP http://www.broadinstitute.org/gsea/msigdb/cards/DELYS_THYROID_CANCER_UP 441 -0.306572 -1.52092 0 0.170316 0 down vs 1BERTUCCI_MEDULLARY_VS_DUCTAL_BREAST_CANCER_UP http://www.broadinstitute.org/gsea/msigdb/cards/BERTUCCI_MEDULLARY_VS_DUCTAL_BREAST_CANCER_UP 199 -0.392891 -1.52187 0.0833333 0.170667 0 down vs 1PEDERSEN_METASTASIS_BY_ERBB2_ISOFORM_4 http://www.broadinstitute.org/gsea/msigdb/cards/PEDERSEN_METASTASIS_BY_ERBB2_ISOFORM_4 108 -0.320031 -1.52686 0 0.170697 0 down vs 1SENGUPTA_NASOPHARYNGEAL_CARCINOMA_WITH_LMP1_DN http://www.broadinstitute.org/gsea/msigdb/cards/SENGUPTA_NASOPHARYNGEAL_CARCINOMA_WITH_LMP1_DN 170 -0.323579 -1.51839 0 0.17084 0 down vs 1TIAN_TNF_SIGNALING_NOT_VIA_NFKB http://www.broadinstitute.org/gsea/msigdb/cards/TIAN_TNF_SIGNALING_NOT_VIA_NFKB 21 -0.540362 -1.51788 0.0285714 0.170916 0 down vs 1QI_PLASMACYTOMA_UP http://www.broadinstitute.org/gsea/msigdb/cards/QI_PLASMACYTOMA_UP 256 -0.418945 -1.51859 0.0714286 0.171264 0 down vs 1ICHIBA_GRAFT_VERSUS_HOST_DISEASE_D7_UP http://www.broadinstitute.org/gsea/msigdb/cards/ICHIBA_GRAFT_VERSUS_HOST_DISEASE_D7_UP 105 -0.490582 -1.51991 0.0666667 0.171331 0 down vs 1SCIAN_INVERSED_TARGETS_OF_TP53_AND_TP73_DN http://www.broadinstitute.org/gsea/msigdb/cards/SCIAN_INVERSED_TARGETS_OF_TP53_AND_TP73_DN 31 -0.386853 -1.51932 0.0526316 0.171476 0 down vs 1SCHURINGA_STAT5A_TARGETS_DN http://www.broadinstitute.org/gsea/msigdb/cards/SCHURINGA_STAT5A_TARGETS_DN 16 -0.539777 -1.51495 0.0625 0.173453 0 down vs 1ABE_VEGFA_TARGETS_2HR http://www.broadinstitute.org/gsea/msigdb/cards/ABE_VEGFA_TARGETS_2HR 33 -0.406852 -1.51299 0.0344828 0.17364 0 down vs 1PHONG_TNF_RESPONSE_VIA_P38_PARTIAL http://www.broadinstitute.org/gsea/msigdb/cards/PHONG_TNF_RESPONSE_VIA_P38_PARTIAL 159 -0.331795 -1.51418 0.0689655 0.173866 0 down vs 1KUROZUMI_RESPONSE_TO_ONCOCYTIC_VIRUS http://www.broadinstitute.org/gsea/msigdb/cards/KUROZUMI_RESPONSE_TO_ONCOCYTIC_VIRUS 44 -0.592875 -1.51304 0.025641 0.174275 0 down vs 1VALK_AML_CLUSTER_4 http://www.broadinstitute.org/gsea/msigdb/cards/VALK_AML_CLUSTER_4 29 -0.446889 -1.50872 0.1 0.178579 0 down vs 1JISON_SICKLE_CELL_DISEASE_UP http://www.broadinstitute.org/gsea/msigdb/cards/JISON_SICKLE_CELL_DISEASE_UP 178 -0.332595 -1.50921 0.0588235 0.178678 0 down vs 1BOYAULT_LIVER_CANCER_SUBCLASS_G5_DN http://www.broadinstitute.org/gsea/msigdb/cards/BOYAULT_LIVER_CANCER_SUBCLASS_G5_DN 26 -0.523126 -1.50704 0.0625 0.179102 0 down vs 1MARKEY_RB1_ACUTE_LOF_UP http://www.broadinstitute.org/gsea/msigdb/cards/MARKEY_RB1_ACUTE_LOF_UP 211 -0.417876 -1.50717 0.0714286 0.179679 0 down vs 1HOSHIDA_LIVER_CANCER_SUBCLASS_S1 http://www.broadinstitute.org/gsea/msigdb/cards/HOSHIDA_LIVER_CANCER_SUBCLASS_S1 235 -0.347693 -1.50726 0.0714286 0.180193 0 down vs 1PEDERSEN_METASTASIS_BY_ERBB2_ISOFORM_7 http://www.broadinstitute.org/gsea/msigdb/cards/PEDERSEN_METASTASIS_BY_ERBB2_ISOFORM_7 385 -0.299609 -1.50451 0 0.181234 0 down vs 1ZHANG_TLX_TARGETS_DN http://www.broadinstitute.org/gsea/msigdb/cards/ZHANG_TLX_TARGETS_DN 88 -0.569165 -1.50087 0.04 0.185369 0 down vs 1WEST_ADRENOCORTICAL_TUMOR_MARKERS_UP http://www.broadinstitute.org/gsea/msigdb/cards/WEST_ADRENOCORTICAL_TUMOR_MARKERS_UP 21 -0.528186 -1.49927 0.0689655 0.185854 0 down vs 1TAKEDA_TARGETS_OF_NUP98_HOXA9_FUSION_16D_UP http://www.broadinstitute.org/gsea/msigdb/cards/TAKEDA_TARGETS_OF_NUP98_HOXA9_FUSION_16D_UP 169 -0.374696 -1.4984 0.0333333 0.186126 0 down vs 1FOSTER_TOLERANT_MACROPHAGE_DN http://www.broadinstitute.org/gsea/msigdb/cards/FOSTER_TOLERANT_MACROPHAGE_DN 399 -0.33695 -1.49952 0.117647 0.18645 0 down vs 1PEDERSEN_TARGETS_OF_611CTF_ISOFORM_OF_ERBB2 http://www.broadinstitute.org/gsea/msigdb/cards/PEDERSEN_TARGETS_OF_611CTF_ISOFORM_OF_ERBB2 74 -0.417688 -1.49661 0.0434783 0.187654 0 down vs 1CHEOK_RESPONSE_TO_MERCAPTOPURINE_DN http://www.broadinstitute.org/gsea/msigdb/cards/CHEOK_RESPONSE_TO_MERCAPTOPURINE_DN 22 -0.423201 -1.49484 0.0869565 0.188708 0 down vs 1TAVOR_CEBPA_TARGETS_DN http://www.broadinstitute.org/gsea/msigdb/cards/TAVOR_CEBPA_TARGETS_DN 30 -0.403617 -1.49039 0.0540541 0.193101 0 down vs 1PLASARI_TGFB1_TARGETS_10HR_UP http://www.broadinstitute.org/gsea/msigdb/cards/PLASARI_TGFB1_TARGETS_10HR_UP 198 -0.327859 -1.48603 0.0434783 0.197783 0 down vs 1HAHTOLA_MYCOSIS_FUNGOIDES_SKIN_DN http://www.broadinstitute.org/gsea/msigdb/cards/HAHTOLA_MYCOSIS_FUNGOIDES_SKIN_DN 26 -0.428839 -1.48019 0.0434783 0.202309 0 down vs 1FARMER_BREAST_CANCER_CLUSTER_3 http://www.broadinstitute.org/gsea/msigdb/cards/FARMER_BREAST_CANCER_CLUSTER_3 16 -0.548883 -1.48057 0.0277778 0.202627 0 down vs 1TAKEDA_TARGETS_OF_NUP98_HOXA9_FUSION_3D_DN http://www.broadinstitute.org/gsea/msigdb/cards/TAKEDA_TARGETS_OF_NUP98_HOXA9_FUSION_3D_DN 31 -0.454562 -1.48117 0.125 0.202754 0 down vs 1TAKEDA_TARGETS_OF_NUP98_HOXA9_FUSION_8D_UP http://www.broadinstitute.org/gsea/msigdb/cards/TAKEDA_TARGETS_OF_NUP98_HOXA9_FUSION_8D_UP 152 -0.340652 -1.47891 0.0357143 0.203141 0 down vs 1BROWNE_HCMV_INFECTION_2HR_DN http://www.broadinstitute.org/gsea/msigdb/cards/BROWNE_HCMV_INFECTION_2HR_DN 48 -0.444178 -1.48163 0.0555556 0.203272 0 down vs 1YANG_BREAST_CANCER_ESR1_BULK_DN http://www.broadinstitute.org/gsea/msigdb/cards/YANG_BREAST_CANCER_ESR1_BULK_DN 22 -0.413442 -1.47806 0.0322581 0.203395 0 down vs 1HINATA_NFKB_TARGETS_FIBROBLAST_UP http://www.broadinstitute.org/gsea/msigdb/cards/HINATA_NFKB_TARGETS_FIBROBLAST_UP 84 -0.366451 -1.48122 0 0.203401 0 down vs 1XU_HGF_SIGNALING_NOT_VIA_AKT1_6HR http://www.broadinstitute.org/gsea/msigdb/cards/XU_HGF_SIGNALING_NOT_VIA_AKT1_6HR 26 -0.43816 -1.47521 0.0740741 0.205534 0 down vs 1GRAESSMANN_RESPONSE_TO_MC_AND_SERUM_DEPRIVATION_UP http://www.broadinstitute.org/gsea/msigdb/cards/GRAESSMANN_RESPONSE_TO_MC_AND_SERUM_DEPRIVATION_UP 206 -0.335598 -1.47522 0 0.206366 0 down vs 1CHIANG_LIVER_CANCER_SUBCLASS_INTERFERON_DN http://www.broadinstitute.org/gsea/msigdb/cards/CHIANG_LIVER_CANCER_SUBCLASS_INTERFERON_DN 48 0.610687 2.1227 0 0.207034 0 up vs 1LEE_LIVER_CANCER_E2F1_UP http://www.broadinstitute.org/gsea/msigdb/cards/LEE_LIVER_CANCER_E2F1_UP 62 -0.356661 -1.47283 0 0.208598 0 down vs 1MAHADEVAN_RESPONSE_TO_MP470_UP http://www.broadinstitute.org/gsea/msigdb/cards/MAHADEVAN_RESPONSE_TO_MP470_UP 19 -0.547177 -1.47111 0.0833333 0.208803 0 down vs 1MORI_LARGE_PRE_BII_LYMPHOCYTE_DN http://www.broadinstitute.org/gsea/msigdb/cards/MORI_LARGE_PRE_BII_LYMPHOCYTE_DN 57 -0.505387 -1.47146 0.108108 0.209201 0 down vs 1COLDREN_GEFITINIB_RESISTANCE_DN http://www.broadinstitute.org/gsea/msigdb/cards/COLDREN_GEFITINIB_RESISTANCE_DN 223 -0.405802 -1.47063 0.05 0.209338 0 down vs 1MCBRYAN_PUBERTAL_BREAST_6_7WK_DN http://www.broadinstitute.org/gsea/msigdb/cards/MCBRYAN_PUBERTAL_BREAST_6_7WK_DN 79 -0.310368 -1.46822 0.0555556 0.211721 0 down vs 1MAHAJAN_RESPONSE_TO_IL1A_UP http://www.broadinstitute.org/gsea/msigdb/cards/MAHAJAN_RESPONSE_TO_IL1A_UP 80 -0.357029 -1.46576 0.0333333 0.214349 0 down vs 1SHIN_B_CELL_LYMPHOMA_CLUSTER_5 http://www.broadinstitute.org/gsea/msigdb/cards/SHIN_B_CELL_LYMPHOMA_CLUSTER_5 18 -0.430046 -1.46525 0.08 0.214366 0 down vs 1GUENTHER_GROWTH_SPHERICAL_VS_ADHERENT_DN http://www.broadinstitute.org/gsea/msigdb/cards/GUENTHER_GROWTH_SPHERICAL_VS_ADHERENT_DN 26 -0.462071 -1.46591 0.0714286 0.214824 0 down vs 1TONKS_TARGETS_OF_RUNX1_RUNX1T1_FUSION_HSC_DN http://www.broadinstitute.org/gsea/msigdb/cards/TONKS_TARGETS_OF_RUNX1_RUNX1T1_FUSION_HSC_DN 181 -0.355673 -1.46343 0.0714286 0.214884 0 down vs 1BEGUM_TARGETS_OF_PAX3_FOXO1_FUSION_DN http://www.broadinstitute.org/gsea/msigdb/cards/BEGUM_TARGETS_OF_PAX3_FOXO1_FUSION_DN 45 -0.422033 -1.46388 0.0689655 0.215052 0 down vs 1NOJIMA_SFRP2_TARGETS_UP http://www.broadinstitute.org/gsea/msigdb/cards/NOJIMA_SFRP2_TARGETS_UP 31 -0.426709 -1.46249 0 0.21514 0 down vs 1SCHOEN_NFKB_SIGNALING http://www.broadinstitute.org/gsea/msigdb/cards/SCHOEN_NFKB_SIGNALING 34 -0.481508 -1.46003 0 0.217262 0 down vs 1NAKAYAMA_SOFT_TISSUE_TUMORS_PCA1_UP http://www.broadinstitute.org/gsea/msigdb/cards/NAKAYAMA_SOFT_TISSUE_TUMORS_PCA1_UP 73 -0.520162 -1.46026 0.05 0.217737 0 down vs 1FRIDMAN_SENESCENCE_UP http://www.broadinstitute.org/gsea/msigdb/cards/FRIDMAN_SENESCENCE_UP 77 -0.374159 -1.45852 0.0434783 0.2184 0 down vs 1SATO_SILENCED_BY_METHYLATION_IN_PANCREATIC_CANCER_2 http://www.broadinstitute.org/gsea/msigdb/cards/SATO_SILENCED_BY_METHYLATION_IN_PANCREATIC_CANCER_2 46 -0.318366 -1.45789 0.107143 0.21852 0 down vs 1BOYAULT_LIVER_CANCER_SUBCLASS_G6_UP http://www.broadinstitute.org/gsea/msigdb/cards/BOYAULT_LIVER_CANCER_SUBCLASS_G6_UP 61 0.578583 2.0159 0 0.219213 0 up vs 1NAKAYAMA_SOFT_TISSUE_TUMORS_PCA1_DN http://www.broadinstitute.org/gsea/msigdb/cards/NAKAYAMA_SOFT_TISSUE_TUMORS_PCA1_DN 74 0.580252 2.03215 0 0.219213 0 up vs 1MURAKAMI_UV_RESPONSE_1HR_UP http://www.broadinstitute.org/gsea/msigdb/cards/MURAKAMI_UV_RESPONSE_1HR_UP 16 -0.47298 -1.45584 0.0571429 0.220533 0 down vs 1SU_KIDNEY http://www.broadinstitute.org/gsea/msigdb/cards/SU_KIDNEY 15 0.673732 1.97229 0 0.225855 0 up vs 1REN_BOUND_BY_E2F http://www.broadinstitute.org/gsea/msigdb/cards/REN_BOUND_BY_E2F 60 -0.515716 -1.44762 0.0740741 0.226771 0 down vs 1IIZUKA_LIVER_CANCER_PROGRESSION_L0_L1_DN http://www.broadinstitute.org/gsea/msigdb/cards/IIZUKA_LIVER_CANCER_PROGRESSION_L0_L1_DN 21 -0.466467 -1.44792 0.166667 0.227034 0 down vs 1WANG_BARRETTS_ESOPHAGUS_AND_ESOPHAGUS_CANCER_DN http://www.broadinstitute.org/gsea/msigdb/cards/WANG_BARRETTS_ESOPHAGUS_AND_ESOPHAGUS_CANCER_DN 37 -0.482435 -1.4484 0.125 0.227416 0 down vs 1CHIARADONNA_NEOPLASTIC_TRANSFORMATION_KRAS_CDC25_UP http://www.broadinstitute.org/gsea/msigdb/cards/CHIARADONNA_NEOPLASTIC_TRANSFORMATION_KRAS_CDC25_UP 56 -0.32873 -1.44893 0 0.227449 0 down vs 1WINZEN_DEGRADED_VIA_KHSRP http://www.broadinstitute.org/gsea/msigdb/cards/WINZEN_DEGRADED_VIA_KHSRP 99 -0.362574 -1.44923 0.0625 0.227953 0 down vs 1WHITFIELD_CELL_CYCLE_G1_S http://www.broadinstitute.org/gsea/msigdb/cards/WHITFIELD_CELL_CYCLE_G1_S 140 -0.388596 -1.44529 0.12 0.230096 0 down vs 1NEMETH_INFLAMMATORY_RESPONSE_LPS_UP http://www.broadinstitute.org/gsea/msigdb/cards/NEMETH_INFLAMMATORY_RESPONSE_LPS_UP 88 -0.372862 -1.4445 0.1 0.230342 0 down vs 1BILANGES_SERUM_RESPONSE_TRANSLATION http://www.broadinstitute.org/gsea/msigdb/cards/BILANGES_SERUM_RESPONSE_TRANSLATION 36 0.718371 1.97876 0 0.231391 0 up vs 1ABBUD_LIF_SIGNALING_1_UP http://www.broadinstitute.org/gsea/msigdb/cards/ABBUD_LIF_SIGNALING_1_UP 46 -0.363712 -1.4423 0.133333 0.232539 0 down vs 1GERY_CEBP_TARGETS http://www.broadinstitute.org/gsea/msigdb/cards/GERY_CEBP_TARGETS 126 -0.312179 -1.44015 0.0416667 0.233127 0 down vs 1ROSTY_CERVICAL_CANCER_PROLIFERATION_CLUSTER http://www.broadinstitute.org/gsea/msigdb/cards/ROSTY_CERVICAL_CANCER_PROLIFERATION_CLUSTER 138 -0.515281 -1.44064 0.09375 0.233465 0 down vs 1VILIMAS_NOTCH1_TARGETS_UP http://www.broadinstitute.org/gsea/msigdb/cards/VILIMAS_NOTCH1_TARGETS_UP 52 -0.567551 -1.44082 0.0606061 0.233919 0 down vs 1TOMLINS_PROSTATE_CANCER_UP http://www.broadinstitute.org/gsea/msigdb/cards/TOMLINS_PROSTATE_CANCER_UP 39 0.622986 2.04131 0 0.235451 0 up vs 1GAL_LEUKEMIC_STEM_CELL_DN http://www.broadinstitute.org/gsea/msigdb/cards/GAL_LEUKEMIC_STEM_CELL_DN 243 -0.309927 -1.43681 0.0416667 0.237829 0 down vs 1STEIN_ESTROGEN_RESPONSE_NOT_VIA_ESRRA http://www.broadinstitute.org/gsea/msigdb/cards/STEIN_ESTROGEN_RESPONSE_NOT_VIA_ESRRA 18 -0.462968 -1.43524 0.0666667 0.239508 0 down vs 1SESTO_RESPONSE_TO_UV_C3 http://www.broadinstitute.org/gsea/msigdb/cards/SESTO_RESPONSE_TO_UV_C3 20 -0.470301 -1.43423 0.142857 0.240108 0 down vs 1FLECHNER_BIOPSY_KIDNEY_TRANSPLANT_REJECTED_VS_OK_UP http://www.broadinstitute.org/gsea/msigdb/cards/FLECHNER_BIOPSY_KIDNEY_TRANSPLANT_REJECTED_VS_OK_UP 85 -0.561019 -1.42775 0.0625 0.248714 0 down vs 1KIM_BIPOLAR_DISORDER_OLIGODENDROCYTE_DENSITY_CORR_DN http://www.broadinstitute.org/gsea/msigdb/cards/KIM_BIPOLAR_DISORDER_OLIGODENDROCYTE_DENSITY_CORR_DN 86 -0.299679 -1.42823 0 0.249213 0 down vs 1WALLACE_PROSTATE_CANCER_RACE_DN http://www.broadinstitute.org/gsea/msigdb/cards/WALLACE_PROSTATE_CANCER_RACE_DN 82 0.551454 1.97991 0 0.253493 0 up vs 1WINNEPENNINCKX_MELANOMA_METASTASIS_DN http://www.broadinstitute.org/gsea/msigdb/cards/WINNEPENNINCKX_MELANOMA_METASTASIS_DN 45 -0.396646 -1.42176 0.09375 0.253778 0 down vs 1LEI_MYB_TARGETS http://www.broadinstitute.org/gsea/msigdb/cards/LEI_MYB_TARGETS 316 -0.274718 -1.42202 0.125 0.254342 0 down vs 1OUYANG_PROSTATE_CANCER_PROGRESSION_UP http://www.broadinstitute.org/gsea/msigdb/cards/OUYANG_PROSTATE_CANCER_PROGRESSION_UP 20 -0.437523 -1.42247 0.0434783 0.254578 0 down vs 1BROCKE_APOPTOSIS_REVERSED_BY_IL6 http://www.broadinstitute.org/gsea/msigdb/cards/BROCKE_APOPTOSIS_REVERSED_BY_IL6 144 -0.35886 -1.42385 0.2 0.254611 0 down vs 1GHANDHI_BYSTANDER_IRRADIATION_UP http://www.broadinstitute.org/gsea/msigdb/cards/GHANDHI_BYSTANDER_IRRADIATION_UP 85 -0.389705 -1.423 0.103448 0.254816 0 down vs 1INGA_TP53_TARGETS http://www.broadinstitute.org/gsea/msigdb/cards/INGA_TP53_TARGETS 17 -0.448874 -1.41848 0.0689655 0.255509 0 down vs 1LINDSTEDT_DENDRITIC_CELL_MATURATION_C http://www.broadinstitute.org/gsea/msigdb/cards/LINDSTEDT_DENDRITIC_CELL_MATURATION_C 67 -0.379648 -1.41767 0.15 0.255759 0 down vs 1DASU_IL6_SIGNALING_UP http://www.broadinstitute.org/gsea/msigdb/cards/DASU_IL6_SIGNALING_UP 59 -0.34195 -1.41871 0.0909091 0.255853 0 down vs 1DORN_ADENOVIRUS_INFECTION_12HR_DN http://www.broadinstitute.org/gsea/msigdb/cards/DORN_ADENOVIRUS_INFECTION_12HR_DN 33 -0.413437 -1.41583 0.176471 0.255912 0 down vs 1PUJANA_BREAST_CANCER_WITH_BRCA1_MUTATED_UP http://www.broadinstitute.org/gsea/msigdb/cards/PUJANA_BREAST_CANCER_WITH_BRCA1_MUTATED_UP 56 -0.500511 -1.41605 0.206897 0.256521 0 down vs 1PARK_TRETINOIN_RESPONSE_AND_RARA_PLZF_FUSION http://www.broadinstitute.org/gsea/msigdb/cards/PARK_TRETINOIN_RESPONSE_AND_RARA_PLZF_FUSION 22 -0.494675 -1.41891 0.0967742 0.256527 0 down vs 1WATANABE_COLON_CANCER_MSI_VS_MSS_UP http://www.broadinstitute.org/gsea/msigdb/cards/WATANABE_COLON_CANCER_MSI_VS_MSS_UP 27 -0.355433 -1.41647 0.107143 0.256866 0 down vs 1AMIT_SERUM_RESPONSE_480_MCF10A http://www.broadinstitute.org/gsea/msigdb/cards/AMIT_SERUM_RESPONSE_480_MCF10A 36 -0.366672 -1.41922 0 0.256933 0 down vs 1MOLENAAR_TARGETS_OF_CCND1_AND_CDK4_UP http://www.broadinstitute.org/gsea/msigdb/cards/MOLENAAR_TARGETS_OF_CCND1_AND_CDK4_UP 64 0.541524 1.98672 0 0.263867 0 up vs 1MCLACHLAN_DENTAL_CARIES_DN http://www.broadinstitute.org/gsea/msigdb/cards/MCLACHLAN_DENTAL_CARIES_DN 237 -0.424574 -1.40496 0.0882353 0.26458 0 down vs 1MORI_IMMATURE_B_LYMPHOCYTE_UP http://www.broadinstitute.org/gsea/msigdb/cards/MORI_IMMATURE_B_LYMPHOCYTE_UP 51 -0.438501 -1.40937 0.0857143 0.264819 0 down vs 1AMIT_SERUM_RESPONSE_40_MCF10A http://www.broadinstitute.org/gsea/msigdb/cards/AMIT_SERUM_RESPONSE_40_MCF10A 32 -0.431861 -1.40729 0.21875 0.264933 0 down vs 1DIRMEIER_LMP1_RESPONSE_EARLY http://www.broadinstitute.org/gsea/msigdb/cards/DIRMEIER_LMP1_RESPONSE_EARLY 64 -0.399986 -1.40586 0.0322581 0.264938 0 down vs 1MOLENAAR_TARGETS_OF_CCND1_AND_CDK4_DN http://www.broadinstitute.org/gsea/msigdb/cards/MOLENAAR_TARGETS_OF_CCND1_AND_CDK4_DN 55 -0.524778 -1.40515 0.12 0.265095 0 down vs 1ONO_FOXP3_TARGETS_DN http://www.broadinstitute.org/gsea/msigdb/cards/ONO_FOXP3_TARGETS_DN 42 -0.466181 -1.40876 0.0810811 0.265139 0 down vs 1DAZARD_UV_RESPONSE_CLUSTER_G28 http://www.broadinstitute.org/gsea/msigdb/cards/DAZARD_UV_RESPONSE_CLUSTER_G28 20 -0.475225 -1.40768 0.103448 0.265142 0 down vs 1MORI_PRE_BI_LYMPHOCYTE_DN http://www.broadinstitute.org/gsea/msigdb/cards/MORI_PRE_BI_LYMPHOCYTE_DN 74 -0.366667 -1.40795 0.0882353 0.265299 0 down vs 1LIU_PROSTATE_CANCER_UP http://www.broadinstitute.org/gsea/msigdb/cards/LIU_PROSTATE_CANCER_UP 93 0.580372 1.94023 0.0135135 0.265428 0 up vs 1WILSON_PROTEASES_AT_TUMOR_BONE_INTERFACE_UP http://www.broadinstitute.org/gsea/msigdb/cards/WILSON_PROTEASES_AT_TUMOR_BONE_INTERFACE_UP 21 -0.465718 -1.40608 0.0833333 0.265719 0 down vs 1LEE_LIVER_CANCER_HEPATOBLAST http://www.broadinstitute.org/gsea/msigdb/cards/LEE_LIVER_CANCER_HEPATOBLAST 16 -0.525944 -1.40093 0.15625 0.269211 0 down vs 1ZIRN_TRETINOIN_RESPONSE_UP http://www.broadinstitute.org/gsea/msigdb/cards/ZIRN_TRETINOIN_RESPONSE_UP 20 -0.458168 -1.40106 0.0588235 0.269996 0 down vs 1CROONQUIST_IL6_DEPRIVATION_DN http://www.broadinstitute.org/gsea/msigdb/cards/CROONQUIST_IL6_DEPRIVATION_DN 98 -0.499895 -1.39906 0.107143 0.27135 0 down vs 1CHIANG_LIVER_CANCER_SUBCLASS_CTNNB1_UP http://www.broadinstitute.org/gsea/msigdb/cards/CHIANG_LIVER_CANCER_SUBCLASS_CTNNB1_UP 170 0.4923 1.87146 0 0.271457 0 up vs 1DAZARD_UV_RESPONSE_CLUSTER_G24 http://www.broadinstitute.org/gsea/msigdb/cards/DAZARD_UV_RESPONSE_CLUSTER_G24 26 -0.429689 -1.39695 0.12 0.271902 0 down vs 1STEARMAN_TUMOR_FIELD_EFFECT_UP http://www.broadinstitute.org/gsea/msigdb/cards/STEARMAN_TUMOR_FIELD_EFFECT_UP 36 -0.453148 -1.39813 0.151515 0.271995 0 down vs 1ODONNELL_TARGETS_OF_MYC_AND_TFRC_UP http://www.broadinstitute.org/gsea/msigdb/cards/ODONNELL_TARGETS_OF_MYC_AND_TFRC_UP 81 -0.40911 -1.39708 0.129032 0.272432 0 down vs 1CADWELL_ATG16L1_TARGETS_DN http://www.broadinstitute.org/gsea/msigdb/cards/CADWELL_ATG16L1_TARGETS_DN 69 -0.351588 -1.39461 0.05 0.272645 0 down vs 1WILENSKY_RESPONSE_TO_DARAPLADIB http://www.broadinstitute.org/gsea/msigdb/cards/WILENSKY_RESPONSE_TO_DARAPLADIB 29 -0.544244 -1.39565 0.116279 0.272741 0 down vs 1MUELLER_COMMON_TARGETS_OF_AML_FUSIONS_DN http://www.broadinstitute.org/gsea/msigdb/cards/MUELLER_COMMON_TARGETS_OF_AML_FUSIONS_DN 30 -0.35782 -1.39469 0.0333333 0.273424 0 down vs 1KEEN_RESPONSE_TO_ROSIGLITAZONE_DN http://www.broadinstitute.org/gsea/msigdb/cards/KEEN_RESPONSE_TO_ROSIGLITAZONE_DN 105 -0.311399 -1.39239 0.0909091 0.273672 0 down vs 1SAKAI_CHRONIC_HEPATITIS_VS_LIVER_CANCER_DN http://www.broadinstitute.org/gsea/msigdb/cards/SAKAI_CHRONIC_HEPATITIS_VS_LIVER_CANCER_DN 36 -0.370588 -1.39269 0.0344828 0.273997 0 down vs 1YIH_RESPONSE_TO_ARSENITE_C4 http://www.broadinstitute.org/gsea/msigdb/cards/YIH_RESPONSE_TO_ARSENITE_C4 18 -0.399198 -1.39272 0.0740741 0.274775 0 down vs 1KYNG_RESPONSE_TO_H2O2_VIA_ERCC6_UP http://www.broadinstitute.org/gsea/msigdb/cards/KYNG_RESPONSE_TO_H2O2_VIA_ERCC6_UP 40 0.557182 1.87544 0 0.276046 0 up vs 1ICHIBA_GRAFT_VERSUS_HOST_DISEASE_D7_DN http://www.broadinstitute.org/gsea/msigdb/cards/ICHIBA_GRAFT_VERSUS_HOST_DISEASE_D7_DN 38 0.524151 1.85532 0 0.276982 0 up vs 1BOYLAN_MULTIPLE_MYELOMA_D_DN http://www.broadinstitute.org/gsea/msigdb/cards/BOYLAN_MULTIPLE_MYELOMA_D_DN 74 -0.336839 -1.38844 0.178571 0.278704 0 down vs 1KIM_RESPONSE_TO_TSA_AND_DECITABINE_UP http://www.broadinstitute.org/gsea/msigdb/cards/KIM_RESPONSE_TO_TSA_AND_DECITABINE_UP 127 -0.310998 -1.38788 0.117647 0.27886 0 down vs 1AMIT_SERUM_RESPONSE_60_MCF10A http://www.broadinstitute.org/gsea/msigdb/cards/AMIT_SERUM_RESPONSE_60_MCF10A 57 -0.404521 -1.38532 0.185185 0.281184 0 down vs 1WANG_BARRETTS_ESOPHAGUS_DN http://www.broadinstitute.org/gsea/msigdb/cards/WANG_BARRETTS_ESOPHAGUS_DN 25 -0.479266 -1.383 0.103448 0.283632 0 down vs 1WALLACE_PROSTATE_CANCER_UP http://www.broadinstitute.org/gsea/msigdb/cards/WALLACE_PROSTATE_CANCER_UP 19 0.631673 1.87768 0.0135135 0.283778 0 up vs 1MATZUK_IMPLANTATION_AND_UTERINE http://www.broadinstitute.org/gsea/msigdb/cards/MATZUK_IMPLANTATION_AND_UTERINE 22 0.53777 1.86225 0.0133333 0.283826 0 up vs 1WANG_METHYLATED_IN_BREAST_CANCER http://www.broadinstitute.org/gsea/msigdb/cards/WANG_METHYLATED_IN_BREAST_CANCER 35 -0.484364 -1.38349 0.0769231 0.283837 0 down vs 1VERHAAK_GLIOBLASTOMA_PRONEURAL http://www.broadinstitute.org/gsea/msigdb/cards/VERHAAK_GLIOBLASTOMA_PRONEURAL 174 0.49926 1.83291 0 0.283894 0 up vs 1SU_THYMUS http://www.broadinstitute.org/gsea/msigdb/cards/SU_THYMUS 20 -0.5483 -1.38109 0.142857 0.285968 0 down vs 1YAO_TEMPORAL_RESPONSE_TO_PROGESTERONE_CLUSTER_14 http://www.broadinstitute.org/gsea/msigdb/cards/YAO_TEMPORAL_RESPONSE_TO_PROGESTERONE_CLUSTER_14 140 0.564117 1.94057 0 0.286871 0 up vs 1RICKMAN_METASTASIS_UP http://www.broadinstitute.org/gsea/msigdb/cards/RICKMAN_METASTASIS_UP 333 0.542503 1.85584 0.0128205 0.287087 0 up vs 1AMUNDSON_GENOTOXIC_SIGNATURE http://www.broadinstitute.org/gsea/msigdb/cards/AMUNDSON_GENOTOXIC_SIGNATURE 102 -0.290962 -1.37799 0.0909091 0.289189 0 down vs 1KENNY_CTNNB1_TARGETS_UP http://www.broadinstitute.org/gsea/msigdb/cards/KENNY_CTNNB1_TARGETS_UP 48 -0.322853 -1.37752 0.15 0.289212 0 down vs 1HENDRICKS_SMARCA4_TARGETS_DN http://www.broadinstitute.org/gsea/msigdb/cards/HENDRICKS_SMARCA4_TARGETS_DN 52 -0.345496 -1.37826 0.0416667 0.289505 0 down vs 1NIKOLSKY_BREAST_CANCER_7Q21_Q22_AMPLICON http://www.broadinstitute.org/gsea/msigdb/cards/NIKOLSKY_BREAST_CANCER_7Q21_Q22_AMPLICON 73 0.621497 1.91463 0.0144928 0.290118 0 up vs 1LEIN_LOCALIZED_TO_PROXIMAL_DENDRITES http://www.broadinstitute.org/gsea/msigdb/cards/LEIN_LOCALIZED_TO_PROXIMAL_DENDRITES 36 0.542115 1.84413 0 0.290179 0 up vs 1CAIRO_HEPATOBLASTOMA_UP http://www.broadinstitute.org/gsea/msigdb/cards/CAIRO_HEPATOBLASTOMA_UP 201 0.541244 1.89378 0 0.290479 0 up vs 1CHARAFE_BREAST_CANCER_BASAL_VS_MESENCHYMAL_DN http://www.broadinstitute.org/gsea/msigdb/cards/CHARAFE_BREAST_CANCER_BASAL_VS_MESENCHYMAL_DN 49 0.571165 1.8342 0 0.290604 0 up vs 1WANG_METASTASIS_OF_BREAST_CANCER_ESR1_DN http://www.broadinstitute.org/gsea/msigdb/cards/WANG_METASTASIS_OF_BREAST_CANCER_ESR1_DN 28 -0.377438 -1.37608 0.103448 0.290778 0 down vs 1BILANGES_RAPAMYCIN_SENSITIVE_VIA_TSC1_AND_TSC2 http://www.broadinstitute.org/gsea/msigdb/cards/BILANGES_RAPAMYCIN_SENSITIVE_VIA_TSC1_AND_TSC2 72 0.576999 1.8387 0 0.291414 0 up vs 1MIKKELSEN_MEF_ICP_WITH_H3K4ME3_AND_H3K27ME3 http://www.broadinstitute.org/gsea/msigdb/cards/MIKKELSEN_MEF_ICP_WITH_H3K4ME3_AND_H3K27ME3 38 0.528399 1.90397 0.015873 0.292284 0 up vs 1WILLERT_WNT_SIGNALING http://www.broadinstitute.org/gsea/msigdb/cards/WILLERT_WNT_SIGNALING 24 0.651224 2.14527 0 0.292284 0 up vs 1XU_CREBBP_TARGETS_DN http://www.broadinstitute.org/gsea/msigdb/cards/XU_CREBBP_TARGETS_DN 44 -0.329803 -1.37408 0.111111 0.293969 0 down vs 1TORCHIA_TARGETS_OF_EWSR1_FLI1_FUSION_TOP20_DN http://www.broadinstitute.org/gsea/msigdb/cards/TORCHIA_TARGETS_OF_EWSR1_FLI1_FUSION_TOP20_DN 18 0.593477 1.87951 0.0151515 0.294313 0 up vs 1DEBOSSCHER_NFKB_TARGETS_REPRESSED_BY_GLUCOCORTICOIDS http://www.broadinstitute.org/gsea/msigdb/cards/DEBOSSCHER_NFKB_TARGETS_REPRESSED_BY_GLUCOCORTICOIDS 24 -0.463964 -1.37075 0.105263 0.296805 0 down vs 1LI_WILMS_TUMOR http://www.broadinstitute.org/gsea/msigdb/cards/LI_WILMS_TUMOR 26 0.602987 1.91936 0.0441176 0.297503 0 up vs 1JECHLINGER_EPITHELIAL_TO_MESENCHYMAL_TRANSITION_DN http://www.broadinstitute.org/gsea/msigdb/cards/JECHLINGER_EPITHELIAL_TO_MESENCHYMAL_TRANSITION_DN 66 -0.321308 -1.37082 0.0869565 0.29752 0 down vs 1LEONARD_HYPOXIA http://www.broadinstitute.org/gsea/msigdb/cards/LEONARD_HYPOXIA 47 -0.394798 -1.37129 0.133333 0.297617 0 down vs 1MACLACHLAN_BRCA1_TARGETS_DN http://www.broadinstitute.org/gsea/msigdb/cards/MACLACHLAN_BRCA1_TARGETS_DN 16 -0.43528 -1.3716 0.12 0.298051 0 down vs 1XU_RESPONSE_TO_TRETINOIN_UP http://www.broadinstitute.org/gsea/msigdb/cards/XU_RESPONSE_TO_TRETINOIN_UP 15 -0.497819 -1.3696 0.171429 0.298131 0 down vs 1LIAN_NEUTROPHIL_GRANULE_CONSTITUENTS http://www.broadinstitute.org/gsea/msigdb/cards/LIAN_NEUTROPHIL_GRANULE_CONSTITUENTS 24 -0.453253 -1.36831 0.138889 0.29959 0 down vs 1ZHAN_MULTIPLE_MYELOMA_MS_UP http://www.broadinstitute.org/gsea/msigdb/cards/ZHAN_MULTIPLE_MYELOMA_MS_UP 47 0.580228 1.88353 0 0.299975 0 up vs 1NIKOLSKY_BREAST_CANCER_8Q12_Q22_AMPLICON http://www.broadinstitute.org/gsea/msigdb/cards/NIKOLSKY_BREAST_CANCER_8Q12_Q22_AMPLICON 128 0.577277 1.8171 0.0133333 0.30294 0 up vs 1ZHU_CMV_ALL_UP http://www.broadinstitute.org/gsea/msigdb/cards/ZHU_CMV_ALL_UP 120 -0.339513 -1.36539 0.047619 0.303675 0 down vs 1NIELSEN_GIST_AND_SYNOVIAL_SARCOMA_UP http://www.broadinstitute.org/gsea/msigdb/cards/NIELSEN_GIST_AND_SYNOVIAL_SARCOMA_UP 20 0.600345 1.89409 0.0169492 0.306134 0 up vs 1KORKOLA_EMBRYONIC_CARCINOMA_VS_SEMINOMA_UP http://www.broadinstitute.org/gsea/msigdb/cards/KORKOLA_EMBRYONIC_CARCINOMA_VS_SEMINOMA_UP 21 0.570266 1.82003 0 0.306164 0 up vs 1LANDIS_BREAST_CANCER_PROGRESSION_UP http://www.broadinstitute.org/gsea/msigdb/cards/LANDIS_BREAST_CANCER_PROGRESSION_UP 44 -0.371678 -1.36161 0.2 0.306894 0 down vs 1DACOSTA_UV_RESPONSE_VIA_ERCC3_COMMON_UP http://www.broadinstitute.org/gsea/msigdb/cards/DACOSTA_UV_RESPONSE_VIA_ERCC3_COMMON_UP 76 -0.287934 -1.36311 0.0625 0.307126 0 down vs 1LI_LUNG_CANCER http://www.broadinstitute.org/gsea/msigdb/cards/LI_LUNG_CANCER 41 -0.347552 -1.3608 0 0.307606 0 down vs 1LAU_APOPTOSIS_CDKN2A_UP http://www.broadinstitute.org/gsea/msigdb/cards/LAU_APOPTOSIS_CDKN2A_UP 55 -0.366599 -1.3623 0.0833333 0.307609 0 down vs 1RUTELLA_RESPONSE_TO_HGF_VS_CSF2RB_AND_IL4_DN http://www.broadinstitute.org/gsea/msigdb/cards/RUTELLA_RESPONSE_TO_HGF_VS_CSF2RB_AND_IL4_DN 239 -0.313067 -1.36167 0.05 0.30767 0 down vs 1ENK_UV_RESPONSE_EPIDERMIS_DN http://www.broadinstitute.org/gsea/msigdb/cards/ENK_UV_RESPONSE_EPIDERMIS_DN 500 -0.274971 -1.35988 0 0.308267 0 down vs 1RICKMAN_HEAD_AND_NECK_CANCER_A http://www.broadinstitute.org/gsea/msigdb/cards/RICKMAN_HEAD_AND_NECK_CANCER_A 98 0.502722 1.75554 0.015625 0.310178 0 up vs 1MEISSNER_BRAIN_HCP_WITH_H3_UNMETHYLATED http://www.broadinstitute.org/gsea/msigdb/cards/MEISSNER_BRAIN_HCP_WITH_H3_UNMETHYLATED 36 -0.339491 -1.35739 0.12 0.311413 0 down vs 1KOBAYASHI_EGFR_SIGNALING_24HR_UP http://www.broadinstitute.org/gsea/msigdb/cards/KOBAYASHI_EGFR_SIGNALING_24HR_UP 100 -0.330164 -1.35539 0.1 0.312793 0 down vs 1ZHOU_CELL_CYCLE_GENES_IN_IR_RESPONSE_24HR http://www.broadinstitute.org/gsea/msigdb/cards/ZHOU_CELL_CYCLE_GENES_IN_IR_RESPONSE_24HR 123 -0.439523 -1.35573 0.185185 0.313116 0 down vs 1MURAKAMI_UV_RESPONSE_6HR_DN http://www.broadinstitute.org/gsea/msigdb/cards/MURAKAMI_UV_RESPONSE_6HR_DN 21 -0.419849 -1.35431 0.0526316 0.313477 0 down vs 1BROWN_MYELOID_CELL_DEVELOPMENT_UP http://www.broadinstitute.org/gsea/msigdb/cards/BROWN_MYELOID_CELL_DEVELOPMENT_UP 160 -0.355873 -1.35298 0.0967742 0.313925 0 down vs 1CHOW_RASSF1_TARGETS_UP http://www.broadinstitute.org/gsea/msigdb/cards/CHOW_RASSF1_TARGETS_UP 27 -0.433102 -1.35192 0.107143 0.314368 0 down vs 1KUROZUMI_RESPONSE_TO_ONCOCYTIC_VIRUS_AND_CYCLIC_RGD http://www.broadinstitute.org/gsea/msigdb/cards/KUROZUMI_RESPONSE_TO_ONCOCYTIC_VIRUS_AND_CYCLIC_RGD 21 -0.588536 -1.35325 0.075 0.31443 0 down vs 1RODWELL_AGING_KIDNEY_NO_BLOOD_DN http://www.broadinstitute.org/gsea/msigdb/cards/RODWELL_AGING_KIDNEY_NO_BLOOD_DN 145 0.484092 1.76248 0.0120482 0.314523 0 up vs 1HOELZEL_NF1_TARGETS_DN http://www.broadinstitute.org/gsea/msigdb/cards/HOELZEL_NF1_TARGETS_DN 106 0.501755 1.75951 0 0.314568 0 up vs 1HAHTOLA_MYCOSIS_FUNGOIDES_CD4_UP http://www.broadinstitute.org/gsea/msigdb/cards/HAHTOLA_MYCOSIS_FUNGOIDES_CD4_UP 61 -0.380952 -1.35234 0.2 0.3146 0 down vs 1SCHAEFFER_SOX9_TARGETS_IN_PROSTATE_DEVELOPMENT_UP http://www.broadinstitute.org/gsea/msigdb/cards/SCHAEFFER_SOX9_TARGETS_IN_PROSTATE_DEVELOPMENT_UP 21 0.540926 1.75638 0 0.315118 0 up vs 1HALMOS_CEBPA_TARGETS_UP http://www.broadinstitute.org/gsea/msigdb/cards/HALMOS_CEBPA_TARGETS_UP 52 -0.325729 -1.35025 0.0909091 0.315254 0 down vs 1TENEDINI_MEGAKARYOCYTE_MARKERS http://www.broadinstitute.org/gsea/msigdb/cards/TENEDINI_MEGAKARYOCYTE_MARKERS 65 -0.330537 -1.34925 0.2 0.31556 0 down vs 1LY_AGING_MIDDLE_DN http://www.broadinstitute.org/gsea/msigdb/cards/LY_AGING_MIDDLE_DN 16 -0.584688 -1.3505 0.157895 0.315623 0 down vs 1YANG_BCL3_TARGETS_UP http://www.broadinstitute.org/gsea/msigdb/cards/YANG_BCL3_TARGETS_UP 353 -0.274451 -1.34878 0 0.315908 0 down vs 1YAMASHITA_LIVER_CANCER_WITH_EPCAM_UP http://www.broadinstitute.org/gsea/msigdb/cards/YAMASHITA_LIVER_CANCER_WITH_EPCAM_UP 52 0.604749 1.80321 0.0128205 0.319267 0 up vs 1SETLUR_PROSTATE_CANCER_TMPRSS2_ERG_FUSION_UP http://www.broadinstitute.org/gsea/msigdb/cards/SETLUR_PROSTATE_CANCER_TMPRSS2_ERG_FUSION_UP 65 0.520464 1.8068 0 0.319347 0 up vs 1POMEROY_MEDULLOBLASTOMA_PROGNOSIS_DN http://www.broadinstitute.org/gsea/msigdb/cards/POMEROY_MEDULLOBLASTOMA_PROGNOSIS_DN 43 0.612128 1.73218 0.0285714 0.320059 0 up vs 1SILIGAN_TARGETS_OF_EWS_FLI1_FUSION_DN http://www.broadinstitute.org/gsea/msigdb/cards/SILIGAN_TARGETS_OF_EWS_FLI1_FUSION_DN 17 0.580279 1.74665 0.025 0.320302 0 up vs 1HUMMEL_BURKITTS_LYMPHOMA_UP http://www.broadinstitute.org/gsea/msigdb/cards/HUMMEL_BURKITTS_LYMPHOMA_UP 40 0.548382 1.74879 0.0133333 0.3207 0 up vs 1BREUHAHN_GROWTH_FACTOR_SIGNALING_IN_LIVER_CANCER http://www.broadinstitute.org/gsea/msigdb/cards/BREUHAHN_GROWTH_FACTOR_SIGNALING_IN_LIVER_CANCER 22 0.541049 1.76295 0.027027 0.320971 0 up vs 1GRAHAM_CML_DIVIDING_VS_NORMAL_QUIESCENT_DN http://www.broadinstitute.org/gsea/msigdb/cards/GRAHAM_CML_DIVIDING_VS_NORMAL_QUIESCENT_DN 94 -0.349158 -1.34514 0.0454545 0.321372 0 down vs 1NIELSEN_SYNOVIAL_SARCOMA_UP http://www.broadinstitute.org/gsea/msigdb/cards/NIELSEN_SYNOVIAL_SARCOMA_UP 18 0.557279 1.74344 0 0.321481 0 up vs 1NIKOLSKY_BREAST_CANCER_8P12_P11_AMPLICON http://www.broadinstitute.org/gsea/msigdb/cards/NIKOLSKY_BREAST_CANCER_8P12_P11_AMPLICON 56 0.547396 1.73307 0.0428571 0.32302 0 up vs 1WARTERS_RESPONSE_TO_IR_SKIN http://www.broadinstitute.org/gsea/msigdb/cards/WARTERS_RESPONSE_TO_IR_SKIN 80 -0.320694 -1.34284 0.0909091 0.324586 0 down vs 1RICKMAN_TUMOR_DIFFERENTIATED_WELL_VS_POORLY_UP http://www.broadinstitute.org/gsea/msigdb/cards/RICKMAN_TUMOR_DIFFERENTIATED_WELL_VS_POORLY_UP 229 0.520284 1.73507 0.0135135 0.32476 0 up vs 1SANSOM_WNT_PATHWAY_REQUIRE_MYC http://www.broadinstitute.org/gsea/msigdb/cards/SANSOM_WNT_PATHWAY_REQUIRE_MYC 58 0.507594 1.73897 0 0.325372 0 up vs 1YANG_MUC2_TARGETS_DUODENUM_6MO_DN http://www.broadinstitute.org/gsea/msigdb/cards/YANG_MUC2_TARGETS_DUODENUM_6MO_DN 21 0.570386 1.73677 0 0.325662 0 up vs 1BERNARD_PPAPDC1B_TARGETS_DN http://www.broadinstitute.org/gsea/msigdb/cards/BERNARD_PPAPDC1B_TARGETS_DN 53 0.507233 1.76426 0.0128205 0.325682 0 up vs 1DEBIASI_APOPTOSIS_BY_REOVIRUS_INFECTION_UP http://www.broadinstitute.org/gsea/msigdb/cards/DEBIASI_APOPTOSIS_BY_REOVIRUS_INFECTION_UP 311 -0.319859 -1.34036 0.2 0.328181 0 down vs 1NIELSEN_GIST_VS_SYNOVIAL_SARCOMA_UP http://www.broadinstitute.org/gsea/msigdb/cards/NIELSEN_GIST_VS_SYNOVIAL_SARCOMA_UP 19 0.571421 1.72609 0.05 0.329239 0 up vs 1EGUCHI_CELL_CYCLE_RB1_TARGETS http://www.broadinstitute.org/gsea/msigdb/cards/EGUCHI_CELL_CYCLE_RB1_TARGETS 23 -0.622512 -1.33835 0.153846 0.32972 0 down vs 1XU_CREBBP_TARGETS_UP http://www.broadinstitute.org/gsea/msigdb/cards/XU_CREBBP_TARGETS_UP 25 0.510477 1.77031 0.0121951 0.329786 0 up vs 1DE_YY1_TARGETS_UP http://www.broadinstitute.org/gsea/msigdb/cards/DE_YY1_TARGETS_UP 20 -0.368066 -1.33882 0.0555556 0.329813 0 down vs 1HUNSBERGER_EXERCISE_REGULATED_GENES http://www.broadinstitute.org/gsea/msigdb/cards/HUNSBERGER_EXERCISE_REGULATED_GENES 31 0.552481 1.76628 0 0.330235 0 up vs 1GEISS_RESPONSE_TO_DSRNA_DN http://www.broadinstitute.org/gsea/msigdb/cards/GEISS_RESPONSE_TO_DSRNA_DN 16 0.550522 1.71365 0.0142857 0.331203 0 up vs 1KANG_FLUOROURACIL_RESISTANCE_DN http://www.broadinstitute.org/gsea/msigdb/cards/KANG_FLUOROURACIL_RESISTANCE_DN 16 -0.431953 -1.33632 0.103448 0.331636 0 down vs 1CERVERA_SDHB_TARGETS_1_UP http://www.broadinstitute.org/gsea/msigdb/cards/CERVERA_SDHB_TARGETS_1_UP 114 -0.28293 -1.33657 0.125 0.332085 0 down vs 1SCHLOSSER_MYC_TARGETS_AND_SERUM_RESPONSE_UP http://www.broadinstitute.org/gsea/msigdb/cards/SCHLOSSER_MYC_TARGETS_AND_SERUM_RESPONSE_UP 46 0.571474 1.71494 0.0540541 0.333533 0 up vs 1JAATINEN_HEMATOPOIETIC_STEM_CELL_DN http://www.broadinstitute.org/gsea/msigdb/cards/JAATINEN_HEMATOPOIETIC_STEM_CELL_DN 218 -0.39881 -1.33408 0.171429 0.334618 0 down vs 1RIZ_ERYTHROID_DIFFERENTIATION http://www.broadinstitute.org/gsea/msigdb/cards/RIZ_ERYTHROID_DIFFERENTIATION 74 -0.390433 -1.33423 0.238095 0.335073 0 down vs 1FIGUEROA_AML_METHYLATION_CLUSTER_6_DN http://www.broadinstitute.org/gsea/msigdb/cards/FIGUEROA_AML_METHYLATION_CLUSTER_6_DN 38 -0.349845 -1.33076 0.1 0.33588 0 down vs 1GAVIN_FOXP3_TARGETS_CLUSTER_P4 http://www.broadinstitute.org/gsea/msigdb/cards/GAVIN_FOXP3_TARGETS_CLUSTER_P4 99 -0.302292 -1.33203 0.130435 0.33588 0 down vs 1VANDESLUIS_NORMAL_EMBRYOS_DN http://www.broadinstitute.org/gsea/msigdb/cards/VANDESLUIS_NORMAL_EMBRYOS_DN 25 0.521617 1.7709 0.0144928 0.336047 0 up vs 1KAMMINGA_EZH2_TARGETS http://www.broadinstitute.org/gsea/msigdb/cards/KAMMINGA_EZH2_TARGETS 41 -0.501479 -1.33251 0.185185 0.336336 0 down vs 1GAVIN_FOXP3_TARGETS_CLUSTER_T4 http://www.broadinstitute.org/gsea/msigdb/cards/GAVIN_FOXP3_TARGETS_CLUSTER_T4 92 -0.305718 -1.3311 0.105263 0.336419 0 down vs 1STEIN_ESRRA_TARGETS_RESPONSIVE_TO_ESTROGEN_UP http://www.broadinstitute.org/gsea/msigdb/cards/STEIN_ESRRA_TARGETS_RESPONSIVE_TO_ESTROGEN_UP 31 -0.371871 -1.32963 0.0689655 0.337318 0 down vs 1UDAYAKUMAR_MED1_TARGETS_DN http://www.broadinstitute.org/gsea/msigdb/cards/UDAYAKUMAR_MED1_TARGETS_DN 233 -0.267425 -1.32863 0.111111 0.338105 0 down vs 1SCHAEFFER_PROSTATE_DEVELOPMENT_12HR_DN http://www.broadinstitute.org/gsea/msigdb/cards/SCHAEFFER_PROSTATE_DEVELOPMENT_12HR_DN 56 0.467983 1.71506 0.0133333 0.338735 0 up vs 1NIELSEN_MALIGNAT_FIBROUS_HISTIOCYTOMA_DN http://www.broadinstitute.org/gsea/msigdb/cards/NIELSEN_MALIGNAT_FIBROUS_HISTIOCYTOMA_DN 17 0.632156 1.7722 0.0149254 0.341403 0 up vs 1ISSAEVA_MLL2_TARGETS http://www.broadinstitute.org/gsea/msigdb/cards/ISSAEVA_MLL2_TARGETS 62 -0.333496 -1.3256 0.178571 0.342773 0 down vs 1MAGRANGEAS_MULTIPLE_MYELOMA_IGG_VS_IGA_DN http://www.broadinstitute.org/gsea/msigdb/cards/MAGRANGEAS_MULTIPLE_MYELOMA_IGG_VS_IGA_DN 25 -0.317141 -1.32187 0.0869565 0.344089 0 down vs 1RORIE_TARGETS_OF_EWSR1_FLI1_FUSION_DN http://www.broadinstitute.org/gsea/msigdb/cards/RORIE_TARGETS_OF_EWSR1_FLI1_FUSION_DN 27 0.499123 1.71533 0 0.34411 0 up vs 1FRASOR_RESPONSE_TO_SERM_OR_FULVESTRANT_DN http://www.broadinstitute.org/gsea/msigdb/cards/FRASOR_RESPONSE_TO_SERM_OR_FULVESTRANT_DN 50 -0.469325 -1.32232 0.148148 0.344216 0 down vs 1TSUNODA_CISPLATIN_RESISTANCE_DN http://www.broadinstitute.org/gsea/msigdb/cards/TSUNODA_CISPLATIN_RESISTANCE_DN 50 -0.421317 -1.32426 0.0666667 0.344308 0 down vs 1GEORGANTAS_HSC_MARKERS http://www.broadinstitute.org/gsea/msigdb/cards/GEORGANTAS_HSC_MARKERS 68 -0.342109 -1.31924 0.0769231 0.344749 0 down vs 1LEE_EARLY_T_LYMPHOCYTE_UP http://www.broadinstitute.org/gsea/msigdb/cards/LEE_EARLY_T_LYMPHOCYTE_UP 104 -0.462084 -1.32258 0.166667 0.344767 0 down vs 1OZANNE_AP1_TARGETS_UP http://www.broadinstitute.org/gsea/msigdb/cards/OZANNE_AP1_TARGETS_UP 15 -0.432784 -1.323 0.117647 0.34477 0 down vs 1JOHANSSON_GLIOMAGENESIS_BY_PDGFB_UP http://www.broadinstitute.org/gsea/msigdb/cards/JOHANSSON_GLIOMAGENESIS_BY_PDGFB_UP 57 -0.308463 -1.32315 0 0.34541 0 down vs 1MANN_RESPONSE_TO_AMIFOSTINE_UP http://www.broadinstitute.org/gsea/msigdb/cards/MANN_RESPONSE_TO_AMIFOSTINE_UP 20 -0.402794 -1.31927 0.16 0.345627 0 down vs 1MASRI_RESISTANCE_TO_TAMOXIFEN_AND_AROMATASE_INHIBITORS_DN http://www.broadinstitute.org/gsea/msigdb/cards/MASRI_RESISTANCE_TO_TAMOXIFEN_AND_AROMATASE_INHIBITORS_DN 20 -0.442413 -1.31966 0.181818 0.346092 0 down vs 1WIEDERSCHAIN_TARGETS_OF_BMI1_AND_PCGF2 http://www.broadinstitute.org/gsea/msigdb/cards/WIEDERSCHAIN_TARGETS_OF_BMI1_AND_PCGF2 56 -0.382962 -1.31975 0.151515 0.346852 0 down vs 1SCHLINGEMANN_SKIN_CARCINOGENESIS_TPA_UP http://www.broadinstitute.org/gsea/msigdb/cards/SCHLINGEMANN_SKIN_CARCINOGENESIS_TPA_UP 39 -0.323138 -1.31712 0.133333 0.34715 0 down vs 1ZEMBUTSU_SENSITIVITY_TO_NIMUSTINE http://www.broadinstitute.org/gsea/msigdb/cards/ZEMBUTSU_SENSITIVITY_TO_NIMUSTINE 17 0.544983 1.78419 0 0.347312 0 up vs 1ELLWOOD_MYC_TARGETS_DN http://www.broadinstitute.org/gsea/msigdb/cards/ELLWOOD_MYC_TARGETS_DN 38 0.509863 1.7167 0 0.347328 0 up vs 1BROWNE_HCMV_INFECTION_2HR_UP http://www.broadinstitute.org/gsea/msigdb/cards/BROWNE_HCMV_INFECTION_2HR_UP 39 -0.379941 -1.31603 0.15625 0.348587 0 down vs 1KYNG_RESPONSE_TO_H2O2_VIA_ERCC6 http://www.broadinstitute.org/gsea/msigdb/cards/KYNG_RESPONSE_TO_H2O2_VIA_ERCC6 17 0.581981 1.77531 0.0263158 0.349544 0 up vs 1SWEET_KRAS_ONCOGENIC_SIGNATURE http://www.broadinstitute.org/gsea/msigdb/cards/SWEET_KRAS_ONCOGENIC_SIGNATURE 89 0.492114 1.77228 0 0.349949 0 up vs 1LIU_IL13_MEMORY_MODEL_UP http://www.broadinstitute.org/gsea/msigdb/cards/LIU_IL13_MEMORY_MODEL_UP 17 0.554189 1.70271 0.0142857 0.351527 0 up vs 1RODRIGUES_THYROID_CARCINOMA_UP http://www.broadinstitute.org/gsea/msigdb/cards/RODRIGUES_THYROID_CARCINOMA_UP 16 0.583277 1.77879 0.0147059 0.352408 0 up vs 1VILIMAS_NOTCH1_TARGETS_DN http://www.broadinstitute.org/gsea/msigdb/cards/VILIMAS_NOTCH1_TARGETS_DN 19 -0.512086 -1.31355 0.121212 0.352696 0 down vs 1RORIE_TARGETS_OF_EWSR1_FLI1_FUSION_UP http://www.broadinstitute.org/gsea/msigdb/cards/RORIE_TARGETS_OF_EWSR1_FLI1_FUSION_UP 30 -0.352232 -1.31242 0.0434783 0.354275 0 down vs 1HUANG_GATA2_TARGETS_UP http://www.broadinstitute.org/gsea/msigdb/cards/HUANG_GATA2_TARGETS_UP 147 -0.320765 -1.31191 0.115385 0.354329 0 down vs 1CHESLER_BRAIN_HIGHEST_EXPRESSION http://www.broadinstitute.org/gsea/msigdb/cards/CHESLER_BRAIN_HIGHEST_EXPRESSION 38 0.54248 1.78563 0.012987 0.354684 0 up vs 1BOYAULT_LIVER_CANCER_SUBCLASS_G56_DN http://www.broadinstitute.org/gsea/msigdb/cards/BOYAULT_LIVER_CANCER_SUBCLASS_G56_DN 17 -0.476338 -1.30973 0.193548 0.355182 0 down vs 1LINDSTEDT_DENDRITIC_CELL_MATURATION_D http://www.broadinstitute.org/gsea/msigdb/cards/LINDSTEDT_DENDRITIC_CELL_MATURATION_D 66 -0.381166 -1.31042 0.107143 0.355733 0 down vs 1HOLLEMAN_ASPARAGINASE_RESISTANCE_B_ALL_DN http://www.broadinstitute.org/gsea/msigdb/cards/HOLLEMAN_ASPARAGINASE_RESISTANCE_B_ALL_DN 15 -0.409044 -1.30673 0.147059 0.355957 0 down vs 1GAJATE_RESPONSE_TO_TRABECTEDIN_UP http://www.broadinstitute.org/gsea/msigdb/cards/GAJATE_RESPONSE_TO_TRABECTEDIN_UP 65 -0.290633 -1.30982 0.1 0.356028 0 down vs 1WIERENGA_STAT5A_TARGETS_UP http://www.broadinstitute.org/gsea/msigdb/cards/WIERENGA_STAT5A_TARGETS_UP 211 -0.289975 -1.30691 0.04 0.356593 0 down vs 1SIMBULAN_UV_RESPONSE_IMMORTALIZED_DN http://www.broadinstitute.org/gsea/msigdb/cards/SIMBULAN_UV_RESPONSE_IMMORTALIZED_DN 31 -0.430064 -1.30732 0.2 0.356828 0 down vs 1SIMBULAN_PARP1_TARGETS_DN http://www.broadinstitute.org/gsea/msigdb/cards/SIMBULAN_PARP1_TARGETS_DN 17 -0.491422 -1.30767 0.1875 0.356903 0 down vs 1KOHOUTEK_CCNT2_TARGETS http://www.broadinstitute.org/gsea/msigdb/cards/KOHOUTEK_CCNT2_TARGETS 57 0.452079 1.69529 0 0.357481 0 up vs 1ZHAN_MULTIPLE_MYELOMA_PR_DN http://www.broadinstitute.org/gsea/msigdb/cards/ZHAN_MULTIPLE_MYELOMA_PR_DN 44 -0.379083 -1.30787 0.3 0.357627 0 down vs 1MULLIGHAN_NPM1_MUTATED_SIGNATURE_2_DN http://www.broadinstitute.org/gsea/msigdb/cards/MULLIGHAN_NPM1_MUTATED_SIGNATURE_2_DN 76 0.460376 1.69774 0 0.35811 0 up vs 1FIGUEROA_AML_METHYLATION_CLUSTER_1_DN http://www.broadinstitute.org/gsea/msigdb/cards/FIGUEROA_AML_METHYLATION_CLUSTER_1_DN 44 -0.347409 -1.30417 0.107143 0.360452 0 down vs 1MORI_PRE_BI_LYMPHOCYTE_UP http://www.broadinstitute.org/gsea/msigdb/cards/MORI_PRE_BI_LYMPHOCYTE_UP 80 -0.357101 -1.30262 0.148148 0.361562 0 down vs 1WU_HBX_TARGETS_2_DN http://www.broadinstitute.org/gsea/msigdb/cards/WU_HBX_TARGETS_2_DN 16 -0.393915 -1.3027 0.111111 0.362407 0 down vs 1TONKS_TARGETS_OF_RUNX1_RUNX1T1_FUSION_GRANULOCYTE_DN http://www.broadinstitute.org/gsea/msigdb/cards/TONKS_TARGETS_OF_RUNX1_RUNX1T1_FUSION_GRANULOCYTE_DN 17 -0.502199 -1.30097 0.189189 0.364219 0 down vs 1TRACEY_RESISTANCE_TO_IFNA2_DN http://www.broadinstitute.org/gsea/msigdb/cards/TRACEY_RESISTANCE_TO_IFNA2_DN 30 -0.398007 -1.297 0.12 0.366845 0 down vs 1SENGUPTA_EBNA1_ANTICORRELATED http://www.broadinstitute.org/gsea/msigdb/cards/SENGUPTA_EBNA1_ANTICORRELATED 158 -0.309245 -1.29878 0.208333 0.366863 0 down vs 1KAAB_FAILED_HEART_VENTRICLE_DN http://www.broadinstitute.org/gsea/msigdb/cards/KAAB_FAILED_HEART_VENTRICLE_DN 41 -0.333754 -1.29721 0.16 0.367496 0 down vs 1CAIRO_HEPATOBLASTOMA_CLASSES_DN http://www.broadinstitute.org/gsea/msigdb/cards/CAIRO_HEPATOBLASTOMA_CLASSES_DN 205 -0.299957 -1.29746 0.192308 0.368149 0 down vs 1LUI_THYROID_CANCER_CLUSTER_2 http://www.broadinstitute.org/gsea/msigdb/cards/LUI_THYROID_CANCER_CLUSTER_2 40 0.50238 1.68845 0.0379747 0.368869 0 up vs 1ONO_AML1_TARGETS_DN http://www.broadinstitute.org/gsea/msigdb/cards/ONO_AML1_TARGETS_DN 41 -0.437653 -1.29471 0.162162 0.369235 0 down vs 1KESHELAVA_MULTIPLE_DRUG_RESISTANCE http://www.broadinstitute.org/gsea/msigdb/cards/KESHELAVA_MULTIPLE_DRUG_RESISTANCE 83 -0.287942 -1.29515 0.0909091 0.369338 0 down vs 1OUYANG_PROSTATE_CANCER_PROGRESSION_DN http://www.broadinstitute.org/gsea/msigdb/cards/OUYANG_PROSTATE_CANCER_PROGRESSION_DN 20 0.56669 1.68306 0.0138889 0.371591 0 up vs 1BENPORATH_ES_2 http://www.broadinstitute.org/gsea/msigdb/cards/BENPORATH_ES_2 40 0.508893 1.68449 0.0273973 0.374667 0 up vs 1OHGUCHI_LIVER_HNF4A_TARGETS_UP http://www.broadinstitute.org/gsea/msigdb/cards/OHGUCHI_LIVER_HNF4A_TARGETS_UP 44 -0.32272 -1.28933 0.192308 0.376427 0 down vs 1WANG_BARRETTS_ESOPHAGUS_UP http://www.broadinstitute.org/gsea/msigdb/cards/WANG_BARRETTS_ESOPHAGUS_UP 51 -0.336592 -1.28874 0.21875 0.376811 0 down vs 1CHICAS_RB1_TARGETS_LOW_SERUM http://www.broadinstitute.org/gsea/msigdb/cards/CHICAS_RB1_TARGETS_LOW_SERUM 93 -0.313541 -1.2896 0.222222 0.37686 0 down vs 1HAHTOLA_CTCL_PATHOGENESIS http://www.broadinstitute.org/gsea/msigdb/cards/HAHTOLA_CTCL_PATHOGENESIS 16 -0.564423 -1.29 0.157895 0.376982 0 down vs 1LABBE_WNT3A_TARGETS_UP http://www.broadinstitute.org/gsea/msigdb/cards/LABBE_WNT3A_TARGETS_UP 110 -0.278934 -1.2874 0.0588235 0.377457 0 down vs 1SCHEIDEREIT_IKK_TARGETS http://www.broadinstitute.org/gsea/msigdb/cards/SCHEIDEREIT_IKK_TARGETS 18 -0.38213 -1.28783 0.133333 0.377657 0 down vs 1UZONYI_RESPONSE_TO_LEUKOTRIENE_AND_THROMBIN http://www.broadinstitute.org/gsea/msigdb/cards/UZONYI_RESPONSE_TO_LEUKOTRIENE_AND_THROMBIN 37 -0.456176 -1.2868 0.242424 0.377721 0 down vs 1WANG_RECURRENT_LIVER_CANCER_DN http://www.broadinstitute.org/gsea/msigdb/cards/WANG_RECURRENT_LIVER_CANCER_DN 16 -0.4173 -1.28445 0.107143 0.381245 0 down vs 1BURTON_ADIPOGENESIS_1 http://www.broadinstitute.org/gsea/msigdb/cards/BURTON_ADIPOGENESIS_1 33 -0.390874 -1.28391 0.21875 0.381574 0 down vs 1DEURIG_T_CELL_PROLYMPHOCYTIC_LEUKEMIA_DN http://www.broadinstitute.org/gsea/msigdb/cards/DEURIG_T_CELL_PROLYMPHOCYTIC_LEUKEMIA_DN 305 -0.335886 -1.28213 0.153846 0.384193 0 down vs 1KONDO_PROSTATE_CANCER_HCP_WITH_H3K27ME3 http://www.broadinstitute.org/gsea/msigdb/cards/KONDO_PROSTATE_CANCER_HCP_WITH_H3K27ME3 97 0.447493 1.66546 0 0.385814 0 up vs 1VETTER_TARGETS_OF_PRKCA_AND_ETS1_DN http://www.broadinstitute.org/gsea/msigdb/cards/VETTER_TARGETS_OF_PRKCA_AND_ETS1_DN 16 -0.40083 -1.28044 0.222222 0.386609 0 down vs 1NIELSEN_LEIOMYOSARCOMA_CNN1_DN http://www.broadinstitute.org/gsea/msigdb/cards/NIELSEN_LEIOMYOSARCOMA_CNN1_DN 20 0.590715 1.66663 0.0588235 0.388285 0 up vs 1VANDESLUIS_COMMD1_TARGETS_GROUP_3_DN http://www.broadinstitute.org/gsea/msigdb/cards/VANDESLUIS_COMMD1_TARGETS_GROUP_3_DN 38 0.471831 1.6699 0.015625 0.388697 0 up vs 1BURTON_ADIPOGENESIS_PEAK_AT_2HR http://www.broadinstitute.org/gsea/msigdb/cards/BURTON_ADIPOGENESIS_PEAK_AT_2HR 50 -0.393052 -1.27804 0.137931 0.389585 0 down vs 1DEMAGALHAES_AGING_UP http://www.broadinstitute.org/gsea/msigdb/cards/DEMAGALHAES_AGING_UP 54 -0.37153 -1.27846 0.147059 0.389887 0 down vs 1BOSCO_ALLERGEN_INDUCED_TH2_ASSOCIATED_MODULE http://www.broadinstitute.org/gsea/msigdb/cards/BOSCO_ALLERGEN_INDUCED_TH2_ASSOCIATED_MODULE 146 -0.297563 -1.27651 0.230769 0.390571 0 down vs 1ZHENG_GLIOBLASTOMA_PLASTICITY_UP http://www.broadinstitute.org/gsea/msigdb/cards/ZHENG_GLIOBLASTOMA_PLASTICITY_UP 244 -0.299622 -1.27656 0.1875 0.391479 0 down vs 1JIANG_AGING_HYPOTHALAMUS_DN http://www.broadinstitute.org/gsea/msigdb/cards/JIANG_AGING_HYPOTHALAMUS_DN 40 0.48303 1.67057 0 0.392456 0 up vs 1THEODOROU_MAMMARY_TUMORIGENESIS http://www.broadinstitute.org/gsea/msigdb/cards/THEODOROU_MAMMARY_TUMORIGENESIS 29 0.509609 1.6668 0.031746 0.39327 0 up vs 1MATTIOLI_MULTIPLE_MYELOMA_SUBGROUPS http://www.broadinstitute.org/gsea/msigdb/cards/MATTIOLI_MULTIPLE_MYELOMA_SUBGROUPS 15 0.597048 1.67158 0 0.395627 0 up vs 1KANG_DOXORUBICIN_RESISTANCE_UP http://www.broadinstitute.org/gsea/msigdb/cards/KANG_DOXORUBICIN_RESISTANCE_UP 53 -0.555628 -1.27118 0.21875 0.399944 0 down vs 1HANN_RESISTANCE_TO_BCL2_INHIBITOR_UP http://www.broadinstitute.org/gsea/msigdb/cards/HANN_RESISTANCE_TO_BCL2_INHIBITOR_UP 35 0.484228 1.64874 0.0307692 0.400336 0 up vs 1BERENJENO_TRANSFORMED_BY_RHOA_FOREVER_DN http://www.broadinstitute.org/gsea/msigdb/cards/BERENJENO_TRANSFORMED_BY_RHOA_FOREVER_DN 31 -0.360054 -1.27022 0.206897 0.400522 0 down vs 1MANTOVANI_VIRAL_GPCR_SIGNALING_DN http://www.broadinstitute.org/gsea/msigdb/cards/MANTOVANI_VIRAL_GPCR_SIGNALING_DN 48 0.44877 1.64225 0 0.401407 0 up vs 1RASHI_RESPONSE_TO_IONIZING_RADIATION_4 http://www.broadinstitute.org/gsea/msigdb/cards/RASHI_RESPONSE_TO_IONIZING_RADIATION_4 59 -0.280732 -1.26921 0.130435 0.401584 0 down vs 1MOOTHA_GLYCOGEN_METABOLISM http://www.broadinstitute.org/gsea/msigdb/cards/MOOTHA_GLYCOGEN_METABOLISM 21 0.513564 1.64387 0.0140845 0.403015 0 up vs 1SMIRNOV_RESPONSE_TO_IR_6HR_DN http://www.broadinstitute.org/gsea/msigdb/cards/SMIRNOV_RESPONSE_TO_IR_6HR_DN 110 -0.342234 -1.26642 0.171429 0.403112 0 down vs 1KYNG_WERNER_SYNDROM_UP http://www.broadinstitute.org/gsea/msigdb/cards/KYNG_WERNER_SYNDROM_UP 19 -0.391205 -1.26688 0.12 0.403179 0 down vs 1MARSON_FOXP3_TARGETS_UP http://www.broadinstitute.org/gsea/msigdb/cards/MARSON_FOXP3_TARGETS_UP 64 -0.319098 -1.26723 0.137931 0.403433 0 down vs 1GALLUZZI_PREVENT_MITOCHONDIAL_PERMEABILIZATION http://www.broadinstitute.org/gsea/msigdb/cards/GALLUZZI_PREVENT_MITOCHONDIAL_PERMEABILIZATION 22 -0.3095 -1.26758 0.181818 0.403763 0 down vs 1DAZARD_UV_RESPONSE_CLUSTER_G2 http://www.broadinstitute.org/gsea/msigdb/cards/DAZARD_UV_RESPONSE_CLUSTER_G2 30 -0.373359 -1.26545 0.227273 0.404118 0 down vs 1CAIRO_LIVER_DEVELOPMENT_UP http://www.broadinstitute.org/gsea/msigdb/cards/CAIRO_LIVER_DEVELOPMENT_UP 162 0.458604 1.64936 0.025974 0.404326 0 up vs 1WOO_LIVER_CANCER_RECURRENCE_DN http://www.broadinstitute.org/gsea/msigdb/cards/WOO_LIVER_CANCER_RECURRENCE_DN 80 0.456736 1.65315 0.0131579 0.4047 0 up vs 1HEIDENBLAD_AMPLICON_8Q24_UP http://www.broadinstitute.org/gsea/msigdb/cards/HEIDENBLAD_AMPLICON_8Q24_UP 37 0.497293 1.65083 0 0.404819 0 up vs 1SUZUKI_AMPLIFIED_IN_ORAL_CANCER http://www.broadinstitute.org/gsea/msigdb/cards/SUZUKI_AMPLIFIED_IN_ORAL_CANCER 16 0.537983 1.65672 0.0169492 0.405202 0 up vs 1NELSON_RESPONSE_TO_ANDROGEN_DN http://www.broadinstitute.org/gsea/msigdb/cards/NELSON_RESPONSE_TO_ANDROGEN_DN 18 0.521514 1.64459 0.0416667 0.405256 0 up vs 1BOYAULT_LIVER_CANCER_SUBCLASS_G3_DN http://www.broadinstitute.org/gsea/msigdb/cards/BOYAULT_LIVER_CANCER_SUBCLASS_G3_DN 50 0.501112 1.65491 0.0142857 0.405422 0 up vs 1FIGUEROA_AML_METHYLATION_CLUSTER_2_UP http://www.broadinstitute.org/gsea/msigdb/cards/FIGUEROA_AML_METHYLATION_CLUSTER_2_UP 52 0.452398 1.63833 0 0.405854 0 up vs 1SHETH_LIVER_CANCER_VS_TXNIP_LOSS_PAM3 http://www.broadinstitute.org/gsea/msigdb/cards/SHETH_LIVER_CANCER_VS_TXNIP_LOSS_PAM3 69 -0.265076 -1.26415 0.0588235 0.405933 0 down vs 1ACOSTA_PROLIFERATION_INDEPENDENT_MYC_TARGETS_DN http://www.broadinstitute.org/gsea/msigdb/cards/ACOSTA_PROLIFERATION_INDEPENDENT_MYC_TARGETS_DN 112 -0.267705 -1.26299 0.157895 0.406559 0 down vs 1YU_MYC_TARGETS_DN http://www.broadinstitute.org/gsea/msigdb/cards/YU_MYC_TARGETS_DN 54 -0.438278 -1.26329 0.1875 0.407002 0 down vs 1PEDERSEN_METASTASIS_BY_ERBB2_ISOFORM_6 http://www.broadinstitute.org/gsea/msigdb/cards/PEDERSEN_METASTASIS_BY_ERBB2_ISOFORM_6 28 -0.341982 -1.2602 0.16129 0.411618 0 down vs 1NIKOLSKY_BREAST_CANCER_15Q26_AMPLICON http://www.broadinstitute.org/gsea/msigdb/cards/NIKOLSKY_BREAST_CANCER_15Q26_AMPLICON 21 -0.443352 -1.25944 0.189189 0.412154 0 down vs 1MENSSEN_MYC_TARGETS http://www.broadinstitute.org/gsea/msigdb/cards/MENSSEN_MYC_TARGETS 51 0.581898 1.63164 0.0909091 0.412295 0 up vs 1CHANDRAN_METASTASIS_TOP50_UP http://www.broadinstitute.org/gsea/msigdb/cards/CHANDRAN_METASTASIS_TOP50_UP 35 0.50822 1.62946 0.0121951 0.4125 0 up vs 1TONKS_TARGETS_OF_RUNX1_RUNX1T1_FUSION_MONOCYTE_DN http://www.broadinstitute.org/gsea/msigdb/cards/TONKS_TARGETS_OF_RUNX1_RUNX1T1_FUSION_MONOCYTE_DN 53 -0.372438 -1.25803 0.15625 0.414002 0 down vs 1CHIARADONNA_NEOPLASTIC_TRANSFORMATION_KRAS_UP http://www.broadinstitute.org/gsea/msigdb/cards/CHIARADONNA_NEOPLASTIC_TRANSFORMATION_KRAS_UP 125 -0.307179 -1.25753 0.2 0.414202 0 down vs 1ZHENG_FOXP3_TARGETS_UP http://www.broadinstitute.org/gsea/msigdb/cards/ZHENG_FOXP3_TARGETS_UP 26 -0.359373 -1.25715 0.147059 0.414219 0 down vs 1HASLINGER_B_CLL_WITH_MUTATED_VH_GENES http://www.broadinstitute.org/gsea/msigdb/cards/HASLINGER_B_CLL_WITH_MUTATED_VH_GENES 17 0.528911 1.63214 0.0508475 0.415673 0 up vs 1SANA_RESPONSE_TO_IFNG_DN http://www.broadinstitute.org/gsea/msigdb/cards/SANA_RESPONSE_TO_IFNG_DN 83 0.485847 1.58174 0.025 0.418648 0 up vs 1HADDAD_T_LYMPHOCYTE_AND_NK_PROGENITOR_DN http://www.broadinstitute.org/gsea/msigdb/cards/HADDAD_T_LYMPHOCYTE_AND_NK_PROGENITOR_DN 62 -0.35451 -1.25019 0.16129 0.419953 0 down vs 1JAATINEN_HEMATOPOIETIC_STEM_CELL_UP http://www.broadinstitute.org/gsea/msigdb/cards/JAATINEN_HEMATOPOIETIC_STEM_CELL_UP 306 0.416133 1.58243 0.0121951 0.420158 0 up vs 1KANG_CISPLATIN_RESISTANCE_UP http://www.broadinstitute.org/gsea/msigdb/cards/KANG_CISPLATIN_RESISTANCE_UP 18 -0.392546 -1.24948 0.185185 0.420206 0 down vs 1RODWELL_AGING_KIDNEY_UP http://www.broadinstitute.org/gsea/msigdb/cards/RODWELL_AGING_KIDNEY_UP 469 -0.316656 -1.25094 0.230769 0.420339 0 down vs 1SARTIPY_BLUNTED_BY_INSULIN_RESISTANCE_UP http://www.broadinstitute.org/gsea/msigdb/cards/SARTIPY_BLUNTED_BY_INSULIN_RESISTANCE_UP 19 -0.383264 -1.25032 0.230769 0.420486 0 down vs 1CHIANG_LIVER_CANCER_SUBCLASS_CTNNB1_DN http://www.broadinstitute.org/gsea/msigdb/cards/CHIANG_LIVER_CANCER_SUBCLASS_CTNNB1_DN 166 -0.296008 -1.24849 0.290323 0.420779 0 down vs 1GREENBAUM_E2A_TARGETS_UP http://www.broadinstitute.org/gsea/msigdb/cards/GREENBAUM_E2A_TARGETS_UP 33 -0.508484 -1.25112 0.222222 0.421056 0 down vs 1VISALA_AGING_LYMPHOCYTE_DN http://www.broadinstitute.org/gsea/msigdb/cards/VISALA_AGING_LYMPHOCYTE_DN 19 -0.37641 -1.25254 0.0869565 0.421522 0 down vs 1LENAOUR_DENDRITIC_CELL_MATURATION_DN http://www.broadinstitute.org/gsea/msigdb/cards/LENAOUR_DENDRITIC_CELL_MATURATION_DN 128 -0.329896 -1.25127 0.125 0.421668 0 down vs 1PELLICCIOTTA_HDAC_IN_ANTIGEN_PRESENTATION_DN http://www.broadinstitute.org/gsea/msigdb/cards/PELLICCIOTTA_HDAC_IN_ANTIGEN_PRESENTATION_DN 49 -0.353882 -1.25156 0.24 0.422246 0 down vs 1LIN_MELANOMA_COPY_NUMBER_UP http://www.broadinstitute.org/gsea/msigdb/cards/LIN_MELANOMA_COPY_NUMBER_UP 69 0.441453 1.58266 0.037037 0.422823 0 up vs 1GAZDA_DIAMOND_BLACKFAN_ANEMIA_MYELOID_DN http://www.broadinstitute.org/gsea/msigdb/cards/GAZDA_DIAMOND_BLACKFAN_ANEMIA_MYELOID_DN 38 0.461461 1.58705 0.045977 0.423275 0 up vs 1RODWELL_AGING_KIDNEY_DN http://www.broadinstitute.org/gsea/msigdb/cards/RODWELL_AGING_KIDNEY_DN 137 0.436892 1.62356 0.0125 0.423556 0 up vs 1ZHAN_MULTIPLE_MYELOMA_MF_DN http://www.broadinstitute.org/gsea/msigdb/cards/ZHAN_MULTIPLE_MYELOMA_MF_DN 37 0.49472 1.58835 0.0285714 0.423577 0 up vs 1LIAO_HAVE_SOX4_BINDING_SITES http://www.broadinstitute.org/gsea/msigdb/cards/LIAO_HAVE_SOX4_BINDING_SITES 40 0.466676 1.58393 0.0394737 0.423968 0 up vs 1BILANGES_SERUM_AND_RAPAMYCIN_SENSITIVE_GENES http://www.broadinstitute.org/gsea/msigdb/cards/BILANGES_SERUM_AND_RAPAMYCIN_SENSITIVE_GENES 68 0.696341 1.57854 0.0857143 0.424114 0 up vs 1MARKEY_RB1_CHRONIC_LOF_UP http://www.broadinstitute.org/gsea/msigdb/cards/MARKEY_RB1_CHRONIC_LOF_UP 113 -0.304794 -1.24587 0.176471 0.424545 0 down vs 1PENG_RAPAMYCIN_RESPONSE_DN http://www.broadinstitute.org/gsea/msigdb/cards/PENG_RAPAMYCIN_RESPONSE_DN 240 0.48034 1.58446 0.0481928 0.426067 0 up vs 1HU_ANGIOGENESIS_UP http://www.broadinstitute.org/gsea/msigdb/cards/HU_ANGIOGENESIS_UP 21 -0.373482 -1.24453 0.185185 0.426664 0 down vs 1MODY_HIPPOCAMPUS_NEONATAL http://www.broadinstitute.org/gsea/msigdb/cards/MODY_HIPPOCAMPUS_NEONATAL 35 0.53841 1.58837 0.0833333 0.427428 0 up vs 1RAMPON_ENRICHED_LEARNING_ENVIRONMENT_LATE_UP http://www.broadinstitute.org/gsea/msigdb/cards/RAMPON_ENRICHED_LEARNING_ENVIRONMENT_LATE_UP 22 0.515787 1.61859 0.0294118 0.428344 0 up vs 1ONDER_CDH1_TARGETS_1_DN http://www.broadinstitute.org/gsea/msigdb/cards/ONDER_CDH1_TARGETS_1_DN 167 -0.292886 -1.24289 0.2 0.429056 0 down vs 1DUTERTRE_ESTRADIOL_RESPONSE_6HR_UP http://www.broadinstitute.org/gsea/msigdb/cards/DUTERTRE_ESTRADIOL_RESPONSE_6HR_UP 223 -0.261111 -1.24015 0.1875 0.429394 0 down vs 1GENTILE_UV_RESPONSE_CLUSTER_D1 http://www.broadinstitute.org/gsea/msigdb/cards/GENTILE_UV_RESPONSE_CLUSTER_D1 18 -0.386585 -1.24126 0.222222 0.430149 0 down vs 1WATTEL_AUTONOMOUS_THYROID_ADENOMA_DN http://www.broadinstitute.org/gsea/msigdb/cards/WATTEL_AUTONOMOUS_THYROID_ADENOMA_DN 53 -0.345493 -1.24019 0.0740741 0.430287 0 down vs 1IVANOVA_HEMATOPOIESIS_INTERMEDIATE_PROGENITOR http://www.broadinstitute.org/gsea/msigdb/cards/IVANOVA_HEMATOPOIESIS_INTERMEDIATE_PROGENITOR 144 0.438954 1.61956 0 0.430487 0 up vs 1ICHIBA_GRAFT_VERSUS_HOST_DISEASE_35D_UP http://www.broadinstitute.org/gsea/msigdb/cards/ICHIBA_GRAFT_VERSUS_HOST_DISEASE_35D_UP 127 -0.372732 -1.24045 0.212121 0.430657 0 down vs 1KEEN_RESPONSE_TO_ROSIGLITAZONE_UP http://www.broadinstitute.org/gsea/msigdb/cards/KEEN_RESPONSE_TO_ROSIGLITAZONE_UP 38 0.491991 1.58868 0.030303 0.430753 0 up vs 1BURTON_ADIPOGENESIS_6 http://www.broadinstitute.org/gsea/msigdb/cards/BURTON_ADIPOGENESIS_6 183 0.432058 1.61565 0 0.431012 0 up vs 1NUTT_GBM_VS_AO_GLIOMA_UP http://www.broadinstitute.org/gsea/msigdb/cards/NUTT_GBM_VS_AO_GLIOMA_UP 46 -0.322565 -1.24129 0.28 0.431085 0 down vs 1MODY_HIPPOCAMPUS_PRENATAL http://www.broadinstitute.org/gsea/msigdb/cards/MODY_HIPPOCAMPUS_PRENATAL 42 0.577087 1.57108 0.0958904 0.431783 0 up vs 1KYNG_RESPONSE_TO_H2O2_VIA_ERCC6_DN http://www.broadinstitute.org/gsea/msigdb/cards/KYNG_RESPONSE_TO_H2O2_VIA_ERCC6_DN 46 0.459661 1.58977 0.0361446 0.43181 0 up vs 1COLLER_MYC_TARGETS_UP http://www.broadinstitute.org/gsea/msigdb/cards/COLLER_MYC_TARGETS_UP 25 0.584088 1.57173 0.118421 0.433554 0 up vs 1CHARAFE_BREAST_CANCER_LUMINAL_VS_BASAL_UP http://www.broadinstitute.org/gsea/msigdb/cards/CHARAFE_BREAST_CANCER_LUMINAL_VS_BASAL_UP 368 0.458773 1.5984 0.0136986 0.434127 0 up vs 1HOSHIDA_LIVER_CANCER_SUBCLASS_S2 http://www.broadinstitute.org/gsea/msigdb/cards/HOSHIDA_LIVER_CANCER_SUBCLASS_S2 114 0.478996 1.58997 0.0240964 0.435466 0 up vs 1ULE_SPLICING_VIA_NOVA2 http://www.broadinstitute.org/gsea/msigdb/cards/ULE_SPLICING_VIA_NOVA2 43 0.439939 1.57218 0.0410959 0.435969 0 up vs 1CHANG_CYCLING_GENES http://www.broadinstitute.org/gsea/msigdb/cards/CHANG_CYCLING_GENES 145 -0.407306 -1.23434 0.285714 0.436464 0 down vs 1CONCANNON_APOPTOSIS_BY_EPOXOMICIN_UP http://www.broadinstitute.org/gsea/msigdb/cards/CONCANNON_APOPTOSIS_BY_EPOXOMICIN_UP 236 -0.242352 -1.23473 0.0555556 0.43667 0 down vs 1WANG_RESPONSE_TO_BEXAROTENE_DN http://www.broadinstitute.org/gsea/msigdb/cards/WANG_RESPONSE_TO_BEXAROTENE_DN 29 -0.383364 -1.23251 0.225806 0.436916 0 down vs 1YAO_TEMPORAL_RESPONSE_TO_PROGESTERONE_CLUSTER_10 http://www.broadinstitute.org/gsea/msigdb/cards/YAO_TEMPORAL_RESPONSE_TO_PROGESTERONE_CLUSTER_10 67 0.481676 1.60533 0.0238095 0.436919 0 up vs 1FOURNIER_ACINAR_DEVELOPMENT_LATE_DN http://www.broadinstitute.org/gsea/msigdb/cards/FOURNIER_ACINAR_DEVELOPMENT_LATE_DN 21 -0.433667 -1.23611 0.366667 0.436979 0 down vs 1LIU_VAV3_PROSTATE_CARCINOGENESIS_UP http://www.broadinstitute.org/gsea/msigdb/cards/LIU_VAV3_PROSTATE_CARCINOGENESIS_UP 89 -0.33895 -1.2329 0.206897 0.436984 0 down vs 1WHITFIELD_CELL_CYCLE_G2 http://www.broadinstitute.org/gsea/msigdb/cards/WHITFIELD_CELL_CYCLE_G2 173 -0.335488 -1.23197 0.333333 0.437123 0 down vs 1SASSON_RESPONSE_TO_GONADOTROPHINS_DN http://www.broadinstitute.org/gsea/msigdb/cards/SASSON_RESPONSE_TO_GONADOTROPHINS_DN 87 -0.278444 -1.23317 0.157895 0.437535 0 down vs 1MARSON_FOXP3_CORE_DIRECT_TARGETS http://www.broadinstitute.org/gsea/msigdb/cards/MARSON_FOXP3_CORE_DIRECT_TARGETS 19 -0.454832 -1.23475 0.25 0.437605 0 down vs 1SHEPARD_CRUSH_AND_BURN_MUTANT_UP http://www.broadinstitute.org/gsea/msigdb/cards/SHEPARD_CRUSH_AND_BURN_MUTANT_UP 191 0.410145 1.5987 0 0.437621 0 up vs 1YEGNASUBRAMANIAN_PROSTATE_CANCER http://www.broadinstitute.org/gsea/msigdb/cards/YEGNASUBRAMANIAN_PROSTATE_CANCER 122 0.415746 1.60633 0 0.438002 0 up vs 1KAUFFMANN_DNA_REPLICATION_GENES http://www.broadinstitute.org/gsea/msigdb/cards/KAUFFMANN_DNA_REPLICATION_GENES 143 -0.335378 -1.23501 0.222222 0.438023 0 down vs 1BOYLAN_MULTIPLE_MYELOMA_C_D_DN http://www.broadinstitute.org/gsea/msigdb/cards/BOYLAN_MULTIPLE_MYELOMA_C_D_DN 247 -0.298829 -1.23074 0.142857 0.438901 0 down vs 1KASLER_HDAC7_TARGETS_2_DN http://www.broadinstitute.org/gsea/msigdb/cards/KASLER_HDAC7_TARGETS_2_DN 32 0.47355 1.56712 0 0.438958 0 up vs 1GAZDA_DIAMOND_BLACKFAN_ANEMIA_PROGENITOR_DN http://www.broadinstitute.org/gsea/msigdb/cards/GAZDA_DIAMOND_BLACKFAN_ANEMIA_PROGENITOR_DN 62 0.456957 1.58999 0.0454545 0.439498 0 up vs 1JECHLINGER_EPITHELIAL_TO_MESENCHYMAL_TRANSITION_UP http://www.broadinstitute.org/gsea/msigdb/cards/JECHLINGER_EPITHELIAL_TO_MESENCHYMAL_TRANSITION_UP 70 -0.355648 -1.22724 0.310345 0.440575 0 down vs 1HOFFMANN_LARGE_TO_SMALL_PRE_BII_LYMPHOCYTE_UP http://www.broadinstitute.org/gsea/msigdb/cards/HOFFMANN_LARGE_TO_SMALL_PRE_BII_LYMPHOCYTE_UP 161 -0.359444 -1.2287 0.24 0.440664 0 down vs 1FINETTI_BREAST_CANCERS_KINOME_BLUE http://www.broadinstitute.org/gsea/msigdb/cards/FINETTI_BREAST_CANCERS_KINOME_BLUE 21 0.534992 1.59411 0.0307692 0.441027 0 up vs 1WELCSH_BRCA1_TARGETS_DN http://www.broadinstitute.org/gsea/msigdb/cards/WELCSH_BRCA1_TARGETS_DN 139 0.472914 1.60677 0.0238095 0.44116 0 up vs 1MULLIGHAN_MLL_SIGNATURE_2_UP http://www.broadinstitute.org/gsea/msigdb/cards/MULLIGHAN_MLL_SIGNATURE_2_UP 402 -0.240349 -1.22896 0.142857 0.441183 0 down vs 1LIM_MAMMARY_LUMINAL_PROGENITOR_UP http://www.broadinstitute.org/gsea/msigdb/cards/LIM_MAMMARY_LUMINAL_PROGENITOR_UP 56 -0.318394 -1.22726 0.222222 0.441531 0 down vs 1VANDESLUIS_COMMD1_TARGETS_GROUP_2_UP http://www.broadinstitute.org/gsea/msigdb/cards/VANDESLUIS_COMMD1_TARGETS_GROUP_2_UP 15 0.560405 1.5988 0.0517241 0.441673 0 up vs 1CROONQUIST_NRAS_VS_STROMAL_STIMULATION_DN http://www.broadinstitute.org/gsea/msigdb/cards/CROONQUIST_NRAS_VS_STROMAL_STIMULATION_DN 99 -0.306448 -1.22732 0.25 0.442253 0 down vs 1LEIN_NEURON_MARKERS http://www.broadinstitute.org/gsea/msigdb/cards/LEIN_NEURON_MARKERS 67 0.450878 1.60943 0 0.442616 0 up vs 1YANG_BREAST_CANCER_ESR1_LASER_UP http://www.broadinstitute.org/gsea/msigdb/cards/YANG_BREAST_CANCER_ESR1_LASER_UP 33 0.559149 1.60754 0.0151515 0.443089 0 up vs 1YAO_TEMPORAL_RESPONSE_TO_PROGESTERONE_CLUSTER_11 http://www.broadinstitute.org/gsea/msigdb/cards/YAO_TEMPORAL_RESPONSE_TO_PROGESTERONE_CLUSTER_11 99 0.471499 1.60144 0.0238095 0.44323 0 up vs 1BHATI_G2M_ARREST_BY_2METHOXYESTRADIOL_DN http://www.broadinstitute.org/gsea/msigdb/cards/BHATI_G2M_ARREST_BY_2METHOXYESTRADIOL_DN 127 0.395999 1.59007 0.0227273 0.443529 0 up vs 1VERHAAK_AML_WITH_NPM1_MUTATED_UP http://www.broadinstitute.org/gsea/msigdb/cards/VERHAAK_AML_WITH_NPM1_MUTATED_UP 181 -0.317495 -1.22524 0.1875 0.444246 0 down vs 1MALONEY_RESPONSE_TO_17AAG_UP http://www.broadinstitute.org/gsea/msigdb/cards/MALONEY_RESPONSE_TO_17AAG_UP 40 0.461377 1.59106 0.027027 0.445217 0 up vs 1KIM_HYPOXIA http://www.broadinstitute.org/gsea/msigdb/cards/KIM_HYPOXIA 25 -0.392881 -1.22353 0.21875 0.44546 0 down vs 1GENTILE_RESPONSE_CLUSTER_D3 http://www.broadinstitute.org/gsea/msigdb/cards/GENTILE_RESPONSE_CLUSTER_D3 61 -0.367771 -1.2229 0.263158 0.445677 0 down vs 1NIKOLSKY_BREAST_CANCER_6P24_P22_AMPLICON http://www.broadinstitute.org/gsea/msigdb/cards/NIKOLSKY_BREAST_CANCER_6P24_P22_AMPLICON 20 0.58876 1.59891 0.0857143 0.44597 0 up vs 1BAUS_TFF2_TARGETS_UP http://www.broadinstitute.org/gsea/msigdb/cards/BAUS_TFF2_TARGETS_UP 31 -0.376294 -1.22361 0.21875 0.446184 0 down vs 1BILANGES_SERUM_SENSITIVE_GENES http://www.broadinstitute.org/gsea/msigdb/cards/BILANGES_SERUM_SENSITIVE_GENES 87 0.405444 1.56284 0.0240964 0.44628 0 up vs 1DUAN_PRDM5_TARGETS http://www.broadinstitute.org/gsea/msigdb/cards/DUAN_PRDM5_TARGETS 77 -0.258273 -1.22127 0.136364 0.447666 0 down vs 1HUANG_FOXA2_TARGETS_DN http://www.broadinstitute.org/gsea/msigdb/cards/HUANG_FOXA2_TARGETS_DN 36 -0.352295 -1.22064 0.26087 0.448012 0 down vs 1WORSCHECH_TUMOR_EVASION_AND_TOLEROGENICITY_UP http://www.broadinstitute.org/gsea/msigdb/cards/WORSCHECH_TUMOR_EVASION_AND_TOLEROGENICITY_UP 30 -0.380868 -1.21999 0.236842 0.448289 0 down vs 1CHEN_LUNG_CANCER_SURVIVAL http://www.broadinstitute.org/gsea/msigdb/cards/CHEN_LUNG_CANCER_SURVIVAL 26 -0.342357 -1.21769 0.190476 0.451423 0 down vs 1AZARE_STAT3_TARGETS http://www.broadinstitute.org/gsea/msigdb/cards/AZARE_STAT3_TARGETS 23 -0.388136 -1.21649 0.321429 0.451595 0 down vs 1MULLIGHAN_MLL_SIGNATURE_1_UP http://www.broadinstitute.org/gsea/msigdb/cards/MULLIGHAN_MLL_SIGNATURE_1_UP 365 -0.247803 -1.21669 0.105263 0.452256 0 down vs 1NIELSEN_GIST_VS_SYNOVIAL_SARCOMA_DN http://www.broadinstitute.org/gsea/msigdb/cards/NIELSEN_GIST_VS_SYNOVIAL_SARCOMA_DN 19 -0.378131 -1.21437 0.222222 0.454901 0 down vs 1KUNINGER_IGF1_VS_PDGFB_TARGETS_DN http://www.broadinstitute.org/gsea/msigdb/cards/KUNINGER_IGF1_VS_PDGFB_TARGETS_DN 45 -0.287368 -1.21362 0.166667 0.454992 0 down vs 1BORCZUK_MALIGNANT_MESOTHELIOMA_DN http://www.broadinstitute.org/gsea/msigdb/cards/BORCZUK_MALIGNANT_MESOTHELIOMA_DN 102 -0.302559 -1.214 0.242424 0.455194 0 down vs 1PIONTEK_PKD1_TARGETS_UP http://www.broadinstitute.org/gsea/msigdb/cards/PIONTEK_PKD1_TARGETS_UP 38 -0.275647 -1.21299 0.166667 0.45525 0 down vs 1FINETTI_BREAST_CANCER_KINOME_GREEN http://www.broadinstitute.org/gsea/msigdb/cards/FINETTI_BREAST_CANCER_KINOME_GREEN 16 -0.555584 -1.20764 0.236842 0.457666 0 down vs 1BILBAN_B_CLL_LPL_DN http://www.broadinstitute.org/gsea/msigdb/cards/BILBAN_B_CLL_LPL_DN 42 -0.343093 -1.21028 0.214286 0.458443 0 down vs 1ZHAN_MULTIPLE_MYELOMA_PR_UP http://www.broadinstitute.org/gsea/msigdb/cards/ZHAN_MULTIPLE_MYELOMA_PR_UP 44 -0.466267 -1.20769 0.289474 0.458517 0 down vs 1TUOMISTO_TUMOR_SUPPRESSION_BY_COL13A1_UP http://www.broadinstitute.org/gsea/msigdb/cards/TUOMISTO_TUMOR_SUPPRESSION_BY_COL13A1_UP 18 -0.380275 -1.20864 0.214286 0.458637 0 down vs 1LINDGREN_BLADDER_CANCER_CLUSTER_3_DN http://www.broadinstitute.org/gsea/msigdb/cards/LINDGREN_BLADDER_CANCER_CLUSTER_3_DN 220 -0.247925 -1.20789 0.0909091 0.459014 0 down vs 1PEDERSEN_METASTASIS_BY_ERBB2_ISOFORM_1 http://www.broadinstitute.org/gsea/msigdb/cards/PEDERSEN_METASTASIS_BY_ERBB2_ISOFORM_1 46 -0.360551 -1.20896 0.258065 0.459072 0 down vs 1MORI_MATURE_B_LYMPHOCYTE_UP http://www.broadinstitute.org/gsea/msigdb/cards/MORI_MATURE_B_LYMPHOCYTE_UP 90 -0.363216 -1.2104 0.275862 0.459077 0 down vs 1YU_MYC_TARGETS_UP http://www.broadinstitute.org/gsea/msigdb/cards/YU_MYC_TARGETS_UP 40 -0.451702 -1.20943 0.206897 0.459182 0 down vs 1TIEN_INTESTINE_PROBIOTICS_24HR_DN http://www.broadinstitute.org/gsea/msigdb/cards/TIEN_INTESTINE_PROBIOTICS_24HR_DN 213 0.39831 1.54684 0.0111111 0.460551 0 up vs 1MCGOWAN_RSP6_TARGETS_UP http://www.broadinstitute.org/gsea/msigdb/cards/MCGOWAN_RSP6_TARGETS_UP 18 0.488251 1.54514 0.0153846 0.462106 0 up vs 1IRITANI_MAD1_TARGETS_DN http://www.broadinstitute.org/gsea/msigdb/cards/IRITANI_MAD1_TARGETS_DN 47 0.557559 1.54735 0.0897436 0.462782 0 up vs 1HUANG_DASATINIB_RESISTANCE_DN http://www.broadinstitute.org/gsea/msigdb/cards/HUANG_DASATINIB_RESISTANCE_DN 66 0.45472 1.55575 0.0133333 0.463895 0 up vs 1CHEN_NEUROBLASTOMA_COPY_NUMBER_GAINS http://www.broadinstitute.org/gsea/msigdb/cards/CHEN_NEUROBLASTOMA_COPY_NUMBER_GAINS 49 0.441815 1.55276 0.0131579 0.464909 0 up vs 1JISON_SICKLE_CELL_DISEASE_DN http://www.broadinstitute.org/gsea/msigdb/cards/JISON_SICKLE_CELL_DISEASE_DN 177 0.415789 1.50049 0.037037 0.465075 0 up vs 1DANG_MYC_TARGETS_UP http://www.broadinstitute.org/gsea/msigdb/cards/DANG_MYC_TARGETS_UP 142 0.498915 1.54808 0.0625 0.465111 0 up vs 1DELACROIX_RAR_TARGETS_UP http://www.broadinstitute.org/gsea/msigdb/cards/DELACROIX_RAR_TARGETS_UP 47 -0.315454 -1.20314 0.185185 0.465709 0 down vs 1NAGY_TFTC_COMPONENTS_HUMAN http://www.broadinstitute.org/gsea/msigdb/cards/NAGY_TFTC_COMPONENTS_HUMAN 19 0.540802 1.54215 0.0379747 0.466201 0 up vs 1SCIBETTA_KDM5B_TARGETS_UP http://www.broadinstitute.org/gsea/msigdb/cards/SCIBETTA_KDM5B_TARGETS_UP 17 0.481557 1.49503 0.0394737 0.466401 0 up vs 1LEIN_ASTROCYTE_MARKERS http://www.broadinstitute.org/gsea/msigdb/cards/LEIN_ASTROCYTE_MARKERS 41 0.449971 1.55351 0.0149254 0.466614 0 up vs 1MODY_HIPPOCAMPUS_POSTNATAL http://www.broadinstitute.org/gsea/msigdb/cards/MODY_HIPPOCAMPUS_POSTNATAL 62 0.410988 1.55016 0 0.46681 0 up vs 1GARGALOVIC_RESPONSE_TO_OXIDIZED_PHOSPHOLIPIDS_BLUE_DN http://www.broadinstitute.org/gsea/msigdb/cards/GARGALOVIC_RESPONSE_TO_OXIDIZED_PHOSPHOLIPIDS_BLUE_DN 57 0.443085 1.50403 0.0533333 0.466842 0 up vs 1TOOKER_GEMCITABINE_RESISTANCE_UP http://www.broadinstitute.org/gsea/msigdb/cards/TOOKER_GEMCITABINE_RESISTANCE_UP 76 0.438524 1.50073 0.0238095 0.46701 0 up vs 1GRUETZMANN_PANCREATIC_CANCER_DN http://www.broadinstitute.org/gsea/msigdb/cards/GRUETZMANN_PANCREATIC_CANCER_DN 200 0.370132 1.50267 0.0470588 0.467158 0 up vs 1GOLDRATH_HOMEOSTATIC_PROLIFERATION http://www.broadinstitute.org/gsea/msigdb/cards/GOLDRATH_HOMEOSTATIC_PROLIFERATION 168 0.412447 1.50153 0 0.467518 0 up vs 1RICKMAN_TUMOR_DIFFERENTIATED_WELL_VS_MODERATELY_UP http://www.broadinstitute.org/gsea/msigdb/cards/RICKMAN_TUMOR_DIFFERENTIATED_WELL_VS_MODERATELY_UP 103 0.461812 1.54846 0.0972222 0.467857 0 up vs 1LANDEMAINE_LUNG_METASTASIS http://www.broadinstitute.org/gsea/msigdb/cards/LANDEMAINE_LUNG_METASTASIS 21 -0.378687 -1.20064 0.233333 0.468096 0 down vs 1WANG_RESPONSE_TO_FORSKOLIN_UP http://www.broadinstitute.org/gsea/msigdb/cards/WANG_RESPONSE_TO_FORSKOLIN_UP 23 0.482523 1.49528 0.0675676 0.468428 0 up vs 1PURBEY_TARGETS_OF_CTBP1_AND_SATB1_UP http://www.broadinstitute.org/gsea/msigdb/cards/PURBEY_TARGETS_OF_CTBP1_AND_SATB1_UP 82 0.404592 1.53262 0.0117647 0.468466 0 up vs 1GESERICK_TERT_TARGETS_DN http://www.broadinstitute.org/gsea/msigdb/cards/GESERICK_TERT_TARGETS_DN 21 -0.424612 -1.20139 0.25 0.46864 0 down vs 1SCHLESINGER_METHYLATED_DE_NOVO_IN_CANCER http://www.broadinstitute.org/gsea/msigdb/cards/SCHLESINGER_METHYLATED_DE_NOVO_IN_CANCER 86 -0.265172 -1.2008 0.217391 0.468704 0 down vs 1HASLINGER_B_CLL_WITH_13Q14_DELETION http://www.broadinstitute.org/gsea/msigdb/cards/HASLINGER_B_CLL_WITH_13Q14_DELETION 24 0.447202 1.5043 0.0135135 0.468785 0 up vs 1LANG_MYB_FAMILY_TARGETS http://www.broadinstitute.org/gsea/msigdb/cards/LANG_MYB_FAMILY_TARGETS 29 0.471509 1.50791 0.0379747 0.470094 0 up vs 1BROWNE_HCMV_INFECTION_16HR_DN http://www.broadinstitute.org/gsea/msigdb/cards/BROWNE_HCMV_INFECTION_16HR_DN 85 0.410886 1.49567 0.025641 0.470197 0 up vs 1PIEPOLI_LGI1_TARGETS_UP http://www.broadinstitute.org/gsea/msigdb/cards/PIEPOLI_LGI1_TARGETS_UP 15 0.475155 1.50476 0.0322581 0.470357 0 up vs 1IVANOVA_HEMATOPOIESIS_STEM_CELL_SHORT_TERM http://www.broadinstitute.org/gsea/msigdb/cards/IVANOVA_HEMATOPOIESIS_STEM_CELL_SHORT_TERM 31 0.509103 1.53309 0.0533333 0.470376 0 up vs 1GOBERT_CORE_OLIGODENDROCYTE_DIFFERENTIATION http://www.broadinstitute.org/gsea/msigdb/cards/GOBERT_CORE_OLIGODENDROCYTE_DIFFERENTIATION 40 0.442946 1.49643 0.057971 0.470618 0 up vs 1NIELSEN_LEIOMYOSARCOMA_DN http://www.broadinstitute.org/gsea/msigdb/cards/NIELSEN_LEIOMYOSARCOMA_DN 17 0.484106 1.50571 0.0416667 0.470851 0 up vs 1STANELLE_E2F1_TARGETS http://www.broadinstitute.org/gsea/msigdb/cards/STANELLE_E2F1_TARGETS 29 -0.335485 -1.19886 0.193548 0.471259 0 down vs 1WATANABE_COLON_CANCER_MSI_VS_MSS_DN http://www.broadinstitute.org/gsea/msigdb/cards/WATANABE_COLON_CANCER_MSI_VS_MSS_DN 79 0.438005 1.5119 0.0298507 0.471367 0 up vs 1CHEOK_RESPONSE_TO_HD_MTX_DN http://www.broadinstitute.org/gsea/msigdb/cards/CHEOK_RESPONSE_TO_HD_MTX_DN 23 0.507567 1.49688 0.144737 0.471756 0 up vs 1DEN_INTERACT_WITH_LCA5 http://www.broadinstitute.org/gsea/msigdb/cards/DEN_INTERACT_WITH_LCA5 26 0.55494 1.53003 0.1 0.47188 0 up vs 1HEDENFALK_BREAST_CANCER_BRACX_UP http://www.broadinstitute.org/gsea/msigdb/cards/HEDENFALK_BREAST_CANCER_BRACX_UP 20 0.501606 1.53883 0.0547945 0.471992 0 up vs 1ROSS_AML_OF_FAB_M7_TYPE http://www.broadinstitute.org/gsea/msigdb/cards/ROSS_AML_OF_FAB_M7_TYPE 68 0.414551 1.5126 0.0235294 0.472202 0 up vs 1STARK_PREFRONTAL_CORTEX_22Q11_DELETION_DN http://www.broadinstitute.org/gsea/msigdb/cards/STARK_PREFRONTAL_CORTEX_22Q11_DELETION_DN 494 0.420067 1.5062 0.0470588 0.472355 0 up vs 1MORI_PLASMA_CELL_UP http://www.broadinstitute.org/gsea/msigdb/cards/MORI_PLASMA_CELL_UP 50 0.423926 1.50826 0.0394737 0.472474 0 up vs 1STARK_HYPPOCAMPUS_22Q11_DELETION_UP http://www.broadinstitute.org/gsea/msigdb/cards/STARK_HYPPOCAMPUS_22Q11_DELETION_UP 52 0.439057 1.51573 0 0.472652 0 up vs 1HOLLEMAN_VINCRISTINE_RESISTANCE_ALL_UP http://www.broadinstitute.org/gsea/msigdb/cards/HOLLEMAN_VINCRISTINE_RESISTANCE_ALL_UP 27 0.493129 1.51661 0.0645161 0.472771 0 up vs 1BOYLAN_MULTIPLE_MYELOMA_C_D_UP http://www.broadinstitute.org/gsea/msigdb/cards/BOYLAN_MULTIPLE_MYELOMA_C_D_UP 134 0.413457 1.51336 0.0232558 0.472839 0 up vs 1BUCKANOVICH_T_LYMPHOCYTE_HOMING_ON_TUMOR_DN http://www.broadinstitute.org/gsea/msigdb/cards/BUCKANOVICH_T_LYMPHOCYTE_HOMING_ON_TUMOR_DN 22 0.490508 1.51457 0.0333333 0.472957 0 up vs 1ELVIDGE_HIF1A_TARGETS_UP http://www.broadinstitute.org/gsea/msigdb/cards/ELVIDGE_HIF1A_TARGETS_UP 64 0.453409 1.51038 0.0641026 0.473008 0 up vs 1WEST_ADRENOCORTICAL_CARCINOMA_VS_ADENOMA_UP http://www.broadinstitute.org/gsea/msigdb/cards/WEST_ADRENOCORTICAL_CARCINOMA_VS_ADENOMA_UP 20 0.458693 1.53335 0.0375 0.473255 0 up vs 1HOFFMANN_SMALL_PRE_BII_TO_IMMATURE_B_LYMPHOCYTE_UP http://www.broadinstitute.org/gsea/msigdb/cards/HOFFMANN_SMALL_PRE_BII_TO_IMMATURE_B_LYMPHOCYTE_UP 70 -0.31766 -1.19718 0.32 0.473677 0 down vs 1LAIHO_COLORECTAL_CANCER_SERRATED_DN http://www.broadinstitute.org/gsea/msigdb/cards/LAIHO_COLORECTAL_CANCER_SERRATED_DN 80 0.485911 1.52423 0.0588235 0.473925 0 up vs 1WIERENGA_PML_INTERACTOME http://www.broadinstitute.org/gsea/msigdb/cards/WIERENGA_PML_INTERACTOME 41 0.475439 1.51705 0.0740741 0.474558 0 up vs 1SCHUHMACHER_MYC_TARGETS_UP http://www.broadinstitute.org/gsea/msigdb/cards/SCHUHMACHER_MYC_TARGETS_UP 78 0.523585 1.50866 0.1375 0.474578 0 up vs 1PENG_GLUTAMINE_DEPRIVATION_DN http://www.broadinstitute.org/gsea/msigdb/cards/PENG_GLUTAMINE_DEPRIVATION_DN 333 0.44787 1.53656 0.0568182 0.474991 0 up vs 1SMITH_LIVER_CANCER http://www.broadinstitute.org/gsea/msigdb/cards/SMITH_LIVER_CANCER 44 0.446474 1.53351 0.037037 0.476413 0 up vs 1HEDENFALK_BREAST_CANCER_BRACX_DN http://www.broadinstitute.org/gsea/msigdb/cards/HEDENFALK_BREAST_CANCER_BRACX_DN 20 0.555835 1.5174 0.0519481 0.477126 0 up vs 1HEDENFALK_BREAST_CANCER_HEREDITARY_VS_SPORADIC http://www.broadinstitute.org/gsea/msigdb/cards/HEDENFALK_BREAST_CANCER_HEREDITARY_VS_SPORADIC 47 0.496869 1.52428 0.0609756 0.47716 0 up vs 1FONTAINE_PAPILLARY_THYROID_CARCINOMA_DN http://www.broadinstitute.org/gsea/msigdb/cards/FONTAINE_PAPILLARY_THYROID_CARCINOMA_DN 78 0.410261 1.51832 0 0.477331 0 up vs 1WANG_ESOPHAGUS_CANCER_VS_NORMAL_DN http://www.broadinstitute.org/gsea/msigdb/cards/WANG_ESOPHAGUS_CANCER_VS_NORMAL_DN 99 -0.288971 -1.19416 0.2 0.47828 0 down vs 1POMEROY_MEDULLOBLASTOMA_DESMOPLASIC_VS_CLASSIC_DN http://www.broadinstitute.org/gsea/msigdb/cards/POMEROY_MEDULLOBLASTOMA_DESMOPLASIC_VS_CLASSIC_DN 59 0.473293 1.5191 0.084507 0.478362 0 up vs 1ZHANG_RESPONSE_TO_CANTHARIDIN_UP http://www.broadinstitute.org/gsea/msigdb/cards/ZHANG_RESPONSE_TO_CANTHARIDIN_UP 19 -0.40005 -1.19239 0.21875 0.478822 0 down vs 1NIELSEN_GIST_AND_SYNOVIAL_SARCOMA_DN http://www.broadinstitute.org/gsea/msigdb/cards/NIELSEN_GIST_AND_SYNOVIAL_SARCOMA_DN 20 -0.473427 -1.19136 0.263158 0.478844 0 down vs 1GAVIN_FOXP3_TARGETS_CLUSTER_P6 http://www.broadinstitute.org/gsea/msigdb/cards/GAVIN_FOXP3_TARGETS_CLUSTER_P6 90 -0.361808 -1.19072 0.37037 0.478886 0 down vs 1MATTHEWS_AP1_TARGETS http://www.broadinstitute.org/gsea/msigdb/cards/MATTHEWS_AP1_TARGETS 17 -0.32301 -1.19336 0.217391 0.478927 0 down vs 1ZHANG_GATA6_TARGETS_DN http://www.broadinstitute.org/gsea/msigdb/cards/ZHANG_GATA6_TARGETS_DN 62 -0.279387 -1.1896 0.0952381 0.479409 0 down vs 1WEIGEL_OXIDATIVE_STRESS_BY_HNE_AND_H2O2 http://www.broadinstitute.org/gsea/msigdb/cards/WEIGEL_OXIDATIVE_STRESS_BY_HNE_AND_H2O2 39 0.45766 1.5336 0.0246914 0.479558 0 up vs 1PARK_APL_PATHOGENESIS_DN http://www.broadinstitute.org/gsea/msigdb/cards/PARK_APL_PATHOGENESIS_DN 49 -0.342225 -1.19144 0.222222 0.479654 0 down vs 1BURTON_ADIPOGENESIS_PEAK_AT_24HR http://www.broadinstitute.org/gsea/msigdb/cards/BURTON_ADIPOGENESIS_PEAK_AT_24HR 43 -0.398301 -1.1924 0.285714 0.479762 0 down vs 1JIANG_AGING_CEREBRAL_CORTEX_DN http://www.broadinstitute.org/gsea/msigdb/cards/JIANG_AGING_CEREBRAL_CORTEX_DN 53 0.446186 1.52453 0.0487805 0.479985 0 up vs 1CHIARADONNA_NEOPLASTIC_TRANSFORMATION_KRAS_CDC25_DN http://www.broadinstitute.org/gsea/msigdb/cards/CHIARADONNA_NEOPLASTIC_TRANSFORMATION_KRAS_CDC25_DN 50 -0.317031 -1.18971 0.206897 0.480183 0 down vs 1RHODES_CANCER_META_SIGNATURE http://www.broadinstitute.org/gsea/msigdb/cards/RHODES_CANCER_META_SIGNATURE 64 0.502596 1.52083 0.106667 0.480299 0 up vs 1ELVIDGE_HYPOXIA_DN http://www.broadinstitute.org/gsea/msigdb/cards/ELVIDGE_HYPOXIA_DN 141 0.428153 1.52533 0.0232558 0.481193 0 up vs 1ELVIDGE_HIF1A_AND_HIF2A_TARGETS_UP http://www.broadinstitute.org/gsea/msigdb/cards/ELVIDGE_HIF1A_AND_HIF2A_TARGETS_UP 39 0.476762 1.51919 0.0657895 0.48134 0 up vs 1HENDRICKS_SMARCA4_TARGETS_UP http://www.broadinstitute.org/gsea/msigdb/cards/HENDRICKS_SMARCA4_TARGETS_UP 55 -0.332057 -1.18762 0.28125 0.481455 0 down vs 1GOLUB_ALL_VS_AML_DN http://www.broadinstitute.org/gsea/msigdb/cards/GOLUB_ALL_VS_AML_DN 24 -0.365498 -1.18788 0.241379 0.481824 0 down vs 1MEISSNER_NPC_ICP_WITH_H3_UNMETHYLATED http://www.broadinstitute.org/gsea/msigdb/cards/MEISSNER_NPC_ICP_WITH_H3_UNMETHYLATED 24 -0.341537 -1.18705 0.205882 0.481896 0 down vs 1WONG_ENDMETRIUM_CANCER_UP http://www.broadinstitute.org/gsea/msigdb/cards/WONG_ENDMETRIUM_CANCER_UP 25 -0.312716 -1.1853 0.272727 0.483702 0 down vs 1KONG_E2F3_TARGETS http://www.broadinstitute.org/gsea/msigdb/cards/KONG_E2F3_TARGETS 96 -0.437323 -1.18342 0.310345 0.485966 0 down vs 1JOHNSTONE_PARVB_TARGETS_2_UP http://www.broadinstitute.org/gsea/msigdb/cards/JOHNSTONE_PARVB_TARGETS_2_UP 133 -0.248886 -1.18236 0.15 0.486859 0 down vs 1MEISSNER_ES_ICP_WITH_H3K4ME3 http://www.broadinstitute.org/gsea/msigdb/cards/MEISSNER_ES_ICP_WITH_H3K4ME3 31 -0.279815 -1.18173 0.136364 0.48704 0 down vs 1FERRANDO_T_ALL_WITH_MLL_ENL_FUSION_DN http://www.broadinstitute.org/gsea/msigdb/cards/FERRANDO_T_ALL_WITH_MLL_ENL_FUSION_DN 87 -0.294609 -1.18112 0.25 0.48725 0 down vs 1WHITFIELD_CELL_CYCLE_S http://www.broadinstitute.org/gsea/msigdb/cards/WHITFIELD_CELL_CYCLE_S 158 -0.329673 -1.17936 0.347826 0.488987 0 down vs 1HERNANDEZ_MITOTIC_ARREST_BY_DOCETAXEL_1_DN http://www.broadinstitute.org/gsea/msigdb/cards/HERNANDEZ_MITOTIC_ARREST_BY_DOCETAXEL_1_DN 38 -0.299386 -1.17981 0.24 0.489058 0 down vs 1CUI_TCF21_TARGETS_UP http://www.broadinstitute.org/gsea/msigdb/cards/CUI_TCF21_TARGETS_UP 37 0.442642 1.48565 0.0606061 0.489169 0 up vs 1NAGY_STAGA_COMPONENTS_HUMAN http://www.broadinstitute.org/gsea/msigdb/cards/NAGY_STAGA_COMPONENTS_HUMAN 15 0.541966 1.48369 0.0789474 0.489237 0 up vs 1VANTVEER_BREAST_CANCER_METASTASIS_UP http://www.broadinstitute.org/gsea/msigdb/cards/VANTVEER_BREAST_CANCER_METASTASIS_UP 54 0.458449 1.48443 0.0454545 0.489846 0 up vs 1FUJII_YBX1_TARGETS_DN http://www.broadinstitute.org/gsea/msigdb/cards/FUJII_YBX1_TARGETS_DN 197 -0.357774 -1.17631 0.4 0.492179 0 down vs 1WEIGEL_OXIDATIVE_STRESS_BY_TBH_AND_H2O2 http://www.broadinstitute.org/gsea/msigdb/cards/WEIGEL_OXIDATIVE_STRESS_BY_TBH_AND_H2O2 36 -0.293971 -1.1764 0.26087 0.492929 0 down vs 1SESTO_RESPONSE_TO_UV_C7 http://www.broadinstitute.org/gsea/msigdb/cards/SESTO_RESPONSE_TO_UV_C7 68 -0.244213 -1.17457 0.166667 0.494757 0 down vs 1SANSOM_APC_TARGETS_UP http://www.broadinstitute.org/gsea/msigdb/cards/SANSOM_APC_TARGETS_UP 122 0.392464 1.47649 0.0574713 0.495791 0 up vs 1DEBIASI_APOPTOSIS_BY_REOVIRUS_INFECTION_DN http://www.broadinstitute.org/gsea/msigdb/cards/DEBIASI_APOPTOSIS_BY_REOVIRUS_INFECTION_DN 281 0.391825 1.47937 0.0126582 0.49616 0 up vs 1LABBE_TGFB1_TARGETS_DN http://www.broadinstitute.org/gsea/msigdb/cards/LABBE_TGFB1_TARGETS_DN 107 -0.222838 -1.17327 0.136364 0.496655 0 down vs 1BOGNI_TREATMENT_RELATED_MYELOID_LEUKEMIA_UP http://www.broadinstitute.org/gsea/msigdb/cards/BOGNI_TREATMENT_RELATED_MYELOID_LEUKEMIA_UP 29 0.40555 1.47516 0.0273973 0.496803 0 up vs 1ROVERSI_GLIOMA_LOH_REGIONS http://www.broadinstitute.org/gsea/msigdb/cards/ROVERSI_GLIOMA_LOH_REGIONS 43 0.42975 1.47988 0.0136986 0.497435 0 up vs 1SUMI_HNF4A_TARGETS http://www.broadinstitute.org/gsea/msigdb/cards/SUMI_HNF4A_TARGETS 32 0.462711 1.47759 0.0847458 0.498264 0 up vs 1BAE_BRCA1_TARGETS_DN http://www.broadinstitute.org/gsea/msigdb/cards/BAE_BRCA1_TARGETS_DN 32 0.456851 1.47653 0.0657895 0.498381 0 up vs 1LE_EGR2_TARGETS_UP http://www.broadinstitute.org/gsea/msigdb/cards/LE_EGR2_TARGETS_UP 107 -0.34254 -1.1679 0.363636 0.499163 0 down vs 1MAGRANGEAS_MULTIPLE_MYELOMA_IGLL_VS_IGLK_DN http://www.broadinstitute.org/gsea/msigdb/cards/MAGRANGEAS_MULTIPLE_MYELOMA_IGLL_VS_IGLK_DN 23 -0.290801 -1.16814 0.173913 0.499702 0 down vs 1BOYAULT_LIVER_CANCER_SUBCLASS_G1_DN http://www.broadinstitute.org/gsea/msigdb/cards/BOYAULT_LIVER_CANCER_SUBCLASS_G1_DN 40 -0.311861 -1.16724 0.259259 0.499912 0 down vs 1SMID_BREAST_CANCER_RELAPSE_IN_PLEURA_DN http://www.broadinstitute.org/gsea/msigdb/cards/SMID_BREAST_CANCER_RELAPSE_IN_PLEURA_DN 27 -0.301402 -1.16652 0.25 0.499972 0 down vs 1TOMLINS_PROSTATE_CANCER_DN http://www.broadinstitute.org/gsea/msigdb/cards/TOMLINS_PROSTATE_CANCER_DN 40 -0.336571 -1.16833 0.275862 0.500304 0 down vs 1ALCALAY_AML_BY_NPM1_LOCALIZATION_DN http://www.broadinstitute.org/gsea/msigdb/cards/ALCALAY_AML_BY_NPM1_LOCALIZATION_DN 183 -0.25803 -1.17078 0.263158 0.50046 0 down vs 1MIKKELSEN_MEF_LCP_WITH_H3K4ME3 http://www.broadinstitute.org/gsea/msigdb/cards/MIKKELSEN_MEF_LCP_WITH_H3K4ME3 124 -0.289471 -1.16662 0.32 0.50048 0 down vs 1LABBE_TARGETS_OF_TGFB1_AND_WNT3A_UP http://www.broadinstitute.org/gsea/msigdb/cards/LABBE_TARGETS_OF_TGFB1_AND_WNT3A_UP 108 -0.247387 -1.16833 0.190476 0.50127 0 down vs 1SESTO_RESPONSE_TO_UV_C1 http://www.broadinstitute.org/gsea/msigdb/cards/SESTO_RESPONSE_TO_UV_C1 72 -0.288428 -1.16529 0.388889 0.501578 0 down vs 1ZHONG_RESPONSE_TO_AZACITIDINE_AND_TSA_UP http://www.broadinstitute.org/gsea/msigdb/cards/ZHONG_RESPONSE_TO_AZACITIDINE_AND_TSA_UP 179 -0.223883 -1.16859 0.133333 0.501846 0 down vs 1TURASHVILI_BREAST_LOBULAR_CARCINOMA_VS_DUCTAL_NORMAL_DN http://www.broadinstitute.org/gsea/msigdb/cards/TURASHVILI_BREAST_LOBULAR_CARCINOMA_VS_DUCTAL_NORMAL_DN 88 -0.28007 -1.16876 0.208333 0.502395 0 down vs 1DASU_IL6_SIGNALING_SCAR_UP http://www.broadinstitute.org/gsea/msigdb/cards/DASU_IL6_SIGNALING_SCAR_UP 30 -0.323013 -1.16901 0.291667 0.502885 0 down vs 1NEWMAN_ERCC6_TARGETS_UP http://www.broadinstitute.org/gsea/msigdb/cards/NEWMAN_ERCC6_TARGETS_UP 24 -0.3461 -1.16332 0.269231 0.503677 0 down vs 1PICCALUGA_ANGIOIMMUNOBLASTIC_LYMPHOMA_DN http://www.broadinstitute.org/gsea/msigdb/cards/PICCALUGA_ANGIOIMMUNOBLASTIC_LYMPHOMA_DN 129 -0.331002 -1.16354 0.277778 0.504129 0 down vs 1ZHAN_MULTIPLE_MYELOMA_HP_DN http://www.broadinstitute.org/gsea/msigdb/cards/ZHAN_MULTIPLE_MYELOMA_HP_DN 43 -0.27896 -1.16149 0.318182 0.505729 0 down vs 1XU_HGF_TARGETS_INDUCED_BY_AKT1_48HR_DN http://www.broadinstitute.org/gsea/msigdb/cards/XU_HGF_TARGETS_INDUCED_BY_AKT1_48HR_DN 27 -0.399822 -1.16153 0.269231 0.506598 0 down vs 1YAMASHITA_LIVER_CANCER_STEM_CELL_DN http://www.broadinstitute.org/gsea/msigdb/cards/YAMASHITA_LIVER_CANCER_STEM_CELL_DN 75 -0.276885 -1.15943 0.275862 0.50896 0 down vs 1CHICAS_RB1_TARGETS_GROWING http://www.broadinstitute.org/gsea/msigdb/cards/CHICAS_RB1_TARGETS_GROWING 236 -0.296129 -1.15715 0.380952 0.509624 0 down vs 1CHUNG_BLISTER_CYTOTOXICITY_DN http://www.broadinstitute.org/gsea/msigdb/cards/CHUNG_BLISTER_CYTOTOXICITY_DN 42 -0.330745 -1.15838 0.275862 0.510179 0 down vs 1FIGUEROA_AML_METHYLATION_CLUSTER_4_DN http://www.broadinstitute.org/gsea/msigdb/cards/FIGUEROA_AML_METHYLATION_CLUSTER_4_DN 15 -0.402779 -1.15727 0.315789 0.510433 0 down vs 1MORI_IMMATURE_B_LYMPHOCYTE_DN http://www.broadinstitute.org/gsea/msigdb/cards/MORI_IMMATURE_B_LYMPHOCYTE_DN 90 -0.382891 -1.15749 0.357143 0.511158 0 down vs 1WATANABE_ULCERATIVE_COLITIS_WITH_CANCER_UP http://www.broadinstitute.org/gsea/msigdb/cards/WATANABE_ULCERATIVE_COLITIS_WITH_CANCER_UP 18 0.492876 1.4691 0.121622 0.51176 0 up vs 1KARAKAS_TGFB1_SIGNALING http://www.broadinstitute.org/gsea/msigdb/cards/KARAKAS_TGFB1_SIGNALING 18 -0.363593 -1.15508 0.235294 0.512006 0 down vs 1MARKS_HDAC_TARGETS_DN http://www.broadinstitute.org/gsea/msigdb/cards/MARKS_HDAC_TARGETS_DN 15 -0.386465 -1.15522 0.34375 0.512613 0 down vs 1YAO_TEMPORAL_RESPONSE_TO_PROGESTERONE_CLUSTER_1 http://www.broadinstitute.org/gsea/msigdb/cards/YAO_TEMPORAL_RESPONSE_TO_PROGESTERONE_CLUSTER_1 67 -0.264687 -1.15409 0.208333 0.513089 0 down vs 1RUTELLA_RESPONSE_TO_HGF_DN http://www.broadinstitute.org/gsea/msigdb/cards/RUTELLA_RESPONSE_TO_HGF_DN 230 -0.250775 -1.15348 0.26087 0.51321 0 down vs 1NIELSEN_GIST http://www.broadinstitute.org/gsea/msigdb/cards/NIELSEN_GIST 93 0.427373 1.45688 0.0394737 0.514216 0 up vs 1JIANG_AGING_CEREBRAL_CORTEX_UP http://www.broadinstitute.org/gsea/msigdb/cards/JIANG_AGING_CEREBRAL_CORTEX_UP 36 0.433039 1.45779 0.0722892 0.514644 0 up vs 1BROWNE_HCMV_INFECTION_12HR_UP http://www.broadinstitute.org/gsea/msigdb/cards/BROWNE_HCMV_INFECTION_12HR_UP 107 -0.280204 -1.15241 0.28 0.514866 0 down vs 1HAHTOLA_CTCL_CUTANEOUS http://www.broadinstitute.org/gsea/msigdb/cards/HAHTOLA_CTCL_CUTANEOUS 25 -0.349159 -1.15091 0.347826 0.515071 0 down vs 1BRACHAT_RESPONSE_TO_METHOTREXATE_UP http://www.broadinstitute.org/gsea/msigdb/cards/BRACHAT_RESPONSE_TO_METHOTREXATE_UP 25 -0.33728 -1.15129 0.1875 0.515128 0 down vs 1LEE_AGING_NEOCORTEX_UP http://www.broadinstitute.org/gsea/msigdb/cards/LEE_AGING_NEOCORTEX_UP 89 -0.246267 -1.14734 0.222222 0.515504 0 down vs 1DARWICHE_PAPILLOMA_RISK_HIGH_UP http://www.broadinstitute.org/gsea/msigdb/cards/DARWICHE_PAPILLOMA_RISK_HIGH_UP 145 -0.203314 -1.1479 0 0.515561 0 down vs 1HOSHIDA_LIVER_CANCER_SURVIVAL_UP http://www.broadinstitute.org/gsea/msigdb/cards/HOSHIDA_LIVER_CANCER_SURVIVAL_UP 73 -0.263185 -1.15146 0.272727 0.515822 0 down vs 1SHIN_B_CELL_LYMPHOMA_CLUSTER_9 http://www.broadinstitute.org/gsea/msigdb/cards/SHIN_B_CELL_LYMPHOMA_CLUSTER_9 19 -0.450328 -1.14818 0.236842 0.515989 0 down vs 1LAIHO_COLORECTAL_CANCER_SERRATED_UP http://www.broadinstitute.org/gsea/msigdb/cards/LAIHO_COLORECTAL_CANCER_SERRATED_UP 108 -0.252858 -1.15004 0.307692 0.516022 0 down vs 1PODAR_RESPONSE_TO_ADAPHOSTIN_UP http://www.broadinstitute.org/gsea/msigdb/cards/PODAR_RESPONSE_TO_ADAPHOSTIN_UP 145 -0.272369 -1.1486 0.375 0.516133 0 down vs 1BOYLAN_MULTIPLE_MYELOMA_PCA3_DN http://www.broadinstitute.org/gsea/msigdb/cards/BOYLAN_MULTIPLE_MYELOMA_PCA3_DN 68 -0.251255 -1.14873 0.310345 0.516765 0 down vs 1CERVERA_SDHB_TARGETS_1_DN http://www.broadinstitute.org/gsea/msigdb/cards/CERVERA_SDHB_TARGETS_1_DN 37 0.409479 1.45802 0.0655738 0.516826 0 up vs 1OSADA_ASCL1_TARGETS_DN http://www.broadinstitute.org/gsea/msigdb/cards/OSADA_ASCL1_TARGETS_DN 24 -0.352777 -1.14893 0.289474 0.517284 0 down vs 1CAIRO_PML_TARGETS_BOUND_BY_MYC_UP http://www.broadinstitute.org/gsea/msigdb/cards/CAIRO_PML_TARGETS_BOUND_BY_MYC_UP 23 0.536886 1.46619 0.116883 0.517345 0 up vs 1MASSARWEH_TAMOXIFEN_RESISTANCE_DN http://www.broadinstitute.org/gsea/msigdb/cards/MASSARWEH_TAMOXIFEN_RESISTANCE_DN 245 0.376337 1.45848 0.0519481 0.518192 0 up vs 1BONOME_OVARIAN_CANCER_POOR_SURVIVAL_DN http://www.broadinstitute.org/gsea/msigdb/cards/BONOME_OVARIAN_CANCER_POOR_SURVIVAL_DN 21 0.479888 1.46012 0.0379747 0.518389 0 up vs 1MISHRA_CARCINOMA_ASSOCIATED_FIBROBLAST_DN http://www.broadinstitute.org/gsea/msigdb/cards/MISHRA_CARCINOMA_ASSOCIATED_FIBROBLAST_DN 23 -0.310521 -1.14351 0.233333 0.518718 0 down vs 1SOTIRIOU_BREAST_CANCER_GRADE_1_VS_3_UP http://www.broadinstitute.org/gsea/msigdb/cards/SOTIRIOU_BREAST_CANCER_GRADE_1_VS_3_UP 148 -0.385976 -1.14383 0.37931 0.51912 0 down vs 1BHATI_G2M_ARREST_BY_2METHOXYESTRADIOL_UP http://www.broadinstitute.org/gsea/msigdb/cards/BHATI_G2M_ARREST_BY_2METHOXYESTRADIOL_UP 115 -0.267444 -1.14414 0.266667 0.519381 0 down vs 1FAELT_B_CLL_WITH_VH3_21_DN http://www.broadinstitute.org/gsea/msigdb/cards/FAELT_B_CLL_WITH_VH3_21_DN 48 -0.283689 -1.14452 0.3125 0.519558 0 down vs 1RODRIGUES_THYROID_CARCINOMA_DN http://www.broadinstitute.org/gsea/msigdb/cards/RODRIGUES_THYROID_CARCINOMA_DN 73 -0.26726 -1.14477 0.307692 0.519849 0 down vs 1CHIANG_LIVER_CANCER_SUBCLASS_POLYSOMY7_DN http://www.broadinstitute.org/gsea/msigdb/cards/CHIANG_LIVER_CANCER_SUBCLASS_POLYSOMY7_DN 24 0.500693 1.45174 0.057971 0.520102 0 up vs 1PETROVA_PROX1_TARGETS_DN http://www.broadinstitute.org/gsea/msigdb/cards/PETROVA_PROX1_TARGETS_DN 63 -0.335646 -1.14115 0.387097 0.520207 0 down vs 1LUI_THYROID_CANCER_CLUSTER_3 http://www.broadinstitute.org/gsea/msigdb/cards/LUI_THYROID_CANCER_CLUSTER_3 28 0.525966 1.45863 0.170732 0.520414 0 up vs 1COLIN_PILOCYTIC_ASTROCYTOMA_VS_GLIOBLASTOMA_DN http://www.broadinstitute.org/gsea/msigdb/cards/COLIN_PILOCYTIC_ASTROCYTOMA_VS_GLIOBLASTOMA_DN 28 0.4239 1.4603 0.0540541 0.52072 0 up vs 1DARWICHE_SKIN_TUMOR_PROMOTER_UP http://www.broadinstitute.org/gsea/msigdb/cards/DARWICHE_SKIN_TUMOR_PROMOTER_UP 138 -0.200355 -1.1414 0 0.52072 0 down vs 1MISSIAGLIA_REGULATED_BY_METHYLATION_DN http://www.broadinstitute.org/gsea/msigdb/cards/MISSIAGLIA_REGULATED_BY_METHYLATION_DN 119 -0.363218 -1.14209 0.24 0.520767 0 down vs 1FAELT_B_CLL_WITH_VH_REARRANGEMENTS_UP http://www.broadinstitute.org/gsea/msigdb/cards/FAELT_B_CLL_WITH_VH_REARRANGEMENTS_UP 48 -0.243086 -1.14168 0.2 0.520786 0 down vs 1JAZAG_TGFB1_SIGNALING_DN http://www.broadinstitute.org/gsea/msigdb/cards/JAZAG_TGFB1_SIGNALING_DN 34 0.401819 1.45236 0.0121951 0.520962 0 up vs 1ZEMBUTSU_SENSITIVITY_TO_DOXORUBICIN http://www.broadinstitute.org/gsea/msigdb/cards/ZEMBUTSU_SENSITIVITY_TO_DOXORUBICIN 17 -0.356388 -1.14033 0.310345 0.521093 0 down vs 1MORI_LARGE_PRE_BII_LYMPHOCYTE_UP http://www.broadinstitute.org/gsea/msigdb/cards/MORI_LARGE_PRE_BII_LYMPHOCYTE_UP 84 -0.383694 -1.13913 0.32 0.521184 0 down vs 1WATANABE_RECTAL_CANCER_RADIOTHERAPY_RESPONSIVE_UP http://www.broadinstitute.org/gsea/msigdb/cards/WATANABE_RECTAL_CANCER_RADIOTHERAPY_RESPONSIVE_UP 103 0.434227 1.45337 0.0625 0.521255 0 up vs 1HADDAD_T_LYMPHOCYTE_AND_NK_PROGENITOR_UP http://www.broadinstitute.org/gsea/msigdb/cards/HADDAD_T_LYMPHOCYTE_AND_NK_PROGENITOR_UP 77 0.421317 1.46221 0.0405405 0.521462 0 up vs 1SASSON_RESPONSE_TO_FORSKOLIN_DN http://www.broadinstitute.org/gsea/msigdb/cards/SASSON_RESPONSE_TO_FORSKOLIN_DN 88 -0.261346 -1.13917 0.222222 0.522031 0 down vs 1MOREIRA_RESPONSE_TO_TSA_DN http://www.broadinstitute.org/gsea/msigdb/cards/MOREIRA_RESPONSE_TO_TSA_DN 18 -0.414127 -1.13831 0.352941 0.522258 0 down vs 1GAZDA_DIAMOND_BLACKFAN_ANEMIA_MYELOID_UP http://www.broadinstitute.org/gsea/msigdb/cards/GAZDA_DIAMOND_BLACKFAN_ANEMIA_MYELOID_UP 29 -0.320375 -1.13785 0.346154 0.522302 0 down vs 1DANG_REGULATED_BY_MYC_UP http://www.broadinstitute.org/gsea/msigdb/cards/DANG_REGULATED_BY_MYC_UP 71 0.437701 1.44484 0.037037 0.522322 0 up vs 1ZHAN_MULTIPLE_MYELOMA_CD1_VS_CD2_DN http://www.broadinstitute.org/gsea/msigdb/cards/ZHAN_MULTIPLE_MYELOMA_CD1_VS_CD2_DN 48 0.430772 1.4607 0.0735294 0.522435 0 up vs 1LEE_METASTASIS_AND_RNA_PROCESSING_UP http://www.broadinstitute.org/gsea/msigdb/cards/LEE_METASTASIS_AND_RNA_PROCESSING_UP 17 0.586578 1.46252 0.105263 0.523545 0 up vs 1DACOSTA_UV_RESPONSE_VIA_ERCC3_XPCS_UP http://www.broadinstitute.org/gsea/msigdb/cards/DACOSTA_UV_RESPONSE_VIA_ERCC3_XPCS_UP 28 0.446732 1.44543 0.0853659 0.523556 0 up vs 1SCHAEFFER_PROSTATE_DEVELOPMENT_AND_CANCER_BOX1_UP http://www.broadinstitute.org/gsea/msigdb/cards/SCHAEFFER_PROSTATE_DEVELOPMENT_AND_CANCER_BOX1_UP 15 0.535921 1.44305 0.138889 0.525119 0 up vs 1TERAO_AOX4_TARGETS_SKIN_DN http://www.broadinstitute.org/gsea/msigdb/cards/TERAO_AOX4_TARGETS_SKIN_DN 27 0.474753 1.46284 0.0983607 0.52539 0 up vs 1MULLIGHAN_NPM1_MUTATED_SIGNATURE_1_DN http://www.broadinstitute.org/gsea/msigdb/cards/MULLIGHAN_NPM1_MUTATED_SIGNATURE_1_DN 123 0.365307 1.44568 0.0568182 0.525477 0 up vs 1NAKAMURA_METASTASIS http://www.broadinstitute.org/gsea/msigdb/cards/NAKAMURA_METASTASIS 45 -0.281233 -1.13537 0.217391 0.5265 0 down vs 1TERAO_AOX4_TARGETS_HG_UP http://www.broadinstitute.org/gsea/msigdb/cards/TERAO_AOX4_TARGETS_HG_UP 28 0.47254 1.44594 0.106061 0.527177 0 up vs 1LI_WILMS_TUMOR_VS_FETAL_KIDNEY_2_UP http://www.broadinstitute.org/gsea/msigdb/cards/LI_WILMS_TUMOR_VS_FETAL_KIDNEY_2_UP 30 0.435 1.4414 0.0810811 0.52719 0 up vs 1GAUSSMANN_MLL_AF4_FUSION_TARGETS_D_UP http://www.broadinstitute.org/gsea/msigdb/cards/GAUSSMANN_MLL_AF4_FUSION_TARGETS_D_UP 36 0.415728 1.44794 0.0147059 0.527212 0 up vs 1SHETH_LIVER_CANCER_VS_TXNIP_LOSS_PAM6 http://www.broadinstitute.org/gsea/msigdb/cards/SHETH_LIVER_CANCER_VS_TXNIP_LOSS_PAM6 46 -0.268814 -1.13411 0.26087 0.528028 0 down vs 1CHIBA_RESPONSE_TO_TSA_UP http://www.broadinstitute.org/gsea/msigdb/cards/CHIBA_RESPONSE_TO_TSA_UP 52 -0.286898 -1.13339 0.357143 0.528724 0 down vs 1ZWANG_EGF_PERSISTENTLY_UP http://www.broadinstitute.org/gsea/msigdb/cards/ZWANG_EGF_PERSISTENTLY_UP 32 0.430243 1.44823 0.027027 0.528906 0 up vs 1JAZAG_TGFB1_SIGNALING_UP http://www.broadinstitute.org/gsea/msigdb/cards/JAZAG_TGFB1_SIGNALING_UP 108 0.363389 1.4462 0.0561798 0.529254 0 up vs 1ROSS_AML_WITH_PML_RARA_FUSION http://www.broadinstitute.org/gsea/msigdb/cards/ROSS_AML_WITH_PML_RARA_FUSION 74 0.414255 1.43755 0.0416667 0.529335 0 up vs 1WIERENGA_STAT5A_TARGETS_GROUP1 http://www.broadinstitute.org/gsea/msigdb/cards/WIERENGA_STAT5A_TARGETS_GROUP1 131 -0.238181 -1.13221 0.2 0.530051 0 down vs 1LIANG_HEMATOPOIESIS_STEM_CELL_NUMBER_LARGE_VS_TINY_DN http://www.broadinstitute.org/gsea/msigdb/cards/LIANG_HEMATOPOIESIS_STEM_CELL_NUMBER_LARGE_VS_TINY_DN 43 0.405581 1.43806 0.025 0.53007 0 up vs 1OLSSON_E2F3_TARGETS_DN http://www.broadinstitute.org/gsea/msigdb/cards/OLSSON_E2F3_TARGETS_DN 47 -0.336729 -1.12969 0.32 0.531234 0 down vs 1LI_ADIPOGENESIS_BY_ACTIVATED_PPARG http://www.broadinstitute.org/gsea/msigdb/cards/LI_ADIPOGENESIS_BY_ACTIVATED_PPARG 17 -0.324462 -1.13047 0.3 0.531427 0 down vs 1BARIS_THYROID_CANCER_UP http://www.broadinstitute.org/gsea/msigdb/cards/BARIS_THYROID_CANCER_UP 23 0.432677 1.43468 0.0864198 0.531813 0 up vs 1FARMER_BREAST_CANCER_CLUSTER_7 http://www.broadinstitute.org/gsea/msigdb/cards/FARMER_BREAST_CANCER_CLUSTER_7 19 -0.325134 -1.13072 0.266667 0.531923 0 down vs 1NUMATA_CSF3_SIGNALING_VIA_STAT3 http://www.broadinstitute.org/gsea/msigdb/cards/NUMATA_CSF3_SIGNALING_VIA_STAT3 22 -0.319067 -1.12979 0.266667 0.532002 0 down vs 1DEURIG_T_CELL_PROLYMPHOCYTIC_LEUKEMIA_UP http://www.broadinstitute.org/gsea/msigdb/cards/DEURIG_T_CELL_PROLYMPHOCYTIC_LEUKEMIA_UP 360 0.354812 1.43826 0.0449438 0.532121 0 up vs 1YAO_TEMPORAL_RESPONSE_TO_PROGESTERONE_CLUSTER_16 http://www.broadinstitute.org/gsea/msigdb/cards/YAO_TEMPORAL_RESPONSE_TO_PROGESTERONE_CLUSTER_16 76 0.424753 1.43528 0.0649351 0.532818 0 up vs 1SINGH_NFE2L2_TARGETS http://www.broadinstitute.org/gsea/msigdb/cards/SINGH_NFE2L2_TARGETS 15 0.627029 1.43344 0.196078 0.532996 0 up vs 1WU_APOPTOSIS_BY_CDKN1A_VIA_TP53 http://www.broadinstitute.org/gsea/msigdb/cards/WU_APOPTOSIS_BY_CDKN1A_VIA_TP53 54 -0.461985 -1.12739 0.346154 0.533221 0 down vs 1NIKOLSKY_BREAST_CANCER_22Q13_AMPLICON http://www.broadinstitute.org/gsea/msigdb/cards/NIKOLSKY_BREAST_CANCER_22Q13_AMPLICON 17 -0.426286 -1.12752 0.361111 0.534042 0 down vs 1BACOLOD_RESISTANCE_TO_ALKYLATING_AGENTS_DN http://www.broadinstitute.org/gsea/msigdb/cards/BACOLOD_RESISTANCE_TO_ALKYLATING_AGENTS_DN 57 0.414717 1.43833 0.0506329 0.534539 0 up vs 1HAHTOLA_MYCOSIS_FUNGOIDES_UP http://www.broadinstitute.org/gsea/msigdb/cards/HAHTOLA_MYCOSIS_FUNGOIDES_UP 18 -0.343195 -1.12525 0.30303 0.534672 0 down vs 1ELVIDGE_HYPOXIA_BY_DMOG_UP http://www.broadinstitute.org/gsea/msigdb/cards/ELVIDGE_HYPOXIA_BY_DMOG_UP 129 -0.284992 -1.12761 0.269231 0.534757 0 down vs 1SCIAN_CELL_CYCLE_TARGETS_OF_TP53_AND_TP73_DN http://www.broadinstitute.org/gsea/msigdb/cards/SCIAN_CELL_CYCLE_TARGETS_OF_TP53_AND_TP73_DN 22 -0.442255 -1.12549 0.393939 0.535003 0 down vs 1PLASARI_TGFB1_SIGNALING_VIA_NFIC_10HR_UP http://www.broadinstitute.org/gsea/msigdb/cards/PLASARI_TGFB1_SIGNALING_VIA_NFIC_10HR_UP 53 -0.286245 -1.12596 0.37037 0.535309 0 down vs 1GENTILE_UV_HIGH_DOSE_UP http://www.broadinstitute.org/gsea/msigdb/cards/GENTILE_UV_HIGH_DOSE_UP 25 -0.317784 -1.1229 0.28 0.538341 0 down vs 1LE_NEURONAL_DIFFERENTIATION_DN http://www.broadinstitute.org/gsea/msigdb/cards/LE_NEURONAL_DIFFERENTIATION_DN 19 -0.399819 -1.12169 0.34375 0.538747 0 down vs 1AMIT_EGF_RESPONSE_240_HELA http://www.broadinstitute.org/gsea/msigdb/cards/AMIT_EGF_RESPONSE_240_HELA 60 -0.271657 -1.12171 0.222222 0.539543 0 down vs 1SHAFFER_IRF4_TARGETS_IN_ACTIVATED_B_LYMPHOCYTE http://www.broadinstitute.org/gsea/msigdb/cards/SHAFFER_IRF4_TARGETS_IN_ACTIVATED_B_LYMPHOCYTE 78 0.41249 1.4219 0.0864198 0.540395 0 up vs 1ONDER_CDH1_TARGETS_3_UP http://www.broadinstitute.org/gsea/msigdb/cards/ONDER_CDH1_TARGETS_3_UP 17 0.502868 1.42907 0.0793651 0.540844 0 up vs 1SMIRNOV_CIRCULATING_ENDOTHELIOCYTES_IN_CANCER_UP http://www.broadinstitute.org/gsea/msigdb/cards/SMIRNOV_CIRCULATING_ENDOTHELIOCYTES_IN_CANCER_UP 155 -0.278812 -1.12002 0.304348 0.541503 0 down vs 1GARGALOVIC_RESPONSE_TO_OXIDIZED_PHOSPHOLIPIDS_YELLOW_DN http://www.broadinstitute.org/gsea/msigdb/cards/GARGALOVIC_RESPONSE_TO_OXIDIZED_PHOSPHOLIPIDS_YELLOW_DN 19 0.462731 1.42209 0.0555556 0.54214 0 up vs 1ACOSTA_PROLIFERATION_INDEPENDENT_MYC_TARGETS_UP http://www.broadinstitute.org/gsea/msigdb/cards/ACOSTA_PROLIFERATION_INDEPENDENT_MYC_TARGETS_UP 79 0.419841 1.42453 0.0731707 0.542297 0 up vs 1HASLINGER_B_CLL_WITH_11Q23_DELETION http://www.broadinstitute.org/gsea/msigdb/cards/HASLINGER_B_CLL_WITH_11Q23_DELETION 23 0.486364 1.42933 0.126761 0.542588 0 up vs 1CHENG_IMPRINTED_BY_ESTRADIOL http://www.broadinstitute.org/gsea/msigdb/cards/CHENG_IMPRINTED_BY_ESTRADIOL 106 0.374762 1.41559 0.0864198 0.543348 0 up vs 1KANNAN_TP53_TARGETS_DN http://www.broadinstitute.org/gsea/msigdb/cards/KANNAN_TP53_TARGETS_DN 21 0.459631 1.41634 0.0285714 0.543798 0 up vs 1GALE_APL_WITH_FLT3_MUTATED_DN http://www.broadinstitute.org/gsea/msigdb/cards/GALE_APL_WITH_FLT3_MUTATED_DN 17 -0.395052 -1.11804 0.266667 0.543799 0 down vs 1KIM_MYC_AMPLIFICATION_TARGETS_UP http://www.broadinstitute.org/gsea/msigdb/cards/KIM_MYC_AMPLIFICATION_TARGETS_UP 196 0.386165 1.417 0.113636 0.543937 0 up vs 1WATTEL_AUTONOMOUS_THYROID_ADENOMA_UP http://www.broadinstitute.org/gsea/msigdb/cards/WATTEL_AUTONOMOUS_THYROID_ADENOMA_UP 72 0.393614 1.42476 0.0422535 0.544301 0 up vs 1BROWN_MYELOID_CELL_DEVELOPMENT_DN http://www.broadinstitute.org/gsea/msigdb/cards/BROWN_MYELOID_CELL_DEVELOPMENT_DN 124 0.396278 1.42292 0.0588235 0.544407 0 up vs 1KAAB_FAILED_HEART_ATRIUM_UP http://www.broadinstitute.org/gsea/msigdb/cards/KAAB_FAILED_HEART_ATRIUM_UP 36 0.407069 1.42552 0.0526316 0.544486 0 up vs 1GROSS_HYPOXIA_VIA_HIF1A_UP http://www.broadinstitute.org/gsea/msigdb/cards/GROSS_HYPOXIA_VIA_HIF1A_UP 76 0.424392 1.42212 0.0941176 0.54455 0 up vs 1LEE_DIFFERENTIATING_T_LYMPHOCYTE http://www.broadinstitute.org/gsea/msigdb/cards/LEE_DIFFERENTIATING_T_LYMPHOCYTE 186 -0.358306 -1.11809 0.382353 0.544653 0 down vs 1MARCINIAK_ER_STRESS_RESPONSE_VIA_CHOP http://www.broadinstitute.org/gsea/msigdb/cards/MARCINIAK_ER_STRESS_RESPONSE_VIA_CHOP 25 0.42406 1.42703 0.0666667 0.544899 0 up vs 1SCHLOSSER_MYC_TARGETS_AND_SERUM_RESPONSE_DN http://www.broadinstitute.org/gsea/msigdb/cards/SCHLOSSER_MYC_TARGETS_AND_SERUM_RESPONSE_DN 46 0.52681 1.41742 0.155844 0.545027 0 up vs 1NGUYEN_NOTCH1_TARGETS_UP http://www.broadinstitute.org/gsea/msigdb/cards/NGUYEN_NOTCH1_TARGETS_UP 29 0.415304 1.41786 0.0821918 0.546231 0 up vs 1HAHTOLA_MYCOSIS_FUNGOIDES_CD4_DN http://www.broadinstitute.org/gsea/msigdb/cards/HAHTOLA_MYCOSIS_FUNGOIDES_CD4_DN 113 0.425807 1.42566 0.0833333 0.546592 0 up vs 1SCHAEFFER_PROSTATE_DEVELOPMENT_6HR_DN http://www.broadinstitute.org/gsea/msigdb/cards/SCHAEFFER_PROSTATE_DEVELOPMENT_6HR_DN 488 0.358658 1.41909 0.0769231 0.547106 0 up vs 1WANG_RESPONSE_TO_ANDROGEN_UP http://www.broadinstitute.org/gsea/msigdb/cards/WANG_RESPONSE_TO_ANDROGEN_UP 29 0.454702 1.41824 0.056338 0.547482 0 up vs 1DACOSTA_LOW_DOSE_UV_RESPONSE_VIA_ERCC3_XPCS_UP http://www.broadinstitute.org/gsea/msigdb/cards/DACOSTA_LOW_DOSE_UV_RESPONSE_VIA_ERCC3_XPCS_UP 17 -0.313263 -1.11425 0.291667 0.54797 0 down vs 1IVANOVSKA_MIR106B_TARGETS http://www.broadinstitute.org/gsea/msigdb/cards/IVANOVSKA_MIR106B_TARGETS 86 -0.296766 -1.11375 0.333333 0.548367 0 down vs 1MATZUK_SPERMATOGONIA http://www.broadinstitute.org/gsea/msigdb/cards/MATZUK_SPERMATOGONIA 24 -0.328358 -1.11447 0.333333 0.548479 0 down vs 1NEMETH_INFLAMMATORY_RESPONSE_LPS_DN http://www.broadinstitute.org/gsea/msigdb/cards/NEMETH_INFLAMMATORY_RESPONSE_LPS_DN 32 -0.304219 -1.11537 0.285714 0.548512 0 down vs 1PLASARI_TGFB1_TARGETS_1HR_UP http://www.broadinstitute.org/gsea/msigdb/cards/PLASARI_TGFB1_TARGETS_1HR_UP 34 -0.364035 -1.11456 0.28125 0.54923 0 down vs 1BOYLAN_MULTIPLE_MYELOMA_PCA1_UP http://www.broadinstitute.org/gsea/msigdb/cards/BOYLAN_MULTIPLE_MYELOMA_PCA1_UP 100 -0.291096 -1.11211 0.314286 0.549659 0 down vs 1SESTO_RESPONSE_TO_UV_C2 http://www.broadinstitute.org/gsea/msigdb/cards/SESTO_RESPONSE_TO_UV_C2 53 -0.318587 -1.11262 0.28 0.549664 0 down vs 1ZHENG_RESPONSE_TO_ARSENITE_DN http://www.broadinstitute.org/gsea/msigdb/cards/ZHENG_RESPONSE_TO_ARSENITE_DN 18 -0.367982 -1.1103 0.461538 0.552324 0 down vs 1LEE_TARGETS_OF_PTCH1_AND_SUFU_DN http://www.broadinstitute.org/gsea/msigdb/cards/LEE_TARGETS_OF_PTCH1_AND_SUFU_DN 83 0.37461 1.40654 0.0724638 0.552527 0 up vs 1VANHARANTA_UTERINE_FIBROID_DN http://www.broadinstitute.org/gsea/msigdb/cards/VANHARANTA_UTERINE_FIBROID_DN 63 -0.280704 -1.10913 0.344828 0.552937 0 down vs 1SENESE_HDAC1_TARGETS_DN http://www.broadinstitute.org/gsea/msigdb/cards/SENESE_HDAC1_TARGETS_DN 244 0.374027 1.4101 0.0740741 0.553222 0 up vs 1GARGALOVIC_RESPONSE_TO_OXIDIZED_PHOSPHOLIPIDS_RED_DN http://www.broadinstitute.org/gsea/msigdb/cards/GARGALOVIC_RESPONSE_TO_OXIDIZED_PHOSPHOLIPIDS_RED_DN 24 0.467522 1.40694 0.126761 0.553439 0 up vs 1FORTSCHEGGER_PHF8_TARGETS_UP http://www.broadinstitute.org/gsea/msigdb/cards/FORTSCHEGGER_PHF8_TARGETS_UP 266 0.362193 1.40756 0.0813954 0.55358 0 up vs 1GRABARCZYK_BCL11B_TARGETS_UP http://www.broadinstitute.org/gsea/msigdb/cards/GRABARCZYK_BCL11B_TARGETS_UP 75 -0.251608 -1.10923 0.291667 0.553765 0 down vs 1HOLLEMAN_VINCRISTINE_RESISTANCE_ALL_DN http://www.broadinstitute.org/gsea/msigdb/cards/HOLLEMAN_VINCRISTINE_RESISTANCE_ALL_DN 19 0.541487 1.40805 0.171429 0.554163 0 up vs 1MOOTHA_PGC http://www.broadinstitute.org/gsea/msigdb/cards/MOOTHA_PGC 409 0.368854 1.41046 0.0674157 0.554499 0 up vs 1CONCANNON_APOPTOSIS_BY_EPOXOMICIN_DN http://www.broadinstitute.org/gsea/msigdb/cards/CONCANNON_APOPTOSIS_BY_EPOXOMICIN_DN 164 0.371034 1.40847 0.0864198 0.555195 0 up vs 1LOPEZ_MESOTHELIOMA_SURVIVAL_OVERALL_DN http://www.broadinstitute.org/gsea/msigdb/cards/LOPEZ_MESOTHELIOMA_SURVIVAL_OVERALL_DN 15 0.507342 1.39191 0.1 0.556432 0 up vs 1CHEN_HOXA5_TARGETS_9HR_DN http://www.broadinstitute.org/gsea/msigdb/cards/CHEN_HOXA5_TARGETS_9HR_DN 39 0.419515 1.3957 0.115385 0.55658 0 up vs 1KOBAYASHI_EGFR_SIGNALING_6HR_DN http://www.broadinstitute.org/gsea/msigdb/cards/KOBAYASHI_EGFR_SIGNALING_6HR_DN 18 -0.389502 -1.10641 0.333333 0.556661 0 down vs 1MONNIER_POSTRADIATION_TUMOR_ESCAPE_UP http://www.broadinstitute.org/gsea/msigdb/cards/MONNIER_POSTRADIATION_TUMOR_ESCAPE_UP 386 0.389377 1.4105 0.0588235 0.55679 0 up vs 1JOSEPH_RESPONSE_TO_SODIUM_BUTYRATE_DN http://www.broadinstitute.org/gsea/msigdb/cards/JOSEPH_RESPONSE_TO_SODIUM_BUTYRATE_DN 63 0.386217 1.39487 0.038961 0.557007 0 up vs 1CHEBOTAEV_GR_TARGETS_DN http://www.broadinstitute.org/gsea/msigdb/cards/CHEBOTAEV_GR_TARGETS_DN 118 0.389955 1.39241 0.028169 0.55742 0 up vs 1WANG_TARGETS_OF_MLL_CBP_FUSION_DN http://www.broadinstitute.org/gsea/msigdb/cards/WANG_TARGETS_OF_MLL_CBP_FUSION_DN 45 0.427456 1.39613 0.0625 0.557781 0 up vs 1RUTELLA_RESPONSE_TO_CSF2RB_AND_IL4_UP http://www.broadinstitute.org/gsea/msigdb/cards/RUTELLA_RESPONSE_TO_CSF2RB_AND_IL4_UP 328 -0.235331 -1.10451 0.333333 0.557998 0 down vs 1DAIRKEE_TERT_TARGETS_DN http://www.broadinstitute.org/gsea/msigdb/cards/DAIRKEE_TERT_TARGETS_DN 120 0.396438 1.39368 0.0921053 0.558188 0 up vs 1SABATES_COLORECTAL_ADENOMA_UP http://www.broadinstitute.org/gsea/msigdb/cards/SABATES_COLORECTAL_ADENOMA_UP 134 -0.240384 -1.10327 0.304348 0.558247 0 down vs 1HOFMANN_MYELODYSPLASTIC_SYNDROM_RISK_UP http://www.broadinstitute.org/gsea/msigdb/cards/HOFMANN_MYELODYSPLASTIC_SYNDROM_RISK_UP 24 0.428898 1.40399 0.126761 0.558306 0 up vs 1PETRETTO_HEART_MASS_QTL_CIS_UP http://www.broadinstitute.org/gsea/msigdb/cards/PETRETTO_HEART_MASS_QTL_CIS_UP 28 0.426192 1.39287 0.0526316 0.558353 0 up vs 1BHATTACHARYA_EMBRYONIC_STEM_CELL http://www.broadinstitute.org/gsea/msigdb/cards/BHATTACHARYA_EMBRYONIC_STEM_CELL 89 0.433276 1.40301 0.121951 0.55838 0 up vs 1BEIER_GLIOMA_STEM_CELL_DN http://www.broadinstitute.org/gsea/msigdb/cards/BEIER_GLIOMA_STEM_CELL_DN 65 -0.283355 -1.10479 0.391304 0.558384 0 down vs 1DIAZ_CHRONIC_MEYLOGENOUS_LEUKEMIA_DN http://www.broadinstitute.org/gsea/msigdb/cards/DIAZ_CHRONIC_MEYLOGENOUS_LEUKEMIA_DN 116 -0.290471 -1.10379 0.392857 0.558423 0 down vs 1MA_MYELOID_DIFFERENTIATION_DN http://www.broadinstitute.org/gsea/msigdb/cards/MA_MYELOID_DIFFERENTIATION_DN 44 -0.264989 -1.10491 0.296296 0.559033 0 down vs 1SCHAEFFER_PROSTATE_DEVELOPMENT_AND_CANCER_BOX4_DN http://www.broadinstitute.org/gsea/msigdb/cards/SCHAEFFER_PROSTATE_DEVELOPMENT_AND_CANCER_BOX4_DN 32 0.491592 1.39002 0.0694444 0.559121 0 up vs 1LU_AGING_BRAIN_DN http://www.broadinstitute.org/gsea/msigdb/cards/LU_AGING_BRAIN_DN 150 0.376191 1.40196 0.0987654 0.559183 0 up vs 1KYNG_ENVIRONMENTAL_STRESS_RESPONSE_NOT_BY_GAMMA_IN_OLD http://www.broadinstitute.org/gsea/msigdb/cards/KYNG_ENVIRONMENTAL_STRESS_RESPONSE_NOT_BY_GAMMA_IN_OLD 30 -0.281828 -1.10168 0.375 0.559534 0 down vs 1HU_GENOTOXIN_ACTION_DIRECT_VS_INDIRECT_24HR http://www.broadinstitute.org/gsea/msigdb/cards/HU_GENOTOXIN_ACTION_DIRECT_VS_INDIRECT_24HR 51 0.403942 1.38912 0.0813954 0.55959 0 up vs 1XU_GH1_EXOGENOUS_TARGETS_UP http://www.broadinstitute.org/gsea/msigdb/cards/XU_GH1_EXOGENOUS_TARGETS_UP 79 0.372447 1.39626 0.0555556 0.559632 0 up vs 1KIM_WT1_TARGETS_8HR_UP http://www.broadinstitute.org/gsea/msigdb/cards/KIM_WT1_TARGETS_8HR_UP 162 -0.232176 -1.10191 0.277778 0.559972 0 down vs 1MAHADEVAN_IMATINIB_RESISTANCE_DN http://www.broadinstitute.org/gsea/msigdb/cards/MAHADEVAN_IMATINIB_RESISTANCE_DN 20 0.431984 1.40026 0.0461538 0.560079 0 up vs 1HILLION_HMGA1_TARGETS http://www.broadinstitute.org/gsea/msigdb/cards/HILLION_HMGA1_TARGETS 88 0.364871 1.38728 0.0823529 0.560179 0 up vs 1HOLLEMAN_VINCRISTINE_RESISTANCE_B_ALL_UP http://www.broadinstitute.org/gsea/msigdb/cards/HOLLEMAN_VINCRISTINE_RESISTANCE_B_ALL_UP 37 0.427458 1.40095 0.116883 0.56021 0 up vs 1LY_AGING_OLD_DN http://www.broadinstitute.org/gsea/msigdb/cards/LY_AGING_OLD_DN 56 -0.330688 -1.09957 0.428571 0.560557 0 down vs 1RODRIGUES_NTN1_AND_DCC_TARGETS http://www.broadinstitute.org/gsea/msigdb/cards/RODRIGUES_NTN1_AND_DCC_TARGETS 34 -0.284128 -1.09821 0.321429 0.560695 0 down vs 1IKEDA_MIR30_TARGETS_DN http://www.broadinstitute.org/gsea/msigdb/cards/IKEDA_MIR30_TARGETS_DN 27 0.415394 1.38781 0.0985916 0.560889 0 up vs 1ABBUD_LIF_SIGNALING_1_DN http://www.broadinstitute.org/gsea/msigdb/cards/ABBUD_LIF_SIGNALING_1_DN 25 0.433797 1.38456 0.0895522 0.561031 0 up vs 1ZHAN_V1_LATE_DIFFERENTIATION_GENES_DN http://www.broadinstitute.org/gsea/msigdb/cards/ZHAN_V1_LATE_DIFFERENTIATION_GENES_DN 15 -0.354862 -1.09984 0.346154 0.561124 0 down vs 1MARSON_FOXP3_TARGETS_DN http://www.broadinstitute.org/gsea/msigdb/cards/MARSON_FOXP3_TARGETS_DN 53 -0.305717 -1.09761 0.26087 0.56116 0 down vs 1ROME_INSULIN_TARGETS_IN_MUSCLE_UP http://www.broadinstitute.org/gsea/msigdb/cards/ROME_INSULIN_TARGETS_IN_MUSCLE_UP 422 0.375749 1.39925 0.0860215 0.561192 0 up vs 1PEART_HDAC_PROLIFERATION_CLUSTER_DN http://www.broadinstitute.org/gsea/msigdb/cards/PEART_HDAC_PROLIFERATION_CLUSTER_DN 75 -0.314536 -1.09872 0.444444 0.561361 0 down vs 1LU_IL4_SIGNALING http://www.broadinstitute.org/gsea/msigdb/cards/LU_IL4_SIGNALING 92 -0.286977 -1.09826 0.36 0.561491 0 down vs 1ONDER_CDH1_TARGETS_2_UP http://www.broadinstitute.org/gsea/msigdb/cards/ONDER_CDH1_TARGETS_2_UP 246 0.43241 1.38514 0.117647 0.561706 0 up vs 1LEIN_LOCALIZED_TO_DISTAL_AND_PROXIMAL_DENDRITES http://www.broadinstitute.org/gsea/msigdb/cards/LEIN_LOCALIZED_TO_DISTAL_AND_PROXIMAL_DENDRITES 15 0.485325 1.39628 0.1 0.561821 0 up vs 1KIM_GLIS2_TARGETS_UP http://www.broadinstitute.org/gsea/msigdb/cards/KIM_GLIS2_TARGETS_UP 84 -0.355393 -1.09999 0.482759 0.561823 0 down vs 1BOYLAN_MULTIPLE_MYELOMA_C_UP http://www.broadinstitute.org/gsea/msigdb/cards/BOYLAN_MULTIPLE_MYELOMA_C_UP 47 0.395059 1.38569 0.0547945 0.562171 0 up vs 1UDAYAKUMAR_MED1_TARGETS_UP http://www.broadinstitute.org/gsea/msigdb/cards/UDAYAKUMAR_MED1_TARGETS_UP 132 0.389964 1.39671 0.0795455 0.562701 0 up vs 1PETROVA_PROX1_TARGETS_UP http://www.broadinstitute.org/gsea/msigdb/cards/PETROVA_PROX1_TARGETS_UP 27 -0.3722 -1.0944 0.346154 0.563006 0 down vs 1DING_LUNG_CANCER_BY_MUTATION_RATE http://www.broadinstitute.org/gsea/msigdb/cards/DING_LUNG_CANCER_BY_MUTATION_RATE 20 0.44791 1.39786 0.0909091 0.563438 0 up vs 1LEE_LIVER_CANCER_MYC_TGFA_UP http://www.broadinstitute.org/gsea/msigdb/cards/LEE_LIVER_CANCER_MYC_TGFA_UP 61 -0.239911 -1.09523 0.166667 0.56352 0 down vs 1TONKS_TARGETS_OF_RUNX1_RUNX1T1_FUSION_ERYTHROCYTE_UP http://www.broadinstitute.org/gsea/msigdb/cards/TONKS_TARGETS_OF_RUNX1_RUNX1T1_FUSION_ERYTHROCYTE_UP 155 -0.259626 -1.09456 0.44 0.563748 0 down vs 1LABBE_TARGETS_OF_TGFB1_AND_WNT3A_DN http://www.broadinstitute.org/gsea/msigdb/cards/LABBE_TARGETS_OF_TGFB1_AND_WNT3A_DN 107 -0.234359 -1.0953 0.304348 0.564317 0 down vs 1WANG_METASTASIS_OF_BREAST_CANCER_ESR1_UP http://www.broadinstitute.org/gsea/msigdb/cards/WANG_METASTASIS_OF_BREAST_CANCER_ESR1_UP 21 -0.383447 -1.09328 0.366667 0.564457 0 down vs 1MARIADASON_RESPONSE_TO_CURCUMIN_SULINDAC_7 http://www.broadinstitute.org/gsea/msigdb/cards/MARIADASON_RESPONSE_TO_CURCUMIN_SULINDAC_7 17 0.463549 1.39682 0.135135 0.564724 0 up vs 1TERAMOTO_OPN_TARGETS_CLUSTER_7 http://www.broadinstitute.org/gsea/msigdb/cards/TERAMOTO_OPN_TARGETS_CLUSTER_7 19 0.431086 1.38268 0.0540541 0.564876 0 up vs 1MCBRYAN_PUBERTAL_BREAST_4_5WK_UP http://www.broadinstitute.org/gsea/msigdb/cards/MCBRYAN_PUBERTAL_BREAST_4_5WK_UP 269 -0.211971 -1.09175 0.3 0.566489 0 down vs 1KAMIKUBO_MYELOID_MN1_NETWORK http://www.broadinstitute.org/gsea/msigdb/cards/KAMIKUBO_MYELOID_MN1_NETWORK 19 -0.353807 -1.09042 0.411765 0.56821 0 down vs 1ASGHARZADEH_NEUROBLASTOMA_POOR_SURVIVAL_DN http://www.broadinstitute.org/gsea/msigdb/cards/ASGHARZADEH_NEUROBLASTOMA_POOR_SURVIVAL_DN 43 0.418324 1.3702 0.084507 0.571554 0 up vs 1ODONNELL_METASTASIS_UP http://www.broadinstitute.org/gsea/msigdb/cards/ODONNELL_METASTASIS_UP 77 0.37023 1.37604 0.0895522 0.572122 0 up vs 1MATZUK_SPERMATID_DIFFERENTIATION http://www.broadinstitute.org/gsea/msigdb/cards/MATZUK_SPERMATID_DIFFERENTIATION 37 0.413961 1.37059 0.0606061 0.572605 0 up vs 1AMIT_EGF_RESPONSE_40_MCF10A http://www.broadinstitute.org/gsea/msigdb/cards/AMIT_EGF_RESPONSE_40_MCF10A 19 -0.373671 -1.08669 0.375 0.572858 0 down vs 1SHETH_LIVER_CANCER_VS_TXNIP_LOSS_PAM4 http://www.broadinstitute.org/gsea/msigdb/cards/SHETH_LIVER_CANCER_VS_TXNIP_LOSS_PAM4 255 0.34402 1.37488 0.0843373 0.573053 0 up vs 1HOLLEMAN_PREDNISOLONE_RESISTANCE_B_ALL_UP http://www.broadinstitute.org/gsea/msigdb/cards/HOLLEMAN_PREDNISOLONE_RESISTANCE_B_ALL_UP 22 0.499143 1.37259 0.214286 0.57306 0 up vs 1MOOTHA_HUMAN_MITODB_6_2002 http://www.broadinstitute.org/gsea/msigdb/cards/MOOTHA_HUMAN_MITODB_6_2002 425 0.390813 1.37637 0.0864198 0.573415 0 up vs 1ROY_WOUND_BLOOD_VESSEL_DN http://www.broadinstitute.org/gsea/msigdb/cards/ROY_WOUND_BLOOD_VESSEL_DN 21 -0.367671 -1.08684 0.323529 0.573482 0 down vs 1GARCIA_TARGETS_OF_FLI1_AND_DAX1_UP http://www.broadinstitute.org/gsea/msigdb/cards/GARCIA_TARGETS_OF_FLI1_AND_DAX1_UP 55 0.40363 1.37089 0.0555556 0.573867 0 up vs 1ROSS_AML_WITH_MLL_FUSIONS http://www.broadinstitute.org/gsea/msigdb/cards/ROSS_AML_WITH_MLL_FUSIONS 76 -0.27595 -1.08701 0.323529 0.57421 0 down vs 1CHESLER_BRAIN_HIGHEST_GENETIC_VARIANCE http://www.broadinstitute.org/gsea/msigdb/cards/CHESLER_BRAIN_HIGHEST_GENETIC_VARIANCE 35 0.402778 1.37902 0.075 0.574215 0 up vs 1OUELLET_CULTURED_OVARIAN_CANCER_INVASIVE_VS_LMP_UP http://www.broadinstitute.org/gsea/msigdb/cards/OUELLET_CULTURED_OVARIAN_CANCER_INVASIVE_VS_LMP_UP 68 0.393549 1.37368 0.104651 0.57433 0 up vs 1BANDRES_RESPONSE_TO_CARMUSTIN_MGMT_48HR_UP http://www.broadinstitute.org/gsea/msigdb/cards/BANDRES_RESPONSE_TO_CARMUSTIN_MGMT_48HR_UP 18 0.439513 1.37279 0.0810811 0.574543 0 up vs 1WENG_POR_DOSAGE http://www.broadinstitute.org/gsea/msigdb/cards/WENG_POR_DOSAGE 21 0.436117 1.37717 0.0945946 0.57471 0 up vs 1XU_RESPONSE_TO_TRETINOIN_AND_NSC682994_DN http://www.broadinstitute.org/gsea/msigdb/cards/XU_RESPONSE_TO_TRETINOIN_AND_NSC682994_DN 15 0.610354 1.37662 0.236842 0.574717 0 up vs 1MATZUK_MEIOTIC_AND_DNA_REPAIR http://www.broadinstitute.org/gsea/msigdb/cards/MATZUK_MEIOTIC_AND_DNA_REPAIR 39 -0.295407 -1.08524 0.448276 0.575011 0 down vs 1HORTON_SREBF_TARGETS http://www.broadinstitute.org/gsea/msigdb/cards/HORTON_SREBF_TARGETS 24 0.51738 1.37109 0.152778 0.575284 0 up vs 1MOOTHA_MITOCHONDRIA http://www.broadinstitute.org/gsea/msigdb/cards/MOOTHA_MITOCHONDRIA 442 0.391901 1.37765 0.0740741 0.575606 0 up vs 1ZHANG_TLX_TARGETS_36HR_DN http://www.broadinstitute.org/gsea/msigdb/cards/ZHANG_TLX_TARGETS_36HR_DN 184 -0.371876 -1.08286 0.458333 0.575833 0 down vs 1MARKEY_RB1_ACUTE_LOF_DN http://www.broadinstitute.org/gsea/msigdb/cards/MARKEY_RB1_ACUTE_LOF_DN 223 -0.300377 -1.08293 0.35 0.576633 0 down vs 1TIAN_BHLHA15_TARGETS http://www.broadinstitute.org/gsea/msigdb/cards/TIAN_BHLHA15_TARGETS 15 -0.347458 -1.08027 0.382353 0.576693 0 down vs 1ZHAN_V1_LATE_DIFFERENTIATION_GENES_UP http://www.broadinstitute.org/gsea/msigdb/cards/ZHAN_V1_LATE_DIFFERENTIATION_GENES_UP 32 -0.310639 -1.07973 0.333333 0.57672 0 down vs 1WINTER_HYPOXIA_UP http://www.broadinstitute.org/gsea/msigdb/cards/WINTER_HYPOXIA_UP 89 -0.327104 -1.08178 0.333333 0.576887 0 down vs 1SHIN_B_CELL_LYMPHOMA_CLUSTER_2 http://www.broadinstitute.org/gsea/msigdb/cards/SHIN_B_CELL_LYMPHOMA_CLUSTER_2 30 -0.317547 -1.08118 0.375 0.577089 0 down vs 1FERRANDO_TAL1_NEIGHBORS http://www.broadinstitute.org/gsea/msigdb/cards/FERRANDO_TAL1_NEIGHBORS 21 -0.387023 -1.08309 0.382353 0.577259 0 down vs 1ONO_AML1_TARGETS_UP http://www.broadinstitute.org/gsea/msigdb/cards/ONO_AML1_TARGETS_UP 24 -0.415487 -1.08358 0.404762 0.577284 0 down vs 1ZHAN_MULTIPLE_MYELOMA_CD1_DN http://www.broadinstitute.org/gsea/msigdb/cards/ZHAN_MULTIPLE_MYELOMA_CD1_DN 44 -0.27455 -1.0804 0.344828 0.577465 0 down vs 1BARIS_THYROID_CANCER_DN http://www.broadinstitute.org/gsea/msigdb/cards/BARIS_THYROID_CANCER_DN 57 -0.268111 -1.07739 0.4375 0.578509 0 down vs 1LINDGREN_BLADDER_CANCER_CLUSTER_3_UP http://www.broadinstitute.org/gsea/msigdb/cards/LINDGREN_BLADDER_CANCER_CLUSTER_3_UP 318 -0.278211 -1.07764 0.3 0.579055 0 down vs 1HOWLIN_CITED1_TARGETS_2_DN http://www.broadinstitute.org/gsea/msigdb/cards/HOWLIN_CITED1_TARGETS_2_DN 17 -0.295006 -1.06908 0.4 0.579124 0 down vs 1HAHTOLA_SEZARY_SYNDROM_DN http://www.broadinstitute.org/gsea/msigdb/cards/HAHTOLA_SEZARY_SYNDROM_DN 40 -0.3729 -1.07797 0.473684 0.579156 0 down vs 1AZARE_NEOPLASTIC_TRANSFORMATION_BY_STAT3_DN http://www.broadinstitute.org/gsea/msigdb/cards/AZARE_NEOPLASTIC_TRANSFORMATION_BY_STAT3_DN 16 -0.352245 -1.07051 0.354839 0.579347 0 down vs 1ELVIDGE_HYPOXIA_UP http://www.broadinstitute.org/gsea/msigdb/cards/ELVIDGE_HYPOXIA_UP 168 -0.240622 -1.06795 0.391304 0.579505 0 down vs 1WEBER_METHYLATED_HCP_IN_FIBROBLAST_DN http://www.broadinstitute.org/gsea/msigdb/cards/WEBER_METHYLATED_HCP_IN_FIBROBLAST_DN 42 -0.274821 -1.06834 0.5 0.579628 0 down vs 1DACOSTA_UV_RESPONSE_VIA_ERCC3_TTD_UP http://www.broadinstitute.org/gsea/msigdb/cards/DACOSTA_UV_RESPONSE_VIA_ERCC3_TTD_UP 64 0.38909 1.36528 0.0759494 0.579639 0 up vs 1KAUFFMANN_MELANOMA_RELAPSE_UP http://www.broadinstitute.org/gsea/msigdb/cards/KAUFFMANN_MELANOMA_RELAPSE_UP 60 -0.38573 -1.06738 0.454545 0.579816 0 down vs 1SASAKI_ADULT_T_CELL_LEUKEMIA http://www.broadinstitute.org/gsea/msigdb/cards/SASAKI_ADULT_T_CELL_LEUKEMIA 172 -0.276796 -1.07421 0.333333 0.579831 0 down vs 1AZARE_NEOPLASTIC_TRANSFORMATION_BY_STAT3_UP http://www.broadinstitute.org/gsea/msigdb/cards/AZARE_NEOPLASTIC_TRANSFORMATION_BY_STAT3_UP 117 -0.280651 -1.06909 0.44 0.58002 0 down vs 1VALK_AML_CLUSTER_5 http://www.broadinstitute.org/gsea/msigdb/cards/VALK_AML_CLUSTER_5 33 -0.415666 -1.07357 0.352941 0.580098 0 down vs 1HANSON_HRAS_SIGNALING_VIA_NFKB http://www.broadinstitute.org/gsea/msigdb/cards/HANSON_HRAS_SIGNALING_VIA_NFKB 22 -0.304988 -1.07073 0.372093 0.580103 0 down vs 1NIELSEN_MALIGNAT_FIBROUS_HISTIOCYTOMA_UP http://www.broadinstitute.org/gsea/msigdb/cards/NIELSEN_MALIGNAT_FIBROUS_HISTIOCYTOMA_UP 18 -0.357951 -1.06614 0.380952 0.580217 0 down vs 1CROONQUIST_IL6_DEPRIVATION_UP http://www.broadinstitute.org/gsea/msigdb/cards/CROONQUIST_IL6_DEPRIVATION_UP 20 -0.32906 -1.0754 0.34375 0.580457 0 down vs 1TORCHIA_TARGETS_OF_EWSR1_FLI1_FUSION_UP http://www.broadinstitute.org/gsea/msigdb/cards/TORCHIA_TARGETS_OF_EWSR1_FLI1_FUSION_UP 261 -0.2077 -1.07443 0.416667 0.580498 0 down vs 1CAFFAREL_RESPONSE_TO_THC_DN http://www.broadinstitute.org/gsea/msigdb/cards/CAFFAREL_RESPONSE_TO_THC_DN 30 -0.312488 -1.07581 0.44 0.58056 0 down vs 1FIGUEROA_AML_METHYLATION_CLUSTER_1_UP http://www.broadinstitute.org/gsea/msigdb/cards/FIGUEROA_AML_METHYLATION_CLUSTER_1_UP 117 0.350781 1.36649 0.0909091 0.580608 0 up vs 1MARTORIATI_MDM4_TARGETS_NEUROEPITHELIUM_DN http://www.broadinstitute.org/gsea/msigdb/cards/MARTORIATI_MDM4_TARGETS_NEUROEPITHELIUM_DN 159 0.364178 1.36576 0.121622 0.580708 0 up vs 1ROESSLER_LIVER_CANCER_METASTASIS_UP http://www.broadinstitute.org/gsea/msigdb/cards/ROESSLER_LIVER_CANCER_METASTASIS_UP 105 -0.226197 -1.07494 0.428571 0.580724 0 down vs 1HUPER_BREAST_BASAL_VS_LUMINAL_DN http://www.broadinstitute.org/gsea/msigdb/cards/HUPER_BREAST_BASAL_VS_LUMINAL_DN 59 -0.315498 -1.0664 0.37037 0.580725 0 down vs 1BASSO_HAIRY_CELL_LEUKEMIA_DN http://www.broadinstitute.org/gsea/msigdb/cards/BASSO_HAIRY_CELL_LEUKEMIA_DN 79 -0.254714 -1.06921 0.407407 0.58075 0 down vs 1LI_CYTIDINE_ANALOG_PATHWAY http://www.broadinstitute.org/gsea/msigdb/cards/LI_CYTIDINE_ANALOG_PATHWAY 16 -0.335635 -1.07169 0.392857 0.580796 0 down vs 1PODAR_RESPONSE_TO_ADAPHOSTIN_DN http://www.broadinstitute.org/gsea/msigdb/cards/PODAR_RESPONSE_TO_ADAPHOSTIN_DN 18 -0.353799 -1.07078 0.4375 0.58086 0 down vs 1DITTMER_PTHLH_TARGETS_DN http://www.broadinstitute.org/gsea/msigdb/cards/DITTMER_PTHLH_TARGETS_DN 73 -0.239458 -1.07226 0.357143 0.580898 0 down vs 1BAKER_HEMATOPOIESIS_STAT3_TARGETS http://www.broadinstitute.org/gsea/msigdb/cards/BAKER_HEMATOPOIESIS_STAT3_TARGETS 16 -0.35052 -1.06484 0.4 0.58141 0 down vs 1LAMB_CCND1_TARGETS http://www.broadinstitute.org/gsea/msigdb/cards/LAMB_CCND1_TARGETS 19 -0.324663 -1.07092 0.333333 0.581474 0 down vs 1LEE_AGING_CEREBELLUM_UP http://www.broadinstitute.org/gsea/msigdb/cards/LEE_AGING_CEREBELLUM_UP 83 -0.26691 -1.07239 0.392857 0.581588 0 down vs 1WHITFIELD_CELL_CYCLE_LITERATURE http://www.broadinstitute.org/gsea/msigdb/cards/WHITFIELD_CELL_CYCLE_LITERATURE 44 -0.423015 -1.06345 0.551724 0.582061 0 down vs 1VANHARANTA_UTERINE_FIBROID_UP http://www.broadinstitute.org/gsea/msigdb/cards/VANHARANTA_UTERINE_FIBROID_UP 44 0.429042 1.36398 0.114286 0.582069 0 up vs 1YANG_BREAST_CANCER_ESR1_UP http://www.broadinstitute.org/gsea/msigdb/cards/YANG_BREAST_CANCER_ESR1_UP 36 0.41597 1.3632 0.119403 0.582134 0 up vs 1ZHENG_FOXP3_TARGETS_IN_T_LYMPHOCYTE_DN http://www.broadinstitute.org/gsea/msigdb/cards/ZHENG_FOXP3_TARGETS_IN_T_LYMPHOCYTE_DN 35 -0.313481 -1.06376 0.368421 0.582306 0 down vs 1DAVIES_MULTIPLE_MYELOMA_VS_MGUS_DN http://www.broadinstitute.org/gsea/msigdb/cards/DAVIES_MULTIPLE_MYELOMA_VS_MGUS_DN 28 -0.351116 -1.0638 0.4 0.583077 0 down vs 1CAIRO_HEPATOBLASTOMA_POOR_SURVIVAL http://www.broadinstitute.org/gsea/msigdb/cards/CAIRO_HEPATOBLASTOMA_POOR_SURVIVAL 16 0.459751 1.36159 0.15493 0.584539 0 up vs 1SENESE_HDAC2_TARGETS_DN http://www.broadinstitute.org/gsea/msigdb/cards/SENESE_HDAC2_TARGETS_DN 126 0.354311 1.36 0.0779221 0.584875 0 up vs 1LEE_AGING_MUSCLE_UP http://www.broadinstitute.org/gsea/msigdb/cards/LEE_AGING_MUSCLE_UP 45 0.379061 1.36052 0.0512821 0.585354 0 up vs 1BURTON_ADIPOGENESIS_4 http://www.broadinstitute.org/gsea/msigdb/cards/BURTON_ADIPOGENESIS_4 46 0.419933 1.35764 0.1375 0.586063 0 up vs 1WAKABAYASHI_ADIPOGENESIS_PPARG_BOUND_36HR http://www.broadinstitute.org/gsea/msigdb/cards/WAKABAYASHI_ADIPOGENESIS_PPARG_BOUND_36HR 29 0.422741 1.35812 0.0810811 0.586708 0 up vs 1KAYO_AGING_MUSCLE_DN http://www.broadinstitute.org/gsea/msigdb/cards/KAYO_AGING_MUSCLE_DN 123 0.354327 1.35862 0.0689655 0.586994 0 up vs 1RAY_TUMORIGENESIS_BY_ERBB2_CDC25A_DN http://www.broadinstitute.org/gsea/msigdb/cards/RAY_TUMORIGENESIS_BY_ERBB2_CDC25A_DN 155 -0.216919 -1.05637 0.473684 0.587838 0 down vs 1TANAKA_METHYLATED_IN_ESOPHAGEAL_CARCINOMA http://www.broadinstitute.org/gsea/msigdb/cards/TANAKA_METHYLATED_IN_ESOPHAGEAL_CARCINOMA 102 0.344836 1.35589 0.109756 0.588117 0 up vs 1LEE_LIVER_CANCER_CIPROFIBRATE_DN http://www.broadinstitute.org/gsea/msigdb/cards/LEE_LIVER_CANCER_CIPROFIBRATE_DN 66 -0.231793 -1.05776 0.413793 0.588143 0 down vs 1KAUFFMANN_DNA_REPAIR_GENES http://www.broadinstitute.org/gsea/msigdb/cards/KAUFFMANN_DNA_REPAIR_GENES 230 -0.277766 -1.05655 0.5 0.588443 0 down vs 1REICHERT_MITOSIS_LIN9_TARGETS http://www.broadinstitute.org/gsea/msigdb/cards/REICHERT_MITOSIS_LIN9_TARGETS 28 -0.444137 -1.05704 0.545455 0.588695 0 down vs 1ODONNELL_METASTASIS_DN http://www.broadinstitute.org/gsea/msigdb/cards/ODONNELL_METASTASIS_DN 24 -0.283631 -1.05782 0.37037 0.588893 0 down vs 1HESS_TARGETS_OF_HOXA9_AND_MEIS1_DN http://www.broadinstitute.org/gsea/msigdb/cards/HESS_TARGETS_OF_HOXA9_AND_MEIS1_DN 76 -0.318036 -1.05892 0.363636 0.588926 0 down vs 1XU_HGF_SIGNALING_NOT_VIA_AKT1_48HR_UP http://www.broadinstitute.org/gsea/msigdb/cards/XU_HGF_SIGNALING_NOT_VIA_AKT1_48HR_UP 35 -0.282563 -1.05839 0.333333 0.588957 0 down vs 1SAGIV_CD24_TARGETS_UP http://www.broadinstitute.org/gsea/msigdb/cards/SAGIV_CD24_TARGETS_UP 22 -0.325924 -1.0531 0.352941 0.589541 0 down vs 1MULLIGHAN_NPM1_SIGNATURE_3_DN http://www.broadinstitute.org/gsea/msigdb/cards/MULLIGHAN_NPM1_SIGNATURE_3_DN 159 0.34722 1.356 0.0697674 0.589897 0 up vs 1DANG_REGULATED_BY_MYC_DN http://www.broadinstitute.org/gsea/msigdb/cards/DANG_REGULATED_BY_MYC_DN 249 -0.213033 -1.05314 0.391304 0.590331 0 down vs 1KORKOLA_YOLK_SAC_TUMOR http://www.broadinstitute.org/gsea/msigdb/cards/KORKOLA_YOLK_SAC_TUMOR 60 0.364424 1.35439 0.0714286 0.590437 0 up vs 1HOSHIDA_LIVER_CANCER_LATE_RECURRENCE_UP http://www.broadinstitute.org/gsea/msigdb/cards/HOSHIDA_LIVER_CANCER_LATE_RECURRENCE_UP 60 -0.26537 -1.05131 0.357143 0.590673 0 down vs 1OHM_METHYLATED_IN_ADULT_CANCERS http://www.broadinstitute.org/gsea/msigdb/cards/OHM_METHYLATED_IN_ADULT_CANCERS 27 -0.293425 -1.05438 0.3125 0.590881 0 down vs 1ONDER_CDH1_TARGETS_1_UP http://www.broadinstitute.org/gsea/msigdb/cards/ONDER_CDH1_TARGETS_1_UP 137 -0.224496 -1.05372 0.416667 0.591026 0 down vs 1TANG_SENESCENCE_TP53_TARGETS_DN http://www.broadinstitute.org/gsea/msigdb/cards/TANG_SENESCENCE_TP53_TARGETS_DN 57 -0.398218 -1.05317 0.5 0.591194 0 down vs 1ZHU_CMV_24_HR_UP http://www.broadinstitute.org/gsea/msigdb/cards/ZHU_CMV_24_HR_UP 93 -0.261347 -1.05132 0.294118 0.591532 0 down vs 1PENG_LEUCINE_DEPRIVATION_DN http://www.broadinstitute.org/gsea/msigdb/cards/PENG_LEUCINE_DEPRIVATION_DN 184 0.410049 1.35331 0.0941176 0.592085 0 up vs 1DALESSIO_TSA_RESPONSE http://www.broadinstitute.org/gsea/msigdb/cards/DALESSIO_TSA_RESPONSE 26 -0.303549 -1.04828 0.433333 0.592359 0 down vs 1AMBROSINI_FLAVOPIRIDOL_TREATMENT_TP53 http://www.broadinstitute.org/gsea/msigdb/cards/AMBROSINI_FLAVOPIRIDOL_TREATMENT_TP53 106 -0.222945 -1.04873 0.413793 0.592473 0 down vs 1MCBRYAN_PUBERTAL_BREAST_3_4WK_UP http://www.broadinstitute.org/gsea/msigdb/cards/MCBRYAN_PUBERTAL_BREAST_3_4WK_UP 212 -0.200974 -1.04902 0.3 0.592936 0 down vs 1LIANG_HEMATOPOIESIS_STEM_CELL_NUMBER_SMALL_VS_HUGE_DN http://www.broadinstitute.org/gsea/msigdb/cards/LIANG_HEMATOPOIESIS_STEM_CELL_NUMBER_SMALL_VS_HUGE_DN 32 0.389102 1.35237 0.0886076 0.593394 0 up vs 1GENTILE_UV_LOW_DOSE_UP http://www.broadinstitute.org/gsea/msigdb/cards/GENTILE_UV_LOW_DOSE_UP 27 -0.31598 -1.04911 0.5 0.593634 0 down vs 1BOYAULT_LIVER_CANCER_SUBCLASS_G23_UP http://www.broadinstitute.org/gsea/msigdb/cards/BOYAULT_LIVER_CANCER_SUBCLASS_G23_UP 50 -0.331413 -1.0461 0.428571 0.595089 0 down vs 1DOANE_RESPONSE_TO_ANDROGEN_DN http://www.broadinstitute.org/gsea/msigdb/cards/DOANE_RESPONSE_TO_ANDROGEN_DN 237 -0.220499 -1.04657 0.333333 0.595138 0 down vs 1ROYLANCE_BREAST_CANCER_16Q_COPY_NUMBER_DN http://www.broadinstitute.org/gsea/msigdb/cards/ROYLANCE_BREAST_CANCER_16Q_COPY_NUMBER_DN 26 0.418363 1.35084 0.140845 0.596026 0 up vs 1KOKKINAKIS_METHIONINE_DEPRIVATION_96HR_UP http://www.broadinstitute.org/gsea/msigdb/cards/KOKKINAKIS_METHIONINE_DEPRIVATION_96HR_UP 116 -0.236841 -1.04191 0.411765 0.601144 0 down vs 1SATO_SILENCED_EPIGENETICALLY_IN_PANCREATIC_CANCER http://www.broadinstitute.org/gsea/msigdb/cards/SATO_SILENCED_EPIGENETICALLY_IN_PANCREATIC_CANCER 49 -0.24808 -1.04218 0.388889 0.601363 0 down vs 1MARSON_FOXP3_TARGETS_STIMULATED_UP http://www.broadinstitute.org/gsea/msigdb/cards/MARSON_FOXP3_TARGETS_STIMULATED_UP 29 -0.368105 -1.03884 0.424242 0.604591 0 down vs 1TSAI_RESPONSE_TO_RADIATION_THERAPY http://www.broadinstitute.org/gsea/msigdb/cards/TSAI_RESPONSE_TO_RADIATION_THERAPY 32 -0.346085 -1.03618 0.40625 0.605058 0 down vs 1PUJANA_XPRSS_INT_NETWORK http://www.broadinstitute.org/gsea/msigdb/cards/PUJANA_XPRSS_INT_NETWORK 167 -0.375324 -1.03754 0.47619 0.605406 0 down vs 1COLINA_TARGETS_OF_4EBP1_AND_4EBP2 http://www.broadinstitute.org/gsea/msigdb/cards/COLINA_TARGETS_OF_4EBP1_AND_4EBP2 353 -0.205749 -1.03888 0.4 0.605436 0 down vs 1BENPORATH_ES_CORE_NINE_CORRELATED http://www.broadinstitute.org/gsea/msigdb/cards/BENPORATH_ES_CORE_NINE_CORRELATED 99 -0.257933 -1.0362 0.421053 0.60592 0 down vs 1SENGUPTA_NASOPHARYNGEAL_CARCINOMA_UP http://www.broadinstitute.org/gsea/msigdb/cards/SENGUPTA_NASOPHARYNGEAL_CARCINOMA_UP 289 -0.293586 -1.03484 0.391304 0.606185 0 down vs 1PETROVA_ENDOTHELIUM_LYMPHATIC_VS_BLOOD_DN http://www.broadinstitute.org/gsea/msigdb/cards/PETROVA_ENDOTHELIUM_LYMPHATIC_VS_BLOOD_DN 160 -0.284038 -1.03764 0.409091 0.606204 0 down vs 1NAKAYAMA_FRA2_TARGETS http://www.broadinstitute.org/gsea/msigdb/cards/NAKAYAMA_FRA2_TARGETS 41 -0.286905 -1.03642 0.454545 0.606487 0 down vs 1HELLEBREKERS_SILENCED_DURING_TUMOR_ANGIOGENESIS http://www.broadinstitute.org/gsea/msigdb/cards/HELLEBREKERS_SILENCED_DURING_TUMOR_ANGIOGENESIS 80 -0.279165 -1.03515 0.391304 0.60659 0 down vs 1FONTAINE_FOLLICULAR_THYROID_ADENOMA_DN http://www.broadinstitute.org/gsea/msigdb/cards/FONTAINE_FOLLICULAR_THYROID_ADENOMA_DN 67 -0.243148 -1.0337 0.26087 0.607143 0 down vs 1SCHLOSSER_MYC_TARGETS_REPRESSED_BY_SERUM http://www.broadinstitute.org/gsea/msigdb/cards/SCHLOSSER_MYC_TARGETS_REPRESSED_BY_SERUM 156 0.48191 1.3453 0.164557 0.608465 0 up vs 1LOPEZ_TRANSLATION_VIA_FN1_SIGNALING http://www.broadinstitute.org/gsea/msigdb/cards/LOPEZ_TRANSLATION_VIA_FN1_SIGNALING 35 0.410884 1.34574 0.0963855 0.608708 0 up vs 1NOUZOVA_METHYLATED_IN_APL http://www.broadinstitute.org/gsea/msigdb/cards/NOUZOVA_METHYLATED_IN_APL 63 0.365464 1.34142 0.0779221 0.610649 0 up vs 1QI_PLASMACYTOMA_DN http://www.broadinstitute.org/gsea/msigdb/cards/QI_PLASMACYTOMA_DN 99 0.365274 1.34161 0.075 0.612225 0 up vs 1VALK_AML_WITH_EVI1 http://www.broadinstitute.org/gsea/msigdb/cards/VALK_AML_WITH_EVI1 23 0.417232 1.31502 0.106061 0.612714 0 up vs 1RAMALHO_STEMNESS_UP http://www.broadinstitute.org/gsea/msigdb/cards/RAMALHO_STEMNESS_UP 201 0.411982 1.34316 0.144578 0.613145 0 up vs 1CHENG_RESPONSE_TO_NICKEL_ACETATE http://www.broadinstitute.org/gsea/msigdb/cards/CHENG_RESPONSE_TO_NICKEL_ACETATE 43 0.40491 1.3141 0.0864198 0.613217 0 up vs 1SHEPARD_BMYB_MORPHOLINO_UP http://www.broadinstitute.org/gsea/msigdb/cards/SHEPARD_BMYB_MORPHOLINO_UP 202 0.32937 1.34169 0.106383 0.613892 0 up vs 1DURCHDEWALD_SKIN_CARCINOGENESIS_DN http://www.broadinstitute.org/gsea/msigdb/cards/DURCHDEWALD_SKIN_CARCINOGENESIS_DN 262 -0.235868 -1.02841 0.375 0.614108 0 down vs 1TIEN_INTESTINE_PROBIOTICS_2HR_DN http://www.broadinstitute.org/gsea/msigdb/cards/TIEN_INTESTINE_PROBIOTICS_2HR_DN 87 0.383751 1.31508 0.155844 0.614227 0 up vs 1CHANG_IMMORTALIZED_BY_HPV31_UP http://www.broadinstitute.org/gsea/msigdb/cards/CHANG_IMMORTALIZED_BY_HPV31_UP 83 0.367051 1.31852 0.105263 0.614297 0 up vs 1GOLUB_ALL_VS_AML_UP http://www.broadinstitute.org/gsea/msigdb/cards/GOLUB_ALL_VS_AML_UP 24 0.466561 1.34222 0.186667 0.614497 0 up vs 1TONKS_TARGETS_OF_RUNX1_RUNX1T1_FUSION_SUSTAINED_IN_MONOCYTE_UP http://www.broadinstitute.org/gsea/msigdb/cards/TONKS_TARGETS_OF_RUNX1_RUNX1T1_FUSION_SUSTAINED_IN_MONOCYTE_UP 21 0.395686 1.33941 0.057971 0.614848 0 up vs 1PLASARI_TGFB1_SIGNALING_VIA_NFIC_10HR_DN http://www.broadinstitute.org/gsea/msigdb/cards/PLASARI_TGFB1_SIGNALING_VIA_NFIC_10HR_DN 30 -0.286463 -1.02848 0.393939 0.614929 0 down vs 1YANG_MUC2_TARGETS_DUODENUM_3MO_DN http://www.broadinstitute.org/gsea/msigdb/cards/YANG_MUC2_TARGETS_DUODENUM_3MO_DN 23 0.422894 1.31153 0.126984 0.614938 0 up vs 1BLALOCK_ALZHEIMERS_DISEASE_INCIPIENT_DN http://www.broadinstitute.org/gsea/msigdb/cards/BLALOCK_ALZHEIMERS_DISEASE_INCIPIENT_DN 162 0.348033 1.31596 0.101124 0.614966 0 up vs 1OSMAN_BLADDER_CANCER_DN http://www.broadinstitute.org/gsea/msigdb/cards/OSMAN_BLADDER_CANCER_DN 374 0.329983 1.33804 0.129032 0.614967 0 up vs 1MILI_PSEUDOPODIA_CHEMOTAXIS_UP http://www.broadinstitute.org/gsea/msigdb/cards/MILI_PSEUDOPODIA_CHEMOTAXIS_UP 69 0.390257 1.31289 0.142857 0.614973 0 up vs 1LOPES_METHYLATED_IN_COLON_CANCER_UP http://www.broadinstitute.org/gsea/msigdb/cards/LOPES_METHYLATED_IN_COLON_CANCER_UP 27 0.394713 1.31216 0.0535714 0.615094 0 up vs 1SUH_COEXPRESSED_WITH_ID1_AND_ID2_UP http://www.broadinstitute.org/gsea/msigdb/cards/SUH_COEXPRESSED_WITH_ID1_AND_ID2_UP 19 -0.341886 -1.02922 0.368421 0.615172 0 down vs 1BERENJENO_ROCK_SIGNALING_NOT_VIA_RHOA_UP http://www.broadinstitute.org/gsea/msigdb/cards/BERENJENO_ROCK_SIGNALING_NOT_VIA_RHOA_UP 28 -0.279427 -1.02869 0.458333 0.615322 0 down vs 1MCCLUNG_DELTA_FOSB_TARGETS_8WK http://www.broadinstitute.org/gsea/msigdb/cards/MCCLUNG_DELTA_FOSB_TARGETS_8WK 47 0.385556 1.31875 0.0972222 0.615438 0 up vs 1ALONSO_METASTASIS_NEURAL_UP http://www.broadinstitute.org/gsea/msigdb/cards/ALONSO_METASTASIS_NEURAL_UP 18 0.442441 1.33526 0.148649 0.61559 0 up vs 1VANOEVELEN_MYOGENESIS_SIN3A_TARGETS http://www.broadinstitute.org/gsea/msigdb/cards/VANOEVELEN_MYOGENESIS_SIN3A_TARGETS 215 0.345722 1.31982 0.101124 0.615718 0 up vs 1KAAB_FAILED_HEART_ATRIUM_DN http://www.broadinstitute.org/gsea/msigdb/cards/KAAB_FAILED_HEART_ATRIUM_DN 140 0.360243 1.31623 0.122222 0.615812 0 up vs 1CHIANG_LIVER_CANCER_SUBCLASS_POLYSOMY7_UP http://www.broadinstitute.org/gsea/msigdb/cards/CHIANG_LIVER_CANCER_SUBCLASS_POLYSOMY7_UP 78 0.35019 1.32922 0.0972222 0.615883 0 up vs 1ACEVEDO_NORMAL_TISSUE_ADJACENT_TO_LIVER_TUMOR_UP http://www.broadinstitute.org/gsea/msigdb/cards/ACEVEDO_NORMAL_TISSUE_ADJACENT_TO_LIVER_TUMOR_UP 162 0.338082 1.3151 0.186047 0.615937 0 up vs 1YAO_TEMPORAL_RESPONSE_TO_PROGESTERONE_CLUSTER_12 http://www.broadinstitute.org/gsea/msigdb/cards/YAO_TEMPORAL_RESPONSE_TO_PROGESTERONE_CLUSTER_12 78 0.372479 1.33706 0.107143 0.615995 0 up vs 1CHANG_POU5F1_TARGETS_UP http://www.broadinstitute.org/gsea/msigdb/cards/CHANG_POU5F1_TARGETS_UP 15 -0.39189 -1.02367 0.363636 0.616194 0 down vs 1BOYLAN_MULTIPLE_MYELOMA_C_DN http://www.broadinstitute.org/gsea/msigdb/cards/BOYLAN_MULTIPLE_MYELOMA_C_DN 56 0.381823 1.33826 0.0657895 0.616226 0 up vs 1GNATENKO_PLATELET_SIGNATURE http://www.broadinstitute.org/gsea/msigdb/cards/GNATENKO_PLATELET_SIGNATURE 46 -0.249258 -1.02681 0.35 0.616287 0 down vs 1CHANDRAN_METASTASIS_TOP50_DN http://www.broadinstitute.org/gsea/msigdb/cards/CHANDRAN_METASTASIS_TOP50_DN 45 0.374462 1.32083 0.115385 0.616292 0 up vs 1MIKKELSEN_IPS_HCP_WITH_H3_UNMETHYLATED http://www.broadinstitute.org/gsea/msigdb/cards/MIKKELSEN_IPS_HCP_WITH_H3_UNMETHYLATED 79 0.346067 1.32968 0.115385 0.61636 0 up vs 1NIELSEN_LIPOSARCOMA_UP http://www.broadinstitute.org/gsea/msigdb/cards/NIELSEN_LIPOSARCOMA_UP 18 0.423173 1.32025 0.106061 0.616391 0 up vs 1FONTAINE_FOLLICULAR_THYROID_ADENOMA_UP http://www.broadinstitute.org/gsea/msigdb/cards/FONTAINE_FOLLICULAR_THYROID_ADENOMA_UP 73 0.355495 1.3105 0.0675676 0.616489 0 up vs 1BOYAULT_LIVER_CANCER_SUBCLASS_G2 http://www.broadinstitute.org/gsea/msigdb/cards/BOYAULT_LIVER_CANCER_SUBCLASS_G2 27 0.394298 1.31901 0.108108 0.616539 0 up vs 1WOTTON_RUNX_TARGETS_UP http://www.broadinstitute.org/gsea/msigdb/cards/WOTTON_RUNX_TARGETS_UP 20 -0.307766 -1.02483 0.4 0.616605 0 down vs 1TSENG_ADIPOGENIC_POTENTIAL_UP http://www.broadinstitute.org/gsea/msigdb/cards/TSENG_ADIPOGENIC_POTENTIAL_UP 29 -0.30764 -1.0229 0.464286 0.616652 0 down vs 1PLASARI_TGFB1_SIGNALING_VIA_NFIC_1HR_DN http://www.broadinstitute.org/gsea/msigdb/cards/PLASARI_TGFB1_SIGNALING_VIA_NFIC_1HR_DN 104 0.367118 1.33556 0.157895 0.616809 0 up vs 1DAZARD_UV_RESPONSE_CLUSTER_G4 http://www.broadinstitute.org/gsea/msigdb/cards/DAZARD_UV_RESPONSE_CLUSTER_G4 21 -0.317374 -1.0238 0.466667 0.61683 0 down vs 1NING_CHRONIC_OBSTRUCTIVE_PULMONARY_DISEASE_DN http://www.broadinstitute.org/gsea/msigdb/cards/NING_CHRONIC_OBSTRUCTIVE_PULMONARY_DISEASE_DN 117 0.328908 1.31636 0.122222 0.617209 0 up vs 1ZHOU_INFLAMMATORY_RESPONSE_LIVE_DN http://www.broadinstitute.org/gsea/msigdb/cards/ZHOU_INFLAMMATORY_RESPONSE_LIVE_DN 365 0.321325 1.32117 0.147727 0.617238 0 up vs 1CHIARADONNA_NEOPLASTIC_TRANSFORMATION_CDC25_DN http://www.broadinstitute.org/gsea/msigdb/cards/CHIARADONNA_NEOPLASTIC_TRANSFORMATION_CDC25_DN 153 -0.204185 -1.02498 0.357143 0.617244 0 down vs 1UEDA_PERIFERAL_CLOCK http://www.broadinstitute.org/gsea/msigdb/cards/UEDA_PERIFERAL_CLOCK 167 0.339302 1.32601 0.1 0.617342 0 up vs 1GENTILE_UV_RESPONSE_CLUSTER_D9 http://www.broadinstitute.org/gsea/msigdb/cards/GENTILE_UV_RESPONSE_CLUSTER_D9 27 0.432111 1.33594 0.1125 0.617409 0 up vs 1PROVENZANI_METASTASIS_DN http://www.broadinstitute.org/gsea/msigdb/cards/PROVENZANI_METASTASIS_DN 135 -0.199746 -1.02381 0.357143 0.617714 0 down vs 1SHIPP_DLBCL_VS_FOLLICULAR_LYMPHOMA_DN http://www.broadinstitute.org/gsea/msigdb/cards/SHIPP_DLBCL_VS_FOLLICULAR_LYMPHOMA_DN 44 -0.335162 -1.02562 0.371429 0.61774 0 down vs 1ROPERO_HDAC2_TARGETS http://www.broadinstitute.org/gsea/msigdb/cards/ROPERO_HDAC2_TARGETS 108 0.333429 1.32977 0.107143 0.61788 0 up vs 1NIKOLSKY_BREAST_CANCER_1Q21_AMPLICON http://www.broadinstitute.org/gsea/msigdb/cards/NIKOLSKY_BREAST_CANCER_1Q21_AMPLICON 38 -0.376774 -1.02501 0.560976 0.618087 0 down vs 1NIKOLSKY_BREAST_CANCER_11Q12_Q14_AMPLICON http://www.broadinstitute.org/gsea/msigdb/cards/NIKOLSKY_BREAST_CANCER_11Q12_Q14_AMPLICON 153 0.359143 1.32762 0.135135 0.618149 0 up vs 1CREIGHTON_ENDOCRINE_THERAPY_RESISTANCE_4 http://www.broadinstitute.org/gsea/msigdb/cards/CREIGHTON_ENDOCRINE_THERAPY_RESISTANCE_4 291 0.325296 1.32635 0.147727 0.618342 0 up vs 1TONKS_TARGETS_OF_RUNX1_RUNX1T1_FUSION_MONOCYTE_UP http://www.broadinstitute.org/gsea/msigdb/cards/TONKS_TARGETS_OF_RUNX1_RUNX1T1_FUSION_MONOCYTE_UP 199 0.351829 1.32147 0.142857 0.618408 0 up vs 1SMID_BREAST_CANCER_LUMINAL_B_UP http://www.broadinstitute.org/gsea/msigdb/cards/SMID_BREAST_CANCER_LUMINAL_B_UP 168 0.357508 1.31659 0.111111 0.618448 0 up vs 1RIZKI_TUMOR_INVASIVENESS_2D_UP http://www.broadinstitute.org/gsea/msigdb/cards/RIZKI_TUMOR_INVASIVENESS_2D_UP 67 0.353664 1.32196 0.0886076 0.618559 0 up vs 1SILIGAN_TARGETS_OF_EWS_FLI1_FUSION_UP http://www.broadinstitute.org/gsea/msigdb/cards/SILIGAN_TARGETS_OF_EWS_FLI1_FUSION_UP 15 0.459736 1.32311 0.169231 0.618691 0 up vs 1LUI_THYROID_CANCER_PAX8_PPARG_UP http://www.broadinstitute.org/gsea/msigdb/cards/LUI_THYROID_CANCER_PAX8_PPARG_UP 43 0.412133 1.33022 0.12987 0.618774 0 up vs 1VANTVEER_BREAST_CANCER_ESR1_UP http://www.broadinstitute.org/gsea/msigdb/cards/VANTVEER_BREAST_CANCER_ESR1_UP 164 0.362657 1.32481 0.170732 0.618856 0 up vs 1MIDORIKAWA_AMPLIFIED_IN_LIVER_CANCER http://www.broadinstitute.org/gsea/msigdb/cards/MIDORIKAWA_AMPLIFIED_IN_LIVER_CANCER 54 0.381604 1.32247 0.101266 0.618932 0 up vs 1GENTILE_UV_RESPONSE_CLUSTER_D8 http://www.broadinstitute.org/gsea/msigdb/cards/GENTILE_UV_RESPONSE_CLUSTER_D8 39 0.429229 1.32669 0.186667 0.618998 0 up vs 1MARIADASON_RESPONSE_TO_BUTYRATE_SULINDAC_4 http://www.broadinstitute.org/gsea/msigdb/cards/MARIADASON_RESPONSE_TO_BUTYRATE_SULINDAC_4 21 0.46221 1.32412 0.16 0.619048 0 up vs 1CUI_TCF21_TARGETS_2_UP http://www.broadinstitute.org/gsea/msigdb/cards/CUI_TCF21_TARGETS_2_UP 420 0.352846 1.33089 0.111111 0.619137 0 up vs 1MA_MYELOID_DIFFERENTIATION_UP http://www.broadinstitute.org/gsea/msigdb/cards/MA_MYELOID_DIFFERENTIATION_UP 39 0.360834 1.32319 0.113924 0.62035 0 up vs 1YOSHIOKA_LIVER_CANCER_EARLY_RECURRENCE_UP http://www.broadinstitute.org/gsea/msigdb/cards/YOSHIOKA_LIVER_CANCER_EARLY_RECURRENCE_UP 37 0.362916 1.33301 0.0779221 0.620353 0 up vs 1GUO_HEX_TARGETS_UP http://www.broadinstitute.org/gsea/msigdb/cards/GUO_HEX_TARGETS_UP 80 0.375623 1.30855 0.153846 0.620769 0 up vs 1IVANOVA_HEMATOPOIESIS_STEM_CELL http://www.broadinstitute.org/gsea/msigdb/cards/IVANOVA_HEMATOPOIESIS_STEM_CELL 245 0.347217 1.33095 0.0909091 0.620834 0 up vs 1TONG_INTERACT_WITH_PTTG1 http://www.broadinstitute.org/gsea/msigdb/cards/TONG_INTERACT_WITH_PTTG1 57 0.385868 1.33216 0.0864198 0.621064 0 up vs 1GAVIN_FOXP3_TARGETS_CLUSTER_P7 http://www.broadinstitute.org/gsea/msigdb/cards/GAVIN_FOXP3_TARGETS_CLUSTER_P7 84 -0.244211 -1.01931 0.44 0.621453 0 down vs 1AMIT_EGF_RESPONSE_480_HELA http://www.broadinstitute.org/gsea/msigdb/cards/AMIT_EGF_RESPONSE_480_HELA 159 -0.249865 -1.01956 0.368421 0.621893 0 down vs 1FONTAINE_THYROID_TUMOR_UNCERTAIN_MALIGNANCY_UP http://www.broadinstitute.org/gsea/msigdb/cards/FONTAINE_THYROID_TUMOR_UNCERTAIN_MALIGNANCY_UP 35 0.396555 1.33117 0.0921053 0.622259 0 up vs 1CHOI_ATL_CHRONIC_VS_ACUTE_DN http://www.broadinstitute.org/gsea/msigdb/cards/CHOI_ATL_CHRONIC_VS_ACUTE_DN 18 0.490226 1.30724 0.182927 0.6228 0 up vs 1WEINMANN_ADAPTATION_TO_HYPOXIA_DN http://www.broadinstitute.org/gsea/msigdb/cards/WEINMANN_ADAPTATION_TO_HYPOXIA_DN 40 -0.30077 -1.0177 0.444444 0.623743 0 down vs 1WONG_MITOCHONDRIA_GENE_MODULE http://www.broadinstitute.org/gsea/msigdb/cards/WONG_MITOCHONDRIA_GENE_MODULE 217 0.426988 1.30633 0.216216 0.623904 0 up vs 1HOLLEMAN_ASPARAGINASE_RESISTANCE_ALL_UP http://www.broadinstitute.org/gsea/msigdb/cards/HOLLEMAN_ASPARAGINASE_RESISTANCE_ALL_UP 22 0.433706 1.30451 0.157895 0.624504 0 up vs 1RICKMAN_TUMOR_DIFFERENTIATED_MODERATELY_VS_POORLY_DN http://www.broadinstitute.org/gsea/msigdb/cards/RICKMAN_TUMOR_DIFFERENTIATED_MODERATELY_VS_POORLY_DN 15 0.507312 1.30534 0.207792 0.625116 0 up vs 1WEST_ADRENOCORTICAL_CARCINOMA_VS_ADENOMA_DN http://www.broadinstitute.org/gsea/msigdb/cards/WEST_ADRENOCORTICAL_CARCINOMA_VS_ADENOMA_DN 24 -0.351322 -1.0163 0.483871 0.62554 0 down vs 1PETROVA_ENDOTHELIUM_LYMPHATIC_VS_BLOOD_UP http://www.broadinstitute.org/gsea/msigdb/cards/PETROVA_ENDOTHELIUM_LYMPHATIC_VS_BLOOD_UP 130 -0.237252 -1.01562 0.444444 0.625893 0 down vs 1LIU_COMMON_CANCER_GENES http://www.broadinstitute.org/gsea/msigdb/cards/LIU_COMMON_CANCER_GENES 74 0.365025 1.30451 0.188235 0.626254 0 up vs 1STOSSI_RESPONSE_TO_ESTRADIOL http://www.broadinstitute.org/gsea/msigdb/cards/STOSSI_RESPONSE_TO_ESTRADIOL 50 -0.263305 -1.01105 0.411765 0.626513 0 down vs 1BURTON_ADIPOGENESIS_PEAK_AT_8HR http://www.broadinstitute.org/gsea/msigdb/cards/BURTON_ADIPOGENESIS_PEAK_AT_8HR 39 -0.244775 -1.0113 0.45 0.626844 0 down vs 1HOWLIN_CITED1_TARGETS_1_DN http://www.broadinstitute.org/gsea/msigdb/cards/HOWLIN_CITED1_TARGETS_1_DN 35 -0.270159 -1.01465 0.4 0.626952 0 down vs 1CHEN_ETV5_TARGETS_TESTIS http://www.broadinstitute.org/gsea/msigdb/cards/CHEN_ETV5_TARGETS_TESTIS 23 -0.344407 -1.01178 0.576923 0.626956 0 down vs 1GAZDA_DIAMOND_BLACKFAN_ANEMIA_PROGENITOR_UP http://www.broadinstitute.org/gsea/msigdb/cards/GAZDA_DIAMOND_BLACKFAN_ANEMIA_PROGENITOR_UP 39 -0.239954 -1.01356 0.423077 0.627078 0 down vs 1MARIADASON_REGULATED_BY_HISTONE_ACETYLATION_DN http://www.broadinstitute.org/gsea/msigdb/cards/MARIADASON_REGULATED_BY_HISTONE_ACETYLATION_DN 49 0.420687 1.302 0.132353 0.627321 0 up vs 1YAGI_AML_SURVIVAL http://www.broadinstitute.org/gsea/msigdb/cards/YAGI_AML_SURVIVAL 127 0.326234 1.29878 0.144444 0.627558 0 up vs 1SHIN_B_CELL_LYMPHOMA_CLUSTER_3 http://www.broadinstitute.org/gsea/msigdb/cards/SHIN_B_CELL_LYMPHOMA_CLUSTER_3 28 -0.342199 -1.01294 0.551724 0.627625 0 down vs 1BOQUEST_STEM_CELL_CULTURED_VS_FRESH_UP http://www.broadinstitute.org/gsea/msigdb/cards/BOQUEST_STEM_CELL_CULTURED_VS_FRESH_UP 416 -0.216966 -1.01183 0.318182 0.627749 0 down vs 1LENAOUR_DENDRITIC_CELL_MATURATION_UP http://www.broadinstitute.org/gsea/msigdb/cards/LENAOUR_DENDRITIC_CELL_MATURATION_UP 113 -0.23829 -1.01359 0.366667 0.627854 0 down vs 1AMUNDSON_POOR_SURVIVAL_AFTER_GAMMA_RADIATION_2G http://www.broadinstitute.org/gsea/msigdb/cards/AMUNDSON_POOR_SURVIVAL_AFTER_GAMMA_RADIATION_2G 166 -0.227931 -1.01213 0.3125 0.628127 0 down vs 1CHO_NR4A1_TARGETS http://www.broadinstitute.org/gsea/msigdb/cards/CHO_NR4A1_TARGETS 32 0.379451 1.30048 0.0882353 0.628137 0 up vs 1CHAUHAN_RESPONSE_TO_METHOXYESTRADIOL_UP http://www.broadinstitute.org/gsea/msigdb/cards/CHAUHAN_RESPONSE_TO_METHOXYESTRADIOL_UP 50 0.390625 1.30206 0.139535 0.62877 0 up vs 1APPIERTO_RESPONSE_TO_FENRETINIDE_DN http://www.broadinstitute.org/gsea/msigdb/cards/APPIERTO_RESPONSE_TO_FENRETINIDE_DN 50 0.382704 1.29897 0.168675 0.628899 0 up vs 1KANG_DOXORUBICIN_RESISTANCE_DN http://www.broadinstitute.org/gsea/msigdb/cards/KANG_DOXORUBICIN_RESISTANCE_DN 18 0.516888 1.30067 0.191176 0.629311 0 up vs 1CONRAD_STEM_CELL http://www.broadinstitute.org/gsea/msigdb/cards/CONRAD_STEM_CELL 39 0.366124 1.297 0.121622 0.629376 0 up vs 1BOUDOUKHA_BOUND_BY_IGF2BP2 http://www.broadinstitute.org/gsea/msigdb/cards/BOUDOUKHA_BOUND_BY_IGF2BP2 106 0.337257 1.29933 0.168539 0.629467 0 up vs 1MADAN_DPPA4_TARGETS http://www.broadinstitute.org/gsea/msigdb/cards/MADAN_DPPA4_TARGETS 45 0.33591 1.29748 0.101266 0.629631 0 up vs 1SCHLESINGER_H3K27ME3_IN_NORMAL_AND_METHYLATED_IN_CANCER http://www.broadinstitute.org/gsea/msigdb/cards/SCHLESINGER_H3K27ME3_IN_NORMAL_AND_METHYLATED_IN_CANCER 28 0.384907 1.29324 0.1 0.63022 0 up vs 1KIM_ALL_DISORDERS_DURATION_CORR_DN http://www.broadinstitute.org/gsea/msigdb/cards/KIM_ALL_DISORDERS_DURATION_CORR_DN 142 0.395782 1.30211 0.162162 0.630431 0 up vs 1TIEN_INTESTINE_PROBIOTICS_6HR_UP http://www.broadinstitute.org/gsea/msigdb/cards/TIEN_INTESTINE_PROBIOTICS_6HR_UP 55 0.447845 1.29205 0.253165 0.630474 0 up vs 1BYSTRYKH_HEMATOPOIESIS_STEM_CELL_AND_BRAIN_QTL_CIS http://www.broadinstitute.org/gsea/msigdb/cards/BYSTRYKH_HEMATOPOIESIS_STEM_CELL_AND_BRAIN_QTL_CIS 62 0.37562 1.29256 0.135802 0.630683 0 up vs 1IGARASHI_ATF4_TARGETS_DN http://www.broadinstitute.org/gsea/msigdb/cards/IGARASHI_ATF4_TARGETS_DN 89 0.368632 1.29363 0.1875 0.630887 0 up vs 1SESTO_RESPONSE_TO_UV_C4 http://www.broadinstitute.org/gsea/msigdb/cards/SESTO_RESPONSE_TO_UV_C4 20 -0.296626 -1.00806 0.4 0.630962 0 down vs 1CASORELLI_APL_SECONDARY_VS_DE_NOVO_UP http://www.broadinstitute.org/gsea/msigdb/cards/CASORELLI_APL_SECONDARY_VS_DE_NOVO_UP 37 0.381429 1.28157 0.188406 0.631303 0 up vs 1HOLLEMAN_ASPARAGINASE_RESISTANCE_B_ALL_UP http://www.broadinstitute.org/gsea/msigdb/cards/HOLLEMAN_ASPARAGINASE_RESISTANCE_B_ALL_UP 26 0.546766 1.29581 0.242424 0.631367 0 up vs 1SCHLOSSER_MYC_AND_SERUM_RESPONSE_SYNERGY http://www.broadinstitute.org/gsea/msigdb/cards/SCHLOSSER_MYC_AND_SERUM_RESPONSE_SYNERGY 31 0.44733 1.2836 0.24 0.63172 0 up vs 1KAAB_HEART_ATRIUM_VS_VENTRICLE_DN http://www.broadinstitute.org/gsea/msigdb/cards/KAAB_HEART_ATRIUM_VS_VENTRICLE_DN 260 0.318324 1.2898 0.172043 0.631824 0 up vs 1ASTON_MAJOR_DEPRESSIVE_DISORDER_UP http://www.broadinstitute.org/gsea/msigdb/cards/ASTON_MAJOR_DEPRESSIVE_DISORDER_UP 46 0.370836 1.29393 0.125 0.631993 0 up vs 1XU_GH1_EXOGENOUS_TARGETS_DN http://www.broadinstitute.org/gsea/msigdb/cards/XU_GH1_EXOGENOUS_TARGETS_DN 116 0.32473 1.28189 0.185185 0.632042 0 up vs 1HOEGERKORP_CD44_TARGETS_DIRECT_UP http://www.broadinstitute.org/gsea/msigdb/cards/HOEGERKORP_CD44_TARGETS_DIRECT_UP 27 -0.272897 -1.00592 0.413793 0.632044 0 down vs 1FLOTHO_PEDIATRIC_ALL_THERAPY_RESPONSE_DN http://www.broadinstitute.org/gsea/msigdb/cards/FLOTHO_PEDIATRIC_ALL_THERAPY_RESPONSE_DN 28 -0.295595 -1.00622 0.555556 0.63225 0 down vs 1ZHU_SKIL_TARGETS_UP http://www.broadinstitute.org/gsea/msigdb/cards/ZHU_SKIL_TARGETS_UP 20 -0.352415 -1.00527 0.529412 0.632403 0 down vs 1GARGALOVIC_RESPONSE_TO_OXIDIZED_PHOSPHOLIPIDS_GREY_DN http://www.broadinstitute.org/gsea/msigdb/cards/GARGALOVIC_RESPONSE_TO_OXIDIZED_PHOSPHOLIPIDS_GREY_DN 68 0.344907 1.28899 0.115385 0.632426 0 up vs 1JAIN_NFKB_SIGNALING http://www.broadinstitute.org/gsea/msigdb/cards/JAIN_NFKB_SIGNALING 74 0.358602 1.2843 0.151163 0.632779 0 up vs 1TIEN_INTESTINE_PROBIOTICS_2HR_UP http://www.broadinstitute.org/gsea/msigdb/cards/TIEN_INTESTINE_PROBIOTICS_2HR_UP 27 0.428736 1.28372 0.189873 0.632989 0 up vs 1RICKMAN_TUMOR_DIFFERENTIATED_MODERATELY_VS_POORLY_UP http://www.broadinstitute.org/gsea/msigdb/cards/RICKMAN_TUMOR_DIFFERENTIATED_MODERATELY_VS_POORLY_UP 117 -0.212057 -1.00636 0.533333 0.633045 0 down vs 1MYLLYKANGAS_AMPLIFICATION_HOT_SPOT_23 http://www.broadinstitute.org/gsea/msigdb/cards/MYLLYKANGAS_AMPLIFICATION_HOT_SPOT_23 18 0.50515 1.28819 0.177419 0.633132 0 up vs 1WIKMAN_ASBESTOS_LUNG_CANCER_UP http://www.broadinstitute.org/gsea/msigdb/cards/WIKMAN_ASBESTOS_LUNG_CANCER_UP 17 0.500988 1.28201 0.191176 0.633157 0 up vs 1LI_AMPLIFIED_IN_LUNG_CANCER http://www.broadinstitute.org/gsea/msigdb/cards/LI_AMPLIFIED_IN_LUNG_CANCER 177 0.356089 1.28538 0.15 0.633218 0 up vs 1RODRIGUES_NTN1_TARGETS_UP http://www.broadinstitute.org/gsea/msigdb/cards/RODRIGUES_NTN1_TARGETS_UP 16 0.427968 1.29413 0.149254 0.633281 0 up vs 1IIZUKA_LIVER_CANCER_PROGRESSION_G2_G3_UP http://www.broadinstitute.org/gsea/msigdb/cards/IIZUKA_LIVER_CANCER_PROGRESSION_G2_G3_UP 28 0.385188 1.29453 0.0757576 0.633369 0 up vs 1BOYLAN_MULTIPLE_MYELOMA_D_CLUSTER_UP http://www.broadinstitute.org/gsea/msigdb/cards/BOYLAN_MULTIPLE_MYELOMA_D_CLUSTER_UP 27 0.400357 1.28984 0.0972222 0.63341 0 up vs 1PACHER_TARGETS_OF_IGF1_AND_IGF2_UP http://www.broadinstitute.org/gsea/msigdb/cards/PACHER_TARGETS_OF_IGF1_AND_IGF2_UP 35 0.355445 1.27872 0.102941 0.633648 0 up vs 1WANG_IMMORTALIZED_BY_HOXA9_AND_MEIS1_UP http://www.broadinstitute.org/gsea/msigdb/cards/WANG_IMMORTALIZED_BY_HOXA9_AND_MEIS1_UP 29 0.377154 1.28443 0.126761 0.634057 0 up vs 1WENG_POR_TARGETS_LIVER_UP http://www.broadinstitute.org/gsea/msigdb/cards/WENG_POR_TARGETS_LIVER_UP 41 0.370564 1.2796 0.146667 0.634245 0 up vs 1NIELSEN_LIPOSARCOMA_DN http://www.broadinstitute.org/gsea/msigdb/cards/NIELSEN_LIPOSARCOMA_DN 19 0.418139 1.28213 0.166667 0.634444 0 up vs 1WANG_NEOPLASTIC_TRANSFORMATION_BY_CCND1_MYC http://www.broadinstitute.org/gsea/msigdb/cards/WANG_NEOPLASTIC_TRANSFORMATION_BY_CCND1_MYC 21 0.41152 1.29008 0.107692 0.634444 0 up vs 1DORMOY_ELAVL1_TARGETS http://www.broadinstitute.org/gsea/msigdb/cards/DORMOY_ELAVL1_TARGETS 17 -0.301028 -1.00318 0.48 0.634503 0 down vs 1HOUSTIS_ROS http://www.broadinstitute.org/gsea/msigdb/cards/HOUSTIS_ROS 36 0.381325 1.27786 0.138889 0.634807 0 up vs 1BYSTRYKH_HEMATOPOIESIS_STEM_CELL_SCP2_QTL_TRANS http://www.broadinstitute.org/gsea/msigdb/cards/BYSTRYKH_HEMATOPOIESIS_STEM_CELL_SCP2_QTL_TRANS 24 0.382339 1.28538 0.1 0.634863 0 up vs 1GOTTWEIN_TARGETS_OF_KSHV_MIR_K12_11 http://www.broadinstitute.org/gsea/msigdb/cards/GOTTWEIN_TARGETS_OF_KSHV_MIR_K12_11 62 -0.241709 -0.999948 0.5 0.634995 0 down vs 1SILIGAN_BOUND_BY_EWS_FLT1_FUSION http://www.broadinstitute.org/gsea/msigdb/cards/SILIGAN_BOUND_BY_EWS_FLT1_FUSION 46 0.363517 1.27992 0.147059 0.635008 0 up vs 1SCHAEFFER_PROSTATE_DEVELOPMENT_48HR_DN http://www.broadinstitute.org/gsea/msigdb/cards/SCHAEFFER_PROSTATE_DEVELOPMENT_48HR_DN 416 0.339607 1.2788 0.146667 0.63504 0 up vs 1KUMAR_TARGETS_OF_MLL_AF9_FUSION http://www.broadinstitute.org/gsea/msigdb/cards/KUMAR_TARGETS_OF_MLL_AF9_FUSION 394 -0.199611 -1.00333 0.555556 0.635079 0 down vs 1KYNG_DNA_DAMAGE_BY_UV http://www.broadinstitute.org/gsea/msigdb/cards/KYNG_DNA_DAMAGE_BY_UV 62 -0.225939 -0.99756 0.458333 0.635182 0 down vs 1VALK_AML_CLUSTER_16 http://www.broadinstitute.org/gsea/msigdb/cards/VALK_AML_CLUSTER_16 25 -0.287753 -1.00198 0.461538 0.635289 0 down vs 1YIH_RESPONSE_TO_ARSENITE_C2 http://www.broadinstitute.org/gsea/msigdb/cards/YIH_RESPONSE_TO_ARSENITE_C2 18 -0.314273 -0.994699 0.518519 0.635331 0 down vs 1BOQUEST_STEM_CELL_DN http://www.broadinstitute.org/gsea/msigdb/cards/BOQUEST_STEM_CELL_DN 214 -0.259564 -1.00076 0.428571 0.635368 0 down vs 1GENTILE_UV_HIGH_DOSE_DN http://www.broadinstitute.org/gsea/msigdb/cards/GENTILE_UV_HIGH_DOSE_DN 306 -0.225844 -1.0014 0.388889 0.635469 0 down vs 1HUI_MAPK14_TARGETS_UP http://www.broadinstitute.org/gsea/msigdb/cards/HUI_MAPK14_TARGETS_UP 21 -0.287755 -0.997769 0.5 0.635495 0 down vs 1SAFFORD_T_LYMPHOCYTE_ANERGY http://www.broadinstitute.org/gsea/msigdb/cards/SAFFORD_T_LYMPHOCYTE_ANERGY 86 -0.236494 -1.00007 0.45 0.635698 0 down vs 1WILCOX_RESPONSE_TO_PROGESTERONE_UP http://www.broadinstitute.org/gsea/msigdb/cards/WILCOX_RESPONSE_TO_PROGESTERONE_UP 146 -0.267027 -0.996188 0.533333 0.635941 0 down vs 1WHITEFORD_PEDIATRIC_CANCER_MARKERS http://www.broadinstitute.org/gsea/msigdb/cards/WHITEFORD_PEDIATRIC_CANCER_MARKERS 116 -0.334175 -0.997997 0.4375 0.63598 0 down vs 1LABBE_TGFB1_TARGETS_UP http://www.broadinstitute.org/gsea/msigdb/cards/LABBE_TGFB1_TARGETS_UP 101 -0.222131 -0.992719 0.545455 0.636005 0 down vs 1BURTON_ADIPOGENESIS_7 http://www.broadinstitute.org/gsea/msigdb/cards/BURTON_ADIPOGENESIS_7 49 -0.239844 -0.994756 0.5 0.636066 0 down vs 1BERNARD_PPAPDC1B_TARGETS_UP http://www.broadinstitute.org/gsea/msigdb/cards/BERNARD_PPAPDC1B_TARGETS_UP 36 -0.233921 -0.993065 0.416667 0.636147 0 down vs 1BURTON_ADIPOGENESIS_5 http://www.broadinstitute.org/gsea/msigdb/cards/BURTON_ADIPOGENESIS_5 119 0.358613 1.28551 0.13253 0.636156 0 up vs 1FAELT_B_CLL_WITH_VH_REARRANGEMENTS_DN http://www.broadinstitute.org/gsea/msigdb/cards/FAELT_B_CLL_WITH_VH_REARRANGEMENTS_DN 45 0.395717 1.27676 0.109756 0.636468 0 up vs 1HUMMERICH_MALIGNANT_SKIN_TUMOR_UP http://www.broadinstitute.org/gsea/msigdb/cards/HUMMERICH_MALIGNANT_SKIN_TUMOR_UP 15 -0.338822 -0.998186 0.486486 0.636553 0 down vs 1HOFMANN_CELL_LYMPHOMA_UP http://www.broadinstitute.org/gsea/msigdb/cards/HOFMANN_CELL_LYMPHOMA_UP 50 -0.273946 -0.993289 0.44 0.636606 0 down vs 1WESTON_VEGFA_TARGETS_3HR http://www.broadinstitute.org/gsea/msigdb/cards/WESTON_VEGFA_TARGETS_3HR 72 -0.209715 -0.996257 0.413793 0.636702 0 down vs 1WAKABAYASHI_ADIPOGENESIS_PPARG_RXRA_BOUND_36HR http://www.broadinstitute.org/gsea/msigdb/cards/WAKABAYASHI_ADIPOGENESIS_PPARG_RXRA_BOUND_36HR 149 0.323788 1.28588 0.159091 0.6368 0 up vs 1CHIANG_LIVER_CANCER_SUBCLASS_UNANNOTATED_UP http://www.broadinstitute.org/gsea/msigdb/cards/CHIANG_LIVER_CANCER_SUBCLASS_UNANNOTATED_UP 79 -0.240898 -0.994781 0.5 0.636866 0 down vs 1REN_ALVEOLAR_RHABDOMYOSARCOMA_DN http://www.broadinstitute.org/gsea/msigdb/cards/REN_ALVEOLAR_RHABDOMYOSARCOMA_DN 404 -0.23125 -0.990954 0.5 0.636884 0 down vs 1HATADA_METHYLATED_IN_LUNG_CANCER_DN http://www.broadinstitute.org/gsea/msigdb/cards/HATADA_METHYLATED_IN_LUNG_CANCER_DN 34 0.370444 1.28634 0.136986 0.637128 0 up vs 1PUJANA_BRCA_CENTERED_NETWORK http://www.broadinstitute.org/gsea/msigdb/cards/PUJANA_BRCA_CENTERED_NETWORK 117 -0.361323 -0.99831 0.571429 0.63717 0 down vs 1LINDGREN_BLADDER_CANCER_CLUSTER_1_DN http://www.broadinstitute.org/gsea/msigdb/cards/LINDGREN_BLADDER_CANCER_CLUSTER_1_DN 370 -0.217622 -0.994989 0.444444 0.637329 0 down vs 1GEORGES_CELL_CYCLE_MIR192_TARGETS http://www.broadinstitute.org/gsea/msigdb/cards/GEORGES_CELL_CYCLE_MIR192_TARGETS 61 -0.33146 -0.991131 0.545455 0.637447 0 down vs 1KORKOLA_TERATOMA_UP http://www.broadinstitute.org/gsea/msigdb/cards/KORKOLA_TERATOMA_UP 16 -0.33152 -0.986987 0.5 0.637957 0 down vs 1TAKEDA_TARGETS_OF_NUP98_HOXA9_FUSION_6HR_DN http://www.broadinstitute.org/gsea/msigdb/cards/TAKEDA_TARGETS_OF_NUP98_HOXA9_FUSION_6HR_DN 39 -0.283106 -0.99125 0.571429 0.638032 0 down vs 1NATSUME_RESPONSE_TO_INTERFERON_BETA_UP http://www.broadinstitute.org/gsea/msigdb/cards/NATSUME_RESPONSE_TO_INTERFERON_BETA_UP 71 -0.24641 -0.986442 0.478261 0.638127 0 down vs 1LIU_CMYB_TARGETS_UP http://www.broadinstitute.org/gsea/msigdb/cards/LIU_CMYB_TARGETS_UP 157 -0.189122 -0.989698 0.416667 0.638252 0 down vs 1OKAMOTO_LIVER_CANCER_MULTICENTRIC_OCCURRENCE_UP http://www.broadinstitute.org/gsea/msigdb/cards/OKAMOTO_LIVER_CANCER_MULTICENTRIC_OCCURRENCE_UP 24 -0.304308 -0.988143 0.448276 0.638323 0 down vs 1DELLA_RESPONSE_TO_TSA_AND_BUTYRATE http://www.broadinstitute.org/gsea/msigdb/cards/DELLA_RESPONSE_TO_TSA_AND_BUTYRATE 21 -0.285254 -0.988639 0.545455 0.638341 0 down vs 1PHONG_TNF_RESPONSE_VIA_P38_COMPLETE http://www.broadinstitute.org/gsea/msigdb/cards/PHONG_TNF_RESPONSE_VIA_P38_COMPLETE 223 -0.223094 -0.987678 0.368421 0.638451 0 down vs 1VANASSE_BCL2_TARGETS_UP http://www.broadinstitute.org/gsea/msigdb/cards/VANASSE_BCL2_TARGETS_UP 39 -0.257433 -0.987242 0.548387 0.638454 0 down vs 1NATSUME_RESPONSE_TO_INTERFERON_BETA_DN http://www.broadinstitute.org/gsea/msigdb/cards/NATSUME_RESPONSE_TO_INTERFERON_BETA_DN 52 -0.249855 -0.988775 0.533333 0.639029 0 down vs 1SHI_SPARC_TARGETS_UP http://www.broadinstitute.org/gsea/msigdb/cards/SHI_SPARC_TARGETS_UP 24 -0.28614 -0.983979 0.472222 0.640951 0 down vs 1SCHEIDEREIT_IKK_INTERACTING_PROTEINS http://www.broadinstitute.org/gsea/msigdb/cards/SCHEIDEREIT_IKK_INTERACTING_PROTEINS 58 -0.239956 -0.984066 0.565217 0.641677 0 down vs 1MITSIADES_RESPONSE_TO_APLIDIN_UP http://www.broadinstitute.org/gsea/msigdb/cards/MITSIADES_RESPONSE_TO_APLIDIN_UP 426 -0.19481 -0.980737 0.6 0.645937 0 down vs 1GARGALOVIC_RESPONSE_TO_OXIDIZED_PHOSPHOLIPIDS_GREY_UP http://www.broadinstitute.org/gsea/msigdb/cards/GARGALOVIC_RESPONSE_TO_OXIDIZED_PHOSPHOLIPIDS_GREY_UP 16 -0.34404 -0.980103 0.5 0.646487 0 down vs 1ZIRN_TRETINOIN_RESPONSE_WT1_UP http://www.broadinstitute.org/gsea/msigdb/cards/ZIRN_TRETINOIN_RESPONSE_WT1_UP 22 -0.281539 -0.978631 0.433333 0.646987 0 down vs 1OSWALD_HEMATOPOIETIC_STEM_CELL_IN_COLLAGEN_GEL_UP http://www.broadinstitute.org/gsea/msigdb/cards/OSWALD_HEMATOPOIETIC_STEM_CELL_IN_COLLAGEN_GEL_UP 225 -0.19114 -0.97909 0.416667 0.64714 0 down vs 1AMUNDSON_RESPONSE_TO_ARSENITE http://www.broadinstitute.org/gsea/msigdb/cards/AMUNDSON_RESPONSE_TO_ARSENITE 216 0.325911 1.27076 0.183908 0.648353 0 up vs 1SHAFFER_IRF4_TARGETS_IN_MYELOMA_VS_MATURE_B_LYMPHOCYTE http://www.broadinstitute.org/gsea/msigdb/cards/SHAFFER_IRF4_TARGETS_IN_MYELOMA_VS_MATURE_B_LYMPHOCYTE 101 0.346329 1.26942 0.182927 0.648879 0 up vs 1BECKER_TAMOXIFEN_RESISTANCE_DN http://www.broadinstitute.org/gsea/msigdb/cards/BECKER_TAMOXIFEN_RESISTANCE_DN 51 0.351389 1.27204 0.135135 0.649397 0 up vs 1GRADE_COLON_VS_RECTAL_CANCER_DN http://www.broadinstitute.org/gsea/msigdb/cards/GRADE_COLON_VS_RECTAL_CANCER_DN 53 0.334735 1.26844 0.135802 0.649639 0 up vs 1MCGARVEY_SILENCED_BY_METHYLATION_IN_COLON_CANCER http://www.broadinstitute.org/gsea/msigdb/cards/MCGARVEY_SILENCED_BY_METHYLATION_IN_COLON_CANCER 42 0.354501 1.27079 0.153846 0.649922 0 up vs 1LEE_AGING_NEOCORTEX_DN http://www.broadinstitute.org/gsea/msigdb/cards/LEE_AGING_NEOCORTEX_DN 78 0.324535 1.26958 0.121951 0.65016 0 up vs 1PAL_PRMT5_TARGETS_DN http://www.broadinstitute.org/gsea/msigdb/cards/PAL_PRMT5_TARGETS_DN 29 -0.260281 -0.976062 0.53125 0.650662 0 down vs 1HOSHIDA_LIVER_CANCER_SURVIVAL_DN http://www.broadinstitute.org/gsea/msigdb/cards/HOSHIDA_LIVER_CANCER_SURVIVAL_DN 111 0.323675 1.26675 0.152941 0.651306 0 up vs 1YAO_TEMPORAL_RESPONSE_TO_PROGESTERONE_CLUSTER_17 http://www.broadinstitute.org/gsea/msigdb/cards/YAO_TEMPORAL_RESPONSE_TO_PROGESTERONE_CLUSTER_17 177 0.383762 1.26728 0.186047 0.65137 0 up vs 1BRUINS_UVC_RESPONSE_MIDDLE http://www.broadinstitute.org/gsea/msigdb/cards/BRUINS_UVC_RESPONSE_MIDDLE 90 0.347759 1.27086 0.158537 0.651397 0 up vs 1HAN_JNK_SINGALING_DN http://www.broadinstitute.org/gsea/msigdb/cards/HAN_JNK_SINGALING_DN 39 -0.263724 -0.973197 0.571429 0.65298 0 down vs 1PASQUALUCCI_LYMPHOMA_BY_GC_STAGE_DN http://www.broadinstitute.org/gsea/msigdb/cards/PASQUALUCCI_LYMPHOMA_BY_GC_STAGE_DN 158 -0.239512 -0.973354 0.44 0.65355 0 down vs 1ODONNELL_TARGETS_OF_MYC_AND_TFRC_DN http://www.broadinstitute.org/gsea/msigdb/cards/ODONNELL_TARGETS_OF_MYC_AND_TFRC_DN 45 -0.366225 -0.97354 0.566667 0.654163 0 down vs 1VECCHI_GASTRIC_CANCER_EARLY_UP http://www.broadinstitute.org/gsea/msigdb/cards/VECCHI_GASTRIC_CANCER_EARLY_UP 409 -0.231076 -0.971153 0.428571 0.655911 0 down vs 1VALK_AML_CLUSTER_2 http://www.broadinstitute.org/gsea/msigdb/cards/VALK_AML_CLUSTER_2 29 0.376236 1.26281 0.114286 0.659238 0 up vs 1DAZARD_RESPONSE_TO_UV_SCC_DN http://www.broadinstitute.org/gsea/msigdb/cards/DAZARD_RESPONSE_TO_UV_SCC_DN 119 -0.238795 -0.968446 0.473684 0.660226 0 down vs 1YAMAZAKI_TCEB3_TARGETS_UP http://www.broadinstitute.org/gsea/msigdb/cards/YAMAZAKI_TCEB3_TARGETS_UP 172 0.364992 1.26287 0.15493 0.660648 0 up vs 1ABRAHAM_ALPC_VS_MULTIPLE_MYELOMA_UP http://www.broadinstitute.org/gsea/msigdb/cards/ABRAHAM_ALPC_VS_MULTIPLE_MYELOMA_UP 26 -0.28309 -0.966454 0.551724 0.662793 0 down vs 1LEE_CALORIE_RESTRICTION_NEOCORTEX_UP http://www.broadinstitute.org/gsea/msigdb/cards/LEE_CALORIE_RESTRICTION_NEOCORTEX_UP 82 0.324299 1.25718 0.180723 0.663645 0 up vs 1GARGALOVIC_RESPONSE_TO_OXIDIZED_PHOSPHOLIPIDS_TURQUOISE_UP http://www.broadinstitute.org/gsea/msigdb/cards/GARGALOVIC_RESPONSE_TO_OXIDIZED_PHOSPHOLIPIDS_TURQUOISE_UP 76 -0.27461 -0.965206 0.565217 0.664131 0 down vs 1HELLER_SILENCED_BY_METHYLATION_DN http://www.broadinstitute.org/gsea/msigdb/cards/HELLER_SILENCED_BY_METHYLATION_DN 104 0.315884 1.25525 0.192308 0.664757 0 up vs 1BERTUCCI_MEDULLARY_VS_DUCTAL_BREAST_CANCER_DN http://www.broadinstitute.org/gsea/msigdb/cards/BERTUCCI_MEDULLARY_VS_DUCTAL_BREAST_CANCER_DN 165 0.386845 1.25735 0.175676 0.664766 0 up vs 1QI_HYPOXIA http://www.broadinstitute.org/gsea/msigdb/cards/QI_HYPOXIA 138 -0.200861 -0.96408 0.380952 0.665179 0 down vs 1SHEDDEN_LUNG_CANCER_GOOD_SURVIVAL_A5 http://www.broadinstitute.org/gsea/msigdb/cards/SHEDDEN_LUNG_CANCER_GOOD_SURVIVAL_A5 66 0.385551 1.26012 0.211268 0.665796 0 up vs 1DARWICHE_PAPILLOMA_RISK_LOW_DN http://www.broadinstitute.org/gsea/msigdb/cards/DARWICHE_PAPILLOMA_RISK_LOW_DN 158 0.306563 1.25854 0.186813 0.665816 0 up vs 1YOSHIMURA_MAPK8_TARGETS_DN http://www.broadinstitute.org/gsea/msigdb/cards/YOSHIMURA_MAPK8_TARGETS_DN 357 0.320396 1.25531 0.172414 0.666123 0 up vs 1MILI_PSEUDOPODIA_CHEMOTAXIS_DN http://www.broadinstitute.org/gsea/msigdb/cards/MILI_PSEUDOPODIA_CHEMOTAXIS_DN 440 0.325921 1.25741 0.209302 0.666146 0 up vs 1HSIAO_HOUSEKEEPING_GENES http://www.broadinstitute.org/gsea/msigdb/cards/HSIAO_HOUSEKEEPING_GENES 387 0.354784 1.25942 0.197802 0.66615 0 up vs 1HOSHIDA_LIVER_CANCER_LATE_RECURRENCE_DN http://www.broadinstitute.org/gsea/msigdb/cards/HOSHIDA_LIVER_CANCER_LATE_RECURRENCE_DN 68 0.332578 1.25891 0.153846 0.666208 0 up vs 1BROWNE_HCMV_INFECTION_4HR_DN http://www.broadinstitute.org/gsea/msigdb/cards/BROWNE_HCMV_INFECTION_4HR_DN 251 0.309468 1.25421 0.211765 0.666448 0 up vs 1KAAB_HEART_ATRIUM_VS_VENTRICLE_UP http://www.broadinstitute.org/gsea/msigdb/cards/KAAB_HEART_ATRIUM_VS_VENTRICLE_UP 245 0.337125 1.25368 0.186667 0.666619 0 up vs 1VERHAAK_GLIOBLASTOMA_NEURAL http://www.broadinstitute.org/gsea/msigdb/cards/VERHAAK_GLIOBLASTOMA_NEURAL 126 0.330357 1.25546 0.192771 0.667265 0 up vs 1YAUCH_HEDGEHOG_SIGNALING_PARACRINE_UP http://www.broadinstitute.org/gsea/msigdb/cards/YAUCH_HEDGEHOG_SIGNALING_PARACRINE_UP 145 0.315503 1.25751 0.182927 0.667533 0 up vs 1ZAMORA_NOS2_TARGETS_DN http://www.broadinstitute.org/gsea/msigdb/cards/ZAMORA_NOS2_TARGETS_DN 94 0.332143 1.2529 0.152941 0.667667 0 up vs 1MIKKELSEN_NPC_ICP_WITH_H3K4ME3 http://www.broadinstitute.org/gsea/msigdb/cards/MIKKELSEN_NPC_ICP_WITH_H3K4ME3 429 0.323067 1.25218 0.155844 0.668082 0 up vs 1LANDIS_ERBB2_BREAST_PRENEOPLASTIC_DN http://www.broadinstitute.org/gsea/msigdb/cards/LANDIS_ERBB2_BREAST_PRENEOPLASTIC_DN 54 0.330472 1.25085 0.16092 0.668678 0 up vs 1ZHAN_LATE_DIFFERENTIATION_GENES_DN http://www.broadinstitute.org/gsea/msigdb/cards/ZHAN_LATE_DIFFERENTIATION_GENES_DN 15 0.42597 1.25024 0.183099 0.668974 0 up vs 1YOKOE_CANCER_TESTIS_ANTIGENS http://www.broadinstitute.org/gsea/msigdb/cards/YOKOE_CANCER_TESTIS_ANTIGENS 37 0.344755 1.24967 0.15493 0.66925 0 up vs 1NAKAJIMA_EOSINOPHIL http://www.broadinstitute.org/gsea/msigdb/cards/NAKAJIMA_EOSINOPHIL 29 -0.293314 -0.959267 0.457143 0.669613 0 down vs 1YAGI_AML_RELAPSE_PROGNOSIS http://www.broadinstitute.org/gsea/msigdb/cards/YAGI_AML_RELAPSE_PROGNOSIS 35 0.365151 1.25095 0.223529 0.670035 0 up vs 1YANG_BREAST_CANCER_ESR1_DN http://www.broadinstitute.org/gsea/msigdb/cards/YANG_BREAST_CANCER_ESR1_DN 25 -0.28956 -0.959464 0.5 0.670035 0 down vs 1DARWICHE_PAPILLOMA_PROGRESSION_RISK http://www.broadinstitute.org/gsea/msigdb/cards/DARWICHE_PAPILLOMA_PROGRESSION_RISK 71 -0.201422 -0.9604 0.521739 0.670234 0 down vs 1SHEDDEN_LUNG_CANCER_POOR_SURVIVAL_A6 http://www.broadinstitute.org/gsea/msigdb/cards/SHEDDEN_LUNG_CANCER_POOR_SURVIVAL_A6 439 -0.260903 -0.960849 0.473684 0.670252 0 down vs 1HORIUCHI_WTAP_TARGETS_UP http://www.broadinstitute.org/gsea/msigdb/cards/HORIUCHI_WTAP_TARGETS_UP 294 -0.202534 -0.959789 0.416667 0.670337 0 down vs 1KLEIN_PRIMARY_EFFUSION_LYMPHOMA_DN http://www.broadinstitute.org/gsea/msigdb/cards/KLEIN_PRIMARY_EFFUSION_LYMPHOMA_DN 57 -0.28863 -0.957278 0.428571 0.672343 0 down vs 1WONG_IFNA2_RESISTANCE_DN http://www.broadinstitute.org/gsea/msigdb/cards/WONG_IFNA2_RESISTANCE_DN 34 0.361204 1.22417 0.208333 0.672772 0 up vs 1EHRLICH_ICF_SYNDROM_DN http://www.broadinstitute.org/gsea/msigdb/cards/EHRLICH_ICF_SYNDROM_DN 15 -0.372988 -0.955756 0.5 0.673977 0 down vs 1TIEN_INTESTINE_PROBIOTICS_6HR_DN http://www.broadinstitute.org/gsea/msigdb/cards/TIEN_INTESTINE_PROBIOTICS_6HR_DN 163 0.329341 1.22337 0.186047 0.674045 0 up vs 1FIGUEROA_AML_METHYLATION_CLUSTER_6_UP http://www.broadinstitute.org/gsea/msigdb/cards/FIGUEROA_AML_METHYLATION_CLUSTER_6_UP 131 0.312786 1.2242 0.197531 0.674097 0 up vs 1GRADE_METASTASIS_DN http://www.broadinstitute.org/gsea/msigdb/cards/GRADE_METASTASIS_DN 45 0.412001 1.22157 0.25 0.674954 0 up vs 1PROVENZANI_METASTASIS_UP http://www.broadinstitute.org/gsea/msigdb/cards/PROVENZANI_METASTASIS_UP 186 0.327529 1.22583 0.215909 0.675112 0 up vs 1PASQUALUCCI_LYMPHOMA_BY_GC_STAGE_UP http://www.broadinstitute.org/gsea/msigdb/cards/PASQUALUCCI_LYMPHOMA_BY_GC_STAGE_UP 274 0.307342 1.22435 0.259259 0.675121 0 up vs 1POMEROY_MEDULLOBLASTOMA_PROGNOSIS_UP http://www.broadinstitute.org/gsea/msigdb/cards/POMEROY_MEDULLOBLASTOMA_PROGNOSIS_UP 44 0.349426 1.24494 0.131579 0.675126 0 up vs 1LIN_NPAS4_TARGETS_DN http://www.broadinstitute.org/gsea/msigdb/cards/LIN_NPAS4_TARGETS_DN 63 0.330986 1.24654 0.179487 0.675154 0 up vs 1WEBER_METHYLATED_IN_COLON_CANCER http://www.broadinstitute.org/gsea/msigdb/cards/WEBER_METHYLATED_IN_COLON_CANCER 18 0.401067 1.24585 0.164179 0.675301 0 up vs 1KAMMINGA_SENESCENCE http://www.broadinstitute.org/gsea/msigdb/cards/KAMMINGA_SENESCENCE 38 0.347515 1.22513 0.194805 0.675572 0 up vs 1HESS_TARGETS_OF_HOXA9_AND_MEIS1_UP http://www.broadinstitute.org/gsea/msigdb/cards/HESS_TARGETS_OF_HOXA9_AND_MEIS1_UP 63 0.371758 1.22651 0.178571 0.675686 0 up vs 1DARWICHE_PAPILLOMA_RISK_HIGH_DN http://www.broadinstitute.org/gsea/msigdb/cards/DARWICHE_PAPILLOMA_RISK_HIGH_DN 172 0.296458 1.22603 0.228261 0.675854 0 up vs 1LIN_APC_TARGETS http://www.broadinstitute.org/gsea/msigdb/cards/LIN_APC_TARGETS 75 0.3524 1.23492 0.1 0.675861 0 up vs 1MATZUK_SPERMATOZOA http://www.broadinstitute.org/gsea/msigdb/cards/MATZUK_SPERMATOZOA 112 0.331292 1.24361 0.184211 0.675975 0 up vs 1CHNG_MULTIPLE_MYELOMA_HYPERPLOID_UP http://www.broadinstitute.org/gsea/msigdb/cards/CHNG_MULTIPLE_MYELOMA_HYPERPLOID_UP 52 0.53004 1.22743 0.304348 0.675988 0 up vs 1MAYBURD_RESPONSE_TO_L663536_UP http://www.broadinstitute.org/gsea/msigdb/cards/MAYBURD_RESPONSE_TO_L663536_UP 28 0.364894 1.2217 0.208333 0.67607 0 up vs 1MULLIGAN_NTF3_SIGNALING_VIA_INSR_AND_IGF1R_UP http://www.broadinstitute.org/gsea/msigdb/cards/MULLIGAN_NTF3_SIGNALING_VIA_INSR_AND_IGF1R_UP 23 0.398535 1.24107 0.197368 0.67608 0 up vs 1VERNOCHET_ADIPOGENESIS http://www.broadinstitute.org/gsea/msigdb/cards/VERNOCHET_ADIPOGENESIS 19 0.37126 1.22787 0.150685 0.676166 0 up vs 1ALONSO_METASTASIS_EMT_UP http://www.broadinstitute.org/gsea/msigdb/cards/ALONSO_METASTASIS_EMT_UP 36 0.395099 1.24509 0.194444 0.676252 0 up vs 1DOANE_RESPONSE_TO_ANDROGEN_UP http://www.broadinstitute.org/gsea/msigdb/cards/DOANE_RESPONSE_TO_ANDROGEN_UP 179 0.304326 1.23538 0.192771 0.67626 0 up vs 1KYNG_RESPONSE_TO_H2O2 http://www.broadinstitute.org/gsea/msigdb/cards/KYNG_RESPONSE_TO_H2O2 70 0.332219 1.22442 0.2 0.676286 0 up vs 1GRADE_COLON_AND_RECTAL_CANCER_DN http://www.broadinstitute.org/gsea/msigdb/cards/GRADE_COLON_AND_RECTAL_CANCER_DN 99 0.331696 1.24668 0.194805 0.676306 0 up vs 1CHESLER_BRAIN_QTL_CIS http://www.broadinstitute.org/gsea/msigdb/cards/CHESLER_BRAIN_QTL_CIS 74 0.350271 1.24194 0.180723 0.676312 0 up vs 1SHAFFER_IRF4_TARGETS_IN_ACTIVATED_DENDRITIC_CELL http://www.broadinstitute.org/gsea/msigdb/cards/SHAFFER_IRF4_TARGETS_IN_ACTIVATED_DENDRITIC_CELL 63 0.347974 1.22208 0.189189 0.676312 0 up vs 1KANG_AR_TARGETS_DN http://www.broadinstitute.org/gsea/msigdb/cards/KANG_AR_TARGETS_DN 19 0.394227 1.23799 0.164179 0.676341 0 up vs 1VANHARANTA_UTERINE_FIBROID_WITH_7Q_DELETION_DN http://www.broadinstitute.org/gsea/msigdb/cards/VANHARANTA_UTERINE_FIBROID_WITH_7Q_DELETION_DN 36 0.363353 1.23743 0.25 0.676353 0 up vs 1NAKAMURA_CANCER_MICROENVIRONMENT_DN http://www.broadinstitute.org/gsea/msigdb/cards/NAKAMURA_CANCER_MICROENVIRONMENT_DN 44 0.412743 1.22675 0.216216 0.676381 0 up vs 1REN_ALVEOLAR_RHABDOMYOSARCOMA_UP http://www.broadinstitute.org/gsea/msigdb/cards/REN_ALVEOLAR_RHABDOMYOSARCOMA_UP 95 0.334394 1.24227 0.169014 0.676854 0 up vs 1GROSS_ELK3_TARGETS_UP http://www.broadinstitute.org/gsea/msigdb/cards/GROSS_ELK3_TARGETS_UP 27 0.408564 1.23575 0.162162 0.67693 0 up vs 1DING_LUNG_CANCER_MUTATED_SIGNIFICANTLY http://www.broadinstitute.org/gsea/msigdb/cards/DING_LUNG_CANCER_MUTATED_SIGNIFICANTLY 26 0.366883 1.22814 0.152778 0.676953 0 up vs 1KOINUMA_COLON_CANCER_MSI_UP http://www.broadinstitute.org/gsea/msigdb/cards/KOINUMA_COLON_CANCER_MSI_UP 16 -0.315509 -0.95341 0.56 0.676998 0 down vs 1LASTOWSKA_COAMPLIFIED_WITH_MYCN http://www.broadinstitute.org/gsea/msigdb/cards/LASTOWSKA_COAMPLIFIED_WITH_MYCN 41 0.432657 1.24127 0.242424 0.677116 0 up vs 1WANG_RESPONSE_TO_BEXAROTENE_UP http://www.broadinstitute.org/gsea/msigdb/cards/WANG_RESPONSE_TO_BEXAROTENE_UP 33 0.337213 1.24263 0.17284 0.677361 0 up vs 1ABDELMOHSEN_ELAVL4_TARGETS http://www.broadinstitute.org/gsea/msigdb/cards/ABDELMOHSEN_ELAVL4_TARGETS 16 0.454483 1.2298 0.243243 0.677366 0 up vs 1TOOKER_GEMCITABINE_RESISTANCE_DN http://www.broadinstitute.org/gsea/msigdb/cards/TOOKER_GEMCITABINE_RESISTANCE_DN 121 0.344148 1.24365 0.181818 0.677377 0 up vs 1MULLIGHAN_MLL_SIGNATURE_1_DN http://www.broadinstitute.org/gsea/msigdb/cards/MULLIGHAN_MLL_SIGNATURE_1_DN 233 0.306576 1.23034 0.229885 0.677426 0 up vs 1KORKOLA_EMBRYONIC_CARCINOMA_VS_SEMINOMA_DN http://www.broadinstitute.org/gsea/msigdb/cards/KORKOLA_EMBRYONIC_CARCINOMA_VS_SEMINOMA_DN 24 0.358673 1.23808 0.148148 0.677509 0 up vs 1BENPORATH_ES_1 http://www.broadinstitute.org/gsea/msigdb/cards/BENPORATH_ES_1 367 0.338759 1.22821 0.22093 0.678197 0 up vs 1RASHI_RESPONSE_TO_IONIZING_RADIATION_5 http://www.broadinstitute.org/gsea/msigdb/cards/RASHI_RESPONSE_TO_IONIZING_RADIATION_5 145 0.315074 1.23583 0.166667 0.67825 0 up vs 1NIKOLSKY_BREAST_CANCER_19Q13.1_AMPLICON http://www.broadinstitute.org/gsea/msigdb/cards/NIKOLSKY_BREAST_CANCER_19Q13.1_AMPLICON 22 0.608894 1.22907 0.344828 0.678268 0 up vs 1KUNINGER_IGF1_VS_PDGFB_TARGETS_UP http://www.broadinstitute.org/gsea/msigdb/cards/KUNINGER_IGF1_VS_PDGFB_TARGETS_UP 78 0.339506 1.231 0.157143 0.678359 0 up vs 1WOOD_EBV_EBNA1_TARGETS_UP http://www.broadinstitute.org/gsea/msigdb/cards/WOOD_EBV_EBNA1_TARGETS_UP 110 0.32668 1.21987 0.189873 0.678465 0 up vs 1FLECHNER_PBL_KIDNEY_TRANSPLANT_OK_VS_DONOR_UP http://www.broadinstitute.org/gsea/msigdb/cards/FLECHNER_PBL_KIDNEY_TRANSPLANT_OK_VS_DONOR_UP 148 0.319443 1.23051 0.215054 0.678508 0 up vs 1BRACHAT_RESPONSE_TO_CAMPTOTHECIN_UP http://www.broadinstitute.org/gsea/msigdb/cards/BRACHAT_RESPONSE_TO_CAMPTOTHECIN_UP 26 0.39291 1.2398 0.171429 0.678513 0 up vs 1YAO_TEMPORAL_RESPONSE_TO_PROGESTERONE_CLUSTER_7 http://www.broadinstitute.org/gsea/msigdb/cards/YAO_TEMPORAL_RESPONSE_TO_PROGESTERONE_CLUSTER_7 75 0.343047 1.22853 0.153846 0.678625 0 up vs 1ACEVEDO_NORMAL_TISSUE_ADJACENT_TO_LIVER_TUMOR_DN http://www.broadinstitute.org/gsea/msigdb/cards/ACEVEDO_NORMAL_TISSUE_ADJACENT_TO_LIVER_TUMOR_DN 345 0.312853 1.23292 0.172414 0.678772 0 up vs 1MOOTHA_GLUCONEOGENESIS http://www.broadinstitute.org/gsea/msigdb/cards/MOOTHA_GLUCONEOGENESIS 32 0.352019 1.23816 0.160494 0.678864 0 up vs 1GUO_TARGETS_OF_IRS1_AND_IRS2 http://www.broadinstitute.org/gsea/msigdb/cards/GUO_TARGETS_OF_IRS1_AND_IRS2 94 0.314386 1.23234 0.177778 0.678904 0 up vs 1MATTIOLI_MGUS_VS_MULTIPLE_MYELOMA http://www.broadinstitute.org/gsea/msigdb/cards/MATTIOLI_MGUS_VS_MULTIPLE_MYELOMA 16 0.436848 1.23868 0.220779 0.678948 0 up vs 1MA_PITUITARY_FETAL_VS_ADULT_DN http://www.broadinstitute.org/gsea/msigdb/cards/MA_PITUITARY_FETAL_VS_ADULT_DN 19 -0.259517 -0.951194 0.6 0.679457 0 down vs 1VALK_AML_CLUSTER_13 http://www.broadinstitute.org/gsea/msigdb/cards/VALK_AML_CLUSTER_13 29 0.381292 1.23593 0.15625 0.679487 0 up vs 1LEE_CALORIE_RESTRICTION_MUSCLE_UP http://www.broadinstitute.org/gsea/msigdb/cards/LEE_CALORIE_RESTRICTION_MUSCLE_UP 42 0.347673 1.23318 0.225352 0.679515 0 up vs 1KYNG_DNA_DAMAGE_BY_4NQO http://www.broadinstitute.org/gsea/msigdb/cards/KYNG_DNA_DAMAGE_BY_4NQO 37 0.345022 1.23101 0.176471 0.679837 0 up vs 1JOHANSSON_BRAIN_CANCER_EARLY_VS_LATE_DN http://www.broadinstitute.org/gsea/msigdb/cards/JOHANSSON_BRAIN_CANCER_EARLY_VS_LATE_DN 45 0.354704 1.23149 0.194444 0.679885 0 up vs 1FIGUEROA_AML_METHYLATION_CLUSTER_3_UP http://www.broadinstitute.org/gsea/msigdb/cards/FIGUEROA_AML_METHYLATION_CLUSTER_3_UP 159 0.304456 1.23888 0.22619 0.679965 0 up vs 1SANSOM_APC_TARGETS_DN http://www.broadinstitute.org/gsea/msigdb/cards/SANSOM_APC_TARGETS_DN 358 -0.22618 -0.950461 0.535714 0.68005 0 down vs 1FOURNIER_ACINAR_DEVELOPMENT_LATE_2 http://www.broadinstitute.org/gsea/msigdb/cards/FOURNIER_ACINAR_DEVELOPMENT_LATE_2 270 -0.238825 -0.949861 0.666667 0.680139 0 down vs 1ROSS_ACUTE_MYELOID_LEUKEMIA_CBF http://www.broadinstitute.org/gsea/msigdb/cards/ROSS_ACUTE_MYELOID_LEUKEMIA_CBF 81 -0.225036 -0.951253 0.428571 0.680212 0 down vs 1TOMLINS_METASTASIS_DN http://www.broadinstitute.org/gsea/msigdb/cards/TOMLINS_METASTASIS_DN 20 0.398778 1.2055 0.214286 0.680489 0 up vs 1HU_GENOTOXIC_DAMAGE_24HR http://www.broadinstitute.org/gsea/msigdb/cards/HU_GENOTOXIC_DAMAGE_24HR 33 0.363656 1.21714 0.17284 0.680711 0 up vs 1ROVERSI_GLIOMA_COPY_NUMBER_UP http://www.broadinstitute.org/gsea/msigdb/cards/ROVERSI_GLIOMA_COPY_NUMBER_UP 97 0.319364 1.20579 0.189873 0.680724 0 up vs 1GINESTIER_BREAST_CANCER_20Q13_AMPLIFICATION_UP http://www.broadinstitute.org/gsea/msigdb/cards/GINESTIER_BREAST_CANCER_20Q13_AMPLIFICATION_UP 115 0.341627 1.20499 0.282051 0.680808 0 up vs 1MARIADASON_RESPONSE_TO_BUTYRATE_SULINDAC_6 http://www.broadinstitute.org/gsea/msigdb/cards/MARIADASON_RESPONSE_TO_BUTYRATE_SULINDAC_6 48 0.379417 1.21862 0.186667 0.680955 0 up vs 1DARWICHE_SKIN_TUMOR_PROMOTER_DN http://www.broadinstitute.org/gsea/msigdb/cards/DARWICHE_SKIN_TUMOR_PROMOTER_DN 179 0.288419 1.20659 0.222222 0.681054 0 up vs 1KYNG_WERNER_SYNDROM_AND_NORMAL_AGING_DN http://www.broadinstitute.org/gsea/msigdb/cards/KYNG_WERNER_SYNDROM_AND_NORMAL_AGING_DN 221 0.307292 1.21803 0.241758 0.681091 0 up vs 1DEMAGALHAES_AGING_DN http://www.broadinstitute.org/gsea/msigdb/cards/DEMAGALHAES_AGING_DN 15 0.39805 1.20607 0.222222 0.681119 0 up vs 1ZHENG_IL22_SIGNALING_DN http://www.broadinstitute.org/gsea/msigdb/cards/ZHENG_IL22_SIGNALING_DN 41 0.355265 1.21744 0.176471 0.681343 0 up vs 1MIKKELSEN_IPS_WITH_HCP_H3K27ME3 http://www.broadinstitute.org/gsea/msigdb/cards/MIKKELSEN_IPS_WITH_HCP_H3K27ME3 101 0.33131 1.20685 0.1875 0.681547 0 up vs 1ZHANG_BREAST_CANCER_PROGENITORS_DN http://www.broadinstitute.org/gsea/msigdb/cards/ZHANG_BREAST_CANCER_PROGENITORS_DN 141 0.299201 1.20689 0.229885 0.682812 0 up vs 1GRAHAM_CML_QUIESCENT_VS_CML_DIVIDING_UP http://www.broadinstitute.org/gsea/msigdb/cards/GRAHAM_CML_QUIESCENT_VS_CML_DIVIDING_UP 23 -0.279383 -0.947678 0.5 0.682912 0 down vs 1RAHMAN_TP53_TARGETS_PHOSPHORYLATED http://www.broadinstitute.org/gsea/msigdb/cards/RAHMAN_TP53_TARGETS_PHOSPHORYLATED 21 0.453786 1.20729 0.278481 0.683102 0 up vs 1RICKMAN_HEAD_AND_NECK_CANCER_F http://www.broadinstitute.org/gsea/msigdb/cards/RICKMAN_HEAD_AND_NECK_CANCER_F 53 0.361661 1.20294 0.181818 0.683288 0 up vs 1LEE_NEURAL_CREST_STEM_CELL_DN http://www.broadinstitute.org/gsea/msigdb/cards/LEE_NEURAL_CREST_STEM_CELL_DN 115 0.314463 1.20753 0.213333 0.683651 0 up vs 1MCBRYAN_PUBERTAL_BREAST_6_7WK_UP http://www.broadinstitute.org/gsea/msigdb/cards/MCBRYAN_PUBERTAL_BREAST_6_7WK_UP 191 0.323541 1.20316 0.25974 0.683985 0 up vs 1DAWSON_METHYLATED_IN_LYMPHOMA_TCL1 http://www.broadinstitute.org/gsea/msigdb/cards/DAWSON_METHYLATED_IN_LYMPHOMA_TCL1 57 0.33168 1.21167 0.181818 0.684254 0 up vs 1GAUSSMANN_MLL_AF4_FUSION_TARGETS_C_DN http://www.broadinstitute.org/gsea/msigdb/cards/GAUSSMANN_MLL_AF4_FUSION_TARGETS_C_DN 19 0.371638 1.19999 0.179104 0.684457 0 up vs 1HOELZEL_NF1_TARGETS_UP http://www.broadinstitute.org/gsea/msigdb/cards/HOELZEL_NF1_TARGETS_UP 134 0.322455 1.20168 0.186667 0.684618 0 up vs 1MOHANKUMAR_TLX1_TARGETS_UP http://www.broadinstitute.org/gsea/msigdb/cards/MOHANKUMAR_TLX1_TARGETS_UP 404 0.316299 1.20039 0.247191 0.684634 0 up vs 1PAPASPYRIDONOS_UNSTABLE_ATEROSCLEROTIC_PLAQUE_DN http://www.broadinstitute.org/gsea/msigdb/cards/PAPASPYRIDONOS_UNSTABLE_ATEROSCLEROTIC_PLAQUE_DN 42 0.427845 1.21529 0.275362 0.684873 0 up vs 1DORSAM_HOXA9_TARGETS_UP http://www.broadinstitute.org/gsea/msigdb/cards/DORSAM_HOXA9_TARGETS_UP 35 0.379932 1.20754 0.225 0.684927 0 up vs 1GRADE_COLON_AND_RECTAL_CANCER_UP http://www.broadinstitute.org/gsea/msigdb/cards/GRADE_COLON_AND_RECTAL_CANCER_UP 276 0.351148 1.21185 0.227273 0.68498 0 up vs 1NIKOLSKY_BREAST_CANCER_20Q11_AMPLICON http://www.broadinstitute.org/gsea/msigdb/cards/NIKOLSKY_BREAST_CANCER_20Q11_AMPLICON 29 0.475774 1.21088 0.276923 0.685229 0 up vs 1MEDINA_SMARCA4_TARGETS http://www.broadinstitute.org/gsea/msigdb/cards/MEDINA_SMARCA4_TARGETS 41 0.339734 1.19932 0.243243 0.685277 0 up vs 1MOREAUX_MULTIPLE_MYELOMA_BY_TACI_DN http://www.broadinstitute.org/gsea/msigdb/cards/MOREAUX_MULTIPLE_MYELOMA_BY_TACI_DN 165 0.375194 1.20785 0.27381 0.685304 0 up vs 1CHNG_MULTIPLE_MYELOMA_HYPERPLOID_DN http://www.broadinstitute.org/gsea/msigdb/cards/CHNG_MULTIPLE_MYELOMA_HYPERPLOID_DN 28 0.402652 1.2006 0.253165 0.685343 0 up vs 1KYNG_NORMAL_AGING_UP http://www.broadinstitute.org/gsea/msigdb/cards/KYNG_NORMAL_AGING_UP 18 -0.281762 -0.945715 0.538462 0.685418 0 down vs 1YAO_HOXA10_TARGETS_VIA_PROGESTERONE_DN http://www.broadinstitute.org/gsea/msigdb/cards/YAO_HOXA10_TARGETS_VIA_PROGESTERONE_DN 17 0.4086 1.21028 0.186441 0.685549 0 up vs 1TSENG_IRS1_TARGETS_UP http://www.broadinstitute.org/gsea/msigdb/cards/TSENG_IRS1_TARGETS_UP 110 0.316925 1.2088 0.195402 0.685657 0 up vs 1SCHLOSSER_SERUM_RESPONSE_AUGMENTED_BY_MYC http://www.broadinstitute.org/gsea/msigdb/cards/SCHLOSSER_SERUM_RESPONSE_AUGMENTED_BY_MYC 102 0.317205 1.20924 0.2 0.685665 0 up vs 1LI_CISPLATIN_RESISTANCE_UP http://www.broadinstitute.org/gsea/msigdb/cards/LI_CISPLATIN_RESISTANCE_UP 27 0.348543 1.20826 0.197183 0.685682 0 up vs 1JAZAG_TGFB1_SIGNALING_VIA_SMAD4_UP http://www.broadinstitute.org/gsea/msigdb/cards/JAZAG_TGFB1_SIGNALING_VIA_SMAD4_UP 108 0.29016 1.19832 0.233333 0.685714 0 up vs 1RAO_BOUND_BY_SALL4 http://www.broadinstitute.org/gsea/msigdb/cards/RAO_BOUND_BY_SALL4 221 0.3135 1.20967 0.235294 0.68577 0 up vs 1PHESSE_TARGETS_OF_APC_AND_MBD2_UP http://www.broadinstitute.org/gsea/msigdb/cards/PHESSE_TARGETS_OF_APC_AND_MBD2_UP 20 0.356694 1.20169 0.228571 0.685897 0 up vs 1HOQUE_METHYLATED_IN_CANCER http://www.broadinstitute.org/gsea/msigdb/cards/HOQUE_METHYLATED_IN_CANCER 56 0.308857 1.20083 0.273973 0.686007 0 up vs 1BOYAULT_LIVER_CANCER_SUBCLASS_G123_DN http://www.broadinstitute.org/gsea/msigdb/cards/BOYAULT_LIVER_CANCER_SUBCLASS_G123_DN 51 0.326155 1.21195 0.207317 0.686137 0 up vs 1MARTORIATI_MDM4_TARGETS_NEUROEPITHELIUM_UP http://www.broadinstitute.org/gsea/msigdb/cards/MARTORIATI_MDM4_TARGETS_NEUROEPITHELIUM_UP 173 0.287985 1.1986 0.247191 0.68628 0 up vs 1BYSTRYKH_HEMATOPOIESIS_STEM_CELL_AND_BRAIN_QTL_TRANS http://www.broadinstitute.org/gsea/msigdb/cards/BYSTRYKH_HEMATOPOIESIS_STEM_CELL_AND_BRAIN_QTL_TRANS 182 0.303397 1.21399 0.202247 0.686993 0 up vs 1VALK_AML_CLUSTER_10 http://www.broadinstitute.org/gsea/msigdb/cards/VALK_AML_CLUSTER_10 32 0.359307 1.21197 0.211268 0.687479 0 up vs 1LUI_TARGETS_OF_PAX8_PPARG_FUSION http://www.broadinstitute.org/gsea/msigdb/cards/LUI_TARGETS_OF_PAX8_PPARG_FUSION 34 0.416277 1.21242 0.271605 0.68749 0 up vs 1COATES_MACROPHAGE_M1_VS_M2_UP http://www.broadinstitute.org/gsea/msigdb/cards/COATES_MACROPHAGE_M1_VS_M2_UP 78 0.33161 1.2134 0.208333 0.687513 0 up vs 1LIU_TOPBP1_TARGETS http://www.broadinstitute.org/gsea/msigdb/cards/LIU_TOPBP1_TARGETS 16 0.36767 1.19436 0.2 0.687805 0 up vs 1VETTER_TARGETS_OF_PRKCA_AND_ETS1_UP http://www.broadinstitute.org/gsea/msigdb/cards/VETTER_TARGETS_OF_PRKCA_AND_ETS1_UP 15 0.43134 1.19448 0.319444 0.688751 0 up vs 1CARDOSO_RESPONSE_TO_GAMMA_RADIATION_AND_3AB http://www.broadinstitute.org/gsea/msigdb/cards/CARDOSO_RESPONSE_TO_GAMMA_RADIATION_AND_3AB 17 0.386266 1.21244 0.182927 0.688857 0 up vs 1FINETTI_BREAST_CANCER_KINOME_RED http://www.broadinstitute.org/gsea/msigdb/cards/FINETTI_BREAST_CANCER_KINOME_RED 16 -0.436625 -0.943007 0.666667 0.689416 0 down vs 1JI_CARCINOGENESIS_BY_KRAS_AND_STK11_DN http://www.broadinstitute.org/gsea/msigdb/cards/JI_CARCINOGENESIS_BY_KRAS_AND_STK11_DN 17 0.460715 1.19147 0.258065 0.689614 0 up vs 1DELASERNA_MYOD_TARGETS_UP http://www.broadinstitute.org/gsea/msigdb/cards/DELASERNA_MYOD_TARGETS_UP 86 0.312358 1.19319 0.25 0.689824 0 up vs 1MIKKELSEN_NPC_HCP_WITH_H3K4ME3_AND_H3K27ME3 http://www.broadinstitute.org/gsea/msigdb/cards/MIKKELSEN_NPC_HCP_WITH_H3K4ME3_AND_H3K27ME3 204 0.294752 1.19449 0.272727 0.690022 0 up vs 1CHANGOLKAR_H2AFY_TARGETS_UP http://www.broadinstitute.org/gsea/msigdb/cards/CHANGOLKAR_H2AFY_TARGETS_UP 46 0.337274 1.19273 0.176471 0.690038 0 up vs 1CHIANG_LIVER_CANCER_SUBCLASS_UNANNOTATED_DN http://www.broadinstitute.org/gsea/msigdb/cards/CHIANG_LIVER_CANCER_SUBCLASS_UNANNOTATED_DN 189 0.356701 1.19169 0.202532 0.690296 0 up vs 1DELYS_THYROID_CANCER_DN http://www.broadinstitute.org/gsea/msigdb/cards/DELYS_THYROID_CANCER_DN 227 0.319005 1.19482 0.246575 0.690483 0 up vs 1WAGNER_APO2_SENSITIVITY http://www.broadinstitute.org/gsea/msigdb/cards/WAGNER_APO2_SENSITIVITY 22 0.372972 1.19201 0.211268 0.690646 0 up vs 1BEGUM_TARGETS_OF_PAX3_FOXO1_FUSION_UP http://www.broadinstitute.org/gsea/msigdb/cards/BEGUM_TARGETS_OF_PAX3_FOXO1_FUSION_UP 60 0.375407 1.19617 0.253521 0.690763 0 up vs 1CROONQUIST_NRAS_SIGNALING_UP http://www.broadinstitute.org/gsea/msigdb/cards/CROONQUIST_NRAS_SIGNALING_UP 41 -0.317481 -0.941697 0.527778 0.69081 0 down vs 1CREIGHTON_AKT1_SIGNALING_VIA_MTOR_UP http://www.broadinstitute.org/gsea/msigdb/cards/CREIGHTON_AKT1_SIGNALING_VIA_MTOR_UP 34 0.388445 1.18948 0.230769 0.691153 0 up vs 1GUTIERREZ_MULTIPLE_MYELOMA_UP http://www.broadinstitute.org/gsea/msigdb/cards/GUTIERREZ_MULTIPLE_MYELOMA_UP 33 0.406559 1.19547 0.236842 0.691318 0 up vs 1DAVICIONI_PAX_FOXO1_SIGNATURE_IN_ARMS_UP http://www.broadinstitute.org/gsea/msigdb/cards/DAVICIONI_PAX_FOXO1_SIGNATURE_IN_ARMS_UP 56 0.335682 1.19033 0.22973 0.691565 0 up vs 1OUILLETTE_CLL_13Q14_DELETION_DN http://www.broadinstitute.org/gsea/msigdb/cards/OUILLETTE_CLL_13Q14_DELETION_DN 58 0.317454 1.18896 0.2375 0.691572 0 up vs 1ABE_INNER_EAR http://www.broadinstitute.org/gsea/msigdb/cards/ABE_INNER_EAR 48 0.365006 1.19486 0.26087 0.691685 0 up vs 1LEI_HOXC8_TARGETS_DN http://www.broadinstitute.org/gsea/msigdb/cards/LEI_HOXC8_TARGETS_DN 17 -0.33807 -0.940482 0.65625 0.692001 0 down vs 1KIM_MYCN_AMPLIFICATION_TARGETS_UP http://www.broadinstitute.org/gsea/msigdb/cards/KIM_MYCN_AMPLIFICATION_TARGETS_UP 90 0.323137 1.18965 0.197368 0.692046 0 up vs 1DUTERTRE_ESTRADIOL_RESPONSE_24HR_DN http://www.broadinstitute.org/gsea/msigdb/cards/DUTERTRE_ESTRADIOL_RESPONSE_24HR_DN 496 -0.231983 -0.935459 0.526316 0.692217 0 down vs 1YIH_RESPONSE_TO_ARSENITE_C1 http://www.broadinstitute.org/gsea/msigdb/cards/YIH_RESPONSE_TO_ARSENITE_C1 23 -0.319811 -0.935935 0.608696 0.692302 0 down vs 1ADDYA_ERYTHROID_DIFFERENTIATION_BY_HEMIN http://www.broadinstitute.org/gsea/msigdb/cards/ADDYA_ERYTHROID_DIFFERENTIATION_BY_HEMIN 72 -0.213756 -0.939893 0.571429 0.692333 0 down vs 1LIU_TARGETS_OF_VMYB_VS_CMYB_DN http://www.broadinstitute.org/gsea/msigdb/cards/LIU_TARGETS_OF_VMYB_VS_CMYB_DN 42 -0.276692 -0.939245 0.607143 0.692446 0 down vs 1KYNG_ENVIRONMENTAL_STRESS_RESPONSE_DN http://www.broadinstitute.org/gsea/msigdb/cards/KYNG_ENVIRONMENTAL_STRESS_RESPONSE_DN 19 -0.284179 -0.935957 0.5 0.693098 0 down vs 1TAKEDA_TARGETS_OF_NUP98_HOXA9_FUSION_16D_DN http://www.broadinstitute.org/gsea/msigdb/cards/TAKEDA_TARGETS_OF_NUP98_HOXA9_FUSION_16D_DN 132 -0.199029 -0.933741 0.607143 0.693383 0 down vs 1HE_PTEN_TARGETS_UP http://www.broadinstitute.org/gsea/msigdb/cards/HE_PTEN_TARGETS_UP 16 -0.291317 -0.938288 0.636364 0.693392 0 down vs 1TARTE_PLASMA_CELL_VS_PLASMABLAST_UP http://www.broadinstitute.org/gsea/msigdb/cards/TARTE_PLASMA_CELL_VS_PLASMABLAST_UP 386 -0.190425 -0.936297 0.588235 0.69346 0 down vs 1LEE_LIVER_CANCER_CIPROFIBRATE_UP http://www.broadinstitute.org/gsea/msigdb/cards/LEE_LIVER_CANCER_CIPROFIBRATE_UP 59 -0.225783 -0.934013 0.555556 0.693803 0 down vs 1BROWNE_HCMV_INFECTION_30MIN_UP http://www.broadinstitute.org/gsea/msigdb/cards/BROWNE_HCMV_INFECTION_30MIN_UP 55 -0.208195 -0.931004 0.518519 0.693852 0 down vs 1FRASOR_TAMOXIFEN_RESPONSE_UP http://www.broadinstitute.org/gsea/msigdb/cards/FRASOR_TAMOXIFEN_RESPONSE_UP 51 -0.232739 -0.936478 0.578947 0.69416 0 down vs 1ALONSO_METASTASIS_DN http://www.broadinstitute.org/gsea/msigdb/cards/ALONSO_METASTASIS_DN 24 -0.264914 -0.936897 0.6 0.694169 0 down vs 1SHEPARD_BMYB_TARGETS http://www.broadinstitute.org/gsea/msigdb/cards/SHEPARD_BMYB_TARGETS 70 -0.244053 -0.932264 0.583333 0.69417 0 down vs 1AMIT_EGF_RESPONSE_240_MCF10A http://www.broadinstitute.org/gsea/msigdb/cards/AMIT_EGF_RESPONSE_240_MCF10A 20 -0.279125 -0.937325 0.53125 0.694217 0 down vs 1AIYAR_COBRA1_TARGETS_DN http://www.broadinstitute.org/gsea/msigdb/cards/AIYAR_COBRA1_TARGETS_DN 28 -0.248382 -0.931174 0.565217 0.694387 0 down vs 1JEON_SMAD6_TARGETS_UP http://www.broadinstitute.org/gsea/msigdb/cards/JEON_SMAD6_TARGETS_UP 24 -0.333525 -0.932336 0.558824 0.694884 0 down vs 1SENESE_HDAC2_TARGETS_UP http://www.broadinstitute.org/gsea/msigdb/cards/SENESE_HDAC2_TARGETS_UP 113 -0.266304 -0.931248 0.5 0.695178 0 down vs 1SHEDDEN_LUNG_CANCER_GOOD_SURVIVAL_A4 http://www.broadinstitute.org/gsea/msigdb/cards/SHEDDEN_LUNG_CANCER_GOOD_SURVIVAL_A4 195 -0.194764 -0.928851 0.47619 0.696123 0 down vs 1JAEGER_METASTASIS_UP http://www.broadinstitute.org/gsea/msigdb/cards/JAEGER_METASTASIS_UP 44 0.377582 1.18217 0.214286 0.696494 0 up vs 1KOYAMA_SEMA3B_TARGETS_DN http://www.broadinstitute.org/gsea/msigdb/cards/KOYAMA_SEMA3B_TARGETS_DN 390 0.289534 1.18222 0.263736 0.697569 0 up vs 1LIN_NPAS4_TARGETS_UP http://www.broadinstitute.org/gsea/msigdb/cards/LIN_NPAS4_TARGETS_UP 153 0.306151 1.18596 0.294118 0.698323 0 up vs 1BILANGES_SERUM_SENSITIVE_VIA_TSC1 http://www.broadinstitute.org/gsea/msigdb/cards/BILANGES_SERUM_SENSITIVE_VIA_TSC1 23 0.356 1.18064 0.228571 0.698409 0 up vs 1ZHENG_GLIOBLASTOMA_PLASTICITY_DN http://www.broadinstitute.org/gsea/msigdb/cards/ZHENG_GLIOBLASTOMA_PLASTICITY_DN 57 0.342253 1.18325 0.228571 0.698515 0 up vs 1WAKABAYASHI_ADIPOGENESIS_PPARG_RXRA_BOUND_WITH_H4K20ME1_MARK http://www.broadinstitute.org/gsea/msigdb/cards/WAKABAYASHI_ADIPOGENESIS_PPARG_RXRA_BOUND_WITH_H4K20ME1_MARK 138 0.319882 1.18229 0.231707 0.698588 0 up vs 1NIKOLSKY_BREAST_CANCER_5P15_AMPLICON http://www.broadinstitute.org/gsea/msigdb/cards/NIKOLSKY_BREAST_CANCER_5P15_AMPLICON 26 -0.339534 -0.917328 0.538462 0.69886 0 down vs 1STEIN_ESRRA_TARGETS_UP http://www.broadinstitute.org/gsea/msigdb/cards/STEIN_ESRRA_TARGETS_UP 370 0.294882 1.18486 0.296703 0.698861 0 up vs 1MILI_PSEUDOPODIA http://www.broadinstitute.org/gsea/msigdb/cards/MILI_PSEUDOPODIA 41 0.355679 1.18357 0.268293 0.698902 0 up vs 1STARK_PREFRONTAL_CORTEX_22Q11_DELETION_UP http://www.broadinstitute.org/gsea/msigdb/cards/STARK_PREFRONTAL_CORTEX_22Q11_DELETION_UP 192 0.344716 1.18524 0.295775 0.698967 0 up vs 1CAFFAREL_RESPONSE_TO_THC_24HR_5_DN http://www.broadinstitute.org/gsea/msigdb/cards/CAFFAREL_RESPONSE_TO_THC_24HR_5_DN 58 -0.247548 -0.919807 0.578947 0.699193 0 down vs 1MOREAUX_B_LYMPHOCYTE_MATURATION_BY_TACI_UP http://www.broadinstitute.org/gsea/msigdb/cards/MOREAUX_B_LYMPHOCYTE_MATURATION_BY_TACI_UP 84 -0.189777 -0.925336 0.55 0.699219 0 down vs 1BONOME_OVARIAN_CANCER_POOR_SURVIVAL_UP http://www.broadinstitute.org/gsea/msigdb/cards/BONOME_OVARIAN_CANCER_POOR_SURVIVAL_UP 31 0.392268 1.18426 0.215385 0.699322 0 up vs 1MCMURRAY_TP53_HRAS_COOPERATION_RESPONSE_UP http://www.broadinstitute.org/gsea/msigdb/cards/MCMURRAY_TP53_HRAS_COOPERATION_RESPONSE_UP 26 0.357507 1.18073 0.191781 0.69941 0 up vs 1POMEROY_MEDULLOBLASTOMA_DESMOPLASIC_VS_CLASSIC_UP http://www.broadinstitute.org/gsea/msigdb/cards/POMEROY_MEDULLOBLASTOMA_DESMOPLASIC_VS_CLASSIC_UP 61 0.29298 1.18243 0.283951 0.699433 0 up vs 1FOSTER_TOLERANT_MACROPHAGE_UP http://www.broadinstitute.org/gsea/msigdb/cards/FOSTER_TOLERANT_MACROPHAGE_UP 148 -0.211224 -0.920159 0.590909 0.699437 0 down vs 1GROSS_HYPOXIA_VIA_ELK3_ONLY_UP http://www.broadinstitute.org/gsea/msigdb/cards/GROSS_HYPOXIA_VIA_ELK3_ONLY_UP 33 -0.275056 -0.926596 0.5 0.699458 0 down vs 1LIU_SMARCA4_TARGETS http://www.broadinstitute.org/gsea/msigdb/cards/LIU_SMARCA4_TARGETS 62 -0.227561 -0.917454 0.5 0.699577 0 down vs 1PEPPER_CHRONIC_LYMPHOCYTIC_LEUKEMIA_DN http://www.broadinstitute.org/gsea/msigdb/cards/PEPPER_CHRONIC_LYMPHOCYTIC_LEUKEMIA_DN 21 0.341577 1.18371 0.222222 0.699693 0 up vs 1MCBRYAN_PUBERTAL_BREAST_3_4WK_DN http://www.broadinstitute.org/gsea/msigdb/cards/MCBRYAN_PUBERTAL_BREAST_3_4WK_DN 37 0.308783 1.17933 0.246914 0.699827 0 up vs 1STAMBOLSKY_RESPONSE_TO_VITAMIN_D3_DN http://www.broadinstitute.org/gsea/msigdb/cards/STAMBOLSKY_RESPONSE_TO_VITAMIN_D3_DN 25 -0.287808 -0.925389 0.615385 0.700049 0 down vs 1AFFAR_YY1_TARGETS_DN http://www.broadinstitute.org/gsea/msigdb/cards/AFFAR_YY1_TARGETS_DN 233 -0.225671 -0.92022 0.583333 0.700219 0 down vs 1NAKAJIMA_MAST_CELL http://www.broadinstitute.org/gsea/msigdb/cards/NAKAJIMA_MAST_CELL 46 -0.239593 -0.925717 0.451613 0.700298 0 down vs 1OLSSON_E2F3_TARGETS_UP http://www.broadinstitute.org/gsea/msigdb/cards/OLSSON_E2F3_TARGETS_UP 28 -0.279225 -0.917468 0.653846 0.700429 0 down vs 1CHYLA_CBFA2T3_TARGETS_UP http://www.broadinstitute.org/gsea/msigdb/cards/CHYLA_CBFA2T3_TARGETS_UP 381 -0.196213 -0.917713 0.444444 0.70075 0 down vs 1MENSE_HYPOXIA_UP http://www.broadinstitute.org/gsea/msigdb/cards/MENSE_HYPOXIA_UP 97 -0.222805 -0.921897 0.466667 0.70086 0 down vs 1BOYAULT_LIVER_CANCER_SUBCLASS_G12_UP http://www.broadinstitute.org/gsea/msigdb/cards/BOYAULT_LIVER_CANCER_SUBCLASS_G12_UP 38 0.389285 1.17937 0.239437 0.700973 0 up vs 1KYNG_ENVIRONMENTAL_STRESS_RESPONSE_NOT_BY_GAMMA_IN_WS http://www.broadinstitute.org/gsea/msigdb/cards/KYNG_ENVIRONMENTAL_STRESS_RESPONSE_NOT_BY_GAMMA_IN_WS 32 -0.218439 -0.918398 0.590909 0.700993 0 down vs 1CHIANG_LIVER_CANCER_SUBCLASS_PROLIFERATION_UP http://www.broadinstitute.org/gsea/msigdb/cards/CHIANG_LIVER_CANCER_SUBCLASS_PROLIFERATION_UP 171 -0.253012 -0.920237 0.47619 0.701041 0 down vs 1TAVAZOIE_METASTASIS http://www.broadinstitute.org/gsea/msigdb/cards/TAVAZOIE_METASTASIS 101 0.310238 1.17803 0.257143 0.701061 0 up vs 1CHARAFE_BREAST_CANCER_LUMINAL_VS_MESENCHYMAL_UP http://www.broadinstitute.org/gsea/msigdb/cards/CHARAFE_BREAST_CANCER_LUMINAL_VS_MESENCHYMAL_UP 435 -0.225157 -0.920735 0.55 0.701134 0 down vs 1BAE_BRCA1_TARGETS_UP http://www.broadinstitute.org/gsea/msigdb/cards/BAE_BRCA1_TARGETS_UP 73 0.321346 1.17365 0.290323 0.701326 0 up vs 1GROSS_ELK3_TARGETS_DN http://www.broadinstitute.org/gsea/msigdb/cards/GROSS_ELK3_TARGETS_DN 32 -0.260708 -0.915243 0.6 0.701329 0 down vs 1ZHANG_BREAST_CANCER_PROGENITORS_UP http://www.broadinstitute.org/gsea/msigdb/cards/ZHANG_BREAST_CANCER_PROGENITORS_UP 411 0.358872 1.17497 0.270588 0.701342 0 up vs 1NIKOLSKY_BREAST_CANCER_17Q11_Q21_AMPLICON http://www.broadinstitute.org/gsea/msigdb/cards/NIKOLSKY_BREAST_CANCER_17Q11_Q21_AMPLICON 131 0.339258 1.1784 0.257143 0.701387 0 up vs 1NAGASHIMA_NRG1_SIGNALING_DN http://www.broadinstitute.org/gsea/msigdb/cards/NAGASHIMA_NRG1_SIGNALING_DN 55 -0.249261 -0.923598 0.5 0.701469 0 down vs 1LE_SKI_TARGETS_UP http://www.broadinstitute.org/gsea/msigdb/cards/LE_SKI_TARGETS_UP 17 -0.292895 -0.921054 0.470588 0.701478 0 down vs 1HERNANDEZ_ABERRANT_MITOSIS_BY_DOCETACEL_2NM_UP http://www.broadinstitute.org/gsea/msigdb/cards/HERNANDEZ_ABERRANT_MITOSIS_BY_DOCETACEL_2NM_UP 78 -0.225864 -0.917762 0.565217 0.70151 0 down vs 1RUTELLA_RESPONSE_TO_HGF_UP http://www.broadinstitute.org/gsea/msigdb/cards/RUTELLA_RESPONSE_TO_HGF_UP 404 -0.204854 -0.922987 0.52381 0.701606 0 down vs 1YAO_TEMPORAL_RESPONSE_TO_PROGESTERONE_CLUSTER_5 http://www.broadinstitute.org/gsea/msigdb/cards/YAO_TEMPORAL_RESPONSE_TO_PROGESTERONE_CLUSTER_5 28 -0.250646 -0.921963 0.5 0.70161 0 down vs 1SARTIPY_NORMAL_AT_INSULIN_RESISTANCE_UP http://www.broadinstitute.org/gsea/msigdb/cards/SARTIPY_NORMAL_AT_INSULIN_RESISTANCE_UP 34 -0.265064 -0.922376 0.652174 0.70182 0 down vs 1MEISSNER_NPC_HCP_WITH_H3K4ME3_AND_H3K27ME3 http://www.broadinstitute.org/gsea/msigdb/cards/MEISSNER_NPC_HCP_WITH_H3K4ME3_AND_H3K27ME3 137 0.296001 1.17379 0.269231 0.702178 0 up vs 1CHIN_BREAST_CANCER_COPY_NUMBER_UP http://www.broadinstitute.org/gsea/msigdb/cards/CHIN_BREAST_CANCER_COPY_NUMBER_UP 26 0.392756 1.17509 0.231884 0.702256 0 up vs 1GARGALOVIC_RESPONSE_TO_OXIDIZED_PHOSPHOLIPIDS_GREEN_DN http://www.broadinstitute.org/gsea/msigdb/cards/GARGALOVIC_RESPONSE_TO_OXIDIZED_PHOSPHOLIPIDS_GREEN_DN 23 0.388898 1.17415 0.233766 0.702343 0 up vs 1GINESTIER_BREAST_CANCER_ZNF217_AMPLIFIED_DN http://www.broadinstitute.org/gsea/msigdb/cards/GINESTIER_BREAST_CANCER_ZNF217_AMPLIFIED_DN 318 0.320673 1.17228 0.253165 0.702885 0 up vs 1ZHAN_MULTIPLE_MYELOMA_CD1_UP http://www.broadinstitute.org/gsea/msigdb/cards/ZHAN_MULTIPLE_MYELOMA_CD1_UP 44 0.333482 1.17511 0.239437 0.703477 0 up vs 1MATZUK_SPERMATOCYTE http://www.broadinstitute.org/gsea/msigdb/cards/MATZUK_SPERMATOCYTE 71 -0.225762 -0.913478 0.611111 0.703528 0 down vs 1RODRIGUES_NTN1_TARGETS_DN http://www.broadinstitute.org/gsea/msigdb/cards/RODRIGUES_NTN1_TARGETS_DN 153 -0.22922 -0.912447 0.608696 0.703583 0 down vs 1MCCLUNG_CREB1_TARGETS_DN http://www.broadinstitute.org/gsea/msigdb/cards/MCCLUNG_CREB1_TARGETS_DN 56 0.323215 1.1754 0.289474 0.703877 0 up vs 1SHAFFER_IRF4_MULTIPLE_MYELOMA_PROGRAM http://www.broadinstitute.org/gsea/msigdb/cards/SHAFFER_IRF4_MULTIPLE_MYELOMA_PROGRAM 36 0.343261 1.17235 0.264706 0.703893 0 up vs 1DAVICIONI_PAX_FOXO1_SIGNATURE_IN_ARMS_DN http://www.broadinstitute.org/gsea/msigdb/cards/DAVICIONI_PAX_FOXO1_SIGNATURE_IN_ARMS_DN 20 0.370139 1.1761 0.213115 0.704319 0 up vs 1CHEMNITZ_RESPONSE_TO_PROSTAGLANDIN_E2_UP http://www.broadinstitute.org/gsea/msigdb/cards/CHEMNITZ_RESPONSE_TO_PROSTAGLANDIN_E2_UP 140 -0.262182 -0.912539 0.631579 0.704359 0 down vs 1MIKKELSEN_IPS_ICP_WITH_H3K27ME3 http://www.broadinstitute.org/gsea/msigdb/cards/MIKKELSEN_IPS_ICP_WITH_H3K27ME3 53 0.32724 1.17556 0.222222 0.704641 0 up vs 1VANDESLUIS_COMMD1_TARGETS_GROUP_4_DN http://www.broadinstitute.org/gsea/msigdb/cards/VANDESLUIS_COMMD1_TARGETS_GROUP_4_DN 15 0.406572 1.17641 0.163934 0.70465 0 up vs 1LIAN_LIPA_TARGETS_3M http://www.broadinstitute.org/gsea/msigdb/cards/LIAN_LIPA_TARGETS_3M 58 -0.324156 -0.909917 0.555556 0.705993 0 down vs 1APPEL_IMATINIB_RESPONSE http://www.broadinstitute.org/gsea/msigdb/cards/APPEL_IMATINIB_RESPONSE 32 -0.328937 -0.910092 0.526316 0.706601 0 down vs 1IWANAGA_E2F1_TARGETS_INDUCED_BY_SERUM http://www.broadinstitute.org/gsea/msigdb/cards/IWANAGA_E2F1_TARGETS_INDUCED_BY_SERUM 30 -0.296221 -0.909088 0.6 0.706724 0 down vs 1PEART_HDAC_PROLIFERATION_CLUSTER_UP http://www.broadinstitute.org/gsea/msigdb/cards/PEART_HDAC_PROLIFERATION_CLUSTER_UP 56 -0.251269 -0.908195 0.578947 0.706907 0 down vs 1HOFFMANN_PRE_BI_TO_LARGE_PRE_BII_LYMPHOCYTE_DN http://www.broadinstitute.org/gsea/msigdb/cards/HOFFMANN_PRE_BI_TO_LARGE_PRE_BII_LYMPHOCYTE_DN 74 0.317425 1.16592 0.210526 0.709086 0 up vs 1LEE_CALORIE_RESTRICTION_NEOCORTEX_DN http://www.broadinstitute.org/gsea/msigdb/cards/LEE_CALORIE_RESTRICTION_NEOCORTEX_DN 88 0.305553 1.16628 0.267442 0.709217 0 up vs 1LE_NEURONAL_DIFFERENTIATION_UP http://www.broadinstitute.org/gsea/msigdb/cards/LE_NEURONAL_DIFFERENTIATION_UP 17 0.402245 1.16658 0.262295 0.709701 0 up vs 1PRAMOONJAGO_SOX4_TARGETS_DN http://www.broadinstitute.org/gsea/msigdb/cards/PRAMOONJAGO_SOX4_TARGETS_DN 50 0.353528 1.16418 0.231707 0.710035 0 up vs 1KYNG_WERNER_SYNDROM_AND_NORMAL_AGING_UP http://www.broadinstitute.org/gsea/msigdb/cards/KYNG_WERNER_SYNDROM_AND_NORMAL_AGING_UP 90 0.313283 1.16678 0.2875 0.710327 0 up vs 1PARK_HSC_VS_MULTIPOTENT_PROGENITORS_DN http://www.broadinstitute.org/gsea/msigdb/cards/PARK_HSC_VS_MULTIPOTENT_PROGENITORS_DN 18 0.386272 1.16921 0.276316 0.710768 0 up vs 1VISALA_RESPONSE_TO_HEAT_SHOCK_AND_AGING_UP http://www.broadinstitute.org/gsea/msigdb/cards/VISALA_RESPONSE_TO_HEAT_SHOCK_AND_AGING_UP 15 0.399009 1.16477 0.219178 0.710943 0 up vs 1MEISSNER_NPC_HCP_WITH_H3K4ME2_AND_H3K27ME3 http://www.broadinstitute.org/gsea/msigdb/cards/MEISSNER_NPC_HCP_WITH_H3K4ME2_AND_H3K27ME3 341 0.291701 1.16427 0.283784 0.710977 0 up vs 1PURBEY_TARGETS_OF_CTBP1_AND_SATB1_DN http://www.broadinstitute.org/gsea/msigdb/cards/PURBEY_TARGETS_OF_CTBP1_AND_SATB1_DN 179 0.29615 1.16699 0.302326 0.711082 0 up vs 1CROONQUIST_NRAS_VS_STROMAL_STIMULATION_UP http://www.broadinstitute.org/gsea/msigdb/cards/CROONQUIST_NRAS_VS_STROMAL_STIMULATION_UP 41 -0.265631 -0.904332 0.558824 0.71138 0 down vs 1JIANG_HYPOXIA_NORMAL http://www.broadinstitute.org/gsea/msigdb/cards/JIANG_HYPOXIA_NORMAL 299 0.291359 1.168 0.271739 0.711831 0 up vs 1PANGAS_TUMOR_SUPPRESSION_BY_SMAD1_AND_SMAD5_DN http://www.broadinstitute.org/gsea/msigdb/cards/PANGAS_TUMOR_SUPPRESSION_BY_SMAD1_AND_SMAD5_DN 152 0.303173 1.16758 0.25641 0.711864 0 up vs 1MALONEY_RESPONSE_TO_17AAG_DN http://www.broadinstitute.org/gsea/msigdb/cards/MALONEY_RESPONSE_TO_17AAG_DN 78 0.397821 1.16263 0.253165 0.711899 0 up vs 1MARZEC_IL2_SIGNALING_DN http://www.broadinstitute.org/gsea/msigdb/cards/MARZEC_IL2_SIGNALING_DN 36 0.348676 1.1683 0.256757 0.712011 0 up vs 1LUND_SILENCED_BY_METHYLATION http://www.broadinstitute.org/gsea/msigdb/cards/LUND_SILENCED_BY_METHYLATION 16 -0.277869 -0.902228 0.6875 0.712067 0 down vs 1GARGALOVIC_RESPONSE_TO_OXIDIZED_PHOSPHOLIPIDS_RED_UP http://www.broadinstitute.org/gsea/msigdb/cards/GARGALOVIC_RESPONSE_TO_OXIDIZED_PHOSPHOLIPIDS_RED_UP 17 -0.285877 -0.903537 0.592593 0.7121 0 down vs 1MEISSNER_NPC_ICP_WITH_H3K4ME3 http://www.broadinstitute.org/gsea/msigdb/cards/MEISSNER_NPC_ICP_WITH_H3K4ME3 17 -0.261629 -0.904368 0.535714 0.712178 0 down vs 1ROESSLER_LIVER_CANCER_METASTASIS_DN http://www.broadinstitute.org/gsea/msigdb/cards/ROESSLER_LIVER_CANCER_METASTASIS_DN 49 0.334741 1.16703 0.2875 0.712208 0 up vs 1MAGRANGEAS_MULTIPLE_MYELOMA_IGLL_VS_IGLK_UP http://www.broadinstitute.org/gsea/msigdb/cards/MAGRANGEAS_MULTIPLE_MYELOMA_IGLL_VS_IGLK_UP 41 -0.234715 -0.902822 0.607143 0.712482 0 down vs 1DAIRKEE_CANCER_PRONE_RESPONSE_BPA http://www.broadinstitute.org/gsea/msigdb/cards/DAIRKEE_CANCER_PRONE_RESPONSE_BPA 51 0.331493 1.16193 0.306818 0.712722 0 up vs 1POS_HISTAMINE_RESPONSE_NETWORK http://www.broadinstitute.org/gsea/msigdb/cards/POS_HISTAMINE_RESPONSE_NETWORK 32 -0.250454 -0.902343 0.653846 0.712788 0 down vs 1JIANG_AGING_HYPOTHALAMUS_UP http://www.broadinstitute.org/gsea/msigdb/cards/JIANG_AGING_HYPOTHALAMUS_UP 46 0.348811 1.16273 0.219512 0.712828 0 up vs 1LANDIS_ERBB2_BREAST_PRENEOPLASTIC_UP http://www.broadinstitute.org/gsea/msigdb/cards/LANDIS_ERBB2_BREAST_PRENEOPLASTIC_UP 19 -0.284631 -0.900302 0.607143 0.714498 0 down vs 1GUTIERREZ_CHRONIC_LYMPHOCYTIC_LEUKEMIA_DN http://www.broadinstitute.org/gsea/msigdb/cards/GUTIERREZ_CHRONIC_LYMPHOCYTIC_LEUKEMIA_DN 56 -0.241122 -0.897072 0.64 0.717356 0 down vs 1YORDY_RECIPROCAL_REGULATION_BY_ETS1_AND_SP100_DN http://www.broadinstitute.org/gsea/msigdb/cards/YORDY_RECIPROCAL_REGULATION_BY_ETS1_AND_SP100_DN 82 0.29541 1.15716 0.283951 0.717543 0 up vs 1GAZDA_DIAMOND_BLACKFAN_ANEMIA_ERYTHROID_UP http://www.broadinstitute.org/gsea/msigdb/cards/GAZDA_DIAMOND_BLACKFAN_ANEMIA_ERYTHROID_UP 24 -0.25751 -0.898214 0.608696 0.71765 0 down vs 1BROWNE_HCMV_INFECTION_6HR_UP http://www.broadinstitute.org/gsea/msigdb/cards/BROWNE_HCMV_INFECTION_6HR_UP 70 -0.195149 -0.895731 0.655172 0.717804 0 down vs 1WHITE_NEUROBLASTOMA_WITH_1P36.3_DELETION http://www.broadinstitute.org/gsea/msigdb/cards/WHITE_NEUROBLASTOMA_WITH_1P36.3_DELETION 21 -0.305151 -0.897091 0.516129 0.718191 0 down vs 1KINSEY_TARGETS_OF_EWSR1_FLII_FUSION_DN http://www.broadinstitute.org/gsea/msigdb/cards/KINSEY_TARGETS_OF_EWSR1_FLII_FUSION_DN 316 0.317925 1.15732 0.315789 0.718386 0 up vs 1DUNNE_TARGETS_OF_AML1_MTG8_FUSION_UP http://www.broadinstitute.org/gsea/msigdb/cards/DUNNE_TARGETS_OF_AML1_MTG8_FUSION_UP 51 -0.292206 -0.89575 0.53125 0.718637 0 down vs 1PENG_GLUTAMINE_DEPRIVATION_UP http://www.broadinstitute.org/gsea/msigdb/cards/PENG_GLUTAMINE_DEPRIVATION_UP 38 -0.246312 -0.89721 0.576923 0.718804 0 down vs 1RHEIN_ALL_GLUCOCORTICOID_THERAPY_DN http://www.broadinstitute.org/gsea/msigdb/cards/RHEIN_ALL_GLUCOCORTICOID_THERAPY_DN 355 0.338976 1.15624 0.255814 0.718907 0 up vs 1TARTE_PLASMA_CELL_VS_B_LYMPHOCYTE_UP http://www.broadinstitute.org/gsea/msigdb/cards/TARTE_PLASMA_CELL_VS_B_LYMPHOCYTE_UP 76 0.33814 1.15873 0.279412 0.719132 0 up vs 1SHIPP_DLBCL_CURED_VS_FATAL_UP http://www.broadinstitute.org/gsea/msigdb/cards/SHIPP_DLBCL_CURED_VS_FATAL_UP 39 0.311971 1.15733 0.267606 0.719576 0 up vs 1TANG_SENESCENCE_TP53_TARGETS_UP http://www.broadinstitute.org/gsea/msigdb/cards/TANG_SENESCENCE_TP53_TARGETS_UP 33 -0.227695 -0.894267 0.548387 0.719579 0 down vs 1HOOI_ST7_TARGETS_UP http://www.broadinstitute.org/gsea/msigdb/cards/HOOI_ST7_TARGETS_UP 86 0.30784 1.15894 0.285714 0.719833 0 up vs 1RIGGI_EWING_SARCOMA_PROGENITOR_UP http://www.broadinstitute.org/gsea/msigdb/cards/RIGGI_EWING_SARCOMA_PROGENITOR_UP 416 0.289931 1.1545 0.341463 0.720247 0 up vs 1IWANAGA_CARCINOGENESIS_BY_KRAS_UP http://www.broadinstitute.org/gsea/msigdb/cards/IWANAGA_CARCINOGENESIS_BY_KRAS_UP 163 0.287221 1.15262 0.241379 0.720465 0 up vs 1FERNANDEZ_BOUND_BY_MYC http://www.broadinstitute.org/gsea/msigdb/cards/FERNANDEZ_BOUND_BY_MYC 181 0.292675 1.1574 0.310345 0.720564 0 up vs 1BONOME_OVARIAN_CANCER_SURVIVAL_OPTIMAL_DEBULKING http://www.broadinstitute.org/gsea/msigdb/cards/BONOME_OVARIAN_CANCER_SURVIVAL_OPTIMAL_DEBULKING 232 0.297731 1.15522 0.291139 0.720581 0 up vs 1JUBAN_TARGETS_OF_SPI1_AND_FLI1_DN http://www.broadinstitute.org/gsea/msigdb/cards/JUBAN_TARGETS_OF_SPI1_AND_FLI1_DN 90 0.331206 1.15466 0.270588 0.721021 0 up vs 1BROWNE_HCMV_INFECTION_48HR_DN http://www.broadinstitute.org/gsea/msigdb/cards/BROWNE_HCMV_INFECTION_48HR_DN 493 0.290972 1.15364 0.329114 0.721203 0 up vs 1ICHIBA_GRAFT_VERSUS_HOST_DISEASE_35D_DN http://www.broadinstitute.org/gsea/msigdb/cards/ICHIBA_GRAFT_VERSUS_HOST_DISEASE_35D_DN 49 0.320071 1.15275 0.246154 0.721234 0 up vs 1URS_ADIPOCYTE_DIFFERENTIATION_UP http://www.broadinstitute.org/gsea/msigdb/cards/URS_ADIPOCYTE_DIFFERENTIATION_UP 74 0.302241 1.15749 0.269231 0.721499 0 up vs 1DAVICIONI_MOLECULAR_ARMS_VS_ERMS_UP http://www.broadinstitute.org/gsea/msigdb/cards/DAVICIONI_MOLECULAR_ARMS_VS_ERMS_UP 320 0.30442 1.15123 0.305882 0.722239 0 up vs 1TERAO_AOX4_TARGETS_SKIN_UP http://www.broadinstitute.org/gsea/msigdb/cards/TERAO_AOX4_TARGETS_SKIN_UP 37 0.337011 1.15277 0.261905 0.722346 0 up vs 1BLUM_RESPONSE_TO_SALIRASIB_DN http://www.broadinstitute.org/gsea/msigdb/cards/BLUM_RESPONSE_TO_SALIRASIB_DN 340 -0.246156 -0.888604 0.444444 0.722592 0 down vs 1DE_YY1_TARGETS_DN http://www.broadinstitute.org/gsea/msigdb/cards/DE_YY1_TARGETS_DN 90 -0.248546 -0.890948 0.608696 0.722781 0 down vs 1TURASHVILI_BREAST_DUCTAL_CARCINOMA_VS_LOBULAR_NORMAL_UP http://www.broadinstitute.org/gsea/msigdb/cards/TURASHVILI_BREAST_DUCTAL_CARCINOMA_VS_LOBULAR_NORMAL_UP 73 -0.231143 -0.891375 0.678571 0.723081 0 down vs 1JEON_SMAD6_TARGETS_DN http://www.broadinstitute.org/gsea/msigdb/cards/JEON_SMAD6_TARGETS_DN 19 -0.277028 -0.8898 0.555556 0.723083 0 down vs 1GRAHAM_CML_QUIESCENT_VS_NORMAL_QUIESCENT_DN http://www.broadinstitute.org/gsea/msigdb/cards/GRAHAM_CML_QUIESCENT_VS_NORMAL_QUIESCENT_DN 46 -0.256862 -0.88879 0.545455 0.723201 0 down vs 1MARCHINI_TRABECTEDIN_RESISTANCE_UP http://www.broadinstitute.org/gsea/msigdb/cards/MARCHINI_TRABECTEDIN_RESISTANCE_UP 20 -0.265585 -0.891647 0.633333 0.7234 0 down vs 1BALDWIN_PRKCI_TARGETS_UP http://www.broadinstitute.org/gsea/msigdb/cards/BALDWIN_PRKCI_TARGETS_UP 33 0.355045 1.15123 0.266667 0.723427 0 up vs 1OXFORD_RALA_OR_RALB_TARGETS_UP http://www.broadinstitute.org/gsea/msigdb/cards/OXFORD_RALA_OR_RALB_TARGETS_UP 48 -0.286641 -0.890088 0.653846 0.723512 0 down vs 1LEE_LIVER_CANCER_MYC_E2F1_DN http://www.broadinstitute.org/gsea/msigdb/cards/LEE_LIVER_CANCER_MYC_E2F1_DN 62 -0.192043 -0.888939 0.615385 0.723812 0 down vs 1ZHANG_GATA6_TARGETS_UP http://www.broadinstitute.org/gsea/msigdb/cards/ZHANG_GATA6_TARGETS_UP 15 0.361227 1.14943 0.228571 0.72513 0 up vs 1VANTVEER_BREAST_CANCER_POOR_PROGNOSIS http://www.broadinstitute.org/gsea/msigdb/cards/VANTVEER_BREAST_CANCER_POOR_PROGNOSIS 51 -0.219076 -0.886482 0.666667 0.725203 0 down vs 1LANDIS_ERBB2_BREAST_TUMORS_65_DN http://www.broadinstitute.org/gsea/msigdb/cards/LANDIS_ERBB2_BREAST_TUMORS_65_DN 36 0.340263 1.14965 0.271605 0.725619 0 up vs 1CHIANG_LIVER_CANCER_SUBCLASS_INTERFERON_UP http://www.broadinstitute.org/gsea/msigdb/cards/CHIANG_LIVER_CANCER_SUBCLASS_INTERFERON_UP 26 -0.304501 -0.885272 0.604651 0.72609 0 down vs 1KIM_GERMINAL_CENTER_T_HELPER_UP http://www.broadinstitute.org/gsea/msigdb/cards/KIM_GERMINAL_CENTER_T_HELPER_UP 63 0.368737 1.14826 0.306667 0.727528 0 up vs 1GREGORY_SYNTHETIC_LETHAL_WITH_IMATINIB http://www.broadinstitute.org/gsea/msigdb/cards/GREGORY_SYNTHETIC_LETHAL_WITH_IMATINIB 143 0.282786 1.14592 0.333333 0.728247 0 up vs 1KAYO_AGING_MUSCLE_UP http://www.broadinstitute.org/gsea/msigdb/cards/KAYO_AGING_MUSCLE_UP 242 0.2756 1.14749 0.375 0.728525 0 up vs 1WANG_LSD1_TARGETS_DN http://www.broadinstitute.org/gsea/msigdb/cards/WANG_LSD1_TARGETS_DN 39 0.325305 1.14619 0.283582 0.728656 0 up vs 1TAYLOR_METHYLATED_IN_ACUTE_LYMPHOBLASTIC_LEUKEMIA http://www.broadinstitute.org/gsea/msigdb/cards/TAYLOR_METHYLATED_IN_ACUTE_LYMPHOBLASTIC_LEUKEMIA 76 0.32403 1.14632 0.275 0.729494 0 up vs 1KUROKAWA_LIVER_CANCER_CHEMOTHERAPY_DN http://www.broadinstitute.org/gsea/msigdb/cards/KUROKAWA_LIVER_CANCER_CHEMOTHERAPY_DN 41 0.319231 1.14419 0.296296 0.72953 0 up vs 1WAMUNYOKOLI_OVARIAN_CANCER_GRADES_1_2_DN http://www.broadinstitute.org/gsea/msigdb/cards/WAMUNYOKOLI_OVARIAN_CANCER_GRADES_1_2_DN 64 0.336371 1.14455 0.297297 0.729979 0 up vs 1LUI_THYROID_CANCER_CLUSTER_1 http://www.broadinstitute.org/gsea/msigdb/cards/LUI_THYROID_CANCER_CLUSTER_1 50 0.346965 1.14653 0.235294 0.73008 0 up vs 1CROMER_METASTASIS_UP http://www.broadinstitute.org/gsea/msigdb/cards/CROMER_METASTASIS_UP 78 0.333907 1.1426 0.28 0.730168 0 up vs 1HOFFMANN_SMALL_PRE_BII_TO_IMMATURE_B_LYMPHOCYTE_DN http://www.broadinstitute.org/gsea/msigdb/cards/HOFFMANN_SMALL_PRE_BII_TO_IMMATURE_B_LYMPHOCYTE_DN 48 0.306729 1.14346 0.269231 0.730259 0 up vs 1YAO_TEMPORAL_RESPONSE_TO_PROGESTERONE_CLUSTER_8 http://www.broadinstitute.org/gsea/msigdb/cards/YAO_TEMPORAL_RESPONSE_TO_PROGESTERONE_CLUSTER_8 48 0.321493 1.14288 0.220779 0.730524 0 up vs 1BURTON_ADIPOGENESIS_9 http://www.broadinstitute.org/gsea/msigdb/cards/BURTON_ADIPOGENESIS_9 92 -0.198332 -0.877952 0.625 0.73064 0 down vs 1GUILLAUMOND_KLF10_TARGETS_UP http://www.broadinstitute.org/gsea/msigdb/cards/GUILLAUMOND_KLF10_TARGETS_UP 51 0.312325 1.14188 0.263158 0.730775 0 up vs 1FLOTHO_PEDIATRIC_ALL_THERAPY_RESPONSE_UP http://www.broadinstitute.org/gsea/msigdb/cards/FLOTHO_PEDIATRIC_ALL_THERAPY_RESPONSE_UP 53 0.383359 1.14462 0.226667 0.730961 0 up vs 1GROSS_HYPOXIA_VIA_HIF1A_DN http://www.broadinstitute.org/gsea/msigdb/cards/GROSS_HYPOXIA_VIA_HIF1A_DN 108 -0.191394 -0.878669 0.529412 0.731049 0 down vs 1HUMMERICH_MALIGNANT_SKIN_TUMOR_DN http://www.broadinstitute.org/gsea/msigdb/cards/HUMMERICH_MALIGNANT_SKIN_TUMOR_DN 18 -0.261567 -0.881448 0.580645 0.731049 0 down vs 1BENPORATH_PROLIFERATION http://www.broadinstitute.org/gsea/msigdb/cards/BENPORATH_PROLIFERATION 138 -0.290171 -0.879697 0.608696 0.731169 0 down vs 1MEISSNER_BRAIN_HCP_WITH_H3K4ME2_AND_H3K27ME3 http://www.broadinstitute.org/gsea/msigdb/cards/MEISSNER_BRAIN_HCP_WITH_H3K4ME2_AND_H3K27ME3 57 -0.208277 -0.880208 0.607143 0.73122 0 down vs 1DUNNE_TARGETS_OF_AML1_MTG8_FUSION_DN http://www.broadinstitute.org/gsea/msigdb/cards/DUNNE_TARGETS_OF_AML1_MTG8_FUSION_DN 19 -0.300288 -0.881851 0.675 0.731246 0 down vs 1WAMUNYOKOLI_OVARIAN_CANCER_LMP_DN http://www.broadinstitute.org/gsea/msigdb/cards/WAMUNYOKOLI_OVARIAN_CANCER_LMP_DN 195 0.339508 1.13674 0.302632 0.731419 0 up vs 1WILLIAMS_ESR1_TARGETS_UP http://www.broadinstitute.org/gsea/msigdb/cards/WILLIAMS_ESR1_TARGETS_UP 26 -0.243393 -0.877954 0.666667 0.731487 0 down vs 1GAUSSMANN_MLL_AF4_FUSION_TARGETS_G_UP http://www.broadinstitute.org/gsea/msigdb/cards/GAUSSMANN_MLL_AF4_FUSION_TARGETS_G_UP 229 0.281412 1.13422 0.3375 0.73154 0 up vs 1MEISSNER_NPC_HCP_WITH_H3K27ME3 http://www.broadinstitute.org/gsea/msigdb/cards/MEISSNER_NPC_HCP_WITH_H3K27ME3 79 0.305608 1.13714 0.309859 0.731627 0 up vs 1SCIBETTA_KDM5B_TARGETS_DN http://www.broadinstitute.org/gsea/msigdb/cards/SCIBETTA_KDM5B_TARGETS_DN 79 -0.250862 -0.878744 0.625 0.731807 0 down vs 1DELACROIX_RAR_BOUND_ES http://www.broadinstitute.org/gsea/msigdb/cards/DELACROIX_RAR_BOUND_ES 444 0.274694 1.1374 0.356322 0.731965 0 up vs 1YAO_TEMPORAL_RESPONSE_TO_PROGESTERONE_CLUSTER_0 http://www.broadinstitute.org/gsea/msigdb/cards/YAO_TEMPORAL_RESPONSE_TO_PROGESTERONE_CLUSTER_0 73 -0.224721 -0.880212 0.565217 0.732072 0 down vs 1MIKKELSEN_MCV6_HCP_WITH_H3K27ME3 http://www.broadinstitute.org/gsea/msigdb/cards/MIKKELSEN_MCV6_HCP_WITH_H3K27ME3 424 0.288709 1.14058 0.305556 0.732309 0 up vs 1MOOTHA_VOXPHOS http://www.broadinstitute.org/gsea/msigdb/cards/MOOTHA_VOXPHOS 87 0.439657 1.13432 0.382353 0.732361 0 up vs 1DING_LUNG_CANCER_EXPRESSION_BY_COPY_NUMBER http://www.broadinstitute.org/gsea/msigdb/cards/DING_LUNG_CANCER_EXPRESSION_BY_COPY_NUMBER 99 0.366711 1.13764 0.303797 0.732499 0 up vs 1STEIN_ESRRA_TARGETS_DN http://www.broadinstitute.org/gsea/msigdb/cards/STEIN_ESRRA_TARGETS_DN 102 0.306807 1.13837 0.325581 0.732752 0 up vs 1OZEN_MIR125B1_TARGETS http://www.broadinstitute.org/gsea/msigdb/cards/OZEN_MIR125B1_TARGETS 25 0.358706 1.13792 0.283784 0.732826 0 up vs 1SERVITJA_ISLET_HNF1A_TARGETS_DN http://www.broadinstitute.org/gsea/msigdb/cards/SERVITJA_ISLET_HNF1A_TARGETS_DN 109 0.317912 1.14076 0.319444 0.732877 0 up vs 1YAUCH_HEDGEHOG_SIGNALING_PARACRINE_DN http://www.broadinstitute.org/gsea/msigdb/cards/YAUCH_HEDGEHOG_SIGNALING_PARACRINE_DN 255 0.27876 1.13962 0.3125 0.732971 0 up vs 1KOBAYASHI_RESPONSE_TO_ROMIDEPSIN http://www.broadinstitute.org/gsea/msigdb/cards/KOBAYASHI_RESPONSE_TO_ROMIDEPSIN 19 0.347827 1.13869 0.266667 0.733055 0 up vs 1BAELDE_DIABETIC_NEPHROPATHY_UP http://www.broadinstitute.org/gsea/msigdb/cards/BAELDE_DIABETIC_NEPHROPATHY_UP 84 0.308656 1.13326 0.329114 0.733326 0 up vs 1LI_INDUCED_T_TO_NATURAL_KILLER_DN http://www.broadinstitute.org/gsea/msigdb/cards/LI_INDUCED_T_TO_NATURAL_KILLER_DN 115 0.30209 1.13433 0.333333 0.733505 0 up vs 1BREDEMEYER_RAG_SIGNALING_NOT_VIA_ATM_DN http://www.broadinstitute.org/gsea/msigdb/cards/BREDEMEYER_RAG_SIGNALING_NOT_VIA_ATM_DN 56 0.315763 1.13883 0.302632 0.733907 0 up vs 1LINDVALL_IMMORTALIZED_BY_TERT_UP http://www.broadinstitute.org/gsea/msigdb/cards/LINDVALL_IMMORTALIZED_BY_TERT_UP 74 0.322049 1.13963 0.285714 0.734074 0 up vs 1KYNG_ENVIRONMENTAL_STRESS_RESPONSE_NOT_BY_4NQO_IN_WS http://www.broadinstitute.org/gsea/msigdb/cards/KYNG_ENVIRONMENTAL_STRESS_RESPONSE_NOT_BY_4NQO_IN_WS 39 -0.213634 -0.875287 0.75 0.734311 0 down vs 1LEE_METASTASIS_AND_ALTERNATIVE_SPLICING_DN http://www.broadinstitute.org/gsea/msigdb/cards/LEE_METASTASIS_AND_ALTERNATIVE_SPLICING_DN 45 0.322029 1.13446 0.297297 0.734357 0 up vs 1MIKKELSEN_MEF_LCP_WITH_H3K27ME3 http://www.broadinstitute.org/gsea/msigdb/cards/MIKKELSEN_MEF_LCP_WITH_H3K27ME3 64 0.309017 1.1325 0.287879 0.734545 0 up vs 1MAHADEVAN_IMATINIB_RESISTANCE_UP http://www.broadinstitute.org/gsea/msigdb/cards/MAHADEVAN_IMATINIB_RESISTANCE_UP 22 0.359118 1.12891 0.276923 0.734969 0 up vs 1VALK_AML_CLUSTER_12 http://www.broadinstitute.org/gsea/msigdb/cards/VALK_AML_CLUSTER_12 30 0.338633 1.13457 0.28169 0.735251 0 up vs 1IKEDA_MIR1_TARGETS_UP http://www.broadinstitute.org/gsea/msigdb/cards/IKEDA_MIR1_TARGETS_UP 53 0.349807 1.12917 0.269231 0.735417 0 up vs 1SOTIRIOU_BREAST_CANCER_GRADE_1_VS_3_DN http://www.broadinstitute.org/gsea/msigdb/cards/SOTIRIOU_BREAST_CANCER_GRADE_1_VS_3_DN 50 0.384827 1.13177 0.3 0.735506 0 up vs 1ZHOU_INFLAMMATORY_RESPONSE_FIMA_DN http://www.broadinstitute.org/gsea/msigdb/cards/ZHOU_INFLAMMATORY_RESPONSE_FIMA_DN 269 0.287031 1.13143 0.35 0.735524 0 up vs 1WELCH_GATA1_TARGETS http://www.broadinstitute.org/gsea/msigdb/cards/WELCH_GATA1_TARGETS 21 0.341213 1.12953 0.289474 0.735537 0 up vs 1YAGI_AML_WITH_INV_16_TRANSLOCATION http://www.broadinstitute.org/gsea/msigdb/cards/YAGI_AML_WITH_INV_16_TRANSLOCATION 415 0.26796 1.13019 0.395604 0.735831 0 up vs 1MIKKELSEN_ES_HCP_WITH_H3_UNMETHYLATED http://www.broadinstitute.org/gsea/msigdb/cards/MIKKELSEN_ES_HCP_WITH_H3_UNMETHYLATED 63 0.28865 1.12979 0.315068 0.735848 0 up vs 1SHIPP_DLBCL_CURED_VS_FATAL_DN http://www.broadinstitute.org/gsea/msigdb/cards/SHIPP_DLBCL_CURED_VS_FATAL_DN 44 0.328966 1.12778 0.285714 0.735971 0 up vs 1TONKS_TARGETS_OF_RUNX1_RUNX1T1_FUSION_SUSTAINED_IN_GRANULOCYTE_UP http://www.broadinstitute.org/gsea/msigdb/cards/TONKS_TARGETS_OF_RUNX1_RUNX1T1_FUSION_SUSTAINED_IN_GRANULOCYTE_UP 15 -0.286759 -0.873661 0.676471 0.735988 0 down vs 1SCHRAMM_INHBA_TARGETS_DN http://www.broadinstitute.org/gsea/msigdb/cards/SCHRAMM_INHBA_TARGETS_DN 24 0.372299 1.1308 0.273973 0.736088 0 up vs 1ZHONG_RESPONSE_TO_AZACITIDINE_AND_TSA_DN http://www.broadinstitute.org/gsea/msigdb/cards/ZHONG_RESPONSE_TO_AZACITIDINE_AND_TSA_DN 67 0.342652 1.13036 0.26506 0.736219 0 up vs 1MCDOWELL_ACUTE_LUNG_INJURY_DN http://www.broadinstitute.org/gsea/msigdb/cards/MCDOWELL_ACUTE_LUNG_INJURY_DN 48 0.315487 1.13463 0.297297 0.736302 0 up vs 1YAMAZAKI_TCEB3_TARGETS_DN http://www.broadinstitute.org/gsea/msigdb/cards/YAMAZAKI_TCEB3_TARGETS_DN 211 0.293565 1.12788 0.337079 0.736833 0 up vs 1PASINI_SUZ12_TARGETS_DN http://www.broadinstitute.org/gsea/msigdb/cards/PASINI_SUZ12_TARGETS_DN 310 -0.21569 -0.871617 0.555556 0.73831 0 down vs 1DAZARD_RESPONSE_TO_UV_NHEK_DN http://www.broadinstitute.org/gsea/msigdb/cards/DAZARD_RESPONSE_TO_UV_NHEK_DN 308 -0.24699 -0.869009 0.5 0.741941 0 down vs 1NADERI_BREAST_CANCER_PROGNOSIS_UP http://www.broadinstitute.org/gsea/msigdb/cards/NADERI_BREAST_CANCER_PROGNOSIS_UP 47 -0.260434 -0.868371 0.653846 0.742256 0 down vs 1GAUSSMANN_MLL_AF4_FUSION_TARGETS_F_UP http://www.broadinstitute.org/gsea/msigdb/cards/GAUSSMANN_MLL_AF4_FUSION_TARGETS_F_UP 183 -0.195693 -0.866787 0.705882 0.744257 0 down vs 1WONG_IFNA2_RESISTANCE_UP http://www.broadinstitute.org/gsea/msigdb/cards/WONG_IFNA2_RESISTANCE_UP 16 -0.252339 -0.865894 0.717949 0.744874 0 down vs 1FIRESTEIN_PROLIFERATION http://www.broadinstitute.org/gsea/msigdb/cards/FIRESTEIN_PROLIFERATION 171 0.28598 1.12356 0.353659 0.745136 0 up vs 1MAHAJAN_RESPONSE_TO_IL1A_DN http://www.broadinstitute.org/gsea/msigdb/cards/MAHAJAN_RESPONSE_TO_IL1A_DN 74 0.31557 1.12364 0.337349 0.745996 0 up vs 1FIGUEROA_AML_METHYLATION_CLUSTER_4_UP http://www.broadinstitute.org/gsea/msigdb/cards/FIGUEROA_AML_METHYLATION_CLUSTER_4_UP 105 0.281445 1.11972 0.333333 0.749373 0 up vs 1BILBAN_B_CLL_LPL_UP http://www.broadinstitute.org/gsea/msigdb/cards/BILBAN_B_CLL_LPL_UP 61 0.311768 1.11925 0.324675 0.74948 0 up vs 1ALFANO_MYC_TARGETS http://www.broadinstitute.org/gsea/msigdb/cards/ALFANO_MYC_TARGETS 234 0.278723 1.12166 0.380435 0.749578 0 up vs 1DARWICHE_SQUAMOUS_CELL_CARCINOMA_DN http://www.broadinstitute.org/gsea/msigdb/cards/DARWICHE_SQUAMOUS_CELL_CARCINOMA_DN 175 0.27071 1.1212 0.384615 0.749674 0 up vs 1BERENJENO_TRANSFORMED_BY_RHOA_FOREVER_UP http://www.broadinstitute.org/gsea/msigdb/cards/BERENJENO_TRANSFORMED_BY_RHOA_FOREVER_UP 19 0.373498 1.1198 0.328125 0.750108 0 up vs 1KORKOLA_YOLK_SAC_TUMOR_UP http://www.broadinstitute.org/gsea/msigdb/cards/KORKOLA_YOLK_SAC_TUMOR_UP 19 0.448349 1.12015 0.348485 0.750361 0 up vs 1VERHAAK_GLIOBLASTOMA_CLASSICAL http://www.broadinstitute.org/gsea/msigdb/cards/VERHAAK_GLIOBLASTOMA_CLASSICAL 160 0.298886 1.12039 0.333333 0.750739 0 up vs 1SMID_BREAST_CANCER_RELAPSE_IN_BRAIN_UP http://www.broadinstitute.org/gsea/msigdb/cards/SMID_BREAST_CANCER_RELAPSE_IN_BRAIN_UP 39 -0.216165 -0.860433 0.678571 0.752096 0 down vs 1MIKKELSEN_ES_HCP_WITH_H3K27ME3 http://www.broadinstitute.org/gsea/msigdb/cards/MIKKELSEN_ES_HCP_WITH_H3K27ME3 41 -0.2276 -0.861024 0.75 0.752152 0 down vs 1PENG_LEUCINE_DEPRIVATION_UP http://www.broadinstitute.org/gsea/msigdb/cards/PENG_LEUCINE_DEPRIVATION_UP 140 0.289997 1.11395 0.329268 0.753374 0 up vs 1JAZAERI_BREAST_CANCER_BRCA1_VS_BRCA2_DN http://www.broadinstitute.org/gsea/msigdb/cards/JAZAERI_BREAST_CANCER_BRCA1_VS_BRCA2_DN 42 0.328594 1.11461 0.348485 0.753711 0 up vs 1TONKS_TARGETS_OF_RUNX1_RUNX1T1_FUSION_SUSTAINDED_IN_ERYTHROCYTE_UP http://www.broadinstitute.org/gsea/msigdb/cards/TONKS_TARGETS_OF_RUNX1_RUNX1T1_FUSION_SUSTAINDED_IN_ERYTHROCYTE_UP 44 0.308104 1.1148 0.328767 0.754247 0 up vs 1BROWNE_HCMV_INFECTION_1HR_UP http://www.broadinstitute.org/gsea/msigdb/cards/BROWNE_HCMV_INFECTION_1HR_UP 59 0.307123 1.114 0.324324 0.754395 0 up vs 1KIM_GASTRIC_CANCER_CHEMOSENSITIVITY http://www.broadinstitute.org/gsea/msigdb/cards/KIM_GASTRIC_CANCER_CHEMOSENSITIVITY 100 0.284328 1.11559 0.316456 0.754404 0 up vs 1BOYAULT_LIVER_CANCER_SUBCLASS_G3_UP http://www.broadinstitute.org/gsea/msigdb/cards/BOYAULT_LIVER_CANCER_SUBCLASS_G3_UP 184 0.351128 1.11583 0.317073 0.754673 0 up vs 1GENTILE_UV_RESPONSE_CLUSTER_D7 http://www.broadinstitute.org/gsea/msigdb/cards/GENTILE_UV_RESPONSE_CLUSTER_D7 39 -0.21827 -0.858234 0.727273 0.75481 0 down vs 1ZEMBUTSU_SENSITIVITY_TO_VINBLASTINE http://www.broadinstitute.org/gsea/msigdb/cards/ZEMBUTSU_SENSITIVITY_TO_VINBLASTINE 18 0.395813 1.11494 0.288136 0.755029 0 up vs 1SARTIPY_BLUNTED_BY_INSULIN_RESISTANCE_DN http://www.broadinstitute.org/gsea/msigdb/cards/SARTIPY_BLUNTED_BY_INSULIN_RESISTANCE_DN 18 -0.287715 -0.857319 0.821429 0.755197 0 down vs 1VANHARANTA_UTERINE_FIBROID_WITH_7Q_DELETION_UP http://www.broadinstitute.org/gsea/msigdb/cards/VANHARANTA_UTERINE_FIBROID_WITH_7Q_DELETION_UP 66 0.325763 1.11688 0.329412 0.755251 0 up vs 1SEIDEN_MET_SIGNALING http://www.broadinstitute.org/gsea/msigdb/cards/SEIDEN_MET_SIGNALING 18 0.440485 1.11593 0.384615 0.75557 0 up vs 1SESTO_RESPONSE_TO_UV_C8 http://www.broadinstitute.org/gsea/msigdb/cards/SESTO_RESPONSE_TO_UV_C8 71 -0.194133 -0.856426 0.809524 0.756243 0 down vs 1COLIN_PILOCYTIC_ASTROCYTOMA_VS_GLIOBLASTOMA_UP http://www.broadinstitute.org/gsea/msigdb/cards/COLIN_PILOCYTIC_ASTROCYTOMA_VS_GLIOBLASTOMA_UP 35 0.333281 1.1161 0.283582 0.756298 0 up vs 1TAKEDA_TARGETS_OF_NUP98_HOXA9_FUSION_8D_DN http://www.broadinstitute.org/gsea/msigdb/cards/TAKEDA_TARGETS_OF_NUP98_HOXA9_FUSION_8D_DN 199 -0.211266 -0.855402 0.678571 0.757394 0 down vs 1WEI_MIR34A_TARGETS http://www.broadinstitute.org/gsea/msigdb/cards/WEI_MIR34A_TARGETS 145 0.307027 1.10787 0.390244 0.75788 0 up vs 1SEITZ_NEOPLASTIC_TRANSFORMATION_BY_8P_DELETION_DN http://www.broadinstitute.org/gsea/msigdb/cards/SEITZ_NEOPLASTIC_TRANSFORMATION_BY_8P_DELETION_DN 30 0.320686 1.10748 0.32 0.758019 0 up vs 1WONG_EMBRYONIC_STEM_CELL_CORE http://www.broadinstitute.org/gsea/msigdb/cards/WONG_EMBRYONIC_STEM_CELL_CORE 333 0.36613 1.11107 0.3375 0.758047 0 up vs 1ZHAN_MULTIPLE_MYELOMA_LB_UP http://www.broadinstitute.org/gsea/msigdb/cards/ZHAN_MULTIPLE_MYELOMA_LB_UP 43 -0.212256 -0.853811 0.666667 0.758212 0 down vs 1BRACHAT_RESPONSE_TO_CAMPTOTHECIN_DN http://www.broadinstitute.org/gsea/msigdb/cards/BRACHAT_RESPONSE_TO_CAMPTOTHECIN_DN 44 0.316533 1.10621 0.325581 0.75828 0 up vs 1GAVIN_FOXP3_TARGETS_CLUSTER_P3 http://www.broadinstitute.org/gsea/msigdb/cards/GAVIN_FOXP3_TARGETS_CLUSTER_P3 158 -0.195604 -0.854116 0.666667 0.758613 0 down vs 1ACEVEDO_FGFR1_TARGETS_IN_PROSTATE_CANCER_MODEL_DN http://www.broadinstitute.org/gsea/msigdb/cards/ACEVEDO_FGFR1_TARGETS_IN_PROSTATE_CANCER_MODEL_DN 299 0.296512 1.10796 0.373333 0.758712 0 up vs 1BASSO_B_LYMPHOCYTE_NETWORK http://www.broadinstitute.org/gsea/msigdb/cards/BASSO_B_LYMPHOCYTE_NETWORK 140 0.312404 1.10647 0.341463 0.758774 0 up vs 1GARY_CD5_TARGETS_DN http://www.broadinstitute.org/gsea/msigdb/cards/GARY_CD5_TARGETS_DN 425 0.326232 1.11047 0.345238 0.758805 0 up vs 1BORCZUK_MALIGNANT_MESOTHELIOMA_UP http://www.broadinstitute.org/gsea/msigdb/cards/BORCZUK_MALIGNANT_MESOTHELIOMA_UP 297 0.335091 1.10678 0.349398 0.758994 0 up vs 1SPIRA_SMOKERS_LUNG_CANCER_UP http://www.broadinstitute.org/gsea/msigdb/cards/SPIRA_SMOKERS_LUNG_CANCER_UP 38 0.362662 1.11109 0.328767 0.759156 0 up vs 1AFFAR_YY1_TARGETS_UP http://www.broadinstitute.org/gsea/msigdb/cards/AFFAR_YY1_TARGETS_UP 211 0.272757 1.10853 0.392857 0.75942 0 up vs 1RASHI_RESPONSE_TO_IONIZING_RADIATION_3 http://www.broadinstitute.org/gsea/msigdb/cards/RASHI_RESPONSE_TO_IONIZING_RADIATION_3 47 0.320389 1.10806 0.358974 0.75957 0 up vs 1AGUIRRE_PANCREATIC_CANCER_COPY_NUMBER_UP http://www.broadinstitute.org/gsea/msigdb/cards/AGUIRRE_PANCREATIC_CANCER_COPY_NUMBER_UP 287 0.297886 1.11135 0.337209 0.759624 0 up vs 1YANG_BREAST_CANCER_ESR1_BULK_UP http://www.broadinstitute.org/gsea/msigdb/cards/YANG_BREAST_CANCER_ESR1_BULK_UP 27 0.370715 1.10973 0.337662 0.759717 0 up vs 1NIKOLSKY_BREAST_CANCER_8Q23_Q24_AMPLICON http://www.broadinstitute.org/gsea/msigdb/cards/NIKOLSKY_BREAST_CANCER_8Q23_Q24_AMPLICON 150 0.355301 1.10865 0.294118 0.760197 0 up vs 1PUJANA_BREAST_CANCER_LIT_INT_NETWORK http://www.broadinstitute.org/gsea/msigdb/cards/PUJANA_BREAST_CANCER_LIT_INT_NETWORK 100 -0.26298 -0.851892 0.6875 0.760563 0 down vs 1MASSARWEH_RESPONSE_TO_ESTRADIOL http://www.broadinstitute.org/gsea/msigdb/cards/MASSARWEH_RESPONSE_TO_ESTRADIOL 60 0.312318 1.10874 0.323944 0.761134 0 up vs 1KLEIN_PRIMARY_EFFUSION_LYMPHOMA_UP http://www.broadinstitute.org/gsea/msigdb/cards/KLEIN_PRIMARY_EFFUSION_LYMPHOMA_UP 50 0.29853 1.10374 0.310811 0.762521 0 up vs 1KORKOLA_EMBRYONAL_CARCINOMA_UP http://www.broadinstitute.org/gsea/msigdb/cards/KORKOLA_EMBRYONAL_CARCINOMA_UP 40 0.376206 1.1041 0.343284 0.762591 0 up vs 1GOUYER_TATI_TARGETS_DN http://www.broadinstitute.org/gsea/msigdb/cards/GOUYER_TATI_TARGETS_DN 16 0.363968 1.10331 0.285714 0.762723 0 up vs 1FLECHNER_PBL_KIDNEY_TRANSPLANT_REJECTED_VS_OK_UP http://www.broadinstitute.org/gsea/msigdb/cards/FLECHNER_PBL_KIDNEY_TRANSPLANT_REJECTED_VS_OK_UP 62 -0.22597 -0.850035 0.866667 0.762998 0 down vs 1SCHAEFFER_SOX9_TARGETS_IN_PROSTATE_DEVELOPMENT_DN http://www.broadinstitute.org/gsea/msigdb/cards/SCHAEFFER_SOX9_TARGETS_IN_PROSTATE_DEVELOPMENT_DN 44 0.35078 1.10269 0.314286 0.763386 0 up vs 1TORCHIA_TARGETS_OF_EWSR1_FLI1_FUSION_DN http://www.broadinstitute.org/gsea/msigdb/cards/TORCHIA_TARGETS_OF_EWSR1_FLI1_FUSION_DN 309 0.285258 1.10418 0.384615 0.763411 0 up vs 1KIM_PTEN_TARGETS_UP http://www.broadinstitute.org/gsea/msigdb/cards/KIM_PTEN_TARGETS_UP 18 -0.26323 -0.848949 0.611111 0.763851 0 down vs 1MARZEC_IL2_SIGNALING_UP http://www.broadinstitute.org/gsea/msigdb/cards/MARZEC_IL2_SIGNALING_UP 112 -0.202311 -0.848468 0.708333 0.763906 0 down vs 1LIEN_BREAST_CARCINOMA_METAPLASTIC http://www.broadinstitute.org/gsea/msigdb/cards/LIEN_BREAST_CARCINOMA_METAPLASTIC 35 0.385899 1.10089 0.285714 0.764301 0 up vs 1HEIDENBLAD_AMPLICON_12P11_12_DN http://www.broadinstitute.org/gsea/msigdb/cards/HEIDENBLAD_AMPLICON_12P11_12_DN 28 0.336359 1.10125 0.363636 0.764303 0 up vs 1SWEET_KRAS_TARGETS_UP http://www.broadinstitute.org/gsea/msigdb/cards/SWEET_KRAS_TARGETS_UP 82 -0.24735 -0.847518 0.64 0.764508 0 down vs 1HSIAO_LIVER_SPECIFIC_GENES http://www.broadinstitute.org/gsea/msigdb/cards/HSIAO_LIVER_SPECIFIC_GENES 242 0.294228 1.10149 0.375 0.764823 0 up vs 1MAHADEVAN_GIST_MORPHOLOGICAL_SWITCH http://www.broadinstitute.org/gsea/msigdb/cards/MAHADEVAN_GIST_MORPHOLOGICAL_SWITCH 15 -0.288642 -0.846837 0.611111 0.764845 0 down vs 1JIANG_VHL_TARGETS http://www.broadinstitute.org/gsea/msigdb/cards/JIANG_VHL_TARGETS 134 0.290071 1.10028 0.318681 0.764863 0 up vs 1CHYLA_CBFA2T3_TARGETS_DN http://www.broadinstitute.org/gsea/msigdb/cards/CHYLA_CBFA2T3_TARGETS_DN 231 0.272097 1.09935 0.426829 0.765337 0 up vs 1HOLLEMAN_PREDNISOLONE_RESISTANCE_ALL_UP http://www.broadinstitute.org/gsea/msigdb/cards/HOLLEMAN_PREDNISOLONE_RESISTANCE_ALL_UP 19 0.406922 1.10166 0.311688 0.765415 0 up vs 1BROWNE_HCMV_INFECTION_18HR_DN http://www.broadinstitute.org/gsea/msigdb/cards/BROWNE_HCMV_INFECTION_18HR_DN 174 0.294844 1.09969 0.351351 0.765446 0 up vs 1WATANABE_RECTAL_CANCER_RADIOTHERAPY_RESPONSIVE_DN http://www.broadinstitute.org/gsea/msigdb/cards/WATANABE_RECTAL_CANCER_RADIOTHERAPY_RESPONSIVE_DN 92 0.304606 1.09788 0.341176 0.766202 0 up vs 1NAKAYAMA_SOFT_TISSUE_TUMORS_PCA2_DN http://www.broadinstitute.org/gsea/msigdb/cards/NAKAYAMA_SOFT_TISSUE_TUMORS_PCA2_DN 78 0.301352 1.09826 0.324324 0.766346 0 up vs 1DAZARD_UV_RESPONSE_CLUSTER_G1 http://www.broadinstitute.org/gsea/msigdb/cards/DAZARD_UV_RESPONSE_CLUSTER_G1 65 0.304624 1.09604 0.373494 0.767273 0 up vs 1YAGI_AML_WITH_11Q23_REARRANGED http://www.broadinstitute.org/gsea/msigdb/cards/YAGI_AML_WITH_11Q23_REARRANGED 340 0.276272 1.09636 0.414634 0.76736 0 up vs 1PURBEY_TARGETS_OF_CTBP1_NOT_SATB1_DN http://www.broadinstitute.org/gsea/msigdb/cards/PURBEY_TARGETS_OF_CTBP1_NOT_SATB1_DN 428 0.263154 1.09829 0.436782 0.767367 0 up vs 1HOLLMANN_APOPTOSIS_VIA_CD40_DN http://www.broadinstitute.org/gsea/msigdb/cards/HOLLMANN_APOPTOSIS_VIA_CD40_DN 256 0.282962 1.0967 0.388235 0.767471 0 up vs 1VANTVEER_BREAST_CANCER_BRCA1_DN http://www.broadinstitute.org/gsea/msigdb/cards/VANTVEER_BREAST_CANCER_BRCA1_DN 42 0.328861 1.09506 0.342105 0.767689 0 up vs 1ONO_FOXP3_TARGETS_UP http://www.broadinstitute.org/gsea/msigdb/cards/ONO_FOXP3_TARGETS_UP 23 -0.297776 -0.844249 0.666667 0.767843 0 down vs 1LEE_AGING_MUSCLE_DN http://www.broadinstitute.org/gsea/msigdb/cards/LEE_AGING_MUSCLE_DN 46 0.312438 1.09459 0.346154 0.76804 0 up vs 1DURCHDEWALD_SKIN_CARCINOGENESIS_UP http://www.broadinstitute.org/gsea/msigdb/cards/DURCHDEWALD_SKIN_CARCINOGENESIS_UP 87 0.277152 1.09524 0.352273 0.768412 0 up vs 1LOPEZ_MBD_TARGETS_IMPRINTED_AND_X_LINKED http://www.broadinstitute.org/gsea/msigdb/cards/LOPEZ_MBD_TARGETS_IMPRINTED_AND_X_LINKED 16 0.384201 1.09675 0.323077 0.768524 0 up vs 1WANG_PROSTATE_CANCER_ANDROGEN_INDEPENDENT http://www.broadinstitute.org/gsea/msigdb/cards/WANG_PROSTATE_CANCER_ANDROGEN_INDEPENDENT 60 0.291701 1.09353 0.363636 0.769015 0 up vs 1LIU_TARGETS_OF_VMYB_VS_CMYB_UP http://www.broadinstitute.org/gsea/msigdb/cards/LIU_TARGETS_OF_VMYB_VS_CMYB_UP 17 0.354513 1.09378 0.295775 0.76953 0 up vs 1KONDO_COLON_CANCER_HCP_WITH_H3K27ME1 http://www.broadinstitute.org/gsea/msigdb/cards/KONDO_COLON_CANCER_HCP_WITH_H3K27ME1 25 0.330998 1.09228 0.338235 0.769847 0 up vs 1ABRAHAM_ALPC_VS_MULTIPLE_MYELOMA_DN http://www.broadinstitute.org/gsea/msigdb/cards/ABRAHAM_ALPC_VS_MULTIPLE_MYELOMA_DN 19 0.371314 1.09267 0.367089 0.769949 0 up vs 1ROZANOV_MMP14_TARGETS_SUBSET http://www.broadinstitute.org/gsea/msigdb/cards/ROZANOV_MMP14_TARGETS_SUBSET 33 0.403743 1.09154 0.292308 0.770651 0 up vs 1HOFFMANN_IMMATURE_TO_MATURE_B_LYMPHOCYTE_UP http://www.broadinstitute.org/gsea/msigdb/cards/HOFFMANN_IMMATURE_TO_MATURE_B_LYMPHOCYTE_UP 43 0.293987 1.08839 0.410256 0.772211 0 up vs 1MARTINEZ_RESPONSE_TO_TRABECTEDIN_UP http://www.broadinstitute.org/gsea/msigdb/cards/MARTINEZ_RESPONSE_TO_TRABECTEDIN_UP 70 0.293669 1.08791 0.390805 0.772368 0 up vs 1LI_CISPLATIN_RESISTANCE_DN http://www.broadinstitute.org/gsea/msigdb/cards/LI_CISPLATIN_RESISTANCE_DN 33 0.321124 1.08846 0.338462 0.773132 0 up vs 1LOCKWOOD_AMPLIFIED_IN_LUNG_CANCER http://www.broadinstitute.org/gsea/msigdb/cards/LOCKWOOD_AMPLIFIED_IN_LUNG_CANCER 210 0.291687 1.09026 0.378049 0.773305 0 up vs 1JIANG_TIP30_TARGETS_DN http://www.broadinstitute.org/gsea/msigdb/cards/JIANG_TIP30_TARGETS_DN 23 0.324866 1.08966 0.363636 0.773793 0 up vs 1HIRSCH_CELLULAR_TRANSFORMATION_SIGNATURE_DN http://www.broadinstitute.org/gsea/msigdb/cards/HIRSCH_CELLULAR_TRANSFORMATION_SIGNATURE_DN 99 0.303886 1.08888 0.369048 0.773832 0 up vs 1TURASHVILI_BREAST_DUCTAL_CARCINOMA_VS_LOBULAR_NORMAL_DN http://www.broadinstitute.org/gsea/msigdb/cards/TURASHVILI_BREAST_DUCTAL_CARCINOMA_VS_LOBULAR_NORMAL_DN 66 -0.221996 -0.838519 0.777778 0.77402 0 down vs 1BACOLOD_RESISTANCE_TO_ALKYLATING_AGENTS_UP http://www.broadinstitute.org/gsea/msigdb/cards/BACOLOD_RESISTANCE_TO_ALKYLATING_AGENTS_UP 26 0.321727 1.08916 0.319444 0.774029 0 up vs 1VALK_AML_WITH_11Q23_REARRANGED http://www.broadinstitute.org/gsea/msigdb/cards/VALK_AML_WITH_11Q23_REARRANGED 21 0.321653 1.0885 0.304348 0.774044 0 up vs 1JACKSON_DNMT1_TARGETS_DN http://www.broadinstitute.org/gsea/msigdb/cards/JACKSON_DNMT1_TARGETS_DN 25 -0.236473 -0.83964 0.692308 0.774092 0 down vs 1LI_WILMS_TUMOR_ANAPLASTIC_UP http://www.broadinstitute.org/gsea/msigdb/cards/LI_WILMS_TUMOR_ANAPLASTIC_UP 19 -0.35531 -0.838786 0.62069 0.774557 0 down vs 1HAHTOLA_MYCOSIS_FUNGOIDES_SKIN_UP http://www.broadinstitute.org/gsea/msigdb/cards/HAHTOLA_MYCOSIS_FUNGOIDES_SKIN_UP 175 0.285768 1.08664 0.366667 0.774651 0 up vs 1MIKKELSEN_IPS_LCP_WITH_H3K4ME3 http://www.broadinstitute.org/gsea/msigdb/cards/MIKKELSEN_IPS_LCP_WITH_H3K4ME3 166 -0.198961 -0.834746 0.818182 0.774678 0 down vs 1CROONQUIST_STROMAL_STIMULATION_UP http://www.broadinstitute.org/gsea/msigdb/cards/CROONQUIST_STROMAL_STIMULATION_UP 59 -0.257394 -0.831883 0.727273 0.774748 0 down vs 1MISHRA_CARCINOMA_ASSOCIATED_FIBROBLAST_UP http://www.broadinstitute.org/gsea/msigdb/cards/MISHRA_CARCINOMA_ASSOCIATED_FIBROBLAST_UP 24 -0.281854 -0.835564 0.675 0.775031 0 down vs 1ZEMBUTSU_SENSITIVITY_TO_CYCLOPHOSPHAMIDE http://www.broadinstitute.org/gsea/msigdb/cards/ZEMBUTSU_SENSITIVITY_TO_CYCLOPHOSPHAMIDE 16 -0.26054 -0.836218 0.666667 0.775147 0 down vs 1LIAN_LIPA_TARGETS_6M http://www.broadinstitute.org/gsea/msigdb/cards/LIAN_LIPA_TARGETS_6M 73 -0.277709 -0.834979 0.714286 0.775248 0 down vs 1HOFMANN_MYELODYSPLASTIC_SYNDROM_HIGH_RISK_DN http://www.broadinstitute.org/gsea/msigdb/cards/HOFMANN_MYELODYSPLASTIC_SYNDROM_HIGH_RISK_DN 20 -0.245368 -0.833197 0.638889 0.775441 0 down vs 1CERIBELLI_GENES_INACTIVE_AND_BOUND_BY_NFY http://www.broadinstitute.org/gsea/msigdb/cards/CERIBELLI_GENES_INACTIVE_AND_BOUND_BY_NFY 41 -0.219265 -0.831905 0.56 0.775575 0 down vs 1GARGALOVIC_RESPONSE_TO_OXIDIZED_PHOSPHOLIPIDS_BLUE_UP http://www.broadinstitute.org/gsea/msigdb/cards/GARGALOVIC_RESPONSE_TO_OXIDIZED_PHOSPHOLIPIDS_BLUE_UP 123 -0.195775 -0.836904 0.684211 0.775713 0 down vs 1GAVIN_PDE3B_TARGETS http://www.broadinstitute.org/gsea/msigdb/cards/GAVIN_PDE3B_TARGETS 22 -0.300994 -0.830857 0.682927 0.775726 0 down vs 1TSUNODA_CISPLATIN_RESISTANCE_UP http://www.broadinstitute.org/gsea/msigdb/cards/TSUNODA_CISPLATIN_RESISTANCE_UP 15 -0.288449 -0.836248 0.675676 0.775929 0 down vs 1RUAN_RESPONSE_TO_TROGLITAZONE_DN http://www.broadinstitute.org/gsea/msigdb/cards/RUAN_RESPONSE_TO_TROGLITAZONE_DN 19 -0.22331 -0.833363 0.772727 0.776028 0 down vs 1SAKAI_TUMOR_INFILTRATING_MONOCYTES_DN http://www.broadinstitute.org/gsea/msigdb/cards/SAKAI_TUMOR_INFILTRATING_MONOCYTES_DN 81 0.331554 1.08576 0.345679 0.776171 0 up vs 1LIEN_BREAST_CARCINOMA_METAPLASTIC_VS_DUCTAL_UP http://www.broadinstitute.org/gsea/msigdb/cards/LIEN_BREAST_CARCINOMA_METAPLASTIC_VS_DUCTAL_UP 79 -0.221202 -0.829568 0.703704 0.776188 0 down vs 1RIZ_ERYTHROID_DIFFERENTIATION_HEMGN http://www.broadinstitute.org/gsea/msigdb/cards/RIZ_ERYTHROID_DIFFERENTIATION_HEMGN 31 -0.22801 -0.832009 0.702703 0.776265 0 down vs 1LEIN_PONS_MARKERS http://www.broadinstitute.org/gsea/msigdb/cards/LEIN_PONS_MARKERS 86 0.29172 1.08539 0.380282 0.776322 0 up vs 1OUELLET_CULTURED_OVARIAN_CANCER_INVASIVE_VS_LMP_DN http://www.broadinstitute.org/gsea/msigdb/cards/OUELLET_CULTURED_OVARIAN_CANCER_INVASIVE_VS_LMP_DN 34 0.302215 1.08465 0.338028 0.77642 0 up vs 1CHEN_HOXA5_TARGETS_9HR_UP http://www.broadinstitute.org/gsea/msigdb/cards/CHEN_HOXA5_TARGETS_9HR_UP 217 -0.223184 -0.829703 0.583333 0.776702 0 down vs 1MIKKELSEN_NPC_LCP_WITH_H3K4ME3 http://www.broadinstitute.org/gsea/msigdb/cards/MIKKELSEN_NPC_LCP_WITH_H3K4ME3 56 0.311211 1.08385 0.364865 0.777435 0 up vs 1KIM_ALL_DISORDERS_OLIGODENDROCYTE_NUMBER_CORR_DN http://www.broadinstitute.org/gsea/msigdb/cards/KIM_ALL_DISORDERS_OLIGODENDROCYTE_NUMBER_CORR_DN 32 0.31672 1.08466 0.4 0.777497 0 up vs 1MARTINEZ_RESPONSE_TO_TRABECTEDIN http://www.broadinstitute.org/gsea/msigdb/cards/MARTINEZ_RESPONSE_TO_TRABECTEDIN 49 0.337166 1.08245 0.355263 0.77893 0 up vs 1BOCHKIS_FOXA2_TARGETS http://www.broadinstitute.org/gsea/msigdb/cards/BOCHKIS_FOXA2_TARGETS 415 0.265391 1.07887 0.465909 0.779556 0 up vs 1WEBER_METHYLATED_HCP_IN_SPERM_DN http://www.broadinstitute.org/gsea/msigdb/cards/WEBER_METHYLATED_HCP_IN_SPERM_DN 28 -0.240923 -0.826906 0.678571 0.779701 0 down vs 1SABATES_COLORECTAL_ADENOMA_DN http://www.broadinstitute.org/gsea/msigdb/cards/SABATES_COLORECTAL_ADENOMA_DN 278 0.290402 1.08257 0.376812 0.779703 0 up vs 1MORI_SMALL_PRE_BII_LYMPHOCYTE_UP http://www.broadinstitute.org/gsea/msigdb/cards/MORI_SMALL_PRE_BII_LYMPHOCYTE_UP 82 0.281936 1.07896 0.397436 0.780298 0 up vs 1SMID_BREAST_CANCER_RELAPSE_IN_LUNG_UP http://www.broadinstitute.org/gsea/msigdb/cards/SMID_BREAST_CANCER_RELAPSE_IN_LUNG_UP 20 0.333752 1.07675 0.352113 0.780313 0 up vs 1LU_TUMOR_ANGIOGENESIS_UP http://www.broadinstitute.org/gsea/msigdb/cards/LU_TUMOR_ANGIOGENESIS_UP 25 0.338028 1.07713 0.301587 0.78049 0 up vs 1MCBRYAN_PUBERTAL_BREAST_4_5WK_DN http://www.broadinstitute.org/gsea/msigdb/cards/MCBRYAN_PUBERTAL_BREAST_4_5WK_DN 189 0.271885 1.07805 0.435294 0.780892 0 up vs 1MA_PITUITARY_FETAL_VS_ADULT_UP http://www.broadinstitute.org/gsea/msigdb/cards/MA_PITUITARY_FETAL_VS_ADULT_UP 27 0.323995 1.0791 0.342105 0.78092 0 up vs 1SPIELMAN_LYMPHOBLAST_EUROPEAN_VS_ASIAN_UP http://www.broadinstitute.org/gsea/msigdb/cards/SPIELMAN_LYMPHOBLAST_EUROPEAN_VS_ASIAN_UP 471 0.285611 1.07946 0.366667 0.781111 0 up vs 1FARMER_BREAST_CANCER_CLUSTER_4 http://www.broadinstitute.org/gsea/msigdb/cards/FARMER_BREAST_CANCER_CLUSTER_4 19 0.522547 1.08085 0.42623 0.781277 0 up vs 1RAY_TARGETS_OF_P210_BCR_ABL_FUSION_UP http://www.broadinstitute.org/gsea/msigdb/cards/RAY_TARGETS_OF_P210_BCR_ABL_FUSION_UP 15 0.361353 1.07722 0.317647 0.781307 0 up vs 1HARRIS_BRAIN_CANCER_PROGENITORS http://www.broadinstitute.org/gsea/msigdb/cards/HARRIS_BRAIN_CANCER_PROGENITORS 43 0.293614 1.0798 0.391304 0.782151 0 up vs 1NIELSEN_SCHWANNOMA_DN http://www.broadinstitute.org/gsea/msigdb/cards/NIELSEN_SCHWANNOMA_DN 16 0.350884 1.07946 0.333333 0.78217 0 up vs 1MELLMAN_TUT1_TARGETS_DN http://www.broadinstitute.org/gsea/msigdb/cards/MELLMAN_TUT1_TARGETS_DN 47 0.300939 1.07727 0.394737 0.782181 0 up vs 1PAL_PRMT5_TARGETS_UP http://www.broadinstitute.org/gsea/msigdb/cards/PAL_PRMT5_TARGETS_UP 198 0.310175 1.0809 0.356322 0.782196 0 up vs 1KIM_WT1_TARGETS_DN http://www.broadinstitute.org/gsea/msigdb/cards/KIM_WT1_TARGETS_DN 440 0.292342 1.08014 0.406977 0.782445 0 up vs 1LEE_LIVER_CANCER http://www.broadinstitute.org/gsea/msigdb/cards/LEE_LIVER_CANCER 47 -0.219602 -0.822639 0.766667 0.783925 0 down vs 1KORKOLA_CORRELATED_WITH_POU5F1 http://www.broadinstitute.org/gsea/msigdb/cards/KORKOLA_CORRELATED_WITH_POU5F1 34 -0.211747 -0.821741 0.75 0.784369 0 down vs 1MCBRYAN_PUBERTAL_TGFB1_TARGETS_DN http://www.broadinstitute.org/gsea/msigdb/cards/MCBRYAN_PUBERTAL_TGFB1_TARGETS_DN 62 -0.199106 -0.822874 0.7 0.784532 0 down vs 1VANTVEER_BREAST_CANCER_METASTASIS_DN http://www.broadinstitute.org/gsea/msigdb/cards/VANTVEER_BREAST_CANCER_METASTASIS_DN 114 -0.222354 -0.823077 0.655172 0.785071 0 down vs 1WINNEPENNINCKX_MELANOMA_METASTASIS_UP http://www.broadinstitute.org/gsea/msigdb/cards/WINNEPENNINCKX_MELANOMA_METASTASIS_UP 156 -0.241022 -0.820511 0.666667 0.785328 0 down vs 1KHETCHOUMIAN_TRIM24_TARGETS_UP http://www.broadinstitute.org/gsea/msigdb/cards/KHETCHOUMIAN_TRIM24_TARGETS_UP 47 -0.251309 -0.819703 0.724138 0.785512 0 down vs 1HEDENFALK_BREAST_CANCER_BRCA1_VS_BRCA2 http://www.broadinstitute.org/gsea/msigdb/cards/HEDENFALK_BREAST_CANCER_BRCA1_VS_BRCA2 157 0.30683 1.07417 0.382022 0.787004 0 up vs 1YAO_TEMPORAL_RESPONSE_TO_PROGESTERONE_CLUSTER_4 http://www.broadinstitute.org/gsea/msigdb/cards/YAO_TEMPORAL_RESPONSE_TO_PROGESTERONE_CLUSTER_4 15 0.36468 1.072 0.358209 0.787836 0 up vs 1LEE_LIVER_CANCER_MYC_TGFA_DN http://www.broadinstitute.org/gsea/msigdb/cards/LEE_LIVER_CANCER_MYC_TGFA_DN 62 0.273587 1.07339 0.434211 0.787914 0 up vs 1SWEET_LUNG_CANCER_KRAS_UP http://www.broadinstitute.org/gsea/msigdb/cards/SWEET_LUNG_CANCER_KRAS_UP 486 0.25998 1.07242 0.428571 0.788656 0 up vs 1OUYANG_PROSTATE_CANCER_MARKERS http://www.broadinstitute.org/gsea/msigdb/cards/OUYANG_PROSTATE_CANCER_MARKERS 19 0.324459 1.06425 0.338235 0.788677 0 up vs 1HATADA_METHYLATED_IN_LUNG_CANCER_UP http://www.broadinstitute.org/gsea/msigdb/cards/HATADA_METHYLATED_IN_LUNG_CANCER_UP 377 0.266982 1.07203 0.487179 0.788797 0 up vs 1STAMBOLSKY_TARGETS_OF_MUTATED_TP53_UP http://www.broadinstitute.org/gsea/msigdb/cards/STAMBOLSKY_TARGETS_OF_MUTATED_TP53_UP 49 0.312311 1.07266 0.361111 0.789052 0 up vs 1SARTIPY_NORMAL_AT_INSULIN_RESISTANCE_DN http://www.broadinstitute.org/gsea/msigdb/cards/SARTIPY_NORMAL_AT_INSULIN_RESISTANCE_DN 20 0.352791 1.06379 0.378378 0.7891 0 up vs 1SANDERSON_PPARA_TARGETS http://www.broadinstitute.org/gsea/msigdb/cards/SANDERSON_PPARA_TARGETS 15 0.371313 1.06304 0.414286 0.789138 0 up vs 1BARRIER_CANCER_RELAPSE_NORMAL_SAMPLE_DN http://www.broadinstitute.org/gsea/msigdb/cards/BARRIER_CANCER_RELAPSE_NORMAL_SAMPLE_DN 29 0.333954 1.06434 0.402778 0.789455 0 up vs 1CREIGHTON_AKT1_SIGNALING_VIA_MTOR_DN http://www.broadinstitute.org/gsea/msigdb/cards/CREIGHTON_AKT1_SIGNALING_VIA_MTOR_DN 23 0.397766 1.06325 0.308824 0.789627 0 up vs 1HALMOS_CEBPA_TARGETS_DN http://www.broadinstitute.org/gsea/msigdb/cards/HALMOS_CEBPA_TARGETS_DN 46 0.309066 1.06449 0.375 0.79 0 up vs 1AMUNDSON_GAMMA_RADIATION_RESPONSE http://www.broadinstitute.org/gsea/msigdb/cards/AMUNDSON_GAMMA_RADIATION_RESPONSE 40 -0.319948 -0.816135 0.65625 0.790154 0 down vs 1MANTOVANI_VIRAL_GPCR_SIGNALING_UP http://www.broadinstitute.org/gsea/msigdb/cards/MANTOVANI_VIRAL_GPCR_SIGNALING_UP 85 0.267187 1.0705 0.445783 0.790237 0 up vs 1MATZUK_MALE_REPRODUCTION_SERTOLI http://www.broadinstitute.org/gsea/msigdb/cards/MATZUK_MALE_REPRODUCTION_SERTOLI 28 0.313881 1.06623 0.275362 0.79037 0 up vs 1GAUSSMANN_MLL_AF4_FUSION_TARGETS_C_UP http://www.broadinstitute.org/gsea/msigdb/cards/GAUSSMANN_MLL_AF4_FUSION_TARGETS_C_UP 167 0.280561 1.06689 0.451219 0.79056 0 up vs 1LEE_CALORIE_RESTRICTION_MUSCLE_DN http://www.broadinstitute.org/gsea/msigdb/cards/LEE_CALORIE_RESTRICTION_MUSCLE_DN 51 0.298071 1.06559 0.357143 0.790879 0 up vs 1BREDEMEYER_RAG_SIGNALING_NOT_VIA_ATM_UP http://www.broadinstitute.org/gsea/msigdb/cards/BREDEMEYER_RAG_SIGNALING_NOT_VIA_ATM_UP 60 0.29207 1.06456 0.432432 0.790909 0 up vs 1MATSUDA_NATURAL_KILLER_DIFFERENTIATION http://www.broadinstitute.org/gsea/msigdb/cards/MATSUDA_NATURAL_KILLER_DIFFERENTIATION 466 0.261306 1.07065 0.460674 0.790981 0 up vs 1FLECHNER_PBL_KIDNEY_TRANSPLANT_REJECTED_VS_OK_DN http://www.broadinstitute.org/gsea/msigdb/cards/FLECHNER_PBL_KIDNEY_TRANSPLANT_REJECTED_VS_OK_DN 51 0.293597 1.06707 0.367816 0.791144 0 up vs 1AKL_HTLV1_INFECTION_UP http://www.broadinstitute.org/gsea/msigdb/cards/AKL_HTLV1_INFECTION_UP 25 0.335342 1.06627 0.346154 0.791306 0 up vs 1ROSS_AML_WITH_AML1_ETO_FUSION http://www.broadinstitute.org/gsea/msigdb/cards/ROSS_AML_WITH_AML1_ETO_FUSION 74 0.297928 1.06195 0.385714 0.791512 0 up vs 1HILLION_HMGA1B_TARGETS http://www.broadinstitute.org/gsea/msigdb/cards/HILLION_HMGA1B_TARGETS 90 0.287551 1.06728 0.385542 0.791623 0 up vs 1BERTUCCI_INVASIVE_CARCINOMA_DUCTAL_VS_LOBULAR_UP http://www.broadinstitute.org/gsea/msigdb/cards/BERTUCCI_INVASIVE_CARCINOMA_DUCTAL_VS_LOBULAR_UP 24 0.3503 1.06961 0.391304 0.791677 0 up vs 1CUI_TCF21_TARGETS_DN http://www.broadinstitute.org/gsea/msigdb/cards/CUI_TCF21_TARGETS_DN 31 0.300606 1.06458 0.373134 0.791852 0 up vs 1GUENTHER_GROWTH_SPHERICAL_VS_ADHERENT_UP http://www.broadinstitute.org/gsea/msigdb/cards/GUENTHER_GROWTH_SPHERICAL_VS_ADHERENT_UP 21 0.34671 1.06485 0.263158 0.792093 0 up vs 1LOPEZ_EPITHELIOID_MESOTHELIOMA http://www.broadinstitute.org/gsea/msigdb/cards/LOPEZ_EPITHELIOID_MESOTHELIOMA 17 -0.253071 -0.81133 0.774194 0.79219 0 down vs 1WU_HBX_TARGETS_1_DN http://www.broadinstitute.org/gsea/msigdb/cards/WU_HBX_TARGETS_1_DN 23 0.338293 1.06745 0.430556 0.7922 0 up vs 1AMIT_EGF_RESPONSE_120_MCF10A http://www.broadinstitute.org/gsea/msigdb/cards/AMIT_EGF_RESPONSE_120_MCF10A 42 -0.246734 -0.811837 0.692308 0.792219 0 down vs 1ZHAN_MULTIPLE_MYELOMA_CD1_AND_CD2_UP http://www.broadinstitute.org/gsea/msigdb/cards/ZHAN_MULTIPLE_MYELOMA_CD1_AND_CD2_UP 86 0.312887 1.06098 0.392405 0.792333 0 up vs 1HUMMERICH_SKIN_CANCER_PROGRESSION_DN http://www.broadinstitute.org/gsea/msigdb/cards/HUMMERICH_SKIN_CANCER_PROGRESSION_DN 100 0.279972 1.05985 0.407407 0.79243 0 up vs 1COULOUARN_TEMPORAL_TGFB1_SIGNATURE_UP http://www.broadinstitute.org/gsea/msigdb/cards/COULOUARN_TEMPORAL_TGFB1_SIGNATURE_UP 109 -0.192997 -0.814029 0.588235 0.792525 0 down vs 1ZHU_CMV_8_HR_DN http://www.broadinstitute.org/gsea/msigdb/cards/ZHU_CMV_8_HR_DN 52 -0.207561 -0.812048 0.85 0.792727 0 down vs 1SHIPP_DLBCL_VS_FOLLICULAR_LYMPHOMA_UP http://www.broadinstitute.org/gsea/msigdb/cards/SHIPP_DLBCL_VS_FOLLICULAR_LYMPHOMA_UP 44 0.368052 1.06008 0.405405 0.792846 0 up vs 1STEARMAN_LUNG_CANCER_EARLY_VS_LATE_UP http://www.broadinstitute.org/gsea/msigdb/cards/STEARMAN_LUNG_CANCER_EARLY_VS_LATE_UP 122 0.301508 1.06826 0.379747 0.793084 0 up vs 1CHEOK_RESPONSE_TO_MERCAPTOPURINE_AND_HD_MTX_DN http://www.broadinstitute.org/gsea/msigdb/cards/CHEOK_RESPONSE_TO_MERCAPTOPURINE_AND_HD_MTX_DN 23 0.323928 1.06109 0.367089 0.793109 0 up vs 1HOFMANN_MYELODYSPLASTIC_SYNDROM_RISK_DN http://www.broadinstitute.org/gsea/msigdb/cards/HOFMANN_MYELODYSPLASTIC_SYNDROM_RISK_DN 23 -0.268191 -0.812412 0.742857 0.793133 0 down vs 1MOOTHA_FFA_OXYDATION http://www.broadinstitute.org/gsea/msigdb/cards/MOOTHA_FFA_OXYDATION 22 0.388795 1.0675 0.338235 0.793134 0 up vs 1BROWNE_HCMV_INFECTION_24HR_UP http://www.broadinstitute.org/gsea/msigdb/cards/BROWNE_HCMV_INFECTION_24HR_UP 144 0.273811 1.06783 0.45679 0.79325 0 up vs 1MIKKELSEN_NPC_HCP_WITH_H3K27ME3 http://www.broadinstitute.org/gsea/msigdb/cards/MIKKELSEN_NPC_HCP_WITH_H3K27ME3 334 0.256299 1.06031 0.475 0.793252 0 up vs 1RUTELLA_RESPONSE_TO_CSF2RB_AND_IL4_DN http://www.broadinstitute.org/gsea/msigdb/cards/RUTELLA_RESPONSE_TO_CSF2RB_AND_IL4_DN 306 -0.20246 -0.812827 0.708333 0.793368 0 down vs 1VERRECCHIA_DELAYED_RESPONSE_TO_TGFB1 http://www.broadinstitute.org/gsea/msigdb/cards/VERRECCHIA_DELAYED_RESPONSE_TO_TGFB1 39 -0.223062 -0.809563 0.708333 0.793982 0 down vs 1CAFFAREL_RESPONSE_TO_THC_24HR_5_UP http://www.broadinstitute.org/gsea/msigdb/cards/CAFFAREL_RESPONSE_TO_THC_24HR_5_UP 32 0.345102 1.06831 0.345679 0.793991 0 up vs 1HWANG_PROSTATE_CANCER_MARKERS http://www.broadinstitute.org/gsea/msigdb/cards/HWANG_PROSTATE_CANCER_MARKERS 28 -0.2228 -0.80857 0.857143 0.794393 0 down vs 1NGO_MALIGNANT_GLIOMA_1P_LOH http://www.broadinstitute.org/gsea/msigdb/cards/NGO_MALIGNANT_GLIOMA_1P_LOH 15 0.393401 1.05683 0.342105 0.795817 0 up vs 1MULLIGHAN_MLL_SIGNATURE_2_DN http://www.broadinstitute.org/gsea/msigdb/cards/MULLIGHAN_MLL_SIGNATURE_2_DN 271 0.271399 1.05638 0.488095 0.796171 0 up vs 1CHANGOLKAR_H2AFY_TARGETS_DN http://www.broadinstitute.org/gsea/msigdb/cards/CHANGOLKAR_H2AFY_TARGETS_DN 38 0.31113 1.05709 0.35443 0.796183 0 up vs 1MIKKELSEN_MEF_ICP_WITH_H3K27ME3 http://www.broadinstitute.org/gsea/msigdb/cards/MIKKELSEN_MEF_ICP_WITH_H3K27ME3 202 0.271135 1.05599 0.472222 0.796201 0 up vs 1BROWNE_HCMV_INFECTION_18HR_UP http://www.broadinstitute.org/gsea/msigdb/cards/BROWNE_HCMV_INFECTION_18HR_UP 175 0.294394 1.0549 0.413333 0.796251 0 up vs 1PARK_HSC_AND_MULTIPOTENT_PROGENITORS http://www.broadinstitute.org/gsea/msigdb/cards/PARK_HSC_AND_MULTIPOTENT_PROGENITORS 49 0.317051 1.05717 0.402299 0.797051 0 up vs 1HANN_RESISTANCE_TO_BCL2_INHIBITOR_DN http://www.broadinstitute.org/gsea/msigdb/cards/HANN_RESISTANCE_TO_BCL2_INHIBITOR_DN 47 0.296052 1.05296 0.357143 0.797165 0 up vs 1KUMAR_AUTOPHAGY_NETWORK http://www.broadinstitute.org/gsea/msigdb/cards/KUMAR_AUTOPHAGY_NETWORK 68 0.262933 1.05491 0.4875 0.797237 0 up vs 1CHUNG_BLISTER_CYTOTOXICITY_UP http://www.broadinstitute.org/gsea/msigdb/cards/CHUNG_BLISTER_CYTOTOXICITY_UP 129 0.284157 1.05526 0.402439 0.797291 0 up vs 1LUI_THYROID_CANCER_PAX8_PPARG_DN http://www.broadinstitute.org/gsea/msigdb/cards/LUI_THYROID_CANCER_PAX8_PPARG_DN 45 0.342815 1.05743 0.426829 0.797345 0 up vs 1BROWNE_HCMV_INFECTION_20HR_UP http://www.broadinstitute.org/gsea/msigdb/cards/BROWNE_HCMV_INFECTION_20HR_UP 232 0.270938 1.05327 0.481481 0.797394 0 up vs 1DORSAM_HOXA9_TARGETS_DN http://www.broadinstitute.org/gsea/msigdb/cards/DORSAM_HOXA9_TARGETS_DN 32 0.30916 1.05355 0.4 0.797541 0 up vs 1MARIADASON_RESPONSE_TO_CURCUMIN_SULINDAC_5 http://www.broadinstitute.org/gsea/msigdb/cards/MARIADASON_RESPONSE_TO_CURCUMIN_SULINDAC_5 22 0.336113 1.05748 0.361446 0.798217 0 up vs 1LA_MEN1_TARGETS http://www.broadinstitute.org/gsea/msigdb/cards/LA_MEN1_TARGETS 24 0.340569 1.05362 0.388889 0.798422 0 up vs 1BYSTROEM_CORRELATED_WITH_IL5_DN http://www.broadinstitute.org/gsea/msigdb/cards/BYSTROEM_CORRELATED_WITH_IL5_DN 63 -0.228026 -0.805367 0.69697 0.798572 0 down vs 1LEE_AGING_CEREBELLUM_DN http://www.broadinstitute.org/gsea/msigdb/cards/LEE_AGING_CEREBELLUM_DN 84 0.266665 1.0521 0.526316 0.798748 0 up vs 1PAPASPYRIDONOS_UNSTABLE_ATEROSCLEROTIC_PLAQUE_UP http://www.broadinstitute.org/gsea/msigdb/cards/PAPASPYRIDONOS_UNSTABLE_ATEROSCLEROTIC_PLAQUE_UP 51 -0.255721 -0.804226 0.714286 0.79935 0 down vs 1WEBER_METHYLATED_ICP_IN_FIBROBLAST http://www.broadinstitute.org/gsea/msigdb/cards/WEBER_METHYLATED_ICP_IN_FIBROBLAST 22 0.290655 1.04995 0.38806 0.799966 0 up vs 1AMUNDSON_GAMMA_RADIATION_RESISTANCE http://www.broadinstitute.org/gsea/msigdb/cards/AMUNDSON_GAMMA_RADIATION_RESISTANCE 20 0.308026 1.04954 0.393939 0.799981 0 up vs 1NAKAYAMA_FGF2_TARGETS http://www.broadinstitute.org/gsea/msigdb/cards/NAKAYAMA_FGF2_TARGETS 29 0.320118 1.04874 0.363636 0.800031 0 up vs 1DAZARD_RESPONSE_TO_UV_SCC_UP http://www.broadinstitute.org/gsea/msigdb/cards/DAZARD_RESPONSE_TO_UV_SCC_UP 118 -0.211608 -0.803045 0.571429 0.800144 0 down vs 1IIZUKA_LIVER_CANCER_PROGRESSION_L1_G1_UP http://www.broadinstitute.org/gsea/msigdb/cards/IIZUKA_LIVER_CANCER_PROGRESSION_L1_G1_UP 24 0.328013 1.04906 0.375 0.8002 0 up vs 1TAKEDA_TARGETS_OF_NUP98_HOXA9_FUSION_10D_DN http://www.broadinstitute.org/gsea/msigdb/cards/TAKEDA_TARGETS_OF_NUP98_HOXA9_FUSION_10D_DN 137 -0.21859 -0.802062 0.777778 0.800597 0 down vs 1ELVIDGE_HYPOXIA_BY_DMOG_DN http://www.broadinstitute.org/gsea/msigdb/cards/ELVIDGE_HYPOXIA_BY_DMOG_DN 58 0.282923 1.04817 0.358025 0.800605 0 up vs 1MOREAUX_B_LYMPHOCYTE_MATURATION_BY_TACI_DN http://www.broadinstitute.org/gsea/msigdb/cards/MOREAUX_B_LYMPHOCYTE_MATURATION_BY_TACI_DN 70 0.356767 1.05008 0.405063 0.800637 0 up vs 1PIONTEK_PKD1_TARGETS_DN http://www.broadinstitute.org/gsea/msigdb/cards/PIONTEK_PKD1_TARGETS_DN 17 0.336929 1.0478 0.342857 0.800701 0 up vs 1BILANGES_SERUM_SENSITIVE_VIA_TSC2 http://www.broadinstitute.org/gsea/msigdb/cards/BILANGES_SERUM_SENSITIVE_VIA_TSC2 39 0.341106 1.05105 0.378788 0.800727 0 up vs 1MCCLUNG_CREB1_TARGETS_UP http://www.broadinstitute.org/gsea/msigdb/cards/MCCLUNG_CREB1_TARGETS_UP 97 0.281729 1.05028 0.443038 0.801002 0 up vs 1ZHANG_TLX_TARGETS_60HR_UP http://www.broadinstitute.org/gsea/msigdb/cards/ZHANG_TLX_TARGETS_60HR_UP 288 0.266729 1.04728 0.518519 0.801121 0 up vs 1BROWNE_HCMV_INFECTION_14HR_UP http://www.broadinstitute.org/gsea/msigdb/cards/BROWNE_HCMV_INFECTION_14HR_UP 156 0.295039 1.05037 0.421053 0.801747 0 up vs 1CLIMENT_BREAST_CANCER_COPY_NUMBER_UP http://www.broadinstitute.org/gsea/msigdb/cards/CLIMENT_BREAST_CANCER_COPY_NUMBER_UP 23 0.304159 1.04264 0.393939 0.803229 0 up vs 1GAUSSMANN_MLL_AF4_FUSION_TARGETS_A_UP http://www.broadinstitute.org/gsea/msigdb/cards/GAUSSMANN_MLL_AF4_FUSION_TARGETS_A_UP 185 0.292637 1.04571 0.428571 0.803284 0 up vs 1RUAN_RESPONSE_TO_TNF_TROGLITAZONE_DN http://www.broadinstitute.org/gsea/msigdb/cards/RUAN_RESPONSE_TO_TNF_TROGLITAZONE_DN 41 0.289042 1.04291 0.417722 0.803589 0 up vs 1BHAT_ESR1_TARGETS_VIA_AKT1_UP http://www.broadinstitute.org/gsea/msigdb/cards/BHAT_ESR1_TARGETS_VIA_AKT1_UP 274 0.263261 1.04353 0.506024 0.803599 0 up vs 1WANG_LMO4_TARGETS_UP http://www.broadinstitute.org/gsea/msigdb/cards/WANG_LMO4_TARGETS_UP 350 0.274315 1.04524 0.5 0.803618 0 up vs 1SERVITJA_LIVER_HNF1A_TARGETS_DN http://www.broadinstitute.org/gsea/msigdb/cards/SERVITJA_LIVER_HNF1A_TARGETS_DN 156 0.267923 1.04373 0.520548 0.804021 0 up vs 1LEE_NEURAL_CREST_STEM_CELL_UP http://www.broadinstitute.org/gsea/msigdb/cards/LEE_NEURAL_CREST_STEM_CELL_UP 143 0.284406 1.04305 0.434783 0.804111 0 up vs 1LEIN_MEDULLA_MARKERS http://www.broadinstitute.org/gsea/msigdb/cards/LEIN_MEDULLA_MARKERS 80 0.260221 1.04405 0.420455 0.804173 0 up vs 1HOFFMANN_PRE_BI_TO_LARGE_PRE_BII_LYMPHOCYTE_UP http://www.broadinstitute.org/gsea/msigdb/cards/HOFFMANN_PRE_BI_TO_LARGE_PRE_BII_LYMPHOCYTE_UP 35 0.288981 1.04572 0.402778 0.804287 0 up vs 1CEBALLOS_TARGETS_OF_TP53_AND_MYC_DN http://www.broadinstitute.org/gsea/msigdb/cards/CEBALLOS_TARGETS_OF_TP53_AND_MYC_DN 37 0.290275 1.04436 0.356164 0.804294 0 up vs 1ZHAN_VARIABLE_EARLY_DIFFERENTIATION_GENES_DN http://www.broadinstitute.org/gsea/msigdb/cards/ZHAN_VARIABLE_EARLY_DIFFERENTIATION_GENES_DN 30 0.346251 1.04463 0.385542 0.804618 0 up vs 1PUJANA_BRCA2_PCC_NETWORK http://www.broadinstitute.org/gsea/msigdb/cards/PUJANA_BRCA2_PCC_NETWORK 414 -0.243616 -0.798438 0.75 0.804887 0 down vs 1AMIT_EGF_RESPONSE_480_MCF10A http://www.broadinstitute.org/gsea/msigdb/cards/AMIT_EGF_RESPONSE_480_MCF10A 41 -0.209571 -0.797026 0.8 0.806381 0 down vs 1GARCIA_TARGETS_OF_FLI1_AND_DAX1_DN http://www.broadinstitute.org/gsea/msigdb/cards/GARCIA_TARGETS_OF_FLI1_AND_DAX1_DN 168 0.305474 1.03851 0.421687 0.806863 0 up vs 1CHARAFE_BREAST_CANCER_LUMINAL_VS_MESENCHYMAL_DN http://www.broadinstitute.org/gsea/msigdb/cards/CHARAFE_BREAST_CANCER_LUMINAL_VS_MESENCHYMAL_DN 449 -0.190698 -0.795503 0.652174 0.807619 0 down vs 1NIKOLSKY_MUTATED_AND_AMPLIFIED_IN_BREAST_CANCER http://www.broadinstitute.org/gsea/msigdb/cards/NIKOLSKY_MUTATED_AND_AMPLIFIED_IN_BREAST_CANCER 93 0.275303 1.03852 0.492958 0.807839 0 up vs 1MUELLER_PLURINET http://www.broadinstitute.org/gsea/msigdb/cards/MUELLER_PLURINET 299 0.317012 1.04019 0.444444 0.808294 0 up vs 1MARCHINI_TRABECTEDIN_RESISTANCE_DN http://www.broadinstitute.org/gsea/msigdb/cards/MARCHINI_TRABECTEDIN_RESISTANCE_DN 48 0.324139 1.03935 0.486842 0.808651 0 up vs 1GUO_HEX_TARGETS_DN http://www.broadinstitute.org/gsea/msigdb/cards/GUO_HEX_TARGETS_DN 64 0.283225 1.03857 0.430233 0.808749 0 up vs 1INGRAM_SHH_TARGETS_UP http://www.broadinstitute.org/gsea/msigdb/cards/INGRAM_SHH_TARGETS_UP 124 0.269988 1.03969 0.506173 0.808857 0 up vs 1VERRECCHIA_RESPONSE_TO_TGFB1_C1 http://www.broadinstitute.org/gsea/msigdb/cards/VERRECCHIA_RESPONSE_TO_TGFB1_C1 19 0.344721 1.04031 0.375 0.809009 0 up vs 1MIKKELSEN_MCV6_ICP_WITH_H3K4ME3_AND_H3K27ME3 http://www.broadinstitute.org/gsea/msigdb/cards/MIKKELSEN_MCV6_ICP_WITH_H3K4ME3_AND_H3K27ME3 33 0.291512 1.03866 0.455882 0.809521 0 up vs 1ODONNELL_TFRC_TARGETS_DN http://www.broadinstitute.org/gsea/msigdb/cards/ODONNELL_TFRC_TARGETS_DN 132 -0.252672 -0.793229 0.68 0.809565 0 down vs 1WEBER_METHYLATED_HCP_IN_SPERM_UP http://www.broadinstitute.org/gsea/msigdb/cards/WEBER_METHYLATED_HCP_IN_SPERM_UP 20 -0.234699 -0.793489 0.777778 0.810068 0 down vs 1ZHAN_MULTIPLE_MYELOMA_CD2_DN http://www.broadinstitute.org/gsea/msigdb/cards/ZHAN_MULTIPLE_MYELOMA_CD2_DN 46 -0.210145 -0.790578 0.821429 0.811755 0 down vs 1KASLER_HDAC7_TARGETS_1_DN http://www.broadinstitute.org/gsea/msigdb/cards/KASLER_HDAC7_TARGETS_1_DN 17 -0.265944 -0.789914 0.833333 0.811772 0 down vs 1DASU_IL6_SIGNALING_SCAR_DN http://www.broadinstitute.org/gsea/msigdb/cards/DASU_IL6_SIGNALING_SCAR_DN 16 -0.291614 -0.791103 0.756757 0.811838 0 down vs 1JUBAN_TARGETS_OF_SPI1_AND_FLI1_UP http://www.broadinstitute.org/gsea/msigdb/cards/JUBAN_TARGETS_OF_SPI1_AND_FLI1_UP 110 0.281076 1.03629 0.4375 0.812245 0 up vs 1DUTERTRE_ESTRADIOL_RESPONSE_6HR_DN http://www.broadinstitute.org/gsea/msigdb/cards/DUTERTRE_ESTRADIOL_RESPONSE_6HR_DN 90 0.289184 1.03551 0.410959 0.813313 0 up vs 1WIERENGA_STAT5A_TARGETS_DN http://www.broadinstitute.org/gsea/msigdb/cards/WIERENGA_STAT5A_TARGETS_DN 206 -0.190707 -0.788142 0.809524 0.813566 0 down vs 1HOSHIDA_LIVER_CANCER_SUBCLASS_S3 http://www.broadinstitute.org/gsea/msigdb/cards/HOSHIDA_LIVER_CANCER_SUBCLASS_S3 264 0.261363 1.03407 0.53012 0.814264 0 up vs 1MATTIOLI_MULTIPLE_MYELOMA_WITH_14Q32_TRANSLOCATIONS http://www.broadinstitute.org/gsea/msigdb/cards/MATTIOLI_MULTIPLE_MYELOMA_WITH_14Q32_TRANSLOCATIONS 34 0.316283 1.03421 0.412698 0.814878 0 up vs 1HELLER_HDAC_TARGETS_DN http://www.broadinstitute.org/gsea/msigdb/cards/HELLER_HDAC_TARGETS_DN 282 0.262526 1.03287 0.493671 0.815319 0 up vs 1FARMER_BREAST_CANCER_CLUSTER_6 http://www.broadinstitute.org/gsea/msigdb/cards/FARMER_BREAST_CANCER_CLUSTER_6 16 0.381725 1.03318 0.380282 0.81552 0 up vs 1GROSS_HIF1A_TARGETS_DN http://www.broadinstitute.org/gsea/msigdb/cards/GROSS_HIF1A_TARGETS_DN 25 0.323135 1.03432 0.38806 0.815534 0 up vs 1PARK_HSC_MARKERS http://www.broadinstitute.org/gsea/msigdb/cards/PARK_HSC_MARKERS 44 -0.195032 -0.785634 0.730769 0.816177 0 down vs 1CREIGHTON_ENDOCRINE_THERAPY_RESISTANCE_2 http://www.broadinstitute.org/gsea/msigdb/cards/CREIGHTON_ENDOCRINE_THERAPY_RESISTANCE_2 433 0.324068 1.03194 0.418919 0.81666 0 up vs 1LE_EGR2_TARGETS_DN http://www.broadinstitute.org/gsea/msigdb/cards/LE_EGR2_TARGETS_DN 107 0.260201 1.02893 0.5 0.816721 0 up vs 1KORKOLA_SEMINOMA_UP http://www.broadinstitute.org/gsea/msigdb/cards/KORKOLA_SEMINOMA_UP 42 0.350522 1.02908 0.338462 0.817253 0 up vs 1GAZIN_EPIGENETIC_SILENCING_BY_KRAS http://www.broadinstitute.org/gsea/msigdb/cards/GAZIN_EPIGENETIC_SILENCING_BY_KRAS 25 0.340512 1.03115 0.386667 0.817792 0 up vs 1NGUYEN_NOTCH1_TARGETS_DN http://www.broadinstitute.org/gsea/msigdb/cards/NGUYEN_NOTCH1_TARGETS_DN 86 0.27686 1.02922 0.511628 0.817942 0 up vs 1BLUM_RESPONSE_TO_SALIRASIB_UP http://www.broadinstitute.org/gsea/msigdb/cards/BLUM_RESPONSE_TO_SALIRASIB_UP 240 0.261678 1.02925 0.466667 0.818847 0 up vs 1EBAUER_TARGETS_OF_PAX3_FOXO1_FUSION_DN http://www.broadinstitute.org/gsea/msigdb/cards/EBAUER_TARGETS_OF_PAX3_FOXO1_FUSION_DN 48 0.291199 1.02948 0.405797 0.819256 0 up vs 1RHODES_UNDIFFERENTIATED_CANCER http://www.broadinstitute.org/gsea/msigdb/cards/RHODES_UNDIFFERENTIATED_CANCER 68 -0.254502 -0.781475 0.653846 0.819869 0 down vs 1NADLER_OBESITY_UP http://www.broadinstitute.org/gsea/msigdb/cards/NADLER_OBESITY_UP 60 -0.251494 -0.782692 0.758621 0.819919 0 down vs 1GAVIN_FOXP3_TARGETS_CLUSTER_T7 http://www.broadinstitute.org/gsea/msigdb/cards/GAVIN_FOXP3_TARGETS_CLUSTER_T7 96 0.277591 1.0296 0.453488 0.81992 0 up vs 1LEE_LIVER_CANCER_DENA_DN http://www.broadinstitute.org/gsea/msigdb/cards/LEE_LIVER_CANCER_DENA_DN 74 0.258147 1.02991 0.481013 0.820184 0 up vs 1HERNANDEZ_MITOTIC_ARREST_BY_DOCETAXEL_1_UP http://www.broadinstitute.org/gsea/msigdb/cards/HERNANDEZ_MITOTIC_ARREST_BY_DOCETAXEL_1_UP 35 -0.226396 -0.781672 0.903226 0.820378 0 down vs 1MCCABE_HOXC6_TARGETS_CANCER_DN http://www.broadinstitute.org/gsea/msigdb/cards/MCCABE_HOXC6_TARGETS_CANCER_DN 20 -0.221683 -0.780298 0.75 0.820878 0 down vs 1COLLIS_PRKDC_SUBSTRATES http://www.broadinstitute.org/gsea/msigdb/cards/COLLIS_PRKDC_SUBSTRATES 20 -0.268821 -0.776068 0.727273 0.824852 0 down vs 1OKAWA_NEUROBLASTOMA_1P36_31_DELETION http://www.broadinstitute.org/gsea/msigdb/cards/OKAWA_NEUROBLASTOMA_1P36_31_DELETION 22 -0.257634 -0.776484 0.647059 0.825132 0 down vs 1WANG_LSD1_TARGETS_UP http://www.broadinstitute.org/gsea/msigdb/cards/WANG_LSD1_TARGETS_UP 23 0.334044 1.02545 0.393939 0.825243 0 up vs 1TRAYNOR_RETT_SYNDROM_UP http://www.broadinstitute.org/gsea/msigdb/cards/TRAYNOR_RETT_SYNDROM_UP 41 -0.201024 -0.774687 0.84375 0.82572 0 down vs 1HOWLIN_CITED1_TARGETS_1_UP http://www.broadinstitute.org/gsea/msigdb/cards/HOWLIN_CITED1_TARGETS_1_UP 34 0.305065 1.02422 0.416667 0.827409 0 up vs 1SUZUKI_RESPONSE_TO_TSA_AND_DECITABINE_1A http://www.broadinstitute.org/gsea/msigdb/cards/SUZUKI_RESPONSE_TO_TSA_AND_DECITABINE_1A 22 -0.25355 -0.772401 0.780488 0.828046 0 down vs 1TORCHIA_TARGETS_OF_EWSR1_FLI1_FUSION_TOP20_UP http://www.broadinstitute.org/gsea/msigdb/cards/TORCHIA_TARGETS_OF_EWSR1_FLI1_FUSION_TOP20_UP 20 -0.250909 -0.770077 0.756757 0.830998 0 down vs 1FERRANDO_LYL1_NEIGHBORS http://www.broadinstitute.org/gsea/msigdb/cards/FERRANDO_LYL1_NEIGHBORS 15 -0.283615 -0.769002 0.764706 0.831177 0 down vs 1MELLMAN_TUT1_TARGETS_UP http://www.broadinstitute.org/gsea/msigdb/cards/MELLMAN_TUT1_TARGETS_UP 19 0.346746 1.02068 0.390244 0.832601 0 up vs 1VALK_AML_CLUSTER_8 http://www.broadinstitute.org/gsea/msigdb/cards/VALK_AML_CLUSTER_8 25 0.313345 1.02176 0.402778 0.833553 0 up vs 1SCHAEFFER_PROSTATE_DEVELOPMENT_12HR_UP http://www.broadinstitute.org/gsea/msigdb/cards/SCHAEFFER_PROSTATE_DEVELOPMENT_12HR_UP 115 0.265389 1.02068 0.538462 0.833582 0 up vs 1MIKKELSEN_IPS_ICP_WITH_H3K4ME3_AND_H327ME3 http://www.broadinstitute.org/gsea/msigdb/cards/MIKKELSEN_IPS_ICP_WITH_H3K4ME3_AND_H327ME3 126 0.269135 1.02095 0.486111 0.833868 0 up vs 1BOYAULT_LIVER_CANCER_SUBCLASS_G12_DN http://www.broadinstitute.org/gsea/msigdb/cards/BOYAULT_LIVER_CANCER_SUBCLASS_G12_DN 15 0.316148 1.02123 0.376812 0.833972 0 up vs 1TIMOFEEVA_GROWTH_STRESS_VIA_STAT1_DN http://www.broadinstitute.org/gsea/msigdb/cards/TIMOFEEVA_GROWTH_STRESS_VIA_STAT1_DN 16 -0.247484 -0.766345 0.676471 0.834103 0 down vs 1PLASARI_TGFB1_TARGETS_10HR_DN http://www.broadinstitute.org/gsea/msigdb/cards/PLASARI_TGFB1_TARGETS_10HR_DN 242 0.279482 1.01952 0.513514 0.834724 0 up vs 1STARK_HYPPOCAMPUS_22Q11_DELETION_DN http://www.broadinstitute.org/gsea/msigdb/cards/STARK_HYPPOCAMPUS_22Q11_DELETION_DN 19 0.420139 1.01906 0.414286 0.835101 0 up vs 1LANDIS_BREAST_CANCER_PROGRESSION_DN http://www.broadinstitute.org/gsea/msigdb/cards/LANDIS_BREAST_CANCER_PROGRESSION_DN 70 0.274899 1.01179 0.481013 0.835184 0 up vs 1SMID_BREAST_CANCER_LUMINAL_A_DN http://www.broadinstitute.org/gsea/msigdb/cards/SMID_BREAST_CANCER_LUMINAL_A_DN 18 -0.28784 -0.764821 0.78125 0.835187 0 down vs 1WANG_TUMOR_INVASIVENESS_DN http://www.broadinstitute.org/gsea/msigdb/cards/WANG_TUMOR_INVASIVENESS_DN 206 0.276128 1.0124 0.534884 0.835389 0 up vs 1GARGALOVIC_RESPONSE_TO_OXIDIZED_PHOSPHOLIPIDS_MAGENTA_UP http://www.broadinstitute.org/gsea/msigdb/cards/GARGALOVIC_RESPONSE_TO_OXIDIZED_PHOSPHOLIPIDS_MAGENTA_UP 26 0.356158 1.01301 0.385714 0.835652 0 up vs 1APPIERTO_RESPONSE_TO_FENRETINIDE_UP http://www.broadinstitute.org/gsea/msigdb/cards/APPIERTO_RESPONSE_TO_FENRETINIDE_UP 38 0.291317 1.01193 0.455696 0.835663 0 up vs 1MARTORIATI_MDM4_TARGETS_FETAL_LIVER_UP http://www.broadinstitute.org/gsea/msigdb/cards/MARTORIATI_MDM4_TARGETS_FETAL_LIVER_UP 220 0.245941 1.01262 0.5 0.835794 0 up vs 1BONOME_OVARIAN_CANCER_SURVIVAL_SUBOPTIMAL_DEBULKING http://www.broadinstitute.org/gsea/msigdb/cards/BONOME_OVARIAN_CANCER_SURVIVAL_SUBOPTIMAL_DEBULKING 497 0.257634 1.01675 0.546512 0.836265 0 up vs 1HUANG_FOXA2_TARGETS_UP http://www.broadinstitute.org/gsea/msigdb/cards/HUANG_FOXA2_TARGETS_UP 43 0.305131 1.01311 0.438356 0.836303 0 up vs 1WHITFIELD_CELL_CYCLE_M_G1 http://www.broadinstitute.org/gsea/msigdb/cards/WHITFIELD_CELL_CYCLE_M_G1 140 0.290384 1.01109 0.433735 0.83635 0 up vs 1FRASOR_RESPONSE_TO_ESTRADIOL_UP http://www.broadinstitute.org/gsea/msigdb/cards/FRASOR_RESPONSE_TO_ESTRADIOL_UP 37 0.278442 1.01708 0.5 0.836476 0 up vs 1MORI_EMU_MYC_LYMPHOMA_BY_ONSET_TIME_DN http://www.broadinstitute.org/gsea/msigdb/cards/MORI_EMU_MYC_LYMPHOMA_BY_ONSET_TIME_DN 17 0.346468 1.01373 0.424658 0.836654 0 up vs 1DAVICIONI_TARGETS_OF_PAX_FOXO1_FUSIONS_DN http://www.broadinstitute.org/gsea/msigdb/cards/DAVICIONI_TARGETS_OF_PAX_FOXO1_FUSIONS_DN 67 0.278717 1.01584 0.522388 0.836819 0 up vs 1MCCLUNG_COCAINE_REWARD_5D http://www.broadinstitute.org/gsea/msigdb/cards/MCCLUNG_COCAINE_REWARD_5D 79 0.257561 1.01322 0.536585 0.837022 0 up vs 1YAO_TEMPORAL_RESPONSE_TO_PROGESTERONE_CLUSTER_13 http://www.broadinstitute.org/gsea/msigdb/cards/YAO_TEMPORAL_RESPONSE_TO_PROGESTERONE_CLUSTER_13 167 0.326846 1.01602 0.480519 0.837173 0 up vs 1LEIN_OLIGODENDROCYTE_MARKERS http://www.broadinstitute.org/gsea/msigdb/cards/LEIN_OLIGODENDROCYTE_MARKERS 71 0.254682 1.01718 0.512195 0.837176 0 up vs 1KAN_RESPONSE_TO_ARSENIC_TRIOXIDE http://www.broadinstitute.org/gsea/msigdb/cards/KAN_RESPONSE_TO_ARSENIC_TRIOXIDE 122 0.263134 1.01746 0.547945 0.837388 0 up vs 1MARKS_ACETYLATED_NON_HISTONE_PROTEINS http://www.broadinstitute.org/gsea/msigdb/cards/MARKS_ACETYLATED_NON_HISTONE_PROTEINS 15 0.344173 1.01376 0.4 0.837489 0 up vs 1PUIFFE_INVASION_INHIBITED_BY_ASCITES_UP http://www.broadinstitute.org/gsea/msigdb/cards/PUIFFE_INVASION_INHIBITED_BY_ASCITES_UP 81 0.288371 1.0177 0.447059 0.837659 0 up vs 1PLASARI_NFIC_TARGETS_BASAL_UP http://www.broadinstitute.org/gsea/msigdb/cards/PLASARI_NFIC_TARGETS_BASAL_UP 27 0.288826 1.01398 0.462687 0.837785 0 up vs 1BOHN_PRIMARY_IMMUNODEFICIENCY_SYNDROM_UP http://www.broadinstitute.org/gsea/msigdb/cards/BOHN_PRIMARY_IMMUNODEFICIENCY_SYNDROM_UP 45 0.295282 1.00991 0.444444 0.838187 0 up vs 1NIELSEN_SCHWANNOMA_UP http://www.broadinstitute.org/gsea/msigdb/cards/NIELSEN_SCHWANNOMA_UP 17 0.328216 1.01409 0.37931 0.838537 0 up vs 1UEDA_CENTRAL_CLOCK http://www.broadinstitute.org/gsea/msigdb/cards/UEDA_CENTRAL_CLOCK 87 0.255197 1.01461 0.534091 0.839134 0 up vs 1HOFMANN_MYELODYSPLASTIC_SYNDROM_LOW_RISK_DN http://www.broadinstitute.org/gsea/msigdb/cards/HOFMANN_MYELODYSPLASTIC_SYNDROM_LOW_RISK_DN 30 0.315283 1.00913 0.381579 0.839168 0 up vs 1SU_PLACENTA http://www.broadinstitute.org/gsea/msigdb/cards/SU_PLACENTA 30 0.280472 1.00869 0.439394 0.839268 0 up vs 1CHIANG_LIVER_CANCER_SUBCLASS_PROLIFERATION_DN http://www.broadinstitute.org/gsea/msigdb/cards/CHIANG_LIVER_CANCER_SUBCLASS_PROLIFERATION_DN 174 0.271461 1.01413 0.513889 0.839435 0 up vs 1BOYAULT_LIVER_CANCER_SUBCLASS_G123_UP http://www.broadinstitute.org/gsea/msigdb/cards/BOYAULT_LIVER_CANCER_SUBCLASS_G123_UP 43 0.342082 1.0075 0.434211 0.841506 0 up vs 1MARTINEZ_RESPONSE_TO_TRABECTEDIN_DN http://www.broadinstitute.org/gsea/msigdb/cards/MARTINEZ_RESPONSE_TO_TRABECTEDIN_DN 267 0.301508 1.00706 0.428571 0.841603 0 up vs 1FERREIRA_EWINGS_SARCOMA_UNSTABLE_VS_STABLE_UP http://www.broadinstitute.org/gsea/msigdb/cards/FERREIRA_EWINGS_SARCOMA_UNSTABLE_VS_STABLE_UP 160 -0.223183 -0.759473 0.772727 0.842032 0 down vs 1MAEKAWA_ATF2_TARGETS http://www.broadinstitute.org/gsea/msigdb/cards/MAEKAWA_ATF2_TARGETS 24 0.281178 1.00623 0.506849 0.842177 0 up vs 1SCHMIDT_POR_TARGETS_IN_LIMB_BUD_UP http://www.broadinstitute.org/gsea/msigdb/cards/SCHMIDT_POR_TARGETS_IN_LIMB_BUD_UP 25 0.371948 1.0057 0.358209 0.842645 0 up vs 1BARRIER_CANCER_RELAPSE_TUMOR_SAMPLE_UP http://www.broadinstitute.org/gsea/msigdb/cards/BARRIER_CANCER_RELAPSE_TUMOR_SAMPLE_UP 16 -0.270914 -0.757552 0.827586 0.842876 0 down vs 1GRAESSMANN_RESPONSE_TO_MC_AND_SERUM_DEPRIVATION_DN http://www.broadinstitute.org/gsea/msigdb/cards/GRAESSMANN_RESPONSE_TO_MC_AND_SERUM_DEPRIVATION_DN 81 0.258964 1.00629 0.533333 0.842921 0 up vs 1WANG_TARGETS_OF_MLL_CBP_FUSION_UP http://www.broadinstitute.org/gsea/msigdb/cards/WANG_TARGETS_OF_MLL_CBP_FUSION_UP 44 0.279129 1.00491 0.485714 0.843122 0 up vs 1ABE_VEGFA_TARGETS http://www.broadinstitute.org/gsea/msigdb/cards/ABE_VEGFA_TARGETS 20 0.3476 1.00519 0.397059 0.843241 0 up vs 1SPIELMAN_LYMPHOBLAST_EUROPEAN_VS_ASIAN_2FC_DN http://www.broadinstitute.org/gsea/msigdb/cards/SPIELMAN_LYMPHOBLAST_EUROPEAN_VS_ASIAN_2FC_DN 21 -0.23797 -0.757894 0.783784 0.843337 0 down vs 1NAM_FXYD5_TARGETS_DN http://www.broadinstitute.org/gsea/msigdb/cards/NAM_FXYD5_TARGETS_DN 18 0.332024 1.00428 0.4625 0.843985 0 up vs 1KANG_FLUOROURACIL_RESISTANCE_UP http://www.broadinstitute.org/gsea/msigdb/cards/KANG_FLUOROURACIL_RESISTANCE_UP 22 -0.231141 -0.753968 0.84 0.844093 0 down vs 1VERRECCHIA_RESPONSE_TO_TGFB1_C5 http://www.broadinstitute.org/gsea/msigdb/cards/VERRECCHIA_RESPONSE_TO_TGFB1_C5 21 0.316964 1.00371 0.438356 0.84444 0 up vs 1HOWLIN_CITED1_TARGETS_2_UP http://www.broadinstitute.org/gsea/msigdb/cards/HOWLIN_CITED1_TARGETS_2_UP 16 -0.234715 -0.754376 0.846154 0.844701 0 down vs 1BERTUCCI_INVASIVE_CARCINOMA_DUCTAL_VS_LOBULAR_DN http://www.broadinstitute.org/gsea/msigdb/cards/BERTUCCI_INVASIVE_CARCINOMA_DUCTAL_VS_LOBULAR_DN 45 -0.226871 -0.752586 0.771429 0.844797 0 down vs 1MORI_MATURE_B_LYMPHOCYTE_DN http://www.broadinstitute.org/gsea/msigdb/cards/MORI_MATURE_B_LYMPHOCYTE_DN 73 -0.217727 -0.754407 0.9375 0.845558 0 down vs 1VANDESLUIS_COMMD1_TARGETS_GROUP_4_UP http://www.broadinstitute.org/gsea/msigdb/cards/VANDESLUIS_COMMD1_TARGETS_GROUP_4_UP 19 0.300891 1.00286 0.537313 0.845743 0 up vs 1NAKAYAMA_SOFT_TISSUE_TUMORS_PCA2_UP http://www.broadinstitute.org/gsea/msigdb/cards/NAKAYAMA_SOFT_TISSUE_TUMORS_PCA2_UP 87 -0.24159 -0.754712 0.827586 0.845955 0 down vs 1WONG_PROTEASOME_GENE_MODULE http://www.broadinstitute.org/gsea/msigdb/cards/WONG_PROTEASOME_GENE_MODULE 49 -0.207633 -0.750157 0.705882 0.846859 0 down vs 1FONTAINE_THYROID_TUMOR_UNCERTAIN_MALIGNANCY_DN http://www.broadinstitute.org/gsea/msigdb/cards/FONTAINE_THYROID_TUMOR_UNCERTAIN_MALIGNANCY_DN 26 -0.213339 -0.74745 0.793103 0.848274 0 down vs 1VANTVEER_BREAST_CANCER_BRCA1_UP http://www.broadinstitute.org/gsea/msigdb/cards/VANTVEER_BREAST_CANCER_BRCA1_UP 34 -0.193319 -0.746565 0.88 0.848367 0 down vs 1GREENBAUM_E2A_TARGETS_DN http://www.broadinstitute.org/gsea/msigdb/cards/GREENBAUM_E2A_TARGETS_DN 20 -0.24475 -0.747542 0.777778 0.84908 0 down vs 1CROMER_TUMORIGENESIS_UP http://www.broadinstitute.org/gsea/msigdb/cards/CROMER_TUMORIGENESIS_UP 62 -0.250735 -0.740962 0.823529 0.851842 0 down vs 1BILD_SRC_ONCOGENIC_SIGNATURE http://www.broadinstitute.org/gsea/msigdb/cards/BILD_SRC_ONCOGENIC_SIGNATURE 61 -0.194129 -0.741206 0.888889 0.852338 0 down vs 1MORI_PLASMA_CELL_DN http://www.broadinstitute.org/gsea/msigdb/cards/MORI_PLASMA_CELL_DN 30 -0.233671 -0.741555 0.84375 0.852739 0 down vs 1DOANE_BREAST_CANCER_CLASSES_DN http://www.broadinstitute.org/gsea/msigdb/cards/DOANE_BREAST_CANCER_CLASSES_DN 32 -0.224504 -0.742079 0.833333 0.852797 0 down vs 1ROLEF_GLIS3_TARGETS http://www.broadinstitute.org/gsea/msigdb/cards/ROLEF_GLIS3_TARGETS 37 0.272761 0.999199 0.514286 0.854831 0 up vs 1BONCI_TARGETS_OF_MIR15A_AND_MIR16_1 http://www.broadinstitute.org/gsea/msigdb/cards/BONCI_TARGETS_OF_MIR15A_AND_MIR16_1 90 0.298338 0.998186 0.465753 0.85548 0 up vs 1OUILLETTE_CLL_13Q14_DELETION_UP http://www.broadinstitute.org/gsea/msigdb/cards/OUILLETTE_CLL_13Q14_DELETION_UP 71 0.288669 0.998304 0.519481 0.856184 0 up vs 1LANDIS_ERBB2_BREAST_TUMORS_65_UP http://www.broadinstitute.org/gsea/msigdb/cards/LANDIS_ERBB2_BREAST_TUMORS_65_UP 22 0.337107 0.996107 0.445946 0.859243 0 up vs 1COLLIS_PRKDC_REGULATORS http://www.broadinstitute.org/gsea/msigdb/cards/COLLIS_PRKDC_REGULATORS 15 0.386759 0.996394 0.460526 0.859471 0 up vs 1HASLINGER_B_CLL_WITH_CHROMOSOME_12_TRISOMY http://www.broadinstitute.org/gsea/msigdb/cards/HASLINGER_B_CLL_WITH_CHROMOSOME_12_TRISOMY 23 0.332825 0.982356 0.458333 0.860542 0 up vs 1CUI_GLUCOSE_DEPRIVATION http://www.broadinstitute.org/gsea/msigdb/cards/CUI_GLUCOSE_DEPRIVATION 59 0.278527 0.982458 0.513889 0.861215 0 up vs 1LASTOWSKA_NEUROBLASTOMA_COPY_NUMBER_UP http://www.broadinstitute.org/gsea/msigdb/cards/LASTOWSKA_NEUROBLASTOMA_COPY_NUMBER_UP 174 0.280403 0.982684 0.506667 0.86165 0 up vs 1ZHONG_SECRETOME_OF_LUNG_CANCER_AND_ENDOTHELIUM http://www.broadinstitute.org/gsea/msigdb/cards/ZHONG_SECRETOME_OF_LUNG_CANCER_AND_ENDOTHELIUM 65 0.278696 0.982869 0.554217 0.86213 0 up vs 1TONKS_TARGETS_OF_RUNX1_RUNX1T1_FUSION_ERYTHROCYTE_DN http://www.broadinstitute.org/gsea/msigdb/cards/TONKS_TARGETS_OF_RUNX1_RUNX1T1_FUSION_ERYTHROCYTE_DN 15 0.317619 0.983677 0.514286 0.862142 0 up vs 1MAYBURD_RESPONSE_TO_L663536_DN http://www.broadinstitute.org/gsea/msigdb/cards/MAYBURD_RESPONSE_TO_L663536_DN 54 0.313058 0.987531 0.432099 0.862303 0 up vs 1GRAESSMANN_APOPTOSIS_BY_SERUM_DEPRIVATION_DN http://www.broadinstitute.org/gsea/msigdb/cards/GRAESSMANN_APOPTOSIS_BY_SERUM_DEPRIVATION_DN 229 0.236782 0.983115 0.523256 0.862505 0 up vs 1RAMASWAMY_METASTASIS_UP http://www.broadinstitute.org/gsea/msigdb/cards/RAMASWAMY_METASTASIS_UP 64 0.280483 0.987785 0.487805 0.862557 0 up vs 1PUIFFE_INVASION_INHIBITED_BY_ASCITES_DN http://www.broadinstitute.org/gsea/msigdb/cards/PUIFFE_INVASION_INHIBITED_BY_ASCITES_DN 142 0.28471 0.991321 0.494118 0.862779 0 up vs 1JEPSEN_SMRT_TARGETS http://www.broadinstitute.org/gsea/msigdb/cards/JEPSEN_SMRT_TARGETS 33 0.281987 0.985003 0.507042 0.862828 0 up vs 1WENG_POR_TARGETS_GLOBAL_UP http://www.broadinstitute.org/gsea/msigdb/cards/WENG_POR_TARGETS_GLOBAL_UP 20 0.319292 0.983738 0.43662 0.862945 0 up vs 1BROWNE_HCMV_INFECTION_6HR_DN http://www.broadinstitute.org/gsea/msigdb/cards/BROWNE_HCMV_INFECTION_6HR_DN 155 0.276196 0.990453 0.493671 0.863119 0 up vs 1GU_PDEF_TARGETS_UP http://www.broadinstitute.org/gsea/msigdb/cards/GU_PDEF_TARGETS_UP 71 0.300124 0.983965 0.5 0.863251 0 up vs 1SERVITJA_LIVER_HNF1A_TARGETS_UP http://www.broadinstitute.org/gsea/msigdb/cards/SERVITJA_LIVER_HNF1A_TARGETS_UP 133 0.256812 0.984438 0.559524 0.863254 0 up vs 1NADLER_OBESITY_DN http://www.broadinstitute.org/gsea/msigdb/cards/NADLER_OBESITY_DN 48 0.277954 0.989043 0.5 0.863271 0 up vs 1BHAT_ESR1_TARGETS_NOT_VIA_AKT1_DN http://www.broadinstitute.org/gsea/msigdb/cards/BHAT_ESR1_TARGETS_NOT_VIA_AKT1_DN 87 0.276434 0.986742 0.5 0.863352 0 up vs 1RIZ_ERYTHROID_DIFFERENTIATION_12HR http://www.broadinstitute.org/gsea/msigdb/cards/RIZ_ERYTHROID_DIFFERENTIATION_12HR 43 0.286293 0.98786 0.558824 0.863352 0 up vs 1VERNELL_RETINOBLASTOMA_PATHWAY_DN http://www.broadinstitute.org/gsea/msigdb/cards/VERNELL_RETINOBLASTOMA_PATHWAY_DN 22 0.328279 0.991495 0.493151 0.863383 0 up vs 1SMID_BREAST_CANCER_RELAPSE_IN_BRAIN_DN http://www.broadinstitute.org/gsea/msigdb/cards/SMID_BREAST_CANCER_RELAPSE_IN_BRAIN_DN 83 0.275977 0.990034 0.520548 0.863407 0 up vs 1LINSLEY_MIR16_TARGETS http://www.broadinstitute.org/gsea/msigdb/cards/LINSLEY_MIR16_TARGETS 198 0.271523 0.988141 0.517241 0.863572 0 up vs 1HERNANDEZ_MITOTIC_ARREST_BY_DOCETAXEL_2_UP http://www.broadinstitute.org/gsea/msigdb/cards/HERNANDEZ_MITOTIC_ARREST_BY_DOCETAXEL_2_UP 63 0.262543 0.98505 0.575 0.863599 0 up vs 1ALCALAY_AML_BY_NPM1_LOCALIZATION_UP http://www.broadinstitute.org/gsea/msigdb/cards/ALCALAY_AML_BY_NPM1_LOCALIZATION_UP 138 0.260809 0.985889 0.56962 0.863615 0 up vs 1YAO_TEMPORAL_RESPONSE_TO_PROGESTERONE_CLUSTER_3 http://www.broadinstitute.org/gsea/msigdb/cards/YAO_TEMPORAL_RESPONSE_TO_PROGESTERONE_CLUSTER_3 15 0.307475 0.994125 0.508197 0.863634 0 up vs 1ZHANG_PROLIFERATING_VS_QUIESCENT http://www.broadinstitute.org/gsea/msigdb/cards/ZHANG_PROLIFERATING_VS_QUIESCENT 51 0.280961 0.989578 0.5 0.863712 0 up vs 1HERNANDEZ_MITOTIC_ARREST_BY_DOCETAXEL_2_DN http://www.broadinstitute.org/gsea/msigdb/cards/HERNANDEZ_MITOTIC_ARREST_BY_DOCETAXEL_2_DN 18 0.315408 0.989217 0.445946 0.863744 0 up vs 1ZEMBUTSU_SENSITIVITY_TO_METHOTREXATE http://www.broadinstitute.org/gsea/msigdb/cards/ZEMBUTSU_SENSITIVITY_TO_METHOTREXATE 18 0.321214 0.99051 0.462687 0.863904 0 up vs 1SNIJDERS_AMPLIFIED_IN_HEAD_AND_NECK_TUMORS http://www.broadinstitute.org/gsea/msigdb/cards/SNIJDERS_AMPLIFIED_IN_HEAD_AND_NECK_TUMORS 37 0.285348 0.988361 0.405797 0.863927 0 up vs 1BURTON_ADIPOGENESIS_12 http://www.broadinstitute.org/gsea/msigdb/cards/BURTON_ADIPOGENESIS_12 32 0.385649 0.985328 0.371429 0.863961 0 up vs 1KIM_GERMINAL_CENTER_T_HELPER_DN http://www.broadinstitute.org/gsea/msigdb/cards/KIM_GERMINAL_CENTER_T_HELPER_DN 23 0.297291 0.98613 0.473684 0.863977 0 up vs 1GAUSSMANN_MLL_AF4_FUSION_TARGETS_G_DN http://www.broadinstitute.org/gsea/msigdb/cards/GAUSSMANN_MLL_AF4_FUSION_TARGETS_G_DN 33 0.27991 0.980653 0.493151 0.863996 0 up vs 1HERNANDEZ_ABERRANT_MITOSIS_BY_DOCETACEL_4NM_UP http://www.broadinstitute.org/gsea/msigdb/cards/HERNANDEZ_ABERRANT_MITOSIS_BY_DOCETACEL_4NM_UP 23 0.295684 0.991944 0.460526 0.864284 0 up vs 1NAKAMURA_ADIPOGENESIS_EARLY_UP http://www.broadinstitute.org/gsea/msigdb/cards/NAKAMURA_ADIPOGENESIS_EARLY_UP 64 0.280064 0.991513 0.506667 0.864316 0 up vs 1XU_HGF_TARGETS_REPRESSED_BY_AKT1_DN http://www.broadinstitute.org/gsea/msigdb/cards/XU_HGF_TARGETS_REPRESSED_BY_AKT1_DN 93 0.258879 0.980126 0.594203 0.864354 0 up vs 1VIETOR_IFRD1_TARGETS http://www.broadinstitute.org/gsea/msigdb/cards/VIETOR_IFRD1_TARGETS 23 0.329301 0.979645 0.42029 0.864703 0 up vs 1NADLER_HYPERGLYCEMIA_AT_OBESITY http://www.broadinstitute.org/gsea/msigdb/cards/NADLER_HYPERGLYCEMIA_AT_OBESITY 58 0.259798 0.99212 0.556818 0.864754 0 up vs 1HASLINGER_B_CLL_WITH_17P13_DELETION http://www.broadinstitute.org/gsea/msigdb/cards/HASLINGER_B_CLL_WITH_17P13_DELETION 19 0.334684 0.992154 0.382716 0.865629 0 up vs 1SUZUKI_RESPONSE_TO_TSA_AND_DECITABINE_1B http://www.broadinstitute.org/gsea/msigdb/cards/SUZUKI_RESPONSE_TO_TSA_AND_DECITABINE_1B 22 0.287624 0.992334 0.530303 0.866138 0 up vs 1LIU_SOX4_TARGETS_DN http://www.broadinstitute.org/gsea/msigdb/cards/LIU_SOX4_TARGETS_DN 297 0.26384 0.992489 0.579545 0.866768 0 up vs 1BURTON_ADIPOGENESIS_PEAK_AT_0HR http://www.broadinstitute.org/gsea/msigdb/cards/BURTON_ADIPOGENESIS_PEAK_AT_0HR 62 -0.198751 -0.728961 0.869565 0.866951 0 down vs 1BOYLAN_MULTIPLE_MYELOMA_C_CLUSTER_DN http://www.broadinstitute.org/gsea/msigdb/cards/BOYLAN_MULTIPLE_MYELOMA_C_CLUSTER_DN 31 -0.213345 -0.725281 0.75 0.869657 0 down vs 1DAZARD_UV_RESPONSE_CLUSTER_G6 http://www.broadinstitute.org/gsea/msigdb/cards/DAZARD_UV_RESPONSE_CLUSTER_G6 147 -0.209978 -0.726127 0.714286 0.869668 0 down vs 1RATTENBACHER_BOUND_BY_CELF1 http://www.broadinstitute.org/gsea/msigdb/cards/RATTENBACHER_BOUND_BY_CELF1 423 0.237896 0.977121 0.551724 0.870149 0 up vs 1ZHAN_MULTIPLE_MYELOMA_UP http://www.broadinstitute.org/gsea/msigdb/cards/ZHAN_MULTIPLE_MYELOMA_UP 64 0.259932 0.966599 0.593407 0.871232 0 up vs 1YIH_RESPONSE_TO_ARSENITE_C3 http://www.broadinstitute.org/gsea/msigdb/cards/YIH_RESPONSE_TO_ARSENITE_C3 36 0.282819 0.97602 0.511905 0.871731 0 up vs 1BROWNE_HCMV_INFECTION_30MIN_DN http://www.broadinstitute.org/gsea/msigdb/cards/BROWNE_HCMV_INFECTION_30MIN_DN 144 0.251216 0.975201 0.623377 0.871812 0 up vs 1ALCALA_APOPTOSIS http://www.broadinstitute.org/gsea/msigdb/cards/ALCALA_APOPTOSIS 86 0.272459 0.965679 0.567901 0.871843 0 up vs 1YAMASHITA_LIVER_CANCER_STEM_CELL_UP http://www.broadinstitute.org/gsea/msigdb/cards/YAMASHITA_LIVER_CANCER_STEM_CELL_UP 47 0.267942 0.967546 0.512821 0.87185 0 up vs 1KAPOSI_LIVER_CANCER_MET_UP http://www.broadinstitute.org/gsea/msigdb/cards/KAPOSI_LIVER_CANCER_MET_UP 17 0.360582 0.966699 0.461538 0.871912 0 up vs 1TUOMISTO_TUMOR_SUPPRESSION_BY_COL13A1_DN http://www.broadinstitute.org/gsea/msigdb/cards/TUOMISTO_TUMOR_SUPPRESSION_BY_COL13A1_DN 17 -0.246873 -0.722683 0.833333 0.872139 0 down vs 1LIANG_HEMATOPOIESIS_STEM_CELL_NUMBER_QTL http://www.broadinstitute.org/gsea/msigdb/cards/LIANG_HEMATOPOIESIS_STEM_CELL_NUMBER_QTL 16 0.310756 0.975401 0.470588 0.872273 0 up vs 1ZHAN_MULTIPLE_MYELOMA_HP_UP http://www.broadinstitute.org/gsea/msigdb/cards/ZHAN_MULTIPLE_MYELOMA_HP_UP 46 0.286088 0.967672 0.480519 0.872473 0 up vs 1RUIZ_TNC_TARGETS_UP http://www.broadinstitute.org/gsea/msigdb/cards/RUIZ_TNC_TARGETS_UP 151 0.275056 0.966841 0.6 0.872517 0 up vs 1HOEGERKORP_CD44_TARGETS_TEMPORAL_DN http://www.broadinstitute.org/gsea/msigdb/cards/HOEGERKORP_CD44_TARGETS_TEMPORAL_DN 25 0.306669 0.965791 0.534247 0.87253 0 up vs 1MEISSNER_BRAIN_HCP_WITH_H3K4ME2 http://www.broadinstitute.org/gsea/msigdb/cards/MEISSNER_BRAIN_HCP_WITH_H3K4ME2 16 0.312317 0.974508 0.43662 0.872695 0 up vs 1MEINHOLD_OVARIAN_CANCER_LOW_GRADE_DN http://www.broadinstitute.org/gsea/msigdb/cards/MEINHOLD_OVARIAN_CANCER_LOW_GRADE_DN 20 -0.25321 -0.721121 0.846154 0.872999 0 down vs 1WANG_MLL_TARGETS http://www.broadinstitute.org/gsea/msigdb/cards/WANG_MLL_TARGETS 282 0.244815 0.97154 0.61039 0.873205 0 up vs 1FUKUSHIMA_TNFSF11_TARGETS http://www.broadinstitute.org/gsea/msigdb/cards/FUKUSHIMA_TNFSF11_TARGETS 16 0.313693 0.973835 0.430769 0.873268 0 up vs 1KANG_IMMORTALIZED_BY_TERT_DN http://www.broadinstitute.org/gsea/msigdb/cards/KANG_IMMORTALIZED_BY_TERT_DN 101 0.260312 0.971898 0.583333 0.873331 0 up vs 1APRELIKOVA_BRCA1_TARGETS http://www.broadinstitute.org/gsea/msigdb/cards/APRELIKOVA_BRCA1_TARGETS 49 0.291228 0.967682 0.560976 0.87339 0 up vs 1IKEDA_MIR133_TARGETS_UP http://www.broadinstitute.org/gsea/msigdb/cards/IKEDA_MIR133_TARGETS_UP 43 0.333286 0.971125 0.472973 0.873426 0 up vs 1KYNG_DNA_DAMAGE_BY_4NQO_OR_UV http://www.broadinstitute.org/gsea/msigdb/cards/KYNG_DNA_DAMAGE_BY_4NQO_OR_UV 63 0.252754 0.964527 0.592593 0.873649 0 up vs 1LEE_METASTASIS_AND_ALTERNATIVE_SPLICING_UP http://www.broadinstitute.org/gsea/msigdb/cards/LEE_METASTASIS_AND_ALTERNATIVE_SPLICING_UP 71 0.271937 0.973215 0.567568 0.873684 0 up vs 1MACLACHLAN_BRCA1_TARGETS_UP http://www.broadinstitute.org/gsea/msigdb/cards/MACLACHLAN_BRCA1_TARGETS_UP 21 0.30753 0.968998 0.519481 0.873919 0 up vs 1CEBALLOS_TARGETS_OF_TP53_AND_MYC_UP http://www.broadinstitute.org/gsea/msigdb/cards/CEBALLOS_TARGETS_OF_TP53_AND_MYC_UP 21 0.300705 0.972015 0.486111 0.873947 0 up vs 1VALK_AML_CLUSTER_9 http://www.broadinstitute.org/gsea/msigdb/cards/VALK_AML_CLUSTER_9 35 0.284855 0.967788 0.506667 0.874006 0 up vs 1LANDIS_ERBB2_BREAST_TUMORS_324_DN http://www.broadinstitute.org/gsea/msigdb/cards/LANDIS_ERBB2_BREAST_TUMORS_324_DN 148 0.254127 0.97226 0.588235 0.874301 0 up vs 1LUCAS_HNF4A_TARGETS_UP http://www.broadinstitute.org/gsea/msigdb/cards/LUCAS_HNF4A_TARGETS_UP 56 0.282408 0.96791 0.5125 0.87465 0 up vs 1WONG_ENDMETRIUM_CANCER_DN http://www.broadinstitute.org/gsea/msigdb/cards/WONG_ENDMETRIUM_CANCER_DN 78 0.315649 0.96937 0.410714 0.874736 0 up vs 1DAIRKEE_CANCER_PRONE_RESPONSE_BPA_E2 http://www.broadinstitute.org/gsea/msigdb/cards/DAIRKEE_CANCER_PRONE_RESPONSE_BPA_E2 114 0.255698 0.969031 0.595506 0.874773 0 up vs 1CLASPER_LYMPHATIC_VESSELS_DURING_METASTASIS_DN http://www.broadinstitute.org/gsea/msigdb/cards/CLASPER_LYMPHATIC_VESSELS_DURING_METASTASIS_DN 35 0.338848 0.969728 0.439394 0.874786 0 up vs 1LIU_CDX2_TARGETS_UP http://www.broadinstitute.org/gsea/msigdb/cards/LIU_CDX2_TARGETS_UP 36 0.280691 0.9636 0.564516 0.874898 0 up vs 1PLASARI_TGFB1_SIGNALING_VIA_NFIC_1HR_UP http://www.broadinstitute.org/gsea/msigdb/cards/PLASARI_TGFB1_SIGNALING_VIA_NFIC_1HR_UP 33 0.282845 0.972382 0.48 0.874989 0 up vs 1NIKOLSKY_BREAST_CANCER_7P22_AMPLICON http://www.broadinstitute.org/gsea/msigdb/cards/NIKOLSKY_BREAST_CANCER_7P22_AMPLICON 37 0.318883 0.968085 0.522388 0.875192 0 up vs 1LU_TUMOR_VASCULATURE_UP http://www.broadinstitute.org/gsea/msigdb/cards/LU_TUMOR_VASCULATURE_UP 29 0.288122 0.962221 0.536232 0.875363 0 up vs 1STEGER_ADIPOGENESIS_UP http://www.broadinstitute.org/gsea/msigdb/cards/STEGER_ADIPOGENESIS_UP 21 -0.221696 -0.717272 0.964286 0.875537 0 down vs 1WEBER_METHYLATED_LCP_IN_SPERM_UP http://www.broadinstitute.org/gsea/msigdb/cards/WEBER_METHYLATED_LCP_IN_SPERM_UP 15 0.323149 0.969776 0.446429 0.875635 0 up vs 1FIRESTEIN_CTNNB1_PATHWAY http://www.broadinstitute.org/gsea/msigdb/cards/FIRESTEIN_CTNNB1_PATHWAY 33 0.307069 0.961633 0.527778 0.875687 0 up vs 1GAUSSMANN_MLL_AF4_FUSION_TARGETS_F_DN http://www.broadinstitute.org/gsea/msigdb/cards/GAUSSMANN_MLL_AF4_FUSION_TARGETS_F_DN 32 -0.196138 -0.717915 0.892857 0.875796 0 down vs 1KOKKINAKIS_METHIONINE_DEPRIVATION_48HR_DN http://www.broadinstitute.org/gsea/msigdb/cards/KOKKINAKIS_METHIONINE_DEPRIVATION_48HR_DN 64 0.259515 0.962766 0.602564 0.876016 0 up vs 1PECE_MAMMARY_STEM_CELL_DN http://www.broadinstitute.org/gsea/msigdb/cards/PECE_MAMMARY_STEM_CELL_DN 137 0.2796 0.96224 0.5 0.876255 0 up vs 1RIGGINS_TAMOXIFEN_RESISTANCE_DN http://www.broadinstitute.org/gsea/msigdb/cards/RIGGINS_TAMOXIFEN_RESISTANCE_DN 216 0.261028 0.960193 0.597561 0.877283 0 up vs 1LEE_INTRATHYMIC_T_PROGENITOR http://www.broadinstitute.org/gsea/msigdb/cards/LEE_INTRATHYMIC_T_PROGENITOR 21 0.302743 0.955675 0.529412 0.877747 0 up vs 1PENG_GLUCOSE_DEPRIVATION_UP http://www.broadinstitute.org/gsea/msigdb/cards/PENG_GLUCOSE_DEPRIVATION_UP 46 0.259662 0.96039 0.5875 0.877758 0 up vs 1ROYLANCE_BREAST_CANCER_16Q_COPY_NUMBER_UP http://www.broadinstitute.org/gsea/msigdb/cards/ROYLANCE_BREAST_CANCER_16Q_COPY_NUMBER_UP 62 -0.197213 -0.711732 0.814815 0.878153 0 down vs 1VERRECCHIA_RESPONSE_TO_TGFB1_C2 http://www.broadinstitute.org/gsea/msigdb/cards/VERRECCHIA_RESPONSE_TO_TGFB1_C2 25 -0.262638 -0.712452 0.851852 0.878253 0 down vs 1DACOSTA_UV_RESPONSE_VIA_ERCC3_UP http://www.broadinstitute.org/gsea/msigdb/cards/DACOSTA_UV_RESPONSE_VIA_ERCC3_UP 307 0.244153 0.959318 0.590909 0.878434 0 up vs 1NING_CHRONIC_OBSTRUCTIVE_PULMONARY_DISEASE_UP http://www.broadinstitute.org/gsea/msigdb/cards/NING_CHRONIC_OBSTRUCTIVE_PULMONARY_DISEASE_UP 154 0.249044 0.955772 0.590361 0.878436 0 up vs 1CHEBOTAEV_GR_TARGETS_UP http://www.broadinstitute.org/gsea/msigdb/cards/CHEBOTAEV_GR_TARGETS_UP 74 0.285558 0.956105 0.5625 0.878589 0 up vs 1KYNG_ENVIRONMENTAL_STRESS_RESPONSE_UP http://www.broadinstitute.org/gsea/msigdb/cards/KYNG_ENVIRONMENTAL_STRESS_RESPONSE_UP 56 0.255524 0.954806 0.604651 0.878809 0 up vs 1NIKOLSKY_BREAST_CANCER_17Q21_Q25_AMPLICON http://www.broadinstitute.org/gsea/msigdb/cards/NIKOLSKY_BREAST_CANCER_17Q21_Q25_AMPLICON 327 0.255772 0.95428 0.623377 0.878974 0 up vs 1NAKAMURA_ADIPOGENESIS_LATE_DN http://www.broadinstitute.org/gsea/msigdb/cards/NAKAMURA_ADIPOGENESIS_LATE_DN 37 -0.200226 -0.71264 0.875 0.879014 0 down vs 1BRUECKNER_TARGETS_OF_MIRLET7A3_DN http://www.broadinstitute.org/gsea/msigdb/cards/BRUECKNER_TARGETS_OF_MIRLET7A3_DN 74 -0.203888 -0.713419 0.714286 0.879179 0 down vs 1ASTIER_INTEGRIN_SIGNALING http://www.broadinstitute.org/gsea/msigdb/cards/ASTIER_INTEGRIN_SIGNALING 56 0.254822 0.956207 0.621951 0.879254 0 up vs 1TOMIDA_METASTASIS_UP http://www.broadinstitute.org/gsea/msigdb/cards/TOMIDA_METASTASIS_UP 26 0.289718 0.949981 0.52381 0.879333 0 up vs 1GHANDHI_DIRECT_IRRADIATION_DN http://www.broadinstitute.org/gsea/msigdb/cards/GHANDHI_DIRECT_IRRADIATION_DN 33 0.27895 0.94946 0.597403 0.879463 0 up vs 1VARELA_ZMPSTE24_TARGETS_DN http://www.broadinstitute.org/gsea/msigdb/cards/VARELA_ZMPSTE24_TARGETS_DN 38 0.274985 0.953523 0.533333 0.879741 0 up vs 1MMS_MOUSE_LYMPH_HIGH_4HRS_UP http://www.broadinstitute.org/gsea/msigdb/cards/MMS_MOUSE_LYMPH_HIGH_4HRS_UP 35 0.302463 0.950226 0.445946 0.87975 0 up vs 1ZHAN_MULTIPLE_MYELOMA_CD1_AND_CD2_DN http://www.broadinstitute.org/gsea/msigdb/cards/ZHAN_MULTIPLE_MYELOMA_CD1_AND_CD2_DN 49 0.257368 0.950983 0.621951 0.879831 0 up vs 1STEGER_ADIPOGENESIS_DN http://www.broadinstitute.org/gsea/msigdb/cards/STEGER_ADIPOGENESIS_DN 25 0.311582 0.956699 0.507692 0.879967 0 up vs 1KYNG_DNA_DAMAGE_BY_4NQO_OR_GAMMA_RADIATION http://www.broadinstitute.org/gsea/msigdb/cards/KYNG_DNA_DAMAGE_BY_4NQO_OR_GAMMA_RADIATION 15 0.310359 0.956288 0.513158 0.880039 0 up vs 1BURTON_ADIPOGENESIS_10 http://www.broadinstitute.org/gsea/msigdb/cards/BURTON_ADIPOGENESIS_10 28 0.305146 0.957029 0.530864 0.88013 0 up vs 1BRACHAT_RESPONSE_TO_CISPLATIN http://www.broadinstitute.org/gsea/msigdb/cards/BRACHAT_RESPONSE_TO_CISPLATIN 21 0.304087 0.951246 0.477612 0.8802 0 up vs 1SYED_ESTRADIOL_RESPONSE http://www.broadinstitute.org/gsea/msigdb/cards/SYED_ESTRADIOL_RESPONSE 19 0.327452 0.950334 0.530864 0.880317 0 up vs 1MEISSNER_BRAIN_ICP_WITH_H3K4ME3 http://www.broadinstitute.org/gsea/msigdb/cards/MEISSNER_BRAIN_ICP_WITH_H3K4ME3 30 0.27851 0.952337 0.597222 0.880352 0 up vs 1VALK_AML_CLUSTER_15 http://www.broadinstitute.org/gsea/msigdb/cards/VALK_AML_CLUSTER_15 31 0.299179 0.957139 0.532468 0.8808 0 up vs 1GABRIELY_MIR21_TARGETS http://www.broadinstitute.org/gsea/msigdb/cards/GABRIELY_MIR21_TARGETS 284 0.298545 0.951331 0.48 0.88091 0 up vs 1DAVICIONI_RHABDOMYOSARCOMA_PAX_FOXO1_FUSION_UP http://www.broadinstitute.org/gsea/msigdb/cards/DAVICIONI_RHABDOMYOSARCOMA_PAX_FOXO1_FUSION_UP 60 0.261806 0.952433 0.589744 0.881148 0 up vs 1GRESHOCK_CANCER_COPY_NUMBER_UP http://www.broadinstitute.org/gsea/msigdb/cards/GRESHOCK_CANCER_COPY_NUMBER_UP 317 0.25361 0.957203 0.604938 0.881573 0 up vs 1ZHOU_TNF_SIGNALING_30MIN http://www.broadinstitute.org/gsea/msigdb/cards/ZHOU_TNF_SIGNALING_30MIN 53 0.275445 0.951433 0.623529 0.881605 0 up vs 1BENPORATH_NOS_TARGETS http://www.broadinstitute.org/gsea/msigdb/cards/BENPORATH_NOS_TARGETS 175 0.263203 0.957528 0.607595 0.881773 0 up vs 1WANG_NFKB_TARGETS http://www.broadinstitute.org/gsea/msigdb/cards/WANG_NFKB_TARGETS 25 0.205966 0.715052 0.853333 0.883422 0 up vs 1SWEET_KRAS_TARGETS_DN http://www.broadinstitute.org/gsea/msigdb/cards/SWEET_KRAS_TARGETS_DN 63 0.254999 0.946928 0.578313 0.883493 0 up vs 1YAO_TEMPORAL_RESPONSE_TO_PROGESTERONE_CLUSTER_2 http://www.broadinstitute.org/gsea/msigdb/cards/YAO_TEMPORAL_RESPONSE_TO_PROGESTERONE_CLUSTER_2 82 0.191309 0.742336 0.707317 0.883559 0 up vs 1BROWNE_HCMV_INFECTION_16HR_UP http://www.broadinstitute.org/gsea/msigdb/cards/BROWNE_HCMV_INFECTION_16HR_UP 222 0.200457 0.715464 0.802632 0.883596 0 up vs 1LI_WILMS_TUMOR_VS_FETAL_KIDNEY_1_UP http://www.broadinstitute.org/gsea/msigdb/cards/LI_WILMS_TUMOR_VS_FETAL_KIDNEY_1_UP 181 0.194452 0.721042 0.780488 0.883758 0 up vs 1CHIARADONNA_NEOPLASTIC_TRANSFORMATION_KRAS_DN http://www.broadinstitute.org/gsea/msigdb/cards/CHIARADONNA_NEOPLASTIC_TRANSFORMATION_KRAS_DN 142 0.18797 0.715907 0.7875 0.883793 0 up vs 1DELACROIX_RAR_TARGETS_DN http://www.broadinstitute.org/gsea/msigdb/cards/DELACROIX_RAR_TARGETS_DN 24 0.227063 0.737706 0.819444 0.883897 0 up vs 1MATZUK_CENTRAL_FOR_FEMALE_FERTILITY http://www.broadinstitute.org/gsea/msigdb/cards/MATZUK_CENTRAL_FOR_FEMALE_FERTILITY 29 0.208935 0.742589 0.90411 0.883908 0 up vs 1GENTILE_UV_RESPONSE_CLUSTER_D2 http://www.broadinstitute.org/gsea/msigdb/cards/GENTILE_UV_RESPONSE_CLUSTER_D2 41 0.323031 0.947114 0.5 0.883945 0 up vs 1VALK_AML_CLUSTER_7 http://www.broadinstitute.org/gsea/msigdb/cards/VALK_AML_CLUSTER_7 27 0.215134 0.716335 0.910448 0.884007 0 up vs 1GAVIN_IL2_RESPONSIVE_FOXP3_TARGETS_UP http://www.broadinstitute.org/gsea/msigdb/cards/GAVIN_IL2_RESPONSIVE_FOXP3_TARGETS_UP 18 0.276046 0.787198 0.661765 0.884261 0 up vs 1BILD_CTNNB1_ONCOGENIC_SIGNATURE http://www.broadinstitute.org/gsea/msigdb/cards/BILD_CTNNB1_ONCOGENIC_SIGNATURE 77 0.232144 0.721192 0.791667 0.884264 0 up vs 1EBAUER_TARGETS_OF_PAX3_FOXO1_FUSION_UP http://www.broadinstitute.org/gsea/msigdb/cards/EBAUER_TARGETS_OF_PAX3_FOXO1_FUSION_UP 206 0.188273 0.737966 0.78481 0.884296 0 up vs 1PARK_HSC_VS_MULTIPOTENT_PROGENITORS_UP http://www.broadinstitute.org/gsea/msigdb/cards/PARK_HSC_VS_MULTIPOTENT_PROGENITORS_UP 19 0.241317 0.741121 0.831169 0.884341 0 up vs 1JOHANSSON_GLIOMAGENESIS_BY_PDGFB_DN http://www.broadinstitute.org/gsea/msigdb/cards/JOHANSSON_GLIOMAGENESIS_BY_PDGFB_DN 21 0.231664 0.716584 0.830769 0.884346 0 up vs 1YAMASHITA_METHYLATED_IN_PROSTATE_CANCER http://www.broadinstitute.org/gsea/msigdb/cards/YAMASHITA_METHYLATED_IN_PROSTATE_CANCER 56 0.195051 0.719764 0.844156 0.884356 0 up vs 1NAKAMURA_CANCER_MICROENVIRONMENT_UP http://www.broadinstitute.org/gsea/msigdb/cards/NAKAMURA_CANCER_MICROENVIRONMENT_UP 24 0.293638 0.945162 0.640625 0.884411 0 up vs 1NOUZOVA_TRETINOIN_AND_H4_ACETYLATION http://www.broadinstitute.org/gsea/msigdb/cards/NOUZOVA_TRETINOIN_AND_H4_ACETYLATION 137 0.208383 0.742661 0.809524 0.884462 0 up vs 1MCCLUNG_COCAIN_REWARD_4WK http://www.broadinstitute.org/gsea/msigdb/cards/MCCLUNG_COCAIN_REWARD_4WK 74 0.213462 0.788149 0.797297 0.884501 0 up vs 1YAGI_AML_WITH_T_9_11_TRANSLOCATION http://www.broadinstitute.org/gsea/msigdb/cards/YAGI_AML_WITH_T_9_11_TRANSLOCATION 128 0.245465 0.945441 0.609756 0.884534 0 up vs 1HAN_SATB1_TARGETS_UP http://www.broadinstitute.org/gsea/msigdb/cards/HAN_SATB1_TARGETS_UP 385 0.189123 0.787526 0.696629 0.884569 0 up vs 1HU_GENOTOXIC_DAMAGE_4HR http://www.broadinstitute.org/gsea/msigdb/cards/HU_GENOTOXIC_DAMAGE_4HR 35 0.260148 0.739509 0.837838 0.884644 0 up vs 1HOEBEKE_LYMPHOID_STEM_CELL_DN http://www.broadinstitute.org/gsea/msigdb/cards/HOEBEKE_LYMPHOID_STEM_CELL_DN 84 0.237165 0.940931 0.55814 0.884677 0 up vs 1BURTON_ADIPOGENESIS_11 http://www.broadinstitute.org/gsea/msigdb/cards/BURTON_ADIPOGENESIS_11 54 0.226297 0.716799 0.797297 0.884714 0 up vs 1LEE_LIVER_CANCER_SURVIVAL_DN http://www.broadinstitute.org/gsea/msigdb/cards/LEE_LIVER_CANCER_SURVIVAL_DN 169 0.226827 0.738104 0.848837 0.884795 0 up vs 1BIDUS_METASTASIS_UP http://www.broadinstitute.org/gsea/msigdb/cards/BIDUS_METASTASIS_UP 211 0.243709 0.721237 0.805195 0.884833 0 up vs 1JAZAERI_BREAST_CANCER_BRCA1_VS_BRCA2_UP http://www.broadinstitute.org/gsea/msigdb/cards/JAZAERI_BREAST_CANCER_BRCA1_VS_BRCA2_UP 49 0.269671 0.944481 0.60241 0.884896 0 up vs 1WHITFIELD_CELL_CYCLE_G2_M http://www.broadinstitute.org/gsea/msigdb/cards/WHITFIELD_CELL_CYCLE_G2_M 208 0.218162 0.744151 0.777778 0.884905 0 up vs 1NADERI_BREAST_CANCER_PROGNOSIS_DN http://www.broadinstitute.org/gsea/msigdb/cards/NADERI_BREAST_CANCER_PROGNOSIS_DN 18 -0.237025 -0.704643 0.828571 0.884941 0 down vs 1BRUECKNER_TARGETS_OF_MIRLET7A3_UP http://www.broadinstitute.org/gsea/msigdb/cards/BRUECKNER_TARGETS_OF_MIRLET7A3_UP 105 0.208776 0.788378 0.782051 0.884957 0 up vs 1DACOSTA_UV_RESPONSE_VIA_ERCC3_TTD_DN http://www.broadinstitute.org/gsea/msigdb/cards/DACOSTA_UV_RESPONSE_VIA_ERCC3_TTD_DN 82 0.23848 0.742788 0.828571 0.884987 0 up vs 1LEE_LIVER_CANCER_ACOX1_DN http://www.broadinstitute.org/gsea/msigdb/cards/LEE_LIVER_CANCER_ACOX1_DN 65 0.187608 0.717744 0.846154 0.884987 0 up vs 1MAINA_VHL_TARGETS_DN http://www.broadinstitute.org/gsea/msigdb/cards/MAINA_VHL_TARGETS_DN 18 0.242031 0.739686 0.895522 0.885063 0 up vs 1MCBRYAN_PUBERTAL_BREAST_5_6WK_UP http://www.broadinstitute.org/gsea/msigdb/cards/MCBRYAN_PUBERTAL_BREAST_5_6WK_UP 115 0.191284 0.71699 0.825581 0.885146 0 up vs 1CAIRO_HEPATOBLASTOMA_DN http://www.broadinstitute.org/gsea/msigdb/cards/CAIRO_HEPATOBLASTOMA_DN 259 0.188524 0.751397 0.74359 0.885206 0 up vs 1SEMENZA_HIF1_TARGETS http://www.broadinstitute.org/gsea/msigdb/cards/SEMENZA_HIF1_TARGETS 35 0.21883 0.744505 0.791045 0.885233 0 up vs 1CHEOK_RESPONSE_TO_MERCAPTOPURINE_AND_LD_MTX_DN http://www.broadinstitute.org/gsea/msigdb/cards/CHEOK_RESPONSE_TO_MERCAPTOPURINE_AND_LD_MTX_DN 20 0.288968 0.939888 0.521127 0.885278 0 up vs 1LEE_RECENT_THYMIC_EMIGRANT http://www.broadinstitute.org/gsea/msigdb/cards/LEE_RECENT_THYMIC_EMIGRANT 215 0.208625 0.788625 0.724138 0.885291 0 up vs 1LIU_VAV3_PROSTATE_CARCINOGENESIS_DN http://www.broadinstitute.org/gsea/msigdb/cards/LIU_VAV3_PROSTATE_CARCINOGENESIS_DN 16 0.258933 0.750614 0.883333 0.885335 0 up vs 1IGLESIAS_E2F_TARGETS_DN http://www.broadinstitute.org/gsea/msigdb/cards/IGLESIAS_E2F_TARGETS_DN 15 0.284489 0.945461 0.54717 0.885348 0 up vs 1ZHAN_MULTIPLE_MYELOMA_MF_UP http://www.broadinstitute.org/gsea/msigdb/cards/ZHAN_MULTIPLE_MYELOMA_MF_UP 46 0.261565 0.94141 0.594203 0.885377 0 up vs 1NELSON_RESPONSE_TO_ANDROGEN_UP http://www.broadinstitute.org/gsea/msigdb/cards/NELSON_RESPONSE_TO_ANDROGEN_UP 86 0.187707 0.721286 0.756098 0.885403 0 up vs 1MITSIADES_RESPONSE_TO_APLIDIN_DN http://www.broadinstitute.org/gsea/msigdb/cards/MITSIADES_RESPONSE_TO_APLIDIN_DN 244 0.234761 0.738108 0.7875 0.885411 0 up vs 1HOFMANN_MYELODYSPLASTIC_SYNDROM_LOW_RISK_UP http://www.broadinstitute.org/gsea/msigdb/cards/HOFMANN_MYELODYSPLASTIC_SYNDROM_LOW_RISK_UP 22 0.293293 0.939427 0.587302 0.885432 0 up vs 1BROWNE_HCMV_INFECTION_10HR_DN http://www.broadinstitute.org/gsea/msigdb/cards/BROWNE_HCMV_INFECTION_10HR_DN 55 0.286016 0.94183 0.532468 0.885435 0 up vs 1CHAUHAN_RESPONSE_TO_METHOXYESTRADIOL_DN http://www.broadinstitute.org/gsea/msigdb/cards/CHAUHAN_RESPONSE_TO_METHOXYESTRADIOL_DN 99 0.256073 0.945741 0.573171 0.885456 0 up vs 1NIKOLSKY_BREAST_CANCER_16Q24_AMPLICON http://www.broadinstitute.org/gsea/msigdb/cards/NIKOLSKY_BREAST_CANCER_16Q24_AMPLICON 48 0.251981 0.717842 0.776119 0.885504 0 up vs 1MIYAGAWA_TARGETS_OF_EWSR1_ETS_FUSIONS_UP http://www.broadinstitute.org/gsea/msigdb/cards/MIYAGAWA_TARGETS_OF_EWSR1_ETS_FUSIONS_UP 250 0.191816 0.751785 0.722892 0.885507 0 up vs 1STAMBOLSKY_RESPONSE_TO_VITAMIN_D3_UP http://www.broadinstitute.org/gsea/msigdb/cards/STAMBOLSKY_RESPONSE_TO_VITAMIN_D3_UP 80 0.199959 0.742929 0.746835 0.885513 0 up vs 1SMID_BREAST_CANCER_RELAPSE_IN_LUNG_DN http://www.broadinstitute.org/gsea/msigdb/cards/SMID_BREAST_CANCER_RELAPSE_IN_LUNG_DN 37 0.301325 0.940949 0.52459 0.885515 0 up vs 1MORI_SMALL_PRE_BII_LYMPHOCYTE_DN http://www.broadinstitute.org/gsea/msigdb/cards/MORI_SMALL_PRE_BII_LYMPHOCYTE_DN 76 0.20762 0.789007 0.786517 0.885532 0 up vs 1PICCALUGA_ANGIOIMMUNOBLASTIC_LYMPHOMA_UP http://www.broadinstitute.org/gsea/msigdb/cards/PICCALUGA_ANGIOIMMUNOBLASTIC_LYMPHOMA_UP 202 0.22989 0.73232 0.757576 0.885553 0 up vs 1ZUCCHI_METASTASIS_UP http://www.broadinstitute.org/gsea/msigdb/cards/ZUCCHI_METASTASIS_UP 41 0.226668 0.744794 0.827586 0.885608 0 up vs 1SAKAI_CHRONIC_HEPATITIS_VS_LIVER_CANCER_UP http://www.broadinstitute.org/gsea/msigdb/cards/SAKAI_CHRONIC_HEPATITIS_VS_LIVER_CANCER_UP 80 0.302411 0.942112 0.487179 0.885615 0 up vs 1ASTON_MAJOR_DEPRESSIVE_DISORDER_DN http://www.broadinstitute.org/gsea/msigdb/cards/ASTON_MAJOR_DEPRESSIVE_DISORDER_DN 156 0.202473 0.781365 0.708861 0.885643 0 up vs 1SETLUR_PROSTATE_CANCER_TMPRSS2_ERG_FUSION_DN http://www.broadinstitute.org/gsea/msigdb/cards/SETLUR_PROSTATE_CANCER_TMPRSS2_ERG_FUSION_DN 19 0.243341 0.782509 0.8 0.885644 0 up vs 1CREIGHTON_ENDOCRINE_THERAPY_RESISTANCE_5 http://www.broadinstitute.org/gsea/msigdb/cards/CREIGHTON_ENDOCRINE_THERAPY_RESISTANCE_5 459 0.189477 0.709313 0.776471 0.885673 0 up vs 1DITTMER_PTHLH_TARGETS_UP http://www.broadinstitute.org/gsea/msigdb/cards/DITTMER_PTHLH_TARGETS_UP 111 0.22128 0.783655 0.744186 0.885676 0 up vs 1ZHENG_BOUND_BY_FOXP3 http://www.broadinstitute.org/gsea/msigdb/cards/ZHENG_BOUND_BY_FOXP3 480 0.189578 0.700251 0.731707 0.8857 0 up vs 1WAMUNYOKOLI_OVARIAN_CANCER_GRADES_1_2_UP http://www.broadinstitute.org/gsea/msigdb/cards/WAMUNYOKOLI_OVARIAN_CANCER_GRADES_1_2_UP 134 0.227672 0.70865 0.833333 0.88574 0 up vs 1HAN_SATB1_TARGETS_DN http://www.broadinstitute.org/gsea/msigdb/cards/HAN_SATB1_TARGETS_DN 433 0.189352 0.734591 0.771084 0.885769 0 up vs 1MOOTHA_GLYCOLYSIS http://www.broadinstitute.org/gsea/msigdb/cards/MOOTHA_GLYCOLYSIS 20 0.231447 0.745917 0.84507 0.885773 0 up vs 1YORDY_RECIPROCAL_REGULATION_BY_ETS1_AND_SP100_UP http://www.broadinstitute.org/gsea/msigdb/cards/YORDY_RECIPROCAL_REGULATION_BY_ETS1_AND_SP100_UP 21 0.238376 0.728243 0.92 0.885786 0 up vs 1IWANAGA_CARCINOGENESIS_BY_KRAS_DN http://www.broadinstitute.org/gsea/msigdb/cards/IWANAGA_CARCINOGENESIS_BY_KRAS_DN 119 0.187862 0.722115 0.804878 0.885792 0 up vs 1WU_SILENCED_BY_METHYLATION_IN_BLADDER_CANCER http://www.broadinstitute.org/gsea/msigdb/cards/WU_SILENCED_BY_METHYLATION_IN_BLADDER_CANCER 55 0.231124 0.784697 0.776119 0.885806 0 up vs 1DARWICHE_PAPILLOMA_RISK_LOW_UP http://www.broadinstitute.org/gsea/msigdb/cards/DARWICHE_PAPILLOMA_RISK_LOW_UP 159 0.18805 0.789287 0.698925 0.885824 0 up vs 1DARWICHE_PAPILLOMA_RISK_HIGH_VS_LOW_DN http://www.broadinstitute.org/gsea/msigdb/cards/DARWICHE_PAPILLOMA_RISK_HIGH_VS_LOW_DN 30 0.275519 0.942495 0.605634 0.885828 0 up vs 1MONNIER_POSTRADIATION_TUMOR_ESCAPE_DN http://www.broadinstitute.org/gsea/msigdb/cards/MONNIER_POSTRADIATION_TUMOR_ESCAPE_DN 360 0.189004 0.733248 0.776471 0.885831 0 up vs 1MULLIGHAN_NPM1_MUTATED_SIGNATURE_2_UP http://www.broadinstitute.org/gsea/msigdb/cards/MULLIGHAN_NPM1_MUTATED_SIGNATURE_2_UP 131 0.203202 0.729405 0.828947 0.885839 0 up vs 1HOFFMANN_IMMATURE_TO_MATURE_B_LYMPHOCYTE_DN http://www.broadinstitute.org/gsea/msigdb/cards/HOFFMANN_IMMATURE_TO_MATURE_B_LYMPHOCYTE_DN 50 0.187538 0.711097 0.891566 0.885844 0 up vs 1PEREZ_TP63_TARGETS http://www.broadinstitute.org/gsea/msigdb/cards/PEREZ_TP63_TARGETS 338 0.240905 0.940023 0.584416 0.885855 0 up vs 1DOUGLAS_BMI1_TARGETS_DN http://www.broadinstitute.org/gsea/msigdb/cards/DOUGLAS_BMI1_TARGETS_DN 309 0.214927 0.778197 0.75 0.885866 0 up vs 1VALK_AML_CLUSTER_1 http://www.broadinstitute.org/gsea/msigdb/cards/VALK_AML_CLUSTER_1 28 0.216758 0.728675 0.825397 0.88589 0 up vs 1MIKKELSEN_ES_LCP_WITH_H3K4ME3 http://www.broadinstitute.org/gsea/msigdb/cards/MIKKELSEN_ES_LCP_WITH_H3K4ME3 135 0.187937 0.725779 0.807692 0.885893 0 up vs 1STEARMAN_LUNG_CANCER_EARLY_VS_LATE_DN http://www.broadinstitute.org/gsea/msigdb/cards/STEARMAN_LUNG_CANCER_EARLY_VS_LATE_DN 59 0.219606 0.745145 0.813333 0.885902 0 up vs 1AKL_HTLV1_INFECTION_DN http://www.broadinstitute.org/gsea/msigdb/cards/AKL_HTLV1_INFECTION_DN 68 0.220953 0.735014 0.771429 0.885972 0 up vs 1DACOSTA_UV_RESPONSE_VIA_ERCC3_XPCS_DN http://www.broadinstitute.org/gsea/msigdb/cards/DACOSTA_UV_RESPONSE_VIA_ERCC3_XPCS_DN 85 0.256457 0.782796 0.757576 0.885976 0 up vs 1THEILGAARD_NEUTROPHIL_AT_SKIN_WOUND_UP http://www.broadinstitute.org/gsea/msigdb/cards/THEILGAARD_NEUTROPHIL_AT_SKIN_WOUND_UP 76 0.209925 0.732544 0.769231 0.885981 0 up vs 1WANG_RECURRENT_LIVER_CANCER_UP http://www.broadinstitute.org/gsea/msigdb/cards/WANG_RECURRENT_LIVER_CANCER_UP 20 0.237851 0.710209 0.857143 0.886013 0 up vs 1LEE_LIVER_CANCER_E2F1_DN http://www.broadinstitute.org/gsea/msigdb/cards/LEE_LIVER_CANCER_E2F1_DN 62 0.190321 0.721291 0.881579 0.886019 0 up vs 1VANDESLUIS_COMMD1_TARGETS_GROUP_3_UP http://www.broadinstitute.org/gsea/msigdb/cards/VANDESLUIS_COMMD1_TARGETS_GROUP_3_UP 87 0.256075 0.9387 0.605634 0.886039 0 up vs 1FINAK_BREAST_CANCER_SDPP_SIGNATURE http://www.broadinstitute.org/gsea/msigdb/cards/FINAK_BREAST_CANCER_SDPP_SIGNATURE 25 0.228641 0.707681 0.822581 0.886055 0 up vs 1BAELDE_DIABETIC_NEPHROPATHY_DN http://www.broadinstitute.org/gsea/msigdb/cards/BAELDE_DIABETIC_NEPHROPATHY_DN 424 0.205516 0.781556 0.752941 0.886073 0 up vs 1SHETH_LIVER_CANCER_VS_TXNIP_LOSS_PAM2 http://www.broadinstitute.org/gsea/msigdb/cards/SHETH_LIVER_CANCER_VS_TXNIP_LOSS_PAM2 149 0.188003 0.75184 0.727273 0.886092 0 up vs 1KYNG_DNA_DAMAGE_DN http://www.broadinstitute.org/gsea/msigdb/cards/KYNG_DNA_DAMAGE_DN 195 0.188221 0.778519 0.726316 0.886111 0 up vs 1GAUSSMANN_MLL_AF4_FUSION_TARGETS_E_UP http://www.broadinstitute.org/gsea/msigdb/cards/GAUSSMANN_MLL_AF4_FUSION_TARGETS_E_UP 92 0.187735 0.726692 0.746835 0.886112 0 up vs 1LIM_MAMMARY_STEM_CELL_UP http://www.broadinstitute.org/gsea/msigdb/cards/LIM_MAMMARY_STEM_CELL_UP 468 0.21431 0.726144 0.771429 0.886129 0 up vs 1GRADE_COLON_CANCER_DN http://www.broadinstitute.org/gsea/msigdb/cards/GRADE_COLON_CANCER_DN 32 0.242929 0.777305 0.794872 0.886158 0 up vs 1LIN_TUMOR_ESCAPE_FROM_IMMUNE_ATTACK http://www.broadinstitute.org/gsea/msigdb/cards/LIN_TUMOR_ESCAPE_FROM_IMMUNE_ATTACK 16 0.241821 0.758941 0.838235 0.886204 0 up vs 1MARTIN_INTERACT_WITH_HDAC http://www.broadinstitute.org/gsea/msigdb/cards/MARTIN_INTERACT_WITH_HDAC 44 0.234844 0.783736 0.765432 0.886205 0 up vs 1MOREIRA_RESPONSE_TO_TSA_UP http://www.broadinstitute.org/gsea/msigdb/cards/MOREIRA_RESPONSE_TO_TSA_UP 28 0.244786 0.766954 0.782609 0.886209 0 up vs 1WESTON_VEGFA_TARGETS http://www.broadinstitute.org/gsea/msigdb/cards/WESTON_VEGFA_TARGETS 106 0.191694 0.702282 0.810811 0.88625 0 up vs 1DACOSTA_ERCC3_ALLELE_XPCS_VS_TTD_UP http://www.broadinstitute.org/gsea/msigdb/cards/DACOSTA_ERCC3_ALLELE_XPCS_VS_TTD_UP 28 0.219971 0.78487 0.823529 0.886256 0 up vs 1SATO_SILENCED_BY_DEACETYLATION_IN_PANCREATIC_CANCER http://www.broadinstitute.org/gsea/msigdb/cards/SATO_SILENCED_BY_DEACETYLATION_IN_PANCREATIC_CANCER 47 0.187523 0.70934 0.833333 0.88626 0 up vs 1WILLIAMS_ESR2_TARGETS_UP http://www.broadinstitute.org/gsea/msigdb/cards/WILLIAMS_ESR2_TARGETS_UP 26 0.248876 0.760591 0.823529 0.88629 0 up vs 1KIM_MYCL1_AMPLIFICATION_TARGETS_DN http://www.broadinstitute.org/gsea/msigdb/cards/KIM_MYCL1_AMPLIFICATION_TARGETS_DN 20 0.257505 0.746014 0.819444 0.886308 0 up vs 1YAO_HOXA10_TARGETS_VIA_PROGESTERONE_UP http://www.broadinstitute.org/gsea/msigdb/cards/YAO_HOXA10_TARGETS_VIA_PROGESTERONE_UP 77 0.187664 0.70026 0.819444 0.886309 0 up vs 1LEIN_CEREBELLUM_MARKERS http://www.broadinstitute.org/gsea/msigdb/cards/LEIN_CEREBELLUM_MARKERS 80 0.187678 0.698902 0.896104 0.886357 0 up vs 1WENG_POR_TARGETS_GLOBAL_DN http://www.broadinstitute.org/gsea/msigdb/cards/WENG_POR_TARGETS_GLOBAL_DN 23 0.223353 0.729582 0.821918 0.886364 0 up vs 1SENESE_HDAC1_AND_HDAC2_TARGETS_UP http://www.broadinstitute.org/gsea/msigdb/cards/SENESE_HDAC1_AND_HDAC2_TARGETS_UP 232 0.188396 0.696938 0.87013 0.886365 0 up vs 1WESTON_VEGFA_TARGETS_6HR http://www.broadinstitute.org/gsea/msigdb/cards/WESTON_VEGFA_TARGETS_6HR 58 0.225395 0.757684 0.849315 0.886374 0 up vs 1DAZARD_RESPONSE_TO_UV_NHEK_UP http://www.broadinstitute.org/gsea/msigdb/cards/DAZARD_RESPONSE_TO_UV_NHEK_UP 241 0.188438 0.733328 0.806818 0.886387 0 up vs 1NAKAMURA_TUMOR_ZONE_PERIPHERAL_VS_CENTRAL_UP http://www.broadinstitute.org/gsea/msigdb/cards/NAKAMURA_TUMOR_ZONE_PERIPHERAL_VS_CENTRAL_UP 276 0.195473 0.722139 0.829545 0.886404 0 up vs 1ELVIDGE_HIF1A_TARGETS_DN http://www.broadinstitute.org/gsea/msigdb/cards/ELVIDGE_HIF1A_TARGETS_DN 90 0.192591 0.711135 0.823529 0.886426 0 up vs 1MCCLUNG_DELTA_FOSB_TARGETS_2WK http://www.broadinstitute.org/gsea/msigdb/cards/MCCLUNG_DELTA_FOSB_TARGETS_2WK 45 0.211433 0.746584 0.8 0.88645 0 up vs 1SMID_BREAST_CANCER_RELAPSE_IN_BONE_UP http://www.broadinstitute.org/gsea/msigdb/cards/SMID_BREAST_CANCER_RELAPSE_IN_BONE_UP 95 0.224217 0.789342 0.787879 0.886456 0 up vs 1RUAN_RESPONSE_TO_TROGLITAZONE_UP http://www.broadinstitute.org/gsea/msigdb/cards/RUAN_RESPONSE_TO_TROGLITAZONE_UP 24 0.246154 0.789852 0.788732 0.886469 0 up vs 1SUNG_METASTASIS_STROMA_UP http://www.broadinstitute.org/gsea/msigdb/cards/SUNG_METASTASIS_STROMA_UP 108 0.206416 0.758182 0.731707 0.886477 0 up vs 1WHITEHURST_PACLITAXEL_SENSITIVITY http://www.broadinstitute.org/gsea/msigdb/cards/WHITEHURST_PACLITAXEL_SENSITIVITY 39 0.209359 0.762991 0.8125 0.886522 0 up vs 1WALLACE_JAK2_TARGETS_UP http://www.broadinstitute.org/gsea/msigdb/cards/WALLACE_JAK2_TARGETS_UP 25 0.236713 0.702611 0.859155 0.886531 0 up vs 1TURASHVILI_BREAST_LOBULAR_CARCINOMA_VS_DUCTAL_NORMAL_UP http://www.broadinstitute.org/gsea/msigdb/cards/TURASHVILI_BREAST_LOBULAR_CARCINOMA_VS_DUCTAL_NORMAL_UP 69 0.233603 0.701445 0.772727 0.88659 0 up vs 1ELVIDGE_HIF1A_AND_HIF2A_TARGETS_DN http://www.broadinstitute.org/gsea/msigdb/cards/ELVIDGE_HIF1A_AND_HIF2A_TARGETS_DN 103 0.187787 0.696042 0.808219 0.886598 0 up vs 1CHIBA_RESPONSE_TO_TSA_DN http://www.broadinstitute.org/gsea/msigdb/cards/CHIBA_RESPONSE_TO_TSA_DN 23 0.263076 0.726823 0.769231 0.886607 0 up vs 1WANG_LMO4_TARGETS_DN http://www.broadinstitute.org/gsea/msigdb/cards/WANG_LMO4_TARGETS_DN 338 0.210401 0.767214 0.776471 0.886626 0 up vs 1MATZUK_EMBRYONIC_GERM_CELL http://www.broadinstitute.org/gsea/msigdb/cards/MATZUK_EMBRYONIC_GERM_CELL 19 0.253331 0.759095 0.777778 0.88667 0 up vs 1BRUINS_UVC_RESPONSE_VIA_TP53_GROUP_C http://www.broadinstitute.org/gsea/msigdb/cards/BRUINS_UVC_RESPONSE_VIA_TP53_GROUP_C 89 0.187721 0.761524 0.795181 0.886675 0 up vs 1CADWELL_ATG16L1_TARGETS_UP http://www.broadinstitute.org/gsea/msigdb/cards/CADWELL_ATG16L1_TARGETS_UP 90 0.194809 0.760895 0.794872 0.88668 0 up vs 1NIELSEN_LEIOMYOSARCOMA_CNN1_UP http://www.broadinstitute.org/gsea/msigdb/cards/NIELSEN_LEIOMYOSARCOMA_CNN1_UP 19 0.335239 0.751873 0.612903 0.88669 0 up vs 1WU_ALZHEIMER_DISEASE_DN http://www.broadinstitute.org/gsea/msigdb/cards/WU_ALZHEIMER_DISEASE_DN 18 0.239363 0.729907 0.890625 0.8867 0 up vs 1NOUSHMEHR_GBM_SILENCED_BY_METHYLATION http://www.broadinstitute.org/gsea/msigdb/cards/NOUSHMEHR_GBM_SILENCED_BY_METHYLATION 47 0.211269 0.778634 0.75 0.886705 0 up vs 1ZHANG_RESPONSE_TO_IKK_INHIBITOR_AND_TNF_DN http://www.broadinstitute.org/gsea/msigdb/cards/ZHANG_RESPONSE_TO_IKK_INHIBITOR_AND_TNF_DN 101 0.217821 0.759638 0.78481 0.886713 0 up vs 1BEIER_GLIOMA_STEM_CELL_UP http://www.broadinstitute.org/gsea/msigdb/cards/BEIER_GLIOMA_STEM_CELL_UP 36 0.258195 0.942497 0.614286 0.886721 0 up vs 1BRUNO_HEMATOPOIESIS http://www.broadinstitute.org/gsea/msigdb/cards/BRUNO_HEMATOPOIESIS 65 0.187608 0.697189 0.888889 0.886728 0 up vs 1HERNANDEZ_ABERRANT_MITOSIS_BY_DOCETACEL_2NM_DN http://www.broadinstitute.org/gsea/msigdb/cards/HERNANDEZ_ABERRANT_MITOSIS_BY_DOCETACEL_2NM_DN 25 0.23388 0.77469 0.828125 0.886739 0 up vs 1ZHANG_RESPONSE_TO_CANTHARIDIN_DN http://www.broadinstitute.org/gsea/msigdb/cards/ZHANG_RESPONSE_TO_CANTHARIDIN_DN 67 0.261784 0.755995 0.84 0.886818 0 up vs 1RUTELLA_RESPONSE_TO_HGF_VS_CSF2RB_AND_IL4_UP http://www.broadinstitute.org/gsea/msigdb/cards/RUTELLA_RESPONSE_TO_HGF_VS_CSF2RB_AND_IL4_UP 395 0.189171 0.700316 0.813333 0.886874 0 up vs 1RODWELL_AGING_KIDNEY_NO_BLOOD_UP http://www.broadinstitute.org/gsea/msigdb/cards/RODWELL_AGING_KIDNEY_NO_BLOOD_UP 214 0.202805 0.763253 0.746835 0.886894 0 up vs 1TURASHVILI_BREAST_CARCINOMA_DUCTAL_VS_LOBULAR_UP http://www.broadinstitute.org/gsea/msigdb/cards/TURASHVILI_BREAST_CARCINOMA_DUCTAL_VS_LOBULAR_UP 20 0.313979 0.936651 0.575758 0.886939 0 up vs 1SUZUKI_RESPONSE_TO_TSA http://www.broadinstitute.org/gsea/msigdb/cards/SUZUKI_RESPONSE_TO_TSA 20 0.259271 0.790035 0.777778 0.886942 0 up vs 1MUNSHI_MULTIPLE_MYELOMA_UP http://www.broadinstitute.org/gsea/msigdb/cards/MUNSHI_MULTIPLE_MYELOMA_UP 81 0.187683 0.697632 0.855422 0.88698 0 up vs 1SMIRNOV_RESPONSE_TO_IR_2HR_DN http://www.broadinstitute.org/gsea/msigdb/cards/SMIRNOV_RESPONSE_TO_IR_2HR_DN 54 0.216129 0.730236 0.768116 0.886995 0 up vs 1URS_ADIPOCYTE_DIFFERENTIATION_DN http://www.broadinstitute.org/gsea/msigdb/cards/URS_ADIPOCYTE_DIFFERENTIATION_DN 30 0.217161 0.72216 0.830986 0.887005 0 up vs 1KOYAMA_SEMA3B_TARGETS_UP http://www.broadinstitute.org/gsea/msigdb/cards/KOYAMA_SEMA3B_TARGETS_UP 277 0.188609 0.746637 0.719512 0.88701 0 up vs 1TURASHVILI_BREAST_LOBULAR_CARCINOMA_VS_LOBULAR_NORMAL_UP http://www.broadinstitute.org/gsea/msigdb/cards/TURASHVILI_BREAST_LOBULAR_CARCINOMA_VS_LOBULAR_NORMAL_UP 94 0.187744 0.702729 0.871795 0.887041 0 up vs 1HELLER_HDAC_TARGETS_UP http://www.broadinstitute.org/gsea/msigdb/cards/HELLER_HDAC_TARGETS_UP 302 0.188728 0.767354 0.690476 0.887091 0 up vs 1OUELLET_OVARIAN_CANCER_INVASIVE_VS_LMP_UP http://www.broadinstitute.org/gsea/msigdb/cards/OUELLET_OVARIAN_CANCER_INVASIVE_VS_LMP_UP 117 0.240469 0.767935 0.802469 0.887099 0 up vs 1GARGALOVIC_RESPONSE_TO_OXIDIZED_PHOSPHOLIPIDS_TURQUOISE_DN http://www.broadinstitute.org/gsea/msigdb/cards/GARGALOVIC_RESPONSE_TO_OXIDIZED_PHOSPHOLIPIDS_TURQUOISE_DN 51 0.254718 0.75641 0.810127 0.887129 0 up vs 1LU_AGING_BRAIN_UP http://www.broadinstitute.org/gsea/msigdb/cards/LU_AGING_BRAIN_UP 258 0.199232 0.761726 0.72619 0.887167 0 up vs 1BREDEMEYER_RAG_SIGNALING_VIA_ATM_NOT_VIA_NFKB_DN http://www.broadinstitute.org/gsea/msigdb/cards/BREDEMEYER_RAG_SIGNALING_VIA_ATM_NOT_VIA_NFKB_DN 37 0.28829 0.942658 0.549296 0.887173 0 up vs 1BREDEMEYER_RAG_SIGNALING_VIA_ATM_NOT_VIA_NFKB_UP http://www.broadinstitute.org/gsea/msigdb/cards/BREDEMEYER_RAG_SIGNALING_VIA_ATM_NOT_VIA_NFKB_UP 47 0.191104 0.70586 0.860759 0.887178 0 up vs 1MCBRYAN_PUBERTAL_TGFB1_TARGETS_UP http://www.broadinstitute.org/gsea/msigdb/cards/MCBRYAN_PUBERTAL_TGFB1_TARGETS_UP 169 0.191772 0.694679 0.8125 0.887213 0 up vs 1WANG_CLIM2_TARGETS_DN http://www.broadinstitute.org/gsea/msigdb/cards/WANG_CLIM2_TARGETS_DN 179 0.217074 0.778723 0.741176 0.887275 0 up vs 1IVANOVA_HEMATOPOIESIS_MATURE_CELL http://www.broadinstitute.org/gsea/msigdb/cards/IVANOVA_HEMATOPOIESIS_MATURE_CELL 277 0.188609 0.751895 0.727273 0.887319 0 up vs 1RAMALHO_STEMNESS_DN http://www.broadinstitute.org/gsea/msigdb/cards/RAMALHO_STEMNESS_DN 73 0.225423 0.774736 0.764706 0.887374 0 up vs 1SASSON_RESPONSE_TO_FORSKOLIN_UP http://www.broadinstitute.org/gsea/msigdb/cards/SASSON_RESPONSE_TO_FORSKOLIN_UP 90 0.188131 0.746833 0.766667 0.887388 0 up vs 1LINDGREN_BLADDER_CANCER_CLUSTER_1_UP http://www.broadinstitute.org/gsea/msigdb/cards/LINDGREN_BLADDER_CANCER_CLUSTER_1_UP 113 0.218772 0.753587 0.802469 0.887413 0 up vs 1PELLICCIOTTA_HDAC_IN_ANTIGEN_PRESENTATION_UP http://www.broadinstitute.org/gsea/msigdb/cards/PELLICCIOTTA_HDAC_IN_ANTIGEN_PRESENTATION_UP 61 0.29897 0.943052 0.441558 0.887414 0 up vs 1SHETH_LIVER_CANCER_VS_TXNIP_LOSS_PAM1 http://www.broadinstitute.org/gsea/msigdb/cards/SHETH_LIVER_CANCER_VS_TXNIP_LOSS_PAM1 226 0.223706 0.936863 0.531915 0.887434 0 up vs 1TAKAO_RESPONSE_TO_UVB_RADIATION_DN http://www.broadinstitute.org/gsea/msigdb/cards/TAKAO_RESPONSE_TO_UVB_RADIATION_DN 98 0.214169 0.763372 0.758621 0.887472 0 up vs 1WEIGEL_OXIDATIVE_STRESS_RESPONSE http://www.broadinstitute.org/gsea/msigdb/cards/WEIGEL_OXIDATIVE_STRESS_RESPONSE 34 0.273861 0.937223 0.602564 0.887485 0 up vs 1OXFORD_RALA_OR_RALB_TARGETS_DN http://www.broadinstitute.org/gsea/msigdb/cards/OXFORD_RALA_OR_RALB_TARGETS_DN 23 0.323359 0.937613 0.507463 0.887495 0 up vs 1STREICHER_LSM1_TARGETS_UP http://www.broadinstitute.org/gsea/msigdb/cards/STREICHER_LSM1_TARGETS_UP 43 0.222126 0.754115 0.753425 0.887522 0 up vs 1KRASNOSELSKAYA_ILF3_TARGETS_DN http://www.broadinstitute.org/gsea/msigdb/cards/KRASNOSELSKAYA_ILF3_TARGETS_DN 44 0.22823 0.790079 0.794521 0.887558 0 up vs 1LIU_VMYB_TARGETS_UP http://www.broadinstitute.org/gsea/msigdb/cards/LIU_VMYB_TARGETS_UP 118 0.203507 0.763858 0.777778 0.887576 0 up vs 1WINTER_HYPOXIA_METAGENE http://www.broadinstitute.org/gsea/msigdb/cards/WINTER_HYPOXIA_METAGENE 236 0.188415 0.752188 0.727273 0.887599 0 up vs 1SENESE_HDAC3_TARGETS_UP http://www.broadinstitute.org/gsea/msigdb/cards/SENESE_HDAC3_TARGETS_UP 485 0.200672 0.722192 0.790123 0.887602 0 up vs 1WILCOX_RESPONSE_TO_PROGESTERONE_DN http://www.broadinstitute.org/gsea/msigdb/cards/WILCOX_RESPONSE_TO_PROGESTERONE_DN 65 0.20188 0.702762 0.815789 0.887631 0 up vs 1TOYOTA_TARGETS_OF_MIR34B_AND_MIR34C http://www.broadinstitute.org/gsea/msigdb/cards/TOYOTA_TARGETS_OF_MIR34B_AND_MIR34C 442 0.223809 0.768004 0.771084 0.887662 0 up vs 1DAVICIONI_RHABDOMYOSARCOMA_PAX_FOXO1_FUSION_DN http://www.broadinstitute.org/gsea/msigdb/cards/DAVICIONI_RHABDOMYOSARCOMA_PAX_FOXO1_FUSION_DN 15 0.254215 0.775096 0.836066 0.887692 0 up vs 1TARTE_PLASMA_CELL_VS_PLASMABLAST_DN http://www.broadinstitute.org/gsea/msigdb/cards/TARTE_PLASMA_CELL_VS_PLASMABLAST_DN 307 0.214179 0.722673 0.865854 0.887724 0 up vs 1BROWNE_HCMV_INFECTION_10HR_UP http://www.broadinstitute.org/gsea/msigdb/cards/BROWNE_HCMV_INFECTION_10HR_UP 99 0.202577 0.747114 0.756757 0.887731 0 up vs 1IWANAGA_CARCINOGENESIS_BY_KRAS_PTEN_UP http://www.broadinstitute.org/gsea/msigdb/cards/IWANAGA_CARCINOGENESIS_BY_KRAS_PTEN_UP 174 0.188121 0.752587 0.771084 0.887737 0 up vs 1KYNG_ENVIRONMENTAL_STRESS_RESPONSE_NOT_BY_UV_IN_OLD http://www.broadinstitute.org/gsea/msigdb/cards/KYNG_ENVIRONMENTAL_STRESS_RESPONSE_NOT_BY_UV_IN_OLD 24 0.234183 0.768448 0.8 0.887742 0 up vs 1EHLERS_ANEUPLOIDY_UP http://www.broadinstitute.org/gsea/msigdb/cards/EHLERS_ANEUPLOIDY_UP 41 0.241708 0.778849 0.833333 0.887796 0 up vs 1GINESTIER_BREAST_CANCER_ZNF217_AMPLIFIED_UP http://www.broadinstitute.org/gsea/msigdb/cards/GINESTIER_BREAST_CANCER_ZNF217_AMPLIFIED_UP 71 0.241087 0.704486 0.833333 0.887828 0 up vs 1ONDER_CDH1_SIGNALING_VIA_CTNNB1 http://www.broadinstitute.org/gsea/msigdb/cards/ONDER_CDH1_SIGNALING_VIA_CTNNB1 81 0.22012 0.723194 0.788732 0.88784 0 up vs 1FOURNIER_ACINAR_DEVELOPMENT_EARLY_UP http://www.broadinstitute.org/gsea/msigdb/cards/FOURNIER_ACINAR_DEVELOPMENT_EARLY_UP 20 0.237078 0.747578 0.861111 0.887851 0 up vs 1WANG_SMARCE1_TARGETS_UP http://www.broadinstitute.org/gsea/msigdb/cards/WANG_SMARCE1_TARGETS_UP 274 0.196259 0.70305 0.779221 0.887992 0 up vs 1SESTO_RESPONSE_TO_UV_C0 http://www.broadinstitute.org/gsea/msigdb/cards/SESTO_RESPONSE_TO_UV_C0 107 0.219551 0.764034 0.780488 0.888017 0 up vs 1PEDRIOLI_MIR31_TARGETS_DN http://www.broadinstitute.org/gsea/msigdb/cards/PEDRIOLI_MIR31_TARGETS_DN 396 0.195491 0.754144 0.75 0.888147 0 up vs 1PANGAS_TUMOR_SUPPRESSION_BY_SMAD1_AND_SMAD5_UP http://www.broadinstitute.org/gsea/msigdb/cards/PANGAS_TUMOR_SUPPRESSION_BY_SMAD1_AND_SMAD5_UP 127 0.214866 0.790157 0.726027 0.888181 0 up vs 1OHGUCHI_LIVER_HNF4A_TARGETS_DN http://www.broadinstitute.org/gsea/msigdb/cards/OHGUCHI_LIVER_HNF4A_TARGETS_DN 145 0.205493 0.768687 0.774648 0.888198 0 up vs 1DANG_MYC_TARGETS_DN http://www.broadinstitute.org/gsea/msigdb/cards/DANG_MYC_TARGETS_DN 31 0.242453 0.790591 0.770115 0.88837 0 up vs 1BRACHAT_RESPONSE_TO_METHOTREXATE_DN http://www.broadinstitute.org/gsea/msigdb/cards/BRACHAT_RESPONSE_TO_METHOTREXATE_DN 27 0.249077 0.794946 0.8125 0.888372 0 up vs 1IKEDA_MIR30_TARGETS_UP http://www.broadinstitute.org/gsea/msigdb/cards/IKEDA_MIR30_TARGETS_UP 115 0.217922 0.692853 0.791667 0.888455 0 up vs 1TURASHVILI_BREAST_DUCTAL_CARCINOMA_VS_DUCTAL_NORMAL_UP http://www.broadinstitute.org/gsea/msigdb/cards/TURASHVILI_BREAST_DUCTAL_CARCINOMA_VS_DUCTAL_NORMAL_UP 43 0.257539 0.796095 0.78125 0.888503 0 up vs 1HUANG_GATA2_TARGETS_DN http://www.broadinstitute.org/gsea/msigdb/cards/HUANG_GATA2_TARGETS_DN 70 0.204253 0.764242 0.809524 0.888506 0 up vs 1HARRIS_HYPOXIA http://www.broadinstitute.org/gsea/msigdb/cards/HARRIS_HYPOXIA 79 0.205919 0.769073 0.763158 0.888509 0 up vs 1RIZKI_TUMOR_INVASIVENESS_3D_DN http://www.broadinstitute.org/gsea/msigdb/cards/RIZKI_TUMOR_INVASIVENESS_3D_DN 266 0.22313 0.935044 0.534884 0.888522 0 up vs 1JI_METASTASIS_REPRESSED_BY_STK11 http://www.broadinstitute.org/gsea/msigdb/cards/JI_METASTASIS_REPRESSED_BY_STK11 26 0.223366 0.703148 0.822581 0.888544 0 up vs 1MASRI_RESISTANCE_TO_TAMOXIFEN_AND_AROMATASE_INHIBITORS_UP http://www.broadinstitute.org/gsea/msigdb/cards/MASRI_RESISTANCE_TO_TAMOXIFEN_AND_AROMATASE_INHIBITORS_UP 20 0.246505 0.794151 0.836066 0.888551 0 up vs 1CARD_MIR302A_TARGETS http://www.broadinstitute.org/gsea/msigdb/cards/CARD_MIR302A_TARGETS 77 0.246114 0.790942 0.808824 0.888578 0 up vs 1FARMER_BREAST_CANCER_APOCRINE_VS_BASAL http://www.broadinstitute.org/gsea/msigdb/cards/FARMER_BREAST_CANCER_APOCRINE_VS_BASAL 326 0.201231 0.792537 0.704545 0.888593 0 up vs 1DELACROIX_RARG_BOUND_MEF http://www.broadinstitute.org/gsea/msigdb/cards/DELACROIX_RARG_BOUND_MEF 360 0.189004 0.792996 0.651685 0.888608 0 up vs 1HONRADO_BREAST_CANCER_BRCA1_VS_BRCA2 http://www.broadinstitute.org/gsea/msigdb/cards/HONRADO_BREAST_CANCER_BRCA1_VS_BRCA2 18 0.261285 0.791489 0.84507 0.888631 0 up vs 1IIZUKA_LIVER_CANCER_PROGRESSION_L0_L1_UP http://www.broadinstitute.org/gsea/msigdb/cards/IIZUKA_LIVER_CANCER_PROGRESSION_L0_L1_UP 17 0.235597 0.7974 0.780488 0.888794 0 up vs 1VALK_AML_CLUSTER_11 http://www.broadinstitute.org/gsea/msigdb/cards/VALK_AML_CLUSTER_11 36 0.249426 0.791799 0.84 0.88892 0 up vs 1BERENJENO_TRANSFORMED_BY_RHOA_REVERSIBLY_DN http://www.broadinstitute.org/gsea/msigdb/cards/BERENJENO_TRANSFORMED_BY_RHOA_REVERSIBLY_DN 29 0.272187 0.796269 0.666667 0.888945 0 up vs 1BARRIER_CANCER_RELAPSE_NORMAL_SAMPLE_UP http://www.broadinstitute.org/gsea/msigdb/cards/BARRIER_CANCER_RELAPSE_NORMAL_SAMPLE_UP 32 0.249851 0.795091 0.828571 0.888951 0 up vs 1BAKKER_FOXO3_TARGETS_UP http://www.broadinstitute.org/gsea/msigdb/cards/BAKKER_FOXO3_TARGETS_UP 60 0.215305 0.76923 0.782051 0.888996 0 up vs 1LIU_NASOPHARYNGEAL_CARCINOMA http://www.broadinstitute.org/gsea/msigdb/cards/LIU_NASOPHARYNGEAL_CARCINOMA 67 0.208636 0.793091 0.765432 0.889192 0 up vs 1LI_DCP2_BOUND_MRNA http://www.broadinstitute.org/gsea/msigdb/cards/LI_DCP2_BOUND_MRNA 89 0.294868 0.935167 0.565789 0.889194 0 up vs 1WANG_THOC1_TARGETS_DN http://www.broadinstitute.org/gsea/msigdb/cards/WANG_THOC1_TARGETS_DN 19 0.246628 0.797664 0.813333 0.889211 0 up vs 1PECE_MAMMARY_STEM_CELL_UP http://www.broadinstitute.org/gsea/msigdb/cards/PECE_MAMMARY_STEM_CELL_UP 141 0.29343 0.933423 0.545455 0.889419 0 up vs 1YU_BAP1_TARGETS http://www.broadinstitute.org/gsea/msigdb/cards/YU_BAP1_TARGETS 29 0.265866 0.769343 0.88 0.88955 0 up vs 1RASHI_RESPONSE_TO_IONIZING_RADIATION_6 http://www.broadinstitute.org/gsea/msigdb/cards/RASHI_RESPONSE_TO_IONIZING_RADIATION_6 81 0.228747 0.796304 0.764706 0.889556 0 up vs 1ZEMBUTSU_SENSITIVITY_TO_VINCRISTINE http://www.broadinstitute.org/gsea/msigdb/cards/ZEMBUTSU_SENSITIVITY_TO_VINCRISTINE 18 0.259601 0.79785 0.753623 0.889617 0 up vs 1ROZANOV_MMP14_TARGETS_DN http://www.broadinstitute.org/gsea/msigdb/cards/ROZANOV_MMP14_TARGETS_DN 34 0.187462 0.690878 0.855422 0.889745 0 up vs 1FLECHNER_BIOPSY_KIDNEY_TRANSPLANT_OK_VS_DONOR_DN http://www.broadinstitute.org/gsea/msigdb/cards/FLECHNER_BIOPSY_KIDNEY_TRANSPLANT_OK_VS_DONOR_DN 25 0.230366 0.769563 0.767442 0.889942 0 up vs 1ABRAMSON_INTERACT_WITH_AIRE http://www.broadinstitute.org/gsea/msigdb/cards/ABRAMSON_INTERACT_WITH_AIRE 45 0.256468 0.689973 0.855263 0.889957 0 up vs 1KATSANOU_ELAVL1_TARGETS_UP http://www.broadinstitute.org/gsea/msigdb/cards/KATSANOU_ELAVL1_TARGETS_UP 166 0.2098 0.79802 0.72973 0.890131 0 up vs 1GRADE_COLON_VS_RECTAL_CANCER_UP http://www.broadinstitute.org/gsea/msigdb/cards/GRADE_COLON_VS_RECTAL_CANCER_UP 34 0.298314 0.933469 0.556962 0.890227 0 up vs 1PASINI_SUZ12_TARGETS_UP http://www.broadinstitute.org/gsea/msigdb/cards/PASINI_SUZ12_TARGETS_UP 109 0.187815 0.769769 0.747126 0.890401 0 up vs 1SCHRAETS_MLL_TARGETS_UP http://www.broadinstitute.org/gsea/msigdb/cards/SCHRAETS_MLL_TARGETS_UP 35 0.207167 0.688877 0.928571 0.890414 0 up vs 1WANG_IMMORTALIZED_BY_HOXA9_AND_MEIS1_DN http://www.broadinstitute.org/gsea/msigdb/cards/WANG_IMMORTALIZED_BY_HOXA9_AND_MEIS1_DN 24 -0.24666 -0.699479 0.828571 0.89056 0 down vs 1CAVARD_LIVER_CANCER_MALIGNANT_VS_BENIGN http://www.broadinstitute.org/gsea/msigdb/cards/CAVARD_LIVER_CANCER_MALIGNANT_VS_BENIGN 31 -0.20896 -0.698535 0.866667 0.890704 0 down vs 1BILANGES_RAPAMYCIN_SENSITIVE_GENES http://www.broadinstitute.org/gsea/msigdb/cards/BILANGES_RAPAMYCIN_SENSITIVE_GENES 39 0.271428 0.933596 0.641975 0.890715 0 up vs 1WEST_ADRENOCORTICAL_TUMOR_UP http://www.broadinstitute.org/gsea/msigdb/cards/WEST_ADRENOCORTICAL_TUMOR_UP 286 0.230085 0.798063 0.738095 0.890764 0 up vs 1JIANG_HYPOXIA_CANCER http://www.broadinstitute.org/gsea/msigdb/cards/JIANG_HYPOXIA_CANCER 80 0.187678 0.687738 0.895349 0.890936 0 up vs 1HOLLMANN_APOPTOSIS_VIA_CD40_UP http://www.broadinstitute.org/gsea/msigdb/cards/HOLLMANN_APOPTOSIS_VIA_CD40_UP 192 0.201213 0.769804 0.756098 0.89103 0 up vs 1INGRAM_SHH_TARGETS_DN http://www.broadinstitute.org/gsea/msigdb/cards/INGRAM_SHH_TARGETS_DN 58 0.218981 0.79958 0.764706 0.891062 0 up vs 1SCHAEFFER_PROSTATE_DEVELOPMENT_48HR_UP http://www.broadinstitute.org/gsea/msigdb/cards/SCHAEFFER_PROSTATE_DEVELOPMENT_48HR_UP 472 0.189539 0.799999 0.673913 0.891231 0 up vs 1IWANAGA_CARCINOGENESIS_BY_KRAS_PTEN_DN http://www.broadinstitute.org/gsea/msigdb/cards/IWANAGA_CARCINOGENESIS_BY_KRAS_PTEN_DN 340 0.202614 0.79818 0.695122 0.891273 0 up vs 1SAKAI_TUMOR_INFILTRATING_MONOCYTES_UP http://www.broadinstitute.org/gsea/msigdb/cards/SAKAI_TUMOR_INFILTRATING_MONOCYTES_UP 27 0.248736 0.805101 0.808219 0.891301 0 up vs 1HOLLEMAN_VINCRISTINE_RESISTANCE_B_ALL_DN http://www.broadinstitute.org/gsea/msigdb/cards/HOLLEMAN_VINCRISTINE_RESISTANCE_B_ALL_DN 15 0.30014 0.800391 0.742857 0.891508 0 up vs 1HOFFMANN_LARGE_TO_SMALL_PRE_BII_LYMPHOCYTE_DN http://www.broadinstitute.org/gsea/msigdb/cards/HOFFMANN_LARGE_TO_SMALL_PRE_BII_LYMPHOCYTE_DN 70 0.21105 0.805464 0.723684 0.891535 0 up vs 1BROWNE_HCMV_INFECTION_14HR_DN http://www.broadinstitute.org/gsea/msigdb/cards/BROWNE_HCMV_INFECTION_14HR_DN 290 0.189741 0.769875 0.738636 0.891661 0 up vs 1RAY_TARGETS_OF_P210_BCR_ABL_FUSION_DN http://www.broadinstitute.org/gsea/msigdb/cards/RAY_TARGETS_OF_P210_BCR_ABL_FUSION_DN 16 0.290075 0.800778 0.714286 0.891684 0 up vs 1MORI_EMU_MYC_LYMPHOMA_BY_ONSET_TIME_UP http://www.broadinstitute.org/gsea/msigdb/cards/MORI_EMU_MYC_LYMPHOMA_BY_ONSET_TIME_UP 103 0.289839 0.930876 0.536585 0.891896 0 up vs 1KYNG_DNA_DAMAGE_UP http://www.broadinstitute.org/gsea/msigdb/cards/KYNG_DNA_DAMAGE_UP 223 0.239287 0.93132 0.621951 0.891951 0 up vs 1SIMBULAN_PARP1_TARGETS_UP http://www.broadinstitute.org/gsea/msigdb/cards/SIMBULAN_PARP1_TARGETS_UP 31 0.255533 0.804117 0.746479 0.891951 0 up vs 1JAERVINEN_AMPLIFIED_IN_LARYNGEAL_CANCER http://www.broadinstitute.org/gsea/msigdb/cards/JAERVINEN_AMPLIFIED_IN_LARYNGEAL_CANCER 37 0.223575 0.798203 0.766234 0.891953 0 up vs 1JI_RESPONSE_TO_FSH_DN http://www.broadinstitute.org/gsea/msigdb/cards/JI_RESPONSE_TO_FSH_DN 57 0.22841 0.685863 0.819444 0.892065 0 up vs 1ROSS_LEUKEMIA_WITH_MLL_FUSIONS http://www.broadinstitute.org/gsea/msigdb/cards/ROSS_LEUKEMIA_WITH_MLL_FUSIONS 77 0.22335 0.805479 0.783784 0.892216 0 up vs 1SCHURINGA_STAT5A_TARGETS_UP http://www.broadinstitute.org/gsea/msigdb/cards/SCHURINGA_STAT5A_TARGETS_UP 21 0.27279 0.931545 0.675 0.892326 0 up vs 1VANLOO_SP3_TARGETS_DN http://www.broadinstitute.org/gsea/msigdb/cards/VANLOO_SP3_TARGETS_DN 85 0.21899 0.802549 0.721519 0.892359 0 up vs 1NUNODA_RESPONSE_TO_DASATINIB_IMATINIB_UP http://www.broadinstitute.org/gsea/msigdb/cards/NUNODA_RESPONSE_TO_DASATINIB_IMATINIB_UP 29 0.268256 0.800792 0.716216 0.892379 0 up vs 1LINDVALL_IMMORTALIZED_BY_TERT_DN http://www.broadinstitute.org/gsea/msigdb/cards/LINDVALL_IMMORTALIZED_BY_TERT_DN 79 0.235158 0.803077 0.767123 0.892498 0 up vs 1SASAI_RESISTANCE_TO_NEOPLASTIC_TRANSFROMATION http://www.broadinstitute.org/gsea/msigdb/cards/SASAI_RESISTANCE_TO_NEOPLASTIC_TRANSFROMATION 50 -0.211618 -0.69534 0.827586 0.892558 0 down vs 1SABATES_COLORECTAL_ADENOMA_SIZE_UP http://www.broadinstitute.org/gsea/msigdb/cards/SABATES_COLORECTAL_ADENOMA_SIZE_UP 22 0.215066 0.684728 0.847222 0.892569 0 up vs 1BERENJENO_ROCK_SIGNALING_NOT_VIA_RHOA_DN http://www.broadinstitute.org/gsea/msigdb/cards/BERENJENO_ROCK_SIGNALING_NOT_VIA_RHOA_DN 45 -0.200831 -0.696163 0.846154 0.89264 0 down vs 1CHIARADONNA_NEOPLASTIC_TRANSFORMATION_CDC25_UP http://www.broadinstitute.org/gsea/msigdb/cards/CHIARADONNA_NEOPLASTIC_TRANSFORMATION_CDC25_UP 119 0.22251 0.801005 0.74359 0.892867 0 up vs 1FOSTER_KDM1A_TARGETS_UP http://www.broadinstitute.org/gsea/msigdb/cards/FOSTER_KDM1A_TARGETS_UP 257 0.192376 0.80548 0.658824 0.89291 0 up vs 1CHEN_LVAD_SUPPORT_OF_FAILING_HEART_DN http://www.broadinstitute.org/gsea/msigdb/cards/CHEN_LVAD_SUPPORT_OF_FAILING_HEART_DN 42 0.223371 0.801449 0.742857 0.893076 0 up vs 1SHEN_SMARCA2_TARGETS_UP http://www.broadinstitute.org/gsea/msigdb/cards/SHEN_SMARCA2_TARGETS_UP 415 0.266601 0.805771 0.708861 0.893331 0 up vs 1VALK_AML_CLUSTER_3 http://www.broadinstitute.org/gsea/msigdb/cards/VALK_AML_CLUSTER_3 32 0.233145 0.80942 0.76 0.893628 0 up vs 1IIZUKA_LIVER_CANCER_PROGRESSION_G1_G2_DN http://www.broadinstitute.org/gsea/msigdb/cards/IIZUKA_LIVER_CANCER_PROGRESSION_G1_G2_DN 24 0.28382 0.805955 0.71831 0.893804 0 up vs 1JIANG_HYPOXIA_VIA_VHL http://www.broadinstitute.org/gsea/msigdb/cards/JIANG_HYPOXIA_VIA_VHL 34 0.269727 0.806372 0.804878 0.893971 0 up vs 1GROSS_HYPOXIA_VIA_ELK3_AND_HIF1A_DN http://www.broadinstitute.org/gsea/msigdb/cards/GROSS_HYPOXIA_VIA_ELK3_AND_HIF1A_DN 102 0.191125 0.682417 0.869048 0.894039 0 up vs 1SESTO_RESPONSE_TO_UV_C6 http://www.broadinstitute.org/gsea/msigdb/cards/SESTO_RESPONSE_TO_UV_C6 37 0.244063 0.809606 0.740741 0.89411 0 up vs 1YAMASHITA_LIVER_CANCER_WITH_EPCAM_DN http://www.broadinstitute.org/gsea/msigdb/cards/YAMASHITA_LIVER_CANCER_WITH_EPCAM_DN 16 0.22981 0.68146 0.923077 0.894242 0 up vs 1IGLESIAS_E2F_TARGETS_UP http://www.broadinstitute.org/gsea/msigdb/cards/IGLESIAS_E2F_TARGETS_UP 149 0.190128 0.678027 0.835443 0.894332 0 up vs 1CORRE_MULTIPLE_MYELOMA_DN http://www.broadinstitute.org/gsea/msigdb/cards/CORRE_MULTIPLE_MYELOMA_DN 60 0.187584 0.679835 0.914286 0.894467 0 up vs 1CHEN_LIVER_METABOLISM_QTL_CIS http://www.broadinstitute.org/gsea/msigdb/cards/CHEN_LIVER_METABOLISM_QTL_CIS 92 0.187735 0.678363 0.853333 0.894613 0 up vs 1MIKKELSEN_MCV6_ICP_WITH_H3K27ME3 http://www.broadinstitute.org/gsea/msigdb/cards/MIKKELSEN_MCV6_ICP_WITH_H3K27ME3 73 0.210806 0.806944 0.75 0.894638 0 up vs 1SCHRAETS_MLL_TARGETS_DN http://www.broadinstitute.org/gsea/msigdb/cards/SCHRAETS_MLL_TARGETS_DN 31 0.249708 0.806377 0.735294 0.894669 0 up vs 1WENDT_COHESIN_TARGETS_UP http://www.broadinstitute.org/gsea/msigdb/cards/WENDT_COHESIN_TARGETS_UP 32 0.285751 0.809646 0.643836 0.894773 0 up vs 1TURASHVILI_BREAST_NORMAL_DUCTAL_VS_LOBULAR_UP http://www.broadinstitute.org/gsea/msigdb/cards/TURASHVILI_BREAST_NORMAL_DUCTAL_VS_LOBULAR_UP 64 0.198197 0.678793 0.815789 0.894884 0 up vs 1TOMIDA_METASTASIS_DN http://www.broadinstitute.org/gsea/msigdb/cards/TOMIDA_METASTASIS_DN 17 0.239693 0.680076 0.873418 0.894898 0 up vs 1RAMPON_ENRICHED_LEARNING_ENVIRONMENT_EARLY_UP http://www.broadinstitute.org/gsea/msigdb/cards/RAMPON_ENRICHED_LEARNING_ENVIRONMENT_EARLY_UP 15 0.225323 0.665177 0.881579 0.895148 0 up vs 1SU_TESTIS http://www.broadinstitute.org/gsea/msigdb/cards/SU_TESTIS 74 0.240396 0.807104 0.786667 0.895171 0 up vs 1BOYLAN_MULTIPLE_MYELOMA_D_UP http://www.broadinstitute.org/gsea/msigdb/cards/BOYLAN_MULTIPLE_MYELOMA_D_UP 85 0.217717 0.809763 0.705882 0.89534 0 up vs 1SERVITJA_ISLET_HNF1A_TARGETS_UP http://www.broadinstitute.org/gsea/msigdb/cards/SERVITJA_ISLET_HNF1A_TARGETS_UP 162 0.215565 0.807573 0.684211 0.895346 0 up vs 1VART_KSHV_INFECTION_ANGIOGENIC_MARKERS_UP http://www.broadinstitute.org/gsea/msigdb/cards/VART_KSHV_INFECTION_ANGIOGENIC_MARKERS_UP 164 0.188074 0.666916 0.833333 0.895441 0 up vs 1MARIADASON_RESPONSE_TO_BUTYRATE_CURCUMIN_SULINDAC_TSA_8 http://www.broadinstitute.org/gsea/msigdb/cards/MARIADASON_RESPONSE_TO_BUTYRATE_CURCUMIN_SULINDAC_TSA_8 16 0.249285 0.667456 0.895522 0.895492 0 up vs 1LIM_MAMMARY_STEM_CELL_DN http://www.broadinstitute.org/gsea/msigdb/cards/LIM_MAMMARY_STEM_CELL_DN 418 0.189281 0.665409 0.822785 0.895519 0 up vs 1CHUANG_OXIDATIVE_STRESS_RESPONSE_UP http://www.broadinstitute.org/gsea/msigdb/cards/CHUANG_OXIDATIVE_STRESS_RESPONSE_UP 28 0.18871 0.664138 0.884058 0.895603 0 up vs 1BENPORATH_OCT4_TARGETS http://www.broadinstitute.org/gsea/msigdb/cards/BENPORATH_OCT4_TARGETS 285 0.241689 0.928112 0.597701 0.895774 0 up vs 1VERHAAK_AML_WITH_NPM1_MUTATED_DN http://www.broadinstitute.org/gsea/msigdb/cards/VERHAAK_AML_WITH_NPM1_MUTATED_DN 242 0.210983 0.809999 0.714286 0.895793 0 up vs 1BIDUS_METASTASIS_DN http://www.broadinstitute.org/gsea/msigdb/cards/BIDUS_METASTASIS_DN 151 0.188013 0.665608 0.864865 0.895918 0 up vs 1KANG_GIST_WITH_PDGFRA_UP http://www.broadinstitute.org/gsea/msigdb/cards/KANG_GIST_WITH_PDGFRA_UP 49 0.204176 0.672284 0.880597 0.895987 0 up vs 1BURTON_ADIPOGENESIS_8 http://www.broadinstitute.org/gsea/msigdb/cards/BURTON_ADIPOGENESIS_8 83 0.198893 0.667559 0.833333 0.896006 0 up vs 1ZHANG_TLX_TARGETS_36HR_UP http://www.broadinstitute.org/gsea/msigdb/cards/ZHANG_TLX_TARGETS_36HR_UP 217 0.235523 0.926644 0.62963 0.896108 0 up vs 1BOQUEST_STEM_CELL_CULTURED_VS_FRESH_DN http://www.broadinstitute.org/gsea/msigdb/cards/BOQUEST_STEM_CELL_CULTURED_VS_FRESH_DN 30 0.188944 0.6682 0.857143 0.896111 0 up vs 1KIM_WT1_TARGETS_12HR_UP http://www.broadinstitute.org/gsea/msigdb/cards/KIM_WT1_TARGETS_12HR_UP 158 0.242953 0.92836 0.632911 0.896128 0 up vs 1SEKI_INFLAMMATORY_RESPONSE_LPS_DN http://www.broadinstitute.org/gsea/msigdb/cards/SEKI_INFLAMMATORY_RESPONSE_LPS_DN 21 -0.211342 -0.68793 0.931035 0.896254 0 down vs 1WAKASUGI_HAVE_ZNF143_BINDING_SITES http://www.broadinstitute.org/gsea/msigdb/cards/WAKASUGI_HAVE_ZNF143_BINDING_SITES 58 0.222495 0.675179 0.855263 0.896299 0 up vs 1ACEVEDO_LIVER_CANCER_WITH_H3K9ME3_DN http://www.broadinstitute.org/gsea/msigdb/cards/ACEVEDO_LIVER_CANCER_WITH_H3K9ME3_DN 117 0.187853 0.672611 0.896104 0.896329 0 up vs 1WANG_BARRETTS_ESOPHAGUS_AND_ESOPHAGUS_CANCER_UP http://www.broadinstitute.org/gsea/msigdb/cards/WANG_BARRETTS_ESOPHAGUS_AND_ESOPHAGUS_CANCER_UP 26 0.242065 0.673196 0.888889 0.896364 0 up vs 1DURAND_STROMA_S_UP http://www.broadinstitute.org/gsea/msigdb/cards/DURAND_STROMA_S_UP 287 0.209546 0.810079 0.701299 0.896388 0 up vs 1GENTILE_UV_RESPONSE_CLUSTER_D6 http://www.broadinstitute.org/gsea/msigdb/cards/GENTILE_UV_RESPONSE_CLUSTER_D6 35 0.231452 0.662611 0.887324 0.896453 0 up vs 1DAIRKEE_CANCER_PRONE_RESPONSE_E2 http://www.broadinstitute.org/gsea/msigdb/cards/DAIRKEE_CANCER_PRONE_RESPONSE_E2 28 0.187434 0.673719 0.866667 0.896465 0 up vs 1FERRANDO_T_ALL_WITH_MLL_ENL_FUSION_UP http://www.broadinstitute.org/gsea/msigdb/cards/FERRANDO_T_ALL_WITH_MLL_ENL_FUSION_UP 85 0.245769 0.927265 0.653846 0.896497 0 up vs 1THEILGAARD_NEUTROPHIL_AT_SKIN_WOUND_DN http://www.broadinstitute.org/gsea/msigdb/cards/THEILGAARD_NEUTROPHIL_AT_SKIN_WOUND_DN 224 0.188358 0.670434 0.8125 0.896535 0 up vs 1FARMER_BREAST_CANCER_CLUSTER_2 http://www.broadinstitute.org/gsea/msigdb/cards/FARMER_BREAST_CANCER_CLUSTER_2 33 -0.286703 -0.688503 0.774194 0.896578 0 down vs 1ENGELMANN_CANCER_PROGENITORS_UP http://www.broadinstitute.org/gsea/msigdb/cards/ENGELMANN_CANCER_PROGENITORS_UP 47 0.187523 0.66964 0.922078 0.896653 0 up vs 1CHEMELLO_SOLEUS_VS_EDL_MYOFIBERS_UP http://www.broadinstitute.org/gsea/msigdb/cards/CHEMELLO_SOLEUS_VS_EDL_MYOFIBERS_UP 35 0.188743 0.668226 0.945946 0.896681 0 up vs 1BOYLAN_MULTIPLE_MYELOMA_D_CLUSTER_DN http://www.broadinstitute.org/gsea/msigdb/cards/BOYLAN_MULTIPLE_MYELOMA_D_CLUSTER_DN 40 0.204241 0.670961 0.868421 0.896691 0 up vs 1LIU_LIVER_CANCER http://www.broadinstitute.org/gsea/msigdb/cards/LIU_LIVER_CANCER 36 0.247625 0.923091 0.653846 0.896725 0 up vs 1PEPPER_CHRONIC_LYMPHOCYTIC_LEUKEMIA_UP http://www.broadinstitute.org/gsea/msigdb/cards/PEPPER_CHRONIC_LYMPHOCYTIC_LEUKEMIA_UP 33 0.187458 0.668864 0.9 0.896755 0 up vs 1VALK_AML_WITH_CEBPA http://www.broadinstitute.org/gsea/msigdb/cards/VALK_AML_WITH_CEBPA 37 0.191311 0.674069 0.918919 0.896786 0 up vs 1MCCABE_HOXC6_TARGETS_DN http://www.broadinstitute.org/gsea/msigdb/cards/MCCABE_HOXC6_TARGETS_DN 21 0.272684 0.810198 0.779412 0.896913 0 up vs 1LINDGREN_BLADDER_CANCER_CLUSTER_2B http://www.broadinstitute.org/gsea/msigdb/cards/LINDGREN_BLADDER_CANCER_CLUSTER_2B 383 -0.192511 -0.689096 0.709677 0.896919 0 down vs 1BROWNE_HCMV_INFECTION_8HR_UP http://www.broadinstitute.org/gsea/msigdb/cards/BROWNE_HCMV_INFECTION_8HR_UP 105 0.248019 0.926675 0.631579 0.896965 0 up vs 1AIYAR_COBRA1_TARGETS_UP http://www.broadinstitute.org/gsea/msigdb/cards/AIYAR_COBRA1_TARGETS_UP 38 0.187481 0.661307 0.906977 0.897113 0 up vs 1LEE_DOUBLE_POLAR_THYMOCYTE http://www.broadinstitute.org/gsea/msigdb/cards/LEE_DOUBLE_POLAR_THYMOCYTE 26 -0.220786 -0.689714 0.961538 0.897117 0 down vs 1NAKAMURA_ADIPOGENESIS_LATE_UP http://www.broadinstitute.org/gsea/msigdb/cards/NAKAMURA_ADIPOGENESIS_LATE_UP 100 0.249408 0.923284 0.670732 0.897211 0 up vs 1LIM_MAMMARY_LUMINAL_MATURE_UP http://www.broadinstitute.org/gsea/msigdb/cards/LIM_MAMMARY_LUMINAL_MATURE_UP 111 0.260017 0.925696 0.636364 0.89722 0 up vs 1WANG_ESOPHAGUS_CANCER_VS_NORMAL_UP http://www.broadinstitute.org/gsea/msigdb/cards/WANG_ESOPHAGUS_CANCER_VS_NORMAL_UP 117 0.187853 0.66027 0.893333 0.897408 0 up vs 1BANDRES_RESPONSE_TO_CARMUSTIN_WITHOUT_MGMT_48HR_DN http://www.broadinstitute.org/gsea/msigdb/cards/BANDRES_RESPONSE_TO_CARMUSTIN_WITHOUT_MGMT_48HR_DN 30 0.2461 0.810248 0.772152 0.897542 0 up vs 1MANALO_HYPOXIA_UP http://www.broadinstitute.org/gsea/msigdb/cards/MANALO_HYPOXIA_UP 202 0.246322 0.92175 0.641026 0.897712 0 up vs 1MYLLYKANGAS_AMPLIFICATION_HOT_SPOT_17 http://www.broadinstitute.org/gsea/msigdb/cards/MYLLYKANGAS_AMPLIFICATION_HOT_SPOT_17 19 -0.229894 -0.689921 0.861111 0.897875 0 down vs 1MARKEY_RB1_CHRONIC_LOF_DN http://www.broadinstitute.org/gsea/msigdb/cards/MARKEY_RB1_CHRONIC_LOF_DN 114 0.187838 0.659118 0.857143 0.897898 0 up vs 1LANDIS_ERBB2_BREAST_TUMORS_324_UP http://www.broadinstitute.org/gsea/msigdb/cards/LANDIS_ERBB2_BREAST_TUMORS_324_UP 147 0.248149 0.923337 0.639535 0.897952 0 up vs 1ZHU_CMV_ALL_DN http://www.broadinstitute.org/gsea/msigdb/cards/ZHU_CMV_ALL_DN 125 0.231598 0.810448 0.74026 0.897978 0 up vs 1TAKADA_GASTRIC_CANCER_COPY_NUMBER_DN http://www.broadinstitute.org/gsea/msigdb/cards/TAKADA_GASTRIC_CANCER_COPY_NUMBER_DN 29 0.26698 0.921973 0.602941 0.898191 0 up vs 1DAIRKEE_TERT_TARGETS_UP http://www.broadinstitute.org/gsea/msigdb/cards/DAIRKEE_TERT_TARGETS_UP 359 0.241018 0.924356 0.613636 0.898356 0 up vs 1GARGALOVIC_RESPONSE_TO_OXIDIZED_PHOSPHOLIPIDS_GREEN_UP http://www.broadinstitute.org/gsea/msigdb/cards/GARGALOVIC_RESPONSE_TO_OXIDIZED_PHOSPHOLIPIDS_GREEN_UP 23 0.309371 0.923914 0.493506 0.898477 0 up vs 1JOHNSTONE_PARVB_TARGETS_1_DN http://www.broadinstitute.org/gsea/msigdb/cards/JOHNSTONE_PARVB_TARGETS_1_DN 59 0.249598 0.810617 0.753247 0.898531 0 up vs 1DACOSTA_UV_RESPONSE_VIA_ERCC3_COMMON_DN http://www.broadinstitute.org/gsea/msigdb/cards/DACOSTA_UV_RESPONSE_VIA_ERCC3_COMMON_DN 470 0.212103 0.657434 0.828947 0.898772 0 up vs 1WANG_ADIPOGENIC_GENES_REPRESSED_BY_SIRT1 http://www.broadinstitute.org/gsea/msigdb/cards/WANG_ADIPOGENIC_GENES_REPRESSED_BY_SIRT1 27 0.303032 0.923346 0.565217 0.898805 0 up vs 1BROWNE_HCMV_INFECTION_12HR_DN http://www.broadinstitute.org/gsea/msigdb/cards/BROWNE_HCMV_INFECTION_12HR_DN 97 0.263042 0.924571 0.631579 0.898854 0 up vs 1SENESE_HDAC1_TARGETS_UP http://www.broadinstitute.org/gsea/msigdb/cards/SENESE_HDAC1_TARGETS_UP 441 0.189391 0.656565 0.835443 0.898984 0 up vs 1GAJATE_RESPONSE_TO_TRABECTEDIN_DN http://www.broadinstitute.org/gsea/msigdb/cards/GAJATE_RESPONSE_TO_TRABECTEDIN_DN 19 0.299632 0.920641 0.536585 0.899032 0 up vs 1CHANG_CORE_SERUM_RESPONSE_DN http://www.broadinstitute.org/gsea/msigdb/cards/CHANG_CORE_SERUM_RESPONSE_DN 199 0.224059 0.821457 0.714286 0.899112 0 up vs 1SAMOLS_TARGETS_OF_KHSV_MIRNAS_DN http://www.broadinstitute.org/gsea/msigdb/cards/SAMOLS_TARGETS_OF_KHSV_MIRNAS_DN 60 0.221133 0.82031 0.7 0.899168 0 up vs 1SMITH_TERT_TARGETS_UP http://www.broadinstitute.org/gsea/msigdb/cards/SMITH_TERT_TARGETS_UP 143 0.228434 0.820843 0.704545 0.899206 0 up vs 1NIKOLSKY_OVERCONNECTED_IN_BREAST_CANCER http://www.broadinstitute.org/gsea/msigdb/cards/NIKOLSKY_OVERCONNECTED_IN_BREAST_CANCER 22 0.24691 0.810631 0.758065 0.899234 0 up vs 1DACOSTA_ERCC3_ALLELE_XPCS_VS_TTD_DN http://www.broadinstitute.org/gsea/msigdb/cards/DACOSTA_ERCC3_ALLELE_XPCS_VS_TTD_DN 36 0.251677 0.818434 0.774194 0.899323 0 up vs 1PARENT_MTOR_SIGNALING_DN http://www.broadinstitute.org/gsea/msigdb/cards/PARENT_MTOR_SIGNALING_DN 43 0.220223 0.811014 0.774648 0.899518 0 up vs 1SANA_TNF_SIGNALING_DN http://www.broadinstitute.org/gsea/msigdb/cards/SANA_TNF_SIGNALING_DN 90 0.233359 0.819508 0.693333 0.899524 0 up vs 1ANASTASSIOU_CANCER_MESENCHYMAL_TRANSITION_SIGNATURE http://www.broadinstitute.org/gsea/msigdb/cards/ANASTASSIOU_CANCER_MESENCHYMAL_TRANSITION_SIGNATURE 64 0.332481 0.825521 0.5625 0.899575 0 up vs 1MANTOVANI_NFKB_TARGETS_UP http://www.broadinstitute.org/gsea/msigdb/cards/MANTOVANI_NFKB_TARGETS_UP 43 0.231836 0.811472 0.797297 0.899691 0 up vs 1WEIGEL_OXIDATIVE_STRESS_BY_HNE_AND_TBH http://www.broadinstitute.org/gsea/msigdb/cards/WEIGEL_OXIDATIVE_STRESS_BY_HNE_AND_TBH 58 0.223822 0.818762 0.744186 0.899721 0 up vs 1HONMA_DOCETAXEL_RESISTANCE http://www.broadinstitute.org/gsea/msigdb/cards/HONMA_DOCETAXEL_RESISTANCE 34 0.2773 0.821511 0.662338 0.899735 0 up vs 1HU_GENOTOXIN_ACTION_DIRECT_VS_INDIRECT_4HR http://www.broadinstitute.org/gsea/msigdb/cards/HU_GENOTOXIN_ACTION_DIRECT_VS_INDIRECT_4HR 35 0.239948 0.816875 0.794521 0.899771 0 up vs 1BANDRES_RESPONSE_TO_CARMUSTIN_MGMT_24HR_DN http://www.broadinstitute.org/gsea/msigdb/cards/BANDRES_RESPONSE_TO_CARMUSTIN_MGMT_24HR_DN 33 0.263173 0.919205 0.6625 0.90002 0 up vs 1SASSON_RESPONSE_TO_GONADOTROPHINS_UP http://www.broadinstitute.org/gsea/msigdb/cards/SASSON_RESPONSE_TO_GONADOTROPHINS_UP 90 0.212941 0.816177 0.705882 0.900065 0 up vs 1BARRIER_COLON_CANCER_RECURRENCE_UP http://www.broadinstitute.org/gsea/msigdb/cards/BARRIER_COLON_CANCER_RECURRENCE_UP 42 0.241146 0.811705 0.717949 0.900066 0 up vs 1OSWALD_HEMATOPOIETIC_STEM_CELL_IN_COLLAGEN_GEL_DN http://www.broadinstitute.org/gsea/msigdb/cards/OSWALD_HEMATOPOIETIC_STEM_CELL_IN_COLLAGEN_GEL_DN 266 0.210465 0.828097 0.642857 0.900079 0 up vs 1MARIADASON_REGULATED_BY_HISTONE_ACETYLATION_UP http://www.broadinstitute.org/gsea/msigdb/cards/MARIADASON_REGULATED_BY_HISTONE_ACETYLATION_UP 79 0.228668 0.825678 0.69863 0.900116 0 up vs 1JI_RESPONSE_TO_FSH_UP http://www.broadinstitute.org/gsea/msigdb/cards/JI_RESPONSE_TO_FSH_UP 71 0.222826 0.826131 0.716049 0.900181 0 up vs 1GAUSSMANN_MLL_AF4_FUSION_TARGETS_E_DN http://www.broadinstitute.org/gsea/msigdb/cards/GAUSSMANN_MLL_AF4_FUSION_TARGETS_E_DN 22 0.279697 0.917771 0.640625 0.900251 0 up vs 1VERRECCHIA_EARLY_RESPONSE_TO_TGFB1 http://www.broadinstitute.org/gsea/msigdb/cards/VERRECCHIA_EARLY_RESPONSE_TO_TGFB1 58 0.245804 0.817097 0.74359 0.900253 0 up vs 1RIZ_ERYTHROID_DIFFERENTIATION_6HR http://www.broadinstitute.org/gsea/msigdb/cards/RIZ_ERYTHROID_DIFFERENTIATION_6HR 40 0.231344 0.821617 0.714286 0.900293 0 up vs 1GENTLES_LEUKEMIC_STEM_CELL_UP http://www.broadinstitute.org/gsea/msigdb/cards/GENTLES_LEUKEMIC_STEM_CELL_UP 27 0.287029 0.812009 0.68254 0.900351 0 up vs 1RAGHAVACHARI_PLATELET_SPECIFIC_GENES http://www.broadinstitute.org/gsea/msigdb/cards/RAGHAVACHARI_PLATELET_SPECIFIC_GENES 69 0.255133 0.915384 0.62963 0.90063 0 up vs 1ENK_UV_RESPONSE_KERATINOCYTE_DN http://www.broadinstitute.org/gsea/msigdb/cards/ENK_UV_RESPONSE_KERATINOCYTE_DN 481 0.238328 0.826324 0.755814 0.900664 0 up vs 1AMIT_SERUM_RESPONSE_20_MCF10A http://www.broadinstitute.org/gsea/msigdb/cards/AMIT_SERUM_RESPONSE_20_MCF10A 21 0.237558 0.8218 0.769231 0.900773 0 up vs 1HU_ANGIOGENESIS_DN http://www.broadinstitute.org/gsea/msigdb/cards/HU_ANGIOGENESIS_DN 37 0.270242 0.828106 0.666667 0.900816 0 up vs 1ZHONG_SECRETOME_OF_LUNG_CANCER_AND_MACROPHAGE http://www.broadinstitute.org/gsea/msigdb/cards/ZHONG_SECRETOME_OF_LUNG_CANCER_AND_MACROPHAGE 76 0.258881 0.91792 0.62963 0.900846 0 up vs 1LIANG_HEMATOPOIESIS_STEM_CELL_NUMBER_LARGE_VS_TINY_UP http://www.broadinstitute.org/gsea/msigdb/cards/LIANG_HEMATOPOIESIS_STEM_CELL_NUMBER_LARGE_VS_TINY_UP 43 0.249964 0.919225 0.662338 0.900869 0 up vs 1MIKKELSEN_ES_ICP_WITH_H3K27ME3 http://www.broadinstitute.org/gsea/msigdb/cards/MIKKELSEN_ES_ICP_WITH_H3K27ME3 41 0.227256 0.812019 0.783333 0.901058 0 up vs 1FARMER_BREAST_CANCER_APOCRINE_VS_LUMINAL http://www.broadinstitute.org/gsea/msigdb/cards/FARMER_BREAST_CANCER_APOCRINE_VS_LUMINAL 317 0.20485 0.823269 0.662791 0.901155 0 up vs 1SCHAEFFER_PROSTATE_DEVELOPMENT_6HR_UP http://www.broadinstitute.org/gsea/msigdb/cards/SCHAEFFER_PROSTATE_DEVELOPMENT_6HR_UP 164 0.218493 0.822592 0.722892 0.901175 0 up vs 1YAO_TEMPORAL_RESPONSE_TO_PROGESTERONE_CLUSTER_15 http://www.broadinstitute.org/gsea/msigdb/cards/YAO_TEMPORAL_RESPONSE_TO_PROGESTERONE_CLUSTER_15 34 0.233492 0.814796 0.767123 0.901175 0 up vs 1LI_WILMS_TUMOR_VS_FETAL_KIDNEY_1_DN http://www.broadinstitute.org/gsea/msigdb/cards/LI_WILMS_TUMOR_VS_FETAL_KIDNEY_1_DN 159 0.265678 0.812463 0.753425 0.901182 0 up vs 1HORIUCHI_WTAP_TARGETS_DN http://www.broadinstitute.org/gsea/msigdb/cards/HORIUCHI_WTAP_TARGETS_DN 300 0.242546 0.82202 0.694118 0.901188 0 up vs 1MEISSNER_NPC_HCP_WITH_H3K4ME2 http://www.broadinstitute.org/gsea/msigdb/cards/MEISSNER_NPC_HCP_WITH_H3K4ME2 479 0.217688 0.918158 0.54023 0.901294 0 up vs 1BOQUEST_STEM_CELL_UP http://www.broadinstitute.org/gsea/msigdb/cards/BOQUEST_STEM_CELL_UP 257 0.257241 0.915467 0.647887 0.901309 0 up vs 1MANALO_HYPOXIA_DN http://www.broadinstitute.org/gsea/msigdb/cards/MANALO_HYPOXIA_DN 284 0.305883 0.916745 0.576923 0.901309 0 up vs 1CHEMELLO_SOLEUS_VS_EDL_MYOFIBERS_DN http://www.broadinstitute.org/gsea/msigdb/cards/CHEMELLO_SOLEUS_VS_EDL_MYOFIBERS_DN 19 0.292571 0.916357 0.619718 0.901325 0 up vs 1GENTILE_UV_RESPONSE_CLUSTER_D5 http://www.broadinstitute.org/gsea/msigdb/cards/GENTILE_UV_RESPONSE_CLUSTER_D5 37 0.297985 0.915885 0.597403 0.901333 0 up vs 1FRASOR_RESPONSE_TO_ESTRADIOL_DN http://www.broadinstitute.org/gsea/msigdb/cards/FRASOR_RESPONSE_TO_ESTRADIOL_DN 79 0.227964 0.826375 0.783784 0.90134 0 up vs 1HELLER_HDAC_TARGETS_SILENCED_BY_METHYLATION_UP http://www.broadinstitute.org/gsea/msigdb/cards/HELLER_HDAC_TARGETS_SILENCED_BY_METHYLATION_UP 442 0.190991 0.813413 0.615385 0.901435 0 up vs 1SHEPARD_CRUSH_AND_BURN_MUTANT_DN http://www.broadinstitute.org/gsea/msigdb/cards/SHEPARD_CRUSH_AND_BURN_MUTANT_DN 177 0.230615 0.828157 0.74359 0.901454 0 up vs 1KASLER_HDAC7_TARGETS_1_UP http://www.broadinstitute.org/gsea/msigdb/cards/KASLER_HDAC7_TARGETS_1_UP 191 0.201481 0.813933 0.658824 0.90152 0 up vs 1HEIDENBLAD_AMPLICON_12P11_12_UP http://www.broadinstitute.org/gsea/msigdb/cards/HEIDENBLAD_AMPLICON_12P11_12_UP 32 0.255805 0.82349 0.777778 0.901558 0 up vs 1FRASOR_RESPONSE_TO_SERM_OR_FULVESTRANT_UP http://www.broadinstitute.org/gsea/msigdb/cards/FRASOR_RESPONSE_TO_SERM_OR_FULVESTRANT_UP 21 0.285009 0.812665 0.626667 0.901689 0 up vs 1BROWNE_HCMV_INFECTION_20HR_DN http://www.broadinstitute.org/gsea/msigdb/cards/BROWNE_HCMV_INFECTION_20HR_DN 98 0.187763 0.652983 0.868421 0.901791 0 up vs 1WOOD_EBV_EBNA1_TARGETS_DN http://www.broadinstitute.org/gsea/msigdb/cards/WOOD_EBV_EBNA1_TARGETS_DN 47 0.187523 0.651527 0.823529 0.901909 0 up vs 1BROWNE_HCMV_INFECTION_24HR_DN http://www.broadinstitute.org/gsea/msigdb/cards/BROWNE_HCMV_INFECTION_24HR_DN 144 0.219591 0.828243 0.7 0.902126 0 up vs 1IZADPANAH_STEM_CELL_ADIPOSE_VS_BONE_DN http://www.broadinstitute.org/gsea/msigdb/cards/IZADPANAH_STEM_CELL_ADIPOSE_VS_BONE_DN 105 0.25033 0.914326 0.69863 0.902136 0 up vs 1OSMAN_BLADDER_CANCER_UP http://www.broadinstitute.org/gsea/msigdb/cards/OSMAN_BLADDER_CANCER_UP 392 0.189156 0.651794 0.82716 0.902299 0 up vs 1GOTZMANN_EPITHELIAL_TO_MESENCHYMAL_TRANSITION_DN http://www.broadinstitute.org/gsea/msigdb/cards/GOTZMANN_EPITHELIAL_TO_MESENCHYMAL_TRANSITION_DN 204 0.211866 0.829715 0.725275 0.902348 0 up vs 1CHOW_RASSF1_TARGETS_DN http://www.broadinstitute.org/gsea/msigdb/cards/CHOW_RASSF1_TARGETS_DN 29 0.263666 0.913689 0.636364 0.902439 0 up vs 1ROY_WOUND_BLOOD_VESSEL_UP http://www.broadinstitute.org/gsea/msigdb/cards/ROY_WOUND_BLOOD_VESSEL_UP 49 0.260496 0.830087 0.764706 0.902523 0 up vs 1SHETH_LIVER_CANCER_VS_TXNIP_LOSS_PAM5 http://www.broadinstitute.org/gsea/msigdb/cards/SHETH_LIVER_CANCER_VS_TXNIP_LOSS_PAM5 91 0.22 0.828342 0.684211 0.902713 0 up vs 1PENG_RAPAMYCIN_RESPONSE_UP http://www.broadinstitute.org/gsea/msigdb/cards/PENG_RAPAMYCIN_RESPONSE_UP 197 0.21337 0.828753 0.692308 0.902807 0 up vs 1HUTTMANN_B_CLL_POOR_SURVIVAL_DN http://www.broadinstitute.org/gsea/msigdb/cards/HUTTMANN_B_CLL_POOR_SURVIVAL_DN 60 0.236879 0.830156 0.676056 0.903119 0 up vs 1LIANG_HEMATOPOIESIS_STEM_CELL_NUMBER_SMALL_VS_HUGE_UP http://www.broadinstitute.org/gsea/msigdb/cards/LIANG_HEMATOPOIESIS_STEM_CELL_NUMBER_SMALL_VS_HUGE_UP 37 0.25428 0.839228 0.7375 0.903297 0 up vs 1LINDGREN_BLADDER_CANCER_HIGH_RECURRENCE http://www.broadinstitute.org/gsea/msigdb/cards/LINDGREN_BLADDER_CANCER_HIGH_RECURRENCE 49 0.283131 0.854282 0.646154 0.90351 0 up vs 1FAELT_B_CLL_WITH_VH3_21_UP http://www.broadinstitute.org/gsea/msigdb/cards/FAELT_B_CLL_WITH_VH3_21_UP 42 0.277349 0.905742 0.519481 0.903638 0 up vs 1SUBTIL_PROGESTIN_TARGETS http://www.broadinstitute.org/gsea/msigdb/cards/SUBTIL_PROGESTIN_TARGETS 36 0.24043 0.854646 0.693333 0.903666 0 up vs 1RIZKI_TUMOR_INVASIVENESS_3D_UP http://www.broadinstitute.org/gsea/msigdb/cards/RIZKI_TUMOR_INVASIVENESS_3D_UP 204 0.206217 0.857569 0.617977 0.903818 0 up vs 1SEIDEN_ONCOGENESIS_BY_MET http://www.broadinstitute.org/gsea/msigdb/cards/SEIDEN_ONCOGENESIS_BY_MET 86 0.282727 0.847959 0.666667 0.903859 0 up vs 1GOTZMANN_EPITHELIAL_TO_MESENCHYMAL_TRANSITION_UP http://www.broadinstitute.org/gsea/msigdb/cards/GOTZMANN_EPITHELIAL_TO_MESENCHYMAL_TRANSITION_UP 68 0.241876 0.830158 0.7375 0.903863 0 up vs 1BYSTRYKH_HEMATOPOIESIS_STEM_CELL_QTL_CIS http://www.broadinstitute.org/gsea/msigdb/cards/BYSTRYKH_HEMATOPOIESIS_STEM_CELL_QTL_CIS 118 0.225024 0.854978 0.728395 0.903919 0 up vs 1QI_HYPOXIA_TARGETS_OF_HIF1A_AND_FOXA2 http://www.broadinstitute.org/gsea/msigdb/cards/QI_HYPOXIA_TARGETS_OF_HIF1A_AND_FOXA2 37 0.239567 0.839268 0.769231 0.903957 0 up vs 1KAYO_CALORIE_RESTRICTION_MUSCLE_DN http://www.broadinstitute.org/gsea/msigdb/cards/KAYO_CALORIE_RESTRICTION_MUSCLE_DN 85 0.223092 0.906047 0.566667 0.903978 0 up vs 1NEWMAN_ERCC6_TARGETS_DN http://www.broadinstitute.org/gsea/msigdb/cards/NEWMAN_ERCC6_TARGETS_DN 38 0.268709 0.904168 0.61194 0.903998 0 up vs 1VANASSE_BCL2_TARGETS_DN http://www.broadinstitute.org/gsea/msigdb/cards/VANASSE_BCL2_TARGETS_DN 70 0.237704 0.838137 0.717949 0.90404 0 up vs 1GROSS_HYPOXIA_VIA_ELK3_AND_HIF1A_UP http://www.broadinstitute.org/gsea/msigdb/cards/GROSS_HYPOXIA_VIA_ELK3_AND_HIF1A_UP 141 0.219751 0.837211 0.691358 0.904083 0 up vs 1MIKKELSEN_ES_ICP_WITH_H3K4ME3_AND_H3K27ME3 http://www.broadinstitute.org/gsea/msigdb/cards/MIKKELSEN_ES_ICP_WITH_H3K4ME3_AND_H3K27ME3 134 0.228077 0.905014 0.594595 0.904088 0 up vs 1FLECHNER_PBL_KIDNEY_TRANSPLANT_OK_VS_DONOR_DN http://www.broadinstitute.org/gsea/msigdb/cards/FLECHNER_PBL_KIDNEY_TRANSPLANT_OK_VS_DONOR_DN 41 0.264126 0.839681 0.7125 0.904122 0 up vs 1SENESE_HDAC1_AND_HDAC2_TARGETS_DN http://www.broadinstitute.org/gsea/msigdb/cards/SENESE_HDAC1_AND_HDAC2_TARGETS_DN 219 0.235319 0.909382 0.64557 0.904146 0 up vs 1NICK_RESPONSE_TO_PROC_TREATMENT_DN http://www.broadinstitute.org/gsea/msigdb/cards/NICK_RESPONSE_TO_PROC_TREATMENT_DN 25 0.219762 0.648388 0.915493 0.90418 0 up vs 1HUMMERICH_BENIGN_SKIN_TUMOR_DN http://www.broadinstitute.org/gsea/msigdb/cards/HUMMERICH_BENIGN_SKIN_TUMOR_DN 18 0.26499 0.847245 0.731343 0.904194 0 up vs 1JIANG_TIP30_TARGETS_UP http://www.broadinstitute.org/gsea/msigdb/cards/JIANG_TIP30_TARGETS_UP 44 0.239007 0.832038 0.721519 0.904214 0 up vs 1FARMER_BREAST_CANCER_BASAL_VS_LULMINAL http://www.broadinstitute.org/gsea/msigdb/cards/FARMER_BREAST_CANCER_BASAL_VS_LULMINAL 324 0.215941 0.848219 0.666667 0.904261 0 up vs 1DURAND_STROMA_NS_UP http://www.broadinstitute.org/gsea/msigdb/cards/DURAND_STROMA_NS_UP 158 0.202415 0.830423 0.666667 0.904293 0 up vs 1ROZANOV_MMP14_TARGETS_UP http://www.broadinstitute.org/gsea/msigdb/cards/ROZANOV_MMP14_TARGETS_UP 261 0.218411 0.835023 0.705128 0.904323 0 up vs 1KYNG_DNA_DAMAGE_BY_GAMMA_AND_UV_RADIATION http://www.broadinstitute.org/gsea/msigdb/cards/KYNG_DNA_DAMAGE_BY_GAMMA_AND_UV_RADIATION 88 0.244421 0.907192 0.670886 0.904363 0 up vs 1BROWNE_HCMV_INFECTION_4HR_UP http://www.broadinstitute.org/gsea/msigdb/cards/BROWNE_HCMV_INFECTION_4HR_UP 54 0.255596 0.906302 0.625 0.904388 0 up vs 1SCHUETZ_BREAST_CANCER_DUCTAL_INVASIVE_DN http://www.broadinstitute.org/gsea/msigdb/cards/SCHUETZ_BREAST_CANCER_DUCTAL_INVASIVE_DN 83 0.221584 0.837508 0.6875 0.90439 0 up vs 1ACEVEDO_LIVER_TUMOR_VS_NORMAL_ADJACENT_TISSUE_DN http://www.broadinstitute.org/gsea/msigdb/cards/ACEVEDO_LIVER_TUMOR_VS_NORMAL_ADJACENT_TISSUE_DN 267 0.209933 0.841364 0.6875 0.904425 0 up vs 1DELASERNA_MYOD_TARGETS_DN http://www.broadinstitute.org/gsea/msigdb/cards/DELASERNA_MYOD_TARGETS_DN 56 0.225412 0.857679 0.653333 0.904437 0 up vs 1FIGUEROA_AML_METHYLATION_CLUSTER_5_DN http://www.broadinstitute.org/gsea/msigdb/cards/FIGUEROA_AML_METHYLATION_CLUSTER_5_DN 48 0.243539 0.855051 0.712121 0.90457 0 up vs 1WU_HBX_TARGETS_2_UP http://www.broadinstitute.org/gsea/msigdb/cards/WU_HBX_TARGETS_2_UP 23 0.268007 0.851311 0.736842 0.904602 0 up vs 1HEIDENBLAD_AMPLICON_8Q24_DN http://www.broadinstitute.org/gsea/msigdb/cards/HEIDENBLAD_AMPLICON_8Q24_DN 44 0.241513 0.849079 0.695122 0.904618 0 up vs 1PURBEY_TARGETS_OF_CTBP1_NOT_SATB1_UP http://www.broadinstitute.org/gsea/msigdb/cards/PURBEY_TARGETS_OF_CTBP1_NOT_SATB1_UP 328 0.215392 0.910393 0.576087 0.904643 0 up vs 1ACEVEDO_LIVER_CANCER_WITH_H3K27ME3_UP http://www.broadinstitute.org/gsea/msigdb/cards/ACEVEDO_LIVER_CANCER_WITH_H3K27ME3_UP 277 0.223667 0.904259 0.61039 0.904651 0 up vs 1TONKS_TARGETS_OF_RUNX1_RUNX1T1_FUSION_GRANULOCYTE_UP http://www.broadinstitute.org/gsea/msigdb/cards/TONKS_TARGETS_OF_RUNX1_RUNX1T1_FUSION_GRANULOCYTE_UP 53 0.257257 0.909534 0.644737 0.904667 0 up vs 1GALLUZZI_PERMEABILIZE_MITOCHONDRIA http://www.broadinstitute.org/gsea/msigdb/cards/GALLUZZI_PERMEABILIZE_MITOCHONDRIA 43 0.237516 0.839786 0.756098 0.904689 0 up vs 1LEE_LIVER_CANCER_MYC_UP http://www.broadinstitute.org/gsea/msigdb/cards/LEE_LIVER_CANCER_MYC_UP 54 0.233018 0.848438 0.775 0.904726 0 up vs 1COWLING_MYCN_TARGETS http://www.broadinstitute.org/gsea/msigdb/cards/COWLING_MYCN_TARGETS 42 0.258469 0.850045 0.771429 0.904728 0 up vs 1YE_METASTATIC_LIVER_CANCER http://www.broadinstitute.org/gsea/msigdb/cards/YE_METASTATIC_LIVER_CANCER 26 0.247792 0.845323 0.716418 0.90477 0 up vs 1LIU_SOX4_TARGETS_UP http://www.broadinstitute.org/gsea/msigdb/cards/LIU_SOX4_TARGETS_UP 132 0.231654 0.850659 0.646341 0.904772 0 up vs 1PATTERSON_DOCETAXEL_RESISTANCE http://www.broadinstitute.org/gsea/msigdb/cards/PATTERSON_DOCETAXEL_RESISTANCE 29 0.255546 0.835192 0.779412 0.904785 0 up vs 1SU_LIVER http://www.broadinstitute.org/gsea/msigdb/cards/SU_LIVER 55 0.237734 0.846228 0.727273 0.904789 0 up vs 1KENNY_CTNNB1_TARGETS_DN http://www.broadinstitute.org/gsea/msigdb/cards/KENNY_CTNNB1_TARGETS_DN 52 0.259171 0.909882 0.679487 0.904795 0 up vs 1KYNG_DNA_DAMAGE_BY_GAMMA_RADIATION http://www.broadinstitute.org/gsea/msigdb/cards/KYNG_DNA_DAMAGE_BY_GAMMA_RADIATION 81 0.223176 0.832086 0.723684 0.904894 0 up vs 1WIKMAN_ASBESTOS_LUNG_CANCER_DN http://www.broadinstitute.org/gsea/msigdb/cards/WIKMAN_ASBESTOS_LUNG_CANCER_DN 27 0.285759 0.840096 0.602941 0.904903 0 up vs 1STONER_ESOPHAGEAL_CARCINOGENESIS_UP http://www.broadinstitute.org/gsea/msigdb/cards/STONER_ESOPHAGEAL_CARCINOGENESIS_UP 37 0.250851 0.907722 0.705128 0.904932 0 up vs 1ZHU_CMV_24_HR_DN http://www.broadinstitute.org/gsea/msigdb/cards/ZHU_CMV_24_HR_DN 88 0.246477 0.830469 0.75 0.904946 0 up vs 1KIM_WT1_TARGETS_12HR_DN http://www.broadinstitute.org/gsea/msigdb/cards/KIM_WT1_TARGETS_12HR_DN 203 0.220772 0.841481 0.764045 0.904994 0 up vs 1CERVERA_SDHB_TARGETS_2 http://www.broadinstitute.org/gsea/msigdb/cards/CERVERA_SDHB_TARGETS_2 112 0.221762 0.83352 0.72973 0.905032 0 up vs 1WELCSH_BRCA1_TARGETS_UP http://www.broadinstitute.org/gsea/msigdb/cards/WELCSH_BRCA1_TARGETS_UP 196 0.234282 0.832523 0.741176 0.905045 0 up vs 1KIM_TIAL1_TARGETS http://www.broadinstitute.org/gsea/msigdb/cards/KIM_TIAL1_TARGETS 32 0.281685 0.90729 0.571429 0.905113 0 up vs 1WANG_CISPLATIN_RESPONSE_AND_XPC_DN http://www.broadinstitute.org/gsea/msigdb/cards/WANG_CISPLATIN_RESPONSE_AND_XPC_DN 223 0.233338 0.906344 0.670455 0.905129 0 up vs 1HOWLIN_PUBERTAL_MAMMARY_GLAND http://www.broadinstitute.org/gsea/msigdb/cards/HOWLIN_PUBERTAL_MAMMARY_GLAND 67 0.241922 0.857735 0.73913 0.905134 0 up vs 1KARLSSON_TGFB1_TARGETS_DN http://www.broadinstitute.org/gsea/msigdb/cards/KARLSSON_TGFB1_TARGETS_DN 203 0.221879 0.833944 0.708861 0.905176 0 up vs 1GARGALOVIC_RESPONSE_TO_OXIDIZED_PHOSPHOLIPIDS_BLACK_UP http://www.broadinstitute.org/gsea/msigdb/cards/GARGALOVIC_RESPONSE_TO_OXIDIZED_PHOSPHOLIPIDS_BLACK_UP 34 0.257331 0.849185 0.739726 0.905236 0 up vs 1KORKOLA_TERATOMA http://www.broadinstitute.org/gsea/msigdb/cards/KORKOLA_TERATOMA 39 0.236342 0.835428 0.75 0.905247 0 up vs 1FONTAINE_PAPILLARY_THYROID_CARCINOMA_UP http://www.broadinstitute.org/gsea/msigdb/cards/FONTAINE_PAPILLARY_THYROID_CARCINOMA_UP 62 0.223301 0.832875 0.678571 0.90525 0 up vs 1KIM_MYC_AMPLIFICATION_TARGETS_DN http://www.broadinstitute.org/gsea/msigdb/cards/KIM_MYC_AMPLIFICATION_TARGETS_DN 93 0.240269 0.85134 0.653333 0.905291 0 up vs 1WANG_SMARCE1_TARGETS_DN http://www.broadinstitute.org/gsea/msigdb/cards/WANG_SMARCE1_TARGETS_DN 362 0.219973 0.842216 0.707865 0.905322 0 up vs 1RIZ_ERYTHROID_DIFFERENTIATION_CCNE1 http://www.broadinstitute.org/gsea/msigdb/cards/RIZ_ERYTHROID_DIFFERENTIATION_CCNE1 39 0.262622 0.85506 0.689189 0.905333 0 up vs 1MOOTHA_TCA http://www.broadinstitute.org/gsea/msigdb/cards/MOOTHA_TCA 16 0.333897 0.841747 0.6 0.905339 0 up vs 1GROSS_HYPOXIA_VIA_ELK3_ONLY_DN http://www.broadinstitute.org/gsea/msigdb/cards/GROSS_HYPOXIA_VIA_ELK3_ONLY_DN 41 0.230165 0.840275 0.702703 0.905356 0 up vs 1GERHOLD_ADIPOGENESIS_UP http://www.broadinstitute.org/gsea/msigdb/cards/GERHOLD_ADIPOGENESIS_UP 49 0.244345 0.910468 0.670732 0.905368 0 up vs 1LEIN_CHOROID_PLEXUS_MARKERS http://www.broadinstitute.org/gsea/msigdb/cards/LEIN_CHOROID_PLEXUS_MARKERS 101 0.243005 0.903035 0.621622 0.90537 0 up vs 1SU_PANCREAS http://www.broadinstitute.org/gsea/msigdb/cards/SU_PANCREAS 52 0.25938 0.908315 0.703125 0.905379 0 up vs 1MIZUSHIMA_AUTOPHAGOSOME_FORMATION http://www.broadinstitute.org/gsea/msigdb/cards/MIZUSHIMA_AUTOPHAGOSOME_FORMATION 19 0.221292 0.646241 0.844156 0.905397 0 up vs 1CHIARETTI_T_ALL_REFRACTORY_TO_THERAPY http://www.broadinstitute.org/gsea/msigdb/cards/CHIARETTI_T_ALL_REFRACTORY_TO_THERAPY 30 0.273227 0.911299 0.59375 0.905446 0 up vs 1RODRIGUES_DCC_TARGETS_DN http://www.broadinstitute.org/gsea/msigdb/cards/RODRIGUES_DCC_TARGETS_DN 120 0.230495 0.845378 0.705128 0.905466 0 up vs 1PENG_GLUCOSE_DEPRIVATION_DN http://www.broadinstitute.org/gsea/msigdb/cards/PENG_GLUCOSE_DEPRIVATION_DN 165 0.24459 0.910783 0.636364 0.905558 0 up vs 1VALK_AML_WITH_FLT3_ITD http://www.broadinstitute.org/gsea/msigdb/cards/VALK_AML_WITH_FLT3_ITD 40 0.251537 0.855374 0.732394 0.905568 0 up vs 1RIZ_ERYTHROID_DIFFERENTIATION_APOBEC2 http://www.broadinstitute.org/gsea/msigdb/cards/RIZ_ERYTHROID_DIFFERENTIATION_APOBEC2 25 0.264712 0.842529 0.764706 0.905571 0 up vs 1CORRE_MULTIPLE_MYELOMA_UP http://www.broadinstitute.org/gsea/msigdb/cards/CORRE_MULTIPLE_MYELOMA_UP 67 0.235481 0.830548 0.736111 0.905579 0 up vs 1VECCHI_GASTRIC_CANCER_ADVANCED_VS_EARLY_UP http://www.broadinstitute.org/gsea/msigdb/cards/VECCHI_GASTRIC_CANCER_ADVANCED_VS_EARLY_UP 169 0.251457 0.835749 0.724638 0.905581 0 up vs 1DARWICHE_SQUAMOUS_CELL_CARCINOMA_UP http://www.broadinstitute.org/gsea/msigdb/cards/DARWICHE_SQUAMOUS_CELL_CARCINOMA_UP 142 0.205199 0.855838 0.623656 0.905592 0 up vs 1AUNG_GASTRIC_CANCER http://www.broadinstitute.org/gsea/msigdb/cards/AUNG_GASTRIC_CANCER 53 0.240681 0.907759 0.697674 0.905699 0 up vs 1TSENG_ADIPOGENIC_POTENTIAL_DN http://www.broadinstitute.org/gsea/msigdb/cards/TSENG_ADIPOGENIC_POTENTIAL_DN 46 0.254192 0.85782 0.690141 0.905755 0 up vs 1THILLAINADESAN_ZNF217_TARGETS_UP http://www.broadinstitute.org/gsea/msigdb/cards/THILLAINADESAN_ZNF217_TARGETS_UP 44 0.286512 0.851364 0.653333 0.90601 0 up vs 1PEREZ_TP53_AND_TP63_TARGETS http://www.broadinstitute.org/gsea/msigdb/cards/PEREZ_TP53_AND_TP63_TARGETS 197 0.237887 0.896661 0.633803 0.906074 0 up vs 1LIN_MELANOMA_COPY_NUMBER_DN http://www.broadinstitute.org/gsea/msigdb/cards/LIN_MELANOMA_COPY_NUMBER_DN 41 0.274264 0.842662 0.6375 0.906184 0 up vs 1GERHOLD_ADIPOGENESIS_DN http://www.broadinstitute.org/gsea/msigdb/cards/GERHOLD_ADIPOGENESIS_DN 63 0.260028 0.852169 0.708333 0.906219 0 up vs 1HOEBEKE_LYMPHOID_STEM_CELL_UP http://www.broadinstitute.org/gsea/msigdb/cards/HOEBEKE_LYMPHOID_STEM_CELL_UP 93 0.18774 0.644473 0.850746 0.90625 0 up vs 1CLASPER_LYMPHATIC_VESSELS_DURING_METASTASIS_UP http://www.broadinstitute.org/gsea/msigdb/cards/CLASPER_LYMPHATIC_VESSELS_DURING_METASTASIS_UP 19 0.293818 0.851664 0.652778 0.906271 0 up vs 1GROSS_HYPOXIA_VIA_ELK3_UP http://www.broadinstitute.org/gsea/msigdb/cards/GROSS_HYPOXIA_VIA_ELK3_UP 202 0.225586 0.858013 0.640449 0.906292 0 up vs 1TING_SILENCED_BY_DICER http://www.broadinstitute.org/gsea/msigdb/cards/TING_SILENCED_BY_DICER 30 0.280535 0.911302 0.642857 0.906314 0 up vs 1ZHAN_MULTIPLE_MYELOMA_SUBGROUPS http://www.broadinstitute.org/gsea/msigdb/cards/ZHAN_MULTIPLE_MYELOMA_SUBGROUPS 30 0.310114 0.842974 0.642857 0.906572 0 up vs 1TRAYNOR_RETT_SYNDROM_DN http://www.broadinstitute.org/gsea/msigdb/cards/TRAYNOR_RETT_SYNDROM_DN 17 0.289617 0.896837 0.628571 0.906623 0 up vs 1WENG_POR_TARGETS_LIVER_DN http://www.broadinstitute.org/gsea/msigdb/cards/WENG_POR_TARGETS_LIVER_DN 20 -0.217683 -0.677116 0.933333 0.906704 0 down vs 1GALE_APL_WITH_FLT3_MUTATED_UP http://www.broadinstitute.org/gsea/msigdb/cards/GALE_APL_WITH_FLT3_MUTATED_UP 56 0.270712 0.843378 0.644737 0.906721 0 up vs 1PETRETTO_HEART_MASS_QTL_CIS_DN http://www.broadinstitute.org/gsea/msigdb/cards/PETRETTO_HEART_MASS_QTL_CIS_DN 24 0.276905 0.884707 0.626667 0.906723 0 up vs 1ZHAN_LATE_DIFFERENTIATION_GENES_UP http://www.broadinstitute.org/gsea/msigdb/cards/ZHAN_LATE_DIFFERENTIATION_GENES_UP 33 0.268561 0.901353 0.632353 0.906811 0 up vs 1RIZ_ERYTHROID_DIFFERENTIATION_HBZ http://www.broadinstitute.org/gsea/msigdb/cards/RIZ_ERYTHROID_DIFFERENTIATION_HBZ 41 0.257731 0.897577 0.64 0.906852 0 up vs 1DAVICIONI_TARGETS_OF_PAX_FOXO1_FUSIONS_UP http://www.broadinstitute.org/gsea/msigdb/cards/DAVICIONI_TARGETS_OF_PAX_FOXO1_FUSIONS_UP 245 0.238407 0.901812 0.654321 0.906885 0 up vs 1MCMURRAY_TP53_HRAS_COOPERATION_RESPONSE_DN http://www.broadinstitute.org/gsea/msigdb/cards/MCMURRAY_TP53_HRAS_COOPERATION_RESPONSE_DN 67 0.233992 0.897108 0.690141 0.906953 0 up vs 1MEISSNER_BRAIN_HCP_WITH_H3K27ME3 http://www.broadinstitute.org/gsea/msigdb/cards/MEISSNER_BRAIN_HCP_WITH_H3K27ME3 266 0.211485 0.858069 0.644737 0.907014 0 up vs 1KRIGE_AMINO_ACID_DEPRIVATION http://www.broadinstitute.org/gsea/msigdb/cards/KRIGE_AMINO_ACID_DEPRIVATION 29 0.294338 0.884999 0.589744 0.907112 0 up vs 1GAUSSMANN_MLL_AF4_FUSION_TARGETS_A_DN http://www.broadinstitute.org/gsea/msigdb/cards/GAUSSMANN_MLL_AF4_FUSION_TARGETS_A_DN 90 0.2372 0.887231 0.684932 0.907191 0 up vs 1SMID_BREAST_CANCER_LUMINAL_A_UP http://www.broadinstitute.org/gsea/msigdb/cards/SMID_BREAST_CANCER_LUMINAL_A_UP 81 0.254642 0.900709 0.642857 0.907232 0 up vs 1YANG_BREAST_CANCER_ESR1_LASER_DN http://www.broadinstitute.org/gsea/msigdb/cards/YANG_BREAST_CANCER_ESR1_LASER_DN 49 0.271611 0.898344 0.6125 0.907272 0 up vs 1TSENG_IRS1_TARGETS_DN http://www.broadinstitute.org/gsea/msigdb/cards/TSENG_IRS1_TARGETS_DN 134 0.233412 0.894536 0.638554 0.90734 0 up vs 1LI_WILMS_TUMOR_VS_FETAL_KIDNEY_2_DN http://www.broadinstitute.org/gsea/msigdb/cards/LI_WILMS_TUMOR_VS_FETAL_KIDNEY_2_DN 51 0.259097 0.89917 0.662162 0.907375 0 up vs 1BURTON_ADIPOGENESIS_2 http://www.broadinstitute.org/gsea/msigdb/cards/BURTON_ADIPOGENESIS_2 72 0.246925 0.895428 0.698795 0.907382 0 up vs 1ROVERSI_GLIOMA_COPY_NUMBER_DN http://www.broadinstitute.org/gsea/msigdb/cards/ROVERSI_GLIOMA_COPY_NUMBER_DN 53 0.253948 0.885742 0.706667 0.907426 0 up vs 1OSADA_ASCL1_TARGETS_UP http://www.broadinstitute.org/gsea/msigdb/cards/OSADA_ASCL1_TARGETS_UP 46 0.243011 0.859274 0.768116 0.907445 0 up vs 1CHIBA_RESPONSE_TO_TSA http://www.broadinstitute.org/gsea/msigdb/cards/CHIBA_RESPONSE_TO_TSA 46 0.248999 0.859762 0.767123 0.907479 0 up vs 1DOANE_BREAST_CANCER_ESR1_UP http://www.broadinstitute.org/gsea/msigdb/cards/DOANE_BREAST_CANCER_ESR1_UP 110 0.254894 0.894901 0.712329 0.907527 0 up vs 1JOHNSTONE_PARVB_TARGETS_2_DN http://www.broadinstitute.org/gsea/msigdb/cards/JOHNSTONE_PARVB_TARGETS_2_DN 321 0.26543 0.897664 0.642857 0.907539 0 up vs 1SHARMA_PILOCYTIC_ASTROCYTOMA_LOCATION_UP http://www.broadinstitute.org/gsea/msigdb/cards/SHARMA_PILOCYTIC_ASTROCYTOMA_LOCATION_UP 24 0.275812 0.858184 0.638889 0.90754 0 up vs 1BILD_E2F3_ONCOGENIC_SIGNATURE http://www.broadinstitute.org/gsea/msigdb/cards/BILD_E2F3_ONCOGENIC_SIGNATURE 238 0.242403 0.885179 0.683544 0.907561 0 up vs 1SMITH_TERT_TARGETS_DN http://www.broadinstitute.org/gsea/msigdb/cards/SMITH_TERT_TARGETS_DN 87 0.232164 0.887497 0.678161 0.907568 0 up vs 1TAKEDA_TARGETS_OF_NUP98_HOXA9_FUSION_6HR_UP http://www.broadinstitute.org/gsea/msigdb/cards/TAKEDA_TARGETS_OF_NUP98_HOXA9_FUSION_6HR_UP 83 0.246576 0.898563 0.720588 0.907642 0 up vs 1SENGUPTA_NASOPHARYNGEAL_CARCINOMA_WITH_LMP1_UP http://www.broadinstitute.org/gsea/msigdb/cards/SENGUPTA_NASOPHARYNGEAL_CARCINOMA_WITH_LMP1_UP 396 0.242139 0.890191 0.654321 0.907737 0 up vs 1BASSO_CD40_SIGNALING_DN http://www.broadinstitute.org/gsea/msigdb/cards/BASSO_CD40_SIGNALING_DN 68 0.232337 0.859987 0.666667 0.907809 0 up vs 1RIGGINS_TAMOXIFEN_RESISTANCE_UP http://www.broadinstitute.org/gsea/msigdb/cards/RIGGINS_TAMOXIFEN_RESISTANCE_UP 65 0.245283 0.885975 0.741935 0.907817 0 up vs 1FINETTI_BREAST_CANCERS_KINOME_GRAY http://www.broadinstitute.org/gsea/msigdb/cards/FINETTI_BREAST_CANCERS_KINOME_GRAY 15 -0.275693 -0.675098 0.862069 0.907862 0 down vs 1KYNG_WERNER_SYNDROM_DN http://www.broadinstitute.org/gsea/msigdb/cards/KYNG_WERNER_SYNDROM_DN 27 0.281782 0.899309 0.643836 0.90789 0 up vs 1FARMER_BREAST_CANCER_CLUSTER_5 http://www.broadinstitute.org/gsea/msigdb/cards/FARMER_BREAST_CANCER_CLUSTER_5 18 0.352295 0.888296 0.5 0.907907 0 up vs 1CAIRO_LIVER_DEVELOPMENT_DN http://www.broadinstitute.org/gsea/msigdb/cards/CAIRO_LIVER_DEVELOPMENT_DN 217 0.213391 0.858385 0.658228 0.907975 0 up vs 1LIU_BREAST_CANCER http://www.broadinstitute.org/gsea/msigdb/cards/LIU_BREAST_CANCER 28 0.254277 0.893676 0.701299 0.908019 0 up vs 1COULOUARN_TEMPORAL_TGFB1_SIGNATURE_DN http://www.broadinstitute.org/gsea/msigdb/cards/COULOUARN_TEMPORAL_TGFB1_SIGNATURE_DN 133 0.221621 0.883549 0.647727 0.90803 0 up vs 1WEINMANN_ADAPTATION_TO_HYPOXIA_UP http://www.broadinstitute.org/gsea/msigdb/cards/WEINMANN_ADAPTATION_TO_HYPOXIA_UP 28 0.263415 0.887709 0.681159 0.908064 0 up vs 1RAMASWAMY_METASTASIS_DN http://www.broadinstitute.org/gsea/msigdb/cards/RAMASWAMY_METASTASIS_DN 60 0.244789 0.893218 0.666667 0.908072 0 up vs 1STAEGE_EWING_FAMILY_TUMOR http://www.broadinstitute.org/gsea/msigdb/cards/STAEGE_EWING_FAMILY_TUMOR 32 0.255879 0.889615 0.677419 0.908096 0 up vs 1LIM_MAMMARY_LUMINAL_MATURE_DN http://www.broadinstitute.org/gsea/msigdb/cards/LIM_MAMMARY_LUMINAL_MATURE_DN 98 0.254307 0.886216 0.643836 0.908193 0 up vs 1LEE_LIVER_CANCER_MYC_DN http://www.broadinstitute.org/gsea/msigdb/cards/LEE_LIVER_CANCER_MYC_DN 61 0.232354 0.888624 0.657534 0.908225 0 up vs 1FERREIRA_EWINGS_SARCOMA_UNSTABLE_VS_STABLE_DN http://www.broadinstitute.org/gsea/msigdb/cards/FERREIRA_EWINGS_SARCOMA_UNSTABLE_VS_STABLE_DN 94 0.242855 0.892128 0.714286 0.908315 0 up vs 1DORSEY_GAB2_TARGETS http://www.broadinstitute.org/gsea/msigdb/cards/DORSEY_GAB2_TARGETS 31 0.188724 0.641499 0.929577 0.908331 0 up vs 1RADMACHER_AML_PROGNOSIS http://www.broadinstitute.org/gsea/msigdb/cards/RADMACHER_AML_PROGNOSIS 78 0.245554 0.890295 0.743243 0.908445 0 up vs 1WOTTON_RUNX_TARGETS_DN http://www.broadinstitute.org/gsea/msigdb/cards/WOTTON_RUNX_TARGETS_DN 28 0.266627 0.888974 0.69863 0.90847 0 up vs 1GRANDVAUX_IRF3_TARGETS_DN http://www.broadinstitute.org/gsea/msigdb/cards/GRANDVAUX_IRF3_TARGETS_DN 19 0.281134 0.860586 0.705882 0.908485 0 up vs 1PYEON_CANCER_HEAD_AND_NECK_VS_CERVICAL_DN http://www.broadinstitute.org/gsea/msigdb/cards/PYEON_CANCER_HEAD_AND_NECK_VS_CERVICAL_DN 29 0.18894 0.640723 0.861538 0.908493 0 up vs 1GRABARCZYK_BCL11B_TARGETS_DN http://www.broadinstitute.org/gsea/msigdb/cards/GRABARCZYK_BCL11B_TARGETS_DN 55 0.236602 0.860034 0.670886 0.908521 0 up vs 1SHAFFER_IRF4_TARGETS_IN_PLASMA_CELL_VS_MATURE_B_LYMPHOCYTE http://www.broadinstitute.org/gsea/msigdb/cards/SHAFFER_IRF4_TARGETS_IN_PLASMA_CELL_VS_MATURE_B_LYMPHOCYTE 67 0.246961 0.899417 0.638889 0.908596 0 up vs 1ZHANG_TARGETS_OF_EWSR1_FLI1_FUSION http://www.broadinstitute.org/gsea/msigdb/cards/ZHANG_TARGETS_OF_EWSR1_FLI1_FUSION 86 0.237753 0.8911 0.670886 0.908773 0 up vs 1SCHLINGEMANN_SKIN_CARCINOGENESIS_TPA_DN http://www.broadinstitute.org/gsea/msigdb/cards/SCHLINGEMANN_SKIN_CARCINOGENESIS_TPA_DN 28 0.187434 0.639699 0.898551 0.908794 0 up vs 1OKUMURA_INFLAMMATORY_RESPONSE_LPS http://www.broadinstitute.org/gsea/msigdb/cards/OKUMURA_INFLAMMATORY_RESPONSE_LPS 180 0.225888 0.892235 0.662791 0.908891 0 up vs 1LINDGREN_BLADDER_CANCER_CLUSTER_2A_DN http://www.broadinstitute.org/gsea/msigdb/cards/LINDGREN_BLADDER_CANCER_CLUSTER_2A_DN 139 0.187956 0.638944 0.884615 0.908897 0 up vs 1GUILLAUMOND_KLF10_TARGETS_DN http://www.broadinstitute.org/gsea/msigdb/cards/GUILLAUMOND_KLF10_TARGETS_DN 30 0.265695 0.890473 0.618421 0.908923 0 up vs 1STAMBOLSKY_BOUND_BY_MUTATED_TP53 http://www.broadinstitute.org/gsea/msigdb/cards/STAMBOLSKY_BOUND_BY_MUTATED_TP53 18 0.269304 0.882358 0.675325 0.909201 0 up vs 1TERAMOTO_OPN_TARGETS_CLUSTER_6 http://www.broadinstitute.org/gsea/msigdb/cards/TERAMOTO_OPN_TARGETS_CLUSTER_6 27 0.271478 0.860641 0.735294 0.90922 0 up vs 1VALK_AML_CLUSTER_6 http://www.broadinstitute.org/gsea/msigdb/cards/VALK_AML_CLUSTER_6 33 0.26141 0.891125 0.68 0.909566 0 up vs 1HELLER_HDAC_TARGETS_SILENCED_BY_METHYLATION_DN http://www.broadinstitute.org/gsea/msigdb/cards/HELLER_HDAC_TARGETS_SILENCED_BY_METHYLATION_DN 272 0.215083 0.860832 0.628205 0.909759 0 up vs 1ZHONG_SECRETOME_OF_LUNG_CANCER_AND_FIBROBLAST http://www.broadinstitute.org/gsea/msigdb/cards/ZHONG_SECRETOME_OF_LUNG_CANCER_AND_FIBROBLAST 130 0.229458 0.860878 0.681818 0.910489 0 up vs 1LABBE_WNT3A_TARGETS_DN http://www.broadinstitute.org/gsea/msigdb/cards/LABBE_WNT3A_TARGETS_DN 97 0.224244 0.881089 0.631579 0.91065 0 up vs 1LU_TUMOR_ENDOTHELIAL_MARKERS_UP http://www.broadinstitute.org/gsea/msigdb/cards/LU_TUMOR_ENDOTHELIAL_MARKERS_UP 22 0.28573 0.862657 0.619048 0.910835 0 up vs 1BANDRES_RESPONSE_TO_CARMUSTIN_WITHOUT_MGMT_48HR_UP http://www.broadinstitute.org/gsea/msigdb/cards/BANDRES_RESPONSE_TO_CARMUSTIN_WITHOUT_MGMT_48HR_UP 18 0.27696 0.86294 0.651515 0.911157 0 up vs 1YAO_TEMPORAL_RESPONSE_TO_PROGESTERONE_CLUSTER_9 http://www.broadinstitute.org/gsea/msigdb/cards/YAO_TEMPORAL_RESPONSE_TO_PROGESTERONE_CLUSTER_9 71 0.238482 0.860927 0.765432 0.911207 0 up vs 1ZHAN_V2_LATE_DIFFERENTIATION_GENES http://www.broadinstitute.org/gsea/msigdb/cards/ZHAN_V2_LATE_DIFFERENTIATION_GENES 43 0.248655 0.863218 0.7 0.911486 0 up vs 1EBAUER_MYOGENIC_TARGETS_OF_PAX3_FOXO1_FUSION http://www.broadinstitute.org/gsea/msigdb/cards/EBAUER_MYOGENIC_TARGETS_OF_PAX3_FOXO1_FUSION 50 0.188814 0.63533 0.883117 0.911652 0 up vs 1SWEET_LUNG_CANCER_KRAS_DN http://www.broadinstitute.org/gsea/msigdb/cards/SWEET_LUNG_CANCER_KRAS_DN 429 0.229093 0.880072 0.666667 0.911715 0 up vs 1JAZAG_TGFB1_SIGNALING_VIA_SMAD4_DN http://www.broadinstitute.org/gsea/msigdb/cards/JAZAG_TGFB1_SIGNALING_VIA_SMAD4_DN 66 0.23833 0.863535 0.693333 0.911774 0 up vs 1COATES_MACROPHAGE_M1_VS_M2_DN http://www.broadinstitute.org/gsea/msigdb/cards/COATES_MACROPHAGE_M1_VS_M2_DN 75 0.223446 0.86101 0.689189 0.911877 0 up vs 1WANG_HCP_PROSTATE_CANCER http://www.broadinstitute.org/gsea/msigdb/cards/WANG_HCP_PROSTATE_CANCER 110 0.215145 0.863857 0.666667 0.911976 0 up vs 1YAGI_AML_FAB_MARKERS http://www.broadinstitute.org/gsea/msigdb/cards/YAGI_AML_FAB_MARKERS 188 0.224601 0.878177 0.6625 0.912441 0 up vs 1BROWNE_HCMV_INFECTION_48HR_UP http://www.broadinstitute.org/gsea/msigdb/cards/BROWNE_HCMV_INFECTION_48HR_UP 178 0.220189 0.864012 0.621951 0.912486 0 up vs 1GOLDRATH_IMMUNE_MEMORY http://www.broadinstitute.org/gsea/msigdb/cards/GOLDRATH_IMMUNE_MEMORY 64 0.234532 0.878686 0.6375 0.912488 0 up vs 1KANG_IMMORTALIZED_BY_TERT_UP http://www.broadinstitute.org/gsea/msigdb/cards/KANG_IMMORTALIZED_BY_TERT_UP 89 0.227 0.879128 0.662651 0.912549 0 up vs 1BYSTROEM_CORRELATED_WITH_IL5_UP http://www.broadinstitute.org/gsea/msigdb/cards/BYSTROEM_CORRELATED_WITH_IL5_UP 49 0.231639 0.861037 0.709302 0.912618 0 up vs 1YAGI_AML_WITH_T_8_21_TRANSLOCATION http://www.broadinstitute.org/gsea/msigdb/cards/YAGI_AML_WITH_T_8_21_TRANSLOCATION 356 0.219714 0.864124 0.662651 0.913082 0 up vs 1KIM_MYCN_AMPLIFICATION_TARGETS_DN http://www.broadinstitute.org/gsea/msigdb/cards/KIM_MYCN_AMPLIFICATION_TARGETS_DN 102 0.247332 0.864256 0.657895 0.913701 0 up vs 1LEE_TARGETS_OF_PTCH1_AND_SUFU_UP http://www.broadinstitute.org/gsea/msigdb/cards/LEE_TARGETS_OF_PTCH1_AND_SUFU_UP 50 0.236759 0.864519 0.662162 0.914113 0 up vs 1DOANE_BREAST_CANCER_CLASSES_UP http://www.broadinstitute.org/gsea/msigdb/cards/DOANE_BREAST_CANCER_CLASSES_UP 71 0.249352 0.8649 0.69863 0.914326 0 up vs 1HADDAD_B_LYMPHOCYTE_PROGENITOR http://www.broadinstitute.org/gsea/msigdb/cards/HADDAD_B_LYMPHOCYTE_PROGENITOR 279 0.23305 0.876364 0.653846 0.914561 0 up vs 1ZHANG_TLX_TARGETS_UP http://www.broadinstitute.org/gsea/msigdb/cards/ZHANG_TLX_TARGETS_UP 108 0.231915 0.865249 0.734177 0.914632 0 up vs 1FOSTER_KDM1A_TARGETS_DN http://www.broadinstitute.org/gsea/msigdb/cards/FOSTER_KDM1A_TARGETS_DN 204 0.227237 0.87525 0.681818 0.914669 0 up vs 1BHAT_ESR1_TARGETS_NOT_VIA_AKT1_UP http://www.broadinstitute.org/gsea/msigdb/cards/BHAT_ESR1_TARGETS_NOT_VIA_AKT1_UP 207 0.215385 0.865526 0.638554 0.914938 0 up vs 1ZHOU_TNF_SIGNALING_4HR http://www.broadinstitute.org/gsea/msigdb/cards/ZHOU_TNF_SIGNALING_4HR 54 0.241032 0.870059 0.666667 0.914983 0 up vs 1ZEMBUTSU_SENSITIVITY_TO_FLUOROURACIL http://www.broadinstitute.org/gsea/msigdb/cards/ZEMBUTSU_SENSITIVITY_TO_FLUOROURACIL 17 0.28101 0.865973 0.671233 0.914986 0 up vs 1MCCABE_HOXC6_TARGETS_CANCER_UP http://www.broadinstitute.org/gsea/msigdb/cards/MCCABE_HOXC6_TARGETS_CANCER_UP 30 0.260121 0.869604 0.735294 0.914993 0 up vs 1HAMAI_APOPTOSIS_VIA_TRAIL_DN http://www.broadinstitute.org/gsea/msigdb/cards/HAMAI_APOPTOSIS_VIA_TRAIL_DN 182 0.215472 0.866476 0.662651 0.915006 0 up vs 1CHANG_CORE_SERUM_RESPONSE_UP http://www.broadinstitute.org/gsea/msigdb/cards/CHANG_CORE_SERUM_RESPONSE_UP 207 0.188277 0.630385 0.870588 0.915288 0 up vs 1ZHAN_MULTIPLE_MYELOMA_MS_DN http://www.broadinstitute.org/gsea/msigdb/cards/ZHAN_MULTIPLE_MYELOMA_MS_DN 43 0.249814 0.875298 0.727273 0.915412 0 up vs 1BOYLAN_MULTIPLE_MYELOMA_C_CLUSTER_UP http://www.broadinstitute.org/gsea/msigdb/cards/BOYLAN_MULTIPLE_MYELOMA_C_CLUSTER_UP 37 0.271339 0.873682 0.68 0.915483 0 up vs 1BARRIER_COLON_CANCER_RECURRENCE_DN http://www.broadinstitute.org/gsea/msigdb/cards/BARRIER_COLON_CANCER_RECURRENCE_DN 19 0.271591 0.870233 0.714286 0.915546 0 up vs 1HOOI_ST7_TARGETS_DN http://www.broadinstitute.org/gsea/msigdb/cards/HOOI_ST7_TARGETS_DN 117 0.228597 0.872637 0.693333 0.915647 0 up vs 1ZHAN_MULTIPLE_MYELOMA_CD2_UP http://www.broadinstitute.org/gsea/msigdb/cards/ZHAN_MULTIPLE_MYELOMA_CD2_UP 44 0.274043 0.866579 0.688525 0.915695 0 up vs 1GENTILE_UV_LOW_DOSE_DN http://www.broadinstitute.org/gsea/msigdb/cards/GENTILE_UV_L
[truncated: 26,846 more chars]
