# Supplementary material for: Identification and characterization of HPV-independent cervical cancers
Source: Oncotarget. 2017 Jan 6;8(8):13375–86. doi: 10.18632/oncotarget.14533 (PMC5355105; doi:10.18632/oncotarget.14533)
Supplement: Supplementary file 6 [file oncotarget-08-13375-s006.docx]

Supplemental table 3: Clinical information
bcr_patient_barcode HPV_express race vital_status last_contact_days_to death_days_to age_at_diagnosis histologic_diagnosis tumor_grade clinical_stage
TCGA-2W-A8YY 0 WHITE Alive 148 [Not Applicable] 51 Adenosquamous G3 Stage IB2
TCGA-4J-AA1J 1 WHITE Alive 226 [Not Applicable] 31 Cervical Squamous Cell Carcinoma G3 Stage IB2
TCGA-C5-A1BQ 1 WHITE Dead [Not Available] 604 65 Cervical Squamous Cell Carcinoma G2 Stage IIIB
TCGA-C5-A2LS 1 WHITE Alive 1345 [Not Applicable] 37 Endocervical Adenocarcinoma of the Usual Type G1 Stage IB2
TCGA-C5-A2LT 1 WHITE Alive 2226 [Not Applicable] 38 Cervical Squamous Cell Carcinoma G3 Stage IB
TCGA-C5-A2LV 1 BLACK OR AFRICAN AMERICAN Alive 2234 [Not Applicable] 36 Cervical Squamous Cell Carcinoma G3 Stage IB
TCGA-C5-A2LX 1 WHITE Alive 491 [Not Applicable] 54 Cervical Squamous Cell Carcinoma G2 Stage IB1
TCGA-C5-A2LY 1 WHITE Alive 2383 [Not Applicable] 30 Cervical Squamous Cell Carcinoma G2 Stage IB1
TCGA-C5-A2LZ 1 WHITE Dead [Not Available] 3046 65 Cervical Squamous Cell Carcinoma G2 Stage IIIB
TCGA-C5-A2M1 1 WHITE Alive 1169 [Not Applicable] 37 Endocervical Type of Adenocarcinoma G2 Stage IB1
TCGA-C5-A2M2 1 WHITE Alive 25 [Not Applicable] 56 Endocervical Type of Adenocarcinoma G2 Stage IB2
TCGA-C5-A3HD 1 WHITE Alive 1582 [Not Applicable] 51 Cervical Squamous Cell Carcinoma G2 Stage IIB
TCGA-C5-A3HE 1 WHITE Alive 548 [Not Applicable] 44 Cervical Squamous Cell Carcinoma G3 Stage IB2
TCGA-C5-A3HF 1 WHITE Dead [Not Available] 543 24 Mucinous Adenocarcinoma of Endocervical Type G2 Stage IB2
TCGA-C5-A3HL 1 WHITE Alive 621 [Not Applicable] 76 Cervical Squamous Cell Carcinoma G2 Stage IB2
TCGA-C5-A7CG 1 WHITE Alive 5564 [Not Applicable] 55 Cervical Squamous Cell Carcinoma G2 Stage IB
TCGA-C5-A7CH 1 WHITE Alive 771 [Not Applicable] 43 Cervical Squamous Cell Carcinoma G2 Stage IIB
TCGA-C5-A7CJ 1 WHITE Dead [Not Available] 3097 42 Cervical Squamous Cell Carcinoma G2 Stage IIA
TCGA-C5-A7CK 1 WHITE Dead [Not Available] 4086 58 Cervical Squamous Cell Carcinoma G2 Stage IVA
TCGA-C5-A7CL 1 WHITE Dead [Not Available] 471 48 Cervical Squamous Cell Carcinoma G2 Stage IIIB
TCGA-C5-A7CM 1 WHITE Alive 294 [Not Applicable] 35 Endocervical Adenocarcinoma of the Usual Type G2 Stage IB2
TCGA-C5-A7CO 1 WHITE Alive 2233 [Not Applicable] 68 Cervical Squamous Cell Carcinoma G2 Stage IB2
TCGA-C5-A7UC 1 WHITE Dead [Not Available] 523 48 Cervical Squamous Cell Carcinoma G3 Stage IB
TCGA-C5-A7UE 1 WHITE Alive 2602 [Not Applicable] 45 Cervical Squamous Cell Carcinoma G2 Stage IB1
TCGA-C5-A7UH 1 WHITE Alive 1846 [Not Applicable] 55 Cervical Squamous Cell Carcinoma G3 Stage IIIB
TCGA-C5-A7UI 1 BLACK OR AFRICAN AMERICAN Dead [Not Available] 2888 42 Cervical Squamous Cell Carcinoma G3 Stage IB1
TCGA-C5-A7X3 1 WHITE Dead [Not Available] 284 70 Cervical Squamous Cell Carcinoma G2 Stage IIIB
TCGA-C5-A7X5 1 BLACK OR AFRICAN AMERICAN Dead [Not Available] 414 72 Cervical Squamous Cell Carcinoma G3 Stage IVB
TCGA-C5-A7X8 1 WHITE Alive 83 [Not Applicable] 35 Mucinous Adenocarcinoma of Endocervical Type G2 Stage IB1
TCGA-C5-A7XC 1 WHITE Alive 1551 [Not Applicable] 26 Cervical Squamous Cell Carcinoma G2 Stage IB1
TCGA-C5-A8XH 1 WHITE Dead [Not Available] 1394 39 Cervical Squamous Cell Carcinoma [Not Available] Stage IB1
TCGA-C5-A8XI 1 BLACK OR AFRICAN AMERICAN Alive 254 [Not Applicable] 69 Cervical Squamous Cell Carcinoma G3 Stage IB2
TCGA-C5-A8XJ 1 WHITE Alive 2305 [Not Applicable] 74 Cervical Squamous Cell Carcinoma [Not Available] Stage IB
TCGA-C5-A8XK 1 BLACK OR AFRICAN AMERICAN Alive 3039 [Not Applicable] 30 Cervical Squamous Cell Carcinoma G3 [Not Available]
TCGA-C5-A8YQ 0 WHITE Dead [Not Available] 715 79 Cervical Squamous Cell Carcinoma G2 Stage IB1
TCGA-C5-A8YR 1 WHITE Dead [Not Available] 837 56 Cervical Squamous Cell Carcinoma G3 Stage IB
TCGA-C5-A8YT 0 WHITE Alive 186 [Not Applicable] 36 Cervical Squamous Cell Carcinoma G3 Stage IB1
TCGA-C5-A8ZZ 1 WHITE Alive 636 [Not Applicable] 41 Cervical Squamous Cell Carcinoma G2 Stage IIB
TCGA-C5-A901 1 WHITE Alive 518 [Not Applicable] 44 Cervical Squamous Cell Carcinoma G2 [Not Available]
TCGA-C5-A902 1 BLACK OR AFRICAN AMERICAN Alive 149 [Not Applicable] 35 Cervical Squamous Cell Carcinoma G3 Stage IB2
TCGA-C5-A905 1 BLACK OR AFRICAN AMERICAN Alive 4879 [Not Applicable] 37 Cervical Squamous Cell Carcinoma G2 Stage IB
TCGA-C5-A907 1 WHITE Alive 136 [Not Applicable] 47 Cervical Squamous Cell Carcinoma G2 Stage IB2
TCGA-DG-A2KH 1 [Not Evaluated] Alive 34 [Not Applicable] 25 Adenosquamous GX Stage IB1
TCGA-DG-A2KJ 1 [Not Evaluated] Alive 1791 [Not Applicable] 50 Cervical Squamous Cell Carcinoma G3 Stage IIIB
TCGA-DG-A2KK 1 [Not Evaluated] Alive 1544 [Not Applicable] 51 Cervical Squamous Cell Carcinoma G3 Stage IIIB
TCGA-DG-A2KL 1 [Not Evaluated] Alive 1367 [Not Applicable] 53 Cervical Squamous Cell Carcinoma G1 Stage IIA
TCGA-DG-A2KM 1 [Not Evaluated] Alive 1218 [Not Applicable] 46 Cervical Squamous Cell Carcinoma G2 Stage IB1
TCGA-DS-A3LQ 0 [Not Available] Alive 183 [Not Applicable] 46 Cervical Squamous Cell Carcinoma G3 Stage IIIB
TCGA-DS-A5RQ 1 WHITE Alive 208 [Not Applicable] 80 Cervical Squamous Cell Carcinoma G2 Stage IB1
TCGA-DS-A7WF 1 [Unknown] Dead [Not Available] 492 41 Adenosquamous G3 Stage IB2
TCGA-DS-A7WH 1 WHITE Alive 223 [Not Applicable] 34 Mucinous Adenocarcinoma of Endocervical Type G2 Stage IB1
TCGA-DS-A7WI 1 WHITE Dead [Not Available] 252 43 Cervical Squamous Cell Carcinoma G2 Stage IIA2
TCGA-EA-A1QS 1 WHITE Alive 40 [Not Applicable] 46 Cervical Squamous Cell Carcinoma G2 Stage IB1
TCGA-EA-A3HQ 1 WHITE Alive 65 [Not Applicable] 60 Cervical Squamous Cell Carcinoma G2 Stage II
TCGA-EA-A3HR 1 WHITE Alive 40 [Not Applicable] 57 Cervical Squamous Cell Carcinoma G2 Stage II
TCGA-EA-A3HS 1 WHITE Alive 40 [Not Applicable] 35 Cervical Squamous Cell Carcinoma G1 Stage IB
TCGA-EA-A3HT 1 WHITE Alive 19 [Not Applicable] 68 Cervical Squamous Cell Carcinoma G1 Stage IB
TCGA-EA-A3HU 1 WHITE Alive 81 [Not Applicable] 43 Cervical Squamous Cell Carcinoma G2 Stage II
TCGA-EA-A3QD 1 WHITE Alive 33 [Not Applicable] 59 Cervical Squamous Cell Carcinoma G3 Stage IIIB
TCGA-EA-A3QE 1 WHITE Alive 47 [Not Applicable] 45 Cervical Squamous Cell Carcinoma G2 Stage IB
TCGA-EA-A3Y4 1 WHITE Alive 35 [Not Applicable] 40 Cervical Squamous Cell Carcinoma G3 Stage IB
TCGA-EA-A410 0 WHITE Alive 56 [Not Applicable] 51 Cervical Squamous Cell Carcinoma G2 Stage IIA2
TCGA-EA-A411 1 WHITE Alive 41 [Not Applicable] 50 Cervical Squamous Cell Carcinoma G2 Stage IB1
TCGA-EA-A439 1 ASIAN Alive 20 [Not Applicable] 50 Cervical Squamous Cell Carcinoma G3 Stage IIA1
TCGA-EA-A43B 1 WHITE Alive 55 [Not Applicable] 43 Cervical Squamous Cell Carcinoma G2 Stage IB1
TCGA-EA-A44S 1 WHITE Alive 9 [Not Applicable] 31 Cervical Squamous Cell Carcinoma G2 Stage IIIB
TCGA-EA-A4BA 0 WHITE Alive 10 [Not Applicable] 49 Endocervical Type of Adenocarcinoma G2 Stage IB2
TCGA-EA-A50E 1 ASIAN Alive 8 [Not Applicable] 45 Cervical Squamous Cell Carcinoma G2 Stage IVA
TCGA-EA-A556 0 WHITE Alive 0 [Not Applicable] 38 Endometrioid Adenocarcinoma of Endocervix G3 Stage IB1
TCGA-EA-A5FO 1 WHITE Alive 45 [Not Applicable] 59 Cervical Squamous Cell Carcinoma G2 Stage IB1
TCGA-EA-A5O9 1 WHITE Alive 27 [Not Applicable] 39 Cervical Squamous Cell Carcinoma G2 Stage IB2
TCGA-EA-A5ZD 1 ASIAN Alive 21 [Not Applicable] 40 Cervical Squamous Cell Carcinoma G2 Stage IB1
TCGA-EA-A5ZE 1 ASIAN Alive 22 [Not Applicable] 54 Cervical Squamous Cell Carcinoma G3 Stage IB1
TCGA-EA-A5ZF 1 ASIAN Alive 26 [Not Applicable] 56 Cervical Squamous Cell Carcinoma G2 Stage IB1
TCGA-EA-A6QX 1 ASIAN Alive 19 [Not Applicable] 49 Cervical Squamous Cell Carcinoma G3 Stage IIIB
TCGA-EA-A78R 1 ASIAN Alive 12 [Not Applicable] 54 Cervical Squamous Cell Carcinoma G2 Stage IB1
TCGA-EA-A97N 1 WHITE Alive 11 [Not Applicable] 38 Cervical Squamous Cell Carcinoma G2 Stage IB2
TCGA-EK-A2GZ 1 BLACK OR AFRICAN AMERICAN Alive 383 [Not Applicable] 64 Cervical Squamous Cell Carcinoma G2 Stage IIIB
TCGA-EK-A2H0 1 WHITE Alive 1398 [Not Applicable] 24 Cervical Squamous Cell Carcinoma G3 Stage IIB
TCGA-EK-A2H1 1 WHITE Alive 799 [Not Applicable] 20 Cervical Squamous Cell Carcinoma G3 Stage IB2
TCGA-EK-A2IP 1 WHITE Alive 202 [Not Applicable] 28 Cervical Squamous Cell Carcinoma G3 Stage IB1
TCGA-EK-A2PG 1 BLACK OR AFRICAN AMERICAN Alive 16 [Not Applicable] 88 Cervical Squamous Cell Carcinoma G3 [Not Available]
TCGA-EK-A2PI 1 WHITE Alive 0 [Not Applicable] 44 Cervical Squamous Cell Carcinoma G2 Stage IIIB
TCGA-EK-A2PK 1 WHITE Alive 0 [Not Applicable] 43 Cervical Squamous Cell Carcinoma G3 Stage IB1
TCGA-EK-A2PL 1 WHITE Alive 0 [Not Applicable] 36 Cervical Squamous Cell Carcinoma [Not Available] Stage IIIB
TCGA-EK-A2PM 0 WHITE Alive 0 [Not Applicable] 81 Cervical Squamous Cell Carcinoma G3 Stage IIB
TCGA-EK-A2R7 1 WHITE Alive 27 [Not Applicable] 45 Cervical Squamous Cell Carcinoma G3 Stage IB
TCGA-EK-A2R8 1 WHITE Alive 40 [Not Applicable] 48 Cervical Squamous Cell Carcinoma G3 Stage IB2
TCGA-EK-A2R9 1 WHITE Alive 4 [Not Applicable] 58 Cervical Squamous Cell Carcinoma G3 Stage IB1
TCGA-EK-A2RA 1 [Not Available] Alive 302 [Not Applicable] 74 Cervical Squamous Cell Carcinoma G3 Stage IIA2
TCGA-EK-A2RB 1 WHITE Alive 9 [Not Applicable] 48 Cervical Squamous Cell Carcinoma G3 Stage IVB
TCGA-EK-A2RC 1 [Not Available] Alive 129 [Not Applicable] 33 Cervical Squamous Cell Carcinoma G3 Stage IB1
TCGA-EK-A2RE 1 WHITE Alive 57 [Not Applicable] 26 Cervical Squamous Cell Carcinoma G2 Stage IIA
TCGA-EK-A2RJ 1 WHITE Alive 53 [Not Applicable] 51 Cervical Squamous Cell Carcinoma G3 Stage IB2
TCGA-EK-A2RK 1 WHITE Alive 13 [Not Applicable] 67 Cervical Squamous Cell Carcinoma G3 Stage IA2
TCGA-EK-A2RL 1 BLACK OR AFRICAN AMERICAN Alive 721 [Not Applicable] 32 Endocervical Type of Adenocarcinoma G2 Stage IB
TCGA-EK-A2RM 1 WHITE Alive 50 [Not Applicable] 40 Cervical Squamous Cell Carcinoma G3 Stage IB
TCGA-EK-A2RN 1 WHITE Alive 71 [Not Applicable] 45 Cervical Squamous Cell Carcinoma G2 Stage IB1
TCGA-EK-A2RO 1 AMERICAN INDIAN OR ALASKA NATIVE Alive 2 [Not Applicable] 59 Cervical Squamous Cell Carcinoma G1 Stage IIB
TCGA-EK-A3GJ 1 NATIVE HAWAIIAN OR OTHER PACIFIC ISLANDER Alive 3 [Not Applicable] 51 Cervical Squamous Cell Carcinoma G3 Stage IB1
TCGA-EK-A3GK 1 WHITE Alive 15 [Not Applicable] 33 Endocervical Type of Adenocarcinoma G3 Stage IB1
TCGA-EK-A3GM 1 WHITE Alive 0 [Not Applicable] 65 Endocervical Type of Adenocarcinoma G2 Stage IIA
TCGA-EK-A3GN 1 WHITE Alive 1 [Not Applicable] 47 Cervical Squamous Cell Carcinoma G3 Stage IIIB
TCGA-EX-A1H6 1 WHITE Alive 241 [Not Applicable] 38 Endocervical Type of Adenocarcinoma G1 Stage IB1
TCGA-EX-A3L1 1 WHITE Alive 211 [Not Applicable] 32 Cervical Squamous Cell Carcinoma G3 Stage IIA1
TCGA-EX-A449 1 WHITE Alive 34 [Not Applicable] 42 Endocervical Type of Adenocarcinoma G1 Stage IVB
TCGA-EX-A69L 1 ASIAN Alive 136 [Not Applicable] 41 Cervical Squamous Cell Carcinoma G3 Stage IB1
TCGA-EX-A69M 1 WHITE Alive 186 [Not Applicable] 62 Cervical Squamous Cell Carcinoma G3 Stage IB2
TCGA-EX-A8YF 1 WHITE Alive 144 [Not Applicable] 44 Cervical Squamous Cell Carcinoma G3 Stage IB1
TCGA-FU-A2QG 1 WHITE Alive 28 [Not Applicable] 29 Cervical Squamous Cell Carcinoma G2 Stage IB1
TCGA-FU-A3EO 1 WHITE Alive 66 [Not Applicable] 55 Endocervical Type of Adenocarcinoma G2 Stage IIB
TCGA-FU-A3HY 1 WHITE Alive 20 [Not Applicable] 47 Cervical Squamous Cell Carcinoma G2 Stage IB2
TCGA-FU-A3HZ 0 WHITE Alive 31 [Not Applicable] 64 Cervical Squamous Cell Carcinoma G3 Stage IIA2
TCGA-FU-A3NI 1 WHITE Alive 44 [Not Applicable] 45 Cervical Squamous Cell Carcinoma G2 Stage IB1
TCGA-FU-A3TQ 1 WHITE Alive 48 [Not Applicable] 55 Cervical Squamous Cell Carcinoma G2 Stage IIIB
TCGA-FU-A3TX 1 [Not Evaluated] Alive 45 [Not Applicable] 78 Cervical Squamous Cell Carcinoma G3 Stage IB2
TCGA-FU-A3WB 1 WHITE Alive 25 [Not Applicable] 43 Cervical Squamous Cell Carcinoma G2 Stage IIA2
TCGA-FU-A3YQ 1 WHITE Alive 107 [Not Applicable] 35 Cervical Squamous Cell Carcinoma G1 Stage IB1
TCGA-FU-A40J 1 WHITE Alive 41 [Not Applicable] 38 Endocervical Type of Adenocarcinoma G3 Stage IIIB
TCGA-FU-A57G 0 WHITE Alive 40 [Not Applicable] 49 Endocervical Type of Adenocarcinoma G2 Stage IB2
TCGA-FU-A5XV 1 WHITE Alive 60 [Not Applicable] 32 Cervical Squamous Cell Carcinoma G3 Stage IIIB
TCGA-FU-A770 1 WHITE Alive 34 [Not Applicable] 33 Endocervical Adenocarcinoma of the Usual Type G2 Stage IIIB
TCGA-GH-A9DA 1 WHITE Alive 274 [Not Applicable] 27 Cervical Squamous Cell Carcinoma G3 Stage IB1
TCGA-HG-A2PA 1 WHITE Alive 773 [Not Applicable] 38 Cervical Squamous Cell Carcinoma G2 Stage IB2
TCGA-HM-A3JJ 1 BLACK OR AFRICAN AMERICAN Dead [Not Available] 659 40 Cervical Squamous Cell Carcinoma G3 Stage IB1
TCGA-HM-A3JK 1 WHITE Alive 168 [Not Applicable] 64 Cervical Squamous Cell Carcinoma G3 Stage IIA2
TCGA-HM-A4S6 1 WHITE Alive 244 [Not Applicable] 51 Cervical Squamous Cell Carcinoma G3 Stage IIIB
TCGA-HM-A6W2 0 BLACK OR AFRICAN AMERICAN Alive 125 [Not Applicable] 34 Adenosquamous G3 Stage IVB
TCGA-IR-A3L7 1 WHITE Alive 3509 [Not Applicable] 37 Cervical Squamous Cell Carcinoma G3 Stage IB1
TCGA-IR-A3LA 0 WHITE Alive 3780 [Not Applicable] 60 Endometrioid Adenocarcinoma of Endocervix G3 Stage IB1
TCGA-IR-A3LB 1 WHITE Dead [Not Available] 2032 53 Endocervical Type of Adenocarcinoma G3 Stage IB1
TCGA-IR-A3LC 1 WHITE Alive 3333 [Not Applicable] 40 Cervical Squamous Cell Carcinoma G3 Stage IB1
TCGA-IR-A3LF 1 WHITE Alive 2949 [Not Applicable] 64 Endocervical Adenocarcinoma of the Usual Type G2 Stage IB1
TCGA-IR-A3LH 1 BLACK OR AFRICAN AMERICAN Alive 2394 [Not Applicable] 49 Cervical Squamous Cell Carcinoma G4 Stage IIA1
TCGA-IR-A3LI 1 WHITE Alive 2445 [Not Applicable] 48 Endocervical Type of Adenocarcinoma G2 Stage IVB
TCGA-IR-A3LK 1 WHITE Alive 808 [Not Applicable] 69 Cervical Squamous Cell Carcinoma G3 Stage IB2
TCGA-IR-A3LL 1 WHITE Alive 720 [Not Applicable] 60 Cervical Squamous Cell Carcinoma G2 Stage IB1
TCGA-JW-A5VG 1 AMERICAN INDIAN OR ALASKA NATIVE Alive 439 [Not Applicable] 35 Cervical Squamous Cell Carcinoma G3 Stage IIA
TCGA-JW-A5VH 0 AMERICAN INDIAN OR ALASKA NATIVE Dead [Not Available] 100 53 Cervical Squamous Cell Carcinoma G2 Stage IVB
TCGA-JW-A5VI 1 AMERICAN INDIAN OR ALASKA NATIVE Alive 506 [Not Applicable] 45 Cervical Squamous Cell Carcinoma G3 Stage IIB
TCGA-JW-A5VJ 1 AMERICAN INDIAN OR ALASKA NATIVE Alive 401 [Not Applicable] 56 Cervical Squamous Cell Carcinoma G3 Stage IIB
TCGA-JW-A5VK 0 BLACK OR AFRICAN AMERICAN Alive 198 [Not Applicable] 43 Cervical Squamous Cell Carcinoma G3 Stage IB2
TCGA-JW-A5VL 1 AMERICAN INDIAN OR ALASKA NATIVE Alive 88 [Not Applicable] 37 Cervical Squamous Cell Carcinoma G1 Stage IB2
TCGA-JW-A69B 1 WHITE Alive 306 [Not Applicable] 44 Endocervical Type of Adenocarcinoma GX Stage IB2
TCGA-JW-A852 1 WHITE Alive 156 [Not Applicable] 42 Cervical Squamous Cell Carcinoma G2 Stage IIB
TCGA-JW-AAVH 1 WHITE Alive 204 [Not Applicable] 46 Cervical Squamous Cell Carcinoma G2 Stage IB1
TCGA-JX-A3PZ 1 WHITE Dead [Not Available] 642 25 Cervical Squamous Cell Carcinoma G2 Stage IB
TCGA-JX-A3Q0 1 BLACK OR AFRICAN AMERICAN Alive 5957 [Not Applicable] 63 Cervical Squamous Cell Carcinoma G2 Stage III
TCGA-JX-A3Q8 1 ASIAN Alive 922 [Not Applicable] 40 Endocervical Type of Adenocarcinoma G3 Stage IB1
TCGA-JX-A5QV 1 WHITE Alive 243 [Not Applicable] 37 Cervical Squamous Cell Carcinoma G3 Stage IB1
TCGA-LP-A4AU 1 ASIAN Alive 3 [Not Applicable] 35 Cervical Squamous Cell Carcinoma G3 Stage IIIB
TCGA-LP-A4AV 1 ASIAN Alive 0 [Not Applicable] 63 Cervical Squamous Cell Carcinoma G2 Stage IB
TCGA-LP-A4AW 1 ASIAN Alive 27 [Not Applicable] 52 Cervical Squamous Cell Carcinoma G1 Stage IA
TCGA-LP-A4AX 1 ASIAN Alive 2 [Not Applicable] 45 Cervical Squamous Cell Carcinoma G3 Stage IB1
TCGA-LP-A5U2 1 ASIAN Alive 9 [Not Applicable] 30 Endocervical Adenocarcinoma of the Usual Type G3 Stage IIIB
TCGA-LP-A5U3 1 ASIAN Alive 25 [Not Applicable] 40 Cervical Squamous Cell Carcinoma G3 Stage IB1
TCGA-LP-A7HU 1 ASIAN Alive 6 [Not Applicable] 53 Endocervical Type of Adenocarcinoma G3 Stage II
TCGA-MA-AA3W 1 WHITE Alive 503 [Not Applicable] 54 Cervical Squamous Cell Carcinoma G3 Stage IB1
TCGA-MA-AA3X 1 BLACK OR AFRICAN AMERICAN Alive 617 [Not Applicable] 50 Cervical Squamous Cell Carcinoma G2 Stage IIIB
TCGA-MA-AA3Y 1 WHITE Alive 542 [Not Applicable] 48 Cervical Squamous Cell Carcinoma G3 Stage IB1
TCGA-MA-AA3Z 1 WHITE Alive 469 [Not Applicable] 43 Cervical Squamous Cell Carcinoma GX Stage IB2
TCGA-MA-AA41 1 WHITE Alive 279 [Not Applicable] 33 Cervical Squamous Cell Carcinoma G2 Stage IIB
TCGA-MA-AA42 1 WHITE Alive 168 [Not Applicable] 75 Cervical Squamous Cell Carcinoma G3 Stage IIB
TCGA-MA-AA43 1 WHITE Alive 227 [Not Applicable] 48 Cervical Squamous Cell Carcinoma G3 Stage IIIB
TCGA-MU-A51Y 1 WHITE Alive 422 [Not Applicable] 27 Cervical Squamous Cell Carcinoma G2 Stage IIA1
TCGA-MU-A5YI 1 BLACK OR AFRICAN AMERICAN Alive 1 [Not Applicable] 60 Cervical Squamous Cell Carcinoma G2 Stage IA1
TCGA-MU-A8JM 1 WHITE Alive 165 [Not Applicable] 46 Cervical Squamous Cell Carcinoma G2 Stage IB1
TCGA-MY-A5BD 1 WHITE Alive 904 [Not Applicable] 62 Cervical Squamous Cell Carcinoma G1 Stage IIB
TCGA-MY-A5BE 1 BLACK OR AFRICAN AMERICAN Alive 2 [Not Applicable] 42 Cervical Squamous Cell Carcinoma G3 Stage IB1
TCGA-MY-A5BF 1 WHITE Alive 132 [Not Applicable] 68 Cervical Squamous Cell Carcinoma G1 Stage IIA2
TCGA-MY-A913 1 BLACK OR AFRICAN AMERICAN Alive 167 [Not Applicable] 28 Cervical Squamous Cell Carcinoma G3 Stage IIA
TCGA-PN-A8MA 1 BLACK OR AFRICAN AMERICAN Alive 90 [Not Applicable] 43 Cervical Squamous Cell Carcinoma G3 Stage IIB
TCGA-Q1-A5R1 1 WHITE Alive 113 [Not Applicable] 32 Mucinous Adenocarcinoma of Endocervical Type G2 Stage IB1
TCGA-Q1-A5R2 1 WHITE Alive 105 [Not Applicable] 64 Cervical Squamous Cell Carcinoma G3 [Not Available]
TCGA-Q1-A5R3 1 WHITE Alive 178 [Not Applicable] 56 Cervical Squamous Cell Carcinoma G2 [Not Available]
TCGA-Q1-A6DT 1 WHITE Alive 103 [Not Applicable] 55 Cervical Squamous Cell Carcinoma GX Stage I
TCGA-Q1-A6DV 1 ASIAN Alive 113 [Not Applicable] 36 Mucinous Adenocarcinoma of Endocervical Type G2 Stage IB1
TCGA-Q1-A6DW 1 WHITE Alive 175 [Not Applicable] 44 Cervical Squamous Cell Carcinoma GX Stage I
TCGA-Q1-A73O 1 [Not Evaluated] Alive 146 [Not Applicable] 37 Cervical Squamous Cell Carcinoma GX Stage IB2
TCGA-Q1-A73P 1 WHITE Alive 146 [Not Applicable] 45 Endocervical Type of Adenocarcinoma G1 Stage IB1
TCGA-Q1-A73Q 1 WHITE Alive 179 [Not Applicable] 46 Cervical Squamous Cell Carcinoma GX Stage I
TCGA-Q1-A73R 1 WHITE Alive 190 [Not Applicable] 45 Endocervical Type of Adenocarcinoma GX Stage I
TCGA-Q1-A73S 1 WHITE Alive 316 [Not Applicable] 33 Adenosquamous G2 Stage IB1
TCGA-R2-A69V 1 WHITE Alive 596 [Not Applicable] 42 Cervical Squamous Cell Carcinoma G3 Stage IB
TCGA-RA-A741 1 WHITE Alive 118 [Not Applicable] 34 Cervical Squamous Cell Carcinoma GX Stage IIB
TCGA-UC-A7PD 1 AMERICAN INDIAN OR ALASKA NATIVE Dead [Not Available] 355 21 Cervical Squamous Cell Carcinoma G2 Stage IB
TCGA-UC-A7PF 1 [Not Evaluated] Dead [Not Available] 2859 44 Cervical Squamous Cell Carcinoma G2 Stage IB1
TCGA-UC-A7PG 1 WHITE Dead [Not Available] 370 44 Cervical Squamous Cell Carcinoma G1 Stage IIIB
TCGA-UC-A7PI 1 WHITE Alive 1905 [Not Applicable] 44 Endometrioid Adenocarcinoma of Endocervix G1 Stage IB1
TCGA-VS-A8EB 1 WHITE Dead [Not Available] 305 41 Cervical Squamous Cell Carcinoma GX Stage IIIB
TCGA-VS-A8EC 1 WHITE Alive 1050 [Not Applicable] 55 Cervical Squamous Cell Carcinoma G2 Stage IIIB
TCGA-VS-A8EG 1 [Unknown] Alive 883 [Not Applicable] 36 Cervical Squamous Cell Carcinoma GX Stage IB1
TCGA-VS-A8EH 1 [Unknown] Alive 615 [Not Applicable] 56 Cervical Squamous Cell Carcinoma G2 Stage IIIB
TCGA-VS-A8EI 1 WHITE Alive 428 [Not Applicable] 38 Cervical Squamous Cell Carcinoma GX Stage IIB
TCGA-VS-A8EJ 0 WHITE Dead [Not Available] 607 60 Cervical Squamous Cell Carcinoma G3 Stage IIB
TCGA-VS-A8EK 1 WHITE Dead [Not Available] 829 65 Cervical Squamous Cell Carcinoma G2 Stage IVA
TCGA-VS-A8EL 1 BLACK OR AFRICAN AMERICAN Alive 1628 [Not Applicable] 38 Cervical Squamous Cell Carcinoma G3 Stage IIB
TCGA-VS-A8Q8 1 WHITE Dead [Not Available] 978 26 Cervical Squamous Cell Carcinoma G2 Stage IB
TCGA-VS-A8Q9 1 WHITE Alive 1400 [Not Applicable] 79 Cervical Squamous Cell Carcinoma G2 Stage IB1
TCGA-VS-A8QA 1 [Unknown] Alive 708 [Not Applicable] 44 Cervical Squamous Cell Carcinoma GX Stage IB1
TCGA-VS-A8QC 1 WHITE Dead [Not Available] 350 51 Cervical Squamous Cell Carcinoma G2 Stage IVA
TCGA-VS-A8QF 1 WHITE Alive 1507 [Not Applicable] 42 Cervical Squamous Cell Carcinoma G2 Stage IIB
TCGA-VS-A8QH 0 WHITE Dead [Not Available] 1210 76 Mucinous Adenocarcinoma of Endocervical Type G2 Stage IB1
TCGA-VS-A8QM 1 [Unknown] Dead [Not Available] 951 47 Cervical Squamous Cell Carcinoma GX Stage IVB
TCGA-VS-A94W 1 WHITE Alive 879 [Not Applicable] 39 Cervical Squamous Cell Carcinoma G2 Stage IIB
TCGA-VS-A94X 1 BLACK OR AFRICAN AMERICAN Dead [Not Available] 506 40 Cervical Squamous Cell Carcinoma G2 Stage IIB
TCGA-VS-A94Y 1 WHITE Dead [Not Available] 144 47 Cervical Squamous Cell Carcinoma GX Stage IIB
TCGA-VS-A94Z 1 WHITE Alive 707 [Not Applicable] 38 Cervical Squamous Cell Carcinoma G2 Stage IIB
TCGA-VS-A950 1 [Unknown] Alive 967 [Not Applicable] 42 Cervical Squamous Cell Carcinoma G3 Stage IIIA
TCGA-VS-A952 1 WHITE Alive 1365 [Not Applicable] 66 Mucinous Adenocarcinoma of Endocervical Type G2 Stage IB1
TCGA-VS-A953 1 [Unknown] Dead [Not Available] 477 63 Cervical Squamous Cell Carcinoma GX Stage IVA
TCGA-VS-A954 1 [Unknown] Alive 1295 [Not Applicable] 67 Cervical Squamous Cell Carcinoma G2 Stage IIIB
TCGA-VS-A957 1 WHITE Alive 1280 [Not Applicable] 64 Cervical Squamous Cell Carcinoma G3 Stage IB1
TCGA-VS-A958 1 WHITE Alive 1333 [Not Applicable] 46 Cervical Squamous Cell Carcinoma G2 Stage IIB
TCGA-VS-A959 1 [Unknown] Alive 1197 [Not Applicable] 76 Cervical Squamous Cell Carcinoma G3 Stage IIB
TCGA-VS-A9U5 1 WHITE Alive 1185 [Not Applicable] 57 Cervical Squamous Cell Carcinoma G3 Stage IIB
TCGA-VS-A9U6 1 WHITE Alive 1213 [Not Applicable] 52 Cervical Squamous Cell Carcinoma GX Stage IVB
TCGA-VS-A9U7 1 WHITE Alive 1108 [Not Applicable] 30 Cervical Squamous Cell Carcinoma G3 Stage IB1
TCGA-VS-A9UB 1 [Unknown] Alive 562 [Not Applicable] 54 Cervical Squamous Cell Carcinoma G3 Stage IIB
TCGA-VS-A9UC 1 [Unknown] Alive 511 [Not Applicable] 32 Cervical Squamous Cell Carcinoma G2 Stage IIB
TCGA-VS-A9UD 1 WHITE Alive 417 [Not Applicable] 73 Cervical Squamous Cell Carcinoma G2 Stage IIIA
TCGA-VS-A9UH 1 BLACK OR AFRICAN AMERICAN Alive 1120 [Not Applicable] 53 Cervical Squamous Cell Carcinoma GX Stage IVA
TCGA-VS-A9UI 1 WHITE Alive 953 [Not Applicable] 76 Cervical Squamous Cell Carcinoma GX Stage IIB
TCGA-VS-A9UJ 0 [Unknown] Dead [Not Available] 52 55 Cervical Squamous Cell Carcinoma GX Stage IIB
TCGA-VS-A9UL 1 WHITE Dead [Not Available] 442 79 Cervical Squamous Cell Carcinoma G3 Stage IIIB
TCGA-VS-A9UM 1 BLACK OR AFRICAN AMERICAN Dead [Not Available] 829 39 Cervical Squamous Cell Carcinoma G2 Stage IVB
TCGA-VS-A9UO 1 WHITE Alive 1080 [Not Applicable] 43 Mucinous Adenocarcinoma of Endocervical Type G2 Stage IIB
TCGA-VS-A9UP 1 [Unknown] Alive 1150 [Not Applicable] 43 Mucinous Adenocarcinoma of Endocervical Type G3 Stage IIA
TCGA-VS-A9UQ 1 [Unknown] Alive 920 [Not Applicable] 32 Mucinous Adenocarcinoma of Endocervical Type G2 Stage IB
TCGA-VS-A9UR 1 WHITE Alive 455 [Not Applicable] 53 Mucinous Adenocarcinoma of Endocervical Type GX Stage IIA
TCGA-VS-A9UT 0 ASIAN Alive 104 [Not Applicable] 72 Mucinous Adenocarcinoma of Endocervical Type G3 Stage IB
TCGA-VS-A9UU 1 [Unknown] Alive 442 [Not Applicable] 42 Cervical Squamous Cell Carcinoma G1 Stage IIB
TCGA-VS-A9UV 1 [Unknown] Dead [Not Available] 104 74 Cervical Squamous Cell Carcinoma GX Stage IVA
TCGA-VS-A9UY 1 [Unknown] Dead [Not Available] 555 29 Cervical Squamous Cell Carcinoma G2 Stage IVB
TCGA-VS-A9UZ 1 WHITE Alive 1939 [Not Applicable] 61 Mucinous Adenocarcinoma of Endocervical Type G2 Stage IB1
TCGA-VS-A9V0 0 [Unknown] Alive 529 [Not Applicable] 58 Mucinous Adenocarcinoma of Endocervical Type G3 Stage IB
TCGA-VS-A9V1 1 WHITE Dead [Not Available] 157 46 Mucinous Adenocarcinoma of Endocervical Type G2 Stage IVB
TCGA-VS-A9V2 1 WHITE Alive 237 [Not Applicable] 29 Cervical Squamous Cell Carcinoma G2 Stage IB1
TCGA-VS-A9V3 1 WHITE Alive 253 [Not Applicable] 62 Cervical Squamous Cell Carcinoma G3 Stage IVB
TCGA-VS-A9V4 0 WHITE Dead [Not Available] 132 63 Mucinous Adenocarcinoma of Endocervical Type G2 Stage IVA
TCGA-VS-A9V5 1 WHITE Dead [Not Available] 494 50 Mucinous Adenocarcinoma of Endocervical Type G2 Stage IIB
TCGA-VS-AA62 1 WHITE Dead [Not Available] 469 51 Cervical Squamous Cell Carcinoma G2 Stage IIB
TCGA-WL-A834 1 WHITE Alive 549 [Not Applicable] 57 Cervical Squamous Cell Carcinoma G3 [Not Available]
TCGA-XS-A8TJ 1 BLACK OR AFRICAN AMERICAN Alive 890 [Not Applicable] 41 Cervical Squamous Cell Carcinoma G2 Stage IB1
TCGA-ZJ-A8QO 1 [Unknown] Alive 0 [Not Applicable] 73 Cervical Squamous Cell Carcinoma [Not Available] [Not Available]
TCGA-ZJ-A8QQ 1 WHITE Alive 2056 [Not Applicable] 24 Cervical Squamous Cell Carcinoma GX Stage IIB
TCGA-ZJ-A8QR 1 NATIVE HAWAIIAN OR OTHER PACIFIC ISLANDER Dead [Not Available] 582 38 Cervical Squamous Cell Carcinoma GX Stage I
TCGA-ZJ-AAX4 1 WHITE Alive 21 [Not Applicable] 85 Cervical Squamous Cell Carcinoma G3 Stage II
TCGA-ZJ-AAX8 1 [Unknown] Alive 0 [Not Applicable] 58 Cervical Squamous Cell Carcinoma G2 Stage IIIB
TCGA-ZJ-AAXA 1 WHITE Alive 43 [Not Applicable] 64 Cervical Squamous Cell Carcinoma G2 Stage IB1
TCGA-ZJ-AAXB 1 WHITE Alive 0 [Not Applicable] 42 Endocervical Adenocarcinoma of the Usual Type G3 Stage IB2
TCGA-ZJ-AAXD 1 [Unknown] Alive 0 [Not Applicable] 35 Cervical Squamous Cell Carcinoma G2 Stage IIIB
TCGA-ZJ-AAXF 1 [Unknown] Alive 0 [Not Applicable] 62 Cervical Squamous Cell Carcinoma G3 Stage IIB
TCGA-ZJ-AAXI 1 [Unknown] Alive 0 [Not Applicable] 67 Cervical Squamous Cell Carcinoma G2 Stage IIB
TCGA-ZJ-AAXJ 1 [Unknown] Alive 0 [Not Applicable] 43 Cervical Squamous Cell Carcinoma [Not Available] Stage IIB
TCGA-ZJ-AAXN 1 WHITE Alive 0 [Not Applicable] 34 Cervical Squamous Cell Carcinoma [Not Available] Stage IB2
TCGA-ZJ-AAXT 1 WHITE Alive 0 [Not Applicable] 54 Cervical Squamous Cell Carcinoma G2 Stage IIIB
TCGA-ZJ-AAXU 1 WHITE Alive 5 [Not Applicable] 51 Cervical Squamous Cell Carcinoma G2 Stage IIB
TCGA-ZJ-AB0H 1 [Unknown] Alive 0 [Not Applicable] 48 Cervical Squamous Cell Carcinoma [Not Available] Stage IIIB
TCGA-ZJ-AB0I 1 WHITE Alive 0 [Not Applicable] 25 Cervical Squamous Cell Carcinoma [Not Available] Stage IIB
TCGA-ZX-AA5X 1 WHITE Alive 119 [Not Applicable] 64 Cervical Squamous Cell Carcinoma G2 Stage IIIB
