# Supplementary material for: Identification and characterization of HPV-independent cervical cancers
Source: Oncotarget. 2017 Jan 6;8(8):13375–86. doi: 10.18632/oncotarget.14533 (PMC5355105; doi:10.18632/oncotarget.14533)
Supplement: Supplementary file 7 [file oncotarget-08-13375-s007.docx]

Supplemental table 4: RNAseq data ANOVA Column # Gene Symbol Significant and 4x HPV-inactive (mean) HPV-active (mean) p-value Ratio of the Means (HPV-inactive vs. HPV-active) FoldChange (HPV-inactive vs. HPV-active) t-statistic (HPV-inactive vs. HPV-active)11 A1CF yes 38.3413 7.58247 0.00431557 5.05658 5.05658 2.8795325 AADACL2 yes 68.7683 8.08538 0.000159465 8.50526 8.50526 3.8321928 AADAT yes 410.965 95.6937 3.60E-08 4.29459 4.29459 5.6803836 AARD yes 15.571 3.20654 0.000396512 4.85602 4.85602 3.5892752 ABCA17P yes 26.2345 122.339 0.000507049 0.214441 -4.66329 -3.5214463 ABCB10P1 yes 72.9017 2.1541 1.31E-05 33.8432 33.8432 4.4445664 ABCB10P3 yes 2.5101 0.0942279 7.62E-05 26.6386 26.6386 4.0207165 ABCB10P4 yes 7.11071 0.19384 2.80E-05 36.6833 36.6833 4.2652176 ABCC12 yes 1.41558 0.345874 2.06E-05 4.09277 4.09277 4.3386686 ABCC8 yes 33.0889 4.13789 5.75E-08 7.99657 7.99657 5.58999111 ABHD12B yes 668.863 70.9656 2.90E-07 9.42517 9.42517 5.26845240 ACSS3 yes 533.446 25.5956 1.29E-15 20.8413 20.8413 8.5247255 ACTBP6 yes 0.107173 0.00329159 6.57E-06 32.5597 32.5597 4.60154256 ACTBP7 yes 0.0377851 0.00165776 0.000252305 22.7929 22.7929 3.7114264 ACTG1P12 yes 0.0459263 0.00764734 0.0113969 6.00552 6.00552 2.54849266 ACTG1P3 yes 0.0372131 0.00166521 0.000174937 22.3474 22.3474 3.80806333 ADAM2 yes 91.9443 1.42501 0.000243481 64.5219 64.5219 3.72088337 ADAM20P3 yes 0.0797845 0.00715805 1.77E-05 11.1461 11.1461 4.37428350 ADAM5 yes 3.39984 0.388419 5.99E-09 8.75301 8.75301 6.01851362 ADAMTS16 yes 144.773 31.8988 0.000234407 4.53849 4.53849 3.73098364 ADAMTS18 yes 201.786 23.1484 0.000146883 8.71706 8.71706 3.85351365 ADAMTS19 yes 334.838 5.23395 4.07E-14 63.9742 63.9742 8.00396373 ADAMTS8 yes 267.813 12.6591 0.000416124 21.1557 21.1557 3.57603374 ADAMTS9 yes 785.496 160.776 1.03E-05 4.88566 4.88566 4.49996405 ADCY8 yes 5.5931 1.21547 0.000118659 4.60158 4.60158 3.90844408 ADCYAP1R1 yes 168.94 12.2131 3.48E-10 13.8327 13.8327 6.52856412 ADD3-AS1 yes 32.7069 7.56142 2.64E-09 4.32549 4.32549 6.16851453 ADRA1A yes 26.6965 2.02605 0.000846482 13.1766 13.1766 3.37664458 ADRA2C yes 618.505 132.782 1.19E-10 4.65806 4.65806 6.71468460 ADRB2 yes 35.834 410.783 0.000242765 0.0872334 -11.4635 -3.72167481 AFF3 yes 220.595 36.0804 4.35E-07 6.11399 6.11399 5.18507541 AGT yes 312.401 69.2739 0.00483416 4.50966 4.50966 2.8424544 AGTR2 yes 29.7995 0.778187 2.31E-06 38.2935 38.2935 4.83255555 AHCYP3 yes 0.0761695 0.00880038 0.00164435 8.65525 8.65525 3.18139581 AIM2 yes 91.3945 662.17 0.00277597 0.138023 -7.24519 -3.0205588 AIRE yes 8.39929 0.952218 3.77E-06 8.82076 8.82076 4.72519604 AK4P2 yes 0.144607 0.015692 0.00124504 9.21533 9.21533 3.26431624 AKAP4 yes 7.9157 0.0422476 0.000409492 187.365 187.365 3.58044639 AKR1B10P1 yes 2.93309 0.371296 7.57E-05 7.8996 7.8996 4.02209648 AKR1B1P7 yes 0.107734 0.01658 0.00114509 6.49784 6.49784 3.28893656 AKR1C7P yes 31.5519 2.67429 0.000695723 11.7982 11.7982 3.43263759 ALPL yes 2060.67 334.638 1.50E-08 6.15789 6.15789 5.84692761 ALPPL2 yes 6085.28 112.72 0.00079604 53.9856 53.9856 3.39426771 AMBN yes 1350.91 0.29897 1.58E-05 4518.54 4518.54 4.40053780 AMELX yes 20.9188 0.045915 0.000278132 455.598 455.598 3.68534783 AMER2 yes 2.94903 0.725719 5.22E-05 4.0636 4.0636 4.11433784 AMER3 yes 1.82286 0.312092 4.76E-06 5.84077 5.84077 4.67355834 ANGPTL1 yes 28.1572 4.48154 7.05E-06 6.28294 6.28294 4.58569872 ANKRD18B yes 41.9492 213.561 0.00121346 0.196427 -5.09095 -3.27188879 ANKRD20A10P yes 0.28933 1.16008 0.000584788 0.249405 -4.00954 -3.48162884 ANKRD20A15P yes 2.64168 0.583722 9.04E-06 4.52558 4.52558 4.52921886 ANKRD20A17P yes 4.40799 0.776284 6.13E-06 5.67832 5.67832 4.61725903 ANKRD26P2 yes 1.43166 0.189535 9.44E-12 7.55356 7.55356 7.13938917 ANKRD33 yes 7.48439 0.529488 0.000133152 14.1351 14.1351 3.87885948 ANKRD62P1-PARP4P3 yes 8.84732 1.20993 6.55E-07 7.31224 7.31224 5.10049952 ANKRD7 yes 16.6381 0.981415 2.94E-10 16.9532 16.9532 6.55841963 ANO1-AS2 yes 7.69114 0.889729 0.0024958 8.64436 8.64436 3.05373967 ANO4 yes 99.3821 23.8979 4.23E-06 4.15861 4.15861 4.69991996 ANXA2R yes 35.2127 141.721 0.000543163 0.248465 -4.02471 -3.502281002 ANXA8 yes 136.332 1253.97 7.17E-06 0.108721 -9.19789 -4.581991003 ANXA8L1 yes 436.034 2132.77 0.000260237 0.204445 -4.8913 -3.703141004 ANXA8L2 yes 1063.19 5496.93 7.23E-06 0.193415 -5.17023 -4.58011011 AOC4P yes 7.18537 0.394931 0.000166138 18.194 18.194 3.821521056 APCDD1 yes 4627 851.919 1.95E-12 5.43127 5.43127 7.396171102 APOF yes 2.39451 0.472888 1.12E-05 5.0636 5.0636 4.480711132 AQP3 yes 2101.81 18351.8 0.000908667 0.114529 -8.73142 -3.356231133 AQP4 yes 40.2896 7.73053 6.64E-06 5.21176 5.21176 4.599321142 AQP8 yes 14.5251 1.08711 6.08E-07 13.3612 13.3612 5.115981161 ARF1P3 yes 0.0550681 0.0016678 6.60E-07 33.0183 33.0183 5.099041253 ARHGEF38-IT1 yes 0.03817 0.00440474 0.00371425 8.66566 8.66566 2.928061298 ARL2BPP8 yes 0.0842548 0.00611378 0.00355067 13.7811 13.7811 2.942511345 ARNT2 yes 1257.49 288.284 3.42E-12 4.36201 4.36201 7.305361376 ARSE yes 335.945 75.2779 0.00227127 4.46274 4.46274 3.082931393 ARX yes 416.823 5.28155 2.07E-08 78.9207 78.9207 5.786441405 ASB10 yes 1.7261 0.299262 0.000904582 5.76785 5.76785 3.357531431 ASCL5 yes 85.1782 3.2335 6.40E-08 26.3424 26.3424 5.569051441 ASIC2 yes 233.255 22.8661 5.47E-18 10.2009 10.2009 9.315631444 ASIC5 yes 7.94232 1.26131 1.16E-06 6.29687 6.29687 4.980341457 ASNSP1 yes 8.03229 0.357493 1.22E-07 22.4684 22.4684 5.441971458 ASNSP2 yes 0.0838132 0.0154875 0.00621506 5.41166 5.41166 2.75881460 ASNSP4 yes 0.435228 0.104036 5.41E-05 4.18342 4.18342 4.105781485 ASS1P7 yes 0.113605 0.0201883 0.00810194 5.62728 5.62728 2.668421489 ASTL yes 21.9785 3.5058 9.62E-10 6.26918 6.26918 6.349591490 ASTN1 yes 65.3129 6.23823 1.07E-17 10.4698 10.4698 9.220421550 ATOH1 yes 13.3958 0.639308 3.72E-05 20.9535 20.9535 4.197151569 ATP1A2 yes 180.403 9.00756 4.55E-08 20.028 20.028 5.635511574 ATP1B2 yes 112.302 16.4416 5.33E-07 6.83033 6.83033 5.143391582 ATP2B2 yes 86.8645 14.1495 8.79E-06 6.13905 6.13905 4.535611610 ATP5F1P2 yes 0.650418 0.0105021 0.000310757 61.9319 61.9319 3.655511697 ATP6V1G3 yes 0.642849 0.144745 7.60E-06 4.44125 4.44125 4.568661744 AURKBPS1 yes 0.752054 0.0671272 3.73E-05 11.2034 11.2034 4.196061750 AVP yes 0.694023 0.163651 0.000801524 4.24088 4.24088 3.392291759 AXIN2 yes 2451.32 132.507 7.76E-16 18.4996 18.4996 8.599581763 AZGP1P2 yes 0.116725 0.0259691 0.00033202 4.49477 4.49477 3.637611767 AZU1 yes 12.3119 1.26461 0.000419824 9.73577 9.73577 3.57361827 BAI3 yes 33.0847 5.98854 1.71E-09 5.52468 5.52468 6.246761836 BAMBI yes 1917.48 230.487 8.81E-12 8.31928 8.31928 7.15081837 BANCR yes 7.37607 1.21488 0.000197712 6.07142 6.07142 3.775971889 BCAT1 yes 3166.39 402.966 3.38E-09 7.85772 7.85772 6.12361904 BCL2L10 yes 29.9768 150.865 0.00771971 0.1987 -5.03271 -2.685071930 BCRP4 yes 0.203004 0.0277901 1.44E-08 7.3049 7.3049 5.855561938 BCYRN1P3 yes 0.27104 0.0643259 0.000651263 4.21354 4.21354 3.451321963 BEST3 yes 282.766 6.67543 2.30E-08 42.3592 42.3592 5.76621967 BEX1 yes 126.97 7.41955 1.39E-07 17.1129 17.1129 5.416231970 BEX5 yes 142.147 21.5856 1.01E-09 6.58529 6.58529 6.340711977 BHLHA9 yes 0.0595047 0.0102819 0.00614948 5.78732 5.78732 2.762371985 BHMT2 yes 241.985 39.4537 0.000197729 6.13339 6.13339 3.775952032 BMP2KL yes 0.063835 0.00710049 0.000618464 8.99022 8.99022 3.465892034 BMP4 yes 1360.64 130.685 2.02E-10 10.4116 10.4116 6.623522035 BMP5 yes 153.371 4.91862 1.19E-08 31.1816 31.1816 5.891292056 BNC1 yes 240.329 1226.62 3.91E-05 0.195928 -5.10391 -4.18492063 BNIP3P2 yes 0.0406172 0.00314473 0.00115044 12.916 12.916 3.287562065 BNIP3P4 yes 8.13077 1.69202 0.000748613 4.80535 4.80535 3.41182126 BRDT yes 80.3951 3.24147 3.16E-06 24.8021 24.8021 4.764572139 BRINP2 yes 57.8195 3.25976 1.51E-14 17.7373 17.7373 8.15522150 BRSK1 yes 263.535 62.2069 0.000311396 4.23642 4.23642 3.654952160 BSN-AS2 yes 6.81642 1.60863 5.17E-12 4.23742 4.23742 7.237962166 BSX yes 4.93463 0.0850101 3.23E-06 58.0475 58.0475 4.759192172 BTBD17 yes 30.3813 0.948284 3.60E-08 32.0382 32.0382 5.680572195 BTF3P13 yes 0.253337 0.0259868 2.54E-05 9.74868 9.74868 4.288792222 BTNL2 yes 3.81865 0.942977 1.89E-07 4.04957 4.04957 5.354862252 C10orf126 yes 119.231 0.326696 0.000149975 364.959 364.959 3.848122262 C10orf40 yes 0.438142 0.0792032 0.00633835 5.53186 5.53186 2.752182272 C10orf82 yes 61.4959 1.49993 1.03E-15 40.9993 40.9993 8.558372281 C11orf21 yes 87.0737 21.1042 2.15E-05 4.12589 4.12589 4.328572309 C11orf85 yes 8.37563 50.3269 0.00237539 0.166424 -6.00873 -3.069072311 C11orf87 yes 19.9476 2.66916 1.49E-05 7.47337 7.47337 4.414752323 C12orf39 yes 9.40865 0.735253 1.01E-07 12.7965 12.7965 5.479142334 C12orf54 yes 22.5445 98.8029 0.00934376 0.228177 -4.38257 -2.618812380 C15orf32 yes 1.41223 0.243365 2.64E-07 5.80292 5.80292 5.287412394 C15orf59 yes 165.419 36.966 0.00337021 4.47488 4.47488 2.959172419 C16orf89 yes 255.313 40.0717 3.49E-05 6.3714 6.3714 4.212222444 C17orf64 yes 22.2259 3.8098 0.00191214 5.83389 5.83389 3.13572524 C1orf127 yes 68.0573 8.26792 1.65E-07 8.23149 8.23149 5.38242528 C1orf141 yes 3.78139 0.399427 8.32E-05 9.46702 9.46702 3.998512549 C1orf195 yes 0.0947149 0.0206311 0.00347762 4.59087 4.59087 2.949162575 C1orf64 yes 32.1917 6.99561 0.00853378 4.60169 4.60169 2.650432581 C1orf87 yes 3.36207 0.796277 3.64E-07 4.22224 4.22224 5.222082583 C1orf95 yes 133.639 8.9393 4.75E-13 14.9497 14.9497 7.621182595 C1QTNF2 yes 11.9115 54.2881 0.000932745 0.219413 -4.55763 -3.348672596 C1QTNF3 yes 855.836 198.902 0.0027893 4.30281 4.30281 3.0192637 C21orf37 yes 2.58793 0.635411 2.19E-06 4.07284 4.07284 4.844572672 C2orf40 yes 49.396 9.03678 5.41E-05 5.46611 5.46611 4.105452695 C2orf80 yes 6.76966 0.203173 2.00E-06 33.3197 33.3197 4.863652718 C3orf55 yes 195.631 8.98949 4.13E-06 21.7621 21.7621 4.705182776 C5orf52 yes 4.84563 1.05084 5.54E-05 4.61122 4.61122 4.099622796 C6orf15 yes 2991.17 52.8471 2.81E-05 56.6004 56.6004 4.264512852 C8orf34 yes 73.6324 11.9548 1.69E-08 6.15925 6.15925 5.825242868 C8orf86 yes 2.647 0.517888 1.08E-05 5.11114 5.11114 4.488432879 C9orf135-AS1 yes 0.0926494 0.0098642 0.000740569 9.39249 9.39249 3.414882887 C9orf169 yes 55.3221 251.537 0.00540108 0.219936 -4.54677 -2.805742891 C9orf173 yes 7.31264 1.42336 9.14E-09 5.13761 5.13761 5.939982920 CA14 yes 101.38 19.1462 3.59E-10 5.29506 5.29506 6.523282924 CA4 yes 22.3916 4.69528 0.000928375 4.76895 4.76895 3.350032925 CA5A yes 19.4324 4.60645 1.04E-08 4.21852 4.21852 5.916592930 CA7 yes 14.4763 1.24975 4.08E-06 11.5834 11.5834 4.708052931 CA8 yes 758.048 40.2926 1.38E-14 18.8136 18.8136 8.168622939 CABP1 yes 41.7516 8.74236 9.76E-10 4.77578 4.77578 6.347062940 CABP2 yes 0.310372 0.0630979 0.00162508 4.91889 4.91889 3.184942943 CABP7 yes 29.499 4.62724 4.85E-08 6.37507 6.37507 5.623142944 CABS1 yes 0.257474 0.0407959 0.000313673 6.31127 6.31127 3.652992956 CACNA1E yes 216.105 11.1523 5.13E-07 19.3776 19.3776 5.151412958 CACNA1G yes 146.641 27.5083 0.000894456 5.33079 5.33079 3.360772962 CACNA1S yes 27.0222 0.79097 4.43E-09 34.1633 34.1633 6.073922964 CACNA2D2 yes 281.607 62.9955 2.40E-12 4.47027 4.47027 7.362312976 CACNG5 yes 4.15727 0.697241 3.37E-06 5.96245 5.96245 4.750312987 CADM1 yes 1785.37 231.515 2.53E-22 7.71171 7.71171 10.68662988 CADM2 yes 42.8857 10.5667 2.19E-06 4.05857 4.05857 4.844473000 CALCB yes 43.7921 0.634655 6.55E-08 69.0014 69.0014 5.564493012 CALM1P2 yes 0.193241 0.0350869 0.00296101 5.5075 5.5075 3.000213023 CALN1 yes 106.287 17.458 9.65E-09 6.08816 6.08816 5.930013042 CAMKV yes 16.5938 2.57545 0.00450498 6.44307 6.44307 2.865523102 CARM1P1 yes 5.11565 1.10704 0.00300263 4.62104 4.62104 2.99583106 CARTPT yes 7.89599 0.387585 4.87E-05 20.3723 20.3723 4.131353169 CBLN4 yes 25.1752 0.735085 8.68E-05 34.2481 34.2481 3.987723195 CBX3P6 yes 0.0563719 0.0029221 8.39E-05 19.2915 19.2915 3.99633244 CCDC129 yes 43.4127 10.0917 0.000801397 4.30182 4.30182 3.392343388 CCKAR yes 6.49476 0.10993 0.000447508 59.0808 59.0808 3.556023389 CCKBR yes 54.7986 5.95083 0.00036521 9.20856 9.20856 3.611733397 CCL17 yes 11.211 56.6355 0.002321 0.19795 -5.05177 -3.076243405 CCL24 yes 63.1958 13.843 7.31E-05 4.56517 4.56517 4.031083422 CCNA1 yes 538.549 92.0659 0.00144518 5.84961 5.84961 3.220093460 CCNYL2 yes 15.6198 1.85936 0.000203007 8.40059 8.40059 3.769023508 CD109 yes 367.118 1833.64 1.18E-05 0.200213 -4.99468 -4.468083628 CDC27P1 yes 4.53637 0.916358 0.00030325 4.95043 4.95043 3.66213629 CDC27P2 yes 0.637221 0.0857472 0.000228154 7.43139 7.43139 3.738153668 CDH10 yes 13.2341 1.64469 0.00150187 8.04652 8.04652 3.208593678 CDH17 yes 1449.97 140.282 0.000157981 10.3361 10.3361 3.834623681 CDH2 yes 685.604 131.001 2.62E-05 5.23359 5.23359 4.281613749 CDKN2AIPNLP3 yes 0.0213091 0.00137032 0.0103953 15.5504 15.5504 2.581243751 CDKN2B-AS1 yes 166.717 716.072 9.51E-06 0.232822 -4.29514 -4.517763756 CDO1 yes 81.331 7.84913 4.72E-09 10.3618 10.3618 6.062323764 CDRT15L2 yes 0.18732 0.007538 9.34E-05 24.8501 24.8501 3.969233776 CDX1 yes 404.233 19.1112 2.48E-06 21.1517 21.1517 4.817163777 CDX2 yes 892.879 38.5593 1.78E-06 23.156 23.156 4.888713810 CEACAM20 yes 41.2681 2.64788 0.000236355 15.5853 15.5853 3.728783825 CEACAMP5 yes 0.937385 10.0151 0.00666468 0.0935971 -10.6841 -2.735213839 CECR3 yes 2.21593 0.181314 6.66E-10 12.2215 12.2215 6.414723854 CELF3 yes 24.1701 3.16668 1.97E-13 7.63262 7.63262 7.759693856 CELF5 yes 34.1791 3.74535 6.08E-17 9.12574 9.12574 8.971143865 CEND1P2 yes 0.13474 0.00451821 0.000148771 29.8214 29.8214 3.850213870 CENPCP1 yes 0.0316831 0.00140433 0.000194656 22.5611 22.5611 3.780073886 CENPVP1 yes 2.75753 0.0494015 3.13E-06 55.8187 55.8187 4.766323887 CENPVP2 yes 1.19936 0.0470962 7.58E-08 25.4662 25.4662 5.536143888 CENPVP3 yes 1.57283 0.0126386 2.03E-05 124.446 124.446 4.342113928 CERS1 yes 118.829 18.3951 8.38E-07 6.45979 6.45979 5.049093941 CES5A yes 8.52361 1.41082 0.00368293 6.04161 6.04161 2.930783949 CFC1 yes 1.55293 0.201624 0.000730291 7.70209 7.70209 3.418853950 CFC1B yes 0.385001 0.0953451 0.00473216 4.03797 4.03797 2.84943957 CFHR4 yes 36.9008 7.922 0.00280343 4.65801 4.65801 3.017413968 CFL1P8 yes 0.0601318 0.0101039 0.00665817 5.95135 5.95135 2.735543980 CGB7 yes 12.114 48.6559 0.000689355 0.248974 -4.01649 -3.435244039 CHGA yes 1047.68 101.927 0.000751679 10.2787 10.2787 3.410634040 CHGB yes 439.061 14.7475 8.38E-07 29.7719 29.7719 5.049064043 CHIA yes 10.4542 0.963431 4.88E-05 10.851 10.851 4.130934058 CHMP1AP1 yes 0.126382 0.00166762 0.000879934 75.786 75.786 3.365494093 CHRM5 yes 19.5153 3.78751 2.28E-08 5.15254 5.15254 5.76844120 CHST5 yes 90.2674 7.38461 0.00025638 12.2237 12.2237 3.707134189 CITED1 yes 110.094 15.059 0.00318593 7.31087 7.31087 2.977054201 CKM yes 94.2253 12.637 1.46E-05 7.45631 7.45631 4.419094243 CLDN16 yes 537.33 61.6746 0.000158863 8.71235 8.71235 3.833174246 CLDN19 yes 64.2129 5.89172 0.000845932 10.8988 10.8988 3.376834256 CLDN6 yes 828.29 12.5844 1.97E-07 65.8186 65.8186 5.346784276 CLEC2B yes 237.62 1609.7 0.00182048 0.147618 -6.77426 -3.150634301 CLIC5 yes 701.476 158.233 2.69E-06 4.43318 4.43318 4.799754340 CLSTN2 yes 182.83 39.7694 1.48E-08 4.59725 4.59725 5.849484356 CLVS2 yes 1.10827 0.225407 0.00124825 4.91674 4.91674 3.263554417 CNN3P1 yes 0.0421275 0.00277248 0.00568633 15.1949 15.1949 2.78864438 CNPY1 yes 45.4796 2.10239 0.000517905 21.6323 21.6323 3.515554454 CNTN3 yes 76.9809 12.4189 3.48E-09 6.19868 6.19868 6.117854490 COL11A2 yes 492.571 53.73 4.12E-11 9.16752 9.16752 6.894214509 COL25A1 yes 74.5215 17.1747 0.00287075 4.33903 4.33903 3.009954510 COL26A1 yes 600.446 14.0313 6.36E-15 42.7934 42.7934 8.286034513 COL2A1 yes 930.109 67.4254 0.000808661 13.7946 13.7946 3.389764532 COL6A5 yes 42.8814 5.41069 0.000263489 7.9253 7.9253 3.699824607 COX11P1 yes 0.212944 0.019927 0.000462548 10.6862 10.6862 3.54694619 COX4I1P1 yes 0.0842548 0.00382279 0.001836 22.0401 22.0401 3.148064630 COX5BP5 yes 0.0258452 0.558291 0.00414901 0.0462934 -21.6013 -2.892324635 COX6A1P1 yes 0.099979 0.0219842 0.00410813 4.54777 4.54777 2.895534668 COX8C yes 7.45279 0.0563079 0.000607905 132.358 132.358 3.470734689 CPLX2 yes 415.276 7.69954 2.96E-08 53.9352 53.9352 5.718024690 CPLX3 yes 28.9475 5.12582 0.00421829 5.64738 5.64738 2.886944693 CPN1 yes 4.69029 0.905858 0.00013409 5.17773 5.17773 3.877044698 CPNE4 yes 54.0591 12.0689 9.09E-06 4.47922 4.47922 4.5284712 CPSF1P1 yes 22.8795 4.26144 7.32E-12 5.36895 5.36895 7.181214727 CPXM2 yes 373.924 83.6318 5.72E-08 4.47107 4.47107 5.59114732 CRABP1 yes 557.446 43.5635 1.18E-08 12.7962 12.7962 5.892184738 CRB2 yes 110.287 18.393 2.11E-05 5.99617 5.99617 4.332494747 CREB3L3 yes 7.8008 1.74554 0.00208192 4.46899 4.46899 3.109714759 CRH yes 1.84834 0.0610249 1.71E-08 30.2883 30.2883 5.821994775 CRISPLD1 yes 385.675 59.8867 2.72E-12 6.44008 6.44008 7.342564807 CRYAA yes 15.272 0.851545 1.48E-07 17.9345 17.9345 5.403134811 CRYBA4 yes 12.4324 0.744812 5.97E-05 16.692 16.692 4.081144812 CRYBB1 yes 42.4997 7.95221 0.000300575 5.3444 5.3444 3.664494817 CRYGA yes 0.123587 0.0279348 0.00110377 4.4241 4.4241 3.29974818 CRYGB yes 0.401086 0.08568 0.000229151 4.68121 4.68121 3.7374821 CRYGEP yes 1.04307 0.0476538 1.45E-05 21.8886 21.8886 4.420254900 CST1 yes 3097.58 34.7738 5.64E-05 89.078 89.078 4.095254906 CST4 yes 38.0363 3.11963 6.00E-06 12.1926 12.1926 4.621914907 CST5 yes 19.0489 1.26045 7.53E-06 15.1127 15.1127 4.57094950 CTAG2 yes 116.116 10.9016 0.00297809 10.6514 10.6514 2.998394970 CTBP2P2 yes 0.359502 0.0877374 5.80E-05 4.09748 4.09748 4.088265000 CTNNA1P1 yes 4.03425 0.954153 3.24E-06 4.2281 4.2281 4.758945001 CTNNA2 yes 568.407 18.0057 2.97E-13 31.5682 31.5682 7.694985047 CTXN2 yes 2.85719 0.642841 3.71E-06 4.44463 4.44463 4.729195050 CUBNP1 yes 0.153777 0.0377374 0.000295673 4.07493 4.07493 3.668925067 CUX2 yes 57.6196 11.6985 5.29E-05 4.92537 4.92537 4.111055090 CXCL17 yes 744.475 3787.33 0.00330888 0.19657 -5.08725 -2.965025118 CXorf51A yes 1.84074 0.0395499 0.000167589 46.5421 46.5421 3.819265119 CXorf51B yes 2.29645 0.0698936 0.000516664 32.8564 32.8564 3.516225161 CYCSP17 yes 0.019358 0.00124871 0.0104966 15.5024 15.5024 2.57785198 CYCSP7 yes 0.0287222 0.0018337 0.0101625 15.6636 15.6636 2.589255200 CYCTP yes 0.0944346 0.00712815 0.000716116 13.2481 13.2481 3.424435212 CYP11B2 yes 0.129926 0.0262306 0.0102938 4.95322 4.95322 2.584715213 CYP17A1 yes 29.072 3.95704 3.25E-10 7.34692 7.34692 6.540475216 CYP1A2 yes 3.47872 0.378087 6.09E-08 9.20085 9.20085 5.578745219 CYP1D1P yes 0.395919 0.0850175 0.00164031 4.65691 4.65691 3.182135229 CYP27C1 yes 32.2946 218.661 0.0012169 0.147693 -6.77082 -3.271055235 CYP2B6 yes 176.728 22.8129 0.000992777 7.74684 7.74684 3.330595281 CYP4F30P yes 2.32512 0.383066 0.00117786 6.06976 6.06976 3.280655309 CYTL1 yes 56.8551 11.3286 2.30E-05 5.01872 5.01872 4.311875318 DACH1 yes 810.322 143.725 5.59E-16 5.63801 5.63801 8.647975319 DACH2 yes 23.9684 5.32456 1.96E-07 4.50147 4.50147 5.347345377 DBX1 yes 3.25147 0.509485 8.89E-05 6.38188 6.38188 3.981945383 DCAF12L2 yes 7.53037 1.01139 2.12E-13 7.44555 7.44555 7.747765387 DCAF13P3 yes 0.0248626 0.00115001 0.00411145 21.6195 21.6195 2.895275406 DCDC2 yes 394.856 92.026 3.74E-06 4.29069 4.29069 4.727495427 DCT yes 13.3765 0.938049 1.15E-07 14.2599 14.2599 5.454615449 DDC yes 172.842 25.8384 0.00458554 6.68934 6.68934 2.859725456 DDIT4L yes 254.46 26.1129 1.57E-11 9.74463 9.74463 7.055375501 DDX39AP1 yes 0.277633 0.0123918 4.64E-09 22.4045 22.4045 6.065495510 DDX4 yes 6.77874 1.6818 2.23E-05 4.03066 4.03066 4.319895548 DEFA5 yes 23626.1 4.12059 0.00064244 5733.67 5733.67 3.455175549 DEFA6 yes 2732.3 0.559849 0.000619089 4880.42 4880.42 3.46565550 DEFA7P yes 0.0374503 0.00117371 0.00189307 31.9076 31.9076 3.138755551 DEFA8P yes 1.90997 0.0267167 0.000836116 71.4895 71.4895 3.380185552 DEFA9P yes 3.42734 0.0382255 0.000732204 89.661 89.661 3.418115578 DEFB116 yes 0.131333 0.0213742 0.00254548 6.14447 6.14447 3.047595594 DEFB133 yes 0.0370669 0.00247521 0.00515993 14.9752 14.9752 2.820885600 DEFT1P yes 0.0755338 0.00881973 0.00134245 8.56418 8.56418 3.242025609 DENND2C yes 69.8466 297.405 0.00115378 0.234853 -4.25798 -3.286715657 DGKB yes 93.623 13.7852 0.00111293 6.79154 6.79154 3.297285663 DGKK yes 2.61251 0.562319 7.60E-06 4.64595 4.64595 4.568625684 DHRS2 yes 1249.91 140.984 0.00466875 8.86564 8.86564 2.853835735 DIRAS2 yes 279.157 13.4434 1.80E-07 20.7654 20.7654 5.365025743 DIS3L2P1 yes 2.11967 0.194905 0.00308845 10.8754 10.8754 2.98695764 DKK1 yes 2816.96 433.172 6.31E-05 6.50309 6.50309 4.067485765 DKK2 yes 257.255 16.1475 0.000307479 15.9316 15.9316 3.658375767 DKK4 yes 663.489 13.5504 1.74E-06 48.9644 48.9644 4.894285793 DLGAP2 yes 94.2138 12.1553 0.000205215 7.75085 7.75085 3.766175798 DLK1 yes 120.359 9.82799 0.00735349 12.2466 12.2466 2.701735805 DLX1 yes 192.492 14.115 2.95E-05 13.6374 13.6374 4.25315806 DLX2 yes 114.018 10.6887 3.72E-06 10.6672 10.6672 4.728435809 DLX5 yes 1561.61 140.974 3.48E-18 11.0773 11.0773 9.379635810 DLX6 yes 289.31 38.8325 1.19E-15 7.45021 7.45021 8.535735811 DLX6-AS1 yes 801.017 72.4769 1.25E-09 11.052 11.052 6.302265813 DMBT1 yes 3586.64 717.115 0.000586004 5.00148 5.00148 3.481035817 DMGDH yes 64.9011 14.6897 3.50E-05 4.41812 4.41812 4.211815823 DMRT3 yes 141.467 21.3298 1.58E-07 6.63236 6.63236 5.390365825 DMRTA2 yes 39.3886 166.636 0.00402969 0.236375 -4.23056 -2.901785840 DNAH10 yes 229.354 43.9961 2.34E-12 5.21306 5.21306 7.366645870 DNAJB1P1 yes 26.0083 0.278047 2.16E-05 93.5392 93.5392 4.32666019 DPEP1 yes 3722.68 89.9247 7.05E-06 41.3978 41.3978 4.585556036 DPP10 yes 78.7992 9.11538 1.99E-14 8.64463 8.64463 8.113496040 DPP6_2 yes 125.853 12.2158 5.83E-06 10.3025 10.3025 4.628476044 DPPA2 yes 3.54977 0.64681 0.00529565 5.48813 5.48813 2.812286062 DPRXP4 yes 0.0462707 0.00226604 0.00259817 20.4192 20.4192 3.04126082 DPYSL4 yes 91.8159 12.4736 1.83E-09 7.36082 7.36082 6.234576083 DPYSL5 yes 183.331 13.6304 0.00033995 13.4501 13.4501 3.631226106 DSC3 yes 2330.11 10670.6 0.000181621 0.218368 -4.57943 -3.798256112 DSCR10 yes 0.673722 0.0576628 1.07E-05 11.6838 11.6838 4.490686114 DSCR4 yes 10.7229 1.29044 2.48E-06 8.30952 8.30952 4.817626116 DSCR8 yes 17.6677 1.76774 0.000127951 9.9945 9.9945 3.88916137 DTD2 yes 1396.28 227.29 0.000307849 6.14315 6.14315 3.658046152 DUOX1 yes 460.064 2112.69 4.46E-07 0.217762 -4.59217 -5.180026154 DUOXA1 yes 214.184 1069.01 7.95E-06 0.200357 -4.9911 -4.558416175 DUSP26 yes 54.3971 3.84885 9.29E-05 14.1333 14.1333 3.970566176 DUSP27 yes 255.265 16.8103 0.00589317 15.1851 15.1851 2.776666199 DUX2 yes 1.40101 0.144749 0.000332929 9.67893 9.67893 3.636876200 DUX4 yes 0.563 0.101005 0.000172945 5.57398 5.57398 3.811056206 DUX4L15 yes 0.461479 0.102113 0.000653823 4.5193 4.5193 3.450216212 DUX4L3 yes 0.438569 0.0880542 0.000347157 4.98067 4.98067 3.625526213 DUX4L4 yes 0.470903 0.11436 0.00191725 4.11773 4.11773 3.134896215 DUX4L6 yes 0.527669 0.122521 0.00201746 4.30674 4.30674 3.119346216 DUX4L7 yes 0.503595 0.116624 0.000504746 4.31811 4.31811 3.522716218 DUXA yes 0.833655 0.168284 1.61E-07 4.95386 4.95386 5.386696223 DUXAP4 yes 0.188824 0.0364127 9.42E-05 5.18567 5.18567 3.967166238 DYNC1I1 yes 344.218 34.1377 7.09E-20 10.0832 10.0832 9.922466276 E2F5 yes 389.989 82.5523 8.34E-33 4.72415 4.72415 13.78826300 ECEL1 yes 312.531 52.5712 0.000322302 5.94491 5.94491 3.645656319 EDA2R yes 49.7324 12.4129 9.63E-10 4.0065 4.0065 6.349396324 EDDM3A yes 1.34354 0.0940419 0.000763454 14.2866 14.2866 3.40626386 EEF1B2P1 yes 0.0777123 0.0143397 0.00267011 5.41939 5.41939 3.032676389 EEF1B2P4 yes 0.317181 0.0554421 2.74E-05 5.72093 5.72093 4.270336390 EEF1B2P5 yes 0.191286 0.0234822 0.000793915 8.146 8.146 3.395026401 EEF1GP2 yes 0.0956731 0.0170808 0.00214454 5.6012 5.6012 3.100616435 EFHD1 yes 1099.75 204.722 3.55E-11 5.37191 5.37191 6.919116444 EFNB3 yes 235.48 22.5206 1.82E-23 10.4562 10.4562 11.03636446 EFR3B yes 270.368 45.131 1.01E-13 5.99074 5.99074 7.86386449 EFTUD1P1 yes 3.79574 0.821497 3.58E-10 4.62052 4.62052 6.52396450 EFTUD1P2 yes 0.111584 0.0137746 0.00138598 8.10074 8.10074 3.232546457 EGFLAM yes 508.616 88.6938 3.27E-06 5.73452 5.73452 4.756926553 EIF4A1P1 yes 0.0772368 0.0131991 0.00198064 5.85168 5.85168 3.124976604 EIF5AP2 yes 0.232655 0.051408 0.000326951 4.52565 4.52565 3.641786613 ELAVL2 yes 11.9158 180.398 8.06E-05 0.0660531 -15.1393 -4.006416619 ELF2P2 yes 7.06388 1.75819 0.000584546 4.01769 4.01769 3.481736628 ELK1P1 yes 0.04161 0.00118585 1.74E-05 35.0886 35.0886 4.378536682 EMID1 yes 1378.75 161.061 9.51E-15 8.56044 8.56044 8.225396700 EMX2 yes 1042.47 86.982 3.38E-20 11.9849 11.9849 10.02416701 EMX2OS yes 821.635 100.918 9.19E-14 8.14161 8.14161 7.878316705 ENAM yes 14.0748 0.961266 3.89E-05 14.6419 14.6419 4.186496794 EPHA7 yes 379.232 21.0468 5.03E-13 18.0185 18.0185 7.612046885 ERVMER34-1 yes 743.791 173.134 2.51E-07 4.29603 4.29603 5.297266932 ETV4 yes 1670.49 373.207 4.09E-17 4.47605 4.47605 9.028227001 F10 yes 118.736 26.6355 3.84E-08 4.45781 4.45781 5.667997007 F2 yes 14.8398 2.47971 0.00159621 5.9845 5.9845 3.190337010 F2RL2 yes 17.2644 1.70624 0.000577265 10.1184 10.1184 3.485257023 FABP1 yes 3607.87 36.3143 6.19E-06 99.3512 99.3512 4.615147045 FABP7 yes 43.0462 1.48769 3.27E-05 28.9349 28.9349 4.228367048 FABP9 yes 8.32411 0.509153 0.000558769 16.3489 16.3489 3.494367086 FAM110B yes 180.886 31.4568 1.20E-13 5.7503 5.7503 7.836557135 FAM138B yes 0.849777 0.132196 3.01E-05 6.42816 6.42816 4.247887146 FAM149B1P1 yes 0.15304 0.0215884 7.28E-05 7.08899 7.08899 4.031997147 FAM150A yes 10.4531 2.54232 0.00280959 4.11163 4.11163 3.016727148 FAM150B yes 308.562 17.0173 0.00050667 18.1323 18.1323 3.521657156 FAM155A yes 153.276 18.0339 1.32E-10 8.49933 8.49933 6.69677165 FAM159B yes 10.4247 1.57529 2.53E-07 6.61764 6.61764 5.296137183 FAM169B yes 35.1046 5.66647 0.00243551 6.19513 6.19513 3.061327187 FAM171A1 yes 1147.52 257.858 2.13E-25 4.4502 4.4502 11.61937188 FAM171A2 yes 497.486 105.766 5.24E-12 4.70367 4.70367 7.235857225 FAM189A2 yes 559.262 96.3996 0.00109461 5.8015 5.8015 3.302147237 FAM197Y2 yes 0.0213091 0.00115001 0.00616966 18.5296 18.5296 2.761277291 FAM218A yes 0.496004 0.109288 1.80E-07 4.53851 4.53851 5.364427329 FAM25G yes 0.614775 3.01851 0.0108593 0.203669 -4.90994 -2.565737360 FAM43B yes 34.3778 6.36973 8.47E-07 5.39706 5.39706 5.046927367 FAM47A yes 0.09073 0.0117637 0.000805968 7.71273 7.71273 3.390717403 FAM69B yes 784.534 132.38 2.00E-24 5.92636 5.92636 11.32737431 FAM83A yes 554.687 3845.5 0.000433179 0.144243 -6.93274 -3.564997465 FAM8A2P yes 0.192496 0.00716975 1.05E-09 26.8484 26.8484 6.33487489 FAM90A27P yes 1.04927 0.0107696 0.000284585 97.4291 97.4291 3.679197490 FAM90A28P yes 2.77347 0.0125318 8.66E-06 221.314 221.314 4.5397574 FBN2 yes 2393.93 368.732 3.57E-10 6.49234 6.49234 6.524217602 FBXO16 yes 107.338 22.9864 8.73E-09 4.66961 4.66961 5.948597656 FCF1P3 yes 0.0657891 0.00773667 0.0011831 8.50355 8.50355 3.279347697 FDPSP9 yes 0.0996569 0.0215077 0.00145035 4.63354 4.63354 3.219027722 FERD3L yes 0.416508 0.02174 0.00010386 19.1585 19.1585 3.942427734 FEZF2 yes 2.35515 0.0341975 0.000630282 68.869 68.869 3.460567740 FGB yes 1093.35 50.936 0.000139105 21.4652 21.4652 3.867587753 FGF13 yes 222.699 33.6782 6.11E-17 6.61256 6.61256 8.970417760 FGF17 yes 44.1328 10.5084 0.000223888 4.19975 4.19975 3.743167761 FGF18 yes 320.496 26.0493 4.27E-13 12.3034 12.3034 7.637997762 FGF19 yes 4414.36 8.31269 2.71E-08 531.039 531.039 5.735137764 FGF20 yes 529.227 1.1928 7.90E-10 443.683 443.683 6.384577765 FGF21 yes 3.52342 0.636004 1.74E-05 5.53994 5.53994 4.377547767 FGF23 yes 13.0783 0.215336 2.36E-10 60.7346 60.7346 6.595927768 FGF3 yes 812.895 3.00536 1.19E-07 270.482 270.482 5.447927769 FGF4 yes 279.003 0.0344844 2.01E-06 8090.72 8090.72 4.862887771 FGF6 yes 1.1083 0.24718 7.31E-07 4.48378 4.48378 5.077647775 FGF8 yes 77.2176 2.19149 2.83E-05 35.2352 35.2352 4.263087776 FGF9 yes 485.116 15.8596 5.83E-17 30.5883 30.5883 8.977097780 FGFR1 yes 3482.88 727.96 3.21E-12 4.78444 4.78444 7.315557792 FGFR4 yes 685.62 168.621 3.50E-07 4.06603 4.06603 5.230097794 FGG yes 273.129 19.7456 0.000635181 13.8324 13.8324 3.458377796 FGL1 yes 134.036 15.7515 3.37E-06 8.50939 8.50939 4.750417797 FGL2 yes 194.318 42.4658 0.0041377 4.57587 4.57587 2.893217886 FLJ26245 yes 14.4417 1.0676 0.00119708 13.5273 13.5273 3.275897895 FLJ30838 yes 49.3045 7.62867 6.22E-14 6.46306 6.46306 7.938487905 FLJ33544 yes 0.447926 0.0397307 6.17E-05 11.2741 11.2741 4.073057906 FLJ33581 yes 7.66222 0.876104 0.00275941 8.74579 8.74579 3.022387919 FLJ37505 yes 9.23018 0.421943 0.000868201 21.8755 21.8755 3.369367921 FLJ37786 yes 2.93093 0.429405 0.00197638 6.82557 6.82557 3.125637927 FLJ39080 yes 30.1187 5.27655 5.55E-12 5.70803 5.70803 7.226547963 FLJ45974 yes 18.0256 3.87036 0.00415528 4.65734 4.65734 2.891837979 FLRT1 yes 1.99934 0.10319 2.84E-07 19.3754 19.3754 5.272297994 FMN2 yes 62.8464 6.35736 1.29E-08 9.88561 9.88561 5.874988037 FOLH1 yes 277.825 45.9878 2.03E-15 6.04127 6.04127 8.457058052 FOXA2 yes 546.488 74.9127 1.63E-11 7.295 7.295 7.049038061 FOXD3 yes 6.6157 39.3935 0.000598486 0.167939 -5.95455 -3.475128094 FOXO1B yes 0.190362 0.0182363 1.71E-06 10.4386 10.4386 4.897288120 FREM1 yes 280.515 49.8877 6.03E-05 5.62293 5.62293 4.078778121 FREM2 yes 689.544 94.4727 3.90E-16 7.29886 7.29886 8.700558125 FRG2 yes 21.5333 0.709334 5.35E-06 30.3571 30.3571 4.647628126 FRG2B yes 8.31616 0.531958 0.000802788 15.6331 15.6331 3.391848127 FRG2C yes 80.1195 0.312695 0.000540088 256.223 256.223 3.503868129 FRMD1 yes 88.0307 3.19731 8.48E-05 27.5327 27.5327 3.993798137 FRMD7 yes 7.24965 1.11737 0.000863275 6.48814 6.48814 3.370998140 FRMPD1 yes 171.365 7.07184 5.29E-06 24.232 24.232 4.650378147 FRRS1L yes 14.5825 1.97566 2.75E-05 7.38107 7.38107 4.26998153 FRZB yes 868.313 115.582 2.71E-07 7.51255 7.51255 5.282298180 FTH1P15 yes 0.301285 0.0678779 0.000103624 4.43862 4.43862 3.9438186 FTH1P21 yes 0.464589 0.00920506 0.000892086 50.471 50.471 3.361548210 FTLP19 yes 0.0616653 0.00511143 0.00227192 12.0642 12.0642 3.082848253 FUT9 yes 32.327 1.87315 3.82E-11 17.2581 17.2581 6.906968265 FXYD6P1 yes 0.109165 0.0224956 0.000764751 4.85271 4.85271 3.405728278 FZD3 yes 578.152 122.695 7.88E-23 4.71209 4.71209 10.84248304 GABBR2 yes 74.7342 15.3157 0.00955781 4.87959 4.87959 2.610878311 GABRA2 yes 45.164 3.62936 9.73E-07 12.444 12.444 5.017698312 GABRA3 yes 498.304 69.7073 2.29E-08 7.14853 7.14853 5.767088316 GABRB1 yes 25.1985 3.35647 1.76E-05 7.50745 7.50745 4.375758321 GABRG1 yes 2.98259 0.284828 3.47E-05 10.4715 10.4715 4.213638322 GABRG2 yes 5.30272 0.319393 1.37E-06 16.6025 16.6025 4.945718324 GABRP yes 583.999 4834.08 0.00892087 0.120809 -8.27755 -2.6358329 GAD1 yes 663.258 158.293 4.90E-08 4.19006 4.19006 5.621248357 GAL3ST3 yes 35.5073 0.156008 1.12E-09 227.6 227.6 6.323358369 GALNT13 yes 30.3802 7.54738 2.00E-07 4.02526 4.02526 5.343778372 GALNT16 yes 281.279 67.1189 1.99E-05 4.19076 4.19076 4.346178380 GALNT8 yes 11.1045 2.31086 7.60E-06 4.80533 4.80533 4.568738385 GALP yes 2.76217 0.399842 1.45E-11 6.90813 6.90813 7.068128386 GALR1 yes 2.63472 0.19912 7.22E-08 13.2319 13.2319 5.545598396 GAP43 yes 29.8399 6.34167 1.72E-08 4.70537 4.70537 5.821698400 GAPDHP15 yes 0.585224 0.0763328 6.95E-08 7.66675 7.66675 5.552938406 GAPDHP21 yes 1.3888 0.249056 0.00627477 5.57625 5.57625 2.755588409 GAPDHP24 yes 0.168381 0.0113951 2.73E-05 14.7767 14.7767 4.271018411 GAPDHP26 yes 0.0387161 0.00152323 0.00285281 25.4171 25.4171 3.011938428 GAPDHP43 yes 0.0248626 0.00195374 0.00822415 12.7256 12.7256 2.663248456 GAPDHP71 yes 7.21127 1.54 3.66E-07 4.68264 4.68264 5.220958525 GCG yes 8.01197 0.831633 0.00861266 9.63402 9.63402 2.647248563 GDF11 yes 569.542 133.302 6.00E-17 4.27257 4.27257 8.973148565 GDF2 yes 0.0421275 0.00414107 0.0102337 10.1731 10.1731 2.586788567 GDF5 yes 63.4758 15.7989 0.000193218 4.01774 4.01774 3.782028569 GDF7 yes 22.5168 2.66733 0.00207861 8.44169 8.44169 3.11028606 GFRA1 yes 435.846 30.8476 0.000764661 14.129 14.129 3.405758609 GFRA4 yes 1.86852 0.0285087 6.09E-08 65.5421 65.5421 5.578958623 GGT2 yes 76.3341 12.9082 1.03E-09 5.91362 5.91362 6.336738624 GGT3P yes 6.59671 1.50981 2.38E-05 4.36922 4.36922 4.304178635 GGTLC5P yes 1.33805 0.332429 2.95E-05 4.02508 4.02508 4.252828667 GIP yes 3.22168 0.714762 1.45E-05 4.50735 4.50735 4.420448696 GJD4 yes 3.22387 0.679515 1.74E-07 4.74437 4.74437 5.371848719 GLI1 yes 100.098 22.6918 1.46E-09 4.41119 4.41119 6.274588730 GLIS3-AS1 yes 0.0635735 0.0105499 0.00465598 6.026 6.026 2.854738735 GLP1R yes 9.4717 1.60192 3.56E-05 5.9127 5.9127 4.207818771 GLUD2 yes 15.6036 1.89901 1.70E-16 8.21671 8.21671 8.821758775 GLULP3 yes 0.154873 0.0122783 0.000598647 12.6136 12.6136 3.475048780 GLYATL1 yes 36.8897 4.79586 2.24E-11 7.69198 7.69198 6.99628796 GMCL1P2 yes 0.433753 0.0458368 4.59E-08 9.463 9.463 5.633598832 GNAT3 yes 53.0547 1.04047 9.27E-05 50.9913 50.9913 3.971238851 GNG5P1 yes 0.0213091 0.00114525 0.0060985 18.6065 18.6065 2.765178917 GOLGA8CP yes 2.48278 0.319354 0.000525243 7.77437 7.77437 3.511638918 GOLGA8DP yes 6.58596 0.428569 0.000346904 15.3673 15.3673 3.625728960 GPA33 yes 480.239 65.8709 0.0052454 7.29061 7.29061 2.815448990 GPHB5 yes 0.481722 0.0476121 1.77E-12 10.1176 10.1176 7.411678996 GPM6A yes 24.3213 5.70286 6.24E-05 4.26476 4.26476 4.070519005 GPR101 yes 4.23989 0.0192714 0.000691325 220.009 220.009 3.434439017 GPR119 yes 0.233709 0.0155155 5.36E-09 15.0629 15.0629 6.038789025 GPR133 yes 103.226 22.0379 5.80E-06 4.68402 4.68402 4.629459038 GPR149 yes 15.5402 2.18938 0.000530329 7.09799 7.09799 3.508959056 GPR173 yes 205.351 48.5512 1.63E-09 4.22958 4.22958 6.255629075 GPR33 yes 1.87541 0.42588 0.00716288 4.40362 4.40362 2.71079089 GPR6 yes 0.169496 0.0259235 8.54E-07 6.5383 6.5383 5.045159095 GPR68 yes 132.632 699.089 0.00154666 0.189721 -5.27089 -3.199799101 GPR83 yes 197.63 5.36807 6.26E-09 36.8159 36.8159 6.010389118 GPRC6A yes 3.527 0.653323 4.37E-06 5.39856 5.39856 4.692799133 GPX3 yes 5129.49 1250.81 9.44E-06 4.10093 4.10093 4.519419135 GPX5 yes 2.35668 0.0779569 0.000423595 30.2306 30.2306 3.571159145 GRAMD4P2 yes 0.809479 0.068381 0.000258504 11.8378 11.8378 3.704939157 GREM2 yes 168.472 10.6652 2.14E-06 15.7964 15.7964 4.849059162 GRIA1 yes 14.316 2.96376 0.000101177 4.83034 4.83034 3.949079163 GRIA2 yes 98.3698 4.75164 1.04E-05 20.7023 20.7023 4.49729165 GRIA4 yes 31.7227 4.80546 4.27E-12 6.60139 6.60139 7.26919175 GRIK3 yes 96.2592 12.7432 1.98E-09 7.55379 7.55379 6.220529177 GRIK5 yes 179.318 25.9687 9.08E-07 6.90518 6.90518 5.032229180 GRIN2B yes 26.1047 3.37177 1.02E-09 7.74213 7.74213 6.339149181 GRIN2C yes 20.0862 95.0762 0.00112065 0.211265 -4.7334 -3.295269190 GRK1_2 yes 6.9284 1.63949 2.58E-07 4.22595 4.22595 5.292059204 GRM8 yes 343.184 34.8848 6.94E-08 9.83762 9.83762 5.553289212 GRPR yes 24.9548 3.80708 4.85E-05 6.55485 6.55485 4.132359223 GSDMC yes 174.885 780.78 0.000178275 0.223987 -4.46454 -3.803129227 GSG1L yes 30.0699 4.65533 1.18E-06 6.45924 6.45924 4.976539249 GSTM2 yes 1462.48 272.828 3.34E-10 5.36045 5.36045 6.536129256 GSTM5P1 yes 1.32346 0.0380328 1.13E-05 34.7978 34.7978 4.477849268 GSX1 yes 1.86192 0.0637292 0.000578681 29.2161 29.2161 3.484569321 GUCA2A yes 68.8763 0.503955 6.48E-05 136.672 136.672 4.061049339 GUSBP10 yes 2.95276 0.145712 9.79E-05 20.2643 20.2643 3.957429341 GUSBP12 yes 0.0574443 0.00139645 0.00135731 41.1359 41.1359 3.238759346 GUSBP7 yes 0.0406016 0.00117121 0.00167588 34.6664 34.6664 3.175679352 GXYLT1P1 yes 0.0171063 0.000950325 0.00669777 18.0005 18.0005 2.733539358 GYG1P3 yes 0.181557 0.0353962 5.36E-05 5.1293 5.1293 4.10779368 GYS2 yes 39.7009 7.02715 4.42E-09 5.64964 5.64964 6.074269395 H2AFZP5 yes 0.165637 0.0370127 0.00380915 4.47513 4.47513 2.919959397 H2BFM yes 1.82301 0.392448 0.000783788 4.64521 4.64521 3.398699399 H2BFWT yes 2.64624 0.429595 3.80E-05 6.15985 6.15985 4.192039401 H3.X yes 2.73999 0.0353577 2.04E-06 77.4932 77.4932 4.859339402 H3.Y yes 28.2205 0.388226 1.46E-06 72.6908 72.6908 4.930899429 HAND1 yes 124.878 1.0939 1.22E-06 114.158 114.158 4.969089430 HAND2 yes 160.439 24.7792 7.61E-06 6.47474 6.47474 4.568339431 HAND2-AS1 yes 57.9056 9.08464 9.27E-05 6.37401 6.37401 3.971299438 HAPLN4 yes 28.9292 6.44716 0.000213205 4.48712 4.48712 3.756099464 HAVCR1 yes 30.342 2.73435 1.31E-08 11.0966 11.0966 5.872389522 HCN1 yes 82.5927 3.5223 4.73E-06 23.4485 23.4485 4.675349529 HCRTR2 yes 9.39896 0.900738 8.59E-08 10.4347 10.4347 5.51159588 HEPACAM yes 11.9279 1.20131 0.000232879 9.92901 9.92901 3.732729590 HEPH yes 794.611 173.715 0.000317432 4.57421 4.57421 3.649779630 HGC6.3 yes 7.23726 0.36527 6.12E-12 19.8135 19.8135 7.210429639 HHIP yes 101.492 16.3569 0.000354898 6.20483 6.20483 3.619539640 HHIP-AS1 yes 32.5105 7.70403 1.90E-06 4.21994 4.21994 4.8759642 HHIPL2 yes 66.8272 13.0001 3.60E-06 5.14051 5.14051 4.735759662 HIGD1AP11 yes 0.0284903 0.00121278 0.00338158 23.4918 23.4918 2.95819676 HIGD1AP8 yes 0.362693 0.0553629 4.10E-05 6.5512 6.5512 4.173589810 HLA-DPB2 yes 56.3319 10.2932 0.000407562 5.47272 5.47272 3.581749858 HMGA1P7 yes 0.424643 0.0750238 2.27E-07 5.66011 5.66011 5.317929882 HMGB1P29 yes 0.113261 0.0250137 0.00340102 4.52795 4.52795 2.956279901 HMGB1P46 yes 0.138006 0.00647775 4.60E-08 21.3046 21.3046 5.633399915 HMGB3P11 yes 0.0762049 0.00180508 6.38E-05 42.2168 42.2168 4.064989916 HMGB3P12 yes 0.062919 0.0074184 0.00335169 8.48149 8.48149 2.960939921 HMGB3P17 yes 0.322848 0.049803 0.000774575 6.4825 6.4825 3.402079922 HMGB3P18 yes 0.201139 0.0146658 0.000552086 13.7149 13.7149 3.497739947 HMGCLL1 yes 9.2811 1.98865 0.000168638 4.66704 4.66704 3.817639959 HMGN1P17 yes 0.608007 0.104716 0.000122304 5.80627 5.80627 3.9006910021 HMGN2P39 yes 0.0248626 0.00111067 0.0037805 22.3853 22.3853 2.9223810047 HMP19 yes 8.41148 1.15267 1.75E-06 7.29738 7.29738 4.8929310048 HMSD yes 10.9375 50.4077 0.00085842 0.216981 -4.60869 -3.3726210049 HMX1 yes 125.8 9.14119 0.000559837 13.7618 13.7618 3.4938310051 HMX3 yes 6.9876 0.517448 9.91E-06 13.504 13.504 4.5082710057 HNF4A yes 703.213 140.24 0.00340176 5.01437 5.01437 2.956210115 HNRNPA1P58 yes 0.0210637 0.00121087 0.0073958 17.3955 17.3955 2.6997710118 HNRNPA1P60 yes 0.0517632 0.00470399 0.00161931 11.0041 11.0041 3.1860110158 HNRNPCP5 yes 0.75905 0.0937998 7.47E-07 8.09224 8.09224 5.0730710180 HNRNPKP3 yes 0.210522 0.0407955 0.000129941 5.16042 5.16042 3.8851310203 HOMER2P1 yes 0.0840477 0.00318012 1.03E-06 26.4291 26.4291 5.0061410222 HOXA11 yes 493.071 118.565 1.23E-07 4.15863 4.15863 5.4398410249 HOXC12 yes 36.7163 1.75824 1.03E-06 20.8824 20.8824 5.0051110252 HOXC5 yes 20.7861 4.47838 3.15E-07 4.64143 4.64143 5.2515910255 HOXC9 yes 61.746 13.2852 1.81E-10 4.64772 4.64772 6.6418810421 HPX yes 60.0456 13.577 0.000172887 4.4226 4.4226 3.8111410550 HSPA9P2 yes 0.588107 0.0150748 6.81E-05 39.0126 39.0126 4.0484610610 HTR3C yes 2.61603 0.619763 0.000400082 4.22102 4.22102 3.5868210649 IBSP yes 32.4692 6.77257 3.25E-06 4.79421 4.79421 4.7583810714 IFITM5 yes 13.7908 1.49396 0.000784235 9.23104 9.23104 3.3985310750 IFNNP1 yes 0.0533529 0.00432142 0.00125889 12.3461 12.3461 3.2610410777 IGBP1P4 yes 0.870051 0.109562 1.65E-06 7.94117 7.94117 4.9049410779 IGDCC3 yes 54.8278 2.47934 5.83E-08 22.1138 22.1138 5.5871610781 IGF1 yes 1503.8 216.633 1.94E-05 6.94171 6.94171 4.3528210783 IGF2 yes 5682.02 785.96 1.17E-08 7.2294 7.2294 5.8931810797 IGFBPL1 yes 1327.77 215.758 1.02E-05 6.15397 6.15397 4.5011610803 IGFL4 yes 9.74059 1.47685 5.55E-09 6.59553 6.59553 6.0324910805 IGFN1 yes 88.104 11.7817 5.18E-12 7.47805 7.47805 7.2377110881 IGHV3-13 yes 12.6265 1.19285 0.00107479 10.5852 10.5852 3.3074710890 IGHV3-29 yes 0.0774324 0.00988963 0.0107585 7.82966 7.82966 2.5690510913 IGHV3-62 yes 0.411288 0.0537303 0.00217996 7.65468 7.65468 3.0955810925 IGHV3-79 yes 0.251391 0.0183914 1.77E-09 13.6689 13.6689 6.2404110927 IGHV3OR15-7 yes 5.24318 0.290541 0.00136913 18.0463 18.0463 3.2361810943 IGHV4-80 yes 0.549658 0.0293688 2.37E-11 18.7157 18.7157 6.9868510945 IGHV5-78 yes 1.15202 0.190655 9.92E-07 6.04243 6.04243 5.0136510951 IGHV7-81 yes 1.30402 0.071312 6.40E-10 18.2861 18.2861 6.4217710973 IGHVII-78-1 yes 0.18907 0.0181025 3.14E-07 10.4444 10.4444 5.2522611013 IGKV1-37 yes 3.275 0.672781 0.0038722 4.86786 4.86786 2.9146611028 IGKV1D-37 yes 3.84183 0.862605 0.00625947 4.45375 4.45375 2.756411064 IGKV2OR2-7 yes 0.933534 0.0539314 1.51E-12 17.3097 17.3097 7.4371311065 IGKV2OR2-7D yes 2.77918 0.149056 2.75E-07 18.6452 18.6452 5.27911066 IGKV2OR2-8 yes 2.94519 0.279149 7.91E-07 10.5506 10.5506 5.0611211105 IGLJCOR18 yes 0.13625 0.0240321 0.00353839 5.66949 5.66949 2.9436211110 IGLON5 yes 128.857 15.858 0.000371986 8.12568 8.12568 3.6067211184 IGSF1 yes 62.3125 15.5533 0.000164372 4.00638 4.00638 3.8243111197 IHH yes 558.771 44.871 2.81E-07 12.4528 12.4528 5.2744211220 IL12RB2 yes 59.8377 290.25 0.00122584 0.206159 -4.85062 -3.2688911224 IL15 yes 74.4714 314.998 0.000206305 0.236418 -4.22979 -3.7647711253 IL1RN yes 731.126 3788.85 0.00201058 0.192968 -5.18222 -3.1203811276 IL31 yes 0.210637 0.00498222 0.00100459 42.2778 42.2778 3.3271611301 IL9 yes 0.609998 0.109219 5.83E-05 5.58509 5.58509 4.0871311334 IMPDH1P3 yes 0.461088 0.0873288 0.000123401 5.27991 5.27991 3.898411360 INHBB yes 801.6 182.771 3.22E-07 4.38582 4.38582 5.2467511372 INPP1 yes 363.618 1578.08 0.000782483 0.230418 -4.33994 -3.3991711386 INSC yes 12.1592 2.969 5.40E-07 4.0954 4.0954 5.1405111447 IQCF6 yes 0.304436 0.0579862 0.000871349 5.25015 5.25015 3.3683211493 IRX5 yes 57.8655 307.619 1.15E-05 0.188108 -5.31609 -4.4733411512 ISLR2 yes 115.047 21.7531 1.03E-11 5.28877 5.28877 7.1247211557 ITGB6 yes 635.904 3389.49 0.000183509 0.18761 -5.3302 -3.7955411573 ITM2C yes 7825.5 1896.41 2.30E-12 4.12649 4.12649 7.3691411593 IYD yes 160.773 23.5406 1.94E-05 6.82961 6.82961 4.352311631 JPH4 yes 35.3785 4.56658 1.12E-05 7.74727 7.74727 4.4802111650 KANK4 yes 683.612 64.849 3.18E-13 10.5416 10.5416 7.6843411675 KBTBD12 yes 65.2711 303.46 0.00978775 0.21509 -4.64922 -2.6025111689 KCNA4 yes 0.8176 0.166622 1.33E-05 4.90691 4.90691 4.4413311691 KCNA6 yes 248.217 5.91465 2.08E-07 41.9665 41.9665 5.3353111692 KCNA7 yes 1.94932 9.00783 0.00140279 0.216403 -4.621 -3.2289611701 KCNC2 yes 177.968 2.05368 0.000325468 86.658 86.658 3.6430111706 KCND3 yes 14.9865 71.809 0.00117225 0.208699 -4.79159 -3.2820511712 KCNF1 yes 81.1523 3.67758 1.88E-08 22.0668 22.0668 5.8047911719 KCNH3 yes 352.979 71.2463 3.18E-07 4.95435 4.95435 5.2496911724 KCNH8 yes 115.666 17.7926 3.66E-10 6.50077 6.50077 6.5200211735 KCNJ13 yes 0.29212 0.0264512 4.22E-07 11.0437 11.0437 5.1914911742 KCNJ4 yes 34.4541 5.15522 1.32E-05 6.68333 6.68333 4.4415211752 KCNK16 yes 9.79191 0.0731737 2.24E-08 133.817 133.817 5.7715711753 KCNK17 yes 30.4776 7.09809 0.000299431 4.29378 4.29378 3.6655211768 KCNN1 yes 75.6304 9.51215 6.19E-21 7.95092 7.95092 10.256111782 KCNS1 yes 60.2734 566.229 8.01E-05 0.106447 -9.39435 -4.0081111788 KCNV1 yes 9.5466 0.128212 0.000212775 74.4595 74.4595 3.7566211832 KDM4E yes 5.46821 0.410464 2.95E-08 13.322 13.322 5.7187711853 KHDRBS2 yes 26.2348 4.18162 8.76E-09 6.27384 6.27384 5.9479511869 KIAA0226L yes 327.681 69.925 5.21E-06 4.68617 4.68617 4.6535111893 KIAA1024L yes 6.54542 0.100753 1.15E-14 64.9653 64.9653 8.1966711895 KIAA1045 yes 61.8419 13.2863 2.77E-05 4.65456 4.65456 4.2682811903 KIAA1210 yes 64.3038 2.17339 0.000252984 29.5869 29.5869 3.7106911904 KIAA1211 yes 292.92 56.6275 1.68E-13 5.17275 5.17275 7.7842711965 KIF1A yes 1740.04 156.481 9.95E-11 11.1198 11.1198 6.744811976 KIF25-AS1 yes 33.8128 1.20022 7.08E-09 28.1721 28.1721 5.9875511992 KIF5C_2 yes 309.267 36.3585 4.44E-11 8.50605 8.50605 6.8815512016 KIRREL3-AS3 yes 0.376442 0.0426621 1.51E-05 8.8238 8.8238 4.4112112018 KISS1R yes 61.4259 10.6159 5.78E-07 5.78621 5.78621 5.1267212055 KLHDC7B yes 85.0347 2495.63 0.000462528 0.0340735 -29.3483 -3.5469112064 KLHL14 yes 453.289 61.7102 7.46E-14 7.34545 7.34545 7.9105412140 KNOP1P1 yes 0.210637 0.0345385 0.0082937 6.09861 6.09861 2.6603312181 KRT14 yes 4263.03 73865.8 0.0112223 0.0577132 -17.3271 -2.5540112214 KRT18P29 yes 0.0648542 0.0102598 0.00711389 6.3212 6.3212 2.7130412230 KRT18P43 yes 0.0619108 0.00669155 0.0041946 9.25208 9.25208 2.8887712244 KRT18P58 yes 0.0945082 0.0124231 0.000262028 7.60744 7.60744 3.7013112252 KRT18P66 yes 0.0435878 0.00321271 0.0008383 13.5673 13.5673 3.3794312262 KRT19P4 yes 0.0910656 0.0191398 0.00338397 4.75793 4.75793 2.9578712264 KRT20 yes 1499.72 11.1027 8.82E-07 135.077 135.077 5.0382112269 KRT25 yes 3.28493 0.136024 6.16E-09 24.1496 24.1496 6.0132412270 KRT26 yes 4.35615 0.0319799 0.000350339 136.215 136.215 3.6230412271 KRT27 yes 153.906 2.37548 1.58E-07 64.7894 64.7894 5.3911312272 KRT28 yes 318.46 0.0583498 2.95E-07 5457.77 5457.77 5.2644512277 KRT33B yes 27.3594 3.71836 0.000613291 7.35793 7.35793 3.4682512283 KRT39 yes 55.0743 2.87437 1.80E-05 19.1605 19.1605 4.3695812290 KRT6A yes 24104.7 97178.9 0.0013559 0.248045 -4.03153 -3.2390612294 KRT71 yes 17.0113 1.05958 8.74E-07 16.0547 16.0547 5.040312295 KRT72 yes 44.6776 5.59438 0.00247698 7.98615 7.98615 3.0560812296 KRT73 yes 26.0234 2.21445 0.000245188 11.7516 11.7516 3.7190212297 KRT74 yes 30.2713 7.546 5.09E-06 4.01158 4.01158 4.6587312300 KRT77 yes 1119.95 11.1558 0.000722953 100.392 100.392 3.4217312311 KRT8P1 yes 0.11569 0.00838646 1.79E-05 13.7949 13.7949 4.3711212318 KRT8P16 yes 0.0834918 0.00515227 0.000218534 16.2048 16.2048 3.7495612324 KRT8P21 yes 0.0903269 0.0171577 0.00674941 5.2645 5.2645 2.7309312338 KRT8P34 yes 0.0235391 0.00145812 0.00925621 16.1435 16.1435 2.6221112352 KRT8P48 yes 0.201125 0.0477057 0.00405807 4.21595 4.21595 2.899512360 KRTAP1-1 yes 2.39959 0.0675316 7.94E-05 35.5329 35.5329 4.0102512361 KRTAP1-3 yes 4.91867 0.064573 4.37E-05 76.1722 76.1722 4.1580912377 KRTAP11-1 yes 4.13265 0.0815071 1.85E-06 50.7029 50.7029 4.8804812389 KRTAP16-1 yes 0.702886 0.141767 0.000466753 4.95803 4.95803 3.544412402 KRTAP2-1 yes 2.95766 0.0248025 0.000327483 119.249 119.249 3.6413412403 KRTAP2-2 yes 4.25287 0.0721488 0.000402744 58.9458 58.9458 3.58512405 KRTAP2-4 yes 1.22554 0.0286003 0.000488466 42.8505 42.8505 3.5318112423 KRTAP3-1 yes 74.7626 2.1053 3.27E-06 35.5116 35.5116 4.7567912424 KRTAP3-2 yes 8.69776 0.833869 0.000231642 10.4306 10.4306 3.7341312425 KRTAP3-3 yes 17.4649 0.124113 0.000218361 140.717 140.717 3.7497712432 KRTAP4-3 yes 0.124313 0.00436886 0.00135644 28.4543 28.4543 3.2389412433 KRTAP4-4 yes 0.217626 0.0125413 0.000665509 17.3527 17.3527 3.445212436 KRTAP4-7 yes 1.9051 0.00751625 4.47E-08 253.464 253.464 5.6388312438 KRTAP4-9 yes 2.89393 0.0155029 2.30E-06 186.67 186.67 4.8339612442 KRTAP5-13P yes 0.216156 0.0515135 0.00210211 4.19611 4.19611 3.1067512445 KRTAP5-3 yes 0.109416 0.0183219 0.00024999 5.97188 5.97188 3.7138612457 KRTAP8-1 yes 0.0497252 0.00484513 0.0104553 10.2629 10.2629 2.579212463 KRTAP9-2 yes 0.745876 0.0132799 0.000844345 56.1657 56.1657 3.3773712464 KRTAP9-3 yes 0.443556 0.00984983 0.000105098 45.0319 45.0319 3.9394112467 KRTAP9-7 yes 0.09945 0.0027792 0.00122985 35.7837 35.7837 3.2679312468 KRTAP9-8 yes 0.786676 0.101254 0.00578556 7.76931 7.76931 2.7828212469 KRTAP9-9 yes 0.200058 0.00745188 1.71E-07 26.8466 26.8466 5.3744712480 KYNU yes 151.546 906.604 0.00769556 0.167158 -5.98236 -2.6861412504 LAMA1 yes 771.763 114.78 2.93E-10 6.72386 6.72386 6.5586312599 LCN10 yes 55.1786 6.6052 1.08E-09 8.35381 8.35381 6.3283912601 LCN15 yes 899.308 10.3418 1.49E-06 86.9582 86.9582 4.9264412644 LDOC1 yes 1006.46 168.612 5.93E-06 5.96911 5.96911 4.6246812647 LECT1 yes 20.0943 2.05947 3.77E-06 9.75699 9.75699 4.7256412649 LEF1 yes 1491 188.731 6.96E-13 7.90013 7.90013 7.5608212650 LEF1-AS1 yes 26.8453 3.91169 9.85E-14 6.86285 6.86285 7.8675312679 LEUTX yes 3.65176 0.120861 0.00011399 30.2145 30.2145 3.918712699 LGI1 yes 7.68474 0.763115 0.00189291 10.0702 10.0702 3.1387812706 LGR5 yes 773.052 101.308 2.92E-06 7.6307 7.6307 4.7817612708 LGSN yes 28.8398 1.99329 1.45E-09 14.4684 14.4684 6.2762812720 LHX1 yes 527.518 13.0634 2.73E-10 40.3814 40.3814 6.5707312721 LHX2 yes 786.557 122.749 0.00191006 6.40782 6.40782 3.1360312726 LHX8 yes 11.7586 0.570396 0.00103663 20.6148 20.6148 3.3180212727 LHX9 yes 416.237 22.8133 2.30E-08 18.2454 18.2454 5.7664512760 LIMS3-LOC440895 yes 292.76 16.6102 1.04E-12 17.6253 17.6253 7.4974412761 LIMS3L yes 145.371 27.5623 6.33E-08 5.27427 5.27427 5.5712612762 LIN28A yes 99.1473 0.756307 1.31E-08 131.094 131.094 5.8725412770 LIN7A yes 89.9919 8.11713 1.26E-08 11.0867 11.0867 5.8806812779 LINC00051 yes 32.0935 2.47341 0.000146069 12.9754 12.9754 3.8549512811 LINC00207 yes 0.196328 0.0416255 0.00675106 4.71654 4.71654 2.7308512817 LINC00229 yes 1.04904 0.167692 2.70E-10 6.25573 6.25573 6.5731312828 LINC00261 yes 240.175 20.2166 2.79E-09 11.8801 11.8801 6.158212841 LINC00273 yes 0.133919 0.0154368 2.42E-05 8.6753 8.6753 4.2998112853 LINC00303 yes 13.6654 0.633083 5.68E-13 21.5855 21.5855 7.5928812863 LINC00314 yes 0.370512 0.0546606 4.19E-05 6.7784 6.7784 4.1682612875 LINC00331 yes 0.508936 0.0655124 3.34E-07 7.76854 7.76854 5.2395812895 LINC00379 yes 2.82054 0.609633 6.57E-06 4.62663 4.62663 4.6016412899 LINC00403 yes 8.87044 0.822194 0.00252015 10.7887 10.7887 3.050712902 LINC00421 yes 0.598719 0.0462365 0.00218025 12.949 12.949 3.0955412909 LINC00442 yes 2.12292 0.0383416 0.000463994 55.3686 55.3686 3.5460412918 LINC00461 yes 178.796 7.02374 6.06E-11 25.456 25.456 6.8290712934 LINC00483 yes 37.8715 3.18896 0.000160621 11.8758 11.8758 3.8303112938 LINC00488 yes 7.05235 0.570859 2.54E-08 12.3539 12.3539 5.7472212944 LINC00502 yes 0.271716 0.0399436 7.82E-08 6.80249 6.80249 5.5298612945 LINC00507 yes 0.670706 0.0796056 0.000125294 8.42536 8.42536 3.8944912953 LINC00523 yes 5.39852 0.0857624 4.12E-09 62.9474 62.9474 6.0874312957 LINC00534 yes 23.4838 4.32326 0.000101665 5.43196 5.43196 3.9478512960 LINC00538 yes 0.182706 0.0252984 1.09E-06 7.22204 7.22204 4.9945312962 LINC00540 yes 67.41 5.52555 3.11E-15 12.1997 12.1997 8.393312969 LINC00552 yes 2.53905 0.377468 2.02E-05 6.72654 6.72654 4.3424912994 LINC00599 yes 0.884406 0.105426 0.00104294 8.38887 8.38887 3.3162512998 LINC00606 yes 0.424125 0.0667335 1.21E-05 6.3555 6.3555 4.462213001 LINC00609 yes 5.66563 0.977752 0.000102682 5.79454 5.79454 3.9453213005 LINC00616 yes 44.9165 0.911917 1.17E-05 49.255 49.255 4.4696413006 LINC00617 yes 27.8731 0.49945 3.80E-05 55.8076 55.8076 4.1921313008 LINC00619 yes 1.12816 0.241424 2.66E-08 4.67295 4.67295 5.7387413012 LINC00624 yes 96.2663 6.28801 2.42E-05 15.3095 15.3095 4.2998413013 LINC00626 yes 16.4724 3.32038 0.00247384 4.96101 4.96101 3.0564713024 LINC00639 yes 40.4595 4.96371 6.50E-08 8.15105 8.15105 5.5662313027 LINC00642 yes 0.745575 0.1141 2.15E-06 6.53441 6.53441 4.848313029 LINC00645 yes 7.27955 0.0992812 1.05E-10 73.3226 73.3226 6.7355313030 LINC00648 yes 24.3475 1.01104 7.52E-11 24.0816 24.0816 6.7925513037 LINC00658 yes 0.694418 0.15715 0.000980253 4.41883 4.41883 3.3342813045 LINC00668 yes 106.693 9.80088 2.10E-05 10.8861 10.8861 4.3332513048 LINC00671 yes 29.7384 1.11101 5.62E-07 26.7669 26.7669 5.1324113053 LINC00676 yes 0.289868 0.0137432 6.84E-08 21.0918 21.0918 5.5562513055 LINC00681 yes 1.47065 0.189906 0.000809382 7.7441 7.7441 3.389513056 LINC00684 yes 0.0830824 0.00770909 8.20E-06 10.7772 10.7772 4.5514113066 LINC00698 yes 3.73631 0.445999 4.99E-08 8.37739 8.37739 5.6175713067 LINC00700 yes 1.73377 0.0595497 0.000679719 29.1147 29.1147 3.4392213077 LINC00837 yes 0.331928 0.0716473 6.84E-05 4.6328 4.6328 4.0475113091 LINC00858 yes 26.5377 1.3049 4.69E-24 20.3369 20.3369 11.215413096 LINC00867 yes 27.088 1.36584 1.64E-09 19.8325 19.8325 6.2545113112 LINC00889 yes 4.48629 0.550182 1.83E-16 8.15419 8.15419 8.8116213123 LINC00901 yes 0.155437 0.0305457 0.00153374 5.08865 5.08865 3.2023113145 LINC00930 yes 25.3212 6.08011 0.000851157 4.16459 4.16459 3.3750613153 LINC00940 yes 3.01696 0.360141 1.82E-10 8.37717 8.37717 6.6413413172 LINC00967 yes 4.12314 0.0130571 0.000134395 315.777 315.777 3.8764613196 LINC01006 yes 76.75 18.9742 3.59E-08 4.04497 4.04497 5.6813113207 LINC01020 yes 0.334092 0.0803584 0.000832285 4.15752 4.15752 3.381513233 LIX1 yes 198.968 9.56759 8.76E-07 20.796 20.796 5.0397713239 LMAN1L yes 23.6676 3.90628 0.00689716 6.05887 6.05887 2.7235813254 LMO1 yes 124.059 24.7917 3.83E-06 5.00408 5.00408 4.7218513276 LOC100093698 yes 0.421719 0.0484322 0.00197718 8.7074 8.7074 3.125513306 LOC100127902 yes 0.183985 0.028761 0.00632893 6.39705 6.39705 2.7526813307 LOC100127903 yes 0.733517 0.153838 0.00122685 4.7681 4.7681 3.2686513349 LOC100128139 yes 0.499087 0.0680863 3.80E-06 7.33021 7.33021 4.7239213391 LOC100128427 yes 0.302862 0.0592298 0.000636794 5.11334 5.11334 3.4576613401 LOC100128505 yes 0.0370669 0.00152228 0.00312391 24.3495 24.3495 2.9832813405 LOC100128540 yes 0.107758 0.0174884 0.00470902 6.16169 6.16169 2.8510113419 LOC100128601 yes 0.0756941 0.0060298 2.88E-05 12.5533 12.5533 4.2588913455 LOC100128833 yes 1.47749 0.113503 0.000906263 13.0171 13.0171 3.3569913463 LOC100128885 yes 34.9886 2.46159 1.50E-14 14.2138 14.2138 8.1564213472 LOC100128948 yes 0.714442 0.012054 5.42E-06 59.2703 59.2703 4.6446313479 LOC100129001 yes 0.0185835 0.00102865 0.00662862 18.0659 18.0659 2.7370513486 LOC100129053 yes 3.16101 0.0312974 2.08E-07 100.999 100.999 5.3353413493 LOC100129098 yes 192.903 13.0108 6.63E-10 14.8264 14.8264 6.4155413495 LOC100129100 yes 1.58305 0.278819 1.91E-11 5.67769 5.67769 7.0231713497 LOC100129108 yes 0.613935 0.0385926 7.34E-06 15.9081 15.9081 4.5764713512 LOC100129184 yes 3.83064 0.760447 0.00826632 5.03735 5.03735 2.6614713526 LOC100129265 yes 2.42429 0.55784 0.000642055 4.34584 4.34584 3.4553413530 LOC100129276 yes 0.716311 0.0755126 0.000936647 9.48598 9.48598 3.3474613532 LOC100129293 yes 1.62358 0.159735 4.47E-15 10.1642 10.1642 8.3390113537 LOC100129331 yes 0.0936257 0.00431693 0.00194651 21.6881 21.6881 3.1302713562 LOC100129473 yes 0.0284903 0.00183365 0.0104225 15.5375 15.5375 2.5803113568 LOC100129507 yes 0.579414 0.0135258 7.01E-12 42.8378 42.8378 7.1883513573 LOC100129527 yes 0.107245 0.0155203 0.000277238 6.90998 6.90998 3.6862113584 LOC100129603 yes 6.93233 1.39692 0.000114472 4.96257 4.96257 3.9176313586 LOC100129611 yes 0.102838 0.0168803 0.0103953 6.0922 6.0922 2.5812413598 LOC100129672 yes 0.046769 0.00248838 0.000750908 18.795 18.795 3.4109313600 LOC100129683 yes 0.0497252 0.00339592 0.00553088 14.6426 14.6426 2.7978413613 LOC100129774 yes 2.12981 0.40108 0.00711794 5.31019 5.31019 2.7128513615 LOC100129779 yes 0.428666 0.00158861 3.56E-08 269.838 269.838 5.6828313632 LOC100129935 yes 0.12898 0.00883658 2.70E-06 14.5962 14.5962 4.7987513643 LOC100129976 yes 0.440808 0.0531142 1.59E-08 8.29926 8.29926 5.8360813645 LOC100129996 yes 0.0992464 0.0104593 0.000743281 9.48878 9.48878 3.4138413649 LOC100130011 yes 0.0842548 0.0106559 0.00985701 7.90687 7.90687 2.6000213650 LOC100130018 yes 0.235604 0.0448607 0.00019613 5.25189 5.25189 3.7780913663 LOC100130088 yes 1.87836 0.317589 0.00133582 5.91442 5.91442 3.2434913687 LOC100130256 yes 13.9708 1.9437 9.91E-14 7.18774 7.18774 7.866513711 LOC100130417 yes 48.3622 8.55864 1.23E-06 5.65069 5.65069 4.9681613720 LOC100130480 yes 3.31606 0.771503 0.000444095 4.29818 4.29818 3.5581413753 LOC100130674 yes 0.0426182 0.0035804 0.0104676 11.9032 11.9032 2.5787813760 LOC100130700 yes 0.0812033 0.00590495 0.00352507 13.7517 13.7517 2.9448313761 LOC100130704 yes 0.180262 0.025188 0.00431682 7.15664 7.15664 2.8794313780 LOC100130871 yes 0.185877 0.0326233 0.00127917 5.69766 5.69766 3.2563213788 LOC100130909 yes 1.40228 0.0935646 3.77E-09 14.9873 14.9873 6.1034813802 LOC100131038 yes 0.0495136 0.00217863 5.41E-05 22.7269 22.7269 4.1054413816 LOC100131138 yes 1.85755 0.308345 0.00741594 6.02428 6.02428 2.6988413832 LOC100131284 yes 0.0478484 0.0048077 0.00184975 9.95246 9.95246 3.1457913854 LOC100131479 yes 0.91121 0.0251821 3.10E-05 36.1848 36.1848 4.2412913858 LOC100131520 yes 0.0894749 0.00838761 6.86E-05 10.6675 10.6675 4.0468613864 LOC100131546 yes 64.4935 7.13634 0.00437542 9.03732 9.03732 2.8750413868 LOC100131562 yes 0.0941567 0.00583772 0.00258225 16.129 16.129 3.0431213877 LOC100131624 yes 0.209377 0.0201812 0.0020025 10.3748 10.3748 3.1216113881 LOC100131641 yes 0.0652683 0.00802095 0.00336457 8.13722 8.13722 2.9597113906 LOC100131890 yes 0.039068 0.00388576 0.00418827 10.0541 10.0541 2.8892613911 LOC100131953 yes 7.54788 1.2801 0.00204498 5.8963 5.8963 3.1151913915 LOC100132009 yes 3.05799 0.573962 2.16E-09 5.32785 5.32785 6.2043113929 LOC100132086 yes 6.0173 1.01868 3.06E-05 5.90695 5.90695 4.2442813936 LOC100132154 yes 0.408892 0.0670052 2.15E-08 6.10238 6.10238 5.7791913953 LOC100132287 yes 52.7537 10.5046 1.36E-07 5.02195 5.02195 5.4209613954 LOC100132304 yes 0.300165 0.0519129 1.40E-07 5.78208 5.78208 5.4152313959 LOC100132354 yes 82.9492 2.25226 9.50E-11 36.8293 36.8293 6.7527613962 LOC100132443 yes 0.187094 0.01026 0.000598902 18.2353 18.2353 3.4749213978 LOC100132626 yes 0.0406016 0.00152824 0.00260967 26.5676 26.5676 3.0398313986 LOC100132698 yes 0.0643703 0.0108858 0.00773259 5.91323 5.91323 2.6844913990 LOC100132735 yes 7.72533 0.544754 0.000108283 14.1813 14.1813 3.9318114005 LOC100132830 yes 0.0811535 0.0157591 0.00217882 5.14964 5.14964 3.0957414023 LOC100133007 yes 0.883751 0.0447292 1.38E-06 19.7578 19.7578 4.9432414031 LOC100133092 yes 0.979084 0.114156 4.68E-14 8.57673 8.57673 7.9824214044 LOC100133203 yes 0.170969 0.0136389 0.000202529 12.5353 12.5353 3.7696414063 LOC100133461 yes 0.183348 0.0152819 0.00157002 11.9977 11.9977 3.195314078 LOC100135064 yes 0.102547 0.0224356 0.00365947 4.57072 4.57072 2.9328414079 LOC100144595 yes 3.88581 0.959215 0.000157746 4.05103 4.05103 3.8350114084 LOC100174950 yes 0.849801 0.0914614 5.59E-06 9.29136 9.29136 4.6378314088 LOC100190922 yes 0.1097 0.0064982 4.60E-05 16.8816 16.8816 4.1455214092 LOC100190940 yes 58.8525 7.34375 2.79E-05 8.01395 8.01395 4.2665514104 LOC100216479 yes 93.7579 7.52764 8.67E-09 12.4552 12.4552 5.9499514117 LOC100270670 yes 0.109648 0.00149766 0.000107809 73.2126 73.2126 3.9329214118 LOC100270679 yes 1.14084 0.078053 2.02E-06 14.6163 14.6163 4.8616114123 LOC100271717 yes 0.0630326 0.0075074 0.000332003 8.39606 8.39606 3.6376314128 LOC100271907 yes 1.3315 0.0791238 0.000909253 16.828 16.828 3.3560414142 LOC100286992 yes 0.141246 0.027558 0.000702905 5.1254 5.1254 3.4297214180 LOC100287413 yes 1.47594 0.306552 0.0102842 4.81464 4.81464 2.5850414197 LOC100287541 yes 8.81517 0.673282 3.32E-05 13.0928 13.0928 4.2244414216 LOC100287840 yes 0.0462707 0.00161121 0.00225616 28.718 28.718 3.0849914291 LOC100288570 yes 56.8656 5.5497 1.58E-10 10.2466 10.2466 6.6657814292 LOC100288590 yes 0.187109 0.00671735 1.16E-10 27.8546 27.8546 6.7182714294 LOC100288623 yes 54.9726 11.1612 0.000919213 4.92535 4.92535 3.3528914295 LOC100288628 yes 0.243237 0.0555083 0.00541315 4.382 4.382 2.80514321 LOC100288897 yes 42.6291 2.791 1.55E-07 15.2738 15.2738 5.3949314354 LOC100289290 yes 10.7762 1.59959 0.00477793 6.73683 6.73683 2.8462414448 LOC100418733 yes 0.0614855 0.00583836 0.00185077 10.5313 10.5313 3.1456314473 LOC100418888 yes 0.511698 0.0832595 3.78E-05 6.14582 6.14582 4.1930214476 LOC100418908 yes 0.0743343 0.00259314 0.00155995 28.6658 28.6658 3.1972314536 LOC100419238 yes 0.855619 0.187186 7.21E-05 4.57097 4.57097 4.0343214543 LOC100419318 yes 0.123795 0.0245485 0.000486355 5.04285 5.04285 3.5330114549 LOC100419366 yes 0.0623129 0.00275715 4.76E-05 22.6005 22.6005 4.1371714580 LOC100419614 yes 0.0463454 0.00720825 0.0081188 6.42949 6.42949 2.667714581 LOC100419615 yes 1.10879 0.0497481 1.89E-07 22.2881 22.2881 5.3543314599 LOC100419678 yes 0.0421275 0.00303335 0.00631513 13.8881 13.8881 2.7534214643 LOC100419780 yes 1.0227 0.080053 0.000822641 12.7752 12.7752 3.3848414644 LOC100419781 yes 0.118849 0.0215077 0.00455952 5.52588 5.52588 2.8615814647 LOC100419784 yes 0.186979 0.0254929 1.60E-06 7.33456 7.33456 4.9125514690 LOC100419859 yes 0.0387161 0.00217849 0.00694857 17.772 17.772 2.7210514691 LOC100419861 yes 0.419645 0.0360878 3.42E-06 11.6284 11.6284 4.7470114695 LOC100419872 yes 0.055929 0.0016691 5.88E-07 33.5084 33.5084 5.1228414743 LOC100419985 yes 19.0173 0.334919 5.12E-06 56.7819 56.7819 4.657614744 LOC100419986 yes 7.97819 0.165822 1.12E-06 48.1131 48.1131 4.9883614747 LOC100419991 yes 0.258827 0.0632505 5.61E-05 4.09209 4.09209 4.0965314780 LOC100420083 yes 0.501106 0.116517 1.34E-05 4.30071 4.30071 4.4381314784 LOC100420095 yes 0.475207 0.117061 0.000327574 4.05949 4.05949 3.6412614786 LOC100420097 yes 0.27235 0.0622191 0.000228191 4.37727 4.37727 3.7381114807 LOC100420171 yes 2.11858 0.430714 2.13E-05 4.91876 4.91876 4.3305714843 LOC100420321 yes 0.04161 0.00306682 0.000740333 13.5678 13.5678 3.4149714856 LOC100420351 yes 0.325587 0.0433636 2.68E-05 7.50831 7.50831 4.2760414864 LOC100420403 yes 1.06733 0.00503712 0.000697616 211.894 211.894 3.4318614891 LOC100420480 yes 0.279896 0.0334745 0.00657123 8.36145 8.36145 2.7399914901 LOC100420510 yes 4.26388 0.357367 0.000193303 11.9314 11.9314 3.781914914 LOC100420545 yes 0.0979693 0.00640311 5.99E-05 15.3003 15.3003 4.0802714956 LOC100420669 yes 0.263707 0.0520803 0.00370758 5.06348 5.06348 2.9286414957 LOC100420670 yes 0.265364 0.0662957 0.000901526 4.00274 4.00274 3.358514966 LOC100420711 yes 0.0655572 0.00398014 8.85E-05 16.4711 16.4711 3.9828915005 LOC100420828 yes 0.0465773 0.00120281 1.88E-05 38.7236 38.7236 4.3593815018 LOC100420863 yes 3.09617 0.194994 3.75E-06 15.8782 15.8782 4.7263615085 LOC100421093 yes 0.443765 0.105028 0.00150245 4.2252 4.2252 3.2084815135 LOC100421288 yes 0.0560339 0.00210548 3.95E-05 26.6134 26.6134 4.1823315162 LOC100421386 yes 0.0581306 0.00669654 0.00469243 8.68069 8.68069 2.8521715201 LOC100421509 yes 0.268491 0.0143615 0.000516752 18.6953 18.6953 3.5161715215 LOC100421548 yes 0.189519 0.0430779 4.79E-05 4.39945 4.39945 4.1356315239 LOC100421618 yes 0.210937 0.00121278 1.20E-05 173.929 173.929 4.4649215245 LOC100421632 yes 0.0415434 0.00247775 0.000175166 16.7666 16.7666 3.8077115256 LOC100421667 yes 1.50503 0.0355359 0.000348074 42.3525 42.3525 3.624815258 LOC100421670 yes 0.0871504 0.0032971 0.000151317 26.4325 26.4325 3.8458115271 LOC100421697 yes 0.064275 0.00775246 0.00603714 8.29091 8.29091 2.7685615277 LOC100421703 yes 16.7022 3.73153 5.40E-16 4.47597 4.47597 8.6528215278 LOC100421704 yes 9.89366 1.24194 4.99E-11 7.96627 7.96627 6.8618815290 LOC100421746 yes 0.0791944 0.00269153 1.18E-05 29.4236 29.4236 4.4687515336 LOC100422021 yes 5.52457 0.175881 1.21E-05 31.4108 31.4108 4.4628615339 LOC100422026 yes 7.62606 0.121108 6.33E-07 62.9691 62.9691 5.1077315340 LOC100422029 yes 0.432474 0.0382222 2.36E-05 11.3147 11.3147 4.3056515346 LOC100422088 yes 0.0381186 0.0031074 0.00134595 12.267 12.267 3.2412515353 LOC100422190 yes 0.713781 0.0609637 0.000125515 11.7083 11.7083 3.8940415364 LOC100422241 yes 0.192671 0.028973 2.83E-05 6.65 6.65 4.2628115365 LOC100422242 yes 0.0656288 0.0123953 0.0109907 5.29466 5.29466 2.5614515367 LOC100422263 yes 1.81068 0.375594 7.10E-05 4.82083 4.82083 4.0381515388 LOC100422319 yes 0.17351 0.0341192 5.54E-05 5.08541 5.08541 4.0999315407 LOC100422375 yes 0.0343382 0.00250681 0.000571121 13.698 13.698 3.4882415458 LOC100422530 yes 0.109289 0.00312519 9.48E-06 34.9705 34.9705 4.5185115465 LOC100422559 yes 0.0258452 0.00106382 0.00313923 24.2947 24.2947 2.9817315489 LOC100422641 yes 0.0248626 0.00143247 0.00744474 17.3565 17.3565 2.6975115521 LOC100499194 yes 0.837476 0.0994774 2.44E-06 8.41875 8.41875 4.8209215541 LOC100505515 yes 0.0180043 0.00116229 0.0105222 15.4903 15.4903 2.5769415553 LOC100505570 yes 0.136063 0.0109575 0.000512215 12.4174 12.4174 3.5186215568 LOC100505663 yes 6.03316 0.0183818 0.000612819 328.213 328.213 3.4684715574 LOC100505685 yes 0.839368 0.0768138 1.52E-06 10.9273 10.9273 4.9224115583 LOC100505727 yes 24.8917 2.96596 2.84E-06 8.39243 8.39243 4.7875815592 LOC100505776 yes 168.062 6.11071 4.18E-06 27.5029 27.5029 4.7024615601 LOC100505811 yes 1.88587 0.234981 6.72E-05 8.02565 8.02565 4.0518315605 LOC100505835 yes 12.05 0.620637 6.25E-06 19.4155 19.4155 4.6129815613 LOC100505878 yes 0.589488 0.00450507 0.000456846 130.85 130.85 3.5503315634 LOC100505995 yes 0.230462 0.0480521 9.33E-06 4.79608 4.79608 4.5219515648 LOC100506055 yes 0.378546 0.0898446 1.13E-08 4.21334 4.21334 5.9012215655 LOC100506085 yes 18.2356 2.00533 0.00364512 9.09356 9.09356 2.934115681 LOC100506216 yes 0.193132 0.0278232 0.0016678 6.94142 6.94142 3.1771315694 LOC100506272 yes 14.7576 1.45494 2.83E-07 10.1431 10.1431 5.2731515710 LOC100506343 yes 0.0845911 0.0091074 8.35E-05 9.28818 9.28818 3.9976515727 LOC100506405 yes 0.058514 0.00149319 2.20E-05 39.1872 39.1872 4.3229215728 LOC100506406 yes 0.063191 0.00549741 0.0069708 11.4947 11.4947 2.7199615730 LOC100506421 yes 35.5686 0.823606 1.44E-09 43.1865 43.1865 6.2779915741 LOC100506474 yes 4.34491 1.07448 3.10E-13 4.04375 4.04375 7.6887215751 LOC100506526 yes 0.302687 0.022245 5.66E-07 13.607 13.607 5.1309515757 LOC100506557 yes 0.208676 0.0459043 0.000402686 4.54589 4.54589 3.5850415778 LOC100506688 yes 35.0767 2.59917 1.06E-10 13.4954 13.4954 6.7340115780 LOC100506700 yes 2.56915 0.469805 6.92E-06 5.46856 5.46856 4.5898715797 LOC100506791 yes 2.76541 0.519757 1.95E-06 5.32058 5.32058 4.8696615805 LOC100506827 yes 5.47124 0.672248 4.94E-06 8.13873 8.13873 4.6654815810 LOC100506858 yes 0.700028 0.116172 0.000727275 6.02579 6.02579 3.4200315812 LOC100506860 yes 16.1791 3.85639 1.51E-15 4.19541 4.19541 8.5009315840 LOC100507012 yes 38.3768 6.84333 0.000942214 5.60791 5.60791 3.3457515859 LOC100507166 yes 0.535715 0.0811407 0.00465568 6.6023 6.6023 2.8547515863 LOC100507194 yes 7.44142 0.353031 4.92E-06 21.0786 21.0786 4.6664915865 LOC100507201 yes 1.28152 0.0377141 0.000269073 33.9797 33.9797 3.6942115875 LOC100507261 yes 0.461121 0.0607547 6.37E-07 7.58989 7.58989 5.1063515877 LOC100507274 yes 2.74441 0.384297 0.0101312 7.14138 7.14138 2.5903415889 LOC100507351 yes 131.264 11.4076 7.90E-12 11.5067 11.5067 7.1687415890 LOC100507353 yes 0.223186 0.0184806 5.31E-06 12.0768 12.0768 4.6493915895 LOC100507377 yes 8.10182 1.94389 0.000657607 4.16783 4.16783 3.4485815913 LOC100507443 yes 1.93696 0.351688 0.00134622 5.50761 5.50761 3.2411915936 LOC100507537 yes 53.3572 0.638263 1.13E-08 83.5975 83.5975 5.9000615950 LOC100507586 yes 0.0390614 0.00277346 0.000472213 14.084 14.084 3.5411815965 LOC100508631 yes 7.08765 1.25344 0.010327 5.65454 5.65454 2.5835715994 LOC100533628 yes 0.0406016 0.00157361 0.00276648 25.8016 25.8016 3.0215716004 LOC100533638 yes 1.90819 0.188046 6.11E-05 10.1475 10.1475 4.0754216010 LOC100533648 yes 7.91837 0.346484 9.13E-06 22.8535 22.8535 4.5270816037 LOC100533712 yes 0.153123 0.0185334 0.00153465 8.26199 8.26199 3.2021316044 LOC100533725 yes 0.0332234 0.00258025 0.0060941 12.876 12.876 2.7654116045 LOC100533727 yes 0.157066 0.0073232 3.43E-07 21.4477 21.4477 5.2337416060 LOC100533843 yes 0.0841452 0.00205255 5.18E-06 40.9955 40.9955 4.6548916061 LOC100533845 yes 2.26566 0.184556 0.00103686 12.2763 12.2763 3.3179616063 LOC100533848 yes 0.138494 0.0289304 0.000186537 4.78714 4.78714 3.7912516076 LOC100631248 yes 5.04618 0.193588 3.93E-08 26.0665 26.0665 5.6636116088 LOC100631378 yes 104.568 14.2309 3.43E-16 7.34792 7.34792 8.719716108 LOC100652833 yes 1.04432 0.154467 0.000535606 6.76078 6.76078 3.5061916127 LOC100653005 yes 1.23296 0.195427 0.000193712 6.30905 6.30905 3.7813516130 LOC100653233 yes 1.12219 0.132708 2.54E-06 8.45607 8.45607 4.8117416135 LOC100736411 yes 0.186376 0.0137298 5.24E-08 13.5746 13.5746 5.6080216145 LOC100862705 yes 0.109578 0.023034 0.000562357 4.75724 4.75724 3.4925716152 LOC100887076 yes 1.36103 0.1192 0.000100699 11.4181 11.4181 3.9502716181 LOC100996301 yes 10.4331 61.3029 0.000173297 0.170189 -5.87581 -3.8105216198 LOC100996354 yes 6.96413 0.962009 0.000591015 7.23915 7.23915 3.4786416207 LOC100996385 yes 8.33338 0.833552 0.000142075 9.99743 9.99743 3.8621216245 LOC100996492 yes 101.379 0.563183 2.07E-12 180.011 180.011 7.3867516279 LOC100996624 yes 0.369616 0.0914215 0.00115713 4.04299 4.04299 3.2858616303 LOC100996694 yes 6.10798 0.303167 1.14E-07 20.1473 20.1473 5.4548716324 LOC101054525 yes 27.9366 0.803384 0.00037177 34.7737 34.7737 3.6068816327 LOC101059915 yes 11.3801 1.33921 3.14E-05 8.49763 8.49763 4.2375216331 LOC101059923 yes 2.20172 0.200067 2.23E-05 11.0049 11.0049 4.3198116335 LOC101059930 yes 0.0619295 0.00192659 3.38E-05 32.1446 32.1446 4.2205316345 LOC101059962 yes 2.07044 0.273558 3.88E-05 7.56856 7.56856 4.1868616355 LOC101059990 yes 1.64073 0.294782 0.000215762 5.56589 5.56589 3.7529416357 LOC101059997 yes 8.40962 1.6465 1.19E-05 5.10757 5.10757 4.4659316374 LOC101060039 yes 0.411698 0.0870493 1.30E-05 4.72948 4.72948 4.4450516380 LOC101060051 yes 46.6353 10.9971 3.40E-05 4.2407 4.2407 4.2187816387 LOC101060067 yes 0.0843309 0.0107512 0.0110912 7.84384 7.84384 2.558216391 LOC101060086 yes 0.0741338 0.00463623 0.00565027 15.9901 15.9901 2.7907316411 LOC101060132 yes 0.0970915 0.0143764 0.000515871 6.75352 6.75352 3.5166416414 LOC101060146 yes 6.6863 1.3896 8.18E-08 4.81167 4.81167 5.5210716435 LOC101060192 yes 0.597747 0.137616 5.95E-09 4.34359 4.34359 6.0197716436 LOC101060193 yes 1.26956 0.0898279 4.17E-07 14.1333 14.1333 5.1941516465 LOC101060825 yes 0.129754 0.0267463 0.00612244 4.8513 4.8513 2.7638516495 LOC101926897 yes 0.499621 0.0980754 1.27E-07 5.09426 5.09426 5.4334116497 LOC101926899 yes 0.0930889 0.0170473 0.0003201 5.46061 5.46061 3.6475116526 LOC101926928 yes 2.48437 0.422808 6.04E-09 5.8759 5.8759 6.016816528 LOC101926930 yes 0.538625 0.0409789 0.000344434 13.144 13.144 3.6276616551 LOC101926953 yes 0.0720171 0.00272605 0.00172236 26.4182 26.4182 3.1674116562 LOC101926964 yes 6.64643 1.37638 2.24E-06 4.82893 4.82893 4.8389416564 LOC101926966 yes 9.09785 0.214426 1.10E-07 42.4288 42.4288 5.4619316606 LOC101927009 yes 2.43663 0.42903 5.36E-06 5.67938 5.67938 4.6471616621 LOC101927024 yes 0.725725 0.154064 1.37E-05 4.71053 4.71053 4.4340716659 LOC101927062 yes 3.06962 0.40177 7.62E-09 7.64023 7.64023 5.9737916671 LOC101927074 yes 0.800193 0.0929916 8.35E-05 8.60501 8.60501 3.9976616715 LOC101927120 yes 1.00269 0.12658 5.16E-05 7.92139 7.92139 4.1170316719 LOC101927124 yes 317.389 78.4241 0.000202846 4.04708 4.04708 3.7692316732 LOC101927136 yes 100.401 10.1202 0.000118993 9.92086 9.92086 3.9077216761 LOC101927166 yes 2.58529 0.563886 1.35E-07 4.58478 4.58478 5.4217716763 LOC101927168 yes 2.55527 0.450728 1.24E-11 5.66922 5.66922 7.094716802 LOC101927207 yes 0.646024 0.053157 0.000214443 12.1531 12.1531 3.7545616804 LOC101927209 yes 5.89022 1.36795 5.58E-05 4.30589 4.30589 4.0980316826 LOC101927231 yes 0.0520295 0.00581894 0.00388522 8.9414 8.9414 2.9135716833 LOC101927238 yes 1.02136 0.182675 2.16E-10 5.59114 5.59114 6.6115116848 LOC101927253 yes 4.18018 0.760363 0.00062909 5.49761 5.49761 3.4610916859 LOC101927264 yes 6.03501 0.114085 0.000259314 52.8994 52.8994 3.7040916860 LOC101927265 yes 0.769527 0.174239 0.00252265 4.4165 4.4165 3.050416867 LOC101927272 yes 0.581897 0.0751192 8.39E-06 7.74632 7.74632 4.546316872 LOC101927277 yes 0.147094 0.00471039 0.00026217 31.2275 31.2275 3.7011616877 LOC101927282 yes 2.78232 0.626663 0.000403142 4.43989 4.43989 3.5847316888 LOC101927293 yes 3.65328 0.535431 1.06E-08 6.82306 6.82306 5.9120216890 LOC101927295 yes 1.42458 0.350404 0.000975392 4.06554 4.06554 3.3357216893 LOC101927298 yes 11.4347 0.743227 9.30E-09 15.3852 15.3852 5.9369216924 LOC101927329 yes 5.5901 0.781335 1.03E-05 7.15455 7.15455 4.4993616926 LOC101927331 yes 0.25988 0.00862589 2.79E-06 30.128 30.128 4.791916928 LOC101927333 yes 25.8313 3.07475 4.13E-05 8.40111 8.40111 4.1718316929 LOC101927334 yes 0.968369 0.0926467 1.28E-07 10.4523 10.4523 5.4330716941 LOC101927346 yes 0.404856 0.0842752 3.18E-05 4.80397 4.80397 4.2347816953 LOC101927358 yes 0.108447 0.0144086 0.00236092 7.5265 7.5265 3.0709616957 LOC101927362 yes 0.0562463 0.00674636 0.00323227 8.33728 8.33728 2.9724716959 LOC101927364 yes 4.00096 0.379862 1.84E-08 10.5327 10.5327 5.8087616963 LOC101927368 yes 0.49117 0.0474913 1.53E-13 10.3423 10.3423 7.7988216976 LOC101927381 yes 12.5919 0.572983 1.51E-09 21.976 21.976 6.2691116978 LOC101927383 yes 2.68657 0.303886 4.68E-08 8.84072 8.84072 5.6299616991 LOC101927396 yes 3.25751 0.515447 1.13E-06 6.31978 6.31978 4.9854716998 LOC101927403 yes 3.41505 0.813173 0.00210897 4.19966 4.19966 3.1057517004 LOC101927409 yes 0.350932 0.0829249 8.79E-06 4.23193 4.23193 4.535517011 LOC101927416 yes 0.0462707 0.00400067 0.0109234 11.5657 11.5657 2.5636417030 LOC101927436 yes 0.187855 0.0357455 0.000187022 5.25534 5.25534 3.7905717046 LOC101927452 yes 3.57721 0.495262 4.52E-05 7.22287 7.22287 4.1497417053 LOC101927459 yes 0.342436 0.0356857 0.000268112 9.59588 9.59588 3.6951717057 LOC101927463 yes 4.54374 0.884059 0.000230491 5.13963 5.13963 3.7354517066 LOC101927473 yes 0.568944 0.102462 1.03E-06 5.55274 5.55274 5.0055917077 LOC101927484 yes 0.170291 0.0120991 3.97E-05 14.0747 14.0747 4.1813517088 LOC101927495 yes 12.6868 2.9649 1.45E-05 4.27901 4.27901 4.4200617176 LOC101927584 yes 0.055547 0.0057817 0.000686362 9.60738 9.60738 3.4364717197 LOC101927605 yes 0.874249 0.0639788 8.70E-06 13.6647 13.6647 4.5378717219 LOC101927627 yes 7.14195 0.12834 0.000360915 55.6488 55.6488 3.6149517234 LOC101927642 yes 0.184585 0.0429798 0.00636273 4.29469 4.29469 2.7508917241 LOC101927650 yes 0.418541 0.0963991 0.00558473 4.34175 4.34175 2.7946217257 LOC101927666 yes 0.214435 0.0479117 0.000149739 4.47563 4.47563 3.8485217270 LOC101927680 yes 328.79 19.6698 1.03E-05 16.7154 16.7154 4.4994517301 LOC101927711 yes 0.137107 0.0272965 0.00143684 5.02287 5.02287 3.2218117334 LOC101927745 yes 9.38884 0.717517 0.00172532 13.0852 13.0852 3.1668917349 LOC101927760 yes 13.8649 0.172968 1.10E-20 80.1587 80.1587 10.17817364 LOC101927775 yes 16.1138 3.10606 5.64E-08 5.18786 5.18786 5.5936717375 LOC101927786 yes 0.708219 0.0451985 1.86E-05 15.6691 15.6691 4.3628317387 LOC101927798 yes 0.0684252 0.00512435 0.00795138 13.353 13.353 2.6748917392 LOC101927803 yes 0.0287222 0.0008657 0.00178444 33.1781 33.1781 3.156717425 LOC101927836 yes 9.61461 1.67361 0.000431529 5.74485 5.74485 3.5660417431 LOC101927842 yes 0.563288 0.036075 7.15E-07 15.6144 15.6144 5.0823417439 LOC101927850 yes 0.066621 0.0129506 0.00360416 5.14425 5.14425 2.9377217447 LOC101927858 yes 0.119899 0.0282021 0.00179311 4.25143 4.25143 3.1552317455 LOC101927866 yes 0.684252 0.00394913 0.000706477 173.267 173.267 3.4282817462 LOC101927873 yes 73.4266 12.2132 4.12E-05 6.01207 6.01207 4.1723717481 LOC101927892 yes 0.17167 0.0189935 0.000406367 9.03834 9.03834 3.5825417489 LOC101927900 yes 1.20816 0.219699 8.89E-06 5.49916 5.49916 4.5330417491 LOC101927902 yes 49.1086 9.90178 6.68E-12 4.95958 4.95958 7.1960917513 LOC101927924 yes 2.12201 0.47986 0.00165942 4.42215 4.42215 3.1786517515 LOC101927926 yes 0.157112 0.0281122 8.25E-05 5.58874 5.58874 4.0005917525 LOC101927936 yes 0.770593 0.110405 2.06E-05 6.9797 6.9797 4.3386117533 LOC101927945 yes 8.3084 1.01236 1.13E-12 8.207 8.207 7.4837417556 LOC101927968 yes 24.6273 2.42945 1.21E-14 10.137 10.137 8.1884317575 LOC101927987 yes 2.19014 0.0255996 2.47E-06 85.5538 85.5538 4.8182317589 LOC101928002 yes 10.3144 2.56964 1.44E-08 4.01396 4.01396 5.8549717593 LOC101928006 yes 1.69685 0.237316 0.00799332 7.15014 7.15014 2.6730717597 LOC101928010 yes 0.0777451 0.00987607 0.00250682 7.87207 7.87207 3.0523617605 LOC101928018 yes 0.1112 0.0126308 0.00557799 8.80391 8.80391 2.7950217627 LOC101928040 yes 2.62639 0.395251 0.00294232 6.64487 6.64487 3.002217634 LOC101928047 yes 82.8297 4.90494 2.45E-05 16.887 16.887 4.2969417653 LOC101928066 yes 8.56143 2.1389 1.61E-05 4.00272 4.00272 4.395417660 LOC101928073 yes 3.28883 0.245421 1.94E-08 13.4008 13.4008 5.7982817685 LOC101928098 yes 0.0462707 0.00197609 0.00204991 23.4153 23.4153 3.1144617695 LOC101928108 yes 2.42424 0.0515822 2.73E-05 46.9976 46.9976 4.2714917700 LOC101928113 yes 15.0237 2.47801 8.47E-06 6.06282 6.06282 4.5439917719 LOC101928132 yes 0.722619 0.162563 0.000241974 4.44516 4.44516 3.7225417724 LOC101928137 yes 3.54486 0.583957 0.000104571 6.07042 6.07042 3.9406917732 LOC101928145 yes 0.0941191 0.00381627 6.10E-07 24.6626 24.6626 5.115517740 LOC101928153 yes 409.359 97.8234 2.32E-10 4.18467 4.18467 6.5994117752 LOC101928165 yes 11.9972 1.54394 4.22E-08 7.77052 7.77052 5.6501817777 LOC101928190 yes 0.247284 0.0334157 8.70E-05 7.40023 7.40023 3.9872417785 LOC101928198 yes 0.154913 0.0339481 0.00160951 4.56322 4.56322 3.1878417788 LOC101928201 yes 0.229678 0.0459527 0.00852364 4.99814 4.99814 2.6508517789 LOC101928202 yes 0.126959 0.00230274 7.63E-08 55.1338 55.1338 5.5347117810 LOC101928223 yes 21.4333 1.81057 9.13E-07 11.8378 11.8378 5.0311117834 LOC101928247 yes 11.8933 1.16541 0.000253784 10.2053 10.2053 3.7098417842 LOC101928255 yes 8.1489 1.34691 1.30E-09 6.05008 6.05008 6.2956717844 LOC101928257 yes 0.123174 0.0247547 0.0103338 4.97577 4.97577 2.5833417852 LOC101928265 yes 0.807426 0.100835 0.00225695 8.00738 8.00738 3.0848817854 LOC101928267 yes 0.0287222 0.00105625 0.00249499 27.1925 27.1925 3.0538317855 LOC101928268 yes 0.1229 0.0236092 0.00230208 5.20559 5.20559 3.0787717866 LOC101928279 yes 96.0299 7.71534 1.34E-08 12.4466 12.4466 5.8678317890 LOC101928303 yes 12.4527 2.38871 1.23E-14 5.21313 5.21313 8.1866617894 LOC101928307 yes 3.55225 0.387985 4.44E-10 9.15563 9.15563 6.486217897 LOC101928310 yes 0.166953 0.00568509 2.40E-07 29.3667 29.3667 5.3064717938 LOC101928353 yes 22.0713 2.16257 1.36E-06 10.206 10.206 4.9472317950 LOC101928365 yes 0.33876 0.0735342 0.000720022 4.60683 4.60683 3.4228817983 LOC101928399 yes 31.5433 135.544 0.000185901 0.232717 -4.29707 -3.7921517985 LOC101928401 yes 1.35489 0.0474263 2.03E-06 28.5683 28.5683 4.8605717989 LOC101928405 yes 0.544929 0.0857308 0.000208553 6.35627 6.35627 3.7619117994 LOC101928410 yes 16.176 2.10516 3.78E-05 7.68398 7.68398 4.1929118000 LOC101928416 yes 1.08672 0.162278 0.00450075 6.69663 6.69663 2.8658218004 LOC101928420 yes 0.481317 0.106701 1.14E-05 4.51089 4.51089 4.47618022 LOC101928439 yes 2.17135 0.492701 0.000142335 4.40703 4.40703 3.8616518057 LOC101928474 yes 4.09489 0.877853 0.00102675 4.66466 4.66466 3.3208118063 LOC101928480 yes 4.82586 0.944774 3.53E-06 5.10795 5.10795 4.7398518078 LOC101928495 yes 0.34929 0.0302894 1.32E-09 11.5318 11.5318 6.2936218101 LOC101928519 yes 5.12315 0.978983 5.56E-09 5.23313 5.23313 6.032218105 LOC101928523 yes 0.0749363 0.0105347 0.00266335 7.11325 7.11325 3.0334718114 LOC101928532 yes 0.271013 0.0220129 0.00430149 12.3116 12.3116 2.8805918120 LOC101928538 yes 9.99893 0.357699 1.58E-05 27.9534 27.9534 4.4007318141 LOC101928559 yes 3.08177 0.386612 3.76E-07 7.97123 7.97123 5.2151718164 LOC101928582 yes 0.0373087 0.00133053 4.87E-05 28.0404 28.0404 4.1316718188 LOC101928607 yes 0.156726 0.0380265 0.00606252 4.12149 4.12149 2.7671618203 LOC101928622 yes 3.33772 0.472048 2.50E-05 7.07073 7.07073 4.2928118206 LOC101928625 yes 0.0518101 0.488548 0.00123619 0.106049 -9.42959 -3.2664218219 LOC101928639 yes 1.03338 0.19514 2.21E-06 5.2956 5.2956 4.842718228 LOC101928648 yes 0.0648542 0.00916466 0.00599875 7.07655 7.07655 2.770718234 LOC101928654 yes 0.066389 0.0139544 0.00691153 4.75757 4.75757 2.7228718258 LOC101928679 yes 1.35529 0.00624788 2.64E-05 216.919 216.919 4.2790918343 LOC101928765 yes 12.1435 2.27793 8.27E-10 5.33093 5.33093 6.3765218348 LOC101928770 yes 1.09877 0.235123 0.000158697 4.67317 4.67317 3.8334518355 LOC101928777 yes 1.30398 0.264106 0.000731593 4.93732 4.93732 3.4183518379 LOC101928801 yes 17.7199 3.42455 0.000888148 5.17439 5.17439 3.3628218410 LOC101928832 yes 0.610991 0.0501687 0.000354346 12.1787 12.1787 3.6199518418 LOC101928840 yes 0.759223 0.147406 0.00133807 5.15057 5.15057 3.2429918429 LOC101928851 yes 0.703498 0.125228 0.000573791 5.61775 5.61775 3.4869418436 LOC101928858 yes 7.98063 1.1831 0.00168569 6.74554 6.74554 3.1739118437 LOC101928859 yes 0.593565 0.111617 2.35E-10 5.31785 5.31785 6.5968718459 LOC101928881 yes 5.48389 0.789211 4.72E-05 6.94858 6.94858 4.1389818466 LOC101928888 yes 0.0745876 0.00514956 0.00399716 14.4843 14.4843 2.904418472 LOC101928894 yes 1.65679 0.185785 5.96E-07 8.91781 8.91781 5.1201318489 LOC101928911 yes 5.24094 1.06313 3.55E-10 4.92975 4.92975 6.5252218516 LOC101928938 yes 0.0462707 0.00137302 0.00174429 33.6999 33.6999 3.1635918523 LOC101928945 yes 2.77458 0.498894 3.21E-07 5.56146 5.56146 5.2477618543 LOC101928965 yes 0.121661 0.66373 0.000117806 0.183299 -5.45558 -3.9102818559 LOC101928982 yes 114.233 20.2105 2.40E-15 5.65219 5.65219 8.4319518560 LOC101928983 yes 41.8324 8.65901 7.12E-13 4.83108 4.83108 7.5570618575 LOC101928999 yes 0.0439975 0.00417522 0.00158043 10.5378 10.5378 3.1933118595 LOC101929019 yes 2.37147 0.308542 0.000230151 7.68605 7.68605 3.7358418603 LOC101929027 yes 10.4307 2.18174 2.23E-08 4.7809 4.7809 5.7721818611 LOC101929036 yes 1.04305 0.0705899 3.27E-05 14.7762 14.7762 4.2282118657 LOC101929083 yes 1.95688 0.295628 1.35E-14 6.61941 6.61941 8.1717718693 LOC101929120 yes 0.426998 0.0292695 8.40E-10 14.5885 14.5885 6.3737318700 LOC101929127 yes 11.1054 2.18037 1.70E-05 5.09334 5.09334 4.3832318701 LOC101929128 yes 61.808 4.69654 0.000805909 13.1603 13.1603 3.3907318716 LOC101929144 yes 0.220579 0.0298729 1.09E-05 7.38393 7.38393 4.4867718729 LOC101929157 yes 4.94576 1.22413 4.76E-06 4.04023 4.04023 4.6739918739 LOC101929167 yes 0.289596 0.0543935 0.00640751 5.32409 5.32409 2.7485218746 LOC101929176 yes 4.16967 0.2786 7.01E-06 14.9665 14.9665 4.5869218790 LOC101929221 yes 7.87825 1.0668 0.00177132 7.38494 7.38494 3.1589318816 LOC101929247 yes 3.66471 0.533123 0.000923988 6.87403 6.87403 3.351418831 LOC101929262 yes 4.14935 0.351231 6.76E-05 11.8138 11.8138 4.0503418834 LOC101929265 yes 28.5449 1.31759 0.000114158 21.6645 21.6645 3.9183318836 LOC101929267 yes 105.955 0.464887 1.48E-05 227.916 227.916 4.4151718874 LOC101929305 yes 3.74988 0.853169 2.53E-10 4.39524 4.39524 6.5844218885 LOC101929316 yes 2.80965 0.0815838 4.06E-08 34.4389 34.4389 5.6573918918 LOC101929350 yes 3.01383 0.136727 0.000253721 22.0426 22.0426 3.7099118999 LOC101929432 yes 4.15664 0.76589 0.000427603 5.4272 5.4272 3.5685619015 LOC101929448 yes 8.72511 1.87855 1.50E-07 4.64458 4.64458 5.4016819024 LOC101929457 yes 5.039 1.22389 6.27E-06 4.11721 4.11721 4.6120419041 LOC101929474 yes 0.562526 0.101914 3.61E-09 5.51961 5.51961 6.1112419047 LOC101929480 yes 0.0852367 0.00534021 0.00315085 15.9613 15.9613 2.9805619081 LOC101929514 yes 0.248983 0.0350684 4.33E-06 7.09991 7.09991 4.6950419096 LOC101929529 yes 0.019358 0.00104174 0.00612057 18.5825 18.5825 2.7639519098 LOC101929531 yes 5.09449 20.6101 0.00159423 0.247184 -4.04556 -3.190719116 LOC101929549 yes 15.0957 2.88362 0.000359028 5.23498 5.23498 3.6163819127 LOC101929560 yes 166.867 27.1248 1.60E-08 6.15182 6.15182 5.8349319129 LOC101929562 yes 0.222669 0.040811 4.44E-05 5.45611 5.45611 4.1542619169 LOC101929602 yes 0.0458285 0.00154923 5.78E-05 29.5815 29.5815 4.0892219171 LOC101929604 yes 49.6694 6.01256 1.13E-05 8.26094 8.26094 4.4774819221 LOC101929654 yes 6.06506 0.580366 2.49E-05 10.4504 10.4504 4.2936719227 LOC101929660 yes 0.510572 0.0560191 0.00526239 9.11425 9.11425 2.8143719241 LOC101929674 yes 0.258867 0.0381203 7.21E-06 6.79079 6.79079 4.580519246 LOC101929680 yes 3.40481 0.377916 1.02E-06 9.00943 9.00943 5.0080119247 LOC101929681 yes 0.163918 0.0135996 1.83E-05 12.0531 12.0531 4.3661119288 LOC101929722 yes 0.080613 0.00441267 0.000349468 18.2685 18.2685 3.6237219311 LOC101929745 yes 0.415423 0.0491946 0.000433552 8.44449 8.44449 3.5647519371 LOC129026 yes 0.23786 0.0154773 2.81E-07 15.3683 15.3683 5.2743119377 LOC133083 yes 3.69745 0.0291597 0.000637221 126.8 126.8 3.4574719386 LOC139957 yes 1.79993 0.111868 8.31E-08 16.0898 16.0898 5.5180319392 LOC145837 yes 78.7443 14.3092 0.00225854 5.50305 5.50305 3.0846719417 LOC150568 yes 2.79076 0.270628 2.55E-11 10.3121 10.3121 6.9749919419 LOC150622 yes 15.95 0.319803 1.35E-05 49.8744 49.8744 4.4364719423 LOC151121 yes 9.0401 0.625407 0.000990763 14.4547 14.4547 3.3311819447 LOC158434 yes 7.99434 0.912833 0.00222613 8.75772 8.75772 3.0891219468 LOC221122 yes 0.358188 0.0735945 0.000227676 4.86705 4.86705 3.7387119500 LOC283194 yes 114.949 24.5554 0.000350739 4.68123 4.68123 3.6227319519 LOC283731 yes 14.1533 1.77914 6.90E-15 7.95511 7.95511 8.2737319520 LOC283804 yes 0.674909 0.0135035 3.45E-05 49.9803 49.9803 4.2149719550 LOC284661 yes 0.612329 0.0288676 4.65E-13 21.2117 21.2117 7.6247419555 LOC284788 yes 1.26678 0.292015 8.76E-05 4.33805 4.33805 3.9855119558 LOC284825 yes 15.3954 2.12103 9.36E-07 7.25847 7.25847 5.0257619566 LOC284998 yes 1.83422 0.120057 8.60E-08 15.2779 15.2779 5.5112419584 LOC285501 yes 37.34 3.36746 0.000309161 11.0885 11.0885 3.656919586 LOC285547 yes 0.183751 0.0327954 8.79E-05 5.60295 5.60295 3.9847519588 LOC285556 yes 54.4285 3.29099 3.03E-05 16.5386 16.5386 4.2461519604 LOC285768 yes 89.6461 13.5494 1.33E-05 6.61625 6.61625 4.440919608 LOC285889 yes 58.533 0.850257 1.21E-06 68.8416 68.8416 4.9718619613 LOC286177 yes 0.763033 0.0953937 0.000154015 7.99878 7.99878 3.8412219617 LOC286189 yes 29.0705 3.97489 9.93E-07 7.31354 7.31354 5.0132519621 LOC286297 yes 0.894637 0.13319 0.00118596 6.71698 6.71698 3.2786319628 LOC286456 yes 0.0931156 0.0216879 0.00497074 4.29345 4.29345 2.8332219642 LOC339298 yes 2.10685 0.476419 0.000276221 4.42227 4.42227 3.6871919650 LOC339568 yes 0.535722 0.0392112 0.000110473 13.6625 13.6625 3.926719671 LOC340268 yes 6.58663 1.27023 0.000525835 5.1854 5.1854 3.5113219694 LOC345571 yes 0.131269 0.0290192 0.00304016 4.52353 4.52353 2.9918819708 LOC359819 yes 4.51384 0.688351 5.74E-06 6.55746 6.55746 4.6320519728 LOC388456 yes 0.109462 0.00935349 0.000198233 11.7028 11.7028 3.7752819743 LOC389033 yes 7.16981 0.693146 0.000795093 10.3439 10.3439 3.394619747 LOC389249 yes 0.210771 0.0416621 3.88E-05 5.05905 5.05905 4.1865319763 LOC390029 yes 0.427287 0.0559755 0.000223774 7.63347 7.63347 3.7432919774 LOC390308 yes 0.062405 0.013296 0.0102462 4.69351 4.69351 2.5863519778 LOC390358 yes 27.6051 2.42514 3.70E-06 11.3829 11.3829 4.7293919803 LOC391020 yes 0.0702872 0.00839988 0.000145762 8.36764 8.36764 3.855519812 LOC391349 yes 0.0624283 0.00459438 0.000427937 13.588 13.588 3.5683419830 LOC391710 yes 0.111968 0.00601379 2.08E-06 18.6186 18.6186 4.8554319831 LOC391711 yes 3.34363 0.0385451 9.15E-05 86.7458 86.7458 3.9746619832 LOC391713 yes 0.653752 0.0347643 2.34E-06 18.8053 18.8053 4.8300719835 LOC391742 yes 0.336487 0.00774617 4.45E-05 43.4391 43.4391 4.1533419836 LOC391744 yes 7.31066 0.0306678 1.05E-06 238.382 238.382 5.0019519837 LOC391746 yes 0.35507 0.0211658 0.000110449 16.7756 16.7756 3.9267619838 LOC391747 yes 0.266683 0.0380704 0.00209367 7.00499 7.00499 3.1079819839 LOC391765 yes 11.1304 0.0580303 1.51E-06 191.803 191.803 4.924619840 LOC391768 yes 0.291409 0.00125597 1.31E-07 232.019 232.019 5.4277819843 LOC391813 yes 0.116781 0.00155491 3.46E-08 75.1044 75.1044 5.6880619855 LOC392268 yes 0.241843 0.00146305 1.14E-06 165.301 165.301 4.9843519905 LOC400682 yes 4.51672 0.866205 2.32E-14 5.21438 5.21438 8.0898519914 LOC400794 yes 16.6692 0.939581 1.38E-19 17.7411 17.7411 9.830819917 LOC400940 yes 7.4914 0.0514718 1.81E-06 145.544 145.544 4.8851819922 LOC401098 yes 1.12735 5.44449 0.0108204 0.207062 -4.82946 -2.5670119936 LOC401437 yes 3.67281 0.278882 3.39E-07 13.1698 13.1698 5.2366619967 LOC402229 yes 0.391745 0.0367593 0.00409189 10.657 10.657 2.8968219977 LOC402715 yes 0.0712795 0.00402494 5.61E-05 17.7094 17.7094 4.0966919989 LOC440180 yes 0.547597 0.0370096 7.90E-05 14.7961 14.7961 4.0116219996 LOC440313 yes 0.72584 0.140656 0.000798754 5.16039 5.16039 3.3932820022 LOC440925 yes 62.1883 13.5463 3.10E-07 4.59081 4.59081 5.2546220026 LOC441025 yes 0.193906 0.0470189 0.000853671 4.12401 4.12401 3.3742120034 LOC441228 yes 1.51453 0.366565 3.39E-08 4.13168 4.13168 5.6922220049 LOC441511 yes 0.0599997 0.00999531 0.00622431 6.00278 6.00278 2.758320054 LOC441666 yes 60.1685 9.95471 1.83E-11 6.04422 6.04422 7.0297720059 LOC441750 yes 0.135953 0.00882443 0.000657023 15.4064 15.4064 3.4488320070 LOC441914 yes 1.59642 0.0698442 0.000577339 22.8569 22.8569 3.4852120082 LOC442087 yes 2.40371 0.368315 9.33E-06 6.52623 6.52623 4.5221520085 LOC442132 yes 1.88652 0.17786 0.000518964 10.6067 10.6067 3.5149820089 LOC442225 yes 0.0172417 0.00107682 0.00949276 16.0116 16.0116 2.6132620092 LOC442272 yes 0.135881 0.0295042 0.00113201 4.60548 4.60548 3.292320093 LOC442292 yes 1.63552 0.377331 0.00129191 4.33444 4.33444 3.2533920101 LOC442427 yes 0.104556 0.0114906 0.00173276 9.09921 9.09921 3.1655920102 LOC442443 yes 28.7562 1.84717 0.000983558 15.5677 15.5677 3.333320132 LOC574538 yes 0.171783 0.0395694 0.00222639 4.34131 4.34131 3.0890920163 LOC642474 yes 0.0577517 0.00163633 2.23E-05 35.2935 35.2935 4.3194920166 LOC642496 yes 3.57466 0.265589 0.00236142 13.4594 13.4594 3.070920170 LOC642554 yes 6.65369 0.579837 3.66E-06 11.4751 11.4751 4.7319820171 LOC642579 yes 0.493772 0.0830155 2.24E-05 5.94795 5.94795 4.3180420174 LOC642633 yes 1.1782 0.232971 0.000181125 5.05729 5.05729 3.7989720175 LOC642635 yes 6.35752 0.300823 3.18E-07 21.1338 21.1338 5.2494720195 LOC643014 yes 0.0375211 0.0010196 0.00154781 36.8 36.8 3.1995720215 LOC643355 yes 4.16578 17.3073 0.00810838 0.240695 -4.15463 -2.6681420226 LOC643486 yes 0.325843 0.0047025 7.61E-07 69.2916 69.2916 5.0693120273 LOC644189 yes 2.02187 0.475399 9.39E-06 4.253 4.253 4.5205720285 LOC644357 yes 0.0620297 0.00597418 0.00079805 10.383 10.383 3.3935420292 LOC644525 yes 0.950471 0.210098 1.45E-05 4.52394 4.52394 4.4203720293 LOC644554 yes 16.8706 2.57424 1.61E-08 6.55363 6.55363 5.834120302 LOC644669 yes 0.252842 0.0485834 0.000382924 5.20428 5.20428 3.5988120306 LOC644717 yes 0.572157 0.101514 0.00078625 5.63622 5.63622 3.397820309 LOC644754 yes 0.512764 0.0564532 3.21E-07 9.083 9.083 5.2477720310 LOC644762 yes 0.159459 0.0122283 1.02E-08 13.0402 13.0402 5.9188620323 LOC645108 yes 0.080613 0.0085951 0.00112778 9.37895 9.37895 3.293420332 LOC645188 yes 0.063191 0.00568842 0.00800573 11.1087 11.1087 2.6725420334 LOC645207 yes 0.21129 0.0228573 0.000993886 9.24387 9.24387 3.3302720341 LOC645319 yes 0.0446029 0.00597744 0.00489439 7.46187 7.46187 2.8383220344 LOC645354 yes 3.66939 0.430486 0.00693859 8.52383 8.52383 2.7215420347 LOC645359 yes 1.8016 0.172458 4.51E-08 10.4466 10.4466 5.6371520350 LOC645397 yes 0.0558224 0.0087009 0.0093218 6.41571 6.41571 2.6196320354 LOC645433 yes 1.75756 0.390852 0.00490815 4.49674 4.49674 2.837420364 LOC645598 yes 0.713301 0.0311622 0.000272779 22.8899 22.8899 3.6905520366 LOC645638 yes 82.0469 359.792 0.00977039 0.22804 -4.3852 -2.6031320368 LOC645749 yes 2.40065 0.0557459 4.04E-07 43.0641 43.0641 5.200420392 LOC646066 yes 0.464061 0.0268369 7.87E-05 17.2919 17.2919 4.0125520395 LOC646103 yes 0.844688 0.0627096 0.00199355 13.4698 13.4698 3.1229820402 LOC646193 yes 0.261463 0.0505419 6.84E-06 5.17318 5.17318 4.5924520415 LOC646358 yes 0.132361 0.0228379 0.000252318 5.79565 5.79565 3.7113920426 LOC646535 yes 0.161615 0.0318035 0.00970019 5.08167 5.08167 2.6056720427 LOC646548 yes 1.086 0.238595 3.12E-05 4.55164 4.55164 4.2394320429 LOC646588 yes 16.8058 1.17292 2.41E-07 14.3282 14.3282 5.30620442 LOC646696 yes 7.30527 0.853644 1.71E-11 8.55775 8.55775 7.0411220446 LOC646709 yes 2.8229 0.4207 1.49E-07 6.71 6.71 5.4019320491 LOC647264 yes 0.0677482 0.00452505 1.12E-05 14.9718 14.9718 4.4795820500 LOC648442 yes 0.0421227 0.00488646 0.00380419 8.62028 8.62028 2.9203720501 LOC648691 yes 0.205737 0.0173675 9.00E-06 11.8461 11.8461 4.5303120510 LOC649324 yes 2.06268 0.0782195 4.04E-07 26.3703 26.3703 5.2002520515 LOC650181 yes 0.118737 0.0146724 0.00828627 8.09253 8.09253 2.6606420548 LOC653924 yes 11.2417 1.71989 9.00E-07 6.53627 6.53627 5.0340220557 LOC724065 yes 0.385073 0.0953923 0.00918969 4.03674 4.03674 2.6246320576 LOC727944 yes 0.146634 0.0183647 0.000102596 7.98457 7.98457 3.9455320582 LOC727982 yes 35.6287 1.06593 1.85E-05 33.4251 33.4251 4.3639120589 LOC728056 yes 0.057494 0.0044084 0.00127318 13.0419 13.0419 3.2577120600 LOC728307 yes 0.213881 0.0143429 0.000876169 14.912 14.912 3.3667320617 LOC728485 yes 11.1559 2.17403 5.17E-14 5.13144 5.13144 7.9669420620 LOC728503 yes 29.9758 3.4717 3.90E-10 8.63431 8.63431 6.5088220634 LOC728602 yes 2.38516 0.176827 0.00354236 13.4887 13.4887 2.9432620635 LOC728606 yes 6.02475 1.16959 6.51E-06 5.15117 5.15117 4.6037920639 LOC728667 yes 0.0522614 0.000993692 5.25E-06 52.5932 52.5932 4.6519420640 LOC728675 yes 0.109462 0.0147986 0.00165255 7.39676 7.39676 3.179920649 LOC728755 yes 64.946 7.30349 2.79E-09 8.89247 8.89247 6.1581320654 LOC728811 yes 16.392 0.939545 1.16E-07 17.4467 17.4467 5.45220657 LOC728819 yes 0.69815 0.170827 2.36E-05 4.08688 4.08688 4.3057420663 LOC728947 yes 0.581822 0.107444 2.13E-05 5.4151 5.4151 4.3308420695 LOC729316 yes 1.11597 0.13278 6.56E-09 8.40467 8.40467 6.0015520701 LOC729458 yes 10.9707 0.07491 5.40E-06 146.452 146.452 4.6458120726 LOC729800 yes 0.302977 0.0297917 0.00211126 10.1698 10.1698 3.1054220741 LOC729960 yes 48.4622 0.462635 0.000583712 104.753 104.753 3.4821320757 LOC730129 yes 0.112214 0.00877195 3.03E-05 12.7924 12.7924 4.2463620778 LOC731631 yes 7.53042 0.496417 1.23E-07 15.1696 15.1696 5.4410920797 LOC791098 yes 0.060888 0.0050868 0.000920039 11.9698 11.9698 3.3526320798 LOC791099 yes 0.169444 0.00461071 2.28E-06 36.7501 36.7501 4.8356620802 LOC84931 yes 14.9091 0.532626 8.39E-22 27.9916 27.9916 10.52620820 LONRF2 yes 255.472 44.8342 2.57E-08 5.69814 5.69814 5.7453620826 LOXHD1 yes 12.271 2.30865 1.17E-06 5.3152 5.3152 4.979520854 LPO yes 34.3069 4.63604 9.95E-07 7.40005 7.40005 5.0128920857 LPPR1 yes 60.4662 7.40859 1.72E-11 8.16163 8.16163 7.0404520859 LPPR3 yes 43.0263 2.1446 3.56E-09 20.0626 20.0626 6.1138120866 LRCH2 yes 107.524 22.5177 1.72E-08 4.77507 4.77507 5.8211220890 LRP2 yes 192.616 32.4574 0.00709714 5.93442 5.93442 2.7138520893 LRP4 yes 1899.84 225.16 9.40E-21 8.43774 8.43774 10.199120894 LRP4-AS1 yes 230.433 33.6146 6.42E-21 6.85515 6.85515 10.25120947 LRRC3B yes 29.3023 1.17022 3.32E-09 25.0399 25.0399 6.1266720962 LRRC52 yes 3.91987 0.728303 0.00271969 5.38219 5.38219 3.0269220971 LRRC66 yes 63.4405 14.2443 2.08E-05 4.45374 4.45374 4.3359320999 LRRTM1 yes 93.696 1.4885 5.26E-07 62.9468 62.9468 5.1461121001 LRRTM3 yes 7.37758 0.10696 0.000496874 68.9754 68.9754 3.5270721004 LRTM1 yes 0.857634 0.0827405 0.00850791 10.3653 10.3653 2.6514921005 LRTM2 yes 1.74696 0.0238619 1.06E-05 73.211 73.211 4.4924421034 LSP1P1 yes 0.0705557 0.0110481 0.00239784 6.38621 6.38621 3.0661621069 LY6D yes 1250.53 13194.6 0.000621785 0.0947757 -10.5512 -3.4643821074 LY6G6D yes 363.162 2.18212 5.49E-13 166.426 166.426 7.5983921075 LY6G6E yes 7.95003 0.438122 2.55E-17 18.1457 18.1457 9.0959521076 LY6G6F yes 41.5079 1.68225 4.40E-10 24.674 24.674 6.4876221077 LY6H yes 17.1633 4.23174 0.00410768 4.05585 4.05585 2.8955721095 LYPD5 yes 83.3909 500.888 0.000615979 0.166486 -6.0065 -3.4670221098 LYPD8 yes 77.6115 0.0964784 0.000396499 804.444 804.444 3.5892821101 LYPLA1P2 yes 0.104505 0.0161051 0.000458776 6.48896 6.48896 3.5491621120 LYZL2 yes 2.65843 0.337085 4.54E-14 7.88652 7.88652 7.9872221164 MAGEA12 yes 153.727 16.2326 1.99E-05 9.47022 9.47022 4.3463321166 MAGEA2 yes 298.017 21.3556 8.74E-07 13.955 13.955 5.0401121167 MAGEA2B yes 4.47158 0.590955 3.16E-09 7.56671 7.56671 6.1356321173 MAGEA8 yes 104.574 0.87776 0.00026727 119.137 119.137 3.6960121177 MAGEB1 yes 2.52704 0.156724 5.21E-06 16.1241 16.1241 4.6534921180 MAGEB17 yes 41.1958 2.51909 0.000456307 16.3534 16.3534 3.5506521186 MAGEB6 yes 2.48776 0.256167 4.29E-06 9.71146 9.71146 4.6968421187 MAGEB6P1 yes 0.168568 0.00438926 0.000261913 38.4048 38.4048 3.7014321190 MAGEC3 yes 4.96549 0.577746 4.89E-10 8.59459 8.59459 6.4693121196 MAGEE2 yes 1.46106 0.177857 4.75E-08 8.2148 8.2148 5.6272721199 MAGEL2 yes 28.7549 5.67112 8.15E-06 5.0704 5.0704 4.5528521245 MANSC4 yes 15.7883 2.25933 3.87E-05 6.98806 6.98806 4.187421297 MAP9 yes 273.017 62.881 6.01E-13 4.3418 4.3418 7.5839321309 MAPK6PS1 yes 0.731624 0.109653 8.31E-06 6.67219 6.67219 4.5484821342 11-Mar yes 7.59513 0.40823 2.98E-07 18.605 18.605 5.262421392 MAT1A yes 243.755 38.7238 0.000126889 6.29472 6.29472 3.8912421413 MBD3L2 yes 3.51923 0.0425378 1.53E-05 82.7319 82.7319 4.4078721414 MBD3L3 yes 6.87705 0.040091 2.35E-06 171.536 171.536 4.8286621415 MBD3L4 yes 0.344925 0.01998 0.000169841 17.2635 17.2635 3.8157721416 MBD3L5 yes 0.896757 0.0215199 3.99E-06 41.671 41.671 4.7129721441 MC4R yes 3.09616 0.293894 2.76E-05 10.5349 10.5349 4.2691221448 MCCD1 yes 1.73649 0.161506 1.99E-06 10.7519 10.7519 4.8652921458 MCHR1 yes 17.2601 3.74325 0.000101674 4.611 4.611 3.9478321519 MED14P1 yes 0.0608711 0.0096955 0.00397001 6.27829 6.27829 2.906621521 MED15P1 yes 1.97028 0.0426941 0.00015537 46.1489 46.1489 3.8389521523 MED15P5 yes 3.25895 0.116913 2.26E-12 27.8749 27.8749 7.372621524 MED15P6 yes 78.7993 1.20012 0.000127253 65.6595 65.6595 3.8905121525 MED15P7 yes 48.4642 0.917389 0.000268129 52.8284 52.8284 3.6951521564 MEG8 yes 2.68712 0.531911 1.25E-05 5.05182 5.05182 4.454921571 MEI1 yes 92.3648 750.98 0.00261711 0.122992 -8.13059 -3.0389421590 MEP1A yes 202.344 20.6713 0.00760934 9.78865 9.78865 2.6900121637 METTL24 yes 63.3209 7.92277 6.31E-10 7.99228 7.99228 6.4242421650 MEX3A yes 1345.04 215.794 1.67E-29 6.23296 6.23296 12.831321658 MFAP3L yes 1305.25 113.19 0.000281766 11.5315 11.5315 3.6818621730 MIAT yes 2581.03 281.034 0.000261932 9.18404 9.18404 3.7014121748 MICU3 yes 141.536 12.4209 6.09E-19 11.395 11.395 9.6242221853 MIR1257 yes 0.326619 0.0369375 1.84E-05 8.84248 8.84248 4.3649321936 MIR1324 yes 0.107614 0.0058887 5.30E-06 18.2746 18.2746 4.6498422018 MIR192 yes 2.10607 0.371934 0.00318957 5.66247 5.66247 2.9766922022 MIR194-2 yes 0.333064 0.0204879 0.000189073 16.2567 16.2567 3.7877122136 MIR3118-2 yes 0.385218 0.0854746 0.000300158 4.50682 4.50682 3.6648622266 MIR345 yes 0.0833376 0.0166749 0.00920583 4.99779 4.99779 2.6240222427 MIR3975 yes 0.0842548 0.00438145 0.00224659 19.2299 19.2299 3.086322455 MIR4267 yes 0.0421275 0.00284998 0.00540747 14.7817 14.7817 2.8053522593 MIR4478 yes 1.09613 0.0344323 2.51E-10 31.8344 31.8344 6.5853323049 MIR550A2 yes 0.0284903 0.00152824 0.00606554 18.6426 18.6426 2.7669923206 MIR656 yes 0.0754486 0.00467943 0.000815346 16.1235 16.1235 3.387423225 MIR7-3HG yes 3.2693 0.366037 0.000776525 8.93161 8.93161 3.4013523328 MKRN3 yes 82.4886 16.7778 2.31E-07 4.91652 4.91652 5.3146723329 MKRN4P yes 3.10518 0.518333 0.000722849 5.9907 5.9907 3.4217723334 MKRN9P yes 4.58441 0.157899 4.91E-08 29.0338 29.0338 5.6205123364 MLXIPL yes 324.725 69.6067 0.000889377 4.66514 4.66514 3.3624223382 MMP16 yes 59.1963 9.80129 4.14E-14 6.03965 6.03965 8.0011123393 MMP26 yes 92.0304 0.093427 0.000266652 985.052 985.052 3.6966323395 MMP28 yes 53.4324 1036.9 0.00155088 0.0515309 -19.4058 -3.1989823423 MOBP yes 28.7817 4.8413 0.00533895 5.94503 5.94503 2.8095823432 MOGAT1 yes 7.33312 1.80613 4.59E-06 4.06012 4.06012 4.6818223434 MOGAT3 yes 67.8358 5.20435 0.000308496 13.0344 13.0344 3.6574823440 MORC1 yes 5.58073 0.957923 7.11E-05 5.82586 5.82586 4.0378423462 MOS yes 0.114889 0.00188551 0.000983169 60.9325 60.9325 3.3334223485 MPO yes 11.8336 2.81101 0.000367492 4.20974 4.20974 3.6100323495 MPPED1 yes 44.67 8.22619 0.00985668 5.43022 5.43022 2.6000323619 MRPL49P2 yes 0.0516387 0.00139645 8.39E-05 36.9786 36.9786 3.9962623728 MRS2P1 yes 0.375658 0.0499047 0.00215579 7.52752 7.52752 3.09923734 MS4A10 yes 1.21444 0.264939 0.00264394 4.58383 4.58383 3.0357523736 MS4A13 yes 1.71975 0.39773 7.63E-05 4.3239 4.3239 4.0202823763 MSI1 yes 575.35 73.3803 5.52E-19 7.84067 7.84067 9.638123791 MSX1 yes 1044.02 165.276 2.60E-05 6.31685 6.31685 4.2827423792 MSX2 yes 1174.5 62.4928 1.61E-21 18.7941 18.7941 10.43823794 MT1A yes 85.2685 7.20429 1.88E-08 11.8358 11.8358 5.8043323795 MT1B yes 1.84215 0.145773 1.53E-09 12.6371 12.6371 6.2670723814 MT4 yes 317.855 1.51833 1.30E-07 209.346 209.346 5.4290323825 MTCO1P1 yes 0.306059 0.0674168 0.000362306 4.5398 4.5398 3.613923857 MTHFD2P1 yes 33.0477 0.350142 1.98E-06 94.3836 94.3836 4.8655623888 MTND1P14 yes 2.19899 0.0735397 0.000371725 29.9021 29.9021 3.6069123890 MTND1P16 yes 0.0213091 0.00116462 0.00639319 18.297 18.297 2.7492823900 MTND1P25 yes 1.15173 0.0939051 0.000440491 12.2648 12.2648 3.5603823902 MTND1P27 yes 4.18188 0.904371 2.95E-07 4.62407 4.62407 5.2649323912 MTND1P4 yes 0.0995718 0.00476439 2.99E-05 20.8992 20.8992 4.2499423919 MTND2P10 yes 0.105319 0.00310354 0.00128983 33.935 33.935 3.2538723949 MTND2P8 yes 0.0604733 0.00794252 0.00597151 7.61387 7.61387 2.7722323950 MTND2P9 yes 0.0553761 0.00542144 0.000982581 10.2143 10.2143 3.3335923951 MTND3P1 yes 0.0789067 0.00389678 0.000177742 20.2492 20.2492 3.803923968 MTND4P16 yes 0.0180043 0.0008657 0.00452961 20.7974 20.7974 2.8637323985 MTND4P31 yes 0.0940659 0.0157629 0.000742529 5.96756 5.96756 3.4141223999 MTND5P16 yes 0.063191 0.00464807 0.00451855 13.5951 13.5951 2.8645324005 MTND5P21 yes 0.0768912 0.00871834 0.000213194 8.81947 8.81947 3.756124013 MTND5P29 yes 0.047264 0.00294454 0.0044574 16.0514 16.0514 2.8689924051 MTTP yes 16.0009 3.29153 1.54E-06 4.86123 4.86123 4.9205324053 MTUS2 yes 122.543 23.0735 5.16E-07 5.31097 5.31097 5.1500224065 MUC17 yes 400.424 24.2999 0.00382595 16.4785 16.4785 2.9185324072 MUC4 yes 719.146 10226.1 0.0109181 0.0703248 -14.2197 -2.5638124076 MUC7 yes 2.84115 0.500518 2.90E-07 5.67642 5.67642 5.2684424083 MURC yes 73.3801 15.8285 2.54E-06 4.63594 4.63594 4.8118624119 MYCBP2-AS1 yes 0.0370669 0.0028233 0.00808389 13.1289 13.1289 2.6691924125 MYCN yes 480.348 69.4478 3.02E-08 6.91667 6.91667 5.7140624129 MYEF2 yes 462.741 95.4403 7.77E-10 4.84849 4.84849 6.3876324133 MYF6 yes 1.98044 0.206393 0.000121772 9.59545 9.59545 3.9018124146 MYH7B yes 1882.36 77.2064 3.13E-09 24.3809 24.3809 6.1376424150 MYL10 yes 1.83901 0.454728 4.90E-05 4.0442 4.0442 4.1298824154 MYL3 yes 9.37736 1.51246 1.67E-08 6.20006 6.20006 5.8273924170 MYLK3 yes 43.3603 8.2401 0.000585689 5.26211 5.26211 3.4811824191 MYO3A yes 62.0032 341.937 0.000148921 0.181329 -5.51483 -3.8499424192 MYO3B yes 434.192 49.188 6.46E-12 8.8272 8.8272 7.2016524206 MYOD1 yes 3.18171 0.236952 3.47E-07 13.4276 13.4276 5.2317324215 MYOZ3 yes 44.4354 6.35405 1.92E-11 6.99325 6.99325 7.0221824308 NAP1L2 yes 38.3461 6.22472 3.33E-09 6.1603 6.1603 6.1263324315 NAP1L6 yes 3.84865 0.306627 0.000286223 12.5515 12.5515 3.6776524324 NAPSA yes 1908.8 33.8907 0.000499509 56.3221 56.3221 3.525624340 NAT16 yes 5.40374 0.710822 9.52E-12 7.60211 7.60211 7.1380324343 NAT8 yes 8.03278 1.57891 0.00850034 5.08756 5.08756 2.651824345 NAT8L yes 521.09 23.3534 5.09E-32 22.3132 22.3132 13.561724388 NCALD yes 983.611 230.576 2.93E-10 4.26588 4.26588 6.5589724404 NCCRP1 yes 116.061 1680.85 0.00232984 0.0690488 -14.4825 -3.0750624465 NDST4 yes 9.97896 2.10095 8.38E-05 4.74973 4.74973 3.996624543 NDUFS5P5 yes 0.121776 0.0216004 0.00306342 5.63766 5.63766 2.9894724583 NEK2P1 yes 1.35269 0.282174 9.45E-08 4.79383 4.79383 5.4926124602 NELL2 yes 1191.53 224.076 1.78E-07 5.31755 5.31755 5.3672224620 NEUROD1 yes 29.8263 2.20503 0.00490214 13.5265 13.5265 2.837824622 NEUROD4 yes 0.547125 0.06391 0.00485873 8.56086 8.56086 2.8407324623 NEUROD6 yes 0.0662433 0.00538057 0.000411377 12.3116 12.3116 3.5791824624 NEUROG1 yes 11.6343 0.667935 3.92E-05 17.4184 17.4184 4.1844724625 NEUROG2 yes 60.9213 4.08784 3.25E-09 14.9031 14.9031 6.1307624626 NEUROG3 yes 1.12002 0.186308 0.00179404 6.01167 6.01167 3.1550724633 NF1P4 yes 0.77622 0.0811666 0.00054577 9.56329 9.56329 3.5009424635 NF1P6 yes 2.18656 0.0428527 0.000233272 51.0249 51.0249 3.7322724636 NF1P7 yes 0.823695 0.0857897 0.000122791 9.60132 9.60132 3.8996724647 NFE2 yes 205.386 32.1895 7.47E-12 6.38054 6.38054 7.1778424675 NFYAP1 yes 0.413814 0.0666108 0.00615092 6.21242 6.21242 2.7622924686 NHEG1 yes 18.2027 0.6017 1.36E-05 30.2521 30.2521 4.43524734 NKAIN1P1 yes 0.119628 0.0190482 0.00327591 6.28028 6.28028 2.9682124736 NKAIN3 yes 68.3829 12.4608 2.94E-08 5.48785 5.48785 5.719824741 NKD1 yes 3372.61 48.8886 2.94E-10 68.9858 68.9858 6.5581624742 NKD2 yes 697.48 160.803 1.37E-07 4.33747 4.33747 5.4196424750 NKX2-1 yes 450.052 7.53455 9.92E-07 59.7318 59.7318 5.0135524751 NKX2-1-AS1 yes 14.8237 1.06027 2.31E-07 13.9811 13.9811 5.3141524755 NKX2-5 yes 173.644 26.6888 0.00194954 6.50624 6.50624 3.129824760 NKX6-1 yes 8.19413 1.27178 2.59E-08 6.44302 6.44302 5.7435524761 NKX6-2 yes 2.99194 0.142774 1.96E-08 20.9557 20.9557 5.7969924762 NKX6-3 yes 11.5101 0.82723 0.000116622 13.914 13.914 3.9128724796 NMD3P1 yes 0.849222 0.199594 8.52E-05 4.25475 4.25475 3.9925424800 NME1P1 yes 0.0889772 0.00617586 2.92E-06 14.4073 14.4073 4.7818524837 NOG yes 60.4977 10.542 0.000445175 5.7387 5.7387 3.5574724880 NOTUM yes 5484.9 46.6471 1.28E-09 117.583 117.583 6.298824882 NOVA1 yes 69.2403 13.6289 2.81E-09 5.08039 5.08039 6.1570324884 NOX1 yes 292.35 35.8154 2.41E-05 8.1627 8.1627 4.3012724885 NOX3 yes 51.0673 0.361861 9.62E-08 141.124 141.124 5.4891624905 NPBWR2 yes 0.0653025 0.00792487 0.000459705 8.2402 8.2402 3.548624960 NPM1P28 yes 0.0762311 0.0121278 0.00113744 6.28565 6.28565 3.290925004 NPSR1 yes 215.065 28.5452 0.00039491 7.53417 7.53417 3.5903825008 NPTX1 yes 504.544 32.3491 6.24E-08 15.5968 15.5968 5.5739725011 NPVF yes 0.14103 0.00727094 4.75E-07 19.3964 19.3964 5.1671125015 NPY2R yes 4.25057 0.0989958 4.78E-08 42.9369 42.9369 5.6260325022 NR0B2 yes 15.6751 3.47833 0.0061855 4.5065 4.5065 2.7604125048 NR5A2 yes 512.102 49.699 0.000108025 10.3041 10.3041 3.9324225051 NRAP yes 149.044 9.02985 1.10E-05 16.5057 16.5057 4.484425062 NRCAM yes 1217.61 151.85 6.78E-16 8.01852 8.01852 8.6193825086 NRSN1 yes 23.2374 0.568045 9.72E-07 40.9077 40.9077 5.0177825091 NRXN3 yes 329.579 37.9267 6.99E-11 8.68989 8.68989 6.8048725189 NUDT19P1 yes 1.55136 0.286253 1.52E-13 5.41953 5.41953 7.8001325275 NXPE1 yes 96.2282 0.638705 0.000280436 150.662 150.662 3.6831325276 NXPE2 yes 14.9112 0.647352 5.29E-06 23.0342 23.0342 4.6501425278 NXPE4 yes 126.047 1.1161 0.000631564 112.935 112.935 3.4599825281 NXPH3 yes 214.874 42.2094 1.01E-08 5.09066 5.09066 5.9219825296 OASL yes 174.863 779.136 0.00584521 0.224431 -4.4557 -2.7793925303 OBP2A yes 24.0459 1.25927 9.60E-05 19.0952 19.0952 3.962325304 OBP2B yes 12.2914 1.31128 0.000533682 9.37353 9.37353 3.5071925307 OC90 yes 3.02053 0.22107 6.16E-06 13.6633 13.6633 4.6161525320 ODC1 yes 14316.4 2269.63 2.18E-14 6.3078 6.3078 8.0991525351 OGDHL yes 543.629 57.5275 6.66E-21 9.4499 9.4499 10.246125360 OGN yes 155.789 15.0982 0.00266161 10.3184 10.3184 3.0336725371 OLFM3 yes 33.4955 1.6694 6.60E-07 20.0644 20.0644 5.0990425397 OPN1LW yes 0.619906 0.104579 0.000305035 5.92763 5.92763 3.6605225405 OPRK1 yes 118.19 25.5053 0.0111441 4.63394 4.63394 2.5565125413 OR10A5 yes 0.0370669 0.0029682 0.00879257 12.488 12.488 2.6400525416 OR10AA1P yes 0.039068 0.00275204 0.000474058 14.196 14.196 3.540125434 OR10G3 yes 0.0599825 0.0025882 9.25E-05 23.1754 23.1754 3.9716525464 OR10S1 yes 0.0284298 0.00259314 0.00256813 10.9635 10.9635 3.0448325481 OR11H13P yes 0.848321 0.187364 9.21E-05 4.52767 4.52767 3.9727925507 OR13C3 yes 0.0258452 0.00109059 0.00331584 23.6983 23.6983 2.9643525525 OR13Z2P yes 0.35405 0.0417157 5.53E-10 8.48722 8.48722 6.4475825565 OR1N1 yes 0.518137 0.0605308 3.11E-05 8.55989 8.55989 4.2403625572 OR1X1P yes 0.29551 0.0511796 5.88E-05 5.77398 5.77398 4.0851725655 OR2S1P yes 1.09465 0.158083 4.74E-05 6.92452 6.92452 4.1378925661 OR2T2 yes 0.0383051 0.00303203 0.00106321 12.6335 12.6335 3.3106425741 OR4C6 yes 0.80209 0.103518 0.00240859 7.74832 7.74832 3.0647725744 OR4D1 yes 0.049023 0.00662278 0.00314896 7.40219 7.40219 2.9807525753 OR4D9 yes 0.063191 0.00333518 0.00300733 18.9468 18.9468 2.9953125755 OR4E2 yes 0.0187252 0.000843529 0.00385656 22.1986 22.1986 2.9159625798 OR4N1P yes 0.342126 0.00261628 0.000733949 130.768 130.768 3.4174325803 OR4P1P yes 0.213972 0.00231277 1.03E-05 92.5176 92.5176 4.5004225817 OR4X1 yes 0.0842548 0.00338857 0.00186939 24.8644 24.8644 3.1425825823 OR51A3P yes 0.039068 0.00285577 0.000611341 13.6804 13.6804 3.4691525840 OR51E1 yes 39.2628 9.60935 2.92E-09 4.0859 4.0859 6.1499825850 OR51H2P yes 0.0390614 0.00105715 1.43E-05 36.9496 36.9496 4.4243625867 OR52B1P yes 0.0370669 0.00186741 0.00511339 19.8494 19.8494 2.8238825877 OR52E4 yes 0.102696 0.00190065 1.09E-07 54.032 54.032 5.465425879 OR52E6 yes 0.418787 0.0218527 0.000152426 19.1641 19.1641 3.8439125880 OR52E7P yes 0.019358 0.00121278 0.00958469 15.9617 15.9617 2.6098825895 OR52M2P yes 0.0392938 0.0029913 0.000867301 13.136 13.136 3.3696625903 OR52Q1P yes 0.0388843 0.00389043 0.00180014 9.99487 9.99487 3.1540425944 OR5AU1 yes 0.501791 0.0353335 1.83E-08 14.2016 14.2016 5.8099125958 OR5BC1P yes 0.0421275 0.00108804 0.00145396 38.7186 38.7186 3.2182825986 OR5G3 yes 0.204318 0.0329708 0.000316061 6.19694 6.19694 3.6509425988 OR5G5P yes 0.166126 0.00853912 1.02E-05 19.4547 19.4547 4.5007126030 OR5T2 yes 0.0336097 0.00183365 0.000607595 18.3294 18.3294 3.4708726063 OR6K2 yes 0.04384 0.00311553 0.000531595 14.0714 14.0714 3.5082826064 OR6K3 yes 0.154865 0.00743181 0.00164981 20.8381 20.8381 3.180426109 OR7E108P yes 3.24375 0.303497 4.56E-13 10.6879 10.6879 7.6275526115 OR7E116P yes 2.26657 0.188632 1.64E-14 12.0158 12.0158 8.1428426118 OR7E121P yes 0.627944 0.0280813 0.000288793 22.3617 22.3617 3.6752526139 OR7E15P yes 0.199289 0.034308 0.0034688 5.80881 5.80881 2.9499726150 OR7E24 yes 2.27762 0.0501788 0.000426619 45.3902 45.3902 3.5691926162 OR7E39P yes 1.57036 0.178846 1.30E-10 8.78051 8.78051 6.6996926164 OR7E43P yes 0.0451634 0.00716813 0.00773368 6.30059 6.30059 2.6844426171 OR7E5P yes 0.456887 0.0307156 4.55E-07 14.8747 14.8747 5.1759726175 OR7E83P yes 0.244492 0.0495095 0.000333212 4.93829 4.93829 3.6366426177 OR7E85P yes 0.733064 0.0963987 9.33E-06 7.6045 7.6045 4.5221126183 OR7E91P yes 40.2823 6.086 2.69E-07 6.61885 6.61885 5.2833326190 OR7G15P yes 0.16851 0.00669342 0.00142977 25.1755 25.1755 3.2232826198 OR8A1 yes 0.0451634 0.00525182 0.00194463 8.59956 8.59956 3.1305726215 OR8D4 yes 0.101496 0.00188551 0.00011885 53.8296 53.8296 3.9080326264 OR9Q2 yes 0.0213091 0.0008657 0.00305181 24.6149 24.6149 2.9906726322 OSTCP6 yes 0.0462707 0.00186184 0.00299011 24.8522 24.8522 2.9971226333 OTOG yes 32.8084 2.30616 3.49E-08 14.2264 14.2264 5.6866526335 OTOL1 yes 21.7121 2.00448 0.000583509 10.8318 10.8318 3.4822326339 OTOR yes 11.5814 0.0879922 1.86E-06 131.618 131.618 4.8797926348 OTUD6A yes 0.110455 0.0270109 0.010716 4.08927 4.08927 2.5704626353 OTX2 yes 162.131 7.54126 0.000211834 21.4992 21.4992 3.7577926354 OTX2-AS1 yes 38.2328 1.9468 5.94E-08 19.6388 19.6388 5.5835726371 OXGR1 yes 191.113 27.5618 1.57E-07 6.93398 6.93398 5.3922726394 P2RY14 yes 27.3808 4.42404 0.000188967 6.1891 6.1891 3.7878626415 PABPC1L2A yes 0.687844 0.0305043 0.000330607 22.5491 22.5491 3.6387726416 PABPC1L2B yes 0.61106 0.0319672 3.59E-05 19.1152 19.1152 4.2054926420 PABPC1P12 yes 0.0210637 0.00108804 0.0054678 19.3593 19.3593 2.8016626422 PABPC1P2 yes 0.134537 0.025947 0.00193874 5.18508 5.18508 3.1314926428 PABPC1P8 yes 0.185083 0.0068544 0.00133154 27.0021 27.0021 3.2444426429 PABPC1P9 yes 0.0622553 0.00868809 0.00284478 7.16559 7.16559 3.0128126441 PACSIN1 yes 265.507 44.0705 1.30E-10 6.0246 6.0246 6.6993626449 PAEP yes 1667.97 34.4644 0.000499527 48.3968 48.3968 3.5255926453 PAFAH1B1P2 yes 0.0829983 0.00969987 9.26E-05 8.55664 8.55664 3.9714926461 PAGE3 yes 0.0377851 0.0030579 0.00181908 12.3566 12.3566 3.1508726462 PAGE4 yes 14.4905 0.891135 7.36E-06 16.2607 16.2607 4.5760226465 PAH yes 98.961 4.32382 3.69E-05 22.8874 22.8874 4.1991726468 PAICSP2 yes 0.228458 0.0257352 0.000877302 8.87725 8.87725 3.3663626484 PAK7 yes 28.1762 5.78972 5.79E-05 4.8666 4.8666 4.0888626576 PAX2 yes 503.818 20.3967 8.55E-08 24.7009 24.7009 5.5124526577 PAX3 yes 14.9586 3.65463 0.00171212 4.09306 4.09306 3.1692126578 PAX4 yes 4.41459 0.103536 1.86E-05 42.6382 42.6382 4.3627326619 PCDH10 yes 58.1375 5.12607 4.48E-08 11.3415 11.3415 5.6383726625 PCDH18 yes 777.856 89.8239 7.32E-11 8.65979 8.65979 6.7970926631 PCDH9 yes 122.912 15.6084 3.25E-09 7.87477 7.87477 6.1305426644 PCDHA6 yes 69.008 11.6727 4.46E-06 5.9119 5.9119 4.6883426663 PCDHB2 yes 65.4845 8.68775 5.38E-13 7.53756 7.53756 7.6014326685 PCDHGB1 yes 198.183 39.2903 5.62E-09 5.04407 5.04407 6.0300326697 PCDP1 yes 218.85 46.7159 0.00234494 4.6847 4.6847 3.0730726712 PCK1 yes 642.56 19.503 1.82E-06 32.9467 32.9467 4.8847126743 PCP4 yes 351.472 41.6802 1.72E-05 8.4326 8.4326 4.3810226746 PCSK1N yes 340.977 40.418 2.88E-13 8.43626 8.43626 7.7001626757 PCYT1B yes 83.0442 16.8922 1.28E-08 4.91613 4.91613 5.8774826789 PDE1C yes 50.3522 9.94487 1.18E-08 5.06313 5.06313 5.8925326834 PDK4 yes 775.489 148.087 0.00117655 5.2367 5.2367 3.2809726860 PDYN yes 5.33875 0.125387 0.000597896 42.5781 42.5781 3.475426863 PDZD3 yes 71.1738 13.1461 0.00810895 5.41406 5.41406 2.6681226885 PEG10 yes 3110.78 616.702 5.14E-09 5.04421 5.04421 6.0465626886 PEG3 yes 68.2118 6.04068 2.60E-05 11.2921 11.2921 4.2828826967 PGAM1P4 yes 1.80183 0.163089 2.02E-08 11.0481 11.0481 5.7910126997 PGBD4P8 yes 0.0388843 0.00390252 0.00191074 9.96391 9.96391 3.1359227007 PGGT1BP2 yes 0.394569 0.039241 0.00185669 10.055 10.055 3.1446527022 PGM5-AS1 yes 11.9508 1.93608 0.000887715 6.17267 6.17267 3.3629627027 PGPEP1L yes 127.51 12.0638 0.00186594 10.5697 10.5697 3.1431427028 PGR yes 1452.95 149.669 2.94E-05 9.70775 9.70775 4.2534527057 PHEX yes 153.439 26.1806 0.0010487 5.86081 5.86081 3.3146427089 PHGR1 yes 161.795 6.52626 2.77E-05 24.7913 24.7913 4.2680227121 PHYH yes 1897.43 341.988 4.39E-06 5.54825 5.54825 4.6915227208 PIN1P1 yes 0.0620254 0.00392093 0.000367748 15.8191 15.8191 3.6098427266 PKHD1L1 yes 478.18 46.5997 0.000151515 10.2614 10.2614 3.8454727282 PKNOX2 yes 184.723 15.9175 1.10E-09 11.605 11.605 6.3255727294 PLA2G12B yes 10.9263 1.05095 1.93E-05 10.3966 10.3966 4.3538827301 PLA2G2E yes 0.318156 0.0763239 0.00466053 4.16849 4.16849 2.8544127307 PLA2G4D yes 774.194 65.5452 7.56E-08 11.8116 11.8116 5.5364827316 PLAC1L yes 0.270777 0.0500402 0.00136022 5.41118 5.41118 3.2381227383 PLEKHH1 yes 913.248 224.491 6.99E-05 4.06808 4.06808 4.0420627433 PLXNA4 yes 454.683 33.8903 5.52E-10 13.4163 13.4163 6.447827455 PMP2 yes 63.52 2.4395 0.000719089 26.0382 26.0382 3.4232527480 PNLIPRP1 yes 5.16805 0.572542 3.88E-07 9.0265 9.0265 5.2088427485 PNMA3 yes 55.2182 12.8221 0.000232997 4.30648 4.30648 3.7325827496 PNOC yes 105.724 14.7742 8.73E-12 7.15599 7.15599 7.1523427504 PNPLA5 yes 13.9521 0.633183 1.60E-11 22.0348 22.0348 7.0523327572 POLR2KP2 yes 0.0832346 0.0031675 0.000284192 26.2777 26.2777 3.6795627612 PON1 yes 44.9424 5.80004 1.86E-05 7.74863 7.74863 4.362327630 POTEE yes 24.5862 3.183 0.000387302 7.72424 7.72424 3.595727632 POTEG yes 10.0105 0.822354 9.26E-05 12.173 12.173 3.9715527633 POTEH yes 7.01006 0.684697 4.35E-05 10.2382 10.2382 4.1590127634 POTEI yes 2.90874 0.67309 0.000707988 4.32147 4.32147 3.4276727635 POTEJ yes 19.3415 0.642735 0.000345886 30.0925 30.0925 3.6265227637 POTEM yes 87.6494 2.49718 1.52E-05 35.0993 35.0993 4.4098127644 POU3F2 yes 335.026 5.97303 3.02E-08 56.0897 56.0897 5.7142327645 POU3F3 yes 74.0117 0.843666 3.56E-10 87.7263 87.7263 6.5246527646 POU3F4 yes 0.370625 0.0181193 6.15E-06 20.4547 20.4547 4.6163627651 POU5F1B yes 44.4729 6.90361 6.51E-13 6.44199 6.44199 7.5713427660 POU6F2 yes 61.4453 6.0719 2.61E-10 10.1196 10.1196 6.5791127667 PP2672 yes 0.547999 0.0920935 6.17E-05 5.95047 5.95047 4.0732627684 PPARGC1A yes 642.026 152.637 0.00294994 4.20624 4.20624 3.0013927716 PPIAP14 yes 1.37368 0.166902 0.00038026 8.23045 8.23045 3.6007127730 PPIAP27 yes 0.179778 0.0125692 5.98E-07 14.3031 14.3031 5.1196527734 PPIAP30 yes 0.0235391 0.00152475 0.0106345 15.438 15.438 2.5731727775 PPM1N yes 20.7555 89.8469 0.00224678 0.23101 -4.32882 -3.0862827801 PPP1R14D yes 147.765 26.9278 0.000319516 5.48745 5.48745 3.64827806 PPP1R17 yes 3.53137 0.253953 3.43E-05 13.9056 13.9056 4.216627808 PPP1R1A yes 72.4543 17.8383 1.21E-05 4.06174 4.06174 4.4631327810 PPP1R1AP2 yes 0.0497252 0.00171182 0.00221101 29.0482 29.0482 3.0912227847 PPP1R9A yes 442.573 106.218 2.90E-10 4.16667 4.16667 6.5607727896 PPY2 yes 3.47677 0.587028 0.00844965 5.92267 5.92267 2.6538727907 PRAME yes 2987.9 208.556 1.59E-19 14.3266 14.3266 9.8110627908 PRAMEF1 yes 2.0807 0.087979 5.18E-06 23.65 23.65 4.6549527909 PRAMEF10 yes 0.383084 0.0434271 4.72E-05 8.82132 8.82132 4.1388627910 PRAMEF11 yes 1.72888 0.143315 6.78E-07 12.0635 12.0635 5.0933227911 PRAMEF12 yes 6.83659 0.292546 6.04E-05 23.3693 23.3693 4.0782727912 PRAMEF13 yes 1.31152 0.0872813 4.11E-05 15.0264 15.0264 4.1727327913 PRAMEF14 yes 0.299196 0.0228154 0.000888635 13.1138 13.1138 3.3626627917 PRAMEF18 yes 0.270658 0.0148055 0.000801172 18.2809 18.2809 3.3924227918 PRAMEF19 yes 0.134524 0.0187958 0.0015515 7.15713 7.15713 3.1988627919 PRAMEF2 yes 2.09332 0.104993 0.00013276 19.9377 19.9377 3.8796127921 PRAMEF21 yes 0.170302 0.0154359 5.27E-06 11.0329 11.0329 4.651127922 PRAMEF22 yes 0.126382 0.0132884 0.0049774 9.51072 9.51072 2.8327827923 PRAMEF23 yes 0.189127 0.0204644 6.04E-05 9.24178 9.24178 4.0785127926 PRAMEF25 yes 0.695428 0.0405199 8.59E-08 17.1626 17.1626 5.5114327929 PRAMEF5 yes 2.33262 0.169526 3.40E-10 13.7597 13.7597 6.5326127930 PRAMEF6 yes 0.936267 0.16486 0.00341495 5.67918 5.67918 2.9549727931 PRAMEF7 yes 2.92582 0.280948 0.00210041 10.4141 10.4141 3.10727934 PRAP1 yes 392.54 48.5704 0.000123441 8.08188 8.08188 3.8983127938 PRB4 yes 0.910078 0.0961921 1.45E-05 9.46105 9.46105 4.4209827986 PREX2 yes 189.193 26.4974 2.42E-11 7.14005 7.14005 6.9834828060 PRLR yes 723.713 160.43 0.000101217 4.5111 4.5111 3.9489728063 PRM3 yes 0.157049 0.0174357 0.000275019 9.00729 9.00729 3.6883628085 PROKR2 yes 1.53841 0.220484 5.08E-06 6.97745 6.97745 4.6590928097 PROX1 yes 437.895 29.8832 4.70E-12 14.6536 14.6536 7.2535628098 PROX1-AS1 yes 28.7151 4.82418 6.65E-08 5.95232 5.95232 5.5615728117 PRPH yes 403.009 20.4151 0.00063032 19.7407 19.7407 3.4605428120 PRPS1L1 yes 0.13685 0.0116088 0.00410773 11.7885 11.7885 2.8955628122 PRPS1P2 yes 0.0657891 0.0120767 0.00664667 5.44762 5.44762 2.7361328140 PRR20E yes 0.131501 0.0121954 0.00143966 10.7828 10.7828 3.2212328144 PRR23B yes 0.105047 0.0141913 0.000329399 7.40216 7.40216 3.6397628156 PRR9 yes 1057.48 25.4724 4.94E-06 41.5146 41.5146 4.6656428184 PRSS35 yes 37.1705 3.99364 1.22E-12 9.30742 9.30742 7.4714628186 PRSS37 yes 10.7193 0.668955 0.000275686 16.0239 16.0239 3.6877128207 PRTG yes 85.4274 12.5592 1.34E-10 6.802 6.802 6.6943928208 PRTN3 yes 4.27415 0.949214 0.00578224 4.50283 4.50283 2.7830228226 PSD yes 238.439 57.2439 8.54E-10 4.16533 4.16533 6.3707328262 PSMA6P3 yes 0.0462707 0.00389765 0.00640416 11.8715 11.8715 2.748728274 PSMB3P2 yes 0.411294 0.0916861 0.000787206 4.4859 4.4859 3.3974528349 PSPC1P2 yes 0.252613 0.0577352 0.000200182 4.37536 4.37536 3.7727128368 PTCH1 yes 1078.91 249.301 3.85E-09 4.32775 4.32775 6.0995128387 PTGDR2 yes 130.49 5.28099 0.000589387 24.7094 24.7094 3.4794228412 PTH yes 3.19729 0.517867 0.000372524 6.17397 6.17397 3.6063228469 PTPN5 yes 52.8477 2.50665 0.000134007 21.083 21.083 3.877228477 PTPRD yes 688.014 88.1642 9.43E-15 7.80378 7.80378 8.2265428488 PTPRO yes 419.037 47.6287 9.62E-08 8.798 8.798 5.4891228569 PZP yes 24.7379 6.13911 0.00067586 4.02955 4.02955 3.4408428597 R3HDML yes 101.287 4.5363 5.24E-06 22.3281 22.3281 4.6521728627 RAB27B yes 222.067 1037.37 3.82E-07 0.214067 -4.67144 -5.2120328645 RAB3B yes 447.669 81.7293 0.00077524 5.47745 5.47745 3.4018328646 RAB3C yes 17.7397 3.93889 1.83E-08 4.50374 4.50374 5.8093828744 RAET1E yes 23.3239 129.239 0.00310945 0.180472 -5.54104 -2.9847528754 RAG2 yes 0.150281 0.0257073 0.000296811 5.84583 5.84583 3.6678828769 RALYL yes 73.7441 6.76764 1.70E-06 10.8966 10.8966 4.8993628779 RANBP20P yes 0.425308 0.0888811 0.000122196 4.78513 4.78513 3.9009128820 RARRES2P1 yes 1.19032 0.286997 0.00321918 4.14751 4.14751 2.9737628852 RASL10B yes 172.685 27.5719 1.73E-23 6.26307 6.26307 11.043128854 RASL11B yes 641.312 62.9891 9.40E-10 10.1813 10.1813 6.3537128870 RAX2 yes 4.57981 0.106567 2.26E-06 42.9759 42.9759 4.8375628920 RBM22P3 yes 0.528165 0.0745456 0.000194727 7.08513 7.08513 3.7799728954 RBM8B yes 16.7338 3.3891 0.000665722 4.93754 4.93754 3.4451129008 RBP2 yes 29.4922 4.86068 7.03E-06 6.06752 6.06752 4.5863129053 RCOR2 yes 587.746 101.782 2.39E-14 5.77458 5.77458 8.0854929057 RD3 yes 6.6794 1.44388 6.82E-05 4.62601 4.62601 4.0481829070 RDXP2 yes 0.203288 0.0489008 8.14E-05 4.15715 4.15715 4.0041229082 REG1A yes 7524.57 252.743 0.00124042 29.7717 29.7717 3.2654129083 REG1B yes 270.83 2.45656 0.000730217 110.247 110.247 3.4188829085 REG3A yes 11120.2 0.971304 0.000652549 11448.8 11448.8 3.4507629086 REG3G yes 2.53264 0.216387 0.00553912 11.7042 11.7042 2.7973529112 RETNLB yes 19.0196 0.121334 0.000491844 156.754 156.754 3.529929137 RFESDP1 yes 2.13806 0.0768986 1.50E-09 27.8036 27.8036 6.2697829145 RFPL2 yes 7.9504 1.72704 2.42E-09 4.6035 4.6035 6.1837629148 RFPL4A yes 5.50156 0.534692 9.35E-05 10.2892 10.2892 3.9690529149 RFPL4AL1 yes 2.39819 0.162026 6.18E-07 14.8013 14.8013 5.1125629156 RFPL4B yes 9.60317 0.397127 5.44E-05 24.1816 24.1816 4.1040429167 RFX4 yes 17.873 2.89197 8.51E-06 6.18022 6.18022 4.5430729169 RFX6 yes 3.45906 0.498011 2.75E-07 6.94575 6.94575 5.2792429193 RGR yes 9.03012 0.320326 3.20E-06 28.1904 28.1904 4.7617229214 RGS7BP yes 54.2579 8.00052 3.52E-05 6.78179 6.78179 4.2104729280 RIMBP2 yes 285.169 70.3827 0.000278272 4.0517 4.0517 3.6852129287 RIMKLBP2 yes 0.0462707 0.00300743 0.00529396 15.3854 15.3854 2.8123929291 RIMS4 yes 162.545 14.1269 6.53E-11 11.506 11.506 6.8165229306 RIPPLY1 yes 8.43005 0.373946 0.000637548 22.5435 22.5435 3.4573229336 RN7SKP3 yes 0.0284903 0.00108804 0.00268561 26.185 26.185 3.0308629345 RN7SL8P yes 0.896818 0.0584507 4.25E-20 15.3431 15.3431 9.9928929644 RNA5SP470 yes 0.412784 0.0093381 0.000286209 44.2043 44.2043 3.6776629726 RNASE9 yes 0.149934 0.0260874 4.32E-05 5.74738 5.74738 4.1604629728 RNASEH1P1 yes 5.55237 0.542389 0.00186914 10.2369 10.2369 3.1426229729 RNASEH1P2 yes 0.306542 0.0198279 9.03E-10 15.4602 15.4602 6.3608229741 RND2 yes 297.7 43.9117 5.56E-22 6.7795 6.7795 10.581229792 RNF180 yes 166.392 23.7746 6.46E-11 6.99871 6.99871 6.8183429797 RNF186 yes 116.603 11.1531 0.000338718 10.4548 10.4548 3.632229869 RNU1-20P yes 0.059147 0.0112885 0.00699502 5.23955 5.23955 2.7187829878 RNU105C yes 0.146713 0.00778432 5.73E-05 18.8472 18.8472 4.0912729894 RNU4-7P yes 0.03817 0.00242556 0.00164751 15.7366 15.7366 3.1808129956 RNU6-67P yes 0.0187606 0.000956058 0.00527225 19.6228 19.6228 2.8137530050 RNY1P8 yes 0.0210637 0.00121966 0.00755477 17.2702 17.2702 2.6924830089 ROBO2 yes 410.296 98.1278 7.37E-12 4.18124 4.18124 7.1800930149 RPL10AP12 yes 0.149922 0.0158893 0.00128616 9.4354 9.4354 3.2547130165 RPL10P14 yes 0.12088 0.0151448 4.39E-05 7.98165 7.98165 4.1570230213 RPL12P37 yes 0.713185 0.136093 1.79E-05 5.24041 5.24041 4.3706730220 RPL12P43 yes 0.0235391 0.00108804 0.0041045 21.6344 21.6344 2.8958230242 RPL13AP21 yes 0.0842548 0.00781315 0.00704476 10.7837 10.7837 2.7163730252 RPL13AP8 yes 0.130711 0.0138076 0.000247567 9.4666 9.4666 3.7164530278 RPL15P15 yes 0.0344799 0.00157361 0.000202731 21.9113 21.9113 3.7693730296 RPL17P1 yes 0.0698431 0.00211251 7.21E-07 33.0616 33.0616 5.0804130311 RPL17P23 yes 0.263725 0.0409464 7.77E-09 6.44074 6.44074 5.9703230324 RPL17P35 yes 0.073199 0.00581482 0.0033051 12.5884 12.5884 2.9653830333 RPL17P43 yes 5.45697 1.21813 1.31E-06 4.4798 4.4798 4.9541530385 RPL19P15 yes 0.121073 0.0083139 0.000671846 14.5627 14.5627 3.4425230389 RPL19P19 yes 0.568271 0.122415 3.06E-09 4.64217 4.64217 6.1416230391 RPL19P20 yes 0.212226 0.0196992 3.47E-08 10.7733 10.7733 5.6878630436 RPL21P130 yes 0.155038 0.029103 6.71E-05 5.32723 5.32723 4.052130463 RPL21P33 yes 0.113272 0.0250847 0.00671669 4.51557 4.51557 2.7325830466 RPL21P36 yes 0.063191 0.00261508 0.00197236 24.1641 24.1641 3.1262530474 RPL21P43 yes 0.542867 0.0438525 0.000348474 12.3794 12.3794 3.6244930523 RPL21P88 yes 0.0676545 0.0129046 0.00473481 5.24268 5.24268 2.8492230549 RPL22P20 yes 0.047264 0.0044378 0.00988307 10.6503 10.6503 2.5990930579 RPL23AP28 yes 0.250724 0.00978281 0.000165777 25.629 25.629 3.8220930605 RPL23AP51 yes 0.121661 0.0224166 0.00242546 5.4273 5.4273 3.062630619 RPL23AP64 yes 0.0375211 0.00295665 0.0082171 12.6904 12.6904 2.6635430644 RPL23P3 yes 0.107517 0.756675 0.00763028 0.142091 -7.03774 -2.6890730659 RPL24P9 yes 0.0531149 0.00139645 3.47E-05 38.0356 38.0356 4.2136430682 RPL26P29 yes 0.0287222 0.0023012 0.00863868 12.4814 12.4814 2.6461930716 RPL27P6 yes 0.0576634 0.00778201 0.00104793 7.40983 7.40983 3.3148630749 RPL29P30 yes 0.440254 0.0992674 0.000926804 4.43503 4.43503 3.3505230771 RPL30P4 yes 0.201176 0.031725 0.00376562 6.34126 6.34126 2.9236530812 RPL31P40 yes 0.131333 0.0197037 0.00685966 6.66541 6.66541 2.7254330823 RPL31P50 yes 0.169429 0.0167998 0.000294107 10.0852 10.0852 3.6703430862 RPL32P28 yes 0.191598 0.0404754 0.000622173 4.73369 4.73369 3.464230863 RPL32P29 yes 0.137952 0.00906157 2.55E-06 15.2239 15.2239 4.8108430880 RPL34P1 yes 0.132 0.0257738 0.0017241 5.12148 5.12148 3.1671130906 RPL34P33 yes 0.429079 0.0966123 1.71E-05 4.44125 4.44125 4.3813930923 RPL35AP15 yes 0.0434461 0.00408905 0.00108353 10.625 10.625 3.3051130939 RPL35AP3 yes 0.149175 0.00340074 0.00105198 43.8655 43.8655 3.3137331027 RPL36P16 yes 0.208679 0.0256187 1.14E-05 8.14557 8.14557 4.4754231045 RPL37AP4 yes 0.0360086 0.0016691 0.00413295 21.5736 21.5736 2.8935831094 RPL39P20 yes 0.127568 0.0212272 0.000178052 6.00965 6.00965 3.8034431161 RPL5P18 yes 1.45333 0.243928 0.000156839 5.95802 5.95802 3.8365131162 RPL5P19 yes 0.0897596 0.008935 0.000838732 10.0458 10.0458 3.3792831190 RPL6P11 yes 0.195742 0.0267102 6.77E-07 7.32837 7.32837 5.0938231211 RPL6P30 yes 0.248953 0.0237158 5.64E-06 10.4973 10.4973 4.6359531212 RPL6P4 yes 0.165507 0.0317189 1.01E-05 5.21794 5.21794 4.5028231213 RPL6P5 yes 0.292213 0.00832029 0.000473431 35.1206 35.1206 3.5404731245 RPL7AP33 yes 1.36285 0.0388694 0.000706692 35.0622 35.0622 3.4281931287 RPL7AP73 yes 0.236815 0.0303698 1.25E-06 7.79772 7.79772 4.9644731297 RPL7L1P4 yes 0.063191 0.00268243 0.00209061 23.5574 23.5574 3.1084331316 RPL7P21 yes 0.0284903 0.0017057 0.0083452 16.703 16.703 2.6581831326 RPL7P30 yes 0.0200437 0.00106217 0.00586423 18.8705 18.8705 2.7783131356 RPL7P58 yes 0.0365709 0.00413141 0.00347614 8.85192 8.85192 2.9492931379 RPL9P20 yes 0.346412 0.0274078 2.56E-09 12.6392 12.6392 6.1740431385 RPL9P26 yes 0.121797 0.0100027 5.23E-05 12.1764 12.1764 4.1140231406 RPLP0P4 yes 3.49566 0.0380127 2.12E-06 91.9603 91.9603 4.8508731421 RPLP1P5 yes 0.0287222 0.00176142 0.00897613 16.3063 16.3063 2.6328531497 RPS12P15 yes 0.0789456 0.00473797 0.000433504 16.6623 16.6623 3.5647931550 RPS15AP15 yes 0.107668 0.0138231 0.000369339 7.78895 7.78895 3.6086731591 RPS15P8 yes 0.0768175 0.0181917 0.0100084 4.22267 4.22267 2.5946531592 RPS15P9 yes 0.879428 0.061468 2.15E-08 14.3071 14.3071 5.7793731600 RPS16P6 yes 0.12973 0.0238391 0.000984767 5.44192 5.44192 3.3329431602 RPS16P8 yes 0.0819515 0.00953321 0.00201317 8.59643 8.59643 3.1199931603 RPS16P9 yes 5.06491 0.51813 3.71E-11 9.77536 9.77536 6.9120931614 RPS17P17 yes 0.225377 0.00897871 1.61E-05 25.1013 25.1013 4.3960331618 RPS17P6 yes 0.279698 0.026054 2.44E-08 10.7353 10.7353 5.7553831643 RPS19P5 yes 0.289783 0.0478796 0.000112462 6.05232 6.05232 3.9221531652 RPS20P13 yes 0.17631 0.0352211 0.000432618 5.0058 5.0058 3.5653531691 RPS21P8 yes 0.0841452 0.00108804 2.79E-06 77.3364 77.3364 4.791931696 RPS23P3 yes 0.149102 0.0217764 0.00209364 6.84692 6.84692 3.1079931698 RPS23P5 yes 0.0619295 0.00417342 0.000153882 14.839 14.839 3.8414531719 RPS24P6 yes 0.0370669 0.00289752 0.00863845 12.7926 12.7926 2.646231786 RPS26P56 yes 0.210637 0.028822 0.00684816 7.3082 7.3082 2.72631830 RPS27P25 yes 4.05777 0.388023 5.04E-09 10.4576 10.4576 6.0503831833 RPS27P28 yes 0.583327 0.0691849 3.70E-11 8.43143 8.43143 6.9125631905 RPS2P31 yes 0.117335 0.011556 1.84E-06 10.1536 10.1536 4.8814831977 RPS3AP46 yes 1.10686 0.0997536 1.93E-09 11.096 11.096 6.2247632010 RPS4XP20 yes 0.105319 0.0159605 0.0105265 6.59872 6.59872 2.5767932018 RPS4XP8 yes 0.180124 0.0211461 0.000127174 8.51807 8.51807 3.8906732021 RPS4Y1P1 yes 0.148991 0.0253377 2.72E-05 5.88019 5.88019 4.2723432028 RPS5P5 yes 0.905144 0.161407 1.39E-05 5.60782 5.60782 4.4309732047 RPS6P12 yes 0.251033 0.0499517 0.000738444 5.02552 5.02552 3.415732050 RPS6P15 yes 0.063924 0.00724589 0.000210923 8.8221 8.8221 3.7589332064 RPS6P4 yes 0.23273 0.0471285 0.00215719 4.9382 4.9382 3.098832091 RPS8P5 yes 0.0351127 0.00277152 0.000977993 12.6691 12.6691 3.3349532122 RPSAP28 yes 0.101987 0.0157028 0.000722061 6.49484 6.49484 3.4220832128 RPSAP33 yes 0.154954 0.0135228 0.00250505 11.4587 11.4587 3.0525832216 RSL24D1P1 yes 0.184818 0.0436007 0.000281614 4.23887 4.23887 3.6820132221 RSL24D1P4 yes 0.0655296 0.00549852 4.31E-05 11.9177 11.9177 4.1614432235 RSPO2 yes 12.6494 2.43781 7.31E-14 5.18884 5.18884 7.9136732237 RSPO4 yes 101.432 14.6735 1.74E-06 6.91259 6.91259 4.8943632256 RTL1 yes 176.194 5.94076 4.18E-05 29.6585 29.6585 4.1684532300 RXFP4 yes 11.3214 1.75197 4.03E-11 6.46214 6.46214 6.8977832303 RXRG yes 107.131 2.4135 3.02E-16 44.3883 44.3883 8.7380132310 S100A1 yes 395.751 72.4061 0.000244204 5.46571 5.46571 3.720132322 S100A3 yes 1591.2 103.894 1.81E-06 15.3157 15.3157 4.8854232331 S100A8 yes 3773.69 47758.2 0.00512953 0.0790167 -12.6556 -2.8228432332 S100A9 yes 13640.6 63316.2 0.00519018 0.215437 -4.64174 -2.8189532334 S100G yes 1.06072 0.0247134 3.69E-07 42.921 42.921 5.2191832358 SALL1 yes 621.056 56.906 4.45E-16 10.9137 10.9137 8.6812432360 SALL2 yes 599.848 124.927 6.24E-14 4.80159 4.80159 7.9381532372 SAMD11 yes 336.507 62.2968 6.72E-07 5.40167 5.40167 5.095232385 SAMD9 yes 317.73 1654.11 3.61E-05 0.192085 -5.20604 -4.2041832420 SATB2-AS1 yes 13.7625 0.0563242 2.01E-06 244.345 244.345 4.8628432430 SBK1 yes 1387.43 342.46 3.53E-19 4.05136 4.05136 9.7002932435 SBSPON yes 578.634 85.3068 5.40E-09 6.78297 6.78297 6.0375932450 SCAND3 yes 82.1926 16.0609 6.45E-13 5.11755 5.11755 7.5727732465 SCARNA15 yes 0.0665715 0.00989715 0.00808116 6.72633 6.72633 2.669332490 SCG2 yes 66.5025 10.8907 7.71E-08 6.10633 6.10633 5.5326232491 SCG3 yes 31.7212 4.84095 4.52E-06 6.55269 6.55269 4.6852532498 SCGB1D4 yes 18.8597 0.349962 0.00038513 53.8908 53.8908 3.5972432499 SCGB1D5P yes 0.0388042 0.00195845 0.000366381 19.8138 19.8138 3.6108632506 SCGN yes 7.40502 1.04358 0.000522229 7.09576 7.09576 3.5132332520 SCN1A yes 145.67 12.7888 1.44E-05 11.3904 11.3904 4.4227832524 SCN3A yes 128.648 9.68525 0.000967757 13.2829 13.2829 3.33832525 SCN3B yes 158.157 25.2188 6.34E-07 6.27141 6.27141 5.1072132526 SCN4A yes 12.6851 2.21331 1.51E-05 5.73128 5.73128 4.4110232528 SCN5A yes 128.338 16.9965 1.67E-10 7.55081 7.55081 6.6556932529 SCN7A yes 30.0821 5.06247 0.00212263 5.94218 5.94218 3.1037732531 SCN9A yes 361.612 64.893 4.78E-08 5.57244 5.57244 5.6260832549 SCRT1 yes 6.77091 1.11725 8.62E-07 6.06033 6.06033 5.0431932550 SCRT2 yes 1.53337 0.204223 0.000295161 7.50828 7.50828 3.6693832552 SCTR yes 397.961 82.0682 0.00545002 4.84915 4.84915 2.8027432553 SCUBE1 yes 79.9941 18.6496 0.00317073 4.28932 4.28932 2.9785732567 SDC2 yes 2046.64 467.656 5.34E-13 4.37637 4.37637 7.6027432598 SDHDP1 yes 0.0462707 0.00423909 0.00858289 10.9152 10.9152 2.6484432607 SDPR yes 574.332 110.195 9.26E-06 5.21195 5.21195 4.5237332626 SEC14L5 yes 108.469 23.4167 5.24E-08 4.63211 4.63211 5.6079232668 SELV yes 10.6946 0.669872 5.32E-12 15.9651 15.9651 7.233432673 SEMA3E yes 281.15 36.1096 1.39E-08 7.78601 7.78601 5.8609732705 SEPHS1P5 yes 1.0192 0.212103 0.00929253 4.80523 4.80523 2.6207432717 14-Sep yes 11.2972 1.05832 1.04E-05 10.6747 10.6747 4.4974132719 SEPT2P1 yes 0.525317 0.0680401 0.000748919 7.7207 7.7207 3.4116832729 SEPT7P4 yes 0.156004 0.0377264 0.00171071 4.13514 4.13514 3.1694632730 SEPT7P5 yes 0.102355 0.0228795 0.00873878 4.47367 4.47367 2.6421832744 SERBP1P4 yes 0.180433 0.00416474 1.52E-09 43.3239 43.3239 6.2679532771 SERPINA6 yes 55.4721 8.07899 0.00115194 6.86622 6.86622 3.2871832772 SERPINA7 yes 1.61742 0.257044 0.00820871 6.29238 6.29238 2.6638932775 SERPINB10 yes 40.4898 8.13498 0.00831121 4.97725 4.97725 2.659632778 SERPINB13 yes 269.044 4221.28 0.00159154 0.0637353 -15.6899 -3.1912132780 SERPINB3 yes 1296.82 14592.8 0.00146983 0.0888668 -11.2528 -3.2150432781 SERPINB4 yes 175.687 5897.72 0.00161111 0.029789 -33.5695 -3.1875432782 SERPINB5 yes 1415.54 5917.47 0.00115051 0.239214 -4.18035 -3.2875432789 SERPIND1 yes 12.7484 0.881877 2.79E-06 14.456 14.456 4.7918932843 SETP7 yes 0.163449 0.00830885 0.0001075 19.6717 19.6717 3.9336632848 SEZ6L yes 568.016 3.0531 2.67E-11 186.046 186.046 6.9671332873 SFRP5 yes 64.9043 4.42164 2.26E-08 14.6788 14.6788 5.7698932880 SFTA3 yes 174.151 0.812802 1.64E-07 214.26 214.26 5.3831132884 SFTPB yes 376.321 5.77819 3.58E-07 65.1277 65.1277 5.2253732932 SH2D6 yes 15.1382 2.74167 0.000253402 5.52151 5.52151 3.7102432966 SHANK1 yes 106.513 17.3908 5.43E-06 6.1247 6.1247 4.6443432987 SHH yes 254.077 10.8365 1.17E-10 23.4465 23.4465 6.7172532989 SHISA3 yes 276.157 2.84014 0.000429673 97.2338 97.2338 3.5672332994 SHISA8 yes 67.9731 13.7624 0.000940862 4.93904 4.93904 3.3461633018 SIAH3 yes 13.8436 1.68496 6.39E-11 8.21594 8.21594 6.8202133027 SIGLEC15 yes 39.5066 5.17986 9.33E-10 7.62696 7.62696 6.3551933036 SIGLEC26P yes 0.0448483 0.00390489 0.000631232 11.4852 11.4852 3.4601333041 SIGLEC31P yes 0.0684647 0.00625627 0.00015602 10.9434 10.9434 3.8378733115 SLC10A2 yes 11.4192 0.220227 0.000540805 51.8521 51.8521 3.5034933117 SLC10A4 yes 78.1476 17.5655 0.000870922 4.44892 4.44892 3.3684633120 SLC10A6 yes 9.14403 138.82 0.00503296 0.0658697 -15.1815 -2.8291233124 SLC12A1 yes 21.2598 4.70422 0.00235352 4.5193 4.5193 3.0719433126 SLC12A3 yes 161.699 5.53385 9.94E-05 29.2199 29.2199 3.9535833139 SLC14A2 yes 52.8513 8.86813 9.99E-11 5.95969 5.95969 6.7442233148 SLC16A12 yes 261.243 14.1339 1.78E-13 18.4834 18.4834 7.775333162 SLC17A1 yes 3.87183 0.375892 4.47E-07 10.3004 10.3004 5.1794933165 SLC17A4 yes 48.1069 5.82347 0.00566762 8.26086 8.26086 2.789733168 SLC17A7 yes 114.898 27.7937 0.00164887 4.13397 4.13397 3.1805733169 SLC17A8 yes 3.47067 0.731848 3.05E-09 4.74233 4.74233 6.1423433171 SLC18A1 yes 7.46087 1.43683 1.10E-08 5.19261 5.19261 5.9057733173 SLC18A3 yes 2.38412 0.408807 0.00019022 5.8319 5.8319 3.7861233177 SLC19A3 yes 149.892 11.7788 2.99E-05 12.7255 12.7255 4.249733184 SLC1A7 yes 79.9208 17.2096 0.00417397 4.64396 4.64396 2.8903733192 SLC22A11 yes 12.7537 2.06211 8.91E-06 6.18482 6.18482 4.5325533197 SLC22A16 yes 22.1328 4.57449 8.01E-08 4.83831 4.83831 5.5252433222 SLC24A5 yes 147.218 30.2087 1.14E-09 4.87337 4.87337 6.3194433235 SLC25A15P5 yes 0.0644989 0.0102365 0.00270728 6.30086 6.30086 3.0283533242 SLC25A1P3 yes 0.163798 0.015517 1.39E-07 10.556 10.556 5.416433274 SLC25A3P1 yes 0.0600897 0.00545805 0.000191731 11.0094 11.0094 3.7840533286 SLC25A48 yes 122.237 15.2437 4.72E-05 8.01884 8.01884 4.1390733303 SLC25A5P9 yes 0.0465773 0.00476066 0.0055591 9.78379 9.78379 2.7961533321 SLC26A7 yes 221.468 12.9103 3.13E-11 17.1544 17.1544 6.9403133329 SLC27A6 yes 71.0313 5.61001 1.24E-14 12.6615 12.6615 8.1846533336 SLC29A4 yes 565.357 55.7868 1.29E-19 10.1342 10.1342 9.8400533337 SLC29A4P1 yes 0.225344 0.033957 6.87E-06 6.63617 6.63617 4.5916633355 SLC2A7 yes 4.90966 0.655971 0.000472953 7.48457 7.48457 3.5407533362 SLC30A3 yes 114.448 24.0437 4.08E-05 4.76 4.76 4.1747333372 SLC32A1 yes 6.6535 0.208007 4.55E-06 31.987 31.987 4.6839933390 SLC35D3 yes 37.4323 4.57291 1.13E-08 8.18568 8.18568 5.8997133421 SLC38A3 yes 81.3993 18.4099 6.72E-07 4.4215 4.4215 5.0953433422 SLC38A4 yes 177.775 29.5345 1.59E-06 6.01926 6.01926 4.9135233426 SLC38A8 yes 73.0477 9.72495 0.00374217 7.51137 7.51137 2.9256633437 SLC39A5 yes 304.811 34.9022 0.000602681 8.73328 8.73328 3.4731633467 SLC4A10 yes 24.326 4.42176 0.000328002 5.50144 5.50144 3.6409133480 SLC52A1 yes 59.3839 284.883 0.000853115 0.20845 -4.79731 -3.374433486 SLC5A12 yes 34.0097 8.42692 3.45E-05 4.03584 4.03584 4.2149733502 SLC6A13 yes 30.9514 4.62486 3.68E-08 6.6924 6.6924 5.6765533521 SLC7A10 yes 19.9797 3.09285 0.000360574 6.45996 6.45996 3.6152133522 SLC7A11 yes 1653.37 299.865 8.14E-08 5.51372 5.51372 5.5219533523 SLC7A11-AS1 yes 15.6928 2.40353 9.12E-09 6.52907 6.52907 5.9405733541 SLC8A3 yes 39.4944 7.05367 7.01E-05 5.59912 5.59912 4.0413633549 SLC9A3P4 yes 0.0925414 0.00513837 0.00218357 18.0099 18.0099 3.0950733552 SLC9A4 yes 87.6145 16.113 7.16E-05 5.43751 5.43751 4.0359733569 SLCO1A2 yes 65.0145 11.1758 8.72E-07 5.81742 5.81742 5.0406233594 SLIT3 yes 734.343 183.228 5.11E-10 4.00781 4.00781 6.461433596 SLITRK2 yes 39.4138 5.80916 0.000299015 6.78478 6.78478 3.6658933599 SLITRK5 yes 52.288 9.51817 4.92E-05 5.49349 5.49349 4.1291633646 SMARCE1P2 yes 0.294123 0.0228171 9.95E-10 12.8905 12.8905 6.3435933647 SMARCE1P3 yes 0.19858 0.0131633 4.75E-11 15.0858 15.0858 6.8702233705 SMLR1 yes 2.95909 0.73411 2.53E-08 4.03085 4.03085 5.7482933729 SMTNL2 yes 131.899 18.3879 2.01E-07 7.17316 7.17316 5.3427233740 SNAI1P1 yes 0.24502 0.0104888 2.30E-06 23.3601 23.3601 4.8336433773 SNAR-B2 yes 0.0497252 0.00313937 0.00469796 15.8392 15.8392 2.8517833938 SNORD113-3 yes 0.113619 0.0191095 0.000691376 5.94567 5.94567 3.4344133958 SNORD114-21 yes 0.0371671 0.00154923 0.0032269 23.9907 23.9907 2.97334023 SNORD115@ yes 7.46961 1.45 7.98E-07 5.15146 5.15146 5.0592434038 SNORD116-22 yes 0.0287222 0.00172984 0.00849619 16.604 16.604 2.6519734254 SNTB1 yes 1295.02 205.425 6.06E-21 6.30409 6.30409 10.258834285 SNX18P20 yes 11.2076 2.34658 0.00034233 4.77614 4.77614 3.6293234342 SOCS2P2 yes 0.157322 0.004103 5.44E-07 38.3431 38.3431 5.1393334360 SOGA2P1 yes 0.0459263 0.00512005 0.00141687 8.9699 8.9699 3.2259834363 SOHLH2 yes 9.58167 1.03574 0.00489294 9.25103 9.25103 2.8384234369 SORCS1 yes 77.6981 19.1513 0.00167508 4.05708 4.05708 3.1758134371 SORCS3 yes 42.4089 4.40543 1.15E-12 9.62652 9.62652 7.4803734390 SOX14 yes 46.4711 1.67791 3.00E-07 27.6958 27.6958 5.2614534392 SOX17 yes 2375.82 156.051 3.02E-09 15.2246 15.2246 6.1436434397 SOX3 yes 9.87098 0.223017 9.91E-07 44.2611 44.2611 5.0136534398 SOX30 yes 49.2348 243.763 2.55E-05 0.201978 -4.95104 -4.2878834402 SOX5P yes 0.0558769 0.00704084 0.00371474 7.93611 7.93611 2.9280234414 SP3P yes 43.6778 1.72508 9.48E-06 25.3194 25.3194 4.5184634416 SP5 yes 507.091 41.9101 5.45E-10 12.0995 12.0995 6.450234418 SP7 yes 22.8048 0.378177 8.47E-07 60.302 60.302 5.0468234419 SP8 yes 143.146 25.3377 0.000674821 5.64953 5.64953 3.4412734424 SPACA3 yes 3.76479 0.480644 8.99E-05 7.8328 7.8328 3.9790634430 SPAG11A yes 24.2052 0.36581 9.51E-05 66.1687 66.1687 3.9646734431 SPAG11B yes 9.84314 0.334626 8.41E-06 29.4154 29.4154 4.5457634447 SPANXC yes 6.19671 0.568628 0.00252491 10.8977 10.8977 3.0501234450 SPANXN2 yes 0.180152 0.0301715 0.00291321 5.97092 5.97092 3.0053334467 SPATA20P1 yes 0.108911 0.0141546 8.76E-05 7.69438 7.69438 3.9854534484 SPATA31C2 yes 2.73572 0.666016 0.0022093 4.10759 4.10759 3.0914634486 SPATA31D2P yes 0.063191 0.00293415 0.00415053 21.5364 21.5364 2.892234492 SPATA31E3P yes 0.0370669 0.00256672 0.00594343 14.4413 14.4413 2.7738134504 SPATA8-AS1 yes 1.94587 0.104736 0.00109445 18.5788 18.5788 3.3021834588 SPON1 yes 1568.68 249.276 2.35E-07 6.29295 6.29295 5.3112334593 SPP2 yes 3.83888 0.23873 0.000703564 16.0804 16.0804 3.4294534620 SPRY4 yes 1319.27 311.014 6.56E-15 4.24182 4.24182 8.2813434637 SPTLC1P2 yes 0.146318 0.0205515 1.11E-05 7.11955 7.11955 4.4825634647 SPZ1 yes 1.29863 0.0556362 2.65E-06 23.3414 23.3414 4.8027734659 SRD5A2 yes 30.6468 3.6741 6.16E-06 8.34132 8.34132 4.6159634742 SS18L2P2 yes 0.0258452 0.00143247 0.00665326 18.0424 18.0424 2.7357934759 SSR1P2 yes 0.105319 0.00561152 0.00240456 18.7683 18.7683 3.0652934776 SSU72P2 yes 4.79937 0.0566843 1.59E-06 84.6684 84.6684 4.9127434777 SSU72P3 yes 2.13975 0.0415527 1.04E-06 51.4948 51.4948 5.0039334778 SSU72P4 yes 3.23676 0.0202513 5.78E-06 159.83 159.83 4.6304734779 SSU72P5 yes 1.45724 0.022383 5.01E-06 65.1045 65.1045 4.6624534780 SSU72P6 yes 0.679057 0.0202674 8.30E-07 33.5048 33.5048 5.0511534781 SSU72P7 yes 7.68144 0.0700556 1.86E-06 109.648 109.648 4.8794434782 SSU72P8 yes 2.55296 0.0386331 2.63E-06 66.0823 66.0823 4.8047834790 SSX4B yes 46.0519 0.767958 0.000719266 59.9667 59.9667 3.4231834791 SSX5 yes 7.17489 0.105028 0.000170337 68.3139 68.3139 3.8150134844 ST6GAL2 yes 301.625 37.8155 2.78E-11 7.97622 7.97622 6.9603934860 ST8SIA2 yes 174.275 18.2397 1.76E-08 9.55471 9.55471 5.816834864 ST8SIA6 yes 106.346 23.2033 1.93E-08 4.58323 4.58323 5.834937 STK32A yes 71.8748 15.6825 9.88E-07 4.58312 4.58312 5.0144534938 STK32B yes 193.356 45.0968 6.37E-06 4.28759 4.28759 4.6085434940 STK33 yes 148.394 29.4264 4.73E-15 5.04288 5.04288 8.3305134954 STMN2 yes 272.395 25.7206 6.83E-06 10.5905 10.5905 4.5929535016 STXBP6 yes 954.606 133.922 1.41E-09 7.12808 7.12808 6.2810335053 SULT1C2P1 yes 4.44625 1.08061 1.21E-05 4.11459 4.11459 4.4620735054 SULT1C3 yes 2.0853 0.295647 2.57E-05 7.05334 7.05334 4.2860535055 SULT1C4 yes 74.7831 13.6304 2.33E-08 5.48651 5.48651 5.7635135118 SV2B yes 81.9218 15.1295 0.00323242 5.4147 5.4147 2.9724535124 SVOP yes 3.76193 0.835155 1.70E-11 4.50447 4.50447 7.0424335138 SYCP2 yes 207.587 1548.93 1.43E-05 0.13402 -7.4616 -4.4238135149 SYN2 yes 322.123 13.8791 1.83E-07 23.2092 23.2092 5.3610335180 SYPL2 yes 49.3479 9.79129 3.87E-05 5.03998 5.03998 4.1871935183 SYT1 yes 439.747 69.8975 5.50E-11 6.29132 6.29132 6.8456535197 SYT6 yes 31.0063 3.97444 8.63E-06 7.80142 7.80142 4.5398935223 TAC1 yes 193.128 2.711 5.02E-05 71.2385 71.2385 4.1239135224 TAC3 yes 22.4209 4.32999 2.57E-07 5.17806 5.17806 5.2924935262 TAF7L yes 16.7773 131.245 0.00490312 0.127832 -7.82276 -2.8377335302 TARDBPP2 yes 5.22105 0.87031 1.40E-06 5.99907 5.99907 4.9400735312 TAS2R1 yes 0.319252 0.056554 3.03E-06 5.64508 5.64508 4.7736535377 TBC1D26 yes 14.0234 2.44376 0.000150256 5.73847 5.73847 3.8476335432 TBX2 yes 492.016 116.268 3.85E-08 4.23175 4.23175 5.6675435438 TBX5 yes 14.7412 1.37985 0.000125223 10.6832 10.6832 3.8946435445 TCAM1P yes 57.5182 312.912 1.02E-05 0.183816 -5.44022 -4.5015235450 TCEA1P3 yes 0.463684 0.110486 0.00123656 4.19678 4.19678 3.2663335459 TCEAL5 yes 23.4497 3.08532 2.57E-06 7.60041 7.60041 4.8091535517 TCHH yes 1197.92 173.29 0.00127394 6.91277 6.91277 3.2575335518 TCHHL1 yes 213.983 4.21859 0.000465187 50.7238 50.7238 3.5453335522 TCL1B yes 2.84839 0.215854 1.44E-07 13.1959 13.1959 5.4087635523 TCL6 yes 24.043 2.9753 3.19E-12 8.08085 8.08085 7.3163135529 TCP10 yes 0.993038 0.211397 0.00344209 4.69751 4.69751 2.9524435550 TDGF1 yes 122.522 23.231 0.00448579 5.27409 5.27409 2.8669135557 TDGF1P7 yes 1.58776 0.277171 3.16E-10 5.72845 5.72845 6.5457535563 TDRD1 yes 105.767 16.8261 0.00212104 6.28591 6.28591 3.10435586 TECTB yes 2.02809 0.244726 0.000165104 8.28716 8.28716 3.8231535604 TENM1 yes 318.314 14.2424 6.20E-05 22.3497 22.3497 4.0721135655 TEX37 yes 0.820001 0.193993 0.00557785 4.22696 4.22696 2.7950335658 TEX41 yes 12.5096 2.53149 4.84E-05 4.94158 4.94158 4.1330935713 TGM1 yes 209.664 2519.93 0.00815826 0.0832021 -12.0189 -2.6660235719 TGM7 yes 24.4781 5.55853 0.00381245 4.4037 4.4037 2.9196735746 THEG5 yes 0.216404 0.011166 2.13E-05 19.3805 19.3805 4.3298535764 THPO yes 26.6133 6.37875 0.000431592 4.17218 4.17218 3.56635838 TISP43 yes 0.750426 0.162919 6.36E-06 4.60613 4.60613 4.608835882 TLX2 yes 10.9951 45.244 7.24E-05 0.243018 -4.11492 -4.0334835896 TM6SF2 yes 35.7608 7.55383 2.76E-09 4.73413 4.73413 6.1603735943 TMEFF1 yes 42.3738 3.08268 5.81E-11 13.7457 13.7457 6.8361535945 TMEM100 yes 267.086 27.0031 2.87E-05 9.89092 9.89092 4.2590635954 TMEM108 yes 94.5286 16.4753 1.18E-11 5.73759 5.73759 7.1024935978 TMEM132C yes 104.625 14.1917 9.97E-06 7.37225 7.37225 4.506936002 TMEM151B yes 39.0139 8.48908 0.000588254 4.59577 4.59577 3.4799636033 TMEM179 yes 8.06771 1.19309 0.00351215 6.76203 6.76203 2.94636067 TMEM200C yes 98.31 15.6139 6.21E-06 6.2963 6.2963 4.6143636092 TMEM225 yes 0.262182 0.0534518 0.000860091 4.90501 4.90501 3.3720636098 TMEM233 yes 31.2396 5.8592 9.70E-05 5.33172 5.33172 3.959836115 TMEM249 yes 37.5365 8.22597 3.37E-07 4.56317 4.56317 5.237836118 TMEM252 yes 54.8991 3.29396 6.39E-10 16.6666 16.6666 6.4221636126 TMEM257 yes 0.857224 0.0773616 4.73E-06 11.0807 11.0807 4.6750336158 TMEM52 yes 220.565 44.5045 2.77E-06 4.95602 4.95602 4.7932536168 TMEM59L yes 93.1609 11.3006 2.85E-08 8.24388 8.24388 5.7254836183 TMEM72 yes 22.4245 1.39574 6.76E-09 16.0664 16.0664 5.9961536184 TMEM72-AS1 yes 81.1848 19.9724 1.43E-13 4.06484 4.06484 7.8092336237 TMPRSS15 yes 119.463 1.28252 0.000309888 93.1476 93.1476 3.6562636288 TNFRSF11B yes 445.842 85.5099 0.000275782 5.21392 5.21392 3.6876236295 TNFRSF19 yes 1260.86 243.919 7.77E-10 5.16918 5.16918 6.3875936304 TNFSF10 yes 2199.66 9206.3 0.00596654 0.23893 -4.18532 -2.7725136326 TNMD yes 3.5717 0.566796 8.92E-06 6.30156 6.30156 4.5324136337 TNP1 yes 0.197171 0.0204111 0.000413625 9.66001 9.66001 3.5776936436 TPD52L3 yes 1.57072 0.0349108 0.000361157 44.9924 44.9924 3.6147736439 TPH1 yes 40.5759 2.56053 1.35E-08 15.8467 15.8467 5.8666636591 TRAM1L1 yes 56.2628 7.97825 1.71E-17 7.05202 7.05202 9.1536636700 TRBV22OR9-2 yes 0.0370669 0.0016691 0.00385281 22.2077 22.2077 2.9162736743 TRBVB yes 0.0370669 0.00148309 0.00295463 24.9931 24.9931 3.0008936762 TREML2 yes 65.1297 6.5774 4.52E-09 9.90204 9.90204 6.0701936793 TRHDE yes 107.756 16.0014 0.000405393 6.73413 6.73413 3.583236836 TRIM43 yes 24.5815 2.22847 3.10E-06 11.0306 11.0306 4.7688236837 TRIM43B yes 19.2692 1.18449 1.61E-05 16.268 16.268 4.3953636838 TRIM43CP yes 2.71816 0.13205 4.60E-06 20.5843 20.5843 4.6811736843 TRIM48 yes 14.4083 0.309991 4.81E-05 46.4798 46.4798 4.134736844 TRIM49 yes 29.2084 2.65655 0.000331073 10.9949 10.9949 3.6383936845 TRIM49B yes 19.1263 1.59282 0.00762795 12.0079 12.0079 2.6891736846 TRIM49C yes 30.1639 1.6879 4.27E-05 17.8707 17.8707 4.1635536847 TRIM49D1 yes 0.967593 0.11234 7.65E-06 8.61307 8.61307 4.5673136848 TRIM49D2P yes 20.3134 0.299424 5.15E-07 67.8415 67.8415 5.1505736851 TRIM51 yes 3.93643 0.0842228 0.000126039 46.7383 46.7383 3.8929736852 TRIM51BP yes 14.1171 1.43298 4.83E-05 9.85151 9.85151 4.1333936854 TRIM51DP yes 0.0422643 0.0038614 0.00302504 10.9454 10.9454 2.9934536856 TRIM51FP yes 15.364 0.134641 0.000608515 114.111 114.111 3.4704536858 TRIM51HP yes 0.0374503 0.00314739 0.0104472 11.8989 11.8989 2.5794736862 TRIM53AP yes 6.74309 0.313519 3.19E-05 21.5078 21.5078 4.2344236880 TRIM60P17 yes 0.774456 0.132142 7.78E-08 5.86077 5.86077 5.5309436882 TRIM60P19 yes 0.117696 0.00154917 0.000879007 75.9736 75.9736 3.365836892 TRIM61 yes 42.1071 7.56677 2.68E-06 5.56474 5.56474 4.8002836895 TRIM64 yes 0.286909 0.0191205 2.74E-08 15.0053 15.0053 5.7330636896 TRIM64B yes 3.30333 0.0771362 8.32E-08 42.8246 42.8246 5.5178636899 TRIM64EP yes 0.0871756 0.0165578 0.0102213 5.26491 5.26491 2.5872136907 TRIM71 yes 9.73474 0.992435 1.96E-05 9.80895 9.80895 4.3500536911 TRIM75 yes 0.478817 0.088074 0.000259065 5.43653 5.43653 3.7043536934 TRMT112P1 yes 0.100258 0.00766936 0.00014682 13.0725 13.0725 3.8536236936 TRMT112P4 yes 0.166333 0.0338052 0.00308558 4.92036 4.92036 2.9871936987 TRNAA9 yes 0.034348 0.0016675 0.000285872 20.5984 20.5984 3.6779836995 TRNAC16 yes 0.220565 0.0146402 0.00196492 15.0658 15.0658 3.127437061 TRNAF10 yes 0.106706 0.0218645 0.0076342 4.88032 4.88032 2.6888937148 TRNAK14 yes 0.0834378 0.0130781 0.00373382 6.37999 6.37999 2.9263737153 TRNAK2 yes 0.0497995 0.00394955 0.000493939 12.6089 12.6089 3.5287237180 TRNAL13 yes 0.0987476 0.0180567 0.000350325 5.46876 5.46876 3.6230537217 TRNAM12 yes 0.11789 0.0154108 3.39E-05 7.64985 7.64985 4.219237221 TRNAM16 yes 0.0377851 0.0016623 0.000255965 22.7307 22.7307 3.7075637234 TRNAN-GUU_2 yes 0.108272 0.0171789 2.19E-05 6.30259 6.30259 4.3239937319 TRNAQ51P yes 0.312338 0.0587554 9.74E-06 5.3159 5.3159 4.5121737331 TRNAR17 yes 0.0462707 0.00281099 0.00413165 16.4606 16.4606 2.8936837364 TRNAS16 yes 0.0248626 0.00140141 0.00698364 17.7411 17.7411 2.7193437385 TRNAS7 yes 0.128858 0.00440318 0.000200547 29.2647 29.2647 3.7722337432 TRNAV29 yes 0.0578871 0.306193 0.00736567 0.189055 -5.28948 -2.7011637470 TRO yes 327.189 70.5232 8.78E-11 4.63945 4.63945 6.7660737485 TRPM1 yes 21.6017 2.46844 0.000151472 8.75113 8.75113 3.8455437487 TRPM3 yes 107.466 17.0314 2.69E-11 6.30988 6.30988 6.9660237516 TSEN2P1 yes 0.105319 0.00171769 0.000979516 61.3142 61.3142 3.334537520 TSG1 yes 2.19737 0.461193 8.51E-12 4.76455 4.76455 7.1564937526 TSHR yes 77.9097 18.6675 6.56E-08 4.17354 4.17354 5.5642437548 TSPAN18 yes 176.667 39.9186 0.000281205 4.42567 4.42567 3.682437553 TSPAN32 yes 202.038 34.9708 4.40E-07 5.77733 5.77733 5.1828537559 TSPAN8 yes 2916.08 637.257 0.00258453 4.57599 4.57599 3.0428537564 TSPO2 yes 4.93214 0.943295 3.11E-06 5.22863 5.22863 4.7677437674 TTLL11-IT1 yes 0.0497995 0.000752654 3.76E-06 66.1652 66.1652 4.7262237687 TTPA yes 48.9431 8.90683 1.58E-07 5.495 5.495 5.3911437729 TTYH1 yes 148.806 34.2661 2.27E-05 4.34265 4.34265 4.3156337737 TUBA3D yes 393.656 0.225065 0.000602139 1749.08 1749.08 3.4734137738 TUBA3E yes 67.5349 0.0643675 0.000348312 1049.21 1049.21 3.6246237753 TUBB2B yes 2308.08 149.42 2.72E-08 15.4469 15.4469 5.7343537758 TUBB4A yes 81.9416 14.0737 7.27E-06 5.82232 5.82232 4.5787237767 TUBB8P1 yes 0.0200437 0.00108121 0.00616165 18.5381 18.5381 2.7617137771 TUBB8P4 yes 0.458776 0.0164107 1.51E-06 27.9559 27.9559 4.9240537772 TUBB8P5 yes 0.29416 0.00924182 6.62E-10 31.8293 31.8293 6.4158337773 TUBB8P6 yes 0.156408 0.016225 0.00113312 9.63993 9.63993 3.2920137783 TUBBP6 yes 0.838634 0.109724 1.99E-07 7.64313 7.64313 5.3444537814 TVP23BP1 yes 0.04161 0.00151193 5.38E-05 27.5212 27.5212 4.1069537824 TWIST1 yes 826.207 108.204 1.02E-06 7.63564 7.63564 5.0074537855 TYMP yes 1650.86 7156.68 1.31E-05 0.230674 -4.33511 -4.4446637858 TYRL yes 1.71063 0.0137865 0.00072407 124.08 124.08 3.4212937862 TYRP1 yes 66.0506 4.73828 6.31E-05 13.9398 13.9398 4.0676737887 UBA52P7 yes 0.0421275 0.00182467 0.00351767 23.0877 23.0877 2.945538028 UBTFL2 yes 0.0841894 0.0108993 0.00102045 7.7243 7.7243 3.322638029 UBTFL3 yes 0.288363 0.0709614 0.00764149 4.06366 4.06366 2.6885638030 UBTFL5 yes 0.969412 0.0982434 0.000466232 9.86745 9.86745 3.5447138045 UCHL1 yes 2674.62 573.096 1.45E-07 4.66697 4.66697 5.4082438053 UCMA yes 5.9567 0.34004 5.11E-07 17.5176 17.5176 5.1518938078 UGT1A12P yes 0.448111 0.00961205 1.33E-05 46.6198 46.6198 4.4410538080 UGT1A2P yes 0.0371671 0.00215268 0.00756075 17.2655 17.2655 2.6922138093 UGT2B11 yes 7.73933 0.629496 4.97E-06 12.2945 12.2945 4.6640138095 UGT2B17 yes 96.6695 13.541 0.00318469 7.13901 7.13901 2.9771738098 UGT2B26P yes 24.8891 1.64286 8.68E-05 15.1498 15.1498 3.9879738099 UGT2B27P yes 0.148268 0.00860618 0.00255127 17.228 17.228 3.0468838104 UGT3A1 yes 24.6601 3.11383 0.00105084 7.91954 7.91954 3.3140538105 UGT3A2 yes 18.7705 3.2711 4.46E-05 5.73828 5.73828 4.1531938139 UNC5C yes 249.529 42.6554 6.84E-12 5.84989 5.84989 7.1922738155 UNGP1 yes 0.444486 0.0923083 0.000214874 4.81524 4.81524 3.7540338195 UQCRFS1P3 yes 0.214609 0.0203044 1.61E-06 10.5696 10.5696 4.9110138203 URAD yes 4.2477 0.10614 8.21E-06 40.0198 40.0198 4.5511738224 USMG5P1 yes 0.200951 0.0380838 1.10E-05 5.27653 5.27653 4.4835438253 USP17L21 yes 0.0519906 0.00683393 0.00090665 7.60771 7.60771 3.3568738256 USP17L25 yes 0.10798 0.00942031 5.98E-06 11.4625 11.4625 4.6228338262 USP17L30 yes 0.082415 0.0102375 4.88E-05 8.05034 8.05034 4.1307738306 USP44 yes 98.205 22.0763 1.33E-05 4.44843 4.44843 4.4401538386 VAT1L yes 83.4175 10.4393 5.06E-05 7.99074 7.99074 4.1219138395 VCAN yes 4704.66 1069.82 5.37E-10 4.3976 4.3976 6.4527438403 VCY yes 0.221045 0.0487414 0.00132237 4.53506 4.53506 3.2464938428 VENTXP1 yes 0.0532923 0.00108804 1.23E-07 48.98 48.98 5.4401238430 VENTXP3 yes 0.538251 0.0622696 1.06E-07 8.64387 8.64387 5.4696638434 VENTXP7 yes 0.357141 0.0615167 0.00473353 5.80559 5.80559 2.8493138439 VGF yes 70.7583 13.4572 6.12E-11 5.25803 5.25803 6.8274538446 VIL1 yes 618.681 138.827 0.00990411 4.45649 4.45649 2.5983438498 VN1R29P yes 0.481871 0.00408979 0.000738081 117.823 117.823 3.4158438504 VN1R34P yes 0.130518 0.00551327 6.28E-09 23.6735 23.6735 6.0097238505 VN1R35P yes 0.161586 0.00469135 9.38E-06 34.4435 34.4435 4.520838538 VN1R67P yes 0.191309 0.0447127 0.000596913 4.27864 4.27864 3.4758638540 VN1R69P yes 0.099762 0.0069389 0.00061408 14.3772 14.3772 3.4678938542 VN1R70P yes 0.0406016 0.00380648 0.00930039 10.6664 10.6664 2.6204438545 VN1R74P yes 0.074588 0.0131814 0.00577996 5.65857 5.65857 2.7831538550 VN1R79P yes 0.0421275 0.00116229 0.00157843 36.2452 36.2452 3.1936938557 VN1R85P yes 0.507811 0.0937102 1.40E-05 5.41895 5.41895 4.4281338558 VN1R86P yes 0.0812033 0.0049146 0.0047267 16.5229 16.5229 2.8497838563 VN1R90P yes 0.130442 0.0156287 2.18E-06 8.3463 8.3463 4.8449238578 VN2R14P yes 0.105557 0.0211243 0.00132234 4.99695 4.99695 3.246538582 VN2R18P yes 0.202055 0.0427498 0.000277788 4.72647 4.72647 3.6856838599 VPREB1 yes 1.47463 0.00368918 0.000269439 399.718 399.718 3.6938538624 VPS37D yes 170.463 29.1685 1.97E-23 5.84407 5.84407 11.025938637 VPS9D1-AS1 yes 0.242995 0.0484298 0.000146924 5.01748 5.01748 3.8534438647 VSIG8 yes 1687.2 60.009 3.52E-06 28.1157 28.1157 4.7405638651 VSTM2B yes 2.5336 0.313898 9.78E-07 8.0714 8.0714 5.016638682 VWC2 yes 27.5571 5.20667 0.00109179 5.29266 5.29266 3.3028938722 WBSCR17 yes 99.3046 22.9795 0.00387032 4.32145 4.32145 2.9148138739 WDR17 yes 17.8996 3.87874 0.000349786 4.6148 4.6148 3.6234738798 WDR86 yes 244.376 54.9681 3.19E-12 4.44578 4.44578 7.3167838838 WIF1 yes 847.556 26.0341 2.60E-07 32.5556 32.5556 5.2902338857 WNT16 yes 74.1955 10.2349 0.000160403 7.24924 7.24924 3.8306738861 WNT3A yes 16.254 85.3229 0.00163014 0.1905 -5.24934 -3.18438875 WRBP1 yes 0.222232 0.0517982 4.57E-05 4.29034 4.29034 4.1468138882 WT1 yes 377.511 34.0126 1.91E-05 11.0992 11.0992 4.3563238883 WT1-AS yes 67.1924 7.14424 7.90E-05 9.40512 9.40512 4.0113938906 XAGE2 yes 72.1025 0.235546 4.85E-05 306.108 306.108 4.1325938907 XAGE3 yes 1.01504 0.11207 2.96E-05 9.05724 9.05724 4.2518338942 XPNPEP2 yes 56.0608 6.55493 0.000418099 8.55247 8.55247 3.5747339020 YPLR6490 yes 0.593889 0.0807412 0.00278595 7.35547 7.35547 3.0193739072 ZBED1P1 yes 0.510407 0.0997629 1.90E-05 5.1162 5.1162 4.3568339130 ZC2HC1B yes 6.76891 1.54325 2.84E-11 4.38613 4.38613 6.9569439134 ZC3H11B yes 10.9749 2.46658 2.06E-05 4.44944 4.44944 4.3380339152 ZC4H2 yes 170.242 27.0582 4.34E-21 6.29168 6.29168 10.304139155 ZCCHC12 yes 91.3808 10.6113 6.93E-06 8.61162 8.61162 4.5896539184 ZDHHC19 yes 44.1687 4.69434 9.88E-07 9.40892 9.40892 5.0144439254 ZFR2 yes 7.37042 265.015 1.30E-06 0.0278114 -35.9565 -4.956839278 ZIC1 yes 96.747 18.2982 0.000707263 5.28725 5.28725 3.4279639283 ZIK1 yes 72.837 16.6883 4.82E-10 4.36456 4.36456 6.4719239284 ZIK1P1 yes 0.0922717 0.00611374 2.62E-07 15.0925 15.0925 5.2885939335 ZNF135 yes 125.738 13.5739 7.40E-22 9.26321 9.26321 10.542939378 ZNF209P yes 0.470515 0.0201259 0.000202864 23.3786 23.3786 3.769239413 ZNF257 yes 40.8839 7.24304 1.41E-13 5.64458 5.64458 7.8117339430 ZNF280A yes 13.7128 0.497308 2.47E-13 27.5741 27.5741 7.7242439431 ZNF280B yes 35.4373 4.07917 2.72E-14 8.68739 8.68739 8.0653639457 ZNF317P1 yes 0.114751 0.0207017 0.00318467 5.54306 5.54306 2.9771839479 ZNF33AP1 yes 0.14171 0.0047972 1.06E-06 29.5402 29.5402 4.9987639525 ZNF418 yes 64.6995 8.44017 3.00E-17 7.66566 7.66566 9.0730539528 ZNF423 yes 256.359 63.5958 1.80E-07 4.03108 4.03108 5.3649939570 ZNF486 yes 184.463 31.1721 9.12E-08 5.91756 5.91756 5.4997539575 ZNF492 yes 29.2676 2.77387 4.05E-24 10.5512 10.5512 11.234639610 ZNF541 yes 21.8656 338.419 1.76E-05 0.064611 -15.4772 -4.3755139629 ZNF560 yes 20.0849 0.884797 0.000442184 22.7 22.7 3.5593239649 ZNF578 yes 51.7506 9.69409 8.00E-10 5.33837 5.33837 6.3824739653 ZNF582 yes 300.688 70.1008 4.17E-19 4.28937 4.28937 9.6771739654 ZNF582-AS1 yes 19.9081 4.58042 6.95E-15 4.34634 4.34634 8.2727439681 ZNF610 yes 84.506 11.5062 4.05E-22 7.34438 7.34438 10.623739696 ZNF626 yes 288.246 40.4506 2.77E-13 7.12587 7.12587 7.7064139706 ZNF645 yes 0.0967549 0.0241362 0.00565657 4.0087 4.0087 2.7903539718 ZNF66 yes 186.012 36.8016 2.32E-15 5.05446 5.05446 8.4370539719 ZNF660 yes 90.6919 13.4229 5.71E-26 6.75649 6.75649 11.790139726 ZNF667-AS1 yes 242.577 58.0931 9.93E-10 4.17566 4.17566 6.3439939736 ZNF676 yes 4.01841 0.915606 4.41E-05 4.3888 4.3888 4.1555539737 ZNF677 yes 116.875 23.3474 2.03E-15 5.0059 5.0059 8.4572139778 ZNF716 yes 3.10376 0.343092 0.000369529 9.04642 9.04642 3.6085339789 ZNF727 yes 65.3639 5.11461 3.12E-13 12.7798 12.7798 7.6876939792 ZNF730 yes 31.7965 2.73395 5.45E-26 11.6303 11.6303 11.796339795 ZNF733P yes 0.376036 0.0654505 2.44E-06 5.74535 5.74535 4.8205139810 ZNF737 yes 305.684 33.1548 4.17E-33 9.21989 9.21989 13.87539884 ZNF835 yes 23.5684 5.13462 4.02E-06 4.5901 4.5901 4.7113239896 ZNF849P yes 1.40918 0.150541 2.61E-11 9.36077 9.36077 6.971139932 ZNF968P yes 0.115987 0.0230048 0.00248338 5.04186 5.04186 3.0552839934 ZNF98 yes 14.7272 1.30103 1.84E-15 11.3196 11.3196 8.4715539967 ZSCAN1 yes 43.807 2.18122 3.77E-18 20.0837 20.0837 9.3681339968 ZSCAN10 yes 8.82952 1.24551 2.76E-07 7.08907 7.08907 5.278339973 ZSCAN18 yes 981.395 148.01 9.81E-27 6.63061 6.63061 12.017639991 ZSWIM2 yes 0.289281 0.0557328 0.000141074 5.1905 5.1905 3.863955 3.8-1.3 no 0.299017 0.702858 0.261633 0.42543 -2.35056 -1.124996 3.8-1.4 no 0.0845447 0.0825169 0.968839 1.02457 1.02457 0.03910197 3.8-1.5 no 0.0487659 0.11376 0.262991 0.428675 -2.33277 -1.121798 5-HT3C2 no 1.13286 0.6312 0.151667 1.79478 1.79478 1.437919 A1BG no 119.601 80.9321 0.056059 1.47779 1.47779 1.9191910 A1BG-AS1 no 32.7094 23.0933 0.0888268 1.4164 1.4164 1.7080412 A2M no 5826.06 3105.92 0.00117045 1.87579 1.87579 3.282513 A2M-AS1 no 18.9245 9.56538 2.49E-05 1.97844 1.97844 4.2930214 A2ML1 no 1268.22 2834.54 0.125214 0.447418 -2.23505 -1.5382315 A2MP1 no 0.707129 0.771518 0.831062 0.916542 -1.09106 -0.21355616 A3GALT2 no 9.32105 35.824 0.000463757 0.26019 -3.84334 -3.5461817 A4GALT no 1037.51 1183.85 0.491891 0.876391 -1.14104 -0.68828318 A4GNT no 1.77906 4.23253 0.055062 0.420331 -2.37908 -1.9270919 AA06 no 0 0 ? 1 1 020 AAAS no 983.621 874.753 0.0504054 1.12446 1.12446 1.9656721 AACP no 0 0.0252272 0.347579 ? ? -0.94100522 AACS no 1061.75 1187.96 0.256923 0.89376 -1.11887 -1.136223 AACSP1 no 11.1315 6.61222 0.218366 1.68347 1.68347 1.2338824 AADAC no 26.2877 14.736 0.173679 1.78391 1.78391 1.3642326 AADACL3 no 3.22303 1.32747 0.462986 2.42796 2.42796 0.7350327 AADACL4 no 0.844801 0.432623 0.0532204 1.95274 1.95274 1.9420129 AAED1 no 147.151 154.13 0.701405 0.954718 -1.04743 -0.3838530 AAGAB no 1220.08 1271.49 0.601375 0.959564 -1.04214 -0.52306631 AAK1 no 742.954 851.805 0.151197 0.872211 -1.14651 -1.4395732 AAMDC no 279.983 283.723 0.929024 0.986818 -1.01336 -0.089159133 AAMP no 2967.49 2181.06 1.49E-05 1.36057 1.36057 4.4137534 AANAT no 8.58309 17.2689 0.11381 0.497025 -2.01197 -1.5866635 AAR2 no 987.588 974.746 0.848617 1.01317 1.01317 0.19107537 AARS no 4113.54 3427.3 0.0306328 1.20023 1.20023 2.1737138 AARS2 no 616.699 614.872 0.972908 1.00297 1.00297 0.033993739 AARSD1 no 624.146 519.689 0.0163367 1.201 1.201 2.4170940 AASDH no 265.893 254.835 0.63558 1.04339 1.04339 0.47444941 AASDHPPT no 791.288 573.65 7.02E-05 1.37939 1.37939 4.0410142 AASS no 254.606 260.93 0.940407 0.975761 -1.02484 -0.074830543 AATF no 1702.86 1471.39 0.0152338 1.15731 1.15731 2.4430144 AATK no 203.932 139.327 0.26591 1.4637 1.4637 1.1149445 AATK-AS1 no 19.5499 5.97847 0.000546184 3.27005 3.27005 3.5007346 ABAT no 317.18 239.481 0.222644 1.32445 1.32445 1.2224647 ABCA1 no 715.745 800.19 0.543793 0.894469 -1.11798 -0.6078948 ABCA10 no 34.7621 43.1085 0.349229 0.806387 -1.2401 -0.93778449 ABCA11P no 115.306 80.7879 0.00219176 1.42727 1.42727 3.0939150 ABCA12 no 217.569 425.589 0.0518888 0.511219 -1.95611 -1.9530651 ABCA13 no 255.826 503.835 0.174831 0.507756 -1.96945 -1.3605753 ABCA2 no 1269.4 987.464 0.117187 1.28551 1.28551 1.5719354 ABCA3 no 432.443 649.888 0.264145 0.665412 -1.50283 -1.1190855 ABCA4 no 97.6868 90.5242 0.956092 1.07912 1.07912 0.055111556 ABCA5 no 433.772 587.705 0.0659072 0.738078 -1.35487 -1.8468757 ABCA6 no 56.7643 23.894 0.00644905 2.37567 2.37567 2.7463458 ABCA7 no 524.301 991.618 0.00447639 0.528733 -1.89131 -2.867659 ABCA8 no 48.8722 21.7009 0.0108657 2.25209 2.25209 2.5655260 ABCA9 no 30.9292 26.5336 0.550253 1.16566 1.16566 0.59816561 ABCB1 no 146.735 57.7675 0.000107477 2.5401 2.5401 3.9337162 ABCB10 no 435.782 501.712 0.160824 0.86859 -1.15129 -1.4063366 ABCB11 no 1.69238 6.01467 0.119431 0.281376 -3.55396 -1.5623367 ABCB4 no 26.0101 14.6088 0.00332336 1.78045 1.78045 2.9636368 ABCB5 no 9.38635 9.61315 0.947283 0.976407 -1.02416 -0.066183669 ABCB6 no 1084.12 910.745 0.213725 1.19036 1.19036 1.2464570 ABCB7 no 462.481 445.812 0.678248 1.03739 1.03739 0.41532471 ABCB8 no 911.358 772.755 0.0361873 1.17936 1.17936 2.1057772 ABCB9 no 256.807 186.232 0.00450979 1.37896 1.37896 2.8651773 ABCC1 no 3914.56 2038.88 0.000121341 1.91996 1.91996 3.9027174 ABCC10 no 710.186 541.229 0.00165573 1.31217 1.31217 3.1793275 ABCC11 no 19.9535 14.2179 0.182429 1.40341 1.40341 1.336977 ABCC13 no 1.68752 0.703604 0.157712 2.39839 2.39839 1.4169178 ABCC2 no 90.2931 29.3974 0.000836013 3.07146 3.07146 3.3802279 ABCC3 no 1427.44 1309.7 0.79682 1.0899 1.0899 0.2577380 ABCC4 no 558.14 290.982 7.98E-05 1.91813 1.91813 4.0090181 ABCC5 no 2022.7 2484.34 0.413459 0.81418 -1.22823 -0.8191482 ABCC5-AS1 no 0 0 ? 1 1 083 ABCC6 no 170.165 107.104 0.124991 1.58879 1.58879 1.5391484 ABCC6P1 no 30.0641 10.9445 0.0109621 2.74695 2.74695 2.5623885 ABCC6P2 no 8.8921 6.8311 0.57082 1.30171 1.30171 0.56756887 ABCC9 no 55.7245 41.2874 0.411508 1.34967 1.34967 0.82257288 ABCD1 no 469.929 523.091 0.496651 0.898369 -1.11313 -0.68072989 ABCD1P2 no 0 0.0237806 0.308592 ? ? -1.0201990 ABCD1P3 no 0 0.00329148 0.768013 ? ? -0.29528491 ABCD1P4 no 0.0326135 0.0499761 0.614273 0.652581 -1.53238 -0.50459492 ABCD1P5 no 0 0.00980878 0.530667 ? ? -0.62783293 ABCD2 no 13.5532 16.7075 0.517966 0.811201 -1.23274 -0.6473794 ABCD3 no 1152.83 1064.54 0.49029 1.08294 1.08294 0.69083295 ABCD4 no 924.955 771.281 0.034977 1.19925 1.19925 2.1197796 ABCE1 no 1793.74 1511.9 0.0459705 1.18641 1.18641 2.0053197 ABCF1 no 2535.81 2260.04 0.171034 1.12202 1.12202 1.372798 ABCF2 no 1774.34 1229.29 8.55E-08 1.44338 1.44338 5.5123699 ABCF2P1 no 0.0344799 0.0379292 0.903435 0.909058 -1.10004 -0.121442100 ABCF2P2 no 0 0.00171148 0.768013 ? ? -0.295284101 ABCF3 no 1716.58 1655.02 0.637797 1.0372 1.0372 0.471336102 ABCG1 no 933.989 952.027 0.908466 0.981053 -1.01931 -0.115086103 ABCG2 no 66.7189 36.7968 0.00228214 1.81317 1.81317 3.08146104 ABCG4 no 11.6102 6.14574 0.0679001 1.88914 1.88914 1.83334105 ABCG5 no 3.68568 2.8915 0.485642 1.27466 1.27466 0.698259106 ABCG8 no 2.54126 1.47975 0.239576 1.71735 1.71735 1.17876107 ABHD1 no 16.3799 9.38923 0.0085864 1.74454 1.74454 2.6483108 ABHD10 no 675.556 590.135 0.0934706 1.14475 1.14475 1.68356109 ABHD11 no 1251.31 727.579 9.70E-05 1.71983 1.71983 3.95981110 ABHD12 no 3170.7 2788.48 0.231033 1.13707 1.13707 1.20052112 ABHD13 no 379.24 364.953 0.634143 1.03915 1.03915 0.476468113 ABHD14A no 370.283 191.176 1.03E-08 1.93686 1.93686 5.91757114 ABHD14A-ACY1 no 7.47126 4.8569 0.0140937 1.53828 1.53828 2.47162115 ABHD14B no 1139.04 1120.69 0.898416 1.01638 1.01638 0.127787116 ABHD15 no 340.196 358.751 0.55332 0.948278 -1.05454 -0.593567117 ABHD16A no 1706.79 1280.76 0.000824936 1.33264 1.33264 3.38404118 ABHD16B no 111.964 128.205 0.333748 0.873324 -1.14505 -0.9684119 ABHD17A no 1232.14 1161.48 0.52323 1.06084 1.06084 0.639243120 ABHD17AP1 no 0.292844 0.348926 0.637745 0.839272 -1.19151 -0.47141121 ABHD17AP2 no 0.401296 0.357301 0.692349 1.12313 1.12313 0.396112122 ABHD17AP3 no 0.0129226 0.00143247 0.061932 9.0212 9.0212 1.8749123 ABHD17AP4 no 5.53884 12.4731 0.265414 0.444063 -2.25194 -1.1161124 ABHD17AP5 no 0.160547 0.0865082 0.369598 1.85586 1.85586 0.898793125 ABHD17AP6 no 5.68223 6.44145 0.781707 0.882136 -1.13361 -0.277384126 ABHD17AP7 no 0 0.00761456 0.573585 ? ? -0.563495127 ABHD17AP8 no 0 0.0144865 0.350261 ? ? -0.935774128 ABHD17AP9 no 0.0187252 0.011467 0.647041 1.63297 1.63297 0.458412129 ABHD17B no 381.66 388.415 0.864612 0.982606 -1.0177 -0.170676130 ABHD17C no 1173.69 1584.73 0.0317051 0.740621 -1.35022 -2.15981131 ABHD2 no 4078.01 2828.68 0.0421551 1.44166 1.44166 2.04209132 ABHD3 no 644.351 690.093 0.586245 0.933716 -1.07099 -0.544968133 ABHD4 no 1024.71 910.423 0.398754 1.12553 1.12553 0.845246134 ABHD5 no 715.537 770.234 0.501695 0.928987 -1.07644 -0.672768135 ABHD6 no 230.509 174.374 0.0291323 1.32192 1.32192 2.19389136 ABHD8 no 430.467 356.767 0.0494798 1.20658 1.20658 1.97369137 ABI1 no 1467.23 1933.59 0.00780701 0.75881 -1.31785 -2.6812138 ABI1P1 no 1.23815 1.27767 0.924862 0.969068 -1.03192 -0.0944034139 ABI2 no 1179.7 763.203 2.55E-05 1.54572 1.54572 4.28748140 ABI3 no 137.69 230.346 0.0309675 0.59775 -1.67294 -2.16933141 ABI3BP no 80.9874 195.192 0.131125 0.414913 -2.41015 -1.51448142 ABL1 no 2118.1 1640.1 0.00216834 1.29145 1.29145 3.09722143 ABL2 no 606.474 678.984 0.32533 0.893207 -1.11956 -0.985435144 ABLIM1 no 2832.43 4271.88 0.028325 0.663039 -1.50821 -2.20512145 ABLIM2 no 217.252 393.191 0.00937428 0.552536 -1.80984 -2.61767146 ABLIM3 no 114.372 356.229 0.0166287 0.321062 -3.11466 -2.41048147 ABO no 102.172 256.768 0.0404279 0.397916 -2.51309 -2.05969148 ABR no 1821.44 1881.67 0.833984 0.967991 -1.03307 -0.209806149 ABRA no 1.66077 1.22199 0.38508 1.35907 1.35907 0.870048150 ABRACL no 1100.79 822.925 0.00702633 1.33765 1.33765 2.71726151 ABT1 no 858.899 798.259 0.312336 1.07596 1.07596 1.01231152 ABT1P1 no 0 0 ? 1 1 0153 ABTB1 no 643.032 751.464 0.301426 0.855706 -1.16863 -1.03545154 ABTB2 no 330.527 250.917 0.0925929 1.31728 1.31728 1.68811155 ACAA1 no 1383.02 892.012 1.71E-08 1.55045 1.55045 5.82213156 ACAA2 no 1494.55 503.658 6.46E-12 2.96739 2.96739 7.20169157 ACACA no 1921.39 1843.41 0.661696 1.0423 1.0423 0.438076158 ACACB no 322.685 321.518 0.98328 1.00363 1.00363 0.0209773159 ACAD10 no 670.866 510.969 0.000349518 1.31293 1.31293 3.62368160 ACAD11 no 410.709 441.862 0.597472 0.929496 -1.07585 -0.528692161 ACAD8 no 532.984 404.217 0.0129793 1.31856 1.31856 2.50165162 ACAD9 no 1277.16 1507 0.0884972 0.847488 -1.17996 -1.70982163 ACADL no 21.9385 36.131 0.391281 0.607193 -1.64692 -0.858734164 ACADM no 860.459 941.666 0.510839 0.913763 -1.09438 -0.658442165 ACADS no 585.985 494.054 0.142865 1.18607 1.18607 1.46967166 ACADSB no 358.916 260.687 0.0744893 1.37681 1.37681 1.79082167 ACADVL no 6357.63 5263.7 0.0478819 1.20783 1.20783 1.98784168 ACAN no 124.945 44.9531 3.88E-06 2.77946 2.77946 4.71886169 ACAP1 no 156.602 314.121 0.0117524 0.49854 -2.00586 -2.53748170 ACAP2 no 1262.73 1560.53 0.0924904 0.809167 -1.23584 -1.68865171 ACAP3 no 968.328 1325.45 0.0233318 0.730565 -1.3688 -2.28148172 ACAT1 no 775.526 518.435 0.000332532 1.4959 1.4959 3.6372173 ACAT2 no 906.875 1012.66 0.424522 0.895542 -1.11664 -0.799866174 ACBD3 no 1287.69 1500.27 0.10559 0.858305 -1.16509 -1.62401175 ACBD4 no 281.571 225.128 0.0975645 1.25072 1.25072 1.66279176 ACBD5 no 603.257 647.466 0.547048 0.93172 -1.07328 -0.602983177 ACBD6 no 879.029 808.393 0.25692 1.08738 1.08738 1.13621178 ACBD7 no 174.969 125.649 0.247955 1.39252 1.39252 1.15794179 ACCS no 308.161 217.965 0.0265936 1.41381 1.41381 2.23017180 ACCSL no 0.473312 0.211477 0.0125393 2.23813 2.23813 2.51414181 ACD no 462.917 535.052 0.104706 0.865182 -1.15583 -1.62816182 ACE no 598.18 425.773 0.202355 1.40493 1.40493 1.27811183 ACE2 no 219.024 107.946 0.0840279 2.02902 2.02902 1.73445184 ACE3P no 0.0794398 0.093256 0.819523 0.851846 -1.17392 -0.228391185 ACER1 no 3.58458 3.59292 0.997176 0.997679 -1.00233 -0.00354318186 ACER2 no 69.8585 72.0766 0.855911 0.969225 -1.03175 -0.181763187 ACER3 no 296.602 324.843 0.506118 0.913063 -1.09522 -0.665821188 ACHE no 83.4188 120.805 0.737823 0.690525 -1.44817 -0.335097189 ACIN1 no 4415.89 3859.35 0.0255803 1.14421 1.14421 2.24549190 ACKR2 no 136.204 148.735 0.870625 0.91575 -1.092 -0.163026191 ACKR3 no 998.415 1375.49 0.33685 0.725861 -1.37767 -0.962193192 ACKR4 no 0.544956 6.50781 0.142758 0.0837387 -11.9419 -1.47006193 ACLY no 4772.99 2622.17 3.15E-07 1.82024 1.82024 5.25131194 ACMSD no 68.6208 42.6351 0.00345769 1.60949 1.60949 2.951195 ACN9 no 454.971 289.784 1.15E-07 1.57003 1.57003 5.45354196 ACO1 no 847.785 873.442 0.756568 0.970625 -1.03026 -0.310319197 ACO2 no 3668.43 2997.86 0.0180671 1.22368 1.22368 2.37937198 ACOT1 no 20.7245 20.6836 0.994054 1.00198 1.00198 0.0074593199 ACOT11 no 396.123 411.81 0.855278 0.961907 -1.0396 -0.18257200 ACOT12 no 1.46977 0.920494 0.104706 1.59671 1.59671 1.62817201 ACOT13 no 924.432 596.721 5.56E-06 1.54919 1.54919 4.63902202 ACOT2 no 547.327 493.6 0.49643 1.10885 1.10885 0.681078203 ACOT4 no 94.3735 124.994 0.114749 0.755025 -1.32446 -1.58253204 ACOT6 no 1.49699 1.4168 0.859223 1.05659 1.05659 0.17754205 ACOT7 no 1184.29 1270.98 0.585186 0.931791 -1.0732 -0.54651206 ACOT8 no 680.132 629.46 0.551891 1.0805 1.0805 0.595707207 ACOT9 no 767.239 836.354 0.522082 0.917361 -1.09008 -0.641012208 ACOX1 no 1408.44 1205.65 0.124451 1.1682 1.1682 1.54136209 ACOX2 no 96.6371 26.4646 1.00E-08 3.65156 3.65156 5.92305210 ACOX3 no 455.39 505.181 0.396536 0.901438 -1.10934 -0.849233211 ACOXL no 50.9416 38.4684 0.183528 1.32424 1.32424 1.33354212 ACP1 no 2115.03 1712.36 0.00342666 1.23515 1.23515 2.95387213 ACP2 no 1144.81 934.545 0.0214744 1.22499 1.22499 2.31358214 ACP5 no 848.048 1122.05 0.199547 0.755803 -1.3231 -1.28613215 ACP6 no 492.095 304.815 0.000406551 1.61441 1.61441 3.58242216 ACPL2 no 461.989 494.618 0.687486 0.934032 -1.07063 -0.402721217 ACPP no 300.353 237.508 0.302608 1.2646 1.2646 1.03291218 ACPT no 3.88498 5.78924 0.175935 0.671069 -1.49016 -1.35709219 ACR no 6.46957 4.16293 0.204127 1.55409 1.55409 1.27309220 ACRBP no 66.9468 42.0406 0.00330797 1.59243 1.59243 2.96511221 ACRC no 38.4622 36.8377 0.840777 1.0441 1.0441 0.201102222 ACRV1 no 20.2116 19.1702 0.74006 1.05432 1.05432 0.332129223 ACSBG1 no 27.6694 32.6389 0.828894 0.847745 -1.1796 -0.216339224 ACSBG2 no 47.1142 52.832 0.434258 0.891774 -1.12136 -0.783145225 ACSF2 no 1100.48 938.852 0.404023 1.17215 1.17215 0.835826226 ACSF3 no 554.825 602.814 0.34361 0.920392 -1.08649 -0.948793227 ACSL1 no 1166.77 1263.31 0.676641 0.923575 -1.08275 -0.417524228 ACSL3 no 1406.77 1528.07 0.506629 0.920618 -1.08623 -0.66502229 ACSL4 no 918.201 984.851 0.752616 0.932324 -1.07259 -0.315526230 ACSL5 no 1797.44 991.064 0.00215727 1.81365 1.81365 3.09879231 ACSL6 no 49.191 19.909 0.0331649 2.47079 2.47079 2.14153232 ACSM1 no 62.4696 30.2249 0.0102735 2.06682 2.06682 2.58541233 ACSM2A no 1.49365 0.898721 0.394148 1.66197 1.66197 0.853541234 ACSM2B no 0.73386 0.707655 0.955303 1.03703 1.03703 0.0561029235 ACSM3 no 335.839 123.151 5.59E-05 2.72706 2.72706 4.09769236 ACSM4 no 5.90724 3.19181 0.0579056 1.85075 1.85075 1.90486237 ACSM5 no 7.32231 6.1331 0.620274 1.1939 1.1939 0.496058238 ACSS1 no 1142.53 742.13 0.0306863 1.53952 1.53952 2.17301239 ACSS2 no 1371.94 1014.17 0.00958836 1.35278 1.35278 2.60974241 ACTA1 no 6.87066 8.94994 0.889491 0.767676 -1.30263 -0.139085242 ACTA2 no 3587.77 2493.97 0.344637 1.43858 1.43858 0.946773243 ACTA2-AS1 no 0.0210637 0.0174679 0.860871 1.20585 1.20585 0.17544244 ACTB no 90425.3 103300 0.0837065 0.875366 -1.14238 -1.73626245 ACTBL2 no 1.63355 7.70869 0.122375 0.21191 -4.71899 -1.54995246 ACTBP1 no 1.0041 1.67458 0.660668 0.599613 -1.66774 -0.439496247 ACTBP10 no 0.196774 0.190654 0.933545 1.0321 1.0321 0.0834663248 ACTBP11 no 0.371169 0.733688 0.279143 0.505895 -1.97669 -1.08452249 ACTBP12 no 0.0462707 0 0.00065245 ? ? 3.4508250 ACTBP13 no 0.772635 2.61963 0.484551 0.29494 -3.39052 -0.700007251 ACTBP14 no 0.194242 0.134048 0.29306 1.44905 1.44905 1.05357252 ACTBP2 no 1.12453 0.868372 0.291785 1.29498 1.29498 1.05637253 ACTBP3 no 0 0 ? 1 1 0254 ACTBP4 no 0 0.00506405 0.624903 ? ? -0.489499257 ACTBP8 no 0.982339 0.819999 0.902011 1.19798 1.19798 0.123242258 ACTBP9 no 1.33903 0.8435 0.0515495 1.58747 1.58747 1.95592259 ACTC1 no 55.1824 15.6944 0.128508 3.51606 3.51606 1.52488260 ACTG1 no 77804.2 71075.8 0.206026 1.09467 1.09467 1.26775261 ACTG1P1 no 2.14038 2.22218 0.894075 0.963192 -1.03821 -0.13328262 ACTG1P10 no 5.7458 8.76693 0.317143 0.655395 -1.5258 -1.00228263 ACTG1P11 no 0 0.0215022 0.291865 ? ? -1.05619265 ACTG1P2 no 0 0.0200799 0.331339 ? ? -0.973246267 ACTG1P4 no 0.0370669 0.0228276 0.539189 1.62377 1.62377 0.614857268 ACTG1P9 no 0.170006 0.0588083 0.0258075 2.89084 2.89084 2.24201269 ACTG2 no 347.956 517.118 0.788887 0.672876 -1.48616 -0.268033270 ACTL10 no 0.0513665 0.0921009 0.431617 0.55772 -1.79301 -0.787659271 ACTL6A no 1929.82 2328.66 0.137459 0.828726 -1.20667 -1.48993272 ACTL6B no 1.24461 1.28373 0.925766 0.969532 -1.03143 -0.0932642273 ACTL7A no 0.0200437 0.0500858 0.40427 0.400186 -2.49884 -0.835386274 ACTL7B no 0.0287222 0.190247 0.0408974 0.150973 -6.6237 -2.05484275 ACTL8 no 185.495 92.4131 0.371431 2.00723 2.00723 0.89535276 ACTL9 no 0.108026 0.0613862 0.660978 1.75977 1.75977 0.439068277 ACTN1 no 3824.05 5748.13 0.0553162 0.665269 -1.50315 -1.92507278 ACTN1-AS1 no 2.0024 3.59431 0.127836 0.557102 -1.795 -1.52759279 ACTN2 no 10.0485 6.09244 0.402353 1.64933 1.64933 0.838804280 ACTN3 no 2.12956 1.23118 0.00192634 1.72969 1.72969 3.13345281 ACTN4 no 11952.4 17075.8 0.00834319 0.699965 -1.42864 -2.65827282 ACTN4P1 no 0.310663 0.390238 0.406191 0.796087 -1.25614 -0.831973283 ACTN4P2 no 0.339043 0.427447 0.819229 0.793183 -1.26074 -0.228768284 ACTR10 no 1033.6 1085.7 0.443922 0.952012 -1.05041 -0.766761285 ACTR1A no 2648.82 2222.84 0.00100827 1.19164 1.19164 3.32609286 ACTR1B no 1596.52 1452.95 0.154614 1.09882 1.09882 1.42759287 ACTR2 no 5268.45 4997.55 0.476421 1.05421 1.05421 0.713108288 ACTR3 no 5343.18 4714.85 0.0644686 1.13326 1.13326 1.85684289 ACTR3B no 352.735 150.07 5.12E-17 2.35047 2.35047 8.99605290 ACTR3BP1 no 0 0.00256473 0.677334 ? ? -0.416576291 ACTR3BP2 no 0 0.00108804 0.768013 ? ? -0.295284292 ACTR3BP3 no 0 0.0126121 0.429805 ? ? -0.790764293 ACTR3BP4 no 0 0 ? 1 1 0294 ACTR3BP5 no 0 0.00116041 0.768013 ? ? -0.295284295 ACTR3BP6 no 0 0.00392891 0.676325 ? ? -0.417957296 ACTR3C no 188.879 113.731 0.000315696 1.66075 1.66075 3.65125297 ACTR3P1 no 0.0925414 0.0181523 0.0219124 5.09804 5.09804 2.3058298 ACTR3P2 no 0.0185835 0.0118587 0.640668 1.56708 1.56708 0.467315299 ACTR3P3 no 0 0.00347122 0.691106 ? ? -0.397799300 ACTR5 no 348.803 385.203 0.319596 0.905503 -1.10436 -0.997204301 ACTR6 no 587.836 515.096 0.20439 1.14122 1.14122 1.27235302 ACTR6P1 no 0 0 ? 1 1 0303 ACTR8 no 663.378 431.263 2.76E-10 1.53822 1.53822 6.56935304 ACTRT1 no 0.060888 0.0428116 0.805702 1.42223 1.42223 0.246226305 ACTRT2 no 0 0.00397685 0.620738 ? ? -0.4954306 ACTRT3 no 57.3637 66.5643 0.30454 0.861779 -1.16039 -1.02879307 ACVR1 no 614.773 680.323 0.378177 0.903649 -1.10662 -0.882774308 ACVR1B no 1159.93 1183.78 0.849185 0.979854 -1.02056 -0.190348309 ACVR1C no 42.0946 81.05 0.0613121 0.519366 -1.92542 -1.8794310 ACVR2A no 307.544 322.764 0.704699 0.952846 -1.04949 -0.379404311 ACVR2B no 419.757 206.89 2.75E-13 2.02889 2.02889 7.7074312 ACVR2B-AS1 no 28.4291 14.8138 5.22E-06 1.9191 1.9191 4.65323313 ACVRL1 no 381.315 219.694 0.0022955 1.73566 1.73566 3.07965314 ACY1 no 909.958 713.2 0.0690868 1.27588 1.27588 1.82544315 ACY3 no 86.7699 149.12 0.492429 0.58188 -1.71857 -0.687427316 ACYP1 no 325.869 417.994 0.0660239 0.779601 -1.28271 -1.84606317 ACYP2 no 142.074 159.309 0.320521 0.891814 -1.12131 -0.995297318 ADA no 266.261 451.298 0.0547098 0.589989 -1.69495 -1.92991319 ADAD1 no 0.770045 0.421534 0.0811169 1.82677 1.82677 1.75107320 ADAD1P1 no 0.0287222 0.0275049 0.967937 1.04426 1.04426 0.040235321 ADAD1P2 no 0 0 ? 1 1 0322 ADAD2 no 45.9274 99.1827 0.0383753 0.463059 -2.15955 -2.08145323 ADAL no 136.487 160.975 0.135642 0.847879 -1.17941 -1.49688324 ADAM10 no 2157.88 2445.47 0.301887 0.882399 -1.13327 -1.03446325 ADAM11 no 41.4028 34.9461 0.568153 1.18476 1.18476 0.571506326 ADAM12 no 512.186 340.514 0.0960162 1.50415 1.50415 1.67056327 ADAM15 no 2784.27 5043.34 0.00149158 0.552068 -1.81137 -3.21065328 ADAM17 no 944.606 1058.84 0.309005 0.892112 -1.12094 -1.01931329 ADAM18 no 3.92539 1.23557 0.0297024 3.17699 3.17699 2.18612330 ADAM19 no 547.479 757.126 0.0904855 0.723101 -1.38293 -1.69918331 ADAM1A no 101.811 68.9815 0.000683563 1.47592 1.47592 3.43763332 ADAM1B no 6.36665 3.58533 0.0101307 1.77575 1.77575 2.59036334 ADAM20 no 7.46773 5.68784 0.0292138 1.31293 1.31293 2.19277335 ADAM20P1 no 11.1167 11.0541 0.975892 1.00567 1.00567 0.0302482336 ADAM20P2 no 0 0.00181195 0.768013 ? ? -0.295284338 ADAM21 no 5.15138 4.73034 0.723758 1.08901 1.08901 0.353824339 ADAM21P1 no 0.299331 0.226017 0.452645 1.32437 1.32437 0.752149340 ADAM22 no 121.101 125.983 0.894438 0.961248 -1.04031 -0.132821341 ADAM23 no 546.003 421.758 0.612154 1.29459 1.29459 0.507617342 ADAM24P no 0 0.0141674 0.462416 ? ? -0.735968343 ADAM28 no 355.065 364.486 0.922186 0.974153 -1.02653 -0.0977753344 ADAM29 no 0.547105 0.366632 0.302654 1.49224 1.49224 1.03282345 ADAM30 no 0.0802972 0.0186484 0.0326642 4.30585 4.30585 2.14772346 ADAM32 no 63.4493 29.5458 2.24E-05 2.14749 2.14749 4.31834347 ADAM33 no 167.461 92.6267 0.0409614 1.80792 1.80792 2.05419348 ADAM3A no 0.458151 0.263154 0.1161 1.741 1.741 1.57663349 ADAM3B no 0 0 ? 1 1 0351 ADAM6 no 0 0 ? 1 1 0352 ADAM7 no 2.49958 1.2134 0.0379107 2.05998 2.05998 2.08651353 ADAM8 no 464.901 1366.4 0.000906593 0.340238 -2.93912 -3.35689354 ADAM9 no 2309.12 2474.58 0.594433 0.933136 -1.07165 -0.533083355 ADAMDEC1 no 164.955 152.215 0.8612 1.0837 1.0837 0.175021356 ADAMTS1 no 699.278 450.149 0.17921 1.55344 1.55344 1.34684357 ADAMTS10 no 150.505 85.6288 0.00960697 1.75764 1.75764 2.60906358 ADAMTS12 no 215.082 112.951 0.014623 1.90421 1.90421 2.4581359 ADAMTS13 no 156.666 82.4726 3.17E-05 1.89962 1.89962 4.23599360 ADAMTS14 no 126.806 158.812 0.518321 0.798463 -1.25241 -0.646821361 ADAMTS15 no 221.857 162.31 0.631189 1.36687 1.36687 0.480625363 ADAMTS17 no 118.32 155.983 0.331439 0.758544 -1.31832 -0.973044366 ADAMTS2 no 762.521 482.904 0.0224422 1.57903 1.57903 2.29656367 ADAMTS20 no 9.35845 4.80779 0.219755 1.94652 1.94652 1.23016368 ADAMTS3 no 30.9515 20.1751 0.170544 1.53414 1.53414 1.37428369 ADAMTS4 no 243.392 209.27 0.627712 1.16306 1.16306 0.485528370 ADAMTS5 no 139.213 48.6021 0.000863824 2.86434 2.86434 3.37081371 ADAMTS6 no 75.2835 46.2526 0.0782776 1.62766 1.62766 1.76776372 ADAMTS7 no 398.128 174.909 0.00035882 2.2762 2.2762 3.61654375 ADAMTS9-AS2 no 24.0511 12.761 0.0126169 1.88473 1.88473 2.51191376 ADAMTSL1 no 242.053 67.2211 0.115326 3.60085 3.60085 1.58377 ADAMTSL2 no 251.957 120.987 0.116961 2.08251 2.08251 1.57291378 ADAMTSL3 no 62.6235 182.887 0.0198635 0.342416 -2.92043 -2.34344379 ADAMTSL4 no 358.924 948.932 0.317113 0.37824 -2.64382 -1.00234380 ADAMTSL5 no 100.973 215.119 0.00538124 0.469382 -2.13046 -2.80696381 ADAP1 no 684.995 568.688 0.441907 1.20452 1.20452 0.77016382 ADAP2 no 141.069 155.543 0.547473 0.906941 -1.10261 -0.602343383 ADAR no 6207.41 7414.62 0.0745857 0.837185 -1.19448 -1.79023384 ADARB1 no 301.049 312.726 0.91683 0.962661 -1.03879 -0.10453385 ADARB2 no 134.411 261.765 0.304577 0.513479 -1.9475 -1.02871386 ADARB2-AS1 no 0 0.00266295 0.701649 ? ? -0.38352387 ADAT1 no 538.464 525.106 0.791629 1.02544 1.02544 0.264469388 ADAT2 no 329.774 233.531 0.0002473 1.41212 1.41212 3.71674389 ADAT3 no 4.57423 4.27594 0.69133 1.06976 1.06976 0.397494390 ADC no 85.3703 102.588 0.378277 0.832168 -1.20168 -0.882588391 ADCK1 no 259.834 216.061 0.0340712 1.2026 1.2026 2.13052392 ADCK2 no 905.022 684.656 0.000532157 1.32186 1.32186 3.50799393 ADCK3 no 910.124 773.391 0.199821 1.1768 1.1768 1.28534394 ADCK4 no 672.606 767.221 0.159969 0.876677 -1.14067 -1.40922395 ADCK5 no 399.008 346.927 0.14279 1.15012 1.15012 1.46995396 ADCY1 no 194.598 85.2537 0.00209683 2.28258 2.28258 3.10752397 ADCY10 no 19.2076 16.1053 0.433831 1.19263 1.19263 0.783873398 ADCY10P1 no 75.2563 47.0332 0.00957328 1.60007 1.60007 2.6103399 ADCY2 no 47.2764 147.793 0.718836 0.319881 -3.12616 -0.360408400 ADCY3 no 1651.9 2078.81 0.0196904 0.794635 -1.25844 -2.34678401 ADCY4 no 146.315 112.635 0.254394 1.29902 1.29902 1.14228402 ADCY5 no 388.035 118.413 0.000172301 3.27697 3.27697 3.81202403 ADCY6 no 1425.99 1191.49 0.252788 1.19681 1.19681 1.14616404 ADCY7 no 699.43 853.206 0.227707 0.819766 -1.21986 -1.20915406 ADCY9 no 427.101 607.398 0.0156119 0.703166 -1.42214 -2.43394407 ADCYAP1 no 2.37035 14.0647 0.492365 0.168532 -5.93359 -0.687529409 ADD1 no 3664.59 2769.54 3.10E-05 1.32317 1.32317 4.24129410 ADD2 no 83.901 68.5258 0.711518 1.22437 1.22437 0.370224411 ADD3 no 2456.09 1690.36 0.00925008 1.453 1.453 2.62234413 ADGB no 10.317 6.08808 0.316172 1.69462 1.69462 1.0043414 ADH1A no 1.24382 3.60309 0.178124 0.345209 -2.8968 -1.35022415 ADH1B no 3.31957 45.6319 0.579485 0.0727468 -13.7463 -0.554837416 ADH1C no 112.289 380.324 0.522668 0.295247 -3.38699 -0.640109417 ADH4 no 2.52223 3.5014 0.694377 0.720349 -1.38822 -0.393361418 ADH5 no 3237.45 2702.96 0.113543 1.19774 1.19774 1.58784419 ADH5P2 no 0.0597245 0.022416 0.123597 2.66437 2.66437 1.54488420 ADH5P3 no 0.0155072 0.0069538 0.458258 2.23003 2.23003 0.74283421 ADH5P4 no 3.36298 1.75279 0.0346246 1.91864 1.91864 2.12392422 ADH6 no 13.0124 14.0909 0.884642 0.923462 -1.08288 -0.145231423 ADH7 no 961.011 1014.47 0.910395 0.947306 -1.05562 -0.112651424 ADHFE1 no 79.5337 102.045 0.232209 0.779396 -1.28305 -1.19749425 ADI1 no 2273.77 2156.21 0.525894 1.05452 1.05452 0.635146426 ADIG no 0.206971 0.243497 0.766489 0.849993 -1.17648 -0.297283427 ADIPOQ no 1.61735 23.0236 0.688342 0.0702475 -14.2354 -0.401556428 ADIPOQ-AS1 no 0.0370638 0.0281609 0.715362 1.31614 1.31614 0.365064429 ADIPOR1 no 2772.82 3342.43 0.0250825 0.829583 -1.20542 -2.25322430 ADIPOR1P1 no 0 0.00164574 0.768013 ? ? -0.295284431 ADIPOR1P2 no 0 0.0225844 0.677092 ? ? -0.416906432 ADIPOR2 no 1738.45 1990.6 0.0964508 0.873329 -1.14504 -1.66837433 ADIRF no 529.331 1332.97 0.0414233 0.397107 -2.51822 -2.04947434 ADK no 902.227 1123.1 0.0248895 0.803337 -1.24481 -2.25625435 ADM no 1310 1821.01 0.298758 0.719381 -1.39008 -1.04119436 ADM2 no 271.786 192.842 0.164476 1.40938 1.40938 1.39412437 ADM5 no 56.9947 42.0192 0.54267 1.3564 1.3564 0.609586438 ADNP no 2725.53 2641.49 0.687955 1.03182 1.03182 0.402082439 ADNP2 no 587.727 655.161 0.263279 0.897072 -1.11474 -1.12111440 ADO no 599.726 493.072 0.00125727 1.21631 1.21631 3.26142441 ADORA1 no 146.853 81.044 0.0055472 1.81201 1.81201 2.79686442 ADORA2A no 107.523 124.029 0.542203 0.866922 -1.15351 -0.610294443 ADORA2A-AS1 no 5.74145 15.9036 0.0719674 0.361015 -2.76997 -1.80671444 ADORA2B no 218.05 313.303 0.028144 0.695972 -1.43684 -2.20767445 ADORA2BP1 no 0.934982 1.20059 0.605867 0.778766 -1.28408 -0.516613446 ADORA3 no 42.3137 73.9117 0.0340986 0.57249 -1.74676 -2.13019447 ADPGK no 1126.78 1318.03 0.054423 0.854901 -1.16973 -1.93222448 ADPGK-AS1 no 1.85666 2.71909 0.0904782 0.682823 -1.46451 -1.69922449 ADPRH no 128.554 140.072 0.571844 0.917771 -1.0896 -0.566059450 ADPRHL1 no 104.086 65.219 0.00471795 1.59595 1.59595 2.85039451 ADPRHL2 no 979.954 1116.61 0.0653625 0.877612 -1.13946 -1.85062452 ADPRM no 114.187 116.102 0.8426 0.983506 -1.01677 -0.198768454 ADRA1B no 27.7463 14.9736 0.163952 1.85302 1.85302 1.39586455 ADRA1D no 7.9002 10.8423 0.701215 0.728649 -1.3724 -0.384106456 ADRA2A no 346.053 146.972 0.0176597 2.35456 2.35456 2.38795457 ADRA2B no 55.3853 36.862 0.524365 1.5025 1.5025 0.637496459 ADRB1 no 44.2486 33.4575 0.382678 1.32253 1.32253 0.874461461 ADRB3 no 1.19834 1.31037 0.932932 0.914505 -1.09349 -0.0842389462 ADRBK1 no 2978.66 2947.04 0.884386 1.01073 1.01073 0.145555463 ADRBK2 no 494.587 282.795 1.34E-05 1.74892 1.74892 4.43831464 ADRM1 no 3454.28 3351.69 0.697063 1.03061 1.03061 0.389721465 ADSL no 1546.35 950.881 2.36E-10 1.62623 1.62623 6.59602466 ADSS no 1602.5 1787.02 0.283338 0.896747 -1.11514 -1.07508467 ADSSL1 no 185.314 433.214 0.0152844 0.427765 -2.33773 -2.44179468 ADTRP no 70.2933 111.096 0.508053 0.632726 -1.58046 -0.662792469 AEBP1 no 4136.27 3679.24 0.615584 1.12422 1.12422 0.502727470 AEBP2 no 741.3 952.939 0.0121802 0.777908 -1.2855 -2.52462471 AEN no 797.141 645.233 0.0229956 1.23543 1.23543 2.28712472 AES no 8049.94 8903.54 0.405222 0.904128 -1.10604 -0.833694473 AF357530 no 0 0.00359088 0.676729 ? ? -0.417404474 AF357533 no 0.0200437 0.0096924 0.601951 2.06798 2.06798 0.522238475 AFAP1 no 1003.93 1018.97 0.928637 0.985239 -1.01498 -0.0896473476 AFAP1-AS1 no 4.96065 5.17726 0.927598 0.958162 -1.04366 -0.090956477 AFAP1L1 no 155.203 218.524 0.192578 0.710234 -1.40799 -1.30639478 AFAP1L2 no 1420.02 1219.79 0.324086 1.16416 1.16416 0.987978479 AFF1 no 2132.54 1685.43 0.0443071 1.26528 1.26528 2.02101480 AFF2 no 169.295 87.76 0.116906 1.92907 1.92907 1.57314482 AFF4 no 1758.18 1523.74 0.0945253 1.15386 1.15386 1.67814483 AFG3L1P no 382.163 411.239 0.529271 0.929298 -1.07608 -0.629968484 AFG3L2 no 2451.43 2095.93 0.0415481 1.16961 1.16961 2.04821485 AFG3L2P1 no 0.52997 0.483035 0.697186 1.09717 1.09717 0.389554486 AFM no 0.204876 0.435179 0.593237 0.470786 -2.12411 -0.534814487 AFMID no 548.453 555.589 0.915225 0.987155 -1.01301 -0.106554488 AFP no 0.808374 3.55058 0.676701 0.227674 -4.39225 -0.417441489 AFTPH no 1308.26 1308.05 0.998493 1.00016 1.00016 0.0018909490 AGA no 432.265 409.64 0.629321 1.05523 1.05523 0.483258491 AGAP1 no 994.199 726.243 0.00844415 1.36896 1.36896 2.6541492 AGAP1-IT1 no 0.0203008 0.0138785 0.725971 1.46275 1.46275 0.350871493 AGAP10 no 134.854 158.077 0.446077 0.853094 -1.1722 -0.763136494 AGAP11 no 83.4172 168.445 0.0253554 0.495218 -2.01931 -2.24896495 AGAP2 no 255.69 431.386 4.01E-05 0.592717 -1.68715 -4.17885496 AGAP2-AS1 no 4.35308 4.64805 0.759331 0.936539 -1.06776 -0.306683497 AGAP3 no 2065.71 1564.85 0.00393749 1.32007 1.32007 2.90926498 AGAP4 no 73.6621 71.9205 0.882732 1.02422 1.02422 0.147652499 AGAP5 no 138.519 144.322 0.760353 0.959791 -1.04189 -0.305339500 AGAP6 no 421.629 377.968 0.356707 1.11552 1.11552 0.923307501 AGAP7 no 1.50826 1.30485 0.653969 1.15589 1.15589 0.448775502 AGAP8 no 0.756289 0.716724 0.799429 1.0552 1.0552 0.254347503 AGAP9 no 23.6453 26.7991 0.532878 0.882317 -1.13338 -0.624455504 AGBL1 no 14.3537 24.7319 0.422786 0.580371 -1.72304 -0.80287505 AGBL1-AS1 no 0 0.011011 0.48539 ? ? -0.698663506 AGBL2 no 47.4642 35.7219 0.182195 1.32871 1.32871 1.33762507 AGBL3 no 102.951 100.209 0.829887 1.02736 1.02736 0.215063508 AGBL4 no 53.3781 360.983 0.777579 0.147869 -6.76275 -0.28277509 AGBL5 no 1064.83 800.858 0.00133376 1.32961 1.32961 3.24395510 AGER no 166.562 107.021 5.25E-05 1.55635 1.55635 4.11305511 AGFG1 no 1452.29 1570.63 0.483553 0.924658 -1.08148 -0.70161512 AGFG2 no 443.135 528.154 0.189402 0.839026 -1.19186 -1.31581513 AGGF1 no 802.228 739.241 0.207899 1.0852 1.0852 1.26251514 AGGF1P1 no 13.3627 8.43766 0.133866 1.58369 1.58369 1.50374515 AGGF1P2 no 7.31557 3.80874 0.00581137 1.92073 1.92073 2.78134516 AGGF1P3 no 1.1531 0.580191 0.173192 1.98744 1.98744 1.36578517 AGGF1P4 no 0 0.00310339 0.67625 ? ? -0.41806518 AGK no 697.381 623.128 0.150592 1.11916 1.11916 1.44171519 AGKP1 no 0 0 ? 1 1 0520 AGL no 761.488 1054.65 0.174804 0.722032 -1.38498 -1.36066521 AGMAT no 141.935 112.159 0.270296 1.26548 1.26548 1.10474522 AGMO no 21.5182 18.396 0.89553 1.16972 1.16972 0.13144523 AGO1 no 1287.89 891.101 1.50E-07 1.44528 1.44528 5.40154524 AGO2 no 361.989 324.5 0.360064 1.11553 1.11553 0.91687525 AGO3 no 253.154 206.955 0.00345279 1.22323 1.22323 2.95145526 AGO4 no 531.291 532.724 0.980264 0.997309 -1.0027 -0.0247622527 AGPAT1 no 1985.51 1323.75 8.10E-08 1.49991 1.49991 5.52301528 AGPAT2 no 2045.42 1597.05 0.0933161 1.28075 1.28075 1.68436529 AGPAT3 no 1841.31 1680.04 0.263488 1.09599 1.09599 1.12062530 AGPAT4 no 377.95 281.408 0.0972329 1.34307 1.34307 1.66444531 AGPAT4-IT1 no 0 0.0244997 0.336163 ? ? -0.963565532 AGPAT5 no 949.128 681.198 0.000978939 1.39332 1.39332 3.33467533 AGPAT5P1 no 0 0 ? 1 1 0534 AGPAT6 no 2452.69 1511.42 3.91E-11 1.62277 1.62277 6.9032535 AGPAT9 no 147.561 91.6326 0.0440026 1.61036 1.61036 2.02394536 AGPS no 1063.41 1246.69 0.14742 0.852982 -1.17236 -1.45305537 AGR2 no 5127.82 6921.46 0.560819 0.740858 -1.34979 -0.582378538 AGR3 no 131.168 329.956 0.266674 0.397531 -2.51553 -1.11316539 AGRN no 7595.02 8939.13 0.264806 0.849638 -1.17697 -1.11752540 AGRP no 1.18612 0.574198 0.0829627 2.0657 2.0657 1.74048542 AGTPBP1 no 456.829 352.982 0.00496363 1.2942 1.2942 2.83369543 AGTR1 no 6.44432 5.13435 0.6403 1.25514 1.25514 0.46783545 AGTRAP no 1089.9 1782.25 0.00077578 0.611529 -1.63524 -3.40163546 AGXT no 6.88131 4.42238 0.488383 1.55602 1.55602 0.693875547 AGXT2 no 4.51942 2.2293 0.0575275 2.02728 2.02728 1.90776548 AHCTF1 no 1467.61 1445.97 0.870596 1.01496 1.01496 0.163063549 AHCTF1P1 no 16.4156 11.983 0.0835226 1.3699 1.3699 1.7373550 AHCY no 5644.32 3831.69 7.74E-05 1.47306 1.47306 4.01649551 AHCYL1 no 3060.22 2512.91 0.0108228 1.2178 1.2178 2.56693552 AHCYL2 no 785.05 588.783 0.0784533 1.33334 1.33334 1.76672553 AHCYP1 no 0.0444769 0.179972 0.0584197 0.247132 -4.04642 -1.90094554 AHCYP2 no 0.474487 0.467712 0.960101 1.01449 1.01449 0.0500754556 AHCYP4 no 0 0.00108804 0.768013 ? ? -0.295284557 AHCYP5 no 0 0.00423816 0.61087 ? ? -0.509451558 AHCYP6 no 0 0.01061 0.655452 ? ? -0.446718559 AHCYP7 no 0 0.0183177 0.41368 ? ? -0.818753560 AHDC1 no 822.752 1184.5 0.0212785 0.694601 -1.43968 -2.3171561 AHI1 no 439.921 385.798 0.278533 1.14029 1.14029 1.0859562 AHNAK no 11241.1 21126.7 0.000406041 0.532079 -1.87942 -3.58276563 AHNAK2 no 1202.44 4041.02 0.000182795 0.297559 -3.36068 -3.79656564 AHR no 1233.38 1577.87 0.1753 0.781672 -1.27931 -1.35909565 AHRR no 176.772 120.567 0.173919 1.46618 1.46618 1.36347566 AHSA1 no 2967.61 2230.25 0.000107267 1.33061 1.33061 3.93421567 AHSA2 no 1300.15 873.359 0.00227392 1.48868 1.48868 3.08257568 AHSG no 2.81507 5.53756 0.487117 0.508358 -1.96712 -0.695897569 AHSP no 0.470105 0.582987 0.823172 0.806374 -1.24012 -0.223694570 AICDA no 0.495302 1.33016 0.113191 0.372362 -2.68556 -1.5894571 AIDA no 1178.53 974.834 0.023095 1.20896 1.20896 2.28545572 AIF1 no 198.696 360.14 0.0474083 0.551717 -1.81252 -1.99211573 AIF1L no 967.988 651.686 0.0354589 1.48536 1.48536 2.11414574 AIFM1 no 1499.82 1055.87 0.0022506 1.42045 1.42045 3.08575575 AIFM1P1 no 0.0400343 0.0208024 0.393312 1.92451 1.92451 0.855053576 AIFM2 no 686.241 405.206 2.64E-07 1.69356 1.69356 5.28732577 AIFM3 no 138.242 60.6633 0.000429484 2.27883 2.27883 3.56735578 AIG1 no 876.845 771.803 0.192285 1.1361 1.1361 1.30726579 AIM1 no 1096.44 2499.64 2.07E-05 0.438638 -2.27978 -4.33745580 AIM1L no 354.956 1244.16 0.000118851 0.285298 -3.50511 -3.90803582 AIMP1 no 1237.95 1216.72 0.772852 1.01745 1.01745 0.288949583 AIMP1P1 no 0 0 ? 1 1 0584 AIMP1P2 no 0 0.0137665 0.440652 ? ? -0.772281585 AIMP2 no 709.866 533.447 5.55E-05 1.33072 1.33072 4.09926586 AIP no 1205.88 1278.61 0.509278 0.94312 -1.06031 -0.660878587 AIPL1 no 1.15198 3.34774 0.182719 0.344108 -2.90607 -1.33601589 AIRN no 0 0 ? 1 1 0590 AJAP1 no 67.9768 30.9284 0.130818 2.19788 2.19788 1.51569591 AJUBA no 877.641 1601.47 0.00565711 0.548023 -1.82474 -2.79032592 AK1 no 850.7 1162.05 0.120762 0.732066 -1.366 -1.5567593 AK2 no 3213.96 4114.51 0.000213613 0.781128 -1.2802 -3.75558594 AK2P1 no 4.74512 4.14775 0.347461 1.14402 1.14402 0.941235595 AK2P2 no 0.261241 0.31937 0.567023 0.817988 -1.22251 -0.573176596 AK3 no 1545.72 1689.79 0.627875 0.914743 -1.0932 -0.485298597 AK3P2 no 0.233934 0.312281 0.789797 0.749113 -1.33491 -0.26685598 AK3P3 no 0 0.00356996 0.682295 ? ? -0.409796599 AK3P4 no 0 0.00905261 0.475199 ? ? -0.715087600 AK3P5 no 0 0 ? 1 1 0601 AK3P6 no 0 0 ? 1 1 0602 AK4 no 1369.61 1266.25 0.591719 1.08163 1.08163 0.537014603 AK4P1 no 0.360353 0.240497 0.180369 1.49837 1.49837 1.34324605 AK4P3 no 7.17423 2.95525 0.00492043 2.42763 2.42763 2.83657606 AK4P4 no 0.544869 0.424211 0.389745 1.28443 1.28443 0.861526607 AK4P5 no 0 0.00396662 0.676526 ? ? -0.417682608 AK4P6 no 0.207604 0.156115 0.482244 1.32982 1.32982 0.703713609 AK5 no 91.2122 22.3458 0.0578722 4.08185 4.08185 1.90512610 AK7 no 104.001 80.4857 0.411225 1.29217 1.29217 0.823071611 AK8 no 150.372 60.8008 0.000134431 2.47318 2.47318 3.87639612 AK9 no 157.5 141.048 0.520198 1.11664 1.11664 0.643919613 AKAP1 no 1870.25 1485.31 0.0715677 1.25916 1.25916 1.80927614 AKAP10 no 492.617 506.331 0.713359 0.972914 -1.02784 -0.367752615 AKAP11 no 962.821 852.668 0.251789 1.12919 1.12919 1.14858616 AKAP12 no 729.121 446.373 0.36015 1.63344 1.63344 0.916705617 AKAP13 no 1798.01 2444.63 0.0143415 0.735493 -1.35963 -2.46523618 AKAP14 no 7.17931 3.01904 0.386763 2.37801 2.37801 0.866967619 AKAP16BP no 0.0157547 0.0107407 0.789169 1.46682 1.46682 0.267666620 AKAP17A_1 no 306.059 311.09 0.864095 0.983826 -1.01644 -0.171334621 AKAP17A_2 no 306.716 310.215 0.904836 0.988719 -1.01141 -0.119671622 AKAP2 no 200.595 269.24 0.487685 0.745041 -1.34221 -0.69499623 AKAP3 no 47.4201 25.0521 0.00010978 1.89286 1.89286 3.92831625 AKAP5 no 0.613046 0.874942 0.472705 0.700671 -1.4272 -0.719137626 AKAP6 no 1076.37 932.561 0.504569 1.15421 1.15421 0.66825627 AKAP7 no 225.932 164.639 0.253577 1.37229 1.37229 1.14425628 AKAP8 no 823.606 852.373 0.563948 0.966251 -1.03493 -0.577731629 AKAP8L no 1405.89 1170.34 0.00348334 1.20126 1.20126 2.94863630 AKAP9 no 1317.23 1077.97 0.0345967 1.22195 1.22195 2.12425631 AKIP1 no 339.94 476.844 0.00351192 0.712897 -1.40273 -2.94602632 AKIRIN1 no 1610.65 1123.92 4.55E-05 1.43306 1.43306 4.14828633 AKIRIN2 no 1311.54 996.527 0.000285243 1.31611 1.31611 3.67857634 AKNA no 690.867 766.365 0.515412 0.901486 -1.10928 -0.651328635 AKNAD1 no 11.2208 4.38847 0.000911033 2.55689 2.55689 3.35548636 AKR1A1 no 2875.81 3280.84 0.194963 0.876546 -1.14084 -1.2994637 AKR1B1 no 1559.57 1265.38 0.189365 1.23249 1.23249 1.31592638 AKR1B10 no 6226.74 2142.05 0.0167448 2.90691 2.90691 2.40788640 AKR1B10P2 no 0 0.00573124 0.629041 ? ? -0.483652641 AKR1B15 no 152.977 250.49 0.216366 0.610713 -1.63743 -1.23927642 AKR1B1P1 no 0 0 ? 1 1 0643 AKR1B1P2 no 0.0926062 0.0355422 0.0472211 2.60553 2.60553 1.99381644 AKR1B1P3 no 0.784073 0.336475 0.0217319 2.33025 2.33025 2.30899645 AKR1B1P4 no 0.0394017 0.0189887 0.363142 2.07501 2.07501 0.911001646 AKR1B1P5 no 0 0 ? 1 1 0647 AKR1B1P6 no 0.0172417 0.0432422 0.632491 0.398723 -2.50801 -0.478791649 AKR1B1P8 no 0 0.0034979 0.677829 ? ? -0.415898650 AKR1C1 no 7738.77 1938.57 0.0189735 3.99201 3.99201 2.36087651 AKR1C2 no 4442.63 3120.33 0.596588 1.42377 1.42377 0.529968652 AKR1C3 no 3550.07 1815.21 0.159529 1.95573 1.95573 1.41071653 AKR1C4 no 32.7574 9.87368 0.00891502 3.31765 3.31765 2.63523654 AKR1C5P no 1.57679 1.60586 0.993554 0.981894 -1.01844 -0.0080873655 AKR1C6P no 4.70274 8.7642 0.057095 0.536586 -1.86364 -1.9111657 AKR1CL1 no 1.74672 0.682113 0.000354454 2.56075 2.56075 3.61987658 AKR1D1 no 1.3548 1.09959 0.555591 1.2321 1.2321 0.590171659 AKR1D1P1 no 12.2819 14.9902 0.487414 0.819329 -1.22051 -0.695423660 AKR1E2 no 69.7811 148.636 0.000658905 0.469475 -2.13004 -3.44802661 AKR7A2 no 1146.76 1081.15 0.529787 1.06068 1.06068 0.629178662 AKR7A2P1 no 0 0.00185667 0.768013 ? ? -0.295284663 AKR7A3 no 104.135 167.732 0.442281 0.620843 -1.61071 -0.769529664 AKR7L no 17.2024 26.3243 0.420594 0.65348 -1.53027 -0.806675665 AKT1 no 4286.58 3544.34 0.0110532 1.20942 1.20942 2.55943666 AKT1S1 no 1234.01 1261.4 0.785669 0.978281 -1.0222 -0.272221667 AKT2 no 2266.44 2704.05 0.229746 0.838165 -1.19308 -1.20385668 AKT3 no 372.542 364.45 0.921778 1.0222 1.0222 0.0982904669 AKTIP no 521.677 509.498 0.783893 1.0239 1.0239 0.274535670 ALAD no 833.244 886.251 0.523109 0.94019 -1.06362 -0.639429671 ALAS1 no 1477.77 1034.44 1.18E-05 1.42857 1.42857 4.46783672 ALAS2 no 13.9433 10.8299 0.648818 1.28749 1.28749 0.455936673 ALB no 17.0352 64.7141 0.469666 0.263238 -3.79884 -0.724086674 ALCAM no 2182.68 2591.43 0.536766 0.84227 -1.18727 -0.618535675 ALDH16A1 no 972.457 1552.79 2.75E-05 0.626266 -1.59677 -4.26956676 ALDH18A1 no 3199.65 1520.69 4.95E-17 2.10408 2.10408 9.00067677 ALDH1A1 no 2844.44 2207.13 0.572488 1.28875 1.28875 0.56511678 ALDH1A2 no 630.462 324.743 0.256086 1.94142 1.94142 1.13821679 ALDH1A3 no 978.824 1345.16 0.418027 0.727662 -1.37427 -0.811145680 ALDH1B1 no 911.246 544.247 0.00305477 1.67432 1.67432 2.99036681 ALDH1L1 no 104.242 375.52 0.0722966 0.277594 -3.60238 -1.80461682 ALDH1L1-AS1 no 0 0.0274759 0.310715 ? ? -1.01571683 ALDH1L1-AS2 no 2.35632 5.4105 0.245663 0.435508 -2.29617 -1.16358684 ALDH1L2 no 182.768 165.535 0.803949 1.10411 1.10411 0.248494685 ALDH2 no 3094.96 2894.31 0.723928 1.06932 1.06932 0.353598686 ALDH3A1 no 3130.16 2115.45 0.405774 1.47966 1.47966 0.832712687 ALDH3A2 no 2431.74 2520.51 0.779405 0.964779 -1.03651 -0.280386688 ALDH3B1 no 509.106 674.462 0.385475 0.754833 -1.3248 -0.869323689 ALDH3B2 no 608.788 1135.75 0.0344758 0.536023 -1.86559 -2.12569690 ALDH4A1 no 619.973 984.912 0.0140023 0.62947 -1.58864 -2.47401691 ALDH5A1 no 599.201 488.615 0.114791 1.22633 1.22633 1.58234692 ALDH6A1 no 524.3 371.713 0.0214831 1.4105 1.4105 2.31342693 ALDH7A1 no 1760.26 1440.38 0.133464 1.22208 1.22208 1.50531694 ALDH7A1P1 no 0.151371 0.068613 0.0452353 2.20616 2.20616 2.01219695 ALDH7A1P2 no 0 0 ? 1 1 0696 ALDH7A1P3 no 0.0210637 0.0150932 0.803594 1.39557 1.39557 0.248953697 ALDH7A1P4 no 0 0.000956058 0.768013 ? ? -0.295284698 ALDH8A1 no 22.2447 204.166 0.780743 0.108954 -9.17817 -0.278641699 ALDH9A1 no 1696.02 1791.23 0.561265 0.946847 -1.05614 -0.581715700 ALDOA no 32843.3 31903.9 0.780239 1.02944 1.02944 0.279298701 ALDOAP1 no 0.0374071 0.0303427 0.77123 1.23282 1.23282 0.291071702 ALDOAP2 no 0 0.00677106 0.519912 ? ? -0.644361703 ALDOB no 140.376 241.52 0.855746 0.581219 -1.72052 -0.181974704 ALDOC no 649.763 665.372 0.903191 0.976542 -1.02402 -0.121751705 ALG1 no 878.385 696.667 0.00700307 1.26084 1.26084 2.71839706 ALG10 no 110.16 135.255 0.0874405 0.814457 -1.22781 -1.71555707 ALG10B no 285.961 297.086 0.73527 0.962553 -1.0389 -0.338488708 ALG11 no 168.802 149.668 0.320841 1.12784 1.12784 0.994638709 ALG12 no 572.854 619.678 0.364643 0.92444 -1.08174 -0.908151710 ALG13 no 504.027 650.408 0.0800866 0.77494 -1.29042 -1.75707711 ALG14 no 206.309 183.665 0.131036 1.12329 1.12329 1.51483712 ALG1L no 394.462 681.383 0.0429455 0.578914 -1.72737 -2.03425713 ALG1L12P no 16.1577 16.7076 0.883937 0.967085 -1.03404 -0.146124714 ALG1L14P no 0.901379 0.753768 0.514063 1.19583 1.19583 0.653424715 ALG1L15P no 1.36159 0.82823 0.00516463 1.64397 1.64397 2.82058716 ALG1L2 no 44.1313 44.883 0.913156 0.983251 -1.01703 -0.109165717 ALG1L3P no 7.09168 5.99926 0.718322 1.18209 1.18209 0.361096718 ALG1L5P no 2.26108 2.0133 0.729156 1.12307 1.12307 0.346623719 ALG1L6P no 114.531 97.7929 0.203841 1.17116 1.17116 1.2739720 ALG1L7P no 11.3318 9.42641 0.262498 1.20213 1.20213 1.12295721 ALG1L8P no 66.6384 47.9671 0.0341399 1.38925 1.38925 2.12969722 ALG1L9P no 81.1216 57.4576 0.00999932 1.41185 1.41185 2.59497723 ALG2 no 789.691 653.988 0.0305603 1.2075 1.2075 2.17467724 ALG3 no 1505.67 1529.45 0.889757 0.984454 -1.01579 -0.138748725 ALG3P1 no 0.0906948 0.096586 0.899858 0.939006 -1.06496 -0.125964726 ALG5 no 799.057 616.327 0.00235043 1.29648 1.29648 3.07234727 ALG6 no 496.647 609.327 0.0235915 0.815075 -1.22688 -2.27718728 ALG8 no 861.28 619.233 0.000178543 1.39088 1.39088 3.80272729 ALG9 no 511.463 343.667 3.09E-07 1.48825 1.48825 5.25507730 ALK no 44.0526 17.544 0.000120248 2.51097 2.51097 3.90503731 ALKBH1 no 248.764 254.174 0.807793 0.978713 -1.02175 -0.243523732 ALKBH2 no 356.818 427.475 0.091829 0.834711 -1.19802 -1.6921733 ALKBH3 no 368.492 323.036 0.278733 1.14071 1.14071 1.08545734 ALKBH3-AS1 no 5.52443 3.91986 0.0881218 1.40934 1.40934 1.71185735 ALKBH4 no 383.848 291.9 0.000111039 1.315 1.315 3.9254736 ALKBH5 no 2495.38 1987.8 0.0014967 1.25535 1.25535 3.20963737 ALKBH6 no 265.446 305.046 0.183426 0.870185 -1.14918 -1.33385738 ALKBH7 no 810.232 645.242 0.313104 1.2557 1.2557 1.0107739 ALKBH8 no 151.679 143.358 0.549402 1.05805 1.05805 0.599442740 ALLC no 3.44479 1.4099 0.019844 2.44328 2.44328 2.34382741 ALMS1 no 628.321 643.52 0.816712 0.976381 -1.02419 -0.232012742 ALMS1P no 3.87349 3.12703 0.659983 1.23871 1.23871 0.440444743 ALOX12 no 71.7606 148.729 0.173539 0.482493 -2.07257 -1.36468744 ALOX12B no 44.2006 208.197 0.157495 0.212302 -4.71028 -1.41765745 ALOX12P1 no 1.07403 1.20931 0.863501 0.888129 -1.12596 -0.17209746 ALOX12P2 no 77.6503 55.4734 0.0691762 1.39978 1.39978 1.82485747 ALOX15 no 73.0634 452.012 0.321347 0.161641 -6.18657 -0.993596748 ALOX15B no 73.7595 301.507 0.0721315 0.244636 -4.0877 -1.80567749 ALOX15P1 no 2.99583 4.78498 0.107264 0.62609 -1.59721 -1.61622750 ALOX15P2 no 0.82461 1.40194 0.776736 0.588193 -1.70012 -0.283872751 ALOX5 no 332.984 472.201 0.176572 0.705174 -1.41809 -1.35508752 ALOX5AP no 130.449 312.208 0.00205372 0.417828 -2.39333 -3.11389753 ALOXE3 no 18.4816 85.8555 0.0894395 0.215264 -4.64546 -1.70475754 ALOXE3P1 no 0.807335 4.42612 0.300797 0.182402 -5.48238 -1.0368755 ALPI no 16.4012 9.47033 0.514903 1.73186 1.73186 0.652118756 ALPK1 no 480.098 589.212 0.164097 0.814814 -1.22727 -1.39538757 ALPK2 no 205.227 55.3487 0.00491208 3.70789 3.70789 2.83713758 ALPK3 no 431.562 667.856 0.343425 0.64619 -1.54753 -0.949159760 ALPP no 646.206 236.349 0.265624 2.73412 2.73412 1.11561762 ALS2 no 582.036 469.264 0.0238599 1.24032 1.24032 2.27277763 ALS2CL no 818.538 2173.88 0.000107802 0.376534 -2.65581 -3.93294764 ALS2CR11 no 36.3585 27.5009 0.244157 1.32208 1.32208 1.16732765 ALS2CR12 no 13.8046 11.4619 0.201695 1.20439 1.20439 1.27999766 ALX1 no 13.8014 17.6648 0.76126 0.781297 -1.27992 -0.304148767 ALX3 no 199.674 194.319 0.812512 1.02756 1.02756 0.237429768 ALX4 no 26.5762 23.9593 0.852916 1.10922 1.10922 0.185584769 ALYREF no 2562.07 2016.51 0.0118277 1.27055 1.27055 2.53519770 AMACR no 796.772 370.893 1.07E-05 2.14825 2.14825 4.4912772 AMBP no 28.0411 18.9742 0.33509 1.47786 1.47786 0.96571773 AMBRA1 no 1946.87 1436.41 2.42E-05 1.35537 1.35537 4.29998774 AMD1 no 2394.32 1893.22 0.00218995 1.26469 1.26469 3.09417775 AMD1P1 no 0.205253 0.160049 0.576487 1.28244 1.28244 0.559232776 AMDHD1 no 14.5499 53.3413 0.159962 0.272769 -3.66611 -1.40925777 AMDHD2 no 406.327 448.418 0.409651 0.906135 -1.10359 -0.825846778 AMDP1_1 no 0 0.00498692 0.686878 ? ? -0.403548779 AMDP1_2 no 0.0315093 0.00853279 0.12825 3.69273 3.69273 1.52592781 AMELY no 0 0.00330115 0.678703 ? ? -0.414702782 AMER1 no 264.008 207.568 0.0705959 1.27192 1.27192 1.81555785 AMFR no 1858.03 1669.44 0.139325 1.11297 1.11297 1.48287786 AMH no 83.6234 154.187 0.121964 0.542352 -1.84382 -1.55166787 AMHR2 no 1.39512 1.9909 0.885508 0.700747 -1.42705 -0.144132788 AMICA1 no 99.6871 261.946 0.015088 0.380563 -2.62768 -2.44656789 AMIGO1 no 67.0458 47.9047 0.0327461 1.39957 1.39957 2.1467790 AMIGO2 no 515.795 439.434 0.601382 1.17377 1.17377 0.523057791 AMIGO3 no 0.0839017 0.107315 0.659969 0.781829 -1.27905 -0.440463792 AMMECR1 no 573.308 750.361 0.0407001 0.764043 -1.30883 -2.05687793 AMMECR1L no 745.252 639.066 0.022717 1.16616 1.16616 2.29185794 AMMECR1LP1 no 0 0.0008657 0.768013 ? ? -0.295284795 AMN no 163.022 225.779 0.51706 0.722042 -1.38496 -0.648773796 AMN1 no 253.144 166.95 0.00126884 1.51629 1.51629 3.25872797 AMOT no 1093.05 464.652 0.000157288 2.3524 2.3524 3.83576798 AMOTL1 no 1132.59 1463.64 0.430404 0.773819 -1.29229 -0.789737799 AMOTL2 no 1173.51 1843.63 0.0557673 0.636522 -1.57104 -1.92149800 AMPD1 no 3.51901 5.25603 0.352389 0.669518 -1.49361 -0.931642801 AMPD2 no 961.195 648.939 1.56E-08 1.48118 1.48118 5.83954802 AMPD3 no 230.124 517.713 3.86E-05 0.444501 -2.24971 -4.18806803 AMPH no 53.5954 29.5278 0.0255072 1.81508 1.81508 2.24662804 AMT no 279.776 181.711 0.017972 1.53967 1.53967 2.38136805 AMTN no 13.0092 306.73 0.236714 0.0424125 -23.5779 -1.18599806 AMY1A no 112.095 45.9038 0.324328 2.44196 2.44196 0.987483807 AMY1B no 435.935 132.46 0.290801 3.29108 3.29108 1.05853808 AMY1C no 94.9657 27.5934 0.0979695 3.44161 3.44161 1.66077809 AMY2A no 20.1702 7.49664 0.275762 2.69056 2.69056 1.0922810 AMY2B no 348.906 291.625 0.544302 1.19642 1.19642 0.607122811 AMYP1 no 0.0945281 0.0741997 0.797313 1.27397 1.27397 0.257091812 AMZ1 no 13.4884 13.631 0.968981 0.989536 -1.01057 -0.0389234813 AMZ2 no 1336.43 1245.06 0.355917 1.07339 1.07339 0.924827814 AMZ2P1 no 109.232 99.287 0.457662 1.10017 1.10017 0.743816815 AMZ2P2 no 0 0 ? 1 1 0816 ANAPC1 no 1374.89 999.924 3.45E-06 1.37499 1.37499 4.74506817 ANAPC10 no 224.671 210.765 0.417635 1.06597 1.06597 0.81183818 ANAPC10P1 no 0 0.00316523 0.676877 ? ? -0.417201819 ANAPC11 no 2173.66 1877.11 0.247375 1.15798 1.15798 1.15937820 ANAPC13 no 1157.74 1032.57 0.159547 1.12122 1.12122 1.41065821 ANAPC15 no 798.501 645.915 0.0545971 1.23623 1.23623 1.93082822 ANAPC16 no 2070.61 2039.57 0.829792 1.01521 1.01521 0.215186823 ANAPC1P1 no 7.59608 2.71674 0.00202196 2.79603 2.79603 3.11866824 ANAPC2 no 967.115 1019.8 0.492826 0.948334 -1.05448 -0.686796825 ANAPC4 no 823.291 630.945 0.00393938 1.30485 1.30485 2.9091826 ANAPC5 no 2813.32 2693.51 0.436782 1.04448 1.04448 0.778845827 ANAPC7 no 1631.02 1526.98 0.30666 1.06813 1.06813 1.02428828 ANG no 41.5929 94.6619 0.159281 0.439384 -2.27591 -1.41156829 ANGEL1 no 1011.87 639.163 2.75E-08 1.58312 1.58312 5.73224830 ANGEL2 no 514.885 515.305 0.991547 0.999187 -1.00081 -0.0106048831 ANGPT1 no 82.7954 32.7413 7.45E-05 2.52878 2.52878 4.02623832 ANGPT2 no 133.877 61.4528 0.00111349 2.17854 2.17854 3.29713833 ANGPT4 no 7.77821 16.6207 0.0570774 0.467984 -2.13683 -1.91124835 ANGPTL2 no 507.734 235.506 0.000170945 2.15592 2.15592 3.81408836 ANGPTL3 no 0.187671 0.248811 0.770428 0.75427 -1.32578 -0.29212837 ANGPTL4 no 572.751 974.373 0.171823 0.587814 -1.70122 -1.37016838 ANGPTL5 no 1.3362 1.87034 0.490314 0.714417 -1.39974 -0.690793839 ANGPTL6 no 25.8808 31.8507 0.177534 0.812566 -1.23067 -1.35207840 ANGPTL7 no 0.917163 0.364598 0.156291 2.51555 2.51555 1.42179841 ANHX no 0.0774693 0.0790445 0.972963 0.980071 -1.02033 -0.0339256842 ANK1 no 88.4781 227.323 0.121641 0.389217 -2.56926 -1.55301843 ANK2 no 377.171 169.085 0.00081474 2.23066 2.23066 3.38761844 ANK3 no 676.008 886.14 0.0926823 0.762868 -1.31084 -1.68765845 ANKAR no 85.836 85.05 0.93244 1.00924 1.00924 0.0848577846 ANKDD1A no 437.917 599.334 0.271224 0.730673 -1.3686 -1.1026847 ANKDD1B no 38.0165 25.4514 0.0620324 1.49369 1.49369 1.87417848 ANKEF1 no 243.854 249.397 0.832957 0.977776 -1.02273 -0.211123849 ANKFN1 no 20.8277 15.0906 0.428581 1.38017 1.38017 0.792867850 ANKFY1 no 1303.77 1403.74 0.384152 0.928782 -1.07668 -0.87175851 ANKH no 1356.07 1362.7 0.979758 0.995129 -1.00489 -0.0253967852 ANKHD1 no 1358.81 1685.54 0.0197244 0.806156 -1.24045 -2.34612853 ANKHD1-EIF4EBP3 no 909.43 747.309 0.0282355 1.21694 1.21694 2.20638854 ANKIB1 no 1187.28 1050.04 0.119612 1.1307 1.1307 1.56156855 ANKK1 no 19.9037 31.8309 0.137672 0.625295 -1.59924 -1.48912856 ANKLE1 no 64.8521 58.116 0.766656 1.11591 1.11591 0.297064857 ANKLE2 no 2048.36 2879.2 0.000326066 0.711433 -1.40561 -3.64251858 ANKMY1 no 311.202 292.827 0.569323 1.06275 1.06275 0.569776859 ANKMY2 no 353.02 356.876 0.9121 0.989193 -1.01093 -0.110497860 ANKRA2 no 302.65 339.97 0.299278 0.890225 -1.12331 -1.04007861 ANKRD1 no 17.9382 19.8198 0.911751 0.905062 -1.1049 -0.110938862 ANKRD10 no 2832.68 2308.5 0.129388 1.22707 1.22707 1.52137863 ANKRD11 no 2767.45 2748.33 0.928985 1.00696 1.00696 0.0892086864 ANKRD12 no 725.231 799.061 0.386866 0.907604 -1.1018 -0.866777865 ANKRD13A no 456.308 826.243 0.000111875 0.552269 -1.81071 -3.92349866 ANKRD13B no 336.563 222.568 0.0124547 1.51218 1.51218 2.51658867 ANKRD13C no 561.186 547.693 0.710484 1.02464 1.02464 0.371613868 ANKRD13D no 1151.6 1048.68 0.294163 1.09814 1.09814 1.05116869 ANKRD16 no 249.918 136.229 7.52E-15 1.83454 1.83454 8.26077870 ANKRD17 no 1835.91 1763.3 0.646582 1.04118 1.04118 0.459052871 ANKRD18A no 154.537 204.26 0.234219 0.756573 -1.32175 -1.19234873 ANKRD18CP no 29.7217 21.839 0.241833 1.36095 1.36095 1.1731874 ANKRD18DP no 1.47441 1.25635 0.616665 1.17356 1.17356 0.501188875 ANKRD18EP no 0.0522614 0.0452767 0.829135 1.15427 1.15427 0.21603876 ANKRD19P no 45.2892 73.5853 0.0917436 0.615465 -1.62479 -1.69255877 ANKRD2 no 12.4737 14.9823 0.592913 0.832565 -1.20111 -0.535283878 ANKRD20A1 no 11.096 15.6758 0.238212 0.707838 -1.41275 -1.1822880 ANKRD20A11P no 2.84859 12.7368 0.0767893 0.22365 -4.47126 -1.77671881 ANKRD20A12P no 15.5715 6.4114 0.00099991 2.42872 2.42872 3.32851882 ANKRD20A13P no 8.04127 3.50563 0.000447132 2.29382 2.29382 3.55626883 ANKRD20A14P no 6.5145 2.61348 3.80E-05 2.49266 2.49266 4.19176885 ANKRD20A16P no 1.31803 0.557892 8.72E-05 2.36252 2.36252 3.9866887 ANKRD20A18P no 4.92984 9.06437 0.252816 0.54387 -1.83867 -1.14609888 ANKRD20A19P no 31.0509 13.4691 0.5873 2.30534 2.30534 0.543432889 ANKRD20A2 no 5.95588 5.49534 0.804291 1.08381 1.08381 0.248051890 ANKRD20A20P no 2.29466 2.8868 0.433272 0.794881 -1.25805 -0.784827891 ANKRD20A3 no 5.83142 7.52189 0.350915 0.775261 -1.28989 -0.934503892 ANKRD20A4 no 4.3352 9.72808 0.0483203 0.445638 -2.24397 -1.98392893 ANKRD20A5P no 20.001 29.6062 0.50764 0.675567 -1.48024 -0.663439894 ANKRD20A6P no 0.218296 0.181944 0.677065 1.1998 1.1998 0.416944895 ANKRD20A7P no 1.49706 1.80914 0.706178 0.827497 -1.20846 -0.37741896 ANKRD20A8P no 3.9272 2.42605 0.284408 1.61876 1.61876 1.07269897 ANKRD20A9P no 0.702605 1.26218 0.293538 0.55666 -1.79643 -1.05253898 ANKRD22 no 445.32 613.639 0.161036 0.725703 -1.37797 -1.40562899 ANKRD23 no 528.118 369.54 0.000217811 1.42912 1.42912 3.75044900 ANKRD24 no 21.0779 32.9761 0.0791261 0.639188 -1.56448 -1.76273901 ANKRD26 no 222.694 217.927 0.832409 1.02187 1.02187 0.211826902 ANKRD26P1 no 2.27001 1.15412 5.64E-05 1.96688 1.96688 4.09526904 ANKRD26P3 no 4.3343 1.62013 8.80E-06 2.67528 2.67528 4.53532905 ANKRD26P4 no 0 0 ? 1 1 0906 ANKRD27 no 970.173 952.442 0.86779 1.01862 1.01862 0.166631907 ANKRD28 no 1101.85 942.128 0.064386 1.16953 1.16953 1.85742908 ANKRD29 no 117.194 167.324 0.260628 0.7004 -1.42776 -1.12737909 ANKRD30A no 2.54206 3.27358 0.847192 0.776536 -1.28777 -0.192896910 ANKRD30B no 5.02884 2.16775 0.151054 2.31984 2.31984 1.44007911 ANKRD30BL no 11104.6 7482.63 0.229871 1.48405 1.48405 1.20353912 ANKRD30BP1 no 0.204902 0.154744 0.637444 1.32414 1.32414 0.471832913 ANKRD30BP2 no 8.91641 3.72197 0.17843 2.39562 2.39562 1.34927914 ANKRD30BP3 no 55.7045 19.4028 0.0691309 2.87095 2.87095 1.82514915 ANKRD31 no 14.5721 8.74921 0.00974665 1.66553 1.66553 2.60399916 ANKRD32 no 264.085 357.213 0.0645131 0.739292 -1.35265 -1.85653918 ANKRD33B no 63.5063 219.989 0.0805243 0.28868 -3.46404 -1.75452919 ANKRD34A no 1.24354 1.24578 0.994611 0.998198 -1.00181 -0.00676128920 ANKRD34B no 94.4941 87.4062 0.865766 1.08109 1.08109 0.169207921 ANKRD34C no 0.811848 0.376677 0.239072 2.15529 2.15529 1.18003922 ANKRD35 no 201.292 318.861 0.303418 0.631285 -1.58407 -1.03118923 ANKRD36 no 198.907 224.07 0.430845 0.887701 -1.12651 -0.788981924 ANKRD36B no 151.396 173.054 0.379032 0.874844 -1.14306 -0.881189925 ANKRD36BP1 no 67.6902 86.5391 0.245938 0.782193 -1.27846 -1.16291926 ANKRD36BP2 no 43.9655 54.3055 0.487625 0.809597 -1.23518 -0.695086927 ANKRD36C no 79.5571 140.641 0.136153 0.565674 -1.7678 -1.49492928 ANKRD36P1 no 0.0739828 0.0458635 0.398942 1.61311 1.61311 0.844908929 ANKRD37 no 291.372 375.292 0.356902 0.776386 -1.28802 -0.922931930 ANKRD39 no 295.249 174.253 2.79E-07 1.69437 1.69437 5.27639931 ANKRD40 no 861.738 803.463 0.328363 1.07253 1.07253 0.979266932 ANKRD42 no 253.093 203.295 0.0951441 1.24495 1.24495 1.67498933 ANKRD44 no 184.24 269.691 0.0330369 0.683153 -1.4638 -2.1431934 ANKRD45 no 26.3595 29.484 0.881599 0.894029 -1.11853 -0.14909935 ANKRD46 no 534.566 317.204 7.59E-09 1.68524 1.68524 5.97467936 ANKRD49 no 253.81 288.606 0.225737 0.879436 -1.13709 -1.21431937 ANKRD50 no 538.869 723.411 0.0811265 0.7449 -1.34246 -1.75102938 ANKRD52 no 1698.71 1179.09 3.96E-05 1.4407 1.4407 4.18202939 ANKRD53 no 12.1973 11.1666 0.697121 1.09231 1.09231 0.389643940 ANKRD54 no 587.117 481.402 0.0104489 1.2196 1.2196 2.57941941 ANKRD54P1 no 0.220594 0.0509091 0.0160353 4.33309 4.33309 2.42401942 ANKRD55 no 15.2915 8.1544 0.0104129 1.87525 1.87525 2.58064943 ANKRD57P1 no 5.7752 4.825 0.482298 1.19693 1.19693 0.703626944 ANKRD6 no 209.612 207.923 0.967189 1.00812 1.00812 0.0411737945 ANKRD60 no 0.145995 0.150497 0.947966 0.970091 -1.03083 -0.065324946 ANKRD61 no 0.519752 0.292412 0.0181525 1.77746 1.77746 2.37759947 ANKRD62 no 1.33089 1.52543 0.803209 0.872472 -1.14617 -0.249452949 ANKRD63 no 0.277225 0.306904 0.916712 0.903296 -1.10706 -0.104678950 ANKRD65 no 311.82 681.804 0.00254127 0.457345 -2.18653 -3.04811951 ANKRD66 no 5.0513 1.83076 0.229024 2.75912 2.75912 1.20573953 ANKRD9 no 207.627 335.149 0.00782664 0.619506 -1.61419 -2.68033954 ANKS1A no 1128 1013.52 0.237719 1.11296 1.11296 1.18344955 ANKS1B no 76.2862 53.6841 0.23473 1.42102 1.42102 1.19103956 ANKS3 no 443.938 382.732 0.0632202 1.15992 1.15992 1.86565957 ANKS4B no 75.3181 50.7302 0.560753 1.48468 1.48468 0.582476958 ANKS6 no 822.744 612.372 0.00798205 1.34354 1.34354 2.67356959 ANKUB1 no 8.16857 12.1317 0.681627 0.673327 -1.48516 -0.410708960 ANKZF1 no 1072.49 829.287 0.0051749 1.29327 1.29327 2.81993961 ANLN no 1289.73 2001.47 0.0032619 0.644391 -1.55185 -2.96957962 ANO1 no 3541.24 1259.9 0.00779843 2.81072 2.81072 2.68158964 ANO10 no 950.738 515.306 1.02E-11 1.845 1.845 7.12636965 ANO2 no 25.8255 24.2089 0.784597 1.06678 1.06678 0.273617966 ANO3 no 34.3777 46.7692 0.791392 0.73505 -1.36045 -0.264776968 ANO5 no 22.4046 34.0819 0.533868 0.657376 -1.5212 -0.622946969 ANO6 no 973.798 1246.9 0.0299033 0.780974 -1.28045 -2.18341970 ANO7 no 87.178 37.2154 4.22E-05 2.34252 2.34252 4.1662971 ANO8 no 467.666 477.981 0.896013 0.978421 -1.02206 -0.130828972 ANO9 no 1034.62 779.044 0.0664955 1.32806 1.32806 1.84284973 ANP32A no 3634.63 2951.97 0.0102957 1.23125 1.23125 2.58465974 ANP32A-IT1 no 0 0 ? 1 1 0975 ANP32AP1 no 0.944634 0.718005 0.123492 1.31564 1.31564 1.54531976 ANP32B no 5088.89 5411.71 0.470473 0.940348 -1.06344 -0.72277977 ANP32BP1 no 0.599385 0.435723 0.16756 1.37561 1.37561 1.38397978 ANP32BP2 no 0.0567204 0.0193185 0.0993824 2.93607 2.93607 1.65379979 ANP32BP3 no 0 0.0391079 0.171633 ? ? -1.37077980 ANP32C no 0.0129226 0.00108121 0.0245954 11.9519 11.9519 2.2609981 ANP32D no 0.0478484 0.0814555 0.51405 0.587418 -1.70237 -0.653443982 ANP32E no 2527.38 2301.59 0.367249 1.0981 1.0981 0.90322983 ANPEP no 2398.89 1646.94 0.69576 1.45657 1.45657 0.391486984 ANTXR1 no 2061.3 1765.27 0.412355 1.1677 1.1677 0.821081985 ANTXR2 no 544.938 435.779 0.309698 1.25049 1.25049 1.01785986 ANTXRL no 0.990552 2.26113 0.287277 0.438079 -2.2827 -1.06631987 ANTXRLP1 no 1.83141 3.78925 0.0876132 0.483319 -2.06903 -1.71461988 ANXA1 no 10177.4 20454.2 0.00909532 0.497568 -2.00977 -2.62824989 ANXA10 no 22.8622 41.6651 0.598106 0.548713 -1.82245 -0.527777990 ANXA11 no 4110.74 5314.07 0.0249592 0.773558 -1.29273 -2.25515991 ANXA13 no 35.9947 18.4016 0.675386 1.95607 1.95607 0.419244992 ANXA2 no 18146.1 37031.2 2.66E-07 0.490022 -2.04072 -5.28559993 ANXA2P1 no 0 0.0465251 0.143395 ? ? -1.46772994 ANXA2P2 no 12.3061 24.6185 0.0325526 0.499874 -2.00051 -2.14911995 ANXA2P3 no 0.0715891 0.190245 0.135289 0.376299 -2.65746 -1.49824997 ANXA3 no 941.092 1525.2 0.0320381 0.617029 -1.62067 -2.15558998 ANXA4 no 2554.78 3769.58 0.245887 0.677734 -1.47551 -1.16303999 ANXA5 no 4871.98 5201.27 0.53707 0.936691 -1.06759 -0.6180731000 ANXA6 no 1803.99 1317.21 0.0941309 1.36955 1.36955 1.680161001 ANXA7 no 2123.76 2613.48 0.0096565 0.812616 -1.23059 -2.607261005 ANXA9 no 219.929 326.41 0.156064 0.673781 -1.48416 -1.422571006 AOAH no 278.041 179.162 0.0908646 1.5519 1.5519 1.697181007 AOAH-IT1 no 0 0 ? 1 1 01008 AOC1 no 962.908 591.244 0.353175 1.62861 1.62861 0.930121009 AOC2 no 56.5661 22.7313 3.77E-06 2.48847 2.48847 4.725451010 AOC3 no 205.273 126.411 0.150255 1.62386 1.62386 1.442911012 AOX1 no 27.0154 56.885 0.228258 0.474913 -2.10565 -1.207721013 AOX2P no 10.5074 4.79544 0.390225 2.19113 2.19113 0.8606541014 AP1AR no 508.493 469.582 0.411249 1.08286 1.08286 0.8230281015 AP1B1 no 3276.45 2964.13 0.164005 1.10537 1.10537 1.395681016 AP1B1P1 no 0.657439 0.466312 0.483721 1.40987 1.40987 0.701341017 AP1B1P2 no 0 0.00202852 0.768013 ? ? -0.2952841018 AP1G1 no 1806.87 1906.89 0.503743 0.947546 -1.05536 -0.6695471019 AP1G2 no 1664.29 2047.81 0.0284884 0.812717 -1.23044 -2.202821020 AP1M1 no 873.152 921.716 0.44549 0.94731 -1.05562 -0.7641231021 AP1M2 no 2016.59 1608.13 0.0199044 1.25399 1.25399 2.342661022 AP1S1 no 1576.28 1113.93 1.83E-05 1.41505 1.41505 4.366561023 AP1S2 no 1639.51 436.353 8.21E-19 3.75729 3.75729 9.582521024 AP1S3 no 340.541 349 0.888526 0.975762 -1.02484 -0.1403081025 AP2A1 no 2103.39 2470.35 0.0559272 0.851453 -1.17446 -1.920231026 AP2A2 no 1755.42 1363.91 0.12716 1.28705 1.28705 1.530311027 AP2B1 no 3879.15 3011.89 0.0782748 1.28794 1.28794 1.767781028 AP2M1 no 8885.23 11607.5 0.0202731 0.765474 -1.30638 -2.335651029 AP2S1 no 1917.89 2678.46 0.00524137 0.716045 -1.39656 -2.81571030 AP3B1 no 1291.81 1292.58 0.992593 0.9994 -1.0006 -0.009292831031 AP3B2 no 97.909 105.716 0.854808 0.926155 -1.07973 -0.1831691032 AP3D1 no 3559.01 3232.44 0.252618 1.10103 1.10103 1.146571033 AP3M1 no 1119.73 960.86 0.0208853 1.16534 1.16534 2.324261034 AP3M2 no 585.477 494.937 0.0535076 1.18293 1.18293 1.939651035 AP3S1 no 1250.87 1349.1 0.403538 0.927195 -1.07852 -0.836691036 AP3S2 no 122.671 100.629 0.0761717 1.21904 1.21904 1.780471037 AP4B1 no 491.197 485.381 0.917555 1.01198 1.01198 0.1036151038 AP4B1-AS1 no 14.1118 15.631 0.684757 0.902808 -1.10766 -0.4064371039 AP4E1 no 250.704 337.905 0.00485198 0.741936 -1.34782 -2.841191040 AP4M1 no 557.223 407.483 6.95E-05 1.36747 1.36747 4.043561041 AP4S1 no 304.797 157.05 8.69E-05 1.94077 1.94077 3.987531042 AP5B1 no 719.785 687.123 0.619523 1.04753 1.04753 0.4971251043 AP5M1 no 717.353 686.099 0.60139 1.04555 1.04555 0.5230451044 AP5S1 no 234.828 231.051 0.847437 1.01635 1.01635 0.1925831045 AP5Z1 no 887.975 808.521 0.142665 1.09827 1.09827 1.470411046 APAF1 no 485.019 537.927 0.349859 0.901644 -1.10908 -0.9365571047 APBA1 no 136.894 109.421 0.17993 1.25108 1.25108 1.34461048 APBA2 no 322.139 103.135 1.21E-08 3.12347 3.12347 5.887491049 APBA3 no 403.057 400.642 0.944149 1.00603 1.00603 0.07012451050 APBB1 no 347.818 182.987 0.000111461 1.90078 1.90078 3.924431051 APBB1IP no 99.555 188.335 0.0262346 0.528606 -1.89177 -2.235541052 APBB2 no 763.46 509.613 0.00120798 1.49812 1.49812 3.273221053 APBB3 no 347.801 307.104 0.233142 1.13252 1.13252 1.19511054 APC no 552.76 573.753 0.78314 0.963411 -1.03798 -0.2755151055 APC2 no 288.684 346.738 0.251295 0.832572 -1.2011 -1.149781057 APCDD1L no 55.9807 38.3757 0.3331 1.45875 1.45875 0.9697021058 APCDD1L-AS1 no 40.6792 34.0563 0.83244 1.19447 1.19447 0.2117871059 APCS no 0.0315093 0.733119 0.323516 0.0429799 -23.2667 -0.9891431060 APEH no 2685.86 1811.43 7.93E-08 1.48273 1.48273 5.527281061 APEX1 no 4195.29 2752.77 4.97E-09 1.52402 1.52402 6.05291062 APEX2 no 936.961 1130.94 0.0327644 0.82848 -1.20703 -2.146471063 APH1A no 5026.35 4514.92 0.108978 1.11328 1.11328 1.608341064 APH1B no 201.834 132.206 0.000818404 1.52667 1.52667 3.386321065 API5 no 2525.53 2115.45 0.00803998 1.19385 1.19385 2.671071066 API5P1 no 26.7234 20.4152 0.00282213 1.309 1.309 3.015321067 API5P2 no 0 0.00301178 0.698384 ? ? -0.3879331068 APIP no 450.131 422.492 0.677236 1.06542 1.06542 0.4167091069 APITD1 no 284.382 318.29 0.263773 0.893468 -1.11923 -1.119951070 APITD1-CORT no 105.698 110.994 0.74525 0.952291 -1.0501 -0.3252561071 APLF no 122.248 101.469 0.133137 1.20479 1.20479 1.506581072 APLN no 245.601 152.479 0.0932565 1.61072 1.61072 1.684671073 APLNR no 325.648 129.997 1.43E-06 2.50505 2.50505 4.935871074 APLP1 no 291.818 211.684 0.253228 1.37856 1.37856 1.145091075 APLP2 no 11130.7 7771.46 0.00123956 1.43225 1.43225 3.265611076 APMAP no 3518.29 2498.19 0.000469202 1.40833 1.40833 3.542951077 APOA1 no 32.3533 12.976 0.0826217 2.49332 2.49332 1.742421078 APOA1BP no 2345.99 1909.58 0.0261493 1.22854 1.22854 2.236821079 APOA2 no 24.7136 11.0646 0.265033 2.23358 2.23358 1.116991080 APOA4 no 0.158988 0.210944 0.87286 0.7537 -1.32679 -0.1601861081 APOA5 no 1.64033 0.507029 0.12417 3.23517 3.23517 1.542511082 APOB no 5.32242 13.561 0.478346 0.392479 -2.5479 -0.7099961083 APOBEC1 no 6.65885 4.33188 0.589847 1.53717 1.53717 0.5397291084 APOBEC2 no 8.73319 7.88874 0.554239 1.10704 1.10704 0.5921921085 APOBEC3A no 45.4562 664.444 0.0540258 0.0684124 -14.6172 -1.935431086 APOBEC3B no 499.275 926.642 0.015721 0.5388 -1.85598 -2.431361087 APOBEC3C no 300.522 519.37 0.00193824 0.578627 -1.72823 -3.131571088 APOBEC3D no 132.372 219.897 0.0159591 0.60197 -1.66121 -2.425781089 APOBEC3F no 255.951 281.653 0.48561 0.908748 -1.10042 -0.6983091090 APOBEC3G no 255.146 522.137 0.00755119 0.488658 -2.04642 -2.692641091 APOBEC3H no 10.9123 25.9704 0.00452541 0.42018 -2.37993 -2.864041092 APOBEC4 no 0.0829451 0.109144 0.903221 0.759962 -1.31586 -0.1217121093 APOBR no 244.681 273.484 0.564102 0.894679 -1.11772 -0.5775031094 APOC1 no 750.113 1186.34 0.249655 0.632291 -1.58155 -1.153781095 APOC1P1 no 1.24744 1.30323 0.889592 0.957196 -1.04472 -0.1389571096 APOC2 no 86.1789 148.445 0.191823 0.580545 -1.72252 -1.308621097 APOC3 no 0.836675 4.51302 0.489552 0.185391 -5.39399 -0.6920081098 APOC4 no 1.53205 1.55613 0.967221 0.984527 -1.01572 -0.04113381099 APOC4-APOC2 no 0.32202 0.262977 0.569936 1.22452 1.22452 0.5688721100 APOD no 338.871 227.014 0.540147 1.49273 1.49273 0.6134051101 APOE no 4567.93 4299.28 0.83706 1.06249 1.06249 0.2058621103 APOH no 3.84119 4.61272 0.855296 0.832739 -1.20086 -0.1825481104 APOL1 no 4213.48 11100.9 0.00852758 0.379563 -2.63461 -2.650691105 APOL2 no 1170.19 1929.83 0.0142844 0.606371 -1.64916 -2.46671106 APOL3 no 419.84 934.104 0.00522548 0.449457 -2.22491 -2.816711107 APOL4 no 522.773 603.876 0.558177 0.865696 -1.15514 -0.5863111108 APOL5 no 0.682714 0.516119 0.3456 1.32278 1.32278 0.9448821109 APOL6 no 973.985 2016.25 0.00028962 0.483067 -2.0701 -3.674481110 APOLD1 no 363.214 267.621 0.0265982 1.3572 1.3572 2.23011111 APOM no 62.8874 64.1779 0.863131 0.979891 -1.02052 -0.1725611112 APOO no 4698.43 4800.07 0.89694 0.978825 -1.02163 -0.1296551113 APOOL no 435.019 382.546 0.211926 1.13717 1.13717 1.251381114 APOOP1 no 0 0 ? 1 1 01115 APOOP2 no 0.0171063 0.0836793 0.724445 0.204427 -4.89173 -0.3529071116 APOOP3 no 0 0 ? 1 1 01117 APOOP4 no 0 0.0508151 0.251098 ? ? -1.150261118 APOOP5 no 0.102786 0.0597793 0.308124 1.71943 1.71943 1.021181119 APOPT1 no 707.455 550.163 0.00199989 1.2859 1.2859 3.122011120 APP no 17423.8 12351.5 0.00263818 1.41066 1.41066 3.036431121 APPBP2 no 771.367 731.881 0.535021 1.05395 1.05395 0.6211891122 APPL1 no 962.296 804.094 0.0915005 1.19675 1.19675 1.693831123 APPL2 no 963.933 1061.22 0.536749 0.908325 -1.10093 -0.6185611124 APRT no 2010.65 2056.43 0.823159 0.977737 -1.02277 -0.2237111125 APTX no 746.17 767.17 0.702488 0.972626 -1.02814 -0.3823861126 AQP1 no 849.788 1115.55 0.760136 0.761764 -1.31274 -0.3056241127 AQP10 no 1.03379 3.08274 0.289186 0.335348 -2.98198 -1.062091128 AQP11 no 82.6654 52.9392 5.53E-05 1.56152 1.56152 4.100391129 AQP12A no 11.3613 4.80393 0.450931 2.36499 2.36499 0.7550071130 AQP12B no 19.236 8.19082 0.35294 2.34848 2.34848 0.9305751131 AQP2 no 21.8272 17.4629 0.774439 1.24992 1.24992 0.2868731134 AQP4-AS1 no 26.0112 66.2198 0.33792 0.392801 -2.54582 -0.960061135 AQP5 no 1964.01 1608.6 0.725687 1.22094 1.22094 0.3512491136 AQP6 no 33.5415 36.7441 0.901294 0.912841 -1.09548 -0.1241491137 AQP7 no 12.9042 35.8113 0.481472 0.360339 -2.77517 -0.7049551138 AQP7P1 no 19.4073 15.6583 0.460543 1.23943 1.23943 0.7390551139 AQP7P2 no 1.61598 1.0094 0.0724339 1.60093 1.60093 1.803741140 AQP7P3 no 57.9504 70.0394 0.621301 0.827397 -1.20861 -0.4946011141 AQP7P4 no 1.14948 0.815146 0.295898 1.41015 1.41015 1.047391143 AQP9 no 88.9054 71.0681 0.490834 1.25099 1.25099 0.6899641144 AQPEP no 5.84152 9.07357 0.70395 0.643795 -1.55329 -0.3804131145 AQR no 1861.91 1800.44 0.670946 1.03414 1.03414 0.4253341146 AR no 469.249 156.463 0.00222348 2.9991 2.9991 3.089491147 ARAF no 1535.15 1540.1 0.97417 0.996783 -1.00323 -0.03240971148 ARAF2P no 0 0.00181195 0.768013 ? ? -0.2952841149 ARAF3P no 0 0 ? 1 1 01150 ARAP1 no 2198.28 2496.12 0.159487 0.880681 -1.13549 -1.410861151 ARAP2 no 328.189 851.993 4.86E-06 0.385202 -2.59604 -4.669341152 ARAP3 no 446.301 613.195 0.024918 0.727829 -1.37395 -2.25581153 ARC no 46.1761 26.078 0.144756 1.77069 1.77069 1.462721154 ARCN1 no 2964.94 2446.44 0.024415 1.21194 1.21194 2.263781155 AREG no 412.203 378.086 0.807021 1.09024 1.09024 0.2445211156 AREGB no 26.7895 47.721 0.343843 0.561377 -1.78133 -0.9483351157 AREL1 no 1054.8 921.394 0.116986 1.14479 1.14479 1.57281158 ARF1 no 10350.5 9812.73 0.483045 1.05481 1.05481 0.7024251159 ARF1P1 no 0.0914094 0.0259125 0.0268159 3.52762 3.52762 2.226871160 ARF1P2 no 0 0.00171182 0.768013 ? ? -0.2952841162 ARF3 no 4602.19 4228.93 0.145223 1.08826 1.08826 1.461021163 ARF4 no 3975.63 3259.93 0.111711 1.21955 1.21955 1.595991164 ARF4P1 no 0.137896 0.235339 0.548394 0.585948 -1.70664 -0.6009581165 ARF4P2 no 0.868763 0.797362 0.702051 1.08955 1.08955 0.3829761166 ARF4P3 no 0 0.0108313 0.50903 ? ? -0.6612651167 ARF4P4 no 0.202243 0.31343 0.762048 0.645257 -1.54977 -0.3031111168 ARF4P5 no 0 0.0281671 0.216533 ? ? -1.238821169 ARF5 no 4419.04 3335 0.000102989 1.32505 1.32505 3.944561170 ARF6 no 3266.69 4627.52 0.00017478 0.705928 -1.41658 -3.808291171 ARFGAP1 no 2289.95 1942.67 0.0785919 1.17877 1.17877 1.765891172 ARFGAP2 no 2520.57 1915.74 0.000351622 1.31572 1.31572 3.622051173 ARFGAP3 no 1371.37 1506.31 0.412293 0.910417 -1.0984 -0.821191174 ARFGEF1 no 2017.17 1539.5 0.000961128 1.31027 1.31027 3.339991175 ARFGEF2 no 1274.13 1174.95 0.449355 1.08441 1.08441 0.7576411176 ARFIP1 no 865.265 735.437 0.0687878 1.17653 1.17653 1.827421177 ARFIP2 no 1311.82 1072.93 0.0154348 1.22265 1.22265 2.438171178 ARFRP1 no 919.727 1066.04 0.323175 0.862751 -1.15908 -0.9898431179 ARG1 no 2.74558 4.96965 0.486398 0.552469 -1.81006 -0.6970491180 ARG2 no 760.413 603.424 0.179336 1.26016 1.26016 1.346451181 ARGFX no 0.99492 0.584755 0.0288081 1.70143 1.70143 2.198361182 ARGFXP1 no 0.143015 0.0457213 0.012963 3.12798 3.12798 2.50211183 ARGFXP2 no 0 0 ? 1 1 01184 ARGLU1 no 2186.54 2160.06 0.909359 1.01226 1.01226 0.1139591185 ARHGAP1 no 2426.52 2448.79 0.920821 0.990906 -1.00918 -0.0994961186 ARHGAP10 no 237.539 357.193 0.00323037 0.665015 -1.50372 -2.972651187 ARHGAP11A no 812.307 936.547 0.201345 0.867342 -1.15295 -1.280981188 ARHGAP11B no 25.4045 37.6404 0.0164127 0.674927 -1.48164 -2.415361189 ARHGAP12 no 673.037 618.272 0.538416 1.08858 1.08858 0.616031190 ARHGAP15 no 70.3664 133.838 0.027635 0.525759 -1.90201 -2.214941191 ARHGAP16P no 0 0 ? 1 1 01192 ARHGAP17 no 1115.52 845.725 1.06E-05 1.31901 1.31901 4.492651193 ARHGAP18 no 480.38 408.807 0.236617 1.17508 1.17508 1.186231194 ARHGAP19 no 437.911 386.593 0.240191 1.13274 1.13274 1.177211195 ARHGAP19-SLIT1 no 11.9853 5.02487 0.000299244 2.38519 2.38519 3.665681196 ARHGAP20 no 37.6 24.6739 0.218059 1.52388 1.52388 1.234711197 ARHGAP21 no 1359.23 1854.19 0.0264412 0.733059 -1.36415 -2.232441198 ARHGAP22 no 133.494 153.894 0.501669 0.867441 -1.15282 -0.6728091199 ARHGAP23 no 1485.64 3204.62 0.000662417 0.463592 -2.15707 -3.446521200 ARHGAP24 no 203.871 238.041 0.575455 0.856454 -1.1676 -0.5607471201 ARHGAP25 no 167.647 235.448 0.107142 0.712034 -1.40443 -1.616791202 ARHGAP26 no 722.123 953.494 0.39369 0.757344 -1.3204 -0.8543691203 ARHGAP26-AS1 no 0.0210637 0.0020615 0.0414627 10.2176 10.2176 2.049071204 ARHGAP27 no 939.498 1240.32 0.0542755 0.757466 -1.32019 -1.933411205 ARHGAP28 no 201.062 56.5733 5.57E-08 3.55401 3.55401 5.596241206 ARHGAP29 no 501.664 471.699 0.85965 1.06353 1.06353 0.1769961207 ARHGAP30 no 290.039 639.786 0.000800617 0.453337 -2.20586 -3.392621208 ARHGAP31 no 251.076 243.645 0.853851 1.0305 1.0305 0.1843921209 ARHGAP32 no 710.522 1087.85 0.0214493 0.653142 -1.53106 -2.314031210 ARHGAP33 no 341.182 451.796 0.199933 0.755167 -1.32421 -1.285021211 ARHGAP35 no 1887.95 2101.78 0.34459 0.89826 -1.11326 -0.9468661212 ARHGAP36 no 5.62053 2.01055 0.186493 2.79552 2.79552 1.324531213 ARHGAP39 no 583.402 351.129 3.79E-05 1.6615 1.6615 4.192261214 ARHGAP4 no 829.54 1986.72 2.46E-05 0.417542 -2.39497 -4.296151215 ARHGAP40 no 109.231 221.321 0.34479 0.493541 -2.02617 -0.9464721216 ARHGAP42 no 150.294 134.049 0.769449 1.12118 1.12118 0.2934031217 ARHGAP42P1 no 0.177538 0.0658727 0.294989 2.69516 2.69516 1.049361218 ARHGAP42P2 no 0.123673 0.0420679 0.0875163 2.93985 2.93985 1.715141219 ARHGAP42P3 no 5.96256 1.6345 3.41E-05 3.64795 3.64795 4.218031220 ARHGAP42P4 no 0 0.0398982 0.374486 ? ? -0.8896381221 ARHGAP42P5 no 0.0157547 0.0276413 0.829265 0.569968 -1.75449 -0.2158631222 ARHGAP44 no 191.7 125.948 0.10027 1.52206 1.52206 1.649441223 ARHGAP5 no 1594.3 1819.56 0.217189 0.876204 -1.14129 -1.237051224 ARHGAP5-AS1 no 80.646 94.3903 0.299341 0.854388 -1.17043 -1.039931225 ARHGAP6 no 83.2038 40.4389 0.0110935 2.05752 2.05752 2.558131226 ARHGAP8 no 631.212 402.615 0.00296845 1.56778 1.56778 2.999411227 ARHGAP9 no 132.19 277.5 0.0102997 0.476362 -2.09924 -2.584511228 ARHGDIA no 7309.68 5781.05 0.000207119 1.26442 1.26442 3.763731229 ARHGDIB no 1528 2137.44 0.0681764 0.714876 -1.39884 -1.831491230 ARHGDIG no 5.38457 5.12005 0.926301 1.05166 1.05166 0.09259031231 ARHGEF1 no 1657.8 2620.35 0.000293609 0.632664 -1.58062 -3.67081232 ARHGEF10 no 450.891 502.134 0.496473 0.897949 -1.11365 -0.6810111233 ARHGEF10L no 826.089 1149.82 0.0412847 0.718451 -1.39188 -2.050881234 ARHGEF11 no 1388.44 1277.28 0.400707 1.08703 1.08703 0.8417461235 ARHGEF12 no 2665.33 2434.3 0.374278 1.09491 1.09491 0.8900261236 ARHGEF15 no 149.508 72.7003 3.48E-06 2.05649 2.05649 4.742881237 ARHGEF16 no 1103.06 1357.47 0.152942 0.812589 -1.23064 -1.433431238 ARHGEF17 no 981.563 628.342 8.01E-05 1.56215 1.56215 4.00791239 ARHGEF18 no 1191.08 1236.42 0.672482 0.963334 -1.03806 -0.4232261240 ARHGEF19 no 1152.85 1258.5 0.523961 0.916051 -1.09164 -0.6381171241 ARHGEF2 no 1983.71 1329.17 1.45E-05 1.49245 1.49245 4.420921242 ARHGEF25 no 281.29 152.539 0.000242942 1.84406 1.84406 3.721471243 ARHGEF26 no 317.151 264.651 0.4326 1.19837 1.19837 0.7859771244 ARHGEF26-AS1 no 40.4327 12.9114 0.000321126 3.13154 3.13154 3.646641245 ARHGEF28 no 445.337 723.391 0.00603504 0.615624 -1.62437 -2.768681246 ARHGEF3 no 758.373 806.147 0.592129 0.940737 -1.063 -0.536421247 ARHGEF3-AS1 no 0 0.00306647 0.676318 ? ? -0.4179661248 ARHGEF33 no 65.6811 179.492 0.000302268 0.365928 -2.73278 -3.662981249 ARHGEF34P no 242.903 237.449 0.876999 1.02297 1.02297 0.1549271250 ARHGEF35 no 406.911 472.339 0.337357 0.861481 -1.16079 -0.9611821251 ARHGEF37 no 369.253 498.334 0.133703 0.740975 -1.34957 -1.504381252 ARHGEF38 no 176.221 103.034 0.14527 1.71032 1.71032 1.460851254 ARHGEF39 no 292.653 294.266 0.964335 0.994519 -1.00551 -0.04475741255 ARHGEF4 no 712.127 1381.18 0.0195671 0.515594 -1.93951 -2.349171256 ARHGEF40 no 848.51 644.274 0.0847824 1.317 1.317 1.730221257 ARHGEF5 no 1649.35 1696.35 0.796115 0.972292 -1.0285 -0.2586441258 ARHGEF6 no 177.061 212.892 0.350128 0.831695 -1.20236 -0.9360341259 ARHGEF7 no 1214.55 1077.32 0.157657 1.12739 1.12739 1.417091260 ARHGEF9 no 407.167 358.22 0.35235 1.13664 1.13664 0.9317191261 ARID1A no 2381.73 2474.57 0.867712 0.962481 -1.03898 -0.1667311262 ARID1B no 1594.83 1566.99 0.84198 1.01777 1.01777 0.1995611263 ARID2 no 818.83 724.581 0.137429 1.13007 1.13007 1.490051264 ARID3A no 342.01 101.925 2.59E-13 3.35551 3.35551 7.716771265 ARID3B no 348.838 239.832 0.000799048 1.45451 1.45451 3.393181266 ARID3C no 3.9648 4.41039 0.71752 0.898968 -1.11239 -0.362171267 ARID4A no 390.825 390.935 0.997568 0.999721 -1.00028 -0.003050481268 ARID4B no 989.138 920.694 0.369352 1.07434 1.07434 0.8992541269 ARID5A no 484.239 485.879 0.978367 0.996625 -1.00339 -0.02714271270 ARID5B no 1153.73 1227.36 0.591691 0.940012 -1.06382 -0.5370551271 ARIH1 no 1257.83 1325.68 0.467574 0.948824 -1.05394 -0.7275051272 ARIH2 no 1934.77 1481.67 2.61E-05 1.30581 1.30581 4.281761273 ARIH2OS no 27.2192 20.8364 0.0145899 1.30633 1.30633 2.458931274 ARIH2P1 no 0 0.0165478 0.41907 ? ? -0.8093271275 ARL1 no 1585.52 1342.28 0.0535051 1.18121 1.18121 1.939671276 ARL10 no 42.0582 41.4496 0.939595 1.01468 1.01468 0.07585191277 ARL11 no 52.228 52.0907 0.990584 1.00264 1.00264 0.01181351278 ARL13A no 22.4273 25.8859 0.322576 0.86639 -1.15421 -0.9910711279 ARL13B no 343.651 442.848 0.0611354 0.776002 -1.28866 -1.88071280 ARL14 no 26.3719 63.7963 0.212646 0.413376 -2.41911 -1.24941281 ARL14EP no 319.297 270.053 0.0675066 1.18235 1.18235 1.835981282 ARL14EPL no 117.04 89.9212 0.241178 1.30159 1.30159 1.174741283 ARL15 no 376.354 280.763 0.0265351 1.34047 1.34047 2.231041284 ARL16 no 687.085 508.771 0.000778629 1.35048 1.35048 3.400581285 ARL17A no 544.664 529.416 0.848245 1.0288 1.0288 0.1915491286 ARL17B no 407.314 430.712 0.764954 0.945677 -1.05744 -0.2992971287 ARL2 no 1406.12 1111.94 0.039978 1.26457 1.26457 2.064371288 ARL2-SNX15 no 1.79565 1.80309 0.979458 0.995874 -1.00414 -0.02577391289 ARL2BP no 993.989 1010.78 0.849188 0.983392 -1.01689 -0.1903451290 ARL2BPP1 no 0 0.0077095 0.594049 ? ? -0.5336391291 ARL2BPP10 no 6.78226 5.78988 0.149891 1.1714 1.1714 1.44421292 ARL2BPP2 no 0 0.00135458 0.768013 ? ? -0.2952841293 ARL2BPP3 no 0 0.0018747 0.677401 ? ? -0.4164841294 ARL2BPP4 no 0.0698099 0.0675153 0.982502 1.03399 1.03399 0.02195361295 ARL2BPP5 no 0 0 ? 1 1 01296 ARL2BPP6 no 0.0735055 0.0404246 0.316972 1.81834 1.81834 1.002641297 ARL2BPP7 no 0.0210637 0.00419583 0.102411 5.02015 5.02015 1.639081299 ARL2BPP9 no 0 0.00319689 0.683534 ? ? -0.4081041300 ARL3 no 853.21 586.662 0.000297878 1.45435 1.45435 3.666921301 ARL4A no 842.87 537.291 0.00176021 1.56874 1.56874 3.160841302 ARL4C no 1713.96 1071.74 0.00182932 1.59923 1.59923 3.149161303 ARL4D no 751.392 898.248 0.33212 0.836508 -1.19545 -0.9716731304 ARL4P no 0 0.00167108 0.768013 ? ? -0.2952841305 ARL4P2 no 0.164556 0.157681 0.916804 1.0436 1.0436 0.1045631306 ARL5A no 873.675 627.354 6.68E-05 1.39264 1.39264 4.053341307 ARL5AP1 no 0.0155072 0.00809132 0.582853 1.91652 1.91652 0.5499131308 ARL5AP2 no 0 0 ? 1 1 01309 ARL5AP4 no 9.83837 2.91788 0.0109681 3.37175 3.37175 2.562181310 ARL5B no 1010.1 824.552 0.042458 1.22503 1.22503 2.039071311 ARL5C no 1.055 0.57713 0.0274307 1.828 1.828 2.217881312 ARL6 no 153.773 147.622 0.723272 1.04167 1.04167 0.3544741313 ARL6IP1 no 6682.51 6300.84 0.565989 1.06058 1.06058 0.5747051314 ARL6IP1P1 no 0.0213091 0.0205976 0.974514 1.03454 1.03454 0.03197771315 ARL6IP1P2 no 0.128922 0.0561486 0.041592 2.29608 2.29608 2.047761316 ARL6IP1P3 no 0.0157547 0.0171019 0.942689 0.921225 -1.08551 -0.07195981317 ARL6IP4 no 3263.21 2863.19 0.319768 1.13971 1.13971 0.9968491318 ARL6IP5 no 1801.65 1856.59 0.755909 0.970406 -1.0305 -0.3111861319 ARL6IP6 no 556.87 673.051 0.0579494 0.827382 -1.20863 -1.904531320 ARL8A no 1196.79 973.843 0.00123168 1.22893 1.22893 3.267491321 ARL8B no 2027.86 2220.98 0.350997 0.913048 -1.09523 -0.9343431322 ARL9 no 44.6053 31.0543 0.201343 1.43637 1.43637 1.280991323 ARMC1 no 974.26 764.144 0.000521687 1.27497 1.27497 3.513521324 ARMC10 no 1117.25 750.113 5.25E-11 1.48944 1.48944 6.853451325 ARMC10P1 no 200.597 136.017 2.34E-07 1.47479 1.47479 5.311891326 ARMC12 no 21.3177 6.60246 1.49E-08 3.22875 3.22875 5.848971327 ARMC2 no 206.685 158.421 0.146653 1.30465 1.30465 1.455821328 ARMC3 no 130.297 141.011 0.912715 0.924018 -1.08223 -0.1097221329 ARMC4 no 18.7248 12.1318 0.210231 1.54345 1.54345 1.256051330 ARMC4P1 no 1.7865 0.995223 0.339695 1.79507 1.79507 0.9565321331 ARMC5 no 487.707 441.981 0.254379 1.10346 1.10346 1.142311332 ARMC6 no 832.907 762.185 0.352944 1.09279 1.09279 0.9305661333 ARMC7 no 455.098 374.847 0.0546595 1.21409 1.21409 1.930321334 ARMC8 no 822.162 962.864 0.0673942 0.853871 -1.17114 -1.836741335 ARMC9 no 503.967 318.711 0.000382346 1.58127 1.58127 3.599221336 ARMCX1 no 195.962 104.294 0.00107145 1.87893 1.87893 3.308381337 ARMCX2 no 784.601 304.614 0.00016945 2.57572 2.57572 3.816381338 ARMCX3 no 1224.6 831.625 0.0282687 1.47254 1.47254 2.205911339 ARMCX4 no 240.891 215.217 0.497959 1.11929 1.11929 0.678661340 ARMCX5 no 79.2634 69.7468 0.378264 1.13644 1.13644 0.8826131341 ARMCX5-GPRASP2 no 547.484 459.332 0.10204 1.19191 1.19191 1.640861342 ARMCX6 no 756.285 680.836 0.401119 1.11082 1.11082 0.8410081343 ARMS2 no 0.931402 0.524839 0.0171867 1.77464 1.77464 2.398141344 ARNT no 1109.34 1083.51 0.766804 1.02384 1.02384 0.2968691346 ARNTL no 206.67 291.454 0.00269261 0.709102 -1.41023 -3.030051347 ARNTL2 no 710.188 1587.85 0.00140072 0.447263 -2.23582 -3.22941348 ARPC1A no 3808.28 3487.29 0.228391 1.09205 1.09205 1.207371349 ARPC1B no 5760.27 5487.23 0.630913 1.04976 1.04976 0.4810131350 ARPC2 no 5341.19 6881.96 0.0270237 0.776114 -1.28847 -2.223811351 ARPC3 no 4920.09 5670.34 0.0574646 0.867689 -1.15249 -1.908251352 ARPC3P1 no 0.0210637 0.0178198 0.870011 1.18204 1.18204 0.1638061353 ARPC3P2 no 0.327165 0.387354 0.654316 0.844615 -1.18397 -0.4482941354 ARPC3P3 no 2.40836 2.47055 0.883404 0.974827 -1.02582 -0.14681355 ARPC3P4 no 0 0.00356366 0.676235 ? ? -0.418081356 ARPC3P5 no 0.403554 0.336371 0.882734 1.19973 1.19973 0.147651357 ARPC4 no 4191.92 3352.7 0.00075375 1.25031 1.25031 3.409851358 ARPC4-TTLL3 no 92.7025 67.3857 0.00237077 1.3757 1.3757 3.069671359 ARPC5 no 4124.75 5169.14 0.00154382 0.797956 -1.2532 -3.200341360 ARPC5L no 1696.54 1796.13 0.522849 0.944553 -1.0587 -0.6398291361 ARPP19 no 2458.44 2906.92 0.0431332 0.845719 -1.18243 -2.03241362 ARPP21 no 7.97044 3.11874 0.531674 2.55566 2.55566 0.6262941363 ARR3 no 12.0132 13.7568 0.375423 0.87326 -1.14513 -0.8878911364 ARRB1 no 460.423 392.935 0.475055 1.17175 1.17175 0.7153211365 ARRB2 no 771.76 976.437 0.0462561 0.790384 -1.26521 -2.002661366 ARRDC1 no 1470.16 1532.53 0.66321 0.959303 -1.04242 -0.4359861367 ARRDC2 no 512.911 977.171 0.000962433 0.524894 -1.90515 -3.33961368 ARRDC3 no 1614.87 2336 0.041464 0.691299 -1.44655 -2.049061369 ARRDC3-AS1 no 8.17082 8.05345 0.94383 1.01457 1.01457 0.07052491370 ARRDC4 no 419.016 515.914 0.365969 0.812182 -1.23125 -0.9056391371 ARRDC5 no 4.38534 9.1952 0.022481 0.476917 -2.0968 -2.29591372 ARSA no 1002.75 954.419 0.640521 1.05063 1.05063 0.4675191373 ARSB no 356.208 236.36 0.000179914 1.50705 1.50705 3.800721374 ARSD no 1605.8 1523.42 0.766485 1.05408 1.05408 0.2972881375 ARSDP1 no 0.0730932 0.0617377 0.760841 1.18393 1.18393 0.3046981377 ARSEP1 no 2.13831 0.736924 0.0666813 2.90166 2.90166 1.841571378 ARSF no 2.02333 26.1564 0.073673 0.0773549 -12.9274 -1.795921379 ARSFP1 no 0 0 ? 1 1 01380 ARSG no 184.338 243.026 0.153829 0.758511 -1.31837 -1.430331381 ARSH no 6.64878 6.44243 0.925185 1.03203 1.03203 0.09399561382 ARSI no 67.2376 284.546 0.021813 0.236298 -4.23194 -2.307551383 ARSJ no 255.925 296.281 0.630686 0.86379 -1.15769 -0.4813331384 ARSK no 159.223 162.377 0.846177 0.980577 -1.01981 -0.1941931385 ART1 no 8.50644 5.67038 0.553286 1.50015 1.50015 0.5936181386 ART2P no 0.130356 0.0701302 0.337106 1.85878 1.85878 0.9616831387 ART3 no 111.705 339.284 0.142622 0.329236 -3.03733 -1.470571388 ART4 no 1.17597 2.54627 0.736485 0.461841 -2.16525 -0.3368731389 ART5 no 19.2974 9.69229 0.11588 1.99101 1.99101 1.577591390 ARTN no 152.954 194.938 0.573795 0.784631 -1.27449 -0.5631871391 ARV1 no 426.351 424.408 0.965507 1.00458 1.00458 0.0432861392 ARVCF no 1020.95 617.088 0.00128727 1.65446 1.65446 3.254451394 AS3MT no 336.702 115.538 5.61E-09 2.91422 2.91422 6.030461395 ASAH1 no 2692.83 2303.29 0.178033 1.16912 1.16912 1.35051396 ASAH2 no 2.84335 1.94446 0.0139506 1.46228 1.46228 2.475361397 ASAH2B no 124.577 132.56 0.549266 0.939782 -1.06408 -0.5996471398 ASAH2C no 0.0835426 0.0895372 0.894086 0.933049 -1.07176 -0.1332661399 ASAP1 no 1027.78 963.307 0.5346 1.06693 1.06693 0.6218311400 ASAP1-IT1 no 0 0 ? 1 1 01401 ASAP1-IT2 no 0 0.00317977 0.675977 ? ? -0.4184331402 ASAP2 no 1278.79 1371.3 0.615219 0.932539 -1.07234 -0.5032461403 ASAP3 no 469.95 559.192 0.316097 0.84041 -1.1899 -1.004451404 ASB1 no 602.941 413.361 2.59E-06 1.45863 1.45863 4.808051406 ASB11 no 2.55888 1.47758 0.0673976 1.7318 1.7318 1.836721407 ASB12 no 9.0289 6.20751 0.31977 1.45451 1.45451 0.9968451408 ASB13 no 519.988 421.215 0.0215092 1.2345 1.2345 2.312951409 ASB14 no 230.582 190.42 0.131759 1.21091 1.21091 1.511981410 ASB15 no 1.13444 0.42292 0.000517094 2.68239 2.68239 3.515981411 ASB16 no 107.894 117.311 0.512549 0.919727 -1.08728 -0.6557791412 ASB16-AS1 no 115.823 140.591 0.130983 0.823834 -1.21384 -1.515041413 ASB17 no 0.38281 0.195406 0.0573466 1.95905 1.95905 1.909161414 ASB18 no 17.9206 12.798 0.292411 1.40027 1.40027 1.054991415 ASB2 no 129.269 292.975 0.00631548 0.44123 -2.26639 -2.75341416 ASB3 no 204.459 239.768 0.187437 0.852736 -1.1727 -1.321691417 ASB4 no 22.3469 8.70332 0.167398 2.56763 2.56763 1.38451418 ASB5 no 0.701128 0.921611 0.7672 0.760763 -1.31447 -0.2963511419 ASB6 no 767.685 818.378 0.314655 0.938057 -1.06603 -1.007461420 ASB7 no 375.243 390.498 0.615243 0.960935 -1.04065 -0.5032121421 ASB8 no 488.522 535.354 0.194936 0.912521 -1.09586 -1.299481422 ASB9 no 73.1549 47.1015 0.0799643 1.55313 1.55313 1.757791423 ASB9P1 no 2.22234 1.12732 0.00184435 1.97136 1.97136 3.146681424 ASCC1 no 514.39 423.602 0.003655 1.21432 1.21432 2.933231425 ASCC2 no 1868.95 1785.4 0.503132 1.04679 1.04679 0.6705061426 ASCC3 no 1003.5 1172.18 0.117228 0.856097 -1.16809 -1.571761427 ASCL1 no 11.9635 16.185 0.920098 0.73917 -1.35287 -0.1004081428 ASCL2 no 627.411 221.922 1.90E-05 2.82718 2.82718 4.357311429 ASCL3 no 1.41147 1.07233 0.37371 1.31627 1.31627 0.8910861430 ASCL4 no 0.583705 1.15 0.631585 0.507568 -1.97018 -0.4800671432 ASF1A no 383.194 274.462 0.000897147 1.39616 1.39616 3.359911433 ASF1B no 1029.99 1752.72 0.000166643 0.587656 -1.70168 -3.820731434 ASGR1 no 40.4328 21.509 0.000975334 1.8798 1.8798 3.335741435 ASGR2 no 6.51076 10.3195 0.0726141 0.630916 -1.585 -1.80261436 ASH1L no 1256.46 1488.87 0.12879 0.843904 -1.18497 -1.523751437 ASH1L-AS1 no 31.2808 36.5549 0.231067 0.855721 -1.16861 -1.200441438 ASH2L no 1218.77 1040.94 0.0653915 1.17083 1.17083 1.850421439 ASH2LP1 no 0 0.00151137 0.768013 ? ? -0.2952841440 ASIC1 no 205.901 157.613 0.151845 1.30637 1.30637 1.437281442 ASIC3 no 33.5082 38.1799 0.5929 0.877639 -1.13942 -0.5353031443 ASIC4 no 4.27423 2.87339 0.073405 1.48753 1.48753 1.79761445 ASIP no 39.1972 16.5508 7.05E-06 2.3683 2.3683 4.585671446 ASL no 811.697 801.872 0.939797 1.01225 1.01225 0.07559791447 ASLP1 no 0.522552 0.440331 0.432091 1.18672 1.18672 0.7868461448 ASMT_1 no 308.234 312.688 0.873021 0.985753 -1.01445 -0.1599811449 ASMT_2 no 310.181 311.595 0.959313 0.99546 -1.00456 -0.05106451452 ASMTL_1 no 481.641 377.045 0.0209061 1.27741 1.27741 2.323881453 ASMTL_2 no 489.065 376.983 0.0140667 1.29732 1.29732 2.472331450 ASMTL-AS1_1 no 59.3478 60.5696 0.880475 0.979829 -1.02059 -0.1505161451 ASMTL-AS1_2 no 60.2485 60.0862 0.984014 1.0027 1.0027 0.02005611454 ASNA1 no 1458.59 1492.98 0.784094 0.976963 -1.02358 -0.2742731455 ASNS no 1337.58 687.492 1.41E-06 1.9456 1.9456 4.93951456 ASNSD1 no 828.574 705.704 0.016551 1.17411 1.17411 2.412231459 ASNSP3 no 1.42197 0.755958 0.0214979 1.88102 1.88102 2.313161461 ASNSP5 no 0.225339 0.0692387 0.000398705 3.25452 3.25452 3.587761462 ASPA no 2.04959 4.08073 0.273777 0.50226 -1.991 -1.096731463 ASPDH no 4.82342 5.11793 0.793221 0.942455 -1.06106 -0.2624011464 ASPG no 40.5047 180.856 0.125592 0.223961 -4.46506 -1.536681465 ASPH no 2883.14 2945.24 0.863335 0.978916 -1.02154 -0.1723011466 ASPHD1 no 284.085 245.392 0.689186 1.15768 1.15768 0.4004091467 ASPHD2 no 173.362 189.29 0.604513 0.915851 -1.09188 -0.5185571468 ASPM no 865.002 1282.26 0.00350091 0.674589 -1.48238 -2.947031469 ASPN no 68.1271 110.741 0.536795 0.615191 -1.62551 -0.6184921470 ASPRV1 no 33.56 160.128 0.608615 0.209582 -4.7714 -0.5126761471 ASPSCR1 no 2492.72 746.025 1.82E-09 3.34133 3.34133 6.235381472 ASRGL1 no 1441.41 882.836 0.18332 1.63271 1.63271 1.334171473 ASS1 no 3591.55 7285.84 0.0228872 0.492948 -2.02861 -2.288961474 ASS1P1 no 0.24828 0.302835 0.593745 0.819851 -1.21973 -0.5340791475 ASS1P10 no 0.590827 1.12039 0.0959901 0.527339 -1.89631 -1.670691476 ASS1P11 no 0.171765 0.47538 0.401827 0.361321 -2.76762 -0.8397441477 ASS1P12 no 5.30144 6.29522 0.351724 0.842137 -1.18746 -0.9329311478 ASS1P13 no 0.339049 0.348583 0.936388 0.972649 -1.02812 -0.07988781479 ASS1P14 no 0.0649333 0.0579588 0.866861 1.12034 1.12034 0.1678141480 ASS1P2 no 0 0.00403914 0.619768 ? ? -0.4967771481 ASS1P3 no 0.177593 0.205231 0.788289 0.865333 -1.15562 -0.2688111482 ASS1P4 no 0 0.0044383 0.630501 ? ? -0.4815941483 ASS1P5 no 0.0849153 0.0839755 0.9829 1.01119 1.01119 0.02145451484 ASS1P6 no 0.0213091 0.00242927 0.0267656 8.77183 8.77183 2.227621486 ASS1P8 no 0 0.0109988 0.454615 ? ? -0.748871487 ASS1P9 no 0.0385508 0.173391 0.0595439 0.222335 -4.49771 -1.892471488 ASTE1 no 159.965 230.059 0.0035776 0.695322 -1.43818 -2.940091491 ASTN2 no 642.613 796.513 0.0795789 0.806783 -1.23949 -1.760051492 ASUN no 733.963 629.812 0.234424 1.16537 1.16537 1.191821493 ASXL1 no 1579.61 1487.05 0.609886 1.06225 1.06225 0.5108571494 ASXL2 no 385.655 442.01 0.362761 0.872502 -1.14613 -0.9117251495 ASXL3 no 52.225 22.49 0.129761 2.32214 2.32214 1.519881496 ASZ1 no 0.726645 0.760707 0.979543 0.955223 -1.04688 -0.02566631497 ATAD1 no 1040.28 916.996 0.108906 1.13444 1.13444 1.608671498 ATAD2 no 1471.5 2213.26 0.00120983 0.664855 -1.50409 -3.272771499 ATAD2B no 428.225 458.754 0.468502 0.933452 -1.07129 -0.7259881500 ATAD3A no 1490.06 1317.96 0.137461 1.13058 1.13058 1.489921501 ATAD3B no 1074.94 990.979 0.413984 1.08472 1.08472 0.818221502 ATAD3C no 121.342 137.972 0.648518 0.87947 -1.13705 -0.4563531503 ATAD5 no 257.007 337.235 0.0159842 0.762101 -1.31216 -2.42521504 ATAT1 no 506.804 319.421 3.73E-05 1.58663 1.58663 4.196181505 ATCAY no 4.31522 2.55553 0.166874 1.68858 1.68858 1.386211506 ATE1 no 610.16 591.963 0.714941 1.03074 1.03074 0.3656281507 ATF1 no 480.866 520.267 0.359793 0.924267 -1.08194 -0.9173881508 ATF1P1 no 0.039026 0.0150568 0.204538 2.59193 2.59193 1.271931509 ATF2 no 844.666 833.164 0.881334 1.01381 1.01381 0.1494251510 ATF3 no 726.013 2138.02 0.0773056 0.339572 -2.94488 -1.773591511 ATF4 no 8608.89 6607.05 0.000334922 1.30299 1.30299 3.635261512 ATF4P1 no 0.753709 0.798383 0.864919 0.944044 -1.05927 -0.1702851513 ATF4P2 no 0.714886 0.870366 0.534046 0.821363 -1.21749 -0.6226751514 ATF4P3 no 0.427796 0.244788 0.0577563 1.74762 1.74762 1.906011515 ATF4P4 no 2.82203 2.26823 0.200809 1.24416 1.24416 1.282511516 ATF5 no 1322.63 1393.86 0.788801 0.948894 -1.05386 -0.2681461517 ATF6 no 1163.42 1322.98 0.0622535 0.879392 -1.13715 -1.872581518 ATF6B no 2701.97 2720.67 0.943632 0.993127 -1.00692 -0.07077461519 ATF7 no 912.839 901.624 0.877431 1.01244 1.01244 0.154381520 ATF7IP no 1534.29 1368.08 0.144072 1.12149 1.12149 1.465231521 ATF7IP2 no 302.702 411.639 0.133582 0.735358 -1.35988 -1.504851522 ATG10 no 250.983 235.836 0.392195 1.06422 1.06422 0.8570771523 ATG12 no 791.602 806.882 0.753792 0.981063 -1.0193 -0.3139761524 ATG12P1 no 0.0187606 0.00819622 0.401646 2.28893 2.28893 0.8400661525 ATG12P2 no 0 0 ? 1 1 01526 ATG13 no 1723.18 1389.84 0.000825058 1.23984 1.23984 3.3841527 ATG14 no 475.506 511.255 0.419267 0.930076 -1.07518 -0.8089841528 ATG16L1 no 693.463 583.367 0.0257607 1.18873 1.18873 2.242731529 ATG16L2 no 643.268 972.07 0.00202083 0.661751 -1.51114 -3.118831530 ATG2A no 903.722 815.53 0.190691 1.10814 1.10814 1.311971531 ATG2B no 586.059 598.628 0.867178 0.979005 -1.02145 -0.167411532 ATG3 no 1105.21 1432.12 0.00631982 0.771728 -1.29579 -2.753171533 ATG3P1 no 0 0.0049017 0.609424 ? ? -0.5115181534 ATG4A no 275.324 382.527 0.00419834 0.719751 -1.38937 -2.888481535 ATG4B no 1566.6 1221.02 0.000905518 1.28303 1.28303 3.357231536 ATG4C no 240.002 255.296 0.402118 0.940093 -1.06372 -0.8392231537 ATG4D no 775.107 681.249 0.321893 1.13777 1.13777 0.9924741538 ATG5 no 945.212 888.967 0.420171 1.06327 1.06327 0.8074111539 ATG7 no 1336.29 1198.82 0.152014 1.11467 1.11467 1.436691540 ATG9A no 1678.65 1495.59 0.139411 1.1224 1.1224 1.482551541 ATG9B no 147.875 297.938 0.178939 0.496327 -2.0148 -1.347681542 ATHL1 no 2389.92 2724.14 0.623396 0.877309 -1.13985 -0.4916321543 ATIC no 2207.37 1498.16 1.11E-05 1.47338 1.47338 4.482741544 ATL1 no 284.838 162.776 1.68E-05 1.74988 1.74988 4.38581545 ATL2 no 1392.91 1351.03 0.771333 1.031 1.031 0.2909361546 ATL3 no 1649.41 2097.38 0.0041264 0.786415 -1.27159 -2.894091547 ATM no 585.423 543.976 0.572585 1.07619 1.07619 0.5649681548 ATMIN no 1209.78 1267.94 0.507367 0.954132 -1.04807 -0.6638651549 ATN1 no 4323.12 3071.62 4.38E-06 1.40744 1.40744 4.692111551 ATOH7 no 5.27747 4.24549 0.690579 1.24308 1.24308 0.3985151552 ATOH8 no 99.6837 55.6489 0.0615292 1.7913 1.7913 1.877821553 ATOX1 no 894.495 900.343 0.964936 0.993505 -1.00654 -0.04400331554 ATP10A no 138.252 111.999 0.533071 1.2344 1.2344 0.6241621555 ATP10B no 584.426 370.167 0.218566 1.57882 1.57882 1.233341556 ATP10D no 317.654 581.658 0.00119158 0.546118 -1.83111 -3.277241557 ATP11A no 1757.87 1121.81 0.0121104 1.56699 1.56699 2.52671558 ATP11A-AS1 no 0 0 ? 1 1 01559 ATP11B no 1389.66 2206.87 0.00393591 0.629698 -1.58806 -2.909391560 ATP11C no 450.618 442.561 0.88949 1.0182 1.0182 0.1390861561 ATP12A no 134.114 48.1655 0.130046 2.78445 2.78445 1.518751562 ATP13A1 no 1848.44 1512.86 0.00166122 1.22182 1.22182 3.178321563 ATP13A2 no 1753.85 1331.83 0.00249951 1.31687 1.31687 3.053261564 ATP13A3 no 2711.67 3057.46 0.402563 0.886903 -1.12752 -0.8384281565 ATP13A4 no 66.2849 159.63 0.159003 0.41524 -2.40825 -1.41251566 ATP13A5 no 10.5761 60.0436 0.460994 0.176141 -5.67728 -0.7383111567 ATP1A1 no 22780.6 18556.4 0.0147633 1.22764 1.22764 2.454581568 ATP1A1OS no 37.9676 23.6418 3.01E-05 1.60595 1.60595 4.247911570 ATP1A3 no 67.1329 40.6119 0.147189 1.65304 1.65304 1.453881571 ATP1A4 no 3.28988 2.09696 0.0582646 1.56888 1.56888 1.902121572 ATP1B1 no 3841.79 5458.8 0.168334 0.703779 -1.4209 -1.381441573 ATP1B1P1 no 0 0 ? 1 1 01575 ATP1B3 no 5375.72 7885.51 0.0519213 0.681722 -1.46687 -1.952791576 ATP1B3P1 no 0.567277 1.13661 0.0692451 0.499094 -2.00363 -1.824391577 ATP1B4 no 0.203069 0.116371 0.200162 1.74502 1.74502 1.284371578 ATP2A1 no 98.4152 92.5403 0.696402 1.06349 1.06349 0.3906161579 ATP2A2 no 6834.1 8328.34 0.04824 0.820585 -1.21864 -1.984641580 ATP2A3 no 790.51 1339.27 0.393681 0.590255 -1.69418 -0.8543861581 ATP2B1 no 1690.88 1127.43 0.0221377 1.49977 1.49977 2.301851583 ATP2B3 no 30.0296 12.8308 0.126572 2.34043 2.34043 1.532691584 ATP2B4 no 3583.86 3295.16 0.535442 1.08761 1.08761 0.6205491585 ATP2C1 no 3641.63 3737.51 0.826102 0.974346 -1.02633 -0.2199271586 ATP2C2 no 534.499 744.041 0.273773 0.718373 -1.39203 -1.096741587 ATP4A no 4.40826 2.28099 0.571881 1.93261 1.93261 0.5660041588 ATP4B no 3.24582 3.37625 0.944645 0.961366 -1.04019 -0.06951589 ATP5A1 no 9797.34 7976.87 0.00264772 1.22822 1.22822 3.035311590 ATP5A1P1 no 2.28935 3.94037 0.276225 0.580998 -1.72118 -1.091141591 ATP5A1P10 no 0.156485 0.639384 0.179019 0.244743 -4.08591 -1.347431592 ATP5A1P2 no 0.132594 0.0927654 0.382547 1.42934 1.42934 0.8747011593 ATP5A1P3 no 0.502399 0.731598 0.307409 0.686714 -1.45621 -1.022691594 ATP5A1P4 no 0.0421275 0.00782548 0.0618552 5.38338 5.38338 1.875451595 ATP5A1P5 no 0.236531 0.65335 0.118948 0.362029 -2.76221 -1.564391596 ATP5A1P6 no 0.0824598 0.303252 0.0803118 0.271919 -3.67757 -1.755761597 ATP5A1P7 no 0.0372853 0.358383 0.0169592 0.104038 -9.61191 -2.403131598 ATP5A1P8 no 0.170567 0.324193 0.241406 0.526127 -1.90068 -1.174171599 ATP5A1P9 no 0.0962185 0.318935 0.0497986 0.301687 -3.31469 -1.970911600 ATP5B no 13939.5 11726.7 0.0197748 1.1887 1.1887 2.345151601 ATP5BP1 no 0 0 ? 1 1 01602 ATP5C1 no 4141.57 3239.95 0.000122281 1.27828 1.27828 3.900741603 ATP5C1P1 no 0.460324 0.173808 0.000193389 2.64846 2.64846 3.781781604 ATP5D no 1593.86 1395.71 0.509726 1.14197 1.14197 0.6601791605 ATP5E no 5658.37 5266.23 0.624884 1.07446 1.07446 0.4895261606 ATP5EP1 no 0.0502345 0.0589072 0.862461 0.852774 -1.17264 -0.1734151607 ATP5EP2 no 5.51695 5.19883 0.807631 1.06119 1.06119 0.2437331608 ATP5F1 no 3388.22 3080.73 0.125895 1.09981 1.09981 1.535451609 ATP5F1P1 no 0.0203008 0 0.00065245 ? ? 3.45081611 ATP5F1P3 no 0.581682 0.0770012 0.0157515 7.55419 7.55419 2.430641612 ATP5F1P4 no 0 0.00143247 0.768013 ? ? -0.2952841613 ATP5F1P5 no 5.87361 3.27062 0.0758436 1.79587 1.79587 1.782471614 ATP5F1P6 no 0.019358 0.00421765 0.135266 4.58977 4.58977 1.498331615 ATP5F1P7 no 0.0185835 0.00523944 0.199792 3.54685 3.54685 1.285431616 ATP5G1 no 1954.85 1816.86 0.526444 1.07595 1.07595 0.6343021617 ATP5G1P1 no 0.153789 0.0778379 0.19076 1.97576 1.97576 1.311771618 ATP5G1P2 no 0 0.0135738 0.424008 ? ? -0.8007541619 ATP5G1P3 no 0.5248 0.370033 0.464913 1.41825 1.41825 0.7318651620 ATP5G1P4 no 14.9513 20.4596 0.199443 0.730773 -1.36841 -1.286431621 ATP5G1P5 no 0.0187252 0.0188711 0.994397 0.992267 -1.00779 -0.007029311622 ATP5G1P6 no 0 0 ? 1 1 01623 ATP5G1P7 no 0 0 ? 1 1 01624 ATP5G1P8 no 0 0.00686897 0.522378 ? ? -0.6405551625 ATP5G2 no 5673.48 5297.1 0.431784 1.07105 1.07105 0.7873731626 ATP5G2P1 no 0 0.0567294 0.318775 ? ? -0.9989021627 ATP5G2P2 no 0.019358 0.0171517 0.907961 1.12864 1.12864 0.1157241628 ATP5G2P3 no 0 0 ? 1 1 01629 ATP5G2P4 no 0.247883 0.112718 0.0258997 2.19915 2.19915 2.240611630 ATP5G3 no 4389.04 3776.54 0.096996 1.16219 1.16219 1.665631631 ATP5H no 3122.66 3061.47 0.844738 1.01999 1.01999 0.1960331632 ATP5HP1 no 0 0.00295081 0.676105 ? ? -0.4182581633 ATP5I no 2035.17 2120.47 0.792088 0.959775 -1.04191 -0.2638731634 ATP5J no 1827.37 1710.17 0.47732 1.06853 1.06853 0.7116541635 ATP5J2 no 3246.76 2547.73 0.00882895 1.27437 1.27437 2.638611636 ATP5J2-PTCD1 no 118.75 86.891 0.00589349 1.36666 1.36666 2.776641637 ATP5J2LP no 0 0 ? 1 1 01638 ATP5J2P2 no 0.0959111 0.0565089 0.385983 1.69727 1.69727 0.8683931639 ATP5J2P3 no 0.0171063 0.0112812 0.824553 1.51635 1.51635 0.2219181640 ATP5J2P4 no 0 0.00186741 0.768013 ? ? -0.2952841641 ATP5J2P5 no 0.230197 0.295531 0.624203 0.778926 -1.28382 -0.4904891642 ATP5J2P6 no 0 0 ? 1 1 01643 ATP5JP1 no 0 0.00195845 0.768013 ? ? -0.2952841644 ATP5L no 3090.69 2638.24 0.0809377 1.1715 1.1715 1.752111645 ATP5L2 no 0 0 ? 1 1 01646 ATP5LP1 no 1.75283 1.59852 0.702768 1.09653 1.09653 0.3820091647 ATP5LP2 no 0.0155072 0.00288528 0.126343 5.37459 5.37459 1.533621648 ATP5LP3 no 0.092724 0.0798943 0.786724 1.16058 1.16058 0.2708481649 ATP5LP4 no 0 0.00121002 0.768013 ? ? -0.2952841650 ATP5LP5 no 0.28236 0.232199 0.648265 1.21603 1.21603 0.4567061651 ATP5LP6 no 0 0 ? 1 1 01652 ATP5LP8 no 0 0 ? 1 1 01653 ATP5O no 3415.7 3219.14 0.489864 1.06106 1.06106 0.6915111654 ATP5S no 397.96 449.676 0.166358 0.884993 -1.12995 -1.387911655 ATP5SL no 801.567 856.429 0.431868 0.935941 -1.06844 -0.7872291656 ATP6AP1 no 4113.34 3393.25 0.030109 1.21221 1.21221 2.180661657 ATP6AP1L no 74.578 46.8087 0.00105058 1.59325 1.59325 3.314121658 ATP6AP2 no 2860.65 3226.51 0.201146 0.886608 -1.12789 -1.281551659 ATP6V0A1 no 1293.75 1043.12 0.00377894 1.24027 1.24027 2.922511660 ATP6V0A2 no 627.43 461.225 1.97E-06 1.36036 1.36036 4.86661661 ATP6V0A4 no 71.88 65.7295 0.899319 1.09357 1.09357 0.1266461662 ATP6V0B no 3454.81 2778.87 0.00600762 1.24324 1.24324 2.770211663 ATP6V0C no 3676 3499.46 0.482643 1.05045 1.05045 0.7030721664 ATP6V0CP1 no 0 0 ? 1 1 01665 ATP6V0CP3 no 0.503138 0.458681 0.802125 1.09692 1.09692 0.2508551666 ATP6V0D1 no 2087.79 2148.29 0.725763 0.971834 -1.02898 -0.3511481667 ATP6V0D2 no 16.6593 11.0704 0.12406 1.50485 1.50485 1.542971668 ATP6V0E1 no 3431.22 2905.69 0.00946585 1.18086 1.18086 2.614261669 ATP6V0E1P1 no 0 0 ? 1 1 01670 ATP6V0E1P2 no 0 0 ? 1 1 01671 ATP6V0E1P3 no 0.0941708 0.0723059 0.682188 1.30239 1.30239 0.4099411672 ATP6V0E1P4 no 0.0315093 0.0215044 0.665249 1.46525 1.46525 0.4331741673 ATP6V0E2 no 1148.08 519.13 4.73E-09 2.21154 2.21154 6.061871674 ATP6V0E2-AS1 no 43.8942 29.4404 0.0418344 1.49095 1.49095 2.045311675 ATP6V1A no 1789.54 2007.22 0.260709 0.89155 -1.12164 -1.127181676 ATP6V1B1 no 487.114 294.135 0.381642 1.65609 1.65609 0.8763691677 ATP6V1B2 no 1987.42 1802.78 0.305976 1.10242 1.10242 1.025731678 ATP6V1C1 no 1439.43 1065.53 4.08E-05 1.3509 1.3509 4.174331679 ATP6V1C2 no 254.381 203.683 0.613912 1.24891 1.24891 0.5051091680 ATP6V1D no 1153.39 1395.42 0.0190913 0.826558 -1.20984 -2.358521681 ATP6V1E1 no 2138.56 2225.1 0.54213 0.961109 -1.04046 -0.6104041682 ATP6V1E1P1 no 0.111881 0.238645 0.678084 0.468819 -2.13302 -0.4155491683 ATP6V1E1P2 no 0 0 ? 1 1 01684 ATP6V1E1P3 no 0 0.0100224 0.451331 ? ? -0.754341685 ATP6V1E2 no 192.275 129.524 0.000744854 1.48447 1.48447 3.413231686 ATP6V1F no 2394.53 2080.52 0.0657777 1.15093 1.15093 1.847761687 ATP6V1G1 no 2869.87 2209.52 0.00203959 1.29886 1.29886 3.1161688 ATP6V1G1P1 no 0 0.0008657 0.768013 ? ? -0.2952841689 ATP6V1G1P2 no 0.360204 0.443572 0.875029 0.812054 -1.23144 -0.1574291690 ATP6V1G1P3 no 0.0235391 0.0024291 0.0198827 9.6905 9.6905 2.343071691 ATP6V1G1P4 no 0 0 ? 1 1 01692 ATP6V1G1P5 no 0 0.00455712 0.613175 ? ? -0.5061591693 ATP6V1G1P6 no 0 0 ? 1 1 01694 ATP6V1G1P7 no 0 0.00196268 0.684004 ? ? -0.4074641695 ATP6V1G2 no 13.9622 8.07776 0.00519225 1.72848 1.72848 2.818821696 ATP6V1G2-DDX39B no 2.95529 2.70204 0.723129 1.09373 1.09373 0.3546651698 ATP6V1H no 1358.41 1003.57 1.24E-05 1.35358 1.35358 4.456651699 ATP7A no 408.724 410.846 0.966661 0.994835 -1.00519 -0.04183631700 ATP7B no 621.152 333.28 0.0842648 1.86375 1.86375 1.733121701 ATP7BP1 no 0 0 ? 1 1 01702 ATP8A1 no 311.375 203.308 0.0697021 1.53154 1.53154 1.821381703 ATP8A2 no 40.7683 24.7593 0.183468 1.64659 1.64659 1.333721704 ATP8A2P1 no 0.285889 0.488626 0.740214 0.585087 -1.70915 -0.3319261705 ATP8A2P2 no 0 0.00442064 0.678458 ? ? -0.4150371706 ATP8A2P3 no 2.49121 1.29101 0.00535773 1.92967 1.92967 2.808421707 ATP8B1 no 1593.91 1667.36 0.757709 0.955951 -1.04608 -0.3088171708 ATP8B2 no 540.42 327.721 0.00495822 1.64902 1.64902 2.834051709 ATP8B3 no 57.8714 87.4222 0.28094 0.661976 -1.51063 -1.080461710 ATP8B4 no 34.5559 64.6423 0.00792555 0.534571 -1.87066 -2.676011711 ATP8B5P no 9.81537 18.4323 0.441502 0.532509 -1.8779 -0.7708441712 ATP9A no 2109.9 1588.79 0.0539083 1.328 1.328 1.936391713 ATP9B no 407.349 409.534 0.948396 0.994665 -1.00536 -0.06478431714 ATPAF1 no 1096.98 868.01 0.0012929 1.26379 1.26379 3.253171715 ATPAF2 no 365.323 284.593 0.000925156 1.28367 1.28367 3.351031716 ATPIF1 no 2617.66 2275.68 0.0940675 1.15028 1.15028 1.680491717 ATR no 792.584 895.406 0.216416 0.885167 -1.12973 -1.239141718 ATRAID no 2333.02 2145.09 0.355031 1.08761 1.08761 0.9265341719 ATRIP no 587.746 774.299 0.0095659 0.759068 -1.3174 -2.610571720 ATRN no 1494.2 1332.35 0.282059 1.12148 1.12148 1.077951721 ATRNL1 no 60.6664 30.8638 0.234709 1.96562 1.96562 1.191091722 ATRX no 1308.99 1182.28 0.296055 1.10717 1.10717 1.047041723 ATXN1 no 855.878 716.273 0.13086 1.19491 1.19491 1.515521724 ATXN10 no 2301.13 2086.17 0.392797 1.10304 1.10304 0.8559851725 ATXN1L no 808.054 903.604 0.302266 0.894257 -1.11825 -1.033651726 ATXN2 no 795.533 632.445 0.000164673 1.25787 1.25787 3.823831727 ATXN2L no 3321.17 2933.99 0.0586215 1.13196 1.13196 1.899411728 ATXN3 no 411.516 470.159 0.16684 0.875269 -1.14251 -1.386321729 ATXN3L no 0.0749005 0.0224559 0.119976 3.33545 3.33545 1.560021730 ATXN7 no 540.312 605.792 0.245634 0.891911 -1.12119 -1.163661731 ATXN7L1 no 288.336 227.935 0.0124646 1.26499 1.26499 2.51631732 ATXN7L2 no 188.892 180.453 0.622717 1.04677 1.04677 0.4925941733 ATXN7L3 no 1761.93 1539.85 0.0213135 1.14422 1.14422 2.316471734 ATXN7L3B no 2457.78 1737.03 3.91E-06 1.41493 1.41493 4.717671735 ATXN8OS no 0.0502667 0.0608116 0.813256 0.826596 -1.20978 -0.2364691736 AUH no 534.73 567.883 0.44008 0.941619 -1.062 -0.773251737 AUNIP no 300.838 357.309 0.0588366 0.841955 -1.18771 -1.897781738 AUP1 no 3686.91 3324.72 0.137287 1.10894 1.10894 1.490591739 AURKA no 866.994 848.551 0.832718 1.02173 1.02173 0.2114311740 AURKAIP1 no 2126.26 2062.92 0.806706 1.0307 1.0307 0.2449281741 AURKAPS1 no 0.0248626 0.0496412 0.465009 0.500845 -1.99662 -0.7317071742 AURKAPS2 no 0 0.0196749 0.371854 ? ? -0.8945581743 AURKB no 944.598 1030.07 0.428972 0.917027 -1.09048 -0.7921951745 AURKC no 17.0865 23.8968 0.241902 0.715011 -1.39858 -1.172931746 AUTS2 no 1249.3 858.257 0.00496249 1.45562 1.45562 2.833771747 AVEN no 323.905 342.862 0.604807 0.944707 -1.05853 -0.5181341748 AVIL no 164.25 112.621 0.0125991 1.45843 1.45843 2.512421749 AVL9 no 1391.52 892.332 1.33E-09 1.55942 1.55942 6.29241751 AVPI1 no 554.122 1157.98 0.0153196 0.478523 -2.08976 -2.440941752 AVPR1A no 78.8238 38.0573 0.000856239 2.07119 2.07119 3.373351753 AVPR1B no 4.03731 6.71565 0.592451 0.601179 -1.6634 -0.5359531754 AVPR2 no 12.9915 26.0732 0.52544 0.498271 -2.00694 -0.6358431755 AWAT1 no 0.456811 0.328038 0.267747 1.39255 1.39255 1.110651756 AWAT2 no 1.67943 0.635129 0.0619183 2.64423 2.64423 1.8751757 AXDND1 no 24.1056 10.875 0.165612 2.2166 2.2166 1.390361758 AXIN1 no 1379.84 1220.76 0.102925 1.13031 1.13031 1.636621760 AXL no 496.079 885.944 0.119823 0.559944 -1.78589 -1.560671761 AZGP1 no 193.224 286.341 0.700998 0.674804 -1.48191 -0.3843991762 AZGP1P1 no 0.39277 0.449138 0.939447 0.874498 -1.14351 -0.07603811764 AZI1 no 818.544 701.429 0.111649 1.16697 1.16697 1.596261765 AZI2 no 681.247 651.031 0.580066 1.04641 1.04641 0.5539871766 AZIN1 no 4005.16 2581.71 3.64E-07 1.55136 1.55136 5.221991768 B2M no 25255.2 68412.7 2.60E-06 0.369159 -2.70886 -4.807091769 B3GALNT1 no 465.327 340.808 0.0293638 1.36536 1.36536 2.190721770 B3GALNT2 no 652.776 505.644 0.0149263 1.29098 1.29098 2.450541771 B3GALT1 no 20.3334 9.10755 0.0443198 2.23259 2.23259 2.020891772 B3GALT2 no 0.728551 0.31262 0.337192 2.33047 2.33047 0.961511773 B3GALT4 no 201.474 335.08 0.000189947 0.60127 -1.66315 -3.78651774 B3GALT5 no 84.2924 113.56 0.673831 0.742271 -1.34722 -0.4213741775 B3GALT6 no 737.066 671.865 0.373501 1.09704 1.09704 0.8914761776 B3GALTL no 274.287 419.447 0.00110805 0.653924 -1.52923 -3.298571777 B3GAT1 no 86.5512 41.4732 0.0556259 2.08692 2.08692 1.922611778 B3GAT2 no 16.5196 7.46248 0.000301914 2.21368 2.21368 3.663291779 B3GAT3 no 1281.84 864.893 3.97E-06 1.48208 1.48208 4.71411780 B3GAT3P1 no 1.97295 4.63904 0.0480885 0.425293 -2.35132 -1.985991781 B3GNT1 no 427.079 267.095 1.79E-07 1.59898 1.59898 5.365451782 B3GNT2 no 755.332 653.847 0.249278 1.15521 1.15521 1.15471783 B3GNT3 no 633.191 999.269 0.205526 0.633654 -1.57815 -1.269151784 B3GNT4 no 84.3923 59.497 0.0416493 1.41843 1.41843 2.047181785 B3GNT5 no 245.773 738.27 0.0643383 0.332904 -3.00387 -1.857761786 B3GNT6 no 4.31898 11.2435 0.496922 0.384131 -2.60328 -0.68031787 B3GNT7 no 354.911 369.953 0.89289 0.959341 -1.04238 -0.134781788 B3GNT8 no 158.889 233.871 0.188149 0.679388 -1.47191 -1.319561789 B3GNT9 no 324.967 423.294 0.0530817 0.767711 -1.30257 -1.943151790 B3GNTL1 no 336.536 312.237 0.532103 1.07782 1.07782 0.6256381791 B4GALNT1 no 80.902 109.587 0.418629 0.738245 -1.35456 -0.8100951792 B4GALNT2 no 20.1637 48.5326 0.579342 0.415466 -2.40693 -0.5550461793 B4GALNT3 no 1483.17 663.459 4.78E-06 2.23551 2.23551 4.672721794 B4GALNT4 no 860.619 224.679 2.31E-11 3.83044 3.83044 6.991141795 B4GALT1 no 5929.32 6957.72 0.411456 0.852193 -1.17344 -0.8226641796 B4GALT2 no 1660.89 1481.92 0.134566 1.12077 1.12077 1.501031797 B4GALT3 no 918.735 756.909 0.0309971 1.2138 1.2138 2.168941798 B4GALT4 no 1200.96 1207.62 0.979254 0.994481 -1.00555 -0.02602891799 B4GALT5 no 2723.23 2303.16 0.281652 1.18239 1.18239 1.078861800 B4GALT6 no 131.226 116.071 0.482372 1.13057 1.13057 0.7035071801 B4GALT7 no 784.81 554.914 1.08E-06 1.41429 1.41429 4.99491802 B9D1 no 368.269 211.37 7.73E-05 1.74229 1.74229 4.017031803 B9D2 no 84.2168 105.289 0.257784 0.799861 -1.25022 -1.134141804 bA255A11.4 no 0 0 ? 1 1 01805 bA395L14.12 no 1.38535 1.00627 0.11954 1.37672 1.37672 1.561871806 BAALC no 414.7 170.788 0.0253029 2.42816 2.42816 2.249781807 BAAT no 13.7198 5.18588 0.0256736 2.6456 2.6456 2.244061808 BAATP1 no 0.185231 0.0690233 0.0264023 2.6836 2.6836 2.233021809 BABAM1 no 1961.16 1801.02 0.21931 1.08891 1.08891 1.231351810 BACE1 no 897.227 728.916 0.0391005 1.23091 1.23091 2.073651811 BACE1-AS no 0.0373441 0.0653452 0.453422 0.571489 -1.74981 -0.7508551812 BACE2 no 1987.44 2633.67 0.346406 0.754625 -1.32516 -0.94331813 BACH1 no 749.07 1132.09 0.00221122 0.661668 -1.51133 -3.091191814 BACH2 no 97.0977 99.886 0.909217 0.972086 -1.02872 -0.1141381815 BAD no 999.312 966.163 0.777573 1.03431 1.03431 0.2827781816 BAG1 no 1838.13 2844.8 0.0237405 0.646138 -1.54766 -2.274721817 BAG2 no 517.466 216.784 5.36E-08 2.38701 2.38701 5.603521818 BAG3 no 1543.18 2300.5 0.00823001 0.670802 -1.49075 -2.662991819 BAG4 no 701.35 539.042 0.00984467 1.3011 1.3011 2.600461820 BAG5 no 1219.79 1187.97 0.743005 1.02678 1.02678 0.3282271821 BAG6 no 5985.02 4773.39 0.00120894 1.25383 1.25383 3.272981822 BAGE2 no 22.3327 5.69284 0.109538 3.92295 3.92295 1.605791823 BAHCC1 no 542.812 388.512 0.0635002 1.39715 1.39715 1.863671824 BAHD1 no 856.592 701.477 0.0313054 1.22113 1.22113 2.164951825 BAI1 no 105.658 57.1138 0.435559 1.84995 1.84995 0.7809271826 BAI2 no 250.962 200.398 0.398858 1.25232 1.25232 0.8450581828 BAIAP2 no 1245.49 2239.73 0.000511884 0.55609 -1.79827 -3.51881829 BAIAP2-AS1 no 518.327 261.194 1.04E-08 1.98446 1.98446 5.915611830 BAIAP2L1 no 2307.57 2271.86 0.858559 1.01572 1.01572 0.1783861831 BAIAP2L2 no 371.342 542.296 0.299133 0.684758 -1.46037 -1.040381832 BAIAP3 no 157.889 134.088 0.65699 1.1775 1.1775 0.4445861833 BAK1 no 1021.96 1357.22 0.0226939 0.752984 -1.32805 -2.292251834 BAK1P1 no 54.5919 81.2284 0.0553808 0.672079 -1.48792 -1.924551835 BAK1P2 no 0.476534 0.151701 0.0106671 3.14126 3.14126 2.572081838 BANF1 no 3821.44 3385.06 0.210986 1.12892 1.12892 1.253961839 BANF1P1 no 0 0 ? 1 1 01840 BANF1P2 no 0.0155072 0.0599568 0.266926 0.258639 -3.86639 -1.112571841 BANF1P3 no 0.288986 0.213255 0.327404 1.35512 1.35512 0.9812121842 BANF1P4 no 0 0.00121087 0.768013 ? ? -0.2952841843 BANF1P5 no 0.0950151 0.0698378 0.566608 1.36051 1.36051 0.5737891844 BANF2 no 1.79661 0.638207 6.04E-06 2.81509 2.81509 4.62051845 BANK1 no 86.5424 64.0223 0.221997 1.35176 1.35176 1.224181846 BANP no 470.225 396.839 0.00575412 1.18493 1.18493 2.784641847 BAP1 no 1984.99 1658.89 0.00862652 1.19658 1.19658 2.646681848 BARD1 no 308.999 529.455 0.000399082 0.583617 -1.71345 -3.58751849 BARHL1 no 0.878492 0.485934 0.022625 1.80784 1.80784 2.293421850 BARHL2 no 0.308699 0.408522 0.809134 0.755648 -1.32337 -0.241791851 BARX1 no 306.309 114.675 0.133063 2.67111 2.67111 1.506871852 BARX2 no 379.963 893.77 0.0505894 0.425124 -2.35226 -1.964091853 BASP1 no 2066.23 1342.93 0.217554 1.5386 1.5386 1.236071854 BASP1P1 no 5.38773 6.12183 0.905159 0.880084 -1.13626 -0.1192631855 BATF no 75.6204 190.813 0.00665648 0.396306 -2.5233 -2.735631856 BATF2 no 142.193 320.118 0.0170231 0.44419 -2.25129 -2.401721857 BATF3 no 38.5941 61.1666 0.0440842 0.630967 -1.58487 -2.023161858 BAX no 1709.32 1593.14 0.322734 1.07293 1.07293 0.9907461859 BAZ1A no 1082.3 1735.62 0.00546492 0.623579 -1.60365 -2.801841860 BAZ1B no 2891.74 2391.96 0.00377031 1.20894 1.20894 2.923251861 BAZ2A no 1643.75 1569.22 0.700643 1.0475 1.0475 0.384881862 BAZ2B no 751.454 705.501 0.533117 1.06513 1.06513 0.6240911863 BBC3 no 268.792 329.733 0.250198 0.815183 -1.22672 -1.152451864 BBIP1 no 262.97 236.004 0.207369 1.11426 1.11426 1.263991865 BBOX1 no 60.8319 204.716 0.0227229 0.297153 -3.36527 -2.291751866 BBS1 no 327.759 254.588 0.0373598 1.28741 1.28741 2.092581867 BBS10 no 260.121 233.836 0.227719 1.11241 1.11241 1.209121868 BBS12 no 75.4003 67.6021 0.415064 1.11535 1.11535 0.8163261869 BBS2 no 1104.86 763.963 4.45E-06 1.44622 1.44622 4.688711870 BBS4 no 278.202 305.581 0.357175 0.910404 -1.09841 -0.9224071871 BBS5 no 187.251 102.922 1.17E-08 1.81935 1.81935 5.893951872 BBS7 no 306.902 286.681 0.382892 1.07054 1.07054 0.8740671873 BBS9 no 223.775 239.766 0.430049 0.933304 -1.07146 -0.7903451874 BBX no 900.724 1194.09 0.0219441 0.754319 -1.3257 -2.305241875 BCAM no 7973.69 4428.74 0.0212896 1.80044 1.80044 2.31691876 BCAN no 260.587 365.175 0.129421 0.713594 -1.40136 -1.521231877 BCAP29 no 933.549 1100.23 0.0555715 0.8485 -1.17855 -1.923041878 BCAP31 no 7908.35 7095.46 0.283863 1.11456 1.11456 1.073911879 BCAR1 no 1729.65 1940.54 0.381182 0.891325 -1.12193 -0.8772161880 BCAR3 no 631.62 940.571 0.0767319 0.671528 -1.48914 -1.777061881 BCAR4 no 7.23511 24.9996 0.719665 0.289409 -3.45532 -0.3592991882 BCAS1 no 209.838 341.416 0.357375 0.614611 -1.62704 -0.9220221883 BCAS2 no 953.315 971.419 0.791892 0.981363 -1.01899 -0.2641281884 BCAS2P1 no 2.05062 1.77746 0.668121 1.15368 1.15368 0.4292191885 BCAS2P2 no 0.0403698 0.0172246 0.242614 2.34373 2.34373 1.171151886 BCAS2P3 no 0 0.00517298 0.566176 ? ? -0.5744291887 BCAS3 no 611.296 675.567 0.312485 0.904862 -1.10514 -1.011991888 BCAS4 no 230.078 247.728 0.72327 0.928751 -1.07671 -0.3544761890 BCAT2 no 838.408 1016.17 0.114294 0.825067 -1.21202 -1.584531891 BCCIP no 1532.12 1313.08 0.0103724 1.16681 1.16681 2.582021892 BCDIN3D no 179.299 150.711 0.0489207 1.18969 1.18969 1.97861893 BCDIN3D-AS1 no 11.1017 6.84307 0.00675173 1.62233 1.62233 2.730811894 BCHE no 63.9813 17.7258 0.000549072 3.60951 3.60951 3.499261895 BCKDHA no 890.214 1248.34 0.00523453 0.713119 -1.40229 -2.816131896 BCKDHB no 426.287 319.309 0.219555 1.33503 1.33503 1.230691897 BCKDK no 1124.18 983.155 0.0697481 1.14345 1.14345 1.821081898 BCL10 no 559.685 763.636 0.0132087 0.732921 -1.3644 -2.495291899 BCL11A no 1977.85 685.918 0.0033049 2.88351 2.88351 2.96541900 BCL11B no 162.306 300.735 0.0306682 0.539697 -1.85289 -2.173251901 BCL2 no 342.179 398.687 0.839526 0.858264 -1.16514 -0.2027041902 BCL2A1 no 90.3193 125.927 0.298081 0.717237 -1.39424 -1.042651903 BCL2L1 no 2763.1 3037.15 0.681143 0.909767 -1.09918 -0.4113681905 BCL2L11 no 813.514 1075.32 0.0136789 0.756529 -1.32183 -2.482541906 BCL2L12 no 676.689 787.717 0.0940994 0.859051 -1.16408 -1.680331907 BCL2L13 no 1020.39 1071.32 0.509 0.952457 -1.04992 -0.6613121908 BCL2L14 no 65.6093 65.8946 0.988018 0.99567 -1.00435 -0.01503261909 BCL2L15 no 168.287 258.732 0.509195 0.65043 -1.53745 -0.6610071910 BCL2L2 no 835.279 1130.78 0.0431299 0.738675 -1.35377 -2.032431911 BCL2L2-PABPN1 no 134.78 168.868 0.228147 0.798137 -1.25292 -1.2081912 BCL3 no 1210.64 2744.1 0.294077 0.441179 -2.26665 -1.051351913 BCL6 no 902.421 1543.42 0.00157823 0.58469 -1.71031 -3.193731914 BCL6B no 151.72 120.424 0.193578 1.25989 1.25989 1.303451915 BCL7A no 662.068 465.838 0.000494757 1.42124 1.42124 3.528261916 BCL7B no 1082.64 1051.73 0.661721 1.02939 1.02939 0.4380431917 BCL7C no 1271.04 1455.13 0.501702 0.873491 -1.14483 -0.6727561918 BCL9 no 1219.69 567.76 2.87E-14 2.14826 2.14826 8.05761919 BCL9L no 1544.66 1840.4 0.221646 0.839307 -1.19146 -1.225111920 BCLAF1 no 2860.98 2302.09 0.000820209 1.24278 1.24278 3.385691921 BCMO1 no 63.9144 62.9927 0.974063 1.01463 1.01463 0.03254491922 BCO2 no 37.1392 52.0006 0.20966 0.714207 -1.40015 -1.257631923 BCOR no 1176.8 741.011 7.61E-07 1.5881 1.5881 5.069221924 BCORL1 no 593.964 336.9 4.49E-08 1.76303 1.76303 5.638121925 BCORP1 no 0 0.0372026 0.21642 ? ? -1.239131926 BCR no 2868.81 2849.68 0.945931 1.00671 1.00671 0.06788341927 BCRP1 no 5.82747 5.79076 0.976228 1.00634 1.00634 0.02982661928 BCRP2 no 10.3271 8.13917 0.452893 1.26881 1.26881 0.7517351929 BCRP3 no 41.4847 25.3281 0.0143175 1.63789 1.63789 2.465851931 BCRP5 no 24.2308 28.8705 0.428706 0.839295 -1.19148 -0.7926521932 BCRP6 no 10.6307 12.1134 0.454759 0.877598 -1.13947 -0.748631933 BCRP7 no 25.6548 28.5663 0.749744 0.898081 -1.11349 -0.3193161934 BCRP8 no 0.552085 1.01836 0.145098 0.542133 -1.84457 -1.461471935 BCS1L no 558.35 482.842 0.0873883 1.15638 1.15638 1.715831936 BCYRN1 no 98.9982 42.052 0.0691203 2.35419 2.35419 1.825211937 BCYRN1P1 no 70.2083 35.3192 0.185053 1.98782 1.98782 1.328891939 BDH1 no 1504.81 1094.33 0.0332627 1.3751 1.3751 2.140331940 BDH2 no 702.485 586.599 0.175005 1.19756 1.19756 1.360031941 BDH2P1 no 0.553147 0.397675 0.674965 1.39095 1.39095 0.419821942 BDKRB1 no 45.8818 159.192 0.00322389 0.288217 -3.46961 -2.973291943 BDKRB2 no 394.801 851.772 0.000337088 0.463505 -2.15747 -3.633511944 BDNF no 123.77 80.1166 0.266377 1.54487 1.54487 1.113851945 BDNF-AS no 32.1655 32.1767 0.998144 0.999654 -1.00035 -0.002328421946 BDP1 no 661.801 717.809 0.459191 0.921974 -1.08463 -0.7412871947 BDP1P no 0.0203008 0.0525145 0.458711 0.386576 -2.58682 -0.742081948 BEAN1 no 41.6397 37.268 0.732488 1.1173 1.1173 0.3421861949 BECN1 no 1645.71 1571.99 0.47828 1.0469 1.0469 0.7101021950 BECN1P1 no 0.805543 1.11124 0.623443 0.724907 -1.37949 -0.4915651951 BEGAIN no 110.257 74.7943 0.315018 1.47414 1.47414 1.00671952 BEND2 no 1.59413 0.804169 0.013471 1.98233 1.98233 2.488131953 BEND3 no 262.027 179.924 0.000193491 1.45632 1.45632 3.781651954 BEND3P1 no 9.10894 7.68172 0.635291 1.18579 1.18579 0.4748541955 BEND3P2 no 0.506916 0.138133 0.00285594 3.66977 3.66977 3.011581956 BEND3P3 no 38.035 42.1214 0.643424 0.902987 -1.10744 -0.463461957 BEND4 no 5.09274 3.55291 0.398118 1.4334 1.4334 0.8463881958 BEND5 no 92.83 213.078 0.837757 0.435661 -2.29536 -0.204971959 BEND6 no 54.3435 53.2113 0.921354 1.02128 1.02128 0.09882441960 BEND7 no 273.674 170.021 0.0339617 1.60965 1.60965 2.131831961 BEST1 no 59.4512 45.0686 0.0562515 1.31913 1.31913 1.917681962 BEST2 no 12.0938 14.5836 0.838442 0.829276 -1.20587 -0.2040921964 BEST4 no 33.1737 22.7783 0.0393566 1.45638 1.45638 2.070921965 BET1 no 529.228 430.377 0.0089048 1.22969 1.22969 2.635631966 BET1L no 1306.91 1052.84 0.00422482 1.24132 1.24132 2.886441968 BEX2 no 254.861 174.41 0.163441 1.46127 1.46127 1.397561969 BEX4 no 556.985 375.485 0.134033 1.48337 1.48337 1.503091971 BFAR no 1150.99 1093.18 0.550941 1.05288 1.05288 0.5971321972 BFSP1 no 107.916 70.1623 0.0199217 1.5381 1.5381 2.342331973 BFSP2 no 10.6719 10.2064 0.884634 1.04561 1.04561 0.1452411974 BGLAP no 7.5957 6.19388 0.798935 1.22632 1.22632 0.2549871975 BGN no 6507.45 5534.57 0.563044 1.17578 1.17578 0.5790721976 BHLHA15 no 157.067 120.84 0.591968 1.29979 1.29979 0.5366541978 BHLHB9 no 149.174 116.578 0.0448506 1.27961 1.27961 2.015831979 BHLHE22 no 19.5266 23.5089 0.525531 0.830605 -1.20394 -0.6357041980 BHLHE23 no 0.0187252 0.0223618 0.90313 0.837374 -1.19421 -0.1218281981 BHLHE40 no 3852.31 8635.39 0.000126902 0.446108 -2.24161 -3.891221982 BHLHE40-AS1 no 66.6732 80.7542 0.155154 0.825631 -1.2112 -1.425721983 BHLHE41 no 437.149 358.425 0.480349 1.21964 1.21964 0.7067641984 BHMT no 11.5904 10.832 0.963745 1.07002 1.07002 0.0454991986 BICC1 no 184.87 143.894 0.335735 1.28476 1.28476 0.964421987 BICD1 no 791.757 431.694 1.05E-06 1.83407 1.83407 5.000811988 BICD2 no 2029.21 2449.55 0.3667 0.828402 -1.20714 -0.9042561989 BID no 853.306 758.692 0.259226 1.12471 1.12471 1.13071990 BIK no 264.541 367.174 0.238004 0.720479 -1.38797 -1.182721991 BIN1 no 972.295 756.923 0.113752 1.28454 1.28454 1.586911992 BIN2 no 153.212 193.086 0.390326 0.793489 -1.26026 -0.8604691993 BIN2P1 no 0.0614855 0.0251596 0.182653 2.44382 2.44382 1.336211994 BIN2P2 no 0 0.00453916 0.617153 ? ? -0.5004921995 BIN3 no 525.952 617.327 0.0390188 0.851984 -1.17373 -2.074521996 BIN3-IT1 no 0 0.00137302 0.768013 ? ? -0.2952841997 BIRC2 no 1277.69 2112.79 0.551797 0.604739 -1.65361 -0.5958491998 BIRC3 no 419.428 2233.29 0.177039 0.187807 -5.32461 -1.353621999 BIRC5 no 1874.45 1634.95 0.175974 1.14649 1.14649 1.356962000 BIRC6 no 1772.38 1652.29 0.394919 1.07268 1.07268 0.8521492001 BIRC7 no 24.5718 69.9006 0.722095 0.351524 -2.84475 -0.3560472002 BIRC8 no 0.0157547 0.00456004 0.267379 3.45494 3.45494 1.111512003 BIVM no 334.123 329.236 0.909294 1.01485 1.01485 0.114042004 BIVM-ERCC5 no 441.783 399.999 0.531969 1.10446 1.10446 0.6258432005 BLACAT1 no 272.017 241.225 0.567719 1.12765 1.12765 0.5721462006 BLACE no 1.28032 1.35093 0.836177 0.947729 -1.05515 -0.2069952007 BLCAP no 1815.76 2978.8 3.45E-05 0.60956 -1.64053 -4.215072008 BLID no 0 0.0141888 0.407906 ? ? -0.8289312009 BLK no 19.4284 20.4302 0.908263 0.950964 -1.05156 -0.1153422010 BLM no 392.196 376.405 0.683301 1.04195 1.04195 0.4084232011 BLMH no 1146.45 795.279 0.00035505 1.44157 1.44157 3.619412012 BLNK no 233.164 480.543 0.00205684 0.485209 -2.06097 -3.113422013 BLOC1S1 no 1169.54 1386.09 0.26293 0.843773 -1.18515 -1.121932014 BLOC1S1-RDH5 no 7.84578 8.66984 0.655518 0.904952 -1.10503 -0.4466272015 BLOC1S2 no 851.275 1090.39 0.0222014 0.780709 -1.28089 -2.300742016 BLOC1S3 no 361.494 396.747 0.298088 0.911143 -1.09752 -1.042642017 BLOC1S4 no 337.802 265.006 0.000721425 1.2747 1.2747 3.422332018 BLOC1S5 no 313.928 265.002 0.0545308 1.18463 1.18463 1.931352019 BLOC1S5-TXNDC5 no 107.563 79.7092 0.00808039 1.34944 1.34944 2.669332020 BLOC1S6 no 841.898 965.752 0.0678681 0.871755 -1.14711 -1.833552021 BLVRA no 697.155 1039.74 0.00134435 0.67051 -1.4914 -3.24162022 BLVRB no 952.09 2072.91 9.87E-05 0.459301 -2.17722 -3.955472023 BLZF1 no 447.915 689.135 5.58E-05 0.649967 -1.53854 -4.098122024 BLZF2P no 0.47007 0.408337 0.683225 1.15118 1.15118 0.4085262025 BMF no 1111.7 602.458 0.0108937 1.84528 1.84528 2.56462026 BMI1 no 1107.15 714.443 0.000131934 1.54967 1.54967 3.881222027 BMP1 no 1124.36 1593.9 0.0577929 0.705412 -1.41761 -1.905732028 BMP10 no 0.0467265 0.107621 0.54436 0.434177 -2.30321 -0.6070352029 BMP15 no 0.0546274 0.0430716 0.780963 1.26829 1.26829 0.2783542030 BMP2 no 648.495 524.273 0.450924 1.23694 1.23694 0.7550192031 BMP2K no 556.246 535.026 0.85398 1.03966 1.03966 0.1842262033 BMP3 no 53.3673 157.693 0.177327 0.338426 -2.95486 -1.352712036 BMP6 no 192.693 54.6917 3.48E-06 3.52325 3.52325 4.743182037 BMP6P1 no 0 0.0061545 0.768013 ? ? -0.2952842038 BMP7 no 1614.51 1260.62 0.307619 1.28073 1.28073 1.022252039 BMP8A no 164.849 135.465 0.397673 1.21692 1.21692 0.8471862040 BMP8B no 89.6843 109.332 0.242618 0.820291 -1.21908 -1.171142041 BMPER no 15.3866 39.7714 0.552919 0.386876 -2.58481 -0.5941672042 BMPR1A no 750.202 488.137 4.78E-07 1.53687 1.53687 5.165692043 BMPR1APS1 no 0.0530634 0.0512543 0.954946 1.0353 1.0353 0.05655162044 BMPR1APS2 no 0.526963 0.238471 0.00250481 2.20976 2.20976 3.052612045 BMPR1B no 463.967 189.276 0.0120329 2.45127 2.45127 2.529012046 BMPR2 no 1559.78 1379.61 0.176783 1.1306 1.1306 1.354422047 BMS1 no 1494.6 1419.62 0.348385 1.05282 1.05282 0.9394312048 BMS1P1 no 20.0514 25.0439 0.405208 0.800653 -1.24898 -0.8337182049 BMS1P2 no 4.35724 5.74175 0.146012 0.758871 -1.31775 -1.458152050 BMS1P3 no 0 0 ? 1 1 02051 BMS1P4 no 51.4285 59.2725 0.237571 0.867662 -1.15252 -1.183822052 BMS1P5 no 39.9902 50.3602 0.315025 0.794085 -1.25931 -1.006692053 BMS1P6 no 5.37228 7.00167 0.226372 0.767285 -1.3033 -1.212642054 BMS1P7 no 5.13446 6.83656 0.17896 0.75103 -1.33151 -1.347622055 BMX no 38.4785 10.2869 3.57E-11 3.74055 3.74055 6.918232057 BNC2 no 141.134 89.0665 0.0636491 1.58459 1.58459 1.862612058 BNIP1 no 267.414 218.809 0.00231125 1.22213 1.22213 3.077542059 BNIP2 no 1027.13 1251.92 0.0168049 0.820446 -1.21885 -2.406552060 BNIP3 no 1672.92 934.134 0.00140898 1.79088 1.79088 3.227652061 BNIP3L no 1819.19 1907.38 0.687935 0.953762 -1.04848 -0.4021092062 BNIP3P1 no 3.38644 1.51144 0.000480111 2.24053 2.24053 3.536592064 BNIP3P3 no 0 0.0151258 0.422556 ? ? -0.8032692066 BNIP3P5 no 0.0449062 0.0165723 0.146574 2.70972 2.70972 1.456112067 BNIP3P6 no 0.0497198 0 5.16E-06 ? ? 4.655992068 BNIPL no 239.165 493.795 0.0711814 0.48434 -2.06466 -1.811762069 BOC no 688.293 357.358 0.115991 1.92606 1.92606 1.577112070 BOD1 no 1166.75 799.59 4.17E-08 1.45918 1.45918 5.652362071 BOD1L1 no 896.832 839.826 0.535514 1.06788 1.06788 0.6204392072 BOD1L2 no 0.0751607 0.0443472 0.561288 1.69482 1.69482 0.581682073 BOK no 1005.22 886.11 0.313691 1.13442 1.13442 1.009472074 BOK-AS1 no 49.7447 12.0736 0.0126004 4.12011 4.12011 2.512382075 BOLA1 no 331.721 390.42 0.171694 0.84965 -1.17696 -1.370582076 BOLA2 no 1955.11 2035.61 0.763237 0.960455 -1.04117 -0.301552077 BOLA2B no 10.0653 11.172 0.499609 0.900942 -1.10995 -0.6760552078 BOLA3 no 389.618 389.455 0.996687 1.00042 1.00042 0.004156092079 BOLA3-AS1 no 70.6112 31.3223 1.46E-05 2.25435 2.25435 4.418852080 BOLL no 12.9575 25.0146 0.0315549 0.517997 -1.93051 -2.161732081 BOP1 no 2324.7 1585.49 0.00055295 1.46623 1.46623 3.497292082 BORA no 363.522 431.519 0.126912 0.842423 -1.18705 -1.531322083 BPESC1 no 0.281907 0.163119 0.196753 1.72823 1.72823 1.294192084 BPGM no 466.352 737.253 0.0509106 0.632553 -1.58089 -1.961342085 BPHL no 562.269 363.919 3.76E-07 1.54504 1.54504 5.215012086 BPI no 9.34127 2.65891 0.000437732 3.5132 3.5132 3.562112087 BPIFA1 no 7.69969 35.3617 0.522332 0.217741 -4.59262 -0.6406272088 BPIFA2 no 1.32863 8.12203 0.668101 0.163583 -6.11309 -0.4292462089 BPIFA3 no 0.059465 0.0399282 0.519382 1.4893 1.4893 0.645182090 BPIFA4P no 0.13114 0.16632 0.751615 0.788481 -1.26826 -0.3168472091 BPIFB1 no 67.0537 3260.04 0.300362 0.0205683 -48.6184 -1.037732092 BPIFB2 no 293.298 34.2292 0.0390225 8.56865 8.56865 2.074482093 BPIFB3 no 0.196838 0.154927 0.765068 1.27053 1.27053 0.2991472094 BPIFB4 no 5.99643 11.8976 0.669115 0.504002 -1.98412 -0.4278512095 BPIFB5P no 0.184706 0.261603 0.612851 0.706056 -1.41632 -0.5066222096 BPIFB6 no 0.837334 0.683002 0.942252 1.22596 1.22596 0.07251032097 BPIFB9P no 2.75178 2.88134 0.812102 0.955035 -1.04708 -0.2379582098 BPIFC no 3.95878 6.72225 0.590614 0.588907 -1.69806 -0.5386162099 BPNT1 no 818.345 719.579 0.143175 1.13726 1.13726 1.468532100 BPTF no 1570.12 1379.39 0.137812 1.13827 1.13827 1.488592101 BPY2 no 0 0 ? 1 1 02102 BPY2B no 0 0.00420954 0.768013 ? ? -0.2952842103 BPY2C no 0 0.00147875 0.768013 ? ? -0.2952842104 BPY2DP no 0 0.00416696 0.768013 ? ? -0.2952842105 BRAF no 758.76 583.438 0.000923189 1.3005 1.3005 3.351652106 BRAFP1 no 0.656752 0.223824 0.000942898 2.93424 2.93424 3.345542107 BRAP no 612.802 555.478 0.0295907 1.1032 1.1032 2.187632108 BRAT1 no 1615.93 1168.37 7.59E-07 1.38307 1.38307 5.069732109 BRCA1 no 612.399 781.657 0.0202842 0.783463 -1.27638 -2.335442110 BRCA1P1 no 0.126335 0.296101 0.0742671 0.426661 -2.34378 -1.792212111 BRCA2 no 242.311 294.928 0.183164 0.821594 -1.21715 -1.334652112 BRCC3 no 736.83 893.945 0.157299 0.824246 -1.21323 -1.418322113 BRD1 no 1074.92 1047.94 0.752312 1.02574 1.02574 0.3159282114 BRD2 no 5569.63 5584.54 0.969775 0.997329 -1.00268 -0.03792672115 BRD3 no 1162.67 1035.25 0.19889 1.12309 1.12309 1.288022116 BRD4 no 1951.02 1898.42 0.740879 1.02771 1.02771 0.3310442117 BRD7 no 2367.85 1574.95 4.19E-07 1.50344 1.50344 5.193022118 BRD7P1 no 0.0187606 0.00371187 0.149359 5.05421 5.05421 1.44612119 BRD7P2 no 2.77653 1.73911 0.000777505 1.59652 1.59652 3.400992120 BRD7P3 no 0 0.00132022 0.768013 ? ? -0.2952842121 BRD7P4 no 0 0 ? 1 1 02122 BRD7P5 no 0.124203 0.0862226 0.440717 1.4405 1.4405 0.7721722123 BRD7P6 no 1.89832 1.28457 0.375801 1.47778 1.47778 0.8871882124 BRD8 no 1519.7 1168.56 0.00227609 1.30049 1.30049 3.082282125 BRD9 no 1086.66 1169.23 0.707282 0.929374 -1.07599 -0.3759232127 BRE no 968.991 901.869 0.329957 1.07443 1.07443 0.9760362128 BRE-AS1 no 10.1133 11.5179 0.794546 0.87805 -1.13889 -0.2606812129 BREA2 no 11.163 9.34487 0.290414 1.19456 1.19456 1.059382130 BRF1 no 2024.91 1771.43 0.127846 1.14309 1.14309 1.527552131 BRF2 no 438.586 298.59 0.000122081 1.46886 1.46886 3.901152132 BRI3 no 2516.06 2194.63 0.0955379 1.14646 1.14646 1.672982133 BRI3BP no 238.21 229.381 0.688955 1.03849 1.03849 0.4007222134 BRI3P1 no 0.0171063 0.0142788 0.872953 1.19802 1.19802 0.1600672135 BRI3P2 no 0 0.00836572 0.536735 ? ? -0.6185822136 BRI3P3 no 0 0 ? 1 1 02137 BRICD5 no 139.545 140.221 0.976933 0.995183 -1.00484 -0.02894212138 BRINP1 no 15.1436 25.1581 0.535255 0.601936 -1.66131 -0.6208342140 BRIP1 no 286.752 555.605 3.05E-06 0.516107 -1.93758 -4.772182141 BRIX1 no 1018.86 1214.08 0.171568 0.839206 -1.1916 -1.370982142 BRK1 no 3461.07 2555.76 1.45E-05 1.35422 1.35422 4.419832143 BRK1P2 no 0 0 ? 1 1 02144 BRMS1 no 1383.87 1481.02 0.49842 0.9344 -1.07021 -0.6779322145 BRMS1L no 212.268 310.842 0.169114 0.682879 -1.46439 -1.37892146 BROX no 1136.61 1311.99 0.119838 0.866326 -1.1543 -1.56062147 BRPF1 no 658.997 585.275 0.132871 1.12596 1.12596 1.507622148 BRPF3 no 907.27 796.38 0.245294 1.13924 1.13924 1.16452149 BRS3 no 1.15736 0.514228 0.00246471 2.25067 2.25067 3.057622151 BRSK2 no 103.191 38.2283 0.00534197 2.69933 2.69933 2.80942152 BRWD1 no 1231.75 1285.42 0.544431 0.958247 -1.04357 -0.6069282153 BRWD1-AS1 no 5.14052 3.53731 0.117591 1.45323 1.45323 1.570192154 BRWD1-IT2 no 15.0826 10.2763 0.0624639 1.4677 1.4677 1.871062155 BRWD3 no 584.264 619.223 0.615071 0.943545 -1.05983 -0.5034572156 BSCL2 no 2068.31 1615.6 0.00298533 1.28021 1.28021 2.997632157 BSDC1 no 1812.68 1670.97 0.16376 1.0848 1.0848 1.39652158 BSG no 17561.6 12394.2 2.54E-05 1.41692 1.41692 4.288992159 BSN no 89.2445 25.8286 5.63E-07 3.45526 3.45526 5.132052161 BSND no 3.10396 3.61379 0.937602 0.858922 -1.16425 -0.07836022162 BSPH1 no 0.415064 0.340569 0.559832 1.21874 1.21874 0.5838452163 BSPRY no 809.038 586.648 0.0582022 1.37909 1.37909 1.90262164 BST1 no 62.2504 36.8375 0.00131185 1.68987 1.68987 3.248862165 BST2 no 2517.4 6337.07 9.53E-05 0.39725 -2.51731 -3.964252167 BTAF1 no 793.452 872.905 0.382906 0.908978 -1.10014 -0.874042168 BTBD1 no 1266.8 1206.14 0.480077 1.05029 1.05029 0.7072012169 BTBD10 no 606.313 816.686 0.00656689 0.742407 -1.34697 -2.740222170 BTBD11 no 246.504 553.244 0.00418647 0.445561 -2.24436 -2.88942171 BTBD16 no 55.8027 31.1969 0.442123 1.78873 1.78873 0.7697962173 BTBD18 no 1.52069 1.49707 0.937093 1.01578 1.01578 0.07900032174 BTBD19 no 80.097 53.7853 0.23225 1.4892 1.4892 1.197392175 BTBD2 no 2374.8 2026.6 0.0476924 1.17182 1.17182 1.989552176 BTBD3 no 840.98 1093.98 0.320922 0.768735 -1.30084 -0.994472177 BTBD6 no 453.907 579.996 0.0507178 0.782604 -1.27779 -1.962992178 BTBD7 no 983.284 932.163 0.598114 1.05484 1.05484 0.5277652179 BTBD7P1 no 5.32187 3.17001 0.0909198 1.67882 1.67882 1.696882180 BTBD7P2 no 0 0.00152323 0.768013 ? ? -0.2952842181 BTBD8 no 14.9256 12.4052 0.203796 1.20317 1.20317 1.274032182 BTBD9 no 118.582 96.0828 0.0501066 1.23416 1.23416 1.968242183 BTC no 30.8979 36.3954 0.503263 0.848949 -1.17793 -0.6703012184 BTD no 267.718 273.261 0.838026 0.979714 -1.02071 -0.2046252185 BTF3 no 9355 8641.67 0.290364 1.08254 1.08254 1.059492186 BTF3L4 no 1672.05 1496.22 0.120215 1.11752 1.11752 1.559012187 BTF3L4P1 no 0.0180043 0.0181842 0.992753 0.990105 -1.00999 -0.009091482188 BTF3L4P2 no 0.484379 0.30147 0.0519175 1.60672 1.60672 1.952822189 BTF3L4P3 no 0.492204 0.423138 0.533932 1.16322 1.16322 0.6228492190 BTF3L4P4 no 0 0 ? 1 1 02191 BTF3P1 no 0 0.00116992 0.768013 ? ? -0.2952842192 BTF3P10 no 0.0563563 0.143178 0.105546 0.393611 -2.54058 -1.624222193 BTF3P11 no 0 0.00376943 0.611649 ? ? -0.5083382194 BTF3P12 no 0 0.00143294 0.768013 ? ? -0.2952842196 BTF3P14 no 0.0577866 0.0254987 0.44242 2.26626 2.26626 0.7692942197 BTF3P15 no 0 0 ? 1 1 02198 BTF3P16 no 0 0.012878 0.437715 ? ? -0.7772592199 BTF3P2 no 0.157674 0.0931334 0.391758 1.69299 1.69299 0.8578692200 BTF3P3 no 0 0.00312568 0.676157 ? ? -0.4181872201 BTF3P4 no 0.138843 0.096383 0.4436 1.44053 1.44053 0.7673042202 BTF3P5 no 0.897535 0.75724 0.402799 1.18527 1.18527 0.8380082203 BTF3P6 no 0 0 ? 1 1 02204 BTF3P7 no 0.632409 3.10277 0.155521 0.203821 -4.90627 -1.424452205 BTF3P8 no 0 0.00274227 0.679096 ? ? -0.4141652206 BTF3P9 no 0.386452 0.205238 0.0196343 1.88295 1.88295 2.347862207 BTG1 no 2373.73 3718.45 0.00142195 0.638366 -1.5665 -3.224922208 BTG1P1 no 0.483003 0.506902 0.899489 0.952854 -1.04948 -0.126432209 BTG2 no 2329.22 2512.78 0.686132 0.926948 -1.07881 -0.4045642210 BTG3 no 1066.66 1421.83 0.0120526 0.7502 -1.33298 -2.528422211 BTG4 no 1.40463 1.48782 0.881946 0.944082 -1.05923 -0.148652212 BTK no 68.7045 95.8687 0.185495 0.716652 -1.39538 -1.327552213 BTLA no 11.4328 18.0101 0.197462 0.6348 -1.5753 -1.292142214 BTN1A1 no 3.91786 2.61919 0.34741 1.49583 1.49583 0.9413362215 BTN2A1 no 681.465 641.639 0.543719 1.06207 1.06207 0.6080012216 BTN2A2 no 278.965 322.088 0.363639 0.866117 -1.15458 -0.9100572217 BTN2A3P no 28.9579 31.9922 0.52367 0.905156 -1.10478 -0.6385662218 BTN3A1 no 565.251 968.028 0.00854897 0.58392 -1.71256 -2.649822219 BTN3A2 no 710.13 1354.51 0.00220283 0.524272 -1.90741 -3.092362220 BTN3A3 no 430.526 779.531 0.00559686 0.552289 -1.81065 -2.793892221 BTNL10 no 2.86396 1.30773 0.0287381 2.19003 2.19003 2.199342223 BTNL3 no 27.6205 3.41063 0.0165774 8.09835 8.09835 2.411632224 BTNL8 no 23.7841 23.8376 0.999285 0.997754 -1.00225 -0.0008964052225 BTNL9 no 163.387 182.521 0.817258 0.895166 -1.11711 -0.2313082226 BTRC no 557.263 510.473 0.299319 1.09166 1.09166 1.039982227 BUB1 no 959.881 816.476 0.0977134 1.17564 1.17564 1.662052228 BUB1B no 780.209 738.02 0.599963 1.05717 1.05717 0.5250992229 BUB3 no 2336.62 2698.7 0.0559795 0.865834 -1.15496 -1.919822230 BUD13 no 534.517 482.266 0.171285 1.10834 1.10834 1.371892231 BUD31 no 2143.81 1677.99 0.00256987 1.27761 1.27761 3.044622232 BVES no 63.9735 93.9503 0.355893 0.68093 -1.46858 -0.9248732233 BVES-AS1 no 5.10759 9.02177 0.477284 0.566141 -1.76634 -0.7117112234 BYSL no 769.411 546.305 4.02E-05 1.40839 1.40839 4.178192235 BZRAP1 no 1093.1 405.625 4.60E-08 2.69484 2.69484 5.633482236 BZRAP1-AS1 no 106.22 107.934 0.939821 0.984115 -1.01614 -0.07556782237 BZW1 no 6134.1 5437.03 0.338881 1.12821 1.12821 0.958152238 BZW1P1 no 0.498985 0.296863 0.069861 1.68086 1.68086 1.820342239 BZW1P2 no 39.918 34.3311 0.445418 1.16274 1.16274 0.7642432240 BZW2 no 1834.53 1574.22 0.102375 1.16536 1.16536 1.639252241 C10orf10 no 126.168 229.827 0.278946 0.548969 -1.8216 -1.084972242 C10orf105 no 0.254776 1.64642 0.756352 0.154745 -6.46225 -0.3106032243 C10orf107 no 29.6305 9.78387 0.00582934 3.02851 3.02851 2.78032244 C10orf11 no 190.192 190.791 0.986835 0.996862 -1.00315 -0.01651682245 C10orf111 no 9.35553 4.8302 9.74E-06 1.93688 1.93688 4.512172246 C10orf112 no 28.8312 10.4314 0.0022177 2.76388 2.76388 3.090292247 C10orf113 no 0.0611334 0.0265356 0.13513 2.30383 2.30383 1.498852248 C10orf115 no 3.2742 3.52233 0.880915 0.929555 -1.07578 -0.1499572249 C10orf118 no 593.783 547.718 0.428363 1.08411 1.08411 0.7932432250 C10orf12 no 81.5627 92.2387 0.338076 0.884257 -1.13089 -0.959752251 C10orf120 no 0.149939 0.0869068 0.232378 1.72528 1.72528 1.197062253 C10orf128 no 24.6768 22.4359 0.65092 1.09988 1.09988 0.4530112254 C10orf129 no 2.46503 2.66021 0.689149 0.926631 -1.07918 -0.4004582255 C10orf131 no 5.78263 5.10225 0.335455 1.13335 1.13335 0.9649792256 C10orf137 no 498.985 434.8 0.106775 1.14762 1.14762 1.618492257 C10orf2 no 477.544 347.885 0.000907946 1.37271 1.37271 3.356462258 C10orf25 no 42.4254 31.9148 0.0450469 1.32933 1.32933 2.013972259 C10orf32 no 392.282 265.912 2.24E-07 1.47524 1.47524 5.32062260 C10orf32-AS3MT no 17.3618 11.8415 0.00434129 1.46617 1.46617 2.877592261 C10orf35 no 241.841 217.736 0.278777 1.11071 1.11071 1.085352263 C10orf53 no 0.508067 0.314636 0.469007 1.61478 1.61478 0.7251632264 C10orf54 no 413.181 1123.99 5.95E-06 0.367602 -2.72033 -4.623832265 C10orf55 no 24.8153 80.3692 0.0100745 0.308766 -3.2387 -2.592322266 C10orf62 no 0.0187252 0.0166859 0.928417 1.12221 1.12221 0.08992362267 C10orf67 no 16.798 19.6479 0.676443 0.854951 -1.16966 -0.4177952268 C10orf68 no 105.955 110.706 0.708312 0.957092 -1.04483 -0.3745362269 C10orf71 no 1.98549 0.789622 0.394922 2.51448 2.51448 0.8521442270 C10orf71-AS1 no 0.132652 0.035301 0.072225 3.75773 3.75773 1.805072271 C10orf76 no 598.594 618.391 0.654159 0.967986 -1.03307 -0.4485112273 C10orf85 no 3.87458 4.78131 0.711344 0.810359 -1.23402 -0.3704582274 C10orf88 no 226.37 199.32 0.0402345 1.13571 1.13571 2.06172275 C10orf90 no 4.79539 4.07374 0.743908 1.17715 1.17715 0.3270312276 C10orf91 no 56.8779 15.2346 1.65E-05 3.73347 3.73347 4.390012277 C10orf95 no 3.64382 3.80674 0.908443 0.957203 -1.04471 -0.1151152278 C10orf99 no 259.942 961.574 0.0439042 0.27033 -3.69919 -2.024892279 C11orf1 no 246.865 160.61 0.000386283 1.53705 1.53705 3.596422280 C11orf16 no 23.0747 17.2099 0.572423 1.34078 1.34078 0.5652062282 C11orf24 no 987.005 777.077 0.0307571 1.27015 1.27015 2.172082283 C11orf30 no 454.457 476.47 0.646887 0.9538 -1.04844 -0.4586272284 C11orf31 no 1774.24 1389.51 0.00529733 1.27688 1.27688 2.812182285 C11orf34 no 1.6439 1.27032 0.165399 1.29408 1.29408 1.391072286 C11orf35 no 218.004 177.6 0.305783 1.2275 1.2275 1.026142287 C11orf40 no 0.267449 0.102649 0.0138766 2.60547 2.60547 2.47732288 C11orf42 no 8.19635 7.32408 0.594174 1.1191 1.1191 0.5334582289 C11orf44 no 0.488697 1.00649 0.222453 0.485545 -2.05954 -1.222972290 C11orf45 no 15.6333 18.7289 0.481802 0.834716 -1.19801 -0.7044232291 C11orf48 no 1284.5 1053.71 0.0284839 1.21902 1.21902 2.202882292 C11orf49 no 1103.6 541.824 1.34E-14 2.03683 2.03683 8.17382293 C11orf52 no 148.252 125.473 0.405563 1.18154 1.18154 0.8330882294 C11orf53 no 12.9014 80.2328 0.093833 0.1608 -6.21892 -1.68172295 C11orf54 no 492.519 363.423 0.013614 1.35522 1.35522 2.484282296 C11orf57 no 635.418 484.402 0.000172314 1.31176 1.31176 3.8122297 C11orf58 no 2848.32 2434.51 0.00936182 1.16998 1.16998 2.618132298 C11orf63 no 149.501 87.8001 0.031665 1.70274 1.70274 2.160322299 C11orf65 no 137.549 100.6 0.0109739 1.36729 1.36729 2.561992300 C11orf68 no 691.158 605.95 0.0624906 1.14062 1.14062 1.870872301 C11orf70 no 49.8984 75.6801 0.413502 0.659334 -1.51668 -0.8190662302 C11orf71 no 237.557 134.152 2.06E-06 1.7708 1.7708 4.857372303 C11orf73 no 599.276 589.572 0.862756 1.01646 1.01646 0.1730392304 C11orf74 no 218.206 153.02 0.00194115 1.426 1.426 3.131112305 C11orf80 no 1255.49 1371.9 0.288228 0.915146 -1.09272 -1.06422306 C11orf82 no 208.817 277.618 0.079901 0.752173 -1.32948 -1.758162307 C11orf83 no 658.858 616.683 0.77049 1.06839 1.06839 0.2920392308 C11orf84 no 1004.52 644.721 3.28E-08 1.55808 1.55808 5.698412310 C11orf86 no 15.1392 11.6527 0.730446 1.2992 1.2992 0.3449052312 C11orf88 no 9.35297 8.55973 0.942603 1.09267 1.09267 0.07206812313 C11orf91 no 6.94878 17.4984 0.0358124 0.39711 -2.51819 -2.110062314 C11orf92 no 87.6719 176.775 0.288705 0.495951 -2.01633 -1.063152315 C11orf93 no 84.1817 159.84 0.213785 0.526663 -1.89875 -1.246292316 C11orf94 no 4.1832 2.52141 0.000529973 1.65907 1.65907 3.509132317 C11orf95 no 695.069 265.236 3.43E-23 2.62057 2.62057 10.95262318 C11orf96 no 317.699 218.061 0.197109 1.45693 1.45693 1.293162319 C12orf10 no 952.036 739.729 0.00454774 1.28701 1.28701 2.862432320 C12orf23 no 1147.9 743.273 0.000774579 1.54438 1.54438 3.402072321 C12orf29 no 315.624 408.311 0.0127342 0.772999 -1.29366 -2.508562322 C12orf36 no 194.832 129.908 0.332236 1.49978 1.49978 0.971442324 C12orf4 no 360.573 424.025 0.0306331 0.850358 -1.17598 -2.173712325 C12orf40 no 2.92366 1.60214 0.218493 1.82485 1.82485 1.233542326 C12orf42 no 21.1291 14.8319 0.312436 1.42457 1.42457 1.01212327 C12orf43 no 261.749 249.113 0.42834 1.05072 1.05072 0.7932812328 C12orf44 no 1286.58 1246.03 0.763515 1.03254 1.03254 0.3011852329 C12orf45 no 307.026 223.096 0.00846457 1.3762 1.3762 2.653262330 C12orf49 no 1036.53 794.233 0.00215654 1.30508 1.30508 3.09892331 C12orf5 no 434.633 395.208 0.295338 1.09976 1.09976 1.04862332 C12orf50 no 1.06625 0.530084 0.00418777 2.01148 2.01148 2.88932333 C12orf52 no 601.154 449.251 2.94E-06 1.33812 1.33812 4.780372335 C12orf55 no 19.8067 37.5712 0.400194 0.527178 -1.89689 -0.8426632336 C12orf56 no 75.301 98.8382 0.376763 0.761861 -1.31258 -0.8853992337 C12orf57 no 1535.81 1523.99 0.967079 1.00776 1.00776 0.04131232338 C12orf60 no 34.8681 31.625 0.369821 1.10255 1.10255 0.8983722339 C12orf61 no 13.0866 10.6611 0.135079 1.22751 1.22751 1.499052340 C12orf65 no 685.697 603.068 0.111168 1.13701 1.13701 1.598422341 C12orf66 no 205.309 181.755 0.173551 1.12959 1.12959 1.364642342 C12orf68 no 41.9943 36.2973 0.635687 1.15695 1.15695 0.4742982343 C12orf71 no 1.19523 1.7976 0.101193 0.664905 -1.50397 -1.644952344 C12orf73 no 250.769 173.348 6.19E-06 1.44663 1.44663 4.615162345 C12orf74 no 4.73925 7.16059 0.491059 0.661851 -1.51091 -0.6896062346 C12orf75 no 1137.56 1139.25 0.995172 0.998521 -1.00148 -0.006056962347 C12orf76 no 565.845 587.472 0.673673 0.963187 -1.03822 -0.4215912348 C12orf77 no 0.366897 0.427907 0.866698 0.857423 -1.16629 -0.1680212349 C12orf79 no 30.3883 57.4714 0.00839676 0.528756 -1.89123 -2.656052350 C12orf80 no 0.944781 2.34016 0.0507681 0.403725 -2.47693 -1.962562351 C13orf35 no 2.92713 1.26892 0.00122842 2.30679 2.30679 3.268272352 C13orf45 no 3.16209 2.7414 0.791843 1.15346 1.15346 0.2641912353 C14orf1 no 1151.33 1174.61 0.825983 0.980174 -1.02023 -0.220082354 C14orf105 no 89.0654 24.8095 0.0400038 3.58997 3.58997 2.06412355 C14orf119 no 1192.19 1295.59 0.432207 0.920189 -1.08673 -0.7866492356 C14orf132 no 295.019 276.349 0.809435 1.06756 1.06756 0.2414022357 C14orf142 no 316.822 280.432 0.245351 1.12976 1.12976 1.164362358 C14orf159 no 969.412 855.874 0.390724 1.13266 1.13266 0.8597472359 C14orf164 no 52.0938 37.902 0.039852 1.37443 1.37443 2.065692360 C14orf166 no 2770.15 2462.27 0.076716 1.12504 1.12504 1.777162361 C14orf166B no 2.43826 0.937484 2.09E-05 2.60085 2.60085 4.334492362 C14orf169 no 4.29001 4.07989 0.776329 1.0515 1.0515 0.2844032363 C14orf177 no 0.0542936 0.0720733 0.715128 0.753311 -1.32747 -0.3653772364 C14orf178 no 10.6897 7.51165 0.0283775 1.42309 1.42309 2.204382365 C14orf180 no 1.01055 2.34479 0.679907 0.430977 -2.32031 -0.4130562366 C14orf182 no 66.4409 81.8019 0.341887 0.812217 -1.2312 -0.9521932367 C14orf183 no 4.19444 2.89966 0.0752752 1.44653 1.44653 1.785962368 C14orf2 no 1552.46 1499.12 0.735027 1.03558 1.03558 0.338812369 C14orf23 no 1.44992 0.998567 0.854438 1.452 1.452 0.1836422370 C14orf28 no 75.8905 82.0166 0.470683 0.925306 -1.08072 -0.7224282371 C14orf37 no 116.01 34.4319 4.42E-15 3.36927 3.36927 8.340642372 C14orf39 no 13.2279 41.17 0.0648966 0.321299 -3.11236 -1.853862373 C14orf64 no 36.6294 67.7745 0.168256 0.540459 -1.85028 -1.381692374 C14orf79 no 351.118 263.43 0.00286879 1.33287 1.33287 3.010172375 C14orf80 no 513.33 655.639 0.0766874 0.782946 -1.27723 -1.777332376 C14orf93 no 285.569 297.8 0.648792 0.958927 -1.04283 -0.4559732377 C14orf99 no 0.3943 0.177223 0.106753 2.22488 2.22488 1.618592378 C15orf26 no 8.69666 4.56014 0.000767132 1.9071 1.9071 3.404832379 C15orf27 no 37.2997 65.283 0.0588221 0.571355 -1.75023 -1.897892381 C15orf37 no 52.8982 37.0527 0.00177756 1.42765 1.42765 3.157872382 C15orf38 no 46.7983 58.6065 0.326857 0.798518 -1.25232 -0.9823242383 C15orf38-AP3S2 no 1914.38 1945.6 0.856966 0.983951 -1.01631 -0.1804182384 C15orf39 no 897.997 1198.29 0.00468381 0.749401 -1.3344 -2.852772385 C15orf40 no 490.943 598.474 0.0588384 0.820323 -1.21903 -1.897772386 C15orf41 no 231.814 358.223 0.00311451 0.647124 -1.5453 -2.984242387 C15orf43 no 0.481435 0.593139 0.533473 0.811673 -1.23202 -0.6235492388 C15orf48 no 409.546 1377.91 0.0101617 0.297223 -3.36448 -2.589282389 C15orf52 no 169.557 380.374 0.00766008 0.445763 -2.24335 -2.687732390 C15orf53 no 0.481776 0.976356 0.0794623 0.493443 -2.02658 -1.760742391 C15orf54 no 0.736591 0.446828 0.0531519 1.64849 1.64849 1.942572392 C15orf56 no 0 0.0572424 0.107524 ? ? -1.615022393 C15orf57 no 391.608 320.888 0.011391 1.22039 1.22039 2.548682395 C15orf60 no 14.5367 15.1104 0.859874 0.962036 -1.03946 -0.1767112396 C15orf61 no 191.344 156.72 0.0224282 1.22093 1.22093 2.296812397 C15orf62 no 0.0613011 0.0442506 0.589707 1.38532 1.38532 0.5399342398 C15orf65 no 21.7892 21.1677 0.891532 1.02936 1.02936 0.13652399 C16orf11 no 0.921398 0.426001 0.00693312 2.1629 2.1629 2.721812400 C16orf13 no 1085.86 899.154 0.13278 1.20765 1.20765 1.507972401 C16orf3 no 0.0398927 0.0142689 0.155376 2.79578 2.79578 1.424952402 C16orf45 no 293.27 138.567 0.000599874 2.11644 2.11644 3.474472403 C16orf46 no 114.499 59.145 1.05E-08 1.93591 1.93591 5.914042404 C16orf47 no 2.17002 1.31169 0.120754 1.65437 1.65437 1.556742405 C16orf52 no 108.295 128.075 0.212383 0.845563 -1.18264 -1.250122406 C16orf54 no 24.9569 77.1021 0.0471434 0.323686 -3.08941 -1.994522407 C16orf58 no 1829.16 1411.26 0.000793525 1.29611 1.29611 3.395162408 C16orf59 no 383.018 348.168 0.352297 1.10009 1.10009 0.931822409 C16orf62 no 1016.16 886.414 0.192131 1.14637 1.14637 1.307712410 C16orf70 no 559.621 565.42 0.895205 0.989744 -1.01036 -0.1318512411 C16orf71 no 37.3745 27.5949 0.136302 1.3544 1.3544 1.494352412 C16orf72 no 2332.06 2062.83 0.110127 1.13051 1.13051 1.603122413 C16orf74 no 210.264 423.406 0.0079685 0.4966 -2.01369 -2.674152414 C16orf78 no 0.70545 0.285327 7.63E-06 2.47243 2.47243 4.56782415 C16orf80 no 725.353 674.721 0.301729 1.07504 1.07504 1.03482416 C16orf82 no 0 0.0503037 0.326114 ? ? -0.9838372417 C16orf86 no 43.9623 31.5604 0.0161035 1.39296 1.39296 2.422442418 C16orf87 no 203.804 131.954 1.06E-05 1.54452 1.54452 4.493242420 C16orf90 no 0.761565 0.563133 0.332091 1.35237 1.35237 0.971732421 C16orf91 no 307.585 289.024 0.457824 1.06422 1.06422 0.7435472422 C16orf92 no 0.0185835 0.0786093 0.233354 0.236404 -4.23005 -1.194562423 C16orf93 no 60.3991 69.8853 0.535206 0.86426 -1.15706 -0.6209082424 C16orf95 no 114.569 71.5653 0.00440088 1.60089 1.60089 2.873152425 C16orf96 no 6.76606 7.43459 0.615877 0.910078 -1.09881 -0.5023092426 C16orf97 no 0.154428 0.132898 0.756823 1.162 1.162 0.3099832427 C16orf98 no 11.3434 21.4862 0.0164373 0.527941 -1.89415 -2.41482428 C17orf100 no 64.1985 66.4517 0.789871 0.966093 -1.0351 -0.2667542429 C17orf102 no 0.221608 0.201104 0.933367 1.10196 1.10196 0.08369032430 C17orf103 no 266.708 199.247 0.0324135 1.33858 1.33858 2.150852431 C17orf104 no 46.5331 88.8346 0.122986 0.523817 -1.90906 -1.547412432 C17orf105 no 0.111823 0.0905349 0.669031 1.23514 1.23514 0.4279662433 C17orf107 no 6.28333 8.04607 0.492619 0.78092 -1.28054 -0.6871242434 C17orf112 no 0.0499895 0.0256535 0.440413 1.94864 1.94864 0.7726862435 C17orf47 no 1.40505 1.27168 0.598551 1.10488 1.10488 0.5271352436 C17orf49 no 1351.15 1309.41 0.769186 1.03187 1.03187 0.2937482437 C17orf50 no 0.017855 0.0771255 0.387904 0.231506 -4.31953 -0.8648822438 C17orf51 no 212.866 180.277 0.35248 1.18077 1.18077 0.9314662439 C17orf53 no 300.317 348.052 0.225136 0.862851 -1.15895 -1.215892440 C17orf58 no 234.838 252.014 0.56973 0.931843 -1.07314 -0.5691772441 C17orf59 no 208.3 226.251 0.419488 0.92066 -1.08618 -0.8085992442 C17orf61-PLSCR3 no 9.70839 8.0524 0.496815 1.20565 1.20565 0.6804692443 C17orf62 no 1652.13 1635.77 0.902158 1.01 1.01 0.1230562445 C17orf66 no 3.63474 5.12361 0.204752 0.709409 -1.40962 -1.271332446 C17orf67 no 215.604 191.317 0.384455 1.12694 1.12694 0.8711942447 C17orf70 no 1114.81 819.542 3.46E-07 1.36028 1.36028 5.232442448 C17orf72 no 45.2907 31.3377 0.328484 1.44525 1.44525 0.979022449 C17orf74 no 0.142619 0.531364 0.645518 0.268402 -3.72575 -0.4605362450 C17orf75 no 518.225 384.191 0.000186018 1.34887 1.34887 3.791982451 C17orf76-AS1 no 6424.04 4190.75 0.000121881 1.53291 1.53291 3.901582452 C17orf77 no 1.5013 29.1911 0.68255 0.0514301 -19.4439 -0.4094482453 C17orf78 no 0.402352 0.959311 0.846346 0.419418 -2.38426 -0.1939772454 C17orf80 no 569.271 462.563 0.012928 1.23069 1.23069 2.503082455 C17orf82 no 12.1945 11.6684 0.882235 1.04509 1.04509 0.1482822456 C17orf85 no 1086.06 918.668 0.025195 1.18221 1.18221 2.251462457 C17orf89 no 515.684 426.038 0.151991 1.21042 1.21042 1.436772458 C17orf96 no 467.748 217.054 1.77E-08 2.15498 2.15498 5.815952459 C17orf97 no 161.65 90.3123 2.09E-05 1.7899 1.7899 4.335322460 C17orf98 no 1.22268 0.582942 0.00136878 2.09742 2.09742 3.236262461 C17orf99 no 7.33543 15.8874 0.255565 0.461713 -2.16585 -1.139462462 C18orf12 no 0 0 ? 1 1 02463 C18orf21 no 327.032 326.148 0.971513 1.00271 1.00271 0.0357452464 C18orf25 no 588.157 764.568 0.00324603 0.769267 -1.29994 -2.971122465 C18orf32 no 869.072 673.137 0.00173301 1.29108 1.29108 3.165552466 C18orf42 no 0.607015 0.231587 0.00309692 2.62111 2.62111 2.986032467 C18orf54 no 175.927 256.586 0.0131791 0.685647 -1.45848 -2.49612468 C18orf56 no 35.2307 42.1251 0.268473 0.836334 -1.19569 -1.108972469 C18orf61 no 20.1461 31.407 0.174257 0.641453 -1.55896 -1.36242470 C18orf63 no 0.499267 0.184235 0.000676205 2.70994 2.70994 3.440692471 C18orf8 no 726.007 797.453 0.26265 0.910408 -1.09841 -1.122592472 C19orf10 no 1942.05 1699.62 0.315201 1.14264 1.14264 1.006322473 C19orf12 no 626.284 382.776 2.66E-07 1.63616 1.63616 5.28552474 C19orf18 no 27.4508 14.3953 3.42E-05 1.90693 1.90693 4.217392475 C19orf24 no 798.38 765.018 0.857311 1.04361 1.04361 0.1799782476 C19orf25 no 448.809 523.672 0.232201 0.857042 -1.1668 -1.197512477 C19orf26 no 79.3057 24.6445 4.74E-11 3.21799 3.21799 6.870712478 C19orf33 no 686.466 3225.05 0.0184208 0.212854 -4.69805 -2.372052479 C19orf35 no 5.06548 7.76667 0.11975 0.652208 -1.53325 -1.560982480 C19orf38 no 32.71 76.1297 0.0105411 0.429661 -2.32742 -2.57632481 C19orf40 no 107.164 145.158 0.00441129 0.73826 -1.35454 -2.872382482 C19orf43 no 2770.8 2843.93 0.827826 0.974285 -1.02639 -0.217712483 C19orf44 no 255.869 215.725 0.139273 1.18609 1.18609 1.483072484 C19orf45 no 37.7062 17.972 0.000162274 2.09805 2.09805 3.827652485 C19orf47 no 334.754 488.654 0.00205005 0.685053 -1.45974 -3.114432486 C19orf48 no 1883.72 2243.58 0.114625 0.839607 -1.19103 -1.583072487 C19orf52 no 331.978 320.063 0.872888 1.03723 1.03723 0.160152488 C19orf53 no 1970.39 1722.19 0.217376 1.14412 1.14412 1.236552489 C19orf54 no 390.937 386.958 0.923498 1.01028 1.01028 0.09612232490 C19orf55 no 167.434 199.442 0.298672 0.839515 -1.19116 -1.041382491 C19orf57 no 189.204 448.486 0.000201252 0.421873 -2.37038 -3.77132492 C19orf59 no 7.1741 10.4634 0.430404 0.685638 -1.45849 -0.7897362493 C19orf60 no 448.863 519.18 0.468097 0.864562 -1.15665 -0.7266492494 C19orf66 no 462.49 807.658 0.000297648 0.572631 -1.74633 -3.667122495 C19orf67 no 3.42712 3.09707 0.779199 1.10657 1.10657 0.2806562496 C19orf68 no 330.337 400.191 0.0295591 0.825448 -1.21146 -2.188062497 C19orf69 no 3.22342 3.7636 0.952076 0.856473 -1.16758 -0.06015822498 C19orf70 no 777.519 833.449 0.688218 0.932893 -1.07193 -0.4017252499 C19orf71 no 2.94211 5.82128 0.0805486 0.505405 -1.97861 -1.754382500 C19orf73 no 27.754 23.6599 0.304437 1.17304 1.17304 1.029012501 C19orf77 no 154.809 84.0527 0.490323 1.84181 1.84181 0.6907782502 C19orf80 no 0.2594 0.24337 0.874279 1.06587 1.06587 0.1583822503 C19orf81 no 11.1092 5.87131 0.184365 1.89212 1.89212 1.330982504 C19orf82 no 112.136 70.0243 2.09E-05 1.60139 1.60139 4.33442505 C1D no 448.519 479.517 0.480982 0.935356 -1.06911 -0.7057442506 C1DP1 no 0.573845 0.607261 0.865348 0.944972 -1.05823 -0.1697392507 C1DP2 no 0.125702 0.1275 0.982852 0.985901 -1.0143 -0.02151422508 C1DP3 no 0.0400873 0.0129363 0.180103 3.09883 3.09883 1.344072509 C1DP4 no 0.171502 0.0947904 0.139439 1.80928 1.80928 1.482442510 C1GALT1 no 599.656 715.53 0.229761 0.838058 -1.19324 -1.203812511 C1GALT1C1 no 620.264 603.519 0.779061 1.02775 1.02775 0.2808362512 C1orf100 no 4.9542 3.83206 0.414386 1.29283 1.29283 0.8175142513 C1orf101 no 36.3198 37.4495 0.84049 0.969834 -1.0311 -0.201472514 C1orf105 no 88.4418 68.0103 0.314896 1.30042 1.30042 1.006962515 C1orf106 no 1110.96 2971.46 3.29E-06 0.373877 -2.67468 -4.755172516 C1orf109 no 407.144 337.103 0.0136185 1.20777 1.20777 2.484162517 C1orf110 no 248.389 100.159 0.0770449 2.47995 2.47995 1.775172518 C1orf111 no 3.04333 4.94221 0.206145 0.615783 -1.62395 -1.267422519 C1orf112 no 418.995 535.295 0.00325093 0.782736 -1.27757 -2.970642520 C1orf115 no 464.784 433.926 0.713335 1.07111 1.07111 0.3677832521 C1orf116 no 610.106 1501.41 0.00131372 0.406357 -2.46089 -3.248432522 C1orf122 no 812.346 702.647 0.178867 1.15612 1.15612 1.34792523 C1orf123 no 701.164 620.16 0.0829628 1.13062 1.13062 1.740482525 C1orf131 no 369.896 380.782 0.723201 0.971413 -1.02943 -0.354572526 C1orf137 no 1.31704 1.72749 0.481836 0.762401 -1.31165 -0.7043692527 C1orf140 no 0.323489 0.151954 0.0599159 2.12886 2.12886 1.889692529 C1orf145 no 25.7959 30.8561 0.433567 0.836008 -1.19616 -0.7843242530 C1orf146 no 2.73062 2.72815 0.996563 1.00091 1.00091 0.004311642531 C1orf148 no 6.7801 8.08115 0.38852 0.839002 -1.19189 -0.8637582532 C1orf158 no 7.18278 2.4665 0.109536 2.91213 2.91213 1.60582533 C1orf159 no 447.742 446.195 0.963843 1.00347 1.00347 0.04537512534 C1orf162 no 102.795 158.672 0.0959736 0.647849 -1.54357 -1.670782535 C1orf167 no 28.8534 85.2843 0.0103043 0.33832 -2.95578 -2.584352536 C1orf168 no 101.701 34.241 0.00148164 2.97014 2.97014 3.212652537 C1orf170 no 38.5871 130.918 0.000236388 0.294742 -3.3928 -3.728752538 C1orf172 no 528.834 622.891 0.0366287 0.849 -1.17786 -2.100762539 C1orf173 no 169.419 37.7175 0.019916 4.49179 4.49179 2.342442540 C1orf174 no 655.43 627.843 0.521354 1.04394 1.04394 0.6421342541 C1orf177 no 29.4708 34.7393 0.49314 0.848343 -1.17877 -0.6862972542 C1orf180 no 1.13721 0.470329 0.0429672 2.4179 2.4179 2.034032543 C1orf185 no 29.7485 66.7489 0.0520602 0.445677 -2.24378 -1.951632544 C1orf186 no 3142.37 1289.29 0.0174754 2.43729 2.43729 2.391892545 C1orf189 no 6.29081 3.72381 0.624631 1.68935 1.68935 0.4898832546 C1orf191 no 0 0.00185863 0.768013 ? ? -0.2952842547 C1orf192 no 183.279 242.839 0.39704 0.754735 -1.32497 -0.8483252548 C1orf194 no 28.2592 29.6541 0.946738 0.952959 -1.04936 -0.06686882550 C1orf198 no 1381.39 1028.36 0.00236073 1.3433 1.3433 3.070992551 C1orf200 no 0 0 ? 1 1 02552 C1orf204 no 67.6647 67.7777 0.992357 0.998332 -1.00167 -0.009588892553 C1orf21 no 797.964 1323.52 0.00198919 0.60291 -1.65862 -3.123652554 C1orf210 no 303.287 312.14 0.882822 0.971638 -1.02919 -0.1475392555 C1orf213 no 55.171 45.877 0.152158 1.20259 1.20259 1.436182556 C1orf216 no 410.053 308.151 0.000268559 1.33069 1.33069 3.694722557 C1orf220 no 42.692 43.9252 0.868469 0.971927 -1.02888 -0.1657682558 C1orf226 no 336.973 360.231 0.727212 0.935437 -1.06902 -0.3492152559 C1orf227 no 2.03045 1.16562 0.000585766 1.74195 1.74195 3.481152560 C1orf228 no 60.1859 54.8699 0.764691 1.09688 1.09688 0.2996412561 C1orf229 no 5.44822 2.16469 3.15E-05 2.51686 2.51686 4.236772562 C1orf233 no 302.966 397.934 0.256183 0.761346 -1.31346 -1.137972563 C1orf234 no 1.7277 2.29687 0.247242 0.752199 -1.32943 -1.159692564 C1orf27 no 703.49 674.575 0.638883 1.04286 1.04286 0.4698142565 C1orf35 no 656.951 657.74 0.989488 0.9988 -1.0012 -0.01318832566 C1orf43 no 4401.99 3852.9 0.0640478 1.14251 1.14251 1.85982567 C1orf50 no 185.497 174.107 0.365606 1.06542 1.06542 0.9063262568 C1orf51 no 174.937 170.787 0.888438 1.0243 1.0243 0.1404192569 C1orf52 no 469.238 459.72 0.755935 1.0207 1.0207 0.3111532570 C1orf53 no 81.085 63.4738 0.0576649 1.27746 1.27746 1.906712571 C1orf54 no 132.086 129.809 0.903448 1.01754 1.01754 0.1214252572 C1orf56 no 254.561 180.76 0.00297377 1.40828 1.40828 2.998852573 C1orf61 no 34.7025 30.0612 0.799721 1.1544 1.1544 0.2539692574 C1orf63 no 1188.13 1245.77 0.646255 0.953731 -1.04851 -0.4595082576 C1orf65 no 3.96752 4.88545 0.71254 0.812108 -1.23136 -0.3688522577 C1orf68 no 0.436756 4.73135 0.0699998 0.0923111 -10.8329 -1.819432578 C1orf74 no 183.187 310.142 0.0773305 0.590654 -1.69304 -1.773442579 C1orf85 no 1048.96 1011.08 0.70952 1.03746 1.03746 0.3729112580 C1orf86 no 1128.86 809.259 0.00805598 1.39493 1.39493 2.670382582 C1orf94 no 4.37871 1.64295 0.400398 2.66514 2.66514 0.8422982584 C1QA no 1653.47 2440.01 0.310565 0.677648 -1.47569 -1.016032585 C1QB no 1870.84 2648.14 0.366289 0.706475 -1.41548 -0.9050332586 C1QBP no 2476.28 1533.69 2.22E-08 1.61459 1.61459 5.773362587 C1QBPP no 0 0.00143247 0.768013 ? ? -0.2952842588 C1QC no 1523.78 2204.5 0.273643 0.691216 -1.44673 -1.097042589 C1QL1 no 181.392 156.681 0.744323 1.15771 1.15771 0.3264822590 C1QL2 no 3.14611 5.2969 0.856153 0.593953 -1.68364 -0.1814542591 C1QL3 no 37.9152 52.2186 0.169902 0.726086 -1.37725 -1.376352592 C1QL4 no 31.8236 17.856 0.259543 1.78223 1.78223 1.129952593 C1QTNF1 no 285.926 539.411 0.467021 0.530071 -1.88654 -0.728412594 C1QTNF1-AS1 no 1.4171 1.9171 0.673181 0.739187 -1.35284 -0.4222662597 C1QTNF3-AMACR no 4.48093 2.06272 0.0019445 2.17234 2.17234 3.130592598 C1QTNF4 no 10.983 16.2063 0.544764 0.677703 -1.47557 -0.6064242599 C1QTNF5 no 171.967 119.928 0.0479993 1.43391 1.43391 1.986792600 C1QTNF6 no 810.181 688.961 0.437415 1.17595 1.17595 0.777772601 C1QTNF7 no 30.2696 30.3323 0.998405 0.997934 -1.00207 -0.002001162602 C1QTNF8 no 1.89927 2.08935 0.94441 0.909025 -1.10008 -0.06979622603 C1QTNF9 no 3.15163 1.64413 0.0531948 1.9169 1.9169 1.942222604 C1QTNF9B no 9.34098 6.21596 0.650341 1.50274 1.50274 0.4538172605 C1QTNF9B-AS1 no 59.4444 26.8738 0.000126739 2.21198 2.21198 3.891552606 C1R_1 no 1733.38 2885.08 0.0339879 0.600809 -1.66442 -2.131522607 C1R_2 no 865.996 1390.04 0.0628699 0.623003 -1.60513 -1.868152608 C1RL no 884.13 883.832 0.9975 1.00034 1.00034 0.00313692609 C1RL-AS1 no 66.208 95.9933 0.0398172 0.689715 -1.44988 -2.066062610 C1S no 2711.11 4363.58 0.100482 0.621305 -1.60952 -1.648412611 C2 no 1990.26 1756.19 0.611487 1.13328 1.13328 0.508572612 C20orf112 no 839.521 489.453 0.0400126 1.71522 1.71522 2.064012613 C20orf141 no 0.661515 6.24353 0.490154 0.105952 -9.43823 -0.6910482614 C20orf144 no 0.0185835 0.0239917 0.818207 0.77458 -1.29102 -0.2300862615 C20orf166 no 0.25304 0.342042 0.530381 0.739793 -1.35173 -0.628272616 C20orf166-AS1 no 3.7628 4.89618 0.565365 0.768518 -1.30121 -0.575632617 C20orf173 no 0.545195 0.617713 0.644269 0.882603 -1.13301 -0.462282618 C20orf181 no 3.32521 1.5146 0.0578048 2.19543 2.19543 1.905632619 C20orf194 no 355.849 372.115 0.76282 0.956289 -1.04571 -0.3020972620 C20orf195 no 21.7586 19.4262 0.752306 1.12007 1.12007 0.3159352621 C20orf196 no 212.629 152.664 0.000797826 1.39279 1.39279 3.393622622 C20orf197 no 7.92944 26.2054 0.215705 0.302588 -3.30482 -1.241072623 C20orf201 no 0.950277 2.24333 0.648681 0.423602 -2.36071 -0.4561272624 C20orf202 no 4.85676 4.43181 0.768709 1.09589 1.09589 0.2943722625 C20orf203 no 5.68284 3.28733 0.00201857 1.72871 1.72871 3.119172626 C20orf24 no 2535.96 2709.89 0.49401 0.935815 -1.06859 -0.6849152627 C20orf26 no 27.0147 27.7667 0.944273 0.972918 -1.02784 -0.06996812628 C20orf27 no 1077.88 796.648 0.00777745 1.35302 1.35302 2.68252629 C20orf78 no 0.605917 0.292629 0.0031561 2.0706 2.0706 2.980032630 C20orf85 no 42.3618 20.2109 0.537194 2.09599 2.09599 0.6178852631 C20orf96 no 392.263 192.717 7.84E-07 2.03544 2.03544 5.063062632 C21orf119 no 123.075 102.795 0.202865 1.19728 1.19728 1.276662633 C21orf128 no 0.0375211 0.0402745 0.929015 0.931635 -1.07338 -0.0891712634 C21orf15 no 17.4379 163.41 0.149807 0.106713 -9.37097 -1.44452635 C21orf2 no 630.81 460.002 0.00622097 1.37132 1.37132 2.758482636 C21orf33 no 2267.45 1645.18 2.06E-05 1.37824 1.37824 4.337762638 C21orf49 no 9.28721 12.3942 0.172806 0.749322 -1.33454 -1.367022639 C21orf54 no 0.205749 0.0862192 0.0374073 2.38635 2.38635 2.092062640 C21orf58 no 299.302 331.071 0.507842 0.904041 -1.10614 -0.6631222641 C21orf59 no 1268.1 1014.43 0.0106591 1.25006 1.25006 2.572352642 C21orf62 no 36.2355 16.7458 0.00452711 2.16385 2.16385 2.863912643 C21orf67 no 38.2996 63.6996 0.00635562 0.601253 -1.66319 -2.751272644 C21orf88 no 0.161598 0.0777628 0.0459972 2.07809 2.07809 2.005062645 C21orf90 no 0.0693233 0.0786454 0.837059 0.881468 -1.13447 -0.2058642646 C21orf91 no 519.33 561.907 0.548049 0.924229 -1.08198 -0.6014772647 C21orf91-OT1 no 5.69235 6.56492 0.517844 0.867086 -1.15329 -0.6475592648 C22orf15 no 7.55616 3.40973 0.101524 2.21606 2.21606 1.643352649 C22orf23 no 475.146 708.726 0.0803051 0.670423 -1.4916 -1.75582650 C22orf24 no 4.24411 1.8943 1.28E-05 2.24046 2.24046 4.45022651 C22orf26 no 11.8121 12.2779 0.825695 0.962065 -1.03943 -0.2204492652 C22orf29 no 19.3245 10.5437 0.000360665 1.8328 1.8328 3.615142653 C22orf31 no 8.50321 5.8498 0.255038 1.45359 1.45359 1.140732654 C22orf34 no 56.9346 32.2194 0.0203709 1.76709 1.76709 2.333812655 C22orf39 no 507.01 422.554 0.0477219 1.19987 1.19987 1.989282656 C22orf42 no 0.570197 0.426004 0.385591 1.33848 1.33848 0.8691112657 C22orf43 no 17.0232 12.0834 0.0451465 1.40881 1.40881 2.013032658 C22orf46 no 362.183 470.321 0.0157189 0.770077 -1.29857 -2.431412659 C2CD2 no 640.14 796.064 0.107115 0.804131 -1.24358 -1.616912660 C2CD2L no 413.399 315.121 0.0342102 1.31187 1.31187 2.128852661 C2CD3 no 616.407 649.748 0.597071 0.948686 -1.05409 -0.529272662 C2CD4A no 225.948 103.89 0.00724026 2.17487 2.17487 2.707032663 C2CD4B no 51.7121 101.658 0.101843 0.508687 -1.96585 -1.641812664 C2CD4C no 56.2932 61.8687 0.802594 0.909881 -1.09904 -0.2502482665 C2CD4D no 20.1309 34.2344 0.0411554 0.588033 -1.70058 -2.05222666 C2CD5 no 1365.07 991.687 0.0224283 1.37652 1.37652 2.29682667 C2orf15 no 37.3471 21.8093 0.000955755 1.71244 1.71244 3.341622668 C2orf16 no 25.4228 21.8507 0.263408 1.16348 1.16348 1.120812669 C2orf27A no 220.934 237.799 0.63029 0.929079 -1.07634 -0.4818912670 C2orf27AP1 no 0.25996 0.243253 0.95571 1.06868 1.06868 0.05559192671 C2orf27B no 2.86241 2.9704 0.910321 0.963644 -1.03773 -0.1127432673 C2orf42 no 220.785 192.093 0.0137907 1.14937 1.14937 2.479572674 C2orf43 no 490.706 273.203 2.30E-06 1.79612 1.79612 4.833492675 C2orf44 no 259.755 232.32 0.151579 1.11809 1.11809 1.438222676 C2orf47 no 383.739 334.193 0.0852716 1.14826 1.14826 1.727492677 C2orf48 no 167.534 217.608 0.310479 0.769892 -1.29888 -1.016212678 C2orf49 no 519.099 429.787 0.00114717 1.2078 1.2078 3.28842679 C2orf50 no 14.6675 25.448 0.472453 0.576373 -1.73499 -0.7195472680 C2orf53 no 0.0567204 1.01252 0.775239 0.056019 -17.8511 -0.2858272681 C2orf54 no 390.545 755.684 0.0889522 0.51681 -1.93495 -1.707372682 C2orf57 no 0.0374858 0.0519416 0.673173 0.72169 -1.38564 -0.4222772683 C2orf61 no 9.62018 16.3593 0.182563 0.588056 -1.70052 -1.336492684 C2orf62 no 18.2649 19.077 0.950871 0.957427 -1.04447 -0.06167272685 C2orf66 no 23.6952 9.64387 0.000119545 2.45702 2.45702 3.906532686 C2orf68 no 146.973 135.842 0.560791 1.08193 1.08193 0.5824192687 C2orf69 no 435.349 349.765 0.00477837 1.24469 1.24469 2.846212688 C2orf70 no 35.8373 28.4525 0.6163 1.25955 1.25955 0.5017062689 C2orf71 no 3.65452 2.71302 0.393804 1.34703 1.34703 0.8541632690 C2orf72 no 70.2375 75.5925 0.909318 0.92916 -1.07624 -0.1140112691 C2orf73 no 2.42931 2.5869 0.918261 0.93908 -1.06487 -0.1027252692 C2orf74 no 154.223 175.035 0.505142 0.881097 -1.13495 -0.667352693 C2orf76 no 174.058 146.987 0.0548847 1.18417 1.18417 1.928512694 C2orf78 no 0.759714 0.438583 0.593947 1.7322 1.7322 0.5337862696 C2orf81 no 151.572 108.909 0.165482 1.39173 1.39173 1.390792697 C2orf82 no 32.1393 16.4648 0.43185 1.952 1.952 0.7872592698 C2orf83 no 0.754261 0.638643 0.856256 1.18104 1.18104 0.1813232699 C2orf88 no 907.623 486.77 0.163058 1.86458 1.86458 1.398842700 C2orf91 no 7.27071 5.9373 0.705371 1.22458 1.22458 0.3784982701 C3 no 2371.96 9278.32 0.114932 0.255646 -3.91166 -1.581732702 C3AR1 no 122.353 165.89 0.26157 0.737559 -1.35582 -1.125142703 C3orf14 no 92.8709 104.907 0.713696 0.885269 -1.1296 -0.3672992704 C3orf17 no 948.408 1060.32 0.239924 0.894458 -1.118 -1.177882705 C3orf18 no 140.718 53.8362 1.83E-08 2.61382 2.61382 5.809932706 C3orf20 no 56.5874 33.9418 0.00439683 1.66719 1.66719 2.873452707 C3orf22 no 5.99081 3.59483 0.503479 1.66651 1.66651 0.6699622708 C3orf27 no 0.533817 0.135179 0.00320876 3.94896 3.94896 2.974792709 C3orf30 no 3.04432 3.14585 0.970321 0.967726 -1.03335 -0.03724112710 C3orf33 no 131.11 90.3098 0.00410823 1.45178 1.45178 2.895522711 C3orf35 no 32.1183 22.5681 0.0179708 1.42317 1.42317 2.381382712 C3orf36 no 23.6421 10.3867 0.0408785 2.27619 2.27619 2.055042713 C3orf37 no 1825.64 2500.67 0.00585371 0.730061 -1.36975 -2.778912714 C3orf38 no 383.877 357.101 0.349662 1.07498 1.07498 0.9369412715 C3orf43 no 1.19071 1.44636 0.456512 0.823246 -1.2147 -0.7457222716 C3orf49 no 16.6958 15.0313 0.424229 1.11074 1.11074 0.8003722717 C3orf52 no 197.718 325.694 0.00496597 0.607066 -1.64727 -2.833542719 C3orf56 no 0 0.0249313 0.371916 ? ? -0.8944422720 C3orf58 no 704.952 901.69 0.274415 0.781812 -1.27908 -1.095272721 C3orf62 no 181.751 171.302 0.578915 1.06099 1.06099 0.5556722722 C3orf65 no 0 0 ? 1 1 02723 C3orf67 no 158.74 145.852 0.705514 1.08837 1.08837 0.3783052724 C3orf70 no 77.9264 117.426 0.184948 0.663619 -1.50689 -1.329212725 C3orf72 no 109.619 38.1312 0.00711095 2.87479 2.87479 2.713182726 C3orf79 no 2.11718 1.34991 0.207789 1.56839 1.56839 1.262822727 C3orf80 no 33.943 37.8231 0.680848 0.897415 -1.11431 -0.4117712728 C3orf83 no 35.8611 73.4232 0.00393343 0.488416 -2.04743 -2.909592729 C3orf84 no 1.05058 2.33012 0.691963 0.450869 -2.21794 -0.3966362730 C3P1 no 1.13724 2.31155 0.686148 0.491981 -2.0326 -0.4045422731 C4A no 1474.25 1347.27 0.843202 1.09425 1.09425 0.1979992732 C4B no 1053.34 1653.03 0.402545 0.637215 -1.56933 -0.8384612733 C4BPA no 166.91 393.422 0.557603 0.424253 -2.35709 -0.5871672734 C4BPAP1 no 0.598083 0.88781 0.678874 0.673661 -1.48443 -0.4144692735 C4BPAP2 no 4.82426 1.48715 0.23462 3.24396 3.24396 1.191322736 C4BPB no 64.3511 213.624 0.433007 0.301236 -3.31966 -0.785282737 C4orf17 no 1.32638 0.735511 0.155999 1.80334 1.80334 1.422792738 C4orf19 no 212.237 215.304 0.968571 0.985751 -1.01445 -0.03943932739 C4orf21 no 231.984 289.538 0.0884152 0.801221 -1.2481 -1.710262740 C4orf22 no 18.7803 12.8726 0.304433 1.45894 1.45894 1.029012741 C4orf26 no 2.56184 7.86555 0.33008 0.325703 -3.07028 -0.9757882742 C4orf27 no 532.848 483.554 0.248534 1.10194 1.10194 1.156522743 C4orf29 no 197.214 170.4 0.167353 1.15736 1.15736 1.384652744 C4orf3 no 2411.3 2186.37 0.2411 1.10288 1.10288 1.174942745 C4orf32 no 127.372 203.973 0.00253292 0.624457 -1.60139 -3.049132746 C4orf33 no 188.899 259.233 0.030845 0.728683 -1.37234 -2.170932747 C4orf36 no 43.5423 83.5146 0.000362395 0.521373 -1.91801 -3.613842748 C4orf40 no 0.903883 0.6627 0.859827 1.36394 1.36394 0.176772749 C4orf45 no 139.262 85.7041 0.00999364 1.62491 1.62491 2.595172750 C4orf46 no 360.21 341.482 0.62507 1.05484 1.05484 0.4892632751 C4orf47 no 54.9176 50.989 0.795213 1.07705 1.07705 0.2598142752 C4orf48 no 167.928 60.6039 1.53E-07 2.77092 2.77092 5.397182753 C4orf50 no 10.6428 4.85202 0.00428109 2.19348 2.19348 2.882142754 C4orf51 no 79.7463 79.9424 0.997523 0.997547 -1.00246 -0.003106852755 C4orf6 no 1.79193 3.38663 0.453102 0.529118 -1.88994 -0.7513872756 C5 no 136.781 182.844 0.123185 0.748075 -1.33676 -1.546582757 C5AR1 no 151.654 200.093 0.148514 0.757919 -1.3194 -1.449122758 C5AR2 no 14.1957 24.7464 0.570642 0.57365 -1.74322 -0.5678312759 C5orf15 no 1594.34 1430.38 0.219725 1.11463 1.11463 1.230242760 C5orf20 no 13.419 19.691 0.327863 0.681479 -1.4674 -0.980282761 C5orf22 no 576.097 623.418 0.492164 0.924094 -1.08214 -0.6878482762 C5orf24 no 1216.23 944.745 0.00155025 1.28736 1.28736 3.19912763 C5orf27 no 5.74475 9.23192 0.344326 0.622271 -1.60702 -0.9473842764 C5orf28 no 307.514 329.59 0.567169 0.933019 -1.07179 -0.572962765 C5orf30 no 276.692 277.439 0.981737 0.997306 -1.0027 -0.02291372766 C5orf34 no 279.775 384.295 0.0226841 0.728021 -1.37359 -2.292412767 C5orf38 no 33.472 114.152 0.163137 0.293223 -3.41038 -1.398582768 C5orf42 no 461.231 482.222 0.793549 0.956471 -1.04551 -0.2619752769 C5orf45 no 386.36 256.318 2.17E-05 1.50735 1.50735 4.326392770 C5orf46 no 7.57093 115.855 0.692179 0.0653483 -15.3026 -0.3963422771 C5orf47 no 0.869789 0.399942 0.00230552 2.17479 2.17479 3.078312772 C5orf48 no 1.77966 0.646907 2.15E-05 2.75103 2.75103 4.328032773 C5orf49 no 59.1002 65.7483 0.888642 0.898887 -1.11249 -0.140162774 C5orf50 no 0.780464 0.37508 0.0254331 2.08079 2.08079 2.247762775 C5orf51 no 761.407 760.249 0.988438 1.00152 1.00152 0.01450552777 C5orf54 no 180.842 143.878 0.0374935 1.25691 1.25691 2.09112778 C5orf55 no 80.6252 69.2747 0.444598 1.16385 1.16385 0.7656232779 C5orf56 no 187.745 332.234 0.0284053 0.565097 -1.76961 -2.203992780 C5orf58 no 4.41434 4.113 0.816067 1.07327 1.07327 0.2328442781 C5orf60 no 4.11711 1.87848 0.000527577 2.19172 2.19172 3.51042782 C5orf63 no 111.3 101.012 0.480552 1.10185 1.10185 0.7064362783 C5orf64 no 0.969295 0.531591 0.0336778 1.82338 1.82338 2.135272784 C6 no 9.43258 7.37589 0.78347 1.27884 1.27884 0.2750862785 C6orf1 no 429.969 354.042 0.082218 1.21446 1.21446 1.744732786 C6orf10 no 7.31194 6.63156 0.865306 1.1026 1.1026 0.1697932787 C6orf100 no 2.05745 1.64276 0.528199 1.25244 1.25244 0.6316092788 C6orf106 no 2908.69 3449.76 0.0507613 0.843159 -1.18602 -1.962622789 C6orf118 no 16.2379 24.8113 0.716345 0.654455 -1.52799 -0.3637462790 C6orf120 no 219.018 170.36 0.0262039 1.28561 1.28561 2.2362791 C6orf123 no 23.6552 11.6603 0.13373 2.0287 2.0287 1.504272792 C6orf132 no 879.141 1423.43 0.00279437 0.617622 -1.61911 -3.018432793 C6orf136 no 474.314 431.571 0.239384 1.09904 1.09904 1.179242794 C6orf141 no 79.8184 174.037 0.0612216 0.458628 -2.18041 -1.880062795 C6orf147 no 1.19816 2.80826 0.00433649 0.426654 -2.34382 -2.877952797 C6orf163 no 26.7913 14.4093 3.47E-05 1.85931 1.85931 4.213732798 C6orf164 no 10.4254 4.59058 6.99E-07 2.27105 2.27105 5.086952799 C6orf165 no 62.3658 28.3022 0.0296212 2.20356 2.20356 2.187222800 C6orf183 no 2.1399 1.4324 0.33552 1.49393 1.49393 0.964852801 C6orf195 no 3.48429 7.59572 0.0189496 0.458718 -2.17999 -2.361352802 C6orf201 no 3.28453 1.88049 0.0958613 1.74663 1.74663 1.671352803 C6orf203 no 354.476 325.522 0.669088 1.08895 1.08895 0.4278882804 C6orf211 no 642.83 522.646 0.0159449 1.22995 1.22995 2.426122805 C6orf222 no 113.089 25.4744 0.0856852 4.43934 4.43934 1.725192806 C6orf223 no 264.596 151.894 0.0429123 1.74197 1.74197 2.034572807 C6orf226 no 121.524 84.9136 0.00575124 1.43115 1.43115 2.784812808 C6orf25 no 25.8473 15.3929 0.0287784 1.67917 1.67917 2.198782809 C6orf47 no 865.298 870.599 0.938945 0.993911 -1.00613 -0.07667022810 C6orf48 no 1983.75 1410.11 0.00254567 1.40681 1.40681 3.047572811 C6orf52 no 111.704 30.1536 1.48E-11 3.70451 3.70451 7.06522812 C6orf57 no 148.183 108.205 3.02E-05 1.36947 1.36947 4.247292813 C6orf58 no 3.3851 1.01363 0.0252864 3.33957 3.33957 2.250042814 C6orf62 no 4168.36 4056.64 0.73249 1.02754 1.02754 0.3421842815 C6orf7 no 0.583416 0.476998 0.562253 1.2231 1.2231 0.5802462816 C6orf70 no 390.186 359.132 0.3238 1.08647 1.08647 0.9885632817 C6orf89 no 1937.64 1785.31 0.263337 1.08533 1.08533 1.120972818 C6orf99 no 21.017 13.7018 0.0119944 1.53389 1.53389 2.530162819 C7 no 99.1138 67.7563 0.688786 1.4628 1.4628 0.4009522820 C7orf10 no 143.362 110.608 0.152814 1.29613 1.29613 1.433872821 C7orf13 no 239.825 233.782 0.892225 1.02585 1.02585 0.1356222822 C7orf25 no 273.158 271.145 0.916913 1.00742 1.00742 0.1044252823 C7orf26 no 716.148 526.613 2.61E-07 1.35991 1.35991 5.289352824 C7orf31 no 135.53 70.5114 1.22E-05 1.9221 1.9221 4.459522825 C7orf33 no 0.962873 0.42212 0.00278883 2.28104 2.28104 3.019052826 C7orf34 no 0.410979 1.32824 0.255089 0.309417 -3.23189 -1.14062827 C7orf41 no 826.231 453.115 0.00038179 1.82344 1.82344 3.599622828 C7orf43 no 539.879 455.643 0.0140055 1.18487 1.18487 2.473922829 C7orf49 no 714.013 658.416 0.291895 1.08444 1.08444 1.056122830 C7orf50 no 2007.24 1606.1 0.030457 1.24976 1.24976 2.176032831 C7orf55 no 387.707 196.019 2.56E-07 1.9779 1.9779 5.293322832 C7orf55-LUC7L2 no 79.6796 64.8924 0.166257 1.22787 1.22787 1.388242833 C7orf57 no 22.4019 58.5047 0.306415 0.382909 -2.61159 -1.02482834 C7orf60 no 330.703 242.279 0.000264083 1.36497 1.36497 3.699222835 C7orf61 no 26.2132 18.0768 0.0239551 1.4501 1.4501 2.271212836 C7orf62 no 0 0 ? 1 1 02837 C7orf63 no 89.0316 71.8732 0.407689 1.23873 1.23873 0.8293172838 C7orf65 no 0.205458 0.663448 0.183515 0.309682 -3.22912 -1.333582839 C7orf66 no 0.0356898 0.0173167 0.403693 2.06101 2.06101 0.8364142840 C7orf69 no 0.568339 0.25704 0.000792068 2.21109 2.21109 3.395692841 C7orf71 no 2.32226 1.92129 0.354715 1.2087 1.2087 0.9271442842 C7orf72 no 0.686823 0.32507 0.00668593 2.11285 2.11285 2.734132843 C7orf73 no 1907.8 1491.54 0.00246305 1.27908 1.27908 3.057832844 C7orf76 no 1.59334 0.739323 0.0189509 2.15514 2.15514 2.361322845 C8A no 0.536633 0.598939 0.891456 0.895972 -1.11611 -0.1365962846 C8B no 1.72658 0.964388 0.237129 1.79034 1.79034 1.184942847 C8G no 10.4449 31.2184 0.145674 0.334573 -2.98888 -1.459372848 C8orf12 no 7.28478 4.56339 0.152294 1.59635 1.59635 1.43572849 C8orf22 no 0.0397889 2.98367 0.526134 0.0133356 -74.9875 -0.6347772850 C8orf31 no 81.3525 107.39 0.738899 0.75754 -1.32006 -0.3336692851 C8orf33 no 1419.88 924.01 1.84E-08 1.53665 1.53665 5.808892853 C8orf37 no 81.6963 73.6608 0.396614 1.10909 1.10909 0.8490932854 C8orf4 no 2098.76 1169.92 0.0762282 1.79393 1.79393 1.780122855 C8orf44 no 108.515 72.8736 0.000745537 1.48909 1.48909 3.412972856 C8orf44-SGK3 no 390.721 327.432 0.0508091 1.19329 1.19329 1.962212857 C8orf46 no 12.368 6.13508 0.00620224 2.01594 2.01594 2.75952858 C8orf47 no 141.062 51.375 0.000227289 2.74573 2.74573 3.739162859 C8orf48 no 7.99274 2.98251 0.00391893 2.67987 2.67987 2.910792860 C8orf49 no 1.085 0.609873 0.501265 1.77906 1.77906 0.6734452861 C8orf56 no 13.1587 4.3449 0.00639932 3.02853 3.02853 2.748952862 C8orf58 no 227.693 228.049 0.989646 0.998439 -1.00156 -0.01298922863 C8orf59 no 1226.11 816.782 3.93E-07 1.50115 1.50115 5.206372864 C8orf69 no 0.768872 1.07025 0.771698 0.718404 -1.39197 -0.2904592865 C8orf74 no 14.278 35.5104 0.119092 0.402079 -2.48707 -1.563772866 C8orf76 no 630.033 518.352 0.00354009 1.21546 1.21546 2.943472867 C8orf82 no 476.288 331.545 0.002647 1.43657 1.43657 3.035392869 C8orf87 no 0.232932 0.241113 0.955196 0.966072 -1.03512 -0.05623722870 C9 no 4.10504 36.6583 0.425967 0.111981 -8.93008 -0.7973692871 C9orf106 no 1.12841 1.01767 0.801547 1.10882 1.10882 0.2516042872 C9orf114 no 701.792 666.504 0.523272 1.05295 1.05295 0.6391772873 C9orf116 no 106.841 62.5206 0.0163092 1.7089 1.7089 2.417712874 C9orf117 no 112.934 93.5669 0.37612 1.20699 1.20699 0.8865942875 C9orf123 no 821.992 688.076 0.417604 1.19462 1.19462 0.8118842876 C9orf129 no 48.2932 17.4716 0.00020142 2.7641 2.7641 3.771082877 C9orf131 no 1.80854 2.1014 0.615463 0.860638 -1.16193 -0.5028982878 C9orf135 no 11.1698 3.45216 0.0565645 3.2356 3.2356 1.915232880 C9orf139 no 16.8054 23.4333 0.24956 0.71716 -1.39439 -1.154012881 C9orf142 no 796.872 922.354 0.239401 0.863954 -1.15747 -1.17922882 C9orf152 no 217.217 227.665 0.924189 0.954108 -1.0481 -0.09525062883 C9orf153 no 4.9482 3.41855 0.0430118 1.44746 1.44746 2.03362884 C9orf156 no 282.558 281.1 0.948339 1.00519 1.00519 0.06485512885 C9orf16 no 1454.12 1218.34 0.18817 1.19352 1.19352 1.319492886 C9orf163 no 11.5954 10.1406 0.426847 1.14346 1.14346 0.7958532888 C9orf170 no 4.47256 8.95359 0.122253 0.499527 -2.00189 -1.550462889 C9orf171 no 35.6275 37.5114 0.932835 0.94978 -1.05288 -0.08435992890 C9orf172 no 35.4808 66.3597 0.340914 0.534673 -1.8703 -0.9541182892 C9orf24 no 30.5087 76.4269 0.41455 0.399187 -2.50509 -0.8172262893 C9orf3 no 1626.71 2220.89 0.185689 0.732458 -1.36527 -1.326962894 C9orf37 no 260.611 262.239 0.948713 0.993792 -1.00625 -0.06438512895 C9orf40 no 224.223 272.947 0.0824223 0.82149 -1.2173 -1.743562896 C9orf41 no 452.331 491.938 0.323562 0.919487 -1.08756 -0.989052897 C9orf43 no 19.0725 14.5129 0.027851 1.31418 1.31418 2.211842898 C9orf47 no 0.359165 0.0930075 0.00497053 3.86167 3.86167 2.833232899 C9orf50 no 3.23494 1.49333 0.00809008 2.16626 2.16626 2.668922900 C9orf53 no 3.38911 9.74071 0.000130104 0.347933 -2.87412 -3.884812901 C9orf57 no 1.30208 0.571635 0.000137456 2.27782 2.27782 3.870652902 C9orf62 no 0 0.0273879 0.666176 ? ? -0.4318962903 C9orf64 no 386.781 379.67 0.877906 1.01873 1.01873 0.1537762904 C9orf66 no 14.3206 10.3635 0.116287 1.38182 1.38182 1.575822905 C9orf69 no 1204.82 1389.52 0.0952673 0.867078 -1.1533 -1.674362906 C9orf72 no 312.674 315.744 0.936419 0.990277 -1.00982 -0.07984952907 C9orf78 no 1373.15 1363.32 0.910034 1.00721 1.00721 0.1131062908 C9orf84 no 71.5619 194.807 0.000295929 0.367347 -2.72222 -3.668682909 C9orf85 no 158.662 175.134 0.285149 0.905948 -1.10382 -1.071042910 C9orf89 no 420.397 518.462 0.0796582 0.810853 -1.23327 -1.759592911 C9orf9 no 158.41 116.189 0.0147323 1.36339 1.36339 2.455362912 C9orf91 no 427.944 461.56 0.508193 0.92717 -1.07855 -0.6625732913 C9orf92 no 4.16232 2.662 0.289338 1.5636 1.5636 1.061752914 C9orf96 no 29.7686 25.298 0.17442 1.17672 1.17672 1.361882915 CA1 no 7.56577 4.65674 0.058372 1.62469 1.62469 1.90132916 CA10 no 47.2428 57.2729 0.943311 0.824871 -1.21231 -0.07117842917 CA11 no 337.489 252.131 0.0611324 1.33855 1.33855 1.880722918 CA12 no 1568.45 2891.37 0.0907623 0.542457 -1.84346 -1.697722919 CA13 no 152.92 117.451 0.139552 1.30199 1.30199 1.482012921 CA15P2 no 0.0155072 0.130484 0.0326899 0.118844 -8.4144 -2.14742922 CA2 no 293.224 1565.73 0.108496 0.187276 -5.3397 -1.610552923 CA3 no 39.4915 32.9534 0.430966 1.1984 1.1984 0.7887732926 CA5AP1 no 0.534782 0.45722 0.596765 1.16964 1.16964 0.5297132927 CA5B no 298.207 197.476 0.00131939 1.51009 1.51009 3.247162928 CA5BP1 no 506.355 539.098 0.510242 0.939264 -1.06466 -0.6593732929 CA6 no 2.89364 2.40266 0.687393 1.20435 1.20435 0.4028472932 CA9 no 946.301 1843.98 0.114006 0.513185 -1.94862 -1.58582933 CAAP1 no 546.667 456.921 0.0289343 1.19642 1.19642 2.196622934 CAB39 no 1880.55 2026.39 0.445144 0.928027 -1.07756 -0.7647052935 CAB39L no 586.199 234.363 2.77E-09 2.50124 2.50124 6.159652936 CABIN1 no 2277.73 1546.39 2.83E-06 1.47293 1.47293 4.788232937 CABLES1 no 663.334 362.766 0.000724919 1.82854 1.82854 3.420952938 CABLES2 no 399.067 481.479 0.105023 0.828836 -1.20651 -1.626672941 CABP4 no 60.3611 68.4009 0.731339 0.882461 -1.13319 -0.3437152942 CABP5 no 0.573827 0.378636 0.393854 1.51551 1.51551 0.8540722945 CABYR no 274.266 116.212 0.000876511 2.36005 2.36005 3.366612946 CABYRP1 no 0.0129226 0.0688447 0.2045 0.187706 -5.32748 -1.272042947 CACFD1 no 892.543 540.144 1.13E-05 1.65242 1.65242 4.479112948 CACHD1 no 478.947 321.66 0.0301808 1.48899 1.48899 2.17972949 CACNA1A no 49.0184 42.084 0.533788 1.16477 1.16477 0.6230692950 CACNA1B no 145.639 60.2021 0.0142334 2.41916 2.41916 2.468012951 CACNA1C no 204.67 114.424 0.00277755 1.78869 1.78869 3.020322952 CACNA1C-AS1 no 0.0675799 0.0609587 0.867886 1.10862 1.10862 0.1665092953 CACNA1C-AS4 no 0 0 ? 1 1 02954 CACNA1C-IT3 no 0 0 ? 1 1 02955 CACNA1D no 300.839 143.529 0.0209724 2.09601 2.09601 2.322672957 CACNA1F no 26.813 31.7337 0.751594 0.844939 -1.18352 -0.3168742959 CACNA1G-AS1 no 4.86221 1.55836 0.00461548 3.12008 3.12008 2.857592960 CACNA1H no 193.426 145.793 0.57698 1.32672 1.32672 0.5585072961 CACNA1I no 50.6462 17.6659 0.00755569 2.86689 2.86689 2.692442963 CACNA2D1 no 233.796 269.05 0.653485 0.86897 -1.15079 -0.4494472965 CACNA2D3 no 272.772 152.516 0.270995 1.78848 1.78848 1.103132966 CACNA2D3-AS1 no 0 0.00236766 0.768013 ? ? -0.2952842967 CACNA2D4 no 54.9579 48.4012 0.371526 1.13547 1.13547 0.8951732968 CACNB1 no 213.245 242.443 0.623553 0.879566 -1.13692 -0.491412969 CACNB2 no 120.933 84.7465 0.00341015 1.42699 1.42699 2.955422970 CACNB3 no 689.789 624.146 0.320351 1.10517 1.10517 0.9956462971 CACNB4 no 53.5758 33.1757 0.0695392 1.61491 1.61491 1.822452972 CACNG1 no 1.80139 2.09955 0.809678 0.85799 -1.16551 -0.2410872973 CACNG2 no 2.40952 1.62666 0.232223 1.48127 1.48127 1.197462974 CACNG3 no 1.6498 0.900496 0.00926534 1.83211 1.83211 2.621762975 CACNG4 no 228.761 279.76 0.628278 0.817706 -1.22293 -0.4847292977 CACNG6 no 28.9217 27.1709 0.955624 1.06444 1.06444 0.05570022978 CACNG7 no 2.66083 1.29309 0.150565 2.05772 2.05772 1.441812979 CACNG8 no 9.29838 6.38865 0.698217 1.45545 1.45545 0.3881592980 CACTIN no 621.176 629.135 0.88275 0.98735 -1.01281 -0.147632981 CACTIN-AS1 no 2.03284 2.41901 0.597792 0.84036 -1.18997 -0.528232982 CACUL1 no 1212.08 1372.68 0.105275 0.883006 -1.13249 -1.625492983 CACYBP no 3015.44 2323.68 0.00168768 1.2977 1.2977 3.173552984 CACYBPP1 no 0 0 ? 1 1 02985 CACYBPP2 no 2.65875 1.85787 0.0438014 1.43108 1.43108 2.025892986 CAD no 2269.68 1712.16 0.000700572 1.32563 1.32563 3.430662989 CADM2-AS1 no 0 0.00553875 0.622703 ? ? -0.4926132990 CADM2-AS2 no 0 0 ? 1 1 02991 CADM3 no 12.1212 27.7124 0.210508 0.437393 -2.28627 -1.255282992 CADM4 no 1203.6 933.072 0.158689 1.28993 1.28993 1.413572993 CADPS no 59.1439 22.3765 0.0153993 2.64312 2.64312 2.439022994 CADPS2 no 848.63 810.755 0.79532 1.04672 1.04672 0.2596762995 CAGE1 no 17.2288 43.8509 0.0365466 0.392895 -2.54521 -2.101692996 CAHM no 9.18184 8.53255 0.698569 1.0761 1.0761 0.3876832997 CALB1 no 127.481 668.782 0.434142 0.190617 -5.24614 -0.7833412998 CALB2 no 186.363 142.431 0.781785 1.30844 1.30844 0.2772822999 CALCA no 27.111 14.8963 0.724359 1.81998 1.81998 0.3530223001 CALCOCO1 no 1046.26 1162.64 0.264485 0.899904 -1.11123 -1.118283002 CALCOCO2 no 1519.9 1901.47 0.00707464 0.799328 -1.25105 -2.714933003 CALCP no 0 0.0395837 0.156906 ? ? -1.419673004 CALCR no 6.36162 2.19275 2.15E-07 2.90121 2.90121 5.328853005 CALCRL no 290.77 215.132 0.20351 1.35159 1.35159 1.274843006 CALD1 no 2719.35 2545.39 0.734629 1.06834 1.06834 0.339343007 CALHM1 no 4.08276 1.47148 0.00938479 2.77459 2.77459 2.617273008 CALHM2 no 277.623 296.946 0.657475 0.934928 -1.0696 -0.4439153009 CALHM3 no 15.6967 9.87394 0.453442 1.58971 1.58971 0.750823010 CALM1 no 7795.82 7493.34 0.669118 1.04037 1.04037 0.4278483011 CALM1P1 no 0 0.00116041 0.768013 ? ? -0.2952843013 CALM2 no 8508.89 8352.53 0.8177 1.01872 1.01872 0.2307393014 CALM2P1 no 0.0706176 0.0399112 0.751042 1.76937 1.76937 0.3176023015 CALM2P2 no 0 0.00275353 0.678163 ? ? -0.4154413016 CALM2P3 no 0 0.0643673 0.061825 ? ? -1.875673017 CALM2P4 no 0 0.0967392 0.0579296 ? ? -1.904683018 CALM3 no 5268.77 5478.27 0.631445 0.961758 -1.03976 -0.4802653019 CALML3 no 8977.3 10838.6 0.586926 0.828269 -1.20734 -0.5439763020 CALML4 no 539.001 648.489 0.366422 0.831165 -1.20313 -0.9047823021 CALML5 no 857.345 3399.17 0.0842976 0.252222 -3.96476 -1.732933022 CALML6 no 12.3841 11.4356 0.698229 1.08294 1.08294 0.3881433024 CALR no 29698.7 24793.9 0.0380903 1.19782 1.19782 2.084553025 CALR3 no 2.48171 1.30305 0.000904607 1.90454 1.90454 3.357523026 CALR4P no 3.83805 2.22405 0.0306706 1.7257 1.7257 2.173223027 CALU no 4033.23 2867.74 0.000476228 1.40641 1.40641 3.538843028 CALY no 12.0832 4.17655 0.0542837 2.89312 2.89312 1.933343029 CAMK1 no 335.726 247.853 0.00845234 1.35454 1.35454 2.653763030 CAMK1D no 175.71 201.849 0.435382 0.870504 -1.14876 -0.7812283031 CAMK1G no 36.0544 13.9911 2.20E-10 2.57695 2.57695 6.608343032 CAMK2A no 9.97355 24.9572 0.113241 0.399626 -2.50234 -1.589183033 CAMK2B no 69.1284 146.779 0.562567 0.470969 -2.12328 -0.579783034 CAMK2D no 675.391 940.853 0.0509896 0.71785 -1.39305 -1.960673035 CAMK2G no 832.329 907.437 0.39467 0.917231 -1.09024 -0.8525993036 CAMK2N1 no 1049.89 974.338 0.779299 1.07755 1.07755 0.2805253037 CAMK2N2 no 14.8358 33.5384 0.321176 0.442353 -2.26064 -0.9939483038 CAMK4 no 168.228 141.984 0.456091 1.18484 1.18484 0.7464193039 CAMKK1 no 391.832 471.462 0.343693 0.8311 -1.20322 -0.948633040 CAMKK2 no 1320.59 1164.95 0.0536937 1.1336 1.1336 1.938133041 CAMKMT no 311.927 250.715 0.056871 1.24415 1.24415 1.912843043 CAMLG no 889.957 653.496 4.02E-06 1.36184 1.36184 4.711413044 CAMP no 11.8827 20.3798 0.806177 0.58306 -1.71509 -0.2456123045 CAMSAP1 no 964.897 1266.88 0.041 0.761636 -1.31296 -2.053793046 CAMSAP2 no 979.56 1094.74 0.296542 0.894789 -1.11758 -1.045993047 CAMSAP3 no 834.075 688.262 0.174783 1.21186 1.21186 1.360733048 CAMTA1 no 935.206 899.804 0.643836 1.03934 1.03934 0.4628843049 CAMTA2 no 872.525 808.466 0.316905 1.07924 1.07924 1.002783050 CAND1 no 2509.78 2562.92 0.806477 0.979269 -1.02117 -0.2452243051 CAND1.11 no 9.12424 7.03516 0.318233 1.29695 1.29695 1.000023052 CAND2 no 186.728 112.328 0.0147254 1.66235 1.66235 2.455533053 CANT1 no 1818.53 1746.21 0.710569 1.04142 1.04142 0.37153054 CANX no 13399.7 10462.5 0.00199423 1.28074 1.28074 3.122883055 CAP1 no 5392.94 6670.17 0.0188269 0.808517 -1.23683 -2.363813056 CAP1P1 no 0.646693 0.836957 0.487716 0.772671 -1.29421 -0.694943057 CAP1P2 no 1.77784 1.79138 0.965377 0.992444 -1.00761 -0.04344873058 CAP2 no 300.032 180.215 0.00312608 1.66486 1.66486 2.983063059 CAP2P1 no 0 0.00154943 0.768013 ? ? -0.2952843060 CAPG no 2944.2 5945.22 2.52E-05 0.495222 -2.0193 -4.290143061 CAPN1 no 5950.27 8196.2 0.00225603 0.725979 -1.37745 -3.085013062 CAPN10 no 581.723 376.8 9.14E-07 1.54385 1.54385 5.03083063 CAPN10-AS1 no 60.4061 40.0892 0.0326886 1.50679 1.50679 2.147413064 CAPN11 no 10.8603 9.92007 0.721505 1.09478 1.09478 0.3568363065 CAPN12 no 183.702 256.457 0.256821 0.716305 -1.39605 -1.136443066 CAPN13 no 595.81 300.353 0.108444 1.9837 1.9837 1.610793067 CAPN14 no 26.1744 256.276 0.21322 0.102133 -9.79113 -1.247833068 CAPN15 no 1479.84 1256.02 0.0660888 1.17819 1.17819 1.845623069 CAPN2 no 3283.13 7530.85 1.53E-08 0.435957 -2.29381 -5.843213070 CAPN3 no 146.185 264.817 0.000966637 0.552023 -1.81152 -3.338333071 CAPN5 no 801.865 755.376 0.875505 1.06154 1.06154 0.1568253072 CAPN6 no 590.218 113.122 0.0225381 5.21752 5.21752 2.294913073 CAPN7 no 672.348 524.886 0.00139246 1.28094 1.28094 3.231153074 CAPN8 no 276.016 501.932 0.441764 0.549907 -1.81849 -0.7704023075 CAPN9 no 77.0903 29.7425 0.0639145 2.59192 2.59192 1.860743076 CAPNS1 no 7381.61 8491.64 0.149938 0.86928 -1.15038 -1.444033077 CAPNS1P1 no 0.152728 0.404296 0.161173 0.377762 -2.64717 -1.405163078 CAPNS2 no 0.498392 1.14016 0.102775 0.437125 -2.28767 -1.637333079 CAPRIN1 no 5170.02 4181.92 0.000841094 1.23628 1.23628 3.378483080 CAPRIN2 no 353.851 330.866 0.658304 1.06947 1.06947 0.4427673081 CAPS no 1506.93 1126.73 0.511992 1.33744 1.33744 0.6566453082 CAPS2 no 133.198 102.535 0.102517 1.29905 1.29905 1.638573083 CAPSL no 23.8955 20.9596 0.877664 1.14007 1.14007 0.1540843084 CAPZA1 no 3326.45 4146.43 0.00453458 0.802244 -1.2465 -2.863383085 CAPZA1P no 0 0.0795141 0.0387074 ? ? -2.077863086 CAPZA2 no 1970.87 1695.11 0.0212836 1.16268 1.16268 2.317013087 CAPZA3 no 0.419272 0.505113 0.85637 0.830055 -1.20474 -0.1811773088 CAPZB no 5039.51 5474.18 0.120117 0.920596 -1.08625 -1.559423089 CARD10 no 704.817 769.184 0.673247 0.916317 -1.09132 -0.4221763090 CARD11 no 234.669 351.783 0.141006 0.667084 -1.49906 -1.476573091 CARD14 no 247.263 441.055 0.0403017 0.560618 -1.78375 -2.0613092 CARD16 no 135.098 220.263 0.0122217 0.61335 -1.63039 -2.52343093 CARD17 no 19.635 21.155 0.866271 0.928152 -1.07741 -0.1685643094 CARD18 no 13.8129 7.36502 0.198023 1.87547 1.87547 1.290513095 CARD6 no 121.167 388.2 1.40E-05 0.312124 -3.20385 -4.428443096 CARD8 no 398.767 456.283 0.238686 0.873946 -1.14424 -1.1813097 CARD9 no 12.418 25.2701 0.284323 0.491412 -2.03495 -1.072883098 CARF no 109.809 107.085 0.844945 1.02544 1.02544 0.1957683099 CARHSP1 no 2217.75 3264.58 0.023958 0.679339 -1.47202 -2.271173100 CARKD no 1084.62 974.936 0.182903 1.1125 1.1125 1.335453101 CARM1 no 2094.05 1646.98 0.00259459 1.27145 1.27145 3.041633103 CARNS1 no 40.8465 45.2358 0.84262 0.902969 -1.10746 -0.1987433104 CARS no 1366.17 1217.08 0.152701 1.12249 1.12249 1.434273105 CARS2 no 1297.92 1004.37 0.00194136 1.29228 1.29228 3.131083107 CASC1 no 46.5353 31.7826 0.41143 1.46418 1.46418 0.8227093108 CASC10 no 53.7982 36.4934 0.13815 1.47419 1.47419 1.487313109 CASC11 no 4.87921 38.3779 0.699867 0.127136 -7.86559 -0.3859283110 CASC16 no 3.26331 3.90509 0.830675 0.835656 -1.19666 -0.2140523111 CASC2 no 74.6144 60.5414 0.270693 1.23245 1.23245 1.103833112 CASC3 no 2104 1732.98 0.0334336 1.2141 1.2141 2.138243113 CASC4 no 1605.26 1339.91 0.110127 1.19803 1.19803 1.603123114 CASC4P1 no 0.553808 0.461298 0.488044 1.20054 1.20054 0.6944163115 CASC5 no 256.054 398.586 0.00889723 0.642405 -1.55665 -2.635923116 CASC8 no 3.33261 60.5144 0.775244 0.0550714 -18.1582 -0.285823117 CASC9 no 165.666 88.9605 0.0365232 1.86225 1.86225 2.101953118 CASD1 no 470.282 276.89 2.22E-08 1.69844 1.69844 5.773423119 CASK no 1250.58 1436.2 0.308993 0.870756 -1.14843 -1.019343120 CASKIN1 no 78.9444 21.9148 8.35E-09 3.60234 3.60234 5.956983121 CASKIN2 no 818.384 632.761 0.00943995 1.29335 1.29335 2.615223122 CASKP1 no 12.5884 10.1927 0.289492 1.23504 1.23504 1.061413123 CASP1 no 397.684 759.919 0.00157431 0.523325 -1.91086 -3.194483124 CASP10 no 380.596 628.607 0.0229093 0.605459 -1.65164 -2.288583125 CASP12 no 1.77166 1.59801 0.676117 1.10867 1.10867 0.4182423126 CASP14 no 514.94 4427.96 0.0738712 0.116293 -8.59899 -1.794683127 CASP16 no 7.42462 4.38456 0.0285613 1.69336 1.69336 2.20183128 CASP1P1 no 0.0157547 0 0.00065245 ? ? 3.45083129 CASP1P2 no 7.58505 27.0145 0.0726505 0.280777 -3.56154 -1.802373130 CASP1P3 no 0 0.00283088 0.680837 ? ? -0.4117853131 CASP2 no 1326.7 1465.09 0.228603 0.905546 -1.10431 -1.206823132 CASP3 no 947.429 999.279 0.595961 0.948113 -1.05473 -0.5308743133 CASP3P1 no 0 0.00181195 0.768013 ? ? -0.2952843134 CASP4 no 673.119 1193.26 0.000284042 0.564103 -1.77273 -3.67973135 CASP5 no 29.6008 8.85771 7.07E-05 3.34181 3.34181 4.039163136 CASP6 no 701.974 427.632 2.34E-09 1.64154 1.64154 6.190463137 CASP7 no 1215.48 1085.43 0.348146 1.11981 1.11981 0.9398973138 CASP8 no 622.632 712.636 0.210187 0.873703 -1.14455 -1.256173139 CASP8AP2 no 422.461 559.226 0.0341899 0.755438 -1.32373 -2.12913140 CASP9 no 529.766 409.535 0.0431235 1.29358 1.29358 2.03253141 CASQ1 no 13.7262 6.60836 0.000470158 2.0771 2.0771 3.542393142 CASQ2 no 10.8468 6.37726 0.121234 1.70085 1.70085 1.554723143 CASR no 6.63075 3.87781 0.143975 1.70992 1.70992 1.465583144 CASS4 no 18.697 19.5429 0.84545 0.956715 -1.04524 -0.1951233145 CAST no 3628.94 5630.22 0.000498095 0.644546 -1.55148 -3.526393146 CASZ1 no 916.321 1136.31 0.128158 0.806398 -1.24008 -1.526293147 CAT no 1475.87 841.195 1.51E-07 1.7545 1.7545 5.400153148 CATSPER1 no 4.83335 10.736 0.0500034 0.450199 -2.22124 -1.969143149 CATSPER2 no 71.8558 129.811 0.0016148 0.553542 -1.80655 -3.186853150 CATSPER2P1 no 56.2799 42.0314 0.0103539 1.339 1.339 2.582653151 CATSPER3 no 97.1911 113.097 0.280242 0.859364 -1.16365 -1.082043152 CATSPER4 no 1.76994 1.04162 0.0121819 1.69923 1.69923 2.524573153 CATSPERB no 37.3147 88.183 0.326111 0.423151 -2.36322 -0.9838433154 CATSPERD no 5.28687 3.32031 0.0915257 1.59228 1.59228 1.693693155 CATSPERG no 49.5525 70.8807 0.143408 0.699096 -1.43042 -1.467673156 CAV1 no 782.673 2294.88 0.0647127 0.341052 -2.9321 -1.855143157 CAV2 no 602.462 1410.32 0.000447625 0.427182 -2.34093 -3.555953158 CAV3 no 0.284212 0.212487 0.556081 1.33755 1.33755 0.5894383159 CBFA2T2 no 1027.36 1071.89 0.767433 0.958454 -1.04335 -0.2960453160 CBFA2T3 no 111.469 224.535 0.098717 0.496445 -2.01432 -1.657073161 CBFB no 1417.32 1416.88 0.996813 1.00031 1.00031 0.003998393162 CBL no 694.257 608.357 0.174803 1.1412 1.1412 1.360663163 CBLB no 753.392 590.985 0.0945059 1.27481 1.27481 1.678243164 CBLC no 556.681 1151.62 0.00200434 0.48339 -2.06872 -3.121333165 CBLL1 no 598.411 479.524 0.000134506 1.24793 1.24793 3.876243166 CBLN1 no 85.2502 24.4338 0.00297573 3.48903 3.48903 2.998643167 CBLN2 no 25.9169 18.7948 0.810165 1.37894 1.37894 0.2404583168 CBLN3 no 45.8403 42.5331 0.697896 1.07776 1.07776 0.3885943170 CBR1 no 2862.97 2366.77 0.334335 1.20965 1.20965 0.9672223171 CBR3 no 321.192 694.418 0.0173131 0.462534 -2.162 -2.39543172 CBR3-AS1 no 48.1438 78.2686 0.0304991 0.61511 -1.62573 -2.175473173 CBR4 no 1808.2 1430.75 0.00981807 1.26381 1.26381 2.601423174 CBS no 1441.58 740.035 0.000277347 1.94798 1.94798 3.68613175 CBWD1 no 1001.13 812.221 0.0184671 1.23258 1.23258 2.371113176 CBWD2 no 963.651 825.478 0.0189984 1.16739 1.16739 2.360373177 CBWD3 no 259.848 273.311 0.590268 0.950742 -1.05181 -0.5391193178 CBWD4P no 12.3322 19.2025 0.0414946 0.642218 -1.5571 -2.048753179 CBWD5 no 572.196 447.269 0.0139287 1.27931 1.27931 2.475933180 CBWD6 no 390.734 440.298 0.237304 0.887429 -1.12685 -1.184493181 CBWD7 no 10.8021 14.0169 0.241713 0.770649 -1.29761 -1.17343182 CBX1 no 2640.75 1844.49 1.42E-05 1.4317 1.4317 4.426033183 CBX1P1 no 3.40187 2.99553 0.304899 1.13565 1.13565 1.028023184 CBX1P2 no 0 0.00454172 0.677427 ? ? -0.4164483185 CBX1P3 no 1.47093 0.767348 0.488476 1.9169 1.9169 0.6937253186 CBX1P4 no 0.607411 0.658357 0.853448 0.922617 -1.08387 -0.1849053187 CBX1P5 no 0.0426182 0.0118853 0.193062 3.58579 3.58579 1.304973188 CBX2 no 918.214 551.409 0.00079578 1.66521 1.66521 3.394353189 CBX3 no 5786.37 4409.41 2.59E-05 1.31228 1.31228 4.284113190 CBX3P1 no 0.215312 0.0799251 0.00637595 2.69393 2.69393 2.750193191 CBX3P2 no 13.6114 15.01 0.558665 0.906825 -1.10275 -0.5855833192 CBX3P3 no 0 0 ? 1 1 03193 CBX3P4 no 0.229612 0.149378 0.235724 1.53712 1.53712 1.18853194 CBX3P5 no 0 0 ? 1 1 03196 CBX3P7 no 0.0171063 0.0128725 0.803197 1.3289 1.3289 0.2494683197 CBX3P8 no 0.0684252 0 0.00065245 ? ? 3.45083198 CBX3P9 no 1.83248 1.52488 0.394204 1.20172 1.20172 0.853443199 CBX4 no 788.342 755.249 0.660389 1.04382 1.04382 0.4398833200 CBX5 no 3399.09 3249.84 0.672604 1.04592 1.04592 0.4230593201 CBX5P1 no 0 0 ? 1 1 03202 CBX6 no 1702.79 995.024 0.000386978 1.71131 1.71131 3.595933203 CBX7 no 471.439 614.088 0.0846182 0.767706 -1.30258 -1.731143204 CBX8 no 306.872 198.831 0.000106053 1.54338 1.54338 3.937113205 CBY1 no 1034.56 643.411 3.71E-08 1.60794 1.60794 5.674953206 CBY3 no 8.23264 10.0132 0.403007 0.822181 -1.21628 -0.8376373207 CC2D1A no 1432.98 1626.35 0.204317 0.881104 -1.13494 -1.272553208 CC2D1B no 886.459 961.786 0.292331 0.92168 -1.08497 -1.055173209 CC2D2A no 250.918 198.226 0.183699 1.26582 1.26582 1.333013210 CC2D2B no 14.1849 15.1213 0.650109 0.938077 -1.06601 -0.454143211 CCAR1 no 1926.85 1661.18 0.0249947 1.15993 1.15993 2.254593212 CCAT1 no 45.7384 14.1524 0.0451514 3.23185 3.23185 2.012983213 CCBE1 no 233.955 78.6658 0.00889937 2.97403 2.97403 2.635843214 CCBL1 no 185.086 246.193 0.0318357 0.751792 -1.33016 -2.158153215 CCBL2 no 1194.91 1076.73 0.206259 1.10977 1.10977 1.26713216 CCDC101 no 402.707 470.297 0.0962816 0.856282 -1.16784 -1.669223217 CCDC102A no 281.965 173.289 1.13E-05 1.62714 1.62714 4.478033218 CCDC102B no 78.211 46.9512 3.06E-05 1.66579 1.66579 4.243773219 CCDC103 no 119.252 87.1908 0.151244 1.36771 1.36771 1.43943220 CCDC104 no 1004.39 672.561 8.99E-05 1.49339 1.49339 3.979023221 CCDC105 no 0.375168 0.374963 0.999186 1.00055 1.00055 0.001021343222 CCDC106 no 496.658 276.605 3.11E-07 1.79555 1.79555 5.253813223 CCDC107 no 513.456 502.809 0.880058 1.02118 1.02118 0.1510443224 CCDC108 no 21.4597 10.0188 0.0936938 2.14195 2.14195 1.682413225 CCDC109B no 312.413 719.965 1.98E-07 0.433928 -2.30453 -5.344953226 CCDC11 no 72.7431 62.8309 0.546076 1.15776 1.15776 0.6044463227 CCDC110 no 34.0827 14.7515 1.43E-05 2.31045 2.31045 4.42343228 CCDC111 no 276.532 297.742 0.481001 0.928765 -1.0767 -0.7057133229 CCDC112 no 266.15 153.026 2.52E-06 1.73925 1.73925 4.813613230 CCDC113 no 185.796 123.954 0.0561246 1.4989 1.4989 1.918673231 CCDC114 no 107.163 157.616 0.56331 0.679895 -1.47082 -0.5786773232 CCDC115 no 779.348 809.271 0.64032 0.963025 -1.03839 -0.4678013233 CCDC116 no 0.340075 0.284754 0.58231 1.19428 1.19428 0.5507063234 CCDC117 no 982.805 762.683 0.00119959 1.28862 1.28862 3.275273235 CCDC11P1 no 0 0 ? 1 1 03236 CCDC12 no 1036.49 969.408 0.507165 1.0692 1.0692 0.6641823237 CCDC120 no 553.668 743.161 0.0176869 0.745017 -1.34225 -2.387373238 CCDC121 no 80.1065 52.2232 0.000157782 1.53392 1.53392 3.834953239 CCDC122 no 131.611 108.603 0.117955 1.21186 1.21186 1.568633240 CCDC124 no 1619.04 1422.57 0.115587 1.13811 1.13811 1.578873241 CCDC125 no 332.854 302.375 0.380292 1.1008 1.1008 0.8788593242 CCDC126 no 218.181 146.881 0.00034945 1.48543 1.48543 3.623733243 CCDC127 no 420.92 414.602 0.907589 1.01524 1.01524 0.1161943245 CCDC13 no 61.611 23.1219 6.66E-07 2.66462 2.66462 5.096953246 CCDC13-AS1 no 0.0344833 0.0166201 0.350271 2.0748 2.0748 0.9357553247 CCDC130 no 683.139 693.054 0.876318 0.985694 -1.01451 -0.1557923248 CCDC132 no 477.433 469.976 0.831383 1.01587 1.01587 0.2131433249 CCDC134 no 449.2 407.529 0.366444 1.10225 1.10225 0.904743250 CCDC135 no 24.025 21.7103 0.910575 1.10662 1.10662 0.1124233251 CCDC136 no 83.1493 64.8656 0.287552 1.28187 1.28187 1.06573252 CCDC137 no 1084.19 964.622 0.107897 1.12395 1.12395 1.61333253 CCDC137P no 0 0.0140059 0.510926 ? ? -0.6583073254 CCDC138 no 233.069 177.189 0.00121853 1.31537 1.31537 3.270663255 CCDC14 no 1300.16 1497.35 0.341501 0.868308 -1.15166 -0.9529563256 CCDC140 no 0.854565 0.24468 0.0542474 3.49258 3.49258 1.933643257 CCDC141 no 5.01333 7.98613 0.179516 0.627755 -1.59298 -1.345893258 CCDC142 no 166.361 196.271 0.119766 0.84761 -1.17979 -1.560913259 CCDC144A no 41.1529 29.9623 0.149846 1.37349 1.37349 1.444363260 CCDC144B no 37.7394 33.5593 0.661756 1.12456 1.12456 0.4379933261 CCDC144CP no 4.91237 5.47168 0.674633 0.897781 -1.11386 -0.4202753262 CCDC144NL no 10.9367 9.55785 0.791294 1.14426 1.14426 0.2649043263 CCDC146 no 625.749 683.063 0.599998 0.916092 -1.09159 -0.5250493264 CCDC147 no 23.579 28.7045 0.489279 0.82144 -1.21737 -0.6924443265 CCDC147-AS1 no 0.401127 0.579464 0.361093 0.692238 -1.44459 -0.9149043266 CCDC148 no 51.2793 26.6113 0.000246588 1.92697 1.92697 3.717513267 CCDC148-AS1 no 2.88871 1.71128 0.084289 1.68804 1.68804 1.732983268 CCDC149 no 290.862 257.958 0.474625 1.12755 1.12755 0.7160193269 CCDC15 no 63.7561 110.935 0.000324473 0.574717 -1.73999 -3.643843270 CCDC150 no 122.384 126.521 0.796109 0.967306 -1.0338 -0.2586533271 CCDC151 no 43.725 25.2754 0.103968 1.72994 1.72994 1.631653272 CCDC152 no 53.7659 165.143 0.0297756 0.325572 -3.07151 -2.185133273 CCDC153 no 44.0889 60.1781 0.393349 0.73264 -1.36493 -0.8549873274 CCDC154 no 48.6617 38.4763 0.536517 1.26472 1.26472 0.6189143275 CCDC155 no 21.2298 36.063 0.312439 0.588685 -1.6987 -1.012093276 CCDC157 no 162.172 103.22 0.000492808 1.57113 1.57113 3.529353277 CCDC158 no 17.4469 16.047 0.71931 1.08724 1.08724 0.3597733278 CCDC159 no 224.34 201.326 0.455877 1.11432 1.11432 0.7467753279 CCDC160 no 69.2975 38.8648 0.144567 1.78304 1.78304 1.463413280 CCDC162P no 7.85468 3.01047 0.000242057 2.60912 2.60912 3.722453281 CCDC163P no 57.1108 53.0264 0.606072 1.07702 1.07702 0.516323282 CCDC166 no 0.57941 0.938287 0.392711 0.617519 -1.61938 -0.856143283 CCDC167 no 578.875 385.855 0.000346391 1.50024 1.50024 3.626123284 CCDC168 no 3.81748 4.35376 0.609177 0.876823 -1.14048 -0.5118723285 CCDC169 no 8.94934 4.47213 0.0048513 2.00114 2.00114 2.841233286 CCDC169-SOHLH2 no 71.5217 18.7534 5.73E-06 3.8138 3.8138 4.632183287 CCDC17 no 43.9381 35.8857 0.601702 1.22439 1.22439 0.5225963288 CCDC170 no 121.666 51.2356 0.0417748 2.37463 2.37463 2.045913289 CCDC171 no 86.331 112.806 0.532285 0.765307 -1.30667 -0.6253613290 CCDC172 no 0.126997 0.097795 0.561318 1.29861 1.29861 0.5816353291 CCDC173 no 131.212 146.765 0.806682 0.894032 -1.11853 -0.244963292 CCDC174 no 457.41 381.274 0.00382571 1.19969 1.19969 2.918553293 CCDC175 no 16.6993 4.498 4.01E-07 3.71261 3.71261 5.20213294 CCDC176 no 271.892 223.605 0.136335 1.21595 1.21595 1.494223295 CCDC177 no 30.15 37.6643 0.486883 0.800494 -1.24923 -0.6962733296 CCDC178 no 7.48013 8.8769 0.817779 0.842651 -1.18673 -0.2306363297 CCDC179 no 0.351018 0.462848 0.475108 0.758388 -1.31859 -0.7152353298 CCDC18 no 363.627 405.028 0.26591 0.897782 -1.11386 -1.114943299 CCDC180 no 85.2726 80.5763 0.803433 1.05828 1.05828 0.2491623300 CCDC181 no 26.3752 33.3656 0.113524 0.790493 -1.26503 -1.587923301 CCDC19 no 121.584 135.954 0.836702 0.894303 -1.11819 -0.2063223302 CCDC22 no 597.118 598.13 0.986185 0.998308 -1.00169 -0.01733243303 CCDC23 no 405.968 340.057 0.0563875 1.19382 1.19382 1.916613304 CCDC24 no 364.446 317.573 0.30877 1.1476 1.1476 1.019813305 CCDC25 no 829.052 789.14 0.527773 1.05058 1.05058 0.6322623306 CCDC27 no 12.1559 4.18434 3.46E-05 2.9051 2.9051 4.214533307 CCDC28A no 384.76 447.034 0.188572 0.860696 -1.16185 -1.318293308 CCDC28B no 211.36 201.827 0.855046 1.04723 1.04723 0.1828673309 CCDC3 no 995.223 344.126 0.00350016 2.89203 2.89203 2.947093310 CCDC30 no 68.371 45.3716 0.0316119 1.50691 1.50691 2.1613311 CCDC33 no 34.8875 23.6465 0.483278 1.47538 1.47538 0.7020513312 CCDC34 no 386.278 516.683 0.119108 0.747611 -1.33759 -1.56373313 CCDC34P1 no 0.0470783 0.00660536 0.0271931 7.12729 7.12729 2.221343314 CCDC36 no 10.4251 6.69715 0.00350772 1.55664 1.55664 2.94643315 CCDC37 no 28.3313 53.747 0.549693 0.527123 -1.89709 -0.5990063316 CCDC38 no 16.939 13.9671 0.617677 1.21278 1.21278 0.4997473317 CCDC39 no 6.8305 9.12176 0.780506 0.748814 -1.33545 -0.278953318 CCDC40 no 296.561 182.609 0.0974899 1.62402 1.62402 1.663163319 CCDC41 no 326.974 322.007 0.864746 1.01543 1.01543 0.1705053320 CCDC41-AS1 no 12.5569 12.728 0.935244 0.98656 -1.01362 -0.08132763321 CCDC42 no 2.6642 1.64153 0.101442 1.623 1.623 1.643743322 CCDC42B no 73.2156 150.614 0.365883 0.486115 -2.05713 -0.9058013323 CCDC43 no 599.061 553.969 0.241843 1.0814 1.0814 1.173083324 CCDC47 no 2577.15 2337.86 0.376868 1.10235 1.10235 0.8852043325 CCDC50 no 1096.94 1434.98 0.0162496 0.764432 -1.30816 -2.419083326 CCDC51 no 472.533 437.93 0.445674 1.07901 1.07901 0.7638123327 CCDC53 no 489.658 490.834 0.975501 0.997604 -1.0024 -0.03073923328 CCDC54 no 0.325717 0.3983 0.602588 0.817767 -1.22284 -0.5213223329 CCDC57 no 714.904 649.591 0.485429 1.10054 1.10054 0.69863330 CCDC58 no 563.851 498.462 0.214266 1.13118 1.13118 1.244983331 CCDC58P1 no 0.204226 0.184607 0.802362 1.10628 1.10628 0.2505483332 CCDC58P2 no 0 0.0199959 0.482575 ? ? -0.7031813333 CCDC58P3 no 0.446409 0.210741 0.000863258 2.11828 2.11828 3.3713334 CCDC58P5 no 0 0 ? 1 1 03335 CCDC59 no 587.702 594.838 0.868915 0.988004 -1.01214 -0.16523336 CCDC6 no 2700.99 2411.86 0.284219 1.11988 1.11988 1.073113337 CCDC60 no 42.1422 198.93 0.052637 0.211845 -4.72044 -1.946823338 CCDC61 no 285.139 326.187 0.193947 0.874158 -1.14396 -1.302373339 CCDC62 no 24.8584 14.3496 5.08E-06 1.73234 1.73234 4.659263340 CCDC63 no 3.26875 4.32805 0.512328 0.755249 -1.32407 -0.6561223341 CCDC64 no 952.053 851.642 0.519081 1.1179 1.1179 0.6456453342 CCDC64B no 675.136 1017.57 0.0397578 0.663481 -1.5072 -2.066683343 CCDC65 no 292.225 228.272 0.0217064 1.28016 1.28016 2.309443344 CCDC66 no 426.443 394.273 0.37538 1.08159 1.08159 0.8879713345 CCDC67 no 23.847 14.3693 0.18996 1.65958 1.65958 1.314153346 CCDC68 no 94.4495 130.491 0.28322 0.723803 -1.38159 -1.075353347 CCDC69 no 224.948 411.142 0.00831818 0.547129 -1.82772 -2.659313348 CCDC7 no 2.22939 1.73237 0.11495 1.2869 1.2869 1.581653349 CCDC70 no 0.408808 0.269309 0.394765 1.51799 1.51799 0.8524263350 CCDC71 no 482.941 413.575 0.0454358 1.16772 1.16772 2.01033351 CCDC71L no 586.607 497.094 0.324637 1.18007 1.18007 0.986853352 CCDC73 no 106.814 86.6102 0.160944 1.23327 1.23327 1.405933353 CCDC74A no 375.103 251.775 0.178887 1.48983 1.48983 1.347843354 CCDC74B no 239.14 70.6016 3.83E-05 3.38718 3.38718 4.189973355 CCDC74B-AS1 no 4.22211 5.84796 0.855867 0.72198 -1.38508 -0.1818193356 CCDC75P1 no 0 0.00329503 0.676801 ? ? -0.4173053357 CCDC77 no 306.623 314.606 0.762837 0.974628 -1.02603 -0.3020753358 CCDC78 no 69.1926 63.7935 0.821983 1.08464 1.08464 0.2252233359 CCDC79 no 3.07942 2.06831 0.0225532 1.48886 1.48886 2.294653360 CCDC8 no 593.644 242.225 4.76E-05 2.45079 2.45079 4.136933361 CCDC80 no 884.672 1615.08 0.152591 0.547756 -1.82563 -1.434663362 CCDC81 no 20.6723 15.0509 0.325003 1.37349 1.37349 0.9861033363 CCDC82 no 480.848 461.002 0.77386 1.04305 1.04305 0.287633364 CCDC83 no 2.17278 1.45145 0.0259068 1.49697 1.49697 2.24053365 CCDC84 no 433.792 376.103 0.196995 1.15339 1.15339 1.293493366 CCDC85A no 32.3243 21.174 0.287276 1.52661 1.52661 1.066313367 CCDC85B no 427.3 345.152 0.261398 1.238 1.238 1.125553368 CCDC85C no 1028.5 1508.53 0.0139722 0.68179 -1.46673 -2.474793369 CCDC86 no 1049.75 607.664 1.57E-08 1.72752 1.72752 5.839073370 CCDC87 no 18.3096 17.8605 0.921974 1.02514 1.02514 0.09804353371 CCDC88A no 604.927 665.271 0.500852 0.909294 -1.09975 -0.6740943372 CCDC88B no 357.787 721.391 0.01001 0.495968 -2.01626 -2.594593373 CCDC88C no 1160.57 1171.69 0.94648 0.990506 -1.00959 -0.06719353374 CCDC89 no 12.9797 4.26503 6.33E-06 3.04329 3.04329 4.60993375 CCDC9 no 618.998 785.766 0.00938789 0.787763 -1.26942 -2.617163376 CCDC90AP1 no 0 0.00198231 0.768013 ? ? -0.2952843377 CCDC90B no 643.234 595.747 0.438937 1.07971 1.07971 0.7751863378 CCDC91 no 419.087 467.683 0.251469 0.896092 -1.11596 -1.149363379 CCDC92 no 885.426 576.963 5.78E-07 1.53463 1.53463 5.126563380 CCDC93 no 776.494 820.137 0.537723 0.946786 -1.0562 -0.6170823381 CCDC94 no 421.879 467.593 0.308592 0.902237 -1.10836 -1.020193382 CCDC96 no 33.5614 39.9753 0.395621 0.839555 -1.19111 -0.8508823383 CCDC97 no 926.249 1047.32 0.219063 0.884402 -1.13071 -1.232013384 CCER1 no 0.0129226 0.00735408 0.639081 1.7572 1.7572 0.4695373385 CCHCR1 no 1093.68 1432.91 0.0486084 0.763261 -1.31017 -1.981363386 CCIN no 3.29596 3.29191 0.997078 1.00123 1.00123 0.00366633387 CCK no 2.38312 23.8802 0.135022 0.0997952 -10.0205 -1.499273390 CCL1 no 0.618413 0.749034 0.703987 0.825614 -1.21122 -0.3803643391 CCL11 no 21.5345 40.1045 0.157141 0.536959 -1.86234 -1.418873392 CCL13 no 20.1919 38.9737 0.236882 0.518091 -1.93016 -1.185563393 CCL14 no 22.5045 60.2848 0.194638 0.373304 -2.67878 -1.300353394 CCL15 no 42.5364 40.0386 0.960254 1.06239 1.06239 0.04988263395 CCL15-CCL14 no 1.06599 1.31647 0.890704 0.809735 -1.23497 -0.1375493396 CCL16 no 0.393266 0.887745 0.143762 0.442994 -2.25737 -1.466373398 CCL18 no 437.279 350.742 0.687287 1.24673 1.24673 0.4029913399 CCL19 no 49.1025 486.623 0.594515 0.100905 -9.91035 -0.5329653400 CCL2 no 317.274 547.446 0.421777 0.579553 -1.72547 -0.804623401 CCL20 no 676.383 575.844 0.725879 1.17459 1.17459 0.3509933402 CCL21 no 99.0333 232.326 0.197232 0.426268 -2.34594 -1.29283403 CCL22 no 37.5436 164.512 0.0381794 0.228212 -4.38189 -2.083583404 CCL23 no 2.98149 6.56461 0.570308 0.454177 -2.20179 -0.5683233406 CCL25 no 13.3063 25.9746 0.855398 0.512281 -1.95205 -0.1824183407 CCL26 no 9.47265 13.6178 0.520666 0.695609 -1.43759 -0.6431973408 CCL27 no 3.43134 4.881 0.792017 0.703 -1.42247 -0.2639653409 CCL28 no 279.6 411.744 0.235904 0.679064 -1.47261 -1.188043410 CCL3 no 143.377 183.255 0.327113 0.782392 -1.27813 -0.9818033411 CCL3L1 no 28.7088 31.4742 0.741512 0.912138 -1.09633 -0.3302053412 CCL3L3 no 4.79395 6.50498 0.471647 0.736965 -1.35692 -0.7208583413 CCL3P1 no 1.88915 5.10366 0.181409 0.370157 -2.70156 -1.340033414 CCL4 no 141.793 224.707 0.157951 0.631014 -1.58475 -1.416093415 CCL4L1 no 17.0066 16.227 0.769095 1.04804 1.04804 0.2938673416 CCL4L2 no 77.1547 95.5046 0.512836 0.807864 -1.23783 -0.6553313417 CCL5 no 405.047 1639.11 0.0192791 0.247113 -4.04673 -2.354813418 CCL7 no 8.77019 8.5012 0.933323 1.03164 1.03164 0.08374553419 CCL8 no 50.8251 63.6327 0.681672 0.798727 -1.25199 -0.4106453420 CCM2 no 1157.97 1345.41 0.201728 0.860679 -1.16187 -1.279893421 CCM2L no 66.3637 39.7875 0.00563264 1.66795 1.66795 2.791773423 CCNA2 no 858.082 975.94 0.306055 0.879236 -1.13735 -1.025563424 CCNB1 no 2400.82 2232.17 0.403418 1.07555 1.07555 0.8369053425 CCNB1IP1 no 1024.89 623.972 1.99E-07 1.64252 1.64252 5.344653426 CCNB1IP1P1 no 0 0 ? 1 1 03427 CCNB1IP1P2 no 0.0171063 0.00414361 0.184456 4.12835 4.12835 1.330713428 CCNB1IP1P3 no 0.0364643 0.00931436 0.110125 3.91485 3.91485 1.603133429 CCNB2 no 942.689 1236.34 0.00719834 0.762483 -1.3115 -2.709023430 CCNB3 no 14.9349 15.2863 0.92601 0.977008 -1.02353 -0.09295673431 CCNC no 1417.59 1082.91 0.000611529 1.30905 1.30905 3.469063432 CCND1 no 5454.77 1650 4.49E-09 3.30592 3.30592 6.071283433 CCND2 no 1457.46 865.991 0.0990599 1.683 1.683 1.655373434 CCND2P no 0 0.473501 0.101302 ? ? -1.644423435 CCND3 no 1193.75 1340.59 0.22377 0.890461 -1.12301 -1.219483436 CCND3P no 0.458642 0.312797 0.122075 1.46626 1.46626 1.55123437 CCNDBP1 no 552.225 728.216 0.00647075 0.758326 -1.31869 -2.745213438 CCNE1 no 553.074 366.698 0.000324766 1.50825 1.50825 3.643593439 CCNE2 no 372.97 594.849 0.00228454 0.626998 -1.5949 -3.081133440 CCNF no 816.532 954.569 0.174792 0.855393 -1.16905 -1.36073441 CCNG1 no 1704.46 1056.86 1.78E-07 1.61275 1.61275 5.366873442 CCNG1P1 no 0 0 ? 1 1 03443 CCNG2 no 923.527 836.737 0.504835 1.10372 1.10372 0.6678323444 CCNH no 476.645 469.714 0.832721 1.01476 1.01476 0.2114263445 CCNI no 5758.33 4945.76 0.0531061 1.1643 1.1643 1.942953446 CCNI2 no 30.059 34.5688 0.697107 0.869542 -1.15003 -0.3896623447 CCNJ no 359.682 160.946 2.88E-18 2.23481 2.23481 9.406383448 CCNJL no 194.159 251.879 0.450234 0.770843 -1.29728 -0.7561713449 CCNJP1 no 0.0344833 0.00728575 0.0767276 4.73298 4.73298 1.777093450 CCNJP2 no 0 0 ? 1 1 03451 CCNK no 1252.24 1366.85 0.298052 0.916148 -1.09153 -1.042723452 CCNL1 no 1750.67 2566.62 0.00260471 0.682089 -1.46608 -3.040423453 CCNL2 no 2958.85 2992.38 0.912779 0.988796 -1.01133 -0.1096423454 CCNO no 246.978 214.295 0.731989 1.15251 1.15251 0.342853455 CCNT1 no 731.707 730.151 0.975273 1.00213 1.00213 0.03102563456 CCNT2 no 843.919 777.013 0.33128 1.08611 1.08611 0.9733643457 CCNT2P1 no 0.212238 0.121006 0.226648 1.75395 1.75395 1.211923458 CCNY no 1729.95 1443.88 0.00476962 1.19813 1.19813 2.846813459 CCNYL1 no 463.985 591.937 0.101694 0.783842 -1.27577 -1.642533461 CCP110 no 552.123 601.152 0.545343 0.918442 -1.0888 -0.6055523462 CCPG1 no 762.98 927.333 0.237441 0.822768 -1.21541 -1.184143463 CCR1 no 128.345 128.512 0.99556 0.998694 -1.00131 -0.005570373464 CCR10 no 21.9617 37.5351 0.36351 0.585096 -1.70912 -0.9103023465 CCR12P no 0.0180043 0.0742957 0.194268 0.242333 -4.12656 -1.301433466 CCR2 no 52.7166 44.7489 0.624254 1.17805 1.17805 0.4904173467 CCR3 no 33.0196 23.2984 0.597065 1.41725 1.41725 0.529283468 CCR4 no 7.1098 16.9435 0.117912 0.419619 -2.38312 -1.568813469 CCR5 no 109.489 203.416 0.106965 0.538254 -1.85786 -1.617613470 CCR6 no 25.637 46.3961 0.0317311 0.552569 -1.80973 -2.159483471 CCR7 no 32.3851 121.791 0.0107493 0.265907 -3.76072 -2.569353472 CCR8 no 3.49103 7.74378 0.0472851 0.450817 -2.2182 -1.993233473 CCR9 no 0.0810229 0.0328545 0.210428 2.46611 2.46611 1.25553474 CCRL1P1 no 0.0203008 0.202634 0.0288629 0.100184 -9.98159 -2.19763475 CCRL2 no 55.8283 52.8364 0.841372 1.05663 1.05663 0.200343476 CCRN4L no 128.439 163.901 0.10655 0.783638 -1.2761 -1.619533477 CCS no 805.58 713.493 0.331897 1.12907 1.12907 0.9721223478 CCSAP no 380.181 452.81 0.116463 0.839603 -1.19104 -1.575063479 CCSER1 no 645.027 333.18 0.0021908 1.93597 1.93597 3.094053480 CCSER2 no 741.981 676.502 0.251309 1.09679 1.09679 1.149753481 CCT2 no 4356.49 3191.38 0.00177537 1.36508 1.36508 3.158243482 CCT3 no 8631.85 6829.14 0.000874763 1.26397 1.26397 3.367193483 CCT3P1 no 0 0.00167828 0.768013 ? ? -0.2952843484 CCT4 no 5548.38 4165.69 0.000119846 1.33192 1.33192 3.905893485 CCT4P1 no 0.089924 0.0170898 0.0347233 5.26185 5.26185 2.122753486 CCT4P2 no 0.312597 0.135767 0.025443 2.30246 2.30246 2.247613487 CCT5 no 6922.85 7065.63 0.870381 0.979793 -1.02062 -0.1633363488 CCT5-2P no 0 0 ? 1 1 03489 CCT5P1 no 0.156866 0.262774 0.25212 0.596963 -1.67515 -1.147783490 CCT5P2 no 0.0497252 0.0424005 0.813729 1.17275 1.17275 0.2358583491 CCT6A no 6167.31 4433.93 9.75E-06 1.39093 1.39093 4.512073492 CCT6B no 435.597 400.336 0.199261 1.08808 1.08808 1.286953493 CCT6P1 no 2.95629 2.78454 0.647429 1.06168 1.06168 0.4578713494 CCT6P2 no 0.32065 0.138408 0.00842939 2.31671 2.31671 2.65473495 CCT6P3 no 168.445 144.88 0.178757 1.16265 1.16265 1.348253496 CCT6P4 no 0 0.00734518 0.634299 ? ? -0.4762493497 CCT6P5 no 0 0.0013228 0.768013 ? ? -0.2952843498 CCT7 no 7044.28 5179.05 6.05E-06 1.36015 1.36015 4.619963499 CCT7P1 no 0 0.15487 0.0148275 ? ? -2.452983500 CCT7P2 no 0.244905 0.225923 0.9521 1.08402 1.08402 0.06012813501 CCT8 no 5019.58 3723.54 1.09E-05 1.34807 1.34807 4.487383502 CCT8L1P no 0.837245 0.306939 4.23E-05 2.72773 2.72773 4.165673503 CCT8L2 no 0.0171063 0.00871975 0.530161 1.96179 1.96179 0.6286073504 CCT8P1 no 0.121596 0.049957 0.0416895 2.43401 2.43401 2.046783505 CCZ1 no 1384.66 1173.1 0.0213545 1.18034 1.18034 2.315733506 CCZ1B no 1031.64 863 0.0683802 1.19542 1.19542 1.830133507 CD101 no 80.1107 142.419 0.00648226 0.5625 -1.77778 -2.74463509 CD14 no 842.68 1087.24 0.251453 0.775062 -1.29022 -1.14943510 CD151 no 5733.88 4975.13 0.119362 1.15251 1.15251 1.562633511 CD160 no 33.8662 47.1056 0.116659 0.718943 -1.39093 -1.574213512 CD163 no 710.153 478.725 0.205803 1.48343 1.48343 1.268383513 CD163L1 no 71.2681 49.6615 0.12915 1.43508 1.43508 1.522313514 CD164 no 5978.41 5183.58 0.215803 1.15334 1.15334 1.24083515 CD164L2 no 31.4353 75.6542 0.0553706 0.415514 -2.40666 -1.924633516 CD177 no 15.0908 243.177 0.372175 0.0620567 -16.1143 -0.8939573517 CD177P1 no 0.295268 1.46743 0.283487 0.201214 -4.96983 -1.074753518 CD180 no 54.6429 72.3449 0.695706 0.755311 -1.32396 -0.3915593519 CD19 no 17.9819 34.8163 0.162933 0.51648 -1.93618 -1.399253520 CD1A no 16.0249 118.943 0.0542498 0.134728 -7.42236 -1.933623521 CD1B no 2.48305 8.96333 0.117106 0.277023 -3.6098 -1.572283522 CD1C no 10.6452 40.4561 0.00833891 0.263131 -3.80039 -2.658443523 CD1D no 40.4295 26.9202 0.124758 1.50183 1.50183 1.540093524 CD1E no 8.9664 42.0298 0.0165341 0.213334 -4.68748 -2.412613525 CD2 no 160.506 412.319 0.0199518 0.389275 -2.56888 -2.341753526 CD200 no 425.461 159.032 0.0173228 2.67532 2.67532 2.395183527 CD200R1 no 22.5807 46.126 0.186996 0.489544 -2.04272 -1.323023528 CD200R1L no 12.8357 10.8655 0.708886 1.18133 1.18133 0.3737633529 CD207 no 23.3915 133.604 0.071492 0.175081 -5.71165 -1.809763530 CD209 no 40.8342 60.6889 0.408885 0.672846 -1.48623 -0.8271993531 CD22 no 30.0431 42.0414 0.329858 0.714607 -1.39937 -0.9762363532 CD226 no 27.01 24.7641 0.69752 1.09069 1.09069 0.3891033533 CD244 no 24.1207 41.1096 0.134472 0.58674 -1.70433 -1.501393534 CD247 no 90.1551 236.81 0.00432889 0.380707 -2.62669 -2.878523535 CD248 no 1059.11 331.495 2.29E-06 3.19494 3.19494 4.83483536 CD24P1 no 0.301796 0.291115 0.905923 1.03669 1.03669 0.1182993537 CD24P2 no 295.912 280.487 0.929898 1.055 1.055 0.08805913538 CD24P3 no 0 0.00539867 0.634399 ? ? -0.4761073539 CD24P4 no 17003.8 18598.2 0.898831 0.914273 -1.09377 -0.1272633540 CD27 no 202.548 297.606 0.0798872 0.680592 -1.46931 -1.758243541 CD27-AS1 no 151.388 80.7062 0.000200995 1.87579 1.87579 3.771643542 CD274 no 109.703 436.827 0.0411995 0.251135 -3.98192 -2.051753543 CD276 no 2257.06 1930 0.286196 1.16946 1.16946 1.068713544 CD28 no 25.2075 41.1812 0.155562 0.612111 -1.63369 -1.424313545 CD2AP no 1569.5 1663.94 0.651536 0.943244 -1.06017 -0.4521553546 CD2BP2 no 1971.48 1625.48 0.00136703 1.21286 1.21286 3.236643547 CD300A no 125.213 164.296 0.157501 0.762116 -1.31214 -1.417633548 CD300C no 18.1345 23.7714 0.246438 0.762871 -1.31084 -1.161673549 CD300E no 35.0968 37.7303 0.82454 0.930202 -1.07504 -0.2219353550 CD300LB no 6.75651 9.35437 0.196492 0.722284 -1.3845 -1.294953551 CD300LD no 0.911211 5.03336 0.742334 0.181035 -5.52381 -0.3291163552 CD300LF no 29.2157 31.7165 0.821185 0.921151 -1.0856 -0.2262513553 CD300LG no 1.31586 3.70852 0.492944 0.354821 -2.81833 -0.6866083554 CD302 no 272.227 153.922 0.000247626 1.7686 1.7686 3.716393555 CD320 no 1328.2 631.746 5.13E-11 2.10242 2.10242 6.857333556 CD33 no 28.8513 36.087 0.393133 0.799493 -1.25079 -0.8553773557 CD34 no 617.84 381.97 0.00373677 1.61751 1.61751 2.926123558 CD36 no 334.616 420.513 0.835353 0.795735 -1.2567 -0.208053559 CD37 no 160.728 285.662 0.0568633 0.562651 -1.7773 -1.91293560 CD38 no 95.1481 122.031 0.434167 0.779702 -1.28254 -0.78333561 CD3D no 73.6562 228.783 0.0232798 0.321948 -3.10609 -2.282353562 CD3E no 164.745 458.344 0.0176366 0.359434 -2.78215 -2.388443563 CD3EAP no 63.6835 80.7137 0.226083 0.789004 -1.26742 -1.21343564 CD3G no 21.8335 43.0901 0.0957564 0.506695 -1.97357 -1.671883565 CD4 no 562.032 792.134 0.176262 0.709517 -1.40941 -1.356063566 CD40 no 414.39 689.421 0.0271902 0.60107 -1.6637 -2.221383567 CD40LG no 8.66162 14.5055 0.174772 0.597128 -1.67468 -1.360763568 CD44 no 6485.87 11987.2 0.149653 0.541067 -1.8482 -1.445053569 CD46 no 5261.94 5291.28 0.9591 0.994455 -1.00558 -0.0513333570 CD46P1 no 0.0210637 0.0172811 0.858538 1.21889 1.21889 0.1784143571 CD47 no 2618.66 3841.65 0.00386347 0.68165 -1.46703 -2.915383572 CD48 no 136.696 282.406 0.042659 0.484041 -2.06594 -2.037083573 CD5 no 128.973 129.459 0.988952 0.996249 -1.00377 -0.01386053574 CD52 no 204.042 415.645 0.0421242 0.490904 -2.03706 -2.04243575 CD53 no 427.336 633.146 0.186524 0.674941 -1.48161 -1.324443576 CD55 no 3041.93 5330.39 0.269162 0.570678 -1.7523 -1.107373577 CD58 no 516.212 796.129 0.00069263 0.648402 -1.54225 -3.433893578 CD59 no 4466.15 7618.01 0.00343563 0.586263 -1.70572 -2.953043579 CD5L no 0.489953 1.1282 0.580793 0.434278 -2.30267 -0.5529233580 CD6 no 146.045 273.249 0.0425287 0.534475 -1.87099 -2.038373581 CD63 no 11726.1 10265.9 0.191049 1.14223 1.14223 1.310913582 CD68 no 1809.72 2518.07 0.0843298 0.718694 -1.39141 -1.732753583 CD69 no 102.172 131.473 0.48392 0.777131 -1.28679 -0.7010193584 CD7 no 119.008 376.047 0.00604642 0.316471 -3.15985 -2.768053585 CD70 no 46.2497 216.202 0.303017 0.213919 -4.67467 -1.032043586 CD72 no 65.9157 71.0891 0.801411 0.927226 -1.07849 -0.2517793587 CD74 no 17090.2 52542.9 0.00177965 0.325262 -3.07444 -3.157513588 CD79A no 94.4044 166.008 0.213935 0.568673 -1.75848 -1.245883589 CD79B no 41.4645 62.3373 0.284842 0.665164 -1.50339 -1.071723590 CD80 no 19.2796 31.4836 0.0725028 0.61237 -1.633 -1.80333591 CD81 no 10222.8 5800.55 3.88E-10 1.76238 1.76238 6.509683592 CD82 no 1512.53 3266.28 0.000163637 0.463076 -2.15947 -3.825473593 CD83 no 341.463 399.117 0.682779 0.855546 -1.16884 -0.4091353594 CD83P1 no 0.137855 0.0461176 0.0120065 2.98921 2.98921 2.52983595 CD84 no 142.433 166.591 0.641682 0.854991 -1.1696 -0.4658963596 CD86 no 106.337 186.941 0.0293287 0.568828 -1.758 -2.19123597 CD8A no 370.126 431.025 0.70637 0.85871 -1.16454 -0.3771523598 CD8B no 843.717 224.653 4.14E-05 3.75565 3.75565 4.171243599 CD8BP no 66.7253 41.6233 0.59047 1.60308 1.60308 0.5388253600 CD9 no 13675.9 11671.3 0.260188 1.17176 1.17176 1.128423601 CD93 no 690.107 459.93 0.0127764 1.50046 1.50046 2.507363602 CD96 no 140.011 342.565 0.00403929 0.408714 -2.4467 -2.901013603 CD97 no 1078.45 1405.38 0.215228 0.767371 -1.30315 -1.242363604 CD99_1 no 1442.54 1967.48 0.0342355 0.733193 -1.3639 -2.128553605 CD99_2 no 1438.74 1962.07 0.0342285 0.733274 -1.36375 -2.128633606 CD99L2 no 717.084 715.751 0.991474 1.00186 1.00186 0.01069613607 CD99P1_1 no 43.0755 53.0666 0.178037 0.811725 -1.23195 -1.350493608 CD99P1_2 no 40.8067 53.037 0.0868888 0.7694 -1.29971 -1.718563609 CDA no 231.765 293.647 0.716828 0.789266 -1.267 -0.3630983610 CDADC1 no 217.815 171.666 0.0013674 1.26883 1.26883 3.236553611 CDAN1 no 497.589 445.743 0.163525 1.11631 1.11631 1.397283612 CDC123 no 1707.07 1308.81 4.10E-06 1.30429 1.30429 4.706823613 CDC14A no 249.748 155.028 0.00229047 1.61098 1.61098 3.080333614 CDC14B no 468.967 350.148 0.0335697 1.33934 1.33934 2.136583615 CDC14BL no 0.0315093 0.0227031 0.698976 1.38789 1.38789 0.3871333616 CDC14C no 0.101051 0.0428122 0.0961389 2.36034 2.36034 1.669943617 CDC16 no 1500.87 1350.93 0.156261 1.11099 1.11099 1.421893618 CDC20 no 2078.86 2224.13 0.482731 0.934683 -1.06988 -0.7029293619 CDC20B no 226.542 133.84 0.0167816 1.69262 1.69262 2.407063620 CDC20P1 no 3.26444 6.5298 0.055578 0.499929 -2.00028 -1.922993621 CDC23 no 821.952 854.197 0.583613 0.962251 -1.03923 -0.5488033622 CDC25A no 492.717 376.496 0.0034264 1.30869 1.30869 2.95393623 CDC25B no 2506.92 3366.71 0.053043 0.744618 -1.34297 -1.943473624 CDC25C no 261.367 283.754 0.483193 0.921105 -1.08565 -0.7021873625 CDC26 no 547.476 496.648 0.303965 1.10234 1.10234 1.030013626 CDC26P1 no 1.26762 1.20416 0.884848 1.0527 1.0527 0.1449693627 CDC27 no 1208.19 1130.73 0.324884 1.0685 1.0685 0.9863463630 CDC34 no 1634.49 986.928 1.68E-10 1.65614 1.65614 6.654683631 CDC37 no 3848.88 3775.32 0.802596 1.01949 1.01949 0.2502453632 CDC37L1 no 365.239 412.996 0.456948 0.884366 -1.13075 -0.7449983633 CDC37P1 no 0 0.0143371 0.352264 ? ? -0.9318843634 CDC37P2 no 0.698357 1.24936 0.158564 0.55897 -1.789 -1.413993635 CDC40 no 589.853 495.899 0.0262624 1.18946 1.18946 2.235123636 CDC42 no 5642.41 6513.88 0.0366943 0.866212 -1.15445 -2.100023637 CDC42BPA no 970.14 1073.47 0.50329 0.903738 -1.10652 -0.6702593638 CDC42BPB no 2944.32 3095.79 0.618311 0.951072 -1.05145 -0.4988463639 CDC42BPG no 714.623 1119.5 0.00399349 0.638341 -1.56656 -2.90473640 CDC42EP1 no 1853.6 2624.14 0.0517443 0.706362 -1.4157 -1.954283641 CDC42EP2 no 271.541 338.254 0.245188 0.802772 -1.24568 -1.164763642 CDC42EP3 no 577.734 798.418 0.215739 0.723598 -1.38198 -1.240973643 CDC42EP4 no 1688.8 2998.1 0.00551929 0.563292 -1.77528 -2.798543644 CDC42EP5 no 361.124 272.071 0.304049 1.32732 1.32732 1.029833645 CDC42P1 no 0 0 ? 1 1 03646 CDC42P2 no 0.25759 0.233834 0.828982 1.10159 1.10159 0.2162263647 CDC42P3 no 0.179642 0.184974 0.929455 0.971175 -1.02968 -0.08861723648 CDC42P4 no 2.61404 2.32416 0.43647 1.12472 1.12472 0.7793763649 CDC42P5 no 0 0.0113239 0.466154 ? ? -0.7298293650 CDC42P6 no 8.30581 9.17073 0.587889 0.905687 -1.10413 -0.5425753651 CDC42SE1 no 2759.18 3761.03 0.00510652 0.733624 -1.3631 -2.824323652 CDC42SE2 no 1352.68 1121.1 0.0500057 1.20657 1.20657 1.969123653 CDC45 no 693.726 791.552 0.214095 0.876412 -1.14102 -1.245443654 CDC5L no 1424.5 1254.3 0.119294 1.13569 1.13569 1.562913655 CDC6 no 936.2 1065.7 0.20202 0.87848 -1.13833 -1.279063656 CDC7 no 504.294 711.634 0.00164573 0.708642 -1.41115 -3.181143657 CDC73 no 1004.25 948.189 0.432151 1.05912 1.05912 0.7867453658 CDCA2 no 319.227 388.978 0.0895458 0.820683 -1.2185 -1.704183659 CDCA3 no 529.636 481.648 0.299262 1.09963 1.09963 1.04013660 CDCA4 no 819.823 1272.24 0.000115851 0.644394 -1.55184 -3.914573661 CDCA5 no 1355.33 1214.98 0.221424 1.11552 1.11552 1.22573662 CDCA7 no 1237.62 1382.94 0.487455 0.89492 -1.11742 -0.6953583663 CDCA7L no 1166.14 991.862 0.18988 1.17571 1.17571 1.314383664 CDCA8 no 841.149 967.314 0.111799 0.869572 -1.14999 -1.59563665 CDCP1 no 1844.83 2783.68 0.00850344 0.662731 -1.50891 -2.651673666 CDCP2 no 1.63176 1.96257 0.480572 0.831441 -1.20273 -0.7064033667 CDH1 no 8750.98 8661.68 0.925864 1.01031 1.01031 0.09314093669 CDH11 no 1133.19 496.978 0.00078923 2.28017 2.28017 3.396723670 CDH12 no 130.928 142.726 0.72811 0.917337 -1.09011 -0.3480173671 CDH12P1 no 1.95026 1.9217 0.926846 1.01486 1.01486 0.09190283672 CDH12P2 no 12.9336 13.8953 0.726568 0.930784 -1.07436 -0.3500743673 CDH12P3 no 1.81546 1.90581 0.760564 0.952591 -1.04977 -0.3050623674 CDH12P4 no 1.10799 1.17701 0.859955 0.941359 -1.06229 -0.1766073675 CDH13 no 402.204 585.439 0.336203 0.687012 -1.45558 -0.9634843676 CDH15 no 10.9664 10.8892 0.992987 1.00709 1.00709 0.008798353677 CDH16 no 152.253 393.898 0.55003 0.386529 -2.58713 -0.5984993679 CDH18 no 31.767 10.0092 0.00017228 3.17378 3.17378 3.812053680 CDH19 no 1.3852 6.56416 0.346065 0.211025 -4.73878 -0.9439693682 CDH20 no 19.4125 14.1428 0.26894 1.37261 1.37261 1.107883683 CDH22 no 64.7291 25.5161 0.0996694 2.5368 2.5368 1.652383684 CDH23 no 149.911 227.633 0.405372 0.658565 -1.51845 -0.8334263685 CDH24 no 537.516 672.024 0.0994478 0.799846 -1.25024 -1.653463686 CDH26 no 144.747 175.452 0.676694 0.824995 -1.21213 -0.4174523687 CDH3 no 3599.98 8161.2 0.00366884 0.441109 -2.26701 -2.932013688 CDH4 no 75.6859 72.3824 0.923337 1.04564 1.04564 0.09632533689 CDH5 no 388.661 293.277 0.38456 1.32524 1.32524 0.8710023690 CDH6 no 451.378 182.981 0.132996 2.46681 2.46681 1.507133691 CDH7 no 2.14767 3.67382 0.635992 0.584589 -1.7106 -0.4738693692 CDH8 no 32.6734 24.5491 0.609567 1.33094 1.33094 0.5113143693 CDH9 no 3.1586 1.65463 0.645478 1.90894 1.90894 0.4605923694 CDHR1 no 309.036 150.622 0.0677555 2.05173 2.05173 1.834313695 CDHR2 no 178.831 116.586 0.498233 1.5339 1.5339 0.6782273696 CDHR3 no 131.005 103.097 0.594839 1.27069 1.27069 0.5324963697 CDHR4 no 16.2031 20.5205 0.829423 0.789605 -1.26646 -0.215663698 CDHR5 no 351.634 110.989 0.0496144 3.16819 3.16819 1.972523699 CDIP1 no 563.246 897.771 0.000786243 0.627383 -1.59392 -3.39783700 CDIPT no 1775.45 1529.01 0.0200883 1.16118 1.16118 2.339153701 CDIPT-AS1 no 5.57873 4.28481 0.337483 1.30198 1.30198 0.9609313702 CDK1 no 1513.8 2006.5 0.00479993 0.754447 -1.32547 -2.844733703 CDK10 no 1389.21 1182.13 0.0685162 1.17517 1.17517 1.829223704 CDK11A no 1210.6 1262.23 0.541575 0.959098 -1.04265 -0.6112423705 CDK11B no 1060.1 1088.29 0.674375 0.974095 -1.02659 -0.4206293706 CDK12 no 1336.28 1126.93 0.0355673 1.18577 1.18577 2.112893707 CDK13 no 1151.66 1094.91 0.384537 1.05183 1.05183 0.8710433708 CDK14 no 371.026 407.809 0.627424 0.909802 -1.09914 -0.4859353709 CDK15 no 7.05033 5.92805 0.403448 1.18932 1.18932 0.8368513710 CDK16 no 3814.89 3023.49 0.0139454 1.26175 1.26175 2.475493711 CDK17 no 454.381 574.69 0.0126049 0.790655 -1.26477 -2.512253712 CDK18 no 620.154 836.955 0.0665465 0.740965 -1.34959 -1.842493713 CDK19 no 679.72 573.031 0.07122 1.18618 1.18618 1.811513714 CDK2 no 1157.11 1804.81 4.23E-05 0.641126 -1.55976 -4.165953715 CDK20 no 194.567 138.188 0.0214475 1.40799 1.40799 2.314063716 CDK2AP1 no 3132.23 2678.28 0.163785 1.16949 1.16949 1.396423717 CDK2AP2 no 1884.38 1766.29 0.679826 1.06686 1.06686 0.4131673718 CDK2AP2P1 no 1.07524 0.537696 0.00194812 1.99972 1.99972 3.130023719 CDK2AP2P3 no 0.8018 0.692621 0.605323 1.15763 1.15763 0.5173933720 CDK3 no 289.796 319.368 0.516339 0.907404 -1.10204 -0.649893721 CDK4 no 3966.92 3483.76 0.08146 1.13869 1.13869 1.749093722 CDK4PS no 0 0 ? 1 1 03723 CDK5 no 540.635 355.841 3.25E-08 1.51932 1.51932 5.700393724 CDK5PS no 0 0.00728488 0.511155 ? ? -0.657953725 CDK5R1 no 214.203 226.308 0.773164 0.946512 -1.05651 -0.2885413726 CDK5R2 no 3.9889 2.428 0.308867 1.64287 1.64287 1.019613727 CDK5RAP1 no 666.77 683.338 0.765564 0.975753 -1.02485 -0.2984963728 CDK5RAP2 no 1659.69 1516.31 0.296465 1.09455 1.09455 1.046153729 CDK5RAP3 no 3129.35 2936.3 0.49779 1.06574 1.06574 0.6789273730 CDK6 no 1196.55 779.576 0.00849796 1.53487 1.53487 2.651893731 CDK7 no 617.373 573.472 0.340328 1.07655 1.07655 0.9552783732 CDK7PS no 0.313251 0.291676 0.812627 1.07397 1.07397 0.2372813733 CDK8 no 522.182 380.013 7.24E-05 1.37412 1.37412 4.033423734 CDK9 no 1265.67 1292.68 0.771421 0.979103 -1.02134 -0.2908213735 CDKAL1 no 483.372 552.119 0.618152 0.875486 -1.14222 -0.4990723736 CDKL1 no 79.0676 79.7593 0.951761 0.991327 -1.00875 -0.06055433737 CDKL2 no 101.981 25.5741 2.32E-06 3.98766 3.98766 4.831993738 CDKL3 no 101.859 83.0167 0.0237269 1.22697 1.22697 2.274953739 CDKL4 no 8.15525 5.30124 0.146565 1.53837 1.53837 1.456143740 CDKL5 no 227.918 247.846 0.624226 0.919595 -1.08743 -0.4904563741 CDKN1A no 3753.2 6698.03 0.00138981 0.560344 -1.78462 -3.231723742 CDKN1B no 1381.63 1374.26 0.958865 1.00537 1.00537 0.05162773743 CDKN1C no 766.003 531.901 0.137858 1.44012 1.44012 1.488423744 CDKN2A no 1346.28 4493.89 2.39E-09 0.29958 -3.33801 -6.186333745 CDKN2AIP no 471.522 487.872 0.74902 0.966488 -1.03467 -0.3202723746 CDKN2AIPNL no 617.571 426.806 0.000259051 1.44696 1.44696 3.704363747 CDKN2AIPNLP1 no 0.0504083 0.0391516 0.728376 1.28752 1.28752 0.3476623748 CDKN2AIPNLP2 no 0 0.0159061 0.326131 ? ? -0.9838013750 CDKN2B no 470.544 1846.55 0.000198558 0.254824 -3.92428 -3.774853752 CDKN2C no 600.65 1473.98 0.000249792 0.407502 -2.45398 -3.714073753 CDKN2D no 256.612 413.643 0.00832092 0.620371 -1.61194 -2.659193754 CDKN3 no 436.36 485.125 0.338809 0.899479 -1.11176 -0.9582923755 CDNF no 27.4443 18.4235 0.00535268 1.48964 1.48964 2.808733757 CDON no 240.262 196.837 0.471304 1.22061 1.22061 0.7214163758 CDPF1 no 213.965 166.473 0.00255803 1.28529 1.28529 3.046063759 CDR1 no 1.93151 0.923492 0.0637285 2.09153 2.09153 1.862053760 CDR2 no 641.97 462.433 0.00279275 1.38825 1.38825 3.018613761 CDR2L no 772.806 795.604 0.887621 0.971345 -1.0295 -0.1414543762 CDRT1 no 30.5151 45.4997 0.179608 0.670664 -1.49106 -1.34563763 CDRT15 no 2.65881 2.99961 0.620091 0.886386 -1.12818 -0.4963183765 CDRT15P1 no 9.97108 12.2001 0.311531 0.817295 -1.22355 -1.0143766 CDRT15P2 no 0.0284903 0.00748327 0.13399 3.80721 3.80721 1.503263767 CDRT4 no 8.91034 5.42075 0.00121156 1.64375 1.64375 3.272353768 CDRT7 no 0.235074 0.0882863 0.0377179 2.66263 2.66263 2.088633769 CDRT8 no 0.0171063 0.00803373 0.624397 2.12931 2.12931 0.4902143770 CDS1 no 798.404 866.921 0.454076 0.920965 -1.08582 -0.7497663771 CDS2 no 1169.99 1006.78 0.069392 1.16211 1.16211 1.823423772 CDSN no 125.254 67.4712 0.638453 1.8564 1.8564 0.4704173773 CDT1 no 945.571 1376.62 0.00218112 0.686879 -1.45586 -3.095413774 CDV3 no 3478.41 3642.02 0.622296 0.955076 -1.04704 -0.4931913775 CDV3P1 no 0.0248626 0.0083072 0.259177 2.99289 2.99289 1.130823778 CDX4 no 0.072777 0.0603121 0.817542 1.20667 1.20667 0.2309433779 CDY1 no 0 0.00208887 0.768013 ? ? -0.2952843780 CDY10P no 0 0 ? 1 1 03781 CDY11P no 0 0 ? 1 1 03782 CDY12P no 0 0 ? 1 1 03783 CDY13P no 0 0 ? 1 1 03784 CDY14P no 0 0 ? 1 1 03785 CDY15P no 0 0 ? 1 1 03786 CDY16P no 0 0 ? 1 1 03787 CDY17P no 0 0 ? 1 1 03788 CDY18P no 0 0 ? 1 1 03789 CDY19P no 0 0.00175168 0.768013 ? ? -0.2952843790 CDY1B no 0 0 ? 1 1 03791 CDY20P no 0 0 ? 1 1 03792 CDY21P no 0 0 ? 1 1 03793 CDY22P no 0 0.00154943 0.768013 ? ? -0.2952843794 CDY23P no 0 0.00106217 0.768013 ? ? -0.2952843795 CDY2A no 0 0 ? 1 1 03796 CDY2B no 0 0 ? 1 1 03797 CDY3P no 0 0.00386395 0.616722 ? ? -0.5011063798 CDY4P no 0 0 ? 1 1 03799 CDY5P no 0 0 ? 1 1 03800 CDY6P no 0 0 ? 1 1 03801 CDY7P no 0 0 ? 1 1 03802 CDY8P no 0 0 ? 1 1 03803 CDY9P no 0 0 ? 1 1 03804 CDYL no 738.973 689.82 0.429976 1.07125 1.07125 0.7904713805 CDYL2 no 55.0377 40.4582 0.144009 1.36036 1.36036 1.465463806 CEACAM1 no 1385.53 1008.87 0.212876 1.37335 1.37335 1.248773807 CEACAM16 no 4.60144 3.87889 0.866277 1.18628 1.18628 0.1685563808 CEACAM18 no 1.09205 0.808742 0.880288 1.35031 1.35031 0.1507533809 CEACAM19 no 180.767 214.392 0.548983 0.843159 -1.18602 -0.6000723811 CEACAM21 no 60.8953 74.3789 0.481832 0.818718 -1.22142 -0.7043753812 CEACAM22P no 6.12027 1.66858 0.00562057 3.66796 3.66796 2.792483813 CEACAM3 no 7.83276 16.6692 0.200611 0.469893 -2.12814 -1.283083814 CEACAM4 no 4.55021 9.53776 0.0650742 0.477073 -2.09612 -1.852623815 CEACAM5 no 13350.9 12437.3 0.894687 1.07345 1.07345 0.1325073816 CEACAM6 no 5060.66 8308.51 0.394542 0.609094 -1.64178 -0.8528293817 CEACAM7 no 1120.35 800.813 0.566668 1.39902 1.39902 0.5737023818 CEACAM8 no 0.638885 0.378155 0.122378 1.68948 1.68948 1.549933819 CEACAMP1 no 0.017855 0.0147745 0.894995 1.20851 1.20851 0.1321173820 CEACAMP10 no 0.0406172 0.0601067 0.667997 0.675752 -1.47983 -0.429393821 CEACAMP11 no 0.0400873 0.373179 0.225017 0.107421 -9.30916 -1.21623822 CEACAMP2 no 0 0.00243018 0.678521 ? ? -0.4149523823 CEACAMP3 no 0 0.00718151 0.514944 ? ? -0.6520553824 CEACAMP4 no 0 0.00689419 0.565739 ? ? -0.5750763826 CEACAMP6 no 0.0210637 0.044876 0.505429 0.469376 -2.13049 -0.6669023827 CEACAMP7 no 0 0.0870314 0.308762 ? ? -1.019833828 CEACAMP8 no 0 0.0384337 0.196266 ? ? -1.29563829 CEACAMP9 no 0.0731172 0.0807267 0.912545 0.905738 -1.10407 -0.1099363830 CEBPA no 497.983 904.185 0.00801153 0.550754 -1.81569 -2.672293831 CEBPA-AS1 no 54.8939 52.5963 0.850258 1.04368 1.04368 0.1889783832 CEBPB no 1345.97 1935.79 0.0124717 0.69531 -1.43821 -2.516093833 CEBPD no 1230.9 1590.38 0.166218 0.773964 -1.29205 -1.388373834 CEBPE no 1.01155 12.135 0.497138 0.0833584 -11.9964 -0.6799593835 CEBPG no 1404.78 1652.79 0.0814502 0.849948 -1.17654 -1.749153836 CEBPZ no 2355.66 1978.37 0.00334934 1.19071 1.19071 2.961153837 CECR1 no 854.488 569.815 0.0599961 1.49959 1.49959 1.88913838 CECR2 no 78.7223 72.0895 0.827945 1.09201 1.09201 0.2175583840 CECR5 no 1097.43 826.795 9.14E-05 1.32733 1.32733 3.974823841 CECR5-AS1 no 1.49621 1.28031 0.523064 1.16863 1.16863 0.6394993842 CECR6 no 18.8848 12.4211 0.0156474 1.52038 1.52038 2.43313843 CECR7 no 31.2132 90.5318 0.00160141 0.344776 -2.90043 -3.189353844 CEL no 201.048 1138.44 0.222423 0.1766 -5.6625 -1.223053845 CELA1 no 2.6812 0.835194 8.30E-05 3.21027 3.21027 3.9993846 CELA2A no 2.90849 2.64982 0.725099 1.09762 1.09762 0.3520343847 CELA2B no 46.7304 37.3789 0.12785 1.25018 1.25018 1.527533848 CELA3A no 1.33961 1.04589 0.836208 1.28083 1.28083 0.2069543849 CELA3B no 0.998621 0.608225 0.351741 1.64186 1.64186 0.93293850 CELF1 no 2864.48 2378.88 0.00142988 1.20413 1.20413 3.223263851 CELF2 no 450.625 327.291 0.165868 1.37683 1.37683 1.389523852 CELF2-AS1 no 0.0171063 0.00171148 0.0445682 9.99501 9.99501 2.018523853 CELF2-AS2 no 0 0 ? 1 1 03855 CELF4 no 44.6645 61.4289 0.360367 0.727093 -1.37534 -0.916293857 CELF6 no 60.2066 45.4412 0.173177 1.32493 1.32493 1.365833858 CELP no 4.78256 1.78567 0.196755 2.6783 2.6783 1.294183859 CELSR1 no 2762.98 2836 0.86027 0.974252 -1.02643 -0.1762063860 CELSR2 no 2094.26 3412.74 0.00523123 0.613661 -1.62957 -2.816343861 CELSR3 no 395.63 753.737 0.0121875 0.524891 -1.90516 -2.524413862 CEMP1 no 113.375 137.861 0.218839 0.822386 -1.21597 -1.232613863 CEND1 no 12.0075 6.78876 0.0983811 1.76873 1.76873 1.658733864 CEND1P1 no 0 0.00190678 0.768013 ? ? -0.2952843866 CENPA no 413.446 480.955 0.160527 0.859634 -1.16329 -1.407343867 CENPB no 2141.57 2592.3 0.0374026 0.826127 -1.21047 -2.092113868 CENPBD1 no 254.589 228.051 0.343601 1.11637 1.11637 0.9488123869 CENPC no 276.686 387.104 0.00538268 0.714758 -1.39907 -2.806873871 CENPE no 580.318 676.347 0.225935 0.858018 -1.16548 -1.213793872 CENPF no 2464.93 2767.14 0.305189 0.890786 -1.1226 -1.02743873 CENPH no 459.136 517.901 0.288512 0.886533 -1.12799 -1.063573874 CENPI no 186.294 255.985 0.0146789 0.727754 -1.37409 -2.456693875 CENPIP1 no 0.14657 0.0895692 0.658941 1.63639 1.63639 0.4418853876 CENPJ no 417.409 544.645 0.0350876 0.766386 -1.30482 -2.118473877 CENPK no 293.942 596.208 9.72E-06 0.493019 -2.02832 -4.512633878 CENPL no 256.678 305.853 0.0671488 0.83922 -1.19158 -1.83843879 CENPM no 480.455 562.483 0.269339 0.854168 -1.17073 -1.106963880 CENPN no 710.742 871.063 0.0273647 0.815948 -1.22557 -2.218843881 CENPO no 259.602 263.844 0.872776 0.983924 -1.01634 -0.1602923882 CENPP no 361.197 540.922 0.0105701 0.667743 -1.49758 -2.575333883 CENPQ no 226.854 304.871 0.00652228 0.744097 -1.34391 -2.742523884 CENPT no 1747.01 1677.87 0.575322 1.04121 1.04121 0.5609423885 CENPV no 483.559 247.241 0.000105597 1.95582 1.95582 3.93823889 CENPW no 626.395 1352.25 0.0321821 0.463225 -2.15878 -2.153763890 CEP104 no 784.092 705.165 0.134764 1.11193 1.11193 1.500263891 CEP112 no 297.62 131.166 1.16E-07 2.26903 2.26903 5.452053892 CEP120 no 445.887 425.885 0.611803 1.04697 1.04697 0.5081183893 CEP128 no 179.768 241.402 0.0195789 0.744684 -1.34285 -2.348943894 CEP135 no 241.123 299.058 0.117481 0.806276 -1.24027 -1.570673895 CEP152 no 191.084 277.676 0.00477883 0.688155 -1.45316 -2.846183896 CEP164 no 608.357 516.357 0.0461688 1.17817 1.17817 2.003473897 CEP164P1 no 0.108564 0.0496778 0.338939 2.18536 2.18536 0.9580343898 CEP170 no 802.662 584.959 0.00714629 1.37217 1.37217 2.711493899 CEP170B no 1861.67 2383.25 0.0499133 0.781147 -1.28017 -1.969923900 CEP170P1 no 0.371904 0.412039 0.749981 0.902594 -1.10792 -0.3190033901 CEP19 no 98.7783 127.441 0.287054 0.775089 -1.29017 -1.06683902 CEP192 no 719.338 769.056 0.551353 0.935353 -1.06912 -0.5965143903 CEP250 no 1355.98 1321.18 0.85446 1.02634 1.02634 0.1836143904 CEP290 no 408.556 330.318 0.0513493 1.23686 1.23686 1.957613905 CEP350 no 945.948 1154.78 0.0239902 0.819155 -1.22077 -2.270643906 CEP41 no 334.101 187.761 8.00E-07 1.7794 1.7794 5.058663907 CEP44 no 366.181 398.879 0.550546 0.918027 -1.08929 -0.5977253908 CEP55 no 835.24 1201.96 0.00505683 0.694898 -1.43906 -2.827553909 CEP57 no 895.883 915.18 0.869521 0.978914 -1.02154 -0.1644313910 CEP57L1 no 170.983 192.351 0.247928 0.88891 -1.12497 -1.158013911 CEP57L1P1 no 0.204275 0.130741 0.203479 1.56244 1.56244 1.274923912 CEP63 no 566.723 647.577 0.1221 0.875143 -1.14267 -1.55113913 CEP68 no 542.699 425.107 0.0222224 1.27662 1.27662 2.300373914 CEP70 no 642.857 734.85 0.25924 0.874815 -1.1431 -1.130673915 CEP72 no 287.1 414.491 0.0355771 0.692657 -1.44372 -2.112773916 CEP76 no 156.738 123.606 0.015112 1.26804 1.26804 2.445983917 CEP78 no 442.069 447.654 0.885039 0.987523 -1.01263 -0.1447273918 CEP85 no 506.776 685.388 0.0132718 0.7394 -1.35245 -2.493553919 CEP85L no 160.05 122.537 0.234525 1.30614 1.30614 1.191563920 CEP89 no 490.059 512.346 0.604487 0.956499 -1.04548 -0.5185933921 CEP95 no 777.418 738.82 0.610877 1.05224 1.05224 0.5094423922 CEP97 no 297.907 345.258 0.210369 0.862854 -1.15895 -1.255663923 CEPT1 no 784.21 839.056 0.321898 0.934634 -1.06994 -0.9924633924 CER1 no 0.814246 0.298703 0.148259 2.72594 2.72594 1.450033925 CERCAM no 1325.65 861.593 0.000970582 1.5386 1.5386 3.337153926 CERK no 1995.41 905.167 8.43E-14 2.20447 2.20447 7.891643927 CERKL no 87.4095 84.7058 0.888619 1.03192 1.03192 0.1401893929 CERS2 no 3735.52 2693.05 2.31E-05 1.3871 1.3871 4.311213930 CERS3 no 207.855 676.135 0.0160767 0.307417 -3.25291 -2.423063931 CERS4 no 860.03 763.959 0.543398 1.12575 1.12575 0.6084873932 CERS5 no 923.444 904.074 0.748979 1.02143 1.02143 0.3203263933 CERS6 no 2047.61 1573.24 0.00514732 1.30152 1.30152 2.821693934 CERS6-AS1 no 21.6899 16.2531 0.0998662 1.33451 1.33451 1.651413935 CES1 no 2865.81 1512.01 0.460265 1.89537 1.89537 0.7395143936 CES1P1 no 0.668199 0.522708 0.900081 1.27834 1.27834 0.1256823937 CES1P2 no 1.27857 0.814958 0.567508 1.56887 1.56887 0.5724593938 CES2 no 1567.82 1942.47 0.347901 0.807131 -1.23896 -0.9403763939 CES3 no 240.064 120.092 0.0287787 1.999 1.999 2.198773940 CES4A no 111.695 113.148 0.941573 0.987155 -1.01301 -0.07336433942 CES5AP1 no 5.5839 4.0849 0.877245 1.36696 1.36696 0.1546153943 CETN1 no 0 0.00398978 0.612639 ? ? -0.5069253944 CETN2 no 1369.25 1315.87 0.811644 1.04057 1.04057 0.2385493945 CETN3 no 460.235 358.641 0.00211307 1.28327 1.28327 3.105153946 CETN4P no 1.48364 2.15515 0.0792098 0.688417 -1.45261 -1.762233947 CETP no 42.1166 43.226 0.895913 0.974336 -1.02634 -0.1309543948 CFB no 2763.44 5816.03 0.118994 0.475143 -2.10463 -1.564193951 CFD no 201.854 348.937 0.544318 0.578483 -1.72866 -0.6070973952 CFDP1 no 1402.35 1397.13 0.968343 1.00374 1.00374 0.03972533953 CFH no 750.062 1833.37 0.0155715 0.409116 -2.4443 -2.43493954 CFHR1 no 2.24206 2.51754 0.885049 0.890578 -1.12287 -0.1447153955 CFHR2 no 0.0822554 0.362218 0.443678 0.227088 -4.40358 -0.7671713956 CFHR3 no 7.76369 6.58546 0.658137 1.17891 1.17891 0.4429993958 CFHR5 no 0.366803 0.149913 0.111584 2.44677 2.44677 1.596563959 CFI no 1396.5 610.949 0.000150358 2.28578 2.28578 3.847463960 CFL1 no 21307.8 17592.5 0.00238379 1.21119 1.21119 3.067983961 CFL1P1 no 1.92627 1.90902 0.970369 1.00904 1.00904 0.03718133962 CFL1P2 no 0.198622 0.189888 0.905222 1.046 1.046 0.1191843963 CFL1P3 no 0 0.00161121 0.768013 ? ? -0.2952843964 CFL1P4 no 0.0844272 0.0412955 0.207715 2.04446 2.04446 1.263033965 CFL1P5 no 0.0235391 0.0462444 0.450821 0.509016 -1.96457 -0.755193966 CFL1P6 no 2.29291 2.00444 0.522193 1.14392 1.14392 0.640843967 CFL1P7 no 0 0 ? 1 1 03969 CFL2 no 395.434 371.975 0.690428 1.06306 1.06306 0.398723970 CFLAR no 1689.54 2250.83 0.0116576 0.750629 -1.33222 -2.540393971 CFLAR-AS1 no 0.0213091 0.0103424 0.485369 2.06036 2.06036 0.6986953972 CFP no 26.3278 34.4627 0.271077 0.763951 -1.30898 -1.102943973 CFTR no 528.363 189.675 0.0474078 2.78563 2.78563 1.992123974 CFTRP1 no 0 0.0158964 0.417612 ? ? -0.8118693975 CGA no 21.5239 238.032 0.758729 0.0904246 -11.0589 -0.3074753976 CGB no 4.54085 11.5714 0.797943 0.392421 -2.54829 -0.2562743977 CGB1 no 0.827657 0.915569 0.836663 0.903981 -1.10622 -0.2063713978 CGB2 no 3.08707 1.54845 0.0332916 1.99365 1.99365 2.139973979 CGB5 no 4.46348 25.8204 0.579633 0.172867 -5.7848 -0.554623981 CGB8 no 1.57852 25.2237 0.5035 0.0625807 -15.9794 -0.6699293982 CGGBP1 no 1937.52 1858.28 0.601373 1.04264 1.04264 0.523073983 CGN no 1354.41 1210 0.608054 1.11935 1.11935 0.5134793984 CGNL1 no 544.395 194.998 4.75E-05 2.7918 2.7918 4.137433985 CGREF1 no 406.785 161.277 6.46E-05 2.52228 2.52228 4.061823986 CGRRF1 no 178.602 147.375 0.0285253 1.21188 1.21188 2.20233987 CH25H no 87.7119 76.9891 0.777981 1.13928 1.13928 0.2822453988 CHAC1 no 202.093 146.501 0.108316 1.37946 1.37946 1.611383989 CHAC2 no 40.2379 38.711 0.828591 1.03944 1.03944 0.2167293990 CHAD no 1.27407 14.1582 0.492341 0.0899884 -11.1125 -0.6875673991 CHADL no 161.73 211.11 0.283192 0.766095 -1.30532 -1.075413992 CHAF1A no 1024.12 1394.91 0.00166483 0.734186 -1.36205 -3.177663993 CHAF1B no 469.202 652.826 0.0083941 0.718725 -1.39135 -2.656163994 CHAMP1 no 540.974 469.944 0.143595 1.15115 1.15115 1.466983995 CHAT no 36.8989 24.6696 0.636775 1.49572 1.49572 0.472773996 CHCHD1 no 864.524 770.238 0.107248 1.12241 1.12241 1.616293997 CHCHD10 no 915.67 804.661 0.423597 1.13796 1.13796 0.8014663998 CHCHD2 no 5952.19 4833.21 0.0406034 1.23152 1.23152 2.057873999 CHCHD2P1 no 0.017855 0.00975838 0.555372 1.82971 1.82971 0.5904984000 CHCHD2P10 no 0.069193 0.0367207 0.272793 1.8843 1.8843 1.098994001 CHCHD2P2 no 82.1576 56.7421 0.00118977 1.44791 1.44791 3.277694002 CHCHD2P3 no 0 0.00383215 0.612236 ? ? -0.50754003 CHCHD2P4 no 0.391771 0.217145 0.213749 1.80419 1.80419 1.246384004 CHCHD2P5 no 0 0.0118971 0.460945 ? ? -0.7383924005 CHCHD2P6 no 11.2529 10.4107 0.553114 1.0809 1.0809 0.5938744006 CHCHD2P7 no 0.0795896 0.0308141 0.19414 2.58289 2.58289 1.30184007 CHCHD2P8 no 0.0203008 0.0262525 0.816164 0.773292 -1.29317 -0.2327184008 CHCHD2P9 no 128.043 102.459 0.0449821 1.2497 1.2497 2.014584009 CHCHD3 no 2774.55 1994.33 3.28E-06 1.39122 1.39122 4.756184010 CHCHD3P1 no 0.0287222 0.016298 0.659108 1.76232 1.76232 0.4416544011 CHCHD3P2 no 0 0.00123738 0.768013 ? ? -0.2952844012 CHCHD3P3 no 0.285236 0.260128 0.759663 1.09652 1.09652 0.3062474013 CHCHD4 no 360.593 287.849 0.00637233 1.25272 1.25272 2.750384014 CHCHD4P1 no 0.0129226 0.0265935 0.586018 0.485931 -2.05791 -0.5452974015 CHCHD4P2 no 0.0377851 0.0308144 0.811057 1.22622 1.22622 0.2393064016 CHCHD4P4 no 0.0171063 0.0020615 0.0800851 8.29796 8.29796 1.757084017 CHCHD5 no 328.082 359.207 0.630353 0.913349 -1.09487 -0.4818034018 CHCHD6 no 436.459 390.398 0.410922 1.11798 1.11798 0.8236044019 CHCHD7 no 964.337 494.764 2.30E-15 1.94908 1.94908 8.438084020 CHD1 no 866.158 828.312 0.564467 1.04569 1.04569 0.5769614021 CHD1L no 1137.27 849.336 0.000477219 1.33901 1.33901 3.538264022 CHD2 no 1942.93 2033.13 0.540284 0.955632 -1.04643 -0.6131974023 CHD3 no 4933.44 3995.12 0.0482337 1.23487 1.23487 1.984694024 CHD4 no 6883.8 4853.31 2.70E-07 1.41837 1.41837 5.283124025 CHD5 no 14.8987 9.51542 0.317821 1.56575 1.56575 1.000884026 CHD6 no 1134.73 1178.27 0.935895 0.963048 -1.03837 -0.08050814027 CHD7 no 1084.16 607.804 9.47E-08 1.78374 1.78374 5.492234028 CHD8 no 1775.43 1625.41 0.248025 1.09229 1.09229 1.157774029 CHD9 no 637.734 975.225 0.0011737 0.653935 -1.5292 -3.281694030 CHDC2 no 3.33056 6.09401 0.440446 0.54653 -1.82972 -0.7726294031 CHDH no 417.616 195.501 0.000147937 2.13613 2.13613 3.851664032 CHEK1 no 649.503 746.862 0.160584 0.869643 -1.1499 -1.407144033 CHEK2 no 606.753 591.577 0.749256 1.02565 1.02565 0.3199614034 CHEK2P1 no 0.0171063 0.0134576 0.820706 1.27112 1.27112 0.2268674035 CHEK2P2 no 0.22656 0.235343 0.957488 0.962678 -1.03877 -0.05335824036 CHEK2P3 no 0 0 ? 1 1 04037 CHERP no 1814.95 1430.13 0.000351806 1.26908 1.26908 3.621914038 CHFR no 611.416 715.345 0.0575933 0.854714 -1.16998 -1.907264041 CHI3L1 no 1010.99 1465.26 0.706264 0.689976 -1.44933 -0.3772954042 CHI3L2 no 33.9626 108.152 0.0899666 0.314026 -3.18445 -1.701944044 CHIAP1 no 0.0677598 0.044998 0.649103 1.50584 1.50584 0.455544045 CHIAP2 no 0.0287222 0.00947757 0.215973 3.03055 3.03055 1.240344046 CHIC1 no 206.862 176.781 0.246986 1.17016 1.17016 1.160324047 CHIC2 no 331.851 504.472 0.480059 0.657818 -1.52018 -0.7072314048 CHID1 no 2121.42 1612.95 9.41E-05 1.31524 1.31524 3.967524049 CHIT1 no 76.9865 269.657 0.443989 0.285498 -3.50265 -0.7666494050 CHKA no 1241.87 515.045 5.83E-10 2.41119 2.41119 6.438364051 CHKB no 586.322 724.719 0.0551274 0.809034 -1.23604 -1.926574052 CHKB-AS1 no 13.9845 14.3313 0.938416 0.975802 -1.0248 -0.07733614053 CHKB-CPT1B no 6.83065 8.69811 0.342922 0.785303 -1.27339 -0.9501494054 CHL1 no 279.429 393.842 0.498248 0.709495 -1.40945 -0.6782034055 CHM no 435.037 413.463 0.629994 1.05218 1.05218 0.4823094056 CHML no 1.60043 0.908379 0.0138032 1.76185 1.76185 2.479244057 CHMP1A no 2526.34 2732.94 0.268485 0.924405 -1.08178 -1.108944059 CHMP1B no 36.7688 19.5867 0.00176849 1.87724 1.87724 3.159424060 CHMP2A no 2449.39 2568.57 0.717301 0.953601 -1.04866 -0.3624644061 CHMP2B no 949.859 9
[truncated: 2,045,128 more chars]
