# Supplementary material for: Identification and characterization of HPV-independent cervical cancers
Source: Oncotarget. 2017 Jan 6;8(8):13375–86. doi: 10.18632/oncotarget.14533 (PMC5355105; doi:10.18632/oncotarget.14533)
Supplement: Supplementary file 8 [file oncotarget-08-13375-s008.docx]

Supplemental Table 5: Methylation ANOVA short list Probeset ID Gene Symbol Mean(0) Mean(1) p-value(0 vs. 1) MeanRatio(0 vs. 1) FoldChange(0 vs. 1) FoldChange(0 vs. 1) (Description) T(0 vs. 1) F(Cluster) SS(Cluster) SS(Error) F(Error)cg12473912 -0.22514 0.299176 1.54E-38 -0.752534 -1.32884 0 down vs 1 -16.0718 258.302 4.9843 4.09083 1cg21827317 -0.157415 0.343348 1.45E-37 -0.45847 -2.18117 0 down vs 1 -15.7637 248.494 4.54656 3.87885 1cg05438378 SMAD3 -0.07625 -0.408825 3.02E-36 0.18651 -5.36164 0 down vs 1 15.3471 235.534 2.00538 1.80501 1cg22099441 0.08462 0.460603 5.19E-36 0.183716 -5.44319 0 down vs 1 -15.2729 233.261 2.56304 2.32943 1cg05024916 0.114515 0.425329 1.18E-35 0.269239 -3.71418 0 down vs 1 -15.16 229.826 1.75153 1.61568 1cg18002947 0.10545 0.444647 4.53E-35 0.237154 -4.21667 0 down vs 1 -14.9763 224.29 2.08604 1.97173 1cg12035325 LRBA 0.0627 0.370498 1.78E-34 0.169232 -5.90906 0 down vs 1 -14.7891 218.718 1.71771 1.66495 1cg19846609 -0.05423 0.434654 2.26E-34 -0.124766 -8.01501 0 down vs 1 -14.7566 217.758 4.33341 4.21883 1cg22617002 0.066145 0.425312 1.34E-33 0.155521 -6.43 0 down vs 1 -14.5133 210.636 2.3389 2.35405 1cg14587524 ZNF781 -0.265945 0.170328 1.40E-33 -1.56137 1.56137 0 up vs 1 -14.5073 210.463 3.45092 3.47613 1cg15978565 LPIN1 -0.084695 -0.427806 1.40E-32 0.197975 -5.05114 0 down vs 1 14.1921 201.416 2.13446 2.24661 1cg11557932 CDYL 0.14204 0.453242 1.76E-32 0.313387 -3.19094 0 down vs 1 -14.161 200.534 1.75591 1.85631 1cg19633390 0.002395 0.39412 2.99E-32 0.00607684 -164.559 0 down vs 1 -14.0886 198.49 2.78214 2.97151 1cg16472853 THADA 0.1406 0.431209 8.45E-32 0.32606 -3.06692 0 down vs 1 -13.9465 194.505 1.53122 1.66894 1cg17120366 MPP7 -0.00697 0.363225 1.78E-31 -0.0191892 -52.1126 0 down vs 1 -13.8447 191.675 2.48473 2.7482 1cg25324105 ZNF781 -0.28168 0.138657 2.24E-31 -2.03148 2.03148 0 up vs 1 -13.8131 190.802 3.20342 3.55932 1cg03387289 0.07124 0.424622 4.09E-31 0.167773 -5.96045 0 down vs 1 -13.7307 188.532 2.26416 2.54599 1cg03468463 SERPINB12 0.150165 0.41636 7.86E-31 0.360662 -2.77268 0 down vs 1 -13.6413 186.084 1.28475 1.46367 1cg07962143 -0.25542 0.324266 1.12E-30 -0.787687 -1.26954 0 down vs 1 -13.5929 184.768 6.09261 6.99056 1cg23476937 ACTL9 0.04018 0.318747 1.87E-30 0.126056 -7.93299 0 down vs 1 -13.5226 182.861 1.40695 1.63115 1cg12015737 HOXA5 0.007315 0.401218 3.03E-30 0.018232 -54.8487 0 down vs 1 -13.4562 181.07 2.81317 3.29372 1cg13100190 0.00742 0.384813 3.63E-30 0.0192821 -51.8616 0 down vs 1 -13.4315 180.406 2.58229 3.03451 1cg03611452 ZNF781 -0.30601 0.0486459 8.47E-30 -6.29056 6.29056 0 up vs 1 -13.3155 177.301 2.28051 2.72682 1cg18705067 SRRM3 0.149985 0.46684 8.91E-30 0.321277 -3.11258 0 down vs 1 -13.3086 177.119 1.82029 2.17877 1cg16264458 HRNBP3 0.13726 0.433124 1.03E-29 0.316907 -3.1555 0 down vs 1 -13.288 176.571 1.58709 1.90554 1cg24080008 -0.139035 0.261953 1.25E-29 -0.530764 -1.88408 0 down vs 1 -13.2618 175.876 2.91528 3.51406 1cg26444351 0.12168 0.389669 1.39E-29 0.312265 -3.20241 0 down vs 1 -13.2478 175.506 1.30212 1.57289 1cg16001422 PLEC1 -0.18556 -0.399146 2.58E-29 0.464892 -2.15104 0 down vs 1 13.1625 173.251 0.827114 1.01211 1cg04332422 STON2 0.17108 0.43123 2.76E-29 0.396725 -2.52064 0 down vs 1 -13.1533 173.009 1.22706 1.5036 1cg08070327 HOXA5 0.087315 0.407722 8.43E-29 0.214153 -4.66955 0 down vs 1 -13.0001 169.002 1.86133 2.33489 1cg11421182 BLCAP -0.08155 -0.345695 9.55E-29 0.235901 -4.23906 0 down vs 1 12.9828 168.553 1.26504 1.59112 1cg26520847 -0.076515 0.273964 9.99E-29 -0.279288 -3.58053 0 down vs 1 -12.9767 168.395 2.22712 2.80381 1cg21477262 ETNK1 -0.008415 0.374792 1.04E-28 -0.0224524 -44.5386 0 down vs 1 -12.9714 168.257 2.66247 3.35466 1cg04650676 9-Sep -0.1049 -0.459203 1.10E-28 0.228439 -4.37753 0 down vs 1 12.9632 168.044 2.27597 2.8713 1cg00532449 -0.33808 0.0995825 1.30E-28 -3.39497 3.39497 0 up vs 1 -12.9403 167.452 3.47293 4.39684 1cg20040306 CLEC12B 0.001095 0.367837 1.76E-28 0.00297687 -335.924 0 down vs 1 -12.8988 166.379 2.43859 3.10725 1cg16615154 -0.233355 0.114382 2.04E-28 -2.04013 2.04013 0 up vs 1 -12.8787 165.86 2.19241 2.80231 1cg16770832 PELI2 0.0224 0.341875 2.17E-28 0.065521 -15.2623 0 down vs 1 -12.8701 165.64 1.85051 2.36844 1cg26047066 0.067465 0.4412 2.25E-28 0.152913 -6.53969 0 down vs 1 -12.8648 165.504 2.53248 3.24395 1cg04922063 CCNY 0.15761 0.368978 2.44E-28 0.427153 -2.34108 0 down vs 1 -12.8537 165.218 0.81002 1.03938 1cg22870307 0.060345 0.443769 3.17E-28 0.135983 -7.35386 0 down vs 1 -12.8176 164.291 2.66548 3.43952 1cg16075268 LTB4R2 -0.122505 -0.367149 3.33E-28 0.333666 -2.99701 0 down vs 1 12.811 164.121 1.08514 1.40171 1cg14013695 HOXA5 -0.00866 0.405731 3.88E-28 -0.0213442 -46.8512 0 down vs 1 -12.79 163.584 3.11343 4.03491 1cg23539753 SP100 -0.203075 -0.444349 4.24E-28 0.457017 -2.1881 0 down vs 1 12.7778 163.273 1.05545 1.37044 1cg18544938 SOSTDC1 0.08626 0.404398 4.59E-28 0.213304 -4.68813 0 down vs 1 -12.7669 162.995 1.83506 2.38678 1cg04086391 DPP6 0.12724 0.376388 7.18E-28 0.338056 -2.95809 0 down vs 1 -12.7053 161.424 1.12546 1.47809 1cg26164879 -0.16836 0.168625 7.77E-28 -0.998427 -1.00158 0 down vs 1 -12.6943 161.146 2.05892 2.70867 1cg08296730 TRIM73 0.008305 0.366868 8.26E-28 0.0226376 -44.1744 0 down vs 1 -12.6858 160.931 2.33104 3.07076 1cg01603456 0.04858 0.318041 8.55E-28 0.152748 -6.54674 0 down vs 1 -12.6812 160.813 1.31646 1.7355 1cg02723291 ETNK1 0.00061 0.422669 1.16E-27 0.00144321 -692.899 0 down vs 1 -12.6392 159.748 3.22971 4.28611 1cg10152627 0.145635 0.420531 1.36E-27 0.346312 -2.88757 0 down vs 1 -12.6176 159.205 1.37011 1.82446 1cg22350070 CTNNA2 -0.3545 0.160033 1.38E-27 -2.21517 2.21517 0 up vs 1 -12.6155 159.15 4.80003 6.39401 1cg03346706 GNA15 0.054395 0.303004 1.89E-27 0.179519 -5.57043 0 down vs 1 -12.5716 158.046 1.1206 1.50315 1cg13294849 SOX2OT -0.23113 0.342115 1.98E-27 -0.675591 -1.48019 0 down vs 1 -12.5655 157.892 5.95798 7.99974 1cg18621142 0.188905 0.430627 1.98E-27 0.438674 -2.2796 0 down vs 1 -12.5651 157.881 1.05938 1.42251 1cg14658493 HOXA5 0.103685 0.406438 2.17E-27 0.255106 -3.91993 0 down vs 1 -12.5529 157.576 1.66186 2.23584 1cg19953038 0.124195 0.438081 3.01E-27 0.283498 -3.52736 0 down vs 1 -12.5073 156.433 1.78633 2.42086 1cg24691255 SERPINB2 0.118365 0.387046 3.05E-27 0.305816 -3.26994 0 down vs 1 -12.5057 156.391 1.30886 1.77426 1cg21641458 HOXA6 0.04719 0.289366 3.48E-27 0.16308 -6.13195 0 down vs 1 -12.4876 155.94 1.06336 1.44564 1cg24713667 C6orf150 -0.31674 -0.468603 5.74E-27 0.675925 -1.47946 0 down vs 1 12.4184 154.216 0.418138 0.574814 1cg15151929 KIAA1751 0.172215 0.450475 6.99E-27 0.382297 -2.61577 0 down vs 1 -12.3912 153.541 1.40384 1.93834 1cg11457640 -0.130055 0.220499 8.08E-27 -0.589821 -1.69543 0 down vs 1 -12.3711 153.044 2.22806 3.08637 1cg18167759 CACNA2D4 0.046725 0.404532 8.91E-27 0.115504 -8.65772 0 down vs 1 -12.3575 152.707 2.32122 3.2225 1cg25875213 ZNF781 -0.33811 0.116717 9.27E-27 -2.89684 2.89684 0 up vs 1 -12.3521 152.574 3.75068 5.21154 1cg00969405 HOXA5 0.10785 0.391095 9.81E-27 0.275764 -3.62629 0 down vs 1 -12.3442 152.38 1.45459 2.02372 1cg25506432 HOXA5 0.042325 0.352069 1.02E-26 0.120218 -8.31822 0 down vs 1 -12.3384 152.235 1.73949 2.42238 1cg07801181 0.09047 0.327795 1.04E-26 0.275996 -3.62324 0 down vs 1 -12.3364 152.186 1.02118 1.42254 1cg25390165 HOXA5 -0.00195 0.281498 1.46E-26 -0.00692723 -144.358 0 down vs 1 -12.2892 151.024 1.45668 2.04481 1cg03239178 -0.337015 0.199758 1.71E-26 -1.68712 1.68712 0 up vs 1 -12.2673 150.487 5.22395 7.35928 1cg21039708 OTX2OS1 -0.134515 0.345602 1.74E-26 -0.38922 -2.56924 0 down vs 1 -12.2646 150.419 4.17937 5.89039 1cg07760773 LTB4R2 -0.15596 -0.393947 1.83E-26 0.395891 -2.52595 0 down vs 1 12.2579 150.255 1.02689 1.44887 1cg27068650 0.15762 0.397059 1.88E-26 0.396968 -2.51909 0 down vs 1 -12.2543 150.167 1.03946 1.46747 1cg19143990 LYSMD2 -0.24331 -0.432282 1.91E-26 0.56285 -1.77667 0 down vs 1 12.2521 150.114 0.647463 0.914385 1cg14113035 0.00751 0.34829 2.11E-26 0.0215625 -46.3769 0 down vs 1 -12.2385 149.78 2.10556 2.98022 1cg04392488 TP73 0.246875 0.462548 2.21E-26 0.533728 -1.87361 0 down vs 1 -12.232 149.621 0.843357 1.19496 1cg17130982 -0.024225 0.41988 2.23E-26 -0.057695 -17.3325 0 down vs 1 -12.2307 149.59 3.57594 5.06785 1cg20553577 COL4A2 0.08718 0.434339 2.31E-26 0.200719 -4.9821 0 down vs 1 -12.2256 149.466 2.18512 3.09935 1cg10021724 0.084395 0.368197 2.33E-26 0.229211 -4.36279 0 down vs 1 -12.2247 149.443 1.46033 2.07163 1cg18716164 VSTM2B -0.30608 0.10655 2.33E-26 -2.87264 2.87264 0 up vs 1 -12.2243 149.434 3.08702 4.37951 1cg11435943 SERPINB12 -0.01503 0.310604 2.40E-26 -0.0483896 -20.6656 0 down vs 1 -12.2204 149.339 1.92255 2.72923 1cg12005122 0.263295 0.415377 2.57E-26 0.633869 -1.57761 0 down vs 1 -12.2107 149.102 0.419349 0.596249 1cg00988806 EPB41 0.19947 0.405537 2.70E-26 0.491867 -2.03307 0 down vs 1 -12.2039 148.934 0.769898 1.09591 1cg24705158 COL23A1 0.0859 0.381942 2.88E-26 0.224903 -4.44636 0 down vs 1 -12.1951 148.72 1.58901 2.26512 1cg25690715 9-Sep -0.14028 -0.42961 2.99E-26 0.326528 -3.06252 0 down vs 1 12.1897 148.589 1.51777 2.16549 1cg06123940 RNF125 0.14625 0.391197 3.04E-26 0.373852 -2.67485 0 down vs 1 -12.1878 148.542 1.08784 1.55257 1cg15929771 VPS13D 0.137105 0.416337 3.64E-26 0.329312 -3.03663 0 down vs 1 -12.1624 147.924 1.41367 2.02603 1cg00005215 SERPINB12 0.03778 0.387805 5.38E-26 0.0974202 -10.2648 0 down vs 1 -12.1084 146.614 2.22134 3.212 1cg13784312 RAPGEF1 0.088085 -0.386575 5.64E-26 -0.22786 -4.38866 0 down vs 1 12.1019 146.456 4.08492 5.91307 1cg05689208 0.01261 0.431632 5.73E-26 0.0292147 -34.2293 0 down vs 1 -12.0997 146.404 3.1834 4.60972 1cg03302088 GNA15 0.016845 0.265208 6.22E-26 0.0635162 -15.744 0 down vs 1 -12.0884 146.128 1.11838 1.62253 1cg01004682 0.041415 0.357885 6.67E-26 0.115722 -8.64142 0 down vs 1 -12.0785 145.891 1.81586 2.6387 1cg15479387 LOC285830 0.14365 0.459033 6.96E-26 0.31294 -3.1955 0 down vs 1 -12.0728 145.751 1.80341 2.62311 1cg01800614 IFITM4P 0.15375 0.415598 7.05E-26 0.369948 -2.70308 0 down vs 1 -12.0709 145.707 1.24313 1.80873 1cg20247911 MYT1L -0.165905 0.267615 7.46E-26 -0.619938 -1.61306 0 down vs 1 -12.0631 145.518 3.40751 4.96428 1cg14600384 0.02628 0.321788 7.47E-26 0.0816688 -12.2446 0 down vs 1 -12.0629 145.514 1.58327 2.30667 1cg09343092 HOXA6 0.136135 0.376584 7.72E-26 0.3615 -2.76625 0 down vs 1 -12.0583 145.403 1.04824 1.52836 1cg02039276 IFITM4P 0.13629 0.418774 7.77E-26 0.32545 -3.07267 0 down vs 1 -12.0574 145.38 1.44679 2.10978 1cg03207666 HOXA5 0.123245 0.408081 8.19E-26 0.302011 -3.31114 0 down vs 1 -12.0501 145.206 1.47098 2.14763 1cg09527362 C6orf150 -0.26533 -0.41518 8.32E-26 0.639072 -1.56477 0 down vs 1 12.0478 145.15 0.407128 0.594636 1cg11267480 SRRM3 0.16037 0.468808 8.79E-26 0.34208 -2.92329 0 down vs 1 -12.0402 144.967 1.72486 2.52245 1cg23017574 TAS2R41 0.05238 0.342456 9.17E-26 0.152954 -6.53791 0 down vs 1 -12.0344 144.826 1.5256 2.23321 1cg00341950 BRSK2 0.23236 0.444473 1.06E-25 0.522776 -1.91286 0 down vs 1 -12.0145 144.349 0.815743 1.19805 1cg13589431 0.199425 0.388453 1.18E-25 0.513383 -1.94786 0 down vs 1 -11.9991 143.979 0.647841 0.953905 1cg04784475 ZNF781 -0.34555 0.0216098 1.24E-25 -15.9904 15.9904 0 up vs 1 -11.992 143.808 2.44415 3.60314 1cg10865238 GRIP1 -0.019145 0.354773 1.25E-25 -0.0539641 -18.5308 0 down vs 1 -11.9908 143.779 2.53495 3.73776 1cg25879102 -0.393805 0.0542119 1.29E-25 -7.26419 7.26419 0 up vs 1 -11.9872 143.694 3.63921 5.36913 1cg19643053 HOXA5 0.08871 0.397908 1.40E-25 0.222941 -4.48549 0 down vs 1 -11.9753 143.407 1.73337 2.56245 1cg17789809 SOX2OT 0.02614 0.384271 1.53E-25 0.068025 -14.7005 0 down vs 1 -11.9636 143.127 2.32542 3.44442 1cg10410142 GRIA2 -0.32658 0.144144 1.56E-25 -2.26565 2.26565 0 up vs 1 -11.9606 143.055 4.01745 5.95365 1cg01389761 TERC -0.224865 -0.438615 1.61E-25 0.512671 -1.95057 0 down vs 1 11.9565 142.959 0.828381 1.22844 1cg10087036 ITPRIPL2 -0.12566 -0.431358 1.62E-25 0.291313 -3.43274 0 down vs 1 11.9552 142.927 1.69435 2.51319 1cg20007021 LTB4R2 -0.04408 -0.401817 1.66E-25 0.109702 -9.11563 0 down vs 1 11.9521 142.852 2.32031 3.44346 1cg16792800 GPC6 -0.26654 0.169711 1.69E-25 -1.57055 1.57055 0 up vs 1 -11.9495 142.79 3.45057 5.12304 1cg06782035 11-Mar -0.222795 0.239328 1.71E-25 -0.930918 -1.07421 0 down vs 1 -11.9479 142.752 3.87199 5.75025 1cg07049592 HOXA5 0.06647 0.293416 2.27E-25 0.226538 -4.41426 0 down vs 1 -11.9084 141.811 0.933819 1.39601 1cg09313482 -0.107475 0.325996 2.37E-25 -0.329682 -3.03323 0 down vs 1 -11.9024 141.666 3.40673 5.09809 1cg00463577 C6orf150 -0.34181 -0.462886 2.58E-25 0.738433 -1.35422 0 down vs 1 11.8903 141.38 0.265785 0.398545 1cg06336984 0.195395 0.424843 2.59E-25 0.459923 -2.17428 0 down vs 1 -11.8899 141.369 0.954521 1.43142 1cg07636952 CUZD1 0.173365 0.406016 2.69E-25 0.42699 -2.34197 0 down vs 1 -11.8844 141.24 0.981363 1.47302 1cg19196335 HOXA5 0.193525 0.421484 2.72E-25 0.459151 -2.17793 0 down vs 1 -11.883 141.206 0.942175 1.41453 1cg06130360 DOCK11 0.00152 0.401763 2.82E-25 0.00378332 -264.318 0 down vs 1 -11.8782 141.092 2.90447 4.36414 1cg05213379 -0.120375 0.275145 3.00E-25 -0.437497 -2.28573 0 down vs 1 -11.8695 140.884 2.83632 4.26804 1cg20631820 -0.08241 0.399877 3.62E-25 -0.206088 -4.85229 0 down vs 1 -11.8432 140.261 4.21725 6.37425 1cg12140755 -0.04065 0.238248 3.83E-25 -0.170621 -5.86096 0 down vs 1 -11.8354 140.076 1.41029 2.13442 1cg16363586 BST2 0.082745 -0.313616 4.07E-25 -0.263841 -3.79016 0 down vs 1 11.8271 139.88 2.8484 4.31699 1cg27310831 -0.03168 0.332031 4.10E-25 -0.0954128 -10.4808 0 down vs 1 -11.8259 139.852 2.39845 3.63579 1cg01323381 HOXA5 0.05398 0.381598 4.51E-25 0.141458 -7.06926 0 down vs 1 -11.8127 139.54 1.94605 2.95659 1cg22721334 ZSCAN18 -0.319955 0.0664722 4.52E-25 -4.81337 4.81337 0 up vs 1 -11.8124 139.532 2.70741 4.11353 1cg15123742 CSGALNACT1 0.12462 0.384529 4.58E-25 0.324085 -3.08561 0 down vs 1 -11.8105 139.488 1.22479 1.86148 1cg26477613 0.00636 0.283974 4.94E-25 0.0223964 -44.65 0 down vs 1 -11.8001 139.242 1.39733 2.12748 1cg12226948 FMNL2 0.07745 0.37004 5.36E-25 0.209302 -4.77779 0 down vs 1 -11.7885 138.969 1.55216 2.36785 1cg03568017 9-Sep -0.202815 -0.42155 5.50E-25 0.481117 -2.0785 0 down vs 1 11.7851 138.888 0.86747 1.32411 1cg17839399 ETNK1 0.03673 0.392876 5.59E-25 0.0934901 -10.6963 0 down vs 1 -11.7828 138.834 2.29971 3.51167 1cg23723410 -0.17828 -0.432163 6.22E-25 0.412529 -2.42407 0 down vs 1 11.7679 138.483 1.16866 1.78907 1cg00409917 HCG4P6 -0.135105 -0.469209 6.47E-25 0.287942 -3.47292 0 down vs 1 11.8097 139.468 2.01784 2.98042 1cg03283421 FAM24B 0.137955 0.408883 6.67E-25 0.337395 -2.96389 0 down vs 1 -11.7581 138.252 1.33084 2.04075 1cg07101841 9-Sep -0.165315 -0.452446 7.19E-25 0.36538 -2.73687 0 down vs 1 11.7476 138.005 1.49479 2.29625 1cg02091168 0.06161 0.31848 7.83E-25 0.19345 -5.16929 0 down vs 1 -11.7357 137.726 1.19631 1.84147 1cg15018934 HLA-F 0.234305 0.426655 8.12E-25 0.549168 -1.82094 0 down vs 1 -11.7307 137.61 0.670812 1.03344 1cg22226804 FLJ43860 0.108385 0.316575 8.25E-25 0.342368 -2.92084 0 down vs 1 -11.7285 137.558 0.785844 1.21111 1cg13750214 0.205785 0.46806 8.26E-25 0.439655 -2.27451 0 down vs 1 -11.7282 137.551 1.24719 1.92221 1cg13369999 LOC285830 0.055335 0.270095 8.45E-25 0.204872 -4.88109 0 down vs 1 -11.7252 137.479 0.836227 1.2895 1cg06271630 -0.050725 0.37007 8.92E-25 -0.137069 -7.29561 0 down vs 1 -11.7176 137.301 3.21039 4.95701 1cg26609631 GSX1 -0.24282 0.11159 1.09E-24 -2.17601 2.17601 0 up vs 1 -11.6893 136.639 2.27735 3.53338 1cg14280171 STRAP 0.07852 0.375241 1.32E-24 0.209252 -4.77893 0 down vs 1 -11.6629 136.023 1.5963 2.48793 1cg26641996 AGAP1 0.27314 0.42248 1.47E-24 0.646516 -1.54675 0 down vs 1 -11.6482 135.68 0.404361 0.631814 1cg20000088 FBLN2 0.150585 0.418054 1.69E-24 0.360205 -2.7762 0 down vs 1 -11.6281 135.214 1.29707 2.03366 1cg15494117 TERC -0.19538 -0.476074 1.73E-24 0.410398 -2.43666 0 down vs 1 11.625 135.14 1.42852 2.24097 1cg05283521 -0.039635 0.397566 1.81E-24 -0.099694 -10.0307 0 down vs 1 -11.6188 134.997 3.46562 5.44242 1cg24694549 GRIP1 -0.07981 0.333392 1.85E-24 -0.239388 -4.17732 0 down vs 1 -11.616 134.931 3.09559 4.86371 1cg15903956 0.056715 -0.251822 1.85E-24 -0.225219 -4.44012 0 down vs 1 11.6158 134.927 1.72596 2.71186 1cg13530474 PTDSS1 0.23775 0.41837 1.85E-24 0.568277 -1.75971 0 down vs 1 -11.6153 134.914 0.591494 0.929453 1cg07532183 0.000235 0.440626 1.88E-24 0.000533332 -1875 0 down vs 1 -11.613 134.862 3.51637 5.52767 1cg07046030 0.12549 0.428869 1.92E-24 0.292607 -3.41755 0 down vs 1 -11.6104 134.802 1.66874 2.62438 1cg15991405 HOXD9 -0.04439 0.32955 1.97E-24 -0.134699 -7.42397 0 down vs 1 -11.6066 134.714 2.53526 3.98975 1cg02633924 9-Sep -0.22864 -0.447812 2.04E-24 0.510571 -1.95859 0 down vs 1 11.6019 134.603 0.870943 1.37174 1cg17714703 UHRF1 -0.08917 -0.431017 2.36E-24 0.206883 -4.83365 0 down vs 1 11.5815 134.131 2.11876 3.34879 1cg24962180 -0.01207 0.343083 2.54E-24 -0.035181 -28.4244 0 down vs 1 -11.5713 133.894 2.28691 3.62095 1cg03587042 CACNB2 0.08789 0.330685 2.64E-24 0.265782 -3.76249 0 down vs 1 -11.5657 133.765 1.0688 1.69391 1cg12610239 0.203465 0.401125 2.75E-24 0.507236 -1.97147 0 down vs 1 -11.56 133.633 0.708361 1.12376 1cg22335882 SLC45A4 0.18579 0.361105 2.92E-24 0.514505 -1.94362 0 down vs 1 -11.5519 133.446 0.557255 0.885289 1cg16970604 0.14246 0.360324 3.07E-24 0.395367 -2.5293 0 down vs 1 -11.5448 133.282 0.860573 1.36884 1cg10683503 0.306965 0.447576 3.21E-24 0.685838 -1.45807 0 down vs 1 -11.5383 133.132 0.358475 0.570837 1cg09851951 -0.250585 0.147837 3.23E-24 -1.69501 1.69501 0 up vs 1 -11.5376 133.116 2.87809 4.58364 1cg26643142 -0.082745 0.265294 3.24E-24 -0.311899 -3.20617 0 down vs 1 -11.5373 133.109 2.19621 3.49786 1cg11592503 CNTNAP2 -0.213565 0.160567 3.27E-24 -1.33007 1.33007 0 up vs 1 -11.5358 133.074 2.53786 4.04305 1cg25416067 9-Sep -0.04281 -0.43238 3.29E-24 0.09901 -10.1 0 down vs 1 11.5349 133.055 2.75163 4.38426 1cg25535999 NR3C1 0.074085 0.371676 3.33E-24 0.199327 -5.01688 0 down vs 1 -11.5332 133.015 1.60567 2.55913 1cg20026524 PDE10A 0.24141 0.419751 3.46E-24 0.575127 -1.73875 0 down vs 1 -11.5282 132.898 0.576658 0.919886 1cg09743615 0.051805 0.335638 4.06E-24 0.154348 -6.47887 0 down vs 1 -11.5057 132.381 1.46064 2.33912 1cg15599946 TERC -0.250795 -0.477545 4.25E-24 0.525175 -1.90413 0 down vs 1 11.499 132.227 0.93221 1.49461 1cg13674662 SYT3 0.0957 0.325701 4.34E-24 0.293828 -3.40335 0 down vs 1 -11.4962 132.162 0.95913 1.53853 1cg04093645 FBN2 0.144555 0.399646 4.39E-24 0.361707 -2.76467 0 down vs 1 -11.4945 132.124 1.1798 1.89306 1cg25148589 GRIA2 -0.259165 0.132496 4.42E-24 -1.95602 1.95602 0 up vs 1 -11.4938 132.107 2.78125 4.46325 1cg26703456 KIAA1486 -0.20875 0.232441 4.48E-24 -0.898078 -1.11349 0 down vs 1 -11.4917 132.06 3.52915 5.66547 1cg20839041 TERC -0.237825 -0.465423 4.57E-24 0.510987 -1.957 0 down vs 1 11.489 131.997 0.939195 1.50844 1cg04670857 CTNNA2 -0.2902 0.121492 4.72E-24 -2.38864 2.38864 0 up vs 1 -11.4846 131.895 3.073 4.93935 1cg02940165 -0.30776 0.107998 4.73E-24 -2.84967 2.84967 0 up vs 1 -11.4841 131.885 3.13401 5.03778 1cg17152524 -0.035185 0.295401 4.77E-24 -0.119109 -8.39565 0 down vs 1 -11.4829 131.858 1.98147 3.18579 1cg21354912 SERPINB7 0.07763 0.383742 4.85E-24 0.202298 -4.94321 0 down vs 1 -11.4808 131.808 1.69894 2.73258 1cg12510614 PTPRN2 0.04094 0.357637 4.92E-24 0.114474 -8.73564 0 down vs 1 -11.4786 131.758 1.81847 2.92595 1cg25426743 HOXA3 0.21089 0.436438 6.20E-24 0.483208 -2.0695 0 down vs 1 -11.4462 131.015 0.922347 1.49248 1cg24713204 ZNF471 -0.26286 0.0794742 6.22E-24 -3.30749 3.30749 0 up vs 1 -11.4458 131.006 2.1248 3.43845 1cg04140525 0.092265 0.295043 6.23E-24 0.312717 -3.19778 0 down vs 1 -11.4455 130.999 0.745523 1.20651 1cg22605290 0.10828 0.439679 6.35E-24 0.24627 -4.06058 0 down vs 1 -11.4429 130.939 1.99123 3.22394 1cg16236157 THADA 0.078635 0.411253 7.85E-24 0.191208 -5.2299 0 down vs 1 -11.4131 130.259 2.0059 3.26465 1cg01758512 FUT9 -0.1953 0.073518 8.99E-24 -2.65649 2.65649 0 up vs 1 -11.3941 129.825 1.31019 2.1395 1cg26748583 0.192715 0.419462 9.12E-24 0.459434 -2.17659 0 down vs 1 -11.392 129.777 0.932182 1.52278 1cg05560575 0.167615 0.447699 9.19E-24 0.374392 -2.671 0 down vs 1 -11.3909 129.752 1.42231 2.32389 1cg09767658 RERE 0.16785 0.397152 9.88E-24 0.422634 -2.36611 0 down vs 1 -11.3807 129.521 0.953309 1.56037 1cg11970204 BTRC 0.10061 0.387922 1.01E-23 0.259356 -3.8557 0 down vs 1 -11.3775 129.447 1.49667 2.45114 1cg05708114 ARID3C -0.09148 0.29402 1.09E-23 -0.311135 -3.21404 0 down vs 1 -11.3669 129.206 2.69443 4.42101 1cg11564377 0.00465 0.309283 1.09E-23 0.0150348 -66.5125 0 down vs 1 -11.3664 129.196 1.68256 2.76095 1cg23464284 RPS6KA2 0.104925 0.358443 1.11E-23 0.292724 -3.41619 0 down vs 1 -11.3639 129.139 1.1653 1.913 1cg08462306 BTBD19 -0.31663 -0.470645 1.13E-23 0.672757 -1.48642 0 down vs 1 11.3619 129.093 0.430077 0.706282 1cg17807172 -0.150425 0.314207 1.20E-23 -0.478745 -2.0888 0 down vs 1 -11.353 128.891 3.91414 6.43798 1cg14479463 ELAVL2 0.187635 0.409227 1.22E-23 0.458511 -2.18097 0 down vs 1 -11.3515 128.857 0.890277 1.46472 1cg00910924 PDE10A 0.13709 0.406649 1.26E-23 0.337121 -2.9663 0 down vs 1 -11.347 128.753 1.31743 2.16922 1cg01535698 FMN2 -0.364365 0.0324392 1.28E-23 -11.2323 11.2323 0 up vs 1 -11.3448 128.705 2.85477 4.70231 1cg21938845 SRRM3 0.30681 0.48522 1.30E-23 0.632311 -1.5815 0 down vs 1 -11.342 128.64 0.577108 0.95108 1cg16101636 LOC285830 0.12625 0.391227 1.43E-23 0.322702 -3.09883 0 down vs 1 -11.3284 128.332 1.27302 2.10298 1cg05957504 ZNF608 0.137895 0.389201 1.45E-23 0.354303 -2.82244 0 down vs 1 -11.3268 128.297 1.14504 1.89209 1cg11043002 COL23A1 0.006405 0.340481 1.57E-23 0.0188116 -53.1587 0 down vs 1 -11.3159 128.05 2.02353 3.35016 1cg00034559 TMEM178 -0.04588 0.3033 1.57E-23 -0.151269 -6.61072 0 down vs 1 -11.3158 128.048 2.21063 3.65998 1cg20640246 0.07779 0.347359 1.57E-23 0.223947 -4.46535 0 down vs 1 -11.3154 128.038 1.31752 2.1815 1cg18238973 MAPK10 0.162665 0.381894 1.68E-23 0.425943 -2.34773 0 down vs 1 -11.3063 127.833 0.871391 1.44513 1cg15628633 LOC285830 0.10954 0.394964 1.70E-23 0.277342 -3.60566 0 down vs 1 -11.3047 127.797 1.47706 2.45026 1cg16403725 DNAH5 0.04363 0.356406 2.03E-23 0.122417 -8.16883 0 down vs 1 -11.2794 127.225 1.77372 2.95563 1cg16536855 HK1 0.14693 0.368113 2.07E-23 0.399144 -2.50536 0 down vs 1 -11.2769 127.168 0.886995 1.4787 1cg26480091 0.063585 0.325735 2.44E-23 0.195205 -5.12282 0 down vs 1 -11.2535 126.642 1.24599 2.08581 1cg12090003 BST2 -0.09656 -0.393325 2.45E-23 0.245497 -4.07337 0 down vs 1 11.2529 126.627 1.59677 2.67332 1cg14298020 LOC285830 -0.072795 0.245508 2.54E-23 -0.296507 -3.3726 0 down vs 1 -11.2477 126.512 1.83696 3.07826 1cg17312277 0.072565 0.411808 2.57E-23 0.176211 -5.67503 0 down vs 1 -11.2461 126.475 2.08661 3.49761 1cg03251356 RREB1 0.16459 0.382597 2.67E-23 0.430192 -2.32455 0 down vs 1 -11.2409 126.358 0.861705 1.44574 1cg18668836 0.101765 0.415166 2.69E-23 0.245119 -4.07965 0 down vs 1 -11.2397 126.331 1.78081 2.98843 1cg15966877 UNC13A 0.219395 0.473836 2.70E-23 0.463019 -2.15974 0 down vs 1 -11.2392 126.319 1.1738 1.96996 1cg01387797 UCN3 0.027265 0.368699 2.75E-23 0.0739492 -13.5228 0 down vs 1 -11.2367 126.264 2.11364 3.54885 1cg06068085 FBN2 0.12306 0.39834 2.79E-23 0.308932 -3.23696 0 down vs 1 -11.2348 126.222 1.37394 2.30765 1cg22122715 CLDN10 0.16382 0.382858 2.84E-23 0.427887 -2.33706 0 down vs 1 -11.232 126.157 0.869873 1.46177 1cg09663145 0.0224 0.314253 2.87E-23 0.0712802 -14.0291 0 down vs 1 -11.2308 126.132 1.54435 2.59571 1cg08082025 0.12726 0.330418 2.87E-23 0.385148 -2.5964 0 down vs 1 -11.2307 126.128 0.748318 1.2578 1cg13398192 FAM155A -0.250035 0.162645 2.91E-23 -1.53731 1.53731 0 up vs 1 -11.2288 126.087 3.08777 5.19171 1cg27241909 LOC285830 0.015135 0.276868 2.92E-23 0.054665 -18.2932 0 down vs 1 -11.2282 126.072 1.24204 2.08858 1cg24053664 GLB1L2 -0.04255 0.335218 3.00E-23 -0.126932 -7.87821 0 down vs 1 -11.2246 125.991 2.58743 4.35375 1cg18423935 STXBP5L -0.154845 0.271572 3.05E-23 -0.570181 -1.75383 0 down vs 1 -11.2224 125.942 3.29675 5.54949 1cg14449180 HCG4P6 -0.069325 -0.462283 3.09E-23 0.149962 -6.66834 0 down vs 1 11.269 126.99 2.78993 4.5038 1cg24389054 HOXA7 0.13596 0.392806 3.09E-23 0.346125 -2.88913 0 down vs 1 -11.2201 125.892 1.19609 2.0142 1cg00394718 0.06435 0.443666 3.30E-23 0.145041 -6.89458 0 down vs 1 -11.2111 125.69 2.60868 4.40005 1cg12160258 TMEM26 -0.26585 0.170576 3.34E-23 -1.55854 1.55854 0 up vs 1 -11.2094 125.65 3.45334 5.82659 1cg14184817 EXOC3L2 0.042225 0.397507 3.35E-23 0.106225 -9.41401 0 down vs 1 -11.2089 125.64 2.28857 3.86163 1cg16026813 BTRC 0.083335 0.407931 3.38E-23 0.204287 -4.89507 0 down vs 1 -11.2078 125.615 1.91031 3.22402 1cg12709372 0.05631 0.378932 3.38E-23 0.148602 -6.7294 0 down vs 1 -11.2075 125.608 1.88715 3.18511 1cg02732915 PRDM8 0.15746 0.417366 3.46E-23 0.37727 -2.65062 0 down vs 1 -11.2043 125.535 1.22476 2.06834 1cg25720793 F11R -0.197835 -0.442623 3.47E-23 0.446961 -2.23733 0 down vs 1 11.2038 125.526 1.08642 1.83485 1cg21126583 CNTNAP2 -0.188965 0.196744 3.50E-23 -0.960462 -1.04117 0 down vs 1 -11.2027 125.501 2.69735 4.55643 1cg05528931 RFTN1 0.271 0.438492 3.50E-23 0.618028 -1.61805 0 down vs 1 -11.2027 125.499 0.508633 0.859209 1cg19705859 ZFR2 0.209825 0.417158 3.58E-23 0.502987 -1.98812 0 down vs 1 -11.1997 125.434 0.779392 1.31727 1cg24361385 HOXA3 0.04684 0.291688 3.68E-23 0.160582 -6.22733 0 down vs 1 -11.1958 125.346 1.08696 1.83839 1cg07750560 -0.267665 0.0640619 3.70E-23 -4.17823 4.17823 0 up vs 1 -11.1948 125.324 1.99517 3.37505 1cg15496335 -0.276855 0.101388 3.99E-23 -2.73064 2.73064 0 up vs 1 -11.1844 125.091 2.59394 4.39611 1cg05076697 0.16981 0.442136 4.01E-23 0.384067 -2.60371 0 down vs 1 -11.1834 125.069 1.34461 2.2792 1cg15924102 0.208105 0.41731 4.08E-23 0.498682 -2.00529 0 down vs 1 -11.1811 125.016 0.79353 1.34566 1cg06542806 0.146875 0.356801 4.23E-23 0.411645 -2.42928 0 down vs 1 -11.1761 124.906 0.799003 1.35613 1cg15747712 SERPINB2 0.01983 0.287676 4.46E-23 0.0689316 -14.5071 0 down vs 1 -11.1685 124.736 1.30074 2.21071 1cg22016094 HLA-DOB 0.139 0.410498 4.65E-23 0.338613 -2.95323 0 down vs 1 -11.1626 124.604 1.33645 2.27382 1cg14058329 HOXA5 0.121685 0.422854 4.74E-23 0.287771 -3.47499 0 down vs 1 -11.1599 124.544 1.64452 2.79931 1cg14972743 ZSCAN18 -0.266285 0.20384 4.78E-23 -1.30635 1.30635 0 up vs 1 -11.1587 124.517 4.00723 6.82265 1cg06186727 0.140805 0.390941 4.90E-23 0.360169 -2.77647 0 down vs 1 -11.1552 124.437 1.13441 1.93266 1cg11296224 0.024255 0.3609 5.01E-23 0.067207 -14.8794 0 down vs 1 -11.1523 124.373 2.05477 3.50246 1cg13392078 RECK -0.06432 0.382236 5.08E-23 -0.168273 -5.94272 0 down vs 1 -11.1501 124.325 3.61551 6.16519 1cg23346462 PEX5L -0.28858 0.128959 5.09E-23 -2.23777 2.23777 0 up vs 1 -11.1498 124.318 3.16091 5.39028 1cg07858905 IKZF1 0.17739 0.428098 5.19E-23 0.414368 -2.41331 0 down vs 1 -11.1471 124.257 1.1396 1.94432 1cg02344833 ZSCAN1 -0.22945 0.15083 5.55E-23 -1.52125 1.52125 0 up vs 1 -11.1376 124.047 2.62195 4.481 1cg12864235 CDH9 -0.03919 0.341977 5.63E-23 -0.114598 -8.72612 0 down vs 1 -11.1357 124.004 2.6342 4.50347 1cg01994486 0.033315 0.341888 5.73E-23 0.0974443 -10.2623 0 down vs 1 -11.1332 123.947 1.72637 2.95278 1cg11239749 HLA-DOB 0.057005 0.381825 6.00E-23 0.149296 -6.69809 0 down vs 1 -11.1266 123.802 1.91295 3.27574 1cg10132208 ZSCAN1 -0.151845 0.112764 6.03E-23 -1.34657 1.34657 0 up vs 1 -11.126 123.787 1.26948 2.17414 1cg23036508 TERC -0.188035 -0.417215 6.36E-23 0.45069 -2.21882 0 down vs 1 11.1185 123.62 0.952299 1.63312 1cg25784220 ZSCAN18 -0.317495 0.11788 6.83E-23 -2.69337 2.69337 0 up vs 1 -11.1084 123.396 3.43673 5.90447 1cg04217498 0.24016 0.417741 6.87E-23 0.574902 -1.73943 0 down vs 1 -11.1075 123.378 0.571754 0.982447 1cg18023674 PLCH2 -0.0515 0.256223 7.09E-23 -0.200997 -4.9752 0 down vs 1 -11.1031 123.278 1.71687 2.95249 1cg09322003 0.08982 0.442632 7.14E-23 0.202923 -4.92799 0 down vs 1 -11.1021 123.256 2.25686 3.88178 1cg12357534 -0.05297 0.326734 7.20E-23 -0.16212 -6.16828 0 down vs 1 -11.1008 123.229 2.61402 4.4971 1cg25880537 -0.211265 0.294378 7.44E-23 -0.717665 -1.39341 0 down vs 1 -11.0962 123.127 4.63561 7.9816 1cg26627760 0.124 0.366707 7.45E-23 0.338144 -2.95732 0 down vs 1 -11.0962 123.125 1.06803 1.83896 1cg13556452 0.02425 0.368407 7.57E-23 0.0658239 -15.1921 0 down vs 1 -11.0938 123.072 2.14749 3.6992 1cg10827139 SORCS2 0.19936 0.429572 7.86E-23 0.46409 -2.15476 0 down vs 1 -11.0886 122.957 0.960892 1.65676 1cg27586249 CLDN10 0.183285 0.369262 7.86E-23 0.496354 -2.01469 0 down vs 1 -11.0886 122.956 0.627102 1.08124 1cg26252167 GPR6 -0.277335 0.0747701 7.88E-23 -3.70917 3.70917 0 up vs 1 -11.0881 122.946 2.24783 3.876 1cg09844094 0.07189 0.391435 8.02E-23 0.183658 -5.44491 0 down vs 1 -11.0857 122.892 1.85132 3.19369 1cg20817131 HOXA5 0.001795 0.338263 8.17E-23 0.00530652 -188.447 0 down vs 1 -11.0831 122.836 2.0526 3.54256 1cg19707040 CTNNA2 -0.35623 0.00905567 8.19E-23 -39.3378 39.3378 0 up vs 1 -11.0826 122.825 2.41926 4.17573 1cg17057098 ACAN 0.30829 0.478743 8.47E-23 0.643957 -1.5529 0 down vs 1 -11.078 122.722 0.526776 0.909997 1cg26854588 0.07414 -0.298567 9.71E-23 -0.248319 -4.02707 0 down vs 1 11.0586 122.293 2.51856 4.36603 1cg00362680 GLI3 0.172955 0.407876 1.01E-22 0.424038 -2.35828 0 down vs 1 -11.0528 122.164 1.0006 1.73641 1cg16997642 HOXA5 0.13969 0.415992 1.02E-22 0.3358 -2.97796 0 down vs 1 -11.0511 122.127 1.38416 2.40276 1cg10429573 0.07473 0.428854 1.04E-22 0.174255 -5.73871 0 down vs 1 -11.0489 122.079 2.27368 3.94843 1cg04800142 -0.065575 0.318758 1.05E-22 -0.205721 -4.86096 0 down vs 1 -11.0475 122.046 2.67814 4.65204 1cg00074145 ONECUT1 -0.210035 0.280312 1.10E-22 -0.749289 -1.3346 0 down vs 1 -11.0416 121.917 4.35939 7.58048 1cg16584604 0.04885 0.378239 1.10E-22 0.129151 -7.74286 0 down vs 1 -11.0409 121.902 1.96714 3.42107 1cg13055709 0.13744 0.390763 1.21E-22 0.351722 -2.84315 0 down vs 1 -11.0281 121.618 1.1635 2.02817 1cg06242102 PTPRN2 0.105935 0.430251 1.22E-22 0.246217 -4.06146 0 down vs 1 -11.0259 121.57 1.90702 3.32556 1cg14100362 0.087125 0.376571 1.31E-22 0.231364 -4.32219 0 down vs 1 -11.0161 121.354 1.51898 2.6536 1cg14662750 JARID2 -0.006005 0.30315 1.32E-22 -0.0198087 -50.4829 0 down vs 1 -11.0156 121.344 1.73289 3.02752 1cg09927251 MYT1L 0.14644 0.430178 1.36E-22 0.340417 -2.93757 0 down vs 1 -11.0112 121.247 1.45966 2.55222 1cg18640250 0.014545 0.411383 1.45E-22 0.0353563 -28.2835 0 down vs 1 -11.0023 121.051 2.85525 5.00049 1cg12964065 KCNG2 0.146765 0.333658 1.57E-22 0.439866 -2.27342 0 down vs 1 -10.9909 120.8 0.633294 1.11141 1cg25221625 CRYBB3 0.17653 0.396937 1.70E-22 0.444731 -2.24855 0 down vs 1 -10.9792 120.543 0.880779 1.54904 1cg24522654 -0.07197 -0.319356 1.73E-22 0.22536 -4.43734 0 down vs 1 10.9772 120.498 1.1096 1.95219 1cg09498948 -0.23137 0.198726 1.83E-22 -1.16426 1.16426 0 up vs 1 -10.9686 120.31 3.35389 5.90992 1cg05308317 0.10174 0.397581 1.84E-22 0.255897 -3.90782 0 down vs 1 -10.9681 120.3 1.58685 2.79645 1cg10647703 LOC285830 0.0927 0.372118 1.92E-22 0.249115 -4.01421 0 down vs 1 -10.9618 120.16 1.41555 2.49747 1cg01076387 0.10069 0.391682 1.95E-22 0.257071 -3.88998 0 down vs 1 -10.9601 120.123 1.53525 2.7095 1cg01179256 CHST11 -0.122975 0.183689 2.05E-22 -0.669475 -1.49371 0 down vs 1 -10.9526 119.96 1.70507 3.01331 1cg18405765 ZNF541 0.19113 0.379132 2.06E-22 0.504125 -1.98363 0 down vs 1 -10.9521 119.948 0.64083 1.13263 1cg13772218 -0.21179 0.130697 2.11E-22 -1.62047 1.62047 0 up vs 1 -10.9488 119.876 2.1267 3.76105 1cg07915730 TSPAN9 0.1193 0.31783 2.19E-22 0.375357 -2.66413 0 down vs 1 -10.9436 119.762 0.714615 1.26499 1cg00969162 UMODL1 0.0741 0.427249 2.32E-22 0.173435 -5.76584 0 down vs 1 -10.9353 119.581 2.26117 4.00874 1cg06533314 -0.21676 0.0793093 2.33E-22 -2.7331 2.7331 0 up vs 1 -10.9343 119.56 1.5893 2.81809 1cg24890964 BLCAP 0.00903 -0.277622 2.36E-22 -0.0325262 -30.7444 0 down vs 1 10.933 119.531 1.4898 2.64232 1cg15784580 0.00871 0.394801 2.36E-22 0.0220617 -45.3273 0 down vs 1 -10.9329 119.528 2.7027 4.79363 1cg08995609 RIN1 -0.289445 -0.441435 2.49E-22 0.655691 -1.52511 0 down vs 1 10.925 119.356 0.41884 0.743941 1cg24186459 0.132185 0.353528 2.58E-22 0.373903 -2.67449 0 down vs 1 -10.92 119.247 0.888278 1.5792 1cg19623438 -0.18383 0.216529 2.77E-22 -0.848986 -1.17788 0 down vs 1 -10.91 119.028 2.90614 5.17612 1cg14230647 PXDN -0.024905 0.290162 2.79E-22 -0.0858313 -11.6508 0 down vs 1 -10.9092 119.01 1.7998 3.2061 1cg21491013 KIAA1409 0.03984 0.362427 3.07E-22 0.109925 -9.09707 0 down vs 1 -10.8956 118.714 1.88674 3.36936 1cg22047387 NEUROD4 -0.29716 0.157194 3.08E-22 -1.8904 1.8904 0 up vs 1 -10.8952 118.706 3.74289 6.68452 1cg18651515 ROPN1B -0.18576 0.0729253 3.10E-22 -2.54727 2.54727 0 up vs 1 -10.894 118.68 1.21328 2.16731 1cg22685966 ZNF781 -0.26132 0.176822 3.11E-22 -1.47787 1.47787 0 up vs 1 -10.8936 118.67 3.48055 6.2179 1cg20936172 0.13346 0.38058 3.27E-22 0.350675 -2.85164 0 down vs 1 -10.8864 118.514 1.10722 1.98063 1cg16691821 -0.286045 0.181651 3.37E-22 -1.5747 1.5747 0 up vs 1 -10.8821 118.419 3.96592 7.09999 1cg01271726 0.041955 0.38607 3.48E-22 0.108672 -9.202 0 down vs 1 -10.8778 118.326 2.14697 3.84664 1cg06951326 C7orf34 0.0987 0.343781 3.76E-22 0.287101 -3.48309 0 down vs 1 -10.8667 118.085 1.08902 1.95513 1cg21402886 0.178705 0.415606 3.83E-22 0.429986 -2.32566 0 down vs 1 -10.864 118.026 1.01754 1.82773 1cg24254387 BTBD19 -0.2743 -0.453808 3.86E-22 0.604441 -1.65442 0 down vs 1 10.8631 118.006 0.584231 1.04958 1cg05211662 ADAMTS19 -0.315035 0.0470649 3.94E-22 -6.69362 6.69362 0 up vs 1 -10.8601 117.942 2.37725 4.27308 1cg17527574 TERC -0.35293 -0.478675 3.97E-22 0.737306 -1.35629 0 down vs 1 10.8588 117.915 0.286682 0.51543 1cg07700843 -0.094185 0.319465 3.99E-22 -0.294821 -3.39189 0 down vs 1 -10.8582 117.901 3.10231 5.57833 1cg24221992 VSTM2A 0.15019 0.398394 4.00E-22 0.376988 -2.6526 0 down vs 1 -10.8579 117.894 1.11696 2.00854 1cg25661961 PDGFC 0.2013 0.379822 4.16E-22 0.529986 -1.88684 0 down vs 1 -10.8525 117.776 0.57783 1.04011 1cg17506588 0.003415 0.26704 4.38E-22 0.0127883 -78.1963 0 down vs 1 -10.845 117.613 1.26006 2.27129 1cg01573321 PSD3 -0.047135 0.344975 4.39E-22 -0.136633 -7.31887 0 down vs 1 -10.8447 117.607 2.78762 5.02501 1cg04749740 -0.02257 0.28133 4.65E-22 -0.080226 -12.4648 0 down vs 1 -10.8365 117.43 1.67448 3.023 1cg03560850 CACNA1H 0.086465 0.43548 4.98E-22 0.198551 -5.03649 0 down vs 1 -10.8267 117.216 2.20854 3.99441 1cg17282592 -0.015285 0.374746 5.04E-22 -0.0407876 -24.5173 0 down vs 1 -10.825 117.18 2.75814 4.98997 1cg01908954 SCG3 -0.32783 0.194619 5.13E-22 -1.68447 1.68447 0 up vs 1 -10.8225 117.127 4.94886 8.95742 1cg13080137 PRIM2 0.26476 0.422107 5.36E-22 0.627234 -1.5943 0 down vs 1 -10.8163 116.992 0.448886 0.813424 1cg10378735 EPHB6 0.060945 0.39181 5.43E-22 0.155547 -6.42891 0 down vs 1 -10.8143 116.949 1.98481 3.59797 1cg05985560 0.163925 0.423912 5.68E-22 0.386696 -2.58601 0 down vs 1 -10.8081 116.816 1.22552 2.22411 1cg08117431 ZNRF4 0.21885 0.464936 5.73E-22 0.47071 -2.12445 0 down vs 1 -10.8067 116.786 1.09797 1.99314 1cg26706077 XYLT1 0.0257 0.331416 5.79E-22 0.0775459 -12.8956 0 down vs 1 -10.8052 116.753 1.69456 3.07698 1cg04462650 0.15073 0.448245 5.90E-22 0.336267 -2.97383 0 down vs 1 -10.8025 116.695 1.60486 2.91555 1cg20546778 GDAP1 0.1376 0.414309 5.94E-22 0.332119 -3.01097 0 down vs 1 -10.8016 116.674 1.38824 2.52247 1cg05983315 ZSCAN1 -0.10251 0.284655 6.06E-22 -0.36012 -2.77685 0 down vs 1 -10.7988 116.615 2.71776 4.94074 1cg18729664 FILIP1 -0.088565 0.322355 6.37E-22 -0.274744 -3.63975 0 down vs 1 -10.7917 116.461 3.06148 5.57297 1cg05005367 RBM20 0.165075 0.418934 6.42E-22 0.394036 -2.53784 0 down vs 1 -10.7907 116.439 1.16843 2.12736 1cg21140931 0.00019 0.34027 6.42E-22 0.000558381 -1790.89 0 down vs 1 -10.7905 116.435 2.09691 3.81797 1cg09624466 OTX2OS1 -0.092735 0.295235 6.45E-22 -0.314106 -3.18364 0 down vs 1 -10.79 116.424 2.72907 4.96944 1cg26844909 0.179465 0.420067 6.48E-22 0.427229 -2.34066 0 down vs 1 -10.7893 116.41 1.04958 1.91145 1cg15998779 -0.181855 0.189185 6.53E-22 -0.961257 -1.0403 0 down vs 1 -10.7882 116.386 2.49608 4.54668 1cg16670307 NTM 0.108695 0.389886 6.82E-22 0.278787 -3.58697 0 down vs 1 -10.782 116.251 1.43357 2.61431 1cg00666903 -0.00839 0.181865 6.90E-22 -0.0461331 -21.6764 0 down vs 1 -10.7803 116.214 0.656281 1.1972 1cg26807616 -0.043365 0.326427 7.08E-22 -0.132847 -7.52744 0 down vs 1 -10.7767 116.137 2.47933 4.52583 1cg15849724 FAM83B 0.19664 0.431174 7.49E-22 0.456057 -2.19271 0 down vs 1 -10.7687 115.965 0.997311 1.82323 1cg26113809 -0.21771 0.140877 7.72E-22 -1.54539 1.54539 0 up vs 1 -10.7643 115.871 2.33135 4.26551 1cg14895183 AGAP1 0.228395 0.398902 8.31E-22 0.572559 -1.74654 0 down vs 1 -10.7539 115.646 0.527112 0.966292 1cg10059410 ARID1B 0.209095 0.412322 8.39E-22 0.507116 -1.97194 0 down vs 1 -10.7524 115.614 0.748827 1.37312 1cg11332928 BTBD19 -0.277975 -0.44967 9.60E-22 0.618176 -1.61766 0 down vs 1 10.7332 115.203 0.53448 0.983569 1cg20449685 ZSCAN1 -0.217885 0.062833 9.85E-22 -3.46768 3.46768 0 up vs 1 -10.7296 115.125 1.42876 2.63103 1cg10170949 EHD2 0.15293 0.32967 1.00E-21 0.463889 -2.15569 0 down vs 1 -10.7274 115.077 0.566351 1.04335 1cg01917209 MST1R 0.328005 -0.0360887 1.01E-21 -9.08887 9.08887 0 up vs 1 10.7254 115.034 2.4035 4.42949 1cg02078690 BSX -0.239985 0.20919 1.04E-21 -1.14721 1.14721 0 up vs 1 -10.7222 114.966 3.65804 6.74549 1cg00348762 DKFZP434H168 -0.248455 0.122449 1.04E-21 -2.02905 2.02905 0 up vs 1 -10.7221 114.964 2.49426 4.59956 1cg02207386 -0.1694 0.256349 1.05E-21 -0.660818 -1.51328 0 down vs 1 -10.7199 114.917 3.28644 6.06284 1cg24762932 -0.24594 0.203546 1.13E-21 -1.20827 1.20827 0 up vs 1 -10.7106 114.716 3.66312 6.76957 1cg13694927 HOXA5 0.0696 0.310973 1.20E-21 0.223814 -4.468 0 down vs 1 -10.7019 114.531 1.05632 1.95528 1cg05522011 PRDM8 0.18393 0.444231 1.25E-21 0.414041 -2.41522 0 down vs 1 -10.6959 114.402 1.22849 2.27653 1cg20114151 0.11616 0.461171 1.30E-21 0.251881 -3.97013 0 down vs 1 -10.6902 114.281 2.15816 4.00356 1cg15480287 PNCK 0.212845 0.382591 1.31E-21 0.556325 -1.79751 0 down vs 1 -10.6889 114.252 0.522418 0.969372 1cg10249507 UNC13A 0.17323 0.377692 1.42E-21 0.458654 -2.18029 0 down vs 1 -10.6775 114.009 0.757953 1.40942 1cg07184643 0.234045 0.422734 1.44E-21 0.553647 -1.80621 0 down vs 1 -10.6753 113.963 0.645519 1.20083 1cg12798492 0.182555 0.455642 1.48E-21 0.400655 -2.49591 0 down vs 1 -10.6716 113.883 1.35213 2.51708 1cg18120446 -0.161515 0.211093 1.49E-21 -0.765136 -1.30696 0 down vs 1 -10.6708 113.867 2.51723 4.68665 1cg07075307 0.177485 0.4527 1.51E-21 0.392059 -2.55064 0 down vs 1 -10.6683 113.814 1.37329 2.55802 1cg26349474 0.12363 0.381945 1.51E-21 0.323685 -3.08942 0 down vs 1 -10.6683 113.813 1.20981 2.25352 1cg05749717 CNTNAP2 -0.222845 0.0709716 1.56E-21 -3.13992 3.13992 0 up vs 1 -10.6636 113.712 1.5652 2.91809 1cg08106847 PTPRN2 0.111725 0.366053 1.57E-21 0.305215 -3.27638 0 down vs 1 -10.6633 113.705 1.17275 2.18656 1cg23708361 CNTNAP2 -0.16322 0.222294 1.64E-21 -0.734253 -1.36193 0 down vs 1 -10.657 113.571 2.69462 5.02997 1cg18476766 0.00943 0.388564 1.64E-21 0.0242688 -41.2051 0 down vs 1 -10.6567 113.565 2.60618 4.86516 1cg12161959 -0.005975 0.396558 1.66E-21 -0.0150672 -66.3695 0 down vs 1 -10.6548 113.526 2.93779 5.48609 1cg27020690 -0.16574 -0.420986 1.71E-21 0.393695 -2.54004 0 down vs 1 10.6505 113.433 1.18123 2.20767 1cg02792538 SLC16A8 -0.11677 -0.365849 1.73E-21 0.319175 -3.13307 0 down vs 1 10.6496 113.414 1.12484 2.10263 1cg23421560 FAM20C 0.1387 0.355816 1.89E-21 0.389808 -2.56537 0 down vs 1 -10.6369 113.143 0.85468 1.60144 1cg20127859 C8orf73 -0.002625 -0.249276 1.89E-21 0.0105305 -94.9622 0 down vs 1 10.6367 113.139 1.10302 2.06684 1cg13487156 RFTN1 0.136465 0.321294 1.89E-21 0.424736 -2.3544 0 down vs 1 -10.6366 113.138 0.61938 1.1606 1cg06060522 0.02772 0.37761 1.90E-21 0.0734091 -13.6223 0 down vs 1 -10.6361 113.126 2.21963 4.15961 1cg23968866 0.241875 0.414783 1.90E-21 0.583136 -1.71487 0 down vs 1 -10.6356 113.116 0.542061 1.01592 1cg16588492 0.16275 0.413802 1.98E-21 0.393304 -2.54256 0 down vs 1 -10.6296 112.989 1.14273 2.1441 1cg02056098 SCN9A 0.10815 0.356215 2.01E-21 0.303608 -3.29372 0 down vs 1 -10.6279 112.952 1.11571 2.09408 1cg15788537 -0.091955 0.295126 2.03E-21 -0.311578 -3.20946 0 down vs 1 -10.6263 112.917 2.71658 5.10033 1cg19093890 -0.02776 0.355137 2.04E-21 -0.078167 -12.7931 0 down vs 1 -10.6258 112.907 2.65817 4.9911 1cg08418978 CLDN10 0.121475 0.378523 2.07E-21 0.320919 -3.11605 0 down vs 1 -10.6239 112.866 1.19797 2.25018 1cg07960138 CPLX1 0.051255 0.407095 2.23E-21 0.125904 -7.94254 0 down vs 1 -10.613 112.637 2.29576 4.32099 1cg18703066 0.14441 0.401705 2.25E-21 0.359493 -2.7817 0 down vs 1 -10.6117 112.609 1.20028 2.25966 1cg07753366 0.000515 0.302156 2.27E-21 0.00170442 -586.711 0 down vs 1 -10.6104 112.582 1.64968 3.10647 1cg25474372 -0.306615 0.104794 2.33E-21 -2.92589 2.92589 0 up vs 1 -10.6068 112.505 3.06878 5.78269 1cg13656752 STK10 -0.136245 -0.429455 2.35E-21 0.317251 -3.15208 0 down vs 1 10.6051 112.469 1.55875 2.93818 1cg11124847 TMPRSS11F 0.23419 0.3958 2.38E-21 0.591688 -1.69008 0 down vs 1 -10.6037 112.438 0.473538 0.892847 1cg14521988 PDE4D 0.17219 0.392233 2.49E-21 0.438999 -2.27791 0 down vs 1 -10.5969 112.294 0.877876 1.65734 1cg19516105 -0.325215 0.115884 2.51E-21 -2.8064 2.8064 0 up vs 1 -10.5962 112.279 3.52768 6.66079 1cg25974167 EEFSEC 0.085435 0.31503 2.58E-21 0.271196 -3.68736 0 down vs 1 -10.5922 112.195 0.955746 1.80595 1cg13253153 KCNQ1 0.07209 0.429848 2.76E-21 0.167711 -5.96265 0 down vs 1 -10.5882 112.109 2.31945 4.36544 1cg08250055 TNXB 0.07789 0.358386 2.82E-21 0.217335 -4.60118 0 down vs 1 -10.5791 111.918 1.4265 2.70215 1cg25241559 SNED1 0.12395 0.449004 2.83E-21 0.276055 -3.62246 0 down vs 1 -10.5787 111.909 1.91571 3.62913 1cg18588768 FAM155A -0.28989 0.141092 2.88E-21 -2.05462 2.05462 0 up vs 1 -10.5764 111.86 3.36772 6.38262 1cg06799422 MGA -0.072495 -0.430326 2.90E-21 0.168465 -5.93594 0 down vs 1 10.5755 111.841 2.32152 4.40057 1cg26949731 0.13878 0.378763 2.90E-21 0.366403 -2.72923 0 down vs 1 -10.5753 111.837 1.04419 1.97938 1cg23814002 SCFD2 0.263285 0.438779 2.99E-21 0.60004 -1.66656 0 down vs 1 -10.5711 111.747 0.558399 1.05936 1cg08728302 FAM20C 0.233625 0.429999 3.09E-21 0.543315 -1.84055 0 down vs 1 -10.5661 111.642 0.699175 1.32768 1cg01568736 SERPINB7 0.19745 0.415134 3.10E-21 0.47563 -2.10247 0 down vs 1 -10.5658 111.637 0.85915 1.63154 1cg13914083 GRIA2 -0.33135 0.103773 3.26E-21 -3.19302 3.19302 0 up vs 1 -10.5586 111.484 3.43275 6.5278 1cg13663861 SURF4 0.354965 0.435507 3.32E-21 0.815061 -1.2269 0 down vs 1 -10.5561 111.43 0.117616 0.223768 1cg07654012 -0.276435 0.174952 3.35E-21 -1.58006 1.58006 0 up vs 1 -10.5545 111.397 3.69416 7.03037 1cg02161761 DSCC1 -0.08572 0.253208 3.39E-21 -0.338536 -2.9539 0 down vs 1 -10.5529 111.363 2.08273 3.96485 1cg02118390 RPS6KA2 0.26263 0.473752 3.39E-21 0.554362 -1.80387 0 down vs 1 -10.5529 111.363 0.808133 1.53843 1cg07102397 FOXN3 0.2474 0.400213 3.42E-21 0.61817 -1.61768 0 down vs 1 -10.5517 111.339 0.42339 0.806178 1cg01400302 ZNF311 0.190885 0.410122 3.42E-21 0.465435 -2.14853 0 down vs 1 -10.5516 111.336 0.871454 1.65937 1cg10193721 LTB4R2 -0.0443 -0.37385 3.44E-21 0.118497 -8.43905 0 down vs 1 10.5509 111.322 1.96907 3.74985 1cg18806140 CTNNA2 -0.130105 0.23437 3.48E-21 -0.555127 -1.80139 0 down vs 1 -10.549 111.282 2.40853 4.58844 1cg16020747 RALYL -0.167625 0.174073 3.71E-21 -0.962957 -1.03847 0 down vs 1 -10.54 111.093 2.11691 4.03975 1cg00463957 PARN 0.16455 0.366147 3.73E-21 0.449409 -2.22514 0 down vs 1 -10.5394 111.078 0.736865 1.40636 1cg20758982 C6orf150 -0.318175 -0.437461 3.89E-21 0.727322 -1.37491 0 down vs 1 10.5333 110.951 0.257986 0.492949 1cg19392831 PRLHR -0.18302 0.0853412 3.92E-21 -2.14457 2.14457 0 up vs 1 -10.532 110.924 1.30574 2.49556 1cg26292021 0.03343 0.301903 3.95E-21 0.110731 -9.03089 0 down vs 1 -10.5309 110.901 1.30683 2.49816 1cg17517613 TSPAN9 0.22805 0.420606 3.95E-21 0.542194 -1.84436 0 down vs 1 -10.5309 110.899 0.67225 1.2851 1cg18700744 NAA25 -0.07536 -0.367271 3.95E-21 0.205189 -4.87356 0 down vs 1 10.5308 110.899 1.54497 2.95344 1cg06034711 0.100745 0.375231 3.96E-21 0.268488 -3.72456 0 down vs 1 -10.5305 110.892 1.36602 2.61153 1cg15959363 C4orf32 0.092375 0.370327 3.99E-21 0.249441 -4.00896 0 down vs 1 -10.5295 110.87 1.40074 2.67843 1cg00854560 0.121115 0.36094 4.15E-21 0.335554 -2.98014 0 down vs 1 -10.5238 110.75 1.04282 1.99618 1cg16518145 0.114 0.41788 4.37E-21 0.272806 -3.66561 0 down vs 1 -10.5166 110.598 1.67426 3.2093 1cg26023912 HOXA5 0.024525 0.266832 4.38E-21 0.0919116 -10.88 0 down vs 1 -10.5163 110.592 1.06451 2.04062 1cg04871875 SFRS13B 0.08631 0.308171 4.42E-21 0.280072 -3.57051 0 down vs 1 -10.5148 110.561 0.892439 1.71124 1cg02579509 LOC286002 -0.059355 0.290981 4.47E-21 -0.203982 -4.90238 0 down vs 1 -10.5133 110.53 2.22529 4.26818 1cg00297075 0.00864 0.328044 4.56E-21 0.0263379 -37.968 0 down vs 1 -10.5104 110.468 1.84969 3.54975 1cg01735384 -0.213495 0.256219 4.75E-21 -0.833252 -1.20012 0 down vs 1 -10.5045 110.345 4.00023 7.68542 1cg07577121 -0.00975 0.296775 4.80E-21 -0.0328531 -30.4385 0 down vs 1 -10.5032 110.317 1.70353 3.27374 1cg10555744 MAN1C1 0.22914 0.426678 4.92E-21 0.537033 -1.86208 0 down vs 1 -10.4994 110.238 0.707487 1.36057 1cg02106682 HOXA5 0.00685 0.227743 4.98E-21 0.0300777 -33.2472 0 down vs 1 -10.4977 110.201 0.884674 1.7019 1cg00699993 GRIA2 -0.337475 0.0741098 5.03E-21 -4.55372 4.55372 0 up vs 1 -10.4963 110.173 3.0714 5.91015 1cg02006142 NSMCE2 0.069545 0.365989 5.07E-21 0.19002 -5.26262 0 down vs 1 -10.4953 110.151 1.59332 3.06656 1cg06448705 TRPC7 -0.15475 0.27186 5.19E-21 -0.569226 -1.75677 0 down vs 1 -10.4917 110.076 3.29975 6.35511 1cg01416317 EXOC3L2 0.11382 0.356901 5.24E-21 0.318912 -3.13566 0 down vs 1 -10.4905 110.051 1.07132 2.06378 1cg19780308 MAML3 0.178505 0.460406 5.24E-21 0.387712 -2.57923 0 down vs 1 -10.4904 110.048 1.44082 2.77565 1cg24389585 HOXA5 0.1825 0.397812 5.35E-21 0.458759 -2.17979 0 down vs 1 -10.4875 109.988 0.840535 1.62011 1cg03585764 RASGEF1C -0.0327 0.272498 5.46E-21 -0.120001 -8.33327 0 down vs 1 -10.4845 109.924 1.68881 3.25705 1cg21631754 EXOC3L2 0.01786 0.369566 5.69E-21 0.0483269 -20.6924 0 down vs 1 -10.4786 109.801 2.24274 4.33019 1cg19382705 UST 0.162185 0.417884 5.77E-21 0.38811 -2.57659 0 down vs 1 -10.4765 109.757 1.18543 2.2897 1cg22834443 TCF7L1 0.09804 0.369266 5.90E-21 0.265499 -3.76649 0 down vs 1 -10.4734 109.691 1.33377 2.57778 1cg04340928 0.133635 0.413687 5.92E-21 0.323034 -3.09565 0 down vs 1 -10.473 109.684 1.42199 2.74845 1cg26523264 ADAMTS10 0.043235 0.254763 6.27E-21 0.169707 -5.89252 0 down vs 1 -10.4648 109.512 0.811247 1.57046 1cg20568390 SSBP3 0.30812 0.430171 6.28E-21 0.716273 -1.39612 0 down vs 1 -10.4644 109.504 0.270086 0.522888 1cg21542881 -0.0039 0.330071 6.29E-21 -0.0118156 -84.6336 0 down vs 1 -10.4642 109.5 2.02225 3.91523 1cg01746118 0.26903 0.426899 6.33E-21 0.630196 -1.58681 0 down vs 1 -10.4633 109.48 0.451868 0.875011 1cg00771217 TMPRSS11D 0.242735 0.38252 6.77E-21 0.634568 -1.57588 0 down vs 1 -10.4536 109.279 0.354274 0.687291 1cg01159194 -0.069955 0.386384 6.85E-21 -0.181051 -5.52332 0 down vs 1 -10.4519 109.242 3.77565 7.32717 1cg05243629 -0.08436 0.311687 7.19E-21 -0.270656 -3.69472 0 down vs 1 -10.445 109.098 2.84388 5.52623 1cg14003265 TRAF2 0.009675 -0.280015 7.28E-21 -0.0345517 -28.9422 0 down vs 1 10.4433 109.062 1.52155 2.95766 1cg02695704 CACNA1H 0.214455 0.469147 7.41E-21 0.457117 -2.18762 0 down vs 1 -10.4407 109.009 1.17611 2.28729 1cg01971940 CACNA1H 0.077085 0.355585 7.91E-21 0.216784 -4.61289 0 down vs 1 -10.4314 108.814 1.40626 2.73979 1cg18892424 0.158075 0.458056 7.95E-21 0.3451 -2.89771 0 down vs 1 -10.4306 108.798 1.63157 3.17922 1cg03061623 0.0652 0.314892 7.97E-21 0.207055 -4.82964 0 down vs 1 -10.4357 108.904 1.12984 2.18906 1cg18657303 0.056215 0.366564 8.14E-21 0.153357 -6.52075 0 down vs 1 -10.4273 108.728 1.7463 3.40496 1cg21246991 0.196885 0.417235 8.27E-21 0.47188 -2.11918 0 down vs 1 -10.4249 108.678 0.880327 1.71726 1cg21886367 LTB4R 0.11989 -0.194568 8.59E-21 -0.616187 -1.62288 0 down vs 1 10.4194 108.563 1.79284 3.50102 1cg00467513 TNXB 0.195405 0.370241 8.66E-21 0.527778 -1.89474 0 down vs 1 -10.4182 108.539 0.554218 1.0825 1cg14613365 0.139085 0.399124 8.70E-21 0.348475 -2.86964 0 down vs 1 -10.4176 108.527 1.22601 2.39494 1cg12509442 VSTM2A 0.086095 0.371427 8.70E-21 0.231795 -4.31416 0 down vs 1 -10.4176 108.527 1.47611 2.88349 1cg23658684 0.027995 0.292283 8.86E-21 0.0957805 -10.4405 0 down vs 1 -10.4149 108.471 1.26641 2.47512 1cg02119363 PEX5L -0.260755 0.0416814 8.88E-21 -6.2559 6.2559 0 up vs 1 -10.4147 108.466 1.65839 3.24138 1cg16774084 0.003065 0.350111 8.95E-21 0.00875437 -114.229 0 down vs 1 -10.4135 108.441 2.18369 4.26908 1cg23645302 FLJ41562 0.010665 0.326753 9.04E-21 0.0326393 -30.6379 0 down vs 1 -10.4121 108.413 1.81148 3.54234 1cg02009585 PEX5L -0.25016 0.117463 9.25E-21 -2.12969 2.12969 0 up vs 1 -10.4088 108.343 2.45032 4.79466 1cg02574509 FMN2 -0.343035 0.0308675 9.29E-21 -11.1131 11.1131 0 up vs 1 -10.4082 108.33 2.53475 4.96047 1cg09221960 0.12811 0.427087 9.80E-21 0.299962 -3.33375 0 down vs 1 -10.4005 108.17 1.62067 3.17632 1cg14742802 ABLIM2 0.173525 0.38264 9.98E-21 0.453495 -2.2051 0 down vs 1 -10.3979 108.115 0.792843 1.55466 1cg15358549 FBN2 0.112845 0.390324 1.08E-20 0.289106 -3.45894 0 down vs 1 -10.387 107.89 1.39597 2.74304 1cg19593660 FLJ43860 0.086155 0.361242 1.08E-20 0.238497 -4.19293 0 down vs 1 -10.3869 107.887 1.37201 2.69602 1cg13280741 PDE4D 0.154135 0.363098 1.11E-20 0.424499 -2.35572 0 down vs 1 -10.3829 107.804 0.791696 1.55689 1cg11683252 0.24357 0.390915 1.17E-20 0.623076 -1.60494 0 down vs 1 -10.375 107.641 0.393633 0.775261 1cg02848074 0.02341 0.274446 1.20E-20 0.085299 -11.7235 0 down vs 1 -10.371 107.558 1.14259 2.25209 1cg14234213 OR5K2 0.20143 0.428781 1.22E-20 0.469773 -2.12869 0 down vs 1 -10.3695 107.526 0.937159 1.84771 1cg14222434 PDE10A 0.084605 0.367611 1.22E-20 0.230148 -4.34502 0 down vs 1 -10.369 107.515 1.45214 2.86335 1cg11206526 0.27195 0.427656 1.23E-20 0.635909 -1.57255 0 down vs 1 -10.3675 107.486 0.439569 0.866985 1cg08825645 -0.11161 0.193916 1.26E-20 -0.575557 -1.73745 0 down vs 1 -10.3639 107.411 1.69245 3.34043 1cg03944501 TLN2 0.25773 0.419954 1.28E-20 0.61371 -1.62943 0 down vs 1 -10.3622 107.375 0.477143 0.942067 1cg25576711 LTB4R2 -0.07932 -0.353245 1.28E-20 0.224547 -4.45341 0 down vs 1 10.3621 107.374 1.36044 2.68608 1cg13760252 ZNRF4 0.293865 0.475094 1.29E-20 0.618541 -1.61671 0 down vs 1 -10.3608 107.347 0.595487 1.17603 1cg20411555 -0.01703 0.288224 1.35E-20 -0.059086 -16.9245 0 down vs 1 -10.3539 107.204 1.68943 3.34092 1cg09235562 0.061675 0.398735 1.36E-20 0.154677 -6.4651 0 down vs 1 -10.3533 107.19 2.05984 4.07394 1cg24805559 0.140865 0.37023 1.37E-20 0.380479 -2.62826 0 down vs 1 -10.3527 107.179 0.953836 1.8867 1cg25024515 0.179235 0.419773 1.42E-20 0.426981 -2.34203 0 down vs 1 -10.3466 107.052 1.04903 2.07744 1cg02879438 ATP11A 0.13682 0.440887 1.45E-20 0.310329 -3.22239 0 down vs 1 -10.3442 107.002 1.67632 3.32126 1cg21198219 -0.025065 0.295392 1.49E-20 -0.0848533 -11.785 0 down vs 1 -10.3397 106.91 1.86191 3.69213 1cg14398661 LMX1B -0.09781 0.195818 1.52E-20 -0.499494 -2.00202 0 down vs 1 -10.3372 106.857 1.56319 3.1013 1cg14036402 EFHD2 -0.144805 -0.365738 1.55E-20 0.395925 -2.52573 0 down vs 1 10.3349 106.809 0.884993 1.75658 1cg11405852 KRT72 0.181305 0.417809 1.57E-20 0.433943 -2.30445 0 down vs 1 -10.3328 106.767 1.01413 2.01369 1cg21570709 -0.139935 -0.377104 1.61E-20 0.371078 -2.69485 0 down vs 1 10.3288 106.685 1.01985 2.0266 1cg01743020 MYT1L 0.13238 0.449294 1.66E-20 0.29464 -3.39397 0 down vs 1 -10.3248 106.602 1.82097 3.62135 1cg10880006 PRDM8 0.07718 0.375192 1.66E-20 0.205708 -4.86126 0 down vs 1 -10.3246 106.598 1.61022 3.20239 1cg06823034 LTB4R -0.01876 -0.360615 1.66E-20 0.0520222 -19.2225 0 down vs 1 10.3244 106.593 2.11886 4.21413 1cg08540929 0.07055 0.412713 1.67E-20 0.170942 -5.84994 0 down vs 1 -10.3241 106.587 2.12268 4.22199 1cg12146673 SST -0.365905 0.0656943 1.67E-20 -5.56981 5.56981 0 up vs 1 -10.324 106.586 3.37738 6.71764 1cg23697780 CYP7B1 0.098005 0.371227 1.70E-20 0.264003 -3.78784 0 down vs 1 -10.3213 106.529 1.35347 2.6935 1cg14913111 HPDL -0.253185 -0.461022 1.79E-20 0.549182 -1.82089 0 down vs 1 10.3134 106.367 0.783185 1.56097 1cg18007268 ABLIM2 -0.03832 0.227169 1.86E-20 -0.168685 -5.9282 0 down vs 1 -10.3081 106.256 1.27794 2.54972 1cg19312058 PEG10 0.26545 0.431797 1.89E-20 0.614756 -1.62666 0 down vs 1 -10.3056 106.206 0.501707 1.00147 1cg16927040 SST -0.23628 0.101274 1.95E-20 -2.33307 2.33307 0 up vs 1 -10.301 106.11 2.06588 4.12748 1cg07569216 ONECUT1 -0.217725 0.17114 1.98E-20 -1.2722 1.2722 0 up vs 1 -10.299 106.07 2.74168 5.47974 1cg22078179 KLHL5 0.052715 0.355965 1.99E-20 0.14809 -6.75263 0 down vs 1 -10.2987 106.063 1.66732 3.33267 1cg22521696 ZNF781 -0.369515 -0.0653448 2.00E-20 5.65485 5.65485 0 up vs 1 -10.2977 106.043 1.67746 3.35354 1cg12178578 HPDL -0.233115 -0.479093 2.01E-20 0.486576 -2.05518 0 down vs 1 10.297 106.029 1.09701 2.19341 1cg01329005 BST2 -0.0831 -0.395505 2.02E-20 0.210111 -4.75938 0 down vs 1 10.2961 106.009 1.76951 3.53873 1cg07035165 KCNN2 -0.164395 0.142904 2.04E-20 -1.15039 1.15039 0 up vs 1 -10.2951 105.99 1.71215 3.42461 1cg03810428 -0.29721 0.0992175 2.04E-20 -2.99554 2.99554 0 up vs 1 -10.2946 105.979 2.84935 5.69982 1cg10751726 LOC150786 -0.348435 -0.0510567 2.05E-20 6.82447 6.82447 0 up vs 1 -10.294 105.967 1.60338 3.20776 1cg11323721 -0.14485 0.213277 2.06E-20 -0.679163 -1.4724 0 down vs 1 -10.2932 105.951 2.32537 4.65291 1cg23479922 11-Mar -0.099435 0.211548 2.08E-20 -0.470034 -2.1275 0 down vs 1 -10.2922 105.928 1.75345 3.50926 1cg05452645 PRDM8 -0.13352 0.206222 2.08E-20 -0.647457 -1.5445 0 down vs 1 -10.292 105.925 2.09275 4.18846 1cg12569246 FILIP1 -0.07852 0.321897 2.09E-20 -0.243929 -4.09956 0 down vs 1 -10.2911 105.907 2.90699 5.81908 1cg23766591 EDNRB -0.294825 0.058068 2.11E-20 -5.07723 5.07723 0 up vs 1 -10.2898 105.879 2.2579 4.52094 1cg00428382 -0.08396 0.264972 2.12E-20 -0.316863 -3.15593 0 down vs 1 -10.2892 105.868 2.2075 4.42049 1cg25273520 0.204085 0.388962 2.16E-20 0.524692 -1.90588 0 down vs 1 -10.2867 105.817 0.619702 1.24155 1cg07899263 0.251225 0.418589 2.24E-20 0.600171 -1.66619 0 down vs 1 -10.2811 105.7 0.507859 1.0186 1cg06067842 TMEM26 -0.294055 0.106128 2.26E-20 -2.77076 2.77076 0 up vs 1 -10.2803 105.684 2.90359 5.82455 1cg04347874 NKX2-1 -0.22166 0.205421 2.26E-20 -1.07905 1.07905 0 up vs 1 -10.2801 105.68 3.30703 6.6341 1cg27314569 BSX -0.22925 0.199787 2.27E-20 -1.14747 1.14747 0 up vs 1 -10.2794 105.666 3.33739 6.6959 1cg07178278 0.25183 0.475839 2.38E-20 0.529234 -1.88952 0 down vs 1 -10.2723 105.52 0.909803 1.82789 1cg14463412 -0.33409 0.0538082 2.45E-20 -6.2089 6.2089 0 up vs 1 -10.2683 105.439 2.72806 5.48515 1cg17496661 0.128425 0.437441 2.47E-20 0.293583 -3.4062 0 down vs 1 -10.2673 105.418 1.73133 3.48179 1cg15174906 -0.01087 0.295513 2.48E-20 -0.0367834 -27.1861 0 down vs 1 -10.2667 105.404 1.70196 3.42316 1cg01708344 10-Mar -0.037595 0.369572 2.50E-20 -0.101726 -9.83035 0 down vs 1 -10.2657 105.384 3.00582 6.0468 1cg17227967 C20orf103 -0.29411 0.0814675 2.51E-20 -3.61015 3.61015 0 up vs 1 -10.2648 105.365 2.55751 5.14582 1cg18518485 TNXB 0.148895 0.347696 2.53E-20 0.428233 -2.33518 0 down vs 1 -10.2639 105.347 0.716567 1.44202 1cg24606807 TFF1 0.11477 0.400937 2.55E-20 0.286254 -3.4934 0 down vs 1 -10.2624 105.316 1.48476 2.9888 1cg22374940 -0.342065 0.0608954 2.60E-20 -5.61726 5.61726 0 up vs 1 -10.2601 105.269 2.94403 5.92897 1cg23878831 0.202915 0.426682 2.60E-20 0.475565 -2.10276 0 down vs 1 -10.2595 105.258 0.907841 1.82849 1cg24862252 SDC2 0.12835 0.375899 2.65E-20 0.341448 -2.92871 0 down vs 1 -10.257 105.206 1.11107 2.23891 1cg03326335 SORCS2 0.132565 0.403323 2.77E-20 0.328682 -3.04246 0 down vs 1 -10.2561 105.188 1.32853 2.66494 1cg23494533 -0.01703 0.340448 2.79E-20 -0.0500223 -19.9911 0 down vs 1 -10.2495 105.052 2.31695 4.67571 1cg00810908 FBLN2 0.300915 0.471009 2.80E-20 0.638873 -1.56526 0 down vs 1 -10.2492 105.046 0.524563 1.05866 1cg13489528 0.14271 0.399112 2.80E-20 0.357568 -2.79667 0 down vs 1 -10.2492 105.045 1.19196 2.40559 1cg07651720 -0.030315 0.328064 2.85E-20 -0.0924058 -10.8218 0 down vs 1 -10.2465 104.991 2.32864 4.70206 1cg09480047 -0.0798 0.254549 2.88E-20 -0.313496 -3.18984 0 down vs 1 -10.2452 104.964 2.02683 4.09366 1cg06553513 -0.060895 0.303626 2.89E-20 -0.200559 -4.98605 0 down vs 1 -10.2447 104.954 2.40914 4.86632 1cg08885633 0.12255 0.401883 2.92E-20 0.30494 -3.27934 0 down vs 1 -10.2429 104.918 1.41469 2.85858 1cg19444859 MYO16 0.10612 0.331092 2.99E-20 0.320515 -3.11998 0 down vs 1 -10.2394 104.846 0.917648 1.8555 1cg13723431 SLC6A3 -0.022065 0.262209 3.13E-20 -0.0841505 -11.8835 0 down vs 1 -10.2328 104.71 1.46518 2.96648 1cg09880291 HOXA5 0.22306 0.427108 3.20E-20 0.522256 -1.91477 0 down vs 1 -10.2297 104.646 0.75489 1.52931 1cg21005369 COL4A2 0.136455 0.391554 3.29E-20 0.348496 -2.86947 0 down vs 1 -10.2258 104.567 1.17987 2.39208 1cg13779907 EPHA10 -0.301165 0.0137562 3.32E-20 -21.8931 21.8931 0 up vs 1 -10.2245 104.54 1.79813 3.64649 1cg07462540 NXPH1 -0.270695 0.154834 3.35E-20 -1.7483 1.7483 0 up vs 1 -10.223 104.51 3.28303 6.65965 1cg23197945 CPN1 0.205575 0.458659 3.36E-20 0.448209 -2.2311 0 down vs 1 -10.2228 104.505 1.16131 2.35584 1cg03371275 MAML2 0.195305 0.386821 3.45E-20 0.504897 -1.9806 0 down vs 1 -10.2188 104.424 0.665011 1.3501 1cg04933640 RBP3 0.24112 0.460388 3.46E-20 0.523732 -1.90937 0 down vs 1 -10.2185 104.417 0.871704 1.76984 1cg14368592 CHRNA4 0.27237 0.467871 3.57E-20 0.582148 -1.71778 0 down vs 1 -10.2141 104.328 0.69297 1.40816 1cg10606698 FILIP1 -0.04501 0.341856 3.62E-20 -0.131664 -7.59512 0 down vs 1 -10.2119 104.283 2.71356 5.51648 1cg26571759 TNXB 0.09091 0.342496 3.71E-20 0.265434 -3.76742 0 down vs 1 -10.2083 104.209 1.1476 2.33464 1cg09414535 GRIP1 -0.009145 0.370585 3.72E-20 -0.0246772 -40.5232 0 down vs 1 -10.2078 104.2 2.61437 5.31905 1cg21824300 KEL 0.18468 0.420471 3.75E-20 0.439222 -2.27676 0 down vs 1 -10.2069 104.18 1.00803 2.05127 1cg18279004 0.074175 0.332075 3.85E-20 0.223368 -4.47691 0 down vs 1 -10.2032 104.105 1.20592 2.45576 1cg16657149 0.20173 0.424144 3.91E-20 0.475616 -2.10253 0 down vs 1 -10.2007 104.055 0.896899 1.82733 1cg03137071 -0.10185 0.250794 3.98E-20 -0.40611 -2.46238 0 down vs 1 -10.1981 104.002 2.25471 4.59605 1cg00811334 CTNNA2 -0.166805 0.21634 4.00E-20 -0.771033 -1.29696 0 down vs 1 -10.1974 103.987 2.6616 5.42624 1cg13473356 PEX5L -0.233695 0.0671005 4.06E-20 -3.48276 3.48276 0 up vs 1 -10.1954 103.947 1.64044 3.34568 1cg00068428 -0.12776 0.239898 4.11E-20 -0.53256 -1.87772 0 down vs 1 -10.1937 103.911 2.45079 5.00011 1cg02087067 0.16185 0.412012 4.30E-20 0.392828 -2.54564 0 down vs 1 -10.1869 103.774 1.13465 2.31799 1cg11952493 MPP7 0.117275 0.364796 4.49E-20 0.321481 -3.11061 0 down vs 1 -10.1808 103.648 1.11082 2.27205 1cg26714388 0.05361 0.432373 4.59E-20 0.12399 -8.06516 0 down vs 1 -10.1776 103.584 2.60108 5.32351 1cg16541979 0.09654 0.37742 4.60E-20 0.25579 -3.90946 0 down vs 1 -10.1771 103.573 1.4304 2.92785 1cg17507952 11-Mar -0.249695 0.075349 4.70E-20 -3.31385 3.31385 0 up vs 1 -10.1742 103.515 1.91559 3.92315 1cg12428514 UCN3 -0.071125 0.362459 4.70E-20 -0.196229 -5.09609 0 down vs 1 -10.174 103.51 3.40851 6.981 1cg06753057 0.29156 0.431707 4.73E-20 0.675366 -1.48068 0 down vs 1 -10.1732 103.494 0.35611 0.729464 1cg08882216 EPHB6 0.153845 0.407485 5.05E-20 0.377548 -2.64867 0 down vs 1 -10.1637 103.301 1.16642 2.39379 1cg02164046 SST -0.20583 0.167845 5.18E-20 -1.22631 1.22631 0 up vs 1 -10.16 103.226 2.53166 5.1994 1cg22706610 PDE4D -0.2159 0.0953845 5.22E-20 -2.26347 2.26347 0 up vs 1 -10.1588 103.202 1.75684 3.60897 1cg13060531 TTLL7 0.156465 0.393377 5.30E-20 0.397748 -2.51415 0 down vs 1 -10.1567 103.159 1.01763 2.09133 1cg19410471 C7orf34 0.12055 0.4088 5.36E-20 0.294887 -3.39112 0 down vs 1 -10.1552 103.127 1.50646 3.09684 1cg19856749 0.25361 0.45451 5.41E-20 0.557985 -1.79216 0 down vs 1 -10.1537 103.099 0.731778 1.50474 1cg08355659 0.016705 0.267738 5.43E-20 0.0623932 -16.0274 0 down vs 1 -10.1533 103.089 1.14256 2.34964 1cg16890879 ARX 0.096355 0.37162 5.48E-20 0.259284 -3.85678 0 down vs 1 -10.1517 103.058 1.37379 2.82602 1cg08533865 ENGASE 0.309025 0.488603 5.52E-20 0.632466 -1.58111 0 down vs 1 -10.1507 103.036 0.584689 1.20302 1cg21969116 -0.014705 0.328796 5.57E-20 -0.0447238 -22.3595 0 down vs 1 -10.1495 103.013 2.13931 4.40267 1cg01827726 TTYH3 -0.315615 -0.455501 5.58E-20 0.692896 -1.44322 0 down vs 1 10.1492 103.007 0.354786 0.730188 1cg02640041 RXFP3 -0.23618 0.0942881 5.69E-20 -2.50487 2.50487 0 up vs 1 -10.1463 102.947 1.98005 4.07755 1cg16742090 0.134235 0.391542 5.73E-20 0.342837 -2.91684 0 down vs 1 -10.1455 102.931 1.20039 2.47237 1cg14672084 PCDHGA4 -0.04928 0.401028 5.85E-20 -0.122884 -8.13775 0 down vs 1 -10.1424 102.868 3.67653 7.57692 1cg00500705 -0.34224 -0.0485608 6.06E-20 7.04766 7.04766 0 up vs 1 -10.1371 102.762 1.56374 3.22604 1cg04945076 -0.097645 0.257824 6.13E-20 -0.378727 -2.64042 0 down vs 1 -10.1355 102.729 2.29098 4.72786 1cg24054700 ABLIM2 0.16189 0.419982 6.16E-20 0.385468 -2.59425 0 down vs 1 -10.1348 102.714 1.20773 2.49273 1cg13842202 KDM4C 0.177 0.410533 6.19E-20 0.431147 -2.3194 0 down vs 1 -10.1342 102.702 0.988814 2.04113 1cg10187559 TTYH1 0.09508 0.349453 6.23E-20 0.272082 -3.67536 0 down vs 1 -10.1332 102.681 1.17317 2.42217 1cg25369070 0.1523 0.379523 6.30E-20 0.401294 -2.49194 0 down vs 1 -10.1316 102.648 0.936098 1.93333 1cg04894619 PEX5L -0.232615 0.0547835 6.36E-20 -4.24608 4.24608 0 up vs 1 -10.1302 102.62 1.49757 3.09379 1cg26673975 CDH5 0.28631 0.483246 6.40E-20 0.592472 -1.68784 0 down vs 1 -10.1293 102.604 0.703185 1.45293 1cg24277733 0.028855 0.339344 6.45E-20 0.0850318 -11.7603 0 down vs 1 -10.1281 102.579 1.74787 3.61232 1cg14447193 NTRK2 -0.01008 0.284728 6.48E-20 -0.0354022 -28.2469 0 down vs 1 -10.1275 102.566 1.57579 3.25709 1cg23248995 SND1 0.251025 0.408665 6.52E-20 0.614255 -1.62799 0 down vs 1 -10.1267 102.551 0.450561 0.931432 1cg00328227 C1orf59 -0.038245 -0.278273 6.52E-20 0.137437 -7.27605 0 down vs 1 10.1266 102.548 1.04458 2.15947 1cg13775295 COL5A1 0.082355 0.347736 6.84E-20 0.236832 -4.2224 0 down vs 1 -10.1197 102.409 1.2769 2.64336 1cg26310551 LTB4R2 -0.03672 -0.373815 6.87E-20 0.0982304 -10.1801 0 down vs 1 10.119 102.394 2.06026 4.26564 1cg02801786 -0.15169 0.20096 6.87E-20 -0.754828 -1.32481 0 down vs 1 -10.119 102.394 2.25479 4.6684 1cg23654821 CSRNP1 -0.13517 -0.403912 6.92E-20 0.334652 -2.98818 0 down vs 1 10.118 102.374 1.30945 2.71165 1cg02108015 0.03826 0.310103 6.98E-20 0.123378 -8.10515 0 down vs 1 -10.1168 102.35 1.33984 2.77525 1cg06225294 ZBTB20 -0.04259 0.31066 7.09E-20 -0.137095 -7.29421 0 down vs 1 -10.1144 102.301 2.26247 4.68854 1cg27633533 CDH4 0.10069 0.393102 7.12E-20 0.256142 -3.90408 0 down vs 1 -10.1139 102.29 1.55027 3.213 1cg19789466 OAS1 -0.25261 -0.428488 7.13E-20 0.589539 -1.69624 0 down vs 1 10.1137 102.287 0.56084 1.16239 1cg23049130 THRB 0.17158 0.399849 7.17E-20 0.429111 -2.3304 0 down vs 1 -10.1129 102.271 0.944743 1.95838 1cg25114913 FUT9 -0.20392 0.226869 7.19E-20 -0.898846 -1.11254 0 down vs 1 -10.1124 102.26 3.3647 6.97553 1cg07509935 LTB4R2 -0.095355 -0.391343 7.38E-20 0.243661 -4.10407 0 down vs 1 10.1086 102.184 1.58843 3.2955 1cg11254700 -0.065005 0.302208 7.71E-20 -0.2151 -4.64899 0 down vs 1 -10.1023 102.057 2.44486 5.07864 1cg10741422 -0.28894 0.168067 7.76E-20 -1.7192 1.7192 0 up vs 1 -10.1014 102.038 3.78672 7.86753 1cg20707222 ST6GALNAC3 -0.387345 0.0290778 7.79E-20 -13.321 13.321 0 up vs 1 -10.1009 102.027 3.14403 6.5329 1cg05599160 -0.117625 0.244104 7.85E-20 -0.481864 -2.07527 0 down vs 1 -10.0997 102.004 2.37238 4.93063 1cg19671120 CNGA3 -0.176955 0.122726 7.85E-20 -1.44187 1.44187 0 up vs 1 -10.0997 102.004 1.6283 3.38418 1cg03799405 0.114065 0.429551 7.93E-20 0.265545 -3.76584 0 down vs 1 -10.0982 101.974 1.80458 3.75164 1cg17972100 ABLIM2 -0.044295 0.307171 7.96E-20 -0.144203 -6.93466 0 down vs 1 -10.0977 101.964 2.23967 4.65665 1cg02889647 IVL 0.03453 0.323232 8.20E-20 0.106827 -9.3609 0 down vs 1 -10.0933 101.874 1.51118 3.14476 1cg17432857 HOXA5 0.11109 0.37941 8.25E-20 0.292797 -3.41534 0 down vs 1 -10.0925 101.859 1.30534 2.71681 1cg15575538 0.004825 0.291648 8.27E-20 0.0165439 -60.4452 0 down vs 1 -10.092 101.849 1.49158 3.10473 1cg05859929 SLC9A3 0.150095 0.392865 8.49E-20 0.382052 -2.61745 0 down vs 1 -10.0882 101.772 1.06859 2.22595 1cg16932094 PRAME -0.111625 0.27835 8.66E-20 -0.401024 -2.49362 0 down vs 1 -10.0854 101.715 2.75735 5.74699 1cg02012703 VSTM2B -0.219205 0.183736 8.79E-20 -1.19304 1.19304 0 up vs 1 -10.0832 101.671 2.94375 6.13816 1cg25183214 EPHA4 0.15945 0.409463 8.83E-20 0.389413 -2.56797 0 down vs 1 -10.0826 101.66 1.13329 2.36336 1cg08396193 HOXA7 0.23374 0.414292 8.85E-20 0.564191 -1.77245 0 down vs 1 -10.0823 101.653 0.591049 1.23265 1cg16150702 RASGEF1C -0.035725 0.28314 9.00E-20 -0.126174 -7.92553 0 down vs 1 -10.0798 101.601 1.84345 3.84651 1cg08903619 0.14191 0.432625 9.09E-20 0.328021 -3.04859 0 down vs 1 -10.0784 101.574 1.53233 3.1982 1cg19911801 PLCH2 -0.0738 0.250035 9.16E-20 -0.295159 -3.388 0 down vs 1 -10.0773 101.552 1.90136 3.96928 1cg01765174 TRIM14 0.053385 -0.217558 9.29E-20 -0.245383 -4.07527 0 down vs 1 10.0753 101.511 1.33099 2.77969 1cg01511828 CCBE1 -0.28014 0.183142 9.34E-20 -1.52963 1.52963 0 up vs 1 -10.0744 101.494 3.89142 8.12834 1cg20168823 BRUNOL4 0.32575 0.482845 9.55E-20 0.674647 -1.48226 0 down vs 1 -10.0712 101.43 0.447447 0.935215 1cg19742623 MYO16 0.11592 0.330563 9.84E-20 0.350674 -2.85165 0 down vs 1 -10.0669 101.342 0.83532 1.74742 1cg00091349 MED12L 0.154185 0.393879 9.95E-20 0.391452 -2.55459 0 down vs 1 -10.0652 101.309 1.04168 2.17983 1cg24676244 THSD7A -0.205525 0.187841 9.95E-20 -1.09415 1.09415 0 up vs 1 -10.0652 101.307 2.8055 5.87091 1cg13289202 ABTB2 0.055735 0.351115 9.96E-20 0.158737 -6.29972 0 down vs 1 -10.065 101.305 1.5819 3.31043 1cg04939026 0.00242 0.276997 1.01E-19 0.00873654 -114.462 0 down vs 1 -10.0637 101.277 1.36693 2.86135 1cg12542255 FOSB -0.0139 0.357616 1.05E-19 -0.0388685 -25.7278 0 down vs 1 -10.058 101.164 2.50249 5.24424 1cg07572317 KNDC1 -0.006065 0.406442 1.08E-19 -0.0149222 -67.0143 0 down vs 1 -10.0537 101.077 3.08518 6.47089 1cg26620747 GTF3C1 0.17257 0.366139 1.08E-19 0.471324 -2.12168 0 down vs 1 -10.0532 101.066 0.679345 1.42501 1cg22560214 -0.151705 0.160379 1.08E-19 -0.945913 -1.05718 0 down vs 1 -10.053 101.062 1.76588 3.70432 1cg01663725 GRIN2B -0.30707 0.0587577 1.10E-19 -5.22604 5.22604 0 up vs 1 -10.0504 101.012 2.42645 5.09256 1cg06484274 XRN1 -0.11685 -0.39686 1.17E-19 0.294436 -3.39632 0 down vs 1 10.0414 100.831 1.42156 2.98887 1cg09008589 GLI3 0.170435 0.369593 1.18E-19 0.461142 -2.16853 0 down vs 1 -10.0408 100.818 0.719142 1.51222 1cg24885556 ITGA7 -0.02502 0.303573 1.19E-19 -0.0824185 -12.1332 0 down vs 1 -10.0388 100.777 1.95764 4.11821 1cg16092834 C4orf32 -0.07035 0.192959 1.21E-19 -0.364586 -2.74284 0 down vs 1 -10.037 100.741 1.25704 2.64531 1cg05493583 0.24356 0.459619 1.21E-19 0.529917 -1.88709 0 down vs 1 -10.037 100.741 0.846375 1.78112 1cg02823803 ZNF471 -0.290735 -0.0130732 1.21E-19 22.239 22.239 0 up vs 1 -10.0365 100.732 1.39782 2.94184 1cg14842771 EPHB6 0.135595 0.406161 1.22E-19 0.333845 -2.9954 0 down vs 1 -10.0356 100.714 1.32729 2.7939 1cg00592643 BTBD3 -0.08426 0.312719 1.25E-19 -0.269444 -3.71135 0 down vs 1 -10.0322 100.644 2.85728 6.01865 1cg18257574 -0.23245 -0.407875 1.26E-19 0.569905 -1.75468 0 down vs 1 10.0305 100.61 0.557959 1.1757 1cg08272731 LHX8 -0.167295 0.174531 1.29E-19 -0.958538 -1.04326 0 down vs 1 -10.0271 100.542 2.1185 4.467 1cg04786142 -0.16211 -0.444624 1.34E-19 0.3646 -2.74273 0 down vs 1 10.0218 100.437 1.44709 3.05449 1cg15737177 0.097125 0.423302 1.35E-19 0.229446 -4.35832 0 down vs 1 -10.0212 100.425 1.92896 4.07208 1cg15425280 GRIA2 -0.291825 0.117715 1.35E-19 -2.47907 2.47907 0 up vs 1 -10.0206 100.412 3.04097 6.42041 1cg02648464 -0.008695 0.314997 1.36E-19 -0.0276034 -36.2274 0 down vs 1 -10.0201 100.402 1.89969 4.0112 1cg04392133 0.20459 0.426796 1.38E-19 0.479362 -2.08611 0 down vs 1 -10.0174 100.348 0.895223 1.89129 1cg23696618 SERPINB10 0.005125 0.333414 1.39E-19 0.0153713 -65.0564 0 down vs 1 -10.017 100.34 1.95403 4.12849 1cg05647859 LIN7A -0.282955 0.196987 1.39E-19 -1.43641 1.43641 0 up vs 1 -10.0166 100.332 4.17634 8.82458 1cg18961681 -0.09948 0.214143 1.41E-19 -0.46455 -2.15262 0 down vs 1 -10.0148 100.296 1.78334 3.76951 1cg02477448 H1F0 0.019625 0.349018 1.42E-19 0.0562293 -17.7843 0 down vs 1 -10.0132 100.264 1.96719 4.15946 1cg14426174 SERPINB7 0.093475 0.357372 1.43E-19 0.261562 -3.82318 0 down vs 1 -10.0127 100.255 1.26266 2.67003 1cg04470054 RPTOR -0.02252 -0.358277 1.43E-19 0.0628564 -15.9093 0 down vs 1 10.0321 100.643 2.03992 4.21594 1cg12400610 0.09735 0.377636 1.44E-19 0.257788 -3.87916 0 down vs 1 -10.011 100.221 1.42436 3.013 1cg10383424 0.072 0.375825 1.45E-19 0.191578 -5.2198 0 down vs 1 -10.0103 100.207 1.67365 3.54082 1cg08872781 TSPAN9 0.215675 0.419991 1.46E-19 0.513523 -1.94733 0 down vs 1 -10.0095 100.19 0.75687 1.60152 1cg10799440 -0.149365 0.225908 1.47E-19 -0.661176 -1.51246 0 down vs 1 -10.0081 100.162 2.55337 5.40437 1cg14966325 SPRR2A -0.002285 0.345846 1.49E-19 -0.00660698 -151.355 0 down vs 1 -10.0067 100.134 2.19738 4.65221 1cg00615473 TIAM1 -0.273825 -0.478691 1.53E-19 0.572028 -1.74816 0 down vs 1 10.0025 100.05 0.760955 1.61241 1cg12984948 0.08169 0.340094 1.54E-19 0.240198 -4.16323 0 down vs 1 -10.0016 100.031 1.21065 2.56576 1cg22588445 PTPRN2 0.018915 0.364147 1.55E-19 0.0519432 -19.2518 0 down vs 1 -10.0007 100.014 2.16093 4.58053 1cg10807461 HLA-G 0.236095 0.398898 1.59E-19 0.591867 -1.68957 0 down vs 1 -9.99713 99.9427 0.480557 1.01937 1cg16783349 -0.000665 0.373526 1.61E-19 -0.00178033 -561.693 0 down vs 1 -9.99534 99.9068 2.53866 5.38697 1cg00457403 SST -0.346825 0.107069 1.61E-19 -3.23926 3.23926 0 up vs 1 -9.99514 99.9028 3.73531 7.92657 1cg20434856 MYO10 -0.03001 0.277684 1.67E-19 -0.108072 -9.25305 0 down vs 1 -9.98988 99.7977 1.71655 3.64646 1cg02270332 0.035265 0.308427 1.72E-19 0.114338 -8.74599 0 down vs 1 -9.98514 99.703 1.35288 2.87665 1cg27601809 CLPB 0.318915 0.43222 1.74E-19 0.737853 -1.35528 0 down vs 1 -9.98396 99.6795 0.232765 0.495048 1cg14108567 -0.001085 0.386685 1.76E-19 -0.0028059 -356.392 0 down vs 1 -9.98251 99.6505 2.72626 5.79993 1cg18591727 -0.02247 0.302578 1.76E-19 -0.0742618 -13.4659 0 down vs 1 -9.98244 99.6492 1.91564 4.07545 1cg13882311 OLFM1 0.09979 0.372441 1.80E-19 0.267935 -3.73225 0 down vs 1 -9.97857 99.5719 1.34782 2.86967 1cg03041696 -0.25311 0.165741 1.82E-19 -1.52714 1.52714 0 up vs 1 -9.97723 99.545 3.1808 6.77412 1cg22883472 EBF2 -0.267075 0.107074 1.84E-19 -2.4943 2.4943 0 up vs 1 -9.97529 99.5064 2.53809 5.40745 1cg04918696 -0.04445 0.313739 1.86E-19 -0.141678 -7.05824 0 down vs 1 -9.97425 99.4857 2.32617 4.95698 1cg23139018 0.02852 0.326202 1.90E-19 0.0874306 -11.4376 0 down vs 1 -9.97089 99.4187 1.60665 3.42602 1cg22920300 SOX2OT -0.16821 0.20083 1.96E-19 -0.837574 -1.19392 0 down vs 1 -9.96669 99.3349 2.46925 5.26985 1cg06372779 OSBPL3 0.201995 0.402046 1.96E-19 0.502417 -1.99038 0 down vs 1 -9.96612 99.3236 0.725606 1.54876 1cg26118906 -0.260255 0.0849144 1.99E-19 -3.06491 3.06491 0 up vs 1 -9.96393 99.2799 2.16014 4.61272 1cg03665212 -0.17492 0.183641 1.99E-19 -0.952512 -1.04986 0 down vs 1 -9.96387 99.2787 2.33101 4.97764 1cg06773295 PRDM16 0.15552 0.403804 2.00E-19 0.385138 -2.59647 0 down vs 1 -9.96333 99.2679 1.11767 2.38694 1cg12258607 PHACTR1 0.26304 0.406009 2.03E-19 0.647868 -1.54352 0 down vs 1 -9.9616 99.2335 0.370596 0.791732 1cg02263813 MT1A -0.23546 0.119839 2.10E-19 -1.9648 1.9648 0 up vs 1 -9.95613 99.1245 2.28879 4.8951 1cg27639662 PRDM8 0.040115 0.258148 2.12E-19 0.155395 -6.4352 0 down vs 1 -9.9553 99.108 0.86191 1.8437 1cg06392956 -0.024435 0.291262 2.18E-19 -0.0838936 -11.9199 0 down vs 1 -9.95108 99.024 1.807 3.8686 1cg00721530 -0.24366 0.0275351 2.20E-19 -8.84908 8.84908 0 up vs 1 -9.94946 98.9918 1.33346 2.85574 1cg25936177 -0.223845 0.0538696 2.21E-19 -4.15531 4.15531 0 up vs 1 -9.94921 98.9869 1.39835 2.99484 1cg15180617 LOC285830 -0.0348947 0.307724 2.24E-19 -0.113396 -8.81864 0 down vs 1 -9.952 99.0424 2.03141 4.32773 1cg22388472 -0.328125 0.0572165 2.27E-19 -5.7348 5.7348 0 up vs 1 -9.94515 98.9059 2.69221 5.77063 1cg27063986 NDST4 0.01022 0.355969 2.28E-19 0.0287104 -34.8306 0 down vs 1 -9.94446 98.8924 2.1674 4.64635 1cg18350249 MDN1 0.324275 0.427462 2.33E-19 0.758606 -1.31821 0 down vs 1 -9.94128 98.8291 0.193049 0.414112 1cg13731936 0.17399 0.382269 2.36E-19 0.455151 -2.19707 0 down vs 1 -9.93906 98.7848 0.786519 1.68793 1cg26187313 0.01425 0.391476 2.39E-19 0.0364007 -27.472 0 down vs 1 -9.93764 98.7567 2.58001 5.53849 1cg25730685 -0.31924 0.127844 2.41E-19 -2.4971 2.4971 0 up vs 1 -9.93605 98.725 3.62407 7.78226 1cg18120576 0.19091 0.399062 2.45E-19 0.478397 -2.09031 0 down vs 1 -9.93364 98.6771 0.785558 1.68771 1cg13832669 -0.163335 0.260326 2.47E-19 -0.627426 -1.59381 0 down vs 1 -9.94272 98.8577 3.25111 6.90621 1cg01485075 ZNF492 -0.201835 0.213043 2.49E-19 -0.94739 -1.05553 0 down vs 1 -9.93128 98.6303 3.12075 6.70788 1cg14037948 GPR144 -0.095815 0.154976 2.54E-19 -0.618258 -1.61745 0 down vs 1 -9.92865 98.578 1.14036 2.45243 1cg05021743 C2CD4D 0.01023 -0.418245 2.54E-19 -0.0244594 -40.8841 0 down vs 1 9.92841 98.5733 3.32865 7.15888 1cg14866863 -0.179455 0.196506 2.55E-19 -0.913231 -1.09501 0 down vs 1 -9.92821 98.5694 2.56273 5.51184 1cg22537280 SSBP3 0.28693 0.435654 2.56E-19 0.658619 -1.51833 0 down vs 1 -9.92766 98.5584 0.401034 0.862627 1cg15015892 C2CD4D 0.04252 -0.404224 2.65E-19 -0.105189 -9.50668 0 down vs 1 9.92258 98.4576 3.61856 7.79153 1cg09655666 -0.199915 0.18073 2.65E-19 -1.10615 1.10615 0 up vs 1 -9.92219 98.4498 2.62699 5.65692 1cg26163319 0.21385 0.401201 2.66E-19 0.533025 -1.87609 0 down vs 1 -9.92172 98.4406 0.6364 1.37054 1cg27124218 0.16322 0.40112 2.69E-19 0.406911 -2.45754 0 down vs 1 -9.92037 98.4137 1.02614 2.21048 1cg02699898 ATP11A 0.22896 0.453758 2.70E-19 0.504586 -1.98182 0 down vs 1 -9.91964 98.3993 0.916228 1.974 1cg12031078 IRS4 -0.104085 0.265436 2.70E-19 -0.392129 -2.55018 0 down vs 1 -9.91958 98.3982 2.47568 5.33389 1cg00039129 0.08777 0.371327 2.72E-19 0.236368 -4.23069 0 down vs 1 -9.91865 98.3797 1.45781 3.14145 1cg23273257 NR3C1 0.30545 0.449423 2.73E-19 0.67965 -1.47135 0 down vs 1 -9.91807 98.368 0.375818 0.809953 1cg24032249 -0.016985 0.24819 2.75E-19 -0.0684354 -14.6123 0 down vs 1 -9.91721 98.351 1.27492 2.74815 1cg03075534 -0.249755 0.0464412 2.77E-19 -5.37787 5.37787 0 up vs 1 -9.91615 98.33 1.59066 3.42947 1cg05774699 HOXA5 0.08238 0.310165 2.77E-19 0.265601 -3.76505 0 down vs 1 -9.91568 98.3208 0.940737 2.02842 1cg17919396 ALDH1A3 0.171765 0.393523 2.78E-19 0.436481 -2.29105 0 down vs 1 -9.91558 98.3186 0.891611 1.92254 1cg04815577 -0.010745 0.324531 2.82E-19 -0.0331093 -30.203 0 down vs 1 -9.91348 98.2771 2.03809 4.39649 1cg03399028 RHCG 0.03068 0.268881 2.84E-19 0.114103 -8.76405 0 down vs 1 -9.91215 98.2507 1.02874 2.21975 1cg25191983 BNIP3L 0.217345 0.370872 3.00E-19 0.586038 -1.70638 0 down vs 1 -9.90418 98.0927 0.427355 0.923608 1cg03534360 SYT16 0.2394 0.436363 3.06E-19 0.548625 -1.82274 0 down vs 1 -9.90117 98.0331 0.703378 1.52108 1cg15071854 HPDL -0.23828 -0.481178 3.07E-19 0.495202 -2.01938 0 down vs 1 9.90094 98.0286 1.06971 2.31339 1cg09237133 SECTM1 0.12896 0.445059 3.10E-19 0.289759 -3.45114 0 down vs 1 -9.8993 97.9961 1.81161 3.91915 1cg25714208 -0.10326 0.259485 3.11E-19 -0.397943 -2.51292 0 down vs 1 -9.8988 97.9863 2.38572 5.16167 1cg24010402 ZNF322A 0.023705 0.293184 3.12E-19 0.0808538 -12.368 0 down vs 1 -9.89872 97.9846 1.31664 2.84868 1cg12873119 CD93 0.097875 0.328958 3.12E-19 0.297531 -3.361 0 down vs 1 -9.89857 97.9817 0.968173 2.09481 1cg11421927 SYTL2 0.11294 0.351922 3.15E-19 0.320924 -3.11601 0 down vs 1 -9.89735 97.9576 1.03549 2.24102 1cg06047040 0.191735 0.393084 3.20E-19 0.487771 -2.05014 0 down vs 1 -9.89491 97.9093 0.73505 1.59158 1cg11549972 -0.282805 0.114364 3.24E-19 -2.47285 2.47285 0 up vs 1 -9.89295 97.8704 2.86002 6.19517 1cg12563644 LOC645323 -0.41271 -0.0120149 3.26E-19 34.3497 34.3497 0 up vs 1 -9.89207 97.853 2.91102 6.30678 1cg19738950 ADARB2 0.15985 0.399313 3.27E-19 0.400312 -2.49805 0 down vs 1 -9.89177 97.847 1.03967 2.2526 1cg19781637 LHFPL2 0.20498 0.401538 3.27E-19 0.510488 -1.95891 0 down vs 1 -9.89159 97.8436 0.700483 1.51775 1cg01364621 0.059715 0.304158 3.32E-19 0.196329 -5.09349 0 down vs 1 -9.88924 97.797 1.08336 2.34846 1cg01889574 CD82 -0.267645 -0.451805 3.33E-19 0.592391 -1.68807 0 down vs 1 9.88907 97.7937 0.614903 1.33301 1cg23675523 ADAMTS17 0.235455 0.426932 3.61E-19 0.551505 -1.81322 0 down vs 1 -9.87708 97.5568 0.664739 1.44454 1cg22014983 MAP4K3 0.338155 0.421138 3.64E-19 0.802955 -1.2454 0 down vs 1 -9.87616 97.5386 0.124853 0.271367 1cg08781681 0.119 0.40806 3.74E-19 0.291624 -3.42908 0 down vs 1 -9.87211 97.4585 1.51494 3.29542 1cg20067780 TBC1D16 -0.173395 0.23546 3.74E-19 -0.736409 -1.35794 0 down vs 1 -9.87184 97.4531 3.0308 6.59322 1cg07678266 GLT1D1 0.16403 0.445924 3.75E-19 0.367843 -2.71855 0 down vs 1 -9.87163 97.4491 1.44075 3.13434 1cg11838827 -0.12176 0.29407 3.76E-19 -0.414052 -2.41516 0 down vs 1 -9.87115 97.4396 3.13508 6.82102 1cg14204430 NDST4 -0.12402 0.257636 3.84E-19 -0.481378 -2.07737 0 down vs 1 -9.86801 97.3776 2.64096 5.7496 1cg11630135 FBLN2 0.21525 0.455572 3.87E-19 0.472483 -2.11648 0 down vs 1 -9.867 97.3576 1.04714 2.28018 1cg07223990 FAM155A -0.32057 0.0114196 3.91E-19 -28.0719 28.0719 0 up vs 1 -9.86535 97.3251 1.99833 4.35289 1cg01910741 PPIL1 -0.000625 0.302699 3.93E-19 -0.00206475 -484.319 0 down vs 1 -9.86471 97.3125 1.66814 3.63413 1cg04057288 SH2D3C -0.05056 0.221519 4.00E-19 -0.228243 -4.3813 0 down vs 1 -9.86208 97.2606 1.34217 2.92554 1cg27645498 -0.06728 0.213151 4.06E-19 -0.315645 -3.16811 0 down vs 1 -9.86005 97.2206 1.42583 3.10918 1cg06899551 FILIP1 -0.11378 0.286465 4.12E-19 -0.397186 -2.51771 0 down vs 1 -9.85776 97.1754 2.9045 6.33651 1cg27433031 HDAC4 0.12876 0.385125 4.16E-19 0.334333 -2.99103 0 down vs 1 -9.85653 97.1511 1.19161 2.60029 1cg13009869 0.02348 0.294649 4.19E-19 0.079688 -12.5489 0 down vs 1 -9.85543 97.1295 1.33321 2.90993 1cg00068316 0.260965 0.427469 4.19E-19 0.610489 -1.63803 0 down vs 1 -9.85522 97.1254 0.502649 1.09716 1cg12427469 KCNQ1 0.044305 0.298177 4.21E-19 0.148586 -6.73009 0 down vs 1 -9.85472 97.1155 1.16855 2.5509 1cg16489926 CDH19 0.114395 0.365689 4.25E-19 0.31282 -3.19672 0 down vs 1 -9.85336 97.0888 1.14494 2.50006 1cg00754357 ZCCHC11 -0.0638 0.302209 4.29E-19 -0.211112 -4.73682 0 down vs 1 -9.85202 97.0624 2.42886 5.30502 1cg10411221 HLA-DMB -0.02647 0.203698 4.37E-19 -0.129947 -7.69545 0 down vs 1 -9.84926 97.0079 0.960527 2.09913 1cg25991569 -0.332005 0.124676 4.44E-19 -2.66295 2.66295 0 up vs 1 -9.84704 96.9642 3.78132 8.26738 1cg06444755 NXPH1 -0.250025 0.0560294 4.49E-19 -4.46239 4.46239 0 up vs 1 -9.84514 96.9267 1.6983 3.71456 1cg24549277 -0.190785 0.194524 4.53E-19 -0.98078 -1.0196 0 down vs 1 -9.84387 96.9019 2.69176 5.88897 1cg07589972 NR3C1 0.148065 0.394239 4.55E-19 0.375572 -2.66261 0 down vs 1 -9.84333 96.8911 1.09876 2.40411 1cg13618529 NRG1 0.172185 0.415635 4.59E-19 0.41427 -2.41389 0 down vs 1 -9.84193 96.8635 1.07458 2.35187 1cg15683166 FAM24B 0.13014 0.384019 4.64E-19 0.338889 -2.95082 0 down vs 1 -9.84052 96.8358 1.16862 2.55842 1cg25357194 KRT72 0.18265 0.402481 4.69E-19 0.45381 -2.20356 0 down vs 1 -9.83897 96.8054 0.876184 1.91881 1cg24033224 GPR133 0.08413 0.405574 4.72E-19 0.207434 -4.8208 0 down vs 1 -9.83798 96.7858 1.87339 4.10349 1cg25980539 0.16377 0.40901 4.84E-19 0.400406 -2.49747 0 down vs 1 -9.83428 96.7131 1.09044 2.3903 1cg08849574 ZNF134 -0.318135 0.0975753 4.89E-19 -3.26041 3.26041 0 up vs 1 -9.83271 96.6821 3.13328 6.87051 1cg06897628 -0.35202 0.115132 4.98E-19 -3.05752 3.05752 0 up vs 1 -9.82996 96.6281 3.95672 8.68096 1cg22530232 PTPRN2 0.0044 0.300355 5.07E-19 0.0146493 -68.2625 0 down vs 1 -9.82746 96.579 1.58807 3.48596 1cg22524864 FXYD3 0.19044 -0.0978273 5.07E-19 -1.9467 1.9467 0 up vs 1 9.82731 96.576 1.50664 3.30732 1cg08444397 0.192785 0.398196 5.14E-19 0.484146 -2.06549 0 down vs 1 -9.82547 96.5399 0.765006 1.67994 1cg26792295 RFTN1 0.121085 0.332414 5.16E-19 0.364259 -2.7453 0 down vs 1 -9.82484 96.5275 0.809726 1.77837 1cg00700039 SMCHD1 -0.21149 -0.388166 5.17E-19 0.544845 -1.83539 0 down vs 1 9.84381 96.9005 0.564831 1.21243 1cg22604218 LOC100132111 0.021055 -0.378839 5.21E-19 -0.0555777 -17.9928 0 down vs 1 9.82331 96.4975 2.89939 6.36981 1cg00253379 -0.220645 0.155964 5.33E-19 -1.41472 1.41472 0 up vs 1 -9.82004 96.4332 2.57157 5.65338 1cg01425762 CMIP -0.064595 -0.314347 5.34E-19 0.20549 -4.86643 0 down vs 1 9.81984 96.4292 1.13093 2.48635 1cg03071124 PTPRN2 0.10421 0.373146 5.36E-19 0.279274 -3.58072 0 down vs 1 -9.8193 96.4187 1.31135 2.88331 1cg18425731 ERMN 0.142665 0.387774 5.40E-19 0.367907 -2.71808 0 down vs 1 -9.81821 96.3972 1.08927 2.39557 1cg13751138 0.066725 0.392589 5.41E-19 0.169962 -5.88368 0 down vs 1 -9.8226 96.4835 1.92433 4.20832 1cg14468408 PBX3 0.223925 0.426897 5.42E-19 0.524541 -1.90643 0 down vs 1 -9.81754 96.3841 0.746947 1.64293 1cg01093088 PGLYRP4 -0.063975 0.253912 5.57E-19 -0.251958 -3.96892 0 down vs 1 -9.81361 96.307 1.83216 4.03312 1cg13470673 ZBTB20 -0.03991 0.31241 5.61E-19 -0.127749 -7.82787 0 down vs 1 -9.81253 96.2857 2.25057 4.95527 1cg09510085 KIAA1609 0.164405 0.399553 5.62E-19 0.411472 -2.4303 0 down vs 1 -9.81242 96.2836 1.00254 2.20742 1cg15730491 -0.164185 0.257006 5.62E-19 -0.638837 -1.56535 0 down vs 1 -9.81236 96.2824 3.21645 7.08216 1cg19708432 DNER 0.05461 0.324662 5.65E-19 0.168206 -5.9451 0 down vs 1 -9.81152 96.2659 1.32225 2.9119 1cg12004787 FGF19 -0.18594 0.223959 5.66E-19 -0.83024 -1.20447 0 down vs 1 -9.81122 96.26 3.0463 6.70907 1cg24103040 GRIP1 -0.141915 0.237212 5.73E-19 -0.598263 -1.67151 0 down vs 1 -9.80947 96.2257 2.60608 5.74159 1cg20092122 BST2 -0.052265 -0.338823 5.82E-19 0.154255 -6.48278 0 down vs 1 9.80724 96.1819 1.48882 3.28159 1cg04154986 LOC400578 -0.012115 0.217947 5.84E-19 -0.0555868 -17.9899 0 down vs 1 -9.80681 96.1734 0.959642 2.11539 1cg09017434 11-Mar -0.134365 0.149948 5.91E-19 -0.896075 -1.11598 0 down vs 1 -9.80496 96.1373 1.46559 3.23189 1cg00089091 DPP10 -0.140265 0.197444 5.98E-19 -0.710403 -1.40765 0 down vs 1 -9.8032 96.1027 2.06778 4.56147 1cg20453845 ABLIM2 0.15099 0.358036 5.99E-19 0.421718 -2.37125 0 down vs 1 -9.80306 96.0999 0.77723 1.7146 1cg09072859 OPCML -0.33328 -0.019068 6.07E-19 17.4785 17.4785 0 up vs 1 -9.80108 96.0611 1.79004 3.9505 1cg03368099 HOXA5 -0.006645 0.258782 6.16E-19 -0.0256779 -38.9439 0 down vs 1 -9.7989 96.0185 1.27735 2.82027 1cg06880923 PRL 0.17364 0.356931 6.21E-19 0.486481 -2.05558 0 down vs 1 -9.7977 95.9948 0.609116 1.3452 1cg02752812 RPS6KA2 0.16555 0.401899 6.23E-19 0.411919 -2.42766 0 down vs 1 -9.79731 95.9874 1.0128 2.2369 1cg01100175 KIF5C -0.262405 0.013267 6.25E-19 -19.7788 19.7788 0 up vs 1 -9.79682 95.9777 1.37785 3.04347 1cg19392994 IQCH 0.24084 0.393288 6.32E-19 0.612376 -1.63298 0 down vs 1 -9.79508 95.9437 0.421366 0.931062 1cg16785381 CSMD1 -0.005205 0.333081 6.36E-19 -0.0156268 -63.9925 0 down vs 1 -9.79414 95.9252 2.07485 4.58552 1cg02988117 0.12979 0.394636 6.56E-19 0.328885 -3.04057 0 down vs 1 -9.78966 95.8374 1.27176 2.81324 1cg05616122 TBC1D16 0.153585 0.354253 6.58E-19 0.433547 -2.30656 0 down vs 1 -9.78926 95.8297 0.730083 1.61513 1cg05541367 ADARB2 0.21335 0.418616 6.62E-19 0.509656 -1.96211 0 down vs 1 -9.78834 95.8117 0.763927 1.69032 1cg10215763 C7orf34 0.09172 0.370015 6.68E-19 0.247882 -4.03418 0 down vs 1 -9.78686 95.7826 1.4042 3.10798 1cg16904348 0.038005 0.324807 6.71E-19 0.117008 -8.54642 0 down vs 1 -9.78635 95.7727 1.49136 3.30123 1cg23009044 PIK3R6 0.042905 0.390302 6.84E-19 0.109928 -9.09688 0 down vs 1 -9.78357 95.7183 2.18811 4.8463 1cg06193239 0.02439 0.262028 6.86E-19 0.0930815 -10.7433 0 down vs 1 -9.78298 95.7067 1.02388 2.26801 1cg23647157 0.171365 0.355761 6.87E-19 0.481685 -2.07604 0 down vs 1 -9.78285 95.7041 0.616485 1.36561 1cg27407935 SREBF1 -0.204245 -0.385283 6.88E-19 0.530117 -1.88638 0 down vs 1 9.78263 95.6999 0.594234 1.31638 1cg04710830 0.102005 0.373963 6.91E-19 0.272768 -3.66612 0 down vs 1 -9.78203 95.6881 1.34098 2.97098 1cg14938419 TBXA2R 0.198075 0.443332 7.12E-19 0.446787 -2.23821 0 down vs 1 -9.77764 95.6023 1.09059 2.41841 1cg02926769 CBFA2T3 0.076315 0.349198 7.15E-19 0.218543 -4.57575 0 down vs 1 -9.77702 95.5901 1.35012 2.9943 1cg26494610 0.04114 0.300604 7.16E-19 0.136858 -7.30685 0 down vs 1 -9.77673 95.5844 1.22059 2.7072 1cg14711743 SERINC5 0.1516 0.398343 7.20E-19 0.380576 -2.62759 0 down vs 1 -9.77586 95.5673 1.10385 2.4487 1cg14519598 PAX5 0.17059 0.371687 7.37E-19 0.458962 -2.17883 0 down vs 1 -9.7725 95.5018 0.733208 1.62762 1cg22023826 BMP5 -0.06333 0.313209 7.41E-19 -0.202197 -4.94566 0 down vs 1 -9.77182 95.4884 2.57062 5.70719 1cg07806674 IFI6 0.201055 0.421636 7.58E-19 0.476845 -2.09712 0 down vs 1 -9.76838 95.4213 0.882174 1.95995 1cg13239420 -0.22208 0.158818 7.71E-19 -1.39833 1.39833 0 up vs 1 -9.76587 95.3723 2.63048 5.8472 1cg00469240 CACNA1H 0.10079 0.410779 7.73E-19 0.245363 -4.07559 0 down vs 1 -9.76542 95.3635 1.74225 3.87315 1cg20925811 MMP9 0.02257 0.328016 7.81E-19 0.0688076 -14.5333 0 down vs 1 -9.76401 95.3359 1.69156 3.76155 1cg07840143 0.19453 0.381247 7.87E-19 0.510246 -1.95984 0 down vs 1 -9.76293 95.3148 0.632103 1.40593 1cg12461287 GRAMD1B -0.285035 0.040067 7.91E-19 -7.11396 7.11396 0 up vs 1 -9.76204 95.2975 1.91627 4.26297 1cg09917805 0.033845 0.274101 7.97E-19 0.123476 -8.09872 0 down vs 1 -9.761 95.2772 1.04657 2.3287 1cg26733846 0.04255 0.354042 8.03E-19 0.120183 -8.32062 0 down vs 1 -9.75984 95.2545 1.75919 3.91528 1cg02954194 HLA-DOB 0.114025 0.323219 8.10E-19 0.352779 -2.83463 0 down vs 1 -9.75868 95.2318 0.793445 1.76633 1cg02655824 RPS6KA2 0.23909 0.443542 8.16E-19 0.539047 -1.85512 0 down vs 1 -9.75752 95.2091 0.757879 1.68755 1cg25344144 WHAMML1 0.15187 0.349165 8.22E-19 0.434951 -2.29911 0 down vs 1 -9.7564 95.1873 0.705752 1.57184 1cg11360755 SLC9A3 0.05693 0.335768 8.49E-19 0.169552 -5.8979 0 down vs 1 -9.75167 95.095 1.40968 3.14267 1cg22336004 PDE4B -0.18725 0.157043 8.52E-19 -1.19235 1.19235 0 up vs 1 -9.75113 95.0846 2.14918 4.79181 1cg04157587 -0.10968 0.204365 8.63E-19 -0.536687 -1.86328 0 down vs 1 -9.74939 95.0506 1.78814 3.98825 1cg25166006 0.158695 0.443098 8.81E-19 0.358148 -2.79214 0 down vs 1 -9.74621 94.9887 1.46652 3.27304 1cg26153410 -0.323355 0.129357 9.01E-19 -2.49971 2.49971 0 up vs 1 -9.74299 94.9258 3.71589 8.29877 1cg26382551 CUGBP2 0.033175 0.342492 9.23E-19 0.0968636 -10.3238 0 down vs 1 -9.73946 94.857 1.7347 3.87696 1cg01601760 0.06063 0.319069 9.48E-19 0.190022 -5.26256 0 down vs 1 -9.7355 94.78 1.21097 2.70865 1cg26371320 -0.188425 0.214436 9.52E-19 -0.8787 -1.13804 0 down vs 1 -9.73483 94.7668 2.94258 6.58276 1cg22900206 FAM171B 0.119925 0.332502 9.55E-19 0.360674 -2.77258 0 down vs 1 -9.73447 94.76 0.819315 1.833 1cg15640734 SLC9A3 0.32529 0.480289 9.57E-19 0.677279 -1.4765 0 down vs 1 -9.73408 94.7523 0.435589 0.974592 1cg00360414 TMEM26 -0.285385 0.112721 9.61E-19 -2.53179 2.53179 0 up vs 1 -9.73356 94.7422 2.87352 6.42994 1cg14213563 SOX6 0.20402 0.401443 9.68E-19 0.508217 -1.96766 0 down vs 1 -9.7325 94.7215 0.706663 1.58161 1cg24150032 0.07455 0.412421 9.76E-19 0.180762 -5.53213 0 down vs 1 -9.73119 94.6961 2.06975 4.63364 1cg12485572 0.01295 0.356424 9.77E-19 0.0363331 -27.5231 0 down vs 1 -9.73114 94.6951 2.13898 4.78866 1cg19205041 PHACTR2 0.109145 0.407475 9.80E-19 0.267857 -3.73333 0 down vs 1 -9.73055 94.6836 1.61366 3.61304 1cg21140628 0.09965 0.345935 9.82E-19 0.28806 -3.4715 0 down vs 1 -9.73036 94.68 1.09975 2.46248 1cg21275690 HCFC1R1 -0.077945 -0.244789 9.85E-19 0.318417 -3.14054 0 down vs 1 9.72993 94.6716 0.504708 1.1302 1cg22618924 0.08198 0.314913 9.87E-19 0.260326 -3.84134 0 down vs 1 -9.72956 94.6643 0.983743 2.20308 1cg17918280 PLCH2 0.23995 0.454038 9.93E-19 0.52848 -1.89222 0 down vs 1 -9.72872 94.6479 0.831 1.86134 1cg17247026 -0.038815 0.402469 9.97E-19 -0.0964422 -10.3689 0 down vs 1 -9.72808 94.6355 3.53065 7.90927 1cg24328125 -0.289735 -0.0265196 1.01E-18 10.9253 10.9253 0 up vs 1 -9.72588 94.5928 1.25615 2.81526 1cg04479219 SLITRK5 -0.285465 0.0857469 1.02E-18 -3.32916 3.32916 0 up vs 1 -9.72488 94.5732 2.4984 5.60053 1cg22939456 0.199895 0.37961 1.04E-18 0.526579 -1.89905 0 down vs 1 -9.72189 94.5152 0.585583 1.31348 1cg09501959 TMEFF2 -0.025435 0.314355 1.08E-18 -0.0809117 -12.3592 0 down vs 1 -9.7168 94.4161 2.09334 4.70034 1cg01654770 -0.013975 0.277602 1.10E-18 -0.0503418 -19.8642 0 down vs 1 -9.71419 94.3655 1.54143 3.46296 1cg03379681 P2RY12 -0.03867 0.283205 1.10E-18 -0.136544 -7.32364 0 down vs 1 -9.71322 94.3466 1.87842 4.22088 1cg15125666 -0.067505 0.281944 1.11E-18 -0.239427 -4.17664 0 down vs 1 -9.71184 94.3197 2.21404 4.97644 1cg09786269 0.24081 0.408247 1.12E-18 0.589863 -1.69531 0 down vs 1 -9.7115 94.3132 0.508303 1.14258 1cg19929638 -0.19626 0.250668 1.13E-18 -0.782948 -1.27722 0 down vs 1 -9.71002 94.2845 3.62154 8.14308 1cg23756251 PEX5L -0.279935 0.0517773 1.14E-18 -5.40652 5.40652 0 up vs 1 -9.70852 94.2554 1.99499 4.48715 1cg09238726 0.107725 0.368297 1.14E-18 0.292495 -3.41886 0 down vs 1 -9.70796 94.2444 1.23104 2.76919 1cg22682172 -0.084415 0.271598 1.16E-18 -0.310809 -3.21741 0 down vs 1 -9.70624 94.211 2.298 5.17111 1cg19035496 CRH -0.01077 0.265435 1.17E-18 -0.040575 -24.6457 0 down vs 1 -9.70422 94.1719 1.38318 3.11382 1cg21868134 HPDL -0.241745 -0.482124 1.18E-18 0.501416 -1.99435 0 down vs 1 9.70345 94.1569 1.04764 2.35882 1cg27535410 PRTN3 0.156605 0.439484 1.19E-18 0.356339 -2.80632 0 down vs 1 -9.70207 94.1301 1.45083 3.26757 1cg20669572 CACNA1B 0.09465 0.325159 1.20E-18 0.291089 -3.43538 0 down vs 1 -9.70061 94.1019 0.963369 2.17035 1cg11703632 -0.30871 0.0878711 1.21E-18 -3.51321 3.51321 0 up vs 1 -9.70016 94.0932 2.85156 6.4248 1cg06627334 PCDH12 0.12524 0.419172 1.21E-18 0.298779 -3.34695 0 down vs 1 -9.69923 94.075 1.56643 3.52999 1cg14426428 AIM1 -0.201175 -0.386346 1.25E-18 0.520712 -1.92045 0 down vs 1 9.69495 93.9921 0.621675 1.40219 1cg00101712 ROPN1 -0.17433 0.213384 1.25E-18 -0.81698 -1.22402 0 down vs 1 -9.69451 93.9834 2.72546 6.14787 1cg12584355 SERPINE1 0.16784 0.354148 1.32E-18 0.473926 -2.11003 0 down vs 1 -9.68688 93.8356 0.629333 1.42183 1cg13278004 SP140L -0.31886 -0.450064 1.32E-18 0.708477 -1.41148 0 down vs 1 9.68669 93.8319 0.312113 0.705175 1cg18442362 OGDH -0.11581 -0.402462 1.33E-18 0.287754 -3.4752 0 down vs 1 9.68612 93.8208 1.4898 3.3664 1cg22243628 0.042545 0.355266 1.33E-18 0.119755 -8.35037 0 down vs 1 -9.68526 93.8043 1.7731 4.00725 1cg15646123 CDKAL1 0.14177 0.351912 1.36E-18 0.402856 -2.48228 0 down vs 1 -9.68236 93.7481 0.800655 1.81058 1cg26292173 C1QTNF1 0.0681 0.310883 1.39E-18 0.219053 -4.5651 0 down vs 1 -9.67925 93.6879 1.0687 2.41828 1cg04075191 DPP10 -0.1814 0.155974 1.40E-18 -1.16301 1.16301 0 up vs 1 -9.67795 93.6627 2.06368 4.67101 1cg07665575 RAG1 0.00937 0.299416 1.44E-18 0.0312942 -31.9548 0 down vs 1 -9.67356 93.5777 1.52529 3.45555 1cg01047586 -0.161395 0.193177 1.47E-18 -0.835476 -1.19692 0 down vs 1 -9.67094 93.5271 2.27944 5.16685 1cg06716419 0.04422 0.306226 1.48E-18 0.144403 -6.92506 0 down vs 1 -9.67005 93.5098 1.24463 2.82176 1cg00625949 MGAT4C 0.156375 0.378144 1.49E-18 0.413533 -2.41819 0 down vs 1 -9.66895 93.4885 0.8917 2.02207 1cg15576869 IKZF1 0.096805 0.320572 1.50E-18 0.301976 -3.31152 0 down vs 1 -9.66837 93.4774 0.907839 2.05891 1cg24915620 0.266135 0.40226 1.50E-18 0.6616 -1.51149 0 down vs 1 -9.66748 93.4601 0.335964 0.762082 1cg09782903 ACCN3 0.21809 0.396903 1.51E-18 0.549479 -1.81991 0 down vs 1 -9.66687 93.4483 0.579718 1.31517 1cg01104717 CACNA1D 0.21272 0.396472 1.51E-18 0.536533 -1.86382 0 down vs 1 -9.66684 93.4479 0.612182 1.38882 1cg12851635 KCNQ1 0.1445 0.405105 1.51E-18 0.356698 -2.80349 0 down vs 1 -9.66678 93.4466 1.23135 2.79354 1cg02850689 -0.258945 0.0544304 1.51E-18 -4.75736 4.75736 0 up vs 1 -9.66657 93.4427 1.78052 4.0396 1cg06800421 CPEB3 0.316325 0.426182 1.52E-18 0.742229 -1.34729 0 down vs 1 -9.66579 93.4275 0.218815 0.496522 1cg06511389 TMEM101 -0.06106 -0.445259 1.52E-18 0.137134 -7.29215 0 down vs 1 9.66558 93.4235 2.67627 6.07309 1cg12954755 -0.007925 0.293716 1.53E-18 -0.0269818 -37.062 0 down vs 1 -9.66507 93.4137 1.64968 3.74391 1cg16145211 0.16691 0.457698 1.53E-18 0.364673 -2.74218 0 down vs 1 -9.66479 93.4082 1.5331 3.47954 1cg23053506 PTPRN2 0.06338 0.383942 1.54E-18 0.165077 -6.05778 0 down vs 1 -9.66374 93.3878 1.86313 4.22949 1cg22719623 OPRM1 -0.14721 0.165915 1.55E-18 -0.887259 -1.12707 0 down vs 1 -9.66344 93.382 1.77768 4.03578 1cg07068768 CRYBB3 0.100225 0.392871 1.55E-18 0.255109 -3.91989 0 down vs 1 -9.66269 93.3675 1.55275 3.52567 1cg17841421 0.205445 0.387563 1.56E-18 0.530095 -1.88646 0 down vs 1 -9.66183 93.351 0.601344 1.36565 1cg19256474 -0.046135 0.269559 1.58E-18 -0.17115 -5.84283 0 down vs 1 -9.65997 93.3149 1.80697 4.1052 1cg11975793 KCNE4 0.07812 0.360041 1.60E-18 0.216975 -4.60882 0 down vs 1 -9.65847 93.286 1.44103 3.27485 1cg04027043 -0.12485 0.25713 1.62E-18 -0.485552 -2.05951 0 down vs 1 -9.65615 93.2412 2.64545 6.01488 1cg16649560 IL4R -0.048485 -0.322463 1.62E-18 0.150358 -6.65078 0 down vs 1 9.65614 93.241 1.36097 3.09441 1cg01464835 VSTM2B -0.273525 0.0759773 1.63E-18 -3.60009 3.60009 0 up vs 1 -9.65538 93.2263 2.21472 5.03635 1cg04948286 TBXA2R 0.073515 0.34973 1.64E-18 0.210205 -4.75727 0 down vs 1 -9.65484 93.2159 1.38329 3.14601 1cg19782061 -0.30242 0.111727 1.65E-18 -2.70677 2.70677 0 up vs 1 -9.65414 93.2025 3.10977 7.07353 1cg06350196 PLCH2 0.1386 0.43022 1.67E-18 0.322161 -3.10404 0 down vs 1 -9.65224 93.1657 1.54189 3.50859 1cg20263510 SORCS2 0.27698 0.421197 1.71E-18 0.657601 -1.52068 0 down vs 1 -9.64857 93.0948 0.377097 0.858744 1cg02469838 -0.25581 0.134366 1.71E-18 -1.90383 1.90383 0 up vs 1 -9.64837 93.091 2.76019 6.2859 1cg23776522 TNXB 0.020095 0.391845 1.71E-18 0.0512831 -19.4996 0 down vs 1 -9.64823 93.0884 2.50565 5.70637 1cg01877524 DSCAML1 0.182725 0.467944 1.72E-18 0.390485 -2.56092 0 down vs 1 -9.64781 93.0802 1.47494 3.35933 1cg23524354 -0.057925 0.272873 1.74E-18 -0.212278 -4.7108 0 down vs 1 -9.64594 93.0442 1.98401 4.52055 1cg23333920 ATP11A 0.183955 0.385485 1.76E-18 0.477204 -2.09554 0 down vs 1 -9.64852 93.094 0.736016 1.6682 1cg09133892 0.088735 0.386734 1.78E-18 0.229447 -4.3583 0 down vs 1 -9.64262 92.9801 1.61008 3.67106 1cg08178991 ITGAX 0.288815 0.46676 1.78E-18 0.618766 -1.61612 0 down vs 1 -9.64253 92.9784 0.574101 1.30901 1cg09080087 MLLT4 0.21614 0.382804 1.78E-18 0.564624 -1.77109 0 down vs 1 -9.64227 92.9733 0.503616 1.14836 1cg18286501 TMEM26 -0.23767 0.248345 1.79E-18 -0.957016 -1.04491 0 down vs 1 -9.64186 92.9655 4.28269 9.76633 1cg01274233 ADAMTS2 -0.25019 -0.0871696 1.81E-18 2.87015 2.87015 0 up vs 1 -9.64034 92.9361 0.481839 1.09914 1cg06290221 0.169605 0.361123 1.81E-18 0.46966 -2.1292 0 down vs 1 -9.64023 92.934 0.665025 1.51705 1cg24567424 FAM155A -0.24162 0.124629 1.82E-18 -1.93872 1.93872 0 up vs 1 -9.63977 92.9251 2.43204 5.54847 1cg17008412 0.155425 0.403043 1.84E-18 0.385629 -2.59317 0 down vs 1 -9.63788 92.8888 1.11169 2.53721 1cg08549573 SRRM3 0.039845 0.260948 1.85E-18 0.152693 -6.54908 0 down vs 1 -9.63709 92.8734 0.886354 2.02326 1cg16835233 ZNF280B -0.23489 0.119779 1.86E-18 -1.96103 1.96103 0 up vs 1 -9.63606 92.8536 2.28068 5.20716 1cg22605936 HIP1 0.329175 0.447293 1.89E-18 0.735927 -1.35883 0 down vs 1 -9.63401 92.8141 0.252958 0.57779 1cg13939462 PPIL1 0.14174 0.410095 1.90E-18 0.345627 -2.89329 0 down vs 1 -9.63284 92.7917 1.30569 2.98308 1cg04073265 SLC6A3 0.176245 0.456051 1.92E-18 0.386459 -2.58759 0 down vs 1 -9.63171 92.7698 1.41948 3.24384 1cg08319778 PSAPL1 0.234625 0.457336 1.94E-18 0.513026 -1.94922 0 down vs 1 -9.62975 92.732 0.89929 2.05592 1cg07768501 FAM20C 0.086465 0.359019 1.94E-18 0.240837 -4.15219 0 down vs 1 -9.62975 92.732 1.34686 3.07914 1cg12302070 KCNH7 -0.234695 0.16378 1.94E-18 -1.43299 1.43299 0 up vs 1 -9.62959 92.729 2.87886 6.58175 1cg17811213 GALNT9 0.297645 0.462208 1.96E-18 0.643963 -1.55288 0 down vs 1 -9.62818 92.7019 0.491002 1.12287 1cg00149716 0.021645 0.307066 1.97E-18 0.0704897 -14.1865 0 down vs 1 -9.6279 92.6965 1.47703 3.37802 1cg05468953 0.000665 0.318799 1.97E-18 0.00208595 -479.398 0 down vs 1 -9.62778 92.6941 1.83501 4.19685 1cg25095994 SP140L -0.152195 -0.410393 2.05E-18 0.370852 -2.6965 0 down vs 1 9.62179 92.5789 1.20872 2.76789 1cg04246521 CLDN10 0.29685 0.448635 2.05E-18 0.661674 -1.51132 0 down vs 1 -9.62174 92.5779 0.417708 0.956537 1cg11596387 0.27947 0.414835 2.05E-18 0.67369 -1.48436 0 down vs 1 -9.6217 92.5771 0.332222 0.760782 1cg04118062 GALNT9 0.19847 0.416049 2.05E-18 0.477035 -2.09628 0 down vs 1 -9.62161 92.5754 0.858325 1.96559 1cg19209911 0.048705 0.410191 2.07E-18 0.118737 -8.42195 0 down vs 1 -9.62065 92.5569 2.3692 5.42661 1cg02495310 PITX1 -0.041885 -0.395299 2.09E-18 0.105958 -9.43772 0 down vs 1 9.61906 92.5264 2.26457 5.18867 1cg06976598 PRKG1 -0.23112 -0.418171 2.09E-18 0.552693 -1.80932 0 down vs 1 9.61877 92.5208 0.634361 1.45356 1cg07660114 ADARB2 0.092645 0.352938 2.11E-18 0.262497 -3.80957 0 down vs 1 -9.61732 92.4929 1.22841 2.81559 1cg00476243 -0.13632 -0.411207 2.12E-18 0.331512 -3.01648 0 down vs 1 9.61686 92.4839 1.37002 3.14047 1cg12749132 LOC100132111 0.001495 -0.419919 2.15E-18 -0.00356021 -280.882 0 down vs 1 9.61454 92.4393 3.21985 7.3844 1cg13138832 BTNL2 0.14065 0.373976 2.21E-18 0.376094 -2.65891 0 down vs 1 -9.61036 92.3591 0.98706 2.26569 1cg12004295 FAM189A1 -0.2807 0.13763 2.22E-18 -2.03953 2.03953 0 up vs 1 -9.61012 92.3545 3.1729 7.28339 1cg13840968 LTB4R 0.031315 -0.212868 2.23E-18 -0.14711 -6.79762 0 down vs 1 9.60961 92.3447 1.08105 2.48183 1cg24127414 PCDHB11 0.02921 0.344481 2.26E-18 0.0847942 -11.7933 0 down vs 1 -9.60725 92.2993 1.80213 4.13926 1cg24564096 SORCS2 0.163495 0.415496 2.29E-18 0.393493 -2.54134 0 down vs 1 -9.60521 92.2601 1.15139 2.64573 1cg27422407 0.16518 0.404097 2.30E-18 0.408763 -2.44641 0 down vs 1 -9.60497 92.2554 1.03494 2.37825 1cg17251713 SERPINB7 -0.095355 0.289984 2.31E-18 -0.328829 -3.04109 0 down vs 1 -9.60426 92.2417 2.69217 6.18744 1cg10710457 PFKP 0.28281 0.447152 2.31E-18 0.632469 -1.5811 0 down vs 1 -9.60403 92.2374 0.489683 1.1255 1cg09680055 CRYBB2 0.20786 0.397369 2.33E-18 0.523091 -1.91171 0 down vs 1 -9.60754 92.3048 0.650829 1.48773 1cg01826354 B3GNTL1 -0.22101 -0.412454 2.34E-18 0.535841 -1.86622 0 down vs 1 9.60241 92.2062 0.664511 1.52784 1cg17380795 0.243115 0.465651 2.36E-18 0.522097 -1.91535 0 down vs 1 -9.60072 92.1739 0.897881 2.06513 1cg13337047 -0.307435 0.120094 2.38E-18 -2.55996 2.55996 0 up vs 1 -9.5995 92.1504 3.31397 7.62408 1cg03932361 RAB37 -0.264605 0.0502273 2.38E-18 -5.26815 5.26815 0 up vs 1 -9.59948 92.15 1.79712 4.13444 1cg03805492 -0.106935 0.269618 2.40E-18 -0.396617 -2.52133 0 down vs 1 -9.59857 92.1326 2.57081 5.91552 1cg00629514 -0.2551 0.165628 2.40E-18 -1.5402 1.5402 0 up vs 1 -9.5983 92.1274 3.20938 7.38531 1cg25306511 PXDN 0.201515 0.361632 2.42E-18 0.557237 -1.79457 0 down vs 1 -9.59703 92.1029 0.464831 1.06994 1cg17712241 0.139395 0.382895 2.45E-18 0.364056 -2.74683 0 down vs 1 -9.5953 92.0697 1.07502 2.47534 1cg06160682 KCND3 0.041385 0.350412 2.46E-18 0.118104 -8.46713 0 down vs 1 -9.59481 92.0603 1.73146 3.98727 1cg05383490 FAM155A -0.29118 0.0341763 2.46E-18 -8.51994 8.51994 0 up vs 1 -9.59467 92.0578 1.91927 4.41989 1cg03131729 0.116895 0.374235 2.47E-18 0.312357 -3.20146 0 down vs 1 -9.59414 92.0476 1.2007 2.76539 1cg07658449 CUX2 -0.03755 0.387962 2.52E-18 -0.0967877 -10.3319 0 down vs 1 -9.59105 91.9883 3.28278 7.56564 1cg00258081 -0.08713 0.274542 2.53E-18 -0.317365 -3.15094 0 down vs 1 -9.59057 91.979 2.37163 5.46631 1cg10978966 0.278655 0.420655 2.54E-18 0.662431 -1.50959 0 down vs 1 -9.59002 91.9684 0.365591 0.842738 1cg27086758 MCTP2 0.2902 0.436446 2.57E-18 0.664916 -1.50395 0 down vs 1 -9.58859 91.941 0.38778 0.894153 1cg04944283 ZNF311 0.175085 0.377262 2.58E-18 0.464094 -2.15474 0 down vs 1 -9.58788 91.9274 0.741107 1.70912 1cg15553958 0.077765 0.311329 2.58E-18 0.249784 -4.00346 0 down vs 1 -9.58776 91.9252 0.989079 2.28104 1cg03643948 UMODL1 -0.001785 0.390826 2.59E-18 -0.00456724 -218.95 0 down vs 1 -9.59158 91.9984 2.7934 6.40672 1cg12544663 0.15478 0.449292 2.65E-18 0.344497 -2.90278 0 down vs 1 -9.58383 91.8499 1.57262 3.6298 1cg07640336 0.072795 0.40578 2.65E-18 0.179395 -5.57428 0 down vs 1 -9.58365 91.8464 2.01033 4.64024 1cg22624278 0.072005 0.295015 2.65E-18 0.244072 -4.09715 0 down vs 1 -9.58356 91.8446 0.901713 2.08138 1cg12817107 0.239025 0.408809 2.72E-18 0.584686 -1.71032 0 down vs 1 -9.58015 91.7793 0.522652 1.20727 1cg26679855 -0.001825 0.360108 2.72E-18 -0.00506793 -197.319 0 down vs 1 -9.57971 91.7708 2.37506 5.48662 1cg00745389 -0.28048 0.0798052 2.73E-18 -3.51456 3.51456 0 up vs 1 -9.57921 91.7613 2.35348 5.43735 1cg16621196 0.119515 0.397303 2.78E-18 0.300816 -3.32429 0 down vs 1 -9.57661 91.7115 1.39909 3.23413 1cg11829371 ABR 0.276605 0.464618 2.80E-18 0.595338 -1.67972 0 down vs 1 -9.57564 91.6928 0.640905 1.48182 1cg18760534 0.105855 0.426435 2.81E-18 0.248233 -4.02848 0 down vs 1 -9.57512 91.6829 1.86333 4.30861 1cg16662451 FBXW10 0.2571 0.412521 2.82E-18 0.623242 -1.60451 0 down vs 1 -9.57459 91.6727 0.437961 1.01282 1cg17250160 0.00655 0.301611 2.87E-18 0.0217167 -46.0475 0 down vs 1 -9.57201 91.6234 1.57849 3.65234 1cg07532782 JARID2 0.385565 0.44582 2.87E-18 0.864844 -1.15628 0 down vs 1 -9.57192 91.6216 0.0658272 0.152315 1cg22264409 PROK2 -0.011435 0.284484 2.88E-18 -0.0401956 -24.8784 0 down vs 1 -9.57168 91.617 1.58768 3.67387 1cg22642498 NLRP12 0.10589 0.360725 2.88E-18 0.293548 -3.4066 0 down vs 1 -9.57127 91.6092 1.17744 2.7248 1cg21341008 0.247605 0.470901 2.91E-18 0.525812 -1.90182 0 down vs 1 -9.56992 91.5834 0.90402 2.09265 1cg09676860 RAB6C -0.32951 0.0148923 2.92E-18 -22.1262 22.1262 0 up vs 1 -9.56935 91.5726 2.15055 4.97875 1cg26010655 0.11473 0.367139 2.97E-18 0.312497 -3.20003 0 down vs 1 -9.56717 91.5308 1.15512 2.67545 1cg19961800 SYT16 0.05844 0.390597 2.97E-18 0.149617 -6.68373 0 down vs 1 -9.56698 91.5271 2.00035 4.63332 1cg04402693 PTPN13 0.22222 0.415448 2.98E-18 0.534892 -1.86954 0 down vs 1 -9.56652 91.5183 0.676955 1.56815 1cg03585734 FHAD1 0.046185 0.320059 3.02E-18 0.144302 -6.92993 0 down vs 1 -9.56459 91.4813 1.35994 3.15154 1cg22611850 0.17405 0.408219 3.03E-18 0.426364 -2.34541 0 down vs 1 -9.56406 91.4713 0.994207 2.30424 1cg09323657 CACNA2D4 -0.23828 -0.420422 3.03E-18 0.566764 -1.7644 0 down vs 1 9.56385 91.4672 0.601501 1.39414 1cg27028202 0.154735 0.419641 3.03E-18 0.368732 -2.712 0 down vs 1 -9.5638 91.4662 1.27234 2.94902 1cg08821431 -0.066985 0.24209 3.05E-18 -0.276694 -3.6141 0 down vs 1 -9.56287 91.4485 1.73199 4.01518 1cg09043456 BAIAP2 0.00384 -0.215695 3.09E-18 -0.0178029 -56.1707 0 down vs 1 9.56132 91.4189 0.87383 2.02641 1cg01548743 C14orf184 0.07907 0.377904 3.16E-18 0.209233 -4.77936 0 down vs 1 -9.55793 91.3541 1.61911 3.75738 1cg19255477 LOC100132111 0.044315 -0.394323 3.20E-18 -0.112383 -8.89818 0 down vs 1 9.55578 91.3129 3.48843 8.09904 1cg13024590 HSPA1A -0.236225 -0.463675 3.23E-18 0.509462 -1.96285 0 down vs 1 9.55885 91.3716 0.937519 2.16497 1cg24301290 -0.23993 0.0176985 3.26E-18 -13.5566 13.5566 0 up vs 1 -9.55334 91.2664 1.20339 2.79531 1cg02945385 SRGAP3 0.20927 0.443507 3.30E-18 0.471853 -2.1193 0 down vs 1 -9.55114 91.2243 0.994782 2.31181 1cg01200651 ABLIM2 0.15482 0.382873 3.41E-18 0.404364 -2.47302 0 down vs 1 -9.54641 91.134 0.942954 2.19354 1cg17012160 FMN2 -0.23573 0.0512479 3.47E-18 -4.59979 4.59979 0 up vs 1 -9.54395 91.087 1.49319 3.47532 1cg06595211 0.18414 0.424414 3.47E-18 0.433868 -2.30485 0 down vs 1 -9.54382 91.0845 1.04673 2.43626 1cg19916212 EDNRB -0.261625 0.135964 3.47E-18 -1.92422 1.92422 0 up vs 1 -9.54371 91.0825 2.86608 6.67097 1cg08699206 0.05678 0.402071 3.48E-18 0.141219 -7.08121 0 down vs 1 -9.54367 91.0817 2.16167 5.03146 1cg25287372 0.2074 0.399112 3.51E-18 0.519653 -1.92436 0 down vs 1 -9.54218 91.0532 0.666374 1.55153 1cg15480367 CHGA -0.189135 0.124864 3.53E-18 -1.51472 1.51472 0 up vs 1 -9.54123 91.0351 1.78762 4.16296 1cg12847373 EDNRB -0.290755 0.0360088 3.55E-18 -8.07456 8.07456 0 up vs 1 -9.54071 91.0252 1.93591 4.50879 1cg15090509 THSD7A -0.21491 0.148498 3.57E-18 -1.44722 1.44722 0 up vs 1 -9.53961 91.0041 2.39446 5.57805 1cg10815835 -0.01175 0.204497 3.59E-18 -0.0574579 -17.404 0 down vs 1 -9.53873 90.9874 0.847852 1.97549 1cg08588180 CMAH -0.319195 -0.452191 3.63E-18 0.705886 -1.41666 0 down vs 1 9.53724 90.959 0.320696 0.747453 1cg08384657 0.216665 0.373498 3.74E-18 0.580097 -1.72385 0 down vs 1 -9.53291 90.8764 0.445957 1.04034 1cg25850181 SNED1 0.346475 0.475196 3.75E-18 0.72912 -1.37152 0 down vs 1 -9.53241 90.8668 0.300413 0.70089 1cg06940614 ZNF781 -0.233755 0.129583 3.81E-18 -1.8039 1.8039 0 up vs 1 -9.53004 90.8216 2.39353 5.5871 1cg00028336 SORBS2 0.190305 0.39106 3.82E-18 0.486638 -2.05491 0 down vs 1 -9.52974 90.8159 0.730722 1.70579 1cg18771659 SPRR1B 0.20387 0.396664 3.85E-18 0.513961 -1.94567 0 down vs 1 -9.52861 90.7943 0.673918 1.57356 1cg16643088 CSRNP1 -0.125475 -0.402543 3.91E-18 0.311706 -3.20816 0 down vs 1 9.52617 90.748 1.39185 3.25155 1cg22351924 IFI6 0.26392 0.439635 3.96E-18 0.600316 -1.66579 0 down vs 1 -9.52435 90.7133 0.559804 1.30828 1cg01530605 SOX2OT -0.154205 0.222022 3.99E-18 -0.694548 -1.43979 0 down vs 1 -9.52327 90.6926 2.56636 5.99905 1cg17485141 0.27066 0.40183 4.07E-18 0.673568 -1.48463 0 down vs 1 -9.52023 90.6349 0.311953 0.729676 1cg07997634 SLC25A13 0.204125 0.385035 4.10E-18 0.530147 -1.88627 0 down vs 1 -9.51926 90.6164 0.593391 1.38826 1cg06513819 0.06657 0.370547 4.15E-18 0.179653 -5.56628 0 down vs 1 -9.51743 90.5815 1.67533 3.921 1cg27445931 -0.03013 0.323386 4.20E-18 -0.0931705 -10.733 0 down vs 1 -9.5155 90.5447 2.26587 5.30527 1cg14120112 VWF 0.252515 0.403863 4.21E-18 0.625249 -1.59936 0 down vs 1 -9.51535 90.5419 0.415311 0.972434 1cg22393371 MBNL3 0.199125 0.398475 4.21E-18 0.499718 -2.00113 0 down vs 1 -9.51522 90.5395 0.720525 1.68712 1cg25422678 BRE 0.11774 0.311505 4.25E-18 0.377972 -2.6457 0 down vs 1 -9.51382 90.5128 0.680718 1.59438 1cg11324832 SOX2OT -0.10244 0.269459 4.30E-18 -0.380169 -2.63041 0 down vs 1 -9.51207 90.4794 2.50766 5.87563 1cg17889682 DYNC1I1 -0.142435 0.222445 4.31E-18 -0.640315 -1.56173 0 down vs 1 -9.51176 90.4735 2.4139 5.65631 1cg18128495 RAB24 0.140665 -0.155701 4.37E-18 -0.90343 -1.10689 0 down vs 1 9.50978 90.436 1.59248 3.7331 1cg08082507 GTF2IRD2P 0.13701 0.329898 4.39E-18 0.41531 -2.40784 0 down vs 1 -9.50915 90.4239 0.674572 1.58154 1cg02947253 ADORA3 0.03678 0.308405 4.39E-18 0.119259 -8.38513 0 down vs 1 -9.50903 90.4216 1.3377 3.13633 1cg00738934 -0.00644 0.321037 4.55E-18 -0.02006 -49.8505 0 down vs 1 -9.5037 90.3203 1.94437 4.56384 1cg17166262 -0.214295 0.198233 4.57E-18 -1.08103 1.08103 0 up vs 1 -9.50315 90.3099 3.08549 7.24311 1cg07673838 SDK1 0.226665 0.411543 4.57E-18 0.550768 -1.81565 0 down vs 1 -9.5031 90.309 0.619712 1.45477 1cg26577252 IGF1R -0.25282 -0.443778 4.57E-18 0.569699 -1.75531 0 down vs 1 9.50301 90.3072 0.661143 1.55206 1cg08928696 -0.228895 -0.425564 4.58E-18 0.537863 -1.85921 0 down vs 1 9.50267 90.3007 0.701277 1.64639 1cg27657537 MED15 -0.284155 -0.406063 4.64E-18 0.699781 -1.42902 0 down vs 1 9.50095 90.268 0.269452 0.632825 1cg02914800 GNG11 0.15796 0.396171 4.68E-18 0.398717 -2.50805 0 down vs 1 -9.49951 90.2407 1.02883 2.41699 1cg07310538 HSPA1A -0.24589 -0.462849 4.70E-18 0.531253 -1.88234 0 down vs 1 9.49898 90.2307 0.85344 2.00519 1cg07539601 0.115275 0.371933 4.93E-18 0.309935 -3.22648 0 down vs 1 -9.49192 90.0966 1.19434 2.81032 1cg03961510 -0.01388 0.395852 5.01E-18 -0.0350636 -28.5196 0 down vs 1 -9.48937 90.0481 3.04381 7.16604 1cg14202937 AMPD2 0.094655 0.307096 5.02E-18 0.308226 -3.24438 0 down vs 1 -9.48909 90.0429 0.818269 1.92656 1cg27464184 -0.362395 0.103562 5.03E-18 -3.49931 3.49931 0 up vs 1 -9.48884 90.0381 3.93649 9.2687 1cg08400319 SNED1 0.13758 0.371631 5.05E-18 0.370205 -2.7012 0 down vs 1 -9.48809 90.0238 0.993209 2.33894 1cg23291534 KCNV1 -0.31376 0.151917 5.07E-18 -2.06534 2.06534 0 up vs 1 -9.48757 90.014 3.93176 9.26005 1cg14636534 LOC645323 -0.034555 0.28148 5.10E-18 -0.122762 -8.14587 0 down vs 1 -9.48675 89.9984 1.81088 4.2657 1cg06493207 0.108225 0.308964 5.11E-18 0.350284 -2.85483 0 down vs 1 -9.48646 89.9929 0.730602 1.72111 1cg01288184 CABLES1 0.047245 -0.37226 5.12E-18 -0.126914 -7.87936 0 down vs 1 9.48614 89.9869 3.19075 7.51709 1cg09340279 SECTM1 0.00626 0.392952 5.14E-18 0.0159307 -62.7718 0 down vs 1 -9.48554 89.9754 2.71111 6.38792 1cg15381080 0.038375 0.361307 5.40E-18 0.106212 -9.41517 0 down vs 1 -9.47829 89.8381 1.89078 4.46186 1cg21901277 -0.316735 0.0623459 5.48E-18 -5.08029 5.08029 0 up vs 1 -9.47616 89.7977 2.60544 6.15109 1cg17457014 TTC22 0.10871 0.332438 5.49E-18 0.327009 -3.05802 0 down vs 1 -9.47571 89.7892 0.907522 2.14274 1cg20067338 0.1889 0.393227 5.53E-18 0.480384 -2.08167 0 down vs 1 -9.47472 89.7704 0.756953 1.7876 1cg25588844 TAF1B 0.01677 -0.309939 5.54E-18 -0.0541075 -18.4817 0 down vs 1 9.47451 89.7663 1.93526 4.57048 1cg13971603 NTNG2 0.11365 0.308561 5.59E-18 0.368323 -2.71501 0 down vs 1 -9.47313 89.7402 0.688795 1.62719 1cg03675047 -0.144505 0.184667 5.60E-18 -0.782517 -1.27793 0 down vs 1 -9.47286 89.735 1.96455 4.64128 1cg08797606 LOC645323 -0.44073 -0.127098 5.64E-18 3.46763 3.46763 0 up vs 1 -9.47181 89.7151 1.78344 4.21432 1cg27403635 KCNN2 -0.247905 0.191668 5.71E-18 -1.29341 1.29341 0 up vs 1 -9.46988 89.6786 3.50331 8.28183 1cg24628676 PDE4D -0.322435 0.0305474 5.76E-18 -10.5552 10.5552 0 up vs 1 -9.46869 89.6562 2.25904 5.3417 1cg23284720 -0.27593 0.0106582 5.80E-18 -25.8889 25.8889 0 up vs 1 -9.46769 89.6371 1.48914 3.52195 1cg00416206 0.179325 0.375485 5.99E-18 0.477583 -2.09388 0 down vs 1 -9.46284 89.5454 0.697649 1.65169 1cg11180069 0.033475 0.336877 6.03E-18 0.0993685 -10.0635 0 down vs 1 -9.46197 89.5288 1.669 3.95211 1cg01063579 0.06705 0.384696 6.05E-18 0.174294 -5.73745 0 down vs 1 -9.46145 89.5191 1.82938 4.33236 1cg11756029 TRAFD1 -0.15467 -0.401797 6.07E-18 0.384945 -2.59777 0 down vs 1 9.46097 89.51 1.10729 2.62255 1cg02998206 IL13RA2 0.15332 0.372587 6.07E-18 0.411502 -2.43012 0 down vs 1 -9.46096 89.5097 0.871692 2.06457 1cg20116929 TNXB 0.18111 0.360944 6.14E-18 0.501767 -1.99296 0 down vs 1 -9.45912 89.4749 0.586358 1.38931 1cg12989574 GPC6 -0.282705 0.0796938 6.20E-18 -3.54739 3.54739 0 up vs 1 -9.45783 89.4506 2.38118 5.64344 1cg01944302 0.1723 0.448416 6.22E-18 0.384241 -2.60253 0 down vs 1 -9.45721 89.4387 1.3823 3.27652 1cg22466716 DNAH5 0.14074 0.383256 6.29E-18 0.367222 -2.72315 0 down vs 1 -9.45559 89.4082 1.06634 2.52846 1cg00442924 SERPINB7 0.183205 0.400868 6.43E-18 0.457021 -2.18808 0 down vs 1 -9.4524 89.3479 0.858984 2.03815 1cg01883757 NUP93 0.21245 0.388657 6.46E-18 0.546626 -1.82941 0 down vs 1 -9.45171 89.3349 0.562944 1.33592 1cg14349531 EPHB6 0.020025 0.268607 6.47E-18 0.0745514 -13.4136 0 down vs 1 -9.45137 89.3284 1.12036 2.6589 1cg26653990 -0.12575 0.250387 6.53E-18 -0.502222 -1.99115 0 down vs 1 -9.45008 89.3039 2.56514 6.08942 1cg21396646 SLC35F3 0.200645 0.392934 6.54E-18 0.510633 -1.95835 0 down vs 1 -9.4497 89.2967 0.670385 1.59157 1cg13479091 PBX3 0.26443 0.438667 6.56E-18 0.602803 -1.65892 0 down vs 1 -9.44942 89.2915 0.550426 1.30685 1cg19591056 FMN2 -0.32215 0.103292 6.56E-18 -3.11884 3.11884 0 up vs 1 -9.44936 89.2904 3.28169 7.79165 1cg12074025 ZNF671 -0.253975 0.186731 6.61E-18 -1.36011 1.36011 0 up vs 1 -9.44822 89.2689 3.52141 8.36281 1cg21601919 FGF18 -0.303995 -0.46753 6.68E-18 0.650215 -1.53795 0 down vs 1 9.44665 89.2392 0.484885 1.15191 1cg15669183 FAM155A -0.336265 0.0355263 6.84E-18 -9.46524 9.46524 0 up vs 1 -9.44314 89.1728 2.5062 5.95826 1cg02066331 RBM24 -0.12418 0.236985 6.88E-18 -0.524 -1.9084 0 down vs 1 -9.44231 89.1571 2.36498 5.62352 1cg10071824 GSX1 -0.303885 0.121123 7.04E-18 -2.50889 2.50889 0 up vs 1 -9.4389 89.0928 3.27501 7.79302 1cg03522799 -0.32765 0.043484 7.12E-18 -7.53495 7.53495 0 up vs 1 -9.43721 89.061 2.49735 5.94467 1cg00232735 OPCML -0.37655 0.0210912 7.13E-18 -17.8534 17.8534 0 up vs 1 -9.43701 89.0571 2.86682 6.82446 1cg11043815 -0.35037 -0.0171165 7.22E-18 20.4697 20.4697 0 up vs 1 -9.43502 89.0195 2.01357 4.79532 1cg14252169 PHF6 0.3484 0.418946 7.23E-18 0.83161 -1.20249 0 down vs 1 -9.43481 89.0157 0.0902335 0.2149 1cg04365609 -0.027865 0.225549 7.36E-18 -0.123543 -8.09437 0 down vs 1 -9.43223 88.967 1.16434 2.77452 1cg11429658 ABCC13 0.08217 0.372124 7.43E-18 0.220813 -4.52871 0 down vs 1 -9.43087 88.9414 1.52432 3.63336 1cg01418539 0.299735 0.424879 7.51E-18 0.705459 -1.41752 0 down vs 1 -9.42925 88.9108 0.283949 0.677052 1cg03217572 EPHB6 0.218065 0.45537 7.52E-18 0.478875 -2.08823 0 down vs 1 -9.42899 88.9058 1.02101 2.43465 1cg24866784 FBLN2 0.01139 0.277863 7.54E-18 0.0409914 -24.3953 0 down vs 1 -9.42853 88.8972 1.28743 3.07024 1cg26956157 CBFA2T3 0.12045 0.442253 7.55E-18 0.272355 -3.67167 0 down vs 1 -9.42838 88.8943 1.87758 4.47775 1cg09286079 CYP2B6 0.062185 0.407041 7.55E-18 0.152773 -6.54564 0 down vs 1 -9.42835 88.8938 2.15622 5.1423 1cg01022121 PLCH2 0.239565 0.465037 7.82E-18 0.515152 -1.94117 0 down vs 1 -9.42308 88.7945 0.92173 2.20066 1cg18108840 -0.10812 0.19192 7.87E-18 -0.563361 -1.77506 0 down vs 1 -9.4222 88.7778 1.63221 3.89768 1cg20740711 -0.21592 -0.431284 7.92E-18 0.500645 -1.99743 0 down vs 1 9.4212 88.759 0.840939 2.00857 1cg09941017 PTPRN2 0.214935 0.455108 8.04E-18 0.472273 -2.11742 0 down vs 1 -9.41901 88.7177 1.04584 2.49914 1cg18484679 NR3C1 0.12746 0.357796 8.05E-18 0.356237 -2.80712 0 down vs 1 -9.4189 88.7157 0.961925 2.29867 1cg01942962 GRIA2 -0.25805 0.150437 8.10E-18 -1.71533 1.71533 0 up vs 1 -9.41798 88.6984 3.02534 7.23094 1cg19099050 LHFPL4 -0.291475 0.126211 8.27E-18 -2.30943 2.30943 0 up vs 1 -9.41489 88.6401 3.16313 7.56525 1cg24265719 DLGAP2 0.152345 0.409897 8.33E-18 0.371667 -2.69058 0 down vs 1 -9.4138 88.6196 1.20267 2.87709 1cg08202743 ABTB2 0.233055 0.372803 8.33E-18 0.625143 -1.59963 0 down vs 1 -9.41369 88.6175 0.354084 0.847077 1cg24669786 SAMD9 0.127545 0.405486 8.34E-18 0.314548 -3.17916 0 down vs 1 -9.41364 88.6166 1.40063 3.35077 1cg17680088 GPR144 -0.13567 0.12207 8.48E-18 -1.11142 1.11142 0 up vs 1 -9.41102 88.5674 1.20443 2.88299 1cg24160178 0.068255 0.341639 8.49E-18 0.199787 -5.00534 0 down vs 1 -9.41101 88.5672 1.35508 3.2436 1cg26390889 RXFP3 -0.261165 0.0849191 8.54E-18 -3.07546 3.07546 0 up vs 1 -9.41006 88.5493 2.17161 5.19915 1cg20598720 0.155565 0.423492 8.69E-18 0.367339 -2.72228 0 down vs 1 -9.40742 88.4995 1.30152 3.11778 1cg08372947 TMEM101 -0.099285 -0.445316 8.70E-18 0.222954 -4.48523 0 down vs 1 9.40729 88.4971 2.17095 5.20063 1cg02715685 ZFR2 -0.26632 0.0720789 8.74E-18 -3.69484 3.69484 0 up vs 1 -9.40662 88.4845 2.07623 4.97444 1cg00562916 PTPN4 0.266115 0.403113 8.77E-18 0.66015 -1.51481 0 down vs 1 -9.40609 88.4746 0.340287 0.815386 1cg14065590 PCDHB11 0.094395 0.359378 8.79E-18 0.262662 -3.80717 0 down vs 1 -9.40569 88.4669 1.27307 3.05076 1cg08092588 0.195715 0.442055 8.81E-18 0.442739 -2.25867 0 down vs 1 -9.4055 88.4634 1.10024 2.6367 1cg27014538 KLHL14 0.006245 0.309312 8.81E-18 0.02019 -49.5295 0 down vs 1 -9.40546 88.4628 1.66531 3.99089 1cg09456829 0.275095 0.449055 8.82E-18 0.612608 -1.63236 0 down vs 1 -9.4052 88.4578 0.548678 1.31497 1cg26591221 CBFA2T3 0.27102 0.454926 8.83E-18 0.595746 -1.67857 0 down vs 1 -9.4051 88.4558 0.613209 1.46966 1cg00388262 0.01148 0.357924 8.88E-18 0.0320738 -31.1781 0 down vs 1 -9.40418 88.4386 2.17613 5.21649 1cg08451469 LOC285830 0.089895 0.366507 9.01E-18 0.245275 -4.07706 0 down vs 1 -9.40203 88.3982 1.38727 3.327 1cg03950476 ZCCHC11 -0.109015 0.210265 9.16E-18 -0.518464 -1.92878 0 down vs 1 -9.39959 88.3523 1.84826 4.43487 1cg13122377 -0.306475 0.15078 9.39E-18 -2.03259 2.03259 0 up vs 1 -9.39591 88.2832 3.79084 9.10319 1cg14760176 0.001135 0.306985 9.46E-18 0.00369725 -270.471 0 down vs 1 -9.39477 88.2618 1.69604 4.07379 1cg17487741 RCBTB1 0.181395 0.379562 9.52E-18 0.477906 -2.09246 0 down vs 1 -9.39384 88.2442 0.712004 1.71054 1cg23906738 NKX2-1 -0.133545 0.22926 9.54E-18 -0.582505 -1.71672 0 down vs 1 -9.39362 88.2401 2.38651 5.73369 1cg22608082 0.05143 0.427259 1.00E-17 0.120372 -8.30759 0 down vs 1 -9.38633 88.1031 2.56094 6.16231 1cg04663932 LIMK1 -0.106065 -0.391301 1.01E-17 0.271057 -3.68926 0 down vs 1 9.38526 88.0831 1.47512 3.55034 1cg05258757 ROPN1B -0.351045 0.0451021 1.01E-17 -7.78335 7.78335 0 up vs 1 -9.38519 88.0818 2.84532 6.84826 1cg23831897 10-Mar 0.286755 0.463711 1.02E-17 0.618392 -1.6171 0 down vs 1 -9.383 88.0407 0.567738 1.3671 1cg17291500 PDGFA 0.277025 0.468307 1.04E-17 0.591545 -1.69049 0 down vs 1 -9.38037 87.9913 0.663387 1.59832 1cg19734870 LOC645323 -0.37142 0.0130294 1.05E-17 -28.5063 28.5063 0 up vs 1 -9.37994 87.9832 2.67976 6.45702 1cg00508604 PF4V1 -0.044745 0.287341 1.05E-17 -0.155721 -6.42175 0 down vs 1 -9.3788 87.962 1.99949 4.81904 1cg04857881 ITFG1 0.104375 0.328015 1.06E-17 0.318202 -3.14266 0 down vs 1 -9.3783 87.9525 0.906811 2.18577 1cg10655021 -0.35628 0.112225 1.06E-17 -3.17469 3.17469 0 up vs 1 -9.37724 87.9326 3.97967 9.59473 1cg07307994 -0.070125 0.270151 1.08E-17 -0.259577 -3.85242 0 down vs 1 -9.37527 87.8957 2.09933 5.06348 1cg05422457 0.07512 0.370226 1.08E-17 0.202903 -4.92846 0 down vs 1 -9.37487 87.8883 1.57897 3.80873 1cg01177168 0.18257 0.372505 1.10E-17 0.490114 -2.04034 0 down vs 1 -9.3719 87.8326 0.654077 1.57873 1cg11672277 MAPRE2 0.032785 0.304572 1.12E-17 0.107643 -9.28999 0 down vs 1 -9.36936 87.785 1.33929 3.23438 1cg03670369 0.20062 0.46588 1.12E-17 0.430626 -2.3222 0 down vs 1 -9.36903 87.7787 1.27574 3.08111 1cg10381036 XAF1 0.306135 0.432631 1.15E-17 0.707612 -1.4132 0 down vs 1 -9.3653 87.7088 0.290116 0.701235 1cg24556395 MYO10 0.048135 0.35812 1.16E-17 0.13441 -7.43991 0 down vs 1 -9.36433 87.6906 1.74221 4.21194 1cg23514374 ETS1 -0.03237 0.282679 1.18E-17 -0.114512 -8.73274 0 down vs 1 -9.36241 87.6548 1.79959 4.35245 1cg26308704 PTPRN2 0.19841 0.437612 1.18E-17 0.453392 -2.2056 0 down vs 1 -9.36185 87.6442 1.03741 2.50935 1cg26007189 -0.280985 0.231942 1.19E-17 -1.21145 1.21145 0 up vs 1 -9.36116 87.6313 4.77011 11.54 1cg18670236 CHCHD6 -0.04261 -0.383854 1.19E-17 0.111006 -9.00853 0 down vs 1 9.36073 87.6233 2.11129 5.10814 1cg03853586 0.1506 0.40374 1.20E-17 0.373013 -2.68087 0 down vs 1 -9.35992 87.6082 1.16182 2.81145 1cg24995083 CLMN 0.14255 0.382974 1.20E-17 0.372219 -2.68659 0 down vs 1 -9.35923 87.5953 1.04803 2.53646 1cg19871722 0.168505 0.404643 1.20E-17 0.416428 -2.40137 0 down vs 1 -9.35913 87.5933 1.011 2.4469 1cg15777781 CUGBP2 0.041465 0.335947 1.23E-17 0.123427 -8.10194 0 down vs 1 -9.35585 87.532 1.5723 3.80806 1cg02757172 GRIA2 -0.144385 0.23067 1.24E-17 -0.625939 -1.5976 0 down vs 1 -9.35428 87.5026 2.55039 6.17905 1cg17237813 TTLL7 0.271405 0.419848 1.24E-17 0.646436 -1.54694 0 down vs 1 -9.354 87.4973 0.399521 0.968013 1cg19246076 ETS1 0.259055 0.418644 1.25E-17 0.618795 -1.61604 0 down vs 1 -9.35312 87.4808 0.46177 1.11905 1cg27622017 SOX2OT -0.24095 0.111507 1.25E-17 -2.16085 2.16085 0 up vs 1 -9.35294 87.4774 2.25232 5.45847 1cg04278702 HTR1E -0.342315 -0.011568 1.26E-17 29.5914 29.5914 0 up vs 1 -9.3525 87.4692 1.9834 4.80718 1cg23218431 PHACTR3 0.068505 0.350402 1.27E-17 0.195504 -5.11499 0 down vs 1 -9.35121 87.4451 1.44078 3.49301 1cg10598034 ZNF701 -0.35149 0.108692 1.27E-17 -3.23382 3.23382 0 up vs 1 -9.3512 87.4449 3.83952 9.30846 1cg26341848 0.0864 0.379524 1.28E-17 0.227654 -4.39264 0 down vs 1 -9.35011 87.4245 1.55783 3.77766 1cg07599881 -0.293275 0.0853366 1.28E-17 -3.43668 3.43668 0 up vs 1 -9.34987 87.4201 2.599 6.30275 1cg06819251 TNXB -0.016485 0.375066 1.29E-17 -0.0439523 -22.752 0 down vs 1 -9.34866 87.3975 2.77968 6.74266 1cg14534279 -0.112045 0.282947 1.29E-17 -0.395992 -2.5253 0 down vs 1 -9.34825 87.3898 2.82876 6.86231 1cg02512724 PTPRN2 0.129395 0.331562 1.29E-17 0.390259 -2.5624 0 down vs 1 -9.34806 87.3862 0.741034 1.79776 1cg08521065 FEZ1 0.19697 0.420485 1.30E-17 0.468435 -2.13477 0 down vs 1 -9.34742 87.3742 0.905798 2.19778 1cg17251384 DTHD1 0.241765 0.423268 1.32E-17 0.571187 -1.75074 0 down vs 1 -9.34455 87.3207 0.597291 1.45012 1cg10142520 EHBP1L1 0.03252 -0.339911 1.33E-17 -0.0956722 -10.4524 0 down vs 1 9.34385 87.3075 2.51483 6.10652 1cg12238343 RXFP3 -0.24276 0.0988273 1.34E-17 -2.45641 2.45641 0 up vs 1 -9.34247 87.2817 2.11554 5.13847 1cg19480937 RFTN1 0.221575 0.386349 1.36E-17 0.573509 -1.74365 0 down vs 1 -9.34082 87.2509 0.492264 1.19609 1cg11863130 ARX 0.15139 0.334997 1.37E-17 0.451914 -2.21281 0 down vs 1 -9.34003 87.2361 0.611221 1.48538 1cg14774511 TSPAN9 0.325855 0.485408 1.37E-17 0.671302 -1.48964 0 down vs 1 -9.33994 87.2344 0.461558 1.12169 1cg25033364 KCNC1 -0.132095 0.155012 1.39E-17 -0.852161 -1.17349 0 down vs 1 -9.33735 87.1861 1.49453 3.63407 1cg26861487 PTPRN2 0.145755 0.379175 1.42E-17 0.384401 -2.60145 0 down vs 1 -9.33364 87.1169 0.987855 2.40396 1cg17907457 HS2ST1 0.22367 0.374848 1.45E-17 0.596694 -1.6759 0 down vs 1 -9.3312 87.0713 0.414379 1.00892 1cg25611723 APCDD1 0.01336 0.286751 1.45E-17 0.0465909 -21.4634 0 down vs 1 -9.33087 87.0652 1.35515 3.29972 1cg01864836 -0.12349 0.152493 1.46E-17 -0.809806 -1.23486 0 down vs 1 -9.33043 87.0569 1.38097 3.36292 1cg22471726 -0.09901 0.288954 1.47E-17 -0.34265 -2.91843 0 down vs 1 -9.32886 87.0276 2.72898 6.64783 1cg25894019 0.23332 0.462666 1.49E-17 0.504295 -1.98297 0 down vs 1 -9.32685 86.9902 0.953675 2.32416 1cg26150514 -0.020095 0.267885 1.51E-17 -0.0750134 -13.331 0 down vs 1 -9.32894 87.0291 1.50291 3.64378 1cg04479860 0.22586 0.388957 1.51E-17 0.580682 -1.72211 0 down vs 1 -9.32455 86.9473 0.48229 1.17595 1cg02268156 -0.038035 0.246163 1.54E-17 -0.154512 -6.47201 0 down vs 1 -9.32175 86.8951 1.4644 3.57273 1cg02983424 -0.2102 0.225108 1.56E-17 -0.933775 -1.07092 0 down vs 1 -9.31975 86.8578 3.43566 8.38567 1cg26675654 -0.1969 0.152337 1.57E-17 -1.29253 1.29253 0 up vs 1 -9.31933 86.85 2.21136 5.3979 1cg18406492 STAR -0.17851 0.156632 1.58E-17 -1.13968 1.13968 0 up vs 1 -9.31863 86.8369 2.03646 4.97172 1cg14429345 ADARB2 0.04962 0.353271 1.58E-17 0.140459 -7.11952 0 down vs 1 -9.31786 86.8225 1.67173 4.08197 1cg18599750 BCL11B 0.093415 0.388325 1.59E-17 0.240559 -4.15699 0 down vs 1 -9.31745 86.8149 1.57688 3.8507 1cg05499367 TMEM26 0.03639 0.283172 1.59E-17 0.128508 -7.78159 0 down vs 1 -9.31682 86.8031 1.10419 2.69678 1cg22931725 FMN2 -0.36209 -0.0602495 1.64E-17 6.00984 6.00984 0 up vs 1 -9.31277 86.7276 1.65186 4.03786 1cg23553867 0.220825 0.454325 1.65E-17 0.486051 -2.0574 0 down vs 1 -9.31139 86.7019 0.988532 2.41712 1cg04918314 KRT75 0.19178 0.394196 1.66E-17 0.486509 -2.05546 0 down vs 1 -9.31044 86.6843 0.742864 1.81679 1cg25869105 WDR37 0.13815 0.332373 1.67E-17 0.415648 -2.40588 0 down vs 1 -9.30994 86.6751 0.68394 1.67286 1cg21199659 ASS1 0.11905 0.225103 1.68E-17 0.52887 -1.89082 0 down vs 1 -9.30923 86.6618 0.20392 0.498848 1cg12691488 -0.27312 0.0834907 1.68E-17 -3.27126 3.27126 0 up vs 1 -9.30881 86.654 2.30572 5.64097 1cg14006096 0.03624 0.347687 1.68E-17 0.104232 -9.594 0 down vs 1 -9.30872 86.6523 1.75867 4.3027 1cg26024874 WDR7 0.15716 0.403556 1.69E-17 0.389438 -2.5678 0 down vs 1 -9.30825 86.6435 1.10074 2.6933 1cg18971999 LOC285830 0.117195 0.382482 1.69E-17 0.306406 -3.26364 0 down vs 1 -9.30823 86.6431 1.276 3.12215 1cg14040899 ADARB2 0.128175 0.390806 1.71E-17 0.327976 -3.049 0 down vs 1 -9.30598 86.6013 1.25057 3.0614 1cg00774005 DLX6AS -0.067015 0.295217 1.73E-17 -0.227003 -4.40524 0 down vs 1 -9.30504 86.5838 2.37898 5.82493 1cg16347279 RAB37 -0.259115 0.0830211 1.73E-17 -3.12107 3.12107 0 up vs 1 -9.30443 86.5724 2.12234 5.19723 1cg01220680 ANGPT4 -0.26415 0.00989897 1.74E-17 -26.6846 26.6846 0 up vs 1 -9.30407 86.5658 1.36168 3.33476 1cg08976646 AP3B1 0.315275 0.426348 1.74E-17 0.739477 -1.35231 0 down vs 1 -9.30372 86.5593 0.223686 0.547849 1cg12582008 OLFM4 -0.038795 0.326884 1.75E-17 -0.118681 -8.42592 0 down vs 1 -9.30303 86.5464 2.42447 5.93886 1cg03944072 CMIP 0.207235 -0.0785258 1.75E-17 -2.63907 2.63907 0 up vs 1 9.30267 86.5396 1.48055 3.62697 1cg14574910 0.129995 0.443935 1.75E-17 0.292824 -3.41502 0 down vs 1 -9.30257 86.5377 1.78695 4.37766 1cg02995770 TMPRSS11F 0.28712 0.419042 1.77E-17 0.685181 -1.45947 0 down vs 1 -9.30159 86.5197 0.31554 0.77317 1cg25592910 PCDH15 -0.160855 0.217632 1.77E-17 -0.739113 -1.35297 0 down vs 1 -9.30091 86.5069 2.59729 6.36511 1cg05241461 ZNF492 -0.294005 0.097666 1.78E-17 -3.01031 3.01031 0 up vs 1 -9.30053 86.4999 2.78138 6.81681 1cg24407133 0.20497 0.38139 1.79E-17 0.537429 -1.86071 0 down vs 1 -9.2999 86.4881 0.564306 1.38323 1cg11325942 0.151535 0.423792 1.79E-17 0.357569 -2.79666 0 down vs 1 -9.29971 86.4846 1.34393 3.29437 1cg15259233 FAM120B 0.13485 0.342431 1.82E-17 0.393802 -2.53935 0 down vs 1 -9.29666 86.4279 0.781255 1.91635 1cg25830307 C10orf11 0.14335 0.425503 1.85E-17 0.336896 -2.96828 0 down vs 1 -9.29443 86.3864 1.4434 3.54223 1cg01020263 NOD2 -0.296355 -0.436423 1.87E-17 0.679054 -1.47264 0 down vs 1 9.29319 86.3634 0.355711 0.873178 1cg22115892 0.092375 0.414823 1.87E-17 0.222686 -4.49064 0 down vs 1 -9.29259 86.3522 1.88511 4.62806 1cg10947826 ADAMTS2 0.280695 0.465389 1.90E-17 0.603141 -1.65799 0 down vs 1 -9.29087 86.3203 0.618475 1.51895 1cg26645225 0.169035 0.437965 1.90E-17 0.385956 -2.59097 0 down vs 1 -9.2905 86.3134 1.31128 3.22073 1cg19942083 -0.24301 -0.43694 1.92E-17 0.556164 -1.79803 0 down vs 1 9.28917 86.2886 0.681878 1.67529 1cg23409074 CRH 0.05788 0.319773 1.93E-17 0.181004 -5.52475 0 down vs 1 -9.28792 86.2655 1.24355 3.05607 1cg08162976 -0.029065 0.295162 1.94E-17 -0.0984714 -10.1552 0 down vs 1 -9.28728 86.2536 1.90597 4.68462 1cg26731008 FAM24A -0.107285 0.196612 1.98E-17 -0.545669 -1.83261 0 down vs 1 -9.28875 86.281 1.67363 4.09287 1cg12410921 -0.11512 0.0996098 2.01E-17 -1.15571 1.15571 0 up vs 1 -9.28242 86.1634 0.835993 2.05691 1cg18058689 GPC6 -0.251015 0.149657 2.05E-17 -1.67727 1.67727 0 up vs 1 -9.27906 86.101 2.91069 7.16676 1cg27573308 0.06482 0.402846 2.06E-17 0.160905 -6.21485 0 down vs 1 -9.27837 86.0881 2.07166 5.10166 1cg04729173 0.00967 0.259318 2.07E-17 0.0372901 -26.8168 0 down vs 1 -9.2777 86.0758 1.12999 2.7831 1cg08204702 PDE4D -0.29515 0.0523727 2.08E-17 -5.63557 5.63557 0 up vs 1 -9.27709 86.0644 2.1897 5.39382 1cg01277511 SSTR4 0.07042 0.280194 2.10E-17 0.251326 -3.9789 0 down vs 1 -9.27533 86.0317 0.797849 1.96606 1cg18947801 LPPR4 -0.35497 0.061583 2.13E-17 -5.76409 5.76409 0 up vs 1 -9.27348 85.9974 3.146 7.75548 1cg02495620 0.109335 0.345426 2.13E-17 0.316522 -3.15934 0 down vs 1 -9.27327 85.9936 1.0106 2.49142 1cg15055165 0.013545 0.295825 2.14E-17 0.0457872 -21.8401 0 down vs 1 -9.27268 85.9826 1.4447 3.56207 1cg00592871 0.159755 0.384344 2.14E-17 0.415656 -2.40584 0 down vs 1 -9.27245 85.9784 0.914526 2.25498 1cg14528751 0.095295 0.375482 2.17E-17 0.253794 -3.94021 0 down vs 1 -9.27455 86.0172 1.42267 3.48981 1cg16624482 UMODL1 -0.10781 0.317326 2.19E-17 -0.339745 -2.94338 0 down vs 1 -9.26947 85.923 3.27698 8.08538 1cg11971771 -0.13317 0.237109 2.19E-17 -0.56164 -1.7805 0 down vs 1 -9.2694 85.9217 2.48586 6.13352 1cg13706582 ZNF385D -0.03183 0.27607 2.19E-17 -0.115297 -8.67325 0 down vs 1 -9.26912 85.9165 1.71884 4.24126 1cg19839798 FAM155A -0.30915 0.183777 2.22E-17 -1.6822 1.6822 0 up vs 1 -9.26701 85.8774 4.40538 10.8753 1cg10658779 C10orf11 0.12493 0.361456 2.23E-17 0.34563 -2.89327 0 down vs 1 -9.2667 85.8718 1.01432 2.50415 1cg15601264 -0.17102 -0.401023 2.24E-17 0.426459 -2.34489 0 down vs 1 9.26569 85.8529 0.959148 2.36846 1cg02538248 ZNRF1 -0.161105 -0.38013 2.25E-17 0.423815 -2.35952 0 down vs 1 9.26522 85.8442 0.869775 2.14799 1cg05101437 CDK6 -0.344945 -0.44939 2.26E-17 0.767585 -1.30279 0 down vs 1 9.26447 85.8305 0.197784 0.488523 1cg17673237 FAM155A -0.329105 0.0600191 2.29E-17 -5.48334 5.48334 0 up vs 1 -9.26277 85.7989 2.74533 6.78342 1cg02897359 HSPA1A -0.293055 -0.468394 2.31E-17 0.625659 -1.59832 0 down vs 1 9.26123 85.7704 0.557412 1.37777 1cg14119999 FAM155A -0.283145 0.0953041 2.32E-17 -2.97096 2.97096 0 up vs 1 -9.26085 85.7634 2.59677 6.41899 1cg22789318 ABR 0.29602 0.466651 2.32E-17 0.634351 -1.57642 0 down vs 1 -9.26054 85.7577 0.527875 1.30495 1cg22721472 TSHZ3 0.17184 0.392682 2.34E-17 0.437605 -2.28516 0 down vs 1 -9.25961 85.7404 0.884266 2.18642 1cg09907521 0.081925 0.278737 2.34E-17 0.293915 -3.40234 0 down vs 1 -9.2596 85.7402 0.702298 1.73649 1cg02805028 GSG1L 0.03641 0.285251 2.35E-17 0.127642 -7.83441 0 down vs 1 -9.25884 85.7261 1.1227 2.77642 1cg09900986 0.142745 0.433991 2.38E-17 0.328912 -3.04033 0 down vs 1 -9.25657 85.6841 1.53794 3.80517 1cg13455509 0.225585 0.435031 2.39E-17 0.518549 -1.92846 0 down vs 1 -9.25634 85.6799 0.79536 1.96798 1cg00059225 GLRA1 -0.26121 0.0748629 2.41E-17 -3.48918 3.48918 0 up vs 1 -9.25494 85.6539 2.04779 5.06843 1cg04539515 0.000705 0.307305 2.48E-17 0.00229414 -435.893 0 down vs 1 -9.25072 85.5758 1.70436 4.22227 1cg11842425 BCL8 0.154125 0.387321 2.48E-17 0.397926 -2.51303 0 down vs 1 -9.25069 85.5752 0.985963 2.44258 1cg01801610 0.137065 0.409985 2.48E-17 0.334317 -2.99117 0 down vs 1 -9.25062 85.574 1.35048 3.34567 1cg26636398 0.149125 0.374446 2.49E-17 0.398255 -2.51095 0 down vs 1 -9.25003 85.563 0.920494 2.28071 1cg00233420 -0.102215 0.215725 2.51E-17 -0.473821 -2.1105 0 down vs 1 -9.24898 85.5436 1.83277 4.54209 1cg15400220 LOC285830 0.19579 0.351422 2.53E-17 0.557137 -1.79489 0 down vs 1 -9.2479 85.5237 0.439151 1.08859 1cg26100214 0.082035 0.29461 2.53E-17 0.278453 -3.59128 0 down vs 1 -9.24749 85.5161 0.819301 2.0311 1cg07211516 HIP1 0.216025 0.423826 2.53E-17 0.509702 -1.96193 0 down vs 1 -9.24748 85.516 0.782911 1.94089 1cg03646889 LPPR4 -0.377035 0.0531778 2.53E-17 -7.09008 7.09008 0 up vs 1 -9.24741 85.5146 3.35571 8.31918 1cg04199473 STRN3 -0.34803 -0.43854 2.58E-17 0.793611 -1.26006 0 down vs 1 9.24452 85.4612 0.148528 0.368447 1cg15111386 TNXB 0.13604 0.367956 2.65E-17 0.369718 -2.70476 0 down vs 1 -9.24077 85.3919 0.975169 2.42103 1cg08839164 0.336 0.471647 2.66E-17 0.712397 -1.40371 0 down vs 1 -9.24009 85.3792 0.333612 0.828371 1cg15091407 -0.39881 -0.00133351 2.68E-17 299.069 299.069 0 up vs 1 -9.23876 85.3547 2.86445 7.11458 1cg16082695 LOC100128239 0.059875 0.386381 2.69E-17 0.154964 -6.45312 0 down vs 1 -9.24663 85.5001 1.93098 4.74274 1cg23227462 -0.09651 0.201294 2.70E-17 -0.479448 -2.08573 0 down vs 1 -9.23805 85.3415 1.60797 3.99442 1cg27209278 ZNRF4 0.34253 0.477463 2.70E-17 0.717396 -1.39393 0 down vs 1 -9.23762 85.3337 0.330106 0.820104 1cg07223952 0.175475 0.446935 2.74E-17 0.392619 -2.547 0 down vs 1 -9.23547 85.294 1.33607 3.32082 1cg14103215 -0.03846 0.303335 2.76E-17 -0.12679 -7.88703 0 down vs 1 -9.23462 85.2782 2.11811 5.26559 1cg17162475 C6orf150 -0.307585 -0.400788 2.79E-17 0.76745 -1.30302 0 down vs 1 9.23292 85.2468 0.157499 0.391685 1cg07974367 -0.056195 0.254274 2.82E-17 -0.221002 -4.52486 0 down vs 1 -9.23116 85.2144 1.74765 4.34789 1cg00989002 -0.09955 0.230144 2.83E-17 -0.432555 -2.31185 0 down vs 1 -9.23082 85.2081 1.97079 4.90339 1cg16383910 TBC1D2B 0.182645 -0.0937784 2.84E-17 -1.94762 1.94762 0 up vs 1 9.23019 85.1963 1.38538 3.44733 1cg25554496 GNA13 0.2597 -0.141116 2.86E-17 -1.84033 1.84033 0 up vs 1 9.22945 85.1827 2.91278 7.24924 1cg20784531 0.14506 0.351493 2.90E-17 0.412697 -2.42309 0 down vs 1 -9.22713 85.14 0.772637 1.92388 1cg20468586 KRT6B 0.22459 0.346125 2.93E-17 0.64887 -1.54114 0 down vs 1 -9.22585 85.1164 0.267805 0.667024 1cg12271981 SLITRK5 -0.201565 0.182044 2.94E-17 -1.10723 1.10723 0 up vs 1 -9.22526 85.1054 2.66806 6.64623 1cg07987311 MCTP2 0.244705 0.404754 2.94E-17 0.604578 -1.65405 0 down vs 1 -9.22499 85.1005 0.464432 1.15698 1cg18315695 HSPA1A -0.295175 -0.448623 2.94E-17 0.657958 -1.51985 0 down vs 1 9.22492 85.0992 0.426912 1.06353 1cg25797455 TSC22D4 0.20754 0.393921 2.96E-17 0.526857 -1.89805 0 down vs 1 -9.22432 85.088 0.629828 1.56924 1cg01134643 UCN3 -0.108585 0.281873 2.96E-17 -0.385227 -2.59587 0 down vs 1 -9.22423 85.0864 2.76418 6.88718 1cg10708793 DHX58 -0.29579 -0.41557 2.97E-17 0.71177 -1.40495 0 down vs 1 9.22346 85.0722 0.260126 0.648234 1cg06349762 0.14465 0.45209 3.01E-17 0.319958 -3.12541 0 down vs 1 -9.22146 85.0353 1.71372 4.27244 1cg26290632 CALB1 -0.319685 0.0575562 3.03E-17 -5.55431 5.55431 0 up vs 1 -9.22078 85.0227 2.58022 6.43364 1cg14191476 P2RY12 -0.033485 0.290408 3.04E-17 -0.115303 -8.67279 0 down vs 1 -9.22023 85.0126 1.90205 4.74323 1cg24304919 KCNN2 -0.19751 0.123786 3.05E-17 -1.59558 1.59558 0 up vs 1 -9.21959 85.0008 1.87167 4.66812 1cg10795614 0.001015 0.296474 3.05E-17 0.00342357 -292.093 0 down vs 1 -9.21949 84.999 1.58275 3.94762 1cg20338386 -0.154965 0.179955 3.05E-17 -0.861131 -1.16126 0 down vs 1 -9.21944 84.998 2.03376 5.07256 1cg12228319 BRUNOL4 0.14881 0.364197 3.06E-17 0.408597 -2.4474 0 down vs 1 -9.21905 84.991 0.841121 2.09808 1cg18000216 ERC2 0.19648 0.410634 3.08E-17 0.47848 -2.08995 0 down vs 1 -9.21825 84.9762 0.831512 2.07447 1cg11654517 ARHGEF6 -0.111455 0.199198 3.09E-17 -0.559517 -1.78725 0 down vs 1 -9.21746 84.9617 1.74973 4.36599 1cg11759875 0.184275 0.442732 3.10E-17 0.416222 -2.40256 0 down vs 1 -9.21698 84.9527 1.21115 3.02242 1cg18458509 SLC22A18AS -0.042905 -0.321937 3.14E-17 0.133271 -7.50349 0 down vs 1 9.21524 84.9206 1.41165 3.52411 1cg06796435 LTB4R2 -0.019615 -0.283243 3.21E-17 0.0692514 -14.4401 0 down vs 1 9.21204 84.8616 1.26009 3.14794 1cg12969193 HOXD9 0.00651 0.306637 3.21E-17 0.0212303 -47.1025 0 down vs 1 -9.21174 84.8562 1.63316 4.08019 1cg03412431 GRM4 0.25198 0.355833 3.23E-17 0.708141 -1.41215 0 down vs 1 -9.21116 84.8454 0.195549 0.488611 1cg27648238 -0.068095 0.197955 3.27E-17 -0.343992 -2.90704 0 down vs 1 -9.20904 84.8064 1.28335 3.20813 1cg07806394 0.042805 0.298823 3.37E-17 0.143245 -6.98102 0 down vs 1 -9.20457 84.724 1.18839 2.97363 1cg25354926 CPLX1 0.23076 0.459637 3.44E-17 0.502049 -1.99184 0 down vs 1 -9.20134 84.6647 0.949775 2.37823 1cg25478614 SST -0.22657 0.0608845 3.45E-17 -3.72131 3.72131 0 up vs 1 -9.20118 84.6618 1.49815 3.7515 1cg11468819 -0.193865 0.149076 3.50E-17 -1.30044 1.30044 0 up vs 1 -9.19879 84.6177 2.13235 5.34235 1cg25285090 -0.19168 0.248154 3.51E-17 -0.772425 -1.29462 0 down vs 1 -9.1984 84.6106 3.50748 8.78832 1cg19084822 GFOD1 0.27882 0.390086 3.52E-17 0.714766 -1.39906 0 down vs 1 -9.19822 84.6072 0.22446 0.56243 1cg20338426 ME3 0.120125 0.370314 3.53E-17 0.324387 -3.08274 0 down vs 1 -9.19754 84.5947 1.13489 2.84411 1cg26673722 TMPRSS11A -0.00919 0.290725 3.55E-17 -0.0316107 -31.6349 0 down vs 1 -9.19682 84.5815 1.63085 4.08765 1cg01165402 -0.197315 0.220406 3.60E-17 -0.895236 -1.11702 0 down vs 1 -9.19453 84.5393 3.16366 7.93354 1cg07486474 PDE6B -0.108735 0.358725 3.60E-17 -0.303115 -3.29908 0 down vs 1 -9.19449 84.5386 3.96193 9.93547 1cg00301239 -0.27807 -0.420611 3.60E-17 0.661109 -1.51261 0 down vs 1 9.19446 84.5382 0.368383 0.92381 1cg09499856 PLCXD3 -0.284 -0.00619536 3.63E-17 45.8408 45.8408 0 up vs 1 -9.19343 84.5191 1.39926 3.50977 1cg23458898 JAKMIP3 0.242015 0.456285 3.63E-17 0.530403 -1.88536 0 down vs 1 -9.19338 84.5182 0.832417 2.08798 1cg27058257 VSTM2B -0.345065 0.0868639 3.64E-17 -3.97248 3.97248 0 up vs 1 -9.19309 84.513 3.38254 8.48506 1cg17420058 -0.06821 0.343725 3.65E-17 -0.198444 -5.03921 0 down vs 1 -9.19253 84.5027 3.07663 7.71863 1cg10321146 VAV1 -0.042465 0.302113 3.67E-17 -0.14056 -7.1144 0 down vs 1 -9.1919 84.4911 2.15275 5.40154 1cg24494114 -0.07423 0.225837 3.70E-17 -0.328689 -3.04239 0 down vs 1 -9.19059 84.467 1.6325 4.09734 1cg15754594 -0.02168 0.371485 3.71E-17 -0.0583604 -17.1349 0 down vs 1 -9.1902 84.4597 2.80264 7.03484 1cg02746340 0.14037 0.392862 3.71E-17 0.357301 -2.79876 0 down vs 1 -9.19009 84.4578 1.15588 2.90142 1cg27185978 DOCK5 0.021065 0.299654 3.76E-17 0.0702978 -14.2252 0 down vs 1 -9.18826 84.4241 1.40716 3.53357 1cg09115646 0.02099 0.403318 3.76E-17 0.0520433 -19.2148 0 down vs 1 -9.18815 84.4222 2.65027 6.65533 1cg20369299 0.18144 0.384574 3.78E-17 0.471794 -2.11957 0 down vs 1 -9.18743 84.4089 0.748142 1.87902 1cg03664527 TP73 0.101425 0.286914 3.82E-17 0.353503 -2.82883 0 down vs 1 -9.1859 84.3808 0.623812 1.56728 1cg16419526 0.220855 0.378184 3.84E-17 0.583988 -1.71236 0 down vs 1 -9.18489 84.3622 0.448782 1.12778 1cg01489256 TDH 0.03303 0.282874 3.91E-17 0.116766 -8.56415 0 down vs 1 -9.18228 84.3143 1.13176 2.8457 1cg11717564 RXRG -0.241585 0.177121 3.93E-17 -1.36396 1.36396 0 up vs 1 -9.1813 84.2963 3.1786 7.99398 1cg02082650 ARX -0.02261 0.280019 4.01E-17 -0.0807446 -12.3847 0 down vs 1 -9.17829 84.2411 1.6605 4.17878 1cg02212846 FNDC1 -0.023695 0.25723 4.04E-17 -0.0921159 -10.8559 0 down vs 1 -9.17724 84.2217 1.43087 3.60174 1cg03331978 -0.1852 0.085718 4.09E-17 -2.16057 2.16057 0 up vs 1 -9.17549 84.1895 1.33074 3.35098 1cg21885159 GNG7 0.23051 0.425432 4.09E-17 0.541826 -1.84561 0 down vs 1 -9.17535 84.1871 0.688874 1.73472 1cg14380774 BEGAIN 0.14173 0.35742 4.11E-17 0.396537 -2.52183 0 down vs 1 -9.17472 84.1754 0.843483 2.12435 1cg03496746 CHRNA4 0.18202 0.400962 4.11E-17 0.453958 -2.20285 0 down vs 1 -9.17464 84.1739 0.869116 2.18895 1cg24985300 KDM5C 0.191815 0.397191 4.20E-17 0.482929 -2.0707 0 down vs 1 -9.1716 84.1182 0.764748 1.92737 1cg23306755 0.087635 0.304645 4.20E-17 0.287663 -3.47629 0 down vs 1 -9.17157 84.1177 0.853841 2.15192 1cg06906472 ST6GAL2 -0.35922 0.0368753 4.21E-17 -9.74149 9.74149 0 up vs 1 -9.17116 84.1103 2.84457 7.16975 1cg12639279 ABLIM2 0.20693 0.366189 4.21E-17 0.565091 -1.76963 0 down vs 1 -9.17094 84.1061 0.459858 1.15913 1cg26784682 KLHL5 0.146935 0.378007 4.25E-17 0.38871 -2.57261 0 down vs 1 -9.16972 84.0837 0.968081 2.44082 1cg13093774 PHYHIP -0.17478 0.183996 4.25E-17 -0.949913 -1.05273 0 down vs 1 -9.1697 84.0833 2.3338 5.88424 1cg05967120 TSC22D4 0.17451 0.389833 4.30E-17 0.447653 -2.23387 0 down vs 1 -9.1679 84.0504 0.840618 2.12029 1cg01094108 GLI3 0.252275 0.404464 4.32E-17 0.623726 -1.60327 0 down vs 1 -9.16728 84.039 0.41994 1.05936 1cg24135553 C11orf41 0.13124 0.3396 4.32E-17 0.386455 -2.58763 0 down vs 1 -9.16717 84.037 0.78713 1.98569 1cg14804593 0.06078 0.300294 4.39E-17 0.202402 -4.94067 0 down vs 1 -9.16469 83.9915 1.04011 2.6253 1cg19824907 GPC6 -0.303235 0.116961 4.43E-17 -2.59261 2.59261 0 up vs 1 -9.16355 83.9707 3.20127 8.08222 1cg05920090 HSPA1A -0.334335 -0.44877 4.43E-17 0.745004 -1.34228 0 down vs 1 9.16351 83.97 0.237428 0.599438 1cg21483929 0.17753 0.401952 4.43E-17 0.44167 -2.26414 0 down vs 1 -9.16351 83.9699 0.913165 2.30548 1cg02613108 PCDH15 -0.25703 0.15871 4.47E-17 -1.61949 1.61949 0 up vs 1 -9.16222 83.9464 3.13373 7.914 1cg05121480 SST -0.33475 0.0737655 4.48E-17 -4.53803 4.53803 0 up vs 1 -9.16175 83.9376 3.02576 7.64212 1cg27106959 SRRM4 -0.113815 0.247314 4.49E-17 -0.460204 -2.17295 0 down vs 1 -9.16134 83.9302 2.36452 5.97257 1cg09017117 HLA-DMB 0.00024 0.312344 4.49E-17 0.000768384 -1301.43 0 down vs 1 -9.16133 83.9299 1.7661 4.46103 1cg23692680 0.09355 0.370025 4.50E-17 0.252821 -3.95537 0 down vs 1 -9.16099 83.9237 1.38589 3.5009 1cg15408073 RXFP3 -0.299935 0.0149299 4.51E-17 -20.0896 20.0896 0 up vs 1 -9.16074 83.9191 1.79749 4.54089 1cg13393830 ZNF577 -0.093775 0.262521 4.52E-17 -0.35721 -2.79947 0 down vs 1 -9.16043 83.9134 2.30165 5.81491 1cg07506599 PTPRN2 0.168585 0.448697 4.54E-17 0.375721 -2.66155 0 down vs 1 -9.15988 83.9035 1.4226 3.5945 1cg01134282 RALYL -0.274 0.140162 4.66E-17 -1.95488 1.95488 0 up vs 1 -9.15568 83.8265 3.10998 7.86525 1cg18786623 HCG4P6 0.1507 -0.252109 4.68E-17 -0.597758 -1.67292 0 down vs 1 9.17887 84.2517 2.93307 7.17151 1cg12359739 0.159935 0.432855 4.71E-17 0.369489 -2.70644 0 down vs 1 -9.15412 83.7979 1.35048 3.41657 1cg16245261 PRKCDBP -0.2136 -0.421289 4.72E-17 0.507015 -1.97233 0 down vs 1 9.15376 83.7913 0.78207 1.97871 1cg27391267 ZSCAN1 -0.190095 0.0575567 4.72E-17 -3.30274 3.30274 0 up vs 1 -9.15375 83.7911 1.11199 2.81345 1cg04373435 CTNNA2 -0.333215 0.108571 4.74E-17 -3.06911 3.06911 0 up vs 1 -9.15333 83.7835 3.53868 8.95403 1cg13436296 MPP3 0.21948 0.358153 4.76E-17 0.612811 -1.63182 0 down vs 1 -9.15269 83.7717 0.348658 0.882343 1cg19813015 0.120465 0.437141 4.77E-17 0.275575 -3.62878 0 down vs 1 -9.15237 83.7659 1.81823 4.60169 1cg00800109 OR2Z1 -0.038985 0.32001 4.78E-17 -0.121824 -8.20854 0 down vs 1 -9.15212 83.7614 2.33665 5.91407 1cg01663008 0.15748 0.401361 4.84E-17 0.392365 -2.54865 0 down vs 1 -9.15016 83.7254 1.07838 2.73056 1cg09650907 FAM104A -0.319835 -0.452598 4.84E-17 0.706665 -1.4151 0 down vs 1 9.15008 83.7239 0.319574 0.809204 1cg16214826 SNTG2 0.060865 0.368485 4.89E-17 0.165176 -6.05414 0 down vs 1 -9.14852 83.6955 1.71572 4.34591 1cg25198340 IL17RD 0.122475 0.35279 4.90E-17 0.347161 -2.88051 0 down vs 1 -9.14822 83.69 0.961752 2.43627 1cg14320852 0.14499 0.378492 4.91E-17 0.383073 -2.61047 0 down vs 1 -9.1479 83.6841 0.988549 2.50433 1cg06202492 TLL1 0.00038 0.338338 4.95E-17 0.00112314 -890.364 0 down vs 1 -9.14685 83.6648 2.07083 5.24731 1cg16018002 NEDD9 0.21675 0.405774 4.98E-17 0.534164 -1.87208 0 down vs 1 -9.14581 83.6459 0.647818 1.64189 1cg22041073 0.14562 0.329681 4.99E-17 0.441699 -2.26398 0 down vs 1 -9.1455 83.6402 0.614248 1.55691 1cg04765483 -0.167625 0.155722 5.04E-17 -1.07644 1.07644 0 up vs 1 -9.14396 83.612 1.89563 4.80642 1cg07876223 0.264525 0.41944 5.05E-17 0.630663 -1.58563 0 down vs 1 -9.14385 83.61 0.435114 1.10327 1cg06446668 FAM155A -0.32973 0.036018 5.05E-17 -9.15458 9.15458 0 up vs 1 -9.14373 83.6078 2.42539 6.14995 1cg25727671 HOXA7 0.239555 0.406963 5.07E-17 0.588641 -1.69883 0 down vs 1 -9.14308 83.5959 0.508124 1.28861 1cg10721556 GFOD1 0.32495 0.397708 5.13E-17 0.817057 -1.2239 0 down vs 1 -9.14138 83.5649 0.095979 0.243494 1cg10942690 0.11536 0.365832 5.19E-17 0.315336 -3.17122 0 down vs 1 -9.13951 83.5307 1.13746 2.88687 1cg12448933 RAB37 -0.162585 0.103198 5.20E-17 -1.57546 1.57546 0 up vs 1 -9.13924 83.5257 1.28078 3.2508 1cg06512316 -0.10861 0.1771 5.22E-17 -0.613269 -1.6306 0 down vs 1 -9.13861 83.5142 1.48002 3.75703 1cg26459859 CNNM1 0.30359 0.43489 5.23E-17 0.698084 -1.43249 0 down vs 1 -9.13833 83.5091 0.312571 0.793507 1cg04825076 0.046365 0.296934 5.28E-17 0.156146 -6.40426 0 down vs 1 -9.13691 83.4832 1.13834 2.89073 1cg19376973 0.1892 0.390109 5.29E-17 0.484993 -2.06189 0 down vs 1 -9.13658 83.4771 0.731839 1.85859 1cg15104158 FGF12 -0.37342 0.0221402 5.30E-17 -16.8661 16.8661 0 up vs 1 -9.13652 83.476 2.83689 7.20472 1cg22371956 SERPINB10 0.251785 0.434407 5.32E-17 0.579606 -1.72531 0 down vs 1 -9.13596 83.4658 0.604679 1.53586 1cg03821311 HIST1H1B 0.299395 0.416832 5.32E-17 0.718263 -1.39225 0 down vs 1 -9.1358 83.4628 0.25005 0.635141 1cg09781932 DSCAML1 0.212355 0.456165 5.44E-17 0.465522 -2.14813 0 down vs 1 -9.13244 83.4015 1.07776 2.73958 1cg26365925 -0.122 0.21791 5.45E-17 -0.559863 -1.78615 0 down vs 1 -9.13229 83.3987 2.09482 5.32505 1cg01679206 WBSCR27 -0.116685 -0.327005 5.47E-17 0.35683 -2.80246 0 down vs 1 9.13164 83.3869 0.802006 2.03899 1cg00746487 0.3082 0.461872 5.48E-17 0.667285 -1.49861 0 down vs 1 -9.13151 83.3845 0.428159 1.08857 1cg16910830 CNTNAP2 -0.194425 0.157311 5.49E-17 -1.23593 1.23593 0 up vs 1 -9.13099 83.375 2.24311 5.70363 1cg06415710 0.161545 0.425422 5.55E-17 0.379729 -2.63346 0 down vs 1 -9.12942 83.3463 1.26247 3.21123 1cg01718116 DPP10 -0.17265 0.143147 5.62E-17 -1.2061 1.2061 0 up vs 1 -9.12752 83.3115 1.80815 4.60113 1cg14999396 CARTPT 0.15151 0.355117 5.71E-17 0.426648 -2.34385 0 down vs 1 -9.12513 83.2679 0.751629 1.91365 1cg20437980 KLHL23 0.112455 0.346994 5.73E-17 0.324083 -3.08563 0 down vs 1 -9.12455 83.2574 0.997354 2.53958 1cg08151596 -0.198975 0.103409 5.83E-17 -1.92416 1.92416 0 up vs 1 -9.12212 83.213 1.65781 4.22357 1cg26918756 S1PR5 -0.22005 0.0279222 5.90E-17 -7.88084 7.88084 0 up vs 1 -9.12033 83.1804 1.11487 2.84144 1cg08373003 PITX1 -0.185265 -0.436734 5.90E-17 0.424206 -2.35734 0 down vs 1 9.12029 83.1797 1.14653 2.92216 1cg20464143 MYO10 -0.05455 0.279219 5.93E-17 -0.195366 -5.11859 0 down vs 1 -9.11948 83.165 2.01981 5.14879 1cg10853409 0.24572 0.39444 5.93E-17 0.622959 -1.60524 0 down vs 1 -9.11942 83.1637 0.401012 1.02226 1cg26288719 FRMPD4 0.183195 0.384753 6.03E-17 0.476137 -2.10024 0 down vs 1 -9.11699 83.1196 0.736574 1.87866 1cg20616779 0.16476 0.446359 6.05E-17 0.36912 -2.70915 0 down vs 1 -9.11648 83.1102 1.43774 3.66743 1cg22863637 -0.02075 0.264955 6.09E-17 -0.0783151 -12.7689 0 down vs 1 -9.11552 83.0926 1.47997 3.77596 1cg07058109 DHX58 -0.297905 -0.433129 6.10E-17 0.687798 -1.45392 0 down vs 1 9.11517 83.0863 0.331531 0.845924 1cg05575273 0.24159 0.452283 6.11E-17 0.534157 -1.87211 0 down vs 1 -9.11505 83.0841 0.804856 2.0537 1cg19433697 -0.27073 0.158932 6.12E-17 -1.70343 1.70343 0 up vs 1 -9.11481 83.0797 3.34713 8.5411 1cg21196136 SNAR-C1 0.18711 0.381074 6.21E-17 0.491007 -2.03663 0 down vs 1 -9.1126 83.0395 0.682121 1.74145 1cg00545229 C1QTNF7 0.113045 0.356162 6.26E-17 0.317397 -3.15062 0 down vs 1 -9.1114 83.0176 1.07164 2.73663 1cg11197630 0.06366 0.328645 6.31E-17 0.193704 -5.16251 0 down vs 1 -9.11006 82.9933 1.2731 3.25203 1cg06680826 MCTP2 0.252845 0.423432 6.43E-17 0.597132 -1.67467 0 down vs 1 -9.10735 82.9438 0.527609 1.34854 1cg12043793 UGT1A5 0.113015 0.270147 6.49E-17 0.418346 -2.39036 0 down vs 1 -9.10581 82.9157 0.447658 1.14458 1cg26411409 TCP10L2 -0.01402 0.329781 6.50E-17 -0.042513 -23.5222 0 down vs 1 -9.10565 82.913 2.14306 5.47957 1cg23182972 MGC87042 0.29707 0.433441 6.52E-17 0.685376 -1.45905 0 down vs 1 -9.10523 82.9052 0.337179 0.862212 1cg23170771 DNAJB6 -0.19821 -0.410834 6.54E-17 0.482458 -2.07272 0 down vs 1 9.10477 82.8969 0.819677 2.09624 1cg09037894 0.17167 0.364958 6.60E-17 0.470383 -2.12593 0 down vs 1 -9.10342 82.8723 0.677371 1.73282 1cg00277165 TRIP13 0.19921 -0.180594 6.65E-17 -1.10308 1.10308 0 up vs 1 9.10228 82.8515 2.6154 6.69227 1cg19224645 -0.31915 -0.00108454 6.69E-17 294.273 294.273 0 up vs 1 -9.10126 82.833 1.83422 4.69444 1cg20803910 XAF1 -0.304475 -0.448419 6.69E-17 0.678997 -1.47276 0 down vs 1 9.10116 82.831 0.375669 0.961498 1cg08623787 RXRG -0.249565 0.17302 6.71E-17 -1.4424 1.4424 0 up vs 1 -9.10091 82.8265 3.23777 8.2873 1cg24258896 0.15749 0.386906 6.73E-17 0.40705 -2.4567 0 down vs 1 -9.1003 82.8154 0.954254 2.4428 1cg27116819 TMEM101 -0.083465 -0.435636 6.75E-17 0.191594 -5.21938 0 down vs 1 9.09981 82.8066 2.24866 5.75698 1cg14095593 0.0366 0.362017 6.77E-17 0.1011 -9.89118 0 down vs 1 -9.09941 82.7993 1.91999 4.91595 1cg03424304 -0.12663 0.156372 6.79E-17 -0.809799 -1.23487 0 down vs 1 -9.09911 82.7938 1.4521 3.71823 1cg14091589 C4orf32 -0.044935 0.210522 6.90E-17 -0.213445 -4.68504 0 down vs 1 -9.09649 82.7462 1.18319 3.03139 1cg27416863 -0.087675 0.349774 6.96E-17 -0.250662 -3.98943 0 down vs 1 -9.0953 82.7245 3.46954 8.89148 1cg05031204 -0.192875 0.0453067 6.98E-17 -4.2571 4.2571 0 up vs 1 -9.09484 82.7162 1.02857 2.63621 1cg10443187 -0.334505 -0.0619613 7.00E-17 5.39861 5.39861 0 up vs 1 -9.09441 82.7082 1.34676 3.45205 1cg18282441 0.23551 0.359044 7.01E-17 0.655936 -1.52454 0 down vs 1 -9.09425 82.7055 0.27669 0.709243 1cg01354961 NXPH1 -0.29373 0.0696268 7.01E-17 -4.21863 4.21863 0 up vs 1 -9.09422 82.7049 2.39378 6.13605 1cg11633102 -0.122795 0.216662 7.03E-17 -0.566759 -1.76442 0 down vs 1 -9.09388 82.6986 2.08923 5.35581 1cg26897717 NCCRP1 0.308265 0.470358 7.06E-17 0.655384 -1.52582 0 down vs 1 -9.09314 82.6852 0.476371 1.22139 1cg24368848 ZSCAN1 -0.10965 0.257373 7.06E-17 -0.426036 -2.34722 0 down vs 1 -9.09309 82.6842 2.44233 6.26206 1cg23483095 NAP1L2 -0.128915 0.239069 7.16E-17 -0.539237 -1.85447 0 down vs 1 -9.09095 82.6453 2.45514 6.29787 1cg06214831 ABLIM2 0.332355 0.474648 7.19E-17 0.700213 -1.42814 0 down vs 1 -9.09036 82.6346 0.367103 0.941806 1cg24224304 -0.2065 0.0845098 7.20E-17 -2.4435 2.4435 0 up vs 1 -9.09024 82.6325 1.53544 3.93929 1cg14751544 MLH1 -0.27143 -0.445479 7.22E-17 0.609299 -1.64123 0 down vs 1 9.0897 82.6227 0.549241 1.40929 1cg14633742 FANCC 0.267345 0.432506 7.23E-17 0.61813 -1.61778 0 down vs 1 -9.08948 82.6186 0.494577 1.26909 1cg27151303 0.023325 0.268795 7.24E-17 0.0867761 -11.5239 0 down vs 1 -9.08931 82.6155 1.09249 2.80343 1cg00674365 ZNF471 -0.310775 0.186944 7.30E-17 -1.66239 1.66239 0 up vs 1 -9.08816 82.5947 4.49145 11.5284 1cg13943133 -0.179235 0.238213 7.45E-17 -0.752414 -1.32906 0 down vs 1 -9.08511 82.5391 3.15954 8.1152 1cg24051554 LOC100132111 -0.03687 -0.406024 7.51E-17 0.0908074 -11.0123 0 down vs 1 9.08385 82.5164 2.47078 6.34789 1cg12630983 FBN2 0.24064 0.427352 7.54E-17 0.563096 -1.7759 0 down vs 1 -9.08313 82.5032 0.632063 1.62415 1cg21565368 SYTL2 0.24476 0.39531 7.57E-17 0.619159 -1.61509 0 down vs 1 -9.08268 82.495 0.410943 1.05606 1cg03536686 0.24664 0.467826 7.57E-17 0.527205 -1.8968 0 down vs 1 -9.0826 82.4936 0.887018 2.27954 1cg15510373 TFR2 0.28817 0.415319 7.65E-17 0.693853 -1.44123 0 down vs 1 -9.08103 82.4651 0.293117 0.75354 1cg02530668 0.32813 0.434187 7.69E-17 0.755734 -1.32322 0 down vs 1 -9.0802 82.4499 0.203938 0.524376 1cg13646855 C9orf144 0.245055 0.409302 7.82E-17 0.598715 -1.67024 0 down vs 1 -9.07773 82.4052 0.489114 1.25832 1cg16167240 BSX -0.22039 0.0967088 7.85E-17 -2.2789 2.2789 0 up vs 1 -9.07713 82.3942 1.82309 4.69079 1cg02987635 C10orf11 -0.147615 0.163403 7.88E-17 -0.903382 -1.10695 0 down vs 1 -9.0765 82.3829 1.75383 4.51322 1cg00931644 KCTD12 -0.178135 0.330332 7.89E-17 -0.539261 -1.85439 0 down vs 1 -9.07627 82.3786 4.68752 12.0633 1cg15289882 PTPRN2 0.13339 0.38488 7.90E-17 0.346575 -2.88538 0 down vs 1 -9.07619 82.3773 1.14673 2.95114 1cg24163668 NFATC1 0.19519 0.374915 7.96E-17 0.520624 -1.92077 0 down vs 1 -9.07501 82.3557 0.585649 1.50758 1cg10542127 -0.28208 -0.0133655 7.97E-17 21.1051 21.1051 0 up vs 1 -9.07481 82.3523 1.30918 3.37024 1cg23472261 EPHB6 0.246085 0.404835 8.08E-17 0.607865 -1.6451 0 down vs 1 -9.0727 82.3139 0.456926 1.17682 1cg14882265 HOXA5 0.093755 0.311472 8.09E-17 0.301007 -3.32219 0 down vs 1 -9.07258 82.3116 0.859412 2.21348 1cg07063890 ZNF469 0.19138 0.403854 8.13E-17 0.473884 -2.11022 0 down vs 1 -9.07177 82.297 0.818521 2.10854 1cg16191088 ADAMTS10 0.091885 0.421511 8.20E-17 0.217989 -4.58738 0 down vs 1 -9.07059 82.2756 1.96998 5.07605 1cg13291607 0.074825 0.41036 8.21E-17 0.18234 -5.48426 0 down vs 1 -9.07036 82.2714 2.04124 5.25993 1cg03518457 BDKRB2 0.061325 0.30282 8.35E-17 0.202513 -4.93795 0 down vs 1 -9.06784 82.2257 1.05738 2.72622 1cg00722329 PLCH2 -0.13583 0.12474 8.35E-17 -1.0889 1.0889 0 up vs 1 -9.06781 82.2252 1.23103 3.17394 1cg22337892 -0.0615 0.194458 8.38E-17 -0.316263 -3.16192 0 down vs 1 -9.06722 82.2145 1.18784 3.06298 1cg25968569 KCTD12 -0.20974 0.30627 8.39E-17 -0.68482 -1.46024 0 down vs 1 -9.06695 82.2096 4.82763 12.4494 1cg07159758 MSI2 0.177125 0.411095 8.41E-17 0.430862 -2.32093 0 down vs 1 -9.06675 82.206 0.992516 2.55959 1cg06353830 0.195655 0.395343 8.47E-17 0.4949 -2.02061 0 down vs 1 -9.06562 82.1854 0.722971 1.86493 1cg13987489 BCL11B 0.07227 0.291798 8.49E-17 0.247671 -4.03761 0 down vs 1 -9.06516 82.1772 0.873771 2.25415 1cg02960418 PDGFA 0.104295 0.328758 8.54E-17 0.31724 -3.15219 0 down vs 1 -9.0643 82.1615 0.913496 2.35708 1cg25735398 DIRAS1 0.27981 0.471553 8.55E-17 0.59338 -1.68526 0 down vs 1 -9.0642 82.1598 0.666588 1.72002 1cg06085204 XAF1 -0.199935 -0.408118 8.59E-17 0.489896 -2.04125 0 down vs 1 9.06355 82.1479 0.78579 2.0279 1cg19630629 CHRM2 -0.214015 0.132056 8.63E-17 -1.62064 1.62064 0 up vs 1 -9.06282 82.1347 2.17144 5.60476 1cg00276443 ASB10 0.212595 0.41797 8.63E-17 0.508637 -1.96604 0 down vs 1 -9.06272 82.1328 0.76474 1.97393 1cg24864831 ZNF774 0.21655 0.420775 8.66E-17 0.514645 -1.94309 0 down vs 1 -9.06233 82.1258 0.7562 1.95206 1cg02287817 EGR4 -0.14268 0.212094 8.67E-17 -0.672721 -1.4865 0 down vs 1 -9.06211 82.1219 2.28203 5.89112 1cg27105446 PSMB5 0.183205 0.373106 8.68E-17 0.491027 -2.03655 0 down vs 1 -9.06184 82.117 0.653839 1.68801 1cg12111783 BCOR -0.187985 0.145168 8.70E-17 -1.29495 1.29495 0 up vs 1 -9.06153 82.1113 2.01236 5.19563 1cg17888086 ZC3H12A -0.27437 -0.43091 8.74E-17 0.636722 -1.57054 0 down vs 1 9.06089 82.0997 0.444294 1.14727 1cg02796638 0.053185 0.340162 8.75E-17 0.156352 -6.39582 0 down vs 1 -9.06071 82.0965 1.49318 3.85587 1cg01461856 -0.1579 -0.383337 8.79E-17 0.41191 -2.42772 0 down vs 1 9.05996 82.0828 0.921439 2.37985 1cg07920195 PSG11 0.235285 0.413394 8.81E-17 0.569155 -1.75699 0 down vs 1 -9.05973 82.0787 0.57516 1.48557 1cg23591139 0.105935 0.359131 8.90E-17 0.294976 -3.39011 0 down vs 1 -9.05808 82.0488 1.16234 3.00329 1cg12449852 LCE1B 0.0935 0.373566 8.93E-17 0.25029 -3.99536 0 down vs 1 -9.05769 82.0417 1.42213 3.67487 1cg17357285 -0.248355 0.188751 8.96E-17 -1.31578 1.31578 0 up vs 1 -9.0571 82.0311 3.4641 8.95257 1cg19384073 0.1186 0.41198 9.04E-17 0.287878 -3.47369 0 down vs 1 -9.05569 82.0055 1.56055 4.03433 1cg15092343 MSX1 -0.056925 0.285945 9.07E-17 -0.199077 -5.02319 0 down vs 1 -9.0552 81.9966 2.13146 5.51083 1cg06168204 0.00835 0.326043 9.09E-17 0.0256101 -39.0471 0 down vs 1 -9.05484 81.9901 1.82993 4.73161 1cg08160462 0.111995 0.295379 9.15E-17 0.379156 -2.63743 0 down vs 1 -9.05394 81.9738 0.609737 1.5769 1cg10273072 C21orf34 -0.010915 0.304785 9.19E-17 -0.0358121 -27.9235 0 down vs 1 -9.05331 81.9623 1.80704 4.674 1cg26693451 CARD14 0.25455 0.387551 9.28E-17 0.656817 -1.52249 0 down vs 1 -9.05185 81.9361 0.320721 0.829829 1cg12387232 0.073095 0.388247 9.40E-17 0.188269 -5.31155 0 down vs 1 -9.04982 81.8993 1.80077 4.66139 1cg07094298 TNIP2 -0.140705 -0.371893 9.42E-17 0.378348 -2.64307 0 down vs 1 9.0495 81.8934 0.969058 2.50863 1cg05842855 -0.199825 0.155175 9.57E-17 -1.28774 1.28774 0 up vs 1 -9.04711 81.8502 2.28494 5.91823 1cg22363327 SFRS13B -0.10935 0.126809 9.57E-17 -0.862319 -1.15966 0 down vs 1 -9.0471 81.8501 1.01118 2.61906 1cg08692733 RBM20 -0.13055 0.199784 9.62E-17 -0.653456 -1.53033 0 down vs 1 -9.04638 81.8371 1.97845 5.12519 1cg15074709 -0.08947 0.138298 9.65E-17 -0.646934 -1.54575 0 down vs 1 -9.04587 81.8277 0.9406 2.43692 1cg24621599 0.275825 0.470118 9.66E-17 0.586714 -1.70441 0 down vs 1 -9.04578 81.8261 0.684435 1.77328 1cg12559119 -0.10036 0.221971 9.67E-17 -0.452132 -2.21174 0 down vs 1 -9.04551 81.8212 1.88374 4.8808 1cg18888520 ZSCAN18 -0.3491 0.0274552 9.70E-17 -12.7153 12.7153 0 up vs 1 -9.0451 81.8139 2.57084 6.66168 1cg11323585 ADARB2 0.17425 0.393714 9.75E-17 0.44258 -2.25948 0 down vs 1 -9.04428 81.799 0.873265 2.26326 1cg22121570 LOC145845 -0.11917 0.252861 9.76E-17 -0.471286 -2.12185 0 down vs 1 -9.04413 81.7963 2.50944 6.50398 1cg13585776 LHX2 -0.08202 0.116386 9.77E-17 -0.704723 -1.419 0 down vs 1 -9.04394 81.7929 0.71372 1.8499 1cg13695954 NPTX2 0.19956 0.405888 9.91E-17 0.491663 -2.03392 0 down vs 1 -9.04187 81.7555 0.771854 2.00149 1cg16003832 -0.27313 0.192563 9.92E-17 -1.41839 1.41839 0 up vs 1 -9.04168 81.752 3.93204 10.1966 1cg03980424 0.04463 0.336346 9.95E-17 0.132691 -7.53633 0 down vs 1 -9.0412 81.7433 1.54291 4.00151 1cg02301319 0.070385 0.36939 9.95E-17 0.190544 -5.24814 0 down vs 1 -9.04119 81.7431 1.62097 4.20397 1cg18940674 -0.01702 0.309086 9.98E-17 -0.0550657 -18.1601 0 down vs 1 -9.04078 81.7357 1.92812 5.00102 1cg14627089 -0.28945 0.0946407 1.01E-16 -3.05841 3.05841 0 up vs 1 -9.03919 81.7069 2.67476 6.94005 1cg10135483 HTR1E -0.244965 0.158966 1.01E-16 -1.54099 1.54099 0 up vs 1 -9.03884 81.7006 2.95824 7.67616 1cg11862714 HOXB3 0.10626 0.310568 1.02E-16 0.342147 -2.92272 0 down vs 1 -9.03688 81.6652 0.756814 1.96466 1cg00905220 ABR 0.209315 0.403262 1.02E-16 0.519055 -1.92658 0 down vs 1 -9.03674 81.6627 0.681999 1.7705 1cg05988291 SHROOM3 0.14359 0.380142 1.03E-16 0.377728 -2.64741 0 down vs 1 -9.03633 81.6552 1.01454 2.63404 1cg09789697 0.30882 0.435337 1.03E-16 0.709382 -1.40968 0 down vs 1 -9.0355 81.6403 0.29021 0.753606 1cg08550260 LDLRAD1 0.155115 0.378076 1.04E-16 0.410274 -2.43739 0 down vs 1 -9.03485 81.6285 0.901316 2.34084 1cg00313685 CUX1 0.10964 0.283553 1.04E-16 0.386665 -2.58622 0 down vs 1 -9.03429 81.6184 0.548381 1.42439 1cg05963604 APPL1 -0.226265 -0.414768 1.04E-16 0.545522 -1.83311 0 down vs 1 9.03429 81.6184 0.64425 1.67341 1cg03113800 GPNMB 0.153595 0.396491 1.06E-16 0.387386 -2.58141 0 down vs 1 -9.0323 81.5824 1.06969 2.77971 1cg16599298 CLRN2 0.29771 0.427794 1.06E-16 0.695919 -1.43695 0 down vs 1 -9.03227 81.5818 0.306806 0.797273 1cg00386586 -0.117815 0.288123 1.06E-16 -0.408905 -2.44556 0 down vs 1 -9.03195 81.5762 2.98771 7.76444 1cg24579377 SORCS2 0.2643 0.441448 1.06E-16 0.598711 -1.67026 0 down vs 1 -9.03096 81.5583 0.568974 1.47897 1cg12297642 LACTB 0.13627 0.32337 1.07E-16 0.421406 -2.37301 0 down vs 1 -9.03075 81.5544 0.634693 1.64988 1cg10298215 HLA-DQB2 0.122885 0.343769 1.07E-16 0.357464 -2.79748 0 down vs 1 -9.03035 81.5472 0.884596 2.2997 1cg08615111 PLCH2 -0.14499 0.218644 1.07E-16 -0.663133 -1.50799 0 down vs 1 -9.03029 81.5461 2.39743 6.23274 1cg20331241 TNXB 0.214705 0.454851 1.08E-16 0.472034 -2.11849 0 down vs 1 -9.02836 81.5113 1.0456 2.71947 1cg04573316 MIR519D -0.18401 0.214697 1.09E-16 -0.857069 -1.16677 0 down vs 1 -9.02719 81.4901 2.88221 7.49819 1cg03801871 LBH -0.164815 0.223359 1.10E-16 -0.737892 -1.35521 0 down vs 1 -9.02658 81.4791 2.73194 7.10822 1cg10157715 -0.284055 0.0075799 1.10E-16 -37.4748 37.4748 0 up vs 1 -9.0265 81.4776 1.54204 4.01231 1cg23677311 DOCK5 0.230875 0.399078 1.10E-16 0.57852 -1.72855 0 down vs 1 -9.02625 81.4732 0.512964 1.33478 1cg08847856 0.02199 0.406437 1.10E-16 0.0541044 -18.4828 0 down vs 1 -9.02597 81.4682 2.67972 6.97329 1cg20418769 0.154515 0.38538 1.10E-16 0.400942 -2.49413 0 down vs 1 -9.02579 81.4649 0.966348 2.51478 1cg25533993 ZNF473 0.174395 0.386433 1.11E-16 0.451294 -2.21585 0 down vs 1 -9.02458 81.443 0.815165 2.12191 1cg09391371 0.31321 0.421861 1.11E-16 0.742448 -1.3469 0 down vs 1 -9.02454 81.4423 0.214037 0.557152 1cg03571393 0.11208 0.360943 1.13E-16 0.31052 -3.22041 0 down vs 1 -9.0296 81.5336 1.1218 2.88935 1cg24006352 KCND3 0.123985 0.359652 1.14E-16 0.344736 -2.90077 0 down vs 1 -9.02091 81.3768 1.00697 2.62332 1cg20623172 SLMO2 0.05161 0.267404 1.14E-16 0.193004 -5.18125 0 down vs 1 -9.02077 81.3743 0.844301 2.19961 1cg11948766 -0.1405 0.323168 1.15E-16 -0.434759 -2.30012 0 down vs 1 -9.01884 81.3395 3.89791 10.1593 1cg25601481 0.15166 0.377581 1.15E-16 0.401662 -2.48966 0 down vs 1 -9.01868 81.3366 0.925407 2.41203 1cg02664177 PDGFA 0.218325 0.437884 1.16E-16 0.498592 -2.00565 0 down vs 1 -9.01799 81.3242 0.874014 2.27842 1cg21757281 0.20126 0.380467 1.16E-16 0.528981 -1.89043 0 down vs 1 -9.0179 81.3224 0.582275 1.51794 1cg17613214 WNT7A 0.21713 0.462522 1.17E-16 0.469448 -2.13016 0 down vs 1 -9.01718 81.3095 1.09179 2.84665 1cg25325645 PDE10A 0.28241 0.422523 1.17E-16 0.668389 -1.49613 0 down vs 1 -9.01703 81.3068 0.355939 0.928079 1cg06866655 SLC9A3 0.17305 0.427807 1.17E-16 0.404505 -2.47216 0 down vs 1 -9.01652 81.2975 1.17671 3.06851 1cg09825080 0.11904 0.290264 1.17E-16 0.410109 -2.43838 0 down vs 1 -9.01603 81.2889 0.531556 1.38629 1cg15861089 KRT86 0.00953 0.199244 1.18E-16 0.0478307 -20.9071 0 down vs 1 -9.01596 81.2876 0.652557 1.70188 1cg02959486 0.03427 0.345485 1.18E-16 0.099194 -10.0813 0 down vs 1 -9.01596 81.2875 1.75605 4.57983 1cg05590053 0.2153 0.421906 1.18E-16 0.510303 -1.95962 0 down vs 1 -9.01583 81.2852 0.773935 2.0185 1cg04601510 0.262845 0.421853 1.19E-16 0.623072 -1.60495 0 down vs 1 -9.01429 81.2575 0.458412 1.19599 1cg16651780 C16orf61 -0.19404 -0.399327 1.20E-16 0.485918 -2.05796 0 down vs 1 9.01234 81.2222 0.764082 1.99435 1cg13077031 JARID2 0.109795 0.329935 1.21E-16 0.332778 -3.00501 0 down vs 1 -9.0119 81.2144 0.87865 2.29361 1cg23210268 GREM2 0.092915 0.373392 1.21E-16 0.24884 -4.01864 0 down vs 1 -9.01187 81.2138 1.42631 3.72322 1cg02106466 PCDH12 -0.20512 -0.00036701 1.21E-16 558.894 558.894 0 up vs 1 -9.0115 81.2072 0.760114 1.98436 1cg24320816 GFOD1 0.232995 0.381506 1.25E-16 0.610725 -1.6374 0 down vs 1 -9.00681 81.1226 0.399883 1.04503 1cg10802444 GRIP1 -0.128955 0.267702 1.25E-16 -0.481711 -2.07593 0 down vs 1 -9.00672 81.1209 2.85265 7.45506 1cg13462275 TCP10 0.14205 0.403236 1.25E-16 0.352275 -2.83869 0 down vs 1 -9.00657 81.1184 1.23685 3.23246 1cg03753331 DPP10 -0.180075 0.178109 1.26E-16 -1.01104 1.01104 0 up vs 1 -9.00513 81.0924 2.32611 6.08115 1cg07802362 DNM3 0.232435 0.409922 1.27E-16 0.567023 -1.7636 0 down vs 1 -9.00483 81.0869 0.571149 1.49326 1cg10613546 PTPRN2 0.078115 0.387883 1.27E-16 0.201388 -4.96554 0 down vs 1 -9.00372 81.0671 1.73977 4.5497 1cg07717083 TNXB -0.017005 0.306573 1.28E-16 -0.055468 -18.0284 0 down vs 1 -9.00289 81.052 1.89835 4.96533 1cg24641417 KCNAB2 0.303465 0.474714 1.28E-16 0.639259 -1.56431 0 down vs 1 -9.00254 81.0458 0.531709 1.39085 1cg00960419 0.097325 0.273537 1.29E-16 0.355802 -2.81055 0 down vs 1 -9.00205 81.0368 0.562972 1.47279 1cg13982956 PBX3 0.036275 0.278241 1.29E-16 0.130373 -7.67032 0 down vs 1 -9.00174 81.0314 1.06151 2.77721 1cg26711406 -0.01317 0.256603 1.29E-16 -0.0513244 -19.4839 0 down vs 1 -9.00139 81.0251 1.31952 3.45248 1cg23620902 -0.157525 0.19717 1.30E-16 -0.798929 -1.25167 0 down vs 1 -9.00127 81.0228 2.28102 5.96838 1cg06665913 ZBTB16 0.1722 0.413999 1.30E-16 0.415943 -2.40418 0 down vs 1 -9.00103 81.0185 1.06005 2.77382 1cg19083774 ABLIM2 0.21004 0.40054 1.30E-16 0.524392 -1.90697 0 down vs 1 -9.00086 81.0155 0.657974 1.72178 1cg22597733 GRIA2 -0.298455 0.0470314 1.33E-16 -6.34586 6.34586 0 up vs 1 -8.9978 80.9604 2.16411 5.66687 1cg02851312 PTPRN2 0.125975 0.35537 1.34E-16 0.35449 -2.82095 0 down vs 1 -8.99646 80.9362 0.954079 2.49906 1cg17533575 IDO2 0.001545 0.306147 1.34E-16 0.0050466 -198.153 0 down vs 1 -8.99639 80.9351 1.68222 4.40638 1cg23095192 ZEB2 -0.020245 0.218582 1.36E-16 -0.0926197 -10.7968 0 down vs 1 -8.99432 80.8978 1.03415 2.71009 1cg05525106 C6orf150 -0.373235 -0.471877 1.36E-16 0.790959 -1.26429 0 down vs 1 8.99415 80.8947 0.176417 0.462334 1cg23805623 TMEM132D 0.075685 0.415796 1.37E-16 0.182024 -5.49378 0 down vs 1 -8.99277 80.8698 2.0973 5.49806 1cg18957463 CORIN 0.12706 0.381093 1.37E-16 0.33341 -2.99931 0 down vs 1 -8.99261 80.867 1.17003 3.06734 1cg04361266 LYRM1 0.180055 0.369456 1.37E-16 0.487352 -2.0519 0 down vs 1 -8.99231 80.8617 0.650401 1.70519 1cg13715631 PITX1 -0.059105 -0.390009 1.38E-16 0.151548 -6.59858 0 down vs 1 8.99187 80.8537 1.98528 5.20546 1cg01411759 MIR498 0.088325 0.376573 1.38E-16 0.23455 -4.26349 0 down vs 1 -8.99126 80.8428 1.50643 3.95043 1cg18608055 SBNO2 -0.058595 -0.292576 1.38E-16 0.200273 -4.99319 0 down vs 1 8.99125 80.8427 0.992609 2.603 1cg09461094 MYLK4 0.244765 0.405435 1.38E-16 0.60371 -1.65643 0 down vs 1 -8.99111 80.84 0.468045 1.22743 1cg03122282 C1orf68 0.09882 0.351218 1.39E-16 0.281364 -3.55412 0 down vs 1 -8.99026 80.8248 1.15502 3.02957 1cg13424029 -0.05452 0.27051 1.40E-16 -0.201545 -4.96166 0 down vs 1 -8.98942 80.8097 1.91542 5.025 1cg13583602 GFOD1 0.336125 0.430802 1.42E-16 0.780231 -1.28167 0 down vs 1 -8.98716 80.769 0.16252 0.426578 1cg14207539 C10orf11 -0.045715 0.299519 1.43E-16 -0.152628 -6.55187 0 down vs 1 -8.98586 80.7456 2.16095 5.67363 1cg10215102 C6orf174 0.142725 0.362736 1.44E-16 0.393468 -2.5415 0 down vs 1 -8.98484 80.7273 0.877617 2.30473 1cg19298856 FLT3LG -0.03321 0.189059 1.46E-16 -0.17566 -5.69283 0 down vs 1 -8.98299 80.694 0.895725 2.35326 1cg03627261 ARL15 0.205695 0.385636 1.48E-16 0.533392 -1.87479 0 down vs 1 -8.98119 80.6618 0.587051 1.54292 1cg07475654 PNKD -0.208855 -0.423551 1.48E-16 0.493105 -2.02796 0 down vs 1 8.9811 80.6602 0.835726 2.19655 1cg03175417 C22orf25 0.06161 -0.192237 1.49E-16 -0.32049 -3.12022 0 down vs 1 8.9804 80.6476 1.16832 3.07118 1cg05232628 CAV1 0.135645 0.346392 1.49E-16 0.391594 -2.55366 0 down vs 1 -8.9799 80.6385 0.805267 2.11706 1cg00469547 0.245035 0.412959 1.49E-16 0.593364 -1.68531 0 down vs 1 -8.97958 80.6328 0.511261 1.34421 1cg10026473 HPDL -0.36252 -0.488804 1.50E-16 0.741647 -1.34835 0 down vs 1 8.97876 80.618 0.289145 0.76036 1cg23571839 BRSK2 0.34682 0.476455 1.51E-16 0.727918 -1.37378 0 down vs 1 -8.97829 80.6098 0.304691 0.801324 1cg01625044 ZNF311 0.27271 0.421322 1.52E-16 0.647272 -1.54495 0 down vs 1 -8.97707 80.5878 0.40043 1.0534 1cg11258452 SRRM4 0.25272 0.447721 1.52E-16 0.564459 -1.77161 0 down vs 1 -8.97689 80.5846 0.68943 1.81373 1cg00425710 MT1A 0.20909 0.373083 1.52E-16 0.560438 -1.78432 0 down vs 1 -8.97677 80.5825 0.487605 1.28281 1cg08763351 SPRR4 0.173555 0.421738 1.52E-16 0.411524 -2.42999 0 down vs 1 -8.97676 80.5821 1.11676 2.93804 1cg02213684 PITX1 -0.05865 -0.364942 1.54E-16 0.16071 -6.22237 0 down vs 1 8.97546 80.5588 1.70094 4.47623 1cg03754403 -0.29307 0.0528263 1.55E-16 -5.54781 5.54781 0 up vs 1 -8.9735 80.5238 2.16925 5.71112 1cg17594003 ZEB2 -0.04151 0.286522 1.57E-16 -0.144875 -6.90249 0 down vs 1 -8.97249 80.5056 1.95097 5.13761 1cg27103937 -0.01116 0.286904 1.58E-16 -0.038898 -25.7083 0 down vs 1 -8.97078 80.4749 1.61078 4.24339 1cg14106046 B3GNTL1 -0.06122 -0.355635 1.58E-16 0.172143 -5.80913 0 down vs 1 8.97071 80.4736 1.57159 4.14019 1cg19354039 0.01636 0.198398 1.61E-16 0.0824603 -12.127 0 down vs 1 -8.96784 80.4221 0.60082 1.58382 1cg19585918 C6orf142 0.224295 0.414533 1.62E-16 0.541079 -1.84816 0 down vs 1 -8.96727 80.4118 0.656164 1.72993 1cg01695225 EMX1 -0.195205 0.149744 1.62E-16 -1.30359 1.30359 0 up vs 1 -8.96716 80.4099 2.15739 5.68794 1cg09171562 MPRIP 0.04596 -0.184815 1.62E-16 -0.248681 -4.02121 0 down vs 1 8.96688 80.405 0.965596 2.54594 1cg26354128 KIAA1409 -0.256955 0.185171 1.63E-16 -1.38766 1.38766 0 up vs 1 -8.96666 80.4009 3.54414 9.34513 1cg26799656 -0.07726 0.379802 1.64E-16 -0.203422 -4.91589 0 down vs 1 -8.96495 80.3703 3.78763 9.99097 1cg14848469 0.24777 0.439968 1.65E-16 0.563155 -1.77571 0 down vs 1 -8.96474 80.3666 0.669755 1.76675 1cg24218935 HCCA2 0.203035 0.409034 1.65E-16 0.496377 -2.0146 0 down vs 1 -8.96421 80.3571 0.769393 2.02983 1cg12659114 0.003695 0.34447 1.66E-16 0.0107266 -93.226 0 down vs 1 -8.96385 80.3507 2.10549 5.5552 1cg04338271 0.192895 0.41722 1.67E-16 0.462334 -2.16294 0 down vs 1 -8.96299 80.3353 0.912376 2.40771 1cg02382016 -0.212095 0.0763747 1.67E-16 -2.77703 2.77703 0 up vs 1 -8.96278 80.3315 1.50875 3.9817 1cg10999992 H1F0 0.022435 0.369767 1.67E-16 0.0606733 -16.4817 0 down vs 1 -8.96278 80.3314 2.1873 5.77243 1cg08481464 C10orf11 0.125585 0.362075 1.67E-16 0.346848 -2.88311 0 down vs 1 -8.96225 80.322 1.01401 2.67636 1cg03494277 CUX1 0.25141 0.398325 1.68E-16 0.631168 -1.58436 0 down vs 1 -8.96144 80.3074 0.391335 1.03307 1cg21858113 SCN4B -0.14921 0.132989 1.68E-16 -1.12197 1.12197 0 up vs 1 -8.96869 80.4374 1.44247 3.76589 1cg27221674 FAM20C 0.16243 0.355077 1.70E-16 0.45745 -2.18603 0 down vs 1 -8.95957 80.2739 0.67289 1.77707 1cg15627072 PLCH2 -0.04735 0.206558 1.70E-16 -0.229234 -4.36236 0 down vs 1 -8.95954 80.2734 1.16888 3.08698 1cg13656878 PAPPA -0.246005 0.128256 1.71E-16 -1.91808 1.91808 0 up vs 1 -8.95883 80.2606 2.53961 6.70812 1cg27528510 EMID2 -0.29114 0.120294 1.72E-16 -2.42024 2.42024 0 up vs 1 -8.95854 80.2555 3.06915 8.10735 1cg12037947 CD93 0.06843 0.307342 1.72E-16 0.222651 -4.49134 0 down vs 1 -8.95847 80.2542 1.03489 2.73378 1cg02260587 PCDHB2 -0.156445 0.131308 1.73E-16 -1.19144 1.19144 0 up vs 1 -8.95708 80.2293 1.50126 3.96698 1cg22699768 0.25882 0.394594 1.74E-16 0.655915 -1.52459 0 down vs 1 -8.95672 80.2228 0.334234 0.883259 1cg17563504 0.10036 0.348425 1.74E-16 0.288039 -3.47175 0 down vs 1 -8.95669 80.2222 1.11571 2.94843 1cg14458619 PTPRD -0.07873 0.248141 1.75E-16 -0.31728 -3.15179 0 down vs 1 -8.95594 80.2089 1.93718 5.12016 1cg20585038 -0.31528 0.0202402 1.75E-16 -15.5769 15.5769 0 up vs 1 -8.95537 80.1986 2.04106 5.39541 1cg02841692 0.060385 0.254691 1.76E-16 0.237091 -4.21778 0 down vs 1 -8.95463 80.1853 0.684525 1.8098 1cg03015498 0.05282 0.340819 1.77E-16 0.15498 -6.45245 0 down vs 1 -8.954 80.1742 1.50383 3.97649 1cg02763002 GLT1D1 0.059945 0.346769 1.77E-16 0.172867 -5.78479 0 down vs 1 -8.95392 80.1726 1.49159 3.9442 1cg16044777 LAMA4 0.162175 0.397126 1.82E-16 0.408372 -2.44875 0 down vs 1 -8.94956 80.0945 1.00086 2.64914 1cg05615464 -0.335955 0.03385 1.82E-16 -9.92482 9.92482 0 up vs 1 -8.94953 80.0941 2.4795 6.56295 1cg14420550 0.27243 0.437651 1.83E-16 0.622483 -1.60647 0 down vs 1 -8.94896 80.0838 0.494932 1.3102 1cg25171014 KRT16 0.14381 0.296123 1.86E-16 0.485642 -2.05913 0 down vs 1 -8.94619 80.0343 0.420623 1.11417 1cg18917640 GGPS1 0.329155 0.412075 1.86E-16 0.798775 -1.25192 0 down vs 1 -8.94612 80.0331 0.124662 0.330218 1cg05005791 PLCH2 0.147945 0.346892 1.87E-16 0.426488 -2.34473 0 down vs 1 -8.94531 80.0186 0.717615 1.90124 1cg24239420 PALLD 0.276485 0.42733 1.88E-16 0.647005 -1.54558 0 down vs 1 -8.945 80.013 0.412555 1.09309 1cg24286765 -0.06689 0.195086 1.88E-16 -0.342875 -2.91651 0 down vs 1 -8.94497 80.0124 1.24434 3.29699 1cg14425564 LHX2 -0.204325 0.17531 1.90E-16 -1.16551 1.16551 0 up vs 1 -8.94342 79.9847 2.61306 6.92595 1cg04562683 TP73 -0.09116 0.320707 1.90E-16 -0.284247 -3.51807 0 down vs 1 -8.94299 79.9771 3.07562 8.15272 1cg12983394 EDNRB -0.232365 0.0694077 1.90E-16 -3.34783 3.34783 0 up vs 1 -8.9428 79.9738 1.65112 4.3769 1cg00175895 0.24085 0.422959 1.90E-16 0.569441 -1.75611 0 down vs 1 -8.94271 79.9721 0.601284 1.59396 1cg05614657 ZMAT4 0.066835 0.333909 1.91E-16 0.200159 -4.99602 0 down vs 1 -8.94225 79.9639 1.29324 3.42864 1cg17826753 KCNV1 -0.317505 0.0609742 1.92E-16 -5.2072 5.2072 0 up vs 1 -8.9415 79.9503 2.59718 6.8868 1cg08316983 0.02537 0.346334 1.92E-16 0.073253 -13.6513 0 down vs 1 -8.94105 79.9424 1.8678 4.95324 1cg26421734 0.047425 0.297499 1.93E-16 0.159412 -6.27305 0 down vs 1 -8.94033 79.9294 1.13385 3.00736 1cg27639457 CTNND2 -0.137415 0.0957371 1.93E-16 -1.43534 1.43534 0 up vs 1 -8.94026 79.9282 0.985591 2.61416 1cg16449808 0.159895 0.407799 1.94E-16 0.392093 -2.55042 0 down vs 1 -8.94006 79.9246 1.11426 2.95556 1cg23068621 -0.147435 0.208616 1.95E-16 -0.706729 -1.41497 0 down vs 1 -8.93907 79.907 2.29849 6.09808 1cg04313565 XYLT1 -0.16018 0.201826 1.96E-16 -0.793653 -1.26 0 down vs 1 -8.9386 79.8986 2.37602 6.30445 1cg01899130 GCH1 -0.164955 -0.425947 1.96E-16 0.387267 -2.5822 0 down vs 1 8.93855 79.8977 1.23501 3.27698 1cg24932628 OLFM4 -0.012165 0.277086 1.96E-16 -0.0439034 -22.7773 0 down vs 1 -8.9382 79.8913 1.51693 4.02534 1cg01627212 0.02706 0.266916 1.97E-16 0.10138 -9.86386 0 down vs 1 -8.93732 79.8757 1.04308 2.76847 1cg10972973 -0.31465 0.073168 1.98E-16 -4.30037 4.30037 0 up vs 1 -8.93713 79.8723 2.72693 7.23792 1cg08541518 BAI3 -0.3579 0.0892954 1.98E-16 -4.00805 4.00805 0 up vs 1 -8.93702 79.8704 3.62587 9.62415 1cg07100000 FGF18 -0.212315 -0.418988 1.98E-16 0.506733 -1.97343 0 down vs 1 8.93684 79.8672 0.774437 2.05567 1cg12409683 0.231005 0.380756 1.98E-16 0.606701 -1.64826 0 down vs 1 -8.93673 79.8652 0.406592 1.07929 1cg27563126 PRRX2 -0.12131 0.156594 1.98E-16 -0.774677 -1.29086 0 down vs 1 -8.93665 79.8638 1.40026 3.71702 1cg27532955 -0.01372 0.30574 1.98E-16 -0.0448748 -22.2842 0 down vs 1 -8.93644 79.86 1.85033 4.91198 1cg25081106 SLITRK5 -0.26291 0.163243 1.98E-16 -1.61054 1.61054 0 up vs 1 -8.9364 79.8592 3.29268 8.74099 1cg02601489 TDH -0.067545 0.209285 1.99E-16 -0.322742 -3.09845 0 down vs 1 -8.93563 79.8455 1.38945 3.68917 1cg02891048 PARK7 0.302605 0.444075 2.00E-16 0.681427 -1.46751 0 down vs 1 -8.93523 79.8383 0.362868 0.963547 1cg01269798 ZNF311 0.08505 0.363115 2.01E-16 0.234224 -4.26942 0 down vs 1 -8.93821 79.8917 1.4012 3.70067 1cg14768765 -0.22196 0.120475 2.01E-16 -1.84238 1.84238 0 up vs 1 -8.93423 79.8205 2.12605 5.64671 1cg26463171 0.174185 0.380055 2.03E-16 0.458316 -2.1819 0 down vs 1 -8.93301 79.7987 0.768427 2.04147 1cg07924874 ZSCAN18 -0.338855 0.0294876 2.03E-16 -11.4914 11.4914 0 up vs 1 -8.93295 79.7976 2.45993 6.53533 1cg14375890 0.211785 -0.196821 2.06E-16 -1.07603 1.07603 0 up vs 1 8.9309 79.761 3.0271 8.04585 1cg06746997 GPR6 0.19324 0.351412 2.06E-16 0.549895 -1.81853 0 down vs 1 -8.93064 79.7563 0.453606 1.20573 1cg26418690 TNXB 0.26337 0.460764 2.07E-16 0.571594 -1.74949 0 down vs 1 -8.93029 79.7501 0.70646 1.87799 1cg09558547 -0.1781 0.21861 2.10E-16 -0.814692 -1.22746 0 down vs 1 -8.92789 79.7073 2.85341 7.58932 1cg24112692 ST8SIA1 0.009345 0.302862 2.10E-16 0.0308556 -32.409 0 down vs 1 -8.92783 79.7061 1.56202 4.15461 1cg04145765 TRIM55 0.050885 0.282339 2.11E-16 0.180226 -5.54857 0 down vs 1 -8.92682 79.6881 0.971288 2.58399 1cg16514818 0.155995 0.44735 2.12E-16 0.348709 -2.86772 0 down vs 1 -8.92634 79.6795 1.53909 4.09499 1cg05190002 -0.273975 -0.412799 2.12E-16 0.6637 -1.5067 0 down vs 1 8.92616 79.6764 0.349422 0.929729 1cg16246661 GALNT2 0.22858 0.39671 2.12E-16 0.576189 -1.73554 0 down vs 1 -8.92603 79.674 0.512519 1.36373 1cg13609065 SLC13A5 0.231805 0.437933 2.13E-16 0.529316 -1.88923 0 down vs 1 -8.92552 79.6649 0.770357 2.05003 1cg21221840 TBXA2R 0.158295 0.430727 2.14E-16 0.367507 -2.72104 0 down vs 1 -8.92515 79.6583 1.34565 3.58128 1cg23431444 JARID2 0.283575 0.404924 2.14E-16 0.700317 -1.42792 0 down vs 1 -8.92469 79.6501 0.266986 0.710621 1cg15961693 0.09107 0.332046 2.16E-16 0.274269 -3.64605 0 down vs 1 -8.92322 79.6238 1.05285 2.80323 1cg06363129 SOSTDC1 -0.27525 0.221459 2.17E-16 -1.2429 1.2429 0 up vs 1 -8.92314 79.6224 4.47323 11.9103 1cg02125316 FGF18 -0.24272 -0.461386 2.19E-16 0.526067 -1.9009 0 down vs 1 8.9211 79.586 0.866924 2.3093 1cg09567180 FAM24B 0.11199 0.372029 2.21E-16 0.301025 -3.32199 0 down vs 1 -8.92006 79.5675 1.22602 3.2666 1cg26813646 SLC18A3 -0.270835 0.00190206 2.22E-16 -142.39 142.39 0 up vs 1 -8.91959 79.5591 1.34867 3.59379 1cg22654055 ABLIM2 0.015445 0.257175 2.23E-16 0.0600564 -16.651 0 down vs 1 -8.91866 79.5424 1.05944 2.82368 1cg24708471 -0.256115 0.174659 2.24E-16 -1.46637 1.46637 0 up vs 1 -8.91793 79.5295 3.36447 8.96859 1cg08332162 0.044625 0.278435 2.25E-16 0.160271 -6.23944 0 down vs 1 -8.91741 79.5203 0.991161 2.64242 1cg23232299 GNG12 0.21426 0.372039 2.25E-16 0.575908 -1.73639 0 down vs 1 -8.91701 79.5131 0.451351 1.2034 1cg22108374 CCDC33 -0.21089 0.152922 2.26E-16 -1.37907 1.37907 0 up vs 1 -8.91694 79.5119 2.39978 6.39845 1cg22806837 ZNF773 -0.44313 -0.0479701 2.26E-16 9.23763 9.23763 0 up vs 1 -8.91674 79.5083 2.83116 7.54896 1cg14583675 ABCG5 -0.310745 0.140364 2.26E-16 -2.21384 2.21384 0 up vs 1 -8.91667 79.507 3.68962 9.83812 1cg23023970 INPP5A -0.308245 -0.414383 2.28E-16 0.743865 -1.34433 0 down vs 1 8.91557 79.4874 0.204249 0.54475 1cg21809447 OLFM3 -0.320365 0.0732046 2.30E-16 -4.37629 4.37629 0 up vs 1 -8.91379 79.4556 2.80841 7.49328 1cg18274480 -0.09456 0.191068 2.30E-16 -0.494904 -2.0206 0 down vs 1 -8.91379 79.4556 1.47917 3.94666 1cg01033356 ADARB2 0.118185 0.414075 2.31E-16 0.285419 -3.50362 0 down vs 1 -8.91342 79.4491 1.58737 4.23571 1cg07589235 CNTNAP3 0.086905 0.321382 2.31E-16 0.27041 -3.69808 0 down vs 1 -8.91314 79.444 0.996824 2.66007 1cg14308479 ASCL2 -0.22492 -0.452389 2.31E-16 0.497183 -2.01133 0 down vs 1 8.91313 79.4438 0.938126 2.50344 1cg08038054 GNG11 -0.1592 0.190306 2.32E-16 -0.836549 -1.19539 0 down vs 1 -8.91282 79.4383 2.21476 5.91061 1cg18658231 -0.024965 0.336351 2.34E-16 -0.0742232 -13.4729 0 down vs 1 -8.91145 79.4139 2.36696 6.31874 1cg26871350 F2RL2 0.06744 0.296959 2.35E-16 0.227102 -4.40331 0 down vs 1 -8.91094 79.4048 0.955116 2.55003 1cg03467555 0.026105 0.281761 2.37E-16 0.0926495 -10.7934 0 down vs 1 -8.90945 79.3783 1.18503 3.16492 1cg02974204 0.05206 0.245666 2.37E-16 0.211914 -4.7189 0 down vs 1 -8.9091 79.3721 0.679603 1.8152 1cg04038089 -0.248385 0.163329 2.38E-16 -1.52076 1.52076 0 up vs 1 -8.90898 79.3699 3.07334 8.209 1cg00546117 RERE 0.25023 0.396863 2.38E-16 0.630519 -1.58599 0 down vs 1 -8.9089 79.3684 0.389838 1.04129 1cg14874750 EMID2 -0.342465 0.0833464 2.38E-16 -4.10894 4.10894 0 up vs 1 -8.90844 79.3602 3.2874 8.78184 1cg09384610 RPH3A -0.19846 0.199038 2.39E-16 -0.997098 -1.00291 0 down vs 1 -8.90823 79.3566 2.86475 7.65315 1cg20100006 FAM20C 0.23027 0.449412 2.39E-16 0.512381 -1.95167 0 down vs 1 -8.90787 79.3502 0.8707 2.32625 1cg26862247 CLYBL 0.20019 0.410616 2.40E-16 0.487536 -2.05113 0 down vs 1 -8.90716 79.3375 0.802817 2.14523 1cg07272677 SORBS2 0.26072 0.4632 2.42E-16 0.562867 -1.77662 0 down vs 1 -8.90645 79.3248 0.743331 1.98659 1cg16370778 ARPP-21 -0.032345 0.309168 2.42E-16 -0.10462 -9.55843 0 down vs 1 -8.906 79.3168 2.11461 5.652 1cg27576971 0.17269 0.381849 2.43E-16 0.452246 -2.21118 0 down vs 1 -8.90554 79.3087 0.793182 2.12026 1cg18426863 0.084495 0.327241 2.45E-16 0.258204 -3.8729 0 down vs 1 -8.90402 79.2816 1.06837 2.85683 1cg15591426 0.011215 0.21731 2.49E-16 0.0516082 -19.3768 0 down vs 1 -8.90192 79.2441 0.770112 2.06026 1cg19374523 -0.12962 0.102249 2.50E-16 -1.26768 1.26768 0 up vs 1 -8.90146 79.2361 0.974777 2.60806 1cg01111713 0.19612 0.40806 2.51E-16 0.480616 -2.08066 0 down vs 1 -8.90091 79.2261 0.81441 2.17927 1cg21854408 -0.266625 -0.00831082 2.51E-16 32.0817 32.0817 0 up vs 1 -8.90032 79.2156 1.2098 3.23772 1cg19711800 ERI3 0.192785 0.360795 2.52E-16 0.534333 -1.87149 0 down vs 1 -8.90003 79.2106 0.511788 1.36975 1cg10494028 C6orf147 0.22946 0.409015 2.52E-16 0.561006 -1.78251 0 down vs 1 -8.90001 79.2102 0.584541 1.56448 1cg16128766 PPYR1 0.175595 0.393701 2.53E-16 0.446011 -2.2421 0 down vs 1 -8.89961 79.2031 0.862488 2.30859 1cg00968890 -0.23397 0.0975701 2.53E-16 -2.39797 2.39797 0 up vs 1 -8.89929 79.1974 1.99292 5.33476 1cg07762474 -0.031725 0.283875 2.53E-16 -0.111757 -8.948 0 down vs 1 -8.89917 79.1952 1.8059 4.83426 1cg01501309 CHD4 0.30021 0.387724 2.54E-16 0.774288 -1.29151 0 down vs 1 -8.89907 79.1935 0.138858 0.371721 1cg08912801 -0.07978 -0.327384 2.54E-16 0.243689 -4.10359 0 down vs 1 8.899 79.1921 1.11156 2.97569 1cg15156029 PRDM16 0.194805 0.399169 2.54E-16 0.488026 -2.04907 0 down vs 1 -8.89875 79.1878 0.757229 2.02724 1cg13893358 ABLIM2 0.072885 0.327026 2.55E-16 0.222872 -4.48688 0 down vs 1 -8.8981 79.1762 1.17103 3.13552 1cg22827833 0.196855 0.391945 2.56E-16 0.502251 -1.99104 0 down vs 1 -8.89743 79.1642 0.690064 1.84798 1cg08083757 0.17744 0.441069 2.57E-16 0.402296 -2.48573 0 down vs 1 -8.89705 79.1575 1.26009 3.37479 1cg25566568 -0.311185 0.20334 2.57E-16 -1.53037 1.53037 0 up vs 1 -8.89674 79.1519 4.79988 12.856 1cg08746033 ADAM29 0.02271 0.349546 2.58E-16 0.06497 -15.3917 0 down vs 1 -8.89659 79.1494 1.93677 5.18759 1cg01220192 0.179385 0.378774 2.59E-16 0.473594 -2.11151 0 down vs 1 -8.89579 79.135 0.720807 1.93102 1cg23766724 0.20583 0.392986 2.62E-16 0.52376 -1.90927 0 down vs 1 -8.89425 79.1076 0.635073 1.70193 1cg11159090 0.187865 0.373116 2.63E-16 0.503502 -1.98609 0 down vs 1 -8.89378 79.0993 0.622216 1.66765 1cg10892950 ANO6 -0.110845 -0.359789 2.63E-16 0.308083 -3.24588 0 down vs 1 8.89341 79.0927 1.12363 3.01177 1cg08401628 0.074375 0.432812 2.65E-16 0.171841 -5.81933 0 down vs 1 -8.89235 79.0739 2.3294 6.24522 1cg23074048 -0.37202 0.0741232 2.65E-16 -5.01894 5.01894 0 up vs 1 -8.89205 79.0686 3.60883 9.67605 1cg15992563 -0.28229 0.202522 2.67E-16 -1.39387 1.39387 0 up vs 1 -8.89501 79.1213 4.25946 11.3591 1cg14080048 -0.165455 0.204432 2.67E-16 -0.809338 -1.23558 0 down vs 1 -8.89102 79.0502 2.4806 6.65258 1cg10055817 ADARB2 0.17093 0.434774 2.68E-16 0.393147 -2.54358 0 down vs 1 -8.89087 79.0476 1.26215 3.385 1cg02360514 FOSB -0.18558 0.132202 2.74E-16 -1.40376 1.40376 0 up vs 1 -8.88737 78.9854 1.83095 4.91435 1cg13224427 0.05149 0.322003 2.80E-16 0.159905 -6.2537 0 down vs 1 -8.88368 78.9197 1.32677 3.56406 1cg17611475 AGAP2 0.21729 0.41851 2.81E-16 0.519199 -1.92604 0 down vs 1 -8.88318 78.9108 0.734107 1.97223 1cg08126551 PDZD4 0.17946 0.327326 2.83E-16 0.548261 -1.82395 0 down vs 1 -8.8825 78.8989 0.396418 1.06517 1cg23836168 ENGASE 0.160775 0.346785 2.83E-16 0.463616 -2.15696 0 down vs 1 -8.88234 78.896 0.627319 1.68566 1cg02703908 LHFPL4 -0.07451 0.180582 2.85E-16 -0.41261 -2.42359 0 down vs 1 -8.88149 78.8809 1.17981 3.17085 1cg18301583 ZNF415 -0.300975 0.104957 2.87E-16 -2.8676 2.8676 0 up vs 1 -8.88011 78.8564 2.98762 8.03201 1cg14778311 GPC6 -0.357525 0.0352773 2.88E-16 -10.1347 10.1347 0 up vs 1 -8.87984 78.8516 2.79747 7.52127 1cg18428688 ZSCAN18 -0.345565 0.111676 2.88E-16 -3.09435 3.09435 0 up vs 1 -8.87976 78.8502 3.79061 10.1916 1cg10824107 -0.04627 0.158619 2.89E-16 -0.291705 -3.42812 0 down vs 1 -8.87932 78.8424 0.761124 2.04659 1cg16494192 SLC18A3 -0.32637 -0.00551701 2.91E-16 59.157 59.157 0 up vs 1 -8.87808 78.8202 1.86651 5.02028 1cg19919383 PLCL2 0.1318 0.37457 2.92E-16 0.351871 -2.84195 0 down vs 1 -8.87763 78.8122 1.06858 2.87441 1cg14462265 0.030265 0.37701 2.92E-16 0.0802764 -12.457 0 down vs 1 -8.87749 78.8099 2.17991 5.86399 1cg03677126 0.199605 0.453289 2.92E-16 0.440348 -2.27093 0 down vs 1 -8.8774 78.8083 1.16682 3.13884 1cg08845028 CCKAR 0.01277 0.28508 2.93E-16 0.0447944 -22.3242 0 down vs 1 -8.87697 78.8005 1.34446 3.61704 1cg13447813 AGAP11 0.180895 0.363437 2.93E-16 0.497735 -2.0091 0 down vs 1 -8.87683 78.798 0.604146 1.62541 1cg24892966 -0.084635 0.261286 2.94E-16 -0.323917 -3.08721 0 down vs 1 -8.8764 78.7904 2.16956 5.8376 1cg24053375 RBP3 0.251605 0.390411 2.99E-16 0.644462 -1.55168 0 down vs 1 -8.87373 78.7431 0.349328 0.940495 1cg24577417 HGC6.3 0.17335 0.402382 3.00E-16 0.430809 -2.32121 0 down vs 1 -8.87353 78.7395 0.951069 2.56068 1cg23424003 EMID2 -0.201485 0.194989 3.02E-16 -1.03331 1.03331 0 up vs 1 -8.8722 78.7159 2.85002 7.67575 1cg05835726 HOXA5 -0.0863474 0.27094 3.04E-16 -0.318696 -3.13779 0 down vs 1 -8.87853 78.8283 2.20806 5.8823 1cg01550545 NFIA 0.2286 0.41848 3.05E-16 0.546262 -1.83062 0 down vs 1 -8.87086 78.6921 0.6537 1.7611 1cg14709049 0.005535 0.263966 3.06E-16 0.0209686 -47.6903 0 down vs 1 -8.87046 78.6851 1.2109 3.2625 1cg09891226 PRDM16 0.009175 0.380247 3.06E-16 0.024129 -41.4439 0 down vs 1 -8.87035 78.6832 2.49652 6.7265 1cg17373343 RFX2 0.243285 0.407492 3.08E-16 0.59703 -1.67496 0 down vs 1 -8.86956 78.6691 0.488877 1.31744 1cg04229851 PSAPL1 0.01306 0.26298 3.09E-16 0.0496615 -20.1363 0 down vs 1 -8.86914 78.6617 1.13246 3.05207 1cg09667775 CHD5 0.186555 0.436896 3.09E-16 0.427001 -2.34192 0 down vs 1 -8.86892 78.6577 1.13627 3.06251 1cg20573218 SOX2OT 0.20104 0.379726 3.11E-16 0.529434 -1.88881 0 down vs 1 -8.86791 78.6399 0.578896 1.56061 1cg00036347 LOC100132111 -0.06129 -0.34356 3.14E-16 0.178397 -5.60549 0 down vs 1 8.86636 78.6123 1.4446 3.89577 1cg08477641 TREX1 -0.325205 -0.465378 3.15E-16 0.698798 -1.43103 0 down vs 1 8.86617 78.609 0.356242 0.960748 1cg14432018 MAGEL2 -0.06855 0.218561 3.15E-16 -0.313642 -3.18835 0 down vs 1 -8.86575 78.6015 1.49458 4.0311 1cg25296804 RPS6KA2 0.230805 0.407375 3.16E-16 0.566567 -1.76502 0 down vs 1 -8.86569 78.6004 0.565263 1.52462 1cg17774672 -0.154695 0.106958 3.16E-16 -1.44631 1.44631 0 up vs 1 -8.86531 78.5937 1.24128 3.34825 1cg00997400 CDH4 0.24667 0.417013 3.19E-16 0.591516 -1.69057 0 down vs 1 -8.86407 78.5718 0.526097 1.4195 1cg17279125 PIK3R6 -0.048195 0.321511 3.19E-16 -0.149902 -6.67104 0 down vs 1 -8.86403 78.5711 2.47817 6.68657 1cg19705450 ZNF311 0.23931 0.403723 3.23E-16 0.592758 -1.68703 0 down vs 1 -8.86203 78.5356 0.490107 1.323 1cg08185576 BDKRB1 0.114995 0.366994 3.24E-16 0.313343 -3.19139 0 down vs 1 -8.86159 78.5279 1.15137 3.10834 1cg24647031 PTPRN2 -0.009835 0.328962 3.24E-16 -0.029897 -33.4481 0 down vs 1 -8.8615 78.5262 2.08112 5.61849 1cg12997538 CNTN4 -0.056395 0.198401 3.27E-16 -0.284248 -3.51806 0 down vs 1 -8.86035 78.5058 1.17707 3.17861 1cg09702591 GALNTL4 0.31169 0.41607 3.28E-16 0.749129 -1.33488 0 down vs 1 -8.8599 78.4978 0.197537 0.533491 1cg07038187 LOC254559 0.28152 -0.0850665 3.28E-16 -3.30941 3.30941 0 up vs 1 8.85962 78.4928 2.43653 6.58077 1cg15594205 POU3F3 -0.218155 0.194181 3.29E-16 -1.12346 1.12346 0 up vs 1 -8.85939 78.4889 3.08262 8.32623 1cg14208875 FBXL20 0.147335 0.362531 3.31E-16 0.406407 -2.46059 0 down vs 1 -8.85835 78.4703 0.839626 2.26838 1cg12287976 TRIM22 0.20139 0.383534 3.32E-16 0.525091 -1.90443 0 down vs 1 -8.85787 78.4619 0.601513 1.62526 1cg11089595 RALYL -0.12804 0.223786 3.33E-16 -0.572154 -1.74778 0 down vs 1 -8.85763 78.4577 2.24426 6.06421 1cg08206156 0.217365 0.411009 3.35E-16 0.528857 -1.89087 0 down vs 1 -8.85667 78.4406 0.679872 1.83748 1cg11521470 -0.12529 0.210872 3.35E-16 -0.594153 -1.68307 0 down vs 1 -8.8566 78.4393 2.04887 5.53753 1cg19164131 CAMTA1 0.06001 0.302187 3.35E-16 0.198586 -5.0356 0 down vs 1 -8.85646 78.4369 1.06336 2.87407 1cg06816106 SPDYA -0.17336 -0.440272 3.37E-16 0.393756 -2.53964 0 down vs 1 8.85569 78.4233 1.29168 3.49177 1cg26534812 -0.00259 0.306808 3.37E-16 -0.00844177 -118.459 0 down vs 1 -8.85555 78.4208 1.73561 4.69198 1cg00474004 IFNA14 0.151505 0.386455 3.38E-16 0.392038 -2.55077 0 down vs 1 -8.85525 78.4155 1.00085 2.70584 1cg09014354 NEUROD4 -0.34234 0.0157711 3.40E-16 -21.7067 21.7067 0 up vs 1 -8.85422 78.3972 2.32516 6.28766 1cg19084281 PCSK1 0.163385 0.384931 3.42E-16 0.424453 -2.35597 0 down vs 1 -8.85339 78.3826 0.889909 2.40692 1cg03433241 0.01326 0.238215 3.43E-16 0.055664 -17.9649 0 down vs 1 -8.8531 78.3774 0.917506 2.48173 1cg04383058 PITPNM1 -0.303925 -0.424428 3.44E-16 0.716082 -1.39649 0 down vs 1 8.85248 78.3664 0.263277 0.712227 1cg09616494 DTHD1 0.23042 0.415719 3.44E-16 0.554269 -1.80418 0 down vs 1 -8.85235 78.3641 0.622532 1.68415 1cg25661892 0.06069 0.263506 3.46E-16 0.230317 -4.34184 0 down vs 1 -8.85152 78.3494 0.745801 2.01801 1cg01101997 PDE10A 0.200515 0.390281 3.47E-16 0.513771 -1.94639 0 down vs 1 -8.85138 78.347 0.652912 1.76672 1cg05713245 SFMBT2 0.14332 0.397538 3.50E-16 0.360519 -2.77378 0 down vs 1 -8.84978 78.3186 1.17174 3.17177 1cg11721464 DEDD2 0.130525 -0.271704 3.55E-16 -0.480394 -2.08163 0 down vs 1 8.84772 78.2822 2.93336 7.94397 1cg07516445 0.209315 0.389686 3.57E-16 0.537138 -1.86172 0 down vs 1 -8.847 78.2694 0.58986 1.59769 1cg00376654 UMOD -0.16623 0.0917582 3.57E-16 -1.81161 1.81161 0 up vs 1 -8.84672 78.2644 1.20675 3.26881 1cg11858450 CCDC105 0.09766 0.347859 3.58E-16 0.280746 -3.56194 0 down vs 1 -8.84643 78.2592 1.13498 3.0746 1cg20541120 LOC100271832 0.32392 0.410603 3.63E-16 0.788888 -1.26761 0 down vs 1 -8.84434 78.2224 0.136234 0.369225 1cg22258892 C1QTNF1 -0.068265 0.246979 3.64E-16 -0.2764 -3.61795 0 down vs 1 -8.84399 78.2162 1.80183 4.88373 1cg12564187 KRT24 0.197235 0.396525 3.65E-16 0.497408 -2.01042 0 down vs 1 -8.8433 78.2039 0.720095 1.95208 1cg03190219 0.175535 0.402096 3.66E-16 0.43655 -2.29069 0 down vs 1 -8.84283 78.1956 0.930657 2.52315 1cg07752120 -0.096695 0.315106 3.77E-16 -0.306865 -3.25876 0 down vs 1 -8.83851 78.1192 3.07463 8.34394 1cg08133919 MYO5A -0.007805 0.276376 3.78E-16 -0.0282405 -35.4101 0 down vs 1 -8.83799 78.11 1.46422 3.97408 1cg09995854 IL1F8 0.359215 0.437046 3.80E-16 0.821915 -1.21667 0 down vs 1 -8.83747 78.1009 0.109832 0.298131 1cg16870351 ZIC3 -0.011145 0.269421 3.82E-16 -0.0413665 -24.1741 0 down vs 1 -8.83667 78.0867 1.42721 3.87477 1cg00344445 FLI1 -0.173695 0.220549 3.82E-16 -0.787558 -1.26975 0 down vs 1 -8.8364 78.0819 2.81805 7.65127 1cg07352917 -0.062275 0.249878 3.90E-16 -0.249222 -4.01249 0 down vs 1 -8.8332 78.0255 1.76666 4.80012 1cg10629020 ABR 0.34787 0.445607 3.91E-16 0.780665 -1.28096 0 down vs 1 -8.83311 78.0238 0.173196 0.470594 1cg11205072 -0.20242 0.220524 3.91E-16 -0.917904 -1.08944 0 down vs 1 -8.83293 78.0206 3.24328 8.81273 1cg07637243 NFATC1 0.18984 0.366768 3.91E-16 0.517602 -1.93199 0 down vs 1 -8.83277 78.0178 0.567559 1.54224 1cg04434871 CLMN 0.254505 0.384697 3.92E-16 0.661572 -1.51155 0 down vs 1 -8.83247 78.0125 0.307319 0.835143 1cg16783478 GRIN2A 0.09287 0.418541 3.93E-16 0.22189 -4.50674 0 down vs 1 -8.83198 78.0039 1.92298 5.22631 1cg22039566 EPHA8 0.046455 0.272976 3.95E-16 0.17018 -5.87614 0 down vs 1 -8.83141 77.9938 0.930328 2.52879 1cg01771462 FMN1 0.2108 0.379506 3.97E-16 0.555459 -1.80031 0 down vs 1 -8.83073 77.9818 0.516036 1.40289 1cg10728451 EBF3 -0.067165 0.221164 3.98E-16 -0.303689 -3.29284 0 down vs 1 -8.83031 77.9744 1.50728 4.09806 1cg18423210 UNC13A 0.06362 0.416021 4.00E-16 0.152925 -6.53915 0 down vs 1 -8.82934 77.9572 2.2516 6.12309 1cg26781166 0.310175 0.435454 4.05E-16 0.712302 -1.4039 0 down vs 1 -8.82763 77.9271 0.284561 0.774146 1cg04942516 FHL2 0.24866 0.403459 4.06E-16 0.616321 -1.62253 0 down vs 1 -8.82729 77.921 0.434463 1.18204 1cg15287895 -0.0741 0.286553 4.06E-16 -0.258591 -3.86711 0 down vs 1 -8.82723 77.9201 2.35829 6.41629 1cg22111038 RAB3C -0.295595 0.0211768 4.06E-16 -13.9584 13.9584 0 up vs 1 -8.82707 77.9171 1.81933 4.9501 1cg18075379 0.0324 0.294758 4.07E-16 0.109921 -9.09746 0 down vs 1 -8.8268 77.9123 1.24797 3.39575 1cg05865340 -0.29302 0.108977 4.07E-16 -2.68882 2.68882 0 up vs 1 -8.82673 77.9112 2.92998 7.9726 1cg09197075 SNN 0.13369 -0.152163 4.13E-16 -0.878595 -1.13818 0 down vs 1 8.82437 77.8694 1.48151 4.03342 1cg25283465 -0.219235 0.224257 4.14E-16 -0.977607 -1.02291 0 down vs 1 -8.82432 77.8686 3.56606 9.70874 1cg11232245 0.109005 0.366227 4.15E-16 0.297643 -3.35972 0 down vs 1 -8.82364 77.8566 1.19959 3.26643 1cg00285343 LOC285954 0.019115 0.316555 4.16E-16 0.0603844 -16.5606 0 down vs 1 -8.82351 77.8544 1.60405 4.36787 1cg24737783 C1orf59 -0.252805 -0.401853 4.17E-16 0.629099 -1.58958 0 down vs 1 8.823 77.8453 0.40278 1.09691 1cg26800525 ATP11A 0.10179 0.370191 4.20E-16 0.274966 -3.63681 0 down vs 1 -8.83988 78.1434 1.30291 3.45137 1cg20707409 CUZD1 0.11836 0.294407 4.21E-16 0.402028 -2.48739 0 down vs 1 -8.82156 77.82 0.561922 1.53081 1cg24993735 0.18285 0.411398 4.22E-16 0.44446 -2.24992 0 down vs 1 -8.82123 77.8141 0.947049 2.58018 1cg26405841 0.13208 0.320646 4.23E-16 0.411919 -2.42766 0 down vs 1 -8.82092 77.8086 0.64468 1.75652 1cg21053982 0.38839 0.438461 4.25E-16 0.885803 -1.12892 0 down vs 1 -8.82031 77.798 0.0454556 0.123867 1cg14961750 0.00845 0.328235 4.27E-16 0.0257437 -38.8444 0 down vs 1 -8.81935 77.781 1.8541 5.05355 1cg08862210 OR12D2 0.03224 0.33469 4.27E-16 0.0963279 -10.3812 0 down vs 1 -8.81932 77.7803 1.65854 4.52056 1cg16488737 OPCML -0.185475 0.205065 4.29E-16 -0.90447 -1.10562 0 down vs 1 -8.81882 77.7716 2.76534 7.53813 1cg12434681 HOXA3 0.092885 0.348849 4.31E-16 0.266261 -3.75571 0 down vs 1 -8.81792 77.7558 1.18789 3.23876 1cg04922803 GLT8D2 -0.144475 0.203911 4.31E-16 -0.708519 -1.4114 0 down vs 1 -8.81784 77.7544 2.2006 6 1cg13565994 C7orf34 0.220135 0.385326 4.32E-16 0.571296 -1.75041 0 down vs 1 -8.81781 77.7537 0.494754 1.34898 1cg25979053 0.24089 0.42726 4.32E-16 0.563802 -1.77367 0 down vs 1 -8.81761 77.7502 0.629751 1.71713 1cg06791091 TSPAN9 0.23816 0.417012 4.33E-16 0.57111 -1.75098 0 down vs 1 -8.81723 77.7435 0.579972 1.58154 1cg05824973 KRT6A 0.104095 0.313478 4.33E-16 0.332064 -3.01146 0 down vs 1 -8.81715 77.7422 0.794881 2.16761 1cg26654934 -0.27119 0.188978 4.34E-16 -1.43503 1.43503 0 up vs 1 -8.81681 77.7361 3.83929 10.4704 1cg05036212 -0.142325 0.264713 4.35E-16 -0.537657 -1.85992 0 down vs 1 -8.81651 77.7308 3.00392 8.19279 1cg07338549 0.260125 0.400257 4.39E-16 0.649895 -1.53871 0 down vs 1 -8.81512 77.7063 0.356033 0.971338 1cg12277789 0.019195 0.346957 4.41E-16 0.0553239 -18.0754 0 down vs 1 -8.81435 77.6927 1.94776 5.31484 1cg26968767 GRIN2C 0.333855 0.453871 4.43E-16 0.735572 -1.35949 0 down vs 1 -8.81371 77.6815 0.261154 0.712714 1cg11705975 PRLHR 0.037255 0.229894 4.44E-16 0.162053 -6.17082 0 down vs 1 -8.81341 77.6763 0.67283 1.83634 1cg18166947 BCL2L13 -0.301355 -0.452269 4.46E-16 0.666318 -1.50079 0 down vs 1 8.81261 77.6621 0.412931 1.12721 1cg21463709 MYT1L -0.34482 0.0147902 4.48E-16 -23.3141 23.3141 0 up vs 1 -8.81213 77.6536 2.34467 6.40112 1cg10356410 0.101725 0.368258 4.49E-16 0.276233 -3.62013 0 down vs 1 -8.81177 77.6473 1.28801 3.51665 1cg05427907 FAM83A 0.07911 0.308737 4.53E-16 0.256238 -3.90262 0 down vs 1 -8.81052 77.6253 0.95601 2.61093 1cg23318063 -0.266435 0.108091 4.55E-16 -2.46491 2.46491 0 up vs 1 -8.80979 77.6123 2.54321 6.94684 1cg18476557 -0.000425 0.240964 4.57E-16 -0.00176375 -566.974 0 down vs 1 -8.80915 77.6011 1.05646 2.88616 1cg12435551 SPAG17 -0.23012 -0.428498 4.57E-16 0.537039 -1.86206 0 down vs 1 8.80909 77.6 0.713518 1.9493 1cg13269522 PNCK 0.26026 0.417019 4.57E-16 0.624097 -1.60232 0 down vs 1 -8.80904 77.5991 0.445534 1.21719 1cg22648929 PXDN 0.19382 0.417222 4.61E-16 0.464549 -2.15263 0 down vs 1 -8.80765 77.5748 0.904884 2.47291 1cg20208979 0.18511 0.360801 4.62E-16 0.513053 -1.94912 0 down vs 1 -8.8073 77.5685 0.559651 1.52956 1cg07028533 CNTNAP2 -0.22386 0.133697 4.64E-16 -1.67438 1.67438 0 up vs 1 -8.80681 77.56 2.31797 6.33587 1cg23119063 0.25798 0.459224 4.64E-16 0.561774 -1.78007 0 down vs 1 -8.8067 77.558 0.734282 2.00711 1cg02474600 KNDC1 0.157885 0.349893 4.65E-16 0.451238 -2.21612 0 down vs 1 -8.80647 77.5539 0.66843 1.82721 1cg13591352 ABCG5 -0.385675 0.0731242 4.67E-16 -5.27424 5.27424 0 up vs 1 -8.80567 77.5397 3.81648 10.4346 1cg01599167 -0.038725 0.275194 4.68E-16 -0.140719 -7.10637 0 down vs 1 -8.80545 77.536 1.78671 4.88525 1cg05667379 DPP10 -0.159025 0.130662 4.70E-16 -1.21707 1.21707 0 up vs 1 -8.80464 77.5216 1.52152 4.16093 1cg11214047 SAMD9L 0.1283 0.380638 4.73E-16 0.337066 -2.96678 0 down vs 1 -8.80377 77.5064 1.15447 3.15778 1cg04933530 0.12601 -0.13636 4.75E-16 -0.924099 -1.08213 0 down vs 1 8.80322 77.4968 1.24809 3.41427 1cg06288154 RAB6C -0.374215 -0.113027 4.79E-16 3.31085 3.31085 0 up vs 1 -8.80198 77.4749 1.23687 3.38454 1cg19972788 NLRP1 0.1878 0.359617 4.84E-16 0.522222 -1.91489 0 down vs 1 -8.80015 77.4427 0.535242 1.46523 1cg08037727 C3orf21 0.19616 0.34653 4.84E-16 0.566069 -1.76657 0 down vs 1 -8.80013 77.4423 0.409961 1.12228 1cg01474424 SGCD -0.212325 0.103546 4.85E-16 -2.05054 2.05054 0 up vs 1 -8.79979 77.4364 1.80899 4.95254 1cg01096886 0.07508 0.340701 4.86E-16 0.220369 -4.53784 0 down vs 1 -8.79954 77.432 1.27921 3.50234 1cg22872857 XPO7 0.215915 0.372191 4.88E-16 0.580118 -1.72379 0 down vs 1 -8.799 77.4224 0.442796 1.21248 1cg09065113 -0.0261 0.313326 4.93E-16 -0.0832997 -12.0048 0 down vs 1 -8.79753 77.3965 2.08886 5.72168 1cg26127479 0.050175 0.299651 4.94E-16 0.167445 -5.97212 0 down vs 1 -8.79709 77.3888 1.12843 3.09125 1cg03707169 FLI1 0.026805 0.336881 4.95E-16 0.0795682 -12.5678 0 down vs 1 -8.79693 77.3859 1.74323 4.7756 1cg17295957 0.094335 0.414625 4.97E-16 0.227519 -4.39524 0 down vs 1 -8.79608 77.371 1.85997 5.09639 1cg04996219 CTNND2 -0.193355 0.216645 4.98E-16 -0.892498 -1.12045 0 down vs 1 -8.796 77.3696 3.04779 8.35124 1cg23500122 GHSR -0.33313 0.0758814 4.98E-16 -4.39014 4.39014 0 up vs 1 -8.79594 77.3685 3.03311 8.31114 1cg23509641 XKR7 -0.348125 -0.025132 4.98E-16 13.8519 13.8519 0 up vs 1 -8.79577 77.3656 1.89149 5.18313 1cg03731303 SOX2OT -0.10702 0.236662 5.00E-16 -0.452206 -2.21138 0 down vs 1 -8.79519 77.3554 2.14156 5.86917 1cg07631359 IRAK2 0.18012 0.380661 5.01E-16 0.473177 -2.11337 0 down vs 1 -8.79489 77.35 0.729161 1.99848 1cg19365166 KRT86 0.33428 0.422831 5.04E-16 0.790575 -1.2649 0 down vs 1 -8.79395 77.3336 0.14217 0.389742 1cg11948055 ZNF737 -0.298235 0.138619 5.05E-16 -2.15148 2.15148 0 up vs 1 -8.79375 77.33 3.46011 9.48588 1cg00779313 WDR37 0.228795 0.415718 5.06E-16 0.550361 -1.81699 0 down vs 1 -8.79351 77.3258 0.633496 1.73682 1cg16329658 -0.2082 0.203615 5.06E-16 -1.02252 1.02252 0 up vs 1 -8.79348 77.3253 3.07485 8.43019 1cg04612055 -0.03617 0.272774 5.10E-16 -0.132601 -7.54145 0 down vs 1 -8.79231 77.3048 1.73053 4.74578 1cg18335796 CALB1 0.10191 0.305367 5.15E-16 0.33373 -2.99644 0 down vs 1 -8.7908 77.2781 0.750522 2.05893 1cg13425960 ZNF473 0.12328 0.30267 5.15E-16 0.407309 -2.45514 0 down vs 1 -8.79078 77.2777 0.583462 1.60064 1cg05440785 0.092355 0.364693 5.17E-16 0.253241 -3.94881 0 down vs 1 -8.79019 77.2674 1.34473 3.68955 1cg22991404 IL1F10 0.25588 0.414697 5.20E-16 0.617028 -1.62067 0 down vs 1 -8.7893 77.2518 0.457314 1.25499 1cg15910502 KAT2B -0.215585 -0.393329 5.23E-16 0.548103 -1.82447 0 down vs 1 8.78851 77.2379 0.572809 1.57223 1cg12853742 LTB4R 0.18231 -0.164174 5.23E-16 -1.11047 1.11047 0 up vs 1 8.78843 77.2365 2.17663 5.97446 1cg08372350 CBFA2T3 0.022 0.315432 5.24E-16 0.0697455 -14.3378 0 down vs 1 -8.78795 77.228 1.56111 4.28544 1cg14190975 OLFM1 0.185885 0.42686 5.26E-16 0.43547 -2.29637 0 down vs 1 -8.78747 77.2197 1.05284 2.89049 1cg20859731 -0.26746 0.14017 5.32E-16 -1.90811 1.90811 0 up vs 1 -8.78562 77.1871 3.01266 8.27449 1cg05000994 -0.0489 0.261796 5.33E-16 -0.186787 -5.3537 0 down vs 1 -8.78557 77.1862 1.75021 4.80712 1cg14072140 DPYD 0.11846 0.366849 5.35E-16 0.322912 -3.09682 0 down vs 1 -8.78502 77.1765 1.11862 3.07279 1cg18093771 ADARB2 -0.081145 0.321942 5.37E-16 -0.252049 -3.96749 0 down vs 1 -8.78789 77.2271 2.94446 8.04485 1cg04228742 0.1701 0.400027 5.39E-16 0.425221 -2.35172 0 down vs 1 -8.78376 77.1545 0.958515 2.63374 1cg23378033 MSRB3 -0.05868 0.27938 5.40E-16 -0.210036 -4.76108 0 down vs 1 -8.78343 77.1487 2.07208 5.69396 1cg16768376 0.16497 0.389161 5.42E-16 0.423912 -2.35898 0 down vs 1 -8.78299 77.1409 0.911288 2.50442 1cg18470427 SLFN12L -0.003805 0.33777 5.44E-16 -0.0112651 -88.7699 0 down vs 1 -8.78238 77.1302 2.11538 5.81434 1cg10004780 ARHGEF4 -0.147905 0.143856 5.46E-16 -1.02814 1.02814 0 up vs 1 -8.78168 77.1178 1.54338 4.24281 1cg11838639 0.17426 0.402697 5.50E-16 0.432732 -2.3109 0 down vs 1 -8.78058 77.0986 0.946134 2.60161 1cg08992872 CALB1 -0.320735 0.0655412 5.53E-16 -4.89364 4.89364 0 up vs 1 -8.77971 77.0834 2.70529 7.44028 1cg16196968 -0.05087 0.260823 5.55E-16 -0.195037 -5.12724 0 down vs 1 -8.77933 77.0766 1.76145 4.8449 1cg11270656 GPIHBP1 0.11668 0.401628 5.59E-16 0.290517 -3.44214 0 down vs 1 -8.77808 77.0546 1.47214 4.0503 1cg09295050 ITGA7 0.222095 0.410062 5.61E-16 0.541613 -1.84634 0 down vs 1 -8.77775 77.0489 0.640594 1.76259 1cg24778248 CNGA3 -0.269025 0.095549 5.62E-16 -2.81557 2.81557 0 up vs 1 -8.77741 77.0428 2.40985 6.63121 1cg23615741 -0.005305 0.333637 5.67E-16 -0.0159005 -62.8911 0 down vs 1 -8.77607 77.0194 2.0829 5.7333 1cg05854261 -0.045835 0.245295 5.68E-16 -0.186857 -5.35169 0 down vs 1 -8.77572 77.0133 1.53671 4.2302 1cg15688396 LSAMP -0.28124 0.0190613 5.68E-16 -14.7545 14.7545 0 up vs 1 -8.7757 77.013 1.63506 4.50095 1cg02477042 LASS3 0.25479 0.424678 5.73E-16 0.599961 -1.66678 0 down vs 1 -8.77445 76.9911 0.52329 1.44091 1cg05710512 0.21415 0.376793 5.73E-16 0.56835 -1.75948 0 down vs 1 -8.77426 76.9877 0.479609 1.32069 1cg08158662 -0.079535 0.158261 5.79E-16 -0.502555 -1.98983 0 down vs 1 -8.77278 76.9617 1.02525 2.82416 1cg00422488 ANP32B -0.15139 -0.385928 5.81E-16 0.392275 -2.54923 0 down vs 1 8.77233 76.9539 0.997346 2.74759 1cg23264016 C10orf11 -0.076265 0.239248 5.82E-16 -0.31877 -3.13706 0 down vs 1 -8.7721 76.9497 1.8049 4.97258 1cg18790143 OTOS -0.03948 0.275942 5.85E-16 -0.143074 -6.98941 0 down vs 1 -8.77131 76.9358 1.80385 4.9706 1cg10821610 PAX7 -0.03343 0.345851 5.87E-16 -0.09666 -10.3455 0 down vs 1 -8.7742 76.9865 2.60694 7.14493 1cg00296182 -0.26221 0.0558943 5.87E-16 -4.69117 4.69117 0 up vs 1 -8.77067 76.9247 1.83467 5.05623 1cg27643910 TNXB 0.089335 0.332442 5.88E-16 0.268724 -3.72129 0 down vs 1 -8.77053 76.9222 1.07155 2.95322 1cg06347739 ASCL2 -0.23373 -0.469342 5.88E-16 0.497995 -2.00805 0 down vs 1 8.77045 76.9209 1.0065 2.77398 1cg26608883 CALCB -0.270275 0.122891 5.91E-16 -2.1993 2.1993 0 up vs 1 -8.76966 76.9069 2.80266 7.72576 1cg07745707 ZNF469 0.19091 0.426803 5.99E-16 0.447303 -2.23562 0 down vs 1 -8.76762 76.8712 1.0089 2.7824 1cg27111250 PRDM8 0.028625 0.289115 6.12E-16 0.0990089 -10.1001 0 down vs 1 -8.76433 76.8134 1.23027 3.39547 1cg17485681 CDH23 -0.06936 0.299138 6.23E-16 -0.231866 -4.31283 0 down vs 1 -8.76165 76.7665 2.462 6.79912 1cg06962787 CDRT1 0.25404 0.389611 6.23E-16 0.652035 -1.53366 0 down vs 1 -8.76147 76.7633 0.333235 0.920307 1cg07387286 ADAM5P 0.0213 0.335997 6.24E-16 0.0633933 -15.7745 0 down vs 1 -8.76121 76.7587 1.79558 4.95921 1cg12937186 0.004675 0.371097 6.26E-16 0.0125978 -79.379 0 down vs 1 -8.76082 76.7519 2.43434 6.724 1cg19659741 ADAM5P 0.035215 0.338757 6.34E-16 0.103954 -9.61969 0 down vs 1 -8.75893 76.7189 1.67054 4.61625 1cg03066788 PNOC -0.09324 0.185672 6.34E-16 -0.502177 -1.99133 0 down vs 1 -8.75874 76.7156 1.41043 3.89766 1cg10604373 -0.09828 0.314641 6.35E-16 -0.312356 -3.20148 0 down vs 1 -8.75865 76.714 3.09138 8.54306 1cg25572565 0.023035 0.277276 6.40E-16 0.083076 -12.0372 0 down vs 1 -8.75747 76.6933 1.17195 3.23958 1cg13654588 PRLHR -0.108545 0.160031 6.41E-16 -0.678273 -1.47433 0 down vs 1 -8.75727 76.6897 1.30784 3.61537 1cg01528913 0.03314 0.328346 6.41E-16 0.10093 -9.90784 0 down vs 1 -8.75715 76.6878 1.58004 4.36795 1cg04606861 GALNT9 -0.24353 0.070867 6.44E-16 -3.43644 3.43644 0 up vs 1 -8.75645 76.6754 1.79215 4.95512 1cg01336629 0.1015 0.354998 6.48E-16 0.285917 -3.49752 0 down vs 1 -8.75559 76.6603 1.16511 3.22206 1cg26267852 PLCH2 0.07165 0.338016 6.49E-16 0.211972 -4.7176 0 down vs 1 -8.75525 76.6545 1.2864 3.55774 1cg18801599 TMEM101 -0.120255 -0.438981 6.51E-16 0.273941 -3.65042 0 down vs 1 8.75471 76.645 1.84185 5.09455 1cg05225593 0.051735 0.315252 6.55E-16 0.164107 -6.09358 0 down vs 1 -8.75389 76.6307 1.25902 3.48311 1cg22896684 0.14444 0.391514 6.55E-16 0.368926 -2.71057 0 down vs 1 -8.75381 76.6293 1.10681 3.06207 1cg07060551 SHANK1 -0.345825 0.0405412 6.55E-16 -8.5302 8.5302 0 up vs 1 -8.7538 76.6291 2.70655 7.48788 1cg12618270 MFHAS1 0.06803 0.332052 6.55E-16 0.204878 -4.88097 0 down vs 1 -8.75375 76.6282 1.26386 3.4966 1cg08962798 CBY3 0.045225 -0.158531 6.56E-16 -0.285275 -3.50539 0 down vs 1 8.75355 76.6247 0.752732 2.08261 1cg14412023 PTPRZ1 0.316575 0.440102 6.58E-16 0.719323 -1.3902 0 down vs 1 -8.75321 76.6188 0.276655 0.76549 1cg06617335 LOC91450 -0.02282 -0.23737 6.61E-16 0.096137 -10.4018 0 down vs 1 8.75255 76.6071 0.83459 2.30962 1cg24740218 -0.3956 -0.131847 6.61E-16 3.00045 3.00045 0 up vs 1 -8.75253 76.6068 1.26128 3.49045 1cg09555914 ZNF773 -0.41923 0.00999021 6.64E-16 -41.9641 41.9641 0 up vs 1 -8.75171 76.5924 3.34024 9.24546 1cg06795634 KNDC1 0.10267 0.351626 6.67E-16 0.291987 -3.42482 0 down vs 1 -8.75098 76.5796 1.12373 3.11089 1cg14495241 0.186085 0.347678 6.71E-16 0.535222 -1.86838 0 down vs 1 -8.75013 76.5648 0.473437 1.3109 1cg14901751 0.205965 0.387187 6.72E-16 0.531952 -1.87987 0 down vs 1 -8.74987 76.5602 0.595443 1.64882 1cg15753394 RBM24 -0.16057 0.247726 6.73E-16 -0.648176 -1.54279 0 down vs 1 -8.74974 76.5579 3.02251 8.36977 1cg26781150 HPDL -0.29194 -0.433379 6.74E-16 0.673636 -1.48448 0 down vs 1 8.74955 76.5547 0.362709 1.00444 1cg04860194 ZCCHC13 0.035885 0.344115 6.75E-16 0.104282 -9.58938 0 down vs 1 -8.7493 76.5502 1.72253 4.77042 1cg00910893 LOC91450 -0.27462 -0.386578 6.77E-16 0.710386 -1.40768 0 down vs 1 8.74866 76.5391 0.227264 0.629482 1cg25447144 MEIS2 0.09239 0.320346 6.78E-16 0.288407 -3.46733 0 down vs 1 -8.74857 76.5374 0.942153 2.60966 1cg25343618 -0.18209 -0.385236 6.78E-16 0.472672 -2.11563 0 down vs 1 8.7485 76.5363 0.748226 2.07253 1cg13763345 TTC7B 0.008475 0.338276 6.82E-16 0.0250535 -39.9145 0 down vs 1 -8.74766 76.5216 1.97207 5.46353 1cg24365121 TNNI2 0.149315 0.366304 6.85E-16 0.407626 -2.45323 0 down vs 1 -8.74691 76.5085 0.853674 2.36547 1cg12309703 DIRC3 0.032565 0.31166 6.87E-16 0.104489 -9.5704 0 down vs 1 -8.74996 76.5619 1.4116 3.89029 1cg22288012 0.258085 0.402188 6.90E-16 0.641703 -1.55835 0 down vs 1 -8.74589 76.4907 0.376497 1.04349 1cg02174588 ZNF704 0.36535 0.437157 6.91E-16 0.835742 -1.19654 0 down vs 1 -8.74559 76.4853 0.0934863 0.259123 1cg09697697 0.24677 0.424397 6.94E-16 0.58146 -1.71981 0 down vs 1 -8.74496 76.4743 0.572052 1.58583 1cg15832905 HCCA2 0.15672 0.373369 6.96E-16 0.419746 -2.38239 0 down vs 1 -8.74459 76.4678 0.851 2.35932 1cg23316360 EDNRB -0.27861 0.0169144 6.96E-16 -16.4717 16.4717 0 up vs 1 -8.74453 76.4669 1.58345 4.39003 1cg07330196 LCE1A -0.0391 0.278633 6.97E-16 -0.140328 -7.12617 0 down vs 1 -8.7478 76.5241 1.8295 5.04449 1cg21526205 ST6GALNAC3 -0.39434 0.0597923 7.01E-16 -6.59517 6.59517 0 up vs 1 -8.74346 76.448 3.73923 10.3694 1cg06458239 ZNF549 -0.331055 0.14808 7.03E-16 -2.23564 2.23564 0 up vs 1 -8.74306 76.441 4.16231 11.5437 1cg22243109 0.16334 0.391975 7.04E-16 0.41671 -2.39975 0 down vs 1 -8.74272 76.4351 0.947773 2.62874 1cg18948899 PTPRN2 0.10329 0.334964 7.08E-16 0.308361 -3.24295 0 down vs 1 -8.74178 76.4187 0.973137 2.69967 1cg24159447 0.13189 0.348693 7.09E-16 0.378241 -2.64382 0 down vs 1 -8.74161 76.4158 0.852212 2.36429 1cg05971894 -0.28755 0.0190098 7.12E-16 -15.1264 15.1264 0 up vs 1 -8.74107 76.4063 1.70392 4.72776 1cg26190686 0.03186 0.356831 7.12E-16 0.0892859 -11.2 0 down vs 1 -8.74101 76.4053 1.91473 5.31277 1cg25397945 ZNF529 -0.32621 0.0580062 7.13E-16 -5.62371 5.62371 0 up vs 1 -8.74081 76.4017 2.67651 7.42681 1cg23167351 KIAA1949 -0.167315 -0.389322 7.21E-16 0.42976 -2.32688 0 down vs 1 8.73909 76.3716 0.893618 2.48059 1cg07576142 GPC6 -0.221045 0.201924 7.28E-16 -1.0947 1.0947 0 up vs 1 -8.73768 76.347 3.24365 9.00696 1cg06984176 JARID2 0.271605 0.393994 7.30E-16 0.689364 -1.45061 0 down vs 1 -8.73714 76.3376 0.271582 0.754222 1cg21648009 HRNBP3 0.025005 0.337362 7.40E-16 0.0741191 -13.4918 0 down vs 1 -8.73516 76.303 1.76897 4.91491 1cg10614021 GRIN2B -0.250375 0.165569 7.44E-16 -1.51221 1.51221 0 up vs 1 -8.7343 76.288 3.1368 8.71699 1cg11683663 ELANE 0.14545 0.433716 7.44E-16 0.335358 -2.98189 0 down vs 1 -8.73429 76.2879 1.50662 4.18683 1cg08471173 0.128245 0.358215 7.45E-16 0.358011 -2.79321 0 down vs 1 -8.73399 76.2826 0.958871 2.66483 1cg04059773 -0.19823 0.168914 7.47E-16 -1.17355 1.17355 0 up vs 1 -8.73369 76.2774 2.44395 6.79254 1cg24926689 0.19537 0.356743 7.54E-16 0.547649 -1.82599 0 down vs 1 -8.73213 76.25 0.472151 1.31274 1cg03715846 -0.009255 0.22313 7.56E-16 -0.041478 -24.1092 0 down vs 1 -8.73188 76.2458 0.979119 2.72242 1cg26756625 LHX2 -0.2572 0.00275979 7.57E-16 -93.1954 93.1954 0 up vs 1 -8.73152 76.2395 1.22527 3.40711 1cg21962562 -0.075195 0.212911 7.75E-16 -0.353176 -2.83145 0 down vs 1 -8.72801 76.1781 1.50495 4.1882 1cg10872967 FAM20C 0.25339 0.41548 7.82E-16 0.609872 -1.63969 0 down vs 1 -8.72656 76.1528 0.476357 1.32612 1cg07150045 KCNH7 -0.136445 0.14819 7.85E-16 -0.920742 -1.08608 0 down vs 1 -8.72594 76.1421 1.46891 4.08984 1cg08759309 0.07169 0.343974 7.94E-16 0.208417 -4.79808 0 down vs 1 -8.72433 76.1139 1.3442 3.74399 1cg09075743 MAD1L1 0.319715 0.416299 7.98E-16 0.767994 -1.30209 0 down vs 1 -8.72343 76.0982 0.169133 0.471183 1cg03365267 0.024965 0.315051 8.01E-16 0.0792411 -12.6197 0 down vs 1 -8.72295 76.0898 1.52571 4.2509 1cg16593628 0.105215 0.375948 8.01E-16 0.279865 -3.57315 0 down vs 1 -8.72282 76.0876 1.32893 3.70274 1cg22673070 RPTOR 0.18606 0.395143 8.03E-16 0.470867 -2.12374 0 down vs 1 -8.72261 76.0839 0.792605 2.20851 1cg09356083 PLCH2 0.086135 0.281343 8.07E-16 0.306157 -3.2663 0 down vs 1 -8.72182 76.0701 0.690895 1.92546 1cg02765085 0.22811 0.453859 8.15E-16 0.502601 -1.98965 0 down vs 1 -8.7202 76.0418 0.923993 2.57604 1cg21252355 0.144015 0.402691 8.18E-16 0.357631 -2.79618 0 down vs 1 -8.71975 76.034 1.2132 3.38267 1cg19060970 ZNF549 -0.297115 0.217481 8.20E-16 -1.36616 1.36616 0 up vs 1 -8.72271 76.0857 4.79889 13.3082 1cg10437931 -0.1509 0.251227 8.27E-16 -0.600651 -1.66486 0 down vs 1 -8.71806 76.0046 2.93187 8.17789 1cg14103680 -0.28168 0.187882 8.27E-16 -1.49924 1.49924 0 up vs 1 -8.71792 76.0022 3.99765 11.151 1cg09560658 TBCK 0.3007 0.413262 8.30E-16 0.727625 -1.37433 0 down vs 1 -8.71738 75.9928 0.229723 0.640867 1cg01275887 FOXK1 0.27977 0.456737 8.31E-16 0.612541 -1.63254 0 down vs 1 -8.71734 75.9919 0.56781 1.58406 1cg04142555 MAGEL2 0.038225 0.341032 8.31E-16 0.112086 -8.92171 0 down vs 1 -8.71725 75.9904 1.66246 4.63797 1cg03476864 PTPRN2 0.01331 0.315829 8.43E-16 0.0421431 -23.7287 0 down vs 1 -8.7151 75.953 1.65929 4.63142 1cg08475096 GRIA2 -0.28818 0.0695253 8.45E-16 -4.14497 4.14497 0 up vs 1 -8.7147 75.946 2.3199 6.47589 1cg22886649 0.37951 0.479173 8.47E-16 0.79201 -1.26261 0 down vs 1 -8.71426 75.9384 0.180089 0.502761 1cg06188870 ADAM8 0.25327 0.455523 8.52E-16 0.555998 -1.79857 0 down vs 1 -8.71345 75.9243 0.741667 2.07092 1cg04503340 AP3B1 0.17421 0.385384 8.54E-16 0.452043 -2.21218 0 down vs 1 -8.71302 75.9168 0.808535 2.25786 1cg06680065 LGR5 0.15536 0.372539 8.58E-16 0.417031 -2.39791 0 down vs 1 -8.7123 75.9041 0.85517 2.38849 1cg04413904 0.111865 0.422045 8.58E-16 0.265055 -3.77281 0 down vs 1 -8.71228 75.9039 1.7444 4.87211 1cg00735667 -0.27803 0.062699 8.63E-16 -4.43436 4.43436 0 up vs 1 -8.71149 75.8901 2.10492 5.88013 1cg24011501 MGAT5B 0.298845 0.457436 8.67E-16 0.653305 -1.53068 0 down vs 1 -8.71065 75.8755 0.456008 1.27411 1cg25650256 STK10 -0.12446 -0.379837 8.69E-16 0.327667 -3.05188 0 down vs 1 8.71031 75.8695 1.18244 3.30406 1cg14726637 CR1 -0.21983 0.106813 8.70E-16 -2.05808 2.05808 0 up vs 1 -8.71021 75.8678 1.93449 5.4056 1cg07868561 SH3TC2 0.15629 0.369848 8.77E-16 0.422579 -2.36642 0 down vs 1 -8.70897 75.8462 0.826893 2.31127 1cg03221366 -0.0074 0.395997 8.86E-16 -0.018687 -53.5132 0 down vs 1 -8.70741 75.8189 2.95042 8.24978 1cg08775211 SORCS2 0.1359 0.382932 8.90E-16 0.354893 -2.81775 0 down vs 1 -8.70677 75.8078 1.10643 3.09419 1cg01588826 KIRREL2 -0.04877 0.220014 8.96E-16 -0.221667 -4.51127 0 down vs 1 -8.70569 75.789 1.30986 3.664 1cg27033231 0.062525 0.262814 8.96E-16 0.237906 -4.20334 0 down vs 1 -8.70566 75.7886 0.72733 2.03453 1cg21312551 0.04118 0.318689 8.96E-16 0.129217 -7.73893 0 down vs 1 -8.70559 75.7873 1.39628 3.90582 1cg26889826 PCYT1B -0.020935 0.285209 9.00E-16 -0.0734022 -13.6236 0 down vs 1 -8.70495 75.7762 1.6993 4.75416 1cg07101782 MLH1 -0.321105 -0.489523 9.01E-16 0.655955 -1.52449 0 down vs 1 8.70481 75.7737 0.514273 1.43883 1cg08217447 KLHL5 0.273995 0.418494 9.18E-16 0.654717 -1.52738 0 down vs 1 -8.70199 75.7247 0.37857 1.05985 1cg04637961 ZCCHC14 0.233175 0.411326 9.20E-16 0.566886 -1.76402 0 down vs 1 -8.70165 75.7187 0.575431 1.61111 1cg00193521 -0.30557 -0.406465 9.25E-16 0.751774 -1.33019 0 down vs 1 8.70075 75.7031 0.18457 0.516873 1cg12647497 GSTM2 0.02899 0.30807 9.26E-16 0.0941021 -10.6268 0 down vs 1 -8.70067 75.7016 1.41213 3.95462 1cg17527766 CRYBG3 0.128815 0.361804 9.36E-16 0.356036 -2.80871 0 down vs 1 -8.69889 75.6708 0.984209 2.75737 1cg13525071 0.171855 0.427178 9.40E-16 0.402303 -2.48569 0 down vs 1 -8.69829 75.6602 1.18195 3.31183 1cg11684930 KNDC1 -0.01865 0.262061 9.42E-16 -0.0711665 -14.0515 0 down vs 1 -8.69789 75.6532 1.42869 4.00356 1cg08297616 0.05831 0.387332 9.43E-16 0.150543 -6.64264 0 down vs 1 -8.69772 75.6503 1.96277 5.5004 1cg13183298 -0.045135 0.204291 9.45E-16 -0.220935 -4.52622 0 down vs 1 -8.6975 75.6465 1.12798 3.16117 1cg06956784 -0.02836 0.238558 9.51E-16 -0.118881 -8.41177 0 down vs 1 -8.69649 75.629 1.29173 3.62093 1cg14498592 KCNQ2 -0.162845 0.27234 9.52E-16 -0.597948 -1.67239 0 down vs 1 -8.70657 75.8043 3.42869 9.45324 1cg15578601 0.26245 0.423421 9.53E-16 0.619832 -1.61334 0 down vs 1 -8.6962 75.6238 0.469801 1.31702 1cg14328535 MYT1L 0.07555 0.357583 9.57E-16 0.21128 -4.73306 0 down vs 1 -8.69544 75.6107 1.44217 4.04362 1cg04402371 PCYT1B 0.07113 0.322867 9.71E-16 0.220307 -4.53912 0 down vs 1 -8.69664 75.6316 1.14843 3.20392 1cg07108754 0.08657 0.34726 9.77E-16 0.249294 -4.01132 0 down vs 1 -8.69229 75.5559 1.23216 3.45729 1cg01405761 CLVS1 -0.390535 -0.0672809 9.90E-16 5.80454 5.80454 0 up vs 1 -8.69033 75.5218 1.89455 5.31826 1cg25208017 FMN2 -0.221125 0.177558 9.92E-16 -1.24537 1.24537 0 up vs 1 -8.69 75.516 2.88187 8.09041 1cg00737970 0.070375 0.359642 9.96E-16 0.195681 -5.11037 0 down vs 1 -8.68933 75.5045 1.51711 4.25971 1cg08695357 RGMA 0.03132 0.322009 1.00E-15 0.0972644 -10.2813 0 down vs 1 -8.6883 75.4866 1.53206 4.30269 1cg02840535 GTF3C1 0.282965 0.464623 1.00E-15 0.60902 -1.64198 0 down vs 1 -8.68814 75.4837 0.598312 1.68039 1cg11172629 CUGBP2 0.081995 0.375193 1.01E-15 0.218541 -4.57581 0 down vs 1 -8.68735 75.47 1.55862 4.37827 1cg04872123 TNXB 0.004865 0.269415 1.01E-15 0.0180576 -55.3783 0 down vs 1 -8.68701 75.4641 1.26892 3.56476 1cg17620251 NHSL2 -0.206645 -0.0172485 1.01E-15 11.9805 11.9805 0 up vs 1 -8.68684 75.4612 0.650372 1.82715 1cg01497613 -0.230835 0.0752036 1.01E-15 -3.06947 3.06947 0 up vs 1 -8.68663 75.4575 1.69813 4.77094 1cg22166633 MIR520E 0.00249 0.345094 1.02E-15 0.00721543 -138.592 0 down vs 1 -8.68533 75.435 2.12815 5.98088 1cg13433278 0.093825 0.322046 1.03E-15 0.29134 -3.43242 0 down vs 1 -8.68453 75.4211 0.944345 2.65445 1cg07085827 LOC150786 -0.379975 -0.0606732 1.03E-15 6.26265 6.26265 0 up vs 1 -8.68361 75.405 1.84851 5.19704 1cg13270055 CACNG2 -0.0391 0.273799 1.05E-15 -0.142806 -7.00253 0 down vs 1 -8.68135 75.3659 1.77511 4.9933 1cg00407546 FLJ45983 -0.17938 0.258964 1.05E-15 -0.692684 -1.44366 0 down vs 1 -8.68804 75.482 3.48036 9.68278 1cg22539450 EMX1 -0.309545 0.0454552 1.05E-15 -6.8099 6.8099 0 up vs 1 -8.68114 75.3623 2.28494 6.42772 1cg21349849 0.22996 0.383633 1.05E-15 0.599427 -1.66826 0 down vs 1 -8.68094 75.3587 0.428167 1.20452 1cg06879302 BMP2 0.235005 0.409499 1.07E-15 0.573884 -1.74251 0 down vs 1 -8.67848 75.316 0.552054 1.55392 1cg09404643 AIM1 -0.377775 -0.466175 1.07E-15 0.810372 -1.234 0 down vs 1 8.67836 75.3139 0.141684 0.398823 1cg11183632 -0.25758 0.0189577 1.07E-15 -13.5871 13.5871 0 up vs 1 -8.67769 75.3022 1.38652 3.90351 1cg07830160 -0.066255 -0.369462 1.08E-15 0.179328 -5.57637 0 down vs 1 8.67661 75.2836 1.66685 4.69389 1cg08577953 0.173935 0.44908 1.08E-15 0.387314 -2.58189 0 down vs 1 -8.67631 75.2783 1.3726 3.86553 1cg14949892 CHFR 0.231155 0.396038 1.08E-15 0.583669 -1.7133 0 down vs 1 -8.67618 75.2761 0.49291 1.38818 1cg16390060 NKX2-2 -0.219875 0.103121 1.09E-15 -2.1322 2.1322 0 up vs 1 -8.67577 75.269 1.89153 5.32761 1cg15129097 SFMBT2 0.174465 0.402494 1.10E-15 0.43346 -2.30702 0 down vs 1 -8.67446 75.2463 0.942752 2.65612 1cg14458578 HRNBP3 0.366305 0.456543 1.11E-15 0.802344 -1.24635 0 down vs 1 -8.67289 75.2189 0.147639 0.41611 1cg02387554 0.062095 0.285862 1.11E-15 0.21722 -4.60363 0 down vs 1 -8.67266 75.2151 0.907845 2.55884 1cg25765464 ZNF536 0.037315 0.296917 1.11E-15 0.125675 -7.95704 0 down vs 1 -8.67256 75.2133 1.2219 3.44409 1cg26841967 -0.13668 0.120109 1.11E-15 -1.13796 1.13796 0 up vs 1 -8.67231 75.2089 1.19556 3.37007 1cg10364513 RXRG -0.31871 0.0817232 1.11E-15 -3.89987 3.89987 0 up vs 1 -8.67227 75.2083 2.90722 8.19498 1cg18457669 0.005075 0.224802 1.12E-15 0.0225755 -44.2959 0 down vs 1 -8.67063 75.1799 0.875353 2.46841 1cg22670503 FRMPD2 0.24092 0.402464 1.13E-15 0.598613 -1.67053 0 down vs 1 -8.67041 75.1761 0.47315 1.33431 1cg18133357 ADARB2 0.04209 0.333489 1.13E-15 0.126211 -7.92324 0 down vs 1 -8.67034 75.1748 1.53955 4.34169 1cg26948895 RHCG 0.226085 0.387743 1.13E-15 0.583079 -1.71503 0 down vs 1 -8.67033 75.1745 0.473821 1.33622 1cg08840152 0.2274 0.398212 1.13E-15 0.571052 -1.75115 0 down vs 1 -8.67012 75.1709 0.529001 1.49191 1cg08725455 GPR173 -0.021125 0.303079 1.13E-15 -0.0697013 -14.3469 0 down vs 1 -8.66983 75.1659 1.9057 5.37488 1cg27192854 0.16691 0.364813 1.13E-15 0.457522 -2.18569 0 down vs 1 -8.66973 75.1642 0.710104 2.00284 1cg22871908 -0.176875 0.126128 1.14E-15 -1.40234 1.40234 0 up vs 1 -8.66856 75.1439 1.66461 4.69629 1cg08889687 -0.299575 0.114288 1.14E-15 -2.62123 2.62123 0 up vs 1 -8.66808 75.1356 3.1055 8.76237 1cg10016783 SLITRK2 -0.14769 0.153557 1.15E-15 -0.961791 -1.03973 0 down vs 1 -8.66718 75.1201 1.64537 4.64348 1cg18497811 0.203305 0.388466 1.15E-15 0.523353 -1.91075 0 down vs 1 -8.66679 75.1133 0.621608 1.75443 1cg19149132 0.01294 0.342941 1.16E-15 0.0377325 -26.5024 0 down vs 1 -8.66647 75.1077 1.97446 5.57313 1cg24740868 C1orf87 -0.02684 0.220844 1.16E-15 -0.121534 -8.22818 0 down vs 1 -8.66632 75.1051 1.11228 3.13965 1cg09797577 -0.24363 0.084268 1.16E-15 -2.89113 2.89113 0 up vs 1 -8.66629 75.1046 1.94938 5.50256 1cg08688169 P2RX7 0.051285 0.341141 1.16E-15 0.150334 -6.65186 0 down vs 1 -8.66582 75.0964 1.52329 4.30029 1cg04538585 LGR5 0.13103 0.37683 1.16E-15 0.347716 -2.87591 0 down vs 1 -8.66571 75.0945 1.09543 3.09251 1cg24510494 TRIM2 -0.274675 -0.441728 1.16E-15 0.621819 -1.60819 0 down vs 1 8.66545 75.0901 0.505974 1.42851 1cg01078772 KIF3B 0.04276 -0.232394 1.17E-15 -0.183998 -5.43485 0 down vs 1 8.66464 75.0761 1.37268 3.87619 1cg23300372 CARTPT -0.298795 0.0412397 1.17E-15 -7.24533 7.24533 0 up vs 1 -8.66463 75.0758 2.09635 5.91971 1cg13330341 ASCL2 -0.259345 -0.467399 1.17E-15 0.554869 -1.80223 0 down vs 1 8.66412 75.0669 0.78482 2.21645 1cg17146473 TNXB 0.23887 0.458173 1.17E-15 0.521353 -1.91809 0 down vs 1 -8.66407 75.0662 0.871983 2.46263 1cg22310279 EDNRB -0.316845 -0.0317335 1.17E-15 9.98456 9.98456 0 up vs 1 -8.66395 75.0641 1.47383 4.16247 1cg07585257 0.25322 0.453706 1.18E-15 0.558114 -1.79175 0 down vs 1 -8.66376 75.0607 0.728764 2.05831 1cg25648746 RGS7BP -0.221185 0.120052 1.18E-15 -1.84242 1.84242 0 up vs 1 -8.66343 75.0551 2.1112 5.96328 1cg03187777 ROPN1 -0.19316 -0.026601 1.18E-15 7.26137 7.26137 0 up vs 1 -8.66293 75.0464 0.502984 1.42089 1cg25705519 SCHIP1 -0.047455 0.18676 1.19E-15 -0.254096 -3.93551 0 down vs 1 -8.66242 75.0376 0.994596 2.80998 1cg27368726 C2orf58 0.06042 0.284555 1.19E-15 0.212331 -4.70962 0 down vs 1 -8.66227 75.035 0.910831 2.57342 1cg02650401 SOX2OT -0.018545 0.284966 1.19E-15 -0.065078 -15.3662 0 down vs 1 -8.66553 75.0915 1.66938 4.69081 1cg03922946 0.05914 0.345281 1.19E-15 0.171281 -5.83837 0 down vs 1 -8.66218 75.0334 1.4845 4.19431 1cg16392213 FEZF1 -0.353045 0.031782 1.19E-15 -11.1083 11.1083 0 up vs 1 -8.66217 75.0332 2.68503 7.58632 1cg22961278 PRDM8 -0.17009 0.209105 1.19E-15 -0.813418 -1.22938 0 down vs 1 -8.66538 75.0888 2.60575 7.32218 1cg11213369 -0.10842 0.197582 1.20E-15 -0.548734 -1.82238 0 down vs 1 -8.66036 75.0018 1.69772 4.79878 1cg13315744 SNTG2 0.027785 0.279928 1.20E-15 0.0992577 -10.0748 0 down vs 1 -8.66021 74.9992 1.15269 3.2583 1cg07821297 LCE3A -0.061895 0.22528 1.21E-15 -0.274747 -3.63971 0 down vs 1 -8.65991 74.994 1.49524 4.22688 1cg16698674 GALNT2 0.230985 0.384495 1.21E-15 0.600748 -1.66459 0 down vs 1 -8.65962 74.9889 0.427261 1.2079 1cg03386869 ITGBL1 -0.04964 0.289629 1.21E-15 -0.171391 -5.8346 0 down vs 1 -8.65916 74.981 2.08693 5.90054 1cg20282837 0.205015 0.389242 1.21E-15 0.526704 -1.8986 0 down vs 1 -8.65898 74.978 0.615352 1.7399 1cg07179075 DPYD 0.101205 0.347757 1.21E-15 0.291022 -3.43616 0 down vs 1 -8.66216 75.033 1.1016 3.09781 1cg18605975 RPTOR 0.243635 0.415353 1.21E-15 0.586573 -1.70482 0 down vs 1 -8.65875 74.974 0.534626 1.51173 1cg26669044 RALYL -0.276405 0.146659 1.22E-15 -1.88468 1.88468 0 up vs 1 -8.65828 74.9658 3.24511 9.17704 1cg07909128 SPRR1A 0.11636 0.385069 1.22E-15 0.30218 -3.30929 0 down vs 1 -8.65813 74.9632 1.30913 3.70229 1cg14896926 TCERG1L 0.271895 0.449759 1.22E-15 0.604535 -1.65416 0 down vs 1 -8.65779 74.9573 0.573579 1.62224 1cg10334489 KSR1 0.028255 -0.176213 1.22E-15 -0.160346 -6.23652 0 down vs 1 8.65776 74.9568 0.757998 2.14384 1cg04756279 VSTM2B -0.38927 0.00674278 1.23E-15 -57.7314 57.7314 0 up vs 1 -8.65702 74.9441 2.84339 8.04331 1cg26164773 TIAL1 0.105435 0.357938 1.23E-15 0.294562 -3.39487 0 down vs 1 -8.65697 74.9432 1.15598 3.27006 1cg03140487 BCOR -0.214025 -0.00838918 1.23E-15 25.512 25.512 0 up vs 1 -8.65642 74.9336 0.766682 2.16908 1cg10407177 BRUNOL4 0.092575 0.321164 1.23E-15 0.288248 -3.46924 0 down vs 1 -8.6564 74.9333 0.947393 2.68035 1cg03269976 0.091155 0.342773 1.24E-15 0.265934 -3.76033 0 down vs 1 -8.65501 74.9091 1.14789 3.24865 1cg20281962 -0.29247 0.179768 1.26E-15 -1.62693 1.62693 0 up vs 1 -8.65353 74.8835 4.04334 11.4469 1cg03356595 -0.15045 0.251718 1.26E-15 -0.597693 -1.6731 0 down vs 1 -8.65275 74.8701 2.93247 8.30349 1cg17072087 VPS37B 0.11985 0.299939 1.28E-15 0.399581 -2.50262 0 down vs 1 -8.65093 74.8387 0.588021 1.66572 1cg18038372 MGMT 0.221395 0.372327 1.28E-15 0.594625 -1.68173 0 down vs 1 -8.65079 74.8362 0.413031 1.17006 1cg19084794 0.07973 0.314846 1.28E-15 0.253235 -3.9489 0 down vs 1 -8.65016 74.8253 1.00226 2.83968 1cg14929805 WISP1 0.23307 0.460967 1.29E-15 0.505611 -1.9778 0 down vs 1 -8.65005 74.8234 0.941662 2.66805 1cg01067190 ZNF311 0.156075 0.380645 1.29E-15 0.410028 -2.43886 0 down vs 1 -8.64985 74.8198 0.914368 2.59084 1cg11081894 0.03579 0.324826 1.29E-15 0.110182 -9.07588 0 down vs 1 -8.6496 74.8157 1.51468 4.29205 1cg09290655 0.003105 0.304768 1.29E-15 0.0101881 -98.154 0 down vs 1 -8.64927 74.8098 1.64992 4.67562 1cg11598178 CPZ 0.093135 0.313356 1.31E-15 0.297218 -3.36454 0 down vs 1 -8.64765 74.7818 0.879298 2.49273 1cg24156796 -0.343555 -0.0432851 1.32E-15 7.93704 7.93704 0 up vs 1 -8.64633 74.759 1.63471 4.63568 1cg08675117 RFX2 -0.03214 0.301572 1.32E-15 -0.106575 -9.38308 0 down vs 1 -8.64566 74.7474 2.01912 5.72666 1cg15736524 -0.067945 0.222514 1.32E-15 -0.305352 -3.27491 0 down vs 1 -8.64562 74.7468 1.52963 4.33841 1cg02629506 CRYBB3 0.25528 0.413981 1.32E-15 0.616647 -1.62167 0 down vs 1 -8.64543 74.7435 0.456643 1.29521 1cg11241498 -0.138275 0.173813 1.33E-15 -0.795539 -1.25701 0 down vs 1 -8.64489 74.7341 1.76592 5.00944 1cg17761419 0.30076 0.417264 1.33E-15 0.720791 -1.38737 0 down vs 1 -8.64463 74.7296 0.246093 0.69814 1cg26715883 PACSIN1 0.181385 0.383128 1.35E-15 0.473432 -2.11224 0 down vs 1 -8.64212 74.6862 0.737928 2.09464 1cg12085698 SPON1 0.25951 0.418246 1.36E-15 0.620472 -1.61168 0 down vs 1 -8.64135 74.6729 0.456844 1.297 1cg08054010 C3orf21 0.171835 0.338961 1.36E-15 0.506946 -1.9726 0 down vs 1 -8.64127 74.6715 0.506416 1.43777 1cg16862102 0.077695 0.299256 1.37E-15 0.259627 -3.85168 0 down vs 1 -8.6402 74.6531 0.890031 2.52751 1cg18864124 0.0494 0.315705 1.37E-15 0.156475 -6.39078 0 down vs 1 -8.64001 74.6498 1.28581 3.6516 1cg15604389 0.25388 0.392214 1.37E-15 0.6473 -1.54488 0 down vs 1 -8.63976 74.6455 0.346957 0.985389 1cg13027280 0.206315 0.381284 1.39E-15 0.541107 -1.84806 0 down vs 1 -8.63788 74.6129 0.555057 1.5771 1cg24859602 -0.07359 0.266789 1.40E-15 -0.275836 -3.62534 0 down vs 1 -8.63708 74.5992 2.1006 5.96959 1cg17008486 0.20968 0.452427 1.41E-15 0.463456 -2.1577 0 down vs 1 -8.63582 74.5774 1.06838 3.03706 1cg16699174 HSPA1A -0.262755 -0.436071 1.41E-15 0.602551 -1.65961 0 down vs 1 8.63528 74.568 0.544623 1.54839 1cg04103377 HTR2C 0.1801 0.396474 1.42E-15 0.454255 -2.20141 0 down vs 1 -8.63517 74.5662 0.848842 2.41335 1cg01427957 TNRC4 -0.224855 0.153304 1.42E-15 -1.46673 1.46673 0 up vs 1 -8.6342 74.5494 2.59279 7.37325 1cg24748548 -0.06711 0.358681 1.42E-15 -0.187102 -5.34468 0 down vs 1 -8.63412 74.5481 3.28709 9.34784 1cg02221053 -0.0978368 0.205279 1.43E-15 -0.476603 -2.09818 0 down vs 1 -8.63657 74.5903 1.58999 4.49774 1cg23036308 0.32555 0.477863 1.44E-15 0.681262 -1.46786 0 down vs 1 -8.63287 74.5264 0.420621 1.19651 1cg12880003 -0.355135 -0.063733 1.44E-15 5.57223 5.57223 0 up vs 1 -8.63239 74.5181 1.53958 4.38003 1cg16362123 C14orf132 0.23516 0.397421 1.45E-15 0.591715 -1.69 0 down vs 1 -8.63093 74.493 0.477361 1.35852 1cg13572911 ACO1 0.148235 0.376985 1.46E-15 0.393212 -2.54316 0 down vs 1 -8.63067 74.4885 0.948725 2.70014 1cg09417355 LCE6A -0.0867 0.283534 1.48E-15 -0.305784 -3.27028 0 down vs 1 -8.62826 74.4468 2.48525 7.07716 1cg06494770 KLHL13 -0.002065 0.298255 1.48E-15 -0.00692361 -144.433 0 down vs 1 -8.62819 74.4456 1.63525 4.65674 1cg13609973 PABPC1P2 0.217015 0.369988 1.48E-15 0.586547 -1.70489 0 down vs 1 -8.62811 74.4443 0.424273 1.20823 1cg02002583 -0.27394 0.0583088 1.48E-15 -4.69809 4.69809 0 up vs 1 -8.62802 74.4427 2.00145 5.69978 1cg27612126 STK32B 0.17162 0.40313 1.48E-15 0.425718 -2.34897 0 down vs 1 -8.62791 74.4409 0.97176 2.76747 1cg13407262 0.30082 0.382698 1.49E-15 0.786051 -1.27218 0 down vs 1 -8.62747 74.4332 0.121549 0.346195 1cg12838303 OPRM1 -0.22004 0.165062 1.49E-15 -1.33307 1.33307 0 up vs 1 -8.62717 74.4281 2.68887 7.65895 1cg26525772 GALNT2 0.19896 0.349199 1.49E-15 0.56976 -1.75512 0 down vs 1 -8.62707 74.4264 0.409248 1.16572 1cg05577810 -0.209395 0.113776 1.49E-15 -1.84041 1.84041 0 up vs 1 -8.62669 74.4197 1.89358 5.39425 1cg03359540 MYLK4 -0.126465 0.220098 1.50E-15 -0.574584 -1.74039 0 down vs 1 -8.62615 74.4105 2.17763 6.20419 1cg12653146 0.12241 0.361407 1.50E-15 0.338704 -2.95243 0 down vs 1 -8.62601 74.408 1.03562 2.95065 1cg17789464 SEMA6B 0.24604 0.441207 1.51E-15 0.557652 -1.79323 0 down vs 1 -8.62563 74.4015 0.690604 1.96781 1cg00840310 -0.326705 0.0701433 1.51E-15 -4.65768 4.65768 0 up vs 1 -8.62527 74.3953 2.8554 8.13687 1cg15488794 BCL2L1 -0.028235 -0.31192 1.52E-15 0.0905201 -11.0473 0 down vs 1 8.62447 74.3816 1.45911 4.15872 1cg13401840 SPRR2C 0.0281 0.271679 1.52E-15 0.103431 -9.66831 0 down vs 1 -8.62398 74.373 1.07572 3.06634 1cg20573931 IL7 -0.024085 0.30662 1.54E-15 -0.0785501 -12.7307 0 down vs 1 -8.62172 74.334 1.98289 5.65518 1cg01681032 MYLK4 0.279425 0.428676 1.55E-15 0.651832 -1.53414 0 down vs 1 -8.62141 74.3287 0.403882 1.15195 1cg14032667 BDKRB2 0.069945 0.243353 1.56E-15 0.287422 -3.47921 0 down vs 1 -8.62015 74.3069 0.545201 1.55548 1cg20986166 0.0608 0.336813 1.56E-15 0.180516 -5.53969 0 down vs 1 -8.61999 74.3042 1.38126 3.94093 1cg09568464 ZNF582 -0.234225 0.0533345 1.56E-15 -4.39162 4.39162 0 up vs 1 -8.61991 74.3029 1.49925 4.27764 1cg25346915 FOLH1 -0.33206 0.0701021 1.58E-15 -4.73681 4.73681 0 up vs 1 -8.61852 74.2788 2.93238 8.36934 1cg16086416 -0.02449 0.311796 1.58E-15 -0.0785448 -12.7316 0 down vs 1 -8.61846 74.2779 2.05039 5.85211 1cg27622679 SOX2OT -0.046015 0.207349 1.59E-15 -0.221921 -4.50612 0 down vs 1 -8.61728 74.2575 1.16388 3.32279 1cg00741624 KIAA1409 -0.17637 0.133557 1.59E-15 -1.32056 1.32056 0 up vs 1 -8.61705 74.2536 1.74155 4.97227 1cg23768829 -0.100395 0.160667 1.60E-15 -0.624864 -1.60035 0 down vs 1 -8.61623 74.2395 1.23568 3.52863 1cg10129768 PLCH2 0.09072 0.277736 1.60E-15 0.326642 -3.06146 0 down vs 1 -8.61593 74.2343 0.634123 1.81094 1cg20986832 -0.20952 0.14996 1.62E-15 -1.39717 1.39717 0 up vs 1 -8.6146 74.2113 2.34298 6.6932 1cg13698778 PCSK2 -0.20238 0.0798423 1.62E-15 -2.53475 2.53475 0 up vs 1 -8.61431 74.2063 1.44411 4.12568 1cg13842421 GPR133 0.13356 0.389502 1.62E-15 0.3429 -2.9163 0 down vs 1 -8.61428 74.2058 1.18768 3.39311 1cg07743805 -0.18811 0.151425 1.64E-15 -1.24226 1.24226 0 up vs 1 -8.61261 74.1771 2.0902 5.97384 1cg24548817 TRPM2 0.08523 0.268434 1.64E-15 0.317508 -3.14953 0 down vs 1 -8.61232 74.1721 0.608538 1.73934 1cg14531038 PARK7 0.35577 0.439103 1.64E-15 0.81022 -1.23423 0 down vs 1 -8.61209 74.1681 0.125908 0.359892 1cg26247373 R3HDM2 0.123445 0.327779 1.67E-15 0.37661 -2.65527 0 down vs 1 -8.61001 74.1323 0.757009 2.16486 1cg15922270 0.202645 0.397481 1.67E-15 0.509823 -1.96146 0 down vs 1 -8.60981 74.1288 0.688266 1.96836 1cg11818438 0.184245 0.365432 1.67E-15 0.504183 -1.98341 0 down vs 1 -8.60964 74.1258 0.595216 1.70232 1cg16142484 -0.230515 0.113786 1.67E-15 -2.02586 2.02586 0 up vs 1 -8.60951 74.1237 2.14929 6.14715 1cg11704005 PAX7 0.00978 0.294513 1.68E-15 0.0332074 -30.1138 0 down vs 1 -8.60849 74.1062 1.46992 4.20508 1cg07385375 RFTN1 0.360335 0.426417 1.68E-15 0.84503 -1.18339 0 down vs 1 -8.60829 74.1027 0.0791743 0.226509 1cg00904548 APEH 0.234935 0.350152 1.68E-15 0.670951 -1.49042 0 down vs 1 -8.60829 74.1027 0.240686 0.688578 1cg01032119 -0.23087 0.121859 1.71E-15 -1.89456 1.89456 0 up vs 1 -8.60623 74.0672 2.2558 6.4567 1cg03915539 CBFA2T3 0.154565 0.367093 1.72E-15 0.421052 -2.37501 0 down vs 1 -8.60528 74.0509 0.818935 2.34453 1cg07176264 SCTR -0.224955 0.153622 1.75E-15 -1.46434 1.46434 0 up vs 1 -8.6027 74.0064 2.59852 7.44377 1cg07821364 PLCH2 0.2505 0.428218 1.75E-15 0.584983 -1.70945 0 down vs 1 -8.60268 74.0061 0.572636 1.64039 1cg06602478 ACAP2 0.156395 -0.172452 1.75E-15 -0.906892 -1.10267 0 down vs 1 8.6021 73.9962 1.96067 5.61734 1cg18131548 TAL1 -0.071885 0.188582 1.75E-15 -0.381186 -2.62339 0 down vs 1 -8.60188 73.9924 1.23006 3.52431 1cg13898875 VIT 0.09459 0.349772 1.76E-15 0.270434 -3.69777 0 down vs 1 -8.6011 73.9789 1.18064 3.38333 1cg12648074 PCDHGA4 -0.181935 0.14827 1.77E-15 -1.22705 1.22705 0 up vs 1 -8.60053 73.969 1.9769 5.66593 1cg21997766 GRIN2C 0.09098 0.35455 1.79E-15 0.256607 -3.89701 0 down vs 1 -8.59852 73.9345 1.25953 3.61159 1cg06294954 CUX1 0.30385 0.403481 1.80E-15 0.753071 -1.3279 0 down vs 1 -8.59832 73.931 0.179974 0.516083 1cg06400319 SPRR2B 0.1016 0.343422 1.80E-15 0.295846 -3.38014 0 down vs 1 -8.59771 73.9206 1.06025 3.04075 1cg01476678 UNC5A 0.04598 0.331065 1.81E-15 0.138885 -7.20019 0 down vs 1 -8.59709 73.91 1.47356 4.22668 1cg14030402 C1orf123 0.325745 0.419569 1.82E-15 0.77638 -1.28803 0 down vs 1 -8.59644 73.8988 0.159605 0.457873 1cg02010152 HLA-DOB -0.005875 0.265729 1.84E-15 -0.022109 -45.2305 0 down vs 1 -8.60094 73.9762 1.33619 3.79311 1cg18163442 -0.023485 0.256666 1.85E-15 -0.0915002 -10.9289 0 down vs 1 -8.59356 73.8492 1.42299 4.085 1cg14880184 GATA6 0.136405 0.370942 1.85E-15 0.367726 -2.71941 0 down vs 1 -8.59349 73.8481 0.997332 2.8631 1cg04406115 KDM4B 0.267435 0.397724 1.86E-15 0.672413 -1.48718 0 down vs 1 -8.59276 73.8355 0.307776 0.883701 1cg13878705 PTPRN2 0.212265 0.373339 1.87E-15 0.568558 -1.75884 0 down vs 1 -8.59226 73.8269 0.470403 1.3508 1cg01745044 HCCA2 0.165195 0.380574 1.88E-15 0.434068 -2.30378 0 down vs 1 -8.59139 73.812 0.841053 2.41564 1cg19453794 PCGF3 -0.26599 -0.143559 1.90E-15 1.85282 1.85282 0 up vs 1 -8.58964 73.7819 0.271768 0.780881 1cg21451930 LAMC2 0.134125 0.37237 1.90E-15 0.360193 -2.77629 0 down vs 1 -8.58945 73.7786 1.02912 2.95712 1cg11891843 0.0496 0.290861 1.91E-15 0.170528 -5.86414 0 down vs 1 -8.5889 73.7693 1.05534 3.03287 1cg14479837 TMEM132C -0.13261 0.199905 1.91E-15 -0.663366 -1.50746 0 down vs 1 -8.58851 73.7625 2.00465 5.76155 1cg02391906 LPPR1 -0.35094 0.0349376 1.91E-15 -10.0448 10.0448 0 up vs 1 -8.58843 73.761 2.69971 7.75936 1cg18676790 -0.26417 0.182029 1.92E-15 -1.45125 1.45125 0 up vs 1 -8.58757 73.7464 3.60974 10.377 1cg21467108 MCTP2 0.013 0.299476 1.93E-15 0.0434091 -23.0366 0 down vs 1 -8.58728 73.7414 1.48797 4.27779 1cg21224025 WNT7A 0.14997 0.331984 1.93E-15 0.451739 -2.21367 0 down vs 1 -8.58705 73.7374 0.600659 1.72693 1cg17014866 0.19903 0.393211 1.94E-15 0.506166 -1.97564 0 down vs 1 -8.58636 73.7256 0.683645 1.96584 1cg03875996 RXFP1 -0.031285 0.329521 1.94E-15 -0.0949408 -10.5329 0 down vs 1 -8.58628 73.7243 2.36029 6.78721 1cg01254505 BST2 -0.323715 -0.4536 1.95E-15 0.713657 -1.40123 0 down vs 1 8.58572 73.7146 0.305869 0.879667 1cg01341655 0.075945 0.317641 1.95E-15 0.23909 -4.18252 0 down vs 1 -8.58569 73.714 1.05915 3.0461 1cg25388715 CASP7 0.20322 0.416479 1.95E-15 0.487948 -2.0494 0 down vs 1 -8.58537 73.7085 0.824579 2.37165 1cg23219336 LELP1 0.096035 0.365951 1.95E-15 0.262426 -3.8106 0 down vs 1 -8.58536 73.7085 1.32091 3.7992 1cg04020735 -0.040015 0.261309 1.96E-15 -0.153133 -6.53027 0 down vs 1 -8.58509 73.7037 1.64621 4.73512 1cg07452842 FAT3 0.240455 0.416009 1.96E-15 0.578005 -1.73009 0 down vs 1 -8.58487 73.7 0.558777 1.60734 1cg11642382 CNTNAP2 -0.341035 0.0042701 1.96E-15 -79.8658 79.8658 0 up vs 1 -8.5845 73.6936 2.16184 6.21913 1cg19241305 -0.09764 0.0773753 1.97E-15 -1.2619 1.2619 0 up vs 1 -8.58435 73.6911 0.555354 1.59768 1cg13620490 0.28328 0.42462 1.97E-15 0.667138 -1.49894 0 down vs 1 -8.58417 73.688 0.362198 1.04204 1cg01393604 LOC285830 0.04517 0.259716 1.97E-15 0.17392 -5.74976 0 down vs 1 -8.58393 73.6838 0.834566 2.40118 1cg13671198 PBX3 0.19688 0.3817 1.98E-15 0.515798 -1.93874 0 down vs 1 -8.5832 73.6714 0.619321 1.78219 1cg20536469 NRXN1 -0.27122 0.048832 1.98E-15 -5.55415 5.55415 0 up vs 1 -8.58297 73.6674 1.8572 5.34465 1cg15398400 C10orf82 -0.008425 0.282866 1.99E-15 -0.0297844 -33.5746 0 down vs 1 -8.58277 73.6639 1.53841 4.42744 1cg08327394 -0.0948 0.274353 1.99E-15 -0.34554 -2.89402 0 down vs 1 -8.58235 73.6567 2.47076 7.11139 1cg08105265 EPX 0.23295 0.391454 1.99E-15 0.595089 -1.68042 0 down vs 1 -8.5822 73.6542 0.455511 1.3111 1cg06600725 -0.15986 0.237197 2.00E-15 -0.673953 -1.48378 0 down vs 1 -8.58139 73.6402 2.85841 8.22897 1cg23166865 -0.33535 0.0270778 2.01E-15 -12.3847 12.3847 0 up vs 1 -8.58066 73.6277 2.38156 6.85734 1cg23229395 ZSCAN18 -0.3215 0.060917 2.01E-15 -5.27767 5.27767 0 up vs 1 -8.58063 73.6272 2.6515 7.63466 1cg04910921 -0.217215 0.131077 2.01E-15 -1.65715 1.65715 0 up vs 1 -8.58061 73.6269 2.19941 6.33294 1cg14040602 0.16714 0.354097 2.01E-15 0.472018 -2.11856 0 down vs 1 -8.58048 73.6247 0.633725 1.82479 1cg08003887 0.1703 0.383516 2.04E-15 0.444049 -2.25201 0 down vs 1 -8.57864 73.5931 0.824251 2.37442 1cg17075096 FAM24B 0.25128 0.419302 2.04E-15 0.599282 -1.66866 0 down vs 1 -8.57827 73.5867 0.511859 1.47464 1cg09612454 C21orf54 0.05085 0.322945 2.05E-15 0.157457 -6.35093 0 down vs 1 -8.57812 73.5842 1.34233 3.86732 1cg03285823 NFATC1 0.20715 0.401859 2.05E-15 0.51548 -1.93994 0 down vs 1 -8.57785 73.5795 0.687367 1.98047 1cg24045590 0.221315 0.45263 2.05E-15 0.488953 -2.04519 0 down vs 1 -8.57769 73.5767 0.970124 2.79526 1cg25923788 0.06616 0.332154 2.05E-15 0.199185 -5.02046 0 down vs 1 -8.57767 73.5765 1.2828 3.69622 1cg03226872 BLCAP 0.230435 -0.093849 2.06E-15 -2.45538 2.45538 0 up vs 1 8.57708 73.5663 1.90664 5.49447 1cg00838829 SSBP3 0.311675 0.427374 2.08E-15 0.72928 -1.37122 0 down vs 1 -8.57562 73.5413 0.242703 0.699648 1cg22474464 NKX2-2 -0.26358 0.15997 2.10E-15 -1.64768 1.64768 0 up vs 1 -8.5742 73.5169 3.25258 9.37943 1cg14321042 0.26908 0.425459 2.11E-15 0.632446 -1.58116 0 down vs 1 -8.57355 73.5057 0.44338 1.27877 1cg02765177 CUZD1 0.143185 0.371576 2.11E-15 0.385345 -2.59507 0 down vs 1 -8.57334 73.5021 0.945747 2.72779 1cg00264419 TNFRSF10B 0.163205 -0.154994 2.15E-15 -1.05297 1.05297 0 up vs 1 8.57058 73.4549 1.83576 5.29824 1cg06060191 LPPR4 -0.368625 0.0982356 2.16E-15 -3.75246 3.75246 0 up vs 1 -8.56976 73.4408 3.95178 11.4075 1cg01431960 ZFR2 0.108175 0.299771 2.17E-15 0.360859 -2.77116 0 down vs 1 -8.56919 73.4311 0.665563 1.92152 1cg13545633 0.01655 0.272762 2.18E-15 0.0606755 -16.4811 0 down vs 1 -8.56813 73.4129 1.1902 3.43702 1cg18057887 IGSF9B 0.18705 0.430646 2.19E-15 0.434348 -2.3023 0 down vs 1 -8.56765 73.4047 1.07587 3.10721 1cg02907374 CALCB -0.28303 0.123287 2.19E-15 -2.29571 2.29571 0 up vs 1 -8.56734 73.3992 2.99328 8.64552 1cg25653995 TNXB 0.283525 0.451214 2.23E-15 0.62836 -1.59144 0 down vs 1 -8.56498 73.3589 0.509832 1.47336 1cg01799791 0.29378 0.451398 2.23E-15 0.650823 -1.53652 0 down vs 1 -8.56494 73.3582 0.450432 1.30172 1cg07220448 SOSTDC1 -0.328145 0.147065 2.23E-15 -2.23129 2.23129 0 up vs 1 -8.56494 73.3582 4.09439 11.8325 1cg26126879 0.269765 0.39621 2.23E-15 0.680863 -1.46872 0 down vs 1 -8.56446 73.35 0.289883 0.837837 1cg12253414 ITGB5 0.321485 0.437251 2.24E-15 0.735241 -1.3601 0 down vs 1 -8.56401 73.3423 0.242985 0.702362 1cg19187616 -0.010525 0.28076 2.25E-15 -0.0374875 -26.6756 0 down vs 1 -8.56348 73.3332 1.53835 4.44724 1cg18469036 DIP2C 0.26095 0.446635 2.25E-15 0.584258 -1.71157 0 down vs 1 -8.56324 73.329 0.625132 1.80731 1cg02948862 CBFA2T3 0.02949 0.312658 2.25E-15 0.0943202 -10.6022 0 down vs 1 -8.56311 73.3269 1.45381 4.20319 1cg19358877 ZNF471 -0.41169 0.0563129 2.26E-15 -7.31076 7.31076 0 up vs 1 -8.56272 73.3202 3.97114 11.4823 1cg26444116 -0.085115 0.245118 2.27E-15 -0.347241 -2.87985 0 down vs 1 -8.56184 73.3051 1.97724 5.71822 1cg21200986 POR 0.31096 0.419049 2.30E-15 0.742061 -1.3476 0 down vs 1 -8.56028 73.2784 0.211827 0.61283 1cg24934063 FEZF1 -0.373715 -0.0355902 2.31E-15 10.5005 10.5005 0 up vs 1 -8.55958 73.2665 2.07287 5.99795 1cg04676766 TIGD4 0.077935 0.313649 2.34E-15 0.248478 -4.02449 0 down vs 1 -8.55745 73.2299 1.00737 2.91633 1cg23698536 FAM83A 0.27145 0.39389 2.34E-15 0.689151 -1.45106 0 down vs 1 -8.55734 73.2281 0.27181 0.786909 1cg17725595 BGN 0.156695 0.343731 2.34E-15 0.455865 -2.19363 0 down vs 1 -8.55711 73.2241 0.634265 1.83634 1cg11232368 DPYD 0.25176 0.417719 2.35E-15 0.602702 -1.6592 0 down vs 1 -8.55635 73.2111 0.499367 1.44603 1cg26690876 WNT2B 0.28463 0.415999 2.36E-15 0.684208 -1.46154 0 down vs 1 -8.55617 73.2081 0.312901 0.906116 1cg02930033 0.22404 0.4076 2.36E-15 0.549657 -1.81932 0 down vs 1 -8.55575 73.2008 0.610906 1.76927 1cg10266211 MSX1 -0.006505 0.209915 2.39E-15 -0.0309887 -32.2698 0 down vs 1 -8.55425 73.1752 0.849205 2.46028 1cg25590826 CCDC33 -0.290385 0.0509098 2.39E-15 -5.70391 5.70391 0 up vs 1 -8.55425 73.1751 2.11192 6.11857 1cg03793270 NOX4 -0.27784 0.104163 2.40E-15 -2.66736 2.66736 0 up vs 1 -8.55322 73.1577 2.64576 7.66703 1cg03952885 0.10646 0.342797 2.41E-15 0.310562 -3.21996 0 down vs 1 -8.55287 73.1515 1.0127 2.93491 1cg25906537 TYRO3 0.35217 0.424072 2.42E-15 0.830449 -1.20417 0 down vs 1 -8.55228 73.1414 0.0937337 0.271687 1cg13941474 -0.08183 0.309021 2.44E-15 -0.264804 -3.77638 0 down vs 1 -8.5509 73.1179 2.76975 8.03069 1cg03371447 SLC6A6 0.363225 0.43286 2.45E-15 0.839129 -1.19171 0 down vs 1 -8.5499 73.1008 0.0879165 0.254967 1cg26102435 0.204845 0.401529 2.46E-15 0.510163 -1.96016 0 down vs 1 -8.54965 73.0965 0.701383 2.03421 1cg14227418 UBAP2 0.233535 0.374309 2.46E-15 0.62391 -1.6028 0 down vs 1 -8.54952 73.0942 0.359303 1.04211 1cg27139419 IGF1R 0.04614 -0.322364 2.46E-15 -0.14313 -6.98666 0 down vs 1 8.54945 73.0931 2.46209 7.14106 1cg25607249 SLC1A5 -0.371365 -0.458345 2.47E-15 0.81023 -1.23422 0 down vs 1 8.54874 73.0809 0.13717 0.397917 1cg09082287 DNAJC6 -0.254545 0.141609 2.47E-15 -1.79752 1.79752 0 up vs 1 -8.54862 73.0789 2.84541 8.25447 1cg08976526 C6orf10 0.16783 0.381609 2.49E-15 0.439795 -2.27378 0 down vs 1 -8.54759 73.0613 0.828608 2.40435 1cg11258089 PDE4D -0.34275 0.0432521 2.49E-15 -7.92448 7.92448 0 up vs 1 -8.5474 73.058 2.70145 7.83908 1cg23787321 -0.27949 -0.424608 2.50E-15 0.65823 -1.51923 0 down vs 1 8.54701 73.0514 0.381823 1.10808 1cg08521684 MUC4 -0.054235 0.247746 2.50E-15 -0.218913 -4.56802 0 down vs 1 -8.54696 73.0505 1.6534 4.79834 1cg08732526 LCE3A -0.06277 0.184749 2.51E-15 -0.339758 -2.94327 0 down vs 1 -8.54649 73.0426 1.1108 3.224 1cg04445570 KIRREL3 0.1013 0.350556 2.51E-15 0.28897 -3.46057 0 down vs 1 -8.54634 73.0398 1.12644 3.26952 1cg12497187 FAM81A 0.23576 0.392188 2.52E-15 0.60114 -1.66351 0 down vs 1 -8.54566 73.0282 0.443657 1.28793 1cg07568841 ZNRF2 -0.04495 -0.332212 2.53E-15 0.135305 -7.39071 0 down vs 1 8.54515 73.0196 1.49615 4.34382 1cg20227714 0.100885 0.389132 2.54E-15 0.259256 -3.85719 0 down vs 1 -8.54445 73.0076 1.50643 4.37438 1cg17374727 LUZP6 0.27967 0.421208 2.55E-15 0.663972 -1.50609 0 down vs 1 -8.54392 72.9986 0.363214 1.05483 1cg20390045 RAB3C -0.2809 0.0918052 2.55E-15 -3.05974 3.05974 0 up vs 1 -8.54373 72.9953 2.51854 7.31459 1cg22319267 GRM8 -0.070235 0.289811 2.56E-15 -0.242348 -4.1263 0 down vs 1 -8.54336 72.989 2.35036 6.82672 1cg03017135 0.00734 0.211301 2.57E-15 0.0347372 -28.7876 0 down vs 1 -8.54273 72.9783 0.754245 2.19106 1cg01931714 -0.078795 0.20265 2.60E-15 -0.388823 -2.57186 0 down vs 1 -8.54095 72.9478 1.43617 4.17377 1cg09842892 ZNF311 0.17289 0.404707 2.60E-15 0.427198 -2.34083 0 down vs 1 -8.54083 72.9458 0.974333 2.83167 1cg01057196 LOC284688 0.0068 0.283808 2.61E-15 0.0239599 -41.7364 0 down vs 1 -8.54058 72.9416 1.39124 4.04355 1cg04347708 -0.01264 0.25719 2.61E-15 -0.0491465 -20.3473 0 down vs 1 -8.54012 72.9336 1.32008 3.83714 1cg25517810 -0.10813 0.205613 2.62E-15 -0.525891 -1.90153 0 down vs 1 -8.53955 72.924 1.7847 5.18837 1cg02892925 TOX 0.063905 0.285648 2.65E-15 0.223719 -4.46988 0 down vs 1 -8.53806 72.8985 0.891492 2.5926 1cg27121661 RFTN1 0.071515 0.337147 2.65E-15 0.212118 -4.71436 0 down vs 1 -8.53785 72.895 1.27932 3.72065 1cg14490819 0.309915 0.468938 2.68E-15 0.660887 -1.51312 0 down vs 1 -8.53639 72.87 0.458496 1.3339 1cg10014874 TNXB 0.235445 0.425297 2.72E-15 0.553601 -1.80636 0 down vs 1 -8.53395 72.8283 0.653507 1.90233 1cg27326032 0.23658 0.46414 2.74E-15 0.509717 -1.96187 0 down vs 1 -8.53275 72.8079 0.938881 2.73381 1cg03213833 ADARB2 0.05941 0.334368 2.75E-15 0.177678 -5.62814 0 down vs 1 -8.53221 72.7987 1.37073 3.99175 1cg13747145 SUSD5 0.237515 0.400181 2.79E-15 0.593519 -1.68487 0 down vs 1 -8.53022 72.7646 0.479746 1.39774 1cg23227149 ACTN2 0.131095 0.398161 2.79E-15 0.329251 -3.0372 0 down vs 1 -8.53021 72.7646 1.29317 3.76766 1cg14182974 RCL1 -0.186975 0.221934 2.79E-15 -0.84248 -1.18697 0 down vs 1 -8.53003 72.7613 3.0316 8.83296 1cg00769882 HSPA6 0.15065 0.367354 2.79E-15 0.410095 -2.43846 0 down vs 1 -8.52981 72.7577 0.851437 2.4809 1cg17391928 SLC27A6 -0.35919 -0.0136155 2.80E-15 26.381 26.381 0 up vs 1 -8.52923 72.7478 2.16522 6.30982 1cg24371954 COL4A2 0.09976 0.357041 2.82E-15 0.279408 -3.579 0 down vs 1 -8.52812 72.7289 1.20015 3.49835 1cg16415819 0.196265 0.39719 2.84E-15 0.494134 -2.02374 0 down vs 1 -8.52728 72.7145 0.731959 2.13403 1cg14011611 TNXB 0.191515 0.400388 2.84E-15 0.478323 -2.09064 0 down vs 1 -8.52722 72.7135 0.791012 2.30624 1cg05138768 0.002675 0.321384 2.84E-15 0.00832339 -120.143 0 down vs 1 -8.527 72.7097 1.84164 5.36968 1cg19091784 TREX1 -0.270655 -0.481444 2.87E-15 0.562173 -1.77881 0 down vs 1 8.52536 72.6818 0.805592 2.34977 1cg10640961 ODZ4 0.193215 0.37734 2.88E-15 0.512045 -1.95295 0 down vs 1 -8.52505 72.6765 0.61467 1.79301 1cg22272282 HIST1H2BK -0.02344 -0.382083 2.88E-15 0.0613479 -16.3005 0 down vs 1 8.52482 72.6725 2.33208 6.80313 1cg15543551 FGF12 -0.31708 0.0093634 2.93E-15 -33.8638 33.8638 0 up vs 1 -8.5222 72.6279 1.93212 5.63983 1cg14415914 APOL3 0.253265 0.400477 2.95E-15 0.632408 -1.58126 0 down vs 1 -8.52148 72.6157 0.392922 1.14713 1cg14617010 0.15367 0.378204 2.95E-15 0.406316 -2.46114 0 down vs 1 -8.5212 72.6109 0.914073 2.66879 1cg26361999 0.00868 0.180729 3.00E-15 0.0480277 -20.8213 0 down vs 1 -8.51846 72.5642 0.536688 1.56796 1cg27538686 ST6GALNAC3 -0.387735 -0.0342428 3.01E-15 11.3231 11.3231 0 up vs 1 -8.51833 72.562 2.26557 6.61918 1cg09235539 DNAJC6 -0.297955 0.119355 3.01E-15 -2.49637 2.49637 0 up vs 1 -8.51823 72.5602 3.15745 9.22515 1cg23463269 INSC -0.239875 0.105975 3.01E-15 -2.2635 2.2635 0 up vs 1 -8.5181 72.5581 2.16867 6.33642 1cg15301316 -0.27467 0.0953459 3.01E-15 -2.88077 2.88077 0 up vs 1 -8.518 72.5562 2.48233 7.25303 1cg19742055 EDNRB -0.27672 0.0146727 3.01E-15 -18.8595 18.8595 0 up vs 1 -8.51794 72.5554 1.53948 4.49823 1cg10782349 ZNF701 -0.36508 -0.015432 3.02E-15 23.6574 23.6574 0 up vs 1 -8.51754 72.5484 2.21656 6.47721 1cg20928986 SP110 0.09677 0.279108 3.03E-15 0.346712 -2.88424 0 down vs 1 -8.51712 72.5413 0.602797 1.76166 1cg26164310 LPPR4 -0.370045 0.0634103 3.04E-15 -5.83572 5.83572 0 up vs 1 -8.5168 72.5359 3.40649 9.9561 1cg13589810 0.013565 0.249631 3.04E-15 0.0543402 -18.4026 0 down vs 1 -8.51671 72.5343 1.01038 2.95309 1cg00852549 NXPH1 -0.31784 0.0955103 3.07E-15 -3.32781 3.32781 0 up vs 1 -8.51521 72.5088 3.09781 9.05732 1cg11419025 C8orf74 0.220535 0.403862 3.07E-15 0.546065 -1.83128 0 down vs 1 -8.51508 72.5066 0.609358 1.78168 1cg04881642 LOC100130017 0.230965 0.373118 3.08E-15 0.619013 -1.61547 0 down vs 1 -8.51471 72.5003 0.366379 1.07134 1cg03301498 MED29 0.15373 -0.160606 3.09E-15 -0.957186 -1.04473 0 down vs 1 8.51425 72.4924 1.79146 5.23902 1cg03851835 SLC35F2 -0.307305 -0.42151 3.09E-15 0.729058 -1.37163 0 down vs 1 8.51394 72.4871 0.236476 0.691611 1cg00940977 -0.27686 -0.396706 3.12E-15 0.697897 -1.43288 0 down vs 1 8.51265 72.4652 0.260415 0.761856 1cg14519115 PLCXD3 -0.33358 0.0108186 3.12E-15 -30.8341 30.8341 0 up vs 1 -8.51264 72.4651 2.15051 6.2914 1cg24708097 0.18766 0.392598 3.12E-15 0.477995 -2.09207 0 down vs 1 -8.51254 72.4634 0.761487 2.22782 1cg10662603 0.1194 0.346874 3.15E-15 0.344217 -2.90514 0 down vs 1 -8.51115 72.4398 0.938172 2.74563 1cg11526778 JAZF1 0.10389 0.373054 3.17E-15 0.278485 -3.59085 0 down vs 1 -8.51003 72.4207 1.31356 3.84524 1cg10129470 0.12868 0.336 3.18E-15 0.382976 -2.61113 0 down vs 1 -8.50975 72.4158 0.779292 2.28141 1cg26029997 TREX1 -0.289245 -0.479144 3.18E-15 0.603671 -1.65653 0 down vs 1 8.50958 72.4129 0.653826 1.91418 1cg04306063 CRHBP -0.13148 0.157644 3.19E-15 -0.834032 -1.19899 0 down vs 1 -8.50911 72.4049 1.5156 4.43766 1cg00750428 -0.35281 -0.039867 3.21E-15 8.84967 8.84967 0 up vs 1 -8.50831 72.3914 1.77561 5.19993 1cg07195011 CTNND2 -0.156785 0.258634 3.23E-15 -0.606205 -1.64961 0 down vs 1 -8.50711 72.3709 3.12889 9.16561 1cg17162031 MYT1L 0.020415 0.305014 3.23E-15 0.0669313 -14.9407 0 down vs 1 -8.50702 72.3694 1.46854 4.30196 1cg02630854 0.25487 0.405714 3.25E-15 0.6282 -1.59185 0 down vs 1 -8.50639 72.3588 0.41255 1.20871 1cg04100971 IL18 0.135565 0.340277 3.31E-15 0.398396 -2.51006 0 down vs 1 -8.50353 72.3101 0.759808 2.22762 1cg23380397 0.11115 0.37677 3.31E-15 0.295008 -3.38974 0 down vs 1 -8.50336 72.3071 1.2792 3.75053 1cg26978064 -0.12271 -0.315973 3.32E-15 0.388356 -2.57496 0 down vs 1 8.50276 72.297 0.677199 1.98578 1cg03722487 PLCH2 -0.074645 0.239481 3.33E-15 -0.311694 -3.20827 0 down vs 1 -8.50264 72.295 1.78907 5.24632 1cg14054842 ADARB2 0.292345 0.468077 3.33E-15 0.624566 -1.60111 0 down vs 1 -8.50254 72.2932 0.559911 1.64194 1cg13596713 GALNT9 0.06971 0.360539 3.34E-15 0.193349 -5.17199 0 down vs 1 -8.50215 72.2866 1.53354 4.49751 1cg00216061 ZFPM1 0.11956 0.325838 3.36E-15 0.366931 -2.72531 0 down vs 1 -8.50098 72.2666 0.771479 2.2632 1cg02953039 CYP21A2 0.03084 0.23944 3.40E-15 0.128801 -7.76393 0 down vs 1 -8.49907 72.2342 0.788942 2.31547 1cg15854666 ARX -0.28542 0.0719108 3.41E-15 -3.96908 3.96908 0 up vs 1 -8.4989 72.2313 2.31504 6.79469 1cg26098871 ENGASE 0.22796 0.374371 3.42E-15 0.608914 -1.64227 0 down vs 1 -8.49824 72.2201 0.388657 1.14089 1cg09467508 -0.31939 0.0975856 3.44E-15 -3.27292 3.27292 0 up vs 1 -8.4975 72.2074 3.15238 9.25535 1cg25230284 ASPA 0.10248 0.327599 3.45E-15 0.312821 -3.19672 0 down vs 1 -8.49705 72.1998 0.918849 2.69801 1cg13802760 -0.0267 0.235264 3.45E-15 -0.113489 -8.8114 0 down vs 1 -8.49687 72.1969 1.24424 3.65359 1cg00798317 ITGA3 0.194175 0.325794 3.47E-15 0.596005 -1.67784 0 down vs 1 -8.49602 72.1824 0.314092 0.92249 1cg13346463 0.281045 0.419449 3.47E-15 0.670034 -1.49246 0 down vs 1 -8.49593 72.1808 0.347308 1.02007 1cg04819096 HOXC13 -0.13606 0.111275 3.48E-15 -1.22273 1.22273 0 up vs 1 -8.49575 72.1778 1.10915 3.25778 1cg13912115 RXFP3 -0.31702 0.0486351 3.48E-15 -6.51834 6.51834 0 up vs 1 -8.49571 72.177 2.42416 7.12029 1cg17069437 TSC22D4 0.120715 0.33323 3.48E-15 0.362257 -2.76047 0 down vs 1 -8.49547 72.1729 0.81884 2.40525 1cg08542640 0.039845 0.250234 3.49E-15 0.159231 -6.28017 0 down vs 1 -8.49507 72.1662 0.802531 2.35757 1cg11554153 FAM20C 0.12434 0.299402 3.51E-15 0.415294 -2.40793 0 down vs 1 -8.49412 72.1501 0.555651 1.63268 1cg23180941 0.30475 0.431732 3.51E-15 0.705878 -1.41668 0 down vs 1 -8.49404 72.1488 0.292349 0.859031 1cg08753468 -0.14091 0.121556 3.52E-15 -1.15922 1.15922 0 up vs 1 -8.49366 72.1423 1.24901 3.67038 1cg18672030 RELL1 -0.24581 -0.423333 3.54E-15 0.580654 -1.7222 0 down vs 1 8.49277 72.1272 0.571383 1.67944 1cg15615793 CRHR2 0.002495 0.267802 3.56E-15 0.00931658 -107.335 0 down vs 1 -8.49214 72.1164 1.27619 3.75161 1cg23676439 LRAT -0.27624 0.0638851 3.56E-15 -4.32402 4.32402 0 up vs 1 -8.49204 72.1147 2.09747 6.16605 1cg04690347 KRT40 0.326885 0.430775 3.57E-15 0.75883 -1.31782 0 down vs 1 -8.49156 72.1067 0.195688 0.575339 1cg25985659 FAM155A -0.32889 -0.0228175 3.58E-15 14.4139 14.4139 0 up vs 1 -8.49122 72.1009 1.6985 4.99415 1cg13677800 GPR26 0.136855 0.345027 3.58E-15 0.39665 -2.52111 0 down vs 1 -8.49105 72.0979 0.785709 2.31034 1cg06651605 0.23551 0.456097 3.58E-15 0.516359 -1.93664 0 down vs 1 -8.49101 72.0972 0.882225 2.59416 1cg10761085 TSSC1 0.20081 0.35923 3.58E-15 0.559001 -1.7889 0 down vs 1 -8.491 72.0972 0.455027 1.338 1cg00246768 CSMD1 -0.002385 0.327062 3.59E-15 -0.0072922 -137.133 0 down vs 1 -8.49081 72.0939 1.96784 5.78664 1cg14989243 FILIP1 -0.13577 0.251609 3.60E-15 -0.539608 -1.8532 0 down vs 1 -8.49042 72.0873 2.72076 8.00141 1cg27310219 0.14284 0.390695 3.62E-15 0.365605 -2.7352 0 down vs 1 -8.48929 72.0681 1.11382 3.27648 1cg23491743 SNED1 0.08625 0.404353 3.62E-15 0.213304 -4.68815 0 down vs 1 -8.48925 72.0674 1.83465 5.39698 1cg14008883 SLC18A3 -0.211195 0.0644711 3.63E-15 -3.27581 3.27581 0 up vs 1 -8.4891 72.0648 1.3778 4.0532 1cg05288711 0.19318 0.403602 3.64E-15 0.47864 -2.08925 0 down vs 1 -8.48876 72.0591 0.802787 2.36182 1cg17084653 0.009255 0.267313 3.64E-15 0.0346223 -28.8831 0 down vs 1 -8.48853 72.0552 1.2074 3.55241 1cg05417162 -0.28297 -0.0145804 3.73E-15 19.4075 19.4075 0 up vs 1 -8.48479 71.9917 1.30602 3.84594 1cg20858622 TNXB 0.18337 0.439441 3.73E-15 0.41728 -2.39647 0 down vs 1 -8.48469 71.9899 1.18888 3.50109 1cg26053881 PRDM16 0.18282 0.386151 3.73E-15 0.473442 -2.11219 0 down vs 1 -8.48464 71.9892 0.749589 2.20745 1cg00852921 EEF1A2 -0.05634 0.264093 3.75E-15 -0.213334 -4.68749 0 down vs 1 -8.48396 71.9777 1.86163 5.48317 1cg16466881 0.11995 0.36857 3.77E-15 0.325447 -3.07269 0 down vs 1 -8.48318 71.9644 1.1207 3.30147 1cg23948240 -0.237445 0.189298 3.77E-15 -1.25434 1.25434 0 up vs 1 -8.48293 71.9601 3.30181 9.72737 1cg16428517 0.03205 0.263082 3.78E-15 0.121825 -8.20849 0 down vs 1 -8.48288 71.9593 0.967748 2.85109 1cg10118210 FAM155A -0.29234 0.152743 3.78E-15 -1.91393 1.91393 0 up vs 1 -8.48275 71.957 3.5917 10.5819 1cg04574046 ESYT2 0.284725 0.413468 3.82E-15 0.688626 -1.45217 0 down vs 1 -8.48118 71.9304 0.300515 0.885705 1cg17799563 DDA1 0.20374 -0.0664309 3.82E-15 -3.06694 3.06694 0 up vs 1 8.4811 71.929 1.32341 3.90056 1cg04394641 SDHB 0.31629 0.439959 3.82E-15 0.718907 -1.391 0 down vs 1 -8.48105 71.9281 0.277295 0.817295 1cg05161980 0.11768 0.335186 3.84E-15 0.351089 -2.84828 0 down vs 1 -8.48034 71.9161 0.857746 2.52853 1cg11537355 DPP6 -0.078605 0.316985 3.86E-15 -0.247977 -4.03263 0 down vs 1 -8.47941 71.9004 2.83732 8.36591 1cg21609106 EGFLAM -0.329275 0.0786402 3.87E-15 -4.18711 4.18711 0 up vs 1 -8.47921 71.897 3.01688 8.89575 1cg01730668 CDH4 0.070675 0.308124 3.88E-15 0.229372 -4.35973 0 down vs 1 -8.47878 71.8896 1.02226 3.01459 1cg13997645 INSC -0.29081 0.0147474 3.89E-15 -19.7194 19.7194 0 up vs 1 -8.47832 71.8819 1.69279 4.99252 1cg23804620 -0.411985 -0.478402 3.89E-15 0.86117 -1.16121 0 down vs 1 8.47821 71.8801 0.079978 0.235884 1cg09412654 0.23092 0.391544 3.90E-15 0.589768 -1.69558 0 down vs 1 -8.47777 71.8726 0.467776 1.37978 1cg15365218 0.142075 0.31848 3.91E-15 0.446103 -2.24164 0 down vs 1 -8.47755 71.8688 0.564211 1.66432 1cg18460938 AGBL1 0.107905 0.368987 3.92E-15 0.292436 -3.41956 0 down vs 1 -8.47712 71.8616 1.23587 3.64595 1cg18943588 0.011135 0.288975 3.92E-15 0.0385327 -25.952 0 down vs 1 -8.47686 71.8572 1.39961 4.12927 1cg05133398 ZNF311 0.151925 0.387218 3.93E-15 0.39235 -2.54874 0 down vs 1 -8.47653 71.8516 1.00377 2.96166 1cg18302890 ADARB2 0.16247 0.349685 3.95E-15 0.464619 -2.1523 0 down vs 1 -8.47593 71.8414 0.635473 1.87525 1cg16790794 NKX2-1 -0.22601 0.0320567 3.97E-15 -7.05032 7.05032 0 up vs 1 -8.47493 71.8245 1.20749 3.56406 1cg09993699 BST2 -0.02138 -0.297065 4.01E-15 0.0719707 -13.8945 0 down vs 1 8.47343 71.799 1.37799 4.06877 1cg26532812 IGSF9B 0.20624 0.450943 4.02E-15 0.457353 -2.1865 0 down vs 1 -8.47309 71.7932 1.08566 3.20589 1cg24475062 TNXB 0.151805 0.304922 4.02E-15 0.497848 -2.00864 0 down vs 1 -8.47293 71.7906 0.425075 1.25526 1cg02475474 MOG -0.02007 0.242522 4.03E-15 -0.0827553 -12.0838 0 down vs 1 -8.47261 71.7852 1.25021 3.69218 1cg25555059 SLC27A6 -0.06832 0.219151 4.07E-15 -0.311748 -3.20771 0 down vs 1 -8.47132 71.7632 1.49833 4.42629 1cg16697438 -0.185485 0.145157 4.08E-15 -1.27782 1.27782 0 up vs 1 -8.4709 71.7561 1.98214 5.85614 1cg11384084 0.163135 0.329264 4.08E-15 0.495453 -2.01836 0 down vs 1 -8.47086 71.7555 0.500393 1.4784 1cg00185189 AKR1B10 0.28886 0.407823 4.08E-15 0.708297 -1.41184 0 down vs 1 -8.47071 71.753 0.256592 0.758122 1cg04913118 PSMB9 0.178195 0.384442 4.10E-15 0.463516 -2.15742 0 down vs 1 -8.47009 71.7425 0.771249 2.27905 1cg05698078 0.04858 0.289893 4.10E-15 0.167579 -5.96733 0 down vs 1 -8.46993 71.7397 1.05579 3.12 1cg05192145 0.083455 0.411205 4.12E-15 0.202952 -4.92727 0 down vs 1 -8.46922 71.7277 1.94762 5.75642 1cg13215579 0.047535 0.402173 4.12E-15 0.118195 -8.46056 0 down vs 1 -8.46917 71.7268 2.28028 6.73973 1cg16914425 EPHB6 0.227315 0.382914 4.13E-15 0.593645 -1.68451 0 down vs 1 -8.46907 71.7252 0.438966 1.29746 1cg01041222 GRIA2 -0.384335 -0.0434974 4.15E-15 8.83581 8.83581 0 up vs 1 -8.46823 71.711 2.10626 6.22677 1cg03018058 RHBDD1 -0.08604 -0.386893 4.17E-15 0.222387 -4.49667 0 down vs 1 8.4674 71.6969 1.64107 4.85247 1cg01138171 ARHGEF4 0.168685 0.37626 4.23E-15 0.44832 -2.23055 0 down vs 1 -8.46516 71.659 0.781213 2.31118 1cg22070855 GHSR -0.1916 0.143179 4.25E-15 -1.33819 1.33819 0 up vs 1 -8.46434 71.645 2.03205 6.0129 1cg14670315 FAM24B 0.203545 0.403263 4.26E-15 0.504745 -1.9812 0 down vs 1 -8.46415 71.6419 0.723189 2.14003 1cg04002454 SP140L -0.37136 -0.452567 4.26E-15 0.820564 -1.21867 0 down vs 1 8.46403 71.6398 0.119565 0.353824 1cg15470261 0.229465 0.456259 4.27E-15 0.502927 -1.98836 0 down vs 1 -8.46378 71.6356 0.932572 2.75987 1cg27235662 CLDN16 0.01438 0.286705 4.28E-15 0.0501561 -19.9378 0 down vs 1 -8.46351 71.631 1.3446 3.9795 1cg16505953 PLIN3 0.329555 0.452144 4.29E-15 0.728871 -1.37198 0 down vs 1 -8.46282 71.6193 0.272473 0.806546 1cg13401823 NFASC -0.06122 0.222315 4.31E-15 -0.275374 -3.63142 0 down vs 1 -8.46241 71.6123 1.45758 4.315 1cg12737588 0.33205 0.473328 4.31E-15 0.701521 -1.42547 0 down vs 1 -8.46238 71.6119 0.361884 1.07132 1cg22891003 PDGFA 0.20414 0.388948 4.31E-15 0.524852 -1.9053 0 down vs 1 -8.46237 71.6118 0.61924 1.8332 1cg23840027 EPB41 0.198695 0.374779 4.34E-15 0.530165 -1.8862 0 down vs 1 -8.46112 71.5905 0.56216 1.66471 1cg14621763 PTPRD 0.081335 0.335503 4.34E-15 0.242427 -4.12495 0 down vs 1 -8.4611 71.5902 1.17128 3.4685 1cg13407144 NRP1 0.149745 0.389741 4.37E-15 0.384217 -2.6027 0 down vs 1 -8.46019 71.5748 1.0443 3.09315 1cg08087868 -0.180785 0.0921 4.37E-15 -1.96292 1.96292 0 up vs 1 -8.46008 71.5729 1.35014 3.99912 1cg25537993 ZSCAN1 -0.13162 0.201279 4.39E-15 -0.653919 -1.52924 0 down vs 1 -8.4594 71.5614 2.00929 5.9525 1cg05857996 EBF4 0.2777 0.455366 4.39E-15 0.609839 -1.63978 0 down vs 1 -8.45923 71.5586 0.572304 1.69551 1cg12212620 0.162025 0.371276 4.43E-15 0.4364 -2.29148 0 down vs 1 -8.45788 71.5357 0.793879 2.3527 1cg20346189 CITED1 -0.145505 0.157137 4.44E-15 -0.925978 -1.07994 0 down vs 1 -8.4577 71.5327 1.66064 4.9216 1cg17274072 ZNF774 0.304985 0.463109 4.46E-15 0.658559 -1.51847 0 down vs 1 -8.457 71.5208 0.453331 1.34375 1cg04385765 0.15299 0.395766 4.48E-15 0.386566 -2.58688 0 down vs 1 -8.45631 71.5091 1.06864 3.16815 1cg10729426 ZNF549 -0.338385 0.163434 4.48E-15 -2.07048 2.07048 0 up vs 1 -8.45611 71.5058 4.56574 13.5365 1cg07993743 WNT9B -0.12602 0.10738 4.49E-15 -1.17358 1.17358 0 up vs 1 -8.45585 71.5013 0.987691 2.92848 1cg11844358 NFATC1 0.355695 0.485509 4.50E-15 0.732622 -1.36496 0 down vs 1 -8.45556 71.4965 0.305536 0.90597 1cg00964997 -0.17582 0.127128 4.51E-15 -1.38302 1.38302 0 up vs 1 -8.45526 71.4915 1.664 4.93441 1cg22692118 0.049855 0.317053 4.51E-15 0.157245 -6.35949 0 down vs 1 -8.45516 71.4898 1.29444 3.83862 1cg20569893 FLOT2 0.103655 -0.197834 4.52E-15 -0.523951 -1.90858 0 down vs 1 8.4548 71.4836 1.64801 4.88753 1cg02390241 ADAMTSL5 0.26913 0.470273 4.53E-15 0.572284 -1.74738 0 down vs 1 -8.45458 71.4799 0.733548 2.17561 1cg07803710 0.062915 0.262622 4.53E-15 0.239565 -4.17424 0 down vs 1 -8.45455 71.4793 0.723111 2.14467 1cg21659431 0.112665 0.371934 4.56E-15 0.302917 -3.30123 0 down vs 1 -8.45346 71.461 1.21876 3.61563 1cg12557133 ZNF311 0.217415 0.409116 4.56E-15 0.531426 -1.88173 0 down vs 1 -8.45329 71.4582 0.666299 1.97675 1cg20248204 KCTD12 -0.325205 0.0278366 4.60E-15 -11.6826 11.6826 0 up vs 1 -8.45215 71.4389 2.2598 6.70612 1cg21675115 EDNRB -0.23299 0.0928876 4.60E-15 -2.5083 2.5083 0 up vs 1 -8.45212 71.4384 1.92543 5.71388 1cg12039490 NIPAL2 0.2582 0.388739 4.60E-15 0.664199 -1.50557 0 down vs 1 -8.45196 71.4356 0.308956 0.916891 1cg14443953 PAX6 -0.341125 0.0502211 4.63E-15 -6.79246 6.79246 0 up vs 1 -8.451 71.4194 2.77677 8.24251 1cg24428653 ITGB1BP3 0.04343 0.399741 4.64E-15 0.108645 -9.20425 0 down vs 1 -8.45078 71.4156 2.30184 6.83311 1cg12754722 CUX2 -0.00958 0.184437 4.66E-15 -0.0519418 -19.2523 0 down vs 1 -8.44999 71.4023 0.682493 2.02638 1cg13769046 HRNBP3 0.295465 0.440569 4.68E-15 0.670644 -1.4911 0 down vs 1 -8.44953 71.3946 0.381748 1.13357 1cg12412598 0.18356 0.363907 4.69E-15 0.504414 -1.9825 0 down vs 1 -8.44908 71.3869 0.589708 1.75127 1cg02049472 MIR1246 0.214705 0.403762 4.71E-15 0.531762 -1.88054 0 down vs 1 -8.45151 71.4279 0.618528 1.82715 1cg26180255 CELSR1 0.23978 0.410828 4.73E-15 0.583651 -1.71335 0 down vs 1 -8.4477 71.3637 0.530461 1.57584 1cg26949612 OSBPL6 0.076005 0.373662 4.74E-15 0.203406 -4.91629 0 down vs 1 -8.4474 71.3586 1.60639 4.77244 1cg17267654 0.132885 0.402631 4.75E-15 0.330041 -3.02992 0 down vs 1 -8.4472 71.3551 1.31926 3.91958 1cg20187173 0.122335 0.359128 4.76E-15 0.340644 -2.93561 0 down vs 1 -8.44665 71.3459 1.01662 3.02081 1cg00242098 KPRP -0.022455 0.282881 4.76E-15 -0.0793797 -12.5977 0 down vs 1 -8.44662 71.3453 1.69034 5.02278 1cg26295626 AP1S3 0.300745 0.426899 4.78E-15 0.704488 -1.41947 0 down vs 1 -8.44611 71.3367 0.288549 0.857516 1cg21144871 0.00611 0.299455 4.81E-15 0.0204037 -49.0107 0 down vs 1 -8.44507 71.3192 1.56018 4.63773 1cg03140788 UMOD -0.217025 0.0234835 4.82E-15 -9.24159 9.24159 0 up vs 1 -8.44464 71.312 1.04877 3.11783 1cg15258754 0.15364 0.351707 4.83E-15 0.436841 -2.28916 0 down vs 1 -8.44446 71.3089 0.711284 2.11464 1cg24478695 BTNL2 0.238155 0.399194 4.83E-15 0.596589 -1.6762 0 down vs 1 -8.44438 71.3075 0.470199 1.39792 1cg14944901 0.028655 0.334466 4.84E-15 0.0856738 -11.6722 0 down vs 1 -8.44404 71.3018 1.69561 5.04152 1cg02316156 -0.043395 0.183638 4.84E-15 -0.236307 -4.23178 0 down vs 1 -8.44403 71.3016 0.934537 2.77864 1cg08052629 PPP1R14A -0.064115 0.189009 4.85E-15 -0.339216 -2.94797 0 down vs 1 -8.44399 71.301 1.16168 3.45403 1cg12432010 IQSEC3 -0.328545 -0.00970515 4.85E-15 33.8526 33.8526 0 up vs 1 -8.44391 71.2996 1.84316 5.4804 1cg14941600 PLCH2 0.03633 0.280893 4.86E-15 0.129337 -7.73172 0 down vs 1 -8.44346 71.2921 1.08443 3.22474 1cg07867133 0.315805 0.473202 4.89E-15 0.667379 -1.4984 0 down vs 1 -8.44247 71.2754 0.44917 1.336 1cg16908208 TMEM132C -0.011975 0.302585 4.90E-15 -0.0395756 -25.2681 0 down vs 1 -8.44548 71.3261 1.79315 5.30457 1cg26039623 RBM47 0.06287 0.332313 4.90E-15 0.189189 -5.28571 0 down vs 1 -8.44231 71.2726 1.31629 3.91529 1cg06322310 PLCH2 -0.129375 0.136303 4.94E-15 -0.949171 -1.05355 0 down vs 1 -8.441 71.2504 1.27976 3.80783 1cg00703843 RAI1 0.297675 0.00280979 4.94E-15 105.942 105.942 0 up vs 1 8.44092 71.2491 1.57639 4.69052 1cg21086940 -0.17486 0.119553 4.94E-15 -1.46261 1.46261 0 up vs 1 -8.44085 71.2479 1.57156 4.67623 1cg15003139 0.266515 0.424247 4.95E-15 0.628207 -1.59183 0 down vs 1 -8.44063 71.2442 0.451084 1.34228 1cg19679327 -0.036515 0.272672 4.96E-15 -0.133915 -7.4674 0 down vs 1 -8.4404 71.2404 1.73325 5.15787 1cg12717612 0.02586 0.25388 4.99E-15 0.101859 -9.81747 0 down vs 1 -8.43954 71.2258 0.942678 2.80583 1cg12086850 0.28005 0.436035 4.99E-15 0.642265 -1.55699 0 down vs 1 -8.43948 71.2249 0.441148 1.31307 1cg16651552 SLC9A3 -0.04343 0.18781 5.03E-15 -0.231244 -4.32444 0 down vs 1 -8.43807 71.201 0.969494 2.88665 1cg06428620 PCDHGA4 -0.18244 0.228097 5.04E-15 -0.799834 -1.25026 0 down vs 1 -8.43774 71.1955 3.05579 9.09927 1cg07884222 0.160315 0.362288 5.04E-15 0.442508 -2.25985 0 down vs 1 -8.43773 71.1952 0.73961 2.20236 1cg22269291 STX18 0.260065 0.368446 5.08E-15 0.705842 -1.41675 0 down vs 1 -8.43646 71.1738 0.212974 0.634371 1cg12457773 NRSN1 -0.22475 0.0771711 5.09E-15 -2.91236 2.91236 0 up vs 1 -8.43614 71.1685 1.65274 4.92326 1cg03483626 KCNA3 -0.05088 0.242257 5.14E-15 -0.210025 -4.76134 0 down vs 1 -8.43481 71.146 1.55797 4.64243 1cg18292904 MST1R 0.41484 -0.0382845 5.15E-15 -10.8357 10.8357 0 up vs 1 8.43431 71.1376 3.72266 11.094 1cg08719486 DAPK1 -0.042415 0.294134 5.18E-15 -0.144203 -6.93466 0 down vs 1 -8.43362 71.1259 2.05359 6.12098 1cg27104173 PTPRN2 0.00444 0.395781 5.18E-15 0.0112183 -89.1398 0 down vs 1 -8.4334 71.1222 2.7767 8.27673 1cg06954535 PGLYRP4 -0.071465 0.213849 5.19E-15 -0.334184 -2.99236 0 down vs 1 -8.43317 71.1184 1.47592 4.39965 1cg02734001 TCF12 0.13736 0.386308 5.21E-15 0.355571 -2.81238 0 down vs 1 -8.43253 71.1076 1.12366 3.35009 1cg18391978 0.243455 0.449799 5.22E-15 0.541253 -1.84757 0 down vs 1 -8.43243 71.1058 0.771972 2.30161 1cg11508406 0.150035 0.425268 5.26E-15 0.352801 -2.83446 0 down vs 1 -8.43103 71.0824 1.37347 4.09631 1cg22720392 PRDM16 0.079395 0.389396 5.31E-15 0.203892 -4.90455 0 down vs 1 -8.42975 71.0607 1.74239 5.19818 1cg22389121 ST20 0.39054 0.445293 5.32E-15 0.877041 -1.1402 0 down vs 1 -8.42952 71.0568 0.0543539 0.162166 1cg12454814 -0.291565 0.014267 5.32E-15 -20.4363 20.4363 0 up vs 1 -8.42938 71.0545 1.69584 5.05974 1cg22702025 PGLYRP3 0.14755 0.333283 5.32E-15 0.442717 -2.25878 0 down vs 1 -8.42935 71.054 0.625455 1.86614 1cg12359279 MX1 -0.376415 -0.456253 5.35E-15 0.825014 -1.2121 0 down vs 1 8.42862 71.0417 0.115568 0.344874 1cg15728909 CDH19 0.0174421 0.286613 5.36E-15 0.0608558 -16.4323 0 down vs 1 -8.43434 71.1381 1.25324 3.69956 1cg19598416 CAPN2 -0.115055 0.202082 5.40E-15 -0.569347 -1.7564 0 down vs 1 -8.4271 71.016 1.82353 5.44368 1cg00741731 LYPD4 0.21395 0.352386 5.40E-15 0.607147 -1.64705 0 down vs 1 -8.42703 71.0148 0.347469 1.0373 1cg02100373 PITX1 -0.087315 -0.356331 5.44E-15 0.245039 -4.08098 0 down vs 1 8.42587 70.9953 1.31212 3.91814 1cg02403395 FGF12 -0.335585 0.150899 5.44E-15 -2.22391 2.22391 0 up vs 1 -8.4258 70.9941 4.29097 12.8135 1cg06701529 -0.015665 0.250239 5.47E-15 -0.0626002 -15.9744 0 down vs 1 -8.42501 70.9808 1.28194 3.82879 1cg02637843 -0.005665 0.277002 5.47E-15 -0.0204511 -48.897 0 down vs 1 -8.425 70.9806 1.44866 4.32676 1cg02801405 0.16951 0.3743 5.49E-15 0.452872 -2.20813 0 down vs 1 -8.42433 70.9693 0.760388 2.27144 1cg23239396 CALCR -0.336075 -0.0207918 5.50E-15 16.1639 16.1639 0 up vs 1 -8.42413 70.966 1.80227 5.38401 1cg22678044 -0.02636 0.242966 5.53E-15 -0.108493 -9.21722 0 down vs 1 -8.42336 70.9529 1.31515 3.92952 1cg10880902 -0.14731 -0.366607 5.53E-15 0.40182 -2.48867 0 down vs 1 8.42329 70.9517 0.871931 2.60528 1cg25926572 INPP5D 0.07779 0.318551 5.54E-15 0.2442 -4.09501 0 down vs 1 -8.42303 70.9474 1.05097 3.14042 1cg19262637 0.040385 0.316089 5.55E-15 0.127765 -7.82688 0 down vs 1 -8.42288 70.945 1.37817 4.11829 1cg26230320 0.126525 0.364365 5.55E-15 0.347248 -2.87979 0 down vs 1 -8.42276 70.9429 1.02563 3.0649 1cg14536812 0.207705 0.436198 5.58E-15 0.476171 -2.10009 0 down vs 1 -8.42197 70.9295 0.946598 2.82927 1cg14598619 KIAA1751 0.03827 0.342944 5.58E-15 0.111592 -8.96118 0 down vs 1 -8.42194 70.9291 1.68302 5.03038 1cg14396421 -0.33481 0.0785686 5.59E-15 -4.26137 4.26137 0 up vs 1 -8.42153 70.9222 3.09823 9.2612 1cg08529825 HIPK1 0.04076 0.290985 5.61E-15 0.140076 -7.13899 0 down vs 1 -8.42118 70.9163 1.13522 3.39367 1cg26705561 SEC31B 0.07739 0.311975 5.62E-15 0.248065 -4.03121 0 down vs 1 -8.42091 70.9117 0.997745 2.98289 1cg09038971 CHST11 0.18442 0.381806 5.68E-15 0.48302 -2.07031 0 down vs 1 -8.41918 70.8826 0.706401 2.11275 1cg26348521 0.144565 0.359468 5.68E-15 0.402164 -2.48655 0 down vs 1 -8.41914 70.8819 0.837342 2.5044 1cg03087203 WDR37 0.260575 0.382762 5.71E-15 0.680775 -1.46891 0 down vs 1 -8.41822 70.8664 0.270689 0.809778 1cg00004996 MTMR7 0.17938 0.393682 5.72E-15 0.455646 -2.19468 0 down vs 1 -8.41792 70.8613 0.832669 2.49115 1cg00162806 VENTX 0.073355 0.299648 5.75E-15 0.244804 -4.0849 0 down vs 1 -8.41734 70.8516 0.928453 2.77809 1cg17406445 0.199625 0.362949 5.75E-15 0.550008 -1.81815 0 down vs 1 -8.41717 70.8488 0.483635 1.44718 1cg16935061 FOXF2 0.00399 0.240418 5.77E-15 0.0165961 -60.2551 0 down vs 1 -8.41673 70.8413 1.01348 3.03295 1cg21724451 MGAT5B 0.14435 0.356839 5.77E-15 0.404525 -2.47204 0 down vs 1 -8.41657 70.8387 0.818633 2.44994 1cg17104700 THEG -0.126015 0.149621 5.80E-15 -0.84223 -1.18732 0 down vs 1 -8.41597 70.8285 1.37749 4.12303 1cg20814158 0.18717 0.408904 5.81E-15 0.457736 -2.18466 0 down vs 1 -8.41549 70.8205 0.891417 2.66844 1cg04427498 -0.1487 0.209636 5.82E-15 -0.709324 -1.40979 0 down vs 1 -8.41522 70.8159 2.32809 6.96954 1cg15063150 0.325895 0.448231 5.83E-15 0.727069 -1.37538 0 down vs 1 -8.41502 70.8125 0.271348 0.812367 1cg09692335 TREX1 -0.384465 -0.471649 5.84E-15 0.815151 -1.22677 0 down vs 1 8.4148 70.8088 0.137813 0.41261 1cg04650238 0.17394 0.368023 5.85E-15 0.472633 -2.11581 0 down vs 1 -8.41443 70.8027 0.682958 2.04494 1cg00509108 MT1A -0.17369 0.139807 5.86E-15 -1.24236 1.24236 0 up vs 1 -8.41429 70.8002 1.7819 5.33563 1cg24734735 LOC154822 0.284595 0.401904 5.88E-15 0.708118 -1.41219 0 down vs 1 -8.41379 70.7919 0.249504 0.747189 1cg16961816 DLX6AS -0.092135 0.205119 5.89E-15 -0.449178 -2.22629 0 down vs 1 -8.41348 70.7866 1.60204 4.79798 1cg04698650 CCDC135 0.244615 0.435852 5.90E-15 0.561234 -1.78179 0 down vs 1 -8.4131 70.7803 0.663074 1.98603 1cg17641046 MLH1 -0.26735 -0.453055 5.91E-15 0.590105 -1.69461 0 down vs 1 8.41303 70.7791 0.625268 1.87282 1cg23025804 SVIL 0.144535 0.361105 5.91E-15 0.400258 -2.49839 0 down vs 1 -8.41295 70.7777 0.85038 2.54714 1cg14801518 -0.010635 0.297533 5.95E-15 -0.0357439 -27.9768 0 down vs 1 -8.41201 70.7619 1.72184 5.15857 1cg13189149 -0.156935 0.18859 5.96E-15 -0.832148 -1.20171 0 down vs 1 -8.41171 70.7569 2.1646 6.48552 1cg06097727 C1QTNF7 0.27285 0.406602 5.96E-15 0.67105 -1.4902 0 down vs 1 -8.4117 70.7568 0.324351 0.971815 1cg27396346 IGF1R 0.306135 0.421956 5.97E-15 0.725515 -1.37833 0 down vs 1 -8.41146 70.7527 0.243215 0.728758 1cg25000308 EPSTI1 0.307855 0.423965 5.98E-15 0.726132 -1.37716 0 down vs 1 -8.41097 70.7444 0.244433 0.732495 1cg22756386 -0.052035 0.253725 5.99E-15 -0.205084 -4.87604 0 down vs 1 -8.41094 70.744 1.69503 5.07955 1cg18504014 ZBTB38 0.31552 0.423681 6.00E-15 0.744711 -1.3428 0 down vs 1 -8.4107 70.7398 0.212109 0.635668 1cg00875511 TACR3 -0.17295 0.125397 6.01E-15 -1.37922 1.37922 0 up vs 1 -8.41031 70.7334 1.61384 4.83696 1cg25952615 GNAL 0.213105 0.411058 6.02E-15 0.51843 -1.9289 0 down vs 1 -8.41009 70.7297 0.710466 2.1295 1cg01706334 0.12778 0.428701 6.04E-15 0.298064 -3.35499 0 down vs 1 -8.40958 70.7211 1.6418 4.92163 1cg06179228 C1QTNF7 0.02231 0.321814 6.05E-15 0.0693258 -14.4246 0 down vs 1 -8.40938 70.7177 1.62638 4.87563 1cg24005949 CSMD2 0.175625 0.311819 6.06E-15 0.563227 -1.77548 0 down vs 1 -8.40907 70.7125 0.336306 1.00826 1cg16248554 GLI2 0.128955 0.376397 6.07E-15 0.342604 -2.91882 0 down vs 1 -8.40874 70.7069 1.11011 3.32842 1cg22025206 SLC9A3 -0.19577 0.166145 6.08E-15 -1.17831 1.17831 0 up vs 1 -8.40862 70.7048 2.37482 7.12062 1cg17187595 MYT1L 0.234855 0.455662 6.10E-15 0.515415 -1.94019 0 down vs 1 -8.40802 70.6949 0.883985 2.6509 1cg20941855 TAGLN3 -0.18007 0.212135 6.11E-15 -0.848848 -1.17807 0 down vs 1 -8.40786 70.6922 2.78897 8.36388 1cg13075444 C11orf41 0.20314 0.362974 6.12E-15 0.559655 -1.78682 0 down vs 1 -8.40757 70.6872 0.463185 1.38915 1cg01033642 CMIP 0.184865 -0.139277 6.14E-15 -1.32732 1.32732 0 up vs 1 8.40692 70.6762 1.90498 5.71415 1cg13610545 PRRX2 -0.0117 0.191111 6.15E-15 -0.0612209 -16.3343 0 down vs 1 -8.40676 70.6735 0.745766 2.23708 1cg13913015 KCNK12 -0.33787 0.138991 6.17E-15 -2.43087 2.43087 0 up vs 1 -8.4062 70.6642 4.12289 12.3691 1cg07592809 0.148175 0.439973 6.18E-15 0.336782 -2.96928 0 down vs 1 -8.40584 70.6582 1.54377 4.63187 1cg06591973 KLHL14 -0.090345 0.223222 6.19E-15 -0.404731 -2.47077 0 down vs 1 -8.40568 70.6554 1.7827 5.34896 1cg04842426 CHRM2 -0.277205 0.0329 6.21E-15 -8.42568 8.42568 0 up vs 1 -8.40515 70.6465 1.74355 5.23216 1cg25282972 0.227425 0.394819 6.22E-15 0.576023 -1.73604 0 down vs 1 -8.40496 70.6433 0.50804 1.52462 1cg22302985 FAM155A -0.32463 0.10451 6.22E-15 -3.1062 3.1062 0 up vs 1 -8.40489 70.6422 3.339 10.0205 1cg12502265 EBF3 0.002975 0.342347 6.23E-15 0.00869002 -115.075 0 down vs 1 -8.40477 70.6401 2.08819 6.26692 1cg10140391 IQSEC1 0.28232 0.361122 6.25E-15 0.781785 -1.27912 0 down vs 1 -8.40422 70.6309 0.112589 0.337937 1cg26190890 HIST1H2BK -0.062755 -0.386737 6.29E-15 0.162268 -6.16265 0 down vs 1 8.40329 70.6153 1.90309 5.71343 1cg14705934 CUZD1 0.075075 0.321012 6.29E-15 0.23387 -4.27588 0 down vs 1 -8.40329 70.6153 1.09664 3.29232 1cg03104428 OLFM3 -0.16788 0.205045 6.37E-15 -0.818748 -1.22138 0 down vs 1 -8.40126 70.5812 2.52151 7.57369 1cg22404125 TSPAN5 0.165785 0.336726 6.38E-15 0.492344 -2.0311 0 down vs 1 -8.40087 70.5746 0.5298 1.59147 1cg05352087 LHX8 -0.264715 0.0942629 6.39E-15 -2.80826 2.80826 0 up vs 1 -8.40062 70.5704 2.33643 7.01886 1cg12111808 GABBR1 0.25371 0.423353 6.40E-15 0.599288 -1.66865 0 down vs 1 -8.40047 70.5679 0.52178 1.56753 1cg19515484 -0.11776 0.159415 6.41E-15 -0.738701 -1.35373 0 down vs 1 -8.40012 70.5621 1.39292 4.18495 1cg26820378 PEX14 0.321 0.433796 6.43E-15 0.739978 -1.35139 0 down vs 1 -8.39964 70.554 0.230679 0.693143 1cg22980306 CHRNA4 0.17845 0.386295 6.48E-15 0.461953 -2.16472 0 down vs 1 -8.39851 70.535 0.783243 2.35412 1cg00076538 EGFLAM -0.314945 0.0438799 6.51E-15 -7.17743 7.17743 0 up vs 1 -8.39774 70.5221 2.33444 7.01769 1cg15062725 ZFR2 0.10138 0.309028 6.55E-15 0.328061 -3.04821 0 down vs 1 -8.3968 70.5062 0.781759 2.35061 1cg16753400 0.072145 0.357628 6.61E-15 0.201732 -4.95708 0 down vs 1 -8.39553 70.4848 1.47768 4.44447 1cg00995520 KCNA3 0.11469 0.329345 6.65E-15 0.348236 -2.87161 0 down vs 1 -8.39442 70.4663 0.835413 2.51337 1cg00833777 ITGAM -0.085065 0.159832 6.67E-15 -0.532213 -1.87895 0 down vs 1 -8.39412 70.4613 1.08739 3.27169 1cg12573791 0.002245 0.244641 6.68E-15 0.0091767 -108.972 0 down vs 1 -8.39385 70.4567 1.06529 3.20541 1cg01795199 FGFR3 0.348155 0.482631 6.68E-15 0.721369 -1.38625 0 down vs 1 -8.3937 70.4542 0.327874 0.986589 1cg14189583 DEDD2 0.057885 -0.237798 6.71E-15 -0.24342 -4.10812 0 down vs 1 8.39308 70.4438 1.58516 4.77051 1cg18279254 CRYBG3 0.26196 0.405581 6.73E-15 0.645888 -1.54826 0 down vs 1 -8.3927 70.4374 0.373987 1.12561 1cg01419136 -0.08622 0.236119 6.73E-15 -0.365155 -2.73856 0 down vs 1 -8.39261 70.4358 1.88384 5.67004 1cg15445725 0.09107 0.31064 6.75E-15 0.293169 -3.411 0 down vs 1 -8.39205 70.4266 0.874103 2.63125 1cg02303399 LELP1 0.103695 0.369199 6.75E-15 0.280864 -3.56044 0 down vs 1 -8.39205 70.4265 1.27809 3.84735 1cg09698471 HTR1A -0.16438 0.0975598 6.76E-15 -1.68492 1.68492 0 up vs 1 -8.39195 70.4247 1.244 3.74482 1cg15688628 0.065855 0.318739 6.77E-15 0.206611 -4.84001 0 down vs 1 -8.39177 70.4218 1.15947 3.49051 1cg22805485 SPON1 0.237235 0.435259 6.77E-15 0.545044 -1.83472 0 down vs 1 -8.39172 70.4209 0.710972 2.14036 1cg26798688 0.21101 0.387989 6.80E-15 0.543855 -1.83872 0 down vs 1 -8.39108 70.4101 0.567887 1.70987 1cg21977377 NKX2-2 -0.255275 0.100867 6.81E-15 -2.53081 2.53081 0 up vs 1 -8.39068 70.4036 2.29966 6.92477 1cg10206873 ELFN1 0.14306 0.398192 6.81E-15 0.359274 -2.78339 0 down vs 1 -8.39066 70.4032 1.18018 3.55379 1cg24638647 TMEM101 -0.08829 -0.41637 6.86E-15 0.212047 -4.71593 0 down vs 1 8.38964 70.386 1.95154 5.87795 1cg14827832 -0.11291 0.238802 6.88E-15 -0.472819 -2.11497 0 down vs 1 -8.38917 70.3781 2.2428 6.75599 1cg26053480 -0.010005 0.252235 6.89E-15 -0.0396654 -25.2109 0 down vs 1 -8.38882 70.3723 1.24686 3.75621 1cg20125424 ZNF701 -0.244365 0.15241 6.90E-15 -1.60334 1.60334 0 up vs 1 -8.38878 70.3716 2.85435 8.59895 1cg04710402 RUNDC3B -0.418375 0.082032 6.92E-15 -5.10015 5.10015 0 up vs 1 -8.38832 70.3639 4.54009 13.6789 1cg21091239 0.264105 0.407707 6.92E-15 0.647782 -1.54373 0 down vs 1 -8.38829 70.3634 0.373884 1.12649 1cg19811761 ZNF471 -0.33393 0.0464119 7.03E-15 -7.19493 7.19493 0 up vs 1 -8.38571 70.3202 2.62281 7.90719 1cg09461545 SEL1L3 0.08659 -0.182748 7.03E-15 -0.473821 -2.1105 0 down vs 1 8.38569 70.3199 1.31527 3.96527 1cg05442477 OLIG1 0.044485 0.299834 7.08E-15 0.148365 -6.74012 0 down vs 1 -8.38474 70.3038 1.18219 3.56487 1cg01601746 ZNF549 -0.41341 -0.0132284 7.08E-15 31.2518 31.2518 0 up vs 1 -8.3846 70.3015 2.90357 8.75596 1cg04243033 ST7 0.27368 0.395316 7.13E-15 0.692307 -1.44445 0 down vs 1 -8.38355 70.284 0.268251 0.809136 1cg26973607 -0.103475 0.175932 7.17E-15 -0.588152 -1.70024 0 down vs 1 -8.38273 70.2702 1.41545 4.2703 1cg11289677 TNXB 0.173715 0.343202 7.22E-15 0.50616 -1.97566 0 down vs 1 -8.38167 70.2525 0.520821 1.57168 1cg03614132 -0.040925 0.275814 7.30E-15 -0.148379 -6.7395 0 down vs 1 -8.37994 70.2234 1.81895 5.4913 1cg25089357 EBF3 0.13933 0.355343 7.32E-15 0.3921 -2.55037 0 down vs 1 -8.38237 70.2641 0.845605 2.53931 1cg03192598 -0.067835 0.246973 7.34E-15 -0.274665 -3.64079 0 down vs 1 -8.37893 70.2064 1.79684 5.42587 1cg13534503 0.265035 0.452104 7.37E-15 0.586226 -1.70583 0 down vs 1 -8.37837 70.1971 0.634486 1.91619 1cg08730348 PYCARD -0.150855 -0.37751 7.41E-15 0.399606 -2.50247 0 down vs 1 8.37748 70.1822 0.931425 2.81356 1cg15082014 -0.168555 0.222521 7.42E-15 -0.757479 -1.32017 0 down vs 1 -8.37725 70.1783 2.77294 8.37672 1cg14528310 LCE1E 0.194355 0.391126 7.43E-15 0.496911 -2.01243 0 down vs 1 -8.37712 70.1762 0.702007 2.12074 1cg15817769 GALNT2 0.187835 0.373146 7.43E-15 0.503382 -1.98656 0 down vs 1 -8.37712 70.1761 0.622619 1.88091 1cg27249422 DHX58 -0.391615 -0.481675 7.44E-15 0.813027 -1.22997 0 down vs 1 8.37691 70.1727 0.147057 0.444275 1cg01270228 ADARB2 0.3121 0.450832 7.45E-15 0.692275 -1.44451 0 down vs 1 -8.37672 70.1695 0.348959 1.05429 1cg06234863 MSI2 0.19516 0.402574 7.46E-15 0.48478 -2.06279 0 down vs 1 -8.37653 70.1662 0.780001 2.35669 1cg13069211 0.0047 0.175692 7.46E-15 0.0267514 -37.3812 0 down vs 1 -8.37644 70.1648 0.530113 1.60171 1cg16294838 DOCK2 0.17049 0.380979 7.52E-15 0.447505 -2.23461 0 down vs 1 -8.37518 70.1436 0.803301 2.42787 1cg07528216 NR3C1 0.237175 0.428802 7.55E-15 0.553111 -1.80796 0 down vs 1 -8.37456 70.1333 0.665781 2.01253 1cg18787437 -0.056385 -0.264986 7.58E-15 0.212785 -4.69958 0 down vs 1 8.37394 70.1228 0.788953 2.38522 1cg20263156 SCARA5 -0.235435 0.0884649 7.58E-15 -2.66134 2.66134 0 up vs 1 -8.3739 70.1222 1.90213 5.75069 1cg25763212 IL20 0.310475 0.423986 7.60E-15 0.732276 -1.3656 0 down vs 1 -8.37364 70.1178 0.233612 0.706321 1cg17215117 MPP7 0.272455 0.426239 7.61E-15 0.639207 -1.56444 0 down vs 1 -8.37331 70.1124 0.428787 1.29653 1cg07002201 FUT9 -0.191665 0.109302 7.62E-15 -1.75354 1.75354 0 up vs 1 -8.37311 70.109 1.64231 4.96613 1cg20123637 TF 0.259515 0.412481 7.68E-15 0.629156 -1.58943 0 down vs 1 -8.37189 70.0886 0.424239 1.28321 1cg06220761 -0.06322 0.29483 7.68E-15 -0.214429 -4.66355 0 down vs 1 -8.37184 70.0876 2.32437 7.03072 1cg08667600 RBMS3 0.25759 0.420349 7.69E-15 0.6128 -1.63185 0 down vs 1 -8.37173 70.0859 0.480298 1.45283 1cg05770446 0.12175 0.376452 7.69E-15 0.323414 -3.09201 0 down vs 1 -8.37167 70.0849 1.1762 3.5579 1cg23125901 DIP2C 0.23509 0.379681 7.77E-15 0.619177 -1.61505 0 down vs 1 -8.3701 70.0585 0.379056 1.14704 1cg16189217 PCCA 0.14731 0.376176 7.77E-15 0.391598 -2.55364 0 down vs 1 -8.37004 70.0575 0.949689 2.87384 1cg24530787 PCDH11X -0.2395 0.0812418 7.78E-15 -2.94799 2.94799 0 up vs 1 -8.36995 70.056 1.86522 5.64442 1cg05596294 LPPR4 -0.3258 0.0687701 7.86E-15 -4.73752 4.73752 0 up vs 1 -8.36835 70.0293 2.82271 8.5452 1cg16093173 KCNH5 0.325565 0.42196 7.87E-15 0.771554 -1.29609 0 down vs 1 -8.36805 70.0242 0.168473 0.510055 1cg25960893 SOX2OT -0.257715 0.0350067 7.88E-15 -7.36188 7.36188 0 up vs 1 -8.36797 70.023 1.55356 4.70352 1cg11479165 RBPMS2 -0.260025 0.016466 7.94E-15 -15.7917 15.7917 0 up vs 1 -8.36673 70.0022 1.38605 4.19763 1cg10185853 0.200815 0.41111 7.96E-15 0.48847 -2.04721 0 down vs 1 -8.36624 69.9939 0.801817 2.42857 1cg18472159 0.07842 0.387785 8.00E-15 0.202225 -4.94498 0 down vs 1 -8.36551 69.9818 1.73524 5.25668 1cg08934319 ROBO1 0.11394 0.369826 8.01E-15 0.308091 -3.2458 0 down vs 1 -8.36526 69.9776 1.18717 3.59657 1cg15182643 EGFLAM -0.325875 -0.0115 8.01E-15 28.337 28.337 0 up vs 1 -8.36525 69.9774 1.7919 5.42865 1cg13006360 -0.085155 0.277212 8.06E-15 -0.307184 -3.25538 0 down vs 1 -8.36425 69.9606 2.38076 7.21435 1cg00717279 FAM20C 0.17222 0.34141 8.14E-15 0.504437 -1.98241 0 down vs 1 -8.36271 69.9349 0.519002 1.5733 1cg24487554 0.04761 0.300407 8.15E-15 0.158485 -6.30975 0 down vs 1 -8.36261 69.9332 1.15868 3.51249 1cg08701621 ZNF135 -0.04715 0.284848 8.18E-15 -0.165527 -6.04132 0 down vs 1 -8.36195 69.9222 1.99844 6.05913 1cg00500229 -0.249675 0.0931134 8.21E-15 -2.68141 2.68141 0 up vs 1 -8.36144 69.9136 2.13044 6.46018 1cg08609445 0.18684 0.456362 8.23E-15 0.409411 -2.44253 0 down vs 1 -8.36108 69.9077 1.31707 3.99409 1cg19976363 ADARB2 -0.02323 0.283479 8.24E-15 -0.081946 -12.2032 0 down vs 1 -8.36096 69.9056 1.70558 5.17245 1cg10331779 CTNND2 -0.193735 0.0868851 8.30E-15 -2.22979 2.22979 0 up vs 1 -8.35979 69.8861 1.42776 4.33112 1cg02078943 -0.246375 0.0660278 8.32E-15 -3.73138 3.73138 0 up vs 1 -8.35938 69.8792 1.76949 5.36829 1cg03593833 -0.335685 -0.024034 8.34E-15 13.9671 13.9671 0 up vs 1 -8.35907 69.874 1.76098 5.34288 1cg16157895 ITPK1 0.309325 0.0238361 8.34E-15 12.9772 12.9772 0 up vs 1 8.35895 69.8721 1.47773 4.48362 1cg17459215 -0.11971 0.324419 8.36E-15 -0.368998 -2.71004 0 down vs 1 -8.35863 69.8666 3.57632 10.8518 1cg00679763 UBE2Z 0.17559 -0.215026 8.36E-15 -0.816598 -1.22459 0 down vs 1 8.35858 69.8658 2.76642 8.3944 1cg26622320 EDNRB -0.18727 0.0760165 8.38E-15 -2.46354 2.46354 0 up vs 1 -8.35831 69.8614 1.25683 3.81394 1cg00449067 CALCRL -0.083415 0.240352 8.40E-15 -0.347054 -2.88139 0 down vs 1 -8.35794 69.8552 1.90056 5.76792 1cg10164640 HPDL -0.342655 -0.458769 8.40E-15 0.746901 -1.33887 0 down vs 1 8.35777 69.8524 0.244449 0.741895 1cg13545297 HOXC8 0.108445 0.35138 8.42E-15 0.308626 -3.24017 0 down vs 1 -8.35744 69.8468 1.07003 3.24779 1cg20739281 PLCH2 0.178415 0.33716 8.43E-15 0.52917 -1.88975 0 down vs 1 -8.35734 69.8452 0.456899 1.38682 1cg05280478 RECQL5 0.101625 0.327769 8.46E-15 0.310051 -3.22527 0 down vs 1 -8.35684 69.8367 0.927228 2.81474 1cg02892388 -0.21808 0.128924 8.48E-15 -1.69154 1.69154 0 up vs 1 -8.35633 69.8282 2.18317 6.62815 1cg06120688 GRB10 0.25806 0.403413 8.51E-15 0.639692 -1.56325 0 down vs 1 -8.3559 69.8211 0.383059 1.16309 1cg12527478 0.016145 0.270425 8.51E-15 0.0597024 -16.7498 0 down vs 1 -8.35576 69.8187 1.17231 3.55964 1cg00989986 0.09871 0.342011 8.52E-15 0.288617 -3.4648 0 down vs 1 -8.35563 69.8165 1.07326 3.25899 1cg22716633 PPP2R2C 0.23267 0.374735 8.52E-15 0.620893 -1.61058 0 down vs 1 -8.35559 69.8158 0.365923 1.11115 1cg12210890 KCNV1 -0.31946 0.101219 8.53E-15 -3.15614 3.15614 0 up vs 1 -8.3555 69.8145 3.20862 9.74337 1cg26677322 -0.02169 0.282433 8.56E-15 -0.076797 -13.0213 0 down vs 1 -8.35481 69.8029 1.67694 5.09306 1cg11539780 ZNF471 -0.346215 0.0633521 8.58E-15 -5.46494 5.46494 0 up vs 1 -8.35462 69.7996 3.04136 9.23742 1cg18242103 PTGDR -0.04086 0.19512 8.58E-15 -0.209409 -4.77533 0 down vs 1 -8.3546 69.7994 1.00965 3.06657 1cg17693604 TLE2 -0.00693 0.245649 8.59E-15 -0.0282109 -35.4473 0 down vs 1 -8.35444 69.7967 1.15668 3.5133 1cg11214544 -0.034615 0.191658 8.59E-15 -0.180608 -5.53684 0 down vs 1 -8.35435 69.7952 0.928287 2.81963 1cg19759804 LDLRAP1 -0.251655 -0.374157 8.63E-15 0.672592 -1.48679 0 down vs 1 8.3537 69.7843 0.272086 0.826578 1cg27076669 CPM -0.044185 -0.354501 8.64E-15 0.12464 -8.02311 0 down vs 1 8.35343 69.7798 1.74593 5.30435 1cg10371560 ITGAM 0.20345 0.367894 8.67E-15 0.553012 -1.80828 0 down vs 1 -8.3529 69.7709 0.490293 1.48976 1cg08637618 -0.290085 0.0962536 8.70E-15 -3.01376 3.01376 0 up vs 1 -8.35241 69.7628 2.70616 8.22368 1cg26901714 THEG 0.11432 0.314234 8.71E-15 0.363806 -2.74872 0 down vs 1 -8.35218 69.7589 0.724606 2.20211 1cg00967201 RELL1 0.149525 0.372103 8.73E-15 0.401838 -2.48856 0 down vs 1 -8.35177 69.752 0.898216 2.72998 1cg06770532 GLI3 -0.064125 0.174863 8.74E-15 -0.366716 -2.72691 0 down vs 1 -8.35168 69.7506 1.03555 3.14744 1cg03080147 MTMR7 0.137815 0.321094 8.80E-15 0.429205 -2.32989 0 down vs 1 -8.35061 69.7327 0.609035 1.85158 1cg16353174 0.241925 0.4519 8.84E-15 0.535351 -1.86793 0 down vs 1 -8.34982 69.7195 0.79938 2.43072 1cg10106639 0.013155 0.306456 8.86E-15 0.0429263 -23.2958 0 down vs 1 -8.34942 69.7128 1.55971 4.74316 1cg07054292 CYP27C1 0.228835 0.385105 8.90E-15 0.594214 -1.68289 0 down vs 1 -8.34881 69.7026 0.442762 1.34666 1cg23808270 DISP1 0.166145 0.356369 8.92E-15 0.466216 -2.14493 0 down vs 1 -8.34851 69.6976 0.656068 1.99557 1cg23680821 C1orf59 -0.321205 -0.442273 8.95E-15 0.726259 -1.37692 0 down vs 1 8.34788 69.6872 0.265753 0.808465 1cg14914552 PTDSS1 0.014575 0.341856 8.98E-15 0.042635 -23.4549 0 down vs 1 -8.34734 69.678 1.94204 5.90879 1cg05355757 0.141985 0.360327 9.01E-15 0.394045 -2.53778 0 down vs 1 -8.34679 69.6688 0.864354 2.6302 1cg13537510 MOG 0.08058 0.296608 9.05E-15 0.271671 -3.68092 0 down vs 1 -8.34618 69.6587 0.846134 2.57513 1cg02834447 0.1457 0.296406 9.05E-15 0.491556 -2.03436 0 down vs 1 -8.34609 69.6573 0.411791 1.25327 1cg25604883 AK5 -0.34682 -0.0463036 9.08E-15 7.49013 7.49013 0 up vs 1 -8.34562 69.6493 1.6374 4.98395 1cg15999067 MAGI2 0.11928 0.360921 9.14E-15 0.330488 -3.02583 0 down vs 1 -8.34454 69.6314 1.05867 3.22323 1cg26646527 0.09118 0.361918 9.15E-15 0.251936 -3.96926 0 down vs 1 -8.34447 69.6302 1.32897 4.04625 1cg10848640 -0.169015 0.16624 9.20E-15 -1.01669 1.01669 0 up vs 1 -8.34363 69.6161 2.03783 6.20574 1cg09229668 CDH23 0.022985 0.276243 9.24E-15 0.0832056 -12.0184 0 down vs 1 -8.34284 69.603 1.16291 3.54204 1cg09937500 0.146415 0.35208 9.31E-15 0.415857 -2.40467 0 down vs 1 -8.34163 69.5828 0.766903 2.33655 1cg05371922 -0.202625 -0.0558655 9.34E-15 3.62702 3.62702 0 up vs 1 -8.34117 69.5752 0.390509 1.1899 1cg13847066 TMEM101 -0.10238 -0.419434 9.36E-15 0.244091 -4.09684 0 down vs 1 8.34095 69.5714 1.82257 5.55379 1cg09658497 GNA12 0.166735 0.424236 9.38E-15 0.393025 -2.54437 0 down vs 1 -8.34051 69.5641 1.20219 3.66375 1cg09851545 -0.233415 0.0810644 9.42E-15 -2.87938 2.87938 0 up vs 1 -8.33995 69.5547 1.79309 5.46527 1cg18771300 RHOJ -0.040455 0.203949 9.47E-15 -0.198358 -5.04139 0 down vs 1 -8.33911 69.5407 1.08302 3.30167 1cg03048372 NCOR2 0.319655 0.47203 9.50E-15 0.677192 -1.47669 0 down vs 1 -8.33848 69.5302 0.420964 1.28353 1cg16495016 0.258335 0.367579 9.51E-15 0.702801 -1.42288 0 down vs 1 -8.3383 69.5273 0.21638 0.659776 1cg27261665 HYDIN -0.03013 0.243801 9.54E-15 -0.123585 -8.09162 0 down vs 1 -8.33789 69.5203 1.3605 4.1488 1cg15703970 ELFN1 -0.08775 0.323794 9.57E-15 -0.271005 -3.68996 0 down vs 1 -8.33736 69.5115 3.0708 9.36548 1cg25657187 LOC645323 -0.32 0.0521557 9.66E-15 -6.13548 6.13548 0 up vs 1 -8.33594 69.4878 2.51112 7.66116 1cg07639489 NBLA00301 -0.233805 0.00768093 9.67E-15 -30.4397 30.4397 0 up vs 1 -8.33583 69.486 1.05731 3.22582 1cg07369950 ZSCAN18 -0.30544 0.0137454 9.70E-15 -22.2213 22.2213 0 up vs 1 -8.33531 69.4774 1.84716 5.63633 1cg13420320 -0.02156 0.27727 9.74E-15 -0.0777583 -12.8604 0 down vs 1 -8.33456 69.4649 1.61907 4.94123 1cg00685984 WIPI1 0.319525 0.438278 9.74E-15 0.729047 -1.37165 0 down vs 1 -8.33456 69.4649 0.255685 0.780327 1cg08378830 -0.30586 0.0525567 9.81E-15 -5.81962 5.81962 0 up vs 1 -8.33348 69.4469 2.32913 7.11013 1cg22273168 10-Mar 0.152105 0.285053 9.81E-15 0.533603 -1.87405 0 down vs 1 -8.33347 69.4467 0.320464 0.978279 1cg14776998 TRIM71 -0.15484 0.226978 9.91E-15 -0.68218 -1.46589 0 down vs 1 -8.33186 69.4199 2.64321 8.07204 1cg24867601 SRPK2 0.35099 0.421834 9.98E-15 0.832057 -1.20184 0 down vs 1 -8.33085 69.4031 0.0909964 0.277959 1cg07508480 0.26228 0.421848 9.98E-15 0.621741 -1.60839 0 down vs 1 -8.33083 69.4027 0.461646 1.41016 1cg04460847 -0.14312 0.0788892 1.00E-14 -1.81419 1.81419 0 up vs 1 -8.33004 69.3896 0.893634 2.73024 1cg20998885 C20orf79 0.21671 0.412718 1.02E-14 0.52508 -1.90447 0 down vs 1 -8.3272 69.3423 0.696572 2.12962 1cg15971980 0.16473 0.371896 1.03E-14 0.442947 -2.25761 0 down vs 1 -8.3265 69.3306 0.778134 2.37939 1cg10523144 0.12879 0.331742 1.03E-14 0.388224 -2.57583 0 down vs 1 -8.32641 69.3291 0.746799 2.28362 1cg07085815 SERPINE2 0.15559 0.385598 1.03E-14 0.403503 -2.4783 0 down vs 1 -8.32598 69.322 0.959188 2.93338 1cg02324249 0.07449 0.341736 1.03E-14 0.217976 -4.58767 0 down vs 1 -8.32514 69.3079 1.29491 3.96088 1cg03744763 HOXA5 0.037065 0.232557 1.04E-14 0.15938 -6.27429 0 down vs 1 -8.32457 69.2984 0.692906 2.11976 1cg03280622 PLEC1 -0.17166 -0.365771 1.04E-14 0.46931 -2.13079 0 down vs 1 8.3239 69.2873 0.683154 2.09026 1cg06937201 ESYT2 0.284695 0.43362 1.04E-14 0.656554 -1.5231 0 down vs 1 -8.3239 69.2873 0.402118 1.23037 1cg06313349 PTPRN2 -0.04246 0.294725 1.05E-14 -0.144067 -6.94123 0 down vs 1 -8.32265 69.2666 2.06136 6.30908 1cg12349884 HIST1H2BK -0.104455 -0.411021 1.06E-14 0.254136 -3.93491 0 down vs 1 8.32192 69.2544 1.70398 5.21619 1cg07508299 PDE10A 0.20825 0.393845 1.06E-14 0.528762 -1.89121 0 down vs 1 -8.32128 69.2438 0.624525 1.91207 1cg11547104 SORCS2 0.142035 0.416341 1.06E-14 0.34115 -2.93126 0 down vs 1 -8.32119 69.2422 1.36424 4.1769 1cg06689180 KCNE1 0.010655 0.319943 1.06E-14 0.0333028 -30.0275 0 down vs 1 -8.32087 69.237 1.73438 5.31059 1cg13571293 0.12033 0.334724 1.06E-14 0.359491 -2.78171 0 down vs 1 -8.32084 69.2363 0.833378 2.55178 1cg09184477 0.05047 0.264637 1.07E-14 0.190714 -5.24345 0 down vs 1 -8.32041 69.2293 0.831617 2.54665 1cg17152981 GPR6 -0.30448 -0.0256046 1.07E-14 11.8916 11.8916 0 up vs 1 -8.31996 69.2218 1.41006 4.31848 1cg23627076 GPR1 0.16078 0.381151 1.07E-14 0.421828 -2.37063 0 down vs 1 -8.31994 69.2215 0.880491 2.69662 1cg14550985 RIN1 -0.033245 -0.297535 1.07E-14 0.111735 -8.94977 0 down vs 1 8.31991 69.2209 1.26643 3.87863 1cg02703627 -0.129475 0.198406 1.07E-14 -0.652577 -1.53239 0 down vs 1 -8.31978 69.2187 1.94917 5.96983 1cg12170314 INSC -0.18798 0.197153 1.07E-14 -0.953472 -1.0488 0 down vs 1 -8.31976 69.2185 2.6893 8.23671 1cg12663535 0.08308 0.277485 1.07E-14 0.299403 -3.33998 0 down vs 1 -8.32241 69.2625 0.684896 2.08646 1cg02331348 PTPRN2 0.083575 0.317025 1.07E-14 0.263623 -3.7933 0 down vs 1 -8.31934 69.2114 0.988113 3.02667 1cg07611666 MBP 0.244935 0.443401 1.08E-14 0.552401 -1.81028 0 down vs 1 -8.31849 69.1973 0.714148 2.18794 1cg01863081 0.008465 0.296647 1.09E-14 0.0285356 -35.0439 0 down vs 1 -8.3176 69.1824 1.50574 4.61415 1cg22993667 GPR123 0.076445 0.395645 1.09E-14 0.193216 -5.17555 0 down vs 1 -8.31674 69.1682 1.84732 5.66204 1cg02133098 C2CD4C 0.11267 0.34992 1.09E-14 0.321988 -3.10571 0 down vs 1 -8.31624 69.1598 1.02054 3.12833 1cg12489896 0.14877 0.369152 1.10E-14 0.403005 -2.48136 0 down vs 1 -8.31577 69.1521 0.880583 2.69961 1cg03142364 OR10H2 0.13043 0.288868 1.10E-14 0.451521 -2.21474 0 down vs 1 -8.3153 69.1441 0.455131 1.39546 1cg01735621 C6orf150 -0.37031 -0.444865 1.10E-14 0.832409 -1.20133 0 down vs 1 8.31519 69.1424 0.100781 0.309007 1cg17752089 PGLYRP2 0.019475 0.228687 1.11E-14 0.08516 -11.7426 0 down vs 1 -8.31469 69.1341 0.793582 2.43352 1cg26364947 TMEM132D -0.163975 0.263662 1.11E-14 -0.621914 -1.60794 0 down vs 1 -8.3143 69.1275 3.31565 10.1684 1cg00030774 FAM189A1 -0.323965 -0.0187448 1.11E-14 17.2829 17.2829 0 up vs 1 -8.31376 69.1186 1.68906 5.18066 1cg07338584 KCNU1 0.17758 0.365953 1.11E-14 0.485253 -2.06078 0 down vs 1 -8.31346 69.1136 0.643362 1.97346 1cg05432034 0.00336 0.285725 1.12E-14 0.0117595 -85.0373 0 down vs 1 -8.31327 69.1105 1.44557 4.43437 1cg04334121 SAA4 0.133955 0.364537 1.13E-14 0.367467 -2.72134 0 down vs 1 -8.31193 69.0881 0.963978 2.95801 1cg03562911 CLYBL 0.21579 0.407807 1.13E-14 0.529147 -1.88983 0 down vs 1 -8.31192 69.088 0.668495 2.05131 1cg13085403 -0.097445 0.23993 1.13E-14 -0.406139 -2.46221 0 down vs 1 -8.31127 69.0772 2.06369 6.33351 1cg04203742 0.022095 0.306848 1.14E-14 0.0720062 -13.8877 0 down vs 1 -8.30989 69.0543 1.47013 4.51337 1cg22161476 FSTL5 -0.302475 0.0653907 1.14E-14 -4.62566 4.62566 0 up vs 1 -8.30923 69.0433 2.45356 7.53375 1cg19922137 SYT14 -0.228035 0.0776314 1.15E-14 -2.93741 2.93741 0 up vs 1 -8.30917 69.0422 1.694 5.20157 1cg08857994 -0.186355 0.0901938 1.15E-14 -2.06616 2.06616 0 up vs 1 -8.30906 69.0405 1.38663 4.25788 1cg19194373 EDN3 -0.284655 0.0802113 1.15E-14 -3.54881 3.54881 0 up vs 1 -8.30895 69.0386 2.41371 7.4119 1cg15364618 LTB4R2 -0.01475 -0.277149 1.15E-14 0.0532204 -18.7898 0 down vs 1 8.30856 69.0322 1.24837 3.83379 1cg19513834 POU3F3 -0.206505 0.212162 1.15E-14 -0.973335 -1.0274 0 down vs 1 -8.3082 69.0262 3.17802 9.76064 1cg26978776 CUZD1 0.171785 0.36864 1.15E-14 0.465997 -2.14594 0 down vs 1 -8.3079 69.0212 0.702602 2.15806 1cg06754664 OR12D2 0.220545 0.39731 1.16E-14 0.555096 -1.80149 0 down vs 1 -8.307 69.0063 0.566512 1.74043 1cg13870494 MAMDC2 -0.07301 0.26921 1.16E-14 -0.271201 -3.6873 0 down vs 1 -8.30692 69.005 2.12338 6.52355 1cg24010336 FBXO17 -0.12212 0.183789 1.16E-14 -0.664457 -1.50499 0 down vs 1 -8.30675 69.0021 1.69669 5.21287 1cg25887811 SH3RF3 0.115015 0.285581 1.17E-14 0.40274 -2.48299 0 down vs 1 -8.30541 68.9799 0.527479 1.62113 1cg26694839 0.037405 0.332747 1.18E-14 0.112413 -8.89579 0 down vs 1 -8.30439 68.9628 1.5815 4.86171 1cg10542336 PTPRN2 0.331335 0.470581 1.18E-14 0.704098 -1.42026 0 down vs 1 -8.30438 68.9627 0.351547 1.0807 1cg24533097 PCDHB11 0.0115 0.296782 1.18E-14 0.038749 -25.8071 0 down vs 1 -8.30412 68.9585 1.47559 4.53643 1cg15498379 ZSCAN18 -0.277 0.0210273 1.19E-14 -13.1733 13.1733 0 up vs 1 -8.30293 68.9387 1.61039 4.95226 1cg19276014 0.15163 0.435389 1.19E-14 0.348264 -2.87139 0 down vs 1 -8.30276 68.9359 1.45988 4.48959 1cg19475870 CDH9 0.070605 0.319755 1.19E-14 0.22081 -4.52879 0 down vs 1 -8.30258 68.9329 1.12549 3.46138 1cg07657064 ZSCAN18 -0.341785 0.0476897 1.20E-14 -7.16685 7.16685 0 up vs 1 -8.30229 68.928 2.75028 8.45895 1cg25653223 -0.214355 0.143191 1.20E-14 -1.49699 1.49699 0 up vs 1 -8.30204 68.9238 2.31783 7.12931 1cg11530659 HLA-DQB2 0.222835 0.392543 1.20E-14 0.56767 -1.76159 0 down vs 1 -8.30186 68.9209 0.522185 1.60624 1cg24168924 BCL11B 0.1071 0.317442 1.20E-14 0.337385 -2.96398 0 down vs 1 -8.30174 68.9188 0.802175 2.46756 1cg23526194 0.071975 0.364257 1.20E-14 0.197594 -5.06089 0 down vs 1 -8.30136 68.9125 1.5489 4.76497 1cg01784063 MUC4 0.29692 0.427272 1.21E-14 0.694921 -1.43901 0 down vs 1 -8.30093 68.9054 0.308071 0.947837 1cg11596090 0.09363 0.400105 1.21E-14 0.234014 -4.27325 0 down vs 1 -8.30056 68.8993 1.70297 5.23996 1cg08647091 GLIS2 0.07342 0.237884 1.21E-14 0.308638 -3.24004 0 down vs 1 -8.3004 68.8966 0.490407 1.50902 1cg21152690 LIN7A -0.27486 0.185141 1.21E-14 -1.4846 1.4846 0 up vs 1 -8.30026 68.8944 3.8365 11.8056 1cg12973315 GPR78 0.05348 0.293208 1.21E-14 0.182396 -5.48257 0 down vs 1 -8.30025 68.8942 1.04197 3.20633 1cg03612683 PSG11 0.00549 0.123306 1.22E-14 0.0445235 -22.46 0 down vs 1 -8.29922 68.8771 0.251666 0.774614 1cg10420746 GLUD2 0.11316 0.325852 1.22E-14 0.347275 -2.87956 0 down vs 1 -8.29911 68.8752 0.820197 2.52459 1cg24871414 -0.175765 0.152182 1.23E-14 -1.15497 1.15497 0 up vs 1 -8.29815 68.8593 1.94996 6.00341 1cg10164367 ITGBL1 0.00877 0.311984 1.23E-14 0.0281104 -35.574 0 down vs 1 -8.29755 68.8494 1.66693 5.13277 1cg14199150 BRD3 -0.18724 -0.376786 1.23E-14 0.49694 -2.01232 0 down vs 1 8.29733 68.8456 0.6514 2.00589 1cg02526981 EBF3 -0.010875 0.317565 1.24E-14 -0.034245 -29.2014 0 down vs 1 -8.29725 68.8444 1.95582 6.02278 1cg06454760 ZNF135 0.02076 0.330394 1.26E-14 0.0628341 -15.9149 0 down vs 1 -8.29417 68.7932 1.73826 5.35679 1cg02279071 MLH1 -0.303165 -0.468069 1.27E-14 0.647693 -1.54394 0 down vs 1 8.29245 68.7648 0.493038 1.52002 1cg27526098 OR11A1 0.05823 0.353613 1.27E-14 0.164671 -6.0727 0 down vs 1 -8.29244 68.7645 1.58194 4.8771 1cg01129958 CSGALNACT1 0.25817 0.419533 1.27E-14 0.615375 -1.62503 0 down vs 1 -8.29237 68.7634 0.472091 1.45547 1cg07656173 PROM1 -0.051285 0.276672 1.28E-14 -0.185364 -5.39479 0 down vs 1 -8.29224 68.7612 1.95007 6.01234 1cg20361154 ZNF280B -0.3771 -0.0426335 1.28E-14 8.84516 8.84516 0 up vs 1 -8.29211 68.759 2.02826 6.25359 1cg25143162 PTPRN2 0.011215 0.303984 1.28E-14 0.0368934 -27.1051 0 down vs 1 -8.29188 68.7553 1.55406 4.79177 1cg01159576 LMX1B -0.209245 0.0305567 1.29E-14 -6.84776 6.84776 0 up vs 1 -8.29094 68.7397 1.04261 3.21551 1cg04359915 CPN1 -0.08417 0.134375 1.29E-14 -0.626383 -1.59647 0 down vs 1 -8.29027 68.7287 0.865962 2.67114 1cg26009192 SCTR -0.009005 0.281036 1.29E-14 -0.0320422 -31.2088 0 down vs 1 -8.28999 68.7239 1.52523 4.70504 1cg08698936 HLA-F -0.017325 0.303074 1.30E-14 -0.0571643 -17.4934 0 down vs 1 -8.28966 68.7184 1.86123 5.74199 1cg12505442 ZNF311 0.18427 0.366202 1.30E-14 0.503192 -1.98731 0 down vs 1 -8.28896 68.7068 0.600118 1.85171 1cg20418725 CASP8 -0.320075 -0.450108 1.30E-14 0.711107 -1.40626 0 down vs 1 8.2888 68.7043 0.306568 0.945973 1cg19673329 HOPX 0.110545 0.34217 1.30E-14 0.323071 -3.0953 0 down vs 1 -8.28868 68.7022 0.972719 3.0016 1cg05769153 ZNF415 -0.15612 0.199175 1.31E-14 -0.783832 -1.27578 0 down vs 1 -8.28851 68.6993 2.28874 7.06285 1cg07653946 GREB1L -0.2972 0.0658206 1.31E-14 -4.5153 4.5153 0 up vs 1 -8.28785 68.6885 2.38935 7.3745 1cg05932360 JARID2 0.27068 0.393369 1.31E-14 0.688108 -1.45326 0 down vs 1 -8.28753 68.6831 0.272914 0.842388 1cg15901783 KCTD12 -0.14993 0.201153 1.32E-14 -0.745355 -1.34164 0 down vs 1 -8.2867 68.6694 2.23479 6.89937 1cg11728747 CPVL 0.058885 0.328345 1.33E-14 0.179339 -5.57604 0 down vs 1 -8.28545 68.6488 1.31646 4.06547 1cg22895083 SDK2 0.03274 0.280632 1.35E-14 0.116665 -8.57153 0 down vs 1 -8.2833 68.6131 1.11415 3.44248 1cg08790440 CNTNAP5 -0.175405 0.0875923 1.35E-14 -2.00252 2.00252 0 up vs 1 -8.28328 68.6128 1.25407 3.87482 1cg18846140 HSPA1A -0.262175 -0.471118 1.36E-14 0.556495 -1.79696 0 down vs 1 8.28266 68.6025 0.791542 2.44607 1cg24429310 CNTNAP2 -0.095975 0.204053 1.36E-14 -0.470344 -2.1261 0 down vs 1 -8.28541 68.6481 1.63129 5.01401 1cg21996068 UMOD -0.147885 0.137051 1.36E-14 -1.07905 1.07905 0 up vs 1 -8.28243 68.5987 1.47202 4.54918 1cg06768939 BAIAP2 0.18768 -0.0744227 1.36E-14 -2.52181 2.52181 0 up vs 1 8.28214 68.5939 1.24555 3.84956 1cg18282791 -0.26998 0.150576 1.36E-14 -1.79298 1.79298 0 up vs 1 -8.28167 68.586 3.20676 9.91212 1cg00668937 0.054125 0.254398 1.37E-14 0.212757 -4.70019 0 down vs 1 -8.28136 68.5809 0.727214 2.24799 1cg02309355 KIAA0513 0.225855 0.406742 1.37E-14 0.555279 -1.8009 0 down vs 1 -8.28065 68.5692 0.593241 1.83416 1cg16380414 0.033775 0.247841 1.37E-14 0.136277 -7.33801 0 down vs 1 -8.28056 68.5677 0.830834 2.5688 1cg15272186 LOC285830 0.218185 0.368708 1.38E-14 0.591755 -1.68989 0 down vs 1 -8.27976 68.5544 0.410795 1.27036 1cg21183256 -0.080725 0.170095 1.38E-14 -0.474587 -2.1071 0 down vs 1 -8.27939 68.5482 1.14063 3.52763 1cg20569034 LOC100130776 0.00042 0.297231 1.39E-14 0.00141304 -707.693 0 down vs 1 -8.279 68.5418 1.59727 4.94035 1cg20670084 0.258595 0.407349 1.39E-14 0.634824 -1.57524 0 down vs 1 -8.27822 68.5289 0.401195 1.24113 1cg26867284 RIMS1 -0.02003 0.255074 1.40E-14 -0.0785262 -12.7346 0 down vs 1 -8.27755 68.5179 1.37218 4.24565 1cg00891541 SMPD3 0.00397 0.337039 1.42E-14 0.011779 -84.8965 0 down vs 1 -8.27572 68.4875 2.01135 6.22603 1cg01062651 0.274855 0.40524 1.42E-14 0.678253 -1.47438 0 down vs 1 -8.27508 68.477 0.308227 0.954251 1cg19519630 TP73 0.17772 0.379882 1.42E-14 0.467829 -2.13753 0 down vs 1 -8.27507 68.4767 0.741001 2.2941 1cg23402467 0.14333 0.365729 1.42E-14 0.391902 -2.55166 0 down vs 1 -8.27482 68.4726 0.896774 2.77653 1cg13511000 ATOH1 -0.27159 0.0702345 1.43E-14 -3.8669 3.8669 0 up vs 1 -8.27456 68.4684 2.11848 6.55949 1cg06868132 RUFY1 0.047295 0.344748 1.43E-14 0.137187 -7.28931 0 down vs 1 -8.27421 68.4625 1.60419 4.9675 1cg22217793 0.07561 0.352752 1.43E-14 0.214343 -4.66541 0 down vs 1 -8.2741 68.4608 1.39258 4.31236 1cg13350140 RET 0.31494 0.465761 1.44E-14 0.676184 -1.47889 0 down vs 1 -8.27363 68.4529 0.412421 1.27728 1cg18298997 IGSF9B 0.13614 0.367382 1.44E-14 0.370568 -2.69856 0 down vs 1 -8.27293 68.4413 0.969508 3.00309 1cg01799671 CMIP -0.14981 -0.40726 1.45E-14 0.367849 -2.71851 0 down vs 1 8.27215 68.4285 1.20172 3.72308 1cg13272412 NRG1 0.078835 0.308842 1.45E-14 0.25526 -3.91758 0 down vs 1 -8.27172 68.4214 0.959182 2.97197 1cg20739526 DPP6 -0.301815 0.0639655 1.46E-14 -4.71841 4.71841 0 up vs 1 -8.27134 68.4151 2.42582 7.51697 1cg27052069 0.104735 0.368326 1.46E-14 0.284354 -3.51674 0 down vs 1 -8.2712 68.4128 1.25973 3.9037 1cg09205190 LOC728392 0.043695 0.320848 1.46E-14 0.136186 -7.34291 0 down vs 1 -8.27071 68.4047 1.3927 4.31627 1cg11354629 GSX1 -0.349005 0.0202691 1.46E-14 -17.2186 17.2186 0 up vs 1 -8.27062 68.4031 2.47238 7.66259 1cg02920604 MYO1E -0.003655 -0.270794 1.47E-14 0.0134974 -74.0886 0 down vs 1 8.27019 68.396 1.29387 4.01048 1cg03218797 -0.279895 0.116059 1.47E-14 -2.41166 2.41166 0 up vs 1 -8.26979 68.3894 2.84255 8.81161 1cg24059119 -0.11061 0.221401 1.48E-14 -0.499592 -2.00163 0 down vs 1 -8.30307 68.9409 1.98742 5.79439 1cg00950718 CCDC19 -0.20519 -0.394306 1.48E-14 0.520383 -1.92166 0 down vs 1 8.26918 68.3793 0.648445 2.01041 1cg22497068 -0.197455 0.126426 1.48E-14 -1.56182 1.56182 0 up vs 1 -8.26915 68.3788 1.90191 5.89663 1cg20707230 -0.01556 0.378394 1.48E-14 -0.0411212 -24.3184 0 down vs 1 -8.2689 68.3747 2.8139 8.72467 1cg06635764 GALP 0.287505 0.466449 1.48E-14 0.61637 -1.6224 0 down vs 1 -8.26885 68.3738 0.580567 1.80011 1cg20525229 CUX2 0.11102 0.423762 1.48E-14 0.261987 -3.81699 0 down vs 1 -8.26842 68.3668 1.77333 5.49896 1cg04642620 EBF3 0.100525 0.35703 1.48E-14 0.281559 -3.55165 0 down vs 1 -8.26829 68.3645 1.19291 3.69925 1cg14158073 -0.22279 0.0923294 1.49E-14 -2.41299 2.41299 0 up vs 1 -8.26815 68.3623 1.8004 5.58325 1cg00787856 CASZ1 -0.300955 -0.0213624 1.49E-14 14.0881 14.0881 0 up vs 1 -8.26805 68.3607 1.41732 4.3954 1cg08839186 FBLN2 0.12419 0.321989 1.49E-14 0.385696 -2.59271 0 down vs 1 -8.26793 68.3587 0.70936 2.19993 1cg10056132 C10orf53 -0.209575 0.0581722 1.49E-14 -3.60267 3.60267 0 up vs 1 -8.26753 68.3521 1.29977 4.03136 1cg25778262 CPM 0.00363 -0.293107 1.50E-14 -0.0123846 -80.7456 0 down vs 1 8.26616 68.3294 1.59647 4.95323 1cg01474741 PSG2 0.174055 0.343195 1.51E-14 0.50716 -1.97176 0 down vs 1 -8.26584 68.3241 0.518695 1.60944 1cg18913923 AOAH 0.19564 0.390077 1.52E-14 0.501542 -1.99385 0 down vs 1 -8.26489 68.3084 0.685449 2.12734 1cg23247879 SLC25A26 0.206405 0.384706 1.52E-14 0.536527 -1.86384 0 down vs 1 -8.26446 68.3013 0.5764 1.78908 1cg07710481 SLITRK5 -0.32627 0.111021 1.52E-14 -2.93881 2.93881 0 up vs 1 -8.26406 68.2947 3.46704 10.7624 1cg22208304 MOCOS 0.280925 -0.0255335 1.53E-14 -11.0022 11.0022 0 up vs 1 8.264 68.2937 1.70279 5.28587 1cg25777818 PTPRN2 0.192745 0.439274 1.53E-14 0.438781 -2.27904 0 down vs 1 -8.26365 68.2879 1.10193 3.42094 1cg24705551 NEFM 0.124005 0.367913 1.53E-14 0.337049 -2.96692 0 down vs 1 -8.26344 68.2845 1.07863 3.34877 1cg06198390 LCE2C 0.041765 0.271394 1.53E-14 0.153891 -6.49812 0 down vs 1 -8.26322 68.2808 0.956028 2.9683 1cg02665570 LYPLAL1 -0.264855 -0.453924 1.53E-14 0.583478 -1.71386 0 down vs 1 8.26306 68.2781 0.648126 2.0124 1cg12705693 TRIP13 0.077175 -0.193409 1.53E-14 -0.399024 -2.50611 0 down vs 1 8.26304 68.2779 1.32746 4.12172 1cg16758317 -0.003175 0.316021 1.54E-14 -0.0100468 -99.5342 0 down vs 1 -8.26278 68.2736 1.84728 5.7361 1cg23645261 ZNF536 -0.07412 0.221939 1.54E-14 -0.333965 -2.99432 0 down vs 1 -8.26255 68.2698 1.58919 4.93494 1cg24286414 FAM20C 0.305505 0.4269 1.54E-14 0.715636 -1.39736 0 down vs 1 -8.26254 68.2696 0.26719 0.829714 1cg27241808 MARCKS -0.25998 -0.455309 1.54E-14 0.570996 -1.75132 0 down vs 1 8.26247 68.2684 0.691756 2.14817 1cg10469774 -0.23786 0.0427005 1.54E-14 -5.57042 5.57042 0 up vs 1 -8.26243 68.2678 1.42715 4.43191 1cg14166485 LHX2 -0.2396 0.0532995 1.54E-14 -4.49535 4.49535 0 up vs 1 -8.2623 68.2655 1.55545 4.83047 1cg05109049 NF1 0.140005 -0.264173 1.55E-14 -0.529974 -1.88688 0 down vs 1 8.26166 68.2551 2.96185 9.19951 1cg19439043 0.18621 0.369499 1.55E-14 0.503952 -1.98432 0 down vs 1 -8.26162 68.2544 0.609106 1.8919 1cg25106304 0.04272 0.319497 1.55E-14 0.13371 -7.47886 0 down vs 1 -8.26144 68.2514 1.38892 4.31422 1cg11421768 TRIP13 0.23495 -0.178925 1.56E-14 -1.31312 1.31312 0 up vs 1 8.26083 68.2414 3.10567 9.64815 1cg01935942 0.0467 0.385627 1.56E-14 0.121102 -8.25753 0 down vs 1 -8.26078 68.2405 2.08271 6.47028 1cg25199254 -0.179485 0.146111 1.57E-14 -1.22842 1.22842 0 up vs 1 -8.2598 68.2243 1.9221 5.97272 1cg14627172 -0.25962 0.163572 1.58E-14 -1.58719 1.58719 0 up vs 1 -8.25886 68.2088 3.24708 10.0923 1cg09997714 PDE10A -0.05497 0.290072 1.58E-14 -0.189505 -5.27692 0 down vs 1 -8.25866 68.2054 2.15855 6.70933 1cg19162841 DGKK 0.059145 0.276821 1.59E-14 0.213658 -4.68038 0 down vs 1 -8.25791 68.1931 0.859092 2.67076 1cg19266396 ELFN1 0.13286 0.402732 1.59E-14 0.329896 -3.03125 0 down vs 1 -8.25741 68.1847 1.32049 4.10567 1cg07950084 ZNRF4 0.007515 0.254238 1.60E-14 0.0295589 -33.8308 0 down vs 1 -8.25601 68.1616 1.10367 3.43268 1cg20260721 -0.02489 0.234079 1.61E-14 -0.106332 -9.40453 0 down vs 1 -8.25515 68.1475 1.21594 3.78267 1cg15185479 HSPA1A -0.299985 -0.476723 1.62E-14 0.629265 -1.58916 0 down vs 1 8.25495 68.1443 0.566342 1.76192 1cg07054095 ZNF549 -0.395675 -0.0182629 1.63E-14 21.6655 21.6655 0 up vs 1 -8.25379 68.1251 2.58256 8.03672 1cg22411784 ZNF773 -0.411435 0.00852887 1.63E-14 -48.2403 48.2403 0 up vs 1 -8.25363 68.1224 3.19773 9.95149 1cg00727675 HOXA3 0.1372 0.362342 1.63E-14 0.378648 -2.64097 0 down vs 1 -8.2534 68.1186 0.919031 2.86022 1cg17596731 0.2385 0.422824 1.64E-14 0.564065 -1.77285 0 down vs 1 -8.25289 68.1102 0.616 1.91736 1cg03276149 -0.0132 0.305291 1.64E-14 -0.0432374 -23.1281 0 down vs 1 -8.25278 68.1084 1.83913 5.72463 1cg07797693 0.346055 0.477296 1.64E-14 0.725032 -1.37925 0 down vs 1 -8.25265 68.1062 0.312291 0.972095 1cg06912227 ADCY9 0.317665 0.481366 1.64E-14 0.659923 -1.51533 0 down vs 1 -8.25256 68.1048 0.485874 1.51245 1cg01720337 CNTN6 -0.130845 0.237987 1.65E-14 -0.549799 -1.81885 0 down vs 1 -8.25202 68.0959 2.46647 7.67875 1cg23685994 0.11303 0.359536 1.65E-14 0.314378 -3.18089 0 down vs 1 -8.25148 68.0869 1.10172 3.43039 1cg10721942 CCDC51 0.05987 -0.201619 1.65E-14 -0.296946 -3.36761 0 down vs 1 8.25114 68.0813 1.23972 3.86041 1cg14043803 0.006305 0.227799 1.66E-14 0.0276779 -36.1299 0 down vs 1 -8.25085 68.0766 0.889491 2.77 1cg20160695 0.169915 0.326038 1.67E-14 0.521151 -1.91883 0 down vs 1 -8.25015 68.065 0.441929 1.37646 1cg09658183 LLGL1 0.128985 -0.174679 1.67E-14 -0.73841 -1.35426 0 down vs 1 8.24981 68.0594 1.67188 5.20779 1cg21105875 KDM5B 0.04616 0.346743 1.67E-14 0.133125 -7.51176 0 down vs 1 -8.2644 68.3003 1.63408 4.95247 1cg24199987 SGMS1 -0.318905 -0.424636 1.67E-14 0.751008 -1.33154 0 down vs 1 8.2495 68.0542 0.202686 0.6314 1cg11413496 0.24958 0.437992 1.68E-14 0.569827 -1.75492 0 down vs 1 -8.2492 68.0493 0.64363 2.00516 1cg10669219 0.164145 0.309535 1.68E-14 0.530295 -1.88574 0 down vs 1 -8.24913 68.0481 0.383255 1.19401 1cg23553442 -0.28678 0.0738258 1.68E-14 -3.88455 3.88455 0 up vs 1 -8.2488 68.0427 2.35767 7.34577 1cg13701109 ADAMTS19 -0.37477 -0.110149 1.69E-14 3.40239 3.40239 0 up vs 1 -8.24825 68.0337 1.2696 3.9562 1cg07279070 EMX1 -0.15264 0.100436 1.70E-14 -1.51978 1.51978 0 up vs 1 -8.24668 68.0078 1.16123 3.61989 1cg20757073 GABBR1 0.20843 0.34289 1.71E-14 0.607863 -1.64511 0 down vs 1 -8.24619 67.9996 0.327795 1.02196 1cg26842775 OSTCL 0.123535 0.372341 1.72E-14 0.331779 -3.01405 0 down vs 1 -8.24461 67.9736 1.12238 3.50055 1cg26363196 ST6GALNAC3 -0.38048 -0.068816 1.73E-14 5.52895 5.52895 0 up vs 1 -8.24456 67.9728 1.76113 5.49278 1cg06548292 MBP 0.20257 0.379793 1.73E-14 0.533369 -1.87487 0 down vs 1 -8.24424 67.9674 0.569455 1.77621 1cg22698904 0.01983 0.321208 1.74E-14 0.0617357 -16.1981 0 down vs 1 -8.24363 67.9574 1.6468 5.13736 1cg02369450 0.11327 0.425821 1.74E-14 0.266004 -3.75934 0 down vs 1 -8.24358 67.9567 1.77116 5.52538 1cg17341326 ZFR2 0.241985 0.463315 1.74E-14 0.522291 -1.91464 0 down vs 1 -8.24358 67.9565 0.888175 2.77079 1cg14074389 0.190945 0.34957 1.75E-14 0.546229 -1.83074 0 down vs 1 -8.24825 68.0337 0.455761 1.4068 1cg01564665 -0.127485 0.186124 1.76E-14 -0.684948 -1.45997 0 down vs 1 -8.24129 67.9189 1.78318 5.56595 1cg07190535 PDE4D -0.381145 0.0269737 1.77E-14 -14.1302 14.1302 0 up vs 1 -8.24092 67.9127 3.01989 9.42705 1cg11362010 SOX2OT -0.06093 0.217848 1.77E-14 -0.279691 -3.57538 0 down vs 1 -8.2408 67.9108 1.40908 4.39878 1cg22360842 PID1 0.193865 0.387126 1.77E-14 0.50078 -1.99689 0 down vs 1 -8.24058 67.9072 0.677186 2.11411 1cg25248155 0.21191 0.350805 1.77E-14 0.604068 -1.65544 0 down vs 1 -8.24025 67.9017 0.349778 1.09206 1cg11313862 SYNE1 0.01628 0.313686 1.77E-14 0.051899 -19.2682 0 down vs 1 -8.24022 67.9012 1.60368 5.00698 1cg14392804 -0.260965 0.0581309 1.77E-14 -4.48926 4.48926 0 up vs 1 -8.24021 67.9011 1.84612 5.76394 1cg01616682 CALCB -0.19978 0.120541 1.78E-14 -1.65737 1.65737 0 up vs 1 -8.23988 67.8956 1.86032 5.80874 1cg10907866 TRAK1 0.01933 0.319311 1.78E-14 0.0605366 -16.5189 0 down vs 1 -8.23949 67.8893 1.63157 5.09495 1cg12094808 FRMD5 -0.140635 0.201715 1.79E-14 -0.697195 -1.43432 0 down vs 1 -8.23909 67.8826 2.125 6.63647 1cg03134083 TNR 0.11232 0.378211 1.79E-14 0.296977 -3.36727 0 down vs 1 -8.239 67.8811 1.28182 4.00326 1cg27165835 PAOX 0.154695 0.31238 1.79E-14 0.495214 -2.01933 0 down vs 1 -8.23887 67.8789 0.450815 1.40799 1cg00429107 -0.34989 0.00997835 1.79E-14 -35.0649 35.0649 0 up vs 1 -8.23882 67.8782 2.34804 7.33349 1cg17062829 -0.285695 0.114912 1.80E-14 -2.4862 2.4862 0 up vs 1 -8.23781 67.8615 2.90975 9.09009 1cg01266853 0.217295 0.397788 1.80E-14 0.546258 -1.83064 0 down vs 1 -8.23756 67.8574 0.590662 1.84535 1cg16764580 MLH1 -0.332075 -0.477594 1.80E-14 0.695308 -1.43821 0 down vs 1 8.23744 67.8555 0.383936 1.19953 1cg21560697 TAF1B 0.202265 -0.16813 1.81E-14 -1.20302 1.20302 0 up vs 1 8.23719 67.8513 2.48742 7.77189 1cg09837243 0.134675 0.372138 1.82E-14 0.361896 -2.76323 0 down vs 1 -8.2364 67.8382 1.02237 3.19499 1cg11339718 CBFA2T3 0.162335 0.407148 1.82E-14 0.398712 -2.50808 0 down vs 1 -8.23616 67.8343 1.08665 3.39606 1cg06486622 LOC285830 -0.145165 0.0785655 1.83E-14 -1.84769 1.84769 0 up vs 1 -8.23528 67.8198 0.907545 2.83692 1cg21302951 KCNH7 -0.24017 0.102232 1.83E-14 -2.34927 2.34927 0 up vs 1 -8.23517 67.818 2.12564 6.64479 1cg08978665 IL32 -0.05176 -0.257159 1.83E-14 0.201276 -4.96829 0 down vs 1 8.23506 67.8161 0.764916 2.3912 1cg03079698 FLJ35220 0.330525 0.462408 1.85E-14 0.714791 -1.39901 0 down vs 1 -8.23352 67.7908 0.315351 0.986187 1cg02948497 0.228835 0.428504 1.85E-14 0.534032 -1.87255 0 down vs 1 -8.23343 67.7894 0.722836 2.26055 1cg05986417 0.27563 0.461972 1.85E-14 0.596638 -1.67606 0 down vs 1 -8.23334 67.7879 0.629564 1.9689 1cg09139491 0.1917 0.385244 1.85E-14 0.497606 -2.00962 0 down vs 1 -8.23312 67.7843 0.679171 2.12415 1cg15019848 ZDHHC14 0.274275 0.393319 1.86E-14 0.697335 -1.43403 0 down vs 1 -8.23292 67.781 0.256941 0.80364 1cg18854392 SERPINI2 0.057165 0.329941 1.86E-14 0.173258 -5.77173 0 down vs 1 -8.23291 67.7808 1.34905 4.21947 1cg14135887 0.195175 0.367612 1.86E-14 0.530926 -1.8835 0 down vs 1 -8.2326 67.7756 0.539114 1.68633 1cg00007326 CACNA1A -0.017865 0.344401 1.87E-14 -0.0518727 -19.278 0 down vs 1 -8.23197 67.7653 2.37943 7.44392 1cg08457620 RTN4RL1 0.17471 0.359287 1.88E-14 0.486269 -2.05647 0 down vs 1 -8.23139 67.7558 0.617691 1.93268 1cg04897431 0.19393 0.368181 1.88E-14 0.526725 -1.89852 0 down vs 1 -8.23136 67.7552 0.550514 1.72251 1cg12974545 HRH1 0.13386 0.369626 1.88E-14 0.36215 -2.76129 0 down vs 1 -8.2312 67.7526 1.00782 3.15349 1cg12986236 0.23632 0.421957 1.89E-14 0.560057 -1.78553 0 down vs 1 -8.22989 67.731 0.62481 1.95567 1cg11629449 OR2H2 -0.093785 0.10584 1.90E-14 -0.886104 -1.12854 0 down vs 1 -8.22955 67.7256 0.722514 2.26167 1cg03612039 ZNF773 -0.38016 0.0821258 1.90E-14 -4.629 4.629 0 up vs 1 -8.22931 67.7215 3.87471 12.1296 1cg03432598 NFATC1 0.16511 0.379432 1.90E-14 0.43515 -2.29806 0 down vs 1 -8.22914 67.7187 0.83282 2.60722 1cg26671873 0.132585 0.390395 1.91E-14 0.339617 -2.94449 0 down vs 1 -8.22876 67.7124 1.20509 3.773 1cg05188150 TMX3 0.264295 0.400251 1.91E-14 0.660324 -1.51441 0 down vs 1 -8.22816 67.7026 0.335129 1.0494 1cg19759051 PRKCZ 0.251445 0.391836 1.92E-14 0.641711 -1.55834 0 down vs 1 -8.22796 67.6993 0.35735 1.11904 1cg05675331 0.032665 0.300091 1.92E-14 0.10885 -9.18694 0 down vs 1 -8.2279 67.6983 1.29666 4.06054 1cg02460314 CPN1 0.344715 0.479844 1.93E-14 0.718389 -1.392 0 down vs 1 -8.22693 67.6824 0.331068 1.037 1cg27252766 0.202135 0.372111 1.93E-14 0.543211 -1.84091 0 down vs 1 -8.22673 67.6792 0.523835 1.64088 1cg09987620 CACNB2 0.09632 0.400742 1.93E-14 0.240354 -4.16052 0 down vs 1 -8.22669 67.6785 1.68023 5.26326 1cg06017490 -0.01404 0.263962 1.93E-14 -0.0531894 -18.8007 0 down vs 1 -8.22662 67.6772 1.40125 4.38943 1cg20887073 -0.189575 0.0555026 1.94E-14 -3.41561 3.41561 0 up vs 1 -8.22597 67.6665 1.08899 3.41183 1cg21041202 0.25744 0.432276 1.95E-14 0.595545 -1.67913 0 down vs 1 -8.22525 67.6547 0.554219 1.73668 1cg21852117 -0.072955 0.171266 1.95E-14 -0.425975 -2.34756 0 down vs 1 -8.22487 67.6485 1.08139 3.38892 1cg11199639 ADAM5P 0.050665 0.330898 1.95E-14 0.153113 -6.53111 0 down vs 1 -8.22481 67.6476 1.42383 4.46212 1cg07591921 0.06675 0.352524 1.97E-14 0.189349 -5.28125 0 down vs 1 -8.224 67.6341 1.48068 4.64122 1cg25745795 ARX -0.086 0.203643 1.98E-14 -0.422307 -2.36795 0 down vs 1 -8.22295 67.617 1.52106 4.76897 1cg17206150 0.02476 0.321207 1.98E-14 0.0770842 -12.9728 0 down vs 1 -8.22289 67.616 1.59336 4.99573 1cg00184203 FAM171B -0.276665 0.108582 1.98E-14 -2.54798 2.54798 0 up vs 1 -8.22273 67.6132 2.69089 8.43724 1cg06153873 ADCY9 0.3303 0.476701 1.98E-14 0.692888 -1.44323 0 down vs 1 -8.2225 67.6095 0.3886 1.21852 1cg04496104 0.10146 0.388961 1.98E-14 0.260849 -3.83364 0 down vs 1 -8.22242 67.6083 1.49864 4.69931 1cg18674980 CA3 -0.078095 0.232804 1.99E-14 -0.335454 -2.98103 0 down vs 1 -8.22233 67.6067 1.75249 5.49543 1cg13685139 MITF 0.157125 0.349679 2.01E-14 0.449341 -2.22548 0 down vs 1 -8.22075 67.5808 0.672237 2.1088 1cg11795921 GALNT2 0.232035 0.38156 2.01E-14 0.608122 -1.64441 0 down vs 1 -8.22059 67.5781 0.405363 1.27167 1cg06610286 EBF3 0.0594 0.345965 2.02E-14 0.171693 -5.82433 0 down vs 1 -8.21998 67.5681 1.4889 4.67153 1cg21474104 0.15525 0.358556 2.02E-14 0.432987 -2.30954 0 down vs 1 -8.21968 67.5631 0.749406 2.35149 1cg01705562 0.10326 0.380191 2.03E-14 0.2716 -3.68188 0 down vs 1 -8.21881 67.5488 1.39047 4.36395 1cg04390523 EDNRB -0.24756 0.0996026 2.03E-14 -2.48548 2.48548 0 up vs 1 -8.21878 67.5483 2.18516 6.85812 1cg13367169 -0.325245 0.0682758 2.04E-14 -4.7637 4.7637 0 up vs 1 -8.21826 67.5399 2.80772 8.81311 1cg08335254 GRAMD1B -0.30946 -0.0365031 2.05E-14 8.47764 8.47764 0 up vs 1 -8.21734 67.5247 1.35085 4.24111 1cg15817287 IL7 0.14522 0.382366 2.06E-14 0.379793 -2.63302 0 down vs 1 -8.21667 67.5136 1.01965 3.20181 1cg26120971 0.09221 0.296931 2.07E-14 0.310543 -3.22017 0 down vs 1 -8.21598 67.5023 0.759879 2.3865 1cg11701868 UHRF1 -0.10539 -0.333958 2.07E-14 0.315578 -3.16879 0 down vs 1 8.22761 67.6935 0.945359 2.90478 1cg19489503 0.059685 0.341911 2.07E-14 0.174563 -5.7286 0 down vs 1 -8.21586 67.5003 1.44415 4.53569 1cg12608702 0.089855 0.334453 2.07E-14 0.268662 -3.72214 0 down vs 1 -8.21576 67.4988 1.08474 3.40694 1cg15489250 -0.349185 0.0743866 2.07E-14 -4.69419 4.69419 0 up vs 1 -8.21566 67.4971 3.25291 10.217 1cg03773809 0.20877 0.390727 2.07E-14 0.534311 -1.87157 0 down vs 1 -8.21551 67.4945 0.600284 1.88549 1cg03847535 MITF 0.220605 0.406305 2.07E-14 0.542954 -1.84178 0 down vs 1 -8.21546 67.4938 0.625234 1.96388 1cg13860360 STARD13 0.09937 0.345426 2.08E-14 0.287674 -3.47616 0 down vs 1 -8.2153 67.4912 1.09771 3.44807 1cg03314100 A2ML1 0.306735 0.432205 2.08E-14 0.709698 -1.40905 0 down vs 1 -8.21514 67.4885 0.285429 0.896613 1cg22768358 ZBTB16 0.016525 0.276819 2.08E-14 0.059696 -16.7515 0 down vs 1 -8.21496 67.4856 1.22842 3.85897 1cg02473540 ZNF135 -0.080135 0.152328 2.08E-14 -0.526069 -1.90089 0 down vs 1 -8.2147 67.4812 0.979772 3.07806 1cg21614682 ABLIM3 -0.049055 0.277042 2.08E-14 -0.177067 -5.64758 0 down vs 1 -8.21462 67.4801 1.92802 6.05721 1cg12446763 RNASEL 0.225875 0.40642 2.09E-14 0.555768 -1.79931 0 down vs 1 -8.2142 67.4731 0.590999 1.85691 1cg27343616 PCSK2 -0.312075 -0.0540881 2.09E-14 5.76975 5.76975 0 up vs 1 -8.21417 67.4727 1.20674 3.79159 1cg01256999 BANP 0.27136 0.412187 2.10E-14 0.658343 -1.51897 0 down vs 1 -8.2163 67.5075 0.359399 1.12333 1cg02593403 SEC31B -0.067365 0.255683 2.10E-14 -0.263471 -3.79549 0 down vs 1 -8.21326 67.4576 1.89213 5.94644 1cg23609682 PRDM16 -0.02137 0.226225 2.12E-14 -0.0944636 -10.5861 0 down vs 1 -8.21209 67.4385 1.11148 3.49405 1cg08391709 PRKCG 0.2489 0.45675 2.12E-14 0.544937 -1.83507 0 down vs 1 -8.21207 67.4382 0.783282 2.46234 1cg10556142 0.045795 0.269369 2.15E-14 0.170009 -5.88205 0 down vs 1 -8.2101 67.4057 0.906273 2.85035 1cg26667946 SIAH3 -0.092465 0.267203 2.15E-14 -0.346048 -2.88977 0 down vs 1 -8.2097 67.3992 2.34542 7.37737 1cg12806613 -0.235365 0.0769232 2.15E-14 -3.05974 3.05974 0 up vs 1 -8.20955 67.3967 1.76819 5.56194 1cg23812660 ZEB2 0.187845 0.416084 2.16E-14 0.45146 -2.21504 0 down vs 1 -8.20908 67.3891 0.944487 2.97127 1cg03838769 SPOCK3 0.05857 0.302267 2.16E-14 0.193769 -5.16078 0 down vs 1 -8.20887 67.3856 1.07676 3.38756 1cg07870237 ACSL6 -0.366515 -0.0439268 2.17E-14 8.34377 8.34377 0 up vs 1 -8.20846 67.3789 1.88675 5.93645 1cg15127832 STOX2 0.330575 0.432105 2.17E-14 0.765035 -1.30713 0 down vs 1 -8.20827 67.3758 0.186898 0.588079 1cg11331739 0.06131 0.275082 2.17E-14 0.222879 -4.48674 0 down vs 1 -8.20796 67.3705 0.828551 2.60727 1cg25001102 0.03222 0.37388 2.19E-14 0.0861773 -11.604 0 down vs 1 -8.2071 67.3565 2.11645 6.66138 1cg24562465 0.17109 0.35979 2.19E-14 0.475528 -2.10293 0 down vs 1 -8.20669 67.3497 0.645595 2.03217 1cg04242655 PRAME -0.11856 0.252042 2.19E-14 -0.470398 -2.12586 0 down vs 1 -8.20649 67.3464 2.49019 7.83888 1cg24344143 0.15688 0.384036 2.21E-14 0.408503 -2.44796 0 down vs 1 -8.20558 67.3316 0.935549 2.94567 1cg17401780 0.22659 0.383676 2.21E-14 0.590577 -1.69326 0 down vs 1 -8.20554 67.3308 0.447396 1.40868 1cg24569447 -0.32694 0.120362 2.21E-14 -2.71631 2.71631 0 up vs 1 -8.20527 67.3264 3.6276 11.4227 1cg07015554 -0.283095 0.101958 2.21E-14 -2.77658 2.77658 0 up vs 1 -8.20519 67.3251 2.68819 8.46483 1cg12053971 GPR123 0.24714 0.457252 2.21E-14 0.54049 -1.85017 0 down vs 1 -8.20518 67.325 0.800424 2.52046 1cg05884870 ADAMTS17 0.057205 0.319003 2.23E-14 0.179324 -5.57649 0 down vs 1 -8.20428 67.3102 1.24266 3.91387 1cg23173466 MAX -0.11271 -0.351061 2.24E-14 0.321055 -3.11473 0 down vs 1 8.20298 67.2888 1.03004 3.24523 1cg26070134 FAM50A 0.320855 0.467146 2.25E-14 0.68684 -1.45594 0 down vs 1 -8.20281 67.286 0.388021 1.22255 1cg00393837 -0.199005 0.136854 2.25E-14 -1.45414 1.45414 0 up vs 1 -8.20271 67.2844 2.04518 6.44397 1cg16118539 0.01817 0.277683 2.26E-14 0.0654343 -15.2825 0 down vs 1 -8.20212 67.2748 1.22106 3.84786 1cg08734395 0.039395 0.322945 2.26E-14 0.121987 -8.19762 0 down vs 1 -8.20157 67.2658 1.45773 4.5943 1cg11230940 -0.05039 0.205646 2.27E-14 -0.245032 -4.0811 0 down vs 1 -8.20146 67.264 1.18856 3.74606 1cg04014889 MAGEL2 0.12354 0.350778 2.29E-14 0.352189 -2.83939 0 down vs 1 -8.20003 67.2405 0.936223 2.95178 1cg13360823 CACNG6 -0.0833 0.162078 2.29E-14 -0.513951 -1.94571 0 down vs 1 -8.19995 67.2392 1.09166 3.44193 1cg11693581 KIAA1598 -0.011015 0.323904 2.29E-14 -0.034007 -29.4057 0 down vs 1 -8.19977 67.2362 2.03375 6.41252 1cg09755181 LOC285830 -0.104735 0.134002 2.30E-14 -0.781592 -1.27944 0 down vs 1 -8.19924 67.2275 1.03337 3.25872 1cg18620542 HRNBP3 0.16547 0.38034 2.30E-14 0.435058 -2.29854 0 down vs 1 -8.20483 67.3193 0.836272 2.60872 1cg25019989 -0.215875 0.0959634 2.30E-14 -2.24956 2.24956 0 up vs 1 -8.19876 67.2196 1.7631 5.56054 1cg19554281 0.064695 0.324199 2.31E-14 0.199553 -5.01119 0 down vs 1 -8.19832 67.2124 1.22097 3.85117 1cg06756211 CAPN2 -0.15262 0.171762 2.31E-14 -0.888553 -1.12543 0 down vs 1 -8.1983 67.2121 1.9078 6.01757 1cg04308185 ORMDL3 -0.22513 0.222525 2.32E-14 -1.01171 1.01171 0 up vs 1 -8.19796 67.2065 3.63333 11.4612 1cg19246110 ZNF671 -0.273585 0.0960103 2.32E-14 -2.84954 2.84954 0 up vs 1 -8.19751 67.1991 2.47669 7.81346 1cg14742937 ADAM5P 0.008025 0.311097 2.32E-14 0.0257958 -38.766 0 down vs 1 -8.1974 67.1974 1.66536 5.25403 1cg18466674 HSPA1A -0.286175 -0.468432 2.34E-14 0.61092 -1.63687 0 down vs 1 8.19659 67.1842 0.602266 1.90046 1cg27314324 KCND3 0.18399 0.408553 2.36E-14 0.450345 -2.22052 0 down vs 1 -8.19502 67.1583 0.914313 2.88623 1cg05169846 CACNA2D4 -0.218125 -0.42709 2.36E-14 0.510724 -1.958 0 down vs 1 8.19782 67.2043 0.791322 2.4845 1cg06489728 0.064885 0.412966 2.36E-14 0.157119 -6.36459 0 down vs 1 -8.19478 67.1545 2.19675 6.93491 1cg02137691 FGFR3 0.176165 0.35475 2.38E-14 0.496589 -2.01374 0 down vs 1 -8.19398 67.1413 0.57824 1.8258 1cg16011250 0.138435 0.33887 2.38E-14 0.408519 -2.44786 0 down vs 1 -8.19378 67.138 0.728393 2.30003 1cg24792289 -0.18569 0.0588196 2.38E-14 -3.15694 3.15694 0 up vs 1 -8.19349 67.1332 1.08395 3.42301 1cg19434087 PTPRN2 0.021195 0.309295 2.38E-14 0.0685267 -14.5928 0 down vs 1 -8.19335 67.131 1.50489 4.75246 1cg12182525 ASB5 0.22013 0.400303 2.39E-14 0.549909 -1.81848 0 down vs 1 -8.19269 67.1201 0.588566 1.859 1cg14625594 0.247145 0.412269 2.40E-14 0.599475 -1.66813 0 down vs 1 -8.19238 67.1151 0.494355 1.56154 1cg00557947 -0.16353 0.203795 2.41E-14 -0.802425 -1.24622 0 down vs 1 -8.19192 67.1076 2.44635 7.72828 1cg00842259 GLI3 0.139975 0.298956 2.42E-14 0.468212 -2.13578 0 down vs 1 -8.19121 67.096 0.458257 1.44793 1cg19577779 SLITRK5 -0.17418 0.219687 2.42E-14 -0.792857 -1.26126 0 down vs 1 -8.19113 67.0945 2.81265 8.8872 1cg26847438 SP140L -0.336935 -0.461009 2.42E-14 0.730865 -1.36824 0 down vs 1 8.19095 67.0917 0.279112 0.881952 1cg05818685 CTNND2 -0.142555 0.126305 2.42E-14 -1.12866 1.12866 0 up vs 1 -8.19091 67.091 1.3106 4.14134 1cg17147317 CR1 -0.02499 0.201802 2.43E-14 -0.123835 -8.07529 0 down vs 1 -8.19059 67.0858 0.932549 2.94698 1cg02969038 0.023205 0.273045 2.43E-14 0.0849859 -11.7667 0 down vs 1 -8.19029 67.0808 1.13173 3.57669 1cg02359773 RSPH6A 0.07194 0.423697 2.43E-14 0.169791 -5.88959 0 down vs 1 -8.19029 67.0808 2.24339 7.08993 1cg10328279 TAS2R60 0.04788 0.331838 2.43E-14 0.144287 -6.93062 0 down vs 1 -8.19027 67.0806 1.46193 4.62025 1cg06434255 OR2H2 0.07601 0.289379 2.43E-14 0.262666 -3.80712 0 down vs 1 -8.19021 67.0796 0.825434 2.60872 1cg10296496 CBFA2T3 -0.015445 0.274695 2.44E-14 -0.0562259 -17.7854 0 down vs 1 -8.1899 67.0744 1.52628 4.82407 1cg25221239 -0.08527 0.21283 2.44E-14 -0.400648 -2.49596 0 down vs 1 -8.18974 67.0719 1.61118 5.09259 1cg02490589 PYHIN1 0.200155 0.411166 2.45E-14 0.486798 -2.05424 0 down vs 1 -8.18893 67.0586 0.807291 2.55218 1cg13710556 -0.12528 0.194921 2.46E-14 -0.642723 -1.55588 0 down vs 1 -8.18873 67.0554 1.85893 5.87712 1cg05649126 LOC100130017 0.25982 0.406925 2.46E-14 0.638496 -1.56618 0 down vs 1 -8.18862 67.0535 0.392351 1.24048 1cg24316982 SPRR4 0.190315 0.395625 2.48E-14 0.481049 -2.07879 0 down vs 1 -8.18737 67.033 0.764257 2.41706 1cg21390082 SLFN12L 0.161145 0.392951 2.50E-14 0.41009 -2.43849 0 down vs 1 -8.18579 67.0071 0.974239 3.08234 1cg11340941 0.16363 0.317243 2.51E-14 0.515787 -1.93878 0 down vs 1 -8.18551 67.0026 0.427834 1.35369 1cg09075268 CACNA1H 0.2009 0.421711 2.51E-14 0.476393 -2.09911 0 down vs 1 -8.18518 66.9972 0.884013 2.79729 1cg14620958 0.01852 0.295575 2.51E-14 0.0626575 -15.9598 0 down vs 1 -8.18513 66.9964 1.39172 4.40388 1cg00233028 -0.251555 0.149057 2.52E-14 -1.68764 1.68764 0 up vs 1 -8.18461 66.9878 2.90982 9.20888 1cg03580872 0.187735 0.394574 2.53E-14 0.475792 -2.10176 0 down vs 1 -8.18416 66.9804 0.775678 2.4551 1cg08331840 KCNU1 0.277895 0.404013 2.53E-14 0.687837 -1.45383 0 down vs 1 -8.1839 66.9763 0.288384 0.912822 1cg12403889 C1orf187 -0.3287 -0.420772 2.54E-14 0.781184 -1.28011 0 down vs 1 8.1834 66.968 0.153699 0.486562 1cg05847075 0.1177 0.320266 2.54E-14 0.367506 -2.72104 0 down vs 1 -8.18334 66.967 0.743966 2.3552 1cg25804280 SDK1 0.34501 0.428505 2.54E-14 0.805149 -1.24201 0 down vs 1 -8.18314 66.9637 0.126397 0.400158 1cg05133179 -0.257635 0.077484 2.55E-14 -3.32501 3.32501 0 up vs 1 -8.18257 66.9544 2.03618 6.44722 1cg24720717 LRRC8D -0.17418 -0.465022 2.56E-14 0.374563 -2.66978 0 down vs 1 8.1824 66.9517 1.53367 4.8563 1cg08200625 F2RL3 0.088145 0.350778 2.56E-14 0.251284 -3.97955 0 down vs 1 -8.18231 66.9503 1.25059 3.96004 1cg26033586 LSAMP -0.2435 0.0176552 2.56E-14 -13.792 13.792 0 up vs 1 -8.18189 66.9433 1.23656 3.91601 1cg01851378 -0.18647 0.197321 2.57E-14 -0.94501 -1.05819 0 down vs 1 -8.18176 66.9412 2.67059 8.45763 1cg23117727 -0.05141 0.271518 2.57E-14 -0.189343 -5.28142 0 down vs 1 -8.18153 66.9375 1.89073 5.9882 1cg10838683 0.09305 0.367143 2.57E-14 0.253443 -3.94566 0 down vs 1 -8.18152 66.9372 1.36212 4.31403 1cg18712752 PTPRN2 0.322815 0.417522 2.57E-14 0.773169 -1.29338 0 down vs 1 -8.18151 66.937 0.162624 0.515054 1cg06173536 GNG4 -0.067735 0.293302 2.57E-14 -0.23094 -4.33013 0 down vs 1 -8.18121 66.9322 2.36331 7.4855 1cg16446738 0.237505 -0.105644 2.58E-14 -2.24817 2.24817 0 up vs 1 8.18103 66.9293 2.13493 6.76242 1cg22032521 HYLS1 -0.224885 -0.439432 2.58E-14 0.511763 -1.95403 0 down vs 1 8.18072 66.9242 0.83457 2.64372 1cg26890354 PCDHGA4 -0.275895 0.0530454 2.58E-14 -5.20111 5.20111 0 up vs 1 -8.18061 66.9224 1.96179 6.21465 1cg18884388 0.327805 0.471795 2.59E-14 0.694803 -1.43926 0 down vs 1 -8.18034 66.9179 0.375911 1.19091 1cg08293528 CDH23 -0.214095 0.0764675 2.59E-14 -2.79982 2.79982 0 up vs 1 -8.18022 66.916 1.53072 4.84957 1cg19109538 SLITRK5 -0.297475 0.0835887 2.62E-14 -3.5588 3.5588 0 up vs 1 -8.17853 66.8884 2.63277 8.34446 1cg24453604 0.174985 0.403001 2.65E-14 0.434205 -2.30306 0 down vs 1 -8.17949 66.904 0.942186 2.97144 1cg15835825 HTR5A -0.33751 0.033866 2.67E-14 -9.96605 9.96605 0 up vs 1 -8.17556 66.8397 2.50061 7.93134 1cg23325570 LOC285830 0.116295 0.342163 2.68E-14 0.339881 -2.9422 0 down vs 1 -8.17491 66.8291 0.924973 2.93426 1cg13742956 KCNQ1 0.077765 0.263935 2.69E-14 0.294637 -3.39401 0 down vs 1 -8.17442 66.8211 0.628402 1.9937 1cg02351840 -0.15792 0.264136 2.70E-14 -0.597874 -1.67259 0 down vs 1 -8.17387 66.8121 3.22967 10.248 1cg13324103 SVIL -0.400505 -0.463379 2.70E-14 0.864313 -1.15699 0 down vs 1 8.17363 66.8083 0.0716746 0.227442 1cg02898293 VSX1 -0.1755 0.0836515 2.72E-14 -2.09799 2.09799 0 up vs 1 -8.17258 66.791 1.21766 3.86495 1cg12060499 0.010365 -0.375503 2.73E-14 -0.027603 -36.228 0 down vs 1 8.17219 66.7847 2.69958 8.56948 1cg04067612 C1QTNF7 0.010075 0.284592 2.73E-14 0.0354015 -28.2474 0 down vs 1 -8.17178 66.7779 1.36634 4.33771 1cg07090758 MYH11 0.29664 0.448933 2.75E-14 0.660767 -1.51339 0 down vs 1 -8.17092 66.7639 0.420511 1.33528 1cg16491530 AIM1L 0.24429 0.382161 2.75E-14 0.639233 -1.56438 0 down vs 1 -8.17072 66.7607 0.34464 1.09441 1cg14986870 SFMBT2 0.097035 0.317679 2.76E-14 0.305449 -3.27386 0 down vs 1 -8.17004 66.7495 0.882681 2.80344 1cg19797739 0.185865 0.395449 2.76E-14 0.470009 -2.12762 0 down vs 1 -8.17002 66.7493 0.796409 2.52945 1cg02992632 FGF12 -0.376155 -0.0139923 2.77E-14 26.8831 26.8831 0 up vs 1 -8.16935 66.7382 2.37807 7.55417 1cg04271617 ADARB2 -0.0116 0.263663 2.78E-14 -0.0439956 -22.7296 0 down vs 1 -8.169 66.7325 1.37377 4.36427 1cg04968532 0.167495 0.363546 2.79E-14 0.460726 -2.17049 0 down vs 1 -8.16831 66.7214 0.696876 2.21425 1cg09274040 KNDC1 0.13049 0.394758 2.80E-14 0.330557 -3.0252 0 down vs 1 -8.16793 66.7151 1.26622 4.02364 1cg20382146 ZIC5 0.19697 0.374017 2.80E-14 0.526634 -1.89885 0 down vs 1 -8.1677 66.7113 0.568323 1.80606 1cg13757081 LOC441204 -0.188305 0.23447 2.81E-14 -0.803109 -1.24516 0 down vs 1 -8.16717 66.7027 3.24068 10.2998 1cg05573550 ARHGAP10 0.0903 0.266716 2.82E-14 0.338562 -2.95367 0 down vs 1 -8.16696 66.6993 0.564279 1.79353 1cg10863207 RFFL 0.0492 -0.232593 2.82E-14 -0.211528 -4.7275 0 down vs 1 8.16656 66.6928 1.43972 4.57651 1cg22983827 0.22447 0.391243 2.83E-14 0.573736 -1.74296 0 down vs 1 -8.16629 66.6883 0.504276 1.60308 1cg07083818 KIAA1683 0.04855 -0.204216 2.83E-14 -0.237738 -4.2063 0 down vs 1 8.16604 66.6842 1.15839 3.68271 1cg09890980 0.186355 0.387185 2.84E-14 0.481308 -2.07767 0 down vs 1 -8.16592 66.6822 0.731262 2.32487 1cg17857691 TMEM206 0.32162 0.411815 2.85E-14 0.780981 -1.28044 0 down vs 1 -8.16523 66.6709 0.147498 0.469015 1cg11517094 HNRNPH1 -0.30721 -0.414247 2.85E-14 0.741611 -1.34842 0 down vs 1 8.16482 66.6643 0.207723 0.660583 1cg03512250 DIRC3 0.19904 0.389135 2.86E-14 0.511494 -1.95506 0 down vs 1 -8.16463 66.6612 0.655175 2.08363 1cg07538634 ZFR2 -0.06003 0.200596 2.86E-14 -0.299258 -3.34159 0 down vs 1 -8.16446 66.6584 1.23155 3.91683 1cg23940999 0.310315 0.471303 2.87E-14 0.65842 -1.51879 0 down vs 1 -8.16413 66.653 0.469897 1.49458 1cg02570061 HNRNPH1 -0.35697 -0.433774 2.87E-14 0.822941 -1.21515 0 down vs 1 8.16396 66.6503 0.10695 0.340186 1cg14994521 0.208365 0.408265 2.87E-14 0.510366 -1.95938 0 down vs 1 -8.16395 66.65 0.724512 2.30452 1cg18943812 PXDN 0.24099 0.41137 2.88E-14 0.585823 -1.707 0 down vs 1 -8.16365 66.6451 0.526327 1.67426 1cg13785068 0.116155 0.347375 2.89E-14 0.33438 -2.99061 0 down vs 1 -8.16298 66.6343 0.969321 3.08394 1cg07336964 ABAT 0.07997 0.327459 2.91E-14 0.244214 -4.09477 0 down vs 1 -8.16157 66.6112 1.11053 3.53441 1cg14825676 UGT1A5 0.172115 0.363583 2.92E-14 0.473386 -2.11244 0 down vs 1 -8.16143 66.609 0.664676 2.1155 1cg17868538 0.161995 0.389484 2.92E-14 0.415922 -2.4043 0 down vs 1 -8.16127 66.6064 0.938294 2.98648 1cg25290307 EMID2 0.00247 0.257201 2.92E-14 0.00960338 -104.13 0 down vs 1 -8.16122 66.6056 1.17647 3.74461 1cg17880210 -0.030055 0.30625 2.92E-14 -0.0981388 -10.1897 0 down vs 1 -8.16107 66.6031 2.05062 6.52719 1cg11852471 0.07804 0.287427 2.93E-14 0.271512 -3.68308 0 down vs 1 -8.16077 66.5982 0.794911 2.53042 1cg14494620 -0.238885 0.115643 2.93E-14 -2.06571 2.06571 0 up vs 1 -8.1607 66.5971 2.27887 7.25439 1cg11071202 -0.13306 0.169759 2.93E-14 -0.783818 -1.27581 0 down vs 1 -8.16065 66.5961 1.66258 5.29262 1cg00324366 NRG1 0.123075 0.356235 2.93E-14 0.345489 -2.89445 0 down vs 1 -8.16043 66.5927 0.985654 3.13786 1cg15037541 0.13483 0.441711 2.94E-14 0.305245 -3.27606 0 down vs 1 -8.16025 66.5897 1.70749 5.4361 1cg25077733 0.114675 0.367914 2.96E-14 0.31169 -3.20832 0 down vs 1 -8.15935 66.575 1.16273 3.70257 1cg18405900 AIM1 -0.379115 -0.461474 2.97E-14 0.821531 -1.21724 0 down vs 1 8.15881 66.5662 0.122981 0.391669 1cg11801524 SVIL 0.22847 0.404674 2.98E-14 0.564578 -1.77123 0 down vs 1 -8.15799 66.5528 0.562922 1.79315 1cg10092394 0.111905 0.376764 2.98E-14 0.297016 -3.36682 0 down vs 1 -8.15788 66.551 1.27188 4.05162 1cg22277103 PTPRN2 0.077795 0.29566 2.99E-14 0.263123 -3.80051 0 down vs 1 -8.15727 66.541 0.860586 2.74183 1cg02909136 -0.33936 0.0311376 3.00E-14 -10.8987 10.8987 0 up vs 1 -8.15674 66.5325 2.48879 7.93033 1cg05163329 -0.063565 0.267286 3.01E-14 -0.237817 -4.20492 0 down vs 1 -8.15667 66.5313 1.98464 6.32399 1cg25669494 0.34121 0.434902 3.01E-14 0.784568 -1.27459 0 down vs 1 -8.15643 66.5274 0.159154 0.507171 1cg12364131 LOC254559 0.017305 -0.24803 3.01E-14 -0.0697698 -14.3328 0 down vs 1 8.15619 66.5234 1.27646 4.06788 1cg05923681 PBRM1 0.371445 0.439395 3.02E-14 0.845356 -1.18293 0 down vs 1 -8.15596 66.5197 0.0837134 0.266797 1cg06911354 HOXA7 -0.051245 0.281625 3.02E-14 -0.181962 -5.49566 0 down vs 1 -8.15594 66.5193 2.00894 6.40259 1cg09921682 ZPBP 0.315065 0.458935 3.03E-14 0.686513 -1.45664 0 down vs 1 -8.15554 66.5128 0.375283 1.19616 1cg05338930 MGAT5B 0.1942 0.432809 3.03E-14 0.448697 -2.22868 0 down vs 1 -8.15546 66.5116 1.03226 3.29025 1cg04181327 -0.046675 0.173548 3.04E-14 -0.268946 -3.71822 0 down vs 1 -8.15497 66.5036 0.879312 2.80307 1cg00344655 ECE1 0.22111 0.330389 3.04E-14 0.669241 -1.49423 0 down vs 1 -8.15488 66.502 0.216517 0.69023 1cg16150863 MAS1L 0.19603 0.379574 3.05E-14 0.516447 -1.93631 0 down vs 1 -8.15453 66.4963 0.610801 1.94732 1cg10966580 -0.222385 0.101391 3.05E-14 -2.19334 2.19334 0 up vs 1 -8.1542 66.491 1.90068 6.06011 1cg03081615 ABLIM2 0.30417 0.464513 3.05E-14 0.654815 -1.52715 0 down vs 1 -8.1542 66.491 0.466141 1.48625 1cg17271970 0.143135 0.369336 3.05E-14 0.387547 -2.58033 0 down vs 1 -8.15415 66.4902 0.927695 2.9579 1cg02630277 0.195755 0.364105 3.06E-14 0.537634 -1.86 0 down vs 1 -8.15404 66.4883 0.513857 1.63845 1cg18207741 -0.141155 0.21816 3.07E-14 -0.647026 -1.54553 0 down vs 1 -8.1533 66.4763 2.34082 7.46512 1cg02931762 -0.17637 0.0418 3.08E-14 -4.21938 4.21938 0 up vs 1 -8.15276 66.4675 0.862994 2.75255 1cg07981495 CGA 0.226325 0.39539 3.08E-14 0.57241 -1.747 0 down vs 1 -8.15263 66.4654 0.518231 1.65297 1cg13079234 0.11873 0.315596 3.11E-14 0.376208 -2.6581 0 down vs 1 -8.15115 66.4412 0.702686 2.24212 1cg23166389 LHX8 -0.277275 0.082099 3.13E-14 -3.37733 3.37733 0 up vs 1 -8.15032 66.4277 2.34159 7.47305 1cg06943853 CACNG2 0.073175 0.298103 3.14E-14 0.245469 -4.07383 0 down vs 1 -8.14989 66.4208 0.917283 2.92776 1cg12799739 CRYBG3 0.230455 0.374028 3.14E-14 0.616144 -1.623 0 down vs 1 -8.14964 66.4167 0.373734 1.19295 1cg06243556 ZSCAN18 -0.357645 0.00845052 3.14E-14 -42.3223 42.3223 0 up vs 1 -8.1496 66.416 2.43 7.75658 1cg00220294 -0.13649 0.0994603 3.14E-14 -1.37231 1.37231 0 up vs 1 -8.14959 66.4158 1.00939 3.22199 1cg22974100 0.133835 0.379517 3.15E-14 0.352646 -2.83571 0 down vs 1 -8.15224 66.459 1.09384 3.47281 1cg23345004 KATNAL2 -0.1071 0.207465 3.15E-14 -0.516232 -1.93711 0 down vs 1 -8.1494 66.4128 1.79407 5.72694 1cg01546568 CTNNA2 -0.31881 0.10966 3.15E-14 -2.90725 2.90725 0 up vs 1 -8.14935 66.4119 3.32858 10.6255 1cg26333618 0.040285 0.267714 3.15E-14 0.150478 -6.6455 0 down vs 1 -8.14923 66.41 0.937798 2.99372 1cg25486506 ZNF187 0.2788 0.376678 3.16E-14 0.740155 -1.35107 0 down vs 1 -8.14888 66.4043 0.173695 0.554531 1cg15448394 DNAH12 0.15594 0.377911 3.17E-14 0.412637 -2.42344 0 down vs 1 -8.14801 66.3901 0.893326 2.85261 1cg15216899 PLD1 0.267055 0.414249 3.18E-14 0.644673 -1.55117 0 down vs 1 -8.14781 66.3868 0.392824 1.25445 1cg18406531 KRTAP26-1 0.19263 0.381996 3.18E-14 0.504272 -1.98305 0 down vs 1 -8.14758 66.3831 0.650162 2.07635 1cg01012242 SLC37A3 0.032905 0.228513 3.19E-14 0.143996 -6.94464 0 down vs 1 -8.14709 66.3751 0.693734 2.21576 1cg09438457 ZNF222 -0.34638 0.0114237 3.19E-14 -30.3211 30.3211 0 up vs 1 -8.14705 66.3745 2.32117 7.41383 1cg18709313 -0.119765 0.217644 3.19E-14 -0.550279 -1.81726 0 down vs 1 -8.147 66.3736 2.06411 6.59284 1cg03778594 0.30497 0.449162 3.20E-14 0.678975 -1.47281 0 down vs 1 -8.14657 66.3667 0.376966 1.20417 1cg22196764 0.234505 0.410021 3.22E-14 0.571934 -1.74845 0 down vs 1 -8.14571 66.3525 0.558537 1.78456 1cg09607548 JPH2 -0.172305 0.133445 3.22E-14 -1.2912 1.2912 0 up vs 1 -8.14566 66.3518 1.69493 5.41546 1cg08642111 CSMD1 -0.24963 0.0895093 3.22E-14 -2.78887 2.78887 0 up vs 1 -8.14559 66.3507 2.08533 6.66292 1cg03240232 OAS2 0.22416 0.355292 3.22E-14 0.630917 -1.58499 0 down vs 1 -8.14554 66.3499 0.311772 0.996168 1cg23043143 IQCE -0.145275 -0.306076 3.23E-14 0.474637 -2.10687 0 down vs 1 8.14545 66.3484 0.468807 1.49796 1cg07152755 -0.141845 0.161739 3.24E-14 -0.877001 -1.14025 0 down vs 1 -8.14479 66.3376 1.67099 5.34012 1cg14259902 EPOR 0.15049 0.314003 3.24E-14 0.479264 -2.08653 0 down vs 1 -8.14468 66.3359 0.484753 1.5492 1cg13209481 SFTA3 -0.14201 0.095951 3.25E-14 -1.48003 1.48003 0 up vs 1 -8.14418 66.3276 1.02667 3.28149 1cg19237063 PTPRN2 0.176215 0.439051 3.26E-14 0.401354 -2.49156 0 down vs 1 -8.14394 66.3238 1.25253 4.00363 1cg21277243 -0.27799 0.0215015 3.26E-14 -12.9288 12.9288 0 up vs 1 -8.14376 66.3209 1.62625 5.19843 1cg18634398 0.23361 0.380294 3.26E-14 0.614288 -1.6279 0 down vs 1 -8.14356 66.3176 0.390106 1.24707 1cg26866014 C7orf33 0.310495 0.436761 3.27E-14 0.710903 -1.40666 0 down vs 1 -8.14335 66.3141 0.289063 0.924109 1cg19635501 ABLIM2 0.25005 0.40632 3.29E-14 0.615402 -1.62495 0 down vs 1 -8.14252 66.3007 0.442758 1.41574 1cg19743317 NLGN4X -0.128405 0.175902 3.30E-14 -0.729982 -1.3699 0 down vs 1 -8.14175 66.288 1.67896 5.36959 1cg07671477 SLC6A18 0.21918 0.444703 3.31E-14 0.492869 -2.02894 0 down vs 1 -8.14143 66.283 0.922142 2.94939 1cg22417566 0.017105 0.395116 3.31E-14 0.0432911 -23.0994 0 down vs 1 -8.14125 66.28 2.59076 8.28668 1cg24952408 LOC285830 0.30818 0.43751 3.33E-14 0.704395 -1.41966 0 down vs 1 -8.14058 66.269 0.303262 0.970161 1cg22598028 ZNF660 -0.23722 0.145455 3.33E-14 -1.63088 1.63088 0 up vs 1 -8.14056 66.2687 2.65509 8.49388 1cg22839476 EBF3 0.17925 0.402468 3.34E-14 0.445378 -2.24529 0 down vs 1 -8.13973 66.2551 0.903388 2.89062 1cg10536803 0.112915 0.325708 3.35E-14 0.346675 -2.88454 0 down vs 1 -8.13944 66.2505 0.820982 2.62712 1cg19638039 VRK1 0.023055 0.299101 3.35E-14 0.0770811 -12.9733 0 down vs 1 -8.13933 66.2487 1.38159 4.42118 1cg07374899 0.272915 0.408356 3.36E-14 0.668327 -1.49627 0 down vs 1 -8.13887 66.2413 0.332595 1.06445 1cg20899781 0.068455 -0.204292 3.37E-14 -0.335085 -2.98432 0 down vs 1 8.13868 66.238 1.34877 4.31684 1cg10724965 0.007775 0.240623 3.37E-14 0.032312 -30.9483 0 down vs 1 -8.1385 66.2352 0.983019 3.14636 1cg08098923 SNX16 0.382785 0.44053 3.38E-14 0.868918 -1.15086 0 down vs 1 -8.13819 66.2301 0.0604579 0.193523 1cg08371852 ARNT2 0.01652 -0.235649 3.39E-14 -0.0701043 -14.2645 0 down vs 1 8.13775 66.2231 1.15293 3.69086 1cg01586609 HTR3A -0.08896 0.223215 3.40E-14 -0.39854 -2.50916 0 down vs 1 -8.13709 66.2122 1.76691 5.65733 1cg06868946 XKR4 -0.16216 0.0653418 3.40E-14 -2.48172 2.48172 0 up vs 1 -8.13701 66.2109 0.938399 3.00465 1cg02499281 RBM33 0.369335 0.426414 3.41E-14 0.866142 -1.15455 0 down vs 1 -8.13642 66.2013 0.0590703 0.189164 1cg00471190 IL32 0.173805 -0.114934 3.43E-14 -1.51222 1.51222 0 up vs 1 8.13563 66.1885 1.51157 4.84151 1cg20684528 GRIN2B -0.339905 0.0352845 3.43E-14 -9.63326 9.63326 0 up vs 1 -8.1356 66.1879 2.55223 8.17478 1cg09684336 LGR6 0.257285 0.395797 3.44E-14 0.650042 -1.53836 0 down vs 1 -8.1353 66.1831 0.347853 1.11425 1cg03827337 RGS7BP -0.301985 0.0656438 3.44E-14 -4.60036 4.60036 0 up vs 1 -8.13519 66.1814 2.4504 7.84941 1cg13368756 CTNND2 -0.21835 0.102219 3.44E-14 -2.13611 2.13611 0 up vs 1 -8.13519 66.1813 1.8632 5.96844 1cg16577208 GLI3 0.188095 0.40294 3.44E-14 0.466807 -2.14221 0 down vs 1 -8.13499 66.178 0.836888 2.68096 1cg20964216 0.09266 0.380788 3.46E-14 0.243338 -4.10951 0 down vs 1 -8.13446 66.1694 1.50518 4.82244 1cg03008707 UNC5C -0.272885 0.0746304 3.47E-14 -3.65649 3.65649 0 up vs 1 -8.13381 66.1588 2.18961 7.01639 1cg11312896 ZSCAN1 -0.136115 0.194555 3.47E-14 -0.699623 -1.42934 0 down vs 1 -8.1393 66.2482 1.98054 6.27812 1cg02886375 -0.04579 0.349627 3.48E-14 -0.130968 -7.63544 0 down vs 1 -8.1334 66.1522 2.83484 9.0849 1cg20408707 OR2H1 0.22663 0.405957 3.49E-14 0.558261 -1.79128 0 down vs 1 -8.13308 66.147 0.583056 1.86868 1cg08492619 SLITRK5 -0.25455 0.120491 3.49E-14 -2.1126 2.1126 0 up vs 1 -8.13279 66.1423 2.55021 8.17396 1cg20620783 OR5E1P 0.13651 0.367993 3.51E-14 0.370958 -2.69572 0 down vs 1 -8.13201 66.1296 0.971532 3.11456 1cg07952813 LOC100271715 -0.182225 -0.396273 3.51E-14 0.459847 -2.17464 0 down vs 1 8.1318 66.1262 0.830694 2.6632 1cg20052010 EFEMP1 0.339565 0.436862 3.52E-14 0.777282 -1.28653 0 down vs 1 -8.13144 66.1203 0.171639 0.550322 1cg18568990 EDNRB -0.34853 -0.00647474 3.54E-14 53.8292 53.8292 0 up vs 1 -8.13083 66.1104 2.12134 6.80263 1cg01700358 0.233835 0.397645 3.54E-14 0.58805 -1.70054 0 down vs 1 -8.13076 66.1093 0.486517 1.56017 1cg04156581 TCTE1 0.04383 0.322498 3.54E-14 0.135908 -7.35794 0 down vs 1 -8.13073 66.1088 1.40797 4.51513 1cg09469870 0.07548 0.316647 3.56E-14 0.238373 -4.19511 0 down vs 1 -8.1296 66.0903 1.05452 3.3826 1cg24760869 ABLIM1 0.05566 0.283693 3.58E-14 0.196198 -5.0969 0 down vs 1 -8.12872 66.0761 0.942789 3.02487 1cg19754520 CPN1 0.11093 0.370222 3.59E-14 0.299631 -3.33743 0 down vs 1 -8.12835 66.0701 1.21898 3.91134 1cg12170787 SBNO2 -0.032325 -0.246979 3.60E-14 0.130881 -7.64051 0 down vs 1 8.128 66.0645 0.835406 2.68081 1cg13215762 PTPRN2 0.205005 0.425101 3.60E-14 0.482251 -2.07361 0 down vs 1 -8.12781 66.0613 0.878295 2.81857 1cg26033870 MAPK14 0.234325 0.375781 3.61E-14 0.623568 -1.60367 0 down vs 1 -8.12771 66.0596 0.362794 1.16429 1cg24090908 LOH12CR1 0.35124 0.480366 3.61E-14 0.731192 -1.36763 0 down vs 1 -8.12744 66.0553 0.302307 0.970235 1cg00295744 TNRC4 -0.28933 0.130562 3.62E-14 -2.21603 2.21603 0 up vs 1 -8.12724 66.052 3.19664 10.2599 1cg17492965 CPZ 0.061055 0.297779 3.62E-14 0.205035 -4.87722 0 down vs 1 -8.12708 66.0495 1.01602 3.26113 1cg24452260 GRIA2 -0.300565 0.0655794 3.63E-14 -4.58322 4.58322 0 up vs 1 -8.12679 66.0448 2.43065 7.80226 1cg21879571 FBRSL1 0.278295 0.400748 3.63E-14 0.694438 -1.44001 0 down vs 1 -8.1267 66.0432 0.271869 0.872706 1cg23028178 NID1 0.089835 0.300735 3.64E-14 0.298719 -3.34763 0 down vs 1 -8.12644 66.039 0.806435 2.58884 1cg06303875 CDX2 -0.233375 0.113834 3.64E-14 -2.05014 2.05014 0 up vs 1 -8.12601 66.0321 2.18574 7.01745 1cg16048517 CTNND2 -0.274095 -0.000427835 3.67E-14 640.656 640.656 0 up vs 1 -8.1251 66.0173 1.35789 4.36055 1cg10556064 SMPD3 0.000655 0.306611 3.67E-14 0.00213626 -468.108 0 down vs 1 -8.12475 66.0115 1.69721 5.45069 1cg25093409 0.24469 0.395932 3.70E-14 0.618009 -1.6181 0 down vs 1 -8.12362 65.9932 0.41473 1.3323 1cg01635193 TMEM101 -0.10346 -0.382044 3.73E-14 0.270807 -3.69267 0 down vs 1 8.12218 65.9698 1.40712 4.52189 1cg07292442 TNXB 0.04846 0.258082 3.74E-14 0.187769 -5.32568 0 down vs 1 -8.12202 65.9672 0.796698 2.56036 1cg16696727 0.062125 0.286835 3.75E-14 0.216588 -4.61706 0 down vs 1 -8.12161 65.9606 0.91551 2.94249 1cg13797425 HSP90AA1 -0.11285 -0.423054 3.75E-14 0.266751 -3.74882 0 down vs 1 8.1215 65.9588 1.74467 5.60759 1cg21241151 0.07813 0.325521 3.75E-14 0.240015 -4.1664 0 down vs 1 -8.12144 65.9578 1.10965 3.56661 1cg21342728 MCHR1 -0.095765 0.149904 3.76E-14 -0.638842 -1.56533 0 down vs 1 -8.12116 65.9532 1.09426 3.51738 1cg09085842 HSPA1A -0.268965 -0.473998 3.78E-14 0.567439 -1.7623 0 down vs 1 8.1203 65.9393 0.762193 2.45051 1cg03084350 PLCD1 0.011605 0.180608 3.79E-14 0.0642553 -15.5629 0 down vs 1 -8.11967 65.929 0.517852 1.66519 1cg04154502 ZFR2 -0.00041 0.248669 3.80E-14 -0.00164878 -606.51 0 down vs 1 -8.11955 65.9271 1.12484 3.61713 1cg00757182 ZNF773 -0.44454 -0.11717 3.80E-14 3.79397 3.79397 0 up vs 1 -8.11929 65.9229 1.9431 6.24878 1cg02781447 ZNF311 0.222315 0.363662 3.81E-14 0.611323 -1.6358 0 down vs 1 -8.11914 65.9204 0.362238 1.16496 1cg09907936 ZNF135 -0.056095 0.247293 3.81E-14 -0.226836 -4.40846 0 down vs 1 -8.11907 65.9194 1.66884 5.36707 1cg14001664 -0.197365 0.0924907 3.81E-14 -2.13389 2.13389 0 up vs 1 -8.11881 65.915 1.52329 4.89929 1cg22723675 -0.218925 0.172802 3.83E-14 -1.26691 1.26691 0 up vs 1 -8.11827 65.9064 2.78218 8.94939 1cg03227338 C11orf49 0.184585 0.364685 3.84E-14 0.506149 -1.9757 0 down vs 1 -8.11791 65.9005 0.588092 1.89188 1cg26544377 0.256295 0.3979 3.84E-14 0.644119 -1.55251 0 down vs 1 -8.11765 65.8963 0.363559 1.16963 1cg24773532 -0.062065 0.245292 3.85E-14 -0.253025 -3.95217 0 down vs 1 -8.12016 65.9369 1.71196 5.47831 1cg21549434 COL23A1 0.134165 0.411779 3.85E-14 0.325818 -3.0692 0 down vs 1 -8.11722 65.8892 1.39734 4.49597 1cg06394058 CBFA2T3 0.18818 0.384268 3.87E-14 0.489711 -2.04202 0 down vs 1 -8.11647 65.8771 0.697137 2.24346 1cg07999953 GJB3 0.152195 -0.149292 3.89E-14 -1.01945 1.01945 0 up vs 1 8.11557 65.8625 1.64799 5.30459 1cg26668276 DLX6AS -0.178815 0.13726 3.93E-14 -1.30274 1.30274 0 up vs 1 -8.1142 65.8402 1.81134 5.83235 1cg02459016 0.28145 0.44354 3.93E-14 0.634554 -1.57591 0 down vs 1 -8.11385 65.8346 0.476353 1.53395 1cg10108612 ALDH4A1 0.37489 0.482224 3.94E-14 0.777418 -1.28631 0 down vs 1 -8.11347 65.8283 0.208879 0.672694 1cg10880203 0.058135 0.401105 3.95E-14 0.144937 -6.89955 0 down vs 1 -8.1134 65.8273 2.1327 6.86848 1cg21653184 ZSCAN18 -0.312625 0.155909 3.95E-14 -2.00518 2.00518 0 up vs 1 -8.11314 65.823 3.98015 12.8191 1cg14945317 BTNL2 0.16144 0.420894 3.95E-14 0.383565 -2.60712 0 down vs 1 -8.11311 65.8226 1.2205 3.93096 1cg05771518 HOXC13 -0.19494 0.0250727 3.97E-14 -7.775 7.775 0 up vs 1 -8.11249 65.8126 0.877634 2.8271 1cg24616296 FAT3 0.05251 0.312606 3.97E-14 0.167975 -5.95326 0 down vs 1 -8.11237 65.8106 1.22655 3.95115 1cg21834048 GRK7 0.225315 0.422724 3.98E-14 0.533008 -1.87615 0 down vs 1 -8.112 65.8045 0.706562 2.27631 1cg10332700 ZNF415 -0.44079 -0.0389778 3.98E-14 11.3087 11.3087 0 up vs 1 -8.11195 65.8038 2.92728 9.43082 1cg09169531 SPRR2A 0.11039 0.336294 3.99E-14 0.328255 -3.04642 0 down vs 1 -8.1118 65.8013 0.925263 2.98103 1cg17426146 EEF1A2 0.110185 0.406308 3.99E-14 0.271186 -3.68751 0 down vs 1 -8.11169 65.7995 1.58987 5.12243 1cg11353300 SNORD89 0.234925 0.370657 3.99E-14 0.633807 -1.57777 0 down vs 1 -8.11154 65.797 0.334029 1.07625 1cg07202353 EHD4 0.10422 -0.159593 3.99E-14 -0.653037 -1.53131 0 down vs 1 8.11146 65.7957 1.26186 4.06582 1cg11842502 0.280275 0.400041 4.05E-14 0.700616 -1.42732 0 down vs 1 -8.10937 65.7619 0.260066 0.838387 1cg02299020 -0.032945 0.263506 4.05E-14 -0.125026 -7.99837 0 down vs 1 -8.10928 65.7604 1.5934 5.13684 1cg14556787 ADARB2 0.09183 0.394806 4.06E-14 0.232595 -4.29931 0 down vs 1 -8.11174 65.8002 1.6635 5.33431 1cg04864441 PTPRN2 -0.01203 0.276403 4.07E-14 -0.0435234 -22.9762 0 down vs 1 -8.10865 65.7502 1.50837 4.86348 1cg23904247 HSP90AA1 -0.134495 -0.373413 4.09E-14 0.360178 -2.77641 0 down vs 1 8.10758 65.7328 1.03494 3.33787 1cg00762830 CBFA2T3 0.180045 0.43721 4.09E-14 0.411805 -2.42834 0 down vs 1 -8.10751 65.7317 1.19906 3.86725 1cg02167055 CBFA2T3 0.207185 0.424334 4.11E-14 0.48826 -2.04809 0 down vs 1 -8.10684 65.7209 0.854932 2.75781 1cg06516502 0.185115 0.446629 4.12E-14 0.414471 -2.41271 0 down vs 1 -8.10664 65.7176 1.23996 4.00003 1cg13036352 KIAA1949 -0.134565 -0.381226 4.12E-14 0.352979 -2.83303 0 down vs 1 8.10641 65.7139 1.10311 3.55876 1cg16359657 RIMS3 0.100855 0.32843 4.13E-14 0.307082 -3.25646 0 down vs 1 -8.10625 65.7113 0.939007 3.02946 1cg09390001 KIAA1024 0.066685 0.277078 4.13E-14 0.240672 -4.15503 0 down vs 1 -8.10602 65.7075 0.802568 2.58942 1cg17436025 0.288345 0.40363 4.14E-14 0.714379 -1.39982 0 down vs 1 -8.10589 65.7054 0.240972 0.777502 1cg08498406 PTK2B 0.20428 0.401412 4.15E-14 0.508904 -1.96501 0 down vs 1 -8.10539 65.6973 0.704582 2.27363 1cg05157420 EPB41L4A 0.372385 0.435651 4.15E-14 0.854778 -1.16989 0 down vs 1 -8.10532 65.6962 0.0725703 0.234183 1cg15952060 MUC4 -0.14103 0.121697 4.15E-14 -1.15886 1.15886 0 up vs 1 -8.10527 65.6955 1.25149 4.03859 1cg13751927 F2RL3 0.078945 0.398261 4.16E-14 0.198224 -5.04479 0 down vs 1 -8.10513 65.6931 1.84867 5.9659 1cg13734106 EBF3 0.15494 0.348016 4.17E-14 0.445209 -2.24613 0 down vs 1 -8.10447 65.6824 0.675888 2.18153 1cg02896776 SARS 0.157945 0.389391 4.18E-14 0.405621 -2.46536 0 down vs 1 -8.10414 65.6771 0.971217 3.135 1cg05214748 PYCARD -0.158295 -0.351169 4.18E-14 0.450766 -2.21844 0 down vs 1 8.10414 65.677 0.674471 2.17714 1cg13325417 0.192285 0.336441 4.19E-14 0.571527 -1.7497 0 down vs 1 -8.10383 65.6721 0.376775 1.21629 1cg08540945 -0.37875 0.118636 4.22E-14 -3.19254 3.19254 0 up vs 1 -8.10275 65.6546 4.48544 14.4836 1cg04393060 KIAA0319L 0.396985 0.441316 4.22E-14 0.899548 -1.11167 0 down vs 1 -8.1026 65.6522 0.0356314 0.115059 1cg12307314 ARHGAP26 -0.179395 -0.336225 4.23E-14 0.533556 -1.87422 0 down vs 1 8.10231 65.6474 0.445941 1.44011 1cg11784799 -0.201335 0.243528 4.23E-14 -0.826743 -1.20957 0 down vs 1 -8.10222 65.646 3.58815 11.5877 1cg10932486 -0.066035 0.236299 4.24E-14 -0.279455 -3.57839 0 down vs 1 -8.10192 65.6411 1.65726 5.35244 1cg16860712 EHD2 0.176735 0.393444 4.24E-14 0.4492 -2.22618 0 down vs 1 -8.10189 65.6406 0.851477 2.75002 1cg00390784 MYLK4 0.194545 0.371344 4.25E-14 0.523895 -1.90878 0 down vs 1 -8.10172 65.6378 0.566731 1.83045 1cg14269301 MAP3K15 -0.240135 0.0360856 4.26E-14 -6.6546 6.6546 0 up vs 1 -8.10132 65.6314 1.38334 4.46842 1cg12639192 WDR37 0.174725 0.311529 4.27E-14 0.560863 -1.78297 0 down vs 1 -8.10071 65.6215 0.339324 1.09624 1cg06558502 -0.15961 0.0822186 4.27E-14 -1.94129 1.94129 0 up vs 1 -8.10067 65.6209 1.06031 3.42552 1cg13590117 0.2732 0.444139 4.29E-14 0.615123 -1.62569 0 down vs 1 -8.10007 65.6111 0.529784 1.71182 1cg16433265 SORBS2 0.167725 0.358216 4.31E-14 0.468223 -2.13573 0 down vs 1 -8.0993 65.5987 0.65791 2.12622 1cg25124433 KRT20 0.277445 0.421205 4.31E-14 0.658694 -1.51816 0 down vs 1 -8.09918 65.5967 0.374707 1.211 1cg14511923 0.07988 -0.311709 4.32E-14 -0.256264 -3.90222 0 down vs 1 8.09912 65.5958 2.78022 8.98545 1cg16086620 CHGA -0.33155 0.0929268 4.33E-14 -3.56786 3.56786 0 up vs 1 -8.09853 65.5861 3.26683 10.5597 1cg25486824 SPON1 0.240875 0.397125 4.34E-14 0.606547 -1.64868 0 down vs 1 -8.09831 65.5826 0.442649 1.43089 1cg12109740 CLPB 0.30348 0.426884 4.34E-14 0.710919 -1.40663 0 down vs 1 -8.09805 65.5784 0.276106 0.892589 1cg12710228 SORCS2 0.10663 0.357118 4.35E-14 0.298585 -3.34913 0 down vs 1 -8.09801 65.5778 1.1376 3.67764 1cg22697034 0.1437 0.41626 4.36E-14 0.345217 -2.89673 0 down vs 1 -8.09737 65.5674 1.34692 4.35503 1cg13572539 FGFR3 0.275895 0.402155 4.37E-14 0.686041 -1.45764 0 down vs 1 -8.09723 65.5651 0.289035 0.934574 1cg15656087 CDH4 0.12787 0.391402 4.37E-14 0.326698 -3.06093 0 down vs 1 -8.09714 65.5637 1.25917 4.07151 1cg03347590 PITX1 -0.175425 -0.39256 4.37E-14 0.446874 -2.23777 0 down vs 1 8.097 65.5613 0.854828 2.76418 1cg18951543 PTPRN2 -0.002865 0.229953 4.38E-14 -0.0124591 -80.2629 0 down vs 1 -8.09689 65.5597 0.982769 3.17797 1cg05450477 E2F3 0.169375 0.385828 4.39E-14 0.438991 -2.27795 0 down vs 1 -8.09634 65.5507 0.849467 2.74729 1cg12765716 TRPV4 0.099195 0.266712 4.42E-14 0.371918 -2.68876 0 down vs 1 -8.09538 65.5351 0.508786 1.64587 1cg08264835 0.27126 0.465245 4.42E-14 0.583048 -1.71513 0 down vs 1 -8.09536 65.5349 0.682266 2.20708 1cg20583640 0.19696 0.414796 4.42E-14 0.474836 -2.10599 0 down vs 1 -8.09515 65.5315 0.860353 2.78332 1cg24562149 BTBD3 0.05301 0.255307 4.43E-14 0.207632 -4.81621 0 down vs 1 -8.09508 65.5302 0.74199 2.40045 1cg13711238 FAM110B 0.04543 0.290161 4.43E-14 0.156568 -6.387 0 down vs 1 -8.10335 65.6643 1.08433 3.45125 1cg03560973 POMC 0.002845 0.262163 4.43E-14 0.010852 -92.1486 0 down vs 1 -8.09497 65.5286 1.21922 3.94446 1cg01304145 EEF2K 0.325195 0.423634 4.44E-14 0.767632 -1.30271 0 down vs 1 -8.09451 65.5211 0.175692 0.56847 1cg03787988 MRVI1 -0.012715 0.23708 4.46E-14 -0.0536317 -18.6457 0 down vs 1 -8.09396 65.5122 1.13132 3.66099 1cg10458080 ACAD10 0.287295 0.407879 4.46E-14 0.704363 -1.41972 0 down vs 1 -8.09379 65.5095 0.263633 0.853163 1cg16957569 IDO2 0.05911 0.316316 4.48E-14 0.18687 -5.35131 0 down vs 1 -8.09326 65.5008 1.19944 3.88212 1cg26078977 PDE4D -0.08305 0.138082 4.48E-14 -0.601452 -1.66264 0 down vs 1 -8.09315 65.499 0.88659 2.86962 1cg01174786 ADCY9 0.217695 0.362187 4.49E-14 0.601057 -1.66374 0 down vs 1 -8.09288 65.4947 0.378535 1.22528 1cg25081552 0.290465 0.425988 4.51E-14 0.681862 -1.46657 0 down vs 1 -8.09199 65.4803 0.333001 1.07813 1cg19514230 SH3RF3 0.197075 0.394903 4.52E-14 0.499047 -2.00382 0 down vs 1 -8.0919 65.4789 0.709564 2.29734 1cg21534264 CADPS -0.21383 0.144097 4.52E-14 -1.48393 1.48393 0 up vs 1 -8.09188 65.4785 2.32278 7.52048 1cg05205813 CACNB2 0.11116 0.364807 4.52E-14 0.304709 -3.28182 0 down vs 1 -8.09173 65.476 1.16648 3.77685 1cg10201685 CAMK2B -0.30494 0.00152268 4.52E-14 -200.265 200.265 0 up vs 1 -8.09158 65.4736 1.70284 5.51369 1cg18013765 0.245425 0.401148 4.53E-14 0.611807 -1.6345 0 down vs 1 -8.09152 65.4726 0.439666 1.42364 1cg01975957 LCE1A 0.02381 0.322943 4.54E-14 0.0737282 -13.5633 0 down vs 1 -8.0912 65.4675 1.62236 5.25359 1cg07303330 TAAR2 0.196835 0.391813 4.54E-14 0.502369 -1.99057 0 down vs 1 -8.09097 65.4637 0.689273 2.23216 1cg13427361 0.106275 -0.289687 4.56E-14 -0.366861 -2.72582 0 down vs 1 8.09294 65.4957 2.84129 9.15344 1cg04417773 ZMIZ1 -0.299095 -0.457055 4.57E-14 0.654396 -1.52813 0 down vs 1 8.08988 65.4461 0.45239 1.46543 1cg02747103 HCN2 0.222955 0.386023 4.58E-14 0.577569 -1.7314 0 down vs 1 -8.08968 65.4429 0.482121 1.56182 1cg04523661 0.182225 0.433734 4.58E-14 0.420131 -2.38021 0 down vs 1 -8.08952 65.4403 1.1469 3.71549 1cg11080731 0.19602 0.379741 4.60E-14 0.516194 -1.93725 0 down vs 1 -8.08901 65.4321 0.611976 1.9828 1cg17321639 0.25075 0.440406 4.61E-14 0.569361 -1.75635 0 down vs 1 -8.08858 65.4252 0.652153 2.1132 1cg06439941 GNG11 0.19401 0.371472 4.63E-14 0.522274 -1.9147 0 down vs 1 -8.08778 65.4122 0.570988 1.85056 1cg19909974 ADARB2 0.055965 0.334104 4.65E-14 0.167508 -5.96988 0 down vs 1 -8.08729 65.4042 1.40263 4.54645 1cg06417426 0.01702 0.272835 4.65E-14 0.062382 -16.0303 0 down vs 1 -8.08716 65.4022 1.18651 3.84604 1cg27077950 -0.14597 0.0838557 4.68E-14 -1.74073 1.74073 0 up vs 1 -8.08637 65.3894 0.957668 3.10487 1cg21269897 HIST1H2BK -0.16257 -0.412035 4.68E-14 0.394554 -2.53451 0 down vs 1 8.08635 65.389 1.12833 3.65821 1cg25580825 COL5A3 0.00594 0.251009 4.70E-14 0.0236645 -42.2575 0 down vs 1 -8.08546 65.3746 1.08892 3.5312 1cg08528444 WNT5B 0.291345 0.412292 4.70E-14 0.706647 -1.41513 0 down vs 1 -8.0854 65.3738 0.265222 0.860087 1cg10156366 -0.246845 0.18293 4.70E-14 -1.34939 1.34939 0 up vs 1 -8.08537 65.3732 3.34889 10.8602 1cg02821998 LETM1 0.105275 0.279884 4.72E-14 0.376138 -2.6586 0 down vs 1 -8.08492 65.3659 0.552779 1.79282 1cg15175129 MSRB3 -0.18054 0.129471 4.74E-14 -1.39444 1.39444 0 up vs 1 -8.0842 65.3543 1.7425 5.65242 1cg17594047 0.2323 0.426314 4.74E-14 0.544903 -1.83519 0 down vs 1 -8.08404 65.3517 0.682474 2.21393 1cg24346629 -0.32666 0.00722474 4.77E-14 -45.2141 45.2141 0 up vs 1 -8.08312 65.3368 2.02121 6.55827 1cg07979388 PALLD 0.313235 0.449349 4.79E-14 0.697086 -1.43454 0 down vs 1 -8.08243 65.3257 0.33591 1.09012 1cg01847441 ACTN1 0.223215 0.373197 4.80E-14 0.598116 -1.67192 0 down vs 1 -8.08232 65.3238 0.407846 1.32361 1cg02863073 SYCN -0.237535 0.0967701 4.80E-14 -2.45463 2.45463 0 up vs 1 -8.08219 65.3217 2.0263 6.57631 1cg23352722 PHYHIP -0.18253 0.112283 4.82E-14 -1.62562 1.62562 0 up vs 1 -8.08158 65.3119 1.57584 5.11511 1cg14441976 SLC27A6 -0.3633 0.0496835 4.83E-14 -7.31229 7.31229 0 up vs 1 -8.08132 65.3077 3.09231 10.0382 1cg07291439 ZIC1 -0.119205 0.199319 4.84E-14 -0.598061 -1.67207 0 down vs 1 -8.08083 65.2998 1.83951 5.9721 1cg14069088 CDKN2BAS -0.073475 -0.335871 4.85E-14 0.218759 -4.57123 0 down vs 1 8.0806 65.2961 1.24834 4.05304 1cg01797106 GLT1D1 -0.0332 0.299389 4.85E-14 -0.110892 -9.01775 0 down vs 1 -8.08043 65.2934 2.00555 6.5118 1cg02464866 -0.011535 0.244884 4.86E-14 -0.047104 -21.2296 0 down vs 1 -8.08015 65.2888 1.19211 3.87092 1cg15155051 ZNF311 0.079025 0.203238 4.88E-14 0.38883 -2.57182 0 down vs 1 -8.0796 65.28 0.279739 0.908466 1cg24672116 0.06464 0.338209 4.89E-14 0.191124 -5.2322 0 down vs 1 -8.07904 65.2709 1.35691 4.40726 1cg07982896 CACNA1A -0.03476 0.305773 4.90E-14 -0.113679 -8.7967 0 down vs 1 -8.0789 65.2687 2.1025 6.82917 1cg18789958 HCN1 -0.16031 0.213917 4.91E-14 -0.749403 -1.3344 0 down vs 1 -8.07849 65.262 2.53915 8.24828 1cg16320885 LCE5A 0.18217 0.35626 4.93E-14 0.51134 -1.95565 0 down vs 1 -8.07803 65.2545 0.549499 1.78522 1cg21801428 0.21969 0.388057 4.93E-14 0.566128 -1.76639 0 down vs 1 -8.07795 65.2533 0.513964 1.66981 1cg24239690 FSCN1 -0.23951 -0.369374 4.95E-14 0.648421 -1.54221 0 down vs 1 8.07712 65.2398 0.305772 0.993619 1cg00074746 FAM26F 0.04144 0.306171 4.96E-14 0.135349 -7.3883 0 down vs 1 -8.07694 65.2369 1.27066 4.12924 1cg00747241 ASPA 0.12217 0.354109 4.97E-14 0.345007 -2.89849 0 down vs 1 -8.07653 65.2303 0.975359 3.16994 1cg27284194 0.02827 0.291293 4.99E-14 0.0970501 -10.304 0 down vs 1 -8.07591 65.2203 1.25431 4.07716 1cg23811057 -0.221225 -0.394446 5.00E-14 0.56085 -1.78301 0 down vs 1 8.07557 65.2148 0.544024 1.76851 1cg11335133 LRRC8D -0.161565 -0.431645 5.00E-14 0.3743 -2.67165 0 down vs 1 8.07552 65.214 1.32253 4.29932 1cg07529353 0.174195 0.352904 5.02E-14 0.493604 -2.02591 0 down vs 1 -8.075 65.2056 0.579044 1.88262 1cg16638540 ZNF135 -0.137945 0.112619 5.03E-14 -1.22489 1.22489 0 up vs 1 -8.07471 65.2009 1.13829 3.70114 1cg21913897 AJAP1 0.15194 0.409929 5.05E-14 0.370649 -2.69797 0 down vs 1 -8.07407 65.1906 1.20676 3.92439 1cg14435059 0.324695 0.469674 5.05E-14 0.69132 -1.44651 0 down vs 1 -8.07403 65.1899 0.381089 1.23932 1cg12037324 NXPH2 0.04757 0.297371 5.05E-14 0.159969 -6.25122 0 down vs 1 -8.07401 65.1897 1.13137 3.67927 1cg02855740 0.203395 0.387362 5.09E-14 0.525078 -1.90448 0 down vs 1 -8.07293 65.1722 0.613617 1.99605 1cg24850323 0.35146 0.468484 5.10E-14 0.750208 -1.33296 0 down vs 1 -8.07252 65.1656 0.248293 0.807758 1cg16305516 DUSP22 0.11862 0.296133 5.15E-14 0.400563 -2.49648 0 down vs 1 -8.07091 65.1396 0.571318 1.85938 1cg22070991 -0.293445 0.119199 5.17E-14 -2.4618 2.4618 0 up vs 1 -8.07025 65.1289 3.08724 10.0492 1cg01655427 LEF1 -0.124125 0.224844 5.18E-14 -0.552049 -1.81143 0 down vs 1 -8.07005 65.1257 2.20797 7.18747 1cg12014886 TCERG1L 0.352485 0.448267 5.19E-14 0.786328 -1.27173 0 down vs 1 -8.06959 65.1183 0.166336 0.541525 1cg19723091 HDAC4 0.290785 0.390509 5.20E-14 0.744631 -1.34295 0 down vs 1 -8.06932 65.1138 0.180308 0.587054 1cg06057566 EDNRB -0.242655 0.0392052 5.23E-14 -6.18936 6.18936 0 up vs 1 -8.06839 65.0989 1.44041 4.69081 1cg26578983 KIAA0513 0.110505 0.392208 5.24E-14 0.281751 -3.54923 0 down vs 1 -8.06807 65.0938 1.4388 4.68594 1cg27273310 EBF3 0.14937 0.389784 5.25E-14 0.383213 -2.60952 0 down vs 1 -8.06786 65.0904 1.04794 3.41314 1cg14676666 0.10223 0.323042 5.26E-14 0.316461 -3.15995 0 down vs 1 -8.06765 65.087 0.88402 2.87941 1cg00348802 PTPRN2 -0.115545 0.205724 5.27E-14 -0.561651 -1.78046 0 down vs 1 -8.06721 65.0799 1.87135 6.09599 1cg14457256 ACSL4 0.120025 0.288498 5.27E-14 0.416034 -2.40365 0 down vs 1 -8.0672 65.0797 0.51461 1.67636 1cg11310950 -0.360585 0.00232629 5.29E-14 -155.004 155.004 0 up vs 1 -8.06659 65.0698 2.38792 7.77992 1cg10469980 KIAA0182 0.078085 0.278254 5.30E-14 0.280625 -3.56348 0 down vs 1 -8.06626 65.0646 0.726461 2.36703 1cg13850887 SNED1 0.283585 0.410415 5.32E-14 0.690971 -1.44724 0 down vs 1 -8.0657 65.0555 0.29165 0.950415 1cg04660829 0.224885 0.35099 5.32E-14 0.640716 -1.56075 0 down vs 1 -8.06563 65.0544 0.288326 0.9396 1cg19954602 FILIP1 -0.06974 0.270339 5.33E-14 -0.257973 -3.87638 0 down vs 1 -8.06538 65.0503 2.0969 6.83381 1cg03542686 CAPZB 0.034365 0.18392 5.35E-14 0.186848 -5.35194 0 down vs 1 -8.06492 65.0429 0.405525 1.32176 1cg13298841 HECW1 -0.20133 0.181951 5.37E-14 -1.10651 1.10651 0 up vs 1 -8.06423 65.0319 2.66349 8.68282 1cg23534802 SCAND3 0.04294 0.271831 5.38E-14 0.157966 -6.33049 0 down vs 1 -8.06403 65.0286 0.949898 3.09677 1cg24797665 CIT 0.167465 0.347034 5.39E-14 0.482561 -2.07227 0 down vs 1 -8.06379 65.0247 0.584626 1.90606 1cg08557523 ACCN4 0.24646 0.374502 5.39E-14 0.658101 -1.51952 0 down vs 1 -8.06363 65.0221 0.297249 0.969157 1cg25616829 0.16963 0.410878 5.39E-14 0.412848 -2.4222 0 down vs 1 -8.06361 65.0219 1.05522 3.4405 1cg14523810 ZNF583 -0.373565 -0.0445814 5.45E-14 8.37938 8.37938 0 up vs 1 -8.06182 64.9929 1.9623 6.40083 1cg21088983 CLVS1 -0.151285 0.180895 5.47E-14 -0.836315 -1.19572 0 down vs 1 -8.06134 64.9851 2.00062 6.52659 1cg00881487 MXRA5 0.063245 0.324171 5.47E-14 0.195098 -5.12564 0 down vs 1 -8.06128 64.9842 1.23439 4.027 1cg14386202 -0.20815 0.165431 5.48E-14 -1.25823 1.25823 0 up vs 1 -8.06097 64.9792 2.5304 8.25562 1cg05890785 -0.309475 0.027151 5.49E-14 -11.3983 11.3983 0 up vs 1 -8.06084 64.9772 2.05453 6.70329 1cg20816699 0.16215 0.392011 5.49E-14 0.413637 -2.41758 0 down vs 1 -8.06069 64.9748 0.957961 3.12564 1cg19457237 0.085795 0.374367 5.49E-14 0.229174 -4.36351 0 down vs 1 -8.06064 64.974 1.50982 4.92632 1cg20062057 NRXN1 -0.279565 0.0365258 5.50E-14 -7.65391 7.65391 0 up vs 1 -8.06051 64.9719 1.81151 5.91088 1cg12700271 HLA-E -0.167475 -0.340576 5.50E-14 0.49174 -2.03359 0 down vs 1 8.06048 64.9713 0.543274 1.77269 1cg21678377 DPP10 -0.196945 0.156252 5.53E-14 -1.26044 1.26044 0 up vs 1 -8.05943 64.9544 2.26178 7.38207 1cg10911747 0.27973 0.424331 5.54E-14 0.659226 -1.51693 0 down vs 1 -8.05937 64.9535 0.379106 1.23735 1cg12755421 PRKCDBP -0.149225 -0.378189 5.54E-14 0.394578 -2.53435 0 down vs 1 8.05937 64.9535 0.950498 3.1023 1cg02123616 0.047525 0.349063 5.54E-14 0.13615 -7.34484 0 down vs 1 -8.05925 64.9515 1.64855 5.38084 1cg05876246 -0.0125 -0.354899 5.54E-14 0.0352213 -28.3919 0 down vs 1 8.05919 64.9506 2.12561 6.93802 1cg19885761 CPLX2 -0.33032 0.063683 5.55E-14 -5.18694 5.18694 0 up vs 1 -8.05913 64.9496 2.8146 9.18706 1cg01254991 -0.06434 0.191258 5.55E-14 -0.336404 -2.97262 0 down vs 1 -8.05896 64.9468 1.1845 3.86645 1cg12874792 RAD51L1 0.343355 0.465851 5.59E-14 0.73705 -1.35676 0 down vs 1 -8.05796 64.9307 0.272056 0.888269 1cg00889599 C10orf140 0.191245 0.391349 5.61E-14 0.488681 -2.04632 0 down vs 1 -8.05731 64.9202 0.725988 2.37075 1cg09052548 CUGBP2 0.080545 0.354976 5.65E-14 0.226902 -4.40718 0 down vs 1 -8.05619 64.9023 1.36548 4.46027 1cg11214001 CAST 0.19531 0.358974 5.68E-14 0.544079 -1.83797 0 down vs 1 -8.05541 64.8897 0.485649 1.58666 1cg06198384 -0.2708 0.127001 5.68E-14 -2.13227 2.13227 0 up vs 1 -8.0554 64.8895 2.86912 9.37367 1cg14716058 TANC1 0.34719 0.425656 5.69E-14 0.815658 -1.226 0 down vs 1 -8.05514 64.8853 0.111631 0.364731 1cg18287067 KIF26A 0.020525 0.335577 5.69E-14 0.0611633 -16.3497 0 down vs 1 -8.05513 64.8851 1.79962 5.87994 1cg03272310 -0.23547 -0.405354 5.70E-14 0.5809 -1.72147 0 down vs 1 8.05468 64.8778 0.523264 1.70986 1cg18706476 MST1R 0.32137 -0.0233526 5.70E-14 -13.7617 13.7617 0 up vs 1 8.05464 64.8772 2.15455 7.04046 1cg15159149 0.29482 0.448428 5.72E-14 0.657452 -1.52102 0 down vs 1 -8.05408 64.8683 0.427807 1.39814 1cg26175448 -0.27977 -0.410488 5.73E-14 0.681554 -1.46723 0 down vs 1 8.05399 64.8668 0.309806 1.01252 1cg05460949 LYPLAL1 -0.257765 -0.446455 5.75E-14 0.57736 -1.73202 0 down vs 1 8.05326 64.8549 0.645526 2.11012 1cg00655094 0.062535 0.322829 5.76E-14 0.193709 -5.16237 0 down vs 1 -8.05317 64.8535 1.22842 4.01558 1cg27436286 IGDCC3 -0.260115 0.0567005 5.77E-14 -4.58752 4.58752 0 up vs 1 -8.05291 64.8494 1.81983 5.94923 1cg05525374 HLA-DMB 0.10945 0.362569 5.77E-14 0.301874 -3.31264 0 down vs 1 -8.05269 64.8458 1.16162 3.79769 1cg11570931 FOXP1 0.295955 0.406127 5.78E-14 0.728725 -1.37226 0 down vs 1 -8.05254 64.8434 0.220071 0.719504 1cg23845153 0.24294 0.379218 5.78E-14 0.640634 -1.56095 0 down vs 1 -8.05247 64.8423 0.336721 1.1009 1cg03530983 HLA-DOB 0.16148 0.391504 5.79E-14 0.412461 -2.42447 0 down vs 1 -8.05214 64.837 0.959323 3.13673 1cg13714832 SH2D3A 0.32607 0.433621 5.81E-14 0.75197 -1.32984 0 down vs 1 -8.05166 64.8292 0.209724 0.685824 1cg24996814 -0.180835 0.146807 5.82E-14 -1.23179 1.23179 0 up vs 1 -8.05151 64.8268 1.94634 6.36501 1cg08606493 PTK2B 0.23753 0.408064 5.83E-14 0.58209 -1.71795 0 down vs 1 -8.05126 64.8228 0.527278 1.72444 1cg02469871 -0.17463 0.108171 5.83E-14 -1.61439 1.61439 0 up vs 1 -8.05117 64.8214 1.45004 4.7424 1cg01889143 -0.21896 0.144984 5.83E-14 -1.51024 1.51024 0 up vs 1 -8.05109 64.8201 2.40152 7.85438 1cg12494529 C10orf110 0.142505 -0.170753 5.83E-14 -0.834568 -1.19823 0 down vs 1 8.05108 64.8199 1.77919 5.81902 1cg13685349 0.348784 0.441422 5.83E-14 0.790138 -1.2656 0 down vs 1 -8.07644 65.2289 0.147941 0.460411 1cg15486123 IVL -0.00399 0.303013 5.84E-14 -0.0131678 -75.9431 0 down vs 1 -8.05093 64.8175 1.70885 5.58915 1cg21054194 0.065985 0.332719 5.85E-14 0.198321 -5.04234 0 down vs 1 -8.05047 64.81 1.28995 4.21956 1cg09367901 BMP4 0.022625 0.334179 5.87E-14 0.0677033 -14.7703 0 down vs 1 -8.04999 64.8023 1.75988 5.75744 1cg04165508 0.17017 -0.130553 5.87E-14 -1.30346 1.30346 0 up vs 1 8.04993 64.8013 1.63965 5.36416 1cg07078735 0.19943 0.363669 5.90E-14 0.548383 -1.82354 0 down vs 1 -8.04911 64.7882 0.48907 1.60034 1cg18032014 DYNC1I1 -0.25439 0.110588 5.94E-14 -2.30034 2.30034 0 up vs 1 -8.04811 64.7721 2.41519 7.90496 1cg26998274 SLITRK1 -0.128045 0.236399 5.96E-14 -0.541648 -1.84622 0 down vs 1 -8.0475 64.7622 2.40813 7.88304 1cg12196045 MAP1LC3B2 -0.107095 -0.358888 5.97E-14 0.298408 -3.35112 0 down vs 1 8.04744 64.7613 1.14949 3.76293 1cg16185299 -0.0984 0.234694 5.97E-14 -0.419269 -2.3851 0 down vs 1 -8.04738 64.7603 2.01165 6.58536 1cg07622833 C11orf41 0.19692 0.428264 5.98E-14 0.45981 -2.17481 0 down vs 1 -8.04712 64.7561 0.970363 3.1768 1cg21074413 GPR133 0.152905 0.383872 5.98E-14 0.398323 -2.51052 0 down vs 1 -8.04701 64.7544 0.9672 3.16653 1cg22878441 -0.25583 0.122362 5.99E-14 -2.09076 2.09076 0 up vs 1 -8.04689 64.7524 2.59324 8.4903 1cg25832180 MAGI2 0.14528 0.374854 6.00E-14 0.387564 -2.58022 0 down vs 1 -8.04643 64.7451 0.955573 3.12891 1cg25520040 LOC283174 0.15233 0.356171 6.10E-14 0.427688 -2.33815 0 down vs 1 -8.04389 64.7042 0.753355 2.46833 1cg00800896 0.17652 0.376859 6.11E-14 0.468398 -2.13494 0 down vs 1 -8.04358 64.6992 0.727696 2.38444 1cg17220278 PRDM16 0.112305 0.424693 6.13E-14 0.264438 -3.7816 0 down vs 1 -8.04299 64.6897 1.76932 5.79838 1cg23457258 BCL11B 0.195085 0.367712 6.14E-14 0.530538 -1.88488 0 down vs 1 -8.04297 64.6893 0.5403 1.77067 1cg26697310 ANK1 0.145725 0.330034 6.14E-14 0.441545 -2.26477 0 down vs 1 -8.04285 64.6874 0.615901 2.01849 1cg03654841 0.272855 0.432919 6.16E-14 0.630268 -1.58663 0 down vs 1 -8.04243 64.6806 0.464521 1.52254 1cg13005636 0.194215 0.417557 6.16E-14 0.465122 -2.14997 0 down vs 1 -8.04237 64.6797 0.904394 2.96432 1cg04387396 SGPL1 -0.249185 -0.424305 6.22E-14 0.587279 -1.70277 0 down vs 1 8.04078 64.6542 0.556016 1.82317 1cg12264626 CA3 -0.06343 0.19244 6.23E-14 -0.32961 -3.03389 0 down vs 1 -8.04057 64.6507 1.18701 3.8924 1cg14385804 MFF 0.108675 0.318175 6.26E-14 0.341558 -2.92776 0 down vs 1 -8.0398 64.6384 0.795765 2.60994 1cg16726706 0.153155 0.39388 6.26E-14 0.388837 -2.57177 0 down vs 1 -8.03978 64.6381 1.05065 3.44594 1cg12443213 SORCS2 0.04917 0.273033 6.27E-14 0.180088 -5.55284 0 down vs 1 -8.03954 64.6342 0.908621 2.98027 1cg18322516 PROKR2 0.128955 0.361467 6.27E-14 0.356755 -2.80305 0 down vs 1 -8.03939 64.6318 0.980187 3.21513 1cg00606880 TMEM26 -8.50E-05 0.286499 6.33E-14 -0.000296685 -3370.58 0 down vs 1 -8.03798 64.6091 1.48909 4.88612 1cg15082983 0.077095 0.279819 6.35E-14 0.275518 -3.62953 0 down vs 1 -8.03756 64.6024 0.74512 2.4452 1cg10281977 TMEM179 -0.29338 0.106807 6.36E-14 -2.74683 2.74683 0 up vs 1 -8.03714 64.5957 2.90364 9.52962 1cg19472254 ATP8B3 0.088965 0.315262 6.37E-14 0.282194 -3.54367 0 down vs 1 -8.03685 64.591 0.928489 3.04748 1cg07022634 0.01681 0.228045 6.38E-14 0.0737134 -13.5661 0 down vs 1 -8.0368 64.5902 0.809005 2.65534 1cg20374317 TMEM132C 0.137065 0.377775 6.38E-14 0.362822 -2.75617 0 down vs 1 -8.03671 64.5886 1.05052 3.44814 1cg08028901 0.049175 0.354947 6.39E-14 0.138542 -7.21804 0 down vs 1 -8.03653 64.5858 1.69517 5.56432 1cg16035036 0.028115 0.402049 6.40E-14 0.0699293 -14.3002 0 down vs 1 -8.0363 64.5821 2.53517 8.32208 1cg22004089 0.226635 0.393635 6.41E-14 0.57575 -1.73687 0 down vs 1 -8.03594 64.5763 0.505648 1.66001 1cg20359994 -0.27995 0.0394582 6.42E-14 -7.09484 7.09484 0 up vs 1 -8.03572 64.5729 1.84974 6.0729 1cg13434989 EDNRB -0.226225 0.133196 6.43E-14 -1.69844 1.69844 0 up vs 1 -8.03552 64.5695 2.3422 7.69012 1cg25003366 0.089115 0.345292 6.45E-14 0.258086 -3.87468 0 down vs 1 -8.03506 64.5621 1.18987 3.90712 1cg05921170 S100A12 0.201645 0.437855 6.45E-14 0.460529 -2.17142 0 down vs 1 -8.03498 64.5609 1.01161 3.32186 1cg27486812 RFX2 0.22796 0.438019 6.49E-14 0.520434 -1.92147 0 down vs 1 -8.03401 64.5453 0.80002 2.62768 1cg14333520 ABLIM2 0.1811 0.36811 6.49E-14 0.491973 -2.03263 0 down vs 1 -8.03391 64.5437 0.634084 2.08271 1cg15165154 CXXC5 -0.027565 -0.30761 6.51E-14 0.0896101 -11.1595 0 down vs 1 8.03355 64.5379 1.42192 4.67085 1cg14659930 ZBTB20 -0.09492 0.24382 6.51E-14 -0.389303 -2.56869 0 down vs 1 -8.03345 64.5363 2.08042 6.83412 1cg14914422 -0.132385 0.108284 6.53E-14 -1.22257 1.22257 0 up vs 1 -8.03294 64.5281 1.05017 3.45021 1cg00613752 GPR6 -0.11843 0.123973 6.55E-14 -0.955287 -1.04681 0 down vs 1 -8.03248 64.5207 1.06536 3.50051 1cg04091325 SH3GL3 0.09482 0.339564 6.56E-14 0.27924 -3.58115 0 down vs 1 -8.03214 64.5153 1.08603 3.56876 1cg10650853 -0.1317 0.172297 6.60E-14 -0.764376 -1.30826 0 down vs 1 -8.03131 64.502 1.67555 5.50707 1cg07972015 -0.060415 0.273804 6.66E-14 -0.22065 -4.53206 0 down vs 1 -8.02976 64.477 2.02526 6.65904 1cg02465887 DCHS2 0.283555 0.428399 6.69E-14 0.661894 -1.51082 0 down vs 1 -8.02914 64.4671 0.380384 1.25089 1cg02981992 COL19A1 -0.36225 0.047749 6.71E-14 -7.58655 7.58655 0 up vs 1 -8.02855 64.4576 3.04778 10.0241 1cg17133833 PSD3 0.29779 0.419158 6.76E-14 0.710448 -1.40756 0 down vs 1 -8.02754 64.4415 0.267072 0.878615 1cg09127540 PTPRN2 -0.072545 0.135676 6.76E-14 -0.534694 -1.87023 0 down vs 1 -8.02742 64.4395 0.786079 2.58613 1cg03549227 0.18808 0.390437 6.78E-14 0.481717 -2.07591 0 down vs 1 -8.02702 64.433 0.742429 2.44277 1cg20042908 0.02868 0.396238 6.79E-14 0.0723807 -13.8158 0 down vs 1 -8.02941 64.4715 2.44828 8.01263 1cg19224787 THRB 0.28776 0.423887 6.79E-14 0.678861 -1.47306 0 down vs 1 -8.02668 64.4276 0.335973 1.10552 1cg25153629 PRDM16 0.15362 0.371365 6.83E-14 0.413663 -2.41743 0 down vs 1 -8.02575 64.4127 0.859639 2.82931 1cg14978172 ELFN1 0.31877 0.453971 6.83E-14 0.702182 -1.42413 0 down vs 1 -8.0257 64.4119 0.331417 1.0908 1cg12622027 TCP10 -0.040025 0.277619 6.84E-14 -0.144173 -6.93613 0 down vs 1 -8.02563 64.4108 1.82936 6.02109 1cg22930275 TARSL2 -0.20899 0.158067 6.84E-14 -1.32216 1.32216 0 up vs 1 -8.02555 64.4095 2.44278 8.04027 1cg00873601 0.01874 0.310138 6.85E-14 0.0604248 -16.5495 0 down vs 1 -8.02542 64.4073 1.53954 5.06746 1cg09007470 PPP1R14A -0.11794 0.123179 6.85E-14 -0.957469 -1.04442 0 down vs 1 -8.02538 64.4067 1.0541 3.46965 1cg13598109 TLE4 0.05233 0.343911 6.85E-14 0.152162 -6.57196 0 down vs 1 -8.02529 64.4053 1.54147 5.074 1cg02960938 -0.06335 0.199199 6.85E-14 -0.318024 -3.14442 0 down vs 1 -8.02523 64.4043 1.24979 4.11396 1cg25868286 TMEM101 -0.12695 -0.390873 6.86E-14 0.324786 -3.07895 0 down vs 1 8.02508 64.4019 1.26291 4.15729 1cg05379541 -0.18154 0.135325 6.88E-14 -1.34151 1.34151 0 up vs 1 -8.02469 64.3956 1.8204 5.99304 1cg08970112 KRT25 0.18084 0.376494 6.93E-14 0.480327 -2.08192 0 down vs 1 -8.02359 64.378 0.694056 2.28556 1cg23873703 KCNAB1 0.038635 0.287792 6.94E-14 0.134246 -7.44899 0 down vs 1 -8.02327 64.3729 1.12555 3.70677 1cg23449696 ZIC1 -0.119345 0.234694 6.96E-14 -0.508512 -1.96652 0 down vs 1 -8.02285 64.3662 2.27259 7.48513 1cg08822417 ADARB2 0.050935 0.39096 6.96E-14 0.130282 -7.67567 0 down vs 1 -8.02273 64.3641 2.09624 6.9045 1cg10264692 0.070185 0.321258 6.97E-14 0.218469 -4.5773 0 down vs 1 -8.02257 64.3616 1.14292 3.76466 1cg24293948 0.09178 0.294442 6.97E-14 0.311709 -3.20813 0 down vs 1 -8.02247 64.3601 0.744666 2.45291 1cg02022733 PCDHGA4 -0.1638 0.17326 6.98E-14 -0.945398 -1.05776 0 down vs 1 -8.02244 64.3596 2.05984 6.78509 1cg17510385 TRIP13 0.22794 -0.224272 6.98E-14 -1.01635 1.01635 0 up vs 1 8.02237 64.3583 3.70768 12.2133 1cg10901805 DLG2 0.174475 0.378273 6.98E-14 0.461241 -2.16806 0 down vs 1 -8.0223 64.3573 0.753037 2.48059 1cg04617755 DPP10 -0.30617 0.0180387 6.99E-14 -16.973 16.973 0 up vs 1 -8.02208 64.3537 1.90576 6.27812 1cg05062446 LYPLAL1 -0.32953 -0.458902 7.02E-14 0.718084 -1.3926 0 down vs 1 8.02144 64.3435 0.303458 0.999838 1cg05056901 -0.02868 0.273885 7.03E-14 -0.104715 -9.54969 0 down vs 1 -8.02115 64.3388 1.6598 5.46913 1cg08368617 -0.152275 0.228055 7.04E-14 -0.667713 -1.49765 0 down vs 1 -8.02085 64.3341 2.62264 8.64237 1cg01021245 SLC16A14 0.246235 0.381527 7.06E-14 0.645393 -1.54944 0 down vs 1 -8.02061 64.3302 0.331867 1.09367 1cg02866106 CHRM2 0.04502 0.308665 7.08E-14 0.145854 -6.85619 0 down vs 1 -8.02015 64.3228 1.26026 4.15364 1cg18731680 COMT 0.130225 0.306833 7.08E-14 0.424417 -2.35618 0 down vs 1 -8.02002 64.3207 0.565508 1.8639 1cg27472176 PXDN 0.15604 0.373312 7.10E-14 0.417988 -2.39241 0 down vs 1 -8.01961 64.3142 0.855908 2.82134 1cg22926923 0.051775 0.350394 7.13E-14 0.147762 -6.76763 0 down vs 1 -8.01895 64.3035 1.61679 5.33032 1cg15147534 -0.00691 0.30166 7.14E-14 -0.0229066 -43.6556 0 down vs 1 -8.01872 64.2999 1.72634 5.69182 1cg03550129 -0.08565 0.209661 7.16E-14 -0.408516 -2.44788 0 down vs 1 -8.01828 64.2927 1.58117 5.21377 1cg24314288 0.35553 0.432118 7.17E-14 0.822762 -1.21542 0 down vs 1 -8.0181 64.2899 0.106349 0.350693 1cg02168857 EPHA7 -0.29165 0.134568 7.18E-14 -2.16731 2.16731 0 up vs 1 -8.01791 64.2868 3.29367 10.8616 1cg02987906 FIP1L1 -0.08989 0.243445 7.18E-14 -0.369241 -2.70826 0 down vs 1 -8.01786 64.286 2.01456 6.64355 1cg13133304 TNNI2 0.193365 0.433279 7.18E-14 0.446282 -2.24073 0 down vs 1 -8.0178 64.2851 1.04359 3.44157 1cg15999188 0.02242 0.389615 7.18E-14 0.0575439 -17.378 0 down vs 1 -8.01772 64.2838 2.44463 8.06208 1cg04157268 CACNA1H 0.229465 0.42476 7.19E-14 0.540222 -1.85109 0 down vs 1 -8.0176 64.2819 0.691515 2.2806 1cg20853943 0.065025 0.311694 7.25E-14 0.208618 -4.79345 0 down vs 1 -8.01627 64.2606 1.10318 3.63948 1cg04087742 -0.030505 0.262035 7.28E-14 -0.116416 -8.58991 0 down vs 1 -8.01833 64.2937 1.55088 5.08971 1cg20335213 ARHGAP29 0.102785 0.322191 7.29E-14 0.319019 -3.13461 0 down vs 1 -8.01541 64.2469 0.872798 2.88004 1cg23914380 TBXA2R 0.14909 0.387517 7.31E-14 0.384731 -2.59922 0 down vs 1 -8.01494 64.2392 1.03069 3.40145 1cg11251200 PTPRN2 0.11516 0.266483 7.35E-14 0.432148 -2.31402 0 down vs 1 -8.01402 64.2246 0.415172 1.37045 1cg17494781 HSPA1A -0.22942 -0.469309 7.36E-14 0.488847 -2.04563 0 down vs 1 8.01393 64.2231 1.04337 3.44415 1cg12666279 DPP10 -0.141215 0.204928 7.38E-14 -0.689096 -1.45118 0 down vs 1 -8.01348 64.2159 2.17234 7.17169 1cg22579691 ATP4A 0.00015 0.287548 7.40E-14 0.000521651 -1916.99 0 down vs 1 -8.01292 64.2069 1.49757 4.94471 1cg22366951 HIVEP3 0.155425 0.374341 7.43E-14 0.415196 -2.4085 0 down vs 1 -8.01243 64.199 0.868908 2.86934 1cg20607331 ZNF549 -0.408955 0.027301 7.46E-14 -14.9795 14.9795 0 up vs 1 -8.01177 64.1885 3.45065 11.3967 1cg17733702 KCNN3 0.08549 0.290369 7.46E-14 0.294418 -3.39653 0 down vs 1 -8.01174 64.188 0.76105 2.51359 1cg02599464 HIST1H2BK -0.110215 -0.41064 7.46E-14 0.268398 -3.72581 0 down vs 1 8.0116 64.1857 1.6364 5.40491 1cg12705352 0.314685 0.474422 7.48E-14 0.663302 -1.50761 0 down vs 1 -8.01122 64.1797 0.462626 1.52816 1cg05562386 C5orf36 0.238125 0.380378 7.49E-14 0.626021 -1.59739 0 down vs 1 -8.011 64.1761 0.366896 1.21201 1cg21244086 SHF -0.020495 0.231329 7.49E-14 -0.0885966 -11.2871 0 down vs 1 -8.01097 64.1757 1.14978 3.79821 1cg03951603 -0.089965 0.245519 7.50E-14 -0.366429 -2.72905 0 down vs 1 -8.01083 64.1734 2.04061 6.74126 1cg00268330 0.12539 0.377215 7.51E-14 0.33241 -3.00833 0 down vs 1 -8.01066 64.1707 1.14978 3.79852 1cg10614768 KNDC1 0.008705 0.29172 7.53E-14 0.0298403 -33.5117 0 down vs 1 -8.01024 64.164 1.45223 4.79822 1cg09076077 C20orf197 -0.09428 0.165461 7.57E-14 -0.569803 -1.75499 0 down vs 1 -8.00938 64.1501 1.2232 4.04238 1cg21600258 LEF1 -0.27064 0.10358 7.57E-14 -2.61285 2.61285 0 up vs 1 -8.00935 64.1497 2.53906 8.39101 1cg21959912 -0.15504 0.178597 7.58E-14 -0.8681 -1.15194 0 down vs 1 -8.00913 64.1462 2.01821 6.67008 1cg12384932 NALCN -0.07802 0.266146 7.59E-14 -0.293147 -3.41126 0 down vs 1 -8.00883 64.1413 2.14761 7.09828 1cg05941247 0.06633 0.341003 7.60E-14 0.194515 -5.141 0 down vs 1 -8.00879 64.1407 1.36788 4.52117 1cg08237117 CHFR 0.32109 0.392934 7.60E-14 0.81716 -1.22375 0 down vs 1 -8.00877 64.1403 0.0935835 0.309317 1cg03556653 -0.314475 0.106677 7.60E-14 -2.94792 2.94792 0 up vs 1 -8.00876 64.1402 3.21585 10.6292 1cg01054891 PEG3 -0.13924 0.127861 7.69E-14 -1.08899 1.08899 0 up vs 1 -8.00678 64.1085 1.29351 4.2775 1cg02227623 CTNNA2 -0.354515 -0.0614706 7.70E-14 5.76723 5.76723 0 up vs 1 -8.00652 64.1044 1.55699 5.14912 1cg18237323 PHYHIP -0.30261 0.0155943 7.73E-14 -19.4051 19.4051 0 up vs 1 -8.00608 64.0973 1.83582 6.07192 1cg23463533 DPP6 0.05811 0.342343 7.73E-14 0.169742 -5.89129 0 down vs 1 -8.00607 64.0972 1.46476 4.84466 1cg14137381 SLC9A3 -0.146435 0.24636 7.80E-14 -0.594394 -1.68239 0 down vs 1 -8.00459 64.0735 2.79737 9.25567 1cg17910813 MEIS3 -0.28746 0.136063 7.81E-14 -2.11269 2.11269 0 up vs 1 -8.0043 64.0688 3.25217 10.7612 1cg02647265 CCDC92 -0.189595 0.192096 7.82E-14 -0.986978 -1.01319 0 down vs 1 -8.00414 64.0662 2.64145 8.74077 1cg05285228 POT1 0.078585 0.308451 7.82E-14 0.254773 -3.92506 0 down vs 1 -8.00405 64.0648 0.958 3.17017 1cg10016380 EDNRB -0.261425 0.0889103 7.83E-14 -2.94032 2.94032 0 up vs 1 -8.00401 64.0642 2.22529 7.36388 1cg24848870 NAV2 0.17268 0.362726 7.83E-14 0.476061 -2.10057 0 down vs 1 -8.00393 64.063 0.654842 2.16703 1cg25071651 DDIT4L -0.229955 0.0871835 7.85E-14 -2.6376 2.6376 0 up vs 1 -8.00359 64.0574 1.82354 6.03507 1cg18404308 CNGA3 -0.32804 0.0145371 7.86E-14 -22.5657 22.5657 0 up vs 1 -8.00323 64.0517 2.12782 7.04271 1cg07338464 0.11622 0.340025 7.87E-14 0.341799 -2.9257 0 down vs 1 -8.0031 64.0496 0.908148 3.00591 1cg06616976 0.061135 0.222437 7.90E-14 0.274842 -3.63845 0 down vs 1 -8.00256 64.041 0.471732 1.56161 1cg22871870 TSPAN3 0.33001 0.416131 7.91E-14 0.793044 -1.26096 0 down vs 1 -8.0023 64.0368 0.134473 0.445186 1cg06059147 PADI1 0.310915 0.446648 7.91E-14 0.696108 -1.43656 0 down vs 1 -8.00224 64.0359 0.334032 1.10586 1cg20838818 0.048655 0.346475 7.94E-14 0.140428 -7.12106 0 down vs 1 -8.00167 64.0268 1.60815 5.32477 1cg04340567 SORCS2 0.280285 0.400137 7.94E-14 0.700473 -1.42761 0 down vs 1 -8.0016 64.0256 0.260439 0.862358 1cg18872497 -0.13235 0.232364 7.96E-14 -0.569581 -1.75568 0 down vs 1 -8.00933 64.1494 2.40816 7.84583 1cg02807192 -0.293285 0.0106943 7.97E-14 -27.4243 27.4243 0 up vs 1 -8.00117 64.0188 1.67535 5.54797 1cg27055156 0.081775 0.304781 7.97E-14 0.268307 -3.72707 0 down vs 1 -8.00105 64.0167 0.901677 2.98602 1cg26406394 PLCH2 0.180345 0.372555 7.99E-14 0.484077 -2.06579 0 down vs 1 -8.00062 64.01 0.669836 2.21848 1cg01844274 SYNE1 0.121875 0.35049 8.01E-14 0.347727 -2.87582 0 down vs 1 -8.00023 64.0037 0.947607 3.13877 1cg20535723 KIF20B 0.22649 0.379508 8.06E-14 0.596799 -1.67561 0 down vs 1 -7.99937 63.99 0.424526 1.40646 1cg03054050 ZDHHC14 0.329165 0.406774 8.06E-14 0.809208 -1.23578 0 down vs 1 -7.9992 63.9872 0.109206 0.361816 1cg00878953 0.24225 0.385859 8.07E-14 0.62782 -1.59281 0 down vs 1 -7.99914 63.9863 0.373924 1.23889 1cg19350679 HLA-DQA2 0.05597 0.291542 8.08E-14 0.191979 -5.2089 0 down vs 1 -7.99887 63.9819 1.00616 3.33384 1cg11052516 LOC645323 -0.40995 -0.0724593 8.09E-14 5.65766 5.65766 0 up vs 1 -7.99871 63.9793 2.0651 6.84286 1cg25724895 C20orf197 -0.02398 0.186397 8.10E-14 -0.12865 -7.77302 0 down vs 1 -7.99857 63.9771 0.802443 2.65904 1cg06922248 -0.01863 0.331894 8.10E-14 -0.0561323 -17.815 0 down vs 1 -7.99839 63.9742 2.22769 7.38219 1cg16272710 OSMR 0.19833 0.370209 8.13E-14 0.535724 -1.86663 0 down vs 1 -7.99796 63.9674 0.53563 1.77518 1cg14223966 -0.25544 0.127473 8.13E-14 -2.00388 2.00388 0 up vs 1 -7.9979 63.9665 2.65838 8.81051 1cg24451653 SUFU 0.220425 0.313774 8.14E-14 0.702497 -1.42349 0 down vs 1 -7.99764 63.9623 0.157992 0.523657 1cg13909178 -0.000575 0.302821 8.15E-14 -0.00189881 -526.645 0 down vs 1 -7.99758 63.9613 1.66893 5.53167 1cg23239891 ACTL9 0.310485 0.463083 8.19E-14 0.670474 -1.49148 0 down vs 1 -7.99679 63.9486 0.422197 1.39965 1cg22242585 0.17817 0.337823 8.20E-14 0.527406 -1.89607 0 down vs 1 -7.99658 63.9453 0.46214 1.53215 1cg15665928 -0.07236 0.210086 8.22E-14 -0.34443 -2.90335 0 down vs 1 -7.99618 63.9389 1.4464 4.79578 1cg03865667 PCDH17 -0.19125 0.0834469 8.22E-14 -2.29188 2.29188 0 up vs 1 -7.99612 63.9379 1.36812 4.53631 1cg05184767 0.07994 0.351757 8.22E-14 0.227259 -4.40026 0 down vs 1 -7.99611 63.9378 1.33958 4.44169 1cg21792399 0.12245 0.345891 8.27E-14 0.354013 -2.82475 0 down vs 1 -7.9951 63.9216 0.9052 3.00216 1cg16409049 AK5 -0.330435 0.0265392 8.30E-14 -12.4508 12.4508 0 up vs 1 -7.9946 63.9136 2.31042 7.66362 1cg17289451 0.050495 0.348454 8.30E-14 0.144912 -6.90075 0 down vs 1 -7.99451 63.9121 1.60964 5.33928 1cg06851122 0.037775 0.331339 8.31E-14 0.114007 -8.77137 0 down vs 1 -7.99439 63.9103 1.56251 5.18307 1cg04486019 -0.25097 0.138485 8.32E-14 -1.81225 1.81225 0 up vs 1 -7.99424 63.9079 2.75 9.1225 1cg18458352 LOC285830 0.002005 0.256271 8.34E-14 0.00782376 -127.816 0 down vs 1 -7.99378 63.9005 1.17218 3.88888 1cg09354454 -0.15598 0.0718871 8.35E-14 -2.16979 2.16979 0 up vs 1 -7.99357 63.8972 0.941415 3.12345 1cg11831981 SCG3 -0.39686 -0.0157041 8.35E-14 25.2711 25.2711 0 up vs 1 -7.99352 63.8963 2.63405 8.73943 1cg27299021 ADARB2 0.213945 0.375892 8.37E-14 0.569166 -1.75696 0 down vs 1 -7.99321 63.8914 0.475516 1.57782 1cg25633284 HCN2 0.177565 0.409892 8.37E-14 0.4332 -2.3084 0 down vs 1 -7.99316 63.8906 0.978625 3.24725 1cg24530480 0.13216 0.378326 8.38E-14 0.349329 -2.86263 0 down vs 1 -7.993 63.8881 1.09869 3.64577 1cg23350904 ZMYND15 -0.121205 0.209987 8.40E-14 -0.577202 -1.7325 0 down vs 1 -7.99258 63.8813 1.98874 6.59994 1cg24794313 SCD5 0.26761 0.397295 8.41E-14 0.67358 -1.4846 0 down vs 1 -7.99246 63.8794 0.304927 1.01198 1cg21784383 ESRRG -0.09317 0.188012 8.41E-14 -0.495554 -2.01794 0 down vs 1 -7.99243 63.879 1.43348 4.75741 1cg12570975 0.052205 0.280263 8.42E-14 0.186271 -5.36852 0 down vs 1 -7.9923 63.8769 0.942997 3.1297 1cg15378866 KRT9 0.259745 0.390199 8.43E-14 0.665672 -1.50224 0 down vs 1 -7.9921 63.8737 0.308557 1.02412 1cg17606558 MAS1L 0.096895 0.362802 8.47E-14 0.267074 -3.74428 0 down vs 1 -7.99139 63.8623 1.28197 4.25568 1cg01418188 OSBPL5 7.50E-05 -0.193715 8.48E-14 -0.000387167 -2582.87 0 down vs 1 7.99122 63.8595 0.680895 2.26043 1cg01800521 -0.178035 0.138178 8.53E-14 -1.28844 1.28844 0 up vs 1 -7.99021 63.8434 1.81292 6.02002 1cg13648127 MAMDC2 0.12912 0.378617 8.56E-14 0.341031 -2.93229 0 down vs 1 -7.98959 63.8336 1.12862 3.74831 1cg13321605 0.193995 0.38246 8.57E-14 0.507229 -1.9715 0 down vs 1 -7.98942 63.8308 0.643992 2.13888 1cg09894929 NXPH1 -0.374565 -0.108523 8.59E-14 3.45149 3.45149 0 up vs 1 -7.98913 63.8262 1.28327 4.26242 1cg18325439 -0.39325 -0.0641299 8.61E-14 6.13209 6.13209 0 up vs 1 -7.98864 63.8184 1.96393 6.52404 1cg04465730 0.08491 0.359264 8.62E-14 0.236344 -4.23112 0 down vs 1 -7.98854 63.8167 1.36471 4.5336 1cg00972313 ADARB2 0.08231 0.321718 8.67E-14 0.255846 -3.90861 0 down vs 1 -7.98755 63.8009 1.03919 3.45305 1cg17489690 PRDM16 0.100795 0.396923 8.69E-14 0.253941 -3.93792 0 down vs 1 -7.98716 63.7947 1.58992 5.28357 1cg23683254 -0.18528 0.128808 8.70E-14 -1.43842 1.43842 0 up vs 1 -7.98698 63.7918 1.78863 5.94418 1cg26628476 0.14896 0.370839 8.71E-14 0.401684 -2.48952 0 down vs 1 -7.98689 63.7904 0.892588 2.96641 1cg03497419 EPM2AIP1 -0.310065 -0.469674 8.74E-14 0.660171 -1.51476 0 down vs 1 7.98636 63.782 0.461882 1.53521 1cg09119881 0.1664 0.379662 8.74E-14 0.438284 -2.28162 0 down vs 1 -7.98625 63.7802 0.824606 2.74092 1cg01814130 UPK1B 0.015025 0.297619 8.74E-14 0.050484 -19.8083 0 down vs 1 -7.98621 63.7796 1.44792 4.8128 1cg09722408 ABCA3 0.282115 0.414346 8.80E-14 0.680868 -1.46871 0 down vs 1 -7.9851 63.7619 0.317018 1.05404 1cg11842415 LHX8 -0.092805 0.223659 8.81E-14 -0.414939 -2.40999 0 down vs 1 -7.98505 63.761 1.8158 6.03738 1cg19451698 RHBDD1 -0.161435 -0.416138 8.82E-14 0.387937 -2.57774 0 down vs 1 7.98477 63.7566 1.17621 3.91107 1cg23698978 -0.143615 0.136852 8.83E-14 -1.04942 1.04942 0 up vs 1 -7.98468 63.7551 1.4262 4.74245 1cg09454925 KCNH7 -0.237285 0.0997314 8.84E-14 -2.37924 2.37924 0 up vs 1 -7.9844 63.7506 2.0593 6.84813 1cg07115879 KRT86 0.24785 0.390842 8.85E-14 0.634144 -1.57693 0 down vs 1 -7.98434 63.7497 0.370715 1.23282 1cg07905696 -0.1209 0.202025 8.86E-14 -0.598442 -1.67101 0 down vs 1 -7.98401 63.7444 1.89069 6.28802 1cg02763409 0.17509 0.394341 8.87E-14 0.444007 -2.25222 0 down vs 1 -7.98384 63.7418 0.871565 2.89876 1cg00589850 PCDHGA4 -0.10354 0.196016 8.89E-14 -0.528222 -1.89314 0 down vs 1 -7.98347 63.7357 1.62695 5.41161 1cg02960016 HRH1 0.144465 0.337912 8.91E-14 0.427522 -2.33906 0 down vs 1 -7.98328 63.7328 0.67849 2.25692 1cg07033292 TBC1D2B 0.172415 -0.0930139 8.91E-14 -1.85365 1.85365 0 up vs 1 7.98313 63.7304 1.27736 4.24917 1cg10356260 SORCS2 0.112575 0.313695 8.94E-14 0.358868 -2.78654 0 down vs 1 -7.98257 63.7215 0.733378 2.43993 1cg19913626 0.072865 0.303201 8.96E-14 0.240319 -4.16113 0 down vs 1 -7.98235 63.7179 0.961926 3.20049 1cg27326026 -0.11964 0.180686 8.99E-14 -0.662145 -1.51024 0 down vs 1 -7.98173 63.708 1.63532 5.44183 1cg14757339 TNXB 0.27746 0.468429 9.06E-14 0.59232 -1.68828 0 down vs 1 -7.98047 63.6879 0.661219 2.20102 1cg10148419 -0.006465 0.277309 9.08E-14 -0.0233134 -42.8939 0 down vs 1 -7.98016 63.683 1.46003 4.86043 1cg03075966 GRK5 0.29187 0.439309 9.09E-14 0.664384 -1.50515 0 down vs 1 -7.97997 63.68 0.394134 1.31213 1cg26239816 CRYBB2 0.29336 0.458587 9.11E-14 0.639705 -1.56322 0 down vs 1 -7.97968 63.6752 0.494969 1.64795 1cg19256656 PPP1R10 0.1375 0.328583 9.12E-14 0.418464 -2.38969 0 down vs 1 -7.97941 63.671 0.662006 2.20423 1cg02374984 CES1 0.07171 0.255033 9.16E-14 0.281179 -3.55645 0 down vs 1 -7.97883 63.6617 0.609329 2.02913 1cg09176893 0.163595 0.42428 9.17E-14 0.385583 -2.59348 0 down vs 1 -7.97856 63.6574 1.23211 4.10333 1cg08285768 AKAP13 0.243725 0.40409 9.20E-14 0.603146 -1.65797 0 down vs 1 -7.97811 63.6503 0.466268 1.553 1cg23202253 ITPR2 -0.023795 -0.299361 9.20E-14 0.079486 -12.5808 0 down vs 1 7.97807 63.6496 1.37679 4.58573 1cg13895046 TMPRSS11F 0.01794 0.307681 9.21E-14 0.0583071 -17.1506 0 down vs 1 -7.99701 63.9521 1.51678 4.86206 1cg08248231 DNAH17 0.04986 0.360061 9.23E-14 0.138476 -7.22145 0 down vs 1 -7.97758 63.6418 1.74464 5.81164 1cg08347471 PRODH2 0.262325 0.442339 9.23E-14 0.59304 -1.68623 0 down vs 1 -7.97749 63.6404 0.587532 1.9572 1cg01781963 0.20759 0.372004 9.24E-14 0.558031 -1.79201 0 down vs 1 -7.97731 63.6374 0.490113 1.63275 1cg05088356 MAST4 -0.00276 0.229025 9.28E-14 -0.0120511 -82.98 0 down vs 1 -7.97667 63.6273 0.974064 3.24549 1cg00364445 OR1I1 0.042915 0.31202 9.31E-14 0.137539 -7.27064 0 down vs 1 -7.97622 63.62 1.31299 4.37524 1cg06442199 BRDT 0.16437 0.378285 9.36E-14 0.434514 -2.30142 0 down vs 1 -7.97528 63.6051 0.829657 2.7653 1cg26772788 0.088675 0.331509 9.36E-14 0.267489 -3.73847 0 down vs 1 -7.97525 63.6046 1.06914 3.56356 1cg12871534 0.364925 0.442205 9.39E-14 0.825239 -1.21177 0 down vs 1 -7.97473 63.5964 0.108281 0.360959 1cg26173773 PTPRN2 -0.034155 0.329956 9.41E-14 -0.103514 -9.66054 0 down vs 1 -7.97443 63.5915 2.40372 8.01348 1cg23864180 ADARB2 -0.122265 0.316119 9.42E-14 -0.386769 -2.58552 0 down vs 1 -7.97432 63.5897 3.48439 11.6165 1cg00461236 0.02056 0.304657 9.42E-14 0.0674857 -14.818 0 down vs 1 -7.97419 63.5877 1.46336 4.87882 1cg12859720 0.315425 0.468495 9.45E-14 0.673273 -1.48528 0 down vs 1 -7.97375 63.5808 0.424812 1.41647 1cg24871165 -0.03177 0.164471 9.47E-14 -0.193165 -5.17693 0 down vs 1 -7.97345 63.576 0.698229 2.32831 1cg08775375 0.30918 0.464887 9.47E-14 0.665065 -1.50361 0 down vs 1 -7.97338 63.5747 0.439574 1.46583 1cg10519925 0.0458 0.337265 9.50E-14 0.135798 -7.36386 0 down vs 1 -7.97285 63.5664 1.54025 5.13687 1cg13117948 PROM1 0.00904 0.277105 9.52E-14 0.032623 -30.6532 0 down vs 1 -7.97252 63.561 1.30286 4.34554 1cg15782228 LAMA5 -0.026465 -0.304709 9.53E-14 0.0868534 -11.5137 0 down vs 1 7.97247 63.5603 1.40368 4.68186 1cg07316166 0.244195 0.377828 9.53E-14 0.646313 -1.54724 0 down vs 1 -7.97234 63.5583 0.323776 1.07996 1cg01232037 0.025415 0.29953 9.55E-14 0.0848495 -11.7856 0 down vs 1 -7.97208 63.5541 1.36234 4.5444 1cg25991026 AIG1 0.253995 0.395242 9.58E-14 0.642631 -1.5561 0 down vs 1 -7.97149 63.5446 0.361725 1.2068 1cg05967001 RPH3A -0.343975 0.0012201 9.59E-14 -281.923 281.923 0 up vs 1 -7.97143 63.5437 2.16046 7.20793 1cg10689409 OR9I1 0.00856 0.334205 9.60E-14 0.025613 -39.0426 0 down vs 1 -7.97127 63.5412 1.92267 6.41485 1cg12012383 EPHA10 -0.06401 0.188534 9.60E-14 -0.339514 -2.94538 0 down vs 1 -7.97125 63.5408 1.15636 3.85812 1cg14074305 0.00295 0.320312 9.70E-14 0.00920977 -108.58 0 down vs 1 -7.96958 63.5142 1.82611 6.09527 1cg27024272 -0.230685 0.0528711 9.70E-14 -4.36316 4.36316 0 up vs 1 -7.96953 63.5135 1.45779 4.86593 1cg03808001 -0.19157 0.203791 9.76E-14 -0.940033 -1.06379 0 down vs 1 -7.9686 63.4986 2.83403 9.46187 1cg01669185 0.13679 0.395059 9.77E-14 0.346252 -2.88807 0 down vs 1 -7.96834 63.4945 1.20938 4.03797 1cg20941737 ZBTB20 0.266795 0.422525 9.78E-14 0.63143 -1.58371 0 down vs 1 -7.96816 63.4916 0.439708 1.4682 1cg24767423 PXDN 0.15441 0.37002 9.81E-14 0.417302 -2.39635 0 down vs 1 -7.96771 63.4844 0.842861 2.81466 1cg00926420 -0.036585 0.223271 9.84E-14 -0.163859 -6.10279 0 down vs 1 -7.96722 63.4766 1.22428 4.08888 1cg18689958 RXFP3 -0.365225 0.0440727 9.90E-14 -8.28688 8.28688 0 up vs 1 -7.96623 63.4609 3.03736 10.1467 1cg05292082 TIAM1 0.078725 0.329504 9.91E-14 0.23892 -4.18551 0 down vs 1 -7.96607 63.4583 1.14025 3.80933 1cg24931520 -0.251415 0.0464361 9.93E-14 -5.41422 5.41422 0 up vs 1 -7.96574 63.453 1.60848 5.37403 1cg14024637 PLXNB3 0.223545 0.37971 9.94E-14 0.588725 -1.69859 0 down vs 1 -7.96555 63.45 0.442168 1.47738 1cg27185976 DLGAP2 0.078345 0.302534 9.95E-14 0.258963 -3.86155 0 down vs 1 -7.96546 63.4485 0.911265 3.0448 1cg05237470 DMD 0.103115 0.33365 9.97E-14 0.309051 -3.23571 0 down vs 1 -7.96517 63.444 0.963589 3.21986 1cg12591454 TRDMT1 0.119575 0.314366 9.97E-14 0.380369 -2.62903 0 down vs 1 -7.96513 63.4433 0.687948 2.29882 1cg10539507 NKX6-1 -0.33747 0.122491 9.99E-14 -2.75507 2.75507 0 up vs 1 -7.96485 63.4388 3.83583 12.8186 1cg12411704 ELFN1 0.160805 0.369377 1.00E-13 0.435341 -2.29705 0 down vs 1 -7.9647 63.4365 0.788731 2.63588 1cg04439513 OR5K4 0.086385 0.316977 1.00E-13 0.272528 -3.66935 0 down vs 1 -7.96691 63.4717 0.963596 3.2033 1cg07076109 FOXK1 0.328745 0.43798 1.01E-13 0.750594 -1.33228 0 down vs 1 -7.96386 63.423 0.216342 0.723152 1cg04169369 ADCY9 0.302535 0.434666 1.01E-13 0.696017 -1.43675 0 down vs 1 -7.96368 63.4202 0.316539 1.05812 1cg13792279 CLDN17 0.204755 0.396345 1.01E-13 0.516608 -1.93571 0 down vs 1 -7.96365 63.4196 0.665526 2.22473 1cg12034488 ZFR2 0.16001 0.422195 1.01E-13 0.378995 -2.63856 0 down vs 1 -7.96353 63.4178 1.24634 4.16639 1cg15202738 ZNF415 -0.414075 0.0272753 1.01E-13 -15.1813 15.1813 0 up vs 1 -7.96347 63.4169 3.53171 11.8064 1cg01318557 LAT2 -0.34556 -0.459949 1.01E-13 0.751301 -1.33102 0 down vs 1 7.96336 63.4152 0.237239 0.793102 1cg04382396 ELANE 0.134975 0.388941 1.01E-13 0.347032 -2.88158 0 down vs 1 -7.96235 63.399 1.16941 3.9104 1cg16772031 0.13748 0.363466 1.02E-13 0.378247 -2.64378 0 down vs 1 -7.96196 63.3928 0.92594 3.09656 1cg24154722 -0.051285 0.217934 1.02E-13 -0.235324 -4.24947 0 down vs 1 -7.96159 63.3869 1.3141 4.39507 1cg22978025 0.02832 0.328416 1.02E-13 0.0862321 -11.5966 0 down vs 1 -7.96401 63.4254 1.63203 5.42935 1cg13870199 C9orf27 0.21281 0.376744 1.02E-13 0.564867 -1.77033 0 down vs 1 -7.96109 63.379 0.487254 1.62984 1cg10823844 FGFR1 0.183895 0.365961 1.02E-13 0.502499 -1.99005 0 down vs 1 -7.96093 63.3765 0.601 2.0104 1cg19606462 ADCY2 0.095145 0.361923 1.03E-13 0.262887 -3.80391 0 down vs 1 -7.96035 63.3672 1.29038 4.31708 1cg06737561 CHAT -0.11173 0.165202 1.03E-13 -0.676323 -1.47858 0 down vs 1 -7.95993 63.3605 1.39048 4.65245 1cg11343811 0.055815 0.250298 1.03E-13 0.222994 -4.48442 0 down vs 1 -7.95984 63.3591 0.685774 2.2946 1cg25386234 -0.16211 0.125004 1.04E-13 -1.29684 1.29684 0 up vs 1 -7.9587 63.341 1.4946 5.00238 1cg18920097 GJD2 -0.192915 0.12276 1.04E-13 -1.57148 1.57148 0 up vs 1 -7.95846 63.3371 1.80675 6.04751 1cg10982851 FOXK1 0.29708 0.420907 1.04E-13 0.70581 -1.41681 0 down vs 1 -7.95803 63.3303 0.278001 0.930617 1cg00675721 0.144395 0.276992 1.04E-13 0.521297 -1.91829 0 down vs 1 -7.95775 63.3258 0.318775 1.06718 1cg24680320 -0.205485 0.0830247 1.05E-13 -2.47499 2.47499 0 up vs 1 -7.95717 63.3165 1.50917 5.0531 1cg13889963 CALB1 -0.222645 0.131494 1.05E-13 -1.6932 1.6932 0 up vs 1 -7.95663 63.3079 2.27387 7.61452 1cg23587176 FOXK1 0.197165 0.35833 1.06E-13 0.550232 -1.81741 0 down vs 1 -7.95554 63.2907 0.470936 1.57746 1cg06792368 0.15679 0.390722 1.06E-13 0.401283 -2.49201 0 down vs 1 -7.95533 63.2873 0.992197 3.32366 1cg13710662 -0.0859 0.272314 1.06E-13 -0.315445 -3.17013 0 down vs 1 -7.9553 63.2868 2.3265 7.79338 1cg23937382 ZSCAN1 -0.17374 0.0474902 1.06E-13 -3.65844 3.65844 0 up vs 1 -7.9551 63.2835 0.887374 2.97271 1cg18942757 LOC100128554 -0.09118 0.279453 1.06E-13 -0.326281 -3.06485 0 down vs 1 -7.95489 63.2802 2.49061 8.34398 1cg04256674 FAM20C 0.344425 0.439536 1.06E-13 0.783611 -1.27614 0 down vs 1 -7.95463 63.2761 0.164012 0.549505 1cg00522048 GRK5 0.106755 0.340458 1.07E-13 0.313563 -3.18916 0 down vs 1 -7.95452 63.2744 0.990256 3.31784 1cg13412545 -0.01298 0.192477 1.07E-13 -0.0674365 -14.8288 0 down vs 1 -7.95451 63.2742 0.765352 2.56431 1cg21211357 CPM -0.044595 -0.290782 1.07E-13 0.153362 -6.52051 0 down vs 1 7.95429 63.2707 1.09887 3.68198 1cg02520816 ADCY9 0.333995 0.483369 1.07E-13 0.690973 -1.44723 0 down vs 1 -7.95412 63.268 0.404546 1.35556 1cg24472965 NXPH2 0.13705 0.354395 1.07E-13 0.386716 -2.58588 0 down vs 1 -7.95359 63.2596 0.856479 2.87029 1cg06861044 UMOD -0.31969 0.0376474 1.07E-13 -8.49168 8.49168 0 up vs 1 -7.95309 63.2516 2.31513 7.7596 1cg11880367 0.152615 0.338567 1.08E-13 0.450767 -2.21844 0 down vs 1 -7.95231 63.2393 0.626931 2.10169 1cg04373359 TMEM30B -0.1355 -0.31121 1.08E-13 0.435397 -2.29676 0 down vs 1 7.95175 63.2303 0.559774 1.87682 1cg15724876 TGFBR2 0.30455 0.416153 1.08E-13 0.731823 -1.36645 0 down vs 1 -7.95171 63.2296 0.225822 0.757149 1cg21167817 PRDM16 0.176615 0.43718 1.08E-13 0.403987 -2.47533 0 down vs 1 -7.95161 63.2281 1.23098 4.12741 1cg17027260 SH3RF3 0.249865 0.4021 1.09E-13 0.6214 -1.60927 0 down vs 1 -7.95101 63.2185 0.420191 1.40909 1cg14968300 -0.131385 0.16401 1.09E-13 -0.801078 -1.24832 0 down vs 1 -7.95092 63.2171 1.58207 5.3055 1cg02341119 MGAT5B -0.12087 0.0810351 1.09E-13 -1.49158 1.49158 0 up vs 1 -7.9509 63.2169 0.739116 2.47865 1cg10211414 RERE 0.09724 0.315403 1.09E-13 0.308304 -3.24355 0 down vs 1 -7.9508 63.2153 0.86294 2.89397 1cg00447646 CACNG6 -0.14806 0.104632 1.09E-13 -1.41506 1.41506 0 up vs 1 -7.95079 63.215 1.15771 3.88255 1cg04879755 TMEM101 -0.100475 -0.400525 1.09E-13 0.250858 -3.98632 0 down vs 1 7.95018 63.2054 1.63232 5.47505 1cg11218330 ATP11A 0.20727 0.371978 1.10E-13 0.55721 -1.79466 0 down vs 1 -7.94973 63.1981 0.491869 1.64999 1cg02487930 0.097325 0.391019 1.10E-13 0.248901 -4.01766 0 down vs 1 -7.94959 63.1959 1.56389 5.24631 1cg25391814 0.288795 0.453436 1.10E-13 0.636903 -1.5701 0 down vs 1 -7.94933 63.1918 0.491467 1.6488 1cg16267202 -0.026395 0.269806 1.10E-13 -0.0978297 -10.2218 0 down vs 1 -7.94932 63.1917 1.59071 5.33662 1cg26802026 FBN2 0.028315 0.326163 1.10E-13 0.0868125 -11.5191 0 down vs 1 -7.94932 63.1917 1.60845 5.39614 1cg06118584 SNN 0.214315 -0.106893 1.10E-13 -2.00495 2.00495 0 up vs 1 7.94897 63.1861 1.87064 6.27631 1cg19127638 TET1 0.226115 0.40002 1.11E-13 0.565259 -1.7691 0 down vs 1 -7.94851 63.1788 0.548331 1.83995 1cg01261007 GPR6 -0.25714 -0.00202784 1.11E-13 126.805 126.805 0 up vs 1 -7.94832 63.1758 1.18 3.95973 1cg07048519 CDRT1 0.292535 0.416608 1.11E-13 0.702183 -1.42413 0 down vs 1 -7.94781 63.1677 0.279107 0.936723 1cg01101865 FLJ43390 -0.216015 0.103298 1.11E-13 -2.09118 2.09118 0 up vs 1 -7.9478 63.1676 1.84863 6.2043 1cg25942572 0.14049 0.38632 1.12E-13 0.363663 -2.7498 0 down vs 1 -7.94679 63.1515 1.09569 3.67823 1cg21617516 CAPN2 -0.11432 0.101427 1.12E-13 -1.12712 1.12712 0 up vs 1 -7.94626 63.143 0.843931 2.83346 1cg18652285 ATP11A 0.17222 0.384373 1.13E-13 0.448055 -2.23187 0 down vs 1 -7.94569 63.134 0.816047 2.74023 1cg21501724 PAK7 -0.32479 -0.0465763 1.13E-13 6.97329 6.97329 0 up vs 1 -7.94514 63.1252 1.40338 4.71311 1cg24089118 PTGDR -0.14995 0.0901402 1.13E-13 -1.66352 1.66352 0 up vs 1 -7.94499 63.1228 1.04512 3.51007 1cg11511443 LMO3 0.0405 0.307832 1.13E-13 0.131565 -7.6008 0 down vs 1 -7.94478 63.1196 1.29575 4.35204 1cg23472379 EBF3 0.142445 0.374426 1.13E-13 0.380436 -2.62856 0 down vs 1 -7.94476 63.1193 0.975713 3.27715 1cg03187190 0.06855 0.350677 1.13E-13 0.195479 -5.11564 0 down vs 1 -7.94466 63.1176 1.44313 4.84722 1cg25497487 CRYBB2 0.315205 0.462995 1.13E-13 0.680795 -1.46887 0 down vs 1 -7.94459 63.1165 0.396014 1.33016 1cg01860897 LRRC8D -0.15267 -0.385938 1.14E-13 0.395582 -2.52792 0 down vs 1 7.94377 63.1036 0.986568 3.31443 1cg08579577 -0.119655 0.209391 1.14E-13 -0.571442 -1.74996 0 down vs 1 -7.94359 63.1006 1.96305 6.59529 1cg02741226 UPK1B -0.03773 0.222227 1.14E-13 -0.169781 -5.88994 0 down vs 1 -7.94358 63.1004 1.22524 4.11648 1cg20544651 SCG3 -0.22897 0.0622139 1.15E-13 -3.68037 3.68037 0 up vs 1 -7.94283 63.0885 1.53728 5.16581 1cg22070156 ITGBL1 -0.14917 0.308675 1.15E-13 -0.483259 -2.06928 0 down vs 1 -7.94254 63.084 3.80062 12.7724 1cg17487010 TCEAL6 -0.091745 0.220758 1.15E-13 -0.415591 -2.40621 0 down vs 1 -7.94186 63.0731 1.77062 5.95137 1cg13202591 -0.31325 0.0171294 1.16E-13 -18.2873 18.2873 0 up vs 1 -7.9408 63.0564 1.97899 6.65351 1cg14984664 0.16586 0.421914 1.17E-13 0.393113 -2.5438 0 down vs 1 -7.93998 63.0434 1.18873 3.99741 1cg03514770 CSGALNACT1 0.10706 0.364469 1.17E-13 0.293742 -3.40434 0 down vs 1 -7.93992 63.0424 1.20134 4.03988 1cg05180522 0.009385 0.265364 1.17E-13 0.0353665 -28.2754 0 down vs 1 -7.93971 63.0391 1.18803 3.99535 1cg04493143 HTR4 0.216225 0.43272 1.17E-13 0.499688 -2.00125 0 down vs 1 -7.93915 63.0301 0.849791 2.85825 1cg10152131 -0.1332 0.213236 1.17E-13 -0.62466 -1.60087 0 down vs 1 -7.93906 63.0288 2.17603 7.31916 1cg19339179 ALPK1 0.197045 0.375237 1.17E-13 0.525121 -1.90432 0 down vs 1 -7.93904 63.0283 0.575698 1.9364 1cg25804146 VAV1 -0.001005 0.261687 1.18E-13 -0.00384046 -260.385 0 down vs 1 -7.93864 63.0219 1.25116 4.20878 1cg16461251 TACR3 -0.254565 0.0738278 1.18E-13 -3.44809 3.44809 0 up vs 1 -7.93845 63.019 1.95526 6.57763 1cg27312916 SYTL2 0.14066 0.354005 1.18E-13 0.397339 -2.51674 0 down vs 1 -7.9383 63.0167 0.825246 2.77628 1cg27179533 EMID2 -0.29785 0.0552299 1.18E-13 -5.39291 5.39291 0 up vs 1 -7.938 63.0119 2.26029 7.60462 1cg02856481 JARID2 0.33086 0.404487 1.18E-13 0.817974 -1.22253 0 down vs 1 -7.93792 63.0106 0.0982864 0.330686 1cg07217951 -0.017935 0.218197 1.19E-13 -0.0821964 -12.166 0 down vs 1 -7.93992 63.0423 1.01046 3.38195 1cg06459662 PHKB 0.16228 0.405638 1.19E-13 0.400062 -2.49962 0 down vs 1 -7.93728 63.0004 1.07376 3.61327 1cg12644668 PLCH2 0.140255 0.381209 1.19E-13 0.367922 -2.71797 0 down vs 1 -7.937 62.9959 1.05265 3.54249 1cg22065894 -0.025815 0.252976 1.19E-13 -0.102045 -9.79959 0 down vs 1 -7.93663 62.9901 1.40921 4.74286 1cg14691729 MIR521-1 0.03706 0.235917 1.20E-13 0.157089 -6.36581 0 down vs 1 -7.93583 62.9775 0.716968 2.41352 1cg13691003 LHX8 -0.282215 0.0577598 1.20E-13 -4.88601 4.88601 0 up vs 1 -7.93567 62.9749 2.09561 7.05472 1cg21434355 MIR29B1 0.38061 0.436657 1.20E-13 0.871646 -1.14725 0 down vs 1 -7.9351 62.9658 0.0569532 0.191756 1cg11815811 0.13809 0.375811 1.20E-13 0.367446 -2.72149 0 down vs 1 -7.93499 62.9641 1.0246 3.44981 1cg19764599 LHX8 -0.29606 0.0227268 1.21E-13 -13.0269 13.0269 0 up vs 1 -7.93439 62.9546 1.84255 6.20479 1cg15192932 PLXNB3 0.14878 0.301155 1.21E-13 0.494031 -2.02416 0 down vs 1 -7.93393 62.9472 0.420965 1.41777 1cg16294013 PDE4B -0.079935 0.174295 1.21E-13 -0.458618 -2.18046 0 down vs 1 -7.93349 62.9403 1.17185 3.94711 1cg21909859 PTDSS1 0.29635 0.422645 1.22E-13 0.701179 -1.42617 0 down vs 1 -7.93279 62.9292 0.289196 0.974264 1cg05913325 MOG 0.23961 0.408136 1.22E-13 0.587084 -1.70333 0 down vs 1 -7.9406 63.0531 0.514177 1.70433 1cg27420736 ACVR1C 0.273465 0.391411 1.23E-13 0.698665 -1.4313 0 down vs 1 -7.93144 62.9078 0.252222 0.849991 1cg17919152 -0.03787 0.353468 1.23E-13 -0.107139 -9.33371 0 down vs 1 -7.93137 62.9066 2.77665 9.35751 1cg05390075 -0.112165 0.0159593 1.23E-13 -7.0282 7.0282 0 up vs 1 -7.9312 62.9039 0.297633 1.00309 1cg11634248 SUV420H1 0.315625 0.39596 1.24E-13 0.797114 -1.25453 0 down vs 1 -7.93065 62.8952 0.117011 0.394406 1cg13156207 0.26826 -0.107676 1.24E-13 -2.49137 2.49137 0 up vs 1 7.9305 62.8928 2.56239 8.63735 1cg16093296 EBF3 0.143805 0.374753 1.24E-13 0.383733 -2.60598 0 down vs 1 -7.92982 62.8821 0.967045 3.26028 1cg02046247 0.06945 0.392362 1.24E-13 0.177005 -5.64956 0 down vs 1 -7.92964 62.8791 1.89054 6.37405 1cg14391855 RIN1 -0.123375 -0.378184 1.24E-13 0.326231 -3.06532 0 down vs 1 7.92959 62.8783 1.17719 3.969 1cg27401543 FTO 0.245595 0.415535 1.24E-13 0.591033 -1.69195 0 down vs 1 -7.9295 62.877 0.523612 1.76544 1cg17285663 -0.299315 0.0776624 1.25E-13 -3.85405 3.85405 0 up vs 1 -7.929 62.869 2.57661 8.68856 1cg19547319 LBX1 -0.160195 0.124291 1.25E-13 -1.28887 1.28887 0 up vs 1 -7.92868 62.864 1.46737 4.94849 1cg24680632 -0.03849 0.289461 1.25E-13 -0.132971 -7.52042 0 down vs 1 -7.92852 62.8614 1.95 6.57639 1cg22891413 SLC6A3 0.103685 0.369349 1.25E-13 0.280724 -3.56222 0 down vs 1 -7.92843 62.86 1.27963 4.31563 1cg10835417 MCTP2 0.236185 0.417061 1.25E-13 0.566308 -1.76582 0 down vs 1 -7.92838 62.8592 0.59317 2.00053 1cg07635744 GFOD1 0.29022 0.379415 1.26E-13 0.764913 -1.30734 0 down vs 1 -7.9281 62.8547 0.144246 0.486521 1cg10659811 -0.314205 -0.0020567 1.26E-13 152.771 152.771 0 up vs 1 -7.92776 62.8494 1.76661 5.95901 1cg18566911 PTPRN2 0.22423 0.360574 1.26E-13 0.621869 -1.60806 0 down vs 1 -7.92752 62.8456 0.337048 1.13698 1cg19759502 EDNRB -0.111275 0.131973 1.26E-13 -0.843167 -1.186 0 down vs 1 -7.92706 62.8383 1.07279 3.61932 1cg12476579 DOCK2 0.06703 0.295732 1.27E-13 0.226658 -4.41193 0 down vs 1 -7.92681 62.8342 0.948326 3.19961 1cg22277972 ISCU -0.25221 -0.41474 1.27E-13 0.608116 -1.64442 0 down vs 1 7.9267 62.8326 0.478942 1.61597 1cg21209859 -0.003195 0.192003 1.27E-13 -0.0166404 -60.0949 0 down vs 1 -7.92669 62.8325 0.690827 2.33088 1cg20797106 PTPRN2 0.23664 0.410071 1.28E-13 0.577071 -1.73289 0 down vs 1 -7.92543 62.8124 0.545346 1.84061 1cg12751456 PAX7 0.148165 0.35169 1.28E-13 0.421294 -2.37364 0 down vs 1 -7.92538 62.8117 0.751025 2.53484 1cg26870744 PDE4D -0.35108 -0.0527923 1.28E-13 6.65022 6.65022 0 up vs 1 -7.925 62.8056 1.6132 5.44535 1cg14766621 MYT1L -0.119605 0.280962 1.28E-13 -0.425698 -2.34908 0 down vs 1 -7.93544 62.9712 2.90345 9.59039 1cg06732976 0.21084 0.324527 1.28E-13 0.649684 -1.53921 0 down vs 1 -7.92457 62.7988 0.234335 0.791084 1cg27174698 0.170905 0.368178 1.29E-13 0.464191 -2.15429 0 down vs 1 -7.92422 62.7933 0.705594 2.3822 1cg19917650 -0.143845 0.156209 1.29E-13 -0.920851 -1.08595 0 down vs 1 -7.92409 62.7911 1.63236 5.51129 1cg23454797 HOXA5 -0.03556 0.275723 1.29E-13 -0.12897 -7.75375 0 down vs 1 -7.92324 62.7777 1.75683 5.9328 1cg26509915 0.17191 0.359643 1.30E-13 0.478001 -2.09204 0 down vs 1 -7.92274 62.7697 0.639 2.15817 1cg01165159 ARHGAP10 0.094845 0.299658 1.30E-13 0.316511 -3.15945 0 down vs 1 -7.92239 62.7643 0.760557 2.56895 1cg01301252 PCDHGA2 0.156085 0.351468 1.31E-13 0.444095 -2.25177 0 down vs 1 -7.92127 62.7465 0.692133 2.33849 1cg15468095 PYCARD -0.281475 -0.420631 1.31E-13 0.669173 -1.49438 0 down vs 1 7.92098 62.7419 0.351095 1.18632 1cg00188704 HOXA2 0.21118 0.397258 1.33E-13 0.531594 -1.88113 0 down vs 1 -7.91937 62.7164 0.627779 2.12208 1cg09771916 0.251365 0.468914 1.33E-13 0.536057 -1.86547 0 down vs 1 -7.91858 62.7039 0.858092 2.90118 1cg16215037 KLHL15 0.246185 0.398598 1.33E-13 0.617627 -1.6191 0 down vs 1 -7.91826 62.6988 0.421177 1.4241 1cg18148349 MGMT 0.12308 0.339089 1.34E-13 0.362972 -2.75503 0 down vs 1 -7.91741 62.6853 0.845984 2.8611 1cg26358950 SORCS2 0.12639 0.292955 1.34E-13 0.431432 -2.31786 0 down vs 1 -7.91697 62.6785 0.503018 1.70138 1cg00324128 0.077155 0.312141 1.35E-13 0.24718 -4.04564 0 down vs 1 -7.91656 62.6719 1.00116 3.38661 1cg15174834 HSPA1A -0.231565 -0.466665 1.35E-13 0.496213 -2.01527 0 down vs 1 7.91628 62.6675 1.00213 3.39013 1cg17015506 CD82 0.25242 0.344992 1.35E-13 0.731669 -1.36674 0 down vs 1 -7.91601 62.6632 0.155375 0.525658 1cg20301677 0.114275 0.34596 1.35E-13 0.330313 -3.02743 0 down vs 1 -7.91587 62.6609 0.973224 3.2927 1cg15736783 0.208345 0.353116 1.36E-13 0.590019 -1.69486 0 down vs 1 -7.91551 62.6554 0.379998 1.28576 1cg11931776 WNT7A 0.25588 0.390944 1.36E-13 0.654519 -1.52784 0 down vs 1 -7.91542 62.6539 0.330747 1.11914 1cg08822494 -0.06195 0.224626 1.36E-
[truncated: 2,164,977 more chars]
