# Supplementary material for: Identification and characterization of HPV-independent cervical cancers
Source: Oncotarget. 2017 Jan 6;8(8):13375–86. doi: 10.18632/oncotarget.14533 (PMC5355105; doi:10.18632/oncotarget.14533)
Supplement: Supplementary file 9 [file oncotarget-08-13375-s009.docx]

Genes methylated and silenced In HPV-inactive cervical cancers
ABHD3
ACAP3
ADAL
AIM1
AKAP10
ANXA11
APOL6
ARAP1
ARF6
ARSG
ATP11B
B3GNTL1
BCL2L1
BST2
C6orf147
CAMK2G
CAPG
CAPN2
CASP1
CASP8
CBY3
CCDC34
CCM2
CD274
CD58
CD82
CLSTN1
CMIP
CSRNP1
CSRP1
DAPP1
DDB2
DEDD2
DEF6
DERL3
DGKA
DHX58
EDEM1
EHBP1L1
EIF4EBP3
ETAA1
FAM53B
GALE
GNB4
GPX1
HIST1H1A
HIST1H2BK
HLA-E
HLA-F
HPDL
HSD17B8
HSPA1A
HSPA1B
HSPA1L
IFFO2
IFI35
IFITM4P
IL32
IL4R
INPP1
IRAK2
IRF7
ITGA6
ITGB2
IVNS1ABP
KHDC1
KIAA0922
KLF6
LAPTM5
LAT2
LBX2
LDLRAP1
LGALS9
LIMD2
LRRFIP1
LTB4R
LTB4R2
LYPLAL1
LYSMD2
MAFK
MAP3K14
MAP7D1
MARK2
MAX
MED29
MFN2
MGA
MST1R
MX1
MX2
NANP
NAPRT1
NEAT1
NEK9
NFIC
NINJ1
NKPD1
NLRC5
NLRP1
NMI
NOD2
NOTCH1
NPTN
NR3C1
OAS1
OAS2
OGDH
PARP14
PCYT1A
PDXK
PHF15
PITX1
PLAC8
PRKCH
PTEN
PTP4A2
PYCARD
RAD9B
RASSF1
RIN1
RNF19A
RNF213
RNLS
RPP25
RPS6KB2
S100A10
SBNO2
SDCBP2
SGMS1
SLC22A4
SLC43A3
SMCHD1
SP1
SP100
SP140L
SPDYA
SPTBN5
STK10
SVIL
SYTL1
TAPBPL
TMEM106A
TMEM173
TMEM185B
TPM4
TRERF1
TREX1
TRIM47
TRIM56
TRIM8
TTC22
UBA7
UBE2D3
UBXN11
UHRF1
UNC13D
WDR90
XAF1
ZC3H12A
ZC3H4
ZNF311
ZNF365
ZXDC
ZYX
